# NLRP3 Inflammasome Activation and Altered Mitophagy Are Key Pathways in Inclusion Body Myositis

**DOI:** 10.1002/jcsm.13672

**Published:** 2024-12-26

**Authors:** Elie Naddaf, Thi Kim Oanh Nguyen, Jens O. Watzlawik, Huanyao Gao, Xu Hou, Fabienne C. Fiesel, Jay Mandrekar, Eileen Kokesh, William S. Harmsen, Ian R. Lanza, Wolfdieter Springer, Eugenia Trushina

**Affiliations:** ^1^ Department of Neurology Mayo Clinic Rochester Minnesota USA; ^2^ Department of Neuroscience Mayo Clinic Jacksonville Florida USA; ^3^ Department of Molecular Pharmacology and Experimental Therapeutics Mayo Clinic Rochester Minnesota USA; ^4^ Neuroscience PhD Program Mayo Clinic Graduate School of Biomedical Sciences Jacksonville Florida USA; ^5^ Department of Quantitative Health Sciences Mayo Clinic Rochester Minnesota USA; ^6^ Division of Endocrinology and Metabolism Mayo Clinic Rochester Minnesota USA

**Keywords:** autophagy, inclusion body myositis, inflammasome, mitochondrial dysfunction, mitophagy

## Abstract

**Background:**

Inclusion body myositis (IBM) is the most prevalent muscle disease in adults for which no current treatment exists. The pathogenesis of IBM remains poorly defined. In this study, we aimed to explore the interplay between inflammation and mitochondrial dysfunction in IBM.

**Methods:**

The study population consisted of 38 IBM patients and 22 age‐ and sex‐matched controls without a myopathy. Mean age was 62.9 years (SD = 9) in IBM group and 59.7 (10) in controls. Bulk RNA sequencing, Meso Scale Discovery electrochemiluminescence (ECL), western blotting, histochemistry and immunohistochemistry were performed on frozen muscle samples from the study participants.

**Results:**

We demonstrated activation of the NLRP3 inflammasome in IBM muscle samples, with the NLRP3 inflammasome being the most upregulated pathway on RNA sequencing, along with increased expression of NLRP3 and ASC proteins in IBM group. *NLRP3* RNA levels most strongly correlated with *TLR7* (correlation coefficient *ρ* = 0.91) and complement activation‐related genes, and inversely correlated with several mitochondria‐related genes among others. On muscle histopathology, there was increased NRLP3 immunoreactivity in both inflammatory cells and muscle fibres. Mitophagy is critical for removing damaged mitochondria and preventing the formation of a vicious cycle of mitochondrial dysfunction—NLRP3 inflammasome activation. Herein, we showed altered mitophagy, as witnessed by the elevated levels of p‐S65‐Ubiquitin, a mitophagy marker, in muscle lysates from IBM patients compared to controls (median of 114.3 vs. 81.25 ECL units, *p* = 0.005). The p‐S65‐Ubiquitin levels were most significantly elevated in IBM males compared to male controls (136 vs. 83.5 ECL units; *p* = 0.013), whereas IBM females had milder nonsignificant elevation compared to female controls (97.25 vs. 69 ECL units, *p* = 0.31). On muscle histopathology, p‐S65‐Ubiquitin aggregates accumulated in muscle fibres that were mostly Type 2 and devoid of cytochrome‐c‐oxidase reactivity. *NLRP3 RNA* levels correlated with p‐S65‐Ubiquitin levels in both sexes (males: *ρ* = 0.48, females: *ρ* = 0.54) but with loss of muscle strength, as reflected by the manual motor test score, only in males (males: *ρ* = 0.62, females: *ρ* = −0.14). Lastly, we identified sex‐specific molecular pathways in IBM. Females had upregulation of pathways related to response to stress, which could conceivably offset some of the pathomechanisms of IBM, while males had upregulation of pathways related to cell adhesion and migration.

**Conclusions:**

There is activation of the NLRP3 inflammasome in IBM, along with altered mitophagy, particularly in males, which is of potential therapeutic significance. These findings suggest sex‐specific mechanisms in IBM that warrant further investigation.

## 1 Introduction

Inclusion body myositis (IBM) is the most common muscle disease in adults, for which there is no treatment. It is characterized by relentlessly progressive muscle weakness, with decreased longevity and high morbidity, as nearly all patients become wheelchair dependent as the disease progresses [1]. The pathomechanisms of IBM remain poorly understood, posing a major challenge for drug development. It is classified as an idiopathic inflammatory myopathy (IIM) mainly based on the associated histopathological findings, characterized by inflammatory infiltration of the endomysium, with highly differentiated, cytotoxic T cells [2]. However, unlike any other IIM, IBM is a disease of ageing that affects males twice as commonly as females and does not respond to immunosuppressant drugs [1, 3]. In addition to the inflammatory reaction, IBM is characterized by the accumulation of autophagic vacuoles and the deposition of proteins such as amyloid‐β peptides, phosphorylated tau protein, p62 and TDP43 [4]. As these features are similar to those of other ageing‐related diseases, such as Alzheimer's disease (ad) and Parkinson's disease (PD), IBM is also considered a degenerative disease. Furthermore, the relationships among these various pathways remain poorly understood.

Histopathological evidence of mitochondrial dysfunction, as indicated by the increased number of cytochrome c oxidase (CCO)‐negative fibres, is observed in almost every IBM muscle biopsy [5]. In addition, multiple mitochondrial DNA deletions occur in IBM muscles, with a greater mutation load in CCO‐negative fibres [6, 7, 8]. Mitochondrial dysfunction followed by endomysial inflammation are the two most commonly encountered histopathological features of IBM [5]. Understanding the intersection between the mitochondria and the immune system is a rapidly evolving field aiming to explore the mitochondrial control of inflammation on one hand, and the impact of inflammation on mitochondrial function on the other [9]. Mitophagy is considered a key mechanism in the mitochondrial control of inflammation [10]. However, a role for mitophagy in IBM has not been established thus far. The best characterized stress‐induced mitophagy pathway is jointly directed by the ubiquitin (Ub) kinase PINK1 and the Ub E3 ligase PRKN (parkin) [11]. In damaged mitochondria, PINK1 builds up on the outer mitochondrial membrane (OMM), activating the PINK1‐PRKN enzymatic pair. PINK1 and PRKN then selectively decorate damaged mitochondria with phosphorylated (p‐S65‐) ubiquitin (Ub) chains that facilitate their degradation by the autophagy‐lysosomal system. As such, the levels of p‐S65‐Ub are highly dynamic, they typically build up on damaged mitochondria, but then rapidly decline upon effective elimination of the organelles [11].

With the conundrum of involved pathways in IBM, systems biology approaches are best suited as exploratory methods for studying disease mechanisms. The results from previous transcriptomics studies were dominated by inflammation‐related genes, highlighting pathways related to T‐cell differentiation and cytotoxicity, the interferon‐ɣ signature, and immunoglobulin secretion [2, 12, 13, 14, 15, 16]. The strong expression of inflammation‐related genes posed a challenge to the exploration of noninflammatory pathways, which are essential for understanding IBM pathogenesis. Most importantly, sex differences in the transcriptome have never been addressed, despite the disease being more common and relatively more severe in males [3, 17]. This is particularly pertinent to mitochondrial pathways given the strong connection between mitochondria and biological sex. In addition to being maternally inherited, the rate limiting steps for the production of sex hormones occur in mitochondria and mitochondria contain receptors for sex hormones that influence mitochondrial function in various tissues, including skeletal muscle [18]. However, our understanding of sex‐specific differences in mitochondrial biology, including in skeletal muscle, remains limited. Female sex has been associated with better oxidative capacity and resilience to oxidative stress, mostly based on findings from animal studies, with limited studies in humans [19]. A recent systematic review and meta‐analysis evaluating various domains of mitochondrial biology in humans, showed that females have higher mitochondrial content in adipose tissue and leukocytes, and lower ROS production in skeletal muscle, but results in other domains were variable [18].

Hence, this study aimed to explore the interplay between mitochondrial dysfunction and inflammation in muscle tissue from male and female IBM patients, highlighting sex‐specific differences.

## 2 Methods

The study was approved by the Mayo Clinic Institutional Review Board. The study was considered minimal risk; therefore, the requirement for informed consent was waived. However, records of any patient who had not provided authorization for their medical records to be used for research, as per Minnesota statute 144.335, were not reviewed.

### 2.1 Patient Selection

A chart review of our electronic medical records was performed to identify IBM patients and controls. IBM diagnosis was established by fulfilling any category of the 2011 European Neuromuscular Centre (ENMC) diagnostic criteria; all participants also met the recently published 2024 ENMC criteria [20]. The control group consisted of participants without a myopathy that were identified from the Muscle laboratory database searchable by diagnosis followed by a chart review to ensure the included patients had no clinical, laboratory or histopathological evidence for a myopathy, including documented normal strength, normal creatine kinase levels, no fibrillation potentials on electromyography and no myopathy or inflammation on muscle biopsy. Controls underwent a muscle biopsy to rule out a muscle disorder, and the results of the workup were unremarkable. Slight denervation atrophy or rare CCO negative fibres (less than three per sample) were allowed as these findings are relatively common in the included age group. The IBM patients and controls were age‐ and sex‐matched at the group level. Disease duration was defined as the time from symptom onset until biopsy. The manual muscle test (MMT) score is a summated motor exam of bilateral shoulder abduction, elbow flexion, elbow extension, finger flexion, hip flexion, knee extension and the tibialis anterior. Each muscle is graded from 0 (*normal*) to 4 (*complete paralysis*), and the total score ranges from 0 (*normal strength*) to 56 (*complete paralysis*) as previously described [21]. MMT was calculated at time of the biopsy. All muscle biopsies were originally obtained for clinical purposes per our clinical practice standards, and residuals were used for this project. After overnight fasting, an open biopsy was performed the following day in the operating room under conscious sedation. The harvested muscle tissue was frozen in isopentane cooled in liquid nitrogen, and stored at −80°C according to our Muscle laboratory protocols.

### 2.2 RNA Extraction and Sequencing

Approximately 20–30 mg of fresh frozen muscle tissue per sample was sent to GENEWIZ, LLC/Azenta US, Inc. (South Plainfield, NJ, USA) for RNA extraction and sequencing. Total RNA was extracted using a Qiagen RNeasy Plus Universal Kit following the manufacturer's instructions (Qiagen, Hilden, Germany). Quantification was performed using a Qubit 2.0 fluorometer (Thermo Fisher Scientific, Waltham, MA, USA) and RNA integrity was checked using TapeStation (Agilent Technologies, Palo Alto, CA, USA). All included samples had a RIN > 6. The RNA sequencing library was prepared using the NEBNext Ultra II RNA Library Prep Kit for Illumina according to the manufacturer's instructions (New England Biolabs, Ipswich, MA, USA). The sequencing library was validated on an Agilent TapeStation (Agilent Technologies, Palo Alto, CA, USA) and quantified by using a Qubit 2.0 fluorometer (Thermo Fisher Scientific, Waltham, MA, USA) as well as by quantitative PCR (KAPA Biosystems, Wilmington, MA, USA). The sequencing libraries were multiplexed and clustered across four flow cell lanes. After clustering, the flow cells were loaded onto the Illumina HiSeq instrument according to the manufacturer's instructions. The samples were sequenced using a 2 × 150 bp paired‐end (PE) configuration. Image analysis and base calling were conducted with HiSeq Control Software (HCS). The raw sequence data (.bcl files) generated via Illumina HiSeq were converted into fastq files and demultiplexed using Illumina bcl2fastq 2.20 software. One mismatch was allowed for index sequence identification.

### 2.3 Transcriptomic Data Analysis

RNA sequencing data analysis was performed using Basepair software (https://www.basepairtech.com/) with a pipeline that included the following steps. The raw reads were trimmed using fastp to remove low‐quality bases from the reads (quality < 10) and adapter sequences. Trimmed reads with a minimum length of 15 bp were aligned to the ENSEMBL GRCh38 genome assembly using STAR with default parameters. The raw read counts were normalized using DESeq2 package. DESeq2 performs an internal normalization where geometric mean is calculated for each gene across all samples. The counts for a gene in each sample is then divided by this mean. The median of these ratios in a sample is the size factor for that sample. Alignments were sorted and indexed using samtools, and normalized bigwig files were created for visualization using deepTools with a bin size of 5 bp. Read counts were collected at gene annotations using feature counts, and the data were visualized using the IGV browser. Differential expression analyses were performed using DESeq2. Pairwise comparisons were performed using the Wald test. The minimum gene expression cutoff was set at 10. Genes with fold change values greater than 1.5 (|Log_2_ fold change| > 0.58) and a Benjamini–Hochberg adjusted *p* value ≤ 0.05 were considered DEGs. Analysis was performed on all IBM patients versus controls, male IBM patients versus male controls and female IBM patients versus female controls. Pathway enrichment analysis was carried out using the GSEA toolkit. Spearman rank correlation analysis was performed for *NLRP3* against the whole dataset of IBM patients. To explore sex‐specific differences, we identified sex‐specific DEGs, defined as genes that were differentially expressed in one sex but not in the other, using the same cutoffs as previously described. Genes with opposite trends were defined as those that were differentially expressed in both sexes but with opposite trends.

### 2.4 Protein Lysate Preparation

For protein lysate preparation, cell lysis buffer (Danvers, MA, USA, cat. #9803) supplemented with PMSF, phosphatase and protease inhibitors and 10–20 mg of fresh frozen muscle tissue were added to a tube with metal beads. Homogenization was performed using a Fisherbrand bead homogenizer followed by sonication. Centrifugation was then performed in a refrigerated (4°C) Beckman centrifuge. For protein quantification, we used a DC protein assay (Bio‐Rad) following the manufacturer's instructions.

### 2.5 Western Blot

The 6X sample buffer was added to the protein lysates, followed by denaturation at 95°C. Total protein lysates (25 μg/well) were separated on 4%–20% Criterion TGX™ Precast Protein Gels (Bio‐Rad, 4561096) and transferred to a PVDF membrane. The following primary antibodies were used: NLRP3 (1:390, Novus Biologicals, NBP2‐12446), ASC (1:500, Cell Signaling, E1E3I), vinculin (1:1000, Cell Signaling, Danvers, MA, USA, cat. #4650) and actin (1:1000, Cell Signaling, D18C11).

The following secondary antibodies were used: peroxidase (HRP), anti‐rabbit IgG (H + L), goat secondary antibody (1:5000, Jackson ImmunoResearch, 111‐035‐003) and rabbit TrueBlot, anti‐rabbit IgG HRP (1:5000, Rockland Immunochemicals, 18‐8816‐31). Membrane development was performed using a ChemiDoc Imaging System from Bio‐Rad. Quantification was performed using ImageJ software.

### 2.6 P‐S65‐Ub Measurement via Meso Scale Discovery‐Based Sandwich ELISA

A sandwich ELISA targeting p‐S65‐Ub was performed via the Meso Scale Discovery (MSD) platform that uses electrochemiluminescence (ECL), as described previously [22]. Briefly, 96‐well plates (Meso Scale Diagnostics, L15XA‐3) were coated overnight with 1‐μg/mL capture p‐S65‐Ub antibody (Cell Signaling Technology, 62802) in 200‐mM sodium carbonate buffer, pH 9.7. MSD plates were washed twice with washing buffer (150 mM Tris, pH 7.4, 150 mM NaCl, 0.1% [v:v] Tween‐20). The plates were then blocked with 1% BSA (w/v) in wash buffer for 1 h. Thirty micrograms of sample was prepared in blocking buffer and incubated for 2 h. The detergent volumes were kept consistent across all samples, and the samples were run in duplicate.

Mouse pink1‐/‐ skeletal muscle lysates were used as negative controls. The plates were washed three times and incubated with 5 μg/mL total Ub antibody (Thermo Fisher, 14‐6078‐37) for 2 h. The plates were washed and incubated with 1‐μg/mL SULFO‐TAG‐labelled goat anti‐mouse antibody (MSD, R32AC‐1) for 1 h. The plates were washed and measured immediately after the addition of 150 μL of MSD GOLD Read Buffer (MSD, R92TG‐2) on a MESO QuickPlex SQ 120 reader (Meso Scale Diagnostics, Rockville, MD, USA).

### 2.7 Muscle Histopathology

Frozen muscle sections (10 μm thick) were stained with haematoxylin and eosin, ATPase (pH 4.3 and 4.6), cytochrome c oxidase, NLRP3 (Novus Biologicals, NBP2–12446) and p‐S65 Ub antibodies. The p‐S65‐Ub antibody was developed in house and fully characterized and validated in cells and human tissue [23, 24]. Immunohistochemistry was performed on adjacent 10‐μm sections of fresh‐frozen human muscle fixed in cold acetone. p‐S65‐Ub‐stained sections were quenched in 0.3% hydrogen peroxide with methanol solution and rinsed with 1X PBS‐X, and all sections were blocked with normal donkey serum (1:10, Jackson ImmunoResearch, 017‐000‐121). Primary antibodies were incubated overnight at 4°C at concentrations of 1:650 p‐S65‐Ub and 1:50 NLRP3, followed by incubation with a biotinylated donkey anti‐rabbit secondary antibody (1:500, Jackson ImmunoResearch, 711‐065‐152). Stains were visualized using Vectastain ABC (Vector Laboratories, PK‐6100) and Dako DAB chromogen (Agilent, K346811‐2). Histomorphological evaluation of the slides was performed by E. N. (muscle pathologist) under light microscopy, and photos were taken with an Olympus DP73 camera.

### 2.8 Statistical Analysis

Continuous data are represented as the mean (SD) or median (interquartile range) as appropriate, and categorical variables are represented as proportions and percentages. Statistical analysis was performed using BlueSky statistics v.7.10 software, GraphPad Prism v.9.3.1 and SAS 9.4 software (SAS Inc., Cary NC) unless otherwise stated (refer to the transcriptomics section for transcriptomic analysis details). Two‐tailed Mann–Whitney tests and Pearson or Spearman correlations were used as appropriate.

### 2.9 Data Sharing Statement

The raw RNA sequencing data will be uploaded to dbGaP. Additional data are available from the corresponding author upon reasonable request.

## 3 Results

### 3.1 Study Cohort Characteristics

The study population consisted of 38 IBM patients and 22 controls without myopathy, with an equal proportion of males and females in each group (Table 1). The mean age at biopsy was 62.9 years in the IBM group and 59.7 years in the control group. The mean disease duration at the time of biopsy in the IBM group was 6 years, and the mean manual motor test (MMT) score was 16.6.

**TABLE 1:** Baseline characteristics and patient demographics.

Variable	Inclusion body myositis (*n* = 38)	Controls (*n* = 22)
**Age at biopsy** (years)	62.6 (9)	59.7 (10)
**Sex:**		
Females	19 (50)	11 (50)
Males	19 (50)	11 (50)
**Race:**		
American Indian/Alaskan Native	1 (2.6)	1 (4.5)
Black	1 (2.6)	0
White	32 (84.2)	19 (86.4)
Unknown/not disclosed	4 (10.5)	2 (9.1)
**Biopsy target**		
Quadriceps	14 (36.8)	8 (36.4)
Biceps brachii	9 (23.7)	5 (22.7)
Triceps brachii	10 (26.3)	3 (13.6)
Others	5 (13.2)	6 (27.3)
**Disease characteristics**		
Disease duration at time of the biopsy (years)	6.1 (4)	NA
Manual Motor Test score	16.6 (9.4)	0
Patients requiring any type of walking assistance at time of the biopsy	4 (10.5)	0

*Note:* Continuous variables are displayed as mean (SD) and categorical variables are displayed as the number of patients (percentage). NA: not applicable.

### 3.2 Transcriptomic Analysis of Samples From IBM Patients Versus Controls

**FIGURE 1:**
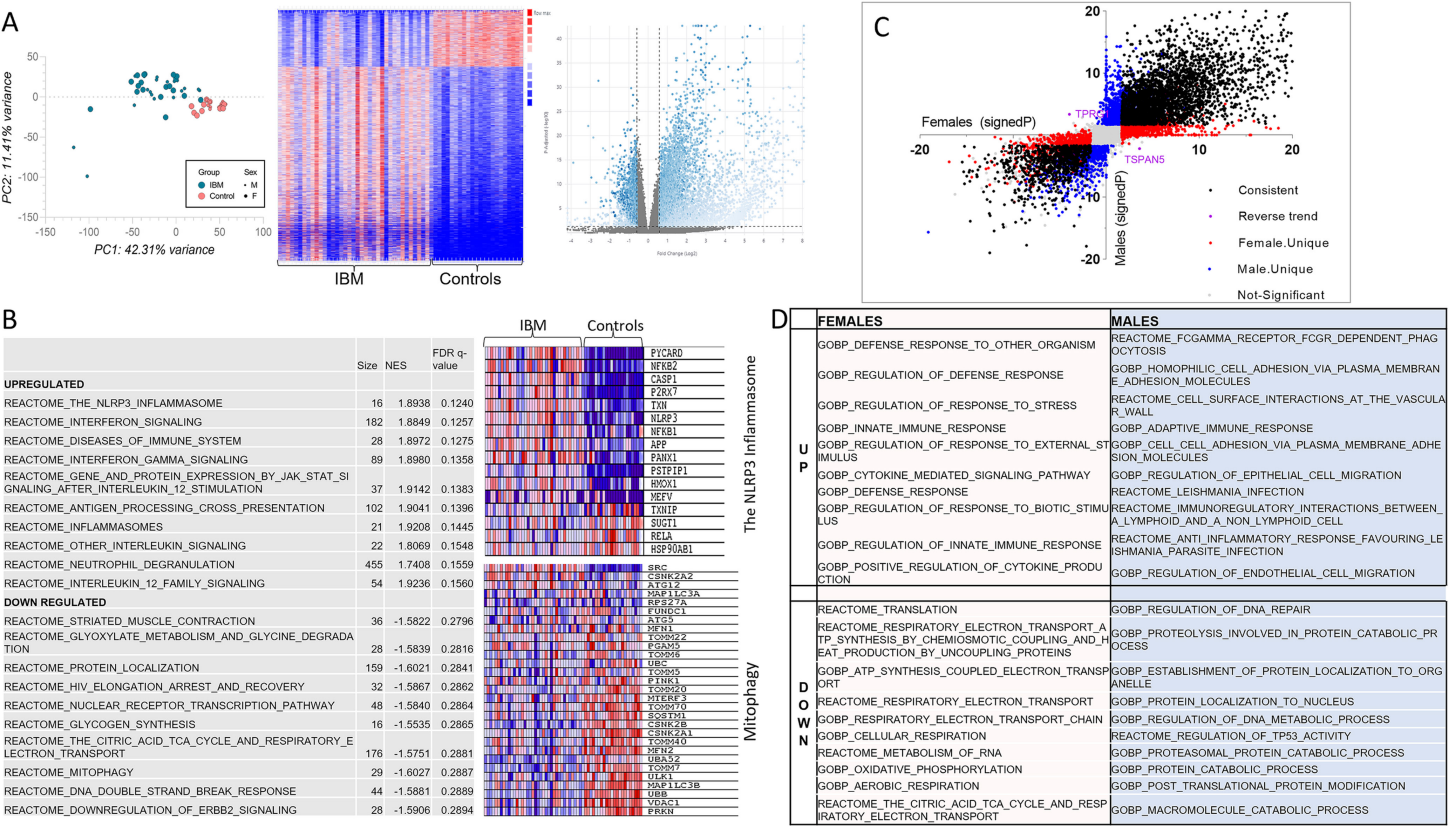
Inclusion body myositis transcriptome. Transcriptomic analysis of muscle tissue from 38 patients with inclusion body myositis and 22 controls. (A) PCA (to the left) and heatmap (middle) showing clear separation between IBM patients and controls. Volcano plot (to the right) representing differentially expressed genes (blue). (B) Top 10 upregulated and top 10 downregulated pathways in IBM. The NLRP3 inflammasome and mitophagy pathways are detailed in heatmaps, with downregulated genes shown in blue and upregulated genes shown in red. Transcriptomic sex differences are shown in C and D. (C) Volcano plot showing the signed ‐log_10_ (*p* value) for each gene in females on the *x*‐axis and in males on *the* y‐axis. Differentially expressed genes that are consistent in both sexes are shown in black, female‐unique genes are shown in red, male‐unique genes are shown in blue and genes with reverse trends are shown in purple. Genes that did not reach fold change or *p* value cutoffs in either sex are shown in grey (not significant). (D) The top 10 upregulated and downregulated pathways for sex‐specific genes.

To explore the molecular pathways involved in IBM pathogenesis, we first compared the IBM group to controls, including both males and females. Principal component analysis (PCA) revealed clear separation between the two groups (Figure 1A). There were 7448 differentially expressed genes (DEGs) in IBM: 5777 upregulated and 1671 downregulated (Figure 1A). A complete list of DEGs can be found in Table S1. GSEA revealed that the NLRP3 inflammasome pathway was the most upregulated pathway in IBM patients, followed by the interferon signalling pathway. Several of the top 10 downregulated pathways were related to mitochondria (the TCA cycle and respiratory electron transport, mitophagy and protein localization) (Figure 1B). To evaluate sex differences, we compared female IBM patients to female controls and male IBM patients to male controls. In females, there were 4743 upregulated and 1544 downregulated genes in the IBM group. In males, there were 4993 upregulated and 1623 downregulated genes in the IBM group. A complete list of DEGs in males and females can be found in Tables S2 and S3. We then identified sex‐specific DEGs, as shown in Figure 1C. There were 1754 female‐specific and 2083 male‐specific genes and 2 genes with opposite trends between the sexes. GSEA of sex‐specific genes (Figure 1D) revealed that the most upregulated female‐specific pathways were related to the response to stress, defence response and regulation of the immune response. However, the most upregulated male‐specific pathways were related to cell adhesion, cell migration and the Fc receptors for IgG‐dependent phagocytosis. The most downregulated sex‐specific pathways were related to RNA metabolism and translation, and electron transport chain and oxidative phosphorylation in females, and protein localization, ubiquitination and degradation, and the regulation of DNA repair in males. Only two genes had opposite trends (Figure 1C): *TPRG1* (Tumour Protein P63 Regulated 1) was upregulated in males and downregulated in females, and *TSPAN5* (Tetraspanin 5) was upregulated in females and downregulated in males. Information about the role of *TPRG1* is sparse. *TPRG1* promoted inflammation and NF‐КB signalling activation in a murine model of cystitis [25]. *TSPAN5* mediates signal transduction events that play a role in the regulation of cell development, migration and senescence [26].

### 3.3 NLRP3 Inflammasome Activation in IBM

**FIGURE 2:**
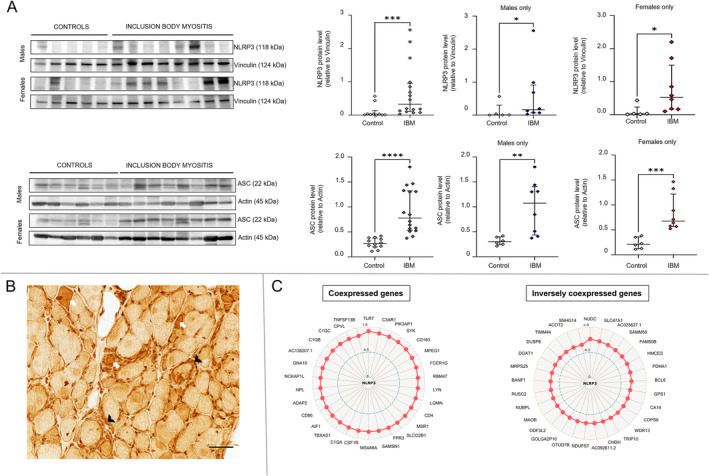
NLRP3 inflammasome activation in inclusion body myositis. (A) Western blot of muscle lysates from IBM patients and controls showing increased NLRP3 and ASC protein expression in samples from both males and females with IBM. The Mann–Whitney test was performed for group comparisons. Western blot results comparing the IBM group to the control group were as follows (Mann–Whitney U, two‐tailed *p* value): NLRP3 for both sexes (20, *p =* 0.0009), NLRP3 for males (6, *p =* 0.045), NLRP3 for females (3, *p =* 0.011), ASC for both sexes (4, *p <* 0.0001), ASC for males (3, *p =* 0.0047) and ASC for females (0, *p =* 0.0007). (B) NLRP3 immunohistochemistry showing increased NLRP3 immunoreactivity in scattered muscle fibres (examples shown as white arrows) as well as in inflammatory cells (examples shown as black arrowheads). (C) Radar maps displaying top coexpressed and inversely coexpressed genes with *NLRP3* according to Spearman rank correlation analysis. The correlation coefficient corresponds to the distance from the centre. All represented genes had a *p value* less than 0.0001.

Next, we validated the overall highest upregulated pathway, the NLRP3 inflammasome pathway, at the protein level in muscle samples from 16 IBM patients and 10 controls by western blotting. There was increased expression of two main NLRP3 inflammasome proteins, NLRP3 and ASC (apoptosis‐associated speck‐like protein containing CARD) (Figure 2A). Upregulation of NLRP3‐related genes and increased expression of the NLRP3 and ASC proteins were observed in samples from both males and females. Immunohistochemistry was performed on four IBM samples and four controls. It demonstrated increased NLRP3 immunoreactivity in both inflammatory cells and scattered muscle fibres in IBM samples (Figure 2B).

The aberrant activation and regulation of the NLRP3 inflammasome are intricate, and there is a growing body of literature elucidating its complexities [27]. To better understand the related genes in our dataset, coexpression analysis of *NLRP3* against all the other genes in the IBM dataset was performed using Spearman's rank correlation. The top 30 coexpressed and inversely coexpressed genes in the IBM samples are shown in Figure 2C. Most of the coexpressed genes were related to the immune system/inflammation, including *TLR7* (highest ρ = 0.91), complement activation (*C3AR1, FPR3*, *C1QA, C1QB* and *C1QC*), as well as genes related to metabolism (*TBXAS1, NPL*), and calcium homeostasis (*GNA15*). The top 30 inversely coexpressed genes included several mitochondria‐associated genes related to the mitochondrial inner and outer membranes (*SAMM50, MAOB* and *TIMM44*), metabolism (*PDHA1, CHDH* and *ACOT2*), complex I (*NDUFS7*) and mitochondrial protein synthesis (*MRPS25*). In addition, there were genes related to protein ubiquitination and degradation (*COPS6* and *SNHG14I*) and several either unidentified genes or genes of unknown function. The gene with the strongest inverse correlation was *NUDC* (*ρ* = −0.77) related to cell division.

### 3.4 Altered Mitophagy in Muscle Tissue From IBM Patients

**FIGURE 3:**
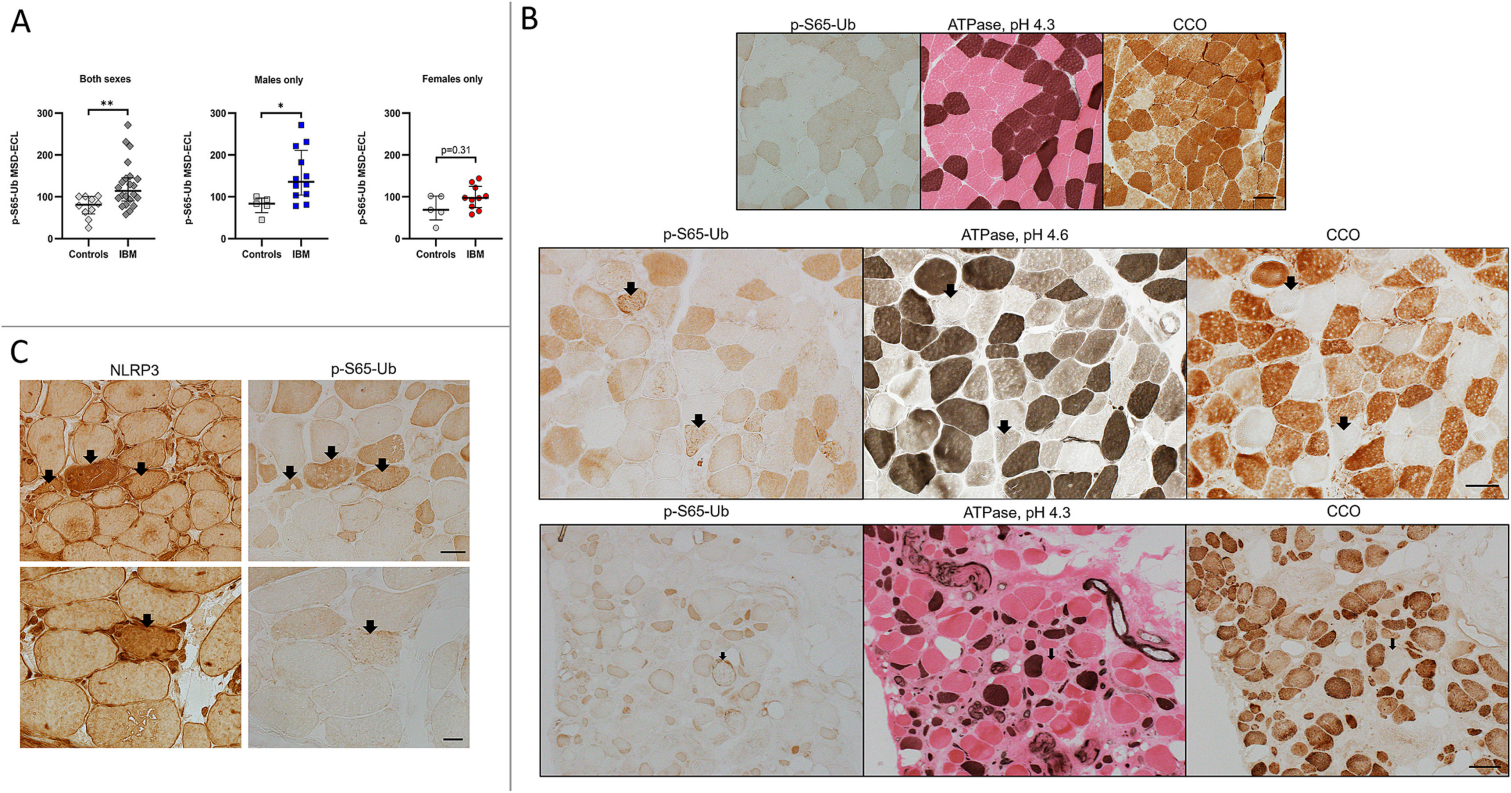
Altered mitophagy in inclusion body myositis patients. (A) Measurement of p‐S65‐Ub levels via sandwich ELISA in muscle samples from IBM patients and controls. The results comparing the IBM samples to the controls were as follows (Mann–Whitney U, two‐tailed *p* value): both sexes (43, *p =* 0.0054), males only (7, *p =* 0.0136) and females only (16, *p =* 0.31). MSD‐ECL:Meso Scale Discovery‐Electrochemiluminescence. (B and C) Frozen muscle sections from IBM samples and controls were reacted to p‐S65‐Ub, NLRP3 and ATPase at pH 4.3 (Type 1 fibres: dark brown, Type 2 fibres: pink) and at pH 4.6 (Type 1 fibres: dark brown; Type 2a fibres: light brown; Type 2b fibres: in between shade), and cytochrome c oxidase. (B) Top row: muscle samples from controls; p‐S65‐Ub shows clear differentiation of fibre types, reflecting the difference in baseline mitophagy and mitochondrial content between fibre types. Type 1 fibres, dark brown on ATPase 4.3, had increased p‐S65‐Ub and CCO reactivity. Middle and bottom rows: muscle samples from male and female IBM patients, respectively, demonstrating p‐S65‐Ub‐positive aggregates in scattered muscle fibres (examples shown as arrows). These fibres were almost exclusively Type 2 fibres (ATPase stains) that were also devoid of CCO activity (CCO‐negative fibres). Additionally, all CCO‐negative fibres, with or without observable abnormal p‐S65‐Ub reactivity, were Type 2 fibres. Scale bar for Panel B: top row: 50 μm; middle row: 50 μm; bottom row: 200 μm. (C) Muscle samples from IBM patients were reacted for NLRP3 and p‐S65‐Ub, demonstrating muscle fibres with increased NLRP3 and p‐S65‐Ub reactivity (arrows). Scale bar: top row 50 μm, bottom row 20 μm.

In the combined male and female analysis, mitophagy was among the top 10 downregulated pathways in the IBM cohort, and mitophagy plays an important role in regulating inflammation in general and NLRP3 inflammasome activation in particular [10]. To further explore whether mitophagy is altered in IBM muscles, we measured p‐S65‐Ub levels in muscle lysates from 22 IBM patients and 10 controls (Figure 3A). The level of p‐S65‐Ub was significantly greater in the IBM group than in the control group (median of 114.3 vs. 81.25 ECL units, *p =* 0.005*)*, indicating altered mitophagy. This can be due to either stronger mitophagy initiation or lower mitophagic degradation in IBM skeletal muscle. Analysis by sex revealed that compared with male controls, IBM males had significantly increased levels of p‐S65‐Ub (median of 136 vs. 83.5 ECL units, *p* = 0.014*)*, whereas compared with female controls, females showed only a mildly nonsignificant increase in p‐S65‐Ub (median of 97.25 vs. 69 ECL units, *p =* 0.31). To further explore the distribution of the findings at the tissue level, we performed muscle enzymatic and immunohistochemical studies on samples from 8 IBM patients and 4 controls (Figure 3B,C). In muscle tissue from controls, Type 2 fibres (fast twitch, lower mitochondrial content) had weak immunoreactivity to p‐S65‐Ub antibodies, while Type 1 fibres (slow twitch, higher mitochondrial content) strongly reacted. This might reflect the difference in baseline mitophagy, which is likely associated with the overall difference in mitochondrial content between fibre types. In contrast to samples from controls, there were p‐S65‐Ub‐positive aggregates in scattered muscle fibres in IBM muscles. These fibres were Type 2 fibres that were also devoid of CCO activity (CCO‐negative fibres). Type 2 fibres are known to be more prone to mitochondrial failure [28]. Additionally, all CCO‐negative fibres, with or without observable p‐S65‐Ub aggregates, were Type 2 fibres.

These data suggest that mitophagy alterations occur mostly in Type 2 fibres in IBM and are more significant in males. Additionally, several fibres with increased NLRP3 signal also exhibited increased p‐S65‐Ub immunoreactivity (Figure 3C), suggesting that these two pathways might be functionally associated.

### 3.5 Correlation of NLRP3 Inflammasome Activation With Altered Mitophagy and Muscle Weakness

**FIGURE 4:**
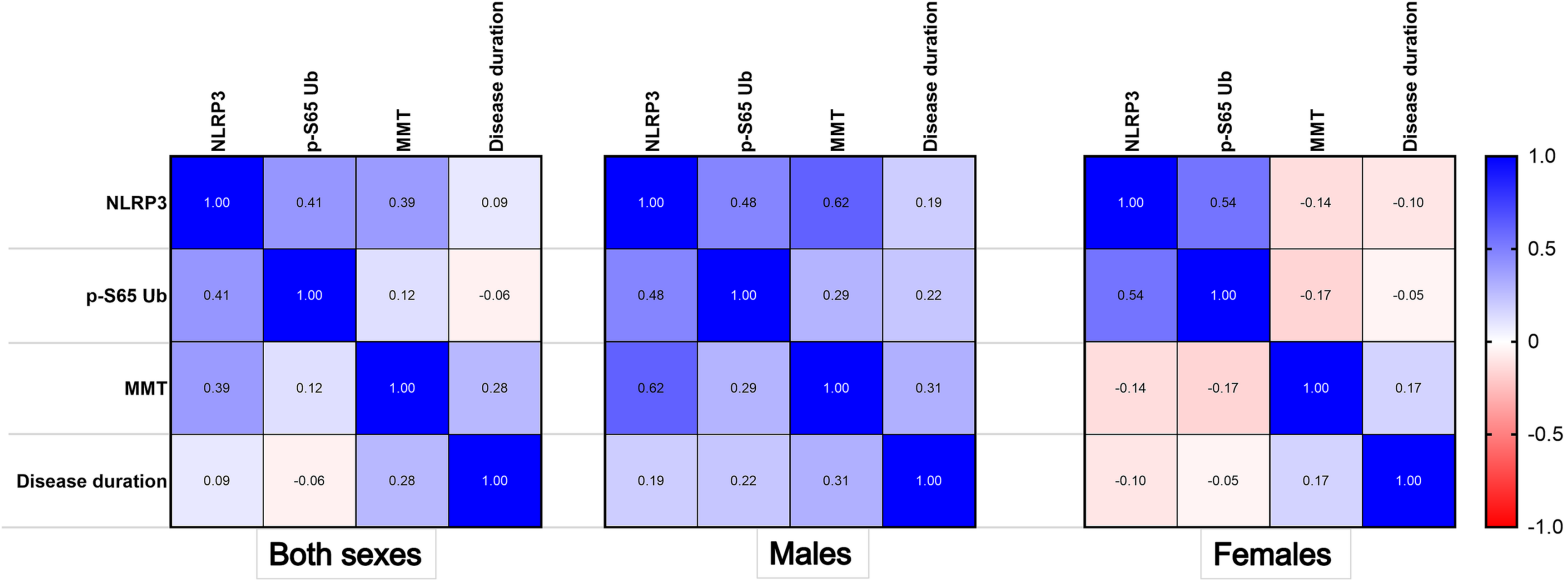
Correlation of NLRP3 inflammasome activation with altered mitophagy and muscle weakness. Pearson correlation plot demonstrating correlation coefficient values for the *NLRP3 RNA* level, p‐S65‐Ub level, manual muscle test (MMT) score and disease duration by sex. Both sexes: *NLRP3* and p‐S65‐Ub, *p*‐value = 0.059; *NLRP3* and MMT score, *p =* 0.072; *NLRP3* and disease duration, *p =* 0.543; p‐S65‐Ub and MMT score, *p =* 0.58; p‐S65‐Ub and disease duration, *p =* 0.699. Males: *NLRP3* and p‐S65‐Ub, *p =* 0.116; *NLRP3* and MMT score, *p =* 0.032; *NLRP3* and disease duration, *p =* 0.699; p‐S65‐Ub and MMT score, *p =* 0.359; p‐S65‐Ub and disease duration, *p =* 0.49. Females: *NLRP3* and p‐S65‐Ub, *p =* 0.106; *NLRP3* and MMT score, *p =* 0.706; *NLRP3* and disease duration, *p* = 0.781; p‐S65‐Ub and MMT score, *p =* 0.642; p‐S65‐Ub and disease duration, *p =* 0.898.

We next determined whether the *NLRP3* level or p‐S65‐Ub level was correlated with disease duration or disease severity, as reflected by the MMT score. The results are shown in Figure 4. The *NLRP3* level had a modest correlation with the p‐S65‐Ub level in both males and females (both sexes: correlation coefficient *ρ* = 0.41, males: *ρ* = 0.48, females: *ρ* = 0.54). *NLRP3* levels strongly correlated with MMT scores in males (*ρ* = 0.62) but not in females (*ρ* = −0.14). Neither the *NLRP3* nor the p‐S65‐Ub level correlated with disease duration. These findings further support the relationship between NLRP3 activation and altered mitophagy in IBM patients. Furthermore, NLRP3 activation is strongly associated with muscle weakness in males.

## 4 Discussion

In this study, we demonstrated the activation of the NLRP3 inflammasome and altered mitophagy in muscles samples from patients with IBM. Despite their intended protective role, aberrant activation of inflammasomes has been linked to the development of various chronic conditions, especially neurodegenerative and other ageing‐related diseases [27, 29]. NLRP3 remains the most studied inflammasome and has been implicated in the pathogenesis of many diseases such as ad, PD and amyotrophic lateral sclerosis, but studies on its role in muscle disorders are limited [27, 29, 30]. In a recent study on IBM, Kummer et al. demonstrated increased *NLRP3* mRNA levels by targeted qt‐PCR in muscle tissue from IBM patients and postulated that NLRP3 may be a key driver of inflammation and protein accumulation in IBM [31]. Herein, we demonstrated via unbiased analysis of transcriptomic data that the NLPR3 inflammasome pathway was indeed the most upregulated pathway in muscle tissue from both males and females with IBM with increased NLRP3 and ASC protein expression. The NLRP3 inflammasome belongs to the NOD‐like receptor (NLR) signalling pathway, which was the most significantly upregulated pathway in the IBM muscle according to the study by Murakami et al., validating our results [15]. However, the authors did not provide further details on the mechanism of NLR signalling pathway and it was grouped under ‘infection pathways’. Similarly, the ‘neuroinflammation signaling pathway’, which includes the NLRP3 inflammasome, was the most significantly upregulated pathway in IBM patients in a study by Pinal Fernandez et al. [13] Notably, the correlation between histopathological and clinical characteristics is typically weak in IBM, given the complexity of the disease and the heterogeneity of the IBM population [21]. However, we demonstrated a strong correlation between the *NLRP3* RNA level and the severity of muscle weakness, reflected by the MMT score, in males but not in females, supporting the clinical relevance of the activation of the NLRP3 inflammasome in IBM.

**FIGURE 5:**
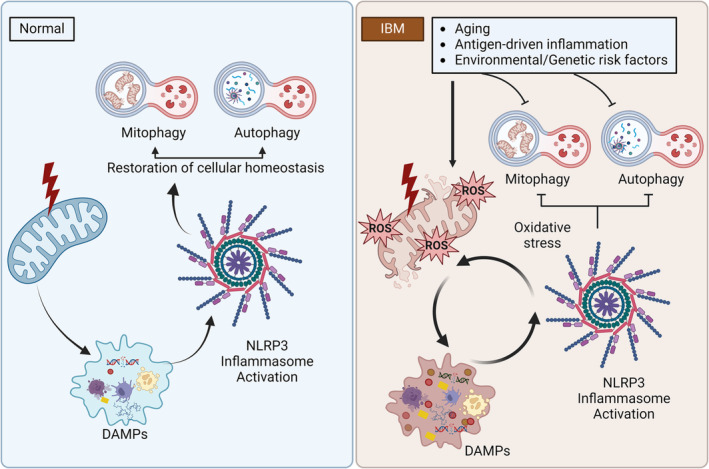
The vicious cycle of inflammasome activation‐mitochondrial dysfunction/altered mitophagy in inclusion body myositis. The release of mitochondrial damage‐associated molecular patterns (DAMPs) results in the activation of the NLRP3 inflammasome. Under normal conditions, damaged mitochondria and the NRLP3 inflammasome are both subsequently removed by mitophagy and autophagy, reestablishing cellular homeostasis. In IBM, mitophagy and autophagy are altered, establishing a feedforward loop in which the inflammatory milieu results in additional oxidative stress and mitochondrial dysfunction with further release of mitochondrial DAMPs and subsequent aberrant NLRP3 inflammasome activation.

Mitophagy removes dysfunctional mitochondria via the autophagic machinery to maintain cellular homeostasis and mitochondrial integrity. While the PINK1‐PRKN mitophagy pathway is genetically linked to PD, both proteins are expressed throughout the body, and this pathway likely plays an important role beyond the brain. However, there are limited studies on mitophagy in human skeletal muscles. PRKN is thought to play an important role in the maintenance of mitochondrial integrity in skeletal muscles, and ageing is associated with a decrease in mitophagy, mitochondrial function and muscle mass [32]. Herein, we show robust alterations in mitophagy in IBM. Elevated p‐S65‐Ub levels could stem from the accumulation of damaged mitochondria or from a block in the degradation of labelled mitochondria. Interestingly, the changes in p‐S65‐Ub levels were pronounced in males, and more research is needed to determine sex‐specific changes in the mechanism of PINK1‐PRKN mitophagy. At the tissue level, altered mitophagy was observed in CCO‐negative fibres. We also showed that loss of CCO activity and altered mitophagy both occur mainly in Type 2 fibres. Type 2 fibres are more prone to mitochondrial failure because they have lower mitochondrial content and lower oxidative capacity, are fast‐twitch fibres that rely on glycolysis, and exhibit lower expression of E3 ubiquitin ligases and proteasome‐mediated protein degradation [28]. CCO‐negative fibres in IBM have also been shown to have increased mitochondrial DNA mutation loads [8]. Moreover, the predilection of Type 2 fibres also raises suspicion for underlying metabolic disturbances, which have not yet been fully explored in IBM. Finally, unbiased coexpression analysis demonstrating that genes with strongest inverse correlation with the *NLRP3* level were mostly related to mitochondria, immunohistochemical studies demonstrating abnormal p‐S65‐Ub aggregation in fibres with increased NLRP3 immunoreactivity and the correlation between p‐S65‐Ub level and *NLRP3* level support that these pathways are functionally related in IBM. Based on these results, a vicious cycle of mitochondrial dysfunction/altered mitophagy and NLRP3 inflammasome activation is likely to occur in IBM (Figure 5). The release of mitochondrial damage‐associated molecular patterns (DAMPs), such as mtDNA, cytochrome c, mitochondrial reactive oxygen species (ROS) and mitochondria‐specific cardiolipin molecules, are known activators of the NLRP3 inflammasome [27]. Under normal conditions, damaged mitochondria and the NRLP3 inflammasome are both removed by mitophagy and autophagy, subsequently reestablishing cellular homeostasis [10, 33]. When mitophagy and autophagy are altered, as in IBM, a feedforward loop is established [10]. Therefore, the inflammatory milieu results in additional oxidative stress and mitochondrial dysfunction with further release of mitochondrial DAMPs and subsequent aberrant NLRP3 inflammasome activation. This vicious, self‐sustaining cycle may be a major contributor to the chronic deterioration of muscle strength in individuals with IBM. Although not directly assessed in our study, the 26S proteasome and autophagy, the two major pathways for protein degradation in eukaryotic cells, have both been shown to be impaired in IBM [34]. Additionally, decreased lysosomal proteolytic activity despite increased maturation of autophagosomes, as well as the upregulation and aggregation of chaperone‐mediated autophagy components, has been demonstrated in IBM muscles [35]. The accumulation of autophagic vacuoles and p62+ aggregates in muscle samples is used to aid in the histopathological diagnosis of IBM; p62 is a shuttle protein that transports polyubiquitinated proteins to either proteasomal or lysosomal degradation [36]. The original trigger for this self‐sustained detrimental loop remains unclear. An ageing skeletal milieu, with ageing mitochondria and an autophagic system, is likely required for these events to occur given the age of the population at risk. Furthermore, antigen‐driven inflammation at a preclinical stage preceding the activation of self‐sustained inflammatory pathways that become nontargeted by conventional immunotherapies is possible.

Another major finding of this study is the demonstration of sex differences in IBM pathomechanisms. Male sex is a known risk factor for IBM, as males are twice as likely to have the disease than females are [3]. Regarding disease severity, males are reported to have more significant limb weakness [17]. Furthermore, additional variations in phenotype by sex have been described [37]. Despite these phenotypic sex differences, none of the previous studies explored the underlying differential disease mechanisms. In this study, we identified sex‐specific genes and pathways that were differentially expressed only in one sex. Interestingly, the upregulated pathways in females could conceivably be protective, likely indicating that females fared to respond better to stress and to immune system activation, whereas in males, the upregulated pathways of cell adhesion and migration might have promoted disease pathogenesis. Increased expression of cell adhesion molecules, such as cadherin 1, has been reported in IBM [16]. For downregulated sex‐specific genes and related pathways, it is possible that females have decreased oxidative phosphorylation as a protection from oxidative stress or as a result of lower mitochondrial mass from more preserved mitophagy. In contrast, the pathways related to protein homeostasis and localization were downregulated in males. Disrupted protein homeostasis with the aggregation of autophagic vacuoles and misfolded protein aggregates is a canonical histopathological feature of IBM. Last, males had more significantly altered mitophagy than females, and *NLRP3* levels correlated with the severity of muscle weakness in males, supporting a direct association between NLPR3 activation and loss of muscle strength in this disease. Taken together, we speculate that more preserved mitophagy in females enable them to better turn down the activation of the NLRP3 inflammasome and limit subsequent tissue damage. This aligns with findings in other disorders where male sex is considered a risk factor, such as gout, aortic abdominal aneurysm and COVID19, in which the NLRP3 inflammasome was found to be overactivated in males and linked to worse disease outcomes [38, 39]. This has been attributed to several sex‐specific factors including a direct role of sex hormones, as testosterone can promote the activation of the NLRP3 inflammasome, whereas oestrogen inhibits it [39]. Furthermore, similar to our findings in the correlation analysis, there is a tight link between the complement cascade and NLRP3 activation [39]. The complement system has been recognized as source of sex‐based differences in vulnerability to various disorders [40]. Lastly, as the NRLP3 inflammasome is a highly regulated pathway that can be activated by various pathogen‐related and endogenous molecules, the interaction between NLRP3 activation and sex can occur at different levels, which likely varies by disease and tissue type [10]. These observations shed light on potential mechanistic differences between sexes, possibly explaining, at least in part, why males are more commonly and severely affected in IBM, and offer solid hypotheses to be explored in future studies and in other neuromuscular disorders.

Limitations of this study include the retrospective nature of the chart review and the lack of a valid disease model for IBM to further investigate the underlying pathomechanisms. The main strength of the study is that the experiments were performed on muscle samples from IBM patients offering a direct window into its pathogenesis. However, there is some heterogeneity inherent to the nature of the disease, stage of the disease at diagnosis, and muscle biopsy target.

The findings of this study may hold major translational significance. Small molecule mitochondria‐targeted therapeutics are being investigated for the treatment of neurodegenerative diseases of ageing [41]. Intervening on mitochondrial pathways requires characterizing the specific level of mitochondrial dysfunction in a particular disorder. Furthermore, there has been rapid advancement in developing strategies for inflammasome blockade and targeting both mitochondrial dysfunction and inflammasome activation in neurodegenerative diseases such as PD and ad [42, 43]. Deciphering the intertwined nature of neurodegenerative diseases resulting in self‐sustaining vicious cycles, such as those involving inflammasome activation and stalled mitophagy, has become of utmost importance, regardless of which is the cause versus the consequence. How to simultaneously target the various interwoven pathways involved in neurodegenerative diseases remains unknown and should be investigated in future studies.

## Conflicts of Interest

Mayo Clinic, F. C. F. and W. S. have filed a patent related to PRKN mitophagy activators. All other authors report no conflicts of interest.

## Supporting information


**Table S1** Differenctially expressed genes in inclusion body myositis, both sexes
**Table S2** Differenctially expressed genes in males with inclusion body myositis
**Table S3** Differenctially expressed genes in females with inclusion body myositis
