# Supplementary material for: NLRP3 Inflammasome Activation and Altered Mitophagy Are Key Pathways in Inclusion Body Myositis
Source: J Cachexia Sarcopenia Muscle. 2024 Dec 26;16(1):e13672. doi: 10.1002/jcsm.13672 (PMC11669947; doi:10.1002/jcsm.13672)
Supplement: Supplementary file 1 — Table S1 Differenctially expressed genes in inclusion body myositis, both sexes Table S2 Differenctially expressed genes in males with inclusion body myositis Table S3 Differenctially expressed genes in females with inclusion body myositis [file JCSM-16-e13672-s001.pdf]

Supplemental Table 1: Differenctially expressed genes in inclusion body myositis, both sexes

name	description	log2FoldChange	pvalue	padj
ENSG00000224373.3	IGHV4-59	9.583232472	42.72995929	1.86E-43
ENSG00000280411.1	IGHV1-69D	9.138777303	27.80319084	1.57E-28
ENSG00000117215.14	PLA2G2D	9.094665005	33.7850304	1.64E-34
ENSG00000130487.7	KLHDC7B	9.090821416	35.67592442	2.11E-36
ENSG00000211904.2	IGHJ2	9.053230975	32.13916234	7.26E-33
ENSG00000090104.11	RGS1	8.767199093	39.14434566	7.17E-40
ENSG00000282651.2	IGHV5-10-1	8.55681575	10.55068085	2.81E-11
ENSG00000251546.1	IGKV1D-39	8.486516344	20.99518657	1.01E-21
ENSG00000211640.4	IGLV6-57	8.348671743	25.75106213	1.77E-26
ENSG00000108700.4	CCL8	8.21622642	40.80691065	1.56E-41
ENSG00000275302.1	CCL4	8.138816945	39.97230698	1.07E-40
ENSG00000211965.4	IGHV3-49	8.116549828	19.9910172	1.02E-20
ENSG00000176083.17	ZNF683	8.078791083	18.76695751	1.71E-19
ENSG00000211638.2	IGLV8-61	8.004633345	20.91609207	1.21E-21
ENSG00000211950.2	IGHV1-24	7.988142103	17.3918035	4.06E-18
ENSG00000172724.11	CCL19	7.977772033	21.89900928	1.26E-22
ENSG00000187908.17	DMBT1	7.958349707	21.76613322	1.71E-22
ENSG00000255833.1	TIFAB	7.950784394	26.57194983	2.68E-27
ENSG00000277089.4	AC243829.4	7.907547026	36.9181146	1.21E-37
ENSG00000232216.1	IGHV3-43	7.839595983	14.4732129	3.36E-15
ENSG00000226777.7	FAM30A	7.803510508	25.22169436	6.00E-26
ENSG00000211970.3	IGHV4-61	7.787424768	21.7711394	1.69E-22
ENSG00000277632.1	CCL3	7.778526097	35.38100978	4.16E-36
ENSG00000188389.10	PDCD1	7.717155092	35.14475526	7.17E-36
ENSG00000211637.2	IGLV4-69	7.708735807	13.9381428	1.15E-14
ENSG00000211611.2	IGKV6-21	7.685161186	14.64639201	2.26E-15
ENSG00000229836.1	AL671883.1	7.611143423	28.12225996	7.55E-29
ENSG00000211685.3	IGLC7	7.559287589	14.94596567	1.13E-15
ENSG00000095970.16	TREM2	7.527907044	25.9167088	1.21E-26
ENSG00000156234.7	CXCL13	7.518351431	22.84891122	1.42E-23
ENSG00000211946.3	IGHV3-20	7.501114797	16.77600049	1.67E-17
ENSG00000231475.3	IGHV4-31	7.475786018	12.05121094	8.89E-13
ENSG00000186891.13	TNFRSF18	7.366523096	29.87905999	1.32E-30
ENSG00000181215.15	C4orf50	7.356140746	26.32706753	4.71E-27
ENSG00000152207.7	CYSLTR2	7.289071956	30.04931023	8.93E-31
ENSG00000133063.15	CHIT1	7.282994819	16.02495058	9.44E-17
ENSG00000239855.1	IGKV1-6	7.265472416	19.17634049	6.66E-20
ENSG00000211652.2	IGLV7-43	7.261549221	12.73406237	1.84E-13
ENSG00000251380.3	DCANP1	7.216445242	19.99393327	1.01E-20
ENSG00000152969.19	JAKMIP1	7.161712299	19.99393327	1.01E-20
ENSG00000166634.6	SERPINB12	7.111727025	10.40095312	3.97E-11
ENSG00000143297.18	FCRL5	7.077059506	15.68237038	2.08E-16
ENSG00000109943.8	CRTAM	7.047782519	19.53345128	2.93E-20
ENSG00000211964.3	IGHV3-48	7.021080329	16.35999705	4.37E-17
ENSG00000243063.1	IGKV3-7	7.012266341	12.59311162	2.55E-13

ENSG00000117560.7	FASLG	7.011631994	19.53498816	2.92E-20
ENSG00000238042.5	LINC02257	7.008731566	29.75356171	1.76E-30
ENSG00000174946.6	GPR171	7.005661485	22.91760955	1.21E-23
ENSG00000211937.3	IGHV2-5	6.977629572	14.02850761	9.36E-15
ENSG00000211670.2	IGLV3-9	6.974214388	11.29387742	5.08E-12
ENSG00000004846.16	ABCB5	6.957672144	7.619316375	2.40E-08
ENSG00000253998.3	IGKV2-29	6.938448862	8.653302287	2.22E-09
ENSG00000233093.5	LINC00892	6.897210902	18.34240805	4.55E-19
ENSG00000211650.2	IGLV5-45	6.886839666	9.195761272	6.37E-10
ENSG00000211972.2	IGHV3-66	6.886701329	8.737259919	1.83E-09
ENSG00000189233.11	NUGGC	6.853307934	18.41132888	3.88E-19
ENSG00000233665.8	AC060234.2	6.84879407	25.34290317	4.54E-26
ENSG00000143184.4	XCL1	6.831264676	12.83409069	1.47E-13
ENSG00000253522.6	MIR3142HG	6.8209061	22.53466136	2.92E-23
ENSG00000049249.8	TNFRSF9	6.818837785	20.55705915	2.77E-21
ENSG00000111537.4	IFNG	6.789247446	18.21359739	6.12E-19
ENSG00000211951.2	IGHV2-26	6.788102526	10.58712439	2.59E-11
ENSG00000251301.6	LINC02384	6.761132256	19.6535117	2.22E-20
ENSG00000281756.1	C2-AS1	6.730966941	23.15883276	6.94E-24
ENSG00000133328.3	HRASLS2	6.730178804	16.62460965	2.37E-17
ENSG00000078081.7	LAMP3	6.726243377	20.93732848	1.16E-21
ENSG00000244158.1	Z84488.1	6.719689404	20.34042816	4.57E-21
ENSG00000183734.4	ASCL2	6.701585365	19.00704993	9.84E-20
ENSG00000241294.1	IGKV2-24	6.693960375	19.41989752	3.80E-20
ENSG00000211941.3	IGHV3-11	6.668329815	18.70247426	1.98E-19
ENSG00000134028.14	ADAMDEC1	6.667830771	12.50790096	3.11E-13
ENSG00000254395.1	IGHV4-55	6.661453664	10.27235092	5.34E-11
ENSG00000136634.6	IL10	6.659304923	22.82573505	1.49E-23
ENSG00000242534.2	IGKV2D-28	6.655375106	8.254771128	5.56E-09
ENSG00000152766.5	ANKRD22	6.640972891	16.57695169	2.65E-17
ENSG00000163599.15	CTLA4	6.638889176	19.43292394	3.69E-20
ENSG00000224041.3	IGKV3D-15	6.633191319	10.33262973	4.65E-11
ENSG00000143185.3	XCL2	6.623803016	9.918508571	1.21E-10
ENSG00000178363.4	CALML3	6.606121935	6.031498044	9.30E-07
ENSG00000226472.8	AC008013.1	6.598063056	15.29829781	5.03E-16
ENSG00000078589.12	P2RY10	6.581353851	15.00248537	9.94E-16
ENSG00000256128.5	LINC00944	6.568973572	18.13102907	7.40E-19
ENSG00000211850.1	TRAJ39	6.55476933	12.63054174	2.34E-13
ENSG00000215853.3	RPTN	6.55463474	9.587506503	2.59E-10
ENSG00000211942.3	IGHV3-13	6.549407784	6.632333364	2.33E-07
ENSG00000007129.17	CEACAM21	6.545048263	20.01710325	9.61E-21
ENSG00000118160.13	SLC8A2	6.54075706	15.88237766	1.31E-16
ENSG00000187808.4	SOWAHD	6.515121455	15.26398309	5.45E-16
ENSG00000273604.1	EPOP	6.512473078	27.6857095	2.06E-28
ENSG00000080031.9	PTPRH	6.489491206	17.45715594	3.49E-18
ENSG00000282173.1	TRBJ1-5	6.480809675	12.7046283	1.97E-13
ENSG00000168389.17	MFSD2A	6.477561331	19.58103694	2.62E-20

ENSG00000256462.1	AL732437.1	6.425373971	5.339889851	4.57E-06
ENSG00000177984.6	LCN15	6.419458438	14.67581606	2.11E-15
ENSG00000211959.2	IGHV4-39	6.417314087	20.84796258	1.42E-21
ENSG00000105989.9	WNT2	6.415437167	5.948911815	1.12E-06
ENSG00000227191.8	TRGC2	6.41339685	13.70284924	1.98E-14
ENSG00000229142.1	HCG4P8	6.410062456	22.43275971	3.69E-23
ENSG00000274576.2	IGHV2-70	6.405792528	7.0168734	9.62E-08
ENSG00000253497.1	IGKV1-13	6.404965621	10.52693896	2.97E-11
ENSG00000265190.6	ANXA8	6.384056983	9.210765365	6.16E-10
ENSG00000135094.10	SDS	6.383813662	16.26332518	5.45E-17
ENSG00000238005.2	AL391832.2	6.382030858	23.76883746	1.70E-24
ENSG00000276070.4	CCL4L2	6.348986453	10.96300784	1.09E-11
ENSG00000211976.2	IGHV3-73	6.340276876	9.659709807	2.19E-10
ENSG00000132938.19	MTUS2	6.336562467	14.63599444	2.31E-15
ENSG00000174500.12	GCSAM	6.330396314	23.47285146	3.37E-24
ENSG00000166920.12	C15orf48	6.329631062	11.1498336	7.08E-12
ENSG00000135925.8	WNT10A	6.327333104	13.23862692	5.77E-14
ENSG00000111199.10	TRPV4	6.308609351	25.79409205	1.61E-26
ENSG00000211642.3	IGLV10-54	6.308516903	5.283716189	5.20E-06
ENSG00000109684.14	CLNK	6.302164974	14.31262494	4.87E-15
ENSG00000186810.7	CXCR3	6.289783556	24.92109518	1.20E-25
ENSG00000234663.6	LINC01934	6.286348204	14.20086889	6.30E-15
ENSG00000176092.15	CRYBG2	6.252140166	13.26354121	5.45E-14
ENSG00000237975.6	FLG-AS1	6.248252013	17.86580966	1.36E-18
ENSG00000240382.3	IGKV1-17	6.24571443	18.52807057	2.96E-19
ENSG00000239975.2	IGKV1D-33	6.243409515	7.283713049	5.20E-08
ENSG00000110848.8	CD69	6.225857638	18.34240805	4.55E-19
ENSG00000163600.12	ICOS	6.20229011	10.82900247	1.48E-11
ENSG00000273962.1	IGKV2-40	6.200704409	4.286888775	5.17E-05
ENSG00000211649.3	IGLV7-46	6.198717613	11.66593862	2.16E-12
ENSG00000276775.1	IGHV4-4	6.193121673	10.76834281	1.70E-11
ENSG00000167914.11	GSDMA	6.187134931	11.78619813	1.64E-12
ENSG00000138944.7	SHISAL1	6.185601223	17.09125948	8.10E-18
ENSG00000186265.9	BTLA	6.185410393	11.80842989	1.55E-12
ENSG00000197057.9	DTHD1	6.167788357	9.942170977	1.14E-10
ENSG00000240505.8	TNFRSF13B	6.160188505	9.422157758	3.78E-10
ENSG00000100298.15	APOBEC3H	6.156413236	11.85215725	1.41E-12
ENSG00000237568.1	AC099063.1	6.144735558	22.04464455	9.02E-23
ENSG00000254366.6	AC062004.1	6.144649394	7.15510515	7.00E-08
ENSG00000105246.5	EBI3	6.131486873	17.16749373	6.80E-18
ENSG00000198829.6	SUCNR1	6.113114495	18.07046742	8.50E-19
ENSG00000211945.2	IGHV1-18	6.111336395	18.36527209	4.31E-19
ENSG00000121933.18	TMIGD3	6.101860304	12.72471599	1.88E-13
ENSG00000123843.12	C4BPB	6.095072041	15.11142995	7.74E-16
ENSG00000211938.2	IGHV3-7	6.083346406	23.98281336	1.04E-24
ENSG00000170476.15	MZB1	6.080236732	22.42903736	3.72E-23
ENSG00000225698.3	IGHV3-72	6.07606893	7.980759914	1.05E-08

ENSG00000232818.2	RPS2P32	6.069754928	21.27474924	5.31E-22
ENSG00000214814.7	FER1L6	6.062560422	14.29526369	5.07E-15
ENSG00000145850.8	TIMD4	6.053668066	4.110969992	7.75E-05
ENSG00000236546.1	AL033527.2	6.053431777	15.33345997	4.64E-16
ENSG00000117090.14	SLAMF1	6.053188853	18.39827703	4.00E-19
ENSG00000268621.5	IGFL2-AS1	6.051425851	7.866376814	1.36E-08
ENSG00000151790.8	TDO2	6.049239707	18.32503761	4.73E-19
ENSG00000136541.14	ERMN	6.037820363	8.850144488	1.41E-09
ENSG00000198019.12	FCGR1B	6.031422776	11.51570021	3.05E-12
ENSG00000196301.3	HLA-DRB9	6.024727678	10.53799425	2.90E-11
ENSG00000211648.2	IGLV1-47	6.019573546	15.46681855	3.41E-16
ENSG00000282600.2	IGHV3-69-1	6.018391328	6.502029393	3.15E-07
ENSG00000211952.3	IGHV4-28	6.012305345	6.661012972	2.18E-07
ENSG00000211892.4	IGHG4	5.994548613	20.90461125	1.25E-21
ENSG00000223648.4	IGHV3-64	5.990267783	7.326514622	4.72E-08
ENSG00000261662.1	AL359752.1	5.986403163	18.0142041	9.68E-19
ENSG00000211947.2	IGHV3-21	5.981614629	12.77812857	1.67E-13
ENSG00000211935.3	IGHV1-3	5.979972284	8.435607446	3.67E-09
ENSG00000237111.1	IGHJ3P	5.973097688	7.879667079	1.32E-08
ENSG00000268758.7	ADGRE4P	5.931865988	8.69685731	2.01E-09
ENSG00000158488.15	CD1E	5.931736076	10.60793483	2.47E-11
ENSG00000147234.10	FRMPD3	5.924401379	12.37724897	4.20E-13
ENSG00000002079.14	MYH16	5.923287128	8.35644712	4.40E-09
ENSG00000186197.14	EDARADD	5.918170998	18.44225073	3.61E-19
ENSG00000060140.8	STYK1	5.916100565	13.69796978	2.00E-14
ENSG00000285681.1	AC091576.1	5.906142384	10.34960294	4.47E-11
ENSG00000267074.1	AC015911.3	5.899181149	9.476224144	3.34E-10
ENSG00000125726.10	CD70	5.888810309	13.07599621	8.39E-14
ENSG00000211653.2	IGLV1-40	5.878753906	23.52384864	2.99E-24
ENSG00000242076.2	IGKV1-33	5.876292421	22.06100574	8.69E-23
ENSG00000278030.1	TRBV7-9	5.874502964	6.38813505	4.09E-07
ENSG00000267757.4	EML2-AS1	5.869226365	16.93028616	1.17E-17
ENSG00000240864.3	IGKV1-16	5.867603785	17.88893502	1.29E-18
ENSG00000267554.1	AC015911.8	5.858517568	10.20259027	6.27E-11
ENSG00000245750.8	DRAIC	5.856367312	14.96179579	1.09E-15
ENSG00000253451.1	IGLV2-28	5.854208221	5.317093607	4.82E-06
ENSG00000140297.12	GCNT3	5.853985422	7.678958461	2.09E-08
ENSG00000234880.1	LINC00163	5.847869356	7.758215996	1.74E-08
ENSG00000243836.5	WDR86-AS1	5.847243582	9.830034139	1.48E-10
ENSG00000276566.1	IGKV1D-13	5.840950531	3.237043637	0.000579
ENSG00000142619.4	PADI3	5.838596275	3.570493471	0.000269
ENSG00000162888.4	C1orf147	5.83850225	12.36952102	4.27E-13
ENSG00000237422.1	AL158071.3	5.826684638	13.17441766	6.69E-14
ENSG00000282320.1	TRBJ1-1	5.814174046	8.469612271	3.39E-09
ENSG00000253755.1	IGHGP	5.801080912	42.03073147	9.32E-43
ENSG00000138311.16	ZNF365	5.790733041	8.633403821	2.33E-09
ENSG00000235576.1	LINC01871	5.788804899	7.518063696	3.03E-08

ENSG00000282608.1	ADORA3	5.774616384	20.00563335	9.87E-21
ENSG00000211966.2	IGHV5-51	5.769470984	20.74937103	1.78E-21
ENSG00000234883.5	MIR155HG	5.766382152	15.9688484	1.07E-16
ENSG00000211956.2	IGHV4-34	5.7656703	12.2840233	5.20E-13
ENSG00000275234.1	AC010503.4	5.761025667	10.95584373	1.11E-11
ENSG00000232810.3	TNF	5.749816633	17.27437045	5.32E-18
ENSG00000175077.5	RTP1	5.749314723	3.158396605	0.000694
ENSG00000227757.3	AP000282.1	5.748230289	7.413065941	3.86E-08
ENSG00000282939.1	TRBV7-2	5.731903044	8.285853957	5.18E-09
ENSG00000223350.2	IGLV9-49	5.729609892	5.759752241	1.74E-06
ENSG00000226674.9	TEX41	5.729599643	9.000964032	9.98E-10
ENSG00000265073.1	AC010761.2	5.723810595	14.75552601	1.76E-15
ENSG00000168081.8	PNOC	5.720282364	6.669486038	2.14E-07
ENSG00000224557.7	HLA-DPB2	5.718790885	4.279763089	5.25E-05
ENSG00000188916.8	INSYN2	5.717805642	12.17207999	6.73E-13
ENSG00000276144.1	AC233309.1	5.711809305	11.94221268	1.14E-12
ENSG00000251442.5	LINC01094	5.708445645	13.23267477	5.85E-14
ENSG00000280135.1	AL096816.1	5.703441453	8.974804385	1.06E-09
ENSG00000244437.1	IGKV3-15	5.697118385	24.08617487	8.20E-25
ENSG00000211673.2	IGLV3-1	5.690474608	23.05954291	8.72E-24
ENSG00000198846.5	TOX	5.687094634	19.8812773	1.31E-20
ENSG00000211955.2	IGHV3-33	5.682613405	19.43292394	3.69E-20
ENSG00000254586.1	AC124301.1	5.682330757	6.92450895	1.19E-07
ENSG00000197992.6	CLEC9A	5.676046758	8.937318186	1.16E-09
ENSG00000276085.1	CCL3L1	5.675397193	6.863522791	1.37E-07
ENSG00000211799.3	TRAV19	5.670605717	9.77855125	1.67E-10
ENSG00000182674.5	KCNB2	5.65465742	5.193427527	6.41E-06
ENSG00000056291.17	NPFFR2	5.654468966	11.02130827	9.52E-12
ENSG00000213057.5	C1orf220	5.648677249	12.95599784	1.11E-13
ENSG00000211595.2	IGKJ3	5.648203144	20.74295345	1.81E-21
ENSG00000256039.1	LINC02446	5.647844257	5.557438162	2.77E-06
ENSG00000211662.2	IGLV3-21	5.644656411	24.89335605	1.28E-25
ENSG00000211765.1	TRBJ2-2	5.642719815	8.156472787	6.97E-09
ENSG00000159871.14	LYPD5	5.635694743	13.56543247	2.72E-14
ENSG00000243290.3	IGKV1-12	5.626180394	20.70890304	1.95E-21
ENSG00000164220.6	F2RL2	5.611924717	7.200693654	6.30E-08
ENSG00000274370.1	AC130371.2	5.604213622	8.816252074	1.53E-09
ENSG00000278857.1	IGKV1D-12	5.603693454	5.005373596	9.88E-06
ENSG00000188282.12	RUFY4	5.60072204	8.154145012	7.01E-09
ENSG00000158485.10	CD1B	5.600330605	7.209241528	6.18E-08
ENSG00000160183.14	TMPRSS3	5.599257517	14.67581606	2.11E-15
ENSG00000177494.5	ZBED2	5.598975622	9.496357323	3.19E-10
ENSG00000205277.9	MUC12	5.594896909	9.87321401	1.34E-10
ENSG00000211678.2	IGLJ3	5.583050496	24.55924615	2.76E-25
ENSG00000236120.6	AC110995.1	5.58151041	14.03610429	9.20E-15
ENSG00000259278.1	AC087878.1	5.575856253	7.384669309	4.12E-08
ENSG00000236581.8	STARD13-AS	5.570508732	10.7731114	1.69E-11

ENSG00000103154.9	NECAB2	5.567916301	6.056962442	8.77E-07
ENSG00000203814.6	HIST2H2BF	5.565802507	8.945668062	1.13E-09
ENSG00000198010.12	DLGAP2	5.563352276	9.977739223	1.05E-10
ENSG00000211658.2	IGLV3-27	5.558458466	3.361310164	0.000435
ENSG00000182010.10	RTKN2	5.551858558	9.598149113	2.52E-10
ENSG00000282420.1	TRBJ1-2	5.547230449	6.679791587	2.09E-07
ENSG00000119283.15	TRIM67	5.529104453	8.647628838	2.25E-09
ENSG00000172673.10	THEMIS	5.525188309	13.1060302	7.83E-14
ENSG00000127561.14	SYNGR3	5.524424584	8.67481281	2.11E-09
ENSG00000285761.1	AL645939.5	5.522610113	8.018894471	9.57E-09
ENSG00000159618.15	ADGRG5	5.512012106	16.17601023	6.67E-17
ENSG00000183542.5	KLRC4	5.511888095	12.54796786	2.83E-13
ENSG00000122733.12	PHF24	5.511463736	9.870300608	1.35E-10
ENSG00000167656.4	LY6D	5.505883154	3.330141316	0.000468
ENSG00000128165.8	ADM2	5.500879019	10.67171292	2.13E-11
ENSG00000243238.1	IGKV2-30	5.4915082	16.63931453	2.29E-17
ENSG00000225871.2	AC245100.2	5.491327648	13.11607091	7.65E-14
ENSG00000281958.1	TRBJ1-4	5.482736576	9.489788568	3.24E-10
ENSG00000165695.9	AK8	5.477863836	11.76657151	1.71E-12
ENSG00000285744.1	AC083837.2	5.47454692	8.613013388	2.44E-09
ENSG00000147138.2	GPR174	5.473016992	14.66472453	2.16E-15
ENSG00000231858.5	AC067945.3	5.467130705	8.69062441	2.04E-09
ENSG00000227644.2	HIGD1AP11	5.454211197	7.175597722	6.67E-08
ENSG00000277941.1	RF01875	5.453159802	7.119205924	7.60E-08
ENSG00000183395.4	PMCH	5.444938234	12.54981335	2.82E-13
ENSG00000211734.3	TRBV5-1	5.443302054	5.931680507	1.17E-06
ENSG00000113088.5	GZMK	5.434026316	14.30101732	5.00E-15
ENSG00000224081.8	SLC44A3-AS1	5.432706761	9.104009954	7.87E-10
ENSG00000211746.3	TRBV19	5.43104137	4.372218807	4.24E-05
ENSG00000235034.6	C19orf81	5.429096777	15.06650608	8.58E-16
ENSG00000170356.9	OR2A20P	5.426207243	6.558526361	2.76E-07
ENSG00000009765.14	IYD	5.424789529	4.040306291	9.11E-05
ENSG00000244116.3	IGKV2-28	5.423978379	24.90778652	1.24E-25
ENSG00000231128.5	AL137856.1	5.417288203	7.808240973	1.56E-08
ENSG00000240498.7	CDKN2B-AS1	5.415516724	8.82042122	1.51E-09
ENSG00000211967.3	IGHV3-53	5.415106019	11.09571434	8.02E-12
ENSG00000284049.1	MIR650	5.405372554	14.61790876	2.41E-15
ENSG00000211639.2	IGLV4-60	5.398379928	3.20132636	0.000629
ENSG00000284336.1	MIR2277	5.396911423	11.47412819	3.36E-12
ENSG00000162676.11	GFI1	5.392923874	14.39781419	4.00E-15
ENSG00000270550.1	IGHV3-30	5.391575846	19.99610011	1.01E-20
ENSG00000156466.9	GDF6	5.38884026	5.753702933	1.76E-06
ENSG00000240535.8	AC034238.1	5.386560395	14.10511859	7.85E-15
ENSG00000178773.14	CPNE7	5.385530758	5.855770269	1.39E-06
ENSG00000261438.1	AL157394.1	5.385194385	12.60394516	2.49E-13
ENSG00000140279.12	DUOX2	5.37028408	2.566532714	0.002713
ENSG00000183347.14	GBP6	5.358680528	22.03446366	9.24E-23

ENSG00000285165.1	AC124014.1	5.356401737	7.789923438	1.62E-08
ENSG00000188396.3	TCTEX1D4	5.354009169	7.870036873	1.35E-08
ENSG00000225465.8	RFPL1S	5.351379739	8.611409831	2.45E-09
ENSG00000284386.1	MIR147B	5.344286936	5.333712239	4.64E-06
ENSG00000285697.1	AC002059.2	5.342351433	11.02878102	9.36E-12
ENSG00000211674.2	IGLJ1	5.333825256	26.64679024	2.26E-27
ENSG00000233393.1	AP000688.2	5.330393176	7.85902661	1.38E-08
ENSG00000281852.1	LINC00891	5.329877731	7.083113892	8.26E-08
ENSG00000266088.5	AC004585.1	5.323944048	7.081855035	8.28E-08
ENSG00000232774.7	FLJ22447	5.323647361	5.260520053	5.49E-06
ENSG00000211949.3	IGHV3-23	5.315570196	27.44295105	3.61E-28
ENSG00000253210.1	AC040970.1	5.314835432	7.701561094	1.99E-08
ENSG00000253811.1	AC136424.2	5.313976302	6.871764744	1.34E-07
ENSG00000275385.1	CCL18	5.309411314	30.22696589	5.93E-31
ENSG00000102962.4	CCL22	5.30775844	8.387477537	4.10E-09
ENSG00000187616.4	MYMK	5.303195504	16.59169481	2.56E-17
ENSG00000259580.1	AC023908.2	5.302887344	7.523139202	3.00E-08
ENSG00000091181.19	IL5RA	5.302168252	5.104667773	7.86E-06
ENSG00000260920.2	AL031985.3	5.299987693	9.253877488	5.57E-10
ENSG00000211896.7	IGHG1	5.294540914	44.04814134	8.95E-45
ENSG00000237286.1	AC004906.1	5.293815624	5.805181088	1.57E-06
ENSG00000230882.1	AC005077.4	5.293489613	7.008948005	9.80E-08
ENSG00000255642.1	PABPC1P4	5.293412932	6.232067133	5.86E-07
ENSG00000225523.2	IGKV6D-21	5.291362624	2.788206693	0.001629
ENSG00000181656.6	GPR88	5.29037746	8.537954404	2.90E-09
ENSG00000211676.2	IGLJ2	5.290374119	35.13863472	7.27E-36
ENSG00000254481.1	PTP4A2P2	5.290261462	6.885034381	1.30E-07
ENSG00000211747.3	TRBV20-1	5.290072424	8.114770167	7.68E-09
ENSG00000211684.2	IGLJ7	5.288814837	3.506268441	0.000312
ENSG00000259363.5	AC090825.1	5.284961808	9.108850497	7.78E-10
ENSG00000189409.13	MMP23B	5.279689376	7.628765746	2.35E-08
ENSG00000241351.3	IGKV3-11	5.279601838	26.09652405	8.01E-27
ENSG00000230729.1	AL160270.1	5.274235224	5.594229104	2.55E-06
ENSG00000169248.12	CXCL11	5.273386869	23.96438616	1.09E-24
ENSG00000132832.10	AL139352.1	5.271420019	5.666577052	2.15E-06
ENSG00000089692.8	LAG3	5.269496131	20.76397613	1.72E-21
ENSG00000154620.5	TMSB4Y	5.267603215	4.717423639	1.92E-05
ENSG00000173404.4	INSM1	5.263758256	5.199668022	6.31E-06
ENSG00000241755.1	IGKV1-9	5.257363183	13.63395002	2.32E-14
ENSG00000241244.1	IGKV1D-16	5.252437881	3.399608221	0.000398
ENSG00000235151.1	AC131097.4	5.251948244	7.054499283	8.82E-08
ENSG00000186897.4	C1QL4	5.25099045	3.104118529	0.000787
ENSG00000035720.7	STAP1	5.250568035	3.739718999	0.000182
ENSG00000242472.1	IGHJ5	5.246740261	24.69937189	2.00E-25
ENSG00000100053.9	CRYBB3	5.245907498	6.969098847	1.07E-07
ENSG00000211593.2	IGKJ5	5.24484946	21.58602159	2.59E-22
ENSG00000185985.9	SLITRK2	5.232624096	4.229861355	5.89E-05

ENSG00000253715.1	AC083841.2	5.228194644	2.792666617	0.001612
ENSG00000264954.1	PRR29-AS1	5.224025208	8.355438291	4.41E-09
ENSG00000270164.1	LINC01480	5.221039996	6.84440896	1.43E-07
ENSG00000010030.13	ETV7	5.21782926	20.26787219	5.40E-21
ENSG00000211900.2	IGHJ6	5.212323026	31.3762941	4.20E-32
ENSG00000160224.16	AIRE	5.211250398	5.037040545	9.18E-06
ENSG00000187862.11	TTC24	5.209222077	20.27414523	5.32E-21
ENSG00000277829.1	AL031651.2	5.207762025	5.469359905	3.39E-06
ENSG00000211679.2	IGLC3	5.199551117	31.66253938	2.18E-32
ENSG00000177272.8	KCNA3	5.194925411	8.471206527	3.38E-09
ENSG00000224238.2	WARS2-IT1	5.192911712	6.144034819	7.18E-07
ENSG00000158089.14	GALNT14	5.190564132	18.58181128	2.62E-19
ENSG00000171903.16	CYP4F11	5.188935194	7.669160787	2.14E-08
ENSG00000242371.1	IGKV1-39	5.176506922	30.32873471	4.69E-31
ENSG00000244493.1	SLC9A9-AS2	5.176217759	18.91070556	1.23E-19
ENSG00000254709.7	IGLL5	5.176038804	30.64262843	2.28E-31
ENSG00000150337.13	FCGR1A	5.173905929	12.55015048	2.82E-13
ENSG00000161905.12	ALOX15	5.173273261	4.786272396	1.64E-05
ENSG00000168754.14	FAM178B	5.168478041	6.197692756	6.34E-07
ENSG00000211675.2	IGLC1	5.160345163	28.91147997	1.23E-29
ENSG00000221955.10	SLC12A8	5.159785529	5.57592337	2.66E-06
ENSG00000280020.1	AC116407.3	5.158461468	7.355008252	4.42E-08
ENSG00000186081.11	KRT5	5.151309967	4.503075372	3.14E-05
ENSG00000211677.2	IGLC2	5.147940319	40.46702966	3.41E-41
ENSG00000172548.14	NIPAL4	5.14468029	5.060411799	8.70E-06
ENSG00000254102.1	AC090136.3	5.133122417	6.243736867	5.71E-07
ENSG00000260719.1	AC009133.3	5.129511391	5.524097197	2.99E-06
ENSG00000234380.1	LINC01426	5.126585073	4.562842684	2.74E-05
ENSG00000144771.7	LRTM1	5.123184336	8.794563113	1.60E-09
ENSG00000261978.1	AC116025.1	5.107015652	6.697589763	2.01E-07
ENSG00000137691.12	CFAP300	5.105368949	5.484260517	3.28E-06
ENSG00000225434.2	LINC01504	5.102557921	14.38527793	4.12E-15
ENSG00000262823.1	AC127521.1	5.102217864	5.527745691	2.97E-06
ENSG00000211592.8	IGKC	5.101557581	35.96728983	1.08E-36
ENSG00000183813.6	CCR4	5.100052174	9.974549697	1.06E-10
ENSG00000126861.4	OMG	5.098786158	9.082472173	8.27E-10
ENSG00000232600.3	TONSL-AS1	5.09837556	8.201221379	6.29E-09
ENSG00000211644.3	IGLV1-51	5.095905532	20.42187472	3.79E-21
ENSG00000281990.1	IGHV1-69-2	5.093675377	1.646303435	0.022579
ENSG00000235290.1	HLA-W	5.093670423	9.805043934	1.57E-10
ENSG00000132704.15	FCRL2	5.085950784	2.644584183	0.002267
ENSG00000128040.10	SPINK2	5.085491331	6.700082723	1.99E-07
ENSG00000182566.13	CLEC4G	5.080504896	11.64122941	2.28E-12
ENSG00000211651.3	IGLV1-44	5.079913708	17.89904162	1.26E-18
ENSG00000137869.14	CYP19A1	5.079148008	6.733970632	1.85E-07
ENSG00000100739.10	BDKRB1	5.076712059	3.368220844	0.000428
ENSG00000250770.3	AC005865.2	5.068713556	6.059068896	8.73E-07

ENSG00000259342.1	AC025580.1	5.068204937	3.343396081	0.000454
ENSG00000171658.8	NMRAL2P	5.065931592	4.006071665	9.86E-05
ENSG00000211899.10	IGHM	5.056427814	35.06299171	8.65E-36
ENSG00000179869.14	ABCA13	5.056101969	3.651456785	0.000223
ENSG00000006606.8	CCL26	5.055585168	4.93171351	1.17E-05
ENSG00000266274.2	RN7SL138P	5.049737624	6.09000823	8.13E-07
ENSG00000240040.6	AC244205.1	5.047370661	34.94706272	1.13E-35
ENSG00000255335.1	AC124301.2	5.046016462	4.078670097	8.34E-05
ENSG00000211596.3	IGKJ2	5.045917466	34.88650889	1.30E-35
ENSG00000134594.4	RAB33A	5.03925547	4.924216317	1.19E-05
ENSG00000226872.1	AC002472.1	5.033639224	5.942177104	1.14E-06
ENSG00000215912.12	TTC34	5.030658123	6.671208626	2.13E-07
ENSG00000102174.8	PHEX	5.027938661	5.990886583	1.02E-06
ENSG00000205045.8	SLFN12L	5.027404201	10.68244389	2.08E-11
ENSG00000273824.1	AC008033.3	5.025679284	4.445133121	3.59E-05
ENSG00000260101.1	AC008074.2	5.025678916	7.292554851	5.10E-08
ENSG00000229694.6	LINC00484	5.02493116	6.05811296	8.75E-07
ENSG00000239819.2	IGKV1D-8	5.024292452	2.601931881	0.002501
ENSG00000211666.2	IGLV2-14	5.021279663	24.68578554	2.06E-25
ENSG00000168811.6	IL12A	5.019834704	5.949182495	1.12E-06
ENSG00000271851.1	AC087501.4	5.018037055	6.5863693	2.59E-07
ENSG00000271327.1	AC010201.2	5.017189978	4.88839533	1.29E-05
ENSG00000268896.1	AC009955.3	5.015961201	7.287400759	5.16E-08
ENSG00000226751.2	AF127936.1	5.013416335	5.376876667	4.20E-06
ENSG00000274767.1	AC243829.1	5.011063089	4.903514368	1.25E-05
ENSG00000188316.13	ENO4	5.004576257	5.35669369	4.40E-06
ENSG00000243264.2	IGKV2D-29	5.000469256	2.628193696	0.002354
ENSG00000214447.4	FAM187A	5.000148548	6.647209253	2.25E-07
ENSG00000284719.1	AL033527.5	4.998910022	6.601253018	2.50E-07
ENSG00000258945.1	AL049775.3	4.993871815	5.89310276	1.28E-06
ENSG00000148604.13	RGR	4.984845632	4.015793555	9.64E-05
ENSG00000211597.2	IGKJ1	4.979770668	34.68830384	2.05E-35
ENSG00000217801.9	AL390719.1	4.973935098	4.850782103	1.41E-05
ENSG00000242887.1	IGHJ3	4.973250436	22.32489593	4.73E-23
ENSG00000128045.6	RASL11B	4.971330502	9.221637991	6.00E-10
ENSG00000163705.12	FANCD2OS	4.970918973	7.981620265	1.04E-08
ENSG00000225383.7	SFTA1P	4.969892644	4.895959675	1.27E-05
ENSG00000174123.10	TLR10	4.963513857	4.359728478	4.37E-05
ENSG00000251002.7	AC244502.1	4.961869292	10.87863135	1.32E-11
ENSG00000151834.15	GABRA2	4.953827604	3.510793528	0.000308
ENSG00000007174.17	DNAH9	4.950897654	4.826417768	1.49E-05
ENSG00000256234.1	AC022509.2	4.947122338	5.915063838	1.22E-06
ENSG00000089356.18	FXYD3	4.934309272	6.911813683	1.23E-07
ENSG00000260082.1	AC106886.1	4.932343134	5.838258397	1.45E-06
ENSG00000211682.2	IGLJ6	4.932281251	2.890794459	0.001286
ENSG00000050730.15	TNIP3	4.930576253	4.828495429	1.48E-05
ENSG00000185610.6	DBX2	4.928678945	4.836834759	1.46E-05

ENSG00000224177.6	LINC00570	4.92761561	3.20582295	0.000623
ENSG00000243466.1	IGKV1-5	4.926847754	24.50283712	3.14E-25
ENSG00000222037.5	IGLC6	4.923018498	2.841473662	0.001441
ENSG00000206159.10	GYG2P1	4.920666767	3.882116633	0.000131
ENSG00000240041.1	IGHJ4	4.918244978	37.14791538	7.11E-38
ENSG00000211598.2	IGKV4-1	4.915366046	27.12617074	7.48E-28
ENSG00000265531.3	FCGR1CP	4.910735318	5.276683959	5.29E-06
ENSG00000271699.5	SNX29P2	4.906937502	4.29555204	5.06E-05
ENSG00000211716.2	TRBV9	4.905880636	2.807587506	0.001557
ENSG00000204165.5	CXorf65	4.901296193	3.904474862	0.000125
ENSG00000253842.1	AP003472.2	4.901196172	4.80363184	1.57E-05
ENSG00000181374.7	CCL13	4.899886521	31.65190609	2.23E-32
ENSG00000249628.3	LINC00942	4.899608928	6.361718572	4.35E-07
ENSG00000138755.5	CXCL9	4.896893942	40.29535723	5.07E-41
ENSG00000188011.5	RTP5	4.896883097	4.715093899	1.93E-05
ENSG00000174236.3	REP15	4.896512185	5.754438383	1.76E-06
ENSG00000233673.7	ANAPC1P1	4.895527562	4.701229289	1.99E-05
ENSG00000278196.3	IGLV2-8	4.888151655	15.22676144	5.93E-16
ENSG00000272463.1	AL357054.4	4.886745157	14.99198323	1.02E-15
ENSG00000282780.1	TRBJ1-6	4.884670847	3.142134283	0.000721
ENSG00000262151.1	AC133065.1	4.884138018	10.37970998	4.17E-11
ENSG00000224796.1	RPL32P1	4.883745414	4.24591411	5.68E-05
ENSG00000197134.11	ZNF257	4.877594914	3.536644268	0.000291
ENSG00000254238.1	AC100782.1	4.870595611	2.814084845	0.001534
ENSG00000225684.3	FAM225B	4.865895159	6.448651028	3.56E-07
ENSG00000130270.16	ATP8B3	4.865313112	11.48425111	3.28E-12
ENSG00000278949.1	AC127070.4	4.863819805	5.688098824	2.05E-06
ENSG00000272449.2	AL139246.5	4.862708994	4.720505017	1.90E-05
ENSG00000269050.1	AC011455.1	4.862564933	5.283217791	5.21E-06
ENSG00000279406.1	AL359183.1	4.86230033	4.281411944	5.23E-05
ENSG00000272282.1	LINC02084	4.859142218	3.470678538	0.000338
ENSG00000147378.11	FATE1	4.85772222	3.833970488	0.000147
ENSG00000211594.2	IGKJ4	4.856456281	33.91730507	1.21E-34
ENSG00000248278.1	SUMO2P17	4.855109078	5.194167612	6.39E-06
ENSG00000284685.1	AC009093.10	4.854463788	3.482400755	0.000329
ENSG00000152292.16	SH2D6	4.850216713	4.711618701	1.94E-05
ENSG00000273162.1	AL133215.2	4.849273546	5.764100431	1.72E-06
ENSG00000167912.5	AC090152.1	4.849065035	6.384676745	4.12E-07
ENSG00000275558.1	RN7SKP175	4.847016943	4.231995224	5.86E-05
ENSG00000254144.3	AC067930.1	4.847016943	4.231995224	5.86E-05
ENSG00000204653.9	ASPDH	4.846891857	4.652230932	2.23E-05
ENSG00000266692.1	RF00285	4.8455202	5.200889167	6.30E-06
ENSG00000105369.9	CD79A	4.844776383	12.20412929	6.25E-13
ENSG00000283440.1	LINC01260	4.843682473	3.463651626	0.000344
ENSG00000189238.5	LINC00943	4.830580483	4.683533586	2.07E-05
ENSG00000232869.2	TRBV29-1	4.825958971	3.836364455	0.000146
ENSG00000211764.1	TRBJ2-1	4.821560637	7.731382158	1.86E-08

ENSG00000246100.3	LINC00900	4.819912322	15.24547189	5.68E-16
ENSG00000223764.2	LINC02593	4.819482414	11.7584205	1.74E-12
ENSG00000259834.1	AL365361.1	4.816463519	12.61495894	2.43E-13
ENSG00000109832.13	DDX25	4.815102613	4.724390074	1.89E-05
ENSG00000244414.6	CFHR1	4.811965111	4.692424773	2.03E-05
ENSG00000134873.9	CLDN10	4.81116325	3.11519775	0.000767
ENSG00000213886.3	UBD	4.808126185	22.49087559	3.23E-23
ENSG00000231441.1	AL512422.1	4.80439525	5.102097839	7.91E-06
ENSG00000224429.7	LINC00539	4.797697618	5.158784473	6.94E-06
ENSG00000244457.2	ENO1P1	4.797419976	6.362553768	4.34E-07
ENSG00000228168.1	HNRNPA1P21	4.794654126	3.814064503	0.000153
ENSG00000271755.1	AL031118.1	4.791715497	5.162017347	6.89E-06
ENSG00000105219.9	CNTD2	4.790134356	3.79430895	0.000161
ENSG00000134545.13	KLRC1	4.782553031	3.75100143	0.000177
ENSG00000121594.11	CD80	4.781649678	3.418172916	0.000382
ENSG00000272159.1	AC087623.3	4.781194481	5.111593028	7.73E-06
ENSG00000278330.1	AC018529.2	4.776292841	4.194539763	6.39E-05
ENSG00000264031.1	ABHD15-AS1	4.776094103	4.587807537	2.58E-05
ENSG00000184731.5	FAM110C	4.766019569	3.76712411	0.000171
ENSG00000073734.9	ABCB11	4.763441314	2.713523106	0.001934
ENSG00000226005.4	AL513303.1	4.75715284	6.398014253	4.00E-07
ENSG00000255987.1	TOMM20P2	4.756606074	4.140150471	7.24E-05
ENSG00000265487.1	AP001021.1	4.751654879	5.125336077	7.49E-06
ENSG00000112799.8	LY86	4.748865797	16.34236659	4.55E-17
ENSG00000284977.1	AL160272.1	4.748594902	12.0899248	8.13E-13
ENSG00000211789.2	TRAV12-2	4.747864687	3.702939887	0.000198
ENSG00000259345.6	AC013652.1	4.747652914	2.977687588	0.001053
ENSG00000249476.1	AC008467.1	4.746682686	4.583212779	2.61E-05
ENSG00000211898.7	IGHD	4.746611237	4.298470269	5.03E-05
ENSG00000108576.9	SLC6A4	4.743145732	4.121570065	7.56E-05
ENSG00000227218.7	AL157935.1	4.739949044	8.076551615	8.38E-09
ENSG00000251039.2	IGKV2D-40	4.737885956	1.686241518	0.020595
ENSG00000116147.16	TNR	4.73692202	1.919367807	0.01204
ENSG00000249852.1	AC145676.1	4.734696627	3.338201485	0.000459
ENSG00000175779.2	C15orf53	4.73341405	11.94457742	1.14E-12
ENSG00000100122.6	CRYBB1	4.726254971	6.840827158	1.44E-07
ENSG00000169594.13	BNC1	4.724704499	4.590610289	2.57E-05
ENSG00000228325.5	IGKV3D-7	4.720269489	2.141868194	0.007213
ENSG00000284671.1	AC009093.9	4.708588389	8.844258338	1.43E-09
ENSG00000283821.1	MIR6875	4.707900263	2.982774329	0.00104
ENSG00000225079.2	FTH1P22	4.707765588	3.324958223	0.000473
ENSG00000224650.2	IGHV3-74	4.706737516	12.91328332	1.22E-13
ENSG00000272908.1	AC006033.2	4.7033566	3.692453598	0.000203
ENSG00000168748.13	CA7	4.702640515	2.682803775	0.002076
ENSG00000160791.13	CCR5	4.701773604	27.23783765	5.78E-28
ENSG00000163508.12	EOMES	4.698988068	11.00940691	9.79E-12
ENSG00000271553.1	AC018638.7	4.69525432	3.336906291	0.00046

ENSG00000115194.10	SLC30A3	4.690963016	2.707433218	0.001961
ENSG00000118004.17	COLEC11	4.686750779	2.408925957	0.0039
ENSG00000162746.14	FCRLB	4.686229947	3.740691746	0.000182
ENSG00000211895.5	IGHA1	4.670817509	33.64206846	2.28E-34
ENSG00000113302.4	IL12B	4.667189415	2.658825276	0.002194
ENSG00000169245.5	CXCL10	4.666904981	31.15817385	6.95E-32
ENSG00000147889.17	CDKN2A	4.666050656	22.71792753	1.91E-23
ENSG00000276232.1	AC006064.5	4.663934241	1.628972787	0.023498
ENSG00000239002.3	SCARNA10	4.663934241	1.628972787	0.023498
ENSG00000227803.2	AL158198.1	4.662948983	4.446835177	3.57E-05
ENSG00000269220.1	LINC00528	4.659390222	4.056051814	8.79E-05
ENSG00000205426.10	KRT81	4.658837368	4.03616852	9.20E-05
ENSG00000254295.1	AC008429.3	4.656344426	3.312134192	0.000487
ENSG00000276308.1	AC078880.4	4.655929217	2.937556964	0.001155
ENSG00000267791.1	AC018761.4	4.654002937	5.005578566	9.87E-06
ENSG00000248964.6	AC131254.1	4.64212834	2.360784186	0.004357
ENSG00000261143.1	ADAMTS7P3	4.641479434	3.649607842	0.000224
ENSG00000237988.5	OR2I1P	4.641401722	29.84688805	1.42E-30
ENSG00000257242.7	LINC01619	4.640630737	3.644397182	0.000227
ENSG00000225720.6	AL031846.1	4.638143536	3.970599404	0.000107
ENSG00000211721.2	TRBV6-5	4.635505345	2.628905019	0.00235
ENSG00000165874.13	SHLD2P1	4.633991344	3.608335482	0.000246
ENSG00000145242.13	EPHA5	4.630515812	1.412938721	0.038642
ENSG00000115919.14	KYNU	4.629611723	15.18862586	6.48E-16
ENSG00000253690.1	AC021678.2	4.628289931	2.587003427	0.002588
ENSG00000279369.1	AC046185.3	4.627429882	9.648071332	2.25E-10
ENSG00000226944.1	AL031847.1	4.625338454	4.977772221	1.05E-05
ENSG00000224592.5	AL139158.2	4.624153311	2.104304808	0.007865
ENSG00000273218.1	AC005776.2	4.623070246	4.920357881	1.20E-05
ENSG00000184524.5	CEND1	4.621593641	4.424524224	3.76E-05
ENSG00000102001.12	CACNA1F	4.619076637	4.434946994	3.67E-05
ENSG00000279479.1	AP005205.3	4.618144767	6.242296467	5.72E-07
ENSG00000149599.15	DUSP15	4.614548056	23.63638523	2.31E-24
ENSG00000180432.5	CYP8B1	4.613588394	2.330940288	0.004667
ENSG00000204044.6	SLC12A5-AS1	4.61271993	11.71485608	1.93E-12
ENSG00000279314.1	AC002525.1	4.611116203	3.618270989	0.000241
ENSG00000281103.1	TRG-AS1	4.609237269	11.39655252	4.01E-12
ENSG00000238000.1	AC116347.1	4.601143342	2.330940288	0.004667
ENSG00000230138.1	AC119428.2	4.601124451	2.913578787	0.00122
ENSG00000279332.1	AC090772.4	4.597599819	3.983111903	0.000104
ENSG00000158525.15	CPA5	4.596654699	3.233255297	0.000584
ENSG00000165591.6	FAAH2	4.590070087	4.385475569	4.12E-05
ENSG00000273691.1	AC087284.1	4.588101388	2.887934157	0.001294
ENSG00000200087.1	SNORA73B	4.584477785	2.570352325	0.002689
ENSG00000181847.11	TIGIT	4.579887964	11.06462691	8.62E-12
ENSG00000234174.1	AC016683.1	4.579175924	3.973787246	0.000106
ENSG00000238121.5	LINC00426	4.577453936	8.876488595	1.33E-09

ENSG00000233901.5	LINC01503	4.575568649	8.374417602	4.22E-09
ENSG00000215270.3	AP000523.1	4.574856351	2.904210359	0.001247
ENSG00000276953.1	TRBV12-4	4.572464634	2.309245388	0.004906
ENSG00000226608.3	FTLP3	4.572289975	4.427422737	3.74E-05
ENSG00000105255.10	FSD1	4.571581443	2.871556093	0.001344
ENSG00000238290.1	AL034417.2	4.563779841	3.222518424	0.000599
ENSG00000237990.3	CNTN4-AS1	4.558935794	2.898674574	0.001263
ENSG00000272666.1	U62317.1	4.558096758	2.586956695	0.002588
ENSG00000171951.4	SCG2	4.557587648	11.35900074	4.38E-12
ENSG00000225914.2	HCG23	4.553889986	2.306599533	0.004936
ENSG00000081148.11	IMPG2	4.553267171	7.87018159	1.35E-08
ENSG00000163734.4	CXCL3	4.552550728	2.545873873	0.002845
ENSG00000198491.3	AC007920.1	4.551050765	1.791152475	0.016175
ENSG00000211810.3	TRAV29DV5	4.550249508	1.817410246	0.015226
ENSG00000268947.1	AC002128.1	4.548426028	4.394647092	4.03E-05
ENSG00000111405.8	ENDOU	4.544948393	3.199662898	0.000631
ENSG00000243806.1	RPL7P18	4.540658311	2.56164836	0.002744
ENSG00000248632.1	AC106872.5	4.537818425	3.558000259	0.000277
ENSG00000229927.2	RHEBP1	4.529735276	2.853900266	0.0014
ENSG00000233609.3	RPL10P19	4.529590457	3.532160276	0.000294
ENSG00000254844.4	AP000757.2	4.529577112	9.412759319	3.87E-10
ENSG00000284649.1	AC009093.8	4.527239708	2.856423556	0.001392
ENSG00000236137.1	AL445231.1	4.520540366	3.876399605	0.000133
ENSG00000137078.8	SIT1	4.519559068	16.37441651	4.22E-17
ENSG00000162739.13	SLAMF6	4.515465088	11.72690808	1.88E-12
ENSG00000181333.11	HEPHL1	4.515439142	2.560474452	0.002751
ENSG00000087510.6	TFAP2C	4.508436167	3.536261908	0.000291
ENSG00000235947.1	EGOT	4.505076262	2.563188614	0.002734
ENSG00000204338.8	CYP21A1P	4.504743631	2.008675383	0.009802
ENSG00000231445.1	TIMM8AP1	4.501122812	1.364447721	0.043207
ENSG00000284523.1	AC004834.1	4.497902939	2.806438041	0.001562
ENSG00000249741.2	AC093890.1	4.497809972	3.180647765	0.00066
ENSG00000211689.7	TRGC1	4.491923222	2.825529878	0.001494
ENSG00000172460.16	PRSS30P	4.485451385	2.806760714	0.00156
ENSG00000122188.12	LAX1	4.484637103	12.7465419	1.79E-13
ENSG00000265743.1	AC138207.5	4.481182379	9.870242765	1.35E-10
ENSG00000202474.1	RNA5SP283	4.476970635	6.674603299	2.12E-07
ENSG00000239571.1	IGKV2D-30	4.472842149	2.516396006	0.003045
ENSG00000272872.1	AP000525.1	4.472321252	2.517401653	0.003038
ENSG00000110195.12	FOLR1	4.464580686	5.282609976	5.22E-06
ENSG00000146001.5	PCDHB18P	4.463267674	3.492763912	0.000322
ENSG00000164483.16	SAMD3	4.461693424	14.79824475	1.59E-15
ENSG00000204539.3	CDSN	4.460637287	1.547154679	0.028369
ENSG00000198535.5	C2CD4A	4.460354074	2.807082978	0.001559
ENSG00000284826.1	AC006518.7	4.452317775	3.498340687	0.000317
ENSG00000207332.1	RNU6-146P	4.450933668	10.99645744	1.01E-11
ENSG00000255571.8	MIR9-3HG	4.448557604	3.456007994	0.00035

ENSG00000135750.14	KCNK1	4.446928659	5.972517612	1.07E-06
ENSG00000156127.6	BATF	4.445017436	14.37070616	4.26E-15
ENSG00000175514.2	GPR152	4.444003227	3.867162025	0.000136
ENSG00000237484.5	LINC01684	4.442409792	2.79845882	0.001591
ENSG00000268743.1	AC008737.1	4.442310552	2.794136062	0.001606
ENSG00000124140.13	SLC12A5	4.439940727	2.772263468	0.001689
ENSG00000253183.1	AC005531.1	4.435600477	1.54094206	0.028778
ENSG00000244459.2	AC147067.1	4.435130379	2.792338074	0.001613
ENSG00000184293.7	CLECL1	4.435043745	2.474653698	0.003352
ENSG00000279155.1	AC233300.1	4.430959609	3.127987418	0.000745
ENSG00000132185.16	FCRLA	4.430350509	4.133704235	7.35E-05
ENSG00000180061.9	TMEM150B	4.427035308	13.95981032	1.10E-14
ENSG00000214376.5	VSTM5	4.426992183	2.797431936	0.001594
ENSG00000225978.3	HAR1A	4.426465966	2.783084976	0.001648
ENSG00000109705.7	NKX3-2	4.425661851	1.532929061	0.029314
ENSG00000239862.1	IGKV1-37	4.425581965	1.723751335	0.018891
ENSG00000073737.16	DHRS9	4.4215202	16.13639638	7.30E-17
ENSG00000243795.1	LINC02044	4.418872662	3.760633438	0.000174
ENSG00000139572.3	GPR84	4.414510455	2.771306713	0.001693
ENSG00000276443.1	RF02164	4.414364455	3.459103852	0.000347
ENSG00000238107.1	AC245100.6	4.413473382	3.392109786	0.000405
ENSG00000180535.3	BHLHA15	4.412269883	2.207722541	0.006198
ENSG00000260517.3	AC009093.2	4.412079777	1.726929812	0.018753
ENSG00000273650.1	AC100793.4	4.409623208	1.958622269	0.011
ENSG00000178934.4	LGALS7B	4.406980264	1.725629683	0.018809
ENSG00000253120.1	IGLV2-34	4.401615027	1.724732856	0.018848
ENSG00000274845.1	RF02271	4.400045547	11.63380102	2.32E-12
ENSG00000258810.1	AL133371.2	4.398810583	3.072765009	0.000846
ENSG00000259088.1	AL137779.2	4.396749499	3.423424754	0.000377
ENSG00000160856.20	FCRL3	4.396004494	6.905980353	1.24E-07
ENSG00000285877.1	AC007448.4	4.392805807	3.068669217	0.000854
ENSG00000255641.1	AC068775.1	4.391619478	2.446597955	0.003576
ENSG00000196468.7	FGF16	4.390436226	6.751319625	1.77E-07
ENSG00000188820.12	CALHM6	4.389996954	13.98716076	1.03E-14
ENSG00000144278.14	GALNT13	4.388073645	3.472241885	0.000337
ENSG00000176406.22	RIMS2	4.387955944	3.05243318	0.000886
ENSG00000181908.5	AP003774.1	4.387123369	3.067846927	0.000855
ENSG00000211893.4	IGHG2	4.387027053	31.39427598	4.03E-32
ENSG00000224864.4	AC011447.1	4.385609723	3.059913284	0.000871
ENSG00000064886.13	CHI3L2	4.37654478	10.38446486	4.13E-11
ENSG00000272808.4	AC015712.6	4.37608267	3.401595417	0.000397
ENSG00000163568.14	AIM2	4.375427181	10.69417935	2.02E-11
ENSG00000249740.2	OSMR-AS1	4.374911102	2.453335848	0.003521
ENSG00000279879.1	AC091152.4	4.374885218	6.351052919	4.46E-07
ENSG00000270379.5	HEATR9	4.372801792	3.031835899	0.000929
ENSG00000161180.10	CCDC116	4.368175356	2.724166943	0.001887
ENSG00000285080.1	AC068724.3	4.366121134	3.379001061	0.000418

ENSG00000204687.4	MAS1L	4.366043664	3.073666959	0.000844
ENSG00000263293.2	THCAT158	4.36500125	1.939741575	0.011488
ENSG00000273272.1	U62317.4	4.364535421	2.442963239	0.003606
ENSG00000242615.1	AC022415.1	4.362522817	7.695016201	2.02E-08
ENSG00000227053.1	AC105446.1	4.362236865	2.173945579	0.0067
ENSG00000211968.3	IGHV1-58	4.360731922	1.93309916	0.011665
ENSG00000211791.2	TRAV13-2	4.360039464	1.925194883	0.01188
ENSG00000260588.1	AC027702.1	4.35029329	3.76021673	0.000174
ENSG00000123500.9	COL10A1	4.350278797	1.689426111	0.020444
ENSG00000234362.5	LINC01914	4.349689525	2.164748104	0.006843
ENSG00000200831.1	SNORD36B	4.34902079	3.029413496	0.000935
ENSG00000205076.4	LGALS7	4.345461673	1.701140665	0.0199
ENSG00000185904.11	LINC00839	4.34483439	2.166823342	0.00681
ENSG00000223725.6	AC009226.1	4.343757354	3.015638949	0.000965
ENSG00000235385.1	LINC02154	4.343567234	2.688607059	0.002048
ENSG00000236849.5	LINC01474	4.342938665	2.682481186	0.002077
ENSG00000247317.3	LY6E-DT	4.339565179	1.920199743	0.012017
ENSG00000213996.12	TM6SF2	4.339361538	3.019066398	0.000957
ENSG00000211771.1	TRBJ2-7	4.338832102	8.40907503	3.90E-09
ENSG00000226660.2	TRBV2	4.336652527	1.896398528	0.012694
ENSG00000285090.1	AC002074.1	4.33181848	1.907034991	0.012387
ENSG00000254872.3	AC139749.1	4.330255367	2.411328537	0.003879
ENSG00000187867.8	PALM3	4.327030552	2.503811539	0.003135
ENSG00000211890.4	IGHA2	4.321502516	16.0699242	8.51E-17
ENSG00000188610.12	FAM72B	4.321276331	6.356709214	4.40E-07
ENSG00000261065.1	AL592146.1	4.317320973	4.85230451	1.41E-05
ENSG00000283063.1	TRBV6-2	4.317094126	1.671417813	0.02131
ENSG00000257824.1	AC068789.1	4.313836977	11.01905912	9.57E-12
ENSG00000088053.11	GP6	4.311771486	2.993927777	0.001014
ENSG00000197935.6	ZNF311	4.308451508	6.479836726	3.31E-07
ENSG00000277710.2	NBEAP5	4.308427827	1.900859379	0.012564
ENSG00000259807.1	AC009093.1	4.308387592	1.471477272	0.033769
ENSG00000231784.8	DBIL5P	4.307206454	3.329718215	0.000468
ENSG00000285062.1	AC245100.9	4.305205825	7.75696347	1.75E-08
ENSG00000070915.9	SLC12A3	4.305032425	2.388332112	0.004089
ENSG00000111262.6	KCNA1	4.300050898	1.671018997	0.02133
ENSG00000280303.2	ERICD	4.299237071	3.742143063	0.000181
ENSG00000248787.2	AC092903.2	4.298544102	2.132859088	0.007364
ENSG00000151025.10	GPR158	4.298148787	2.682803775	0.002076
ENSG00000255847.5	AP003717.1	4.293020583	2.676254283	0.002107
ENSG00000116824.4	CD2	4.292278866	16.94913686	1.12E-17
ENSG00000253669.3	GASAL1	4.286542741	5.502423605	3.14E-06
ENSG00000182557.7	SPNS3	4.284041033	6.367758451	4.29E-07
ENSG00000179299.16	NSUN7	4.273235033	2.380123276	0.004168
ENSG00000201134.1	RF00019	4.270805552	2.371689653	0.004249
ENSG00000204277.1	LINC01993	4.270275732	2.110632734	0.007751
ENSG00000121101.15	TEX14	4.268357903	2.380123276	0.004168

ENSG00000102271.13	KLHL4	4.267120421	12.33713663	4.60E-13
ENSG00000145649.7	GZMA	4.26097196	14.48522483	3.27E-15
ENSG00000209582.1	SNORA48	4.260016605	2.942977277	0.00114
ENSG00000225655.5	BX255923.1	4.259673376	6.106376721	7.83E-07
ENSG00000237461.1	AL359710.1	4.259506436	2.63025354	0.002343
ENSG00000211788.2	TRAV13-1	4.259073864	1.852446404	0.014046
ENSG00000242611.2	AC093627.6	4.257269792	3.194200714	0.000639
ENSG00000211797.2	TRAV17	4.256376118	2.350745789	0.004459
ENSG00000237702.2	TRBV3-1	4.253784015	1.867293382	0.013574
ENSG00000166035.10	LIPC	4.252155361	4.41972439	3.80E-05
ENSG00000259772.6	AC012236.1	4.249419146	2.092033579	0.00809
ENSG00000128438.10	TBC1D27P	4.246159497	2.087300174	0.008179
ENSG00000256897.1	AC018410.2	4.24172645	3.256251191	0.000554
ENSG00000223652.2	AC106786.1	4.233312995	2.6323415	0.002332
ENSG00000261036.1	AC113418.1	4.23282781	2.088499275	0.008156
ENSG00000163606.10	CD200R1	4.228892328	13.31242512	4.87E-14
ENSG00000181036.13	FCRL6	4.228770404	13.46946975	3.39E-14
ENSG00000227155.7	AL161725.1	4.226223813	2.897402312	0.001266
ENSG00000224215.1	AL606469.1	4.225896447	2.081230989	0.008294
ENSG00000057593.13	F7	4.225838471	2.949209023	0.001124
ENSG00000163564.14	PYHIN1	4.224751306	10.41433269	3.85E-11
ENSG00000058335.15	RASGRF1	4.223379135	2.086828461	0.008188
ENSG00000227925.1	LINC01655	4.220978892	2.331752688	0.004659
ENSG00000259347.6	AC087482.1	4.21854365	2.082610771	0.008268
ENSG00000160654.10	CD3G	4.217661338	9.89420415	1.28E-10
ENSG00000213424.8	KRT222	4.216878124	6.019685932	9.56E-07
ENSG00000159374.17	M1AP	4.215524571	3.250235647	0.000562
ENSG00000270670.1	MARK2P16	4.213495784	2.338800709	0.004584
ENSG00000197177.15	ADGRA1	4.212393851	1.838372446	0.014509
ENSG00000261265.1	AC025271.2	4.210175451	3.712011111	0.000194
ENSG00000235724.8	AC009299.3	4.208214536	2.884798386	0.001304
ENSG00000211829.9	TRDC	4.205712711	7.321561837	4.77E-08
ENSG00000161653.10	NAGS	4.205557793	7.366799641	4.30E-08
ENSG00000100985.7	MMP9	4.205000843	14.22481846	5.96E-15
ENSG00000158477.6	CD1A	4.198400803	2.565346497	0.002721
ENSG00000163121.9	NEURL3	4.196956658	2.602327232	0.002498
ENSG00000214776.12	AC092821.1	4.196851719	2.621356207	0.002391
ENSG00000237520.1	AL391832.1	4.196626645	2.925307351	0.001188
ENSG00000181408.3	UTS2R	4.190285891	12.59692801	2.53E-13
ENSG00000152910.18	CNTNAP4	4.188270482	6.616406679	2.42E-07
ENSG00000186766.7	FOXI2	4.186693655	6.321152199	4.77E-07
ENSG00000261468.1	AC096921.2	4.182357275	4.415699958	3.84E-05
ENSG00000251081.1	AC104663.1	4.181015107	1.411045605	0.038811
ENSG00000172116.21	CD8B	4.179506204	10.53187527	2.94E-11
ENSG00000278404.1	RF01874	4.177802363	2.035545759	0.009214
ENSG00000268357.1	VN1R81P	4.176677548	3.185294081	0.000653
ENSG00000225137.1	DYNC1I2P1	4.176484285	2.896183597	0.00127

ENSG00000261656.5	BEAN1-AS1	4.175947393	1.818684101	0.015182
ENSG00000137747.15	TMPRSS13	4.175582463	4.39210196	4.05E-05
ENSG00000006555.10	TTC22	4.171345156	4.776344678	1.67E-05
ENSG00000260166.1	AC134312.3	4.170937125	2.04236702	0.009071
ENSG00000261335.1	AC005837.1	4.169467081	2.580320222	0.002628
ENSG00000235770.5	LINC00607	4.16820725	2.049414698	0.008925
ENSG00000118194.18	TNNT2	4.163528556	34.20884798	6.18E-35
ENSG00000168421.12	RHOH	4.161963776	9.70834586	1.96E-10
ENSG00000179750.15	APOBEC3B	4.161665003	4.221573585	6.00E-05
ENSG00000074706.13	IPCEF1	4.161003632	12.98172125	1.04E-13
ENSG00000103522.15	IL21R	4.158788781	12.34457181	4.52E-13
ENSG00000232504.4	ST3GAL5-AS1	4.158718877	1.594514361	0.025438
ENSG00000099960.12	SLC7A4	4.158311451	7.367329211	4.29E-08
ENSG00000260645.2	AL359715.2	4.15774069	2.298360065	0.005031
ENSG00000178199.13	ZC3H12D	4.157575217	12.97610369	1.06E-13
ENSG00000285954.1	AC119428.3	4.156714913	2.033967002	0.009248
ENSG00000213023.10	SYT3	4.155051688	1.588465932	0.025795
ENSG00000205784.2	ARRDC5	4.154491744	1.807508681	0.015577
ENSG00000268262.1	AC011445.1	4.151205149	2.560844575	0.002749
ENSG00000242258.1	LINC00996	4.151001793	2.027042511	0.009396
ENSG00000179902.12	C1orf194	4.14869178	1.803470022	0.015723
ENSG00000124159.15	MATN4	4.148616663	2.58882941	0.002577
ENSG00000246898.1	LINC00920	4.145202537	1.797086298	0.015956
ENSG00000275152.4	CCL16	4.144468877	1.79845223	0.015906
ENSG00000177455.12	CD19	4.143570738	2.035534614	0.009214
ENSG00000277156.1	AL589743.5	4.143205292	2.027057235	0.009396
ENSG00000173391.8	OLR1	4.142588938	1.585953619	0.025945
ENSG00000274678.1	AC106886.3	4.133532941	2.533068791	0.00293
ENSG00000231528.2	FAM225A	4.131978477	2.27669327	0.005288
ENSG00000142959.4	BEST4	4.128242006	2.003485023	0.00992
ENSG00000250893.1	AC098869.2	4.12576799	1.790855366	0.016186
ENSG00000259655.1	AC090825.2	4.122428618	2.274319452	0.005317
ENSG00000179331.2	RAB39A	4.122130776	2.27406362	0.00532
ENSG00000133048.12	CHI3L1	4.116568483	16.11985828	7.59E-17
ENSG00000130751.9	NPAS1	4.115991009	2.535350086	0.002915
ENSG00000181634.7	TNFSF15	4.114676728	3.803555355	0.000157
ENSG00000184860.9	SDR42E1	4.113765136	1.560281352	0.027524
ENSG00000160200.17	CBS	4.111967712	1.788619584	0.01627
ENSG00000124203.6	ZNF831	4.111389754	11.4467377	3.57E-12
ENSG00000226491.1	FTOP1	4.110384865	2.292158603	0.005103
ENSG00000227508.6	LINC01624	4.109962499	2.282388729	0.005219
ENSG00000280132.1	AC026471.6	4.109252779	2.008025053	0.009817
ENSG00000169495.4	HTRA4	4.108483082	1.778643801	0.016648
ENSG00000268729.1	AC020922.2	4.107946557	1.984395403	0.010366
ENSG00000235499.1	AC073046.1	4.107439143	2.831274808	0.001475
ENSG00000258955.1	LINC00519	4.106803313	2.01612582	0.009635
ENSG00000264230.8	ANXA8L1	4.105044343	1.963222177	0.010884

ENSG00000204403.9	CASP12	4.104260424	2.258726796	0.005512
ENSG00000250174.5	MYLK-AS2	4.103627313	2.264904348	0.005434
ENSG00000183837.9	PNMA3	4.103174184	12.08518904	8.22E-13
ENSG00000182612.10	TSPAN10	4.102070104	6.313891508	4.85E-07
ENSG00000275559.1	RF02110	4.101057062	2.244924127	0.00569
ENSG00000232320.6	AC009299.2	4.100307311	1.991116481	0.010207
ENSG00000282133.1	TRBJ1-3	4.098646443	1.763412836	0.017242
ENSG00000233902.1	AL645933.1	4.097353958	23.82369098	1.50E-24
ENSG00000269072.1	AC063977.6	4.089256435	1.984395403	0.010366
ENSG00000187758.7	ADH1A	4.087805956	3.892708395	0.000128
ENSG00000079263.18	SP140	4.083010234	12.20097399	6.30E-13
ENSG00000140284.10	SLC27A2	4.082933068	1.984619004	0.010361
ENSG00000253250.2	C8orf88	4.078646795	7.689102641	2.05E-08
ENSG00000261504.1	LINC01686	4.077470648	2.233450945	0.005842
ENSG00000253123.3	AC091182.1	4.076465009	12.9829617	1.04E-13
ENSG00000197191.4	CYSRT1	4.076164679	5.57931475	2.63E-06
ENSG00000267453.7	LINC01835	4.074825509	1.986339876	0.01032
ENSG00000158816.15	VWA5B1	4.074794353	3.793277019	0.000161
ENSG00000144406.18	UNC80	4.072579943	2.466774946	0.003414
ENSG00000205584.6	AC211476.1	4.07117684	1.751760928	0.017711
ENSG00000211794.3	TRAV12-3	4.064994583	1.353840918	0.044275
ENSG00000198417.6	MT1F	4.064241571	8.387257141	4.10E-09
ENSG00000255306.1	AC004923.4	4.062917345	2.214229247	0.006106
ENSG00000271590.1	AC108463.3	4.062725663	1.980509245	0.010459
ENSG00000140527.14	WDR93	4.061659293	1.541854423	0.028717
ENSG00000177688.6	SUMO4	4.058084112	2.217803155	0.006056
ENSG00000160185.14	UBASH3A	4.056006135	7.026231849	9.41E-08
ENSG00000170498.8	KISS1	4.055899637	1.343801991	0.04531
ENSG00000204475.9	NCR3	4.05141559	6.475675253	3.34E-07
ENSG00000146250.6	PRSS35	4.049796467	1.354154893	0.044243
ENSG00000105550.9	FGF21	4.047991228	2.564166445	0.002728
ENSG00000229400.1	AL596330.1	4.047731129	2.494229568	0.003205
ENSG00000277583.1	RF02180	4.047588852	2.764737686	0.001719
ENSG00000102245.7	CD40LG	4.040469818	4.74605016	1.79E-05
ENSG00000109625.18	CPZ	4.039015368	9.961013042	1.09E-10
ENSG00000225402.1	AC010878.1	4.037882955	1.529433712	0.029551
ENSG00000273213.2	AC239798.4	4.037219129	1.533432117	0.02928
ENSG00000231767.4	AL136454.1	4.036299875	1.726579027	0.018768
ENSG00000135898.9	GPR55	4.035814636	1.955009502	0.011092
ENSG00000259444.1	AL161669.1	4.035643219	1.527025808	0.029715
ENSG00000133477.16	FAM83F	4.03526844	2.463675647	0.003438
ENSG00000122824.10	NUDT10	4.03510273	1.958300032	0.011008
ENSG00000134258.16	VTCN1	4.033898039	1.339605479	0.04575
ENSG00000211753.4	TRBV28	4.033332175	2.066472711	0.008581
ENSG00000240929.2	HIST2H2BB	4.032149494	1.740752186	0.018166
ENSG00000248322.1	AL117348.1	4.030792708	1.333684172	0.046378
ENSG00000229474.6	PATL2	4.028893092	15.06768938	8.56E-16

ENSG00000122859.4	NEUROG3	4.02744889	1.527025808	0.029715
ENSG00000198576.3	ARC	4.025759444	6.577518235	2.65E-07
ENSG00000085552.16	IGSF9	4.024405182	2.19170376	0.006431
ENSG00000241163.7	LINC00877	4.023774774	1.727957624	0.018709
ENSG00000205622.9	AP001043.1	4.023652005	3.502377203	0.000315
ENSG00000155961.4	RAB39B	4.022826756	5.070296041	8.51E-06
ENSG00000092200.12	RPGRIP1	4.020030124	4.540840319	2.88E-05
ENSG00000211769.1	TRBJ2-5	4.017251132	5.350204561	4.46E-06
ENSG00000237356.6	AL365295.1	4.016668369	2.479122885	0.003318
ENSG00000258691.1	AL355102.2	4.013763028	8.82042122	1.51E-09
ENSG00000258352.1	AC119044.1	4.012005847	1.948848246	0.01125
ENSG00000102970.10	CCL17	4.012005267	1.511339961	0.030808
ENSG00000280417.1	AC096887.2	4.010992887	2.44912767	0.003555
ENSG00000232063.1	AL691447.2	4.007592439	1.7179012	0.019147
ENSG00000073756.11	PTGS2	4.004763616	2.465696631	0.003422
ENSG00000122223.12	CD244	4.003354836	7.87126755	1.35E-08
ENSG00000131019.10	ULBP3	4.002515287	2.189466821	0.006464
ENSG00000228252.9	COL6A4P2	4.00104554	2.168588846	0.006783
ENSG00000124664.10	SPDEF	3.998807315	1.925523615	0.011871
ENSG00000256660.5	CLEC12B	3.997751931	1.323809217	0.047445
ENSG00000254418.1	SPON1-AS1	3.997508402	2.365771152	0.004308
ENSG00000274667.1	AC090517.2	3.99529377	1.932850004	0.011672
ENSG00000167094.15	TTC16	3.993848142	6.043490333	9.05E-07
ENSG00000136244.11	IL6	3.993436365	1.500123361	0.031614
ENSG00000187123.14	LYPD6	3.992964908	1.711905955	0.019413
ENSG00000125409.12	TEKT3	3.992143878	1.715158707	0.019268
ENSG00000258479.5	LINC00640	3.991650373	1.946265049	0.011317
ENSG00000256713.7	PGA5	3.991440754	1.693755122	0.020242
ENSG00000182255.6	KCNA4	3.990225959	1.499463225	0.031662
ENSG00000255968.1	AC024145.1	3.987485642	2.161456801	0.006895
ENSG00000234779.1	BNC2-AS1	3.984807157	1.7047216	0.019737
ENSG00000265485.6	LINC01915	3.983998648	5.324162888	4.74E-06
ENSG00000211776.2	TRAV2	3.98371038	1.922111381	0.011964
ENSG00000211825.1	TRDJ1	3.977522734	1.696248273	0.020126
ENSG00000124116.18	WFDC3	3.976887255	4.697196751	2.01E-05
ENSG00000224003.1	YES1P1	3.975159221	1.496400074	0.031886
ENSG00000141433.12	ADCYAP1	3.973407781	3.09679695	0.0008
ENSG00000261193.1	AC134312.4	3.971917885	1.502582286	0.031435
ENSG00000155269.11	GPR78	3.971237607	9.651918629	2.23E-10
ENSG00000211672.2	IGLV4-3	3.97071376	1.307974042	0.049207
ENSG00000211699.2	TRGV3	3.969375936	1.908162208	0.012355
ENSG00000165566.12	AMER2	3.968417547	1.485703982	0.032681
ENSG00000280157.1	AL359510.2	3.968235837	1.921521605	0.011981
ENSG00000283795.1	MIR4426	3.967160914	1.683188604	0.02074
ENSG00000205562.2	AL049775.1	3.964342445	6.649418578	2.24E-07
ENSG00000249096.6	LINC02362	3.96341828	3.703362861	0.000198
ENSG00000198963.10	RORB	3.961334506	3.087351734	0.000818

ENSG00000285906.1	AC083855.2	3.960389383	1.498407213	0.031739
ENSG00000269486.2	ERVK9-11	3.95992008	1.9082313	0.012353
ENSG00000071909.18	MYO3B	3.958058404	1.908963249	0.012332
ENSG00000145244.11	CORIN	3.956305743	1.304669962	0.049583
ENSG00000285417.1	BX571818.1	3.955959444	1.897736609	0.012655
ENSG00000116299.16	KIAA1324	3.950233721	9.432089319	3.70E-10
ENSG00000261678.2	SCRT1	3.949321252	2.123182089	0.00753
ENSG00000166448.14	TMEM130	3.949072525	1.482598019	0.032916
ENSG00000154451.14	GBP5	3.94858831	29.55614702	2.78E-30
ENSG00000253882.6	AC099548.2	3.948005903	5.071092157	8.49E-06
ENSG00000233058.1	LINC00884	3.945771738	1.681427755	0.020824
ENSG00000103241.6	FOXF1	3.945543897	1.683001878	0.020749
ENSG00000171954.12	CYP4F22	3.945146068	3.704125382	0.000198
ENSG00000259006.1	AC092143.2	3.941285828	6.998365015	1.00E-07
ENSG00000233967.6	AL359715.1	3.941042134	1.477729067	0.033287
ENSG00000278977.1	AC003101.3	3.939808605	1.896587922	0.012689
ENSG00000213967.10	ZNF726	3.937565603	1.91245087	0.012233
ENSG00000269035.1	AC010319.2	3.937171493	2.153105127	0.007029
ENSG00000277406.2	SEC22B4P	3.933728615	5.051187931	8.89E-06
ENSG00000137265.14	IRF4	3.932749401	15.7347698	1.84E-16
ENSG00000142677.3	IL22RA1	3.92667995	4.608765957	2.46E-05
ENSG00000259343.6	TMC3-AS1	3.924136126	1.665703188	0.021592
ENSG00000251623.2	AC139491.5	3.921996954	2.109257727	0.007776
ENSG00000183439.7	TRIM61	3.921956191	1.895109205	0.012732
ENSG00000245522.2	AC026250.1	3.920630148	1.884885474	0.013035
ENSG00000276972.1	AC078880.5	3.920289457	1.472286562	0.033706
ENSG00000138135.6	CH25H	3.91978394	1.463017233	0.034434
ENSG00000261997.1	AC007336.1	3.913071265	1.658139986	0.021972
ENSG00000236411.1	NDUFAF4P3	3.909670624	1.66645497	0.021555
ENSG00000189120.4	SP6	3.908530886	2.10159923	0.007914
ENSG00000277678.1	RF00003	3.904387468	4.792647542	1.61E-05
ENSG00000231106.2	LINC01436	3.903544641	1.935761762	0.011594
ENSG00000259426.5	AC027237.3	3.903262525	1.663262755	0.021714
ENSG00000182950.2	ODF3L1	3.902244287	2.109765862	0.007767
ENSG00000099822.2	HCN2	3.900474622	3.964668116	0.000108
ENSG00000223345.3	HIST2H2BA	3.894832255	1.655159438	0.022123
ENSG00000117069.14	ST6GALNAC5	3.894268999	1.859452184	0.013821
ENSG00000226091.7	LINC00937	3.893680898	3.865165088	0.000136
ENSG00000147724.11	FAM135B	3.89181345	5.930217877	1.17E-06
ENSG00000279611.1	AC012313.10	3.890657956	5.42426516	3.76E-06
ENSG00000267551.3	AC005264.1	3.889635899	8.042436982	9.07E-09
ENSG00000279965.1	AL390755.1	3.887860209	5.507029928	3.11E-06
ENSG00000163519.13	TRAT1	3.880258616	5.56487564	2.72E-06
ENSG00000231407.5	AL354732.1	3.879722972	1.652264927	0.022271
ENSG00000270933.1	AC010719.1	3.879671072	1.447515087	0.035685
ENSG00000134242.15	PTPN22	3.877731903	12.55217529	2.80E-13
ENSG00000096996.15	IL12RB1	3.875634997	15.50203583	3.15E-16

ENSG00000272701.3	MESTIT1	3.872433094	1.646915248	0.022547
ENSG00000122877.15	EGR2	3.870644655	13.2630796	5.46E-14
ENSG00000100450.12	GZMH	3.869141093	11.91359017	1.22E-12
ENSG00000280173.1	AC104447.1	3.868509343	4.693116356	2.03E-05
ENSG00000278743.1	AC087239.1	3.868037481	2.081592484	0.008287
ENSG00000117400.17	MPL	3.865425478	1.638228218	0.023002
ENSG00000227906.7	SNAP25-AS1	3.864682245	1.8411443	0.014416
ENSG00000153976.2	HS3ST3A1	3.864175231	3.688798495	0.000205
ENSG00000254827.5	SLC22A18AS	3.860395347	6.041751602	9.08E-07
ENSG00000153563.15	CD8A	3.859268425	20.84723332	1.42E-21
ENSG00000264452.1	RF00285	3.858247769	3.609437852	0.000246
ENSG00000196632.10	WNK3	3.857964628	4.223206363	5.98E-05
ENSG00000272092.1	AC087623.2	3.856512694	2.077448001	0.008367
ENSG00000212153.1	RNU1-82P	3.855317115	1.857572412	0.013881
ENSG00000256340.8	ABCC6P1	3.854841105	1.84689898	0.014227
ENSG00000137819.13	PAQR5	3.853564605	2.859664246	0.001381
ENSG00000167077.12	MEI1	3.850467503	12.76625145	1.71E-13
ENSG00000175894.16	TSPEAR	3.843826727	1.437206968	0.036542
ENSG00000236733.1	AL354794.1	3.842340249	1.609152607	0.024595
ENSG00000169313.9	P2RY12	3.835592871	8.402528464	3.96E-09
ENSG00000243250.1	RPS6P16	3.834285201	1.828617206	0.014838
ENSG00000149527.17	PLCH2	3.832147521	10.70888544	1.95E-11
ENSG00000202343.1	RF00410	3.830553871	1.614122947	0.024315
ENSG00000257243.1	AC020612.1	3.830215905	2.060014879	0.008709
ENSG00000269161.1	AC010618.2	3.827761425	1.605124087	0.024824
ENSG00000170396.7	ZNF804A	3.820321062	1.606344049	0.024755
ENSG00000062524.15	LTK	3.818330487	11.47659432	3.34E-12
ENSG00000170927.14	PKHD1	3.817756709	4.465238797	3.43E-05
ENSG00000134256.12	CD101	3.817533856	7.170705173	6.75E-08
ENSG00000259954.1	IL21R-AS1	3.815612293	9.999238615	1.00E-10
ENSG00000186818.12	LILRB4	3.814539871	16.53299595	2.93E-17
ENSG00000254760.1	AC008750.1	3.811038248	1.410379169	0.038871
ENSG00000114013.15	CD86	3.810930072	13.52654762	2.97E-14
ENSG00000133101.9	CCNA1	3.808874617	3.702662223	0.000198
ENSG00000260135.6	MMP2-AS1	3.804534396	2.732588143	0.001851
ENSG00000165923.15	AGBL2	3.803429315	1.597225629	0.02528
ENSG00000155886.11	SLC24A2	3.802857851	6.751913028	1.77E-07
ENSG00000267069.1	AP005264.1	3.801575526	1.409512713	0.038948
ENSG00000226287.8	TMEM191A	3.799640821	3.232934145	0.000585
ENSG00000233952.1	FTLP15	3.79671291	1.401877262	0.039639
ENSG00000106952.7	TNFSF8	3.795456419	11.18777326	6.49E-12
ENSG00000189045.13	ANKDD1B	3.79500095	1.595484189	0.025381
ENSG00000111796.3	KLRB1	3.794097768	5.973794619	1.06E-06
ENSG00000248483.6	POU5F2	3.791832244	1.402217488	0.039608
ENSG00000232310.7	AL078590.3	3.788660988	1.586797055	0.025894
ENSG00000244676.5	AL109761.1	3.786993214	4.454850672	3.51E-05
ENSG00000232725.1	U52111.1	3.782446131	1.400322335	0.039781

ENSG00000265874.1	MIR4489	3.782293385	1.585953619	0.025945
ENSG00000185972.5	CCIN	3.780575685	1.398182601	0.039978
ENSG00000101349.16	PAK5	3.777994069	1.398570946	0.039942
ENSG00000101280.7	ANGPT4	3.777685245	1.392540252	0.0405
ENSG00000231305.3	AC112484.1	3.776881121	1.586797055	0.025894
ENSG00000233718.7	MYCNOS	3.775892653	4.646660761	2.26E-05
ENSG00000168995.13	SIGLEC7	3.775566742	4.219813711	6.03E-05
ENSG00000196366.3	C9orf163	3.774893043	1.582727214	0.026138
ENSG00000149571.11	KIRREL3	3.774702198	3.439491035	0.000364
ENSG00000257042.1	AC008011.2	3.774423199	5.743793859	1.80E-06
ENSG00000101440.9	ASIP	3.773893444	1.384535756	0.041254
ENSG00000266538.1	AC005838.2	3.773375057	1.576276493	0.026529
ENSG00000225210.10	DUXAP9	3.771146545	6.050081521	8.91E-07
ENSG00000284585.1	MIR4722	3.769059514	1.576720047	0.026502
ENSG00000174776.11	WDR49	3.765329382	1.387052516	0.041015
ENSG00000231752.6	EMBP1	3.765204448	3.175158288	0.000668
ENSG00000237989.1	LINC01679	3.764556395	9.986323616	1.03E-10
ENSG00000142233.11	NTN5	3.764135104	3.760314245	0.000174
ENSG00000285525.1	AC099670.3	3.760082751	1.380221693	0.041666
ENSG00000136573.13	BLK	3.759303204	3.125450332	0.000749
ENSG00000179141.9	MTUS2-AS1	3.756567538	1.564610188	0.027251
ENSG00000135114.12	OASL	3.755408324	14.74167717	1.81E-15
ENSG00000164621.5	SMAD5-AS1	3.755036622	1.783695589	0.016455
ENSG00000248799.1	AC022118.1	3.754504853	1.562906455	0.027359
ENSG00000198354.6	DCAF12L2	3.753528804	4.212265975	6.13E-05
ENSG00000261420.1	AL022069.1	3.753110924	1.775351241	0.016774
ENSG00000089012.14	SIRPG	3.748827316	4.69296614	2.03E-05
ENSG00000171320.14	ESCO2	3.745324744	1.999248889	0.010017
ENSG00000272551.1	AC017048.3	3.74478728	1.572382346	0.026768
ENSG00000261040.7	WFDC21P	3.744512966	8.651826021	2.23E-09
ENSG00000105967.15	TFEC	3.743716065	14.2108212	6.15E-15
ENSG00000246223.8	LINC01550	3.741643277	13.17710897	6.65E-14
ENSG00000232283.1	HSD17B3-AS1	3.740469916	1.368406212	0.042815
ENSG00000213892.11	CEACAM16	3.739749191	4.15855182	6.94E-05
ENSG00000240143.1	AL023653.1	3.738762117	7.956597715	1.11E-08
ENSG00000148123.14	PLPPR1	3.737085878	6.947407496	1.13E-07
ENSG00000236908.2	AC005865.1	3.737016971	1.37833392	0.041847
ENSG00000173083.14	HPSE	3.736150019	10.42435479	3.76E-11
ENSG00000136011.14	STAB2	3.734788899	4.411043823	3.88E-05
ENSG00000284595.1	MIR6785	3.733258204	1.763412836	0.017242
ENSG00000258128.2	MKRN9P	3.72841504	1.370453148	0.042613
ENSG00000205436.7	EXOC3L4	3.728369115	20.49417572	3.20E-21
ENSG00000267369.1	AC015911.7	3.726933014	6.756558419	1.75E-07
ENSG00000254288.1	AC087672.2	3.726876431	1.370453148	0.042613
ENSG00000261208.1	AL365475.1	3.726113541	6.017673673	9.60E-07
ENSG00000211698.2	TRGV4	3.723018139	1.355161791	0.044141
ENSG00000172955.17	ADH6	3.722629507	1.366641066	0.042989

ENSG00000110777.11	POU2AF1	3.7205255	8.699622647	2.00E-09
ENSG00000204758.7	AC008429.1	3.720407771	7.955119147	1.11E-08
ENSG00000150201.14	FXYD4	3.718149552	2.608590856	0.002463
ENSG00000196735.11	HLA-DQA1	3.718126743	33.76092193	1.73E-34
ENSG00000187474.4	FPR3	3.716436089	25.28705627	5.16E-26
ENSG00000228401.4	HSPC324	3.716141479	1.75056927	0.01776
ENSG00000164749.11	HNF4G	3.71507372	1.550465425	0.028154
ENSG00000226754.1	AL606760.1	3.713769326	1.549767215	0.028199
ENSG00000168356.11	SCN11A	3.708565024	2.464013029	0.003435
ENSG00000272764.1	AL596094.1	3.706945118	5.283716189	5.20E-06
ENSG00000271605.5	MILR1	3.704978061	16.86599489	1.36E-17
ENSG00000002726.20	AOC1	3.702806908	1.354020949	0.044257
ENSG00000100079.6	LGALS2	3.702312837	11.14652603	7.14E-12
ENSG00000101670.11	LIPG	3.698662121	11.6014843	2.50E-12
ENSG00000197980.12	LEKR1	3.697540673	1.545248544	0.028494
ENSG00000226007.2	BX005266.2	3.697083717	1.348351205	0.044838
ENSG00000284258.1	MIR8085	3.693479024	5.20471392	6.24E-06
ENSG00000237357.2	BX088651.4	3.691537631	1.538555044	0.028936
ENSG00000279968.2	GVQW2	3.690872204	1.74182743	0.018121
ENSG00000187726.8	DNAJB13	3.688037386	3.120689898	0.000757
ENSG00000235961.5	PNMA6A	3.686225439	6.816872958	1.52E-07
ENSG00000261439.1	DKFZP434H168	3.685263511	3.089325211	0.000814
ENSG00000283991.1	AC017000.1	3.676817256	2.855620425	0.001394
ENSG00000231551.8	AC245100.4	3.674471523	11.80052343	1.58E-12
ENSG00000174255.6	ZNF80	3.672876512	1.519898308	0.030207
ENSG00000186188.10	FFAR4	3.672238247	3.023459653	0.000947
ENSG00000270069.1	MIR222HG	3.669729346	5.922113824	1.20E-06
ENSG00000255320.1	AP000759.1	3.667313087	5.318756244	4.80E-06
ENSG00000253988.1	AC079015.1	3.666964913	1.338110503	0.045908
ENSG00000113763.11	UNC5A	3.666414057	1.52324689	0.029975
ENSG00000089723.9	OTUB2	3.664425639	4.421353585	3.79E-05
ENSG00000105717.13	PBX4	3.663634961	10.73065817	1.86E-11
ENSG00000225606.1	AC005281.1	3.662825441	1.339914582	0.045718
ENSG00000152315.4	KCNK13	3.661832327	1.331681325	0.046593
ENSG00000106327.12	TFR2	3.660758407	3.404271336	0.000394
ENSG00000254503.1	AC010319.1	3.66073844	1.339388705	0.045773
ENSG00000256250.1	AC073912.1	3.660552762	1.33878957	0.045836
ENSG00000186076.5	AC012085.1	3.659871153	1.515741012	0.030497
ENSG00000246334.2	PRR7-AS1	3.659308358	3.462561727	0.000345
ENSG00000272696.1	AL359091.4	3.658614467	1.516048682	0.030476
ENSG00000234492.4	RPL34-AS1	3.653890572	1.512085307	0.030755
ENSG00000224389.9	C4B	3.650536573	13.71195706	1.94E-14
ENSG00000161640.15	SIGLEC11	3.647219629	7.246141111	5.67E-08
ENSG00000274752.1	TRBV12-3	3.64613801	1.50578827	0.031204
ENSG00000180828.2	BHLHE22	3.643611135	13.99595295	1.01E-14
ENSG00000173208.3	ABCD2	3.641125621	8.687166337	2.06E-09
ENSG00000231187.2	AL356056.2	3.640536841	1.50528346	0.03124

ENSG00000244752.2	CRYBB2	3.637665805	2.650537799	0.002236
ENSG00000176912.3	TYMSOS	3.636542859	3.062835909	0.000865
ENSG00000116990.10	MYCL	3.635919382	12.30555624	4.95E-13
ENSG00000200983.1	SNORA3A	3.633702456	1.497542841	0.031802
ENSG00000173369.15	C1QB	3.630012672	30.68947549	2.04E-31
ENSG00000130054.4	FAM155B	3.629062442	1.315298003	0.048384
ENSG00000164604.12	GPR85	3.625735173	3.225567559	0.000595
ENSG00000197465.13	GYPE	3.623076462	3.057146754	0.000877
ENSG00000117586.10	TNFSF4	3.621817836	13.48428917	3.28E-14
ENSG00000177822.7	AC098864.1	3.619959374	13.94129092	1.14E-14
ENSG00000161405.16	IKZF3	3.619395778	18.88023749	1.32E-19
ENSG00000259628.1	AC007000.3	3.618816888	1.309985949	0.048979
ENSG00000180767.9	CHST13	3.61765708	2.800376845	0.001584
ENSG00000183840.6	GPR39	3.614973401	5.533757772	2.93E-06
ENSG00000264785.1	AC005722.3	3.609643956	2.965239439	0.001083
ENSG00000227082.2	AC244021.1	3.605372724	9.599480129	2.51E-10
ENSG00000151882.11	CCL28	3.604413948	4.16599329	6.82E-05
ENSG00000250654.7	AC023794.3	3.602022238	4.567744389	2.71E-05
ENSG00000108602.17	ALDH3A1	3.600020015	7.433218835	3.69E-08
ENSG00000214553.10	LRRC37A11P	3.599518268	1.30469687	0.04958
ENSG00000272430.1	AL356056.3	3.598953359	2.562264461	0.00274
ENSG00000246662.6	LINC00535	3.598153078	1.303861116	0.049675
ENSG00000254944.1	ATP5PBP5	3.592970082	1.683718087	0.020715
ENSG00000122224.17	LY9	3.589646109	5.861530284	1.38E-06
ENSG00000197299.11	BLM	3.587388244	9.804047824	1.57E-10
ENSG00000200463.1	SNORD118	3.586785167	1.303036571	0.04977
ENSG00000277702.1	AC239859.6	3.585247957	2.566968514	0.00271
ENSG00000284353.1	MIR142	3.584748068	4.47833462	3.32E-05
ENSG00000278266.1	AC079949.2	3.58050938	3.027635816	0.000938
ENSG00000231105.1	AL031728.1	3.580398582	1.472846878	0.033663
ENSG00000122025.14	FLT3	3.57864193	5.341511198	4.56E-06
ENSG00000237976.1	AL391069.3	3.57833835	5.645218992	2.26E-06
ENSG00000074276.10	CDHR2	3.577203376	1.472846878	0.033663
ENSG00000169508.6	GPR183	3.575841177	9.945087223	1.13E-10
ENSG00000236914.3	LINC01852	3.574760911	3.122956728	0.000753
ENSG00000282386.1	AL358472.4	3.574270937	1.469844886	0.033897
ENSG00000250167.1	AC034206.1	3.573696108	1.921781474	0.011973
ENSG00000255150.2	EID3	3.573518716	11.38208844	4.15E-12
ENSG00000276115.1	AC026356.2	3.567912093	2.541284895	0.002876
ENSG00000120457.11	KCNJ5	3.567904889	14.82315868	1.50E-15
ENSG00000155307.18	SAMSN1	3.561223628	12.46007538	3.47E-13
ENSG00000237887.1	RPL23AP32	3.560852135	2.845089864	0.001429
ENSG00000258521.1	AL157871.2	3.560565852	3.326689265	0.000471
ENSG00000135127.11	BICDL1	3.558394373	10.7120883	1.94E-11
ENSG00000277152.1	AC110048.2	3.555444524	2.085465286	0.008214
ENSG00000260997.1	AC004847.1	3.555067841	2.259015828	0.005508
ENSG00000230333.6	AC004160.1	3.551592297	2.097222474	0.007994

ENSG00000251359.4	WWC2-AS2	3.549551026	2.316464011	0.004825
ENSG00000279085.1	AL022323.3	3.539751651	3.025160734	0.000944
ENSG00000166278.14	C2	3.536274023	54.70931685	1.95E-55
ENSG00000265134.1	MIR3190	3.534606749	1.451390246	0.035368
ENSG00000143851.15	PTPN7	3.53132716	15.07364607	8.44E-16
ENSG00000271387.1	AL445228.2	3.530903738	1.445283286	0.035869
ENSG00000250328.5	MGC32805	3.52280665	2.739339979	0.001822
ENSG00000197540.7	GZMM	3.520823741	9.836448837	1.46E-10
ENSG00000211766.1	TRBJ2-2P	3.520801203	4.198484665	6.33E-05
ENSG00000284411.1	MIR3191	3.52068172	1.443543798	0.036013
ENSG00000260339.1	HEXA-AS1	3.519960348	1.439718811	0.036331
ENSG00000259129.5	LINC00648	3.513888272	1.876693955	0.013283
ENSG00000283844.1	MIR214	3.513749083	4.381028634	4.16E-05
ENSG00000213468.6	FIRRE	3.513032232	3.941923745	0.000114
ENSG00000261346.1	AC116348.3	3.512909142	9.385125119	4.12E-10
ENSG00000215146.5	BX322639.1	3.509953725	2.950020769	0.001122
ENSG00000014257.15	ACPP	3.505374509	2.469454916	0.003393
ENSG00000241106.7	HLA-DOB	3.500771736	12.49860245	3.17E-13
ENSG00000019169.10	MARCO	3.499091017	15.91065538	1.23E-16
ENSG00000165171.10	METTL27	3.4975439	5.639391879	2.29E-06
ENSG00000253865.1	AC131025.1	3.493800159	2.879985889	0.001318
ENSG00000197768.10	STPG3	3.49074707	2.189058521	0.006471
ENSG00000105251.10	SHD	3.487342818	8.121793461	7.55E-09
ENSG00000278449.1	MIR6892	3.484584558	2.717454324	0.001917
ENSG00000170549.3	IRX1	3.479677367	2.889091111	0.001291
ENSG00000270177.1	AC104109.2	3.479427988	1.617687438	0.024116
ENSG00000169413.2	RNASE6	3.478119545	12.22912893	5.90E-13
ENSG00000118491.9	ZC2HC1B	3.477945467	2.37586136	0.004209
ENSG00000124466.8	LYPD3	3.477529877	2.686802951	0.002057
ENSG00000143674.10	MAP3K21	3.474840912	3.006305985	0.000986
ENSG00000117228.9	GBP1	3.471061472	36.361291	4.35E-37
ENSG00000232629.8	HLA-DQB2	3.466084829	12.91868772	1.21E-13
ENSG00000272138.1	LINC01607	3.464914878	1.409092651	0.038986
ENSG00000198336.9	MYL4	3.46402556	11.85870937	1.38E-12
ENSG00000271503.5	CCL5	3.459041108	23.45141343	3.54E-24
ENSG00000276991.1	RF01877	3.454903404	4.157028814	6.97E-05
ENSG00000120262.9	CCDC170	3.453701293	10.10342726	7.88E-11
ENSG00000112299.7	VNN1	3.452550198	6.018959012	9.57E-07
ENSG00000268049.1	AC012313.2	3.450146634	3.007140947	0.000984
ENSG00000258407.1	AL157955.1	3.449540068	3.864404009	0.000137
ENSG00000124256.14	ZBP1	3.447265277	8.455908503	3.50E-09
ENSG00000121807.5	CCR2	3.445768137	9.464382013	3.43E-10
ENSG00000026751.16	SLAMF7	3.442824311	26.17099728	6.75E-27
ENSG00000147145.12	LPAR4	3.442516992	3.457249198	0.000349
ENSG00000270571.2	AC007681.1	3.442166603	2.195165461	0.00638
ENSG00000155897.9	ADCY8	3.44002374	2.526934459	0.002972
ENSG00000133321.10	RARRES3	3.439590921	42.46343564	3.44E-43

ENSG00000283734.1	MIR4785		3.438522831	2.635766999	0.002313
ENSG00000134061.5	CD180		3.43731597	8.487321619	3.26E-09
ENSG00000007264.14	MATK		3.434874417	11.56794328	2.70E-12
ENSG00000211664.3	IGLV2-18		3.432077583	2.699448407	0.001998
ENSG00000102575.11	ACP5		3.427143247	28.93984829	1.15E-29
ENSG00000285921.1	AP000779.1		3.42522357	4.570178642	2.69E-05
ENSG00000265415.1	AC099850.3		3.424266062	3.757309189	0.000175
ENSG00000175697.10	GPR156		3.420983832	5.573825165	2.67E-06
ENSG00000178789.8	CD300LB		3.420093785	4.988850242	1.03E-05
ENSG00000255478.1	AP000944.1		3.411635204	2.911356941	0.001226
ENSG00000171847.10	FAM90A1		3.400687901	5.695488289	2.02E-06
ENSG00000105855.9	ITGB8		3.399361645	8.882769801	1.31E-09
ENSG00000079931.14	MOXD1		3.396064975	7.743953147	1.80E-08
ENSG00000180044.5	C3orf80		3.395912728	6.617232071	2.41E-07
ENSG00000173077.15		1-Dec	3.39236284	8.204449056	6.25E-09
ENSG00000130948.10	HSD17B3		3.391966881	7.342265794	4.55E-08
ENSG00000181097.5	BREA2		3.385734723	2.225469355	0.00595
ENSG00000171428.13	NAT1		3.382835112	14.20018925	6.31E-15
ENSG00000167850.3	CD300C		3.381117304	5.781397666	1.65E-06
ENSG00000216490.3	IFI30		3.376308314	28.9212023	1.20E-29
ENSG00000234840.1	LINC01239		3.372203408	6.532395075	2.93E-07
ENSG00000185842.15	DNAH14		3.371779848	4.856698539	1.39E-05
ENSG00000126264.9	HCST		3.371389656	15.62135976	2.39E-16
ENSG00000211751.9	TRBC1		3.366222404	11.71474914	1.93E-12
ENSG00000244219.6	TMEM225B		3.364157	5.301813082	4.99E-06
ENSG00000259448.2	LINC02352		3.363528222	1.790702633	0.016192
ENSG00000256612.7	CYP2B7P		3.362517458	2.372017679	0.004246
ENSG00000142621.19	FHAD1		3.360879467	2.668419819	0.002146
ENSG00000198691.12	ABCA4		3.357703778	5.191276106	6.44E-06
ENSG00000265533.1	AC114689.3		3.353885975	2.264320664	0.005441
ENSG00000269985.1	AL021328.1		3.352831048	4.054959014	8.81E-05
ENSG00000272379.1	AL008729.2		3.352369718	3.337168117	0.00046
ENSG00000197744.5	PTMAP2		3.351058316	5.468712332	3.40E-06
ENSG00000237886.1	NALT1		3.349210823	2.972702229	0.001065
ENSG00000269600.1	AC016629.2		3.348815633	2.680402869	0.002087
ENSG00000140968.10	IRF8		3.348296403	28.93510393	1.16E-29
ENSG00000265688.1	MAFG-DT		3.347619979	4.145684125	7.15E-05
ENSG00000253174.2	AC009630.2		3.346532194	2.93376314	0.001165
ENSG00000227486.1	AL445472.1		3.345061277	2.462098708	0.003451
ENSG00000245479.2	LINC01585		3.342236243	3.236329106	0.00058
ENSG00000283602.1	AC116353.5		3.34097528	1.896559746	0.012689
ENSG00000245667.2	AC006064.1		3.340733383	2.552601806	0.002802
ENSG00000283667.1	AC009802.1		3.337544389	6.239532828	5.76E-07
ENSG00000184574.9	LPAR5		3.336538231	7.124477732	7.51E-08
ENSG00000205702.10	CYP2D7		3.336508798	5.743793859	1.80E-06
ENSG00000150551.10	LYPD1		3.336081484	4.510077376	3.09E-05
ENSG00000244255.5	AL645922.1		3.335461488	35.81826951	1.52E-36

ENSG00000232519.2	AL353807.2	3.333696241	2.154116905	0.007013
ENSG00000152213.3	ARL11	3.333462901	6.469948185	3.39E-07
ENSG00000177602.5	HASPIN	3.3333223	5.821470761	1.51E-06
ENSG00000152076.18	CCDC74B	3.332515581	2.346424768	0.004504
ENSG00000119866.21	BCL11A	3.328754888	3.633729306	0.000232
ENSG00000100162.14	CENPM	3.328119747	9.738185475	1.83E-10
ENSG00000226087.1	AC073283.2	3.325396525	4.467808283	3.41E-05
ENSG00000225792.1	AC004540.2	3.322950049	5.779293413	1.66E-06
ENSG00000249695.6	AC026369.1	3.319717125	2.159249821	0.00693
ENSG00000235890.2	TSPEAR-AS1	3.31891411	1.327148187	0.047082
ENSG00000173372.16	C1QA	3.311655002	30.07751532	8.37E-31
ENSG00000182132.13	KCNIP1	3.306412881	1.803527029	0.015721
ENSG00000271360.1	AL512631.2	3.303782343	2.286035589	0.005176
ENSG00000285524.1	AC073236.1	3.301959539	1.545379902	0.028485
ENSG00000146285.13	SCML4	3.297414333	4.799451783	1.59E-05
ENSG00000283848.1	MIR6872	3.294677106	2.178550446	0.006629
ENSG00000253227.1	AC090192.2	3.294193257	2.972775981	0.001065
ENSG00000236308.1	AL138921.2	3.288861073	1.90903693	0.01233
ENSG00000146070.16	PLA2G7	3.288813852	5.700998968	1.99E-06
ENSG00000179133.13	C10orf67	3.285905659	2.47661128	0.003337
ENSG00000280194.1	AD000864.1	3.284643308	16.20682524	6.21E-17
ENSG00000167103.11	PIP5KL1	3.283442759	2.367656813	0.004289
ENSG00000184949.15	FAM227A	3.281763771	5.774336363	1.68E-06
ENSG00000204252.13	HLA-DOA	3.280333498	31.09825196	7.98E-32
ENSG00000178826.10	TMEM139	3.279924003	1.84706753	0.014221
ENSG00000127074.14	RGS13	3.278028411	3.74628049	0.000179
ENSG00000173578.7	XCR1	3.278012174	7.515375635	3.05E-08
ENSG00000125245.12	GPR18	3.277311806	3.016851415	0.000962
ENSG00000253917.4	AC226119.1	3.276770875	2.063814232	0.008633
ENSG00000127472.10	PLA2G5	3.272563372	4.884065759	1.31E-05
ENSG00000260542.1	AL499627.1	3.267284251	8.618141069	2.41E-09
ENSG00000185527.11	PDE6G	3.266682923	3.288988762	0.000514
ENSG00000225511.7	LINC00475	3.264353087	4.917874426	1.21E-05
ENSG00000223534.1	HLA-DQB1-AS1	3.264019648	22.91446494	1.22E-23
ENSG00000224481.2	AC245100.1	3.263654797	2.359808507	0.004367
ENSG00000109956.12	B3GAT1	3.260833297	5.883525641	1.31E-06
ENSG00000066468.22	FGFR2	3.258494022	1.639765494	0.022921
ENSG00000038945.14	MSR1	3.256356009	19.88695691	1.30E-20
ENSG00000225407.3	AC025188.1	3.253993474	4.668921627	2.14E-05
ENSG00000230869.1	AGAP10P	3.253788628	1.914041446	0.012189
ENSG00000285526.1	AC020907.6	3.253665836	2.664161977	0.002167
ENSG00000186952.14	TMEM232	3.252028455	2.55741883	0.002771
ENSG00000268027.5	AC243960.1	3.247822782	3.898383716	0.000126
ENSG00000137825.10	ITPKA	3.246453214	4.77070103	1.70E-05
ENSG00000234630.1	AC245060.2	3.244986867	2.749420934	0.001781
ENSG00000206337.10	HCP5	3.244186896	41.6851775	2.06E-42
ENSG00000164743.4	C8orf48	3.24382748	2.422283995	0.003782

ENSG00000243829.1	AC011495.1	3.242080109	1.951982119	0.011169
ENSG00000272009.1	AL121944.1	3.241626816	3.618416661	0.000241
ENSG00000224577.1	LINC01117	3.240381377	2.862957024	0.001371
ENSG00000213846.5	AC098614.1	3.239123691	8.991300942	1.02E-09
ENSG00000235649.2	MXRA5Y	3.23557906	2.737990601	0.001828
ENSG00000267629.3	AC138430.1	3.231160115	1.826706384	0.014904
ENSG00000277734.7	TRAC	3.228321246	21.03211117	9.29E-22
ENSG00000234775.1	AC105940.2	3.227651381	2.19057864	0.006448
ENSG00000073861.2	TBX21	3.227575209	7.259689333	5.50E-08
ENSG00000273061.1	CDC37L1-DT	3.225706565	1.867747726	0.01356
ENSG00000211767.1	TRBJ2-3	3.224549818	6.730985357	1.86E-07
ENSG00000253653.1	AC009185.1	3.223462602	2.014317396	0.009676
ENSG00000248254.1	AC107398.2	3.222675745	2.096861794	0.008001
ENSG00000280137.1	AC116348.4	3.222547712	5.582835358	2.61E-06
ENSG00000136250.11	AOAH	3.219024482	11.07711775	8.37E-12
ENSG00000281357.2	ARRDC3-AS1	3.217278834	3.921178162	0.00012
ENSG00000272817.1	AL359198.1	3.215733063	2.509803848	0.003092
ENSG00000100453.12	GZMB	3.214377987	8.718941504	1.91E-09
ENSG00000196664.4	TLR7	3.214011609	10.41641315	3.83E-11
ENSG00000188573.7	FBLL1	3.210863969	2.47651983	0.003338
ENSG00000105492.15	SIGLEC6	3.210249606	1.770330994	0.016969
ENSG00000125384.6	PTGER2	3.208412172	7.251239104	5.61E-08
ENSG00000276672.1	AL161891.1	3.204531728	1.686643604	0.020576
ENSG00000211768.1	TRBJ2-4	3.204058757	2.418891563	0.003812
ENSG00000173947.13	PIFO	3.200683913	1.735498429	0.018387
ENSG00000126860.11	EVI2A	3.1984407	11.45028548	3.55E-12
ENSG00000266865.6	AC138207.8	3.198377763	4.902443431	1.25E-05
ENSG00000283152.1	MIR3120	3.196462136	2.850507469	0.001411
ENSG00000284407.1	MIR5001	3.195735	6.615949786	2.42E-07
ENSG00000151490.13	PTPRO	3.194580782	12.53050276	2.95E-13
ENSG00000141293.15	SKAP1	3.194317174	8.622161253	2.39E-09
ENSG00000143631.10	FLG	3.193061513	5.159481527	6.93E-06
ENSG00000064787.13	BCAS1	3.192066415	8.998418819	1.00E-09
ENSG00000159189.11	C1QC	3.188450811	29.50108016	3.15E-30
ENSG00000225706.1	PTPRD-AS1	3.186726186	1.698625746	0.020016
ENSG00000188257.11	PLA2G2A	3.177556324	9.968920676	1.07E-10
ENSG00000240137.5	ERICH6-AS1	3.175833274	1.942467546	0.011416
ENSG00000265474.1	AC010761.4	3.174365852	9.043252689	9.05E-10
ENSG00000204287.13	HLA-DRA	3.171558479	34.95941868	1.10E-35
ENSG00000122254.6	HS3ST2	3.171486154	2.927717241	0.001181
ENSG00000152380.9	FAM151B	3.171274119	4.460788948	3.46E-05
ENSG00000228863.8	AL121985.1	3.165717577	8.359245204	4.37E-09
ENSG00000136944.17	LMX1B	3.158748907	5.037985985	9.16E-06
ENSG00000183918.16	SH2D1A	3.153497315	8.697878857	2.01E-09
ENSG00000260063.1	AL512408.1	3.149339861	2.976414231	0.001056
ENSG00000239194.1	RNU1-123P	3.14919433	2.208604395	0.006186
ENSG00000227660.1	UST-AS1	3.148927119	1.641410912	0.022834

ENSG00000169442.8	CD52	3.148321437	10.66451041	2.17E-11
ENSG00000196668.3	LINC00173	3.148237586	8.089210595	8.14E-09
ENSG00000133020.4	MYH8	3.14780982	15.83368853	1.47E-16
ENSG00000247774.6	PCED1B-AS1	3.146957624	9.343058276	4.54E-10
ENSG00000124772.11	CPNE5	3.136923723	8.650529682	2.24E-09
ENSG00000156475.18	PPP2R2B	3.13559872	8.790645789	1.62E-09
ENSG00000167157.10	PRRX2	3.135132261	7.83355855	1.47E-08
ENSG00000283897.1	AC011416.3	3.131197706	2.931656835	0.00117
ENSG00000179344.16	HLA-DQB1	3.12740741	24.26772782	5.40E-25
ENSG00000116254.17	CHD5	3.125682874	1.714795161	0.019284
ENSG00000211752.3	TRBV27	3.123181285	1.559242386	0.02759
ENSG00000267681.1	AC135721.1	3.122228899	3.901938657	0.000125
ENSG00000254815.5	AP006284.1	3.121143866	5.387518922	4.10E-06
ENSG00000268798.1	AC027307.3	3.118800067	1.76080806	0.017346
ENSG00000180938.5	ZNF572	3.118758254	3.249534823	0.000563
ENSG00000163462.17	TRIM46	3.115697215	7.31124	4.88E-08
ENSG00000196109.7	ZNF676	3.114865629	1.34568968	0.045114
ENSG00000262681.2	AC005722.2	3.114608381	1.459274985	0.034732
ENSG00000279320.1	AC069528.2	3.113661654	1.324915874	0.047324
ENSG00000163792.6	TCF23	3.108615386	5.008324596	9.81E-06
ENSG00000258851.1	AL139300.2	3.108463001	1.687203151	0.020549
ENSG00000171044.10	XKR6	3.106655092	5.891935902	1.28E-06
ENSG00000142235.9	LMTK3	3.105958952	3.912773401	0.000122
ENSG00000253161.5	LINC01605	3.103308133	1.824066787	0.014995
ENSG00000147168.12	IL2RG	3.093004715	21.42369572	3.77E-22
ENSG00000125735.10	TNFSF14	3.091060694	10.08297235	8.26E-11
ENSG00000269915.1	AP006621.4	3.090969391	8.557436659	2.77E-09
ENSG00000095397.13	WHRN	3.087512997	12.70054904	1.99E-13
ENSG00000285171.1	AL590764.2	3.087376718	20.93862704	1.15E-21
ENSG00000258449.1	AC023510.1	3.086596124	3.053441379	0.000884
ENSG00000131401.11	NAPSB	3.086234188	10.33552518	4.62E-11
ENSG00000120280.5	CXorf21	3.086143437	6.940200039	1.15E-07
ENSG00000196460.13	RFX8	3.08148544	2.957832456	0.001102
ENSG00000176920.11	FUT2	3.07918662	1.730250713	0.01861
ENSG00000120332.15	TNN	3.078104929	1.492791106	0.032152
ENSG00000141497.13	ZMYND15	3.077709712	7.606968221	2.47E-08
ENSG00000275638.1	AC011939.3	3.077471118	1.833839718	0.014661
ENSG00000166793.10	YPEL4	3.075996548	11.61425182	2.43E-12
ENSG00000231113.2	AL035587.1	3.074769457	4.77070103	1.70E-05
ENSG00000104921.14	FCER2	3.074561976	4.993766046	1.01E-05
ENSG00000265118.5	AC134669.1	3.073058602	9.098066816	7.98E-10
ENSG00000234546.3	LNCTAM34A	3.071421421	2.984863125	0.001035
ENSG00000156738.17	MS4A1	3.070838906	2.399637213	0.003984
ENSG00000099365.10	STX1B	3.06865938	23.41996712	3.80E-24
ENSG00000140254.12	DUOXA1	3.068151714	1.488521704	0.03247
ENSG00000172543.7	CTSW	3.067970774	17.21982986	6.03E-18
ENSG00000279532.1	AC002094.5	3.066426516	1.579703826	0.026321

ENSG00000272917.1	AC010186.4	3.06451268	6.020507881	9.54E-07
ENSG00000258744.1	AL132800.1	3.063396294	1.5642146	0.027276
ENSG00000121552.3	CSTA	3.062974741	4.036443415	9.20E-05
ENSG00000253347.1	AC040934.1	3.062575073	2.472538393	0.003369
ENSG00000272301.1	AP002360.3	3.062280876	1.839024311	0.014487
ENSG00000204264.9	PSMB8	3.061065654	47.38113218	4.16E-48
ENSG00000198520.11	ARMH1	3.059290228	6.568259449	2.70E-07
ENSG00000110448.10	CD5	3.058754147	9.38009222	4.17E-10
ENSG00000077984.5	CST7	3.055101439	10.46554305	3.42E-11
ENSG00000027869.11	SH2D2A	3.054415433	8.063910976	8.63E-09
ENSG00000225361.3	PPP1R26-AS1	3.054157342	3.579502259	0.000263
ENSG00000115956.9	PLEK	3.052909539	28.57727128	2.65E-29
ENSG00000268201.1	AC020915.2	3.051846001	5.431071972	3.71E-06
ENSG00000181195.10	PENK	3.047575458	21.07825794	8.35E-22
ENSG00000248869.5	LINC02511	3.047209184	1.409512713	0.038948
ENSG00000237669.1	AL671277.2	3.04478107	7.355605272	4.41E-08
ENSG00000251429.1	AC098679.2	3.044509234	1.904303817	0.012465
ENSG00000161681.15	SHANK1	3.043108659	7.846639404	1.42E-08
ENSG00000173805.15	HAP1	3.041868372	2.533569788	0.002927
ENSG00000284964.1	AC242842.2	3.041592861	5.098964634	7.96E-06
ENSG00000281741.2	AC241377.3	3.037263591	6.443400682	3.60E-07
ENSG00000168913.6	ENHO	3.034176236	4.122232283	7.55E-05
ENSG00000240065.7	PSMB9	3.029371436	38.91362018	1.22E-39
ENSG00000013725.14	CD6	3.026271164	11.99471081	1.01E-12
ENSG00000176826.15	FKBP9P1	3.024594016	3.314446539	0.000485
ENSG00000225964.5	NRIR	3.022618972	1.313658047	0.048567
ENSG00000159261.10	CLDN14	3.01913243	2.146648388	0.007134
ENSG00000213809.8	KLRK1	3.014904214	18.26290647	5.46E-19
ENSG00000206432.4	TMEM200C	3.014615841	2.435862858	0.003666
ENSG00000161643.12	SIGLEC16	3.011004534	6.759429506	1.74E-07
ENSG00000177575.12	CD163	3.010956135	20.95560439	1.11E-21
ENSG00000211772.11	TRBC2	3.010315763	19.56730605	2.71E-20
ENSG00000229891.1	LINC01315	3.009022457	6.613441111	2.44E-07
ENSG00000251576.1	LINC01267	3.008619685	1.903451659	0.01249
ENSG00000255819.7	KLRC4-KLRK1	3.008137494	20.37010859	4.26E-21
ENSG00000106772.17	PRUNE2	3.007987173	35.04784827	8.96E-36
ENSG00000182866.16	LCK	3.006891356	13.03720791	9.18E-14
ENSG00000283445.1	AL136985.3	3.006868162	1.499481836	0.031661
ENSG00000010932.16	FMO1	3.006539363	4.248123471	5.65E-05
ENSG00000225886.3	AL445490.1	3.005686468	3.368286853	0.000428
ENSG00000213512.2	GBP7	3.001557298	4.431412239	3.70E-05
ENSG00000132514.13	CLEC10A	3.001485944	23.62670014	2.36E-24
ENSG00000143036.16	SLC44A3	2.998423577	5.541357267	2.88E-06
ENSG00000166111.9	SVOP	2.995431816	8.268292264	5.39E-09
ENSG00000139055.6	ERP27	2.994175963	2.124314953	0.007511
ENSG00000140379.7	BCL2A1	2.99179115	2.764087076	0.001722
ENSG00000108691.9	CCL2	2.991551156	20.34465903	4.52E-21

ENSG00000271774.1	AL109930.1	2.991486816	5.482878475	3.29E-06
ENSG00000234211.2	AL451067.1	2.990919106	6.355528699	4.41E-07
ENSG00000102524.11	TNFSF13B	2.990849217	20.9722353	1.07E-21
ENSG00000117477.12	CCDC181	2.990107248	1.485120229	0.032725
ENSG00000159860.7	TCAF2P1	2.989136758	3.700427752	0.000199
ENSG00000133067.17	LGR6	2.988119469	5.183379273	6.56E-06
ENSG00000274878.1	RF02107	2.987548516	1.526302628	0.029764
ENSG00000141505.11	ASGR1	2.987335523	2.94561217	0.001133
ENSG00000090382.6	LYZ	2.983417106	16.88374597	1.31E-17
ENSG00000223834.3	AL161935.1	2.983383582	2.730781972	0.001859
ENSG00000110079.17	MS4A4A	2.980908469	21.93575995	1.16E-22
ENSG00000115415.18	STAT1	2.979752045	34.10610475	7.83E-35
ENSG00000279259.1	AC087741.3	2.978947872	1.71774299	0.019154
ENSG00000230387.2	AL118508.1	2.977500293	5.070192318	8.51E-06
ENSG00000151322.18	NPAS3	2.975872575	7.684893945	2.07E-08
ENSG00000127152.17	BCL11B	2.974249406	5.58861926	2.58E-06
ENSG00000223865.10	HLA-DPB1	2.973403034	31.52507958	2.98E-32
ENSG00000267102.1	AC060766.1	2.972764012	5.064083063	8.63E-06
ENSG00000259031.1	AL845552.2	2.970997106	3.119190676	0.00076
ENSG00000225937.2	PCA3	2.969753432	14.42189717	3.79E-15
ENSG00000231389.7	HLA-DPA1	2.968407265	32.49223205	3.22E-33
ENSG00000224358.1	AL451074.2	2.964474196	3.29823428	0.000503
ENSG00000258590.5	NBEAP1	2.962406591	1.466297885	0.034174
ENSG00000224261.2	RPSAP18	2.960772963	1.360140034	0.043638
ENSG00000149090.11	PAMR1	2.959735318	22.71738116	1.92E-23
ENSG00000229380.1	AC147651.1	2.956222033	1.331268858	0.046637
ENSG00000259523.1	AC022613.2	2.956103799	1.613494515	0.02435
ENSG00000154589.6	LY96	2.95468182	9.89420415	1.28E-10
ENSG00000281883.1	AL512506.3	2.954470896	3.880512378	0.000132
ENSG00000133110.14	POSTN	2.952087266	22.10213293	7.90E-23
ENSG00000123685.8	BATF3	2.951336636	10.30110261	5.00E-11
ENSG00000280184.2	AL023806.3	2.948263456	2.52685407	0.002973
ENSG00000198106.8	SNX29P2	2.944889565	2.980071676	0.001047
ENSG00000182397.14	DNM1P46	2.944854459	2.851074368	0.001409
ENSG00000144488.14	ESPNL	2.941594102	4.360164524	4.36E-05
ENSG00000279673.1	AC092919.2	2.940183142	4.27441864	5.32E-05
ENSG00000155659.14	VSIG4	2.937921251	16.96508442	1.08E-17
ENSG00000285804.1	AC093218.1	2.93663799	1.488190724	0.032494
ENSG00000176771.16	NCKAP5	2.93663238	6.675111388	2.11E-07
ENSG00000203780.10	FANK1	2.936249496	1.936752816	0.011568
ENSG00000105376.4	ICAM5	2.935813509	3.533901583	0.000292
ENSG00000198851.9	CD3E	2.93380871	14.98131393	1.04E-15
ENSG00000166927.12	MS4A7	2.933639908	34.66085918	2.18E-35
ENSG00000272871.1	AL159169.2	2.931911868	3.939692544	0.000115
ENSG00000234166.1	ARHGEF19-AS1	2.923766091	1.431986104	0.036984
ENSG00000225864.1	AL645939.1	2.9227757	36.98404137	1.04E-37
ENSG00000257681.1	AC025265.1	2.922418919	1.522393706	0.030034

ENSG00000257764.2	AC020656.1	2.922069341	16.08456659	8.23E-17
ENSG00000270006.2	AC010531.6	2.921120981	2.171970252	0.00673
ENSG00000139626.15	ITGB7	2.917220854	19.89821664	1.26E-20
ENSG00000272986.1	AC009570.1	2.915544094	1.666854609	0.021535
ENSG00000258701.1	LINC00638	2.913464588	2.808154374	0.001555
ENSG00000128510.11	CPA4	2.91271043	4.153535658	7.02E-05
ENSG00000123485.11	HJURP	2.912342746	5.016080597	9.64E-06
ENSG00000276317.1	AL357033.3	2.912078725	1.637143962	0.02306
ENSG00000129295.8	LRRC6	2.91111874	2.508025607	0.003104
ENSG00000231345.3	BEND3P1	2.909985068	1.568788949	0.026991
ENSG00000127743.5	IL17B	2.908468036	4.423808095	3.77E-05
ENSG00000257591.5	ZNF625	2.908434701	11.49753378	3.18E-12
ENSG00000249825.5	CTD-2201I18.1	2.90778704	13.63823698	2.30E-14
ENSG00000283201.1	AC092329.3	2.907504363	3.992874956	0.000102
ENSG00000284644.1	AC074386.1	2.907177942	4.122767448	7.54E-05
ENSG00000275557.1	AC242842.1	2.905283362	5.931309675	1.17E-06
ENSG00000257202.1	AC084398.2	2.903666307	6.981731547	1.04E-07
ENSG00000111863.12	ADTRP	2.903369435	2.715809584	0.001924
ENSG00000263528.7	IKBKE	2.900572285	11.93767919	1.15E-12
ENSG00000172817.3	CYP7B1	2.899205783	3.005450465	0.000988
ENSG00000167286.9	CD3D	2.897606227	11.11030689	7.76E-12
ENSG00000275332.1	AC103691.2	2.897562183	1.757692397	0.017471
ENSG00000207112.1	SNORA25	2.897199248	2.235273505	0.005817
ENSG00000167083.6	GNGT2	2.895256909	8.553194848	2.80E-09
ENSG00000239713.8	APOBEC3G	2.895176621	31.76826343	1.71E-32
ENSG00000140839.11	CLEC18B	2.894249246	1.338814685	0.045834
ENSG00000135914.5	HTR2B	2.891815721	3.819161091	0.000152
ENSG00000167207.13	NOD2	2.887072524	13.13877439	7.26E-14
ENSG00000271579.1	AC078880.3	2.886103138	2.703823399	0.001978
ENSG00000229754.1	CXCR2P1	2.885317807	2.650207052	0.002238
ENSG00000172247.3	C1QTNF4	2.884370743	6.270904273	5.36E-07
ENSG00000133106.14	EPSTI1	2.879740202	29.52123632	3.01E-30
ENSG00000221887.5	HMSD	2.877683802	9.559938166	2.75E-10
ENSG00000203747.10	FCGR3A	2.876409886	13.57874248	2.64E-14
ENSG00000121380.12	BCL2L14	2.875818711	1.308906321	0.049101
ENSG00000230113.1	AC138207.1	2.872097608	12.65400561	2.22E-13
ENSG00000175175.5	PPM1E	2.872067626	8.339257686	4.58E-09
ENSG00000188015.9	S100A3	2.870116441	6.361849452	4.35E-07
ENSG00000171860.4	C3AR1	2.869970523	15.34744938	4.49E-16
ENSG00000101916.11	TLR8	2.868412075	6.498847995	3.17E-07
ENSG00000284393.1	AC092111.3	2.866046575	3.703745844	0.000198
ENSG00000284142.1	MIR6845	2.865406794	2.385058914	0.00412
ENSG00000231852.7	CYP21A2	2.865099465	3.939264379	0.000115
ENSG00000099958.14	DERL3	2.863784104	16.65365511	2.22E-17
ENSG00000166928.10	MS4A14	2.86354146	10.22620938	5.94E-11
ENSG00000110077.14	MS4A6A	2.862185521	27.47008134	3.39E-28
ENSG00000147443.12	DOK2	2.861622682	24.5545619	2.79E-25

ENSG00000010610.9	CD4	2.858335536	28.86148709	1.38E-29
ENSG00000178562.17	CD28	2.856136424	6.494104684	3.21E-07
ENSG00000140030.5	GPR65	2.854066933	8.542684249	2.87E-09
ENSG00000100336.17	APOL4	2.853989988	32.05870081	8.74E-33
ENSG00000230658.1	KLHL7-DT	2.85125696	1.445225474	0.035874
ENSG00000172602.10	RND1	2.851166268	3.154291615	0.000701
ENSG00000100385.13	IL2RB	2.849728885	10.06927003	8.53E-11
ENSG00000102445.18	RUBCNL	2.84784628	11.56323952	2.73E-12
ENSG00000262001.1	DLGAP1-AS2	2.847497546	5.331951823	4.66E-06
ENSG00000196126.11	HLA-DRB1	2.847064871	33.07724226	8.37E-34
ENSG00000226032.2	AL035530.1	2.847009084	3.407357952	0.000391
ENSG00000122691.12	TWIST1	2.844595443	6.359762901	4.37E-07
ENSG00000187955.11	COL14A1	2.84159473	14.40620164	3.92E-15
ENSG00000124216.3	SNAI1	2.834964446	4.752448371	1.77E-05
ENSG00000221949.5	LINC01465	2.833767756	1.412029187	0.038723
ENSG00000237718.2	AC009095.1	2.831902897	1.41002243	0.038903
ENSG00000205037.2	AC134312.1	2.83114251	1.909286385	0.012323
ENSG00000129450.8	SIGLEC9	2.829172889	4.691597259	2.03E-05
ENSG00000172322.13	CLEC12A	2.828541296	4.086522371	8.19E-05
ENSG00000110324.10	IL10RA	2.827179731	29.39512015	4.03E-30
ENSG00000105963.14	ADAP1	2.826354931	18.86398789	1.37E-19
ENSG00000143119.13	CD53	2.825213855	23.19089504	6.44E-24
ENSG00000274897.2	PANO1	2.824795315	6.397584487	4.00E-07
ENSG00000168062.9	BATF2	2.824769572	20.51838661	3.03E-21
ENSG00000011600.11	TYROBP	2.823685849	22.69991049	2.00E-23
ENSG00000244926.6	ALKBH3-AS1	2.815800059	1.521627669	0.030087
ENSG00000100285.9	NEFH	2.815491379	7.939759111	1.15E-08
ENSG00000278324.1	RF02166	2.815404602	2.346146756	0.004507
ENSG00000259275.2	AC087477.2	2.812121235	5.779293413	1.66E-06
ENSG00000139194.7	RBP5	2.810272826	13.43345106	3.69E-14
ENSG00000136167.13	LCP1	2.808460754	22.46828013	3.40E-23
ENSG00000126709.14	IFI6	2.807179493	18.90403916	1.25E-19
ENSG00000087589.16	CASS4	2.806765817	8.659314547	2.19E-09
ENSG00000242574.8	HLA-DMB	2.806648856	33.42496784	3.76E-34
ENSG00000133055.8	MYBPH	2.806383075	11.56734611	2.71E-12
ENSG00000245648.1	AC022075.1	2.806298937	11.99886034	1.00E-12
ENSG00000056487.15	PHF21B	2.805716105	4.532331263	2.94E-05
ENSG00000161243.8	FBXO27	2.805211997	8.770888977	1.69E-09
ENSG00000117091.9	CD48	2.804790674	9.536447141	2.91E-10
ENSG00000261552.1	AC109460.4	2.804735348	5.51289761	3.07E-06
ENSG00000186648.14	CARMIL3	2.799840429	3.555758254	0.000278
ENSG00000158113.12	LRRC43	2.796706427	1.828366598	0.014847
ENSG00000166153.16	DEPDC4	2.795094151	1.375461026	0.042125
ENSG00000284280.1	MIR6843	2.794423385	1.489368533	0.032406
ENSG00000185905.3	C16orf54	2.794054791	7.462117342	3.45E-08
ENSG00000066294.14	CD84	2.793932369	24.68578554	2.06E-25
ENSG00000123338.12	NCKAP1L	2.793304984	28.69789985	2.00E-29

ENSG00000285230.1	RALY-AS1	2.791378587	6.111335399	7.74E-07
ENSG00000149054.15	ZNF215	2.786436377	4.476255701	3.34E-05
ENSG00000078725.12	BRINP1	2.785229516	3.002374679	0.000995
ENSG00000124491.15	F13A1	2.783150066	22.5335114	2.93E-23
ENSG00000230918.1	AC008063.1	2.782878456	3.028963288	0.000935
ENSG00000239636.1	AC004865.2	2.778404264	3.95610771	0.000111
ENSG00000258572.1	AL133467.1	2.774113547	4.051589637	8.88E-05
ENSG00000232237.3	ASCL5	2.770203326	6.196586025	6.36E-07
ENSG00000272360.1	AC116036.2	2.767805623	2.959162798	0.001099
ENSG00000226025.9	LGALS17A	2.766954677	11.21880476	6.04E-12
ENSG00000183484.11	GPR132	2.765514869	11.99715302	1.01E-12
ENSG00000177108.5	ZDHHC22	2.765310936	3.186954655	0.00065
ENSG00000227698.1	AP001619.1	2.765205906	1.608182889	0.02465
ENSG00000181085.14	MAPK15	2.764749877	2.556813087	0.002775
ENSG00000239552.2	HOXB-AS2	2.764471548	1.376550273	0.042019
ENSG00000204525.16	HLA-C	2.763948359	45.76954944	1.70E-46
ENSG00000204261.8	PSMB8-AS1	2.756064543	38.20351794	6.26E-39
ENSG00000267594.5	CYP4F24P	2.755142717	1.453542771	0.035193
ENSG00000183578.7	TNFAIP8L3	2.754357432	21.57966991	2.63E-22
ENSG00000175352.10	NRIP3	2.754069862	3.413132392	0.000386
ENSG00000256673.1	AC141557.1	2.75308658	2.765396579	0.001716
ENSG00000278727.1	AC000403.1	2.751921204	1.948220733	0.011266
ENSG00000158481.12	CD1C	2.751787718	7.652274165	2.23E-08
ENSG00000254254.5	AC012349.1	2.750806092	15.36872313	4.28E-16
ENSG00000264514.1	AP000915.1	2.750745441	6.857753755	1.39E-07
ENSG00000204642.13	HLA-F	2.748421316	36.79661278	1.60E-37
ENSG00000248993.1	AL645941.2	2.748152341	32.58430225	2.60E-33
ENSG00000271109.1	AC008555.5	2.747575238	3.811176863	0.000154
ENSG00000113263.12	ITK	2.744843179	10.83530669	1.46E-11
ENSG00000169989.2	TIGD4	2.742882829	1.715706896	0.019244
ENSG00000148600.14	CDHR1	2.741611983	3.106249727	0.000783
ENSG00000168394.11	TAP1	2.741468336	35.60253254	2.50E-36
ENSG00000109063.14	MYH3	2.740102191	18.56414798	2.73E-19
ENSG00000162692.11	VCAM1	2.739482621	27.7564218	1.75E-28
ENSG00000275097.1	AC024940.5	2.736263897	1.843308619	0.014345
ENSG00000020633.18	RUNX3	2.735736447	16.02598616	9.42E-17
ENSG00000225556.1	C2CD4D	2.735497731	3.542596765	0.000287
ENSG00000271581.1	AL671883.2	2.734691876	33.5579887	2.77E-34
ENSG00000166707.10	ZCCHC18	2.734661165	4.380156523	4.17E-05
ENSG00000130475.14	FCHO1	2.732171246	8.720127492	1.90E-09
ENSG00000280649.2	AC245100.8	2.731336431	9.170786973	6.75E-10
ENSG00000101213.6	PTK6	2.730252818	6.579898525	2.63E-07
ENSG00000267049.1	AC002398.1	2.730058493	5.110701959	7.75E-06
ENSG00000170571.11	EMB	2.729928345	25.77290068	1.69E-26
ENSG00000227039.6	ITGB2-AS1	2.729743268	5.693531149	2.03E-06
ENSG00000272155.1	AC055822.1	2.729154681	1.76881854	0.017029
ENSG00000100228.12	RAB36	2.728928782	2.673546436	0.002121

ENSG00000234062.7	AL390879.1	2.727934534	2.326397608	0.004716
ENSG00000115232.13	ITGA4	2.725826343	10.61022878	2.45E-11
ENSG00000204472.12	AIF1	2.724780982	20.40354059	3.95E-21
ENSG00000105991.8	HOXA1	2.724713563	1.480675121	0.033062
ENSG00000236234.1	AC091132.2	2.720994573	2.287381354	0.00516
ENSG00000006747.14	SCIN	2.720938828	4.810177991	1.55E-05
ENSG00000163131.10	CTSS	2.718240748	23.23380453	5.84E-24
ENSG00000122122.9	SASH3	2.717645483	11.23823389	5.78E-12
ENSG00000262185.2	AC005736.1	2.717458452	2.861496079	0.001376
ENSG00000151079.7	KCNA6	2.717195951	3.685257391	0.000206
ENSG00000275198.1	AL512791.2	2.716284584	4.215767012	6.08E-05
ENSG00000134516.16	DOCK2	2.715719551	20.47785646	3.33E-21
ENSG00000272902.2	TBC1D8-AS1	2.714344423	2.596630651	0.002531
ENSG00000234745.10	HLA-B	2.712054453	40.10041686	7.94E-41
ENSG00000007350.16	TKTL1	2.709311906	3.270635979	0.000536
ENSG00000186806.5	VSIG10L	2.705745896	7.980273265	1.05E-08
ENSG00000176490.4	DIRAS1	2.7043711	13.25108266	5.61E-14
ENSG00000140557.11	ST8SIA2	2.704054309	12.01265921	9.71E-13
ENSG00000010310.8	GIPR	2.703930912	3.13550317	0.000732
ENSG00000214262.4	ANKRD36BP1	2.70243904	2.968635952	0.001075
ENSG00000260455.1	NBAT1	2.701376655	2.037893331	0.009164
ENSG00000104972.15	LILRB1	2.700413199	10.97379379	1.06E-11
ENSG00000153283.12	CD96	2.697787042	8.645257259	2.26E-09
ENSG00000106819.11	ASPN	2.696663534	12.10163106	7.91E-13
ENSG00000112981.4	NME5	2.69352263	2.068829343	0.008534
ENSG00000101447.14	FAM83D	2.693050003	1.714942724	0.019278
ENSG00000087494.15	PTHLH	2.692572013	4.669990947	2.14E-05
ENSG00000198286.9	CARD11	2.692429362	8.986075444	1.03E-09
ENSG00000164484.11	TMEM200A	2.690843822	9.98815767	1.03E-10
ENSG00000162511.7	LAPTM5	2.690291867	22.38696794	4.10E-23
ENSG00000090659.17	CD209	2.689723469	23.23323832	5.84E-24
ENSG00000145703.15	IQGAP2	2.68810345	19.38992777	4.07E-20
ENSG00000186470.13	BTN3A2	2.686026619	28.16968082	6.77E-29
ENSG00000168546.10	GFRA2	2.68291872	13.69301846	2.03E-14
ENSG00000264456.1	AC138207.4	2.682348663	7.419946938	3.80E-08
ENSG00000095585.16	BLNK	2.681753826	13.29385644	5.08E-14
ENSG00000088882.7	CPXM1	2.680731391	12.05915545	8.73E-13
ENSG00000105374.9	NKG7	2.680073889	8.044291393	9.03E-09
ENSG00000255920.2	CCND2-AS1	2.679281619	7.414520277	3.85E-08
ENSG00000100342.20	APOL1	2.679087967	39.92073804	1.20E-40
ENSG00000260160.1	AC011468.1	2.678586036	2.741751935	0.001812
ENSG00000185862.6	EVI2B	2.676287017	11.31144631	4.88E-12
ENSG00000134539.16	KLRD1	2.67306685	7.929279138	1.18E-08
ENSG00000258376.2	AC004846.1	2.672030056	7.290906006	5.12E-08
ENSG00000115155.17	OTOF	2.670459325	6.514437102	3.06E-07
ENSG00000136267.13	DGKB	2.670426477	3.125167915	0.00075
ENSG00000260617.1	AC138028.5	2.670165752	5.810914769	1.55E-06

ENSG00000232229.5	LINC00865	2.669922043	2.262720062	0.005461
ENSG00000119917.13	IFIT3	2.664028283	29.14427674	7.17E-30
ENSG00000060558.3	GNA15	2.663867004	11.32916575	4.69E-12
ENSG00000113296.14	THBS4	2.663090443	13.02741719	9.39E-14
ENSG00000130513.6	GDF15	2.661824569	5.645693734	2.26E-06
ENSG00000256377.5	AC009509.1	2.661022761	2.281222567	0.005233
ENSG00000165168.7	CYBB	2.660692129	21.56972145	2.69E-22
ENSG00000136514.2	RTP4	2.658275925	9.129202491	7.43E-10
ENSG00000255197.5	AC090559.1	2.65572831	6.934735305	1.16E-07
ENSG00000266466.1	AC233702.7	2.655018722	2.770474281	0.001696
ENSG00000243819.4	RN7SL832P	2.653321836	1.971533606	0.010677
ENSG00000198133.8	TMEM229B	2.651149316	20.23299338	5.85E-21
ENSG00000233360.4	Z83844.2	2.649018606	12.64439497	2.27E-13
ENSG00000165457.13	FOLR2	2.64898814	20.29761458	5.04E-21
ENSG00000254966.1	AC103974.1	2.647788546	3.591925412	0.000256
ENSG00000089041.16	P2RX7	2.647298079	18.32266818	4.76E-19
ENSG00000162490.6	DRAXIN	2.643822622	3.109288267	0.000778
ENSG00000075035.9	WSCD2	2.642245144	3.944439466	0.000114
ENSG00000254859.1	AC067930.4	2.640547674	2.244924127	0.00569
ENSG00000185101.12	ANO9	2.640404695	7.139700443	7.25E-08
ENSG00000197629.5	MPEG1	2.64028765	21.49141685	3.23E-22
ENSG00000149548.14	CCDC15	2.638908089	2.497559566	0.00318
ENSG00000019582.14	CD74	2.638092347	29.4029447	3.95E-30
ENSG00000225492.6	GBP1P1	2.637742677	16.47838735	3.32E-17
ENSG00000105383.14	CD33	2.63761759	10.20972069	6.17E-11
ENSG00000149633.11	KIAA1755	2.635628434	18.24305384	5.71E-19
ENSG00000234511.9	C5orf58	2.635328173	8.427074871	3.74E-09
ENSG00000025708.13	TYMP	2.632681937	27.11046155	7.75E-28
ENSG00000246526.2	LINC002481	2.6323929	2.527431338	0.002969
ENSG00000103472.10	RRN3P2	2.632340257	5.341104063	4.56E-06
ENSG00000165891.15	E2F7	2.630923677	1.401331084	0.039689
ENSG00000203734.11	ECT2L	2.630895822	1.402479525	0.039584
ENSG00000272814.1	AC093732.2	2.630484952	5.931509404	1.17E-06
ENSG00000146006.7	LRRTM2	2.630071862	2.608512993	0.002463
ENSG00000154118.12	JPH3	2.629880487	1.730899155	0.018582
ENSG00000266903.1	AC243964.2	2.62851315	1.594096585	0.025463
ENSG00000260293.2	AC106820.4	2.626444456	1.985837613	0.010331
ENSG00000179082.3	C9orf106	2.624018219	1.661346632	0.02181
ENSG00000082293.12	COL19A1	2.621788307	4.026884952	9.40E-05
ENSG00000257452.1	AC004551.1	2.621316603	8.497313954	3.18E-09
ENSG00000081237.19	PTPRC	2.619224881	15.61929509	2.40E-16
ENSG00000163393.12	SLC22A15	2.618963802	9.760381437	1.74E-10
ENSG00000283312.1	AC017104.4	2.617790441	1.481072673	0.033031
ENSG00000115165.9	CYTIP	2.614076634	9.409084431	3.90E-10
ENSG00000043591.5	ADRB1	2.613571375	7.038279581	9.16E-08
ENSG00000244731.7	C4A	2.613464799	12.7739854	1.68E-13
ENSG00000262655.3	SPON1	2.612887342	6.580307867	2.63E-07

ENSG00000123610.4	TNFAIP6	2.610516089	9.727005842	1.87E-10
ENSG00000248027.1	AP001351.1	2.609196717	1.418621977	0.03814
ENSG00000179921.14	GPBAR1	2.608966498	5.506563744	3.11E-06
ENSG00000166710.18	B2M	2.607728027	42.66642805	2.16E-43
ENSG00000265840.1	AC010761.5	2.604825486	7.503731925	3.14E-08
ENSG00000156966.6	B3GNT7	2.603380672	5.217407435	6.06E-06
ENSG00000211770.1	TRBJ2-6	2.597106671	2.539036354	0.00289
ENSG00000206503.12	HLA-A	2.594995732	43.00501136	9.89E-44
ENSG00000227766.1	AL671277.1	2.594043198	40.23405546	5.83E-41
ENSG00000148948.7	LRRC4C	2.591556423	10.07935662	8.33E-11
ENSG00000266368.1	AC005410.2	2.591288352	1.811267287	0.015443
ENSG00000279805.1	Z95114.3	2.589814906	2.78883199	0.001626
ENSG00000086205.17	FOLH1	2.58944034	1.916561468	0.012118
ENSG00000153446.15	C16orf89	2.586679809	5.931099038	1.17E-06
ENSG00000261416.1	AC012645.3	2.584718615	15.75216368	1.77E-16
ENSG00000277402.1	MIR6891	2.58236419	28.55919832	2.76E-29
ENSG00000185338.5	SOCS1	2.580335694	7.901220042	1.26E-08
ENSG00000265206.5	AC004687.1	2.578964523	7.887849088	1.29E-08
ENSG00000250486.4	FAM218A	2.57765829	3.742532995	0.000181
ENSG00000215915.9	ATAD3C	2.576753444	3.191917431	0.000643
ENSG00000177675.8	CD163L1	2.575707296	17.12493911	7.50E-18
ENSG00000188042.7	ARL4C	2.573239971	30.69505052	2.02E-31
ENSG00000128011.4	LRFN1	2.572644778	7.752722653	1.77E-08
ENSG00000156587.15	UBE2L6	2.56840214	45.03759008	9.17E-46
ENSG00000165178.9	NCF1C	2.568347801	10.68128863	2.08E-11
ENSG00000106809.10	OGN	2.567212784	12.45627854	3.50E-13
ENSG00000142178.8	SIK1	2.566627834	2.11967673	0.007591
ENSG00000144395.17	CCDC150	2.563203352	8.158649068	6.94E-09
ENSG00000185811.18	IKZF1	2.562586797	14.89257145	1.28E-15
ENSG00000160255.17	ITGB2	2.562291433	18.80845422	1.55E-19
ENSG00000261332.1	AC116348.2	2.561782121	7.823547701	1.50E-08
ENSG00000167613.15	LAIR1	2.561278421	27.70240744	1.98E-28
ENSG00000166523.7	CLEC4E	2.560895341	2.333010538	0.004645
ENSG00000119915.4	ELOVL3	2.560522111	1.537784498	0.028988
ENSG00000280054.1	AC004241.5	2.559874312	1.64605869	0.022591
ENSG00000132749.10	TESMIN	2.559761832	1.422841568	0.037771
ENSG00000159958.6	TNFRSF13C	2.559608247	3.597020744	0.000253
ENSG00000148483.8	TMEM236	2.553606901	4.503850261	3.13E-05
ENSG00000110427.15	KIAA1549L	2.550405076	5.05133035	8.89E-06
ENSG00000186517.13	ARHGAP30	2.549811461	21.23786754	5.78E-22
ENSG00000169224.12	GCSAML	2.548349193	1.352977728	0.044363
ENSG00000159166.13	LAD1	2.54795968	17.29525377	5.07E-18
ENSG00000088827.12	SIGLEC1	2.546769966	25.80673802	1.56E-26
ENSG00000268995.1	VN1R82P	2.546418625	2.391116655	0.004063
ENSG00000105122.12	RASAL3	2.543409383	18.12647009	7.47E-19
ENSG00000177519.3	RPRM	2.543389878	1.858541632	0.01385
ENSG00000244509.3	APOBEC3C	2.542791423	51.79363824	1.61E-52

ENSG00000196368.4	NUDT11	2.542722595	3.419455101	0.000381
ENSG00000170458.13	CD14	2.541670292	19.40654251	3.92E-20
ENSG00000136689.18	IL1RN	2.53703477	2.145607534	0.007151
ENSG00000182487.12	NCF1B	2.534646709	6.451831518	3.53E-07
ENSG00000155926.13	SLA	2.533964872	13.78161951	1.65E-14
ENSG00000130768.14	SMPDL3B	2.532769526	1.388021641	0.040924
ENSG00000140368.12	PSTPIP1	2.530149757	16.45185743	3.53E-17
ENSG00000266036.1	AC016888.1	2.528407819	21.46929576	3.39E-22
ENSG00000187210.13	GCNT1	2.527542072	44.40537999	3.93E-45
ENSG00000276136.1	AC016957.2	2.527059796	2.015008341	0.00966
ENSG00000158869.10	FCER1G	2.526972216	16.10038952	7.94E-17
ENSG00000180999.10	C1orf105	2.522665182	3.697034734	0.000201
ENSG00000272669.1	AL021707.6	2.521801156	3.419873299	0.00038
ENSG00000229873.1	OGFR-AS1	2.520241143	6.169772049	6.76E-07
ENSG00000260314.2	MRC1	2.519178678	16.93023738	1.17E-17
ENSG00000121005.8	CRISPLD1	2.519026018	6.985091882	1.03E-07
ENSG00000223551.1	TMSB4XP4	2.516217266	5.284079581	5.20E-06
ENSG00000213402.2	PTPRCAP	2.514657757	19.166903	6.81E-20
ENSG00000238113.6	LINC01410	2.51149728	4.270336219	5.37E-05
ENSG00000263873.1	AP003396.5	2.511209308	13.69778473	2.01E-14
ENSG00000250320.5	AC113383.1	2.511057797	1.632222522	0.023323
ENSG00000135917.14	SLC19A3	2.510769405	2.979302388	0.001049
ENSG00000241484.9	ARHGAP8	2.509322513	4.574817107	2.66E-05
ENSG00000234614.1	AL450992.2	2.507860747	5.013304747	9.70E-06
ENSG00000258405.9	ZNF578	2.507812134	2.534014719	0.002924
ENSG00000005844.17	ITGAL	2.503411151	16.00369483	9.92E-17
ENSG00000129226.13	CD68	2.503006553	25.66657833	2.15E-26
ENSG00000010671.15	BTK	2.502474518	13.03720791	9.18E-14
ENSG00000251023.1	AC114980.1	2.501878635	2.248389985	0.005644
ENSG00000196550.10	FAM72A	2.501061015	4.36600137	4.31E-05
ENSG00000146722.11	AC211486.1	2.500691919	2.551495459	0.002809
ENSG00000186652.9	PRG2	2.50027704	2.173400902	0.006708
ENSG00000206195.10	DUXAP8	2.499414529	5.568841988	2.70E-06
ENSG00000188060.7	RAB42	2.497897589	6.754202274	1.76E-07
ENSG00000283073.1	SMUG1-AS1	2.496527917	1.592925261	0.025531
ENSG00000267607.1	AC011511.5	2.496017887	24.62884468	2.35E-25
ENSG00000155966.13	AFF2	2.49555129	1.820109762	0.015132
ENSG00000136286.15	MYO1G	2.494957473	12.94854348	1.13E-13
ENSG00000243449.6	C4orf48	2.494284502	4.309227837	4.91E-05
ENSG00000224307.1	AL161785.1	2.492859416	3.7984352	0.000159
ENSG00000082074.16	FYB1	2.492541204	14.77740479	1.67E-15
ENSG00000259658.5	AC027559.1	2.491712668	2.323899664	0.004744
ENSG00000284554.2	AL022318.4	2.488815359	34.26451191	5.44E-35
ENSG00000077585.13	GPR137B	2.484816953	13.82305878	1.50E-14
ENSG00000251586.1	TET2-AS1	2.484778092	1.735999681	0.018365
ENSG00000145569.5	OTULINL	2.484217999	21.38672008	4.10E-22
ENSG00000133083.14	DCLK1	2.480016851	35.24863994	5.64E-36

ENSG00000163154.5	TNFAIP8L2	2.478779796	5.612817125	2.44E-06
ENSG00000236801.1	RPL24P8	2.47716417	2.549626934	0.002821
ENSG00000090339.8	ICAM1	2.476216772	33.45637292	3.50E-34
ENSG00000168398.6	BDKRB2	2.474668405	11.37055082	4.26E-12
ENSG00000039139.9	DNAH5	2.473447832	2.685038749	0.002065
ENSG00000159753.13	CARMIL2	2.471977227	8.648095911	2.25E-09
ENSG00000249577.1	AC010424.1	2.469148464	1.936558153	0.011573
ENSG00000163823.3	CCR1	2.467741873	13.93499948	1.16E-14
ENSG00000166963.12	MAP1A	2.467491871	42.78192084	1.65E-43
ENSG00000183873.15	SCN5A	2.467189864	8.756671294	1.75E-09
ENSG00000143387.12	CTSK	2.465992255	12.49886664	3.17E-13
ENSG00000128815.19	WDFY4	2.460375873	18.15031063	7.07E-19
ENSG00000102879.15	CORO1A	2.458687463	16.28813174	5.15E-17
ENSG00000254204.1	AC022034.3	2.457365496	3.644991294	0.000226
ENSG00000154096.13	THY1	2.453392593	13.39611044	4.02E-14
ENSG00000257829.1	AC121757.1	2.452732898	2.51278977	0.003071
ENSG00000141574.7	SECTM1	2.451338958	16.88876142	1.29E-17
ENSG00000170379.20	TCAF2	2.45013696	7.89419509	1.28E-08
ENSG00000089127.12	OAS1	2.449539798	18.65586174	2.21E-19
ENSG00000273183.1	AC093726.2	2.447543697	1.425833393	0.037512
ENSG00000187653.11	TMSB4XP8	2.447099295	6.725809897	1.88E-07
ENSG00000204959.4	ARHGEF34P	2.446781492	2.001969739	0.009955
ENSG00000149243.15	KLHL35	2.443828695	6.896294235	1.27E-07
ENSG00000237773.6	AC073332.1	2.443807621	5.100826737	7.93E-06
ENSG00000284186.1	MIR3615	2.443168985	20.6602003	2.19E-21
ENSG00000132965.9	ALOX5AP	2.440639287	17.20610614	6.22E-18
ENSG00000064218.4	DMRT3	2.440611124	3.585027286	0.00026
ENSG00000124253.10	PCK1	2.439985278	2.071563772	0.008481
ENSG00000284734.1	AC099063.4	2.439979899	16.21216646	6.14E-17
ENSG00000155629.14	PIK3AP1	2.437894843	10.14002969	7.24E-11
ENSG00000042493.15	CAPG	2.436775294	27.41687192	3.83E-28
ENSG00000272625.1	AP000919.4	2.436544162	4.903016533	1.25E-05
ENSG00000273472.1	AC096733.2	2.435156453	1.392505385	0.040504
ENSG00000145779.7	TNFAIP8	2.433975106	25.65100415	2.23E-26
ENSG00000086300.15	SNX10	2.429796759	18.31365268	4.86E-19
ENSG00000169583.12	CLIC3	2.42976193	1.978043327	0.010519
ENSG00000223358.5	EHHADH-AS1	2.428134507	1.594528067	0.025437
ENSG00000279933.1	AL031595.1	2.427841773	11.64097287	2.29E-12
ENSG00000111341.9	MGP	2.426261921	14.41824334	3.82E-15
ENSG00000277511.1	AC116407.2	2.425593596	4.099155553	7.96E-05
ENSG00000243811.9	APOBEC3D	2.425256726	18.39512653	4.03E-19
ENSG00000208024.1	MIR199A2	2.424985959	3.438199655	0.000365
ENSG00000237541.3	HLA-DQA2	2.423556894	4.874620292	1.33E-05
ENSG00000138435.15	CHRNA1	2.42217514	15.59742762	2.53E-16
ENSG00000183570.16	PCBP3	2.419973948	4.558956718	2.76E-05
ENSG00000173198.5	CYSLTR1	2.417973414	7.731382158	1.86E-08
ENSG00000141506.13	PIK3R5	2.417764387	11.10099343	7.93E-12

ENSG00000206028.1	Z99774.1	2.415967909	6.297662571	5.04E-07
ENSG00000203279.3	AL590705.1	2.4158098	1.838858256	0.014492
ENSG00000138964.16	PARVG	2.414782296	15.58621699	2.59E-16
ENSG00000169515.6	CCDC8	2.414372209	21.31623144	4.83E-22
ENSG00000111801.15	BTN3A3	2.41435378	40.78088232	1.66E-41
ENSG00000169896.17	ITGAM	2.413199	23.11996081	7.59E-24
ENSG00000105609.16	LILRB5	2.409488153	27.07272326	8.46E-28
ENSG00000157303.10	SUSD3	2.408508137	3.082960645	0.000826
ENSG00000105851.10	PIK3CG	2.407418474	7.733746281	1.85E-08
ENSG00000269107.1	AC092329.1	2.406976186	1.906114484	0.012413
ENSG00000140749.8	IGSF6	2.406433718	15.08380671	8.25E-16
ENSG00000115828.16	QPCT	2.405244552	5.138710111	7.27E-06
ENSG00000234235.1	BOK-AS1	2.405058273	5.310913525	4.89E-06
ENSG00000260549.1	MT1L	2.40430819	1.408084075	0.039077
ENSG00000213801.4	ZNF321P	2.404287377	6.736234668	1.84E-07
ENSG00000159251.7	ACTC1	2.404128831	9.086396955	8.20E-10
ENSG00000262678.1	AC004771.5	2.404071548	1.512460485	0.030728
ENSG00000205744.9	DENND1C	2.403852057	10.08795312	8.17E-11
ENSG00000262223.7	AC110285.1	2.403162928	1.628554837	0.02352
ENSG00000253931.1	AC105118.1	2.402061401	2.427464061	0.003737
ENSG00000284633.1	AL031590.1	2.401755319	1.645412509	0.022625
ENSG00000128604.19	IRF5	2.39669859	15.10368523	7.88E-16
ENSG00000162645.12	GBP2	2.395580095	37.27084799	5.36E-38
ENSG00000247416.3	AP000802.1	2.393504349	6.707222544	1.96E-07
ENSG00000180539.7	C9orf139	2.393463047	5.073282225	8.45E-06
ENSG00000185565.11	LSAMP	2.391499361	5.427806883	3.73E-06
ENSG00000256967.1	AC018653.3	2.390178087	6.808666065	1.55E-07
ENSG00000172575.11	RASGRP1	2.389026132	8.556600586	2.78E-09
ENSG00000134460.17	IL2RA	2.386859749	3.011056677	0.000975
ENSG00000271474.1	AC106881.1	2.386749239	1.328293756	0.046958
ENSG00000232855.6	AF165147.1	2.386172976	1.459976214	0.034676
ENSG00000260727.1	SLC7A5P1	2.386138685	1.764164487	0.017212
ENSG00000039537.13	C6	2.385404455	7.630264089	2.34E-08
ENSG00000274515.1	AC105020.5	2.38520281	1.305478104	0.049491
ENSG00000226576.1	AC060234.1	2.384541413	6.180749434	6.60E-07
ENSG00000184060.10	ADAP2	2.383162218	21.73601932	1.84E-22
ENSG00000198673.10	FAM19A2	2.382892694	2.169942409	0.006762
ENSG00000276649.1	AL117335.1	2.381833481	1.607734841	0.024675
ENSG00000140285.9	FGF7	2.38121716	18.11392187	7.69E-19
ENSG00000239922.1	AC092958.1	2.380934565	3.056498392	0.000878
ENSG00000143494.15	VASH2	2.380073507	19.16088178	6.90E-20
ENSG00000177337.7	DLGAP1-AS1	2.379837211	17.28492374	5.19E-18
ENSG00000164647.8	STEAP1	2.379533232	3.895920952	0.000127
ENSG00000269054.1	AC012313.6	2.378415375	1.801164558	0.015806
ENSG00000198502.5	HLA-DRB5	2.377813915	16.18414309	6.54E-17
ENSG00000101825.7	MXRA5	2.375068452	17.12067778	7.57E-18
ENSG00000146192.14	FGD2	2.374271956	23.19167042	6.43E-24

ENSG00000182993.4	C12orf60	2.373960871	13.88702094	1.30E-14
ENSG00000255629.1	AC025576.1	2.372240219	2.691631851	0.002034
ENSG00000100505.13	TRIM9	2.370654719	13.19625852	6.36E-14
ENSG00000262312.2	AC004494.1	2.370071972	4.1173401	7.63E-05
ENSG00000229323.1	DLEU1-AS1	2.368648797	1.567239644	0.027087
ENSG00000171659.14	GPR34	2.368425583	9.280150387	5.25E-10
ENSG00000236772.1	AL034550.1	2.366949948	3.35530787	0.000441
ENSG00000109944.10	JHY	2.366757386	4.642131445	2.28E-05
ENSG00000159403.16	C1R	2.366523873	24.71002461	1.95E-25
ENSG00000247081.7	BAALC-AS1	2.366107039	2.475408365	0.003347
ENSG00000174370.9	C11orf45	2.362294082	2.857545607	0.001388
ENSG00000267898.1	AC026803.2	2.362250453	1.571928713	0.026796
ENSG00000249328.2	AC036214.1	2.362010409	1.462650581	0.034463
ENSG00000228784.8	LINC00954	2.361914445	2.925719184	0.001187
ENSG00000187801.14	ZFP69B	2.359820954	3.006781268	0.000985
ENSG00000093072.16	ADA2	2.358308419	27.44403313	3.60E-28
ENSG00000123219.12	CENPK	2.35818075	3.464182356	0.000343
ENSG00000140853.15	NLRC5	2.356956997	28.5324539	2.93E-29
ENSG00000233608.3	TWIST2	2.356001508	3.804353754	0.000157
ENSG00000179057.13	IGSF22	2.354139306	6.095129895	8.03E-07
ENSG00000111181.12	SLC6A12	2.353714331	2.308008567	0.00492
ENSG00000227620.4	ALG1L8P	2.352841221	1.769212588	0.017013
ENSG00000266923.1	AC063949.2	2.351271499	5.192639836	6.42E-06
ENSG00000273188.1	AL022328.3	2.350775963	1.328451569	0.046941
ENSG00000165140.10	FBP1	2.349880729	16.36515663	4.31E-17
ENSG00000171303.6	KCNK3	2.346227778	2.762455038	0.001728
ENSG00000124920.13	MYRF	2.345580662	3.962230191	0.000109
ENSG00000232815.1	DUX4L50	2.345483032	2.644205389	0.002269
ENSG00000153064.11	BANK1	2.344886044	3.32404801	0.000474
ENSG00000107130.9	NCS1	2.342538069	43.17628434	6.66E-44
ENSG00000277117.4	FP565260.3	2.342473611	3.506268441	0.000312
ENSG00000169884.13	WNT10B	2.34228639	4.421958905	3.78E-05
ENSG00000141968.7	VAV1	2.341645921	12.52560785	2.98E-13
ENSG00000182782.7	HCAR2	2.340285504	1.793695019	0.016081
ENSG00000164520.11	RAET1E	2.338136679	3.239981856	0.000575
ENSG00000126759.13	CFP	2.338135336	10.44419248	3.60E-11
ENSG00000203710.11	CR1	2.335844058	5.187099613	6.50E-06
ENSG00000174599.5	TRAM1L1	2.335571343	5.05133035	8.89E-06
ENSG00000259755.1	AC090907.2	2.333547888	7.916442275	1.21E-08
ENSG00000106565.17	TMEM176B	2.330963949	23.38826881	4.09E-24
ENSG00000235568.6	NFAM1	2.33069459	7.964402649	1.09E-08
ENSG00000072694.20	FCGR2B	2.329908256	11.14567098	7.15E-12
ENSG00000023445.13	BIRC3	2.328509209	33.7443425	1.80E-34
ENSG00000112149.9	CD83	2.328283213	16.21573918	6.09E-17
ENSG00000265907.1	AP000919.2	2.328107187	5.221560918	6.00E-06
ENSG00000148344.10	PTGES	2.328024342	8.437027548	3.66E-09
ENSG00000127249.14	ATP13A4	2.327061921	1.463702782	0.034379

ENSG00000267416.1	AC025048.4	2.32567116	5.603027631	2.49E-06
ENSG00000125347.13	IRF1	2.325432626	29.15915363	6.93E-30
ENSG00000105464.3	GRIN2D	2.321492897	3.269863182	0.000537
ENSG00000204632.11	HLA-G	2.321073937	11.27410731	5.32E-12
ENSG00000244482.10	LILRA6	2.320040815	7.392812396	4.05E-08
ENSG00000087237.11	CETP	2.319953179	3.878614819	0.000132
ENSG00000164188.8	RANBP3L	2.319783585	2.172401343	0.006724
ENSG00000179071.4	CCDC89	2.319701862	3.696303838	0.000201
ENSG00000204659.4	CBY3	2.319380088	2.486226742	0.003264
ENSG00000177989.13	ODF3B	2.318585939	24.0562733	8.78E-25
ENSG00000182584.4	ACTL10	2.317795863	2.948162355	0.001127
ENSG00000234028.3	AC062029.1	2.317433529	3.896637737	0.000127
ENSG00000135773.12	CAPN9	2.317135052	2.166954081	0.006808
ENSG00000229391.7	HLA-DRB6	2.316916502	15.16769488	6.80E-16
ENSG00000234518.2	PTGES3P1	2.315266147	24.45792919	3.48E-25
ENSG00000109062.11	SLC9A3R1	2.314686461	33.5579887	2.77E-34
ENSG00000110876.9	SELPLG	2.314459619	12.64485929	2.27E-13
ENSG00000187608.9	ISG15	2.312705413	14.65445451	2.22E-15
ENSG00000007968.6	E2F2	2.312179107	2.794178172	0.001606
ENSG00000204257.14	HLA-DMA	2.310806009	30.24641596	5.67E-31
ENSG00000183773.15	AIFM3	2.31044892	2.931611424	0.001171
ENSG00000130876.11	SLC7A10	2.309702178	2.740937503	0.001816
ENSG00000147255.18	IGSF1	2.308234086	6.931112082	1.17E-07
ENSG00000026950.16	BTN3A1	2.307536287	32.59383837	2.55E-33
ENSG00000253958.1	CLDN23	2.307212441	5.896091393	1.27E-06
ENSG00000161682.14	FAM171A2	2.306001696	6.90529221	1.24E-07
ENSG00000116690.12	PRG4	2.305816592	6.932475167	1.17E-07
ENSG00000168004.9	HRASLS5	2.304866616	4.053294926	8.85E-05
ENSG00000143228.12	NUF2	2.304529775	3.960723439	0.000109
ENSG00000139734.18	DIAPH3	2.304202766	1.60110008	0.025055
ENSG00000184613.10	NELL2	2.302698068	4.692206904	2.03E-05
ENSG00000250007.6	AC087457.1	2.301693769	2.166823342	0.00681
ENSG00000196172.9	ZNF681	2.301037367	2.71646366	0.001921
ENSG00000157999.5	ANKRD61	2.300041497	1.648066265	0.022487
ENSG00000162777.16	DENND2D	2.299845644	11.04472745	9.02E-12
ENSG00000163624.5	CDS1	2.29983009	2.609733794	0.002456
ENSG00000242337.5	TFP1	2.299181019	1.860145832	0.013799
ENSG00000100092.22	SH3BP1	2.298222243	17.98616255	1.03E-18
ENSG00000123454.11	DBH	2.298006153	4.949165022	1.12E-05
ENSG00000080293.9	SCTR	2.295063148	1.973724617	0.010624
ENSG00000165259.13	HDX	2.295032278	7.719546101	1.91E-08
ENSG00000188305.5	PEAK3	2.293251234	2.025192651	0.009436
ENSG00000134183.11	GNAT2	2.292221982	3.366413276	0.00043
ENSG00000272482.1	AC254633.1	2.290788564	3.603424744	0.000249
ENSG00000112936.18	C7	2.290120472	8.475194303	3.35E-09
ENSG00000176194.17	CIDEA	2.289138746	2.58488194	0.002601
ENSG00000035499.12	DEPDC1B	2.288590734	1.416344936	0.03834

ENSG00000203706.8	SERTAD4-AS1	2.28743239	3.00752579	0.000983
ENSG00000182013.17	PNMA8A	2.285191045	15.32419906	4.74E-16
ENSG00000246575.2	AC093162.2	2.283052435	4.373558804	4.23E-05
ENSG00000128340.14	RAC2	2.282280966	14.23418413	5.83E-15
ENSG00000170909.13	OSCAR	2.282001133	4.409243492	3.90E-05
ENSG00000128606.12	LRRC17	2.281977568	18.46617752	3.42E-19
ENSG00000204516.9	MICB	2.28171459	11.6478734	2.25E-12
ENSG00000153012.11	LGI2	2.281600956	12.21192822	6.14E-13
ENSG00000002933.8	TMEM176A	2.280741339	25.9167088	1.21E-26
ENSG00000179388.8	EGR3	2.279479641	7.548239337	2.83E-08
ENSG00000181790.11	ADGRB1	2.278940107	6.264461954	5.44E-07
ENSG00000217648.1	AL136116.3	2.278008918	2.181566489	0.006583
ENSG00000162654.8	GBP4	2.276872367	24.63130076	2.34E-25
ENSG00000181291.7	TMEM132E	2.275916247	2.466535974	0.003416
ENSG00000278921.2	EPB41L4A-DT	2.275661665	3.239889571	0.000576
ENSG00000224905.6	AP001347.1	2.274511653	1.408700546	0.039021
ENSG00000226482.1	ADIPOQ-AS1	2.274172721	4.276132474	5.30E-05
ENSG00000186235.10	AC016757.1	2.273291433	1.473757089	0.033593
ENSG00000100055.20	CYTH4	2.273263159	21.16671596	6.81E-22
ENSG00000206341.7	HLA-H	2.272579223	12.46577712	3.42E-13
ENSG00000184661.13	CDCA2	2.27237707	1.412332905	0.038696
ENSG00000165949.12	IFI27	2.271038592	17.9210535	1.20E-18
ENSG00000221986.6	MYBPHL	2.270570728	2.63149461	0.002336
ENSG00000043462.11	LCP2	2.268360688	20.06362208	8.64E-21
ENSG00000119632.3	IFI27L2	2.26831874	30.78429172	1.64E-31
ENSG00000196209.12	SIRPB2	2.26510221	5.993866322	1.01E-06
ENSG00000261779.1	AC113208.3	2.2641534	1.653396585	0.022213
ENSG00000161149.12	TUBA3FP	2.262973206	4.362602732	4.34E-05
ENSG00000105639.18	JAK3	2.262521449	10.70366271	1.98E-11
ENSG00000103490.13	PYCARD	2.260697714	24.57828881	2.64E-25
ENSG00000163710.8	PCOLCE2	2.259147708	6.456496296	3.50E-07
ENSG00000059377.16	TBXAS1	2.2584978	14.22997867	5.89E-15
ENSG00000250318.1	AC003072.1	2.258434657	3.666084216	0.000216
ENSG00000198246.8	SLC29A3	2.258183833	12.38275111	4.14E-13
ENSG00000075884.13	ARHGAP15	2.256617692	8.45764325	3.49E-09
ENSG00000118513.18	MYB	2.252899625	1.390206685	0.040719
ENSG00000104783.12	KCNN4	2.251838616	11.58528729	2.60E-12
ENSG00000279036.1	AC015656.1	2.251539801	3.242443276	0.000572
ENSG00000279265.1	AC000123.2	2.250328699	1.709703066	0.019512
ENSG00000182508.13	LHFPL1	2.250326673	1.802592202	0.015755
ENSG00000177551.5	NHLH2	2.250285363	6.156156209	6.98E-07
ENSG00000164099.3	PRSS12	2.249521155	5.185881866	6.52E-06
ENSG00000221963.5	APOL6	2.247539198	38.87927395	1.32E-39
ENSG00000261448.1	AC109446.3	2.244789855	1.547471788	0.028348
ENSG00000250234.1	AC025754.1	2.244700478	1.801380335	0.015799
ENSG00000181631.6	P2RY13	2.244019722	4.439732535	3.63E-05
ENSG00000204622.11	HLA-J	2.240991661	11.35242358	4.44E-12

ENSG00000111339.11	ART4	2.239829441	1.350745104	0.044592
ENSG00000154102.10	C16orf74	2.239597355	1.548895805	0.028256
ENSG00000165025.14	SYK	2.238734676	17.47372024	3.36E-18
ENSG00000138795.9	LEF1	2.237491283	2.328719367	0.004691
ENSG00000237276.8	ANO7L1	2.237444964	5.420324242	3.80E-06
ENSG00000169403.11	PTAFR	2.233175342	14.15879041	6.94E-15
ENSG00000269918.1	AF131215.6	2.232804552	5.116856023	7.64E-06
ENSG00000115884.10	SDC1	2.23180353	11.34015759	4.57E-12
ENSG00000139155.8	SLCO1C1	2.231750152	1.325478048	0.047263
ENSG00000182578.13	CSF1R	2.231482318	20.47553941	3.35E-21
ENSG00000136052.9	SLC41A2	2.230925859	10.8562628	1.39E-11
ENSG00000280202.1	AC005831.1	2.230638912	3.501520208	0.000315
ENSG00000225177.5	FLJ46906	2.229504188	4.490842925	3.23E-05
ENSG00000267731.1	AC005332.2	2.229258278	2.095034524	0.008035
ENSG00000244479.7	OR2A1-AS1	2.22778357	2.309861398	0.004899
ENSG00000171596.6	NMUR1	2.225378101	1.975787325	0.010573
ENSG00000258839.3	MC1R	2.222339372	17.68530165	2.06E-18
ENSG00000158517.13	NCF1	2.221561611	9.993609196	1.01E-10
ENSG00000261359.2	PYCARD-AS1	2.221559438	24.00400055	9.91E-25
ENSG00000176998.4	HCG4	2.218448553	2.155230721	0.006995
ENSG00000168490.13	PHYHIP	2.217031588	2.015713378	0.009645
ENSG00000281404.1	LINC01176	2.216840815	4.809588355	1.55E-05
ENSG00000102096.9	PIM2	2.216585563	17.77664021	1.67E-18
ENSG00000267270.5	PARD6G-AS1	2.215526704	1.878032255	0.013242
ENSG00000138316.10	ADAMTS14	2.215294466	6.95206492	1.12E-07
ENSG00000266495.1	AC011731.1	2.214855823	4.110143498	7.76E-05
ENSG00000264633.1	MIR4271	2.214440693	3.469922315	0.000339
ENSG00000116711.9	PLA2G4A	2.214429361	8.058135256	8.75E-09
ENSG00000121797.9	CCRL2	2.213302369	7.144953351	7.16E-08
ENSG00000117226.11	GBP3	2.211917004	20.60451693	2.49E-21
ENSG00000180353.10	HCLS1	2.211163148	19.59397415	2.55E-20
ENSG00000054219.10	LY75	2.210845372	9.70834766	1.96E-10
ENSG00000134955.11	SLC37A2	2.207614754	19.15219785	7.04E-20
ENSG00000197471.11	SPN	2.206337218	12.67622372	2.11E-13
ENSG00000125637.15	PSD4	2.204564146	18.63713473	2.31E-19
ENSG00000132205.10	EMILIN2	2.201764422	15.37093872	4.26E-16
ENSG00000118849.9	RARRES1	2.201752341	8.321287432	4.77E-09
ENSG00000124915.10	DKFZP434K028	2.199319381	2.209776889	0.006169
ENSG00000185274.11	GALNT17	2.196782476	17.36778838	4.29E-18
ENSG00000026508.18	CD44	2.196019156	28.83319932	1.47E-29
ENSG00000264739.1	AC093484.2	2.193874552	4.198999991	6.32E-05
ENSG00000186496.11	ZNF396	2.193803997	4.291010852	5.12E-05
ENSG00000175463.11	TBC1D10C	2.193583821	14.19037554	6.45E-15
ENSG00000272717.1	AC112236.2	2.193040046	2.601198414	0.002505
ENSG00000259953.1	AL138756.1	2.19239913	5.625482159	2.37E-06
ENSG00000266405.3	CBX3P2	2.189925303	2.008239038	0.009812
ENSG00000172183.14	ISG20	2.189432324	13.07796667	8.36E-14

ENSG00000130775.15	THEMIS2	2.188876029	16.83257992	1.47E-17
ENSG00000214223.4	HNRNPA1P10	2.186581294	1.33313587	0.046437
ENSG00000269460.1	AC005515.1	2.185737807	3.777361203	0.000167
ENSG00000227484.1	CR559946.1	2.18331646	2.408576124	0.003903
ENSG00000172243.17	CLEC7A	2.181882168	7.111338779	7.74E-08
ENSG00000108846.15	ABCC3	2.179606002	11.9884449	1.03E-12
ENSG00000047457.13	CP	2.177680464	20.28886093	5.14E-21
ENSG00000167767.13	KRT80	2.177050971	6.226884065	5.93E-07
ENSG00000266573.5	AC018697.1	2.175825185	1.708321048	0.019574
ENSG00000115353.10	TACR1	2.172429028	5.04730242	8.97E-06
ENSG00000042980.12	ADAM28	2.169605615	4.807547841	1.56E-05
ENSG00000143344.15	RGL1	2.167947935	21.79301161	1.61E-22
ENSG00000276753.1	MIR6821	2.167850159	6.915893017	1.21E-07
ENSG00000229692.3	SOS1-IT1	2.167748878	1.329260583	0.046853
ENSG00000181218.5	HIST3H2A	2.167466416	7.199862313	6.31E-08
ENSG00000116701.14	NCF2	2.166545873	10.11626981	7.65E-11
ENSG00000126467.10	TSKS	2.166145131	3.287849872	0.000515
ENSG00000230521.1	AL645929.1	2.164693464	3.782532183	0.000165
ENSG00000285671.1	AL139405.1	2.163803821	1.33878957	0.045836
ENSG00000175985.9	PLEKHD1	2.161891644	1.645909222	0.022599
ENSG00000197813.5	AC011450.1	2.160877571	8.68529644	2.06E-09
ENSG00000124508.16	BTN2A2	2.159444897	30.8664873	1.36E-31
ENSG00000278238.1	AL359513.1	2.159258631	1.95940018	0.01098
ENSG00000130208.9	APOC1	2.157052885	14.54709177	2.84E-15
ENSG00000182326.14	C1S	2.156377518	24.25196816	5.60E-25
ENSG00000022556.15	NLRP2	2.15356628	2.269382676	0.005378
ENSG00000102265.11	TIMP1	2.153321758	22.29770247	5.04E-23
ENSG00000120075.5	HOXB5	2.152983566	2.822357599	0.001505
ENSG00000091986.15	CCDC80	2.152471982	13.902288	1.25E-14
ENSG00000056998.19	GYG2	2.151687078	3.430208931	0.000371
ENSG00000181092.9	ADIPOQ	2.148925973	3.891062832	0.000129
ENSG00000271746.1	AL031848.2	2.148740498	2.225686599	0.005947
ENSG00000171310.10	CHST11	2.147771618	15.03466052	9.23E-16
ENSG00000273771.1	AC024337.2	2.147641941	2.38889865	0.004084
ENSG00000273112.1	AL590385.2	2.14606822	9.430038786	3.72E-10
ENSG00000213145.9	CRIP1	2.145527118	16.95137774	1.12E-17
ENSG00000205054.7	LINC01121	2.144930918	2.697104968	0.002009
ENSG00000163106.10	HPGDS	2.144540074	6.402850344	3.96E-07
ENSG00000250011.1	HMGB1P3	2.14272877	4.609491104	2.46E-05
ENSG00000231991.4	ANXA2P2	2.142391888	6.330634363	4.67E-07
ENSG00000280087.1	AC011481.3	2.142235843	15.80294497	1.57E-16
ENSG00000262179.2	MYMX	2.141178347	1.627877508	0.023557
ENSG00000183822.3	NCF4-AS1	2.139367602	4.391565496	4.06E-05
ENSG00000272324.5	AC012629.2	2.138399112	4.093201716	8.07E-05
ENSG00000184811.3	TRARG1	2.138352189	3.740260496	0.000182
ENSG00000167895.14	TMC8	2.137271773	12.81226986	1.54E-13
ENSG00000171101.13	SIGLEC17P	2.137176177	1.638339691	0.022996

ENSG00000143226.13	FCGR2A	2.136993016	9.834996964	1.46E-10
ENSG00000049089.14	COL9A2	2.136817779	6.100591765	7.93E-07
ENSG00000077420.15	APBB1IP	2.135621065	11.29082652	5.12E-12
ENSG00000117266.15	CDK18	2.135436988	22.42912916	3.72E-23
ENSG00000268895.5	A1BG-AS1	2.135292329	11.54742582	2.84E-12
ENSG00000164694.16	FNDC1	2.132376581	9.582656109	2.61E-10
ENSG00000011590.13	ZBTB32	2.130067355	1.359176228	0.043734
ENSG00000259668.5	AC066613.1	2.129592632	6.829402933	1.48E-07
ENSG00000198216.11	CACNA1E	2.121158748	2.422283995	0.003782
ENSG00000259250.1	AC018904.1	2.118082453	1.75591212	0.017542
ENSG00000179270.6	PCARE	2.116968677	1.364408596	0.043211
ENSG00000184221.12	OLIG1	2.116906747	4.247570693	5.65E-05
ENSG00000128394.16	APOBEC3F	2.116873972	16.10832481	7.79E-17
ENSG00000185015.7	CA13	2.114132925	4.530726795	2.95E-05
ENSG00000124343.13	XG	2.113674008	10.09261502	8.08E-11
ENSG00000215483.10	LINC00598	2.113656067	3.596611047	0.000253
ENSG00000023892.10	DEF6	2.113022364	14.29467946	5.07E-15
ENSG00000253392.2	AC119403.1	2.112376056	3.314706279	0.000485
ENSG00000273456.1	AC064836.3	2.112352277	3.933662131	0.000117
ENSG00000183160.8	TMEM119	2.110930065	16.27570725	5.30E-17
ENSG00000279355.1	AGPAT4-IT1	2.110197734	1.880758127	0.01316
ENSG00000108679.12	LGALS3BP	2.109003832	31.67845718	2.10E-32
ENSG00000197099.8	AC068631.1	2.107107264	2.585397931	0.002598
ENSG00000110900.14	TSPAN11	2.106448899	6.682855339	2.08E-07
ENSG00000131080.14	EDA2R	2.105114076	15.10962745	7.77E-16
ENSG00000103528.16	SYT17	2.104615035	4.751824255	1.77E-05
ENSG00000261329.5	AC016597.1	2.104577968	2.757571543	0.001748
ENSG00000265806.1	MIR4292	2.103968998	2.373605018	0.004231
ENSG00000125730.16	C3	2.102976559	21.51036477	3.09E-22
ENSG00000255733.5	IFNG-AS1	2.102535093	1.65981766	0.021887
ENSG00000257027.1	AC010186.3	2.101730299	2.487323913	0.003256
ENSG00000152495.10	CAMK4	2.101152837	3.935840556	0.000116
ENSG00000197415.11	VEPH1	2.100654595	1.517505283	0.030373
ENSG00000186105.7	LRRC70	2.09924312	2.52247588	0.003003
ENSG00000139329.4	LUM	2.099020768	9.881547624	1.31E-10
ENSG00000179528.15	LBX2	2.097533323	6.524525369	2.99E-07
ENSG00000122121.10	XPNPEP2	2.096887713	5.879982916	1.32E-06
ENSG00000227986.1	TRIM60P18	2.09595532	2.332942748	0.004646
ENSG00000280498.1	SNORA16A	2.095894442	2.496797813	0.003186
ENSG00000133317.14	LGALS12	2.091738312	1.458999866	0.034754
ENSG00000179715.12	PCED1B	2.090964784	13.81634833	1.53E-14
ENSG00000128262.8	POM121L9P	2.089571713	1.703827338	0.019778
ENSG00000280721.1	LINC01943	2.088994953	17.02239975	9.50E-18
ENSG00000101194.17	SLC17A9	2.087899967	10.06816787	8.55E-11
ENSG00000278962.1	AC092645.1	2.086244062	3.874171913	0.000134
ENSG00000274092.1	AC106739.1	2.085765625	3.175526342	0.000668
ENSG00000250740.1	AC109361.2	2.085041645	4.01950149	9.56E-05

ENSG00000272432.1	AL031432.3	2.085011761	1.403186417	0.03952
ENSG00000151650.7	VENTX	2.084143218	7.619316375	2.40E-08
ENSG00000279693.1	AC099521.2	2.082751604	2.896967312	0.001268
ENSG00000104043.14	ATP8B4	2.081154902	13.48428917	3.28E-14
ENSG00000133937.4	GSC	2.079649684	3.959175263	0.00011
ENSG00000103187.7	COTL1	2.077232817	19.85691823	1.39E-20
ENSG00000198626.15	RYR2	2.075879942	2.875347809	0.001332
ENSG00000170509.11	HSD17B13	2.075844231	1.559957904	0.027545
ENSG00000255443.1	CD44-AS1	2.075617114	15.08500382	8.22E-16
ENSG00000174871.10	CNIH2	2.073852465	1.524278916	0.029903
ENSG00000231205.11	ZNF826P	2.073713531	4.493504771	3.21E-05
ENSG00000196666.4	FAM180B	2.07338668	7.080652502	8.31E-08
ENSG00000146386.7	ABRACL	2.07300646	11.37152421	4.25E-12
ENSG00000102032.12	RENBP	2.071931603	18.68318247	2.07E-19
ENSG00000224259.6	LINC01133	2.071731356	3.307638113	0.000492
ENSG00000097046.12	CDC7	2.071188988	7.729323129	1.86E-08
ENSG00000285585.1	AC069444.2	2.069636943	2.560896778	0.002749
ENSG00000237940.3	LINC01238	2.068923127	3.084888426	0.000822
ENSG00000257341.5	AL928654.3	2.068262074	16.78246684	1.65E-17
ENSG00000127083.7	OMD	2.06811813	8.444534904	3.59E-09
ENSG00000087076.8	HSD17B14	2.067720542	8.62331751	2.38E-09
ENSG00000177300.6	CLDN22	2.067382729	2.469360571	0.003393
ENSG00000230596.3	GPAA1P2	2.067118283	1.551470965	0.028089
ENSG00000273471.1	AC112229.4	2.063655579	1.668125911	0.021472
ENSG00000277287.1	AL109976.1	2.063030838	1.342423725	0.045454
ENSG00000214102.7	WEE2	2.062518626	2.814801984	0.001532
ENSG00000239264.8	TXNDC5	2.062132636	14.06133054	8.68E-15
ENSG00000127585.11	FBXL16	2.061386322	4.194439801	6.39E-05
ENSG00000187164.19	SHTN1	2.061339038	20.89852557	1.26E-21
ENSG00000225783.7	MIAT	2.059487552	13.68056501	2.09E-14
ENSG00000155465.18	SLC7A7	2.057897315	17.50737781	3.11E-18
ENSG00000275395.5	FCGBP	2.056916301	5.655779386	2.21E-06
ENSG00000233483.3	AC008105.2	2.056847492	8.573331333	2.67E-09
ENSG00000091129.19	NRCAM	2.056116228	8.82606771	1.49E-09
ENSG00000127951.6	FGL2	2.055254579	18.72554414	1.88E-19
ENSG00000091490.10	SEL1L3	2.054903939	15.63968396	2.29E-16
ENSG00000225217.1	HSPA7	2.051787142	12.86466564	1.37E-13
ENSG00000162482.4	AKR7A3	2.05165104	2.991449048	0.00102
ENSG00000185567.6	AHNAK2	2.04944778	10.84577487	1.43E-11
ENSG00000015285.10	WAS	2.049272021	12.61817728	2.41E-13
ENSG00000139178.10	C1RL	2.049062892	21.97975049	1.05E-22
ENSG00000135905.19	DOCK10	2.047452656	19.44041353	3.63E-20
ENSG00000066336.11	SPI1	2.045689915	14.36321307	4.33E-15
ENSG00000131042.14	LILRB2	2.044539854	11.70235716	1.98E-12
ENSG00000100351.16	GRAP2	2.040567531	5.095241621	8.03E-06
ENSG00000177106.15	EPS8L2	2.040004741	22.55118525	2.81E-23
ENSG00000101938.14	CHRDL1	2.039032584	10.65832842	2.20E-11

ENSG00000241572.1	PRICKLE2-AS1	2.038013906	1.98819055	0.010276
ENSG00000230795.3	HLA-K	2.038004257	11.65671356	2.20E-12
ENSG00000065361.15	ERBB3	2.037277514	8.692238916	2.03E-09
ENSG00000213928.8	IRF9	2.03647998	35.52457395	2.99E-36
ENSG00000139182.14	CLSTN3	2.03614238	29.68281725	2.08E-30
ENSG00000238279.1	BX470102.1	2.036090988	2.945209232	0.001134
ENSG00000228397.2	LINC01635	2.035081156	1.745858002	0.017953
ENSG00000164932.12	CTHRC1	2.03453798	8.487578548	3.25E-09
ENSG00000174600.13	CMKLR1	2.033930188	26.94517626	1.13E-27
ENSG00000162931.11	TRIM17	2.032102658	2.133973409	0.007346
ENSG00000196407.11	THEM5	2.032019817	3.45623923	0.00035
ENSG00000224220.1	AC104699.1	2.031338228	2.539068402	0.00289
ENSG00000125510.15	OPRL1	2.030636992	5.968879123	1.07E-06
ENSG00000162630.5	B3GALT2	2.030517482	2.805433701	0.001565
ENSG00000197140.14	ADAM32	2.029748445	2.91056491	0.001229
ENSG00000073598.5	FNDC8	2.029625173	1.487639308	0.032536
ENSG00000029153.14	ARNTL2	2.029383316	9.740761019	1.82E-10
ENSG00000064205.10	WISP2	2.029098982	9.322960506	4.75E-10
ENSG00000141655.16	TNFRSF11A	2.028748947	5.989539867	1.02E-06
ENSG00000250264.1	AL669918.1	2.027028532	27.70240744	1.98E-28
ENSG00000285545.1	AC124798.2	2.026114945	1.54925694	0.028232
ENSG00000237181.1	AC147651.3	2.023522673	4.340018061	4.57E-05
ENSG00000047365.11	ARAP2	2.021819354	19.96364294	1.09E-20
ENSG00000125657.4	TNFSF9	2.02094365	3.643361671	0.000227
ENSG00000068079.7	IFI35	2.019839518	28.4823884	3.29E-29
ENSG00000163040.14	CCDC74A	2.019597525	2.892059619	0.001282
ENSG00000173698.17	ADGRG2	2.019536087	3.757498935	0.000175
ENSG00000276975.3	HYDIN2	2.018183567	2.066119814	0.008588
ENSG00000113083.13	LOX	2.016888532	7.63283525	2.33E-08
ENSG00000166819.11	PLIN1	2.0168494	3.814674297	0.000153
ENSG00000197479.6	PCDHB11	2.016390535	4.722353224	1.90E-05
ENSG00000121904.17	CSMD2	2.01433244	1.582315641	0.026163
ENSG00000249906.1	AC006487.1	2.013647184	4.96484958	1.08E-05
ENSG00000003137.8	CYP26B1	2.011675744	15.0787395	8.34E-16
ENSG00000087116.15	ADAMTS2	2.01156614	10.94753805	1.13E-11
ENSG00000064692.18	SNCAIP	2.010886778	5.329638557	4.68E-06
ENSG00000180549.7	FUT7	2.010761364	1.911049396	0.012273
ENSG00000156414.18	TDRD9	2.009752666	1.514013238	0.030619
ENSG00000164488.11	DACT2	2.009451424	3.455272621	0.000351
ENSG00000134121.9	CHL1	2.005435313	6.793651805	1.61E-07
ENSG00000102048.15	ASB9	2.004397771	3.305988117	0.000494
ENSG00000204136.10	GGTA1P	2.003982605	16.47158352	3.38E-17
ENSG00000244682.7	FCGR2C	2.001262569	11.70988044	1.95E-12
ENSG00000172578.11	KLHL6	2.00115147	10.42293652	3.78E-11
ENSG00000267282.1	AC011481.2	2.001093505	1.85026066	0.014117
ENSG00000111679.16	PTPN6	2.000686416	16.45372574	3.52E-17
ENSG00000197249.13	SERPINA1	2.000199927	5.897715407	1.27E-06

ENSG00000271725.1	AC103858.1	2.000022938	9.071402191	8.48E-10
ENSG00000239257.1	RPL23AP1	1.999800898	15.16814939	6.79E-16
ENSG00000227051.6	C14orf132	1.999421168	22.72763692	1.87E-23
ENSG00000285802.1	AL450043.1	1.997753197	1.553623813	0.02795
ENSG00000164691.17	TAGAP	1.996815481	8.894989149	1.27E-09
ENSG00000135318.11	NT5E	1.996420569	10.67769839	2.10E-11
ENSG00000101336.13	HCK	1.996374683	8.806891763	1.56E-09
ENSG00000178078.11	STAP2	1.996131055	8.310950699	4.89E-09
ENSG00000130203.9	APOE	1.991057654	16.28195715	5.22E-17
ENSG00000108405.3	P2RX1	1.990212181	4.489472953	3.24E-05
ENSG00000162627.16	SNX7	1.989891549	23.67542057	2.11E-24
ENSG00000187288.10	CIDEC	1.989278015	2.939376857	0.00115
ENSG00000069188.16	SDK2	1.988072166	3.702662223	0.000198
ENSG00000258754.7	LINC01579	1.987508348	1.817028937	0.01524
ENSG00000197646.7	PDCD1LG2	1.987413569	11.5588246	2.76E-12
ENSG00000223396.4	RPS10P7	1.983597777	7.505378817	3.12E-08
ENSG00000216775.3	AL109918.1	1.983434238	6.611394166	2.45E-07
ENSG00000132334.16	PTPRE	1.983313301	21.57421959	2.67E-22
ENSG00000119714.10	GPR68	1.983182695	19.30674244	4.93E-20
ENSG00000131979.18	GCH1	1.983073084	17.73704856	1.83E-18
ENSG00000149294.16	NCAM1	1.982904591	19.86014522	1.38E-20
ENSG00000173193.14	PARP14	1.981088898	22.06100574	8.69E-23
ENSG00000254703.2	SENCR	1.97907479	2.027287763	0.009391
ENSG00000030304.13	MUSK	1.977733705	7.55314723	2.80E-08
ENSG00000186654.20	PRR5	1.977392975	10.41185403	3.87E-11
ENSG00000248405.10	PRR5-ARHGAP8	1.977377064	10.36126788	4.35E-11
ENSG00000102678.6	FGF9	1.976285166	6.973008862	1.06E-07
ENSG00000115738.9	ID2	1.975913524	30.47600128	3.34E-31
ENSG00000271218.1	AL033384.2	1.974374523	1.307003738	0.049317
ENSG00000135124.14	P2RX4	1.974359327	18.91235947	1.22E-19
ENSG00000143768.12	LEFTY2	1.973822266	1.614690077	0.024283
ENSG00000115607.9	IL18RAP	1.973023782	1.842562416	0.014369
ENSG00000273987.1	AC121761.2	1.97245497	1.780029105	0.016595
ENSG00000159713.10	TPPP3	1.971270027	14.82315868	1.50E-15
ENSG00000119922.9	IFIT2	1.969245673	17.50804936	3.10E-18
ENSG00000182667.14	NTM	1.968822598	9.331078299	4.67E-10
ENSG00000034053.14	APBA2	1.968656841	3.290664486	0.000512
ENSG00000111729.14	CLEC4A	1.968153275	4.100622649	7.93E-05
ENSG00000148803.11	FUOM	1.96797295	10.29341228	5.09E-11
ENSG00000180638.17	SLC47A2	1.964969843	5.068741739	8.54E-06
ENSG00000146232.16	NFKBIE	1.964272444	24.91616716	1.21E-25
ENSG00000186056.10	MATN1-AS1	1.960665391	1.327148187	0.047082
ENSG00000253930.1	TNFRSF10A-AS1	1.959651009	2.848967246	0.001416
ENSG00000143178.12	TBX19	1.95964363	6.562823205	2.74E-07
ENSG00000151883.17	PARP8	1.959610047	16.60663454	2.47E-17
ENSG00000137731.13	FXYD2	1.958921345	5.060759437	8.69E-06
ENSG00000279196.1	AC135048.4	1.95852026	30.49054036	3.23E-31

ENSG00000099377.13	HSD3B7	1.958416516	31.09825196	7.98E-32
ENSG00000139629.15	GALNT6	1.957645764	9.97386725	1.06E-10
ENSG00000162711.17	NLRP3	1.955947992	6.733920075	1.85E-07
ENSG00000196684.12	HSH2D	1.955830027	4.191854652	6.43E-05
ENSG00000163694.14	RBM47	1.954061944	11.09973427	7.95E-12
ENSG00000204764.13	RANBP17	1.953941803	1.678692299	0.020956
ENSG00000215417.12	MIR17HG	1.9535744	2.100687459	0.007931
ENSG00000172137.18	CALB2	1.952345557	1.677139577	0.021031
ENSG00000204267.13	TAP2	1.952345127	27.62060224	2.40E-28
ENSG00000196154.11	S100A4	1.952217692	19.27062871	5.36E-20
ENSG00000174125.7	TLR1	1.949762977	4.891194706	1.28E-05
ENSG00000009790.14	TRAF3IP3	1.949629997	13.28149492	5.23E-14
ENSG00000275004.3	ZNF280B	1.949575744	4.246365876	5.67E-05
ENSG00000204850.4	AC011484.1	1.949248298	3.038780746	0.000915
ENSG00000106823.12	ECM2	1.948651667	13.23879156	5.77E-14
ENSG00000146215.13	CRIP3	1.948118215	1.534857764	0.029184
ENSG00000186074.18	CD300LF	1.94723448	1.636771872	0.02308
ENSG00000128203.6	ASPHD2	1.946078257	5.656712836	2.20E-06
ENSG00000129538.13	RNASE1	1.943857498	14.60129551	2.50E-15
ENSG00000253540.5	FAM86HP	1.943805574	5.003355867	9.92E-06
ENSG00000205056.8	LINC02397	1.943327921	6.163787381	6.86E-07
ENSG00000137491.14	SLCO2B1	1.943304961	25.29675971	5.05E-26
ENSG00000203497.2	PDCD4-AS1	1.941777189	4.025921716	9.42E-05
ENSG00000137462.7	TLR2	1.940650016	4.632203097	2.33E-05
ENSG00000167604.14	NFKBID	1.938687299	8.191914268	6.43E-09
ENSG00000100368.13	CSF2RB	1.938357254	12.65431527	2.22E-13
ENSG00000244198.6	AC004889.1	1.938307305	1.533885473	0.029249
ENSG00000270099.1	AL365273.2	1.937900201	4.911637392	1.23E-05
ENSG00000159216.18	RUNX1	1.937509663	26.65702747	2.20E-27
ENSG00000280594.1	BTG3-AS1	1.937426241	3.25182153	0.00056
ENSG00000270578.1	AP000787.2	1.937116153	1.324185104	0.047404
ENSG00000236199.1	AL359076.1	1.936254972	2.323084543	0.004752
ENSG00000214787.9	MS4A4E	1.935871757	7.763201481	1.73E-08
ENSG00000274020.2	LINC01138	1.935607271	9.928747464	1.18E-10
ENSG00000279182.1	CR559946.2	1.935035853	3.218648893	0.000604
ENSG00000163449.10	TMEM169	1.934267302	4.573623548	2.67E-05
ENSG00000110628.14	SLC22A18	1.932765282	12.90796682	1.24E-13
ENSG00000181804.14	SLC9A9	1.932099752	23.0735739	8.44E-24
ENSG00000163357.10	DCST1	1.93208957	3.71521106	0.000193
ENSG00000160883.10	HK3	1.931628175	5.027055581	9.40E-06
ENSG00000267545.1	AC005779.2	1.93101604	3.50129463	0.000315
ENSG00000227262.3	HCG4B	1.930137542	6.160825893	6.91E-07
ENSG00000142227.10	EMP3	1.927000264	16.55655945	2.78E-17
ENSG00000235590.7	GNAS-AS1	1.925397616	5.147772107	7.12E-06
ENSG00000283703.2	VSIG10L2	1.924989082	10.24370523	5.71E-11
ENSG00000138496.16	PARP9	1.924742093	28.30643023	4.94E-29
ENSG00000269825.1	AC022150.4	1.92252804	1.971103783	0.010688

ENSG00000196811.11	CHRNG	1.92214227	7.124137949	7.51E-08
ENSG00000188112.8	C6orf132	1.916895474	4.110656374	7.75E-05
ENSG00000139832.4	RAB20	1.916870766	20.19147309	6.43E-21
ENSG00000276842.1	AC023510.2	1.916170418	1.549767215	0.028199
ENSG00000117122.13	MFAP2	1.915068355	7.459545931	3.47E-08
ENSG00000136235.16	GPNMB	1.914835928	11.72259083	1.89E-12
ENSG00000234465.10	PINLYP	1.914350766	4.114142362	7.69E-05
ENSG00000219529.2	AP000580.1	1.913468433	3.112883404	0.000771
ENSG00000161921.14	CXCL16	1.912188435	22.50519344	3.12E-23
ENSG00000259018.1	AL049829.2	1.911815028	3.290419193	0.000512
ENSG00000106948.16	AKNA	1.910913311	19.03331138	9.26E-20
ENSG00000235641.4	LINC00484	1.910603493	4.177488711	6.65E-05
ENSG00000224596.7	ZMIZ1-AS1	1.909742723	1.439821889	0.036323
ENSG00000188452.13	CERKL	1.909118919	5.318062287	4.81E-06
ENSG00000000971.15	CFH	1.908977024	16.90944548	1.23E-17
ENSG00000259040.5	BLOC1S5-TXNDC5	1.905899533	12.90302476	1.25E-13
ENSG00000244242.1	IFITM10	1.905656072	13.52052631	3.02E-14
ENSG00000160013.8	PTGIR	1.9036717	8.552557374	2.80E-09
ENSG00000110665.11	C11orf21	1.902761545	5.346338229	4.50E-06
ENSG00000205549.9	C9orf92	1.902177894	2.228753965	0.005905
ENSG00000138207.13	RBP4	1.900740764	2.641028128	0.002285
ENSG00000272913.1	AC009237.14	1.900413073	1.900852522	0.012565
ENSG00000181458.10	TMEM45A	1.900158138	9.889653237	1.29E-10
ENSG00000137841.11	PLCB2	1.899457995	13.98716076	1.03E-14
ENSG00000204397.7	CARD16	1.899306807	10.36329596	4.33E-11
ENSG00000110057.7	UNC93B1	1.898660428	19.77896873	1.66E-20
ENSG00000284121.1	MIR198	1.895256201	9.612324817	2.44E-10
ENSG00000283944.1	MIR4709	1.894674528	16.25064006	5.62E-17
ENSG00000171223.5	JUNB	1.892871378	15.59742762	2.53E-16
ENSG00000235501.5	AC105942.1	1.892141786	10.42783643	3.73E-11
ENSG00000116741.7	RGS2	1.891096621	12.24216063	5.73E-13
ENSG00000135046.13	ANXA1	1.891057939	16.26637178	5.42E-17
ENSG00000133800.8	LYVE1	1.890402701	7.123476824	7.53E-08
ENSG00000142149.8	HUNK	1.889792401	4.95518602	1.11E-05
ENSG00000076944.15	STXBP2	1.889539924	11.77834115	1.67E-12
ENSG00000225265.1	TAF1A-AS1	1.888775897	2.576742685	0.00265
ENSG00000186684.12	CYP27C1	1.888068276	3.253996799	0.000557
ENSG00000181126.13	HLA-V	1.887984386	4.041047747	9.10E-05
ENSG00000168743.12	NPNT	1.887157452	17.556078	2.78E-18
ENSG00000196136.17	SERPINA3	1.884114811	5.420130872	3.80E-06
ENSG00000130755.12	GMFG	1.883745892	11.18876184	6.47E-12
ENSG00000005059.15	MCUB	1.882342306	19.15680514	6.97E-20
ENSG00000138615.5	CILP	1.88226755	13.16522629	6.84E-14
ENSG00000167513.8	CDT1	1.882239251	4.01982772	9.55E-05
ENSG00000132688.10	NES	1.881818144	17.46724358	3.41E-18
ENSG00000268475.1	AC011462.2	1.881778301	1.796858	0.015964
ENSG00000166401.14	SERPINB8	1.881672138	16.25401098	5.57E-17

ENSG00000115380.19	EFEMP1	1.881342562	12.8314771	1.47E-13
ENSG00000248712.7	CCDC153	1.881221173	3.01054457	0.000976
ENSG00000110934.10	BIN2	1.88021513	7.246141111	5.67E-08
ENSG00000169258.6	GPRIN1	1.879856457	3.929631981	0.000118
ENSG00000120875.8	DUSP4	1.878099071	14.37070616	4.26E-15
ENSG00000138166.5	DUSP5	1.877919679	12.7465419	1.79E-13
ENSG00000164626.8	KCNK5	1.876308342	4.665728039	2.16E-05
ENSG00000100979.14	PLTP	1.876079842	17.29381199	5.08E-18
ENSG00000121898.12	CPXM2	1.875629539	10.38719925	4.10E-11
ENSG00000174944.8	P2RY14	1.875629054	13.60699275	2.47E-14
ENSG00000160307.9	S100B	1.874650373	3.174223199	0.00067
ENSG00000153303.17	FRMD1	1.874531884	5.714123769	1.93E-06
ENSG00000267121.5	AC008105.3	1.874051271	15.95390789	1.11E-16
ENSG00000178031.16	ADAMTSL1	1.872820689	6.245730723	5.68E-07
ENSG00000249835.2	VCAN-AS1	1.872458026	9.668791862	2.14E-10
ENSG00000188921.13	HACD4	1.871971783	11.70159023	1.99E-12
ENSG00000246582.2	AC100861.1	1.870214399	2.903248313	0.00125
ENSG00000175544.13	CABP4	1.869699191	14.97622539	1.06E-15
ENSG00000158292.6	GPR153	1.869675969	29.05276299	8.86E-30
ENSG00000279030.1	AC007336.3	1.867017383	9.883017894	1.31E-10
ENSG00000089639.10	GMIP	1.866886353	15.48283727	3.29E-16
ENSG00000091513.15	TF	1.865717122	3.735820568	0.000184
ENSG00000168071.21	CCDC88B	1.865354761	13.37484725	4.22E-14
ENSG00000026103.21	FAS	1.864812806	36.95413641	1.11E-37
ENSG00000197168.12	NEK5	1.864381414	1.415991629	0.038371
ENSG00000050628.20	PTGER3	1.861997788	2.041963615	0.009079
ENSG00000196639.6	HRH1	1.861593806	13.83090797	1.48E-14
ENSG00000235313.1	HM13-IT1	1.860293994	1.948200992	0.011267
ENSG00000167851.14	CD300A	1.857799886	5.153192154	7.03E-06
ENSG00000176887.6	SOX11	1.855040781	7.367329211	4.29E-08
ENSG00000258985.1	AL352979.2	1.853714529	1.747856651	0.017871
ENSG00000142765.17	SYTL1	1.853496673	6.379203116	4.18E-07
ENSG00000102057.9	KCND1	1.852848092	3.480348275	0.000331
ENSG00000150594.6	ADRA2A	1.852771345	6.067188508	8.57E-07
ENSG00000108821.13	COL1A1	1.852083011	8.908311305	1.24E-09
ENSG00000069424.14	KCNAB2	1.851870081	18.64971045	2.24E-19
ENSG00000115935.17	WIPF1	1.850735601	20.19221362	6.42E-21
ENSG00000136999.4	NOV	1.85015241	6.337464992	4.60E-07
ENSG00000279753.1	AC011558.1	1.8487403	7.635719511	2.31E-08
ENSG00000235865.2	GSN-AS1	1.847462657	30.49054036	3.23E-31
ENSG00000269481.1	AC010319.4	1.84716034	4.509431689	3.09E-05
ENSG00000103269.13	RHBDL1	1.84700694	2.194243073	0.006394
ENSG00000104415.14	WISP1	1.844876812	1.424758451	0.037605
ENSG00000172382.9	PRSS27	1.844089496	1.976472402	0.010557
ENSG00000106066.14	CPVL	1.843036685	16.83496706	1.46E-17
ENSG00000126950.7	TMEM35A	1.842777617	3.064880687	0.000861
ENSG00000124191.17	TOX2	1.841216498	5.392651143	4.05E-06

ENSG00000100889.11	PCK2		1.839883835	19.57985942	2.63E-20
ENSG00000134962.6	KLB		1.83986281	1.319313635	0.047939
ENSG00000100379.17	KCTD17		1.83769976	21.90652978	1.24E-22
ENSG00000145416.13		1-Mar	1.835990313	14.38789627	4.09E-15
ENSG00000160588.9	MPZL3		1.835179449	3.89230594	0.000128
ENSG00000175906.4	ARL4D		1.835155361	21.53525276	2.92E-22
ENSG00000173762.7	CD7		1.834203661	2.958635239	0.0011
ENSG00000154027.18	AK5		1.834187368	2.686687582	0.002057
ENSG00000182492.15	BGN		1.833433422	11.46375503	3.44E-12
ENSG00000184557.4	SOCS3		1.832342339	11.10903895	7.78E-12
ENSG00000128886.11	ELL3		1.831754913	22.74545169	1.80E-23
ENSG00000224287.2	MSL3P1		1.829921197	3.660392462	0.000219
ENSG00000138646.8	HERC5		1.829800833	12.78482554	1.64E-13
ENSG00000136492.8	BRIP1		1.829013161	1.635258885	0.02316
ENSG00000110446.10	SLC15A3		1.828886074	25.28630112	5.17E-26
ENSG00000277986.1	RF00015		1.827759743	1.391492974	0.040598
ENSG00000285622.1	AL135926.1		1.827068459	16.6123473	2.44E-17
ENSG00000185522.8	LMNTD2		1.826336647	4.638815946	2.30E-05
ENSG00000275993.2	SIK1B		1.825904421	4.012836411	9.71E-05
ENSG00000260992.1	DOCK9-DT		1.825357118	1.775673655	0.016762
ENSG00000087245.12	MMP2		1.824069026	9.947848127	1.13E-10
ENSG00000160180.15	TFF3		1.823893623	2.273241641	0.00533
ENSG00000233175.2	AC008105.1		1.823280623	17.46591526	3.42E-18
ENSG00000091844.7	RGS17		1.822783848	2.984974568	0.001035
ENSG00000118508.4	RAB32		1.82278118	14.91319637	1.22E-15
ENSG00000143196.4	DPT		1.821023298	16.95989427	1.10E-17
ENSG00000142949.16	PTPRF		1.820991262	10.50657381	3.11E-11
ENSG00000270087.5	ZNF503		1.820334884	3.752690394	0.000177
ENSG00000054392.12	HHAT		1.819752907	6.466612717	3.41E-07
ENSG00000137752.23	CASP1		1.819685173	16.69413705	2.02E-17
ENSG00000012779.10	ALOX5		1.819021203	12.0213041	9.52E-13
ENSG00000154175.16	ABI3BP		1.818380723	13.42513096	3.76E-14
ENSG00000181004.9	BBS12		1.818094913	4.744501246	1.80E-05
ENSG00000106789.12	CORO2A		1.817538291	10.73371878	1.85E-11
ENSG00000281571.2	AC241585.2		1.816593084	3.067483288	0.000856
ENSG00000256576.2	LINC02361		1.815980435	1.840114336	0.014451
ENSG00000230438.6	SERPINB9P1		1.814665437	3.830965006	0.000148
ENSG00000127311.9	HELB		1.814541082	13.09313802	8.07E-14
ENSG00000184922.13	FMNL1		1.811152329	17.82210604	1.51E-18
ENSG00000224397.5	SMIM25		1.810647736	1.430095804	0.037145
ENSG00000226067.6	LINC00623		1.810423854	10.33965086	4.57E-11
ENSG00000122694.15	GLIPR2		1.809738805	17.85727662	1.39E-18
ENSG00000250138.4	AC139495.3		1.8086995	3.017789204	0.00096
ENSG00000198910.13	L1CAM		1.808612203	18.68750308	2.05E-19
ENSG00000166825.13	ANPEP		1.808440785	7.727820355	1.87E-08
ENSG00000128342.4	LIF		1.808435339	4.405350937	3.93E-05
ENSG00000237499.6	AL357060.1		1.808380843	11.77467065	1.68E-12

ENSG00000164692.17	COL1A2	1.807827104	10.52851242	2.96E-11
ENSG00000114405.10	C3orf14	1.80586182	8.662122037	2.18E-09
ENSG00000166073.10	GPR176	1.804998895	9.823403772	1.50E-10
ENSG00000115598.9	IL1RL2	1.804226494	1.541872359	0.028716
ENSG00000162004.16	CCDC78	1.803501073	1.559235421	0.027591
ENSG00000178093.13	TSSK6	1.803200648	3.265225821	0.000543
ENSG00000123119.11	NECAB1	1.802317726	3.061392515	0.000868
ENSG00000168234.12	TTC39C	1.801856006	13.69778473	2.01E-14
ENSG00000164430.15	CGAS	1.800497853	7.35479105	4.42E-08
ENSG00000239203.1	AC093484.1	1.798727898	5.0605578	8.70E-06
ENSG00000138356.13	AOX1	1.798344779	7.041118205	9.10E-08
ENSG00000148677.6	ANKRD1	1.797562344	5.409166657	3.90E-06
ENSG00000118971.7	CCND2	1.796623916	16.6055481	2.48E-17
ENSG00000163191.5	S100A11	1.796279474	20.9722353	1.07E-21
ENSG00000138378.18	STAT4	1.795509134	8.765126536	1.72E-09
ENSG00000118193.11	KIF14	1.795351029	1.412452032	0.038685
ENSG00000236383.8	CCDC200	1.795317956	11.03864972	9.15E-12
ENSG00000135476.11	ESPL1	1.795200792	3.093359403	0.000807
ENSG00000148053.15	NTRK2	1.794496406	8.653302287	2.22E-09
ENSG00000147256.11	ARHGAP36	1.792127756	5.099683885	7.95E-06
ENSG00000198821.10	CD247	1.791726334	6.035234094	9.22E-07
ENSG00000114529.12	C3orf52	1.791467776	3.674259296	0.000212
ENSG00000224536.2	AC096677.1	1.79129609	5.572423183	2.68E-06
ENSG00000163430.11	FSTL1	1.790805022	9.011789382	9.73E-10
ENSG00000279809.1	AC005538.2	1.790541892	3.529089007	0.000296
ENSG00000170345.9	FOS	1.790449955	4.73431605	1.84E-05
ENSG00000110697.12	PITPNM1	1.790103815	24.75753703	1.75E-25
ENSG00000065809.13	FAM107B	1.7896497	16.22190692	6.00E-17
ENSG00000141682.11	PMAIP1	1.789440458	6.004101376	9.91E-07
ENSG00000260219.2	AC106782.2	1.789398395	1.91951793	0.012036
ENSG00000222041.11	CYTOR	1.789364674	17.56462049	2.73E-18
ENSG00000260038.1	AC009090.1	1.786511266	1.606927785	0.024721
ENSG00000185008.17	ROBO2	1.785193216	2.937294402	0.001155
ENSG00000186998.15	EMID1	1.784119379	9.923939821	1.19E-10
ENSG00000141448.8	GATA6	1.783118621	2.861420608	0.001376
ENSG00000198121.13	LPAR1	1.783063253	9.771484088	1.69E-10
ENSG00000128849.10	CGNL1	1.781817544	11.65395865	2.22E-12
ENSG00000183423.11	LRIT3	1.781501662	1.640883933	0.022862
ENSG00000196754.10	S100A2	1.78061344	4.383045253	4.14E-05
ENSG00000168461.12	RAB31	1.780499601	19.3242351	4.74E-20
ENSG00000213190.3	MLLT11	1.780406413	21.62854011	2.35E-22
ENSG00000196954.13	CASP4	1.778102485	21.65861261	2.19E-22
ENSG00000268088.1	AC093063.1	1.777713088	2.255487661	0.005553
ENSG00000259678.1	AC066613.2	1.77701603	7.962217157	1.09E-08
ENSG00000197405.7	C5AR1	1.776989464	3.683100403	0.000207
ENSG00000232527.7	AC245595.1	1.776706776	2.19127135	0.006438
ENSG00000040608.13	RTN4R	1.775829165	4.171301806	6.74E-05

ENSG00000277369.1	AC010654.1	1.77569932	1.734995173	0.018408
ENSG00000064666.14	CNN2	1.773910554	18.98732384	1.03E-19
ENSG00000249141.1	AL159163.1	1.773248913	18.21939011	6.03E-19
ENSG00000149781.12	FERMT3	1.773114478	17.35252809	4.44E-18
ENSG00000170915.8	PAQR8	1.772335842	18.54117863	2.88E-19
ENSG00000173868.11	PHOSPHO1	1.771859099	11.93159559	1.17E-12
ENSG00000140678.16	ITGAX	1.771791814	7.561094627	2.75E-08
ENSG00000279983.1	AC244517.10	1.771092734	2.474472954	0.003354
ENSG00000112343.10	TRIM38	1.770716817	45.03759008	9.17E-46
ENSG00000284194.1	SCO2	1.769857203	19.34385858	4.53E-20
ENSG00000276855.1	AC015922.3	1.767671895	4.13793298	7.28E-05
ENSG00000144893.12	MED12L	1.767626617	9.358438311	4.38E-10
ENSG00000228919.5	AC097381.1	1.766926194	2.138655582	0.007267
ENSG00000260711.2	AL121839.2	1.766823168	3.275655516	0.00053
ENSG00000240219.1	AL512306.2	1.766685556	3.270904456	0.000536
ENSG00000285867.1	BX470102.2	1.766110656	3.156855426	0.000697
ENSG00000051523.10	CYBA	1.764790449	19.01259441	9.71E-20
ENSG00000124762.13	CDKN1A	1.764257863	10.87779629	1.32E-11
ENSG00000109861.15	CTSC	1.764203175	16.11906613	7.60E-17
ENSG00000262089.1	AC040977.1	1.763881937	2.260141698	0.005494
ENSG00000146072.6	TNFRSF21	1.763666186	24.04789068	8.96E-25
ENSG00000057657.16	PRDM1	1.762258643	13.15676916	6.97E-14
ENSG00000138119.16	MYOF	1.762084017	23.07546926	8.40E-24
ENSG00000189184.11	PCDH18	1.761960642	10.43279123	3.69E-11
ENSG00000282851.2	BISPR	1.761669693	9.75687148	1.75E-10
ENSG00000215277.8	RNF212B	1.761322042	1.316477703	0.048253
ENSG00000276600.4	RAB7B	1.761195573	14.29467946	5.07E-15
ENSG00000107821.14	KAZALD1	1.761052698	14.90239149	1.25E-15
ENSG00000255568.3	BRWD1-AS2	1.760045773	2.253398164	0.00558
ENSG00000136048.13	DRAM1	1.759875598	18.39538568	4.02E-19
ENSG00000150540.13	HNMT	1.759698234	15.69653208	2.01E-16
ENSG00000107105.14	ELAVL2	1.758558376	3.421512335	0.000379
ENSG00000180644.7	PRF1	1.758493399	9.26453855	5.44E-10
ENSG00000164342.12	TLR3	1.758461717	16.69711618	2.01E-17
ENSG00000107099.15	DOCK8	1.757931354	16.16957945	6.77E-17
ENSG00000163013.11	FBXO41	1.757181551	9.879497186	1.32E-10
ENSG00000205795.4	CYS1	1.756502653	5.821739765	1.51E-06
ENSG00000100365.14	NCF4	1.756402559	6.452355816	3.53E-07
ENSG00000170684.8	ZNF296	1.755723965	2.947379119	0.001129
ENSG00000182580.2	EPHB3	1.754976054	7.972352459	1.07E-08
ENSG00000145536.15	ADAMTS16	1.754971983	2.705782441	0.001969
ENSG00000185480.11	PARPBP	1.754705337	4.860618679	1.38E-05
ENSG00000204482.10	LST1	1.753165282	6.761532529	1.73E-07
ENSG00000106785.14	TRIM14	1.752240621	21.7456509	1.80E-22
ENSG00000213222.3	AC093724.1	1.752139452	1.328937203	0.046888
ENSG00000255176.1	AP000941.1	1.751822124	1.935723714	0.011595
ENSG00000050438.16	SLC4A8	1.751081889	4.114856072	7.68E-05

ENSG00000187116.13	LILRA5	1.749937366	1.333590433	0.046388
ENSG00000283498.1	MIR1244-2	1.749677863	2.048022522	0.008953
ENSG00000099194.5	SCD	1.74951755	3.375522908	0.000421
ENSG00000111335.12	OAS2	1.748716131	9.572541837	2.68E-10
ENSG00000179639.10	FCER1A	1.748416895	3.310096329	0.00049
ENSG00000263235.1	AC006111.2	1.746538447	1.36978295	0.042679
ENSG00000158315.10	RHBDL2	1.746393887	2.123119351	0.007531
ENSG00000157368.10	IL34	1.745180285	15.43354747	3.69E-16
ENSG00000177679.15	SRRM3	1.74497683	4.506543495	3.11E-05
ENSG00000145936.8	KCNMB1	1.744411078	5.408600972	3.90E-06
ENSG00000157680.15	DGKI	1.743825886	1.805951147	0.015633
ENSG00000281371.1	INE2	1.741028637	4.294634435	5.07E-05
ENSG00000118523.5	CTGF	1.740853727	7.522224818	3.00E-08
ENSG00000134830.5	C5AR2	1.740363762	3.465819049	0.000342
ENSG00000129038.15	LOXL1	1.739080251	15.21315217	6.12E-16
ENSG00000108830.9	RND2	1.738600159	6.449659977	3.55E-07
ENSG00000283856.1	MIR7703	1.73830623	26.02184716	9.51E-27
ENSG00000120254.15	MTHFD1L	1.737971368	15.39897764	3.99E-16
ENSG00000144152.12	FBLN7	1.737578439	8.757247748	1.75E-09
ENSG00000081320.10	STK17B	1.737107146	19.72596662	1.88E-20
ENSG00000005249.12	PRKAR2B	1.737066681	6.166055322	6.82E-07
ENSG00000175643.9	RMI2	1.734825412	7.074151036	8.43E-08
ENSG00000011465.16	DCN	1.733708113	8.557430298	2.77E-09
ENSG00000182463.15	TSHZ2	1.73337433	15.30852722	4.91E-16
ENSG00000223701.3	RAET1E-AS1	1.732935235	2.999743507	0.001001
ENSG00000273612.1	RF02108	1.732263517	1.667705825	0.021493
ENSG00000168542.14	COL3A1	1.731953235	7.848717007	1.42E-08
ENSG00000079691.17	CARMIL1	1.731599842	14.04216044	9.07E-15
ENSG00000142634.12	EFHD2	1.730444178	18.5206849	3.02E-19
ENSG00000176845.12	METRNL	1.728782381	12.46215299	3.45E-13
ENSG00000178718.6	RPP25	1.728120778	8.986416299	1.03E-09
ENSG00000228078.1	HLA-U	1.726365096	1.591912012	0.025591
ENSG00000185215.8	TNFAIP2	1.725524105	22.68062547	2.09E-23
ENSG00000149177.12	PTPRJ	1.725392188	16.41850066	3.82E-17
ENSG00000284713.1	AP003071.5	1.724153882	1.654447524	0.022159
ENSG00000166557.12	TMED3	1.723774307	14.35069704	4.46E-15
ENSG00000123609.10	NMI	1.723738471	24.14808684	7.11E-25
ENSG00000106976.20	DNM1	1.722671843	8.968449814	1.08E-09
ENSG00000112378.11	PERP	1.722660499	17.67306555	2.12E-18
ENSG00000152422.15	XRCC4	1.719760976	15.18208839	6.58E-16
ENSG00000048540.14	LMO3	1.719651392	5.920979256	1.20E-06
ENSG00000137077.7	CCL21	1.719343838	2.229911152	0.00589
ENSG00000131944.9	FAAP24	1.719108131	2.146075345	0.007144
ENSG00000144648.15	ACKR2	1.718591759	2.387089047	0.004101
ENSG00000082196.20	C1QTNF3	1.718289844	7.323639911	4.75E-08
ENSG00000154655.15	L3MBTL4	1.71802376	15.17282491	6.72E-16
ENSG00000276718.1	AC005840.4	1.717075317	2.497685142	0.003179

ENSG00000184828.9	ZBTB7C	1.717008239	14.2108212	6.15E-15
ENSG00000277299.1	AC084876.1	1.716978557	4.331020333	4.67E-05
ENSG00000268297.1	CLEC4GP1	1.716725465	4.26458099	5.44E-05
ENSG00000168961.16	LGALS9	1.716187516	13.89122296	1.28E-14
ENSG00000267291.1	AC008649.1	1.716000448	9.504163481	3.13E-10
ENSG00000256508.2	MRGPRF-AS1	1.715909526	2.016525082	0.009627
ENSG00000038295.7	TLL1	1.715755593	5.805680508	1.56E-06
ENSG00000163565.18	IFI16	1.71503615	16.0095661	9.78E-17
ENSG00000163364.9	LINC01116	1.712652213	5.572410099	2.68E-06
ENSG00000168447.10	SCNN1B	1.711752258	1.432807895	0.036914
ENSG00000225766.10	DHRS4L1	1.711558806	1.455728464	0.035016
ENSG00000257530.1	AC048344.1	1.711150717	2.139501967	0.007253
ENSG00000185880.12	TRIM69	1.710845816	19.00584389	9.87E-20
ENSG00000268050.2	AL158151.3	1.70887739	3.708065581	0.000196
ENSG00000164530.14	PI16	1.706938595	6.808522566	1.55E-07
ENSG00000147257.13	GPC3	1.705007746	8.919018086	1.20E-09
ENSG00000248672.5	LY75-CD302	1.704575628	14.83942229	1.45E-15
ENSG00000181264.8	TMEM136	1.703854123	16.18157373	6.58E-17
ENSG00000169432.15	SCN9A	1.702993286	8.814165568	1.53E-09
ENSG00000164181.13	ELOVL7	1.701392114	3.662159701	0.000218
ENSG00000172936.13	MYD88	1.701276924	17.34527892	4.52E-18
ENSG00000284180.1	MIR3606	1.700911621	7.229388491	5.90E-08
ENSG00000101000.5	PROCR	1.700711809	6.005542841	9.87E-07
ENSG00000250461.1	AC122718.1	1.70047546	1.878482914	0.013229
ENSG00000197457.9	STMN3	1.695311422	24.35804147	4.38E-25
ENSG00000267125.2	AC012615.3	1.694551496	8.446741922	3.57E-09
ENSG00000214922.9	HLA-F-AS1	1.694489782	22.20827394	6.19E-23
ENSG00000100600.14	LGMN	1.694298914	28.08409551	8.24E-29
ENSG00000184371.13	CSF1	1.694094017	26.50300121	3.14E-27
ENSG00000153071.14	DAB2	1.693743373	17.5299081	2.95E-18
ENSG00000110436.12	SLC1A2	1.693371494	4.897950937	1.26E-05
ENSG00000155511.17	GRIA1	1.693006929	2.057378458	0.008762
ENSG00000278367.1	AL356652.1	1.692023559	4.514972541	3.06E-05
ENSG00000267737.1	AC087645.2	1.691986945	1.47084285	0.033819
ENSG00000170214.4	ADRA1B	1.690701141	3.118312203	0.000762
ENSG00000206538.8	VGLL3	1.68962624	11.70980924	1.95E-12
ENSG00000167550.10	RHEBL1	1.68955582	3.429160894	0.000372
ENSG00000130429.14	ARPC1B	1.689085679	20.33287313	4.65E-21
ENSG00000168016.14	TRANK1	1.689044298	20.84005897	1.45E-21
ENSG00000115085.13	ZAP70	1.688828261	9.838065245	1.45E-10
ENSG00000163687.13	DNASE1L3	1.687351383	2.979302388	0.001049
ENSG00000121966.6	CXCR4	1.685970329	10.6135549	2.43E-11
ENSG00000102109.8	PCSK1N	1.685859392	1.62992506	0.023446
ENSG00000064309.14	CDON	1.685761809	11.26602938	5.42E-12
ENSG00000123892.11	RAB38	1.685351768	1.783193231	0.016474
ENSG00000118242.15	MREG	1.685066496	13.63552472	2.31E-14
ENSG00000162706.12	CADM3	1.683857191	9.186221421	6.51E-10

ENSG00000244306.11	DUXAP10	1.683818901	3.499831471	0.000316
ENSG00000213719.8	CLIC1	1.682720878	21.41955213	3.81E-22
ENSG00000110002.15	VWA5A	1.682580518	26.1044402	7.86E-27
ENSG00000134247.9	PTGFRN	1.682433499	16.68528797	2.06E-17
ENSG00000071967.11	CYBRD1	1.682054103	8.815424782	1.53E-09
ENSG00000115226.9	FNDC4	1.680921978	6.663632669	2.17E-07
ENSG00000115604.10	IL18R1	1.679101719	8.993967186	1.01E-09
ENSG00000158714.10	SLAMF8	1.677853187	12.24495814	5.69E-13
ENSG00000197122.11	SRC	1.677731777	23.06787101	8.55E-24
ENSG00000160593.18	JAML	1.677676437	5.820425391	1.51E-06
ENSG00000106546.13	AHR	1.677246282	19.51377244	3.06E-20
ENSG00000152527.13	PLEKHH2	1.676936184	7.111070344	7.74E-08
ENSG00000254978.2	ALG1L9P	1.674045594	2.116923361	0.00764
ENSG00000248290.1	TNXA	1.673037356	3.420213659	0.00038
ENSG00000214425.7	LRRC37A4P	1.672930663	8.420313615	3.80E-09
ENSG00000132109.9	TRIM21	1.672268105	27.15765467	6.96E-28
ENSG00000176720.5	BOK	1.671800518	9.701983343	1.99E-10
ENSG00000229851.1	ARSD-AS1	1.671581441	7.766423045	1.71E-08
ENSG00000227678.7	AL355581.1	1.670458998	6.716851413	1.92E-07
ENSG00000232415.1	ELN-AS1	1.668965024	10.58055059	2.63E-11
ENSG00000234807.6	LINC01135	1.668834728	2.67176067	0.002129
ENSG00000135362.13	PRR5L	1.668560388	12.22908289	5.90E-13
ENSG00000115267.6	IFIH1	1.668474278	16.64285973	2.28E-17
ENSG00000123453.17	SARDH	1.668107695	15.09256912	8.08E-16
ENSG00000049192.14	ADAMTS6	1.667068593	2.304210234	0.004964
ENSG00000131459.12	GFPT2	1.666959068	7.533090627	2.93E-08
ENSG00000183486.12	MX2	1.66609188	8.920094486	1.20E-09
ENSG00000284987.1	U52112.1	1.665966268	17.28350486	5.21E-18
ENSG00000125430.8	HS3ST3B1	1.665720419	6.311923261	4.88E-07
ENSG00000128335.13	APOL2	1.663076972	27.27989619	5.25E-28
ENSG00000276180.1	HIST1H4I	1.662399134	7.924142004	1.19E-08
ENSG00000232573.1	RPL3P4	1.662189356	2.086892484	0.008187
ENSG00000064201.15	TSPAN32	1.658176298	4.546793541	2.84E-05
ENSG00000183049.12	CAMK1D	1.658017374	14.61790876	2.41E-15
ENSG00000224138.1	AC000123.1	1.65649112	1.696130675	0.020131
ENSG00000196730.12	DAPK1	1.655887987	17.95626978	1.11E-18
ENSG00000104974.11	LILRA1	1.65587456	1.597060958	0.025289
ENSG00000113600.10	C9	1.655069968	15.90286385	1.25E-16
ENSG00000204055.4	AL158151.1	1.654514903	14.94194085	1.14E-15
ENSG00000019991.16	HGF	1.653701059	8.895487743	1.27E-09
ENSG00000121410.11	A1BG	1.651991336	13.663108	2.17E-14
ENSG00000106560.10	GIMAP2	1.651938902	15.94176276	1.14E-16
ENSG00000140511.11	HAPLN3	1.65150358	13.22799876	5.92E-14
ENSG00000133216.16	EPHB2	1.650813511	14.21944497	6.03E-15
ENSG00000258875.5	AL135818.1	1.650143768	4.157028814	6.97E-05
ENSG00000284906.1	AC091057.6	1.647852177	2.521383641	0.00301
ENSG00000267272.5	LINC01140	1.647681387	11.95176603	1.12E-12

ENSG00000245164.6	LINC00861	1.644762602	1.471535058	0.033765
ENSG00000064300.8	NGFR	1.64466493	9.36411898	4.32E-10
ENSG00000241839.9	PLEKHO2	1.644376186	18.97209435	1.07E-19
ENSG00000249437.7	NAIP	1.643784685	3.200570213	0.00063
ENSG00000197614.10	MFAP5	1.643736021	7.582971069	2.61E-08
ENSG00000135838.13	NPL	1.64220107	16.17764464	6.64E-17
ENSG00000267534.3	S1PR2	1.64209734	9.531796348	2.94E-10
ENSG00000251136.8	AF117829.1	1.64150011	6.220768947	6.01E-07
ENSG00000225670.4	CADM3-AS1	1.641280025	9.5728173	2.67E-10
ENSG00000229214.2	LINC00242	1.641043102	1.761158379	0.017332
ENSG00000226900.1	AL451069.1	1.640321964	1.557079204	0.027728
ENSG00000106483.11	SFRP4	1.636971608	6.078316003	8.35E-07
ENSG00000284634.1	AC092821.3	1.636332765	6.033122005	9.27E-07
ENSG00000115325.13	DOK1	1.636282175	14.16280016	6.87E-15
ENSG00000261801.5	LOXL1-AS1	1.635962856	14.82006447	1.51E-15
ENSG00000173559.12	NABP1	1.635657699	16.30959024	4.90E-17
ENSG00000237798.1	AC010894.4	1.634733031	11.07426795	8.43E-12
ENSG00000062282.14	DGAT2	1.633974882	2.986745495	0.001031
ENSG00000170006.11	TMEM154	1.633769341	3.416374783	0.000383
ENSG00000236675.1	MTX1P1	1.633719693	6.09712101	8.00E-07
ENSG00000170579.16	DLGAP1	1.632453188	1.302357299	0.049847
ENSG00000108950.11	FAM20A	1.632190186	17.57220602	2.68E-18
ENSG00000184986.10	TMEM121	1.631501572	2.470665567	0.003383
ENSG00000273544.1	SNORA44	1.631436143	1.83695218	0.014556
ENSG00000126353.3	CCR7	1.6306505	1.720562584	0.01903
ENSG00000164877.18	MICALL2	1.62992531	18.34530101	4.52E-19
ENSG00000164845.16	FAM86FP	1.629893753	1.411045605	0.038811
ENSG00000205559.4	CHKB-DT	1.627865792	1.410094904	0.038896
ENSG00000026025.15	VIM	1.627395617	13.28762005	5.16E-14
ENSG00000064012.21	CASP8	1.626984375	19.84379805	1.43E-20
ENSG00000128594.7	LRRC4	1.626847492	5.099764152	7.95E-06
ENSG00000092621.12	PHGDH	1.626804022	7.87650031	1.33E-08
ENSG00000174807.3	CD248	1.624844748	7.891015999	1.29E-08
ENSG00000105270.14	CLIP3	1.624148263	21.64483109	2.27E-22
ENSG00000131242.17	RAB11FIP4	1.623886067	8.25366863	5.58E-09
ENSG00000259709.1	AC023906.4	1.623396788	5.068570477	8.54E-06
ENSG00000164294.13	GPX8	1.623040101	11.50957557	3.09E-12
ENSG00000166501.13	PRKCB	1.622813127	5.999752835	1.00E-06
ENSG00000128284.19	APOL3	1.622700699	23.48908667	3.24E-24
ENSG00000267648.1	AC060766.5	1.622305471	1.34085255	0.045619
ENSG00000153551.13	CMTM7	1.622230078	17.0485588	8.94E-18
ENSG00000132274.15	TRIM22	1.622182385	18.56898688	2.70E-19
ENSG00000259775.1	AL138976.2	1.621976269	1.524426826	0.029893
ENSG00000285016.1	AC017002.6	1.621562892	11.45147849	3.54E-12
ENSG00000206597.1	SNORA57	1.6211886	2.351459684	0.004452
ENSG00000180448.10	ARHGAP45	1.620968494	15.00248537	9.94E-16
ENSG00000101255.10	TRIB3	1.620866102	7.692924109	2.03E-08

ENSG00000061455.10	PRDM6	1.620537656	1.828299933	0.014849
ENSG00000198794.11	SCAMP5	1.619363442	10.85846234	1.39E-11
ENSG00000213658.11	LAT	1.618903254	14.16717374	6.80E-15
ENSG00000280153.1	AC133065.3	1.618508541	14.14776159	7.12E-15
ENSG00000256229.7	ZNF486	1.617800003	6.060509097	8.70E-07
ENSG00000059378.12	PARP12	1.616791394	26.2647581	5.44E-27
ENSG00000166741.7	NNMT	1.61619025	8.460989732	3.46E-09
ENSG00000197747.8	S100A10	1.616169953	16.69711618	2.01E-17
ENSG00000106333.12	PCOLCE	1.614835952	14.85545981	1.39E-15
ENSG00000258520.1	AL359317.1	1.613398131	3.49882179	0.000317
ENSG00000231908.1	IDH1-AS1	1.613299426	1.33741368	0.045982
ENSG00000205542.10	TMSB4X	1.613027504	15.60609431	2.48E-16
ENSG00000082397.17	EPB41L3	1.612068674	23.11996081	7.59E-24
ENSG00000165072.9	MAMDC2	1.611754338	14.77288154	1.69E-15
ENSG00000166033.12	HTRA1	1.611573057	9.141813858	7.21E-10
ENSG00000129596.4	CDO1	1.611268392	6.891492277	1.28E-07
ENSG00000028277.21	POU2F2	1.610785386	7.402649014	3.96E-08
ENSG00000027644.4	INSRR	1.61067755	1.764164487	0.017212
ENSG00000038427.15	VCAN	1.61067179	8.489450205	3.24E-09
ENSG00000006534.15	ALDH3B1	1.609675211	23.57193822	2.68E-24
ENSG00000256512.1	AC009511.1	1.60961694	3.553582131	0.00028
ENSG00000116852.14	KIF21B	1.609300579	14.06326469	8.64E-15
ENSG00000107798.17	LIPA	1.608614507	23.51352384	3.07E-24
ENSG00000143515.16	ATP8B2	1.608312473	14.98894319	1.03E-15
ENSG00000101230.5	ISM1	1.607735461	5.508867993	3.10E-06
ENSG00000117676.13	RPS6KA1	1.607556601	11.05928624	8.72E-12
ENSG00000234961.1	AL133415.1	1.607508294	12.71737137	1.92E-13
ENSG00000166165.12	CKB	1.606732618	11.45396336	3.52E-12
ENSG00000213062.4	AL021068.1	1.60623503	1.356879613	0.043966
ENSG00000181467.4	RAP2B	1.604844969	27.52107521	3.01E-28
ENSG00000228526.7	MIR34AHG	1.604247439	13.66437009	2.17E-14
ENSG00000101160.13	CTSZ	1.602934584	22.9376439	1.15E-23
ENSG00000147251.15	DOCK11	1.601245203	10.21208917	6.14E-11
ENSG00000121281.12	ADCY7	1.601096601	23.10958214	7.77E-24
ENSG00000184988.8	TMEM106A	1.601048694	15.05717393	8.77E-16
ENSG00000164611.12	PTTG1	1.599544818	5.895664517	1.27E-06
ENSG00000204176.13	SYT15	1.598695396	4.335177011	4.62E-05
ENSG00000142347.17	MYO1F	1.597904764	7.774166342	1.68E-08
ENSG00000146112.11	PPP1R18	1.597742318	25.52571766	2.98E-26
ENSG00000174004.5	NRROS	1.597221368	15.11142995	7.74E-16
ENSG00000169750.8	RAC3	1.596515912	2.008148603	0.009814
ENSG00000163145.12	C1QTNF7	1.595934889	3.680245303	0.000209
ENSG00000182836.9	PLCXD3	1.595767606	3.150661205	0.000707
ENSG00000151917.17	BEND6	1.595745372	4.897308317	1.27E-05
ENSG00000225756.1	DBH-AS1	1.594722359	3.741252922	0.000181
ENSG00000255121.2	AP003392.4	1.594421792	3.153292262	0.000703
ENSG00000149131.15	SERPING1	1.594120843	12.17207999	6.73E-13

ENSG00000182718.16	ANXA2	1.593780647	20.8721015	1.34E-21
ENSG00000140563.14	MCTP2	1.593015749	2.686345753	0.002059
ENSG00000153029.14	MR1	1.591925915	20.15213297	7.04E-21
ENSG00000152580.8	IGSF10	1.591792428	6.493093035	3.21E-07
ENSG00000172296.12	SPTLC3	1.59099244	13.67947324	2.09E-14
ENSG00000227507.2	LTB	1.590862796	4.20428024	6.25E-05
ENSG00000266916.5	ZNF793-AS1	1.59040607	1.551470965	0.028089
ENSG00000197355.10	UAP1L1	1.590210669	22.06100574	8.69E-23
ENSG00000164949.7	GEM	1.589799553	12.32881253	4.69E-13
ENSG00000111490.13	TBC1D30	1.589738008	2.851313322	0.001408
ENSG00000171700.13	RGS19	1.589491806	10.82988119	1.48E-11
ENSG00000140931.19	CMTM3	1.588781361	14.97944997	1.05E-15
ENSG00000161955.16	TNFSF13	1.586991895	17.52728842	2.97E-18
ENSG00000254814.1	AP003031.1	1.586031292	1.796514169	0.015977
ENSG00000183784.7	C9orf66	1.585428615	1.613346805	0.024359
ENSG00000177335.10	C8orf31	1.585022108	2.17093531	0.006746
ENSG00000187688.14	TRPV2	1.583248115	17.46836074	3.40E-18
ENSG00000229124.6	VIM-AS1	1.583093434	12.77855279	1.67E-13
ENSG00000283321.1	AC019117.2	1.583004019	7.009391522	9.79E-08
ENSG00000100095.18	SEZ6L	1.582100534	2.044571636	0.009025
ENSG00000114541.14	FRMD4B	1.581582649	15.58653632	2.59E-16
ENSG00000156508.17	EEF1A1	1.581304031	21.66307602	2.17E-22
ENSG00000026297.15	RNASET2	1.581303848	17.32191877	4.77E-18
ENSG00000198785.4	GRIN3A	1.581288302	3.210884497	0.000615
ENSG00000171227.6	TMEM37	1.58116489	6.98324351	1.04E-07
ENSG00000141524.15	TMC6	1.581065502	17.25296684	5.59E-18
ENSG00000251669.5	FAM86EP	1.58061145	2.875932358	0.001331
ENSG00000112541.14	PDE10A	1.579763736	5.934838659	1.16E-06
ENSG00000204592.8	HLA-E	1.579167028	24.55924615	2.76E-25
ENSG00000232533.1	AC093673.1	1.578461986	13.35821665	4.38E-14
ENSG00000263961.7	RHEX	1.578214307	2.082908227	0.008262
ENSG00000110492.15	MDK	1.577591468	16.74367341	1.80E-17
ENSG00000092009.10	CMA1	1.577529609	2.843536005	0.001434
ENSG00000145708.10	CRHBP	1.576927263	2.612025197	0.002443
ENSG00000204706.14	MAMDC2-AS1	1.57551739	10.00011372	1.00E-10
ENSG00000111885.6	MAN1A1	1.575066136	14.61260138	2.44E-15
ENSG00000154134.14	ROBO3	1.574268204	14.23531205	5.82E-15
ENSG00000267745.1	AC060766.7	1.573310332	3.91017205	0.000123
ENSG00000164620.8	RELL2	1.573252333	9.430277725	3.71E-10
ENSG00000256594.8	AC010186.2	1.572411767	4.867028038	1.36E-05
ENSG00000142185.16	TRPM2	1.572364774	9.868535441	1.35E-10
ENSG00000260807.6	AC009041.2	1.572131294	16.75421409	1.76E-17
ENSG00000113212.6	PCDHB7	1.571918264	4.174915032	6.68E-05
ENSG00000123329.17	ARHGAP9	1.571917835	7.321605814	4.77E-08
ENSG00000205279.8	CTXN3	1.571883488	2.967008059	0.001079
ENSG00000152778.8	IFIT5	1.570122302	23.77427437	1.68E-24
ENSG00000117519.15	CNN3	1.569009903	13.7090713	1.95E-14

ENSG00000171608.15	PIK3CD	1.567030067	14.19905597	6.32E-15
ENSG00000184378.2	ACTRT3	1.566882366	2.649425501	0.002242
ENSG00000253645.1	AC108863.1	1.566429265	20.94769478	1.13E-21
ENSG00000173110.7	HSPA6	1.56533723	2.50478491	0.003128
ENSG00000118640.10	VAMP8	1.564946048	18.08396703	8.24E-19
ENSG00000242539.2	AC007620.2	1.564605348	15.63968396	2.29E-16
ENSG00000135077.8	HAVCR2	1.563135376	13.96261912	1.09E-14
ENSG00000246640.1	PICART1	1.562773845	1.799417771	0.01587
ENSG00000164308.16	ERAP2	1.56244471	5.456969136	3.49E-06
ENSG00000137809.16	ITGA11	1.562365366	7.929279138	1.18E-08
ENSG00000115129.13	TP53I3	1.562359646	17.86261546	1.37E-18
ENSG00000138778.11	CENPE	1.561386874	2.105244087	0.007848
ENSG00000250742.2	LINC02381	1.559689809	6.138150131	7.28E-07
ENSG00000169499.14	PLEKHA2	1.558855771	24.00400055	9.91E-25
ENSG00000178226.10	PRSS36	1.558350591	13.13385971	7.35E-14
ENSG00000277694.1	RF01979	1.558348494	2.761772713	0.001731
ENSG00000161040.16	FBXL13	1.557425527	6.174286408	6.69E-07
ENSG00000249240.2	AC069368.1	1.557403713	18.12604	7.48E-19
ENSG00000088340.15	FER1L4	1.55726799	6.041491266	9.09E-07
ENSG00000034510.5	TMSB10	1.554016992	19.44272234	3.61E-20
ENSG00000197903.7	HIST1H2BK	1.553737914	11.0023507	9.95E-12
ENSG00000149050.9	ZNF214	1.552786491	2.190889741	0.006443
ENSG00000139354.10	GAS2L3	1.55278071	4.26139116	5.48E-05
ENSG00000179029.14	TMEM107	1.551867987	11.50442024	3.13E-12
ENSG00000254087.7	LYN	1.551769156	11.42061649	3.80E-12
ENSG00000272186.1	AP003392.6	1.551637597	1.309392939	0.049046
ENSG00000186854.10	TRABD2A	1.551432008	16.23467759	5.83E-17
ENSG00000134285.10	FKBP11	1.551048552	13.52406354	2.99E-14
ENSG00000101265.15	RASSF2	1.550865204	12.44857518	3.56E-13
ENSG00000105281.12	SLC1A5	1.548424539	17.75378152	1.76E-18
ENSG00000257108.1	NHLRC4	1.547708451	2.288180393	0.00515
ENSG00000049540.16	ELN	1.547394356	9.900872947	1.26E-10
ENSG00000124549.14	BTN2A3P	1.54566536	13.08796796	8.17E-14
ENSG00000117600.12	PLPPR4	1.545544144	6.064802987	8.61E-07
ENSG00000267698.1	AC002116.2	1.545492151	16.5783599	2.64E-17
ENSG00000131171.12	SH3BGRL	1.545435076	15.30502259	4.95E-16
ENSG00000271664.1	AC004890.3	1.544227934	1.511735666	0.03078
ENSG00000053918.16	KCNQ1	1.543996789	8.092097257	8.09E-09
ENSG00000157227.12	MMP14	1.543487937	10.65323346	2.22E-11
ENSG00000247157.6	LINC01252	1.543128601	1.824661389	0.014974
ENSG00000258659.6	TRIM34	1.542118305	14.35003396	4.47E-15
ENSG00000169026.12	SLC49A3	1.538705211	22.63466893	2.32E-23
ENSG00000162704.15	ARPC5	1.538622687	18.57344062	2.67E-19
ENSG00000092470.11	WDR76	1.538429581	8.551467616	2.81E-09
ENSG00000117595.11	IRF6	1.538126394	2.895228886	0.001273
ENSG00000160602.13	NEK8	1.537691459	8.097319569	7.99E-09
ENSG00000162636.15	FAM102B	1.537051213	16.13639638	7.30E-17

ENSG00000102287.18	GABRE	1.536646736	5.890682666	1.29E-06
ENSG00000089225.19	TBX5	1.536634243	1.772352776	0.016891
ENSG00000135324.5	MRAP2	1.535554002	3.633828656	0.000232
ENSG00000197496.5	SLC2A10	1.535200679	9.303017886	4.98E-10
ENSG00000035862.12	TIMP2	1.534598649	7.158674493	6.94E-08
ENSG00000172794.19	RAB37	1.534554266	8.822708439	1.50E-09
ENSG00000134321.11	RSAD2	1.533672521	11.58988641	2.57E-12
ENSG00000106258.14	CYP3A5	1.533356652	2.057418637	0.008762
ENSG00000268364.1	SMC5-AS1	1.533284608	11.3255744	4.73E-12
ENSG00000239998.5	LILRA2	1.533122753	1.403913444	0.039454
ENSG00000187550.8	SBK2	1.531606847	1.65735296	0.022011
ENSG00000240694.8	PNMA2	1.531548647	3.969560627	0.000107
ENSG00000196576.14	PLXNB2	1.530424095	25.49648807	3.19E-26
ENSG00000163840.9	DTX3L	1.529611242	23.66451869	2.17E-24
ENSG00000278887.2	AC106782.7	1.529319985	2.636058968	0.002312
ENSG00000272419.6	LINC01145	1.52832089	7.57470022	2.66E-08
ENSG00000158270.11	COLEC12	1.527685376	13.05057813	8.90E-14
ENSG00000170049.9	KCNAB3	1.5272151	1.865043776	0.013644
ENSG00000278611.1	ZNF426-DT	1.527008446	1.753509749	0.01764
ENSG00000090530.9	P3H2	1.526707502	8.43771496	3.65E-09
ENSG00000224729.5	PCOLCE-AS1	1.526172773	14.1115815	7.73E-15
ENSG00000154237.12	LRRK1	1.525645383	16.64395453	2.27E-17
ENSG00000154760.13	SLFN13	1.525422232	15.5486753	2.83E-16
ENSG00000197956.9	S100A6	1.524616137	17.54807796	2.83E-18
ENSG00000063127.15	SLC6A16	1.524269172	3.21255303	0.000613
ENSG00000148848.14	ADAM12	1.524212187	10.38999178	4.07E-11
ENSG00000156299.13	TIAM1	1.523973812	12.56738641	2.71E-13
ENSG00000205413.7	SAMD9	1.522825456	10.14532152	7.16E-11
ENSG00000179954.15	SSC5D	1.522110707	8.009700889	9.78E-09
ENSG00000150627.15	WDR17	1.521155125	1.776218009	0.016741
ENSG00000205885.7	C1RL-AS1	1.520806333	14.17924185	6.62E-15
ENSG00000230630.5	DNM3OS	1.52038705	8.130435138	7.41E-09
ENSG00000137675.4	MMP27	1.520049866	1.495520908	0.031951
ENSG00000155962.12	CLIC2	1.520021137	13.16455823	6.85E-14
ENSG00000166250.11	CLMP	1.519461224	8.115536781	7.66E-09
ENSG00000255310.2	AF131215.5	1.517289468	5.434249945	3.68E-06
ENSG00000122644.12	ARL4A	1.516640906	12.50090645	3.16E-13
ENSG00000080986.12	NDC80	1.515857364	2.524902821	0.002986
ENSG00000144619.14	CNTN4	1.515655817	3.492834558	0.000321
ENSG00000111728.10	ST8SIA1	1.515649838	8.531376334	2.94E-09
ENSG00000240661.3	AC063952.1	1.514757296	6.905367283	1.24E-07
ENSG00000168405.17	CMAHP	1.513045133	16.14865715	7.10E-17
ENSG00000204991.10	SPIRE2	1.512870195	4.221359783	6.01E-05
ENSG00000122862.4	SRGN	1.51265747	10.4662188	3.42E-11
ENSG00000226237.1	GAS1RR	1.511753788	2.164037996	0.006854
ENSG00000259125.1	LRP1-AS	1.511446985	3.646157289	0.000226
ENSG00000225746.11	MEG8	1.508913097	6.842713321	1.44E-07

ENSG00000104312.7	RIPK2	1.508858646	14.10271187	7.89E-15
ENSG00000076258.9	FMO4	1.508663131	6.455553965	3.50E-07
ENSG00000224101.1	ELMO1-AS1	1.508459898	1.322731094	0.047563
ENSG00000087495.16	PHACTR3	1.508280071	1.551470965	0.028089
ENSG00000283674.2	AC068587.4	1.507491756	2.136503091	0.007303
ENSG00000259792.1	AC104758.2	1.507330563	4.208925748	6.18E-05
ENSG00000213722.8	DDAH2	1.507205711	13.15478332	7.00E-14
ENSG00000131203.12	IDO1	1.507116761	4.842219304	1.44E-05
ENSG00000196205.8	EEF1A1P5	1.506833919	20.11770454	7.63E-21
ENSG00000277147.5	LINC00869	1.50606193	21.57200171	2.68E-22
ENSG00000134531.9	EMP1	1.505910541	9.717547959	1.92E-10
ENSG00000143429.10	LSP1P4	1.505652277	4.36008629	4.36E-05
ENSG00000106538.9	RARRES2	1.504685663	6.6166633	2.42E-07
ENSG00000179761.11	PIPOX	1.504527488	3.221702342	0.0006
ENSG00000110031.12	LPXN	1.504457674	17.29073365	5.12E-18
ENSG00000185404.16	SP140L	1.504298391	36.02629352	9.41E-37
ENSG00000100448.3	CTSG	1.503826108	5.015987282	9.64E-06
ENSG00000133962.7	CATSPERB	1.50332945	1.582480369	0.026153
ENSG00000266094.7	RASSF5	1.50222617	11.69658875	2.01E-12
ENSG00000213088.10	ACKR1	1.501854652	8.495164905	3.20E-09
ENSG00000213988.10	ZNF90	1.501775343	1.475701618	0.033442
ENSG00000185885.15	IFITM1	1.501106742	11.31670543	4.82E-12
ENSG00000188483.7	IER5L	1.500612447	15.01994291	9.55E-16
ENSG00000125910.5	S1PR4	1.49993844	2.201368667	0.00629
ENSG00000271009.3	AC116667.1	1.499780372	2.293872793	0.005083
ENSG00000101144.12	BMP7	1.498871244	1.754786991	0.017588
ENSG00000145908.12	ZNF300	1.498163299	6.468330746	3.40E-07
ENSG00000183801.7	OLFML1	1.497336722	10.63136991	2.34E-11
ENSG00000157734.13	SNX22	1.49729923	14.67166234	2.13E-15
ENSG00000152270.8	PDE3B	1.497120425	4.010546611	9.76E-05
ENSG00000277496.1	AL357033.4	1.497053692	4.142663243	7.20E-05
ENSG00000135148.11	TRAFD1	1.495377234	25.89717606	1.27E-26
ENSG00000101911.12	PRPS2	1.494458454	13.97961164	1.05E-14
ENSG00000184584.12	TMEM173	1.494201033	21.02861471	9.36E-22
ENSG00000179630.10	LACC1	1.493628393	15.6003984	2.51E-16
ENSG00000137628.16	DDX60	1.4931543	14.75963137	1.74E-15
ENSG00000113532.12	ST8SIA4	1.493115457	10.66376211	2.17E-11
ENSG00000111331.12	OAS3	1.493089388	9.307710513	4.92E-10
ENSG00000259539.1	AC051619.6	1.492965887	1.908900141	0.012334
ENSG00000087903.12	RFX2	1.492707691	13.04534474	9.01E-14
ENSG00000168038.10	ULK4	1.492500559	2.24528416	0.005685
ENSG00000160469.16	BRSK1	1.49242082	4.522793467	3.00E-05
ENSG00000213185.6	FAM24B	1.491909911	3.465819049	0.000342
ENSG00000095303.15	PTGS1	1.489908872	8.804579697	1.57E-09
ENSG00000139289.13	PHLDA1	1.487976333	21.1080402	7.80E-22
ENSG00000109610.5	SOD3	1.487279611	8.41594352	3.84E-09
ENSG00000167552.13	TUBA1A	1.487124962	18.65637461	2.21E-19

ENSG00000178404.9	CEP295NL	1.486308832	6.751213942	1.77E-07
ENSG00000176788.8	BASP1	1.485429529	8.49661208	3.19E-09
ENSG00000085265.10	FCN1	1.483281886	2.745160118	0.001798
ENSG00000100077.14	GRK3	1.482850162	11.48322254	3.29E-12
ENSG00000197948.10	FCHSD1	1.482512225	17.15604319	6.98E-18
ENSG00000092010.14	PSME1	1.482355041	33.17202704	6.73E-34
ENSG00000105939.12	ZC3HAV1	1.48232712	31.38500686	4.12E-32
ENSG00000275074.1	NUDT18	1.48174426	13.78161951	1.65E-14
ENSG00000118308.15	LRMP	1.48163292	5.018336536	9.59E-06
ENSG00000138180.15	CEP55	1.481202621	2.588364901	0.00258
ENSG00000104894.11	CD37	1.480752036	13.40222858	3.96E-14
ENSG00000147324.10	MFHAS1	1.479673621	28.71794099	1.91E-29
ENSG00000256061.7	DNAAF4	1.477441597	2.793290475	0.00161
ENSG00000135414.9	GDF11	1.477217087	16.10146825	7.92E-17
ENSG00000116717.12	GADD45A	1.4762799	11.3160698	4.83E-12
ENSG00000091536.17	MYO15A	1.476113091	3.969170487	0.000107
ENSG00000158792.15	SPATA2L	1.475905752	8.33708458	4.60E-09
ENSG00000157873.17	TNFRSF14	1.47577205	17.18771443	6.49E-18
ENSG00000008394.12	MGST1	1.475677727	4.460209162	3.47E-05
ENSG00000174130.12	TLR6	1.475454053	3.099196249	0.000796
ENSG00000257453.1	AC011611.3	1.474826952	14.83756151	1.45E-15
ENSG00000158258.16	CLSTN2	1.474821688	8.897746601	1.27E-09
ENSG00000175505.10	CLCF1	1.474791982	7.326809746	4.71E-08
ENSG00000102802.9	MEDAG	1.474789258	7.904076443	1.25E-08
ENSG00000168918.13	INPP5D	1.474340286	12.44520418	3.59E-13
ENSG00000203392.3	AC105020.1	1.47368198	2.211464951	0.006145
ENSG00000127084.18	FGD3	1.473559864	8.977770804	1.05E-09
ENSG00000162641.18	AKNAD1	1.473372119	1.493950892	0.032066
ENSG00000278893.1	AC010533.1	1.472989223	3.419873299	0.00038
ENSG00000228624.7	HDAC2-AS2	1.472981508	2.538725539	0.002893
ENSG00000250786.1	SNHG18	1.471827443	1.301207511	0.04998
ENSG00000124496.12	TRERF1	1.471806834	15.65386299	2.22E-16
ENSG00000256043.2	CTSO	1.471473887	16.52775577	2.97E-17
ENSG00000267601.1	AC022966.1	1.469826746	6.650334878	2.24E-07
ENSG00000182704.7	TSKU	1.468304787	6.692226389	2.03E-07
ENSG00000101955.14	SRPX	1.466631155	10.14962688	7.09E-11
ENSG00000120708.16	TGFBI	1.466055675	16.00369483	9.92E-17
ENSG00000188517.15	COL25A1	1.466039911	1.611947306	0.024437
ENSG00000132821.11	VSTM2L	1.465110695	6.384190609	4.13E-07
ENSG00000184730.10	APOBR	1.464278861	5.245919248	5.68E-06
ENSG00000186185.13	KIF18B	1.463889298	1.502504684	0.031441
ENSG00000126246.9	IGFLR1	1.460594101	14.20760111	6.20E-15
ENSG00000238057.9	ZEB2-AS1	1.460591894	3.829379759	0.000148
ENSG00000223478.1	AL441992.1	1.46048718	2.112606254	0.007716
ENSG00000113749.7	HRH2	1.459948915	2.381412427	0.004155
ENSG00000177409.11	SAMD9L	1.459885276	13.78493869	1.64E-14
ENSG00000156970.12	BUB1B	1.45919986	1.910320729	0.012294

ENSG00000184232.8	OAF	1.458992382	8.577409419	2.65E-09
ENSG00000077238.13	IL4R	1.458726247	17.15149833	7.06E-18
ENSG00000166794.4	PPIB	1.458514814	14.14774551	7.12E-15
ENSG00000122420.9	PTGFR	1.458285482	6.210509269	6.16E-07
ENSG00000005513.9	SOX8	1.457183242	12.01294561	9.71E-13
ENSG00000255839.1	AC117503.1	1.457024098	3.973040596	0.000106
ENSG00000270823.1	AC007938.2	1.456991256	6.180100587	6.61E-07
ENSG00000171943.11	SRGAP2C	1.456856931	18.46617752	3.42E-19
ENSG00000101577.9	LPIN2	1.456837731	21.74212978	1.81E-22
ENSG00000067066.16	SP100	1.456770357	35.51349972	3.07E-36
ENSG00000185339.8	TCN2	1.455732575	14.64127678	2.28E-15
ENSG00000165698.15	SPACA9	1.455345741	6.755942359	1.75E-07
ENSG00000125841.12	NRSN2	1.453358078	17.32235252	4.76E-18
ENSG00000129474.15	AJUBA	1.452955561	9.929302872	1.18E-10
ENSG00000124813.21	RUNX2	1.452766011	6.33606159	4.61E-07
ENSG00000227220.1	AL133346.1	1.451944112	5.980382315	1.05E-06
ENSG00000274582.1	SNORA16A	1.451327588	1.498792123	0.031711
ENSG00000104332.11	SFRP1	1.4510421	5.30243195	4.98E-06
ENSG00000112977.15	DAP	1.450909913	14.39211964	4.05E-15
ENSG00000172725.13	CORO1B	1.450637596	18.15362877	7.02E-19
ENSG00000159231.5	CBR3	1.450055209	9.167521064	6.80E-10
ENSG00000187922.13	LCN10	1.449826661	2.373605018	0.004231
ENSG00000204099.11	NEU4	1.449424185	2.50807443	0.003104
ENSG00000100911.15	PSME2	1.449352212	27.47389844	3.36E-28
ENSG00000011422.11	PLAUR	1.449305585	11.24712901	5.66E-12
ENSG00000167525.13	PROCA1	1.449193832	6.806655121	1.56E-07
ENSG00000167553.15	TUBA1C	1.449011731	36.0212552	9.52E-37
ENSG00000131435.12	PDLIM4	1.448978391	13.1387152	7.27E-14
ENSG00000159228.12	CBR1	1.447109494	22.81667009	1.53E-23
ENSG00000136295.14	TTYH3	1.446939925	19.82358206	1.50E-20
ENSG00000280187.1	AC022107.1	1.446675845	3.746304997	0.000179
ENSG00000272558.1	U91328.2	1.446607168	25.96975168	1.07E-26
ENSG00000065357.19	DGKA	1.44518901	14.74298004	1.81E-15
ENSG00000074370.17	ATP2A3	1.444123676	12.2125495	6.13E-13
ENSG00000006756.15	ARSD	1.443758784	18.55883518	2.76E-19
ENSG00000151789.11	ZNF385D	1.442772617	15.24229643	5.72E-16
ENSG00000099337.4	KCNK6	1.442750368	16.90944548	1.23E-17
ENSG00000254928.1	AP001372.3	1.442689348	2.983892742	0.001038
ENSG00000232480.1	TGFB2-AS1	1.442497197	4.532339107	2.94E-05
ENSG00000162458.12	FBLIM1	1.442297201	16.44525884	3.59E-17
ENSG00000105928.14	GSDME	1.442137368	16.15027389	7.07E-17
ENSG00000090554.12	FLT3LG	1.441096159	16.75500742	1.76E-17
ENSG00000174697.4	LEP	1.438647523	1.434513382	0.036769
ENSG00000226471.6	Z93930.2	1.4375869	2.071059614	0.008491
ENSG00000284594.1	MIR7847	1.436370034	16.561764	2.74E-17
ENSG00000166147.13	FBN1	1.436104967	5.861530284	1.38E-06
ENSG00000168685.14	IL7R	1.435891336	2.90850141	0.001235

ENSG00000258232.2	AC125611.3	1.435368953	23.63638523	2.31E-24
ENSG00000164088.17	PPM1M	1.435017482	17.36809434	4.28E-18
ENSG00000275719.1	AC008622.2	1.434804811	8.740543962	1.82E-09
ENSG00000171970.12	ZNF57	1.434695364	4.981210516	1.04E-05
ENSG00000154645.13	CHODL	1.434125255	2.948258729	0.001127
ENSG00000206052.10	DOK6	1.43356787	1.96058874	0.01095
ENSG00000197536.11	C5orf56	1.433277582	22.06279874	8.65E-23
ENSG00000157193.16	LRP8	1.43300321	13.01628438	9.63E-14
ENSG00000128383.12	APOBEC3A	1.432282074	1.309143287	0.049075
ENSG00000165816.12	VWA2	1.431724857	1.581843125	0.026191
ENSG00000131398.13	KCNC3	1.430232319	10.36126788	4.35E-11
ENSG00000159239.13	AC005041.1	1.429518016	5.486100048	3.27E-06
ENSG00000185022.11	MAFF	1.429010326	7.132267428	7.37E-08
ENSG00000204681.10	GABBR1	1.428954397	12.50444499	3.13E-13
ENSG00000188729.6	OSTN	1.428811497	1.501599153	0.031507
ENSG00000060982.14	BCAT1	1.427410214	9.221916128	6.00E-10
ENSG00000136869.14	TLR4	1.426335995	10.03960493	9.13E-11
ENSG00000121064.12	SCPEP1	1.425563046	20.55820322	2.77E-21
ENSG00000277669.1	AC009086.2	1.424949469	13.50278909	3.14E-14
ENSG00000197093.10	GAL3ST4	1.424907644	8.502802141	3.14E-09
ENSG00000177542.10	SLC25A22	1.424643874	20.14580194	7.15E-21
ENSG00000163219.11	ARHGAP25	1.424580363	11.64870917	2.25E-12
ENSG00000131188.11	PRR7	1.424539533	4.220357523	6.02E-05
ENSG00000138134.11	STAMBPL1	1.423979227	9.724755747	1.88E-10
ENSG00000142512.14	SIGLEC10	1.423593743	1.324011762	0.047423
ENSG00000235897.1	TM4SF19-AS1	1.42348147	2.899068352	0.001262
ENSG00000133256.12	PDE6B	1.42326755	4.507200613	3.11E-05
ENSG00000275367.1	AC092111.1	1.422983864	1.421415268	0.037895
ENSG00000136490.8	LIMD2	1.42170435	9.752436733	1.77E-10
ENSG00000257702.3	LBX2-AS1	1.421701131	8.815424782	1.53E-09
ENSG00000120669.15	SOHLH2	1.42122692	2.062300318	0.008664
ENSG00000278869.1	BX539320.1	1.420821315	2.033682922	0.009254
ENSG00000285518.1	AC004900.1	1.420388791	2.04803713	0.008953
ENSG00000013364.18	MVP	1.420316523	31.4794807	3.32E-32
ENSG00000114450.9	GNB4	1.420079617	16.44966005	3.55E-17
ENSG00000172901.19	LVRN	1.419823978	4.141749992	7.22E-05
ENSG00000104093.13	DMXL2	1.419506369	15.40742856	3.91E-16
ENSG00000163932.13	PRKCD	1.418811509	12.98679096	1.03E-13
ENSG00000237949.1	LINC00844	1.418674379	2.44709523	0.003572
ENSG00000277758.4	FO681492.1	1.417541987	2.113395055	0.007702
ENSG00000265519.1	AC015922.2	1.416588363	6.042763263	9.06E-07
ENSG00000181234.9	TMEM132C	1.416399619	3.786527728	0.000163
ENSG00000176049.15	JAKMIP2	1.416225549	2.261245508	0.00548
ENSG00000115339.13	GALNT3	1.416179564	3.270035988	0.000537
ENSG00000015133.18	CCDC88C	1.416138544	18.95593199	1.11E-19
ENSG00000280734.2	LINC01232	1.415531523	16.34949454	4.47E-17
ENSG00000123342.15	MMP19	1.414937938	10.95159947	1.12E-11

ENSG00000258588.3	TRIM6-TRIM34	1.414639022	11.47873262	3.32E-12
ENSG00000254287.1	AC007991.4	1.414324626	2.086892484	0.008187
ENSG00000230319.1	AL022476.1	1.413863444	2.660612761	0.002185
ENSG00000215808.3	LINC01139	1.413224605	2.53287877	0.002932
ENSG00000284308.1	C2orf81	1.411795724	5.707175148	1.96E-06
ENSG00000204262.12	COL5A2	1.411435146	10.67351039	2.12E-11
ENSG00000280073.1	AL157996.1	1.411286556	2.070052819	0.00851
ENSG00000136960.12	ENPP2	1.411161588	8.959076203	1.10E-09
ENSG00000181104.6	F2R	1.411109788	12.06438873	8.62E-13
ENSG00000082512.14	TRAF5	1.411030686	21.02073842	9.53E-22
ENSG00000285162.1	AC004593.3	1.410142213	16.15801317	6.95E-17
ENSG00000237807.3	AC022034.1	1.409998396	5.512649938	3.07E-06
ENSG00000196975.15	ANXA4	1.409753643	14.23693096	5.80E-15
ENSG00000189129.13	PLAC9	1.406699562	9.135663378	7.32E-10
ENSG00000125675.17	GRIA3	1.406250202	1.699831154	0.01996
ENSG00000260193.1	AL138781.1	1.406025528	1.826452984	0.014912
ENSG00000254838.5	GVINP1	1.40506918	6.410100992	3.89E-07
ENSG00000103855.17	CD276	1.404968913	14.40111492	3.97E-15
ENSG00000160991.15	ORAI2	1.404774129	21.06038937	8.70E-22
ENSG00000259327.1	AC023906.3	1.404490207	6.240385919	5.75E-07
ENSG00000204054.13	LINC00963	1.403942238	24.228067	5.91E-25
ENSG00000138161.13	CUZD1	1.403681142	2.844913522	0.001429
ENSG00000223403.4	MEG9	1.403171007	9.565919341	2.72E-10
ENSG00000100626.16	GALNT16	1.402006599	6.693604669	2.02E-07
ENSG00000185585.19	OLFML2A	1.401409065	13.01959152	9.56E-14
ENSG00000245213.6	AC105285.1	1.401321717	4.208699017	6.18E-05
ENSG00000157601.13	MX1	1.401303488	7.774655895	1.68E-08
ENSG00000169439.11	SDC2	1.400653428	12.46274513	3.45E-13
ENSG00000261888.1	AC144831.1	1.400568021	5.178347101	6.63E-06
ENSG00000227028.6	SLC8A1-AS1	1.399904078	8.875846408	1.33E-09
ENSG00000156103.15	MMP16	1.399625572	3.91560509	0.000121
ENSG00000241399.6	CD302	1.399573492	11.83512901	1.46E-12
ENSG00000246090.6	AP002026.1	1.399359666	3.790558424	0.000162
ENSG00000178860.8	MSC	1.399302405	20.21669986	6.07E-21
ENSG00000269837.1	IPO5P1	1.398638143	3.056172091	0.000879
ENSG00000160345.12	C9orf116	1.398524017	1.992343967	0.010178
ENSG00000178531.5	CTXN1	1.398194599	1.511280382	0.030812
ENSG00000267801.1	AC087289.5	1.398161614	21.44870576	3.56E-22
ENSG00000235944.8	ZNF815P	1.397763952	2.913506304	0.00122
ENSG00000274712.1	AC005332.4	1.397614408	10.88583757	1.30E-11
ENSG00000233117.2	LINC00702	1.396466039	10.26479588	5.44E-11
ENSG00000049768.14	FOXP3	1.396451038	6.409569693	3.89E-07
ENSG00000056558.10	TRAF1	1.396393375	14.78976955	1.62E-15
ENSG00000153406.13	NMRAL1	1.394904867	13.12593261	7.48E-14
ENSG00000236841.7	AC007750.1	1.394388601	7.579103589	2.64E-08
ENSG00000243444.7	PALM2	1.393855538	2.449119382	0.003555
ENSG00000176438.12	SYNE3	1.392733455	12.59635725	2.53E-13

ENSG00000168779.19	SHOX2	1.392504977	5.776441019	1.67E-06
ENSG00000275688.4	CCL15-CCL14	1.392251336	7.525854094	2.98E-08
ENSG00000238650.1	SNORD54	1.391521654	1.339290597	0.045784
ENSG00000284570.1	MIR7705	1.390423145	8.36443828	4.32E-09
ENSG00000258944.1	AC004846.2	1.390257814	5.741951262	1.81E-06
ENSG00000261586.2	AC068987.4	1.38957085	1.334137484	0.04633
ENSG00000106484.15	MEST	1.389478515	9.065158955	8.61E-10
ENSG00000258745.1	AL358334.3	1.389224217	6.040874642	9.10E-07
ENSG00000225472.1	AL136366.1	1.388918405	1.484987921	0.032735
ENSG00000268894.6	PLCE1-AS1	1.387309169	2.405918895	0.003927
ENSG00000145685.13	LHFPL2	1.385729725	12.00026738	9.99E-13
ENSG00000267510.1	AC011451.1	1.385600461	2.106768043	0.00782
ENSG00000155428.12	TRIM74	1.385241263	1.412332905	0.038696
ENSG00000100767.16	PAPLN	1.384394887	8.912716965	1.22E-09
ENSG00000130812.10	ANGPTL6	1.384157964	14.40071917	3.97E-15
ENSG00000258818.3	RNASE4	1.384131006	6.483043817	3.29E-07
ENSG00000104432.13	IL7	1.383903268	11.34015759	4.57E-12
ENSG00000006042.11	TMEM98	1.383580397	8.708524484	1.96E-09
ENSG00000112414.14	ADGRG6	1.383240893	11.99047054	1.02E-12
ENSG00000114698.14	PLSCR4	1.381040053	8.074571302	8.42E-09
ENSG00000284428.1	IPO5P1	1.380935966	1.689464665	0.020443
ENSG00000133739.15	LRRCC1	1.380894953	6.115043959	7.67E-07
ENSG00000135144.7	DTX1	1.380543974	6.593200167	2.55E-07
ENSG00000150961.14	SEC24D	1.380068515	16.03798851	9.16E-17
ENSG00000186469.8	GNG2	1.378856989	15.9064899	1.24E-16
ENSG00000101342.9	TLDC2	1.378512916	17.46734363	3.41E-18
ENSG00000164050.12	PLXNB1	1.378218373	10.50373611	3.14E-11
ENSG00000285082.1	AL160272.2	1.378143715	3.023074803	0.000948
ENSG00000164778.4	EN2	1.377969152	1.343475672	0.045344
ENSG00000188167.8	TMPPE	1.377822615	2.357608275	0.004389
ENSG00000283726.1	MIR5193	1.376811979	14.64670939	2.26E-15
ENSG00000141756.18	FKBP10	1.376765255	10.09288393	8.07E-11
ENSG00000259171.1	AL163636.2	1.376497349	6.414901069	3.85E-07
ENSG00000230212.6	AP000688.1	1.376474597	19.18113546	6.59E-20
ENSG00000172349.17	IL16	1.375591893	10.4586364	3.48E-11
ENSG00000162576.16	MXRA8	1.375359284	10.32430853	4.74E-11
ENSG00000285520.1	AL353586.1	1.375205289	3.821448986	0.000151
ENSG00000275431.1	AC244100.2	1.375192644	7.429935577	3.72E-08
ENSG00000206190.11	ATP10A	1.374781357	10.59724196	2.53E-11
ENSG00000101347.9	SAMHD1	1.374752587	17.8363217	1.46E-18
ENSG00000161298.17	ZNF382	1.37453375	5.62930165	2.35E-06
ENSG00000276409.4	CCL14	1.372585068	7.4276959	3.74E-08
ENSG00000132481.6	TRIM47	1.37194735	21.03211117	9.29E-22
ENSG00000099869.7	IGF2-AS	1.371915162	1.772015526	0.016904
ENSG00000137573.13	SULF1	1.371885418	7.985637195	1.03E-08
ENSG00000130066.16	SAT1	1.371562098	12.86579026	1.36E-13
ENSG00000227487.3	NCAM1-AS1	1.371119702	5.850001761	1.41E-06

ENSG00000102034.16	ELF4	1.370965672	16.18414309	6.54E-17
ENSG00000182179.12	UBA7	1.370058885	19.42008241	3.80E-20
ENSG00000099256.18	PRTFDC1	1.36962431	9.029900727	9.33E-10
ENSG00000144730.17	IL17RD	1.369439946	6.197692756	6.34E-07
ENSG00000273485.1	AL139339.2	1.369391608	7.402425631	3.96E-08
ENSG00000075275.16	CELSR1	1.368651836	8.211694966	6.14E-09
ENSG00000273117.1	AC144652.1	1.368585262	3.746033498	0.000179
ENSG00000113504.20	SLC12A7	1.367442204	20.47394265	3.36E-21
ENSG00000077264.14	PAK3	1.367203981	3.980696525	0.000105
ENSG00000012660.13	ELOVL5	1.36640917	9.747662924	1.79E-10
ENSG00000196155.12	PLEKHG4	1.365555807	6.747527738	1.79E-07
ENSG00000171729.13	TMEM51	1.363717232	8.344153874	4.53E-09
ENSG00000013016.15	EHD3	1.363232994	16.05041161	8.90E-17
ENSG00000244953.1	AC087521.1	1.36314909	1.59746403	0.025266
ENSG00000182916.7	TCEAL7	1.361270157	7.94216315	1.14E-08
ENSG00000138172.10	CALHM2	1.361180565	13.20192315	6.28E-14
ENSG00000112139.15	MDGA1	1.360289111	11.32289103	4.75E-12
ENSG00000110318.13	CEP126	1.360017748	5.49316525	3.21E-06
ENSG00000130813.17	C19orf66	1.359623765	16.56815319	2.70E-17
ENSG00000151117.8	TMEM86A	1.358843146	17.08781069	8.17E-18
ENSG00000112290.12	WASF1	1.358462825	3.859941686	0.000138
ENSG00000150867.13	PIP4K2A	1.358184638	16.92648471	1.18E-17
ENSG00000251675.1	AC010260.1	1.358038232	1.964332305	0.010856
ENSG00000225969.2	ABHD11-AS1	1.358024574	1.624585185	0.023736
ENSG00000174740.7	PABPC5	1.357937908	2.765892753	0.001714
ENSG00000186665.9	C17orf58	1.35749128	6.792419989	1.61E-07
ENSG00000160360.12	GPSM1	1.357350182	10.91636236	1.21E-11
ENSG00000153832.11	FBXO36	1.357079896	3.894028852	0.000128
ENSG00000137812.19	KNL1	1.355648323	1.846162186	0.014251
ENSG00000162543.5	UBXN10	1.355630625	3.35530787	0.000441
ENSG00000101307.15	SIRPB1	1.355496953	2.036220742	0.0092
ENSG00000099953.9	MMP11	1.355425553	8.25926489	5.50E-09
ENSG00000129911.8	KLF16	1.355227551	19.39825623	4.00E-20
ENSG00000176170.13	SPHK1	1.353937306	13.33893337	4.58E-14
ENSG00000044524.10	EPHA3	1.353903384	5.557438162	2.77E-06
ENSG00000279006.1	AL022323.2	1.353576609	2.073258002	0.008448
ENSG00000153208.16	MERTK	1.352994489	11.81209256	1.54E-12
ENSG00000237424.1	FOXD2-AS1	1.352758715	1.402479525	0.039584
ENSG00000258876.1	TGFB3-AS1	1.352624563	7.494087624	3.21E-08
ENSG00000105559.11	PLEKHA4	1.352494634	18.78564757	1.64E-19
ENSG00000254726.2	MEX3A	1.352418303	2.531415296	0.002942
ENSG00000261644.2	AC007728.2	1.351539098	10.32314546	4.75E-11
ENSG00000131981.15	LGALS3	1.350675586	15.70989033	1.95E-16
ENSG00000167291.15	TBC1D16	1.350289571	22.16263284	6.88E-23
ENSG00000115414.18	FN1	1.35026692	10.62566523	2.37E-11
ENSG00000174175.16	SELP	1.350049039	5.989934476	1.02E-06
ENSG00000168404.12	MLKL	1.349700069	14.39223435	4.05E-15

ENSG00000268230.5	AC012313.3	1.348736667	20.11673686	7.64E-21
ENSG00000165655.16	ZNF503	1.348532144	8.078301222	8.35E-09
ENSG00000055118.14	KCNH2	1.347746919	3.286303956	0.000517
ENSG00000140534.13	TICRR	1.347186277	6.387896104	4.09E-07
ENSG00000145246.13	ATP10D	1.347154799	10.57279134	2.67E-11
ENSG00000155254.12	MARVELD1	1.347128471	9.481885078	3.30E-10
ENSG00000134201.11	GSTM5	1.346941173	5.865292006	1.36E-06
ENSG00000273008.1	AC010864.1	1.345692728	1.602707975	0.024963
ENSG00000130592.15	LSP1	1.345337968	16.67199976	2.13E-17
ENSG00000101439.8	CST3	1.345141178	17.45715594	3.49E-18
ENSG00000180616.8	SSTR2	1.344716384	1.998980137	0.010024
ENSG00000076604.14	TRAF4	1.34461678	17.95733075	1.10E-18
ENSG00000129009.12	ISLR	1.344588818	9.263332242	5.45E-10
ENSG00000157456.7	CCNB2	1.344364725	1.732225265	0.018526
ENSG00000166128.12	RAB8B	1.343470175	14.11304661	7.71E-15
ENSG00000267387.1	AC020931.1	1.34319916	14.35122172	4.45E-15
ENSG00000107819.13	SFXN3	1.342916655	13.63266869	2.33E-14
ENSG00000134508.12	CABLES1	1.342640067	7.558274249	2.77E-08
ENSG00000189057.10	FAM111B	1.342001544	1.3523747	0.044425
ENSG00000136379.11	ABHD17C	1.341509054	5.938485984	1.15E-06
ENSG00000149212.11	SESN3	1.341373336	12.79967108	1.59E-13
ENSG00000183023.18	SLC8A1	1.340634647	16.03443414	9.24E-17
ENSG00000170962.12	PDGFD	1.34033721	5.158114268	6.95E-06
ENSG00000106003.12	LFNG	1.340061121	10.54245163	2.87E-11
ENSG00000136193.16	SCRN1	1.339397286	9.06432678	8.62E-10
ENSG00000106804.7	C5	1.339189844	3.559410125	0.000276
ENSG00000125618.16	PAX8	1.338939874	2.610200086	0.002454
ENSG00000111432.4	FZD10	1.338367433	6.021345195	9.52E-07
ENSG00000184408.9	KCND2	1.338113646	1.898501251	0.012633
ENSG00000096092.5	TMEM14A	1.337153258	10.83990366	1.45E-11
ENSG00000258920.1	FOXN3-AS1	1.336666293	2.421590892	0.003788
ENSG00000106366.8	SERPINE1	1.335485214	6.271986689	5.35E-07
ENSG00000164330.16	EBF1	1.334143777	8.977658712	1.05E-09
ENSG00000128656.13	CHN1	1.333391	14.15879041	6.94E-15
ENSG00000123560.13	PLP1	1.332428785	4.195891377	6.37E-05
ENSG00000088305.18	DNMT3B	1.331246701	8.944101342	1.14E-09
ENSG00000232878.3	DPYD-AS1	1.330770251	2.603126967	0.002494
ENSG00000163485.16	ADORA1	1.330719928	10.66560229	2.16E-11
ENSG00000177103.13	DSCAML1	1.330499707	3.224917175	0.000596
ENSG00000165124.17	SVEP1	1.330232494	7.131232117	7.39E-08
ENSG00000181418.7	DDN	1.32978714	2.946907736	0.00113
ENSG00000155011.8	DKK2	1.328515816	4.942948761	1.14E-05
ENSG00000255929.5	AP000943.3	1.327902143	6.422561156	3.78E-07
ENSG00000243749.1	TMEM35B	1.326797629	12.50703686	3.11E-13
ENSG00000196338.12	NLGN3	1.326535957	2.097222474	0.007994
ENSG00000156049.6	GNA14	1.326448571	3.955131118	0.000111
ENSG00000104518.10	GSDMD	1.326415615	19.65022755	2.24E-20

ENSG00000103723.13	AP3B2	1.326061479	2.731619754	0.001855
ENSG00000259201.1	AC090971.2	1.325962825	1.78591481	0.016371
ENSG00000111371.15	SLC38A1	1.325473445	6.452849895	3.52E-07
ENSG00000159674.11	SPON2	1.325010515	9.88453434	1.30E-10
ENSG00000134326.11	CMPK2	1.324764963	11.19695039	6.35E-12
ENSG00000072133.10	RPS6KA6	1.324645688	3.602910337	0.00025
ENSG00000154548.8	SRSF12	1.324559982	1.73312991	0.018487
ENSG00000076641.4	PAG1	1.323675361	10.92590159	1.19E-11
ENSG00000228436.2	AL139260.1	1.322571777	3.147811927	0.000712
ENSG00000073331.17	ALPK1	1.322527304	21.09176081	8.10E-22
ENSG00000232993.1	AL445183.1	1.322023205	6.921376251	1.20E-07
ENSG00000261276.1	AP003071.3	1.32118034	4.016910901	9.62E-05
ENSG00000204962.5	PCDHA8	1.320746639	2.307575064	0.004925
ENSG00000138642.14	HERC6	1.320639076	10.71473558	1.93E-11
ENSG00000118503.14	TNFAIP3	1.320248827	18.94844751	1.13E-19
ENSG00000177096.8	PHETA2	1.31976142	9.094960112	8.04E-10
ENSG00000267454.5	ZNF582-AS1	1.319075971	3.710654896	0.000195
ENSG00000198715.12	GLMP	1.319065731	16.21348122	6.12E-17
ENSG00000269973.1	AC010969.2	1.318572161	2.796511275	0.001598
ENSG00000254429.1	AP001972.1	1.318368773	5.836222354	1.46E-06
ENSG00000142089.15	IFITM3	1.317939467	9.797923872	1.59E-10
ENSG00000224818.1	AC096677.2	1.31763139	5.023640437	9.47E-06
ENSG00000197380.10	DACT3	1.317339671	7.972930859	1.06E-08
ENSG00000069966.18	GNB5	1.316721842	16.4441947	3.60E-17
ENSG00000172201.11	ID4	1.316388274	8.04169912	9.08E-09
ENSG00000101871.14	MID1	1.316032334	11.14114764	7.23E-12
ENSG00000118777.11	ABCG2	1.315981453	2.932510414	0.001168
ENSG00000248383.4	PCDHAC1	1.314362822	3.038175879	0.000916
ENSG00000148700.14	ADD3	1.314279873	8.977770804	1.05E-09
ENSG00000186767.6	SPIN4	1.314222814	4.507909076	3.11E-05
ENSG00000150637.8	CD226	1.313088044	6.425379565	3.76E-07
ENSG00000069493.14	CLEC2D	1.312855981	12.44169448	3.62E-13
ENSG00000103811.16	CTSH	1.312638707	9.870594001	1.35E-10
ENSG00000140406.3	TLNRD1	1.312317064	21.76485343	1.72E-22
ENSG00000135540.11	NHSL1	1.312285596	7.541429005	2.87E-08
ENSG00000119969.14	HELLS	1.311288656	8.149625606	7.09E-09
ENSG00000204970.9	PCDHA1	1.310742791	2.261319803	0.005479
ENSG00000250208.6	FZD10-DT	1.310062704	10.48850476	3.25E-11
ENSG00000163815.5	CLEC3B	1.309974994	6.977151917	1.05E-07
ENSG00000041353.9	RAB27B	1.309942166	1.466350868	0.03417
ENSG00000260103.2	AC012435.1	1.309195736	3.844248251	0.000143
ENSG00000254979.5	AP000781.2	1.308527868	9.07699431	8.38E-10
ENSG00000166888.11	STAT6	1.308010205	14.68287492	2.08E-15
ENSG00000183943.5	PRKX	1.307938265	15.46855263	3.40E-16
ENSG00000125746.16	EML2	1.307866804	21.64995243	2.24E-22
ENSG00000007237.18	GAS7	1.30785443	10.76734368	1.71E-11
ENSG00000156265.15	MAP3K7CL	1.307043466	4.468221107	3.40E-05

ENSG00000157617.16	C2CD2	1.30652552	18.62662932	2.36E-19
ENSG00000091317.7	CMTM6	1.30612645	13.19092023	6.44E-14
ENSG00000168079.16	SCARA5	1.305694672	6.394690863	4.03E-07
ENSG00000274922.1	AL139384.1	1.304893095	3.548421824	0.000283
ENSG00000283789.1	MIR7846	1.304152678	3.303280317	0.000497
ENSG00000228318.3	AP001610.1	1.30406373	6.958603569	1.10E-07
ENSG00000017427.16	IGF1	1.304016706	10.54422852	2.86E-11
ENSG00000163751.3	CPA3	1.303843513	3.350579319	0.000446
ENSG00000225216.6	AC007362.1	1.303196735	2.479122885	0.003318
ENSG00000204577.11	LILRB3	1.302844963	5.825844605	1.49E-06
ENSG00000137033.11	IL33	1.302616187	9.374184484	4.22E-10
ENSG00000110811.19	P3H3	1.302315876	7.575509523	2.66E-08
ENSG00000050327.14	ARHGEF5	1.301158854	4.246554048	5.67E-05
ENSG00000258130.8	AC106782.1	1.301140877	2.25080654	0.005613
ENSG00000262061.5	AC129507.1	1.300495983	17.85484573	1.40E-18
ENSG00000284762.1	AC022414.1	1.299979694	2.785177051	0.00164
ENSG00000077713.18	SLC25A43	1.299493305	11.84060831	1.44E-12
ENSG00000160293.16	VAV2	1.299312478	23.8542971	1.40E-24
ENSG00000088726.15	TMEM40	1.299001744	2.625170621	0.00237
ENSG00000166813.14	KIF7	1.298641696	11.07488442	8.42E-12
ENSG00000134802.17	SLC43A3	1.29848685	9.151087314	7.06E-10
ENSG00000186564.5	FOXD2	1.298014277	1.316979978	0.048197
ENSG00000243753.5	HLA-L	1.297963893	8.791781624	1.62E-09
ENSG00000197142.10	ACSL5	1.297752677	17.26546386	5.43E-18
ENSG00000097021.19	ACOT7	1.297680108	16.6625995	2.17E-17
ENSG00000119699.7	TGFB3	1.29731966	12.34795724	4.49E-13
ENSG00000149557.13	FEZ1	1.297250531	8.310360961	4.89E-09
ENSG00000077063.10	CTTNBP2	1.297087186	3.531000128	0.000294
ENSG00000154640.14	BTG3	1.297059903	12.56475721	2.72E-13
ENSG00000116774.11	OLFML3	1.296710515	7.68421632	2.07E-08
ENSG00000166086.12	JAM3	1.296313725	16.68030102	2.09E-17
ENSG00000158050.4	DUSP2	1.295745249	3.555420979	0.000278
ENSG00000135604.9	STX11	1.294980173	6.17292211	6.72E-07
ENSG00000167123.18	CERCAM	1.294504421	8.985855658	1.03E-09
ENSG00000059804.15	SLC2A3	1.294351507	10.36715615	4.29E-11
ENSG00000167554.14	ZNF610	1.294285028	3.407412523	0.000391
ENSG00000173546.7	CSPG4	1.293826716	12.9552666	1.11E-13
ENSG00000168453.14	HR	1.293760773	6.467756875	3.41E-07
ENSG00000148180.19	GSN	1.293446519	11.38436629	4.13E-12
ENSG00000134215.15	VAV3	1.292928266	4.221381936	6.01E-05
ENSG00000165272.15	AQP3	1.292697848	7.022257738	9.50E-08
ENSG00000267026.5	AL136084.2	1.29244255	2.07455604	0.008423
ENSG00000164946.19	FREM1	1.291672464	7.947676757	1.13E-08
ENSG00000258283.1	AC011603.3	1.29151805	2.897402312	0.001266
ENSG00000187994.13	RINL	1.29105601	13.24789589	5.65E-14
ENSG00000112559.13	MDFI	1.29070068	2.274777221	0.005312
ENSG00000261243.1	AC022165.1	1.290472383	1.857727558	0.013876

ENSG00000125089.16	SH3TC1		1.290418369	17.63860392	2.30E-18
ENSG00000017483.14	SLC38A5		1.290359001	1.892408588	0.012811
ENSG00000052126.14	PLEKHA5		1.290057139	11.34131191	4.56E-12
ENSG00000106571.13	GLI3		1.2900072	10.19846526	6.33E-11
ENSG00000111348.8	ARHGDIB		1.289780727	10.97119745	1.07E-11
ENSG00000141519.14	CCDC40		1.289317638	17.30301332	4.98E-18
ENSG00000279296.1	PRAL		1.288480735	6.530935264	2.94E-07
ENSG00000226445.1	BX322234.1		1.288052288	1.66127046	0.021814
ENSG00000204965.8	PCDHA5		1.287688878	2.422321688	0.003782
ENSG00000154736.5	ADAMTS5		1.287294779	3.941026989	0.000115
ENSG00000172197.10	MBOAT1		1.287226597	6.25104928	5.61E-07
ENSG00000225075.1	AL603832.1		1.286220746	4.195026495	6.38E-05
ENSG00000281344.1	HELLPAR		1.286027006	12.32133322	4.77E-13
ENSG00000119655.10	NPC2		1.285991197	12.47719779	3.33E-13
ENSG00000180096.11		1-Sep	1.285792475	7.531667223	2.94E-08
ENSG00000239389.7	PCDHA13		1.285396068	2.189434887	0.006465
ENSG00000100504.16	PYGL		1.283746195	10.1344232	7.34E-11
ENSG00000134363.11	FST		1.283498643	6.241427583	5.74E-07
ENSG00000197253.13	TPSB2		1.283115209	3.250798658	0.000561
ENSG00000134602.15	STK26		1.283108465	7.915963537	1.21E-08
ENSG00000203727.3	SAMD5		1.282849868	4.062901399	8.65E-05
ENSG00000103995.13	CEP152		1.282708801	6.004713746	9.89E-07
ENSG00000197696.9	NMB		1.281963514	2.617868808	0.002411
ENSG00000196169.14	KIF19		1.281955326	1.392688107	0.040487
ENSG00000166482.11	MFAP4		1.28114164	5.700152263	1.99E-06
ENSG00000256185.1	AC055720.2		1.280942619	3.326799725	0.000471
ENSG00000152061.23	RABGAP1L		1.280568382	14.99824204	1.00E-15
ENSG00000163814.7	CDCP1		1.280514761	4.436300753	3.66E-05
ENSG00000139514.12	SLC7A1		1.280368635	19.59919671	2.52E-20
ENSG00000184384.13	MAML2		1.280315947	14.67389495	2.12E-15
ENSG00000118707.9	TGIF2		1.280214075	16.90063286	1.26E-17
ENSG00000249859.9	PVT1		1.279454591	6.883870018	1.31E-07
ENSG00000089327.14	FXYD5		1.278935675	11.55822026	2.77E-12
ENSG00000141480.17	ARRB2		1.278764522	12.56721641	2.71E-13
ENSG00000280047.1	AC091825.1		1.277642727	3.428965988	0.000372
ENSG00000109743.10	BST1		1.277124112	7.345769156	4.51E-08
ENSG00000182218.9	HHIPL1		1.27708313	7.615148211	2.43E-08
ENSG00000269086.2	AC008555.2		1.276614552	1.575116665	0.0266
ENSG00000140090.17	SLC24A4		1.275707708	2.75850851	0.001744
ENSG00000125354.22		6-Sep	1.275112689	14.27055817	5.36E-15
ENSG00000212978.6	AC016747.1		1.274208949	9.67798962	2.10E-10
ENSG00000068724.15	TTC7A		1.27338353	13.7735202	1.68E-14
ENSG00000243232.4	PCDHAC2		1.273344054	3.335433555	0.000462
ENSG00000105472.12	CLEC11A		1.272487334	10.00476223	9.89E-11
ENSG00000113248.5	PCDHB15		1.27213276	5.749178999	1.78E-06
ENSG00000269190.5	FBXO17		1.271723911	7.842226934	1.44E-08
ENSG00000272154.4	AC244517.2		1.271560468	1.495068374	0.031984

ENSG00000162976.12	PQLC3	1.271187441	13.23879156	5.77E-14
ENSG00000232935.2	AL359094.2	1.271085293	3.150725171	0.000707
ENSG00000281453.1	TGFB2-OT1	1.270647464	13.17067097	6.75E-14
ENSG00000213793.5	ZNF888	1.268968034	7.045204956	9.01E-08
ENSG00000268516.2	AC020915.3	1.268636762	18.16350125	6.86E-19
ENSG00000145832.14	SLC25A48	1.26809648	1.355089391	0.044148
ENSG00000156427.7	FGF18	1.267985057	2.337975353	0.004592
ENSG00000066279.17	ASPM	1.267973831	2.283911017	0.005201
ENSG00000263874.2	LINC00672	1.26784485	2.67234066	0.002126
ENSG00000226137.5	BAIAP2-DT	1.267193538	12.425353	3.76E-13
ENSG00000111962.7	UST	1.266460431	8.744641094	1.80E-09
ENSG00000181649.6	PHLDA2	1.266346035	4.169445201	6.77E-05
ENSG00000240875.5	LINC00886	1.266343068	1.535238299	0.029158
ENSG00000184194.5	GPR173	1.266134224	9.218477537	6.05E-10
ENSG00000010327.10	STAB1	1.264918697	10.92586873	1.19E-11
ENSG00000169504.14	CLIC4	1.26387362	28.56948282	2.69E-29
ENSG00000072840.12	EVC	1.263157976	12.02679869	9.40E-13
ENSG00000135678.11	CPM	1.262124477	14.94434733	1.14E-15
ENSG00000275516.1	AC100791.3	1.261797829	10.30004528	5.01E-11
ENSG00000174348.13	PODN	1.261733304	6.754587149	1.76E-07
ENSG00000130590.13	SAMD10	1.261623567	7.602254884	2.50E-08
ENSG00000160318.6	CLDND2	1.260901541	2.392684593	0.004049
ENSG00000173040.12	EVC2	1.260801693	7.409254259	3.90E-08
ENSG00000183833.16	MAATS1	1.260696265	1.989666374	0.010241
ENSG00000142552.7	RCN3	1.260440739	8.54008014	2.88E-09
ENSG00000139910.19	NOVA1	1.259594002	7.190332138	6.45E-08
ENSG00000182957.15	SPATA13	1.258985289	16.44754543	3.57E-17
ENSG00000204963.5	PCDHA7	1.258413907	2.30215035	0.004987
ENSG00000112297.14	CRYBG1	1.258222943	17.220603	6.02E-18
ENSG00000214274.9	ANG	1.258146586	5.557438162	2.77E-06
ENSG00000173715.16	C11orf80	1.258132394	13.07203319	8.47E-14
ENSG00000137727.12	ARHGAP20	1.258058011	3.547248506	0.000284
ENSG00000172005.10	MAL	1.258032591	3.120814666	0.000757
ENSG00000153246.12	PLA2R1	1.256980511	4.960226621	1.10E-05
ENSG00000163297.16	ANTXR2	1.256766713	14.49092028	3.23E-15
ENSG00000121895.7	TMEM156	1.256561284	1.459575094	0.034708
ENSG00000235505.7	CASP17P	1.256427073	9.407285269	3.91E-10
ENSG00000139410.14	SDSL	1.256247123	3.968925348	0.000107
ENSG00000230587.1	LINC02580	1.25609776	1.566908449	0.027108
ENSG00000266441.1	AP005205.2	1.255667797	4.579707543	2.63E-05
ENSG00000103485.17	QPRT	1.255410528	11.66287918	2.17E-12
ENSG00000258761.1	AC116903.1	1.255001168	10.91391072	1.22E-11
ENSG00000254192.1	AC011365.2	1.254716035	4.091465299	8.10E-05
ENSG00000100362.12	PVALB	1.254367528	1.561003025	0.027479
ENSG00000248905.8	FMN1	1.253484314	7.515375635	3.05E-08
ENSG00000129521.13	EGLN3	1.252926273	9.073953472	8.43E-10
ENSG00000260892.1	AC105020.4	1.252902404	12.53683072	2.91E-13

ENSG00000165071.14	TMEM71	1.252720655	2.106728405	0.007821
ENSG00000162687.17	KCNT2	1.252441557	2.053376735	0.008843
ENSG00000112837.16	TBX18	1.252429791	6.05982184	8.71E-07
ENSG00000104324.15	CPQ	1.252178387	10.46099559	3.46E-11
ENSG00000273340.1	MICE	1.25212478	2.263188043	0.005455
ENSG00000108828.15	VAT1	1.251316043	9.561229137	2.75E-10
ENSG00000262246.5	CORO7	1.250698553	17.61103748	2.45E-18
ENSG00000099994.10	SUSD2	1.250595351	10.27099877	5.36E-11
ENSG00000151651.15	ADAM8	1.250482968	4.980559689	1.05E-05
ENSG00000127990.17	SGCE	1.250314471	10.75411926	1.76E-11
ENSG00000285269.2	AL160269.1	1.250306053	17.43235162	3.70E-18
ENSG00000178222.12	RNF212	1.250251199	4.180365631	6.60E-05
ENSG00000105419.17	MEIS3	1.249480877	9.955510493	1.11E-10
ENSG00000213064.9	SFT2D2	1.249358835	23.21805916	6.05E-24
ENSG00000232934.7	AL157786.1	1.249349607	9.53717311	2.90E-10
ENSG00000253194.2	AL137009.1	1.248604826	8.247244183	5.66E-09
ENSG00000177363.4	LRRN4CL	1.248599073	6.779358081	1.66E-07
ENSG00000198865.9	CCDC152	1.248587317	10.0378551	9.17E-11
ENSG00000177465.4	ACOT4	1.246359983	2.169287777	0.006772
ENSG00000083857.13	FAT1	1.246145548	13.95113879	1.12E-14
ENSG00000102409.9	BEX4	1.246141723	11.35346654	4.43E-12
ENSG00000165533.18	TTC8	1.245730345	9.207044571	6.21E-10
ENSG00000121236.20	TRIM6	1.245159956	3.541843895	0.000287
ENSG00000130958.12	SLC35D2	1.245097128	18.06558744	8.60E-19
ENSG00000081842.17	PCDHA6	1.244857766	2.221308392	0.006007
ENSG00000204967.10	PCDHA4	1.2444416	3.133477339	0.000735
ENSG00000204961.6	PCDHA9	1.24376512	2.202921913	0.006267
ENSG00000258451.1	AL163636.1	1.243112989	5.279976486	5.25E-06
ENSG00000213443.2	AC007068.1	1.243002337	10.33163513	4.66E-11
ENSG00000250120.6	PCDHA10	1.242416786	3.172522172	0.000672
ENSG00000197769.5	MAP1LC3C	1.242378204	2.565021474	0.002723
ENSG00000130589.16	HELZ2	1.241553018	11.21545254	6.09E-12
ENSG00000251664.3	PCDHA12	1.241425383	2.002091157	0.009952
ENSG00000163755.8	HPS3	1.240660183	17.67812916	2.10E-18
ENSG00000285218.1	AC026316.5	1.240068716	2.059833878	0.008713
ENSG00000157851.16	DPYSL5	1.239307653	2.101142817	0.007922
ENSG00000148154.9	UGCG	1.238525516	18.85585482	1.39E-19
ENSG00000165511.6	C10orf25	1.238131439	3.037758995	0.000917
ENSG00000153814.12	JAZF1	1.237030467	9.963823392	1.09E-10
ENSG00000099860.8	GADD45B	1.236908669	12.30688264	4.93E-13
ENSG00000170542.5	SERPINB9	1.236822717	13.18747332	6.49E-14
ENSG00000101773.18	RBBP8	1.236483359	9.76476933	1.72E-10
ENSG00000132256.18	TRIM5	1.236189953	15.16814939	6.79E-16
ENSG00000140937.13	CDH11	1.235688311	6.79946136	1.59E-07
ENSG00000205364.3	MT1M	1.234922714	2.551646035	0.002808
ENSG00000069399.14	BCL3	1.234550614	14.054012	8.83E-15
ENSG00000172345.13	STARD5	1.233355935	8.218914691	6.04E-09

ENSG00000279204.1	AC134043.2	1.233042225	4.378728715	4.18E-05
ENSG00000196935.8	SRGAP1	1.232767253	11.55417645	2.79E-12
ENSG00000134323.11	MYCN	1.232682577	4.270231598	5.37E-05
ENSG00000057252.12	SOAT1	1.229870675	14.61481007	2.43E-15
ENSG00000273413.1	AL136982.7	1.228963034	6.210100486	6.16E-07
ENSG00000082497.11	SERTAD4	1.228463356	5.968899446	1.07E-06
ENSG00000078098.13	FAP	1.226770305	6.407970509	3.91E-07
ENSG00000079435.9	LIPE	1.226604149	3.199851564	0.000631
ENSG00000188185.11	LINC00265	1.225272757	6.702904121	1.98E-07
ENSG00000152503.9	TRIM36	1.225236305	1.761511986	0.017318
ENSG00000129465.15	RIPK3	1.224406485	9.418546226	3.81E-10
ENSG00000183935.5	HTR7P1	1.224396476	4.25242529	5.59E-05
ENSG00000161929.14	SCIMP	1.224383965	10.12286635	7.54E-11
ENSG00000081803.15	CADPS2	1.224077868	9.504163481	3.13E-10
ENSG00000168140.4	VASN	1.224038756	3.91918979	0.00012
ENSG00000123473.15	STIL	1.223694447	1.63826776	0.023
ENSG00000267598.1	AC011446.2	1.223681771	10.46787073	3.41E-11
ENSG00000139970.16	RTN1	1.2231563	6.069971614	8.51E-07
ENSG00000158710.14	TAGLN2	1.222339238	11.87805257	1.32E-12
ENSG00000197766.7	CFD	1.22220381	5.726167491	1.88E-06
ENSG00000138061.11	CYP1B1	1.221604426	7.189023343	6.47E-08
ENSG00000205593.11	DENND6B	1.221505914	11.16918018	6.77E-12
ENSG00000175471.19	MCTP1	1.221363692	8.648645745	2.25E-09
ENSG00000181381.13	DDX60L	1.221108141	9.353158848	4.43E-10
ENSG00000236830.6	CBR3-AS1	1.220329714	8.18249626	6.57E-09
ENSG00000172260.14	NEGR1	1.219578078	7.434241463	3.68E-08
ENSG00000184349.12	EFNA5	1.219577191	4.977699487	1.05E-05
ENSG00000198753.11	PLXNB3	1.217711963	6.029059632	9.35E-07
ENSG00000109113.19	RAB34	1.217056454	8.977979032	1.05E-09
ENSG00000141391.13	PRELID3A	1.215917261	1.59534877	0.025389
ENSG00000092969.11	TGFB2	1.215640904	11.05236595	8.86E-12
ENSG00000143867.6	OSR1	1.215611887	9.187398297	6.50E-10
ENSG00000284060.1	AC002472.2	1.21560029	4.383991884	4.13E-05
ENSG00000184500.15	PROS1	1.215458543	7.227270442	5.93E-08
ENSG00000261485.1	PAN3-AS1	1.21523022	1.553320543	0.027969
ENSG00000188313.12	PLSCR1	1.213597951	9.25104294	5.61E-10
ENSG00000189143.9	CLDN4	1.213196149	3.206431821	0.000622
ENSG00000146094.14	DOK3	1.212960432	11.04472745	9.02E-12
ENSG00000231064.7	AC234582.1	1.21279907	9.37121975	4.25E-10
ENSG00000237649.7	KIFC1	1.21251241	4.516469424	3.04E-05
ENSG00000279425.1	AC092279.2	1.21200412	1.734838648	0.018415
ENSG00000124212.5	PTGIS	1.210621795	3.76934766	0.00017
ENSG00000272398.5	CD24	1.210587892	3.227639646	0.000592
ENSG00000137161.16	CNPY3	1.209438852	15.45915212	3.47E-16
ENSG00000177839.6	PCDHB9	1.208653046	1.905845945	0.012421
ENSG00000274396.1	RF01978	1.206981082	2.353079243	0.004435
ENSG00000204969.6	PCDHA2	1.206922889	3.382291887	0.000415

ENSG00000070190.12	DAPP1	1.20682016	4.538449705	2.89E-05
ENSG00000148671.13	ADIRF	1.206625705	6.201198194	6.29E-07
ENSG00000166801.15	FAM111A	1.20631162	15.95011949	1.12E-16
ENSG00000221874.4	ZNF816-ZNF321P	1.205117582	2.914043487	0.001219
ENSG00000277481.1	PKD1L3	1.20441622	1.80376024	0.015712
ENSG00000263424.1	AC110597.3	1.202584643	7.239022194	5.77E-08
ENSG00000277443.2	MARCKS	1.202147651	11.03521094	9.22E-12
ENSG00000198805.11	PNP	1.201601571	10.95616563	1.11E-11
ENSG00000123572.16	NRK	1.201110916	3.177458059	0.000665
ENSG00000204851.6	PNMA8B	1.201068247	5.042206667	9.07E-06
ENSG00000118961.14	LDAH	1.200988098	7.26416358	5.44E-08
ENSG00000168477.19	TNXB	1.199267586	6.236677473	5.80E-07
ENSG00000089472.16	HEPH	1.199215697	6.18213859	6.57E-07
ENSG00000267580.1	AC008738.3	1.199137615	6.145638313	7.15E-07
ENSG00000263050.1	AC090617.5	1.198535253	1.421415268	0.037895
ENSG00000149418.10	ST14	1.198443151	5.394913519	4.03E-06
ENSG00000235531.9	MSC-AS1	1.197970272	17.04441542	9.03E-18
ENSG00000162745.10	OLFML2B	1.197868293	10.6656929	2.16E-11
ENSG00000185201.16	IFITM2	1.197714071	8.52199189	3.01E-09
ENSG00000177663.13	IL17RA	1.197579975	10.14005778	7.24E-11
ENSG00000135426.15	TESPA1	1.197015896	2.912294147	0.001224
ENSG00000105245.9	NUMBL	1.196268031	15.6700839	2.14E-16
ENSG00000005448.16	WDR54	1.195798184	11.62890109	2.35E-12
ENSG00000134533.6	RERG	1.195359065	4.895153463	1.27E-05
ENSG00000156011.16	PSD3	1.195202307	7.119205924	7.60E-08
ENSG00000135333.13	EPHA7	1.194925679	1.890071809	0.01288
ENSG00000132357.13	CARD6	1.194822526	9.777511795	1.67E-10
ENSG00000111644.7	ACRBP	1.193666543	1.717709897	0.019155
ENSG00000284052.1	AC006460.2	1.193623358	2.879685493	0.001319
ENSG00000226145.7	KRT16P6	1.19304465	2.117084113	0.007637
ENSG00000010810.17	FYN	1.192984801	11.54728199	2.84E-12
ENSG00000261731.2	AC074050.3	1.191661405	1.350050927	0.044663
ENSG00000163584.17	RPL22L1	1.191203059	7.380947504	4.16E-08
ENSG00000165359.15	INTS6L	1.190709237	9.537125504	2.90E-10
ENSG00000143369.14	ECM1	1.190420117	6.996061688	1.01E-07
ENSG00000120820.12	GLT8D2	1.190275325	6.100540998	7.93E-07
ENSG00000273179.1	AC092535.4	1.189868473	3.608255386	0.000246
ENSG00000235718.8	MFRP	1.189206208	4.489877011	3.24E-05
ENSG00000169231.13	THBS3	1.189016459	9.797757816	1.59E-10
ENSG00000101104.12	PABPC1L	1.188785429	6.217874583	6.06E-07
ENSG00000180921.6	FAM83H	1.188267277	5.673068705	2.12E-06
ENSG00000100842.12	EFS	1.187904493	7.136526206	7.30E-08
ENSG00000232811.1	AL360270.1	1.187101209	1.658941101	0.021931
ENSG00000223953.5	C1QTNF5	1.186528638	4.458406127	3.48E-05
ENSG00000178927.17	CYBC1	1.186084681	16.86027989	1.38E-17
ENSG00000185561.9	TLCD2	1.185233801	8.498627989	3.17E-09
ENSG00000105011.8	ASF1B	1.184378947	3.278980435	0.000526

ENSG00000116337.15	AMPD2	1.183868367	14.23418413	5.83E-15
ENSG00000149534.8	MS4A2	1.183537658	3.150179257	0.000708
ENSG00000118855.18	MFSD1	1.183302341	15.27534552	5.30E-16
ENSG00000270228.1	AC079880.1	1.182770351	6.735406559	1.84E-07
ENSG00000118402.5	ELOVL4	1.182455892	1.525930683	0.02979
ENSG00000134996.11	OSTF1	1.182414238	15.1902438	6.45E-16
ENSG00000196139.13	AKR1C3	1.182137292	7.055344029	8.80E-08
ENSG00000171940.13	ZNF217	1.181925917	20.12507954	7.50E-21
ENSG00000245848.2	CEBPA	1.181602076	5.852937905	1.40E-06
ENSG00000148288.12	GBGT1	1.181562554	8.123723845	7.52E-09
ENSG00000259007.1	AL358333.3	1.181559098	10.10787731	7.80E-11
ENSG00000197702.12	PARVA	1.181500485	10.63571877	2.31E-11
ENSG00000018408.14	WWTR1	1.18095663	22.71792753	1.91E-23
ENSG00000174705.12	SH3PXD2B	1.180843399	10.85378689	1.40E-11
ENSG00000167106.11	FAM102A	1.18078026	17.7242409	1.89E-18
ENSG00000284882.1	AL359762.1	1.179981318	3.525415544	0.000298
ENSG00000142669.14	SH3BGRL3	1.179922756	10.96282701	1.09E-11
ENSG00000213799.12	ZNF845	1.178765271	14.01985469	9.55E-15
ENSG00000259948.2	AC124068.1	1.178575901	19.40734638	3.91E-20
ENSG00000284191.1	MIR671	1.178449378	9.439973379	3.63E-10
ENSG00000179820.15	MYADM	1.178442688	12.63843182	2.30E-13
ENSG00000167994.11	RAB3IL1	1.178150071	8.224607117	5.96E-09
ENSG00000183722.8	LHFPL6	1.178092075	5.302905533	4.98E-06
ENSG00000171357.5	LURAP1	1.178062796	1.752143754	0.017695
ENSG00000231925.11	TAPBP	1.177992886	22.87802999	1.32E-23
ENSG00000108771.12	DHX58	1.177370016	11.79535096	1.60E-12
ENSG00000257086.1	AP001453.3	1.177231872	3.330547409	0.000467
ENSG00000185222.9	TCEAL9	1.177085708	8.555278239	2.78E-09
ENSG00000146242.8	TPBG	1.176107572	10.27240111	5.34E-11
ENSG00000179431.6	FJX1	1.175859118	7.821374429	1.51E-08
ENSG00000235978.6	AC018816.1	1.175571054	9.579088011	2.64E-10
ENSG00000107968.9	MAP3K8	1.174626283	11.46141652	3.46E-12
ENSG00000266028.7	SRGAP2	1.174602749	19.80914788	1.55E-20
ENSG00000078596.10	ITM2A	1.174488024	9.830292476	1.48E-10
ENSG00000143850.14	PLEKHA6	1.174230802	9.140383252	7.24E-10
ENSG00000172935.8	MRGPRF	1.173685152	5.003273919	9.92E-06
ENSG00000168792.4	ABHD15	1.173197187	15.00380695	9.91E-16
ENSG00000005884.17	ITGA3	1.172825351	10.48217366	3.29E-11
ENSG00000137509.10	PRCP	1.172787817	10.66452444	2.17E-11
ENSG00000008283.15	CYB561	1.172508948	12.69669031	2.01E-13
ENSG00000143845.14	ETNK2	1.17233478	4.282960129	5.21E-05
ENSG00000154277.12	UCHL1	1.172327808	7.479888667	3.31E-08
ENSG00000206384.10	COL6A6	1.170553397	3.74064861	0.000182
ENSG00000203644.3	AC083799.1	1.169646802	4.164373422	6.85E-05
ENSG00000237187.8	NR2F1-AS1	1.169537272	1.971950464	0.010667
ENSG00000028137.18	TNFRSF1B	1.169421532	12.25601057	5.55E-13
ENSG00000011028.13	MRC2	1.16934863	7.430639433	3.71E-08

ENSG00000258908.1	AL355075.3	1.169205164	6.32824113	4.70E-07
ENSG00000153815.16	CMIP	1.168743139	15.07364607	8.44E-16
ENSG00000100918.12	REC8	1.167195261	9.049676248	8.92E-10
ENSG00000110660.14	SLC35F2	1.167137418	11.82927094	1.48E-12
ENSG00000261115.5	TMEM178B	1.167004811	5.441371987	3.62E-06
ENSG00000228889.6	UBAC2-AS1	1.166484537	4.227396777	5.92E-05
ENSG00000232220.2	AC008440.2	1.166114131	10.38090396	4.16E-11
ENSG00000103966.10	EHD4	1.16604308	14.01692991	9.62E-15
ENSG00000134250.19	NOTCH2	1.165658176	11.37257622	4.24E-12
ENSG00000170955.9	CAVIN3	1.16555969	15.54002389	2.88E-16
ENSG00000157191.19	NECAP2	1.165095737	13.37676574	4.20E-14
ENSG00000113758.13	DBN1	1.164939391	10.93755327	1.15E-11
ENSG00000132530.16	XAF1	1.164083315	5.918584764	1.21E-06
ENSG00000137269.14	LRRC1	1.163496445	5.798813156	1.59E-06
ENSG00000131650.13	KREMEN2	1.162620087	1.504760462	0.031278
ENSG00000223855.1	HRAT92	1.162445243	2.166655575	0.006813
ENSG00000163235.15	TGFA	1.161352164	1.511396863	0.030804
ENSG00000147485.12	PXDNL	1.161026511	4.04686475	8.98E-05
ENSG00000174007.7	CEP19	1.160759133	3.138552934	0.000727
ENSG00000281358.1	RASSF1-AS1	1.160362839	10.50657381	3.11E-11
ENSG00000225963.7	AC009950.1	1.160309135	9.164033124	6.85E-10
ENSG00000283938.1	MIR3917	1.160220555	13.90136918	1.25E-14
ENSG00000234390.4	USP27X-AS1	1.159913645	1.88197331	0.013123
ENSG00000164176.12	EDIL3	1.159887636	4.319166206	4.80E-05
ENSG00000243364.7	EFNA4	1.159607592	3.629780834	0.000235
ENSG00000183098.10	GPC6	1.159552203	8.529107875	2.96E-09
ENSG00000270021.1	AC026691.1	1.158546585	2.985873053	0.001033
ENSG00000122861.15	PLAU	1.158150915	16.17710193	6.65E-17
ENSG00000145050.16	MANF	1.157978076	14.67478582	2.11E-15
ENSG00000101224.17	CDC25B	1.157742186	13.46557646	3.42E-14
ENSG00000245812.2	LINC02202	1.157554815	5.076343346	8.39E-06
ENSG00000130303.12	BST2	1.157381555	7.692813469	2.03E-08
ENSG00000224888.4	AC138028.2	1.157192807	11.81358508	1.54E-12
ENSG00000238164.6	TNFRSF14-AS1	1.156234547	12.76179905	1.73E-13
ENSG00000139117.13	CPNE8	1.156016357	11.13465295	7.33E-12
ENSG00000198429.9	ZNF69	1.155664947	1.667045415	0.021526
ENSG00000121858.10	TNFSF10	1.154876322	12.48464695	3.28E-13
ENSG00000119514.6	GALNT12	1.153613264	3.838063528	0.000145
ENSG00000284773.1	AC114490.3	1.152823437	11.0321158	9.29E-12
ENSG00000172346.14	CSDC2	1.152122616	20.41903224	3.81E-21
ENSG00000203876.9	ADD3-AS1	1.151781663	4.344800163	4.52E-05
ENSG00000269652.1	AC011510.1	1.150758663	5.95355985	1.11E-06
ENSG00000116661.10	FBXO2	1.150572409	3.307143561	0.000493
ENSG00000242715.7	CCDC169	1.150522722	1.51298269	0.030691
ENSG00000276384.1	AC016876.3	1.150283051	4.237235745	5.79E-05
ENSG00000255867.1	DENND5B-AS1	1.150208174	1.820878909	0.015105
ENSG00000250722.5	SELENOP	1.149969183	9.011789382	9.73E-10

ENSG00000280206.1	AC026401.3	1.149641573	1.318624868	0.048015
ENSG00000249307.5	LINC01088	1.149321785	1.379598494	0.041725
ENSG00000258525.1	AL049830.3	1.149244804	1.875407689	0.013323
ENSG00000135378.3	PRRG4	1.148389956	5.194167612	6.39E-06
ENSG00000152784.15	PRDM8	1.148312001	12.85355863	1.40E-13
ENSG00000130147.15	SH3BP4	1.14808111	13.10522169	7.85E-14
ENSG00000173846.12	PLK3	1.14788963	12.32254821	4.76E-13
ENSG00000077150.18	NFKB2	1.147793749	13.86514579	1.36E-14
ENSG00000116678.19	LEPR	1.147583134	4.537572241	2.90E-05
ENSG00000152056.16	AP1S3	1.147498812	1.416428733	0.038333
ENSG00000231437.3	LINC01750	1.147396741	2.166823342	0.00681
ENSG00000269001.2	AC092070.2	1.147001519	9.979730052	1.05E-10
ENSG00000222047.8	C10orf55	1.146943015	13.82708963	1.49E-14
ENSG00000223768.2	LINC00205	1.146682082	14.29526369	5.07E-15
ENSG00000254042.1	AC011365.1	1.145739663	2.104304808	0.007865
ENSG00000141753.6	IGFBP4	1.14509224	9.910346222	1.23E-10
ENSG00000274259.2	SYNGAP1-AS1	1.144792786	3.606014671	0.000248
ENSG00000188707.5	ZBED6CL	1.144331048	2.485041487	0.003273
ENSG00000162733.17	DDR2	1.144161267	4.81072269	1.55E-05
ENSG00000198189.10	HSD17B11	1.143903418	8.313391033	4.86E-09
ENSG00000240891.7	PLCXD2	1.143810677	3.442709662	0.000361
ENSG00000118762.7	PKD2	1.143754462	8.337897688	4.59E-09
ENSG00000260092.1	AC009163.2	1.143701787	1.561570125	0.027443
ENSG00000197497.10	ZNF665	1.143517633	4.034432222	9.24E-05
ENSG00000257878.1	AC007298.2	1.142583796	2.308325828	0.004917
ENSG00000228703.1	AL355310.2	1.142033421	10.66451041	2.17E-11
ENSG00000118257.16	NRP2	1.141934937	9.333421781	4.64E-10
ENSG00000227107.1	AC104667.1	1.14153672	1.31917006	0.047955
ENSG00000110852.4	CLEC2B	1.14112221	10.53011933	2.95E-11
ENSG00000139668.8	WDFY2	1.140728989	12.77051903	1.70E-13
ENSG00000110719.9	TCIRG1	1.140717195	9.735937731	1.84E-10
ENSG00000158246.7	TENT5B	1.140327688	1.6685065	0.021453
ENSG00000176387.6	HSD11B2	1.140325322	3.365651002	0.000431
ENSG00000235257.8	ITGA9-AS1	1.140134928	11.33764328	4.60E-12
ENSG00000177674.15	AGTRAP	1.140108573	17.00303983	9.93E-18
ENSG00000153707.16	PTPRD	1.138798734	7.087357116	8.18E-08
ENSG00000136205.16	TNS3	1.138776651	10.79669564	1.60E-11
ENSG00000145824.12	CXCL14	1.138324014	6.188068011	6.49E-07
ENSG00000258583.6	LINC01500	1.137551734	3.301836189	0.000499
ENSG00000123384.13	LRP1	1.136294486	6.440678562	3.63E-07
ENSG00000284627.1	AC079781.4	1.135967654	1.381372204	0.041555
ENSG00000164054.15	SHISA5	1.135565702	12.85071202	1.41E-13
ENSG00000111912.19	NCOA7	1.13500251	10.71558496	1.92E-11
ENSG00000181894.14	ZNF329	1.134919354	12.13068359	7.40E-13
ENSG00000166997.7	CNPY4	1.134693242	9.000964032	9.98E-10
ENSG00000178662.15	CSRNP3	1.133848964	1.376034678	0.042069
ENSG00000160888.6	IER2	1.133523089	10.66764734	2.15E-11

ENSG00000105088.8	OLFM2	1.133509441	4.374947331	4.22E-05
ENSG00000175538.10	KCNE3	1.133397405	6.009914248	9.77E-07
ENSG00000250602.6	AC093535.1	1.13317124	2.852636346	0.001404
ENSG00000248774.1	AC097534.1	1.132933592	2.620974251	0.002393
ENSG00000255882.1	AC091814.1	1.132787774	8.851964297	1.41E-09
ENSG00000261114.1	AC012181.1	1.132658591	3.982820485	0.000104
ENSG00000116396.14	KCNC4	1.132474511	6.353047632	4.44E-07
ENSG00000268996.3	MAN1B1-DT	1.132434904	10.87884818	1.32E-11
ENSG00000276919.1	RF01872	1.132309268	7.978320932	1.05E-08
ENSG00000203326.11	ZNF525	1.132194161	3.740596639	0.000182
ENSG00000249158.6	PCDHA11	1.131519795	1.983590873	0.010385
ENSG00000265168.1	AC005726.4	1.13131741	2.965253587	0.001083
ENSG00000120324.8	PCDHB10	1.130884561	3.717250983	0.000192
ENSG00000142694.6	EVA1B	1.130225948	8.346937313	4.50E-09
ENSG00000137441.7	FGFBP2	1.130180475	1.327131587	0.047083
ENSG00000116819.7	TFAP2E	1.12971979	5.454099327	3.51E-06
ENSG00000000938.12	FGR	1.12967823	4.035574414	9.21E-05
ENSG00000104611.11	SH2D4A	1.128009201	3.536644268	0.000291
ENSG00000139679.15	LPAR6	1.128005354	9.493691302	3.21E-10
ENSG00000269243.1	AC008894.2	1.127852662	12.2993156	5.02E-13
ENSG00000102359.6	SRPX2	1.127454972	4.848800135	1.42E-05
ENSG00000103335.22	PIEZO1	1.126746476	13.93841038	1.15E-14
ENSG00000267561.2	AC093155.3	1.12625263	9.052898558	8.85E-10
ENSG00000153558.15	FBXL2	1.126049049	4.516795803	3.04E-05
ENSG00000174586.10	ZNF497	1.125898237	13.45959027	3.47E-14
ENSG00000014914.20	MTMR11	1.12581716	4.8320593	1.47E-05
ENSG00000203760.8	CENPW	1.125765615	3.238530152	0.000577
ENSG00000117632.22	STMN1	1.125428895	16.5655865	2.72E-17
ENSG00000179277.9	MEIS3P1	1.125288825	5.072847146	8.46E-06
ENSG00000135596.17	MICAL1	1.124999016	15.06387975	8.63E-16
ENSG00000087086.14	FTL	1.12495842	12.05714101	8.77E-13
ENSG00000171219.8	CDC42BPG	1.124771601	2.008675383	0.009802
ENSG00000001036.13	FUCA2	1.124396627	12.15782391	6.95E-13
ENSG00000104321.10	TRPA1	1.124363545	5.710662751	1.95E-06
ENSG00000224958.5	PGM5-AS1	1.123880218	3.747918343	0.000179
ENSG00000165633.12	VSTM4	1.123555441	12.15305652	7.03E-13
ENSG00000026652.13	AGPAT4	1.123446058	9.035250563	9.22E-10
ENSG00000153790.11	C7orf31	1.122927326	2.612025197	0.002443
ENSG00000234336.6	JAZF1-AS1	1.122625987	2.688338602	0.00205
ENSG00000171451.13	DSEL	1.121524875	10.28596045	5.18E-11
ENSG00000182240.15	BACE2	1.121490786	8.994759481	1.01E-09
ENSG00000184117.11	NIPSNAP1	1.12060928	25.83931873	1.45E-26
ENSG00000136826.14	KLF4	1.120346867	7.369084122	4.27E-08
ENSG00000256072.1	AC078889.1	1.120053233	1.457640674	0.034863
ENSG00000282556.2	AC068733.3	1.120017632	15.11905626	7.60E-16
ENSG00000196381.10	ZNF781	1.119374299	2.122732241	0.007538
ENSG00000142606.15	MMEL1	1.119371139	7.643131672	2.27E-08

ENSG00000100479.12	POLE2	1.119118077	3.46300974	0.000344
ENSG00000140526.17	ABHD2	1.118964528	19.99004126	1.02E-20
ENSG00000128805.14	ARHGAP22	1.11868938	9.664784286	2.16E-10
ENSG00000228288.6	PCAT6	1.118515926	2.045937362	0.008996
ENSG00000189190.10	ZNF600	1.118363317	9.25104294	5.61E-10
ENSG00000135899.17	SP110	1.118092797	13.50613821	3.12E-14
ENSG00000248144.5	ADH1C	1.11777888	4.761252228	1.73E-05
ENSG00000169851.15	PCDH7	1.117020109	5.893323229	1.28E-06
ENSG00000272040.1	AC010245.2	1.115633182	2.178613514	0.006628
ENSG00000142173.14	COL6A2	1.115419985	9.244667876	5.69E-10
ENSG00000102871.15	TRADD	1.11465644	18.32523729	4.73E-19
ENSG00000267727.1	AC008738.5	1.114616834	4.854632705	1.40E-05
ENSG00000152804.10	HHEX	1.113566031	6.988644568	1.03E-07
ENSG00000181896.11	ZNF101	1.113336637	11.65920636	2.19E-12
ENSG00000128228.4	SDF2L1	1.113134726	12.24653942	5.67E-13
ENSG00000184347.14	SLIT3	1.112709101	4.050657806	8.90E-05
ENSG00000146350.13	TBC1D32	1.1126181	2.618701018	0.002406
ENSG00000240758.2	AC010655.2	1.112558115	4.818870635	1.52E-05
ENSG00000120328.6	PCDHB12	1.112393243	2.450067309	0.003548
ENSG00000205133.11	TRIQK	1.112024743	7.816559976	1.53E-08
ENSG00000077274.8	CAPN6	1.111718381	4.785222122	1.64E-05
ENSG00000013288.8	MAN2B2	1.111510623	11.35983242	4.37E-12
ENSG00000167004.12	PDIA3	1.111215409	16.90039369	1.26E-17
ENSG00000110076.18	NRXN2	1.111052639	9.828524272	1.48E-10
ENSG00000160410.14	SHKBP1	1.110662611	15.43304213	3.69E-16
ENSG00000279713.1	AC080038.3	1.110522648	6.361718572	4.35E-07
ENSG00000251246.1	AL691442.1	1.110386916	2.140680507	0.007233
ENSG00000109814.11	UGDH	1.109812076	7.942424381	1.14E-08
ENSG00000089505.17	CMTM1	1.109473997	4.013606936	9.69E-05
ENSG00000253276.2	CCDC71L	1.109186033	11.4039301	3.95E-12
ENSG00000167601.11	AXL	1.108872877	6.756875036	1.75E-07
ENSG00000167642.12	SPINT2	1.108859687	7.794441707	1.61E-08
ENSG00000081059.19	TCF7	1.108802084	5.517851565	3.03E-06
ENSG00000255723.1	AC108516.2	1.108427564	6.929902931	1.18E-07
ENSG00000008517.16	IL32	1.107375375	4.124447298	7.51E-05
ENSG00000136378.14	ADAMTS7	1.106894256	8.316995266	4.82E-09
ENSG00000243055.1	GK-AS1	1.106211522	3.082861091	0.000826
ENSG00000142875.19	PRKACB	1.106107757	17.04056153	9.11E-18
ENSG00000120327.6	PCDHB14	1.105888195	8.335246239	4.62E-09
ENSG00000138801.8	PAPSS1	1.10549221	15.74864906	1.78E-16
ENSG00000214226.8	C17orf67	1.105257735	2.786536573	0.001635
ENSG00000138080.13	EMILIN1	1.105063992	8.861621839	1.38E-09
ENSG00000099204.19	ABLIM1	1.104526874	10.50657381	3.11E-11
ENSG00000223546.6	LINC00630	1.104480684	1.510898968	0.030839
ENSG00000161888.11	SPC24	1.10413714	3.625205689	0.000237
ENSG00000108798.8	ABI3	1.10373665	8.285853957	5.18E-09
ENSG00000087253.12	LPCAT2	1.103720647	10.3021943	4.99E-11

ENSG00000030582.17	GRN	1.103049289	17.7071748	1.96E-18
ENSG00000255408.3	PCDHA3	1.102906579	2.343353213	0.004536
ENSG00000162512.15	SDC3	1.102716327	11.82781878	1.49E-12
ENSG00000204003.8	AL355987.1	1.101919197	2.334100132	0.004633
ENSG00000104903.4	LYL1	1.101158255	7.671101087	2.13E-08
ENSG00000143502.14	SUSD4	1.101127006	3.253748116	0.000558
ENSG00000182732.17	RGS6	1.100449857	4.809309494	1.55E-05
ENSG00000260121.1	AC138028.4	1.100045278	11.14948217	7.09E-12
ENSG00000120899.17	PTK2B	1.100031789	16.15765935	6.96E-17
ENSG00000284879.1	AC133644.3	1.10002334	12.68696989	2.06E-13
ENSG00000183580.9	FBXL7	1.099129441	8.07497464	8.41E-09
ENSG00000070882.12	OSBPL3	1.098645511	6.327121242	4.71E-07
ENSG00000090661.11	CERS4	1.09858195	6.911648338	1.23E-07
ENSG00000136059.14	VILL	1.098320013	10.29434635	5.08E-11
ENSG00000155849.15	ELMO1	1.096107782	9.668791862	2.14E-10
ENSG00000111404.6	RERGL	1.096033482	2.464818654	0.003429
ENSG00000186310.9	NAP1L3	1.095421956	4.841215301	1.44E-05
ENSG00000187554.12	TLR5	1.095326988	9.354707044	4.42E-10
ENSG00000144857.14	BOC	1.095011796	8.980476149	1.05E-09
ENSG00000069702.10	TGFBR3	1.094987734	4.788300736	1.63E-05
ENSG00000272821.1	U62317.2	1.094906739	10.9639943	1.09E-11
ENSG00000168679.17	SLC16A4	1.094240753	6.754587149	1.76E-07
ENSG00000283078.1	AL137077.2	1.093260795	1.416344936	0.03834
ENSG00000112655.15	PTK7	1.092636587	8.554085853	2.79E-09
ENSG00000196369.11	SRGAP2B	1.091615191	7.762875422	1.73E-08
ENSG00000169554.19	ZEB2	1.091274794	17.11061738	7.75E-18
ENSG00000245556.2	SCAMP1-AS1	1.091090057	6.981271307	1.04E-07
ENSG00000240207.6	AC080013.1	1.090991719	2.549939703	0.002819
ENSG00000198346.10	ZNF813	1.089947412	6.818596859	1.52E-07
ENSG00000245571.6	FAM111A-DT	1.089689927	6.994273341	1.01E-07
ENSG00000084070.11	SMAP2	1.089509728	13.36278492	4.34E-14
ENSG00000145107.15	TM4SF19	1.089474477	2.293588653	0.005086
ENSG00000119782.13	FKBP1B	1.08924688	2.793929197	0.001607
ENSG00000168060.15	NAALADL1	1.089115262	7.044201222	9.03E-08
ENSG00000184979.9	USP18	1.088572511	10.81156062	1.54E-11
ENSG00000261270.1	AC012181.2	1.088433862	4.358096435	4.38E-05
ENSG00000100599.15	RIN3	1.08828721	11.40909482	3.90E-12
ENSG00000146555.18	SDK1	1.087681512	5.50147412	3.15E-06
ENSG00000196743.8	GM2A	1.08754729	17.63165457	2.34E-18
ENSG00000138639.17	ARHGAP24	1.087208259	15.12558244	7.49E-16
ENSG00000180801.13	ARSJ	1.087191348	3.198962259	0.000632
ENSG00000135902.9	CHRND	1.085968118	3.64125126	0.000228
ENSG00000232931.5	LINC00342	1.085658327	7.241518094	5.73E-08
ENSG00000227751.1	AC004824.1	1.084685792	8.08611369	8.20E-09
ENSG00000244405.7	ETV5	1.082910336	7.789093111	1.63E-08
ENSG00000149256.15	TENM4	1.08240939	9.63416982	2.32E-10
ENSG00000136040.8	PLXNC1	1.081593576	8.906387054	1.24E-09

ENSG00000134287.9	ARF3	1.081305183	14.19905597	6.32E-15
ENSG00000272734.1	ADIRF-AS1	1.08021734	5.714385242	1.93E-06
ENSG00000013297.11	CLDN11	1.079820547	3.322954996	0.000475
ENSG00000254693.1	AC010768.1	1.079738437	10.00011372	1.00E-10
ENSG00000239911.2	PRKAG2-AS1	1.079371097	2.287317055	0.00516
ENSG00000224721.1	AC007182.1	1.079308364	7.995528023	1.01E-08
ENSG00000050405.13	LIMA1	1.078808316	8.491186139	3.23E-09
ENSG00000130635.15	COL5A1	1.078707742	6.282553417	5.22E-07
ENSG00000099864.17	PALM	1.078265558	5.071092157	8.49E-06
ENSG00000111716.12	LDHB	1.078004426	7.551136903	2.81E-08
ENSG00000135245.9	HILPDA	1.077952567	5.751066557	1.77E-06
ENSG00000163629.12	PTPN13	1.077505232	5.955140451	1.11E-06
ENSG00000134222.16	PSRC1	1.075927682	3.849841453	0.000141
ENSG00000167202.11	TBC1D2B	1.075407815	9.098066816	7.98E-10
ENSG00000174514.12	MFSD4A	1.075195133	5.078879267	8.34E-06
ENSG00000059573.8	ALDH18A1	1.074859866	18.67307683	2.12E-19
ENSG00000172716.16	SLFN11	1.073444704	12.22580391	5.95E-13
ENSG00000255863.2	AC073610.2	1.073196355	13.80912708	1.55E-14
ENSG00000109466.13	KLHL2	1.071578427	9.703733534	1.98E-10
ENSG00000285446.1	Z84488.2	1.071240796	7.877578332	1.33E-08
ENSG00000106348.17	IMPDH1	1.071125289	10.38954066	4.08E-11
ENSG00000285854.1	AC010197.2	1.07111672	7.484216386	3.28E-08
ENSG00000167306.19	MYO5B	1.070980199	3.610223884	0.000245
ENSG00000065308.4	TRAM2	1.070807533	9.223628487	5.98E-10
ENSG00000169299.13	PGM2	1.070735147	9.346237904	4.51E-10
ENSG00000155093.18	PTPRN2	1.070421487	9.529344081	2.96E-10
ENSG00000283881.1	MIR3916	1.070073657	3.171393075	0.000674
ENSG00000104774.12	MAN2B1	1.068835786	14.44621532	3.58E-15
ENSG00000151778.10	SERP2	1.068158413	5.777388242	1.67E-06
ENSG00000252561.1	RNU1-125P	1.068095571	3.558131493	0.000277
ENSG00000105750.14	ZNF85	1.06808755	4.007258053	9.83E-05
ENSG00000118898.15	PPL	1.068057964	3.589442399	0.000257
ENSG00000237149.5	ZNF503-AS2	1.067648492	5.803484204	1.57E-06
ENSG00000179362.14	HMGN2P46	1.06740887	1.716466297	0.01921
ENSG00000259134.6	LINC00924	1.067286709	2.66624847	0.002157
ENSG00000111674.8	ENO2	1.06722843	7.70548753	1.97E-08
ENSG00000285957.1	AC019257.7	1.066886129	5.485147088	3.27E-06
ENSG00000166546.13	BEAN1	1.066866973	2.490249058	0.003234
ENSG00000132561.13	MATN2	1.066820797	3.607071262	0.000247
ENSG00000214063.10	TSPAN4	1.066269317	9.869458878	1.35E-10
ENSG00000137474.20	MYO7A	1.06625006	7.467622084	3.41E-08
ENSG00000276116.2	FUT8-AS1	1.065911095	1.586432755	0.025916
ENSG00000140287.10	HDC	1.065072475	2.6680566	0.002148
ENSG00000206567.9	AC022007.1	1.063708159	3.107305886	0.000781
ENSG00000165929.12	TC2N	1.062970306	7.409254259	3.90E-08
ENSG00000256151.1	ADGRD1-AS1	1.062849005	2.172335854	0.006725
ENSG00000106069.22	CHN2	1.062842014	8.96785317	1.08E-09

ENSG00000167065.13	DUSP18	1.062173233	12.27845699	5.27E-13
ENSG00000179542.15	SLITRK4	1.061419604	4.555888613	2.78E-05
ENSG00000114670.13	NEK11	1.061354718	10.00510637	9.88E-11
ENSG00000118729.11	CASQ2	1.061216271	5.308905972	4.91E-06
ENSG00000198400.11	NTRK1	1.060944624	2.93376314	0.001165
ENSG00000155660.10	PDIA4	1.060781745	16.07425114	8.43E-17
ENSG00000173114.12	LRRN3	1.059264844	5.8865839	1.30E-06
ENSG00000244607.6	CCDC13	1.058235582	1.342276086	0.04547
ENSG00000130816.15	DNMT1	1.057776195	30.32873471	4.69E-31
ENSG00000176463.13	SLCO3A1	1.057735583	12.70311692	1.98E-13
ENSG00000231672.6	DIRC3	1.057046825	2.844628029	0.00143
ENSG00000168229.3	PTGDR	1.056870336	3.16021397	0.000691
ENSG00000229116.2	AL137026.1	1.056354514	1.760552246	0.017356
ENSG00000124243.17	BCAS4	1.056234034	5.68274976	2.08E-06
ENSG00000188321.13	ZNF559	1.056146025	8.996772887	1.01E-09
ENSG00000158106.13	RHPN1	1.055691816	5.838972966	1.45E-06
ENSG00000163472.18	TMEM79	1.055522085	13.21265422	6.13E-14
ENSG00000234500.1	AC008267.3	1.055243669	1.383022391	0.041398
ENSG00000248996.1	AC145098.1	1.054965573	1.3142708	0.048499
ENSG00000070404.9	FSTL3	1.054691769	5.802893414	1.57E-06
ENSG00000109436.7	TBC1D9	1.053918272	10.08121277	8.29E-11
ENSG00000148926.9	ADM	1.053778148	4.858772443	1.38E-05
ENSG00000155363.18	MOV10	1.053747241	15.42866576	3.73E-16
ENSG00000174171.5	AC020659.1	1.05318127	4.602379975	2.50E-05
ENSG00000126787.12	DLGAP5	1.053150785	1.305118714	0.049531
ENSG00000151012.13	SLC7A11	1.053013342	1.602496192	0.024975
ENSG00000261971.7	MMP25-AS1	1.052917086	5.875076159	1.33E-06
ENSG00000173890.16	GPR160	1.052890755	2.797704565	0.001593
ENSG00000144668.11	ITGA9	1.052195268	8.815424782	1.53E-09
ENSG00000091972.18	CD200	1.051871591	6.035234094	9.22E-07
ENSG00000213626.12	LBH	1.05118815	14.13367371	7.35E-15
ENSG00000198915.11	RASGEF1A	1.051134689	3.60398348	0.000249
ENSG00000064199.6	SPA17	1.050159594	4.843458259	1.43E-05
ENSG00000167461.11	RAB8A	1.049571507	11.56837251	2.70E-12
ENSG00000128271.21	ADORA2A	1.049496382	6.84386956	1.43E-07
ENSG00000174327.6	SLC16A13	1.049187924	1.320711577	0.047785
ENSG00000131669.9	NINJ1	1.049138651	15.12879285	7.43E-16
ENSG00000188643.10	S100A16	1.048816063	16.75423051	1.76E-17
ENSG00000177732.8	SOX12	1.048021697	11.58746494	2.59E-12
ENSG00000137193.13	PIM1	1.045891489	9.950075372	1.12E-10
ENSG00000204323.5	SMIM5	1.045740685	1.347622958	0.044914
ENSG00000142731.10	PLK4	1.04562453	1.353260282	0.044334
ENSG00000106723.16	SPIN1	1.045528424	12.32132491	4.77E-13
ENSG00000284292.1	AC004922.1	1.045347167	15.85483019	1.40E-16
ENSG00000167711.13	SERPINF2	1.04514693	3.893692046	0.000128
ENSG00000199080.1	MIR133B	1.044570298	2.217519423	0.00606
ENSG00000213983.11	AP1G2	1.044473731	10.86844369	1.35E-11

ENSG00000169230.9	PRELID1	1.044185962	15.62368758	2.38E-16
ENSG00000167460.15	TPM4	1.043492969	9.432003696	3.70E-10
ENSG00000162595.6	DIRAS3	1.043315915	2.624342777	0.002375
ENSG00000178695.5	KCTD12	1.043038043	10.7879938	1.63E-11
ENSG00000144935.14	TRPC1	1.042859196	6.715446231	1.93E-07
ENSG00000121152.9	NCAPH	1.042674776	1.761314273	0.017325
ENSG00000144554.10	FANCD2	1.042225775	6.541549425	2.87E-07
ENSG00000250709.1	CCDC169-SOHLH2	1.042118686	1.379159312	0.041768
ENSG00000183092.16	BEGAIN	1.042009274	5.734742102	1.84E-06
ENSG00000165617.14	DACT1	1.041908613	5.4741664	3.36E-06
ENSG00000155324.9	GRAMD2B	1.040017849	5.818679245	1.52E-06
ENSG00000188897.9	AC099489.1	1.039534054	3.510627451	0.000309
ENSG00000163513.17	TGFBR2	1.039027671	8.151776577	7.05E-09
ENSG00000157064.10	NMNAT2	1.039026954	5.529414284	2.96E-06
ENSG00000139318.7	DUSP6	1.038930929	6.575095056	2.66E-07
ENSG00000177182.10	CLVS1	1.03874899	5.216680912	6.07E-06
ENSG00000112773.15	TENT5A	1.037772414	6.779977106	1.66E-07
ENSG00000198795.10	ZNF521	1.037101118	5.766695791	1.71E-06
ENSG00000277978.1	AC010542.5	1.036737833	9.34498681	4.52E-10
ENSG00000225420.1	AC104134.1	1.036703145	7.071164379	8.49E-08
ENSG00000171291.8	ZNF439	1.036660033	2.705393085	0.001971
ENSG00000270629.5	NBPF14	1.036569877	11.36171132	4.35E-12
ENSG00000258017.1	AC011603.2	1.03637915	14.33104175	4.67E-15
ENSG00000224940.8	PRRT4	1.035037857	4.837143272	1.45E-05
ENSG00000134532.16	SOX5	1.034246068	1.942665228	0.011411
ENSG00000152518.7	ZFP36L2	1.034236651	18.54554465	2.85E-19
ENSG00000233622.3	CYP2T1P	1.03418453	3.058546457	0.000874
ENSG00000143797.11	MBOAT2	1.033890977	10.31342773	4.86E-11
ENSG00000067057.16	PFKP	1.033879156	12.4421395	3.61E-13
ENSG00000219607.3	PPP1R3G	1.033396329	5.731545233	1.86E-06
ENSG00000242732.4	RTL5	1.033012533	8.017848215	9.60E-09
ENSG00000064547.13	LPAR2	1.032804494	2.466820012	0.003413
ENSG00000130038.9	CRACR2A	1.032734256	6.671591773	2.13E-07
ENSG00000133639.4	BTG1	1.032218721	15.39774602	4.00E-16
ENSG00000065413.19	ANKRD44	1.031769852	8.169671135	6.77E-09
ENSG00000167984.17	NLRC3	1.031270268	7.100006304	7.94E-08
ENSG00000092820.17	EZR	1.030962283	14.18129771	6.59E-15
ENSG00000278129.1	ZNF8	1.030886424	13.91186134	1.23E-14
ENSG00000284482.1	MIR4680	1.030090723	8.519456774	3.02E-09
ENSG00000085117.11	CD82	1.029714868	10.62408274	2.38E-11
ENSG00000254894.1	NAV2-AS1	1.029611857	4.493027765	3.21E-05
ENSG00000163931.15	TKT	1.029543194	5.904086627	1.25E-06
ENSG00000140807.6	NKD1	1.029403206	2.848556709	0.001417
ENSG00000242797.3	GLYCTK-AS1	1.029058563	4.937892913	1.15E-05
ENSG00000163637.12	PRICKLE2	1.028777064	10.3206367	4.78E-11
ENSG00000140488.15	CELF6	1.027964225	4.850701855	1.41E-05
ENSG00000196950.13	SLC39A10	1.027259946	12.34795724	4.49E-13

ENSG00000235098.8	ANKRD65	1.027182672	9.693496645	2.03E-10
ENSG00000196616.13	ADH1B	1.027069491	4.33685229	4.60E-05
ENSG00000123416.15	TUBA1B	1.026831479	14.45393218	3.52E-15
ENSG00000081377.16	CDC14B	1.026722566	12.10636647	7.83E-13
ENSG00000198832.10	SELENOM	1.026704175	9.256950466	5.53E-10
ENSG00000144810.15	COL8A1	1.026609865	4.63881251	2.30E-05
ENSG00000135269.17	TES	1.025806777	10.98531177	1.03E-11
ENSG00000142920.16	AZIN2	1.025682145	6.747604751	1.79E-07
ENSG00000258727.1	AL135999.1	1.025046175	9.997628517	1.01E-10
ENSG00000101544.8	ADNP2	1.024953739	18.51677232	3.04E-19
ENSG00000111452.12	ADGRD1	1.024875067	4.27691556	5.29E-05
ENSG00000101052.12	IFT52	1.024155571	7.073847959	8.44E-08
ENSG00000130224.14	LRCH2	1.023926953	3.529382487	0.000296
ENSG00000069974.15	RAB27A	1.023578503	16.23530118	5.82E-17
ENSG00000072786.12	STK10	1.022876692	8.968550206	1.08E-09
ENSG00000148082.9	SHC3	1.022699383	4.161154968	6.90E-05
ENSG00000113231.13	PDE8B	1.022555164	4.201007489	6.29E-05
ENSG00000110218.8	PANX1	1.022381846	6.198670279	6.33E-07
ENSG00000139537.10	CCDC65	1.02228228	2.638618105	0.002298
ENSG00000072832.14	CRMP1	1.021252726	6.214432597	6.10E-07
ENSG00000213654.9	GPSM3	1.021217778	8.233591878	5.84E-09
ENSG00000092445.11	TYRO3	1.020936745	4.235119585	5.82E-05
ENSG00000131238.17	PPT1	1.020923536	14.82256118	1.50E-15
ENSG00000168282.5	MGAT2	1.020002824	19.53480987	2.92E-20
ENSG00000115363.13	EVA1A	1.019877373	1.73895462	0.018241
ENSG00000123989.13	CHPF	1.019639463	12.77855279	1.67E-13
ENSG00000088298.12	EDEM2	1.019630333	10.01847877	9.58E-11
ENSG00000135931.17	ARMC9	1.019559188	10.04956128	8.92E-11
ENSG00000002549.12	LAP3	1.019411143	13.31674111	4.82E-14
ENSG00000075914.12	EXOSC7	1.018926601	7.063471813	8.64E-08
ENSG00000135525.18	MAP7	1.018870743	2.324835149	0.004733
ENSG00000266261.1	AC005324.5	1.018537555	1.626093733	0.023654
ENSG00000123643.12	SLC36A1	1.017898772	12.07327578	8.45E-13
ENSG00000133026.12	MYH10	1.017426309	4.603377522	2.49E-05
ENSG00000135185.11	TMEM243	1.017119139	7.707476455	1.96E-08
ENSG00000039560.13	RAI14	1.016678779	7.360699528	4.36E-08
ENSG00000143320.8	CRABP2	1.01593703	6.859464762	1.38E-07
ENSG00000280334.1	AC009084.2	1.015815455	2.604788816	0.002484
ENSG00000003436.15	TFPI	1.015629742	11.96632256	1.08E-12
ENSG00000279803.1	AC009090.5	1.015273317	1.46475265	0.034296
ENSG00000107201.9	DDX58	1.015007721	13.90136918	1.25E-14
ENSG00000124126.13	PREX1	1.014570204	11.33283308	4.65E-12
ENSG00000167723.14	TRPV3	1.014393179	3.909048679	0.000123
ENSG00000258377.1	AL139099.1	1.013994342	19.15440416	7.01E-20
ENSG00000274417.1	MIR6515	1.013516988	3.271159019	0.000536
ENSG00000265784.1	AC006441.3	1.013445143	13.1648677	6.84E-14
ENSG00000104689.9	TNFRSF10A	1.013340561	9.828529625	1.48E-10

ENSG00000131236.16	CAP1	1.013326284	12.86337489	1.37E-13
ENSG00000172236.16	TPSAB1	1.013016154	3.590148707	0.000257
ENSG00000163563.7	MNDA	1.012415847	1.622046539	0.023876
ENSG00000214402.6	LCNL1	1.012390376	2.047087732	0.008972
ENSG00000164211.12	STARD4	1.011722334	8.319884333	4.79E-09
ENSG00000237172.3	B3GNT9	1.011607238	20.96805724	1.08E-21
ENSG00000282057.1	AC092807.3	1.010994193	1.875970804	0.013305
ENSG00000134013.15	LOXL2	1.01095159	7.820287149	1.51E-08
ENSG00000088756.12	ARHGAP28	1.01080403	7.652274165	2.23E-08
ENSG00000284378.1	MIR1244-1	1.010645681	10.18589457	6.52E-11
ENSG00000170017.12	ALCAM	1.010620499	7.00783579	9.82E-08
ENSG00000273259.3	AL049839.2	1.009838865	2.953661159	0.001113
ENSG00000172123.12	SLFN12	1.008838899	6.714688054	1.93E-07
ENSG00000184743.12	ATL3	1.008569216	7.956597715	1.11E-08
ENSG00000163923.9	RPL39L	1.00802799	2.617815844	0.002411
ENSG00000246763.6	RGMB-AS1	1.007998826	3.322950966	0.000475
ENSG00000152661.8	GJA1	1.00795338	5.917756553	1.21E-06
ENSG00000204282.4	TNRC6C-AS1	1.007647551	8.176908588	6.65E-09
ENSG00000166886.12	NAB2	1.007470629	14.76560504	1.72E-15
ENSG00000104549.11	SQLE	1.006923824	8.328953053	4.69E-09
ENSG00000162757.4	C1orf74	1.006500322	9.094960112	8.04E-10
ENSG00000267787.6	AC027097.2	1.006369377	8.327229199	4.71E-09
ENSG00000163359.15	COL6A3	1.005811834	5.805680508	1.56E-06
ENSG00000160219.11	GAB3	1.00477984	11.35052118	4.46E-12
ENSG00000134627.11	PIWIL4	1.004551787	7.190332138	6.45E-08
ENSG00000164111.14	ANXA5	1.00427795	10.60538044	2.48E-11
ENSG00000163132.6	MSX1	1.004059047	4.517637658	3.04E-05
ENSG00000267344.1	AC003070.1	1.003632462	4.227833691	5.92E-05
ENSG00000172667.10	ZMAT3	1.003518212	17.24489218	5.69E-18
ENSG00000279086.1	AC073130.3	1.003140752	5.559751426	2.76E-06
ENSG00000207652.1	MIR621	1.003006264	1.92943581	0.011764
ENSG00000272822.1	AC073610.3	1.002591717	13.51085142	3.08E-14
ENSG00000164114.18	MAP9	1.00213202	4.804383845	1.57E-05
ENSG00000197943.9	PLCG2	1.001641715	8.123101048	7.53E-09
ENSG00000133138.19	TBC1D8B	1.001628475	2.268998867	0.005383
ENSG00000279198.1	AC008894.3	1.001291025	7.716564604	1.92E-08
ENSG00000183250.11	LINC01547	0.999717268	4.236783659	5.80E-05
ENSG00000023902.13	PLEKHO1	0.999634316	11.65533887	2.21E-12
ENSG00000259753.1	AC068234.1	0.999302653	4.718212762	1.91E-05
ENSG00000142156.14	COL6A1	0.999248461	9.124848093	7.50E-10
ENSG00000248049.6	UBA6-AS1	0.998788903	8.73496681	1.84E-09
ENSG00000274021.1	AC024909.2	0.998581136	5.967311262	1.08E-06
ENSG00000101310.16	SEC23B	0.997972041	14.67273861	2.12E-15
ENSG00000132003.9	ZSWIM4	0.997853471	14.54171571	2.87E-15
ENSG00000269469.1	AC010619.1	0.997796342	15.24547189	5.68E-16
ENSG00000279117.1	AP001972.5	0.997077214	8.561333637	2.75E-09
ENSG00000214455.4	RCN1P2	0.996939647	8.524547734	2.99E-09

ENSG00000259409.1	BMF-AS1	0.996446291	3.339923851	0.000457
ENSG00000151746.13	BICD1	0.996082057	7.937466979	1.15E-08
ENSG00000117016.9	RIMS3	0.996028047	5.045804281	9.00E-06
ENSG00000169302.15	STK32A	0.995544488	1.619405026	0.024021
ENSG00000122952.16	ZWINT	0.994987246	4.38367199	4.13E-05
ENSG00000163577.7	EIF5A2	0.994677568	6.180932348	6.59E-07
ENSG00000273331.1	TM4SF19-TCTEX1D2	0.994326921	5.368539928	4.28E-06
ENSG00000264772.6	AC016876.2	0.99410569	12.16395515	6.86E-13
ENSG00000186417.13	GLDN	0.99410559	2.499618165	0.003165
ENSG00000135519.7	KCNH3	0.993848624	2.725348806	0.001882
ENSG00000126970.15	ZC4H2	0.993651439	10.00273646	9.94E-11
ENSG00000117834.12	SLC5A9	0.99320601	1.485794579	0.032674
ENSG00000133789.14	SWAP70	0.993180274	11.35972721	4.37E-12
ENSG00000188158.15	NHS	0.992462927	3.687340789	0.000205
ENSG00000259207.7	ITGB3	0.992086118	5.355708823	4.41E-06
ENSG00000171777.15	RASGRP4	0.992044516	5.841125431	1.44E-06
ENSG00000100316.15	RPL3	0.99192363	7.642005162	2.28E-08
ENSG00000163521.15	GLB1L	0.991913613	4.802899316	1.57E-05
ENSG00000214189.9	ZNF788P	0.990588744	4.442143205	3.61E-05
ENSG00000135069.13	PSAT1	0.990023955	4.05466067	8.82E-05
ENSG00000172932.14	ANKRD13D	0.989948559	10.61535443	2.42E-11
ENSG00000109107.13	ALDOC	0.989825604	4.389509322	4.08E-05
ENSG00000136859.9	ANGPTL2	0.989269767	11.3657782	4.31E-12
ENSG00000086730.16	LAT2	0.989216555	6.192894846	6.41E-07
ENSG00000110651.11	CD81	0.989164788	11.93693549	1.16E-12
ENSG00000102699.5	PARP4	0.987963942	21.3751284	4.22E-22
ENSG00000276250.1	AC127024.6	0.987918341	6.116283487	7.65E-07
ENSG00000183853.17	KIRREL1	0.987768763	6.228908086	5.90E-07
ENSG00000109586.11	GALNT7	0.986836552	12.13288281	7.36E-13
ENSG00000100219.16	XBP1	0.986456362	11.73509548	1.84E-12
ENSG00000259865.1	AL390728.6	0.985470119	7.10610018	7.83E-08
ENSG00000162944.10	RFTN2	0.985204093	5.331172063	4.66E-06
ENSG00000011426.10	ANLN	0.984710176	4.229237891	5.90E-05
ENSG00000110042.7	DTX4	0.984618914	12.76073501	1.73E-13
ENSG00000241313.2	WWTR1-AS1	0.984335087	13.00032931	9.99E-14
ENSG00000269242.1	AC010422.3	0.984066562	11.01041907	9.76E-12
ENSG00000170442.11	KRT86	0.983804326	2.254756993	0.005562
ENSG00000182220.14	ATP6AP2	0.98350799	12.23226896	5.86E-13
ENSG00000139146.13	SINHCAF	0.982457472	13.24789589	5.65E-14
ENSG00000215845.10	TSTD1	0.982176095	10.42251124	3.78E-11
ENSG00000146151.13	HMGCLL1	0.982106281	2.953669304	0.001113
ENSG00000225398.3	PGM5P4	0.980508561	2.321704936	0.004768
ENSG00000145623.12	OSMR	0.980248177	8.200351872	6.30E-09
ENSG00000166845.14	C18orf54	0.980119479	3.636948766	0.000231
ENSG00000197622.12	CDC42SE1	0.979473657	23.38512619	4.12E-24
ENSG00000213977.7	TAX1BP3	0.978512024	10.00094176	9.98E-11
ENSG00000196776.15	CD47	0.977976621	16.8274584	1.49E-17

ENSG00000175093.4	SPSB4	0.97788168	7.342265794	4.55E-08
ENSG00000200320.1	SNORA63	0.977220458	4.023674814	9.47E-05
ENSG00000269480.1	AC020913.2	0.976992509	7.190332138	6.45E-08
ENSG00000132170.20	PPARG	0.976413534	2.800376845	0.001584
ENSG00000214193.10	SH3D21	0.976175761	7.72246796	1.89E-08
ENSG00000136010.13	ALDH1L2	0.97598656	7.267356171	5.40E-08
ENSG00000126218.11	F10	0.975120957	3.848860628	0.000142
ENSG00000130489.14	SCO2	0.974357098	9.164604394	6.85E-10
ENSG00000133574.9	GIMAP4	0.974147909	8.048926013	8.93E-09
ENSG00000204131.9	NHSL2	0.974142824	6.559674394	2.76E-07
ENSG00000157870.15	PRXL2B	0.973770436	10.46829886	3.40E-11
ENSG00000243701.5	DUBR	0.973607365	6.246102821	5.67E-07
ENSG00000102924.11	CBLN1	0.972834814	2.010808123	0.009754
ENSG00000164305.18	CASP3	0.972460075	12.18172435	6.58E-13
ENSG00000162881.6	OXER1	0.972298601	1.786221749	0.01636
ENSG00000002834.17	LASP1	0.972254105	12.74814332	1.79E-13
ENSG00000145365.10	TIFA	0.972011172	8.807380118	1.56E-09
ENSG00000197256.10	KANK2	0.971825885	10.39706513	4.01E-11
ENSG00000169255.14	B3GALNT1	0.970680198	6.282609912	5.22E-07
ENSG00000217555.12	CKLF	0.970621122	10.24130428	5.74E-11
ENSG00000167617.2	CDC42EP5	0.970404363	5.142820979	7.20E-06
ENSG00000135736.5	CCDC102A	0.970013129	7.729630799	1.86E-08
ENSG00000254964.1	AP001458.1	0.969851208	4.136538317	7.30E-05
ENSG00000116793.15	PHTF1	0.969480859	16.05055805	8.90E-17
ENSG00000162882.14	HAAO	0.969445405	7.037936699	9.16E-08
ENSG00000072609.17	CHFR	0.969164053	24.74813758	1.79E-25
ENSG00000197927.12	C2orf27A	0.968547287	5.217348641	6.06E-06
ENSG00000180447.6	GAS1	0.968415125	4.536931114	2.90E-05
ENSG00000174307.6	PHLDA3	0.967878484	11.81358508	1.54E-12
ENSG00000225613.2	LINCMD1	0.967836189	2.404707352	0.003938
ENSG00000177426.20	TGIF1	0.967502559	12.07512707	8.41E-13
ENSG00000150995.19	ITPR1	0.966637089	13.23387046	5.84E-14
ENSG00000100292.16	HMOX1	0.966390626	5.390090878	4.07E-06
ENSG00000196371.3	FUT4	0.966193955	9.366418246	4.30E-10
ENSG00000256007.1	ARAP1-AS1	0.964889103	10.49521808	3.20E-11
ENSG00000136810.12	TXN	0.964641737	13.18894539	6.47E-14
ENSG00000049323.15	LTBP1	0.964525494	6.678700091	2.10E-07
ENSG00000106415.12	GLCCI1	0.96400326	10.01733517	9.61E-11
ENSG00000277288.4	C10orf142	0.963585695	1.74109715	0.018151
ENSG00000255498.1	AC068385.1	0.963297782	3.986535993	0.000103
ENSG00000117525.13	F3	0.963278118	6.853551714	1.40E-07
ENSG00000162433.14	AK4	0.963034533	5.169935147	6.76E-06
ENSG00000134470.20	IL15RA	0.962781468	13.92654278	1.18E-14
ENSG00000002587.9	HS3ST1	0.962389307	1.330446558	0.046725
ENSG00000234231.4	AC093616.1	0.962380846	1.773336792	0.016852
ENSG00000101003.9	GINS1	0.962093577	2.559754606	0.002756
ENSG00000008735.13	MAPK8IP2	0.961647237	2.101142817	0.007922

ENSG00000077782.20	FGFR1	0.961258437	8.149712653	7.08E-09
ENSG00000143924.18	EML4	0.961239739	13.89729156	1.27E-14
ENSG00000169902.14	TPST1	0.961167643	4.731401594	1.86E-05
ENSG00000166598.14	HSP90B1	0.961075553	11.31385129	4.85E-12
ENSG00000178752.15	ERFE	0.960988066	2.409164636	0.003898
ENSG00000072110.13	ACTN1	0.960512343	9.218898929	6.04E-10
ENSG00000258399.7	AL117190.1	0.960512308	5.629503872	2.35E-06
ENSG00000205403.13	CFI	0.960508693	6.647092109	2.25E-07
ENSG00000145284.11	SCD5	0.960422323	3.660883826	0.000218
ENSG00000106686.16	SPATA6L	0.96018781	3.73147577	0.000186
ENSG00000224063.5	AC007319.1	0.960099297	6.925401126	1.19E-07
ENSG00000170385.9	SLC30A1	0.959687863	9.089924009	8.13E-10
ENSG00000100196.10	KDELR3	0.959559443	6.084813118	8.23E-07
ENSG00000229036.7	VDAC1P8	0.959056088	9.43118572	3.71E-10
ENSG00000160211.17	G6PD	0.958949772	10.78461415	1.64E-11
ENSG00000170004.16	CHD3	0.958418792	7.693553956	2.03E-08
ENSG00000276231.4	PIK3R6	0.958175374	2.187645995	0.006492
ENSG00000175147.12	TMEM51-AS1	0.957939562	5.073476384	8.44E-06
ENSG00000272234.1	AC008945.1	0.957882792	6.379237468	4.18E-07
ENSG00000187607.15	ZNF286A	0.957868999	21.34467744	4.52E-22
ENSG00000127507.17	ADGRE2	0.957810282	1.482278026	0.03294
ENSG00000260027.4	HOXB7	0.957688815	3.449095997	0.000356
ENSG00000009694.13	TENM1	0.957524772	3.278816492	0.000526
ENSG00000147065.16	MSN	0.957396648	14.59031992	2.57E-15
ENSG00000109103.11	UNC119	0.957371109	14.59701443	2.53E-15
ENSG00000119686.9	FLVCR2	0.956992489	12.34188728	4.55E-13
ENSG00000151239.13	TWF1	0.95698581	8.269136462	5.38E-09
ENSG00000171522.5	PTGER4	0.956717692	8.589106071	2.58E-09
ENSG00000198001.13	IRAK4	0.956318548	11.31112132	4.89E-12
ENSG00000110092.3	CCND1	0.956225043	7.628884702	2.35E-08
ENSG00000233893.2	EZR-AS1	0.955979305	8.833764894	1.47E-09
ENSG00000125844.15	RRBP1	0.955814005	8.585067672	2.60E-09
ENSG00000103653.16	CSK	0.955323322	12.72785697	1.87E-13
ENSG00000130300.8	PLVAP	0.955149287	6.307253058	4.93E-07
ENSG00000198538.10	ZNF28	0.954988458	6.812415152	1.54E-07
ENSG00000158062.20	UBXN11	0.954420955	6.950804561	1.12E-07
ENSG00000278419.1	AL451164.3	0.954064295	7.879642979	1.32E-08
ENSG00000101856.9	PGRMC1	0.953207477	7.8304028	1.48E-08
ENSG00000236528.1	AL033528.2	0.952856475	6.759702048	1.74E-07
ENSG00000177628.15	GBA	0.952673117	10.34413537	4.53E-11
ENSG00000173267.13	SNCG	0.952525705	5.058463599	8.74E-06
ENSG00000140092.14	FBLN5	0.952462825	6.565610678	2.72E-07
ENSG00000015475.18	BID	0.952362192	21.68379676	2.07E-22
ENSG00000166839.16	ANKDD1A	0.952280625	5.21238254	6.13E-06
ENSG00000141582.14	CBX4	0.951068239	10.86855354	1.35E-11
ENSG00000183856.10	IQGAP3	0.950609141	1.84706753	0.014221
ENSG00000173548.8	SNX33	0.950427176	7.929927984	1.18E-08

ENSG00000254788.7	CKLF-CMTM1	0.949857365	10.54461727	2.85E-11
ENSG00000104368.17	PLAT	0.949853441	6.532395075	2.93E-07
ENSG00000104998.3	IL27RA	0.949034352	6.412221985	3.87E-07
ENSG00000172469.15	MANEA	0.948767217	5.603952449	2.49E-06
ENSG00000264343.5	NOTCH2NLA	0.948309539	7.97314458	1.06E-08
ENSG00000093217.10	XYLB	0.947891127	4.176045472	6.67E-05
ENSG00000166068.12	SPRED1	0.947843911	9.305636225	4.95E-10
ENSG00000172071.11	EIF2AK3	0.946696883	11.13956172	7.25E-12
ENSG00000116815.15	CD58	0.946359546	11.6681876	2.15E-12
ENSG00000101109.11	STK4	0.945883554	13.71606986	1.92E-14
ENSG00000253837.1	AC090197.1	0.945645919	4.863130438	1.37E-05
ENSG00000182809.10	CRIP2	0.945114645	9.449059486	3.56E-10
ENSG00000143554.13	SLC27A3	0.945008738	8.379870605	4.17E-09
ENSG00000118620.12	ZNF430	0.944932557	11.08591812	8.21E-12
ENSG00000249992.1	TMEM158	0.944595442	3.793657169	0.000161
ENSG00000259583.2	AC015712.2	0.944485227	6.101432052	7.92E-07
ENSG00000108932.11	SLC16A6	0.943571701	1.598240076	0.025221
ENSG00000223776.5	LGALS8-AS1	0.943389139	2.956912957	0.001104
ENSG00000111229.15	ARPC3	0.943384439	12.30770046	4.92E-13
ENSG00000138670.17	RASGEF1B	0.943223402	5.67002132	2.14E-06
ENSG00000158163.14	DZIP1L	0.943205777	3.370553491	0.000426
ENSG00000130518.16	IQCN	0.943069287	6.173758847	6.70E-07
ENSG00000168026.18	TTC21A	0.942798449	4.298805653	5.03E-05
ENSG00000183671.12	GPR1	0.942273708	2.292121562	0.005104
ENSG00000283196.2	AC006453.2	0.94208315	4.65189818	2.23E-05
ENSG00000151693.10	ASAP2	0.942067564	6.044784953	9.02E-07
ENSG00000261759.1	AC099518.5	0.941706342	5.981958596	1.04E-06
ENSG00000102547.18	CAB39L	0.941359033	4.595018861	2.54E-05
ENSG00000167766.18	ZNF83	0.940890028	6.860877659	1.38E-07
ENSG00000272639.1	AC015712.5	0.940831536	2.043803529	0.009041
ENSG00000266208.1	AC080112.1	0.940809881	8.8352433	1.46E-09
ENSG00000188760.10	TMEM198	0.94063384	10.68174643	2.08E-11
ENSG00000111775.2	COX6A1	0.940606221	8.942492309	1.14E-09
ENSG00000139193.3	CD27	0.940210185	10.65216316	2.23E-11
ENSG00000181019.12	NQO1	0.938368588	4.943241427	1.14E-05
ENSG00000181588.16	MEX3D	0.938286888	11.4077071	3.91E-12
ENSG00000168476.11	REEP4	0.937962625	10.69231744	2.03E-11
ENSG00000185187.12	SIGIRR	0.937183214	10.437599	3.65E-11
ENSG00000156711.16	MAPK13	0.937143124	9.358715281	4.38E-10
ENSG00000254129.1	AC108449.1	0.937129883	2.050243481	0.008908
ENSG00000072071.16	ADGRL1	0.936916345	9.07388181	8.44E-10
ENSG00000205221.12	VIT	0.936700949	4.752664433	1.77E-05
ENSG00000069849.10	ATP1B3	0.935756072	19.23184583	5.86E-20
ENSG00000241111.1	PRICKLE2-AS1	0.935533319	7.77335628	1.69E-08
ENSG00000118495.19	PLAGL1	0.935191563	8.371046803	4.26E-09
ENSG00000148429.14	USP6NL	0.934832902	8.831198474	1.48E-09
ENSG00000146858.7	ZC3HAV1L	0.933747704	3.005244993	0.000988

ENSG00000107249.22	GLIS3	0.933539952	2.896548145	0.001269
ENSG00000109927.10	TECTA	0.932792847	3.491028266	0.000323
ENSG00000267169.1	AC022098.1	0.932671182	7.210836137	6.15E-08
ENSG00000138623.10	SEMA7A	0.93257526	9.320799369	4.78E-10
ENSG00000182575.7	NXPH3	0.932574761	3.426151391	0.000375
ENSG00000188641.13	DPYD	0.932574127	8.86523398	1.36E-09
ENSG00000258666.1	AL157871.4	0.932507797	7.208123289	6.19E-08
ENSG00000173013.5	CCDC96	0.932376827	2.138083087	0.007276
ENSG00000099849.14	RASSF7	0.932328842	5.794094192	1.61E-06
ENSG00000283936.1	MIR3658	0.932054582	5.818389398	1.52E-06
ENSG00000250751.1	AC015795.1	0.931782672	7.095021939	8.03E-08
ENSG00000268173.3	AC007192.1	0.931758722	8.944288037	1.14E-09
ENSG00000106511.5	MEOX2	0.931515873	5.673244324	2.12E-06
ENSG00000134138.19	MEIS2	0.931495398	2.798077088	0.001592
ENSG00000118690.12	ARMC2	0.931145307	6.539340685	2.89E-07
ENSG00000129534.13	MIS18BP1	0.931106749	8.091807584	8.09E-09
ENSG00000144837.8	PLA1A	0.930965506	3.506023208	0.000312
ENSG00000140105.17	WARS	0.930672237	8.516102475	3.05E-09
ENSG00000142046.14	TMEM91	0.929469916	6.406309901	3.92E-07
ENSG00000135749.18	PCNX2	0.929393207	7.519436011	3.02E-08
ENSG00000172965.15	MIR4435-2HG	0.929029323	11.87124978	1.35E-12
ENSG00000122417.15	ODF2L	0.928703839	7.570451149	2.69E-08
ENSG00000145861.7	C1QTNF2	0.928671025	2.880171994	0.001318
ENSG00000247796.2	AC008966.1	0.928509016	4.369157468	4.27E-05
ENSG00000168268.10	NT5DC2	0.927552429	6.904160629	1.25E-07
ENSG00000004660.14	CAMKK1	0.927534667	5.477290839	3.33E-06
ENSG00000170775.2	GPR37	0.926373429	1.488406329	0.032478
ENSG00000203965.12	EFCAB7	0.926195641	3.998921113	0.0001
ENSG00000111664.10	GNB3	0.925909755	3.90984835	0.000123
ENSG00000182749.5	PAQR7	0.925570038	18.06456734	8.62E-19
ENSG00000116833.13	NR5A2	0.924939578	2.470665567	0.003383
ENSG00000119314.15	PTBP3	0.924920377	13.31242528	4.87E-14
ENSG00000102554.13	KLF5	0.924912665	5.240225724	5.75E-06
ENSG00000092964.17	DPYSL2	0.924427926	7.480248173	3.31E-08
ENSG00000113140.10	SPARC	0.924324356	6.080350515	8.31E-07
ENSG00000122966.15	CIT	0.924065498	5.242514999	5.72E-06
ENSG00000176046.8	NUPR1	0.923976182	5.971268145	1.07E-06
ENSG00000256661.1	A2ML1-AS1	0.923363794	3.674412956	0.000212
ENSG00000128268.11	MGAT3	0.923045543	5.50140435	3.15E-06
ENSG00000240972.1	MIF	0.922700461	17.33666159	4.61E-18
ENSG00000257913.2	DDN-AS1	0.922140735	2.966602726	0.00108
ENSG00000184254.16	ALDH1A3	0.921901031	5.622608701	2.38E-06
ENSG00000138031.14	ADCY3	0.921865369	10.77604548	1.67E-11
ENSG00000104419.14	NDRG1	0.921239472	7.12250928	7.54E-08
ENSG00000126603.8	GLIS2	0.920114773	6.51416059	3.06E-07
ENSG00000139269.2	INHBE	0.91936113	2.117084113	0.007637
ENSG00000072858.10	SIDT1	0.919168045	6.996289801	1.01E-07

ENSG00000163975.11	MELTF	0.919006234	5.537105943	2.90E-06
ENSG00000283842.1	MIR4751	0.918936195	10.80341707	1.57E-11
ENSG00000235863.3	B3GALT4	0.918534414	8.218482666	6.05E-09
ENSG00000166173.10	LARP6	0.917929112	12.71708968	1.92E-13
ENSG00000189337.16	KAZN	0.917911851	7.106384209	7.83E-08
ENSG00000218537.1	MIF-AS1	0.917738501	17.52226677	3.00E-18
ENSG00000066322.14	ELOVL1	0.917461349	15.41152744	3.88E-16
ENSG00000102010.14	BMX	0.916984483	2.139178522	0.007258
ENSG00000224420.3	ADM5	0.916692583	2.261649611	0.005475
ENSG00000087088.19	BAX	0.915954443	15.71905383	1.91E-16
ENSG00000011105.13	TSPAN9	0.915883451	10.92947667	1.18E-11
ENSG00000144824.20	PHLDB2	0.915810487	3.977216787	0.000105
ENSG00000083799.17	CYLD	0.915651861	9.798227992	1.59E-10
ENSG00000157379.13	DHRS1	0.915399421	10.5892105	2.58E-11
ENSG00000175489.9	LRRC25	0.915355423	4.473221746	3.36E-05
ENSG00000099331.13	MYO9B	0.91472035	12.46995911	3.39E-13
ENSG00000067167.7	TRAM1	0.914254651	10.33449634	4.63E-11
ENSG00000135549.14	PKIB	0.913871251	1.96871759	0.010747
ENSG00000178685.13	PARP10	0.913332467	19.35730803	4.39E-20
ENSG00000214338.10	SOGA3	0.912807913	2.140962216	0.007228
ENSG00000136147.17	PHF11	0.912600485	11.47678654	3.34E-12
ENSG00000196924.15	FLNA	0.912597062	9.542774738	2.87E-10
ENSG00000006468.13	ETV1	0.912583608	5.615686869	2.42E-06
ENSG00000099992.15	TBC1D10A	0.912419823	8.139120724	7.26E-09
ENSG00000197670.6	AL157838.1	0.912230904	3.270185856	0.000537
ENSG00000125753.13	VASP	0.912186023	9.112266423	7.72E-10
ENSG00000131711.14	MAP1B	0.911822624	4.664205316	2.17E-05
ENSG00000241014.2	AC114490.1	0.91135351	4.773964355	1.68E-05
ENSG00000099139.13	PCSK5	0.91087894	4.573623548	2.67E-05
ENSG00000243646.9	IL10RB	0.9108691	11.73482327	1.84E-12
ENSG00000167595.15	PROSER3	0.910582299	10.78723964	1.63E-11
ENSG00000270055.1	AC127502.2	0.908985717	3.650144814	0.000224
ENSG00000114923.16	SLC4A3	0.908772039	5.152197677	7.04E-06
ENSG00000172819.16	RARG	0.907529768	5.11105236	7.74E-06
ENSG00000261602.1	AC092115.2	0.907278944	3.93930201	0.000115
ENSG00000189334.8	S100A14	0.907211448	1.649024808	0.022438
ENSG00000065485.19	PDIA5	0.907049445	7.83204802	1.47E-08
ENSG00000242479.1	AC109992.1	0.906526834	3.584180243	0.000261
ENSG00000117013.15	KCNQ4	0.906064135	4.342961213	4.54E-05
ENSG00000120833.13	SOCS2	0.905666078	4.372787873	4.24E-05
ENSG00000249035.6	CLMAT3	0.905494736	4.892890442	1.28E-05
ENSG00000196262.13	PPIA	0.905450154	11.92107205	1.20E-12
ENSG00000181007.8	ZFP82	0.905368869	8.627579347	2.36E-09
ENSG00000135587.8	SMPD2	0.90518817	6.502943229	3.14E-07
ENSG00000166340.16	TPP1	0.904806091	19.17598491	6.67E-20
ENSG00000255201.1	AC087623.1	0.904117288	5.63397041	2.32E-06
ENSG00000259939.1	AC022167.1	0.903481406	7.15510515	7.00E-08

ENSG00000137404.14	NRM	0.90286067	10.04170507	9.08E-11
ENSG00000156218.12	ADAMTSL3	0.902757452	5.004158805	9.90E-06
ENSG00000050767.16	COL23A1	0.902534102	1.703231701	0.019805
ENSG00000054793.13	ATP9A	0.902414834	9.899219906	1.26E-10
ENSG00000231770.5	TMEM44-AS1	0.902391849	7.935917047	1.16E-08
ENSG00000184304.14	PRKD1	0.902366456	5.342288998	4.55E-06
ENSG00000144802.11	NFKBIZ	0.902318153	6.608866204	2.46E-07
ENSG00000143333.6	RGS16	0.902184482	4.154335429	7.01E-05
ENSG00000255114.1	AP003392.3	0.902018036	11.64992582	2.24E-12
ENSG00000145632.14	PLK2	0.900823628	9.698144859	2.00E-10
ENSG00000042088.13	TDP1	0.900524919	9.376424362	4.20E-10
ENSG00000204272.12	NBDY	0.90020038	13.3635715	4.33E-14
ENSG00000149489.8	ROM1	0.899919696	8.252230505	5.59E-09
ENSG00000213240.8	AC239799.1	0.899781913	7.03975055	9.13E-08
ENSG00000137575.11	SDCBP	0.899754449	11.50530016	3.12E-12
ENSG00000134874.17	DZIP1	0.899735668	5.645189926	2.26E-06
ENSG00000248871.1	TNFSF12-TNFSF13	0.899580993	10.75940351	1.74E-11
ENSG00000229689.3	AC009237.3	0.898225676	3.510925803	0.000308
ENSG00000280798.1	LINC00294	0.897977254	9.456356006	3.50E-10
ENSG00000172687.13	ZNF738	0.89777315	8.810823443	1.55E-09
ENSG00000146776.14	ATXN7L1	0.897535023	13.67609664	2.11E-14
ENSG00000132881.11	CPLANE2	0.897415533	2.507333382	0.003109
ENSG00000160606.10	TLCD1	0.897094867	1.532428743	0.029348
ENSG00000166681.13	BEX3	0.897020333	7.506824391	3.11E-08
ENSG00000248124.7	RRN3P1	0.896586122	9.254674045	5.56E-10
ENSG00000139174.11	PRICKLE1	0.896529833	8.273657951	5.33E-09
ENSG00000149257.14	SERPINH1	0.896524515	7.080652502	8.31E-08
ENSG00000106404.13	CLDN15	0.896363711	5.630229918	2.34E-06
ENSG00000070669.16	ASNS	0.896318486	9.159812354	6.92E-10
ENSG00000261061.1	AC092718.4	0.896180519	2.025180819	0.009437
ENSG00000120370.12	GORAB	0.895564182	8.964158877	1.09E-09
ENSG00000256029.6	SNHG28	0.895483954	6.082842062	8.26E-07
ENSG00000169991.10	IFFO2	0.895303295	11.9608928	1.09E-12
ENSG00000182195.7	LDOC1	0.895104913	7.819965434	1.51E-08
ENSG00000267040.6	AC027097.1	0.894998564	6.64433917	2.27E-07
ENSG00000239704.10	CDRT4	0.89413169	8.362522337	4.34E-09
ENSG00000259607.1	AC108449.3	0.89396863	12.34585585	4.51E-13
ENSG00000115594.11	IL1R1	0.893837912	8.45368229	3.52E-09
ENSG00000164733.20	CTSB	0.893786004	13.27611825	5.30E-14
ENSG00000073350.13	LLGL2	0.893650322	8.202216832	6.28E-09
ENSG00000240184.6	PCDHGC3	0.892943387	7.816226659	1.53E-08
ENSG00000163545.8	NUAK2	0.892790977	2.328719367	0.004691
ENSG00000136169.16	SETDB2	0.892462741	13.05313575	8.85E-14
ENSG00000123810.7	B9D2	0.892452811	4.045381345	9.01E-05
ENSG00000125898.12	FAM110A	0.892397304	4.33025825	4.67E-05
ENSG00000131471.6	AOC3	0.891581832	2.503645529	0.003136
ENSG00000134901.12	KDELC1	0.891565136	3.977638156	0.000105

ENSG00000138160.5	KIF11	0.891543964	4.071818782	8.48E-05
ENSG00000182796.14	TMEM198B	0.89138646	7.391497884	4.06E-08
ENSG00000076928.17	ARHGEF1	0.891334801	9.61353506	2.43E-10
ENSG00000158859.9	ADAMTS4	0.89088714	2.23380345	0.005837
ENSG00000122786.19	CALD1	0.89026666	7.563736048	2.73E-08
ENSG00000149591.16	TAGLN	0.890231517	4.01719686	9.61E-05
ENSG00000142871.16	CYR61	0.889894671	3.172621853	0.000672
ENSG00000113273.16	ARSB	0.888544605	8.3687676	4.28E-09
ENSG00000159314.11	ARHGAP27	0.888168323	8.764385925	1.72E-09
ENSG00000198856.12	OSTC	0.887329923	9.740191558	1.82E-10
ENSG00000197965.11	MPZL1	0.88732186	7.776709709	1.67E-08
ENSG00000170522.9	ELOVL6	0.887224739	4.308596435	4.91E-05
ENSG00000138193.15	PLCE1	0.886737932	4.494647218	3.20E-05
ENSG00000268056.5	AC020913.1	0.886551589	6.794555474	1.60E-07
ENSG00000135916.15	ITM2C	0.886361653	7.128744867	7.43E-08
ENSG00000169282.17	KCNAB1	0.88625129	3.496705005	0.000319
ENSG00000163239.12	TDRD10	0.886207176	6.046960435	8.98E-07
ENSG00000141959.16	PFKL	0.885967877	14.31456832	4.85E-15
ENSG00000189067.12	LITAF	0.885677308	8.360789213	4.36E-09
ENSG00000175938.6	ORAI3	0.885654576	9.266301588	5.42E-10
ENSG00000099998.17	GGT5	0.885606441	7.278674052	5.26E-08
ENSG00000078900.14	TP73	0.885194286	3.560207977	0.000275
ENSG00000148426.12	PROSER2	0.885115282	3.987107633	0.000103
ENSG00000263155.5	MYZAP	0.884657075	4.148099376	7.11E-05
ENSG00000162542.13	TMCO4	0.884367827	10.83299414	1.47E-11
ENSG00000088986.10	DYNLL1	0.884040952	12.07360621	8.44E-13
ENSG00000150593.17	PDCD4	0.883969807	11.46870586	3.40E-12
ENSG00000271787.1	AC104794.4	0.883934578	2.322263458	0.004761
ENSG00000268603.1	AC053503.5	0.883855218	2.867759693	0.001356
ENSG00000119986.6	AVPI1	0.883289323	6.442533456	3.61E-07
ENSG00000099338.22	CATSPERG	0.883060647	2.92222329	0.001196
ENSG00000279519.1	AC007382.1	0.882801894	3.201460243	0.000629
ENSG00000236104.2	ZBTB22	0.88269099	18.21204798	6.14E-19
ENSG00000126391.13	FRMD8	0.882686043	14.92258619	1.20E-15
ENSG00000111252.10	SH2B3	0.881371722	13.33439109	4.63E-14
ENSG00000143179.15	UCK2	0.88131626	11.10179742	7.91E-12
ENSG00000133313.14	CNDP2	0.880902157	16.96993474	1.07E-17
ENSG00000073849.14	ST6GAL1	0.880744692	10.57917698	2.64E-11
ENSG00000238184.1	CD81-AS1	0.880189109	9.753051914	1.77E-10
ENSG00000076770.14	MBNL3	0.879606007	4.4521394	3.53E-05
ENSG00000141696.12	P3H4	0.879561795	8.994565701	1.01E-09
ENSG00000166130.14	IKBIP	0.879462292	6.448883716	3.56E-07
ENSG00000247746.4	USP51	0.879339327	3.960081207	0.00011
ENSG00000136141.14	LRCH1	0.879258199	8.707068301	1.96E-09
ENSG00000101017.13	CD40	0.879193947	9.329236303	4.69E-10
ENSG00000204623.9	ZNRD1ASP	0.878801148	5.087567674	8.17E-06
ENSG00000144455.13	SUMF1	0.878527553	6.906643055	1.24E-07

ENSG00000203865.9	ATP1A1-AS1	0.87849046	12.88719599	1.30E-13
ENSG00000153048.10	CARHSP1	0.878275026	8.542612416	2.87E-09
ENSG00000265666.1	RARA-AS1	0.878096094	6.847168659	1.42E-07
ENSG00000229969.1	AL136982.2	0.877919282	1.574183077	0.026657
ENSG00000166803.12	PCLAF	0.877352691	1.696437737	0.020117
ENSG00000198890.8	PRMT6	0.877346862	11.51327752	3.07E-12
ENSG00000109220.10	CHIC2	0.877240895	11.8884564	1.29E-12
ENSG00000163683.11	SMIM14	0.876923208	6.267681721	5.40E-07
ENSG00000100300.17	TSPO	0.876892792	10.07623457	8.39E-11
ENSG00000221818.8	EBF2	0.876833891	5.990543094	1.02E-06
ENSG00000101974.14	ATP11C	0.876368528	6.914681458	1.22E-07
ENSG00000116191.17	RALGPS2	0.876126578	6.109266657	7.78E-07
ENSG00000197566.9	ZNF624	0.875660883	4.102703972	7.89E-05
ENSG00000164953.15	TMEM67	0.875593544	3.85413064	0.00014
ENSG00000227719.1	AC006042.1	0.875418528	2.069706231	0.008517
ENSG00000125775.14	SDCBP2	0.87530109	3.209184964	0.000618
ENSG00000117280.12	RAB29	0.874743804	13.65620456	2.21E-14
ENSG00000145882.10	PCYOX1L	0.874478643	8.756671294	1.75E-09
ENSG00000113368.11	LMNB1	0.874298005	5.74183266	1.81E-06
ENSG00000162772.16	ATF3	0.87423834	2.261741462	0.005473
ENSG00000127863.15	TNFRSF19	0.874132867	4.942595332	1.14E-05
ENSG00000187815.9	ZFP69	0.87407566	6.556887091	2.77E-07
ENSG00000119865.8	CNRIP1	0.873349266	6.237500371	5.79E-07
ENSG00000105329.9	TGFB1	0.873169334	15.77848359	1.67E-16
ENSG00000170581.13	STAT2	0.873055441	11.83612925	1.46E-12
ENSG00000112699.10	GMDS	0.873052458	15.67938321	2.09E-16
ENSG00000152377.13	SPOCK1	0.873039893	3.347906945	0.000449
ENSG00000173852.14	DPY19L1	0.87248182	9.695913527	2.01E-10
ENSG00000131370.15	SH3BP5	0.872213728	9.456356006	3.50E-10
ENSG00000152582.13	SPEF2	0.872122818	2.745952744	0.001795
ENSG00000100003.17	SEC14L2	0.871742945	5.187543656	6.49E-06
ENSG00000162267.12	ITIH3	0.871457208	3.070179652	0.000851
ENSG00000245148.2	ARAP1-AS2	0.871296067	13.51556999	3.05E-14
ENSG00000111424.10	VDR	0.871219795	6.521793311	3.01E-07
ENSG00000121297.6	TSHZ3	0.871139686	9.213210411	6.12E-10
ENSG00000188511.12	C22orf34	0.871121634	3.196718477	0.000636
ENSG00000119397.16	CNTRL	0.870935173	6.588258526	2.58E-07
ENSG00000255104.8	AC005324.4	0.870867871	9.031269376	9.31E-10
ENSG00000213297.8	ZNF625-ZNF20	0.870673914	8.361737066	4.35E-09
ENSG00000249042.5	AC008771.1	0.870191038	1.890973169	0.012854
ENSG00000149451.17	ADAM33	0.86968818	2.821202299	0.001509
ENSG00000005022.5	SLC25A5	0.869566728	17.4647999	3.43E-18
ENSG00000179051.13	RCC2	0.869530906	13.13724291	7.29E-14
ENSG00000166145.14	SPINT1	0.869118859	1.645128062	0.02264
ENSG00000166833.20	NAV2	0.869115273	6.732008816	1.85E-07
ENSG00000104814.12	MAP4K1	0.869088092	5.698658485	2.00E-06
ENSG00000232973.12	CYP1B1-AS1	0.868959013	3.552787557	0.00028

ENSG00000215788.10	TNFRSF25	0.868489701	6.390513483	4.07E-07
ENSG00000070756.15	PABPC1	0.868161478	10.27023786	5.37E-11
ENSG00000171314.8	PGAM1	0.868147009	18.40119181	3.97E-19
ENSG00000134198.9	TSPAN2	0.867739603	7.36433978	4.32E-08
ENSG00000142279.12	WTIP	0.867391639	6.586518952	2.59E-07
ENSG00000135205.14	CCDC146	0.867070308	7.328927333	4.69E-08
ENSG00000227671.4	AL390728.4	0.866969629	7.636716599	2.31E-08
ENSG00000182158.14	CREB3L2	0.866812479	11.17452627	6.69E-12
ENSG00000257225.1	AC079601.1	0.866802981	7.934654688	1.16E-08
ENSG00000117448.13	AKR1A1	0.866535272	13.14403009	7.18E-14
ENSG00000173621.8	LRFN4	0.865947107	8.680710409	2.09E-09
ENSG00000274565.1	AC080038.1	0.865894419	3.175927977	0.000667
ENSG00000280088.1	AC126474.2	0.865857629	6.94825177	1.13E-07
ENSG00000179978.11	AC140134.1	0.865820405	1.85798874	0.013868
ENSG00000186635.14	ARAP1	0.865800383	16.03443414	9.24E-17
ENSG00000106803.9	SEC61B	0.865784867	6.796938296	1.60E-07
ENSG00000182511.11	FES	0.865624802	8.101729852	7.91E-09
ENSG00000177054.13	ZDHHC13	0.865467201	10.35461346	4.42E-11
ENSG00000135828.11	RNASEL	0.865094496	13.5621446	2.74E-14
ENSG00000172780.16	RAB43	0.864866757	9.01453272	9.67E-10
ENSG00000105810.9	CDK6	0.864359836	6.425099399	3.76E-07
ENSG00000131016.16	AKAP12	0.864352183	4.159361977	6.93E-05
ENSG00000120889.12	TNFRSF10B	0.864257151	13.12469888	7.50E-14
ENSG00000260822.1	AC004656.1	0.863128849	5.884061561	1.31E-06
ENSG00000145431.10	PDGFC	0.863074988	9.991601615	1.02E-10
ENSG00000272638.1	AC006027.1	0.862888825	8.149513743	7.09E-09
ENSG00000128791.11	TWSG1	0.862413862	7.021894968	9.51E-08
ENSG00000277967.1	RF02217	0.86238247	5.924069663	1.19E-06
ENSG00000278206.1	AL031320.2	0.862121343	9.325538735	4.73E-10
ENSG00000154114.12	TBCEL	0.861703714	15.03767796	9.17E-16
ENSG00000179859.9	RNF227	0.861339754	3.876555035	0.000133
ENSG00000153214.10	TMEM87B	0.861058396	8.760638822	1.74E-09
ENSG00000092051.16	JPH4	0.8609495	1.539416863	0.028879
ENSG00000103056.11	SMPD3	0.860836135	5.411998617	3.87E-06
ENSG00000258425.1	AC013451.1	0.860806327	1.630030788	0.023441
ENSG00000196329.11	GIMAP5	0.860320447	9.868422569	1.35E-10
ENSG00000196511.14	TPK1	0.86024642	7.997066665	1.01E-08
ENSG00000136811.16	ODF2	0.860151106	12.80458607	1.57E-13
ENSG00000165806.19	CASP7	0.859892753	17.15046998	7.07E-18
ENSG00000107742.12	SPOCK2	0.859857861	6.069974089	8.51E-07
ENSG00000198142.4	SOWAHC	0.859566281	8.368217332	4.28E-09
ENSG00000234409.6	CCDC188	0.85895858	1.871720885	0.013436
ENSG00000113391.18	FAM172A	0.858744994	10.70692805	1.96E-11
ENSG00000148655.14	LRMDA	0.858537519	2.330815112	0.004669
ENSG00000196517.11	SLC6A9	0.857784881	4.115559114	7.66E-05
ENSG00000271913.6	AL035530.2	0.857573056	5.473652734	3.36E-06
ENSG00000108511.9	HOXB6	0.85726319	3.780585072	0.000166

ENSG00000005108.15	THSD7A	0.85672757	4.079036343	8.34E-05
ENSG00000236364.3	AL358115.1	0.856220506	8.954213298	1.11E-09
ENSG00000102053.12	ZC3H12B	0.856099838	1.591800253	0.025598
ENSG00000075213.10	SEMA3A	0.855910029	2.612371337	0.002441
ENSG00000159433.11	STARD9	0.85585386	5.827535171	1.49E-06
ENSG00000033100.16	CHPF2	0.854880186	14.75963137	1.74E-15
ENSG00000019549.11	SNAI2	0.854856416	4.960454468	1.10E-05
ENSG00000159212.12	CLIC6	0.854640645	1.617134129	0.024147
ENSG00000114423.20	CBLB	0.854473844	11.99389527	1.01E-12
ENSG00000167578.17	RAB4B	0.854271618	13.39128933	4.06E-14
ENSG00000196189.12	SEMA4A	0.854137774	5.586300516	2.59E-06
ENSG00000205089.7	CCNI2	0.854011529	6.100002116	7.94E-07
ENSG00000123360.11	PDE1B	0.85367046	4.116052618	7.66E-05
ENSG00000225377.5	NRSN2-AS1	0.85353047	3.823223408	0.00015
ENSG00000149639.14	SOGA1	0.853450669	11.53597283	2.91E-12
ENSG00000106868.16	SUSD1	0.853386781	5.596591081	2.53E-06
ENSG00000154642.10	C21orf91	0.853384576	11.83612925	1.46E-12
ENSG00000230928.1	AL139241.1	0.853097329	2.443302957	0.003603
ENSG00000163704.11	PRRT3	0.853071867	9.095970619	8.02E-10
ENSG00000099251.14	HSD17B7P2	0.853041501	1.507818953	0.031059
ENSG00000170779.10	CDCA4	0.852867707	12.85511124	1.40E-13
ENSG00000284010.1	MIR675	0.852783866	3.746033498	0.000179
ENSG00000142657.20	PGD	0.852780587	15.02752691	9.39E-16
ENSG00000158825.5	CDA	0.852468107	1.87203847	0.013426
ENSG00000168993.14	CPLX1	0.852309952	4.114797265	7.68E-05
ENSG00000104361.9	NIPAL2	0.851983108	5.711288336	1.94E-06
ENSG00000025434.18	NR1H3	0.851452095	8.196803585	6.36E-09
ENSG00000177614.10	PGBD5	0.850729951	4.664434492	2.17E-05
ENSG00000152926.14	ZNF117	0.850569246	4.776947632	1.67E-05
ENSG00000264672.5	SEPT4-AS1	0.850375393	6.698610232	2.00E-07
ENSG00000269113.3	TRABD2B	0.850293125	2.931025527	0.001172
ENSG00000152952.11	PLOD2	0.849793553	5.229478407	5.90E-06
ENSG00000122986.13	HVCN1	0.848258633	8.468891711	3.40E-09
ENSG00000092096.16	SLC22A17	0.847556383	5.367847033	4.29E-06
ENSG00000249624.9	AP000295.1	0.847505232	13.81291314	1.54E-14
ENSG00000090539.15	CHRD	0.847305801	6.034494201	9.24E-07
ENSG00000046653.14	GPM6B	0.847103521	5.312401593	4.87E-06
ENSG00000271383.6	NBPF19	0.846951683	9.483527255	3.28E-10
ENSG00000131941.7	RHPN2	0.846091993	1.550917705	0.028124
ENSG00000237310.1	GS1-124K5.4	0.845830636	1.674121095	0.021178
ENSG00000169239.12	CA5B	0.845379271	5.636827457	2.31E-06
ENSG00000185650.9	ZFP36L1	0.845377681	12.44497971	3.59E-13
ENSG00000164574.15	GALNT10	0.845373038	7.349341498	4.47E-08
ENSG00000250510.7	GPR162	0.844353398	4.978415003	1.05E-05
ENSG00000122180.4	MYOG	0.844217026	3.141778145	0.000721
ENSG00000251357.4	AP000350.4	0.843526042	15.48642527	3.26E-16
ENSG00000116584.17	ARHGEF2	0.842710664	14.23418413	5.83E-15

ENSG00000198618.5	PPIAP22	0.842398938	8.821696413	1.51E-09
ENSG00000169871.12	TRIM56	0.842378374	12.41675263	3.83E-13
ENSG00000147883.10	CDKN2B	0.842241974	3.78854185	0.000163
ENSG00000187134.13	AKR1C1	0.841802769	4.36807899	4.28E-05
ENSG00000140464.19	PML	0.841216555	14.31262494	4.87E-15
ENSG00000242265.5	PEG10	0.84117888	4.055674553	8.80E-05
ENSG00000279528.1	AC115618.3	0.841087606	3.298503435	0.000503
ENSG00000175274.18	TP53I11	0.84086492	9.752040149	1.77E-10
ENSG00000165752.16	STK32C	0.840822752	19.8096635	1.55E-20
ENSG00000146674.14	IGFBP3	0.840618009	2.933890512	0.001164
ENSG00000129219.13	PLD2	0.839668867	8.998418819	1.00E-09
ENSG00000116017.10	ARID3A	0.839030639	9.297636343	5.04E-10
ENSG00000160326.13	SLC2A6	0.838644349	3.900608178	0.000126
ENSG00000147119.3	CHST7	0.838365681	7.025469161	9.43E-08
ENSG00000130150.11	MOSPD2	0.837526567	8.159534323	6.93E-09
ENSG00000102218.5	RP2	0.837371164	10.63480726	2.32E-11
ENSG00000171533.11	MAP6	0.836613677	2.67656565	0.002106
ENSG00000258086.1	AC079313.1	0.836461111	5.897715407	1.27E-06
ENSG00000095380.10	NANS	0.836385483	10.20739237	6.20E-11
ENSG00000107485.16	GATA3	0.835297707	3.433971085	0.000368
ENSG00000144476.5	ACKR3	0.835279296	3.000231261	0.000999
ENSG00000005238.19	FAM214B	0.835213596	18.17154089	6.74E-19
ENSG00000142867.13	BCL10	0.835193282	8.823660912	1.50E-09
ENSG00000179241.12	LDLRAD3	0.83442853	7.937809061	1.15E-08
ENSG00000102024.17	PLS3	0.834060978	3.166963917	0.000681
ENSG00000137501.17	SYTL2	0.833677986	6.055024894	8.81E-07
ENSG00000130309.10	COLGALT1	0.833594096	12.06943554	8.52E-13
ENSG00000131697.17	NPHP4	0.83355742	9.898223689	1.26E-10
ENSG00000158985.13	CDC42SE2	0.833437786	8.970516606	1.07E-09
ENSG00000168487.18	BMP1	0.833402358	6.218850774	6.04E-07
ENSG00000145794.16	MEGF10	0.832878413	3.418172916	0.000382
ENSG00000147100.10	SLC16A2	0.832746457	7.892885424	1.28E-08
ENSG00000151702.16	FLI1	0.832440996	8.746691666	1.79E-09
ENSG00000115252.18	PDE1A	0.832125899	4.083139135	8.26E-05
ENSG00000267458.1	AC092069.1	0.831505853	9.467646757	3.41E-10
ENSG00000173207.12	CKS1B	0.831337372	8.691453682	2.03E-09
ENSG00000162946.22	DISC1	0.831097833	7.370216252	4.26E-08
ENSG00000138131.3	LOXL4	0.830815921	2.842754927	0.001436
ENSG00000160310.17	PRMT2	0.830765087	16.99684322	1.01E-17
ENSG00000076344.15	RGS11	0.83065304	4.212269337	6.13E-05
ENSG00000225951.1	ODF2-AS1	0.830624118	7.355720504	4.41E-08
ENSG00000134490.13	TMEM241	0.830622891	5.233064603	5.85E-06
ENSG00000094804.10	CDC6	0.829951578	2.597076603	0.002529
ENSG00000104888.9	SLC17A7	0.829050234	5.666524087	2.16E-06
ENSG00000033327.12	GAB2	0.828234931	6.607845183	2.47E-07
ENSG00000147144.12	CCDC120	0.828134075	8.197300012	6.35E-09
ENSG00000163520.13	FBLN2	0.828047025	3.734519771	0.000184

ENSG00000173950.15	XXYLT1	0.827997389	7.051275627	8.89E-08
ENSG00000198131.13	ZNF544	0.827806334	10.95302124	1.11E-11
ENSG00000137486.16	ARRB1	0.826859479	8.740236708	1.82E-09
ENSG00000127415.12	IDUA	0.826855722	8.561037556	2.75E-09
ENSG00000107738.19	VSIR	0.826839573	7.162503999	6.88E-08
ENSG00000114473.13	IQCG	0.826755228	8.586400901	2.59E-09
ENSG00000245904.3	AC025164.1	0.826495143	6.115581899	7.66E-07
ENSG00000283149.1	AC068631.2	0.82636199	13.14994865	7.08E-14
ENSG00000105173.13	CCNE1	0.826004287	1.97969223	0.010479
ENSG00000122778.9	KIAA1549	0.825664471	5.361803561	4.35E-06
ENSG00000276101.1	AC027601.4	0.825080291	7.108830934	7.78E-08
ENSG00000178700.7	DHFR2	0.82475155	7.012065063	9.73E-08
ENSG00000115271.10	GCA	0.824527437	5.036678279	9.19E-06
ENSG00000268362.5	AC092279.1	0.824478171	3.669921065	0.000214
ENSG00000149499.11	EML3	0.823074389	7.758157785	1.75E-08
ENSG00000078124.11	ACER3	0.822940599	10.44094742	3.62E-11
ENSG00000272870.1	AC097534.2	0.8228666	1.828617206	0.014838
ENSG00000175567.8	UCP2	0.82277192	8.630882614	2.34E-09
ENSG00000180964.16	TCEAL8	0.8224299	7.868490363	1.35E-08
ENSG00000268975.2	MIA-RAB4B	0.82175679	12.23490646	5.82E-13
ENSG00000160233.7	LRRC3	0.8216603	3.504810508	0.000313
ENSG00000116652.6	DLEU2L	0.821640415	1.391084295	0.040636
ENSG00000271936.1	AC012073.1	0.821573417	4.965644979	1.08E-05
ENSG00000253159.2	PCDHGA12	0.821132364	6.265199557	5.43E-07
ENSG00000163399.15	ATP1A1	0.82087695	11.68238203	2.08E-12
ENSG00000151773.12	CCDC122	0.820223443	2.379033134	0.004178
ENSG00000160710.16	ADAR	0.819975853	11.84266922	1.44E-12
ENSG00000261934.2	PCDHGA9	0.819823759	6.366565862	4.30E-07
ENSG00000254221.2	PCDHGB1	0.819785663	6.301915374	4.99E-07
ENSG00000266524.2	GDF10	0.819134544	2.116999657	0.007638
ENSG00000170961.6	HAS2	0.819078519	2.966602726	0.00108
ENSG00000106665.15	CLIP2	0.818708957	6.377871632	4.19E-07
ENSG00000102595.19	UGGT2	0.818604481	5.217596751	6.06E-06
ENSG00000274760.1	RF02216	0.818376583	5.765573013	1.72E-06
ENSG00000105287.12	PRKD2	0.818057557	8.589766055	2.57E-09
ENSG00000259529.1	AL136295.5	0.817896727	12.40493073	3.94E-13
ENSG00000033170.16	FUT8	0.817877269	7.15601415	6.98E-08
ENSG00000157637.12	SLC38A10	0.817849054	9.712687186	1.94E-10
ENSG00000134109.10	EDEM1	0.817721401	10.40958683	3.89E-11
ENSG00000112175.7	BMP5	0.817187953	4.51258725	3.07E-05
ENSG00000117643.14	MAN1C1	0.817063318	5.096559033	8.01E-06
ENSG00000188825.13	LINC00910	0.817043488	7.045081331	9.01E-08
ENSG00000259024.6	TVP23C-CDRT4	0.81685521	9.15668333	6.97E-10
ENSG00000123612.15	ACVR1C	0.816464615	1.474374377	0.033545
ENSG00000062716.12	VMP1	0.816363777	13.19758595	6.34E-14
ENSG00000285509.1	AP000646.1	0.816270758	6.93521712	1.16E-07
ENSG00000060491.16	OGFR	0.816176531	14.47195742	3.37E-15

ENSG00000154065.16	ANKRD29	0.815762046	5.145343749	7.16E-06
ENSG00000105722.9	ERF	0.815672858	10.27950686	5.25E-11
ENSG00000262209.2	PCDHGB3	0.815377127	6.30913963	4.91E-07
ENSG00000280758.1	AL356481.3	0.815364513	7.163781617	6.86E-08
ENSG00000077616.10	NAALAD2	0.815158547	3.114662378	0.000768
ENSG00000197594.12	ENPP1	0.815040395	3.188466775	0.000648
ENSG00000115750.16	TAF1B	0.814883938	6.257524192	5.53E-07
ENSG00000133805.15	AMPD3	0.814702393	3.113008294	0.000771
ENSG00000140575.12	IQGAP1	0.814593333	8.559301972	2.76E-09
ENSG00000253485.2	PCDHGA5	0.814540919	6.272112799	5.34E-07
ENSG00000123146.19	ADGRE5	0.814537041	12.19477498	6.39E-13
ENSG00000253910.2	PCDHGB2	0.814018789	6.316332134	4.83E-07
ENSG00000226925.1	IL1R1-AS1	0.813953244	2.540608378	0.00288
ENSG00000130244.12	FAM98C	0.813080911	11.10906867	7.78E-12
ENSG00000147010.17	SH3KBP1	0.813035053	9.257602484	5.53E-10
ENSG00000253537.2	PCDHGA7	0.812785744	6.280777187	5.24E-07
ENSG00000010704.18	HFE	0.812547399	8.154592063	7.00E-09
ENSG00000262576.2	PCDHGA4	0.812486521	6.253903063	5.57E-07
ENSG00000267436.1	AC005786.3	0.812358056	6.847168659	1.42E-07
ENSG00000253767.2	PCDHGA8	0.812301088	6.235071051	5.82E-07
ENSG00000183153.6	GJD3	0.812301011	4.019679462	9.56E-05
ENSG00000239672.7	NME1	0.811771581	12.36561813	4.31E-13
ENSG00000158220.13	ESYT3	0.811708312	2.628336884	0.002353
ENSG00000262712.1	AC012676.1	0.811646423	4.900175332	1.26E-05
ENSG00000112763.16	BTN2A1	0.811133996	25.51500911	3.05E-26
ENSG00000188004.10	SNHG28	0.811117731	4.464765734	3.43E-05
ENSG00000148057.15	IDNK	0.810867832	7.449116759	3.56E-08
ENSG00000229299.2	AL121845.1	0.810775984	4.614874027	2.43E-05
ENSG00000228106.5	AL392172.1	0.810646275	3.301855374	0.000499
ENSG00000152475.6	ZNF837	0.810603538	5.231906766	5.86E-06
ENSG00000138764.14	CCNG2	0.810475958	6.413510522	3.86E-07
ENSG00000100060.17	MFNG	0.810246524	7.026231849	9.41E-08
ENSG00000104427.11	ZC2HC1A	0.809989907	4.6577743	2.20E-05
ENSG00000162695.11	SLC30A7	0.809835809	7.505297407	3.12E-08
ENSG00000175745.12	NR2F1	0.809818278	1.809695187	0.015499
ENSG00000101335.9	MYL9	0.809611166	6.470106833	3.39E-07
ENSG00000166002.6	SMCO4	0.809545231	4.483675822	3.28E-05
ENSG00000253846.2	PCDHGA10	0.80952071	6.240459001	5.75E-07
ENSG00000169410.9	PTPN9	0.808824519	8.823413359	1.50E-09
ENSG00000013810.18	TACC3	0.808505144	8.058967832	8.73E-09
ENSG00000152192.7	POU4F1	0.808161669	1.681181609	0.020836
ENSG00000231768.1	LINC01354	0.80814733	2.061610606	0.008677
ENSG00000138587.5	MNS1	0.807138953	3.125348649	0.000749
ENSG00000111049.3	MYF5	0.807093326	2.67717731	0.002103
ENSG00000157404.15	KIT	0.807035251	2.589696922	0.002572
ENSG00000185745.9	IFIT1	0.807002336	7.762122896	1.73E-08
ENSG00000136783.9	NIPSNAP3A	0.806661507	16.32270304	4.76E-17

ENSG00000255438.2	AL354813.1	0.806607729	3.980211057	0.000105
ENSG00000108819.10	PPP1R9B	0.806493533	12.95788393	1.10E-13
ENSG00000107521.18	HPS1	0.806435971	9.567140708	2.71E-10
ENSG00000137460.8	FHDC1	0.806434307	4.765890917	1.71E-05
ENSG00000272668.2	AL590560.1	0.805581995	2.488084183	0.00325
ENSG00000254245.2	PCDHGA3	0.805564191	6.149925494	7.08E-07
ENSG00000136830.11	FAM129B	0.80551469	12.86235285	1.37E-13
ENSG00000184867.13	ARMCX2	0.805477517	13.16237437	6.88E-14
ENSG00000145022.4	TCTA	0.804926975	16.08456659	8.23E-17
ENSG00000272760.1	AC093726.1	0.804876243	3.313159893	0.000486
ENSG00000174718.11	KIAA1551	0.804522671	4.892305566	1.28E-05
ENSG00000253873.5	PCDHGA11	0.804502605	6.193812507	6.40E-07
ENSG00000111879.19	FAM184A	0.804391124	3.280576651	0.000524
ENSG00000245468.3	LINC02447	0.804348471	1.649968504	0.022389
ENSG00000074842.7	MYDGF	0.804095881	8.897678796	1.27E-09
ENSG00000244041.7	LINC01011	0.803880412	8.618006464	2.41E-09
ENSG00000253953.2	PCDHGB4	0.803843906	6.177473622	6.65E-07
ENSG00000276547.1	PCDHGB5	0.803483778	6.216451667	6.08E-07
ENSG00000265982.1	AC103810.3	0.8033908	2.496365018	0.003189
ENSG00000265393.1	AC084125.4	0.803292905	2.018780573	0.009577
ENSG00000147536.11	GINS4	0.803273729	4.180365631	6.60E-05
ENSG00000242419.5	PCDHGC4	0.802895584	6.195975774	6.37E-07
ENSG00000124602.9	UNC5CL	0.802866213	2.156709546	0.006971
ENSG00000137807.15	KIF23	0.80279792	2.268828702	0.005385
ENSG00000198300.13	PEG3	0.801947449	6.048733718	8.94E-07
ENSG00000170340.10	B3GNT2	0.80173957	7.328927333	4.69E-08
ENSG00000109339.21	MAPK10	0.801646211	10.65626023	2.21E-11
ENSG00000160796.16	NBEAL2	0.801328087	8.056666732	8.78E-09
ENSG00000184785.5	SMIM10	0.801117205	3.174638332	0.000669
ENSG00000232133.1	IMPDH1P10	0.800162892	7.305293179	4.95E-08
ENSG00000281887.3	GIMAP1-GIMAP5	0.799994706	8.557248534	2.77E-09
ENSG00000253731.2	PCDHGA6	0.79933788	6.087765032	8.17E-07
ENSG00000139192.11	TAPBPL	0.79920223	13.10637439	7.83E-14
ENSG00000139187.9	KLRG1	0.798954193	3.903030062	0.000125
ENSG00000178694.9	NSUN3	0.79823049	9.574455942	2.66E-10
ENSG00000155304.5	HSPA13	0.79815886	6.705052693	1.97E-07
ENSG00000144645.13	OSBPL10	0.797838968	6.414571285	3.85E-07
ENSG00000114790.12	ARHGEF26	0.797734152	2.554970018	0.002786
ENSG00000143479.16	DYRK3	0.797495217	2.534125907	0.002923
ENSG00000152127.8	MGAT5	0.796977073	8.507066843	3.11E-09
ENSG00000228192.7	AL512353.1	0.796473675	8.447780294	3.57E-09
ENSG00000196358.10	NTNG2	0.796332911	1.70084432	0.019914
ENSG00000243660.9	ZNF487	0.796231703	3.750063419	0.000178
ENSG00000241288.7	AC092902.2	0.796126154	1.58373203	0.026078
ENSG00000240764.3	PCDHGC5	0.796126147	6.136185011	7.31E-07
ENSG00000183496.5	MEX3B	0.795997323	5.782334563	1.65E-06
ENSG00000147535.16	PLPP5	0.795884702	8.754500378	1.76E-09

ENSG00000079150.17	FKBP7	0.795531244	6.510608509	3.09E-07
ENSG00000137821.11	LRRC49	0.795438611	4.843982839	1.43E-05
ENSG00000038210.12	PI4K2B	0.794587795	7.065544002	8.60E-08
ENSG00000104870.12	FCGRT	0.794365882	8.711391433	1.94E-09
ENSG00000149260.16	CAPN5	0.794334637	5.788330262	1.63E-06
ENSG00000270504.1	AL391422.4	0.794282122	4.254862335	5.56E-05
ENSG00000186480.12	INSIG1	0.794192946	7.383942089	4.13E-08
ENSG00000152558.14	TMEM123	0.794094161	11.33161587	4.66E-12
ENSG00000223804.5	AC244669.1	0.793998196	2.474002026	0.003357
ENSG00000187049.9	TMEM216	0.793829615	7.183135715	6.56E-08
ENSG00000163389.11	POGLUT1	0.793656001	10.30566499	4.95E-11
ENSG00000197077.13	KIAA1671	0.793634539	4.942304915	1.14E-05
ENSG00000141012.12	GALNS	0.793580981	10.35475117	4.42E-11
ENSG00000112769.18	LAMA4	0.793574321	3.956720855	0.00011
ENSG00000087258.14	GNAO1	0.793202941	6.282852775	5.21E-07
ENSG00000104951.15	IL4I1	0.793056267	9.747662924	1.79E-10
ENSG00000096093.15	EFHC1	0.793030392	7.674210597	2.12E-08
ENSG00000068489.12	PRR11	0.792827808	6.911788435	1.23E-07
ENSG00000260912.1	AL158206.1	0.791848896	2.384435742	0.004126
ENSG00000114115.9	RBP1	0.790633148	3.57890429	0.000264
ENSG00000083720.12	OXCT1	0.790495981	3.627285451	0.000236
ENSG00000283122.1	HYMAI	0.790297749	3.627774068	0.000236
ENSG00000255680.1	AC091564.6	0.790245892	10.63431571	2.32E-11
ENSG00000132793.11	LPIN3	0.789917855	4.177703952	6.64E-05
ENSG00000125355.15	TMEM255A	0.789885576	2.20326711	0.006262
ENSG00000167208.14	SNX20	0.789658175	3.912176011	0.000122
ENSG00000245552.6	AP000787.1	0.789357485	2.780951697	0.001656
ENSG00000090520.11	DNAJB11	0.789039297	16.72605721	1.88E-17
ENSG00000204956.5	PCDHGA1	0.789037871	5.983138632	1.04E-06
ENSG00000151575.14	TEX9	0.788753287	2.136608938	0.007301
ENSG00000081853.14	PCDHGA2	0.788559623	5.900911216	1.26E-06
ENSG00000150977.10	RILPL2	0.788369793	7.409351974	3.90E-08
ENSG00000132718.8	SYT11	0.788340404	7.132966474	7.36E-08
ENSG00000091592.15	NLRP1	0.788303778	5.853273484	1.40E-06
ENSG00000108784.9	NAGLU	0.788193881	8.551467616	2.81E-09
ENSG00000008516.16	MMP25	0.788030737	2.677549918	0.002101
ENSG00000143079.14	CTTNBP2NL	0.787703147	10.26672874	5.41E-11
ENSG00000089685.14	BIRC5	0.787329678	1.620825411	0.023943
ENSG00000121653.11	MAPK8IP1	0.787268665	8.681737317	2.08E-09
ENSG00000100156.10	SLC16A8	0.786944729	2.175604765	0.006674
ENSG00000187514.16	PTMA	0.786702731	9.964274958	1.09E-10
ENSG00000197372.9	ZNF675	0.78652013	5.944137355	1.14E-06
ENSG00000104883.7	PEX11G	0.786502177	6.857084927	1.39E-07
ENSG00000254122.2	PCDHGB7	0.786427834	6.133059303	7.36E-07
ENSG00000166689.15	PLEKHA7	0.786364042	8.235536192	5.81E-09
ENSG00000163879.10	DNALI1	0.786155007	7.100006304	7.94E-08
ENSG00000282951.1	AC008537.4	0.786027789	7.209661557	6.17E-08

ENSG00000163701.18	IL17RE		0.785687687	1.656626796	0.022048
ENSG00000170323.8	FABP4		0.785243287	1.401731291	0.039652
ENSG00000162639.15	HENMT1		0.785217205	5.567940455	2.70E-06
ENSG00000260729.1	AC009690.1		0.785128781	11.35762999	4.39E-12
ENSG00000102362.15	SYTL4		0.785108152	3.250045964	0.000562
ENSG00000197183.14	NOL4L		0.784681239	7.065116839	8.61E-08
ENSG00000075624.14	ACTB		0.784514724	8.353817332	4.43E-09
ENSG00000254963.1	AP001972.2		0.784163747	2.619374953	0.002402
ENSG00000090971.4	NAT14		0.783797827	9.012245297	9.72E-10
ENSG00000138395.14	CDK15		0.783646486	3.736975661	0.000183
ENSG00000144369.12	FAM171B		0.783397827	3.358620052	0.000438
ENSG00000177990.11	DPY19L2		0.783338493	1.460662813	0.034621
ENSG00000100100.12	PIK3IP1		0.783177787	5.581905864	2.62E-06
ENSG00000008300.16	CELSR3		0.782978131	3.35530787	0.000441
ENSG00000152359.14	POC5		0.782922168	8.184886791	6.53E-09
ENSG00000075618.17	FSCN1		0.782746879	4.763554148	1.72E-05
ENSG00000103489.11	XYLT1		0.782673639	3.313976302	0.000485
ENSG00000104856.13	RELB		0.782570433	6.759741443	1.74E-07
ENSG00000129667.12	RHBDF2		0.782474319	7.299686139	5.02E-08
ENSG00000284542.1	MIR3614		0.782297395	7.40777025	3.91E-08
ENSG00000183741.11	CBX6		0.782234608	15.55121189	2.81E-16
ENSG00000123975.4	CKS2		0.78126401	4.680225137	2.09E-05
ENSG00000270011.7	ZNF559-ZNF177		0.780815142	5.920125274	1.20E-06
ENSG00000280339.1	AP001528.3		0.780708409	4.268629563	5.39E-05
ENSG00000173821.19	RNF213		0.779599208	11.95529704	1.11E-12
ENSG00000139211.6	AMIGO2		0.779517687	5.718798221	1.91E-06
ENSG00000104765.15	BNIP3L		0.779142188	6.831449115	1.47E-07
ENSG00000152953.12	STK32B		0.77853469	2.592286226	0.002557
ENSG00000134574.11	DDB2		0.778486416	10.27622173	5.29E-11
ENSG00000253305.2	PCDHGB6		0.778358115	5.99203904	1.02E-06
ENSG00000146828.17	SLC12A9		0.778356078	10.08333043	8.25E-11
ENSG00000164124.10	TMEM144		0.778002798	8.838397528	1.45E-09
ENSG00000087077.13	TRIP6		0.777624675	5.661076923	2.18E-06
ENSG00000119333.11	WDR34		0.777427735	7.613567491	2.43E-08
ENSG00000138758.11		11-Sep	0.777096368	12.17953762	6.61E-13
ENSG00000188629.12	ZNF177		0.777088083	2.935103194	0.001161
ENSG00000250397.2	AP006623.1		0.77615333	2.460357902	0.003465
ENSG00000086570.12	FAT2		0.775457942	2.357925232	0.004386
ENSG00000079819.18	EPB41L2		0.775359841	5.620586694	2.40E-06
ENSG00000198211.8	AC092143.1		0.77517956	2.397104999	0.004008
ENSG00000165169.10	DYNLT3		0.7747846	10.669527	2.14E-11
ENSG00000153363.12	LINC00467		0.773670125	6.813540782	1.54E-07
ENSG00000153250.19	RBMS1		0.773574926	9.452938133	3.52E-10
ENSG00000131323.14	TRAF3		0.77326335	12.51893054	3.03E-13
ENSG00000010295.19	IFFO1		0.772985867	10.98357638	1.04E-11
ENSG00000130827.6	PLXNA3		0.772082653	8.465742509	3.42E-09
ENSG00000154370.15	TRIM11		0.771869223	9.692474185	2.03E-10

ENSG00000197860.9	SGTB	0.771123567	11.11059525	7.75E-12
ENSG00000272168.6	CASC15	0.770973872	2.565574781	0.002719
ENSG00000104723.20	TUSC3	0.770889054	7.561094627	2.75E-08
ENSG00000150760.12	DOCK1	0.770457604	7.330059737	4.68E-08
ENSG00000197283.16	SYNGAP1	0.770354315	9.405090911	3.93E-10
ENSG00000075651.15	PLD1	0.770301009	5.059048671	8.73E-06
ENSG00000115687.13	PASK	0.7701202	7.020136615	9.55E-08
ENSG00000158286.12	RNF207	0.769769269	5.987342785	1.03E-06
ENSG00000183114.7	FAM43B	0.769697243	2.317687455	0.004812
ENSG00000274630.1	AC125257.2	0.769596611	5.872307691	1.34E-06
ENSG00000149798.4	CDC42EP2	0.769592154	7.955119147	1.11E-08
ENSG00000130600.18	H19	0.769455843	3.423584521	0.000377
ENSG00000111780.8	AL021546.1	0.769270106	8.903169923	1.25E-09
ENSG00000157240.3	FZD1	0.768987438	3.128512708	0.000744
ENSG00000136098.16	NEK3	0.768951227	8.272489051	5.34E-09
ENSG00000153714.5	LURAP1L	0.76876795	2.921280095	0.001199
ENSG00000171631.14	P2RY6	0.768531499	4.115644754	7.66E-05
ENSG00000225077.3	LINC00337	0.76849533	3.198771523	0.000633
ENSG00000261460.1	AC009690.2	0.768382316	9.233158326	5.85E-10
ENSG00000149346.14	SLX4IP	0.768279998	10.03512763	9.22E-11
ENSG00000138698.14	RAP1GDS1	0.767706453	8.485003442	3.27E-09
ENSG00000259185.1	AC090971.1	0.767357193	5.527745691	2.97E-06
ENSG00000187231.13	SESTD1	0.767267341	4.753224807	1.77E-05
ENSG00000082438.15	COBLL1	0.767087938	3.869245674	0.000135
ENSG00000115841.19	RMDN2	0.766929298	7.146895044	7.13E-08
ENSG00000196116.7	TDRD7	0.766452442	14.46058179	3.46E-15
ENSG00000162825.16	NBPF20	0.765856455	3.470148609	0.000339
ENSG00000284976.1	BX255925.3	0.765746556	11.21880476	6.04E-12
ENSG00000275371.1	AC012645.4	0.764308857	1.34568968	0.045114
ENSG00000130313.6	PGLS	0.764226912	8.931405227	1.17E-09
ENSG00000126821.7	SGPP1	0.763974439	9.137972451	7.28E-10
ENSG00000067992.13	PDK3	0.763941748	2.019495183	0.009561
ENSG00000160209.18	PDXK	0.762833559	9.119143426	7.60E-10
ENSG00000273749.4	CYFIP1	0.762736304	16.5655865	2.72E-17
ENSG00000101096.19	NFATC2	0.762338094	5.888847331	1.29E-06
ENSG00000184009.11	ACTG1	0.762259747	9.00826035	9.81E-10
ENSG00000090534.19	THPO	0.762259425	5.803004368	1.57E-06
ENSG00000248668.2	OXCT1-AS1	0.762056035	2.313342398	0.00486
ENSG00000196562.14	SULF2	0.761952623	4.586911545	2.59E-05
ENSG00000161638.10	ITGA5	0.76115811	7.929279138	1.18E-08
ENSG00000111269.2	CREBL2	0.761121632	8.603784178	2.49E-09
ENSG00000111665.11	CDCA3	0.760968026	5.598237803	2.52E-06
ENSG00000267552.6	AC093227.2	0.760408556	1.781747588	0.016529
ENSG00000197054.11	ZNF763	0.760241995	4.720669825	1.90E-05
ENSG00000166473.17	PKD1L2	0.760058272	1.884294745	0.013053
ENSG00000111450.13	STX2	0.759841234	7.805029972	1.57E-08
ENSG00000112576.12	CCND3	0.759668518	9.008778235	9.80E-10

ENSG00000114993.16	RTKN	0.758821631	17.12493911	7.50E-18
ENSG00000163354.14	DCST2	0.758764585	1.545821119	0.028456
ENSG00000255410.1	AC091564.5	0.758738656	2.300331925	0.005008
ENSG00000152457.17	DCLRE1C	0.758598284	7.975969652	1.06E-08
ENSG00000148468.16	FAM171A1	0.758578707	8.630067656	2.34E-09
ENSG00000275266.1	RF01972	0.758469841	3.293506144	0.000509
ENSG00000110274.15	CEP164	0.758352751	6.788447154	1.63E-07
ENSG00000188522.14	FAM83G	0.758170425	4.021706303	9.51E-05
ENSG00000104728.15	ARHGEF10	0.75816341	4.182174519	6.57E-05
ENSG00000154262.12	ABCA6	0.757617637	2.9658116	0.001082
ENSG00000189223.14	PAX8-AS1	0.757427094	1.346776771	0.045001
ENSG00000158966.14	CACHD1	0.756923619	3.434184612	0.000368
ENSG00000177410.12	ZFAS1	0.756755242	5.392849301	4.05E-06
ENSG00000141380.13	SS18	0.756742922	12.08775992	8.17E-13
ENSG00000134853.11	PDGFRA	0.756699893	4.000009177	1.00E-04
ENSG00000124766.6	SOX4	0.756433932	5.782500416	1.65E-06
ENSG00000159322.17	ADPGK	0.75583472	9.418546226	3.81E-10
ENSG00000188467.10	SLC24A5	0.755094911	1.35004473	0.044664
ENSG00000196839.12	ADA	0.75498235	7.623134797	2.38E-08
ENSG00000171680.21	PLEKHG5	0.754784193	6.712736016	1.94E-07
ENSG00000274072.1	RF01684	0.75473898	2.804295196	0.001569
ENSG00000116514.16	RNF19B	0.754613663	10.7468109	1.79E-11
ENSG00000160172.10	FAM86C2P	0.753866785	1.868346383	0.013541
ENSG00000280161.1	AC022413.1	0.753379898	1.559817887	0.027554
ENSG00000152763.16	WDR78	0.75318798	2.245144661	0.005687
ENSG00000165507.8	DEPP1	0.753101658	3.696764169	0.000201
ENSG00000203485.12	INF2	0.753090649	11.91173575	1.23E-12
ENSG00000261487.1	AC135048.1	0.753036046	7.952969896	1.11E-08
ENSG00000148773.13	MKI67	0.752964567	2.478507953	0.003323
ENSG00000162734.12	PEA15	0.752962239	9.141569894	7.22E-10
ENSG00000144681.10	STAC	0.752755473	2.574790321	0.002662
ENSG00000236472.1	AC002401.1	0.752590286	9.098066816	7.98E-10
ENSG00000106100.10	NOD1	0.752401346	10.41127025	3.88E-11
ENSG00000261011.1	AL136982.4	0.752133101	2.500048492	0.003162
ENSG00000169379.15	ARL13B	0.751914455	5.852045964	1.41E-06
ENSG00000204161.13	TMEM273	0.75189284	6.443258461	3.60E-07
ENSG00000263776.1	SNORA4	0.75179101	1.659501921	0.021903
ENSG00000155366.16	RHOC	0.751577074	9.75848575	1.74E-10
ENSG00000205771.6	CATSPER2P1	0.751571796	12.45968005	3.47E-13
ENSG00000169188.4	APEX2	0.751363979	15.40574459	3.93E-16
ENSG00000142632.16	ARHGEF19	0.751318274	3.34520784	0.000452
ENSG00000107562.16	CXCL12	0.750554382	10.20854819	6.19E-11
ENSG00000213123.10	TCTEX1D2	0.750541824	4.724833898	1.88E-05
ENSG00000143341.11	HMCN1	0.750455458	2.584931291	0.002601
ENSG00000267379.1	AC008569.1	0.750281336	8.629529764	2.35E-09
ENSG00000132359.14	RAP1GAP2	0.750107564	3.976584065	0.000106
ENSG00000198848.12	CES1	0.750028391	1.35794811	0.043858

ENSG00000257594.3	GALNT4	0.749531696	6.256504605	5.54E-07
ENSG00000167371.19	PRRT2	0.749309939	3.232219869	0.000586
ENSG00000187017.16	ESPN	0.749052182	1.574578425	0.026633
ENSG00000115091.11	ACTR3	0.749006308	19.55369749	2.79E-20
ENSG00000163874.10	ZC3H12A	0.748849326	5.636827457	2.31E-06
ENSG00000144485.10	HES6	0.748512199	2.270456762	0.005365
ENSG00000167397.14	VKORC1	0.748043352	5.587960352	2.58E-06
ENSG00000120594.16	PLXDC2	0.74788242	2.6297089	0.002346
ENSG00000178996.13	SNX18	0.747474831	12.17714847	6.65E-13
ENSG00000284503.1	MIR3652	0.747282236	9.15973444	6.92E-10
ENSG00000225778.5	PROSER2-AS1	0.746742588	3.282860284	0.000521
ENSG00000204520.12	MICA	0.74654321	5.403137774	3.95E-06
ENSG00000136710.9	CCDC115	0.746051433	11.34682591	4.50E-12
ENSG00000102383.13	ZDHHC15	0.74591684	2.169726629	0.006765
ENSG00000213713.3	PIGCP1	0.745841351	5.630082919	2.34E-06
ENSG00000100299.17	ARSA	0.745569923	7.279938288	5.25E-08
ENSG00000170801.9	HTRA3	0.745538659	2.582809714	0.002613
ENSG00000170266.15	GLB1	0.745518683	9.733730581	1.85E-10
ENSG00000138594.13	TMOD3	0.745432827	9.551865034	2.81E-10
ENSG00000134851.12	TMEM165	0.745319986	12.04255389	9.07E-13
ENSG00000101350.7	KIF3B	0.745129027	6.310187892	4.90E-07
ENSG00000101474.11	APMAP	0.744297604	9.260933448	5.48E-10
ENSG00000187240.14	DYNC2H1	0.744181349	3.222733035	0.000599
ENSG00000179144.4	GIMAP7	0.744081799	5.49388549	3.21E-06
ENSG00000243335.9	KCTD7	0.74397349	14.22792416	5.92E-15
ENSG00000085563.14	ABCB1	0.743784932	2.298914744	0.005024
ENSG00000145247.11	OCIAD2	0.743752006	7.36535888	4.31E-08
ENSG00000196937.10	FAM3C	0.743692729	6.299125562	5.02E-07
ENSG00000163617.10	CCDC191	0.743577783	4.862235106	1.37E-05
ENSG00000183255.11	PTTG1IP	0.743268039	12.23926962	5.76E-13
ENSG00000128283.6	CDC42EP1	0.743198316	6.285911734	5.18E-07
ENSG00000135052.16	GOLM1	0.743085122	8.299623739	5.02E-09
ENSG00000146021.14	KLHL3	0.742853449	6.425098786	3.76E-07
ENSG00000185070.10	FLRT2	0.742686348	6.722470632	1.89E-07
ENSG00000167654.17	ATCAY	0.742603666	2.440786	0.003624
ENSG00000159110.19	IFNAR2	0.742358579	12.00162766	9.96E-13
ENSG00000233033.1	CASK-AS1	0.742034189	3.827152892	0.000149
ENSG00000168350.7	DEGS2	0.741802122	7.470135063	3.39E-08
ENSG00000196405.12	EVL	0.741776876	8.930848732	1.17E-09
ENSG00000197892.12	KIF13B	0.741660852	16.75439125	1.76E-17
ENSG00000205220.11	PSMB10	0.741456356	6.923662476	1.19E-07
ENSG00000089057.14	SLC23A2	0.740783112	10.31273278	4.87E-11
ENSG00000132669.13	RIN2	0.740460523	5.278386023	5.27E-06
ENSG00000276166.1	AC092118.2	0.740314425	1.881088708	0.01315
ENSG00000164402.13	8-Sep	0.739983424	7.836622678	1.46E-08
ENSG00000124357.12	NAGK	0.739838729	12.92798854	1.18E-13
ENSG00000175220.11	ARHGAP1	0.739734839	9.231615501	5.87E-10

ENSG00000139974.15	SLC38A6	0.739589467	8.095619124	8.02E-09
ENSG00000270149.5	AL591806.3	0.739266379	11.49259575	3.22E-12
ENSG00000005381.7	MPO	0.739174279	3.244062791	0.00057
ENSG00000149485.18	FADS1	0.739134493	4.977877267	1.05E-05
ENSG00000156398.12	SFXN2	0.738626375	4.947526281	1.13E-05
ENSG00000149428.18	HYOU1	0.73787693	15.67985226	2.09E-16
ENSG00000183260.7	ABHD16B	0.737545345	5.74063537	1.82E-06
ENSG00000198053.11	SIRPA	0.737411717	4.330626277	4.67E-05
ENSG00000091039.16	OSBPL8	0.737053936	7.2006655	6.30E-08
ENSG00000205740.2	AL359878.1	0.736821427	1.344706917	0.045216
ENSG00000155893.12	PXYLP1	0.736403229	5.063026022	8.65E-06
ENSG00000127314.17	RAP1B	0.735906538	8.706631566	1.97E-09
ENSG00000068366.19	ACSL4	0.735756029	16.57001705	2.69E-17
ENSG00000284055.1	MIR4315-1	0.735148452	1.63245999	0.02331
ENSG00000267179.1	AC008770.2	0.734982468	6.403303028	3.95E-07
ENSG00000139641.12	ESYT1	0.734821787	11.41395932	3.86E-12
ENSG00000168807.16	SNTB2	0.734425304	6.49584737	3.19E-07
ENSG00000153395.9	LPCAT1	0.734293239	6.440202898	3.63E-07
ENSG00000115419.12	GLS	0.73423246	15.16814939	6.79E-16
ENSG00000234936.1	AC010883.1	0.734107534	2.825708569	0.001494
ENSG00000134072.10	CAMK1	0.734092486	5.778292681	1.67E-06
ENSG00000114353.16	GNAI2	0.733828827	9.279868857	5.25E-10
ENSG00000179218.13	CALR	0.733130267	11.7948028	1.60E-12
ENSG00000110455.13	ACCS	0.733032509	4.033744765	9.25E-05
ENSG00000198521.11	ZNF43	0.73293765	6.926236223	1.19E-07
ENSG00000180257.13	ZNF816	0.732504283	4.614874027	2.43E-05
ENSG00000196110.7	ZNF699	0.732444134	1.547254106	0.028363
ENSG00000187091.13	PLCD1	0.73196086	7.585605847	2.60E-08
ENSG00000213903.8	LTB4R	0.731828241	7.652702676	2.22E-08
ENSG00000223799.1	IL10RB-DT	0.731239594	9.443584235	3.60E-10
ENSG00000140332.15	TLE3	0.731207135	6.149836814	7.08E-07
ENSG00000048740.18	CELF2	0.730911353	8.749550597	1.78E-09
ENSG00000196526.10	AFAP1	0.730320359	6.046123896	8.99E-07
ENSG00000232149.1	FERP1	0.730018102	4.653890902	2.22E-05
ENSG00000206527.9	HACD2	0.729949861	4.2097164	6.17E-05
ENSG00000221890.3	NPTXR	0.729891593	3.705510589	0.000197
ENSG00000182247.9	UBE2E2	0.729858886	15.85249793	1.40E-16
ENSG00000260778.5	AC009065.4	0.729794527	1.723681357	0.018894
ENSG00000125877.12	ITPA	0.729755363	17.6110883	2.45E-18
ENSG00000094975.13	SUCO	0.729337741	10.00006297	1.00E-10
ENSG00000185379.20	RAD51D	0.729321044	9.658746106	2.19E-10
ENSG00000146859.6	TMEM140	0.728564499	8.864143882	1.37E-09
ENSG00000246523.7	AP001528.1	0.728277466	1.352016945	0.044461
ENSG00000254505.9	CHMP4A	0.728075189	15.26401023	5.44E-16
ENSG00000224875.2	AC083949.1	0.727659565	3.100542047	0.000793
ENSG00000074527.11	NTN4	0.727398492	5.485382154	3.27E-06
ENSG00000123870.10	ZNF137P	0.727197295	1.466163088	0.034185

ENSG00000180385.8	EMC3-AS1	0.72685864	4.453762003	3.52E-05
ENSG00000020181.17	ADGRA2	0.725793521	6.478997474	3.32E-07
ENSG00000240963.1	AL645465.1	0.725766616	6.324600558	4.74E-07
ENSG00000168778.11	TCTN2	0.725515045	4.396298139	4.02E-05
ENSG00000138744.14	NAAA	0.725303545	5.212942665	6.12E-06
ENSG00000261063.1	AC009139.2	0.725201739	3.432238704	0.00037
ENSG00000101004.14	NINL	0.72517089	5.529603418	2.95E-06
ENSG00000110880.10	CORO1C	0.724858458	8.762571743	1.73E-09
ENSG00000138386.16	NAB1	0.724366128	9.788753562	1.63E-10
ENSG00000271741.1	AC114490.2	0.724330126	9.060632064	8.70E-10
ENSG00000076706.16	MCAM	0.724161755	3.420213659	0.00038
ENSG00000110080.18	ST3GAL4	0.724084755	7.232092103	5.86E-08
ENSG00000257342.1	AC025165.2	0.724005963	8.838862941	1.45E-09
ENSG00000198885.9	ITPRIPL1	0.723769703	2.781204538	0.001655
ENSG00000116977.18	LGALS8	0.723448913	10.41116563	3.88E-11
ENSG00000166483.10	WEE1	0.722493099	7.123718625	7.52E-08
ENSG00000143367.15	TUFT1	0.722255042	8.58284157	2.61E-09
ENSG00000240801.1	AC132217.1	0.722190814	2.897774975	0.001265
ENSG00000149541.9	B3GAT3	0.722052108	10.69787529	2.01E-11
ENSG00000133195.11	SLC39A11	0.721703626	8.129341466	7.42E-09
ENSG00000151136.14	BTBD11	0.721546962	1.4554813	0.035036
ENSG00000141527.17	CARD14	0.721520982	4.685824319	2.06E-05
ENSG00000260755.1	AC010542.2	0.721134763	3.535976319	0.000291
ENSG00000183726.10	TMEM50A	0.720887711	14.04656487	8.98E-15
ENSG00000277462.1	ZNF670	0.720647218	1.717661532	0.019157
ENSG00000167695.14	FAM57A	0.72061309	5.297599346	5.04E-06
ENSG00000181744.8	C3orf58	0.720580595	11.42657386	3.74E-12
ENSG00000110881.11	ASIC1	0.720535663	3.797708779	0.000159
ENSG00000237036.4	ZEB1-AS1	0.720396344	13.07004926	8.51E-14
ENSG00000184898.6	RBM43	0.720105246	9.590101517	2.57E-10
ENSG00000268858.2	AL118506.1	0.720018357	5.731381501	1.86E-06
ENSG00000143315.7	PIGM	0.719832012	11.08407703	8.24E-12
ENSG00000261094.2	AC007066.2	0.719669109	1.994576284	0.010126
ENSG00000110400.10	NECTIN1	0.719567337	5.95724329	1.10E-06
ENSG00000176014.12	TUBB6	0.719471662	13.36587822	4.31E-14
ENSG00000100271.16	TTLL1	0.719422751	12.91969801	1.20E-13
ENSG00000152240.12	HAUS1	0.719369122	5.854043536	1.40E-06
ENSG00000120693.13	SMAD9	0.719180033	3.533482251	0.000293
ENSG00000100997.18	ABHD12	0.718891718	10.70366271	1.98E-11
ENSG00000220323.4	HIST2H2BD	0.718752189	2.636490442	0.002309
ENSG00000248751.6	AC004997.1	0.718449273	6.291275293	5.11E-07
ENSG00000214021.15	TTLL3	0.718085076	5.413003953	3.86E-06
ENSG00000258656.1	AL160236.1	0.717968213	1.419403892	0.038071
ENSG00000106397.11	PLOD3	0.71766732	11.89706101	1.27E-12
ENSG00000135506.15	OS9	0.717661336	9.555711746	2.78E-10
ENSG00000049449.9	RCN1	0.717567548	18.26207496	5.47E-19
ENSG00000111261.13	MANSC1	0.717462599	3.371847414	0.000425

ENSG00000075223.13	SEMA3C	0.717390144	3.636704699	0.000231
ENSG00000165244.6	ZNF367	0.717353529	5.862482565	1.37E-06
ENSG00000156345.17	CDK20	0.717302542	4.57784333	2.64E-05
ENSG00000169641.13	LUZP1	0.717208917	11.23237103	5.86E-12
ENSG00000196757.7	ZNF700	0.716379089	8.484423801	3.28E-09
ENSG00000177380.13	PPFIA3	0.715625799	4.737091895	1.83E-05
ENSG00000260898.5	ADPGK-AS1	0.715046454	4.323230503	4.75E-05
ENSG00000206561.12	COLQ	0.714883473	2.884626903	0.001304
ENSG00000228509.5	AC006460.1	0.714116305	6.163787381	6.86E-07
ENSG00000171466.9	ZNF562	0.714066084	12.44497971	3.59E-13
ENSG00000186409.15	CCDC30	0.713721079	1.60899266	0.024604
ENSG00000270959.1	LPP-AS2	0.713201399	6.229183613	5.90E-07
ENSG00000178202.12	KDELC2	0.712920098	4.208662761	6.18E-05
ENSG00000131797.12	CLUHP3	0.712838257	5.838258397	1.45E-06
ENSG00000167173.18	C15orf39	0.712637089	6.041325194	9.09E-07
ENSG00000118655.5	DCLRE1B	0.711838822	8.568954157	2.70E-09
ENSG00000271270.6	TMCC1-AS1	0.711754757	4.161605834	6.89E-05
ENSG00000260276.2	AC022167.2	0.711632083	7.590372471	2.57E-08
ENSG00000076662.9	ICAM3	0.711010741	4.857796619	1.39E-05
ENSG00000164638.10	SLC29A4	0.710923358	1.431238213	0.037048
ENSG00000174059.16	CD34	0.710761986	4.118993725	7.60E-05
ENSG00000104081.13	BMF	0.710009737	4.546099076	2.84E-05
ENSG00000266929.1	AC067852.1	0.709171909	6.761532529	1.73E-07
ENSG00000271425.7	NBPF10	0.708838499	4.507917427	3.11E-05
ENSG00000145075.12	CCDC39	0.708345215	4.156002965	6.98E-05
ENSG00000180773.14	SLC36A4	0.70814345	5.043313091	9.05E-06
ENSG00000115963.13	RND3	0.707531078	3.336177163	0.000461
ENSG00000162522.10	KIAA1522	0.707346643	10.26479588	5.44E-11
ENSG00000128274.16	A4GALT	0.707093984	6.713722764	1.93E-07
ENSG00000123427.16	EEF1AKMT3	0.706356174	5.648318899	2.25E-06
ENSG00000223528.7	AL359094.1	0.706124623	4.228011225	5.92E-05
ENSG00000139880.19	CDH24	0.705954376	2.828033677	0.001486
ENSG00000280160.1	AC135050.7	0.705654861	5.049232562	8.93E-06
ENSG00000055163.19	CYFIP2	0.705624272	5.694574921	2.02E-06
ENSG00000072135.12	PTPN18	0.70535886	6.511102408	3.08E-07
ENSG00000187372.11	PCDHB13	0.705249304	1.70426977	0.019757
ENSG00000115041.12	KCNIP3	0.704955274	1.535181298	0.029162
ENSG00000004948.14	CALCR	0.704941808	1.479614939	0.033142
ENSG00000072042.12	RDH11	0.704224518	8.455908503	3.50E-09
ENSG00000168214.20	RBPJ	0.70382191	7.388830693	4.08E-08
ENSG00000161328.10	LRRC56	0.703736171	1.737552867	0.0183
ENSG00000272848.2	AL135910.1	0.703263368	3.121913315	0.000755
ENSG00000021355.12	SERPINB1	0.703108652	10.8303022	1.48E-11
ENSG00000082781.11	ITGB5	0.703076976	2.605093828	0.002483
ENSG00000169241.18	SLC50A1	0.702496133	9.631264339	2.34E-10
ENSG00000198960.10	ARMCX6	0.702170735	8.894918871	1.27E-09
ENSG00000143061.17	IGSF3	0.701977847	3.586935922	0.000259

ENSG00000172824.15	CES4A	0.70176891	3.854805001	0.00014
ENSG00000267980.1	AC007292.1	0.701516324	8.407475219	3.91E-09
ENSG00000131873.6	CHSY1	0.701504026	9.272341045	5.34E-10
ENSG00000134548.10	SPX	0.701130534	2.244098039	0.0057
ENSG00000196247.11	ZNF107	0.700662656	4.707620577	1.96E-05
ENSG00000146833.15	TRIM4	0.700433415	7.014328573	9.68E-08
ENSG00000114861.20	FOXP1	0.700277554	6.932829164	1.17E-07
ENSG00000256262.1	USP30-AS1	0.699953754	4.180742036	6.60E-05
ENSG00000054598.7	FOXC1	0.699634132	4.540776013	2.88E-05
ENSG00000143622.10	RIT1	0.699458617	18.09580171	8.02E-19
ENSG00000222046.2	DCDC2B	0.699454532	3.064117761	0.000863
ENSG00000198951.11	NAGA	0.699276493	7.679196389	2.09E-08
ENSG00000134755.15	DSC2	0.699032151	3.420726423	0.00038
ENSG00000255252.3	AL078612.1	0.698912253	15.25968721	5.50E-16
ENSG00000144959.9	NCEH1	0.698634961	6.817788189	1.52E-07
ENSG00000282826.1	FRG1CP	0.698581111	3.83807139	0.000145
ENSG00000143110.11	C1orf162	0.69846826	3.890490929	0.000129
ENSG00000180155.19	LYNX1	0.697392256	3.881353108	0.000131
ENSG00000197045.12	GMFB	0.697232326	7.127730573	7.45E-08
ENSG00000205730.6	ITPRIPL2	0.697215714	4.987480319	1.03E-05
ENSG00000169136.10	ATF5	0.697103644	11.83419606	1.46E-12
ENSG00000100234.11	TIMP3	0.696978187	4.38798647	4.09E-05
ENSG00000170540.14	ARL6IP1	0.696600894	7.306359664	4.94E-08
ENSG00000253523.1	AC104350.1	0.696557513	1.550421354	0.028156
ENSG00000160190.13	SLC37A1	0.696392096	6.936355269	1.16E-07
ENSG00000260616.6	AC007728.1	0.696332213	1.536924602	0.029045
ENSG00000248734.2	AC008906.1	0.695874853	6.709104141	1.95E-07
ENSG00000144677.14	CTDSPL	0.695801222	7.50207967	3.15E-08
ENSG00000172380.5	GNG12	0.695719867	5.459744419	3.47E-06
ENSG00000029993.14	HMGB3	0.695500727	4.525928678	2.98E-05
ENSG00000104976.11	SNAPC2	0.695086933	7.904076443	1.25E-08
ENSG00000255772.5	LINC01479	0.694986194	1.917372087	0.012096
ENSG00000102003.10	SYP	0.694843387	4.166554485	6.81E-05
ENSG00000205581.10	HMGN1	0.694143366	10.28104173	5.24E-11
ENSG00000186862.18	PDZD7	0.693949329	5.085370093	8.22E-06
ENSG00000164930.11	FZD6	0.693890492	4.099446559	7.95E-05
ENSG00000279118.1	AC093535.2	0.69359742	2.879488716	0.00132
ENSG00000151229.12	SLC2A13	0.693493012	2.013499494	0.009694
ENSG00000144218.18	AFF3	0.6934263	1.72938075	0.018647
ENSG00000170876.7	TMEM43	0.692912759	11.58028142	2.63E-12
ENSG00000175354.19	PTPN2	0.692875661	6.511102408	3.08E-07
ENSG00000068796.16	KIF2A	0.692825818	11.43430037	3.68E-12
ENSG00000137267.5	TUBB2A	0.692742084	8.103514014	7.88E-09
ENSG00000257176.2	AC009318.1	0.692701748	1.807964584	0.015561
ENSG00000138756.17	BMP2K	0.692540882	6.785586838	1.64E-07
ENSG00000267317.2	AC027307.2	0.69241228	4.12581782	7.48E-05
ENSG00000160973.7	FOXH1	0.692293135	2.081590368	0.008287

ENSG00000260669.2	AL096870.2	0.691956141	13.27636199	5.29E-14
ENSG00000234290.2	AC116366.1	0.691874449	5.603668979	2.49E-06
ENSG00000106477.18	CEP41	0.691771979	6.569169698	2.70E-07
ENSG00000255439.6	AC135050.2	0.691655533	5.102097546	7.91E-06
ENSG00000110395.6	CBL	0.691421526	8.940961708	1.15E-09
ENSG00000147437.9	GNRH1	0.691376631	2.097370704	0.007992
ENSG00000165046.12	LETM2	0.690997765	2.003017065	0.009931
ENSG00000270049.2	AC009061.2	0.690727082	2.06456379	0.008619
ENSG00000284707.1	AC079781.5	0.690548605	8.174636777	6.69E-09
ENSG00000141985.9	SH3GL1	0.690317181	8.203852284	6.25E-09
ENSG00000177879.15	AP3S1	0.690169555	5.919428183	1.20E-06
ENSG00000139624.12	CERS5	0.690008666	7.582975983	2.61E-08
ENSG00000138448.11	ITGAV	0.689994047	7.761491127	1.73E-08
ENSG00000168306.12	ACOX2	0.689618618	5.226938849	5.93E-06
ENSG00000284917.1	EEF1AKMT4-ECE2	0.688836216	2.825708569	0.001494
ENSG00000185483.11	ROR1	0.688726537	5.088658249	8.15E-06
ENSG00000233223.2	AC016876.1	0.688647147	6.302080432	4.99E-07
ENSG00000259992.1	AC025287.1	0.688577343	6.905367283	1.24E-07
ENSG00000106617.13	PRKAG2	0.688528016	4.453466544	3.52E-05
ENSG00000227741.1	AL121987.2	0.688440815	6.671591773	2.13E-07
ENSG00000214182.5	PTMAP5	0.688186821	1.315742634	0.048335
ENSG00000112796.9	ENPP5	0.687916202	2.50742325	0.003109
ENSG00000169169.14	CPT1C	0.687900883	2.701333007	0.001989
ENSG00000154025.15	SLC5A10	0.687832963	5.626625662	2.36E-06
ENSG00000162998.4	FRZB	0.687517622	1.365345369	0.043118
ENSG00000197694.15	SPTAN1	0.687165528	8.329518374	4.68E-09
ENSG00000198604.10	BAZ1A	0.686462883	7.768944505	1.70E-08
ENSG00000095015.5	MAP3K1	0.686373104	4.275329557	5.30E-05
ENSG00000117594.9	HSD11B1	0.685945604	2.235335469	0.005817
ENSG00000176903.4	PNMA1	0.685419444	10.22767276	5.92E-11
ENSG00000138182.14	KIF20B	0.684889673	3.002568432	0.000994
ENSG00000280416.1	AC009084.3	0.684501069	6.510045454	3.09E-07
ENSG00000249459.8	ZNF286B	0.684239499	1.974082924	0.010615
ENSG00000100644.16	HIF1A	0.684034602	9.754245148	1.76E-10
ENSG00000198453.12	ZNF568	0.684016797	4.888423623	1.29E-05
ENSG00000101846.6	STS	0.683821534	5.993122886	1.02E-06
ENSG00000125170.10	DOK4	0.683716208	5.428265311	3.73E-06
ENSG00000197329.11	PELI1	0.68312826	8.997653662	1.01E-09
ENSG00000147439.12	BIN3	0.683124645	12.26714256	5.41E-13
ENSG00000119899.12	SLC17A5	0.683058349	6.057821182	8.75E-07
ENSG00000184307.14	ZDHHC23	0.682900641	4.609857842	2.46E-05
ENSG00000270094.1	AL670729.2	0.68273126	4.268506008	5.39E-05
ENSG00000100596.6	SPTLC2	0.682620996	7.697267297	2.01E-08
ENSG00000164078.12	MST1R	0.682613228	4.845043233	1.43E-05
ENSG00000259877.2	AC009113.1	0.68231574	8.376624898	4.20E-09
ENSG00000156500.15	FAM122C	0.682277541	11.36754553	4.29E-12
ENSG00000170921.15	TANC2	0.681987747	8.449355057	3.55E-09

ENSG00000081818.3	PCDHB4	0.681450778	6.896713297	1.27E-07
ENSG00000158555.14	GDPD5	0.680592825	4.195843427	6.37E-05
ENSG00000039068.18	CDH1	0.680543922	1.784289555	0.016433
ENSG00000182481.8	KPNA2	0.680174184	6.493004925	3.21E-07
ENSG00000267519.6	AC020916.1	0.680165959	6.479492934	3.32E-07
ENSG00000100592.15	DAAM1	0.679970312	8.671901466	2.13E-09
ENSG00000285868.1	AC008676.3	0.679871639	2.814084845	0.001534
ENSG00000050344.8	NFE2L3	0.679836547	4.602379975	2.50E-05
ENSG00000173918.14	C1QTNF1	0.679687496	3.362288512	0.000434
ENSG00000109667.11	SLC2A9	0.679521239	1.566288255	0.027146
ENSG00000140104.13	CLBA1	0.679469385	5.862820887	1.37E-06
ENSG00000135951.14	TSGA10	0.679449665	2.330385667	0.004673
ENSG00000228109.1	MELTF-AS1	0.67940402	2.940516656	0.001147
ENSG00000169105.7	CHST14	0.679398125	6.054358202	8.82E-07
ENSG00000104154.6	SLC30A4	0.679281295	6.387683244	4.10E-07
ENSG00000160789.19	LMNA	0.679072987	15.79808425	1.59E-16
ENSG00000137478.14	FCHSD2	0.678790479	12.41302449	3.86E-13
ENSG00000243943.9	ZNF512	0.678701436	16.20981344	6.17E-17
ENSG00000262370.5	AC108134.3	0.677982218	3.769570683	0.00017
ENSG00000198814.12	GK	0.677948841	6.029637356	9.34E-07
ENSG00000172986.12	GXYLT2	0.6775939	7.035335767	9.22E-08
ENSG00000112984.11	KIF20A	0.677563263	1.399689098	0.039839
ENSG00000284691.1	AC073111.5	0.677497272	7.721774798	1.90E-08
ENSG00000189114.6	BLOC1S3	0.677306102	10.38082535	4.16E-11
ENSG00000165355.7	FBXO33	0.677156007	7.856758805	1.39E-08
ENSG00000132670.20	PTPRA	0.677065098	8.471206527	3.38E-09
ENSG00000196476.11	C20orf96	0.676440333	3.914157825	0.000122
ENSG00000104213.12	PDGFRL	0.676411169	3.682378027	0.000208
ENSG00000129048.6	ACKR4	0.67611476	3.309633772	0.00049
ENSG00000044574.7	HSPA5	0.675760488	15.24422565	5.70E-16
ENSG00000117399.13	CDC20	0.675611444	1.678937121	0.020944
ENSG00000166532.15	RIMKLB	0.675602597	10.269018	5.38E-11
ENSG00000148158.16	SNX30	0.675499281	5.914847138	1.22E-06
ENSG00000151303.11	AL136982.1	0.67528068	2.174609854	0.006689
ENSG00000285283.1	AL035078.4	0.674981536	16.852571	1.40E-17
ENSG00000161513.11	FDXR	0.674646142	3.652031955	0.000223
ENSG00000048342.15	CC2D2A	0.674519313	4.891872932	1.28E-05
ENSG00000151388.10	ADAMTS12	0.673796814	3.946856221	0.000113
ENSG00000271811.1	Z97200.1	0.673706337	1.891834151	0.012828
ENSG00000236824.2	BCYRN1	0.673062269	2.558409222	0.002764
ENSG00000167992.12	VWCE	0.673055654	4.103880152	7.87E-05
ENSG00000132010.15	ZNF20	0.67267823	3.27566967	0.00053
ENSG00000123136.14	DDX39A	0.672649984	13.14771004	7.12E-14
ENSG00000166592.11	RRAD	0.672193394	2.074289567	0.008428
ENSG00000135472.8	FAIM2	0.672106396	1.800451629	0.015832
ENSG00000164120.13	HPGD	0.672031569	2.250931742	0.005611
ENSG00000139083.10	ETV6	0.671480148	6.971713003	1.07E-07

ENSG00000108960.8	MMD	0.671256889	1.605068439	0.024827
ENSG00000182185.18	RAD51B	0.671178641	4.524252198	2.99E-05
ENSG00000213096.10	ZNF254	0.670961846	8.538505613	2.89E-09
ENSG00000149573.8	MPZL2	0.67071238	2.25510986	0.005558
ENSG00000114978.17	MOB1A	0.670709427	11.14189966	7.21E-12
ENSG00000173200.12	PARP15	0.670447644	2.475083824	0.003349
ENSG00000196505.10	GDAP2	0.670170949	6.300788929	5.00E-07
ENSG00000154721.14	JAM2	0.670059114	5.629280043	2.35E-06
ENSG00000023516.8	AKAP11	0.670027245	8.462492394	3.45E-09
ENSG00000129173.12	E2F8	0.669730078	2.255820844	0.005549
ENSG00000248671.7	ALG1L9P	0.669347751	1.442978796	0.03606
ENSG00000167778.8	SPRYD3	0.669197598	7.390325123	4.07E-08
ENSG00000132470.13	ITGB4	0.669188081	3.228048227	0.000591
ENSG00000240356.6	RPL23AP7	0.66918647	2.829253425	0.001482
ENSG00000136026.13	CKAP4	0.668911803	11.38618028	4.11E-12
ENSG00000271880.1	AGAP11	0.668614092	2.341415734	0.004556
ENSG00000167535.7	CACNB3	0.668552046	3.212848861	0.000613
ENSG00000174899.10	PQLC2L	0.668226538	1.391898816	0.04056
ENSG00000117602.11	RCAN3	0.66820045	4.67250959	2.13E-05
ENSG00000054938.15	CHRDL2	0.667944029	1.485848671	0.03267
ENSG00000116237.15	ICMT	0.667665778	3.60360491	0.000249
ENSG00000153317.14	ASAP1	0.667269544	10.9799286	1.05E-11
ENSG00000156504.16	FAM122B	0.66724025	9.222456518	5.99E-10
ENSG00000169220.17	RGS14	0.667223016	5.46838387	3.40E-06
ENSG00000197124.11	ZNF682	0.667160508	2.206502233	0.006216
ENSG00000130055.13	GDPD2	0.66659235	1.407842694	0.039098
ENSG00000215302.8	AC127502.1	0.666308769	1.526850782	0.029727
ENSG00000170312.15	CDK1	0.6662155	1.955617735	0.011076
ENSG00000102316.16	MAGED2	0.666208452	7.455346087	3.50E-08
ENSG00000164924.17	YWHAZ	0.66598877	13.55292377	2.80E-14
ENSG00000108774.14	RAB5C	0.665530286	7.762213295	1.73E-08
ENSG00000196968.10	FUT11	0.665389083	14.74298004	1.81E-15
ENSG00000167925.15	GHDC	0.665203203	8.382577174	4.14E-09
ENSG00000172831.11	CES2	0.665155787	7.633140357	2.33E-08
ENSG00000080823.22	MOK	0.66455817	4.250983024	5.61E-05
ENSG00000140451.12	PIF1	0.664340169	2.359135849	0.004374
ENSG00000138185.19	ENTPD1	0.663977091	7.544481271	2.85E-08
ENSG00000198551.9	ZNF627	0.663912859	9.02704153	9.40E-10
ENSG00000177556.11	ATOX1	0.66372703	7.25767133	5.52E-08
ENSG00000141338.13	ABCA8	0.663154521	3.375885651	0.000421
ENSG00000122574.10	WIPF3	0.66307216	2.429536168	0.003719
ENSG00000166548.15	TK2	0.663016699	6.004713746	9.89E-07
ENSG00000138346.14	DNA2	0.662766805	3.130454309	0.000741
ENSG00000173218.14	VANGL1	0.662400875	6.118460936	7.61E-07
ENSG00000144331.19	ZNF385B	0.662118775	2.904066592	0.001247
ENSG00000142192.20	APP	0.662012445	5.929250099	1.18E-06
ENSG00000283239.1	KBTBD11-OT1	0.661346883	4.174564104	6.69E-05

ENSG00000214548.16	MEG3	0.661115018	4.728023315	1.87E-05
ENSG00000197208.5	SLC22A4	0.660863264	4.67250959	2.13E-05
ENSG00000080608.9	PUM3	0.660348114	8.89483605	1.27E-09
ENSG00000158715.5	SLC45A3	0.660155975	2.489625554	0.003239
ENSG00000065135.10	GNAI3	0.659890741	9.141569894	7.22E-10
ENSG00000105327.17	BBC3	0.659727522	3.382291887	0.000415
ENSG00000108518.7	PFN1	0.659591643	12.2187554	6.04E-13
ENSG00000213445.9	SIPA1	0.659463481	5.727325199	1.87E-06
ENSG00000174501.14	ANKRD36C	0.659419894	2.784164414	0.001644
ENSG00000138079.13	SLC3A1	0.658820499	4.268765111	5.39E-05
ENSG00000023171.17	GRAMD1B	0.658817232	2.955119981	0.001109
ENSG00000116489.12	CAPZA1	0.658572746	7.963435808	1.09E-08
ENSG00000154920.14	EME1	0.658483466	4.932254571	1.17E-05
ENSG00000100523.14	DDHD1	0.658295487	13.63471874	2.32E-14
ENSG00000224660.1	SH3BP5-AS1	0.658217908	6.347570232	4.49E-07
ENSG00000072818.11	ACAP1	0.658100172	4.245441336	5.68E-05
ENSG00000117151.12	CTBS	0.657958734	4.72463778	1.89E-05
ENSG00000125148.6	MT2A	0.657706551	3.830965006	0.000148
ENSG00000115318.11	LOXL3	0.657690729	7.342265794	4.55E-08
ENSG00000162407.8	PLPP3	0.657672277	3.950195523	0.000112
ENSG00000268279.4	AC090004.1	0.657582792	9.774034382	1.68E-10
ENSG00000171365.16	CLCN5	0.657496217	6.783617309	1.65E-07
ENSG00000122432.17	SPATA1	0.65742835	5.317283271	4.82E-06
ENSG00000215126.10	CBWD6	0.657352948	2.946100607	0.001132
ENSG00000267493.3	CIRBP-AS1	0.657310019	1.355249841	0.044132
ENSG00000138613.13	APH1B	0.657229874	6.757708469	1.75E-07
ENSG00000119608.12	PROX2	0.657039952	4.954475778	1.11E-05
ENSG00000197291.8	RAMP2-AS1	0.657014349	2.77254086	0.001688
ENSG00000281028.1	AC104662.2	0.656893166	5.548285607	2.83E-06
ENSG00000106089.11	STX1A	0.656344085	6.081021227	8.30E-07
ENSG00000171492.14	LRRC8D	0.656311704	8.15325975	7.03E-09
ENSG00000089123.15	TASP1	0.655736326	5.241427987	5.74E-06
ENSG00000106367.14	AP1S1	0.655405992	9.762889216	1.73E-10
ENSG00000140743.7	CDR2	0.655396674	6.521984124	3.01E-07
ENSG00000198835.3	GJC2	0.655131874	2.168650684	0.006782
ENSG00000088836.13	SLC4A11	0.655050547	2.31333118	0.00486
ENSG00000168237.17	GLYCTK	0.654588471	6.634940039	2.32E-07
ENSG00000147799.11	ARHGAP39	0.654348826	5.242843973	5.72E-06
ENSG00000006327.13	TNFRSF12A	0.654180441	1.729813415	0.018629
ENSG00000285336.1	AC108734.4	0.654101444	3.029422215	0.000934
ENSG00000159176.13	CSRP1	0.654002162	4.715773512	1.92E-05
ENSG00000175866.15	BAIAP2	0.653987089	3.77045505	0.00017
ENSG00000160213.7	CSTB	0.653945834	11.22741016	5.92E-12
ENSG00000117385.15	P3H1	0.653505497	5.972604159	1.07E-06
ENSG00000262112.1	AC015912.1	0.653497413	8.240131888	5.75E-09
ENSG00000272654.1	AL358472.2	0.653012437	1.898821972	0.012623
ENSG00000198648.10	STK39	0.652899033	5.179001427	6.62E-06

ENSG00000260851.6	AC010542.3	0.652565359	4.880230926	1.32E-05
ENSG00000109323.9	MANBA	0.652368345	6.396470223	4.01E-07
ENSG00000198719.8	DLL1	0.652211486	4.849036905	1.42E-05
ENSG00000134070.4	IRAK2	0.652191631	5.93156871	1.17E-06
ENSG00000104177.17	MYEF2	0.652064605	3.560725016	0.000275
ENSG00000138078.15	PREPL	0.65130319	4.135271197	7.32E-05
ENSG00000121060.17	TRIM25	0.651008072	9.248357431	5.64E-10
ENSG00000158195.10	WASF2	0.650615609	7.363964322	4.33E-08
ENSG00000275964.1	AL355001.2	0.650592188	3.154236865	0.000701
ENSG00000270084.1	GAS5-AS1	0.650493147	3.187889169	0.000649
ENSG00000129680.15	MAP7D3	0.649662962	7.766964491	1.71E-08
ENSG00000196502.11	SULT1A1	0.649460084	6.151769987	7.05E-07
ENSG00000214826.5	DDX12P	0.649393041	3.245444938	0.000568
ENSG00000200090.1	RF00019	0.648994958	1.736956594	0.018325
ENSG00000106537.7	TSPAN13	0.648884105	5.218541644	6.05E-06
ENSG00000267748.4	AC011479.1	0.648883339	2.877162706	0.001327
ENSG00000142303.13	ADAMTS10	0.648804208	4.351192689	4.45E-05
ENSG00000248429.5	FAM198B-AS1	0.648647705	3.065544599	0.00086
ENSG00000170439.6	METTL7B	0.648469556	2.543440801	0.002861
ENSG00000095203.14	EPB41L4B	0.648195438	1.549880706	0.028192
ENSG00000265678.1	AC129510.1	0.647967377	1.586521753	0.025911
ENSG00000284862.1	CCDC39	0.647469076	5.218256609	6.05E-06
ENSG00000166347.18	CYB5A	0.647258803	4.945481416	1.13E-05
ENSG00000272991.1	AF129408.1	0.647176728	1.377836545	0.041895
ENSG00000259984.1	AL928711.1	0.646373436	2.945653399	0.001133
ENSG00000165895.18	ARHGAP42	0.64621992	4.332562772	4.65E-05
ENSG00000184445.11	KNTC1	0.646215905	5.158072964	6.95E-06
ENSG00000047578.12	KIAA0556	0.646111092	7.878172854	1.32E-08
ENSG00000136840.18	ST6GALNAC4	0.645956171	5.457707396	3.49E-06
ENSG00000268509.2	AC026202.3	0.645888284	2.17145914	0.006738
ENSG00000101935.9	AMMECR1	0.645862848	6.053403047	8.84E-07
ENSG00000266642.2	AC024267.6	0.6456805	2.461662801	0.003454
ENSG00000158483.15	FAM86C1	0.645560545	4.015385259	9.65E-05
ENSG00000269145.2	AC007192.2	0.645445404	1.87300982	0.013396
ENSG00000230082.1	PRRT3-AS1	0.645170225	3.174638332	0.000669
ENSG00000136108.14	CKAP2	0.645147415	4.300907642	5.00E-05
ENSG00000139190.16	VAMP1	0.644973907	8.707956482	1.96E-09
ENSG00000081870.11	HSPB11	0.644723884	6.354101918	4.42E-07
ENSG00000285577.1	AC019127.1	0.644590672	3.075126538	0.000841
ENSG00000172638.12	EFEMP2	0.644175464	5.636827457	2.31E-06
ENSG00000213073.4	AL353625.1	0.644008183	2.27669327	0.005288
ENSG00000156535.14	CD109	0.643837928	2.706061438	0.001968
ENSG00000065600.12	TMEM206	0.643656045	4.930390275	1.17E-05
ENSG00000168411.13	RFWD3	0.643556881	8.953864695	1.11E-09
ENSG00000164125.15	FAM198B	0.643081153	3.663316669	0.000217
ENSG00000256069.7	A2MP1	0.642792786	3.433962279	0.000368
ENSG00000167680.15	SEMA6B	0.642496684	3.536581081	0.000291

ENSG00000264443.1	AL445686.2	0.642486927	1.670199226	0.02137
ENSG00000201675.1	SNORD32A	0.642342307	1.32496594	0.047319
ENSG00000127946.16	HIP1	0.642217689	6.931795966	1.17E-07
ENSG00000064989.12	CALCRL	0.641989229	3.866570289	0.000136
ENSG00000136104.20	RNASEH2B	0.641744798	5.963329907	1.09E-06
ENSG00000114026.21	OGG1	0.641696113	6.94081977	1.15E-07
ENSG00000137124.7	ALDH1B1	0.639741914	2.805744698	0.001564
ENSG00000185477.4	GPRIN3	0.639709324	2.895376664	0.001272
ENSG00000053747.16	LAMA3	0.639255502	2.991945824	0.001019
ENSG00000136156.13	ITM2B	0.638864351	8.243810215	5.70E-09
ENSG00000090924.14	PLEKHG2	0.638812519	6.088261039	8.16E-07
ENSG00000250312.7	ZNF718	0.638421609	4.588505721	2.58E-05
ENSG00000255390.1	AC091564.4	0.638317229	3.398406028	0.0004
ENSG00000156869.13	FRRS1	0.638092594	4.992612548	1.02E-05
ENSG00000270075.1	AL162742.2	0.637953705	1.632098879	0.023329
ENSG00000173898.12	SPTBN2	0.637862975	4.948453792	1.13E-05
ENSG00000160055.19	TMEM234	0.637705283	5.411824607	3.87E-06
ENSG00000159335.15	PTMS	0.637194793	5.768826709	1.70E-06
ENSG00000099308.10	MAST3	0.637156862	3.789520678	0.000162
ENSG00000162869.15	PPP1R21	0.636958082	13.31242512	4.87E-14
ENSG00000107551.20	RASSF4	0.636837906	5.738775712	1.82E-06
ENSG00000159842.15	ABR	0.636597983	10.99046407	1.02E-11
ENSG00000123353.9	ORMDL2	0.636538243	10.00788115	9.82E-11
ENSG00000186716.20	BCR	0.636109126	4.570263849	2.69E-05
ENSG00000166402.8	TUB	0.63588063	3.997866086	0.0001
ENSG00000188501.11	LCTL	0.635444834	3.835134468	0.000146
ENSG00000221792.1	MIR1282	0.635346856	8.70666276	1.96E-09
ENSG00000150471.16	ADGRL3	0.635077716	1.375949825	0.042078
ENSG00000272674.3	PCDHB16	0.634783377	1.68019841	0.020883
ENSG00000117009.11	KMO	0.634282248	4.614951513	2.43E-05
ENSG00000157927.16	RADIL	0.634205807	3.410744132	0.000388
ENSG00000074800.15	ENO1	0.634066991	9.900700137	1.26E-10
ENSG00000135338.13	LCA5	0.633495302	3.578219784	0.000264
ENSG00000154016.13	GRAP	0.633144834	3.107077533	0.000781
ENSG00000272636.3	DOC2B	0.632814253	3.697624267	0.000201
ENSG00000170271.10	FAXDC2	0.632768123	3.649805616	0.000224
ENSG00000004700.15	RECQL	0.632129417	10.33333592	4.64E-11
ENSG00000162849.15	KIF26B	0.631463442	1.365059658	0.043146
ENSG00000186088.15	GSAP	0.631454212	4.950796964	1.12E-05
ENSG00000245067.6	IGFBP7-AS1	0.630986856	3.83340136	0.000147
ENSG00000137845.14	ADAM10	0.630195434	10.39286954	4.05E-11
ENSG00000127191.17	TRAF2	0.630034762	8.67128453	2.13E-09
ENSG00000148484.17	RSU1	0.630029202	7.867205295	1.36E-08
ENSG00000213614.9	HEXA	0.629453113	8.676769359	2.10E-09
ENSG00000235823.2	OLMALINC	0.62917684	4.196242757	6.36E-05
ENSG00000188779.10	SKOR1	0.629003668	1.813331173	0.01537
ENSG00000144136.10	SLC20A1	0.628725082	10.84136702	1.44E-11

ENSG00000196267.12	ZNF836	0.628655923	5.955888233	1.11E-06
ENSG00000122035.6	RASL11A	0.628597153	3.604079648	0.000249
ENSG00000198598.6	MMP17	0.62850836	1.451920151	0.035325
ENSG00000187605.15	TET3	0.62845596	7.038829119	9.14E-08
ENSG00000283803.1	MIR3198-2	0.628374847	2.79108545	0.001618
ENSG00000143498.17	TAF1A	0.627376623	4.332507327	4.65E-05
ENSG00000272508.1	AL136982.6	0.626635331	1.577559221	0.026451
ENSG00000232767.1	AC016825.1	0.626525242	2.882798109	0.00131
ENSG00000183161.5	FANCF	0.625328242	4.771925334	1.69E-05
ENSG00000197568.13	HHLA3	0.6250726	2.284402513	0.005195
ENSG00000163453.11	IGFBP7	0.625006084	4.192067272	6.43E-05
ENSG00000167394.12	ZNF668	0.624948415	8.105804854	7.84E-09
ENSG00000284779.1	IGF2	0.624883774	2.275259099	0.005306
ENSG00000172803.17	SNX32	0.624726151	11.89532401	1.27E-12
ENSG00000179403.11	VWA1	0.624460263	5.816852419	1.52E-06
ENSG00000172757.12	CFL1	0.624371119	11.2761895	5.29E-12
ENSG00000187479.6	C11orf96	0.624348308	2.305878545	0.004944
ENSG00000164038.14	SLC9B2	0.624139742	4.091387101	8.10E-05
ENSG00000233429.9	HOTAIRM1	0.623906407	2.83122376	0.001475
ENSG00000106125.14	MINDY4	0.623871392	3.350579319	0.000446
ENSG00000126458.3	RRAS	0.623468707	5.496786557	3.19E-06
ENSG00000175130.6	MARCKSL1	0.623294384	4.790665673	1.62E-05
ENSG00000262468.6	LINC01569	0.62311701	2.744305616	0.001802
ENSG00000196659.9	TTC30B	0.623078827	1.463253458	0.034415
ENSG00000108106.13	UBE2S	0.622979766	3.695915373	0.000201
ENSG00000117318.8	ID3	0.622904568	4.107642931	7.80E-05
ENSG00000232748.3	AC135050.1	0.622833715	6.922004399	1.20E-07
ENSG00000183397.5	C19orf71	0.622716022	4.544105028	2.86E-05
ENSG00000143545.8	RAB13	0.622599086	6.104195687	7.87E-07
ENSG00000167244.19	IGF2	0.622297139	2.25080654	0.005613
ENSG00000169855.19	ROBO1	0.622174336	4.717213208	1.92E-05
ENSG00000166669.13	ATF7IP2	0.62207634	4.176561495	6.66E-05
ENSG00000237440.8	ZNF737	0.621953095	3.833970488	0.000147
ENSG00000184840.11	TMED9	0.620736197	15.18006783	6.61E-16
ENSG00000109452.12	INPP4B	0.61966519	3.697788274	0.000201
ENSG00000134954.14	ETS1	0.619347717	7.694595397	2.02E-08
ENSG00000018236.14	CNTN1	0.618848589	1.594096585	0.025463
ENSG00000138685.15	FGF2	0.618308865	4.927422663	1.18E-05
ENSG00000196418.12	ZNF124	0.618036316	2.67895898	0.002094
ENSG00000154127.9	UBASH3B	0.617918291	2.235369993	0.005816
ENSG00000197442.9	MAP3K5	0.61788273	5.231281661	5.87E-06
ENSG00000177105.9	RHOG	0.617690751	6.369915871	4.27E-07
ENSG00000224645.1	AL391069.1	0.617657458	3.586181629	0.000259
ENSG00000101367.8	MAPRE1	0.617369935	17.73694746	1.83E-18
ENSG00000234741.7	GAS5	0.617336895	5.14962346	7.09E-06
ENSG00000178904.18	DPY19L3	0.617273041	4.835789574	1.46E-05
ENSG00000141837.19	CACNA1A	0.617227175	2.573261323	0.002671

ENSG00000134308.13	YWHAQ		0.617148183	8.574385568	2.66E-09
ENSG00000163947.11	ARHGEF3		0.616794346	6.91184578	1.23E-07
ENSG00000279227.1	AC009303.4		0.61675307	1.55120522	0.028106
ENSG00000140950.15	TLDC1		0.616309741	7.766023525	1.71E-08
ENSG00000284146.1	MIR922		0.61621074	1.839247645	0.014479
ENSG00000261427.6	AC099518.3		0.615332973	3.653308475	0.000222
ENSG00000205352.10	PRR13		0.615294941	8.450529445	3.54E-09
ENSG00000182534.13	MXRA7		0.615244956	4.483285875	3.29E-05
ENSG00000138111.14	MFSD13A		0.614937477	3.760314245	0.000174
ENSG00000074621.13	SLC24A1		0.614774935	6.472312231	3.37E-07
ENSG00000133121.20	STARD13		0.61475804	7.527229721	2.97E-08
ENSG00000144847.12	IGSF11		0.613795708	2.084546841	0.008231
ENSG00000204856.11	FAM216A		0.613699044	5.871331669	1.34E-06
ENSG00000205078.5	SYCE1L		0.613645179	4.013606936	9.69E-05
ENSG00000263069.5	AC124319.2		0.613482813	7.767757899	1.71E-08
ENSG00000114446.4	IFT57		0.613326817	5.085145521	8.22E-06
ENSG00000173486.12	FKBP2		0.613203623	6.915413163	1.22E-07
ENSG00000284829.1	AL662884.2		0.613120233	6.314997788	4.84E-07
ENSG00000163872.15	YEATS2		0.613040906	12.02316524	9.48E-13
ENSG00000133657.15	ATP13A3		0.612963327	8.969015418	1.07E-09
ENSG00000184702.19		5-Sep	0.612859338	3.505266899	0.000312
ENSG00000159733.13	ZFYVE28		0.612428505	5.049232562	8.93E-06
ENSG00000006210.6	CX3CL1		0.612035459	3.146056932	0.000714
ENSG00000131389.17	SLC6A6		0.611799899	6.073185948	8.45E-07
ENSG00000108387.14		4-Sep	0.611457127	5.314062087	4.85E-06
ENSG00000178809.11	TRIM73		0.611378336	2.23663374	0.005799
ENSG00000228343.1	AC115618.2		0.610981539	4.119081894	7.60E-05
ENSG00000280254.1	AC233723.2		0.610696578	3.430124533	0.000371
ENSG00000112029.9	FBXO5		0.610438973	3.925455804	0.000119
ENSG00000084731.14	KIF3C		0.610115471	5.691869775	2.03E-06
ENSG00000266820.1	KPNA2P3		0.609627233	3.988653765	0.000103
ENSG00000198682.12	PAPSS2		0.609504097	2.144462523	0.00717
ENSG00000176148.15	TCP11L1		0.609464969	11.41739718	3.82E-12
ENSG00000142408.4	CACNG8		0.60943632	1.735104303	0.018403
ENSG00000104626.14	ERI1		0.609321707	5.978088095	1.05E-06
ENSG00000160867.14	FGFR4		0.608919991	2.008239038	0.009812
ENSG00000117724.12	CENPF		0.608812287	2.293152858	0.005092
ENSG00000197608.11	ZNF841		0.608084375	5.344946905	4.52E-06
ENSG00000158122.11	PRXL2C		0.607913425	7.302806803	4.98E-08
ENSG00000187189.10	TSPYL4		0.607910815	9.546475733	2.84E-10
ENSG00000127124.15	HIVEP3		0.607893735	6.173224895	6.71E-07
ENSG00000126838.9	PZP		0.607589615	1.529103438	0.029573
ENSG00000181135.15	ZNF707		0.607082372	9.640278917	2.29E-10
ENSG00000161618.9	ALDH16A1		0.606437451	7.433937415	3.68E-08
ENSG00000234684.6	SDCBP2-AS1		0.605770526	6.905296573	1.24E-07
ENSG00000105137.12	SYDE1		0.605173539	3.366305978	0.00043
ENSG00000197872.11	FAM49A		0.605120339	6.285447065	5.18E-07

ENSG00000182197.11	EXT1	0.605100616	5.116668741	7.64E-06
ENSG00000249786.7	EAF1-AS1	0.604736288	5.013304747	9.70E-06
ENSG00000285304.1	Z83844.3	0.604505385	5.75168404	1.77E-06
ENSG00000273136.6	NBPF26	0.603950251	3.841823827	0.000144
ENSG00000135709.12	KIAA0513	0.603930544	3.959626049	0.00011
ENSG00000116729.13	WLS	0.603899469	6.626545653	2.36E-07
ENSG00000145545.11	SRD5A1	0.603801915	10.17311657	6.71E-11
ENSG00000116962.14	NID1	0.603477285	1.957651995	0.011024
ENSG00000197933.12	ZNF823	0.603476639	5.24506386	5.69E-06
ENSG00000090054.14	SPTLC1	0.603200311	6.014194187	9.68E-07
ENSG00000268713.1	AC005261.3	0.603145491	1.876403126	0.013292
ENSG00000106025.8	TSPAN12	0.603135044	3.532985837	0.000293
ENSG00000240211.1	AC092849.1	0.602828533	4.563367502	2.73E-05
ENSG00000157800.17	SLC37A3	0.602657738	3.742284551	0.000181
ENSG00000177721.4	ANXA2R	0.60261058	6.150924187	7.06E-07
ENSG00000245105.3	A2M-AS1	0.602425608	4.991585085	1.02E-05
ENSG00000181885.18	CLDN7	0.602127747	2.066852872	0.008573
ENSG00000285991.1	AL355312.5	0.602044173	4.142184946	7.21E-05
ENSG00000168701.18	TMEM208	0.60187226	5.788711397	1.63E-06
ENSG00000196482.16	ESRRG	0.601791473	1.71677336	0.019197
ENSG00000138101.18	DTNB	0.601632283	4.620846561	2.39E-05
ENSG00000000003.14	TSPAN6	0.601126939	3.244593482	0.000569
ENSG00000198780.11	FAM169A	0.600814531	1.37740239	0.041937
ENSG00000285427.1	SOD2-OT1	0.600549473	3.599337181	0.000252
ENSG00000271447.5	MMP28	0.599855004	2.378437347	0.004184
ENSG00000106853.19	PTGR1	0.599853482	4.195891377	6.37E-05
ENSG00000204713.10	TRIM27	0.599769987	16.75500742	1.76E-17
ENSG00000274425.1	AC114271.1	0.599754259	4.54674501	2.84E-05
ENSG00000158691.14	ZSCAN12	0.599623233	3.839977716	0.000145
ENSG00000163946.13	FAM208A	0.599564657	15.58172533	2.62E-16
ENSG00000165449.11	SLC16A9	0.599444346	2.159395589	0.006928
ENSG00000175106.16	TVP23C	0.599372176	5.269960149	5.37E-06
ENSG00000058668.14	ATP2B4	0.599267763	7.315561786	4.84E-08
ENSG00000130508.10	PXDN	0.599083045	2.907377401	0.001238
ENSG00000137331.11	IER3	0.598902177	3.871378689	0.000134
ENSG00000106080.10	FKBP14	0.598832363	5.266220988	5.42E-06
ENSG00000151892.14	GFRA1	0.598159757	2.11801922	0.00762
ENSG00000240089.2	BMS1P3	0.597913783	1.532154983	0.029366
ENSG00000213203.2	GIMAP1	0.597418917	4.219910683	6.03E-05
ENSG00000254528.7	AP000757.1	0.597146964	3.242443276	0.000572
ENSG00000176390.11	CRLF3	0.59654895	6.909906066	1.23E-07
ENSG00000268970.1	AC022150.2	0.596483976	2.043162405	0.009054
ENSG00000110888.17	CAPRIN2	0.59633296	4.383533532	4.13E-05
ENSG00000224281.4	SLC25A5-AS1	0.596090308	11.8157149	1.53E-12
ENSG00000284753.1	EEF1AKMT4	0.595970128	3.43992966	0.000363
ENSG00000281005.1	LINC00921	0.595825783	2.335471391	0.004619
ENSG00000131591.17	C1orf159	0.595687059	5.505283575	3.12E-06

ENSG00000110931.18	CAMKK2	0.595342642	7.519417903	3.02E-08
ENSG00000117139.17	KDM5B	0.595078944	8.53162393	2.94E-09
ENSG00000164136.16	IL15	0.594674964	3.432198955	0.00037
ENSG00000274422.1	AC245060.5	0.594537642	2.206194701	0.00622
ENSG00000221829.9	FANCG	0.594481915	7.464960563	3.43E-08
ENSG00000171502.14	COL24A1	0.594270222	1.424842637	0.037597
ENSG00000174799.10	CEP135	0.594192736	4.598604156	2.52E-05
ENSG00000085831.15	TTC39A	0.593290648	2.58859145	0.002579
ENSG00000175183.9	CSRP2	0.592517254	3.023007914	0.000948
ENSG00000164938.13	TP53INP1	0.591989651	3.96144003	0.000109
ENSG00000143390.17	RFX5	0.591932231	4.982142277	1.04E-05
ENSG00000248356.1	AC093864.1	0.591917854	1.470141911	0.033873
ENSG00000185869.14	ZNF829	0.591883669	2.728296181	0.001869
ENSG00000171862.10	PTEN	0.59173661	9.301931554	4.99E-10
ENSG00000255343.1	AC234917.1	0.591701585	2.128213359	0.007444
ENSG00000272160.1	RNU4-5P	0.59166723	11.9122091	1.22E-12
ENSG00000139220.16	PPFIA2	0.591541772	1.559694863	0.027562
ENSG00000131831.17	RAI2	0.591535898	3.338936687	0.000458
ENSG00000255248.8	MIR100HG	0.591510185	3.701561232	0.000199
ENSG00000257181.1	AC025423.4	0.590807136	4.903478671	1.25E-05
ENSG00000166822.12	TMEM170A	0.590410764	7.815595196	1.53E-08
ENSG00000119977.20	TCTN3	0.590114531	6.882032279	1.31E-07
ENSG00000168246.5	UBTD2	0.58993683	10.01612766	9.64E-11
ENSG00000061337.15	LZTS1	0.5897917	2.825766705	0.001494
ENSG00000101695.8	RNF125	0.589528598	4.316006081	4.83E-05
ENSG00000258663.1	AL117190.2	0.58939346	2.836560987	0.001457
ENSG00000139926.15	FRMD6	0.588506698	2.997233278	0.001006
ENSG00000124224.16	PPP4R1L	0.5881016	5.224340089	5.97E-06
ENSG00000111961.17	SASH1	0.587854607	4.421289067	3.79E-05
ENSG00000178301.3	AQP11	0.587742078	1.678637306	0.020959
ENSG00000260257.2	AL035071.1	0.587631423	3.595853006	0.000254
ENSG00000163053.10	SLC16A14	0.587628902	2.07391509	0.008435
ENSG00000120742.10	SERP1	0.587367036	6.847294456	1.42E-07
ENSG00000111602.11	TIMELESS	0.587299397	7.56330364	2.73E-08
ENSG00000213462.4	ERV3-1	0.587239079	2.899009939	0.001262
ENSG00000171159.4	C9orf16	0.587192759	6.635779691	2.31E-07
ENSG00000263956.6	NBPF11	0.586745239	8.28379858	5.20E-09
ENSG00000163909.7	HEYL	0.586679384	2.616680341	0.002417
ENSG00000205084.10	TMEM231	0.586311215	4.548178104	2.83E-05
ENSG00000121316.10	PLBD1	0.58626333	3.063722864	0.000864
ENSG00000257038.1	AP002761.3	0.586044596	3.117430136	0.000763
ENSG00000261371.5	PECAM1	0.58603896	3.663548162	0.000217
ENSG00000180776.15	ZDHHC20	0.585361478	5.779840108	1.66E-06
ENSG00000214575.9	CPEB1	0.585234347	1.347618819	0.044914
ENSG00000116985.11	BMP8B	0.584998152	1.703827338	0.019778
ENSG00000058600.15	POLR3E	-0.585036735	10.88989629	1.29E-11
ENSG00000273372.1	SFTPD-AS1	-0.585067398	1.415281427	0.038434

ENSG00000228801.5	AC064807.1	-0.585719092	3.014176091	0.000968
ENSG00000252690.3	AC105339.2	-0.586098577	5.596526481	2.53E-06
ENSG00000285589.1	AC010422.8	-0.586485549	27.41687192	3.83E-28
ENSG00000264501.2	RN7SL731P	-0.586683253	3.032292977	0.000928
ENSG00000281207.1	SLFNL1-AS1	-0.586776446	9.420979473	3.79E-10
ENSG00000136478.7	TEX2	-0.586847926	7.047423554	8.97E-08
ENSG00000147687.18	TATDN1	-0.586901474	5.695832202	2.01E-06
ENSG00000036257.12	CUL3	-0.58744393	7.838936586	1.45E-08
ENSG00000065989.15	PDE4A	-0.587720597	5.807647217	1.56E-06
ENSG00000262429.1	AC004771.4	-0.587998337	11.51881446	3.03E-12
ENSG00000276965.1	RF01891	-0.588182304	3.64991696	0.000224
ENSG00000260272.1	AC093525.2	-0.588286242	15.17267719	6.72E-16
ENSG00000003249.13	DBNDD1	-0.588980768	1.522160624	0.03005
ENSG00000110700.6	RPS13	-0.589050487	8.822708439	1.50E-09
ENSG00000135108.14	FBXO21	-0.589195397	7.122374576	7.54E-08
ENSG00000105520.10	PLPPR2	-0.589340094	5.567555691	2.71E-06
ENSG00000185633.10	NDUFA4L2	-0.589557921	5.871377959	1.34E-06
ENSG00000250748.6	AC025419.1	-0.589569983	3.881018981	0.000132
ENSG00000283764.1	MIR6850	-0.589604073	5.682391638	2.08E-06
ENSG00000143318.12	CASQ1	-0.589966566	1.83854228	0.014503
ENSG00000154122.13	ANKH	-0.590491017	7.843547979	1.43E-08
ENSG00000117479.13	SLC19A2	-0.590496948	2.024918227	0.009442
ENSG00000146707.14	POMZP3	-0.590668906	2.850507469	0.001411
ENSG00000129675.15	ARHGEF6	-0.590715073	5.572121675	2.68E-06
ENSG00000196683.10	TOMM7	-0.590829929	6.572904974	2.67E-07
ENSG00000196878.14	LAMB3	-0.590964162	3.463897557	0.000344
ENSG00000164715.5	LMTK2	-0.590991702	6.030347213	9.33E-07
ENSG00000171385.9	KCND3	-0.591081995	3.279330038	0.000526
ENSG00000175662.17	TOM1L2	-0.591454366	5.997082712	1.01E-06
ENSG00000134769.21	DTNA	-0.591551268	3.480705129	0.000331
ENSG00000228672.3	PROB1	-0.591634402	2.097539457	0.007988
ENSG00000116688.16	MFN2	-0.591673178	3.571645653	0.000268
ENSG00000178127.12	NDUFV2	-0.591687538	5.400907759	3.97E-06
ENSG00000184983.10	NDUFA6	-0.591878482	5.459100425	3.47E-06
ENSG00000093100.13	AC016026.1	-0.591966155	6.133191602	7.36E-07
ENSG00000273432.1	AC004951.4	-0.592044504	9.022618611	9.49E-10
ENSG00000138095.18	LRPPRC	-0.592845655	5.677681337	2.10E-06
ENSG00000198718.12	TOGARAM1	-0.59306079	8.920815618	1.20E-09
ENSG00000143156.13	NME7	-0.593166702	5.321803126	4.77E-06
ENSG00000139350.11	NEDD1	-0.59331021	3.586351528	0.000259
ENSG00000230534.6	AL392046.1	-0.593391852	3.842929777	0.000144
ENSG00000130559.18	CAMSAP1	-0.594506111	4.982135049	1.04E-05
ENSG00000133687.15	TMTC1	-0.595009888	6.541475979	2.87E-07
ENSG00000159267.14	HLCS	-0.595256333	8.906506842	1.24E-09
ENSG00000185104.19	FAF1	-0.595306524	7.661203001	2.18E-08
ENSG00000142197.12	DOP1B	-0.5953716	5.064409994	8.62E-06
ENSG00000250251.6	PKD1P6	-0.595694629	6.145638313	7.15E-07

ENSG00000113448.18	PDE4D		-0.595829936	3.278667761	0.000526
ENSG00000132563.15	REEP2		-0.596058088	2.612430688	0.002441
ENSG00000150281.6	CTF1		-0.596466345	5.465914424	3.42E-06
ENSG00000008282.8	SYPL1		-0.596867922	18.8557839	1.39E-19
ENSG00000117791.15		2-Mar	-0.596893462	7.96783866	1.08E-08
ENSG00000144749.13	LRIG1		-0.597008376	6.233013377	5.85E-07
ENSG00000108176.14	DNAJC12		-0.597131995	1.809513392	0.015506
ENSG00000267426.5	AC087289.3		-0.597576832	10.8995491	1.26E-11
ENSG00000155265.10	GOLGA7B		-0.597989935	2.265552005	0.005426
ENSG00000065150.18	IPO5		-0.598283188	9.170130048	6.76E-10
ENSG00000169047.5	IRS1		-0.598327579	2.881645796	0.001313
ENSG00000215440.11	NPEPL1		-0.598414511	8.681737317	2.08E-09
ENSG00000215041.9	NEURL4		-0.598455463	10.54422852	2.86E-11
ENSG00000075702.17	WDR62		-0.598514102	1.775351241	0.016774
ENSG00000266931.2	AC092651.2		-0.598813655	4.061718864	8.68E-05
ENSG00000167674.14	HDGFL2		-0.599028971	14.19189602	6.43E-15
ENSG00000163833.7	FBXO40		-0.599351279	1.695205304	0.020174
ENSG00000118181.10	RPS25		-0.600040584	7.790472712	1.62E-08
ENSG00000285803.1	AL442003.1		-0.600101768	5.837149977	1.45E-06
ENSG00000100412.15	ACO2		-0.600170797	3.437584319	0.000365
ENSG00000198168.8	SVIP		-0.600187992	4.979402719	1.05E-05
ENSG00000225614.2	ZNF469		-0.600262456	2.615055685	0.002426
ENSG00000276550.4	HERC2P2		-0.600376287	3.288141319	0.000515
ENSG00000261168.1	AL592424.1		-0.600450357	2.146523016	0.007136
ENSG00000274068.1	AL449266.1		-0.600500327	6.836377907	1.46E-07
ENSG00000068024.16	HDAC4		-0.601010468	6.712556986	1.94E-07
ENSG00000114302.15	PRKAR2A		-0.601434314	5.220448097	6.02E-06
ENSG00000260111.1	AC012184.1		-0.601472606	4.639344991	2.29E-05
ENSG00000103423.13	DNAJA3		-0.601748794	6.20009784	6.31E-07
ENSG00000227591.5	AL031316.1		-0.601864926	2.63523103	0.002316
ENSG00000129682.15	FGF13		-0.601866782	6.39406945	4.04E-07
ENSG00000172336.4	POP7		-0.601872891	18.59489021	2.54E-19
ENSG00000185883.11	ATP6V0C		-0.602217762	14.79908014	1.59E-15
ENSG00000162413.16	KLHL21		-0.602255653	4.382999008	4.14E-05
ENSG00000253741.1	LNCOC1		-0.602448285	3.170822943	0.000675
ENSG00000241316.7	SUCLG2-AS1		-0.602449263	3.254167835	0.000557
ENSG00000141664.9	ZCCHC2		-0.602533793	7.309427698	4.90E-08
ENSG00000008710.19	PKD1		-0.602627216	7.508125748	3.10E-08
ENSG00000163064.6	EN1		-0.602897904	3.082441341	0.000827
ENSG00000176407.17	KCMF1		-0.602920454	7.069724946	8.52E-08
ENSG00000106771.12	TMEM245		-0.602943187	10.07236078	8.47E-11
ENSG00000269068.1	AC009955.4		-0.603097877	4.847866644	1.42E-05
ENSG00000151729.10	SLC25A4		-0.603195113	2.09513435	0.008033
ENSG00000146147.14	MLIP		-0.603377451	2.45034374	0.003545
ENSG00000136206.3	SPDYE1		-0.603549855	1.576477983	0.026517
ENSG00000197312.11	DDI2		-0.603601066	8.187283094	6.50E-09
ENSG00000129152.3	MYOD1		-0.603677849	1.667443969	0.021506

ENSG00000062485.18	CS	-0.603919607	4.441492363	3.62E-05
ENSG00000137177.19	KIF13A	-0.603936083	5.567012567	2.71E-06
ENSG00000243156.7	MICAL3	-0.604265212	7.044074042	9.03E-08
ENSG00000232640.1	AL354892.2	-0.604267317	2.53988244	0.002885
ENSG00000139044.11	B4GALNT3	-0.604411086	1.86524947	0.013638
ENSG00000157881.13	PANK4	-0.60441524	7.422922619	3.78E-08
ENSG00000161664.6	ASB16	-0.604450659	2.646383034	0.002257
ENSG00000101665.9	SMAD7	-0.604484137	4.051147002	8.89E-05
ENSG00000110200.8	ANAPC15	-0.604752268	8.224130313	5.97E-09
ENSG00000277247.1	AC083809.1	-0.604799893	3.116162631	0.000765
ENSG00000205309.13	NT5M	-0.605024006	3.643361671	0.000227
ENSG00000275665.1	AC006449.3	-0.605025158	6.428775323	3.73E-07
ENSG00000140548.9	ZNF710	-0.605114891	3.254855629	0.000556
ENSG00000153956.15	CACNA2D1	-0.60528381	3.138393953	0.000727
ENSG00000267342.1	AC087289.2	-0.605530861	9.255145323	5.56E-10
ENSG00000119471.14	HSDL2	-0.60572572	3.697325539	0.000201
ENSG00000071205.11	ARHGAP10	-0.605737158	11.98590969	1.03E-12
ENSG00000100181.22	TPTEP1	-0.605738571	4.17060888	6.75E-05
ENSG00000121310.16	ECHDC2	-0.60618231	6.756960933	1.75E-07
ENSG00000051620.10	HEBP2	-0.606215591	12.10160135	7.91E-13
ENSG00000169564.6	PCBP1	-0.606317009	11.08127896	8.29E-12
ENSG00000272269.1	AL138724.1	-0.606391735	7.200693654	6.30E-08
ENSG00000029725.16	RABEP1	-0.606455069	9.039207739	9.14E-10
ENSG00000086015.20	MAST2	-0.606768433	5.213778474	6.11E-06
ENSG00000100664.10	EIF5	-0.607256145	14.85545981	1.39E-15
ENSG00000175701.10	MTLN	-0.607346416	3.00915371	0.000979
ENSG00000130811.11	EIF3G	-0.607398707	11.58895755	2.58E-12
ENSG00000255164.1	AF235103.1	-0.608173401	1.568773897	0.026991
ENSG00000115268.9	RPS15	-0.608269744	10.70469786	1.97E-11
ENSG00000251569.1	AC093899.2	-0.608359815	2.480800133	0.003305
ENSG00000210112.1	MT-TM	-0.608414586	1.667847582	0.021486
ENSG00000136603.13	SKIL	-0.608677393	4.993612496	1.01E-05
ENSG00000272679.1	AL355987.2	-0.608941277	5.165373844	6.83E-06
ENSG00000274859.1	AC131238.1	-0.609217471	3.904822237	0.000125
ENSG00000100036.12	SLC35E4	-0.609224261	6.102253916	7.90E-07
ENSG00000012211.12	PRICKLE3	-0.609710382	8.131972715	7.38E-09
ENSG00000112294.12	ALDH5A1	-0.609850095	2.870589596	0.001347
ENSG00000210049.1	MT-TF	-0.610315784	2.863841448	0.001368
ENSG00000148842.17	CNNM2	-0.61039083	9.329137759	4.69E-10
ENSG00000104946.12	TBC1D17	-0.610944129	10.02269806	9.49E-11
ENSG00000168710.17	AHCYL1	-0.610964846	14.60554934	2.48E-15
ENSG00000119912.16	IDE	-0.61098905	5.865292006	1.36E-06
ENSG00000179766.19	ATP8B5P	-0.611038024	1.325616986	0.047248
ENSG00000204390.9	HSPA1L	-0.611096132	4.290959551	5.12E-05
ENSG00000273275.1	AC017083.2	-0.611368539	8.911732578	1.23E-09
ENSG00000257337.6	AC068888.1	-0.611434798	11.74814194	1.79E-12
ENSG00000131153.8	GINS2	-0.611527852	4.466542522	3.42E-05

ENSG00000136842.13	TMOD1	-0.61168901	4.387306373	4.10E-05
ENSG00000127241.16	MASP1	-0.611925202	1.604429234	0.024864
ENSG00000156381.8	ANKRD9	-0.612293925	3.58980278	0.000257
ENSG00000232656.7	IDI2-AS1	-0.612331428	1.840724193	0.01443
ENSG00000116604.17	MEF2D	-0.612342368	5.933050695	1.17E-06
ENSG00000117625.13	RCOR3	-0.612524725	14.60055942	2.51E-15
ENSG00000239474.6	KLHL41	-0.61295814	2.458975981	0.003476
ENSG00000129245.11	FXR2	-0.61322645	5.24575273	5.68E-06
ENSG00000170871.11	KIAA0232	-0.613255153	6.673582105	2.12E-07
ENSG00000141744.3	PNMT	-0.613304308	2.676861768	0.002104
ENSG00000114416.17	FXR1	-0.613547692	4.844748393	1.43E-05
ENSG00000151623.14	NR3C2	-0.613859993	5.739813627	1.82E-06
ENSG00000102178.12	UBL4A	-0.613986884	7.084822742	8.23E-08
ENSG00000167774.2	AC010323.1	-0.614734867	7.020923468	9.53E-08
ENSG00000198837.9	DENND4B	-0.61486631	6.927751504	1.18E-07
ENSG00000102755.11	FLT1	-0.614935262	4.857684005	1.39E-05
ENSG00000274776.1	AC090241.3	-0.614954713	1.387899078	0.040936
ENSG00000256364.1	AC069234.2	-0.615045914	4.833524733	1.47E-05
ENSG00000171443.6	ZNF524	-0.615249565	13.5609965	2.75E-14
ENSG00000143499.13	SMYD2	-0.615584665	6.835051025	1.46E-07
ENSG00000168427.8	KLHL30	-0.616472082	2.23579434	0.00581
ENSG00000199053.3	MIR324	-0.61670662	2.8982228	0.001264
ENSG00000170906.15	NDUFA3	-0.616797806	7.021680097	9.51E-08
ENSG00000198515.13	CNGA1	-0.616801062	1.943489744	0.01139
ENSG00000063245.14	EPN1	-0.616887982	13.02741719	9.39E-14
ENSG00000111231.8	GPN3	-0.617150449	13.07599621	8.39E-14
ENSG00000215021.8	PHB2	-0.61734993	13.21564919	6.09E-14
ENSG00000127418.14	FGFRL1	-0.617705737	4.966228301	1.08E-05
ENSG00000241326.1	AL603962.1	-0.617851884	4.134787428	7.33E-05
ENSG00000011201.11	ANOS1	-0.617922656	4.087444963	8.18E-05
ENSG00000242612.6	DECR2	-0.617927086	8.917213783	1.21E-09
ENSG00000145332.13	KLHL8	-0.618029549	9.162684062	6.88E-10
ENSG00000105879.11	CBLL1	-0.6180299	13.75244757	1.77E-14
ENSG00000237174.7	AL445465.1	-0.618033663	3.041015938	0.00091
ENSG00000171813.13	PWWP2B	-0.618427757	4.026603963	9.41E-05
ENSG00000280434.1	AL031595.3	-0.618494549	7.320745129	4.78E-08
ENSG00000137804.12	NUSAP1	-0.618834029	5.458525481	3.48E-06
ENSG00000145494.11	NDUFS6	-0.618886484	7.698790463	2.00E-08
ENSG00000256928.1	AP000763.4	-0.61899439	3.08940772	0.000814
ENSG00000185641.6	AC034236.1	-0.619562606	4.473187646	3.36E-05
ENSG00000156232.7	WHAMM	-0.619829817	13.29350384	5.09E-14
ENSG00000267952.1	AC008878.1	-0.619855492	6.80159099	1.58E-07
ENSG00000260017.1	AC138811.1	-0.619893262	3.493633322	0.000321
ENSG00000005882.11	PDK2	-0.620047871	3.953274667	0.000111
ENSG00000177731.15	FLII	-0.620682024	6.406746036	3.92E-07
ENSG00000168255.20	POLR2J3	-0.6209009	4.506985269	3.11E-05
ENSG00000138376.10	BARD1	-0.620987416	5.49147437	3.22E-06

ENSG00000166971.16	AKTIP	-0.621013815	14.95974539	1.10E-15
ENSG00000250938.5	AC108866.1	-0.621581947	3.398237092	0.0004
ENSG00000265399.1	AP005329.2	-0.621635242	2.209473871	0.006173
ENSG00000260097.2	SPDYE6	-0.621693453	2.066595717	0.008578
ENSG00000197576.13	HOXA4	-0.621864653	3.021539804	0.000952
ENSG00000272434.1	AC137630.3	-0.621902339	8.682967088	2.08E-09
ENSG00000265750.1	AC090772.3	-0.621954997	7.268154272	5.39E-08
ENSG00000108509.20	CAMTA2	-0.62202809	12.7123539	1.94E-13
ENSG00000131055.4	COX4I2	-0.622213209	2.0378391	0.009166
ENSG00000138796.16	HADH	-0.622328833	5.090141328	8.13E-06
ENSG00000217930.7	PAM16	-0.622354382	10.2521555	5.60E-11
ENSG00000250012.1	AC079848.1	-0.622369739	3.269253327	0.000538
ENSG00000224490.5	TTC21B-AS1	-0.622475665	2.349186513	0.004475
ENSG00000169599.12	NFU1	-0.622668249	9.885703716	1.30E-10
ENSG00000175756.13	AURKAIP1	-0.622807687	7.776596513	1.67E-08
ENSG00000285539.1	AC006059.3	-0.623008986	2.284313945	0.005196
ENSG00000130024.14	PHF10	-0.623011101	12.93839805	1.15E-13
ENSG00000126267.9	COX6B1	-0.623062924	5.866319955	1.36E-06
ENSG00000144674.16	GOLGA4	-0.623544529	5.224992339	5.96E-06
ENSG00000107929.14	LARP4B	-0.623793104	16.29163298	5.11E-17
ENSG00000261167.1	AC107027.3	-0.623964205	5.259102892	5.51E-06
ENSG00000159069.13	FBXW5	-0.62415837	9.074566226	8.42E-10
ENSG00000184863.10	RBM33	-0.624245452	11.94801611	1.13E-12
ENSG00000175536.6	LIPT2	-0.62453482	4.252600374	5.59E-05
ENSG00000147654.14	EBAG9	-0.62465629	16.0684397	8.54E-17
ENSG00000234814.8	SVIL2P	-0.624703119	1.533734253	0.029259
ENSG00000104960.15	PTOV1	-0.624805078	17.83548649	1.46E-18
ENSG00000175879.8	HOXD8	-0.624832871	1.900859379	0.012564
ENSG00000184281.14	TSSC4	-0.624872567	16.85639552	1.39E-17
ENSG00000184220.11	CMSS1	-0.625151308	7.302067863	4.99E-08
ENSG00000284738.1	AL358472.5	-0.625253835	5.898585194	1.26E-06
ENSG00000171766.15	GATM	-0.62545119	4.021674708	9.51E-05
ENSG00000076984.17	MAP2K7	-0.62554777	9.905006461	1.24E-10
ENSG00000188677.14	PARVB	-0.625770217	5.993866322	1.01E-06
ENSG00000218175.2	AC016739.1	-0.625826106	4.284031222	5.20E-05
ENSG00000278202.1	AC243919.1	-0.625934059	3.326355683	0.000472
ENSG00000259863.1	SH3RF3-AS1	-0.626142847	1.808363935	0.015547
ENSG00000233762.3	AC007969.1	-0.626166985	3.76102465	0.000173
ENSG00000100908.13	EMC9	-0.626187109	7.099313454	7.96E-08
ENSG00000233426.3	EIF3FP3	-0.626346069	1.312507105	0.048696
ENSG00000136149.6	RPL13AP25	-0.627418474	1.326124546	0.047193
ENSG00000176225.13	RTTN	-0.627428593	6.050754405	8.90E-07
ENSG00000165626.17	BEND7	-0.627582769	11.23823389	5.78E-12
ENSG00000197756.9	RPL37A	-0.627590493	9.000490285	9.99E-10
ENSG00000198816.6	ZNF358	-0.627968444	7.473196454	3.36E-08
ENSG00000131196.17	NFATC1	-0.627987368	6.935085735	1.16E-07
ENSG00000239486.1	AC091390.3	-0.628197427	2.741589536	0.001813

ENSG00000178397.12	FAM220A	-0.628462373	4.408204555	3.91E-05
ENSG00000116761.11	CTH	-0.628653919	3.095043609	0.000803
ENSG00000152078.9	TMEM56	-0.628725695	3.168209532	0.000679
ENSG00000118689.14	FOXO3	-0.628899394	3.364856069	0.000432
ENSG00000143028.8	SYPL2	-0.628927668	2.36120062	0.004353
ENSG00000126003.6	PLAGL2	-0.628952525	7.238215664	5.78E-08
ENSG00000140988.15	RPS2	-0.629585196	12.96986263	1.07E-13
ENSG00000285471.1	AC007846.2	-0.629998318	1.957744869	0.011022
ENSG00000214783.9	POLR2J4	-0.630171569	9.701779357	1.99E-10
ENSG00000232611.1	AL683813.1	-0.630252085	5.810914769	1.55E-06
ENSG00000165028.11	NIPSNAP3B	-0.630274303	3.424909733	0.000376
ENSG00000261532.1	AC009065.7	-0.630862833	6.271346377	5.35E-07
ENSG00000131061.13	ZNF341	-0.631078538	7.5884298	2.58E-08
ENSG00000151240.16	DIP2C	-0.631098999	6.564713708	2.72E-07
ENSG00000173641.17	HSPB7	-0.631178832	2.192723113	0.006416
ENSG00000186432.8	KPNA4	-0.63122188	4.539841378	2.89E-05
ENSG00000185532.16	PRKG1	-0.631661323	3.109164786	0.000778
ENSG00000103067.13	ESRP2	-0.63206787	6.064293788	8.62E-07
ENSG00000165238.16	WNK2	-0.632343186	1.79544708	0.016016
ENSG00000117640.17	MTFR1L	-0.632469799	6.322565924	4.76E-07
ENSG00000203506.5	RBMS3-AS2	-0.63301849	1.534097358	0.029235
ENSG00000169727.12	GPS1	-0.633209169	16.26121885	5.48E-17
ENSG00000010282.14	HHATL	-0.633224369	2.325789323	0.004723
ENSG00000130433.7	CACNG6	-0.633366067	2.156854802	0.006969
ENSG00000163517.14	HDAC11	-0.633431318	4.281846325	5.23E-05
ENSG00000179242.15	CDH4	-0.63393526	4.640037574	2.29E-05
ENSG00000205213.13	LGR4	-0.634150139	6.459146814	3.47E-07
ENSG00000101605.12	MYOM1	-0.634320529	2.09112447	0.008107
ENSG00000136848.17	DAB2IP	-0.634354697	8.738048899	1.83E-09
ENSG00000269371.1	AC008878.4	-0.634524726	6.980878438	1.05E-07
ENSG00000127423.10	AUNIP	-0.635187682	5.969035041	1.07E-06
ENSG00000213442.5	RPL18AP3	-0.635246611	8.429492514	3.72E-09
ENSG00000165495.15	PKNOX2	-0.63553912	4.290997825	5.12E-05
ENSG00000167705.11	RILP	-0.636292738	6.247831386	5.65E-07
ENSG00000146411.5	SLC2A12	-0.636425616	2.943153037	0.00114
ENSG00000229638.1	RPL4P4	-0.636726343	10.21555014	6.09E-11
ENSG00000108528.13	SLC25A11	-0.636960909	4.366154762	4.30E-05
ENSG00000242259.8	C22orf39	-0.637089338	10.81010182	1.55E-11
ENSG00000272478.1	AL020996.3	-0.637487862	5.84067669	1.44E-06
ENSG00000261864.1	AC130462.1	-0.637650473	7.470706962	3.38E-08
ENSG00000141404.15	GNAL	-0.637821011	10.20259027	6.27E-11
ENSG00000139977.13	NAA30	-0.637890791	6.446270433	3.58E-07
ENSG00000172340.14	SUCLG2	-0.638006818	6.262793004	5.46E-07
ENSG00000070182.20	SPTB	-0.638048242	2.015458924	0.00965
ENSG00000241666.2	AL031733.2	-0.638085547	3.822944356	0.00015
ENSG00000236526.1	AL035448.1	-0.63821824	4.675882671	2.11E-05
ENSG00000189283.9	FHIT	-0.638381709	5.974003743	1.06E-06

ENSG00000167186.10	COQ7	-0.638488881	9.087397881	8.18E-10
ENSG00000167100.14	SAMD14	-0.638539265	4.430701067	3.71E-05
ENSG00000198771.10	RCSD1	-0.639053211	3.930566295	0.000117
ENSG00000213413.2	PVRIG	-0.639082066	2.729077878	0.001866
ENSG00000142784.15	WDTC1	-0.639246904	6.31619243	4.83E-07
ENSG00000168517.10	HEXIM2	-0.639254629	3.944532371	0.000114
ENSG00000169083.16	AR	-0.639478547	8.033815422	9.25E-09
ENSG00000151090.18	THRB	-0.639573409	8.010298617	9.77E-09
ENSG00000164855.15	TMEM184A	-0.639873832	7.940326772	1.15E-08
ENSG00000157350.12	ST3GAL2	-0.640126163	6.089781825	8.13E-07
ENSG00000116539.12	ASH1L	-0.640224336	15.23763529	5.79E-16
ENSG00000270110.1	AL353593.3	-0.640519687	1.341333462	0.045569
ENSG00000273540.3	AGBL1	-0.64064389	1.410596652	0.038851
ENSG00000213366.12	GSTM2	-0.640812339	8.36785026	4.29E-09
ENSG00000224597.10	SVIL-AS1	-0.640867704	3.677119335	0.00021
ENSG00000284041.1	AC073111.3	-0.640868182	5.590889692	2.57E-06
ENSG00000178502.5	KLHL11	-0.640986931	3.488597221	0.000325
ENSG00000132612.15	VPS4A	-0.641299083	11.10880979	7.78E-12
ENSG00000122873.11	CISD1	-0.641410797	6.162465004	6.88E-07
ENSG00000254783.1	AP003084.1	-0.641703257	3.914896632	0.000122
ENSG00000204444.10	APOM	-0.641890224	6.601877897	2.50E-07
ENSG00000188559.14	RALGAPA2	-0.642226107	5.859678596	1.38E-06
ENSG00000054967.12	RELT	-0.642345045	3.385874998	0.000411
ENSG00000134419.15	RPS15A	-0.642853326	6.631831974	2.33E-07
ENSG00000100344.10	PNPLA3	-0.642953727	6.644984947	2.26E-07
ENSG00000105640.12	RPL18A	-0.643072689	12.62679152	2.36E-13
ENSG00000283663.1	AC008687.8	-0.64353435	1.772070693	0.016902
ENSG00000137393.9	RNF144B	-0.644125836	3.288402394	0.000515
ENSG00000132570.14	PCBD2	-0.644138894	5.977725791	1.05E-06
ENSG00000177752.14	YIPF7	-0.644547847	3.232020464	0.000586
ENSG00000178974.9	FBXO34	-0.644674762	11.65139916	2.23E-12
ENSG00000132361.16	CLUH	-0.644899069	4.558448496	2.76E-05
ENSG00000164086.9	DUSP7	-0.645211868	8.409402804	3.90E-09
ENSG00000174444.14	RPL4	-0.645498343	16.47111986	3.38E-17
ENSG00000173991.5	TCAP	-0.646029007	2.025192651	0.009436
ENSG00000254473.1	AL354920.1	-0.646414766	7.732440671	1.85E-08
ENSG00000148935.11	GAS2	-0.646672224	5.209602787	6.17E-06
ENSG00000176020.8	AMIGO3	-0.646913223	8.500153683	3.16E-09
ENSG00000146066.2	HIGD2A	-0.647178941	10.05166013	8.88E-11
ENSG00000270617.1	URGCP-MRPS24	-0.647250292	7.976260338	1.06E-08
ENSG00000095464.9	PDE6C	-0.647422452	2.452576455	0.003527
ENSG00000221823.10	PPP3R1	-0.647479703	10.0341694	9.24E-11
ENSG00000152413.14	HOMER1	-0.647535334	2.785433723	0.001639
ENSG00000183520.11	UTP11	-0.64756771	5.193427527	6.41E-06
ENSG00000119787.13	ATL2	-0.647976699	7.610601996	2.45E-08
ENSG00000272031.2	ANKRD34A	-0.648041204	4.134316294	7.34E-05
ENSG00000204316.12	MRPL38	-0.648077375	10.65252669	2.23E-11

ENSG00000134463.14	ECHDC3	-0.648081715	3.702662223	0.000198
ENSG00000215218.3	UBE2QL1	-0.648194211	2.268304449	0.005391
ENSG00000135390.18	ATP5MC2	-0.64826798	12.26371976	5.45E-13
ENSG00000108840.15	HDAC5	-0.648330649	17.09476195	8.04E-18
ENSG00000237380.6	HOXD-AS2	-0.648477561	1.685121542	0.020648
ENSG00000189060.5	H1F0	-0.64869194	16.1165011	7.65E-17
ENSG00000247828.7	TMEM161B-AS1	-0.648760783	5.885757662	1.30E-06
ENSG00000185761.10	ADAMTSL5	-0.648957079	3.693312817	0.000203
ENSG00000004142.11	POLDIP2	-0.648979062	6.041325194	9.09E-07
ENSG00000218052.5	ADAMTS7P4	-0.6492418	2.516507784	0.003044
ENSG00000136717.14	BIN1	-0.649378387	3.435750208	0.000367
ENSG00000170185.9	USP38	-0.649381047	5.22309913	5.98E-06
ENSG00000128309.16	MPST	-0.649490737	6.151104898	7.06E-07
ENSG00000198796.6	ALPK2	-0.649520223	2.081868235	0.008282
ENSG00000102935.11	ZNF423	-0.649760108	12.93058824	1.17E-13
ENSG00000171914.16	TLN2	-0.650130836	5.743793859	1.80E-06
ENSG00000267469.1	AC005944.1	-0.650261051	8.207801161	6.20E-09
ENSG00000132681.16	ATP1A4	-0.650320782	2.597654736	0.002525
ENSG00000176946.11	THAP4	-0.6509011	12.03643153	9.20E-13
ENSG00000284269.1	MIR7855	-0.65343049	1.553277907	0.027972
ENSG00000180182.10	MED14	-0.653662873	9.442472038	3.61E-10
ENSG00000118507.16	AKAP7	-0.653764172	7.568154961	2.70E-08
ENSG00000136451.8	VEZF1	-0.653845268	18.26698445	5.41E-19
ENSG00000284008.1	MIR6511B1	-0.654161791	5.584750733	2.60E-06
ENSG00000186868.15	MAPT	-0.65428232	2.778427032	0.001666
ENSG00000255362.1	AP000785.2	-0.655336218	1.954273197	0.01111
ENSG00000186687.15	LYRM7	-0.655352145	6.018291827	9.59E-07
ENSG00000163684.11	RPP14	-0.655715437	5.762827166	1.73E-06
ENSG00000209082.1	MT-TL1	-0.655920062	2.866664186	0.001359
ENSG00000104964.14	AES	-0.65599664	8.46953665	3.39E-09
ENSG00000203867.7	RBM20	-0.656226147	2.258726796	0.005512
ENSG00000251920.1	RNA5SP216	-0.656337201	5.361124045	4.35E-06
ENSG00000283297.1	TEX52	-0.656425669	2.848556709	0.001417
ENSG00000116983.12	HPCAL4	-0.656921354	1.832801884	0.014696
ENSG00000114737.15	CISH	-0.6569319	1.596654557	0.025313
ENSG00000285188.1	AC008397.2	-0.656999883	1.31962252	0.047905
ENSG00000006432.15	MAP3K9	-0.657324546	3.090463245	0.000812
ENSG00000175741.5	RWDD4P2	-0.658076551	10.19802907	6.34E-11
ENSG00000082014.16	SMARCD3	-0.659025125	6.230045722	5.89E-07
ENSG00000146278.10	PNRC1	-0.659215699	11.10179742	7.91E-12
ENSG00000205959.3	AC105345.1	-0.659397953	4.614623928	2.43E-05
ENSG00000030419.16	IKZF2	-0.659439718	5.575698666	2.66E-06
ENSG00000267855.5	NDUFA7	-0.659576149	6.131162457	7.39E-07
ENSG00000225339.3	AL354740.1	-0.659641826	10.68603416	2.06E-11
ENSG00000231587.1	SNORD62B	-0.660256116	3.726816127	0.000188
ENSG00000252106.2	RNY3P15	-0.660278347	1.337374387	0.045986
ENSG00000255154.7	HTD2	-0.660301624	5.404568456	3.94E-06

ENSG00000148826.8	NKX6-2	-0.660321047	6.91199791	1.22E-07
ENSG00000104341.16	LAPTM4B	-0.661636675	4.172178568	6.73E-05
ENSG00000218018.2	AL109955.1	-0.662032143	2.707765411	0.00196
ENSG00000246922.8	UBAP1L	-0.662679639	8.1086366	7.79E-09
ENSG00000062582.13	MRPS24	-0.662787789	7.902149401	1.25E-08
ENSG00000162373.12	BEND5	-0.662846782	12.11487132	7.68E-13
ENSG00000102225.15	CDK16	-0.6629617	8.833829327	1.47E-09
ENSG00000011638.10	TMEM159	-0.663453242	5.119444836	7.60E-06
ENSG00000285437.1	POLR2J3	-0.663975528	5.011549167	9.74E-06
ENSG00000128272.14	ATF4	-0.66428415	9.595849447	2.54E-10
ENSG00000153391.15	INO80C	-0.664449943	16.47111986	3.38E-17
ENSG00000235205.1	TATDN2P3	-0.664611537	1.469219071	0.033945
ENSG00000120729.9	MYOT	-0.665101983	2.386654157	0.004105
ENSG00000104973.17	MED25	-0.665731244	6.511024569	3.08E-07
ENSG00000153531.13	ADPRHL1	-0.665905014	2.35253113	0.004441
ENSG00000099954.18	CECR2	-0.666160836	3.300340249	0.000501
ENSG00000125967.16	NECAB3	-0.666350291	6.443810881	3.60E-07
ENSG00000183230.16	CTNNA3	-0.666943853	2.376408117	0.004203
ENSG00000115840.13	SLC25A12	-0.667237182	4.071645565	8.48E-05
ENSG00000158528.11	PPP1R9A	-0.667252518	7.447402149	3.57E-08
ENSG00000283745.1	MIR196B	-0.667276066	2.530427102	0.002948
ENSG00000166682.11	TMPRSS5	-0.667385471	5.469828924	3.39E-06
ENSG00000141030.12	COPS3	-0.667658075	9.583700868	2.61E-10
ENSG00000234203.1	AC004771.2	-0.667687514	11.98304239	1.04E-12
ENSG00000147454.13	SLC25A37	-0.667988243	9.659709807	2.19E-10
ENSG00000145757.15	SPATA9	-0.66822454	3.830013282	0.000148
ENSG00000179008.8	C14orf39	-0.668277634	3.846192033	0.000142
ENSG00000139620.12	KANSL2	-0.668533452	6.050315849	8.91E-07
ENSG00000152229.18	PSTPIP2	-0.669181205	2.97905156	0.001049
ENSG00000285628.1	AC126755.7	-0.669513274	2.375726609	0.00421
ENSG00000135063.19	FAM189A2	-0.669604246	3.651456785	0.000223
ENSG00000234771.3	SLC25A25-AS1	-0.669627827	5.164353714	6.85E-06
ENSG00000226380.9	LINC-PINT	-0.669947931	4.684242567	2.07E-05
ENSG00000173614.13	NMNAT1	-0.670585175	4.779180968	1.66E-05
ENSG00000114378.16	HYAL1	-0.670855616	4.85657959	1.39E-05
ENSG00000130783.13	CCDC62	-0.671244732	1.694560924	0.020204
ENSG00000010803.16	SCMH1	-0.67205649	18.50441614	3.13E-19
ENSG00000163092.19	XIRP2	-0.67289705	1.685813648	0.020615
ENSG00000248527.1	MTATP6P1	-0.672922462	2.785301849	0.001639
ENSG00000184719.11	RNLS	-0.673210575	9.349255907	4.47E-10
ENSG00000104529.17	EEF1D	-0.673408431	18.93100192	1.17E-19
ENSG00000173812.10	EIF1	-0.673532474	16.04108424	9.10E-17
ENSG00000143612.20	C1orf43	-0.673859064	10.72460812	1.89E-11
ENSG00000273433.1	AC004080.6	-0.674739475	3.825396345	0.000149
ENSG00000185105.5	MYADML2	-0.67475617	2.280278282	0.005245
ENSG00000268149.1	AC010336.4	-0.674891347	9.353687479	4.43E-10
ENSG00000054654.16	SYNE2	-0.675232967	8.488149124	3.25E-09

ENSG00000167588.12	GPD1	-0.675478695	3.104542343	0.000786
ENSG00000070495.14	JMJD6	-0.675985223	8.769789579	1.70E-09
ENSG00000200201.1	RF00019	-0.676540988	1.787601033	0.016308
ENSG00000269352.1	PTOV1-AS2	-0.676830497	18.32800184	4.70E-19
ENSG00000147853.16	AK3	-0.677101877	4.32830556	4.70E-05
ENSG00000165060.12	FXN	-0.677292318	7.244741891	5.69E-08
ENSG00000138347.15	MYPN	-0.677370276	2.721099863	0.001901
ENSG00000063046.17	EIF4B	-0.677627431	13.45141421	3.54E-14
ENSG00000138768.14	USO1	-0.677663304	6.714034461	1.93E-07
ENSG00000259371.2	AL136295.3	-0.678088976	4.811809503	1.54E-05
ENSG00000215105.4	TTC3P1	-0.678164011	4.664205316	2.17E-05
ENSG00000167978.16	SRRM2	-0.678170537	10.86100828	1.38E-11
ENSG00000145358.6	DDIT4L	-0.678560272	2.503064272	0.00314
ENSG00000284015.1	MIR1281	-0.679021495	1.519296839	0.030248
ENSG00000163380.15	LMOD3	-0.679287933	2.781204538	0.001655
ENSG00000167965.17	MLST8	-0.679662823	12.27679586	5.29E-13
ENSG00000060656.19	PTPRU	-0.679664009	5.073374028	8.45E-06
ENSG00000134744.13	TUT4	-0.679833158	13.77566574	1.68E-14
ENSG00000256053.7	APOPT1	-0.680310427	7.70375229	1.98E-08
ENSG00000271895.2	AL109811.3	-0.681147716	7.498772518	3.17E-08
ENSG00000185122.10	HSF1	-0.68147228	12.6185415	2.41E-13
ENSG00000078967.12	UBE2D4	-0.68150605	8.333138609	4.64E-09
ENSG00000244270.1	RPL32P29	-0.681808859	4.259581895	5.50E-05
ENSG00000079999.13	KEAP1	-0.681891455	8.762571743	1.73E-09
ENSG00000116133.12	DHCR24	-0.682640919	1.900915442	0.012563
ENSG00000182054.9	IDH2	-0.682972357	3.03357799	0.000926
ENSG00000267092.2	AC027307.1	-0.683515678	3.860035587	0.000138
ENSG00000099797.14	TECR	-0.683565646	7.331783407	4.66E-08
ENSG00000183134.4	PTGDR2	-0.683813949	2.619238759	0.002403
ENSG00000142599.17	RERE	-0.684308224	7.853281084	1.40E-08
ENSG00000080561.13	MID2	-0.684338499	7.448912253	3.56E-08
ENSG00000094963.13	FMO2	-0.684407452	4.756857652	1.75E-05
ENSG00000184313.19	MROH7	-0.684432906	2.382748043	0.004142
ENSG00000125648.14	SLC25A23	-0.684456007	5.271825874	5.35E-06
ENSG00000154553.14	PDLIM3	-0.684459747	3.152321196	0.000704
ENSG00000280214.1	AC027682.7	-0.684559128	3.327224437	0.000471
ENSG00000256632.3	AC135586.2	-0.68474421	7.792053703	1.61E-08
ENSG00000250159.6	AC106791.1	-0.68554712	2.549982892	0.002818
ENSG00000111860.13	CEP85L	-0.685581613	6.285817022	5.18E-07
ENSG00000080345.17	RIF1	-0.686643185	2.975113644	0.001059
ENSG00000259961.1	AL354712.1	-0.686892788	1.753775208	0.017629
ENSG00000130653.15	PNPLA7	-0.686892857	5.677565059	2.10E-06
ENSG00000114544.16	SLC41A3	-0.686949498	14.39007199	4.07E-15
ENSG00000236208.1	C10orf71-AS1	-0.687199564	1.911616985	0.012257
ENSG00000117419.15	ERI3	-0.68792785	13.64450047	2.27E-14
ENSG00000162669.15	HFM1	-0.688401902	2.473148885	0.003364
ENSG00000235706.7	DICER1-AS1	-0.688412527	7.310840668	4.89E-08

ENSG00000260914.3	AC026464.4	-0.688989041	10.46188559	3.45E-11
ENSG00000006016.10	CRLF1	-0.689164877	5.862442097	1.37E-06
ENSG00000284981.1	AC093668.3	-0.689297906	3.75054875	0.000178
ENSG00000171016.12	PYGO1	-0.689729727	5.628510268	2.35E-06
ENSG00000171714.11	ANO5	-0.689752305	2.738799715	0.001825
ENSG00000189227.5	C15orf61	-0.689830643	5.764100431	1.72E-06
ENSG00000163159.12	VPS72	-0.689965087	4.394647092	4.03E-05
ENSG00000132382.14	MYBBP1A	-0.690335804	6.900633721	1.26E-07
ENSG00000008441.16	NFIX	-0.690389113	7.1744687	6.69E-08
ENSG00000132819.16	RBM38	-0.6906303	3.163458613	0.000686
ENSG00000262454.3	MIR193BHG	-0.69105843	4.638017833	2.30E-05
ENSG00000234636.2	MED14OS	-0.691396434	5.009421369	9.79E-06
ENSG00000232079.6	LINC01697	-0.691601686	1.828366598	0.014847
ENSG00000275645.1	AC068338.3	-0.691833319	5.013195898	9.70E-06
ENSG00000179526.16	SHARPIN	-0.692081077	15.0218033	9.51E-16
ENSG00000125652.7	ALKBH7	-0.692288096	7.742518335	1.81E-08
ENSG00000185010.14	F8	-0.692419743	12.05080751	8.90E-13
ENSG00000128573.24	FOXP2	-0.693172133	4.684813895	2.07E-05
ENSG00000100376.11	FAM118A	-0.69330702	3.627884164	0.000236
ENSG00000131368.7	MRPS25	-0.693356231	17.56462049	2.73E-18
ENSG00000267288.2	AC138150.2	-0.693850609	4.125962859	7.48E-05
ENSG00000254806.5	SYS1-DBNDD2	-0.693867909	6.90772261	1.24E-07
ENSG00000157077.14	ZFYVE9	-0.694112275	9.196101067	6.37E-10
ENSG00000258952.2	SALRNA1	-0.694139496	4.820465124	1.51E-05
ENSG00000276664.1	RF01882	-0.694154669	1.402495891	0.039583
ENSG00000268163.1	AC004076.1	-0.694197528	2.897536021	0.001266
ENSG00000261915.6	AC026954.2	-0.69435636	19.95470761	1.11E-20
ENSG00000150054.18	MPP7	-0.694371103	5.27441407	5.32E-06
ENSG00000004478.7	FKBP4	-0.694472501	10.05166013	8.88E-11
ENSG00000197483.9	ZNF628	-0.694641098	14.13370082	7.35E-15
ENSG00000168286.2	THAP11	-0.694870145	17.59085186	2.57E-18
ENSG00000162191.13	UBXN1	-0.695403455	18.94314004	1.14E-19
ENSG00000263327.6	TAPT1-AS1	-0.695574202	6.616415467	2.42E-07
ENSG00000077522.12	ACTN2	-0.695898745	2.215830386	0.006084
ENSG00000185669.5	SNAI3	-0.696014926	1.777070119	0.016708
ENSG00000254740.2	AP003396.3	-0.696033561	2.635920425	0.002312
ENSG00000112293.14	GPLD1	-0.696212631	7.402425631	3.96E-08
ENSG00000255330.9	AL096711.2	-0.696371894	4.782097638	1.65E-05
ENSG00000244274.7	DBNDD2	-0.697041765	6.655138004	2.21E-07
ENSG00000161671.16	EMC10	-0.698090819	4.621201123	2.39E-05
ENSG00000188153.13	COL4A5	-0.698245484	5.195558487	6.37E-06
ENSG00000279605.1	AC067930.6	-0.698383966	17.35256376	4.44E-18
ENSG00000254741.1	AC067930.2	-0.699031465	16.05041161	8.90E-17
ENSG00000143858.11	SYT2	-0.699450936	5.049999385	8.91E-06
ENSG00000005379.16	TSPOAP1	-0.699476401	5.719545061	1.91E-06
ENSG00000176182.5	MYPOP	-0.699586763	10.19052478	6.45E-11
ENSG00000160886.13	LY6K	-0.699729251	3.293341348	0.000509

ENSG00000253293.4	HOXA10	-0.699820115	6.955835149	1.11E-07
ENSG00000263823.1	AC009831.1	-0.699865323	3.156288123	0.000698
ENSG00000158856.18	DMTN	-0.700464067	4.54959664	2.82E-05
ENSG00000178104.19	PDE4DIP	-0.70104017	3.425220478	0.000376
ENSG00000164406.7	LEAP2	-0.701306516	6.613093953	2.44E-07
ENSG00000156574.9	NODAL	-0.701490023	1.394347914	0.040332
ENSG00000185436.11	IFNLR1	-0.701752077	3.106555015	0.000782
ENSG00000261423.1	TMEM202-AS1	-0.702212132	6.687746934	2.05E-07
ENSG00000132429.9	POPDC3	-0.702663175	3.130383137	0.000741
ENSG00000267318.1	AC005702.1	-0.702783901	8.44643516	3.58E-09
ENSG00000130962.17	PRRG1	-0.702802243	5.832671094	1.47E-06
ENSG00000184887.13	BTBD6	-0.703115732	6.503547211	3.14E-07
ENSG00000005075.15	POLR2J	-0.703700278	7.948735234	1.13E-08
ENSG00000261240.1	AC009065.6	-0.704586189	6.22612046	5.94E-07
ENSG00000168152.12	THAP9	-0.704768813	8.818638032	1.52E-09
ENSG00000143171.12	RXRG	-0.705196072	2.65928924	0.002191
ENSG00000112782.16	CLIC5	-0.705287929	2.8873004	0.001296
ENSG00000262967.1	AC005921.2	-0.705343527	9.117387376	7.63E-10
ENSG00000175395.15	ZNF25	-0.705804184	14.9379981	1.15E-15
ENSG00000272106.1	AL691432.2	-0.706539375	6.132088294	7.38E-07
ENSG00000269194.1	AC006942.1	-0.707196686	8.099223413	7.96E-09
ENSG00000273356.1	LINC02019	-0.707304366	3.210918322	0.000615
ENSG00000125246.15	CLYBL	-0.707317315	6.141976707	7.21E-07
ENSG00000174428.16	GTF2IRD2B	-0.707328744	15.65195297	2.23E-16
ENSG00000066629.17	EML1	-0.707884611	12.25750558	5.53E-13
ENSG00000008513.15	ST3GAL1	-0.708289297	4.98161693	1.04E-05
ENSG00000276293.4	PIP4K2B	-0.708753861	7.631714444	2.33E-08
ENSG00000138759.18	FRAS1	-0.708877019	3.768558434	0.00017
ENSG00000154945.6	ANKRD40	-0.709362719	7.592016372	2.56E-08
ENSG00000177084.16	POLE	-0.710337837	13.19092023	6.44E-14
ENSG00000131773.13	KHDRBS3	-0.710348427	9.529793621	2.95E-10
ENSG00000134324.11	LPIN1	-0.710982694	4.803900318	1.57E-05
ENSG00000226416.1	MRPL23-AS1	-0.711480208	2.13045601	0.007405
ENSG00000108878.4	CACNG1	-0.711919765	2.95122023	0.001119
ENSG00000264589.3	MAPT-AS1	-0.712073528	2.703823399	0.001978
ENSG00000123411.14	IKZF4	-0.712269466	8.094534203	8.04E-09
ENSG00000272114.1	AL136131.3	-0.712308743	2.457523923	0.003487
ENSG00000120709.10	FAM53C	-0.712349773	8.095841598	8.02E-09
ENSG00000162244.11	RPL29	-0.71242011	11.63756162	2.30E-12
ENSG00000272325.1	NUDT3	-0.713406361	12.54680579	2.84E-13
ENSG00000143994.13	ABHD1	-0.713532916	6.005542841	9.87E-07
ENSG00000165632.7	TAF3	-0.713588262	16.0629909	8.65E-17
ENSG00000153561.12	RMND5A	-0.713822022	7.858558749	1.38E-08
ENSG00000249971.1	AC104116.1	-0.71404473	7.382568181	4.14E-08
ENSG00000170745.11	KCNS3	-0.714128174	4.195891377	6.37E-05
ENSG00000124151.18	NCOA3	-0.714416062	7.695605175	2.02E-08
ENSG00000136546.14	SCN7A	-0.714782513	2.366216002	0.004303

ENSG00000228649.8	SNHG26	-0.714920393	5.228556732	5.91E-06
ENSG00000248492.1	ZFAT-AS1	-0.714980706	1.800686358	0.015824
ENSG00000171033.12	PKIA	-0.715001074	3.08106758	0.00083
ENSG00000163806.15	SPDYA	-0.715234871	5.781377584	1.65E-06
ENSG00000144061.12	NPHP1	-0.715528717	3.715776221	0.000192
ENSG00000226329.2	AC005682.1	-0.71588348	2.270637732	0.005362
ENSG00000084733.10	RAB10	-0.715939854	7.491301887	3.23E-08
ENSG00000091542.8	ALKBH5	-0.716235763	6.823461282	1.50E-07
ENSG00000147872.9	PLIN2	-0.716315674	7.245326002	5.68E-08
ENSG00000179456.10	ZBTB18	-0.717025027	4.948453792	1.13E-05
ENSG00000102119.10	EMD	-0.717026306	17.25330576	5.58E-18
ENSG00000162139.9	NEU3	-0.717410868	6.883870018	1.31E-07
ENSG00000136732.15	GYPC	-0.717603345	11.10879591	7.78E-12
ENSG00000214026.10	MRPL23	-0.717641617	15.42127904	3.79E-16
ENSG00000111817.17	DSE	-0.717930453	8.7746315	1.68E-09
ENSG00000124164.15	VAPB	-0.718016257	10.73906339	1.82E-11
ENSG00000170162.13	VGLL2	-0.718130213	1.936721634	0.011569
ENSG00000128655.17	PDE11A	-0.718231048	2.254305519	0.005568
ENSG00000285564.1	DKFZp451B082	-0.718366201	1.860393341	0.013791
ENSG00000141034.9	GID4	-0.718600052	10.90393487	1.25E-11
ENSG00000184601.10	C14orf180	-0.718716046	1.594977862	0.025411
ENSG00000259037.1	BX927359.1	-0.718848944	1.33678109	0.046049
ENSG00000167434.9	CA4	-0.71944571	2.302653275	0.004981
ENSG00000266993.3	AL050343.1	-0.719706597	10.83898766	1.45E-11
ENSG00000197321.14	SVIL	-0.719922576	3.14901498	0.00071
ENSG00000198853.11	RUSC2	-0.720396979	9.022936564	9.49E-10
ENSG00000179564.3	LSMEM2	-0.720981234	9.073124123	8.45E-10
ENSG00000076685.18	NT5C2	-0.721523291	9.000157964	1.00E-09
ENSG00000143126.7	CELSR2	-0.721979418	3.387368379	0.00041
ENSG00000115896.15	PLCL1	-0.722879021	4.042765763	9.06E-05
ENSG00000166266.13	CUL5	-0.723015635	8.131557761	7.39E-09
ENSG00000152705.7	CATSPER3	-0.723132182	4.864476258	1.37E-05
ENSG00000266677.1	AC087164.1	-0.723360542	5.134877603	7.33E-06
ENSG00000267257.1	AC105105.1	-0.723564116	2.44784879	0.003566
ENSG00000152217.18	SETBP1	-0.723697435	9.604680291	2.48E-10
ENSG00000111911.6	HINT3	-0.723922469	6.167782726	6.80E-07
ENSG00000114738.10	MAPKAPK3	-0.723998025	5.527291187	2.97E-06
ENSG00000166949.15	SMAD3	-0.724126628	11.64840081	2.25E-12
ENSG00000205090.8	TMEM240	-0.724176124	4.262407504	5.47E-05
ENSG00000130560.8	UBAC1	-0.724230633	8.151381637	7.06E-09
ENSG00000205746.9	AC126755.1	-0.724959669	4.295295463	5.07E-05
ENSG00000270392.2	PFN1P2	-0.724978744	2.60413098	0.002488
ENSG00000165526.8	RPUSD4	-0.724985449	7.539859517	2.88E-08
ENSG00000123124.13	WWP1	-0.725650848	5.902795477	1.25E-06
ENSG00000144034.14	TPRKB	-0.725654948	11.07857118	8.35E-12
ENSG00000164880.15	INTS1	-0.726017231	10.42798909	3.73E-11
ENSG00000260751.2	AC008870.2	-0.726643102	5.914669811	1.22E-06

ENSG00000159208.15	CIART	-0.72717703	1.86952898	0.013504
ENSG00000254913.1	AC239802.1	-0.727287621	3.051325264	0.000889
ENSG00000213639.9	PPP1CB	-0.727554294	7.744151259	1.80E-08
ENSG00000175356.13	SCUBE2	-0.727818033	2.828190917	0.001485
ENSG00000186106.11	ANKRD46	-0.728045058	7.551043081	2.81E-08
ENSG00000283971.1	MIR4442	-0.728225751	1.604920012	0.024836
ENSG00000165338.16	HECTD2	-0.728236852	6.095756688	8.02E-07
ENSG00000176909.11	MAMSTR	-0.728375161	3.575556732	0.000266
ENSG00000103994.17	ZNF106	-0.728402786	3.703360187	0.000198
ENSG00000131069.19	ACSS2	-0.728414391	6.042593791	9.07E-07
ENSG00000009724.16	MASP2	-0.728824914	9.41251095	3.87E-10
ENSG00000205609.12	EIF3CL	-0.728903198	5.567552732	2.71E-06
ENSG00000124702.17	KLHDC3	-0.729647713	7.246369395	5.67E-08
ENSG00000279365.1	AP000695.3	-0.729903312	1.434714377	0.036752
ENSG00000107262.21	BAG1	-0.730911979	9.471193085	3.38E-10
ENSG00000132155.11	RAF1	-0.731497966	14.74298004	1.81E-15
ENSG00000119414.11	PPP6C	-0.731619566	23.11613302	7.65E-24
ENSG00000185271.8	KLHL33	-0.731623279	3.053140497	0.000885
ENSG00000183655.12	KLHL25	-0.731737105	6.971924734	1.07E-07
ENSG00000166582.9	CENPV	-0.731791037	7.966426643	1.08E-08
ENSG00000103319.11	EEF2K	-0.732074794	11.76205006	1.73E-12
ENSG00000135740.16	SLC9A5	-0.732090835	4.430484615	3.71E-05
ENSG00000054523.17	KIF1B	-0.732354394	5.053818696	8.83E-06
ENSG00000141447.17	OSBPL1A	-0.732494332	9.61909068	2.40E-10
ENSG00000259291.2	ZNF710-AS1	-0.733433706	4.139585872	7.25E-05
ENSG00000242735.1	RPSAP26	-0.73346675	1.415014239	0.038458
ENSG00000279722.1	AC007342.7	-0.733517411	6.113914671	7.69E-07
ENSG00000129744.2	ART1	-0.733681469	1.849596915	0.014138
ENSG00000163827.12	LRRC2	-0.733921237	2.904893487	0.001245
ENSG00000072401.14	UBE2D1	-0.734702622	4.41781909	3.82E-05
ENSG00000175084.11	DES	-0.73492765	2.825643019	0.001494
ENSG00000275342.4	PRAG1	-0.735302081	10.84594645	1.43E-11
ENSG00000135972.8	MRPS9	-0.735897509	10.39497462	4.03E-11
ENSG00000180113.15	TDRD6	-0.735913963	3.073022046	0.000845
ENSG00000227409.1	ZMYM4-AS1	-0.736641157	3.341542111	0.000455
ENSG00000231851.5	UTAT33	-0.736806549	11.29127124	5.11E-12
ENSG00000196923.13	PDLIM7	-0.73689515	5.031908874	9.29E-06
ENSG00000225400.1	RAB28P5	-0.737043605	6.058478099	8.74E-07
ENSG00000134333.13	LDHA	-0.737344762	3.600688133	0.000251
ENSG00000115694.14	STK25	-0.737806396	9.931920362	1.17E-10
ENSG00000225950.8	NTF4	-0.737959093	1.867015967	0.013583
ENSG00000165731.18	RET	-0.738126562	2.760560663	0.001736
ENSG00000284114.1	MIR6800	-0.738379884	6.740379101	1.82E-07
ENSG00000163157.14	TMOD4	-0.738670817	3.531899515	0.000294
ENSG00000227128.4	LBX1-AS1	-0.738817072	2.535661146	0.002913
ENSG00000172366.19	MCRIP2	-0.739694898	5.735519201	1.84E-06
ENSG00000189367.14	KIAA0408	-0.739729348	4.964233567	1.09E-05

ENSG00000143434.15	SEMA6C	-0.739839115	3.970182101	0.000107
ENSG00000112183.14	RBM24	-0.740887283	3.148892052	0.00071
ENSG00000167658.15	EEF2	-0.741425963	14.09753415	7.99E-15
ENSG00000106554.12	CHCHD3	-0.741649927	6.04951553	8.92E-07
ENSG00000179636.14	TPPP2	-0.741757422	4.396713816	4.01E-05
ENSG00000213585.10	VDAC1	-0.742050413	5.24204815	5.73E-06
ENSG00000123358.19	NR4A1	-0.742275104	2.56164836	0.002744
ENSG00000228049.7	POLR2J2	-0.742392156	3.266162434	0.000542
ENSG00000112715.21	VEGFA	-0.742433338	3.437945866	0.000365
ENSG00000158158.11	CNNM4	-0.742635402	5.520068143	3.02E-06
ENSG00000126934.13	MAP2K2	-0.743002568	14.29848063	5.03E-15
ENSG00000091436.16	MAP3K20	-0.74373941	4.763554148	1.72E-05
ENSG00000166317.11	SYNPO2L	-0.743946414	2.925719184	0.001187
ENSG00000272267.2	AC021242.3	-0.744094854	2.994947124	0.001012
ENSG00000284431.1	AL022238.4	-0.744698624	13.39995485	3.98E-14
ENSG00000185246.17	PRPF39	-0.744788684	11.51973176	3.02E-12
ENSG00000036448.9	MYOM2	-0.745058097	2.298095435	0.005034
ENSG00000210154.1	MT-TD	-0.745587313	4.460209162	3.47E-05
ENSG00000175591.11	P2RY2	-0.745890093	3.43034952	0.000371
ENSG00000102471.14	NDFIP2	-0.746268862	12.0346526	9.23E-13
ENSG00000115361.7	ACADL	-0.746354909	5.661988652	2.18E-06
ENSG00000187715.13	KBTBD12	-0.746510311	3.410345273	0.000389
ENSG00000036672.15	USP2	-0.746888348	3.368022158	0.000429
ENSG00000231628.1	AL133406.2	-0.74710653	7.4463849	3.58E-08
ENSG00000083814.13	ZNF671	-0.748067035	7.121027898	7.57E-08
ENSG00000205683.11	DPF3	-0.748105826	3.752957679	0.000177
ENSG00000231933.7	Z98949.1	-0.748445608	2.592286226	0.002557
ENSG00000102893.15	PHKB	-0.748820566	7.032203743	9.29E-08
ENSG00000112186.11	CAP2	-0.75020935	3.861212051	0.000138
ENSG00000133454.15	MYO18B	-0.750394494	2.668312196	0.002146
ENSG00000092148.12	HECTD1	-0.750599545	8.377036689	4.20E-09
ENSG00000168907.13	PLA2G4F	-0.750685899	1.958640192	0.010999
ENSG00000224546.2	EIF4BP3	-0.750762019	3.634938232	0.000232
ENSG00000180229.12	HERC2P3	-0.751317988	2.069083031	0.008529
ENSG00000205669.3	ACOT6	-0.751427937	2.781350224	0.001654
ENSG00000151552.11	QDPR	-0.751500083	11.57469836	2.66E-12
ENSG00000163050.16	COQ8A	-0.752424392	3.26102501	0.000548
ENSG00000170935.7	NCBP2L	-0.753198113	6.50787344	3.11E-07
ENSG00000233593.8	AL590094.1	-0.753379234	6.362553768	4.34E-07
ENSG00000253187.2	HOXA10-AS	-0.753551553	8.663504005	2.17E-09
ENSG00000234175.1	AL355355.2	-0.753614904	3.935911427	0.000116
ENSG00000275441.1	AC020765.2	-0.753869736	13.25011063	5.62E-14
ENSG00000135842.16	FAM129A	-0.753974178	4.141171465	7.22E-05
ENSG00000166405.14	RIC3	-0.754074247	8.25623023	5.54E-09
ENSG00000269926.1	DDIT4-AS1	-0.754163433	4.139066076	7.26E-05
ENSG00000258092.1	AC005841.1	-0.754190454	11.24681488	5.66E-12
ENSG00000118515.11	SGK1	-0.75441855	3.708854483	0.000195

ENSG00000187840.4	EIF4EBP1	-0.754472659	5.513380492	3.07E-06
ENSG00000178460.17	MCMDC2	-0.754589105	9.759977042	1.74E-10
ENSG00000272906.1	AL353708.3	-0.755002728	9.865651205	1.36E-10
ENSG00000260855.1	AL591848.4	-0.755359493	5.184073194	6.55E-06
ENSG00000137309.19	HMGA1	-0.756384222	7.80441821	1.57E-08
ENSG00000137818.11	RPLP1	-0.75773491	12.88989856	1.29E-13
ENSG00000144357.16	UBR3	-0.758236627	5.674633439	2.12E-06
ENSG00000258603.3	AC005225.2	-0.759016117	2.736824929	0.001833
ENSG00000234638.1	AC053503.4	-0.759074743	3.03157019	0.00093
ENSG00000067177.14	PHKA1	-0.75918488	2.845897045	0.001426
ENSG00000112319.18	EYA4	-0.759659673	5.996320179	1.01E-06
ENSG00000079393.20	DUSP13	-0.75980607	3.055922214	0.000879
ENSG00000164093.16	PITX2	-0.760173211	3.021798231	0.000951
ENSG00000179698.13	WDR97	-0.760353035	4.483642993	3.28E-05
ENSG00000179364.13	PACS2	-0.761051019	15.4340903	3.68E-16
ENSG00000242396.1	AC096536.2	-0.761239792	1.980133681	0.010468
ENSG00000235837.1	AC073333.1	-0.761401492	5.372001261	4.25E-06
ENSG00000206559.7	ZCWPW2	-0.761529094	6.388931672	4.08E-07
ENSG00000167632.15	TRAPPC9	-0.762058025	9.245601235	5.68E-10
ENSG00000129749.3	CHRNA10	-0.762192533	3.105771928	0.000784
ENSG00000152104.11	PTPN14	-0.763318817	10.56215529	2.74E-11
ENSG00000065518.7	NDUFB4	-0.763872301	10.01561408	9.65E-11
ENSG00000073711.10	PPP2R3A	-0.763878214	4.47849106	3.32E-05
ENSG00000276710.4	CSPG4P10	-0.76427434	5.819102518	1.52E-06
ENSG00000005471.17	ABCB4	-0.764353266	5.561953773	2.74E-06
ENSG00000283041.1	AC008038.1	-0.7644283	11.55787737	2.77E-12
ENSG00000258604.1	AL161668.4	-0.76454741	4.609857842	2.46E-05
ENSG00000139209.15	SLC38A4	-0.765003349	2.306511831	0.004937
ENSG00000162494.5	LRRC38	-0.765404851	2.486116028	0.003265
ENSG00000258940.2	AL132639.2	-0.76545604	7.770882508	1.69E-08
ENSG00000242902.1	FLNC-AS1	-0.765840819	2.748273901	0.001785
ENSG00000175826.11	CTDNEP1	-0.766060988	10.94289399	1.14E-11
ENSG00000178741.11	COX5A	-0.766767008	5.171641253	6.74E-06
ENSG00000078618.21	NRDC	-0.766812311	9.429457897	3.72E-10
ENSG00000267809.1	NDUFV2P1	-0.766966653	4.648336877	2.25E-05
ENSG00000167799.9	NUDT8	-0.767186207	4.332435215	4.65E-05
ENSG00000166295.8	ANAPC16	-0.767231916	14.07703891	8.37E-15
ENSG00000172809.12	RPL38	-0.767522885	13.08749787	8.18E-14
ENSG00000197258.5	EIF4BP6	-0.767593752	7.642005162	2.28E-08
ENSG00000006757.11	PNPLA4	-0.767659238	7.119956231	7.59E-08
ENSG00000223923.1	AC010136.1	-0.767885919	2.727049457	0.001875
ENSG00000006576.16	PHTF2	-0.768044028	4.460381505	3.46E-05
ENSG00000112425.14	EPM2A	-0.768160872	5.530044889	2.95E-06
ENSG00000262302.1	AC003688.1	-0.768472816	9.542457006	2.87E-10
ENSG00000102313.8	ITIH6	-0.768570608	2.226134374	0.005941
ENSG00000213889.10	PPM1N	-0.768806818	4.49104359	3.23E-05
ENSG00000213160.9	KLHL23	-0.768958377	9.34498681	4.52E-10

ENSG00000198624.12	CCDC69	-0.768973767	4.756917395	1.75E-05
ENSG00000144596.12	GRIP2	-0.769332376	4.4745913	3.35E-05
ENSG00000176476.8	SGF29	-0.77016648	17.08043583	8.31E-18
ENSG00000196323.13	ZBTB44	-0.770783544	12.78592705	1.64E-13
ENSG00000174032.16	SLC25A30	-0.771956764	4.546614828	2.84E-05
ENSG00000131097.6	HIGD1B	-0.772164181	4.415699958	3.84E-05
ENSG00000068383.18	INPP5A	-0.772225476	7.642627104	2.28E-08
ENSG00000067225.17	PKM	-0.773116479	4.755527445	1.76E-05
ENSG00000168209.4	DDIT4	-0.774159782	4.319720129	4.79E-05
ENSG00000149646.12	CNBD2	-0.774240325	7.772851837	1.69E-08
ENSG00000198933.9	TBKBP1	-0.774497204	7.857708267	1.39E-08
ENSG00000121769.7	FABP3	-0.774578691	2.462208872	0.00345
ENSG00000118197.13	DDX59	-0.774585296	14.61772353	2.41E-15
ENSG00000235296.1	AC145207.1	-0.774794782	2.692085158	0.002032
ENSG00000181016.9	LSMEM1	-0.775449456	4.0352397	9.22E-05
ENSG00000132849.20	PATJ	-0.776001845	4.224321397	5.97E-05
ENSG00000143248.12	RGS5	-0.776253668	9.08525212	8.22E-10
ENSG00000128591.15	FLNC	-0.77700455	2.868378384	0.001354
ENSG00000268047.1	AC018766.1	-0.777696876	13.49566072	3.19E-14
ENSG00000152700.13	SAR1B	-0.778109286	6.656223722	2.21E-07
ENSG00000180891.12	CUEDC1	-0.778278754	11.99047054	1.02E-12
ENSG00000100380.13	ST13	-0.778420775	28.41511041	3.84E-29
ENSG00000188716.5	DUPD1	-0.778521595	2.975158376	0.001059
ENSG00000112964.13	GHR	-0.779015963	9.829296692	1.48E-10
ENSG00000185418.15	TARSL2	-0.779294498	6.897201247	1.27E-07
ENSG00000281183.1	NPTN-IT1	-0.779537631	5.4741664	3.36E-06
ENSG00000271848.1	AC073389.2	-0.779887395	3.611944416	0.000244
ENSG00000265690.7	AC074143.1	-0.780461178	9.098066816	7.98E-10
ENSG00000162104.9	ADCY9	-0.780480057	6.722737765	1.89E-07
ENSG00000133619.17	KRBA1	-0.780499745	7.765484229	1.72E-08
ENSG00000224982.3	TMEM233	-0.78179942	4.370369667	4.26E-05
ENSG00000250309.2	AC008453.1	-0.782093712	2.418664611	0.003814
ENSG00000116748.21	AMPD1	-0.782172943	3.122396373	0.000754
ENSG00000284584.1	MIR5006	-0.782360893	4.531659249	2.94E-05
ENSG00000162402.13	USP24	-0.782505658	7.808240973	1.56E-08
ENSG00000146729.9	NIPSNAP2	-0.782677268	5.766404393	1.71E-06
ENSG00000261221.3	ZNF865	-0.783162406	8.42446787	3.76E-09
ENSG00000267140.1	AC007998.2	-0.783418323	14.53399733	2.92E-15
ENSG00000168528.11	SERINC2	-0.783546814	5.435995982	3.66E-06
ENSG00000182621.17	PLCB1	-0.783895356	8.930465434	1.17E-09
ENSG00000224424.7	PRKAR2A-AS1	-0.784140746	4.795360431	1.60E-05
ENSG00000152556.16	PFKM	-0.784303178	4.058787392	8.73E-05
ENSG00000107854.5	TNKS2	-0.784367219	11.89201513	1.28E-12
ENSG00000119138.4	KLF9	-0.784587988	7.983365503	1.04E-08
ENSG00000284372.1	MIR6808	-0.784779581	6.555823165	2.78E-07
ENSG00000137831.14	UACA	-0.785277921	6.592108645	2.56E-07
ENSG00000136261.14	BZW2	-0.78534662	5.924069663	1.19E-06

ENSG00000275307.1	RF01892	-0.785422653	5.360122002	4.36E-06
ENSG00000185267.9	CDNF	-0.785505628	8.973997539	1.06E-09
ENSG00000078061.12	ARAF	-0.786208935	13.6554813	2.21E-14
ENSG00000051382.8	PIK3CB	-0.786895255	8.262619707	5.46E-09
ENSG00000184481.16	FOXO4	-0.786959484	6.826468358	1.49E-07
ENSG00000224195.1	AC022400.1	-0.787786593	7.34950685	4.47E-08
ENSG00000279026.1	AC005225.4	-0.788094357	2.147134222	0.007126
ENSG00000279792.1	AC015909.5	-0.788409787	2.67166262	0.00213
ENSG00000259884.1	AC025259.3	-0.788550516	2.778694484	0.001665
ENSG00000143164.15	DCAF6	-0.78942939	6.666347649	2.16E-07
ENSG00000035664.11	DAPK2	-0.789442296	5.392663762	4.05E-06
ENSG00000284135.1	MIR3654	-0.790098443	9.550956262	2.81E-10
ENSG00000126091.20	ST3GAL3	-0.790168861	6.981271307	1.04E-07
ENSG00000167969.12	ECI1	-0.790407524	9.494666816	3.20E-10
ENSG00000135723.13	FHOD1	-0.790928625	4.93171351	1.17E-05
ENSG00000168890.13	TMEM150A	-0.791489102	13.01052453	9.76E-14
ENSG00000085433.15	WDR47	-0.791622135	9.580454935	2.63E-10
ENSG00000155313.15	USP25	-0.792261093	9.186909426	6.50E-10
ENSG00000257252.5	AC124947.1	-0.792287326	3.572116093	0.000268
ENSG00000163820.14	FYCO1	-0.792583776	4.098768014	7.97E-05
ENSG00000186566.12	GPATCH8	-0.792625459	15.12616958	7.48E-16
ENSG00000184203.7	PPP1R2	-0.793048958	11.23090926	5.88E-12
ENSG00000230953.2	AC099677.1	-0.793055284	4.871166879	1.35E-05
ENSG00000119048.7	UBE2B	-0.793115388	12.78020264	1.66E-13
ENSG00000176974.19	SHMT1	-0.795084211	8.965847908	1.08E-09
ENSG00000250899.3	AC125807.2	-0.795338965	3.666412897	0.000216
ENSG00000175390.13	EIF3F	-0.795401269	26.21929758	6.04E-27
ENSG00000233024.7	AC126755.2	-0.795986209	4.238444409	5.78E-05
ENSG00000198894.7	CIPC	-0.796049147	5.877953839	1.32E-06
ENSG00000100347.14	SAMM50	-0.796361933	11.45694766	3.49E-12
ENSG00000171105.13	INSR	-0.796559555	10.41702092	3.83E-11
ENSG00000170035.15	UBE2E3	-0.797056306	14.82983491	1.48E-15
ENSG00000152642.10	GPD1L	-0.797419319	4.228011225	5.92E-05
ENSG00000177354.11	C10orf71	-0.797515114	2.8927424	0.00128
ENSG00000279348.1	AC012513.3	-0.797755211	5.284508302	5.19E-06
ENSG00000103710.10	RASL12	-0.798253935	8.093497773	8.06E-09
ENSG00000070371.15	CLTCL1	-0.799263466	4.386390672	4.11E-05
ENSG00000229212.8	AC044860.1	-0.800822664	2.11318965	0.007706
ENSG00000162885.12	B3GALNT2	-0.8008842	6.817766224	1.52E-07
ENSG00000125166.12	GOT2	-0.801094869	4.064242507	8.62E-05
ENSG00000270249.1	AC093668.1	-0.801407413	6.584788471	2.60E-07
ENSG00000214706.10	IFRD2	-0.802554264	12.67098964	2.13E-13
ENSG00000237412.6	PRSS56	-0.80279979	2.671954678	0.002128
ENSG00000107954.10	NEURL1	-0.803287324	3.272172886	0.000534
ENSG00000235663.1	SAPCD1-AS1	-0.803383877	7.817247072	1.52E-08
ENSG00000125744.11	RTN2	-0.803807469	4.629488583	2.35E-05
ENSG00000142453.11	CARM1	-0.803851126	10.61041194	2.45E-11

ENSG00000108352.12	RAPGEFL1	-0.803919391	6.338426165	4.59E-07
ENSG00000108984.14	MAP2K6	-0.804249296	4.346596849	4.50E-05
ENSG00000255769.7	GOLGA2P10	-0.804349536	5.513380492	3.07E-06
ENSG00000140990.14	NDUFB10	-0.804591814	6.747036652	1.79E-07
ENSG00000103353.15	UBFD1	-0.804619844	11.972154	1.07E-12
ENSG00000158402.19	CDC25C	-0.804630099	5.984706366	1.04E-06
ENSG00000278709.1	NKILA	-0.804766571	3.295056996	0.000507
ENSG00000165113.12	GKAP1	-0.806023424	8.563748903	2.73E-09
ENSG00000110717.12	NDUFS8	-0.806174301	9.582656109	2.61E-10
ENSG00000148660.20	CAMK2G	-0.806307709	8.243856872	5.70E-09
ENSG00000261087.1	AP003469.4	-0.806322188	7.235360879	5.82E-08
ENSG00000107404.19	DVL1	-0.806829974	6.347451383	4.49E-07
ENSG00000178201.4	VN1R1	-0.807036495	3.706908596	0.000196
ENSG00000185946.15	RNPC3	-0.807612389	11.20703323	6.21E-12
ENSG00000136813.14	ECPAS	-0.808207754	10.0001139	1.00E-10
ENSG00000256222.2	MTRNR2L3	-0.808737474	1.36973576	0.042684
ENSG00000065675.14	PRKCQ	-0.808850177	4.239595536	5.76E-05
ENSG00000230970.3	HHATL-AS1	-0.808884229	3.255126578	0.000556
ENSG00000169570.9	DTWD2	-0.809783946	6.934124936	1.16E-07
ENSG00000259351.1	AC015914.1	-0.810060133	6.902284017	1.25E-07
ENSG00000168530.15	MYL1	-0.810185338	3.405773283	0.000393
ENSG00000069535.13	MAOB	-0.810241972	6.029380101	9.35E-07
ENSG00000255372.1	AC107886.1	-0.810590782	2.536920301	0.002905
ENSG00000179632.9	MAF1	-0.810825741	14.17983719	6.61E-15
ENSG00000233021.2	AL669841.1	-0.810987613	3.035308936	0.000922
ENSG00000284989.1	AL451062.3	-0.811131227	12.44497971	3.59E-13
ENSG00000283662.1	AC138904.3	-0.811280739	2.620723444	0.002395
ENSG00000152601.17	MBNL1	-0.811877796	12.37840027	4.18E-13
ENSG00000106078.18	COBL	-0.812431576	4.18031088	6.60E-05
ENSG00000230561.4	CCDC192	-0.812513958	7.02743384	9.39E-08
ENSG00000259827.1	AC026461.1	-0.812667234	3.954479068	0.000111
ENSG00000187193.8	MT1X	-0.812781196	3.943231889	0.000114
ENSG00000162378.12	ZYG11B	-0.812829641	5.900501598	1.26E-06
ENSG00000067141.16	NEO1	-0.81303758	9.789193335	1.62E-10
ENSG00000175606.10	TMEM70	-0.814061951	3.478640875	0.000332
ENSG00000013563.13	DNASE1L1	-0.814450821	11.10975342	7.77E-12
ENSG00000170153.10	RNF150	-0.815765387	5.356834959	4.40E-06
ENSG00000136383.6	ALPK3	-0.816619737	3.096990745	0.0008
ENSG00000130695.15	CEP85	-0.816654997	4.842067494	1.44E-05
ENSG00000274265.4	AC245297.3	-0.816679528	4.025470082	9.43E-05
ENSG00000112246.9	SIM1	-0.816761393	2.061022026	0.008689
ENSG00000178538.9	CA8	-0.818385697	3.928511297	0.000118
ENSG00000138772.12	ANXA3	-0.818416058	4.376824838	4.20E-05
ENSG00000173801.16	JUP	-0.818524216	8.426109078	3.75E-09
ENSG00000245768.6	AC092378.1	-0.81917241	3.613096978	0.000244
ENSG00000185262.8	UBALD2	-0.81934728	10.38747698	4.10E-11
ENSG00000279317.2	AC006994.2	-0.819396357	2.227273981	0.005926

ENSG00000122367.19	LDB3	-0.820166368	3.270058785	0.000537
ENSG00000160299.16	PCNT	-0.820900619	5.017069492	9.61E-06
ENSG00000280401.1	AC022532.1	-0.821325292	1.571901584	0.026798
ENSG00000188385.11	JAKMIP3	-0.821496909	7.451869872	3.53E-08
ENSG00000171617.13	ENC1	-0.821703656	5.145933403	7.15E-06
ENSG00000186350.10	RXRA	-0.822165776	10.39825658	4.00E-11
ENSG00000127527.13	EPS15L1	-0.823047368	11.25461144	5.56E-12
ENSG00000283175.1	AC007920.2	-0.823475255	2.292030594	0.005105
ENSG00000184489.11	PTP4A3	-0.8236372	4.214710656	6.10E-05
ENSG00000176842.14	IRX5	-0.824156004	1.347510005	0.044925
ENSG00000263489.1	AC127029.2	-0.825333427	2.650008373	0.002239
ENSG00000273455.1	AC072039.2	-0.825472771	4.762950605	1.73E-05
ENSG00000135407.10	AVIL	-0.825662364	8.575197921	2.66E-09
ENSG00000223837.2	AL645941.1	-0.825778414	5.933865909	1.16E-06
ENSG00000245910.8	SNHG6	-0.826026853	11.959936	1.10E-12
ENSG00000225178.5	RPSAP58	-0.826075039	2.305410573	0.00495
ENSG00000102753.9	KPNA3	-0.826437611	7.740257473	1.82E-08
ENSG00000147123.10	NDUFB11	-0.827137924	11.82600843	1.49E-12
ENSG00000212916.4	MAP10	-0.82717673	8.643195685	2.27E-09
ENSG00000238123.1	MID1IP1-AS1	-0.827258932	7.065116839	8.61E-08
ENSG00000169926.10	KLF13	-0.827923482	10.61153094	2.45E-11
ENSG00000275770.1	MIR6505	-0.827997792	2.396957256	0.004009
ENSG00000215193.12	PEX26	-0.828602265	7.862472961	1.37E-08
ENSG00000125247.15	TMTC4	-0.828997106	12.38180385	4.15E-13
ENSG00000069869.16	NEDD4	-0.829402262	5.894619771	1.27E-06
ENSG00000164068.15	RNF123	-0.829464899	6.631496385	2.34E-07
ENSG00000263342.1	AC003688.2	-0.829523416	3.578631735	0.000264
ENSG00000231806.3	PCAT7	-0.829569386	2.518509039	0.00303
ENSG00000274705.2	MIR486-1	-0.829589541	1.314952458	0.048423
ENSG00000115221.11	ITGB6	-0.829603557	3.575141082	0.000266
ENSG00000274070.1	CASTOR2	-0.829720754	3.996157727	0.000101
ENSG00000187486.5	KCNJ11	-0.830736991	3.438748119	0.000364
ENSG00000185739.13	SRL	-0.830924457	3.665380167	0.000216
ENSG00000106070.18	GRB10	-0.830928991	10.10105724	7.92E-11
ENSG00000237190.3	CDKN2AIPNL	-0.831956482	12.30054763	5.01E-13
ENSG00000250761.2	AC093305.1	-0.832213506	5.251172785	5.61E-06
ENSG00000105258.8	POLR2I	-0.832510991	12.15325922	7.03E-13
ENSG00000227954.6	TARID	-0.832981574	7.041638922	9.09E-08
ENSG00000273466.1	AC012510.1	-0.834849464	6.031069825	9.31E-07
ENSG00000235387.3	SPAAR	-0.835710746	7.128993987	7.43E-08
ENSG00000118298.11	CA14	-0.836617925	1.603449292	0.02492
ENSG00000103852.12	TTC23	-0.836756475	12.00913986	9.79E-13
ENSG00000115556.13	PLCD4	-0.837764886	5.860277226	1.38E-06
ENSG00000165192.13	ASB11	-0.838271727	3.174985554	0.000668
ENSG00000273381.1	AL158071.4	-0.838427059	1.605535434	0.024801
ENSG00000175556.16	LONRF3	-0.838952662	6.235071051	5.82E-07
ENSG00000182685.7	BRICD5	-0.839371011	9.934846147	1.16E-10

ENSG00000164967.9	RPP25L	-0.839742431	8.854988109	1.40E-09
ENSG00000115592.11	PRKAG3	-0.839903176	3.136838414	0.00073
ENSG00000144868.13	TMEM108	-0.841594663	7.265290759	5.43E-08
ENSG00000104848.1	KCNA7	-0.843197739	3.18212626	0.000657
ENSG00000141905.18	NFIC	-0.843223645	13.10637439	7.83E-14
ENSG00000279504.1	AD001527.2	-0.843785314	12.19477498	6.39E-13
ENSG00000164742.15	ADCY1	-0.843844264	6.334127574	4.63E-07
ENSG00000169314.14	C22orf15	-0.844145172	4.899033325	1.26E-05
ENSG00000239900.12	ADSL	-0.84426692	8.917086139	1.21E-09
ENSG00000179922.5	ZNF784	-0.844530789	7.491301887	3.23E-08
ENSG00000257151.1	PWAR6	-0.845546107	5.470614542	3.38E-06
ENSG00000162688.16	AGL	-0.847145077	3.766946857	0.000171
ENSG00000109906.13	ZBTB16	-0.847772893	4.058787392	8.73E-05
ENSG00000116981.3	NT5C1A	-0.84786292	2.222954609	0.005985
ENSG00000175449.13	RFESD	-0.847981054	5.339794629	4.57E-06
ENSG00000157445.14	CACNA2D3	-0.848628195	4.252635702	5.59E-05
ENSG00000259164.1	AC007375.1	-0.849222531	4.800528687	1.58E-05
ENSG00000164591.13	MYOZ3	-0.849719034	3.560614056	0.000275
ENSG00000148702.14	HABP2	-0.850042017	3.42910745	0.000372
ENSG00000138136.6	LBX1	-0.850809502	3.282439491	0.000522
ENSG00000205238.9	SPDYE2	-0.851047195	4.738459123	1.83E-05
ENSG00000276102.1	MIR6747	-0.852117816	9.953768838	1.11E-10
ENSG00000271420.1	AL109936.2	-0.852709041	1.775437808	0.016771
ENSG00000179796.12	LRRC3B	-0.85301226	1.541854423	0.028717
ENSG00000283450.1	MIR486-2	-0.854263868	1.382944334	0.041405
ENSG00000065882.15	TBC1D1	-0.854570286	7.007881365	9.82E-08
ENSG00000182253.14	SYNM	-0.854715847	3.396774531	0.000401
ENSG00000176595.3	KBTBD11	-0.854768809	4.541905132	2.87E-05
ENSG00000255508.7	AP002990.1	-0.855497438	16.72209349	1.90E-17
ENSG00000276488.1	AC008735.4	-0.85628886	2.400908289	0.003973
ENSG00000167311.13	ART5	-0.857987057	3.241276172	0.000574
ENSG00000248015.6	AC005329.1	-0.858422432	8.302328035	4.99E-09
ENSG00000066382.16	MPPED2	-0.858903761	3.454023925	0.000352
ENSG00000120913.23	PDLIM2	-0.859104689	8.247477278	5.66E-09
ENSG00000107537.13	PHYH	-0.859599679	7.701851395	1.99E-08
ENSG00000148377.5	IDI2	-0.859629998	2.09963608	0.00795
ENSG00000132388.12	UBE2G1	-0.859736535	7.83204802	1.47E-08
ENSG00000226833.5	AC092164.1	-0.859841227	4.613998378	2.43E-05
ENSG00000188807.12	TMEM201	-0.860239586	8.339257686	4.58E-09
ENSG00000188735.12	TMEM120B	-0.860968114	13.73564103	1.84E-14
ENSG00000254772.9	EEF1G	-0.861967629	16.60369029	2.49E-17
ENSG00000255650.5	FAM222A-AS1	-0.862574654	1.807753233	0.015569
ENSG00000272894.5	AC004982.2	-0.8634326	8.783764913	1.65E-09
ENSG00000170011.13	MYRIP	-0.863579184	3.724191608	0.000189
ENSG00000229939.1	AL589880.1	-0.863619079	3.230233218	0.000589
ENSG00000259288.6	BUB1B-PAK6	-0.867256255	4.474532812	3.35E-05
ENSG00000184990.12	SIVA1	-0.867434105	21.41134393	3.88E-22

ENSG00000255970.1	LINC02421	-0.867728371	1.362304659	0.043421
ENSG00000130957.4	FBP2	-0.868024164	2.428503887	0.003728
ENSG00000206418.4	RAB12	-0.868063576	12.74652687	1.79E-13
ENSG00000160539.5	PLPP7	-0.868187469	5.152152731	7.04E-06
ENSG00000236901.6	MIR600HG	-0.870097913	8.14552105	7.15E-09
ENSG00000139835.13	GRTP1	-0.870572639	11.52444412	2.99E-12
ENSG00000078295.16	ADCY2	-0.870994845	6.477425644	3.33E-07
ENSG00000276376.1	RF00066	-0.871929591	3.781612783	0.000165
ENSG00000160392.13	C19orf47	-0.872360694	7.9262947	1.18E-08
ENSG00000277449.1	CEBPB-AS1	-0.872667799	5.827747064	1.49E-06
ENSG00000254681.6	PKD1P5	-0.872781735	4.365788735	4.31E-05
ENSG00000176894.9	PXMP2	-0.873051301	8.894918871	1.27E-09
ENSG00000161281.10	COX7A1	-0.873216106	5.574101666	2.67E-06
ENSG00000184545.10	DUSP8	-0.873884467	5.273959736	5.32E-06
ENSG00000188322.4	SBK1	-0.873947441	4.501731448	3.15E-05
ENSG00000149089.12	APIP	-0.874032243	11.65323692	2.22E-12
ENSG00000163293.11	NIPAL1	-0.875710004	4.111833137	7.73E-05
ENSG00000197971.14	MBP	-0.876081401	10.48896051	3.24E-11
ENSG00000070423.17	RNF126	-0.876515901	18.54329139	2.86E-19
ENSG00000129250.11	KIF1C	-0.876971155	5.600686234	2.51E-06
ENSG00000225243.5	AL021026.1	-0.877076803	4.885538915	1.30E-05
ENSG00000224078.13	SNHG14	-0.877163352	7.249240791	5.63E-08
ENSG00000197893.13	NRAP	-0.877389687	3.585351557	0.00026
ENSG00000115286.19	NDUFS7	-0.877521338	8.159319281	6.93E-09
ENSG00000277864.1	SCARNA15	-0.877586641	4.044790884	9.02E-05
ENSG00000280486.1	AC005329.3	-0.877633417	7.633156685	2.33E-08
ENSG00000259820.1	AC083843.2	-0.8782591	5.370105343	4.26E-06
ENSG00000145945.6	FAM50B	-0.880499795	13.19645928	6.36E-14
ENSG00000095059.15	DHPS	-0.881090741	29.80566252	1.56E-30
ENSG00000007314.12	SCN4A	-0.881192608	4.16377348	6.86E-05
ENSG00000175600.15	SUGCT	-0.881214924	8.575197921	2.66E-09
ENSG00000130734.9	ATG4D	-0.881767933	10.05166013	8.88E-11
ENSG00000270605.1	AL353622.1	-0.882939065	3.944646452	0.000114
ENSG00000189366.9	ALG1L	-0.883115688	4.082434794	8.27E-05
ENSG00000069696.6	DRD4	-0.883519141	4.49465121	3.20E-05
ENSG00000284741.1	PDE11A	-0.88353969	2.480800133	0.003305
ENSG00000275131.3	AC241952.1	-0.883884443	6.490840561	3.23E-07
ENSG00000136002.18	ARHGEF4	-0.885788345	12.74652687	1.79E-13
ENSG00000025156.12	HSF2	-0.886315405	12.21488499	6.10E-13
ENSG00000114942.13	EEF1B2	-0.887490299	17.47764582	3.33E-18
ENSG00000047597.6	XK	-0.888661013	3.860269071	0.000138
ENSG00000285815.1	WRB-SH3BGR	-0.888930032	5.212014836	6.14E-06
ENSG00000275329.1	AL138781.2	-0.889134942	2.495084438	0.003198
ENSG00000186051.6	TAL2	-0.889810089	3.717059887	0.000192
ENSG00000259589.2	AC073167.1	-0.89037451	3.916445978	0.000121
ENSG00000267924.1	AC139769.2	-0.891421759	1.872748844	0.013405
ENSG00000139914.6	FITM1	-0.891787362	4.461761789	3.45E-05

ENSG00000257022.1	AC008250.1	-0.89189122	3.679543694	0.000209
ENSG00000100485.11	SOS2	-0.892132237	12.64397217	2.27E-13
ENSG00000182175.14	RGMA	-0.892139038	9.302682284	4.98E-10
ENSG00000100442.10	FKBP3	-0.892322703	7.650021544	2.24E-08
ENSG00000125266.7	EFNB2	-0.893270024	14.86063078	1.38E-15
ENSG00000116898.11	MRPS15	-0.893357422	14.76913991	1.70E-15
ENSG00000279312.1	AL136164.4	-0.895241666	7.384954096	4.12E-08
ENSG00000188981.10	MSANTD1	-0.895275682	6.417622805	3.82E-07
ENSG00000170085.17	SIMC1	-0.895874899	12.44497971	3.59E-13
ENSG00000272070.1	AC005618.1	-0.896855314	2.766722139	0.001711
ENSG00000265728.1	AP001099.1	-0.89687859	1.673073941	0.021229
ENSG00000169762.16	TAPT1	-0.89699059	15.31599797	4.83E-16
ENSG00000164440.14	TXLNB	-0.897405396	4.807311862	1.56E-05
ENSG00000181856.14	SLC2A4	-0.898107364	4.284661051	5.19E-05
ENSG00000270876.1	ZNF30-AS1	-0.899206122	1.860889988	0.013776
ENSG00000133315.10	MACROD1	-0.899921839	6.830733843	1.48E-07
ENSG00000274750.2	HIST1H3E	-0.900091044	1.786670341	0.016343
ENSG00000279989.1	AC011815.3	-0.90025324	1.350682081	0.044598
ENSG00000112561.17	TFEB	-0.901229412	10.99336587	1.02E-11
ENSG00000183571.10	PGPEP1L	-0.901472166	3.065485707	0.00086
ENSG00000065029.14	ZNF76	-0.901867427	17.20181448	6.28E-18
ENSG00000065717.14	TLE2	-0.901896724	10.12716172	7.46E-11
ENSG00000251015.1	SLC25A30-AS1	-0.902539069	4.606988975	2.47E-05
ENSG00000198948.11	MFAP3L	-0.902955037	1.874294664	0.013357
ENSG00000234567.1	AL024497.2	-0.903045743	3.625477138	0.000237
ENSG00000164776.9	PHKG1	-0.903650901	7.525829091	2.98E-08
ENSG00000227495.1	AC004771.1	-0.903673801	5.807549352	1.56E-06
ENSG00000248275.1	TRIM52-AS1	-0.903791514	8.023603953	9.47E-09
ENSG00000169752.16	NRG4	-0.903883235	6.350445461	4.46E-07
ENSG00000180785.9	OR51E1	-0.904835507	2.50972625	0.003092
ENSG00000164398.12	ACSL6	-0.905939306	5.191957326	6.43E-06
ENSG00000170325.14	PRDM10	-0.906064772	17.24405142	5.70E-18
ENSG00000188176.11	SMTNL2	-0.906343928	3.88595784	0.00013
ENSG00000167874.6	TMEM88	-0.906392849	6.771434703	1.69E-07
ENSG00000266708.1	AP001793.1	-0.906457532	11.6704557	2.14E-12
ENSG00000150401.14	DCUN1D2	-0.906477132	8.603461278	2.49E-09
ENSG00000185437.13	SH3BGR	-0.906613806	4.29555204	5.06E-05
ENSG00000169894.17	MUC3A	-0.906662475	3.475889428	0.000334
ENSG00000179909.15	ZNF154	-0.906973604	7.059639415	8.72E-08
ENSG00000188130.13	MAPK12	-0.907109413	8.643195685	2.27E-09
ENSG00000173334.3	TRIB1	-0.90761843	7.08584779	8.21E-08
ENSG00000143153.12	ATP1B1	-0.908063682	5.268590018	5.39E-06
ENSG00000229980.4	TOB1-AS1	-0.909302206	5.334691175	4.63E-06
ENSG00000213707.2	HMGB1P10	-0.909633507	4.210724102	6.16E-05
ENSG00000102878.16	HSF4	-0.911542827	9.340174675	4.57E-10
ENSG00000138162.18	TACC2	-0.912029698	6.470194457	3.39E-07
ENSG00000166896.8	ATP23	-0.912269097	6.509432748	3.09E-07

ENSG00000226314.8	ZNF192P1	-0.912637798	7.060825437	8.69E-08
ENSG00000260604.2	AL590004.3	-0.913889542	1.520152217	0.030189
ENSG00000259661.1	AC068831.4	-0.91504283	12.28683275	5.17E-13
ENSG00000099624.7	ATP5F1D	-0.915276321	8.521957692	3.01E-09
ENSG00000236609.3	ZNF853	-0.916476129	9.464376726	3.43E-10
ENSG00000263011.1	AC108134.4	-0.91668018	3.492924759	0.000321
ENSG00000248235.6	AC037459.1	-0.916961663	9.260474793	5.49E-10
ENSG00000127824.13	TUBA4A	-0.918204814	5.098964634	7.96E-06
ENSG00000161558.10	TMEM143	-0.918209924	6.716543613	1.92E-07
ENSG00000243715.1	CACNA2D3-AS1	-0.918511365	1.599952694	0.025122
ENSG00000225549.3	SELENOKP3	-0.91930649	1.823535117	0.015013
ENSG00000116138.12	DNAJC16	-0.91949455	9.868973366	1.35E-10
ENSG00000065978.18	YBX1	-0.919522817	21.18098133	6.59E-22
ENSG00000123240.16	OPTN	-0.919817674	7.69719661	2.01E-08
ENSG00000172216.5	CEBPB	-0.919848209	6.669027438	2.14E-07
ENSG00000135903.19	PAX3	-0.920216428	4.453105403	3.52E-05
ENSG00000182759.3	MAFA	-0.920266921	3.039399548	0.000913
ENSG00000235070.3	AC062015.1	-0.92377184	1.758405681	0.017442
ENSG00000204789.4	ZNF204P	-0.926368363	7.980531404	1.05E-08
ENSG00000136111.13	TBC1D4	-0.926920048	8.256612443	5.54E-09
ENSG00000261054.1	AC036108.2	-0.927903121	3.612260427	0.000244
ENSG00000135821.18	GLUL	-0.92799391	5.998304666	1.00E-06
ENSG00000278603.1	AC245033.3	-0.92808348	7.820287149	1.51E-08
ENSG00000115159.15	GPD2	-0.930008434	9.997834809	1.00E-10
ENSG00000234132.2	AL358473.2	-0.93075731	3.393179547	0.000404
ENSG00000154358.20	OBSCN	-0.93091843	3.623975109	0.000238
ENSG00000112394.16	SLC16A10	-0.931195073	3.918759978	0.000121
ENSG00000068137.14	PLEKHH3	-0.931289744	12.51016912	3.09E-13
ENSG00000071282.11	LMCD1	-0.931985166	6.775920351	1.68E-07
ENSG00000091262.15	ABCC6	-0.933316892	2.689792222	0.002043
ENSG00000213866.3	YBX1P10	-0.934819297	14.28613068	5.17E-15
ENSG00000269604.1	AC005523.2	-0.938701251	4.434784275	3.67E-05
ENSG00000198467.14	TPM2	-0.939092106	4.904330077	1.25E-05
ENSG00000224861.1	YBX1P1	-0.940002907	15.80549854	1.56E-16
ENSG00000179862.6	CITED4	-0.94089808	6.428718528	3.73E-07
ENSG00000228232.1	GAPDHP1	-0.941631401	4.264466764	5.44E-05
ENSG00000180758.11	GPR157	-0.942461776	5.13265529	7.37E-06
ENSG00000285976.1	AL135905.2	-0.9438532	7.846639404	1.42E-08
ENSG00000162913.9	OBSCN-AS1	-0.944894313	2.989798641	0.001024
ENSG00000228794.8	LINC01128	-0.945449289	7.410597119	3.89E-08
ENSG00000188037.11	CLCN1	-0.946513524	4.234589285	5.83E-05
ENSG00000112245.11	PTP4A1	-0.94677268	7.900455735	1.26E-08
ENSG00000102763.17	VWA8	-0.947073871	7.693863048	2.02E-08
ENSG00000108861.8	DUSP3	-0.947795151	9.368278143	4.28E-10
ENSG00000144354.13	CDCA7	-0.948408779	5.294529107	5.08E-06
ENSG00000141965.4	FEM1A	-0.949123031	4.531764999	2.94E-05
ENSG00000232472.1	EEF1B2P3	-0.949652943	10.55551661	2.78E-11

ENSG00000183091.19	NEB	-0.951493809	4.139711974	7.25E-05
ENSG00000174099.11	MSRB3	-0.951871875	13.18408518	6.55E-14
ENSG00000259429.5	UBE2Q2P2	-0.953489897	4.383339791	4.14E-05
ENSG00000186439.12	TRDN	-0.953706172	3.43034952	0.000371
ENSG00000180730.4	SHISA2	-0.954228712	2.364468406	0.00432
ENSG00000006704.10	GTF2IRD1	-0.955731069	9.754245148	1.76E-10
ENSG00000196547.14	MAN2A2	-0.955978899	12.4460967	3.58E-13
ENSG00000279518.1	AC083843.3	-0.955992732	5.444423212	3.59E-06
ENSG00000185924.6	RTN4RL1	-0.956247975	4.415829727	3.84E-05
ENSG00000188747.8	NOXA1	-0.95848763	9.651136414	2.23E-10
ENSG00000278978.1	AC092611.3	-0.959376447	4.51917862	3.03E-05
ENSG00000267302.5	RNFT1-DT	-0.960409132	6.625725538	2.37E-07
ENSG00000226094.1	RPL7P3	-0.96139135	3.757437393	0.000175
ENSG00000168509.19	HJV	-0.96167647	4.981210516	1.04E-05
ENSG00000269890.1	AL353593.1	-0.96175076	3.50047144	0.000316
ENSG00000175727.13	MLXIP	-0.961977093	11.27983876	5.25E-12
ENSG00000234281.5	LANCL1-AS1	-0.962027205	4.44623685	3.58E-05
ENSG00000120051.14	CFAP58	-0.962745899	4.521690037	3.01E-05
ENSG00000285155.1	AC092153.1	-0.963302709	4.397332434	4.01E-05
ENSG00000137872.16	SEMA6D	-0.96334811	9.482713331	3.29E-10
ENSG00000137843.11	PAK6	-0.964369194	5.343900764	4.53E-06
ENSG00000113328.18	CCNG1	-0.964658809	10.93697559	1.16E-11
ENSG00000162409.10	PRKAA2	-0.965351445	5.740068552	1.82E-06
ENSG00000272975.1	MYHAS	-0.965444513	3.015817146	0.000964
ENSG00000177981.10	ASB8	-0.96547425	12.55334253	2.80E-13
ENSG00000260139.6	CSPG4P13	-0.965603221	1.504284596	0.031312
ENSG00000016391.10	CHDH	-0.966793538	6.777803967	1.67E-07
ENSG00000157502.13	MUM1L1	-0.968629564	6.081536855	8.29E-07
ENSG00000267364.1	AC022706.1	-0.968929888	4.669353722	2.14E-05
ENSG00000278662.4	GOLGA6L10	-0.969292931	2.090007116	0.008128
ENSG00000006025.11	OSBPL7	-0.971735063	6.357983254	4.39E-07
ENSG00000268536.1	AC005523.1	-0.973755245	1.557270104	0.027716
ENSG00000165810.16	BTNL9	-0.976030767	6.903300742	1.25E-07
ENSG00000250479.8	CHCHD10	-0.97623974	6.007762632	9.82E-07
ENSG00000178053.17	MLF1	-0.977909606	3.803790559	0.000157
ENSG00000185028.3	LRRC14B	-0.978010758	3.836336395	0.000146
ENSG00000130822.15	PNCK	-0.979039779	6.299286815	5.02E-07
ENSG00000267480.1	AP001542.3	-0.982293989	12.61870561	2.41E-13
ENSG00000184207.8	PGP	-0.983238137	8.843247261	1.43E-09
ENSG00000170500.12	LONRF2	-0.984297034	8.342627394	4.54E-09
ENSG00000198947.15	DMD	-0.984438551	7.714618105	1.93E-08
ENSG00000165175.15	MID1IP1	-0.984463544	12.57259165	2.68E-13
ENSG00000284966.2	AL138689.2	-0.984620769	15.39897764	3.99E-16
ENSG00000269934.1	AL353593.2	-0.985596598	3.378366727	0.000418
ENSG00000271730.1	AL390208.1	-0.988358559	10.63919404	2.30E-11
ENSG00000224614.1	TNK2-AS1	-0.990581471	15.27653669	5.29E-16
ENSG00000218891.4	ZNF579	-0.992062864	9.254674045	5.56E-10

ENSG00000254833.1	AP001893.3	-0.992647457	2.39849065	0.003995
ENSG00000146701.11	MDH2	-0.993090976	9.852770335	1.40E-10
ENSG00000281938.1	AC026398.1	-0.993846488	4.903019715	1.25E-05
ENSG00000081052.12	COL4A4	-0.993879326	9.860341998	1.38E-10
ENSG00000102804.14	TSC22D1	-0.994132212	10.40043762	3.98E-11
ENSG00000235535.7	TRDN-AS1	-0.99516867	4.842909886	1.44E-05
ENSG00000126882.12	FAM78A	-0.996055578	9.115737162	7.66E-10
ENSG00000061938.17	TNK2	-0.996081313	12.12462137	7.51E-13
ENSG00000146926.10	ASB10	-0.99705256	4.65371757	2.22E-05
ENSG00000215695.1	RSC1A1	-0.997295456	7.567114533	2.71E-08
ENSG00000163126.14	ANKRD23	-0.997559928	5.428794881	3.73E-06
ENSG00000213337.8	ANKRD39	-0.997666217	5.224179391	5.97E-06
ENSG00000105707.13	HPN	-0.999821319	4.813063537	1.54E-05
ENSG00000155090.14	KLF10	-1.000169572	5.143504823	7.19E-06
ENSG00000099875.14	MKNK2	-1.001272373	6.869896477	1.35E-07
ENSG00000162878.12	PKDCC	-1.001471282	8.309093016	4.91E-09
ENSG00000145675.14	PIK3R1	-1.001651104	6.520836183	3.01E-07
ENSG00000080546.13	SESN1	-1.003293588	11.08320032	8.26E-12
ENSG00000160999.10	SH2B2	-1.004132564	7.378790956	4.18E-08
ENSG00000273149.1	AL138963.3	-1.004391909	13.5604763	2.75E-14
ENSG00000272446.5	AL158850.1	-1.004672086	5.288182291	5.15E-06
ENSG00000222371.1	RN7SKP202	-1.004678685	3.114662378	0.000768
ENSG00000165434.7	PGM2L1	-1.00739226	9.676252889	2.11E-10
ENSG00000133112.16	TPT1	-1.008006043	14.05427206	8.83E-15
ENSG00000133135.13	RNF128	-1.008842003	4.016147334	9.64E-05
ENSG00000227392.1	HPN-AS1	-1.009644011	4.216145184	6.08E-05
ENSG00000185345.20	PRKN	-1.009744299	7.94216315	1.14E-08
ENSG00000236085.1	ACTG1P4	-1.010168322	6.305553879	4.95E-07
ENSG00000138379.4	MSTN	-1.011527629	3.875957708	0.000133
ENSG00000205106.4	DKFZp779M0652	-1.012643764	1.965085067	0.010837
ENSG00000213171.2	LINGO4	-1.013801613	3.130315162	0.000741
ENSG00000095587.8	TLL2	-1.0142442	2.619162049	0.002403
ENSG00000154415.7	PPP1R3A	-1.014520555	4.599506096	2.51E-05
ENSG00000261434.1	AC021087.3	-1.01520081	3.388015515	0.000409
ENSG00000275529.1	SNORD116-4	-1.015239377	2.231793017	0.005864
ENSG00000164841.4	TMEM74	-1.016892713	1.50578827	0.031204
ENSG00000236432.7	AC097662.1	-1.019275078	7.747559542	1.79E-08
ENSG00000096060.14	FKBP5	-1.020056331	3.650882657	0.000223
ENSG00000259475.1	AC036108.1	-1.020170511	4.001164776	9.97E-05
ENSG00000283899.1	MIR761	-1.021827831	9.144186683	7.17E-10
ENSG00000172828.12	CES3	-1.022867735	4.930421706	1.17E-05
ENSG00000245330.4	AP005717.1	-1.02321948	8.626055679	2.37E-09
ENSG00000137700.18	SLC37A4	-1.023367324	9.112586803	7.72E-10
ENSG00000183864.4	TOB2	-1.024299812	12.3458744	4.51E-13
ENSG00000283927.1	MIR133A1	-1.025495144	4.036347805	9.20E-05
ENSG00000264490.3	BX640514.1	-1.025612341	2.703547284	0.001979
ENSG00000018625.14	ATP1A2	-1.025723091	5.579891484	2.63E-06

ENSG00000130037.4	KCNA5	-1.026235116	3.330141316	0.000468
ENSG00000147231.13	CXorf57	-1.026306062	6.750017337	1.78E-07
ENSG00000183785.14	TUBA8	-1.026679067	5.616643907	2.42E-06
ENSG00000280007.1	AC008079.1	-1.027046664	6.004713746	9.89E-07
ENSG00000178980.14	SELENOW	-1.027303408	10.72465363	1.89E-11
ENSG00000284453.1	MIR1-2	-1.029191517	4.2348312	5.82E-05
ENSG00000224568.1	LINC01886	-1.030617295	1.491532439	0.032245
ENSG00000268518.1	AC020909.2	-1.031459314	4.639943127	2.29E-05
ENSG00000279168.2	AC105052.4	-1.03407923	3.226983134	0.000593
ENSG00000259065.1	AC005520.2	-1.035477924	8.82606771	1.49E-09
ENSG00000137094.14	DNAJB5	-1.035997828	7.170802405	6.75E-08
ENSG00000280152.1	AC009078.3	-1.038285064	7.468375321	3.40E-08
ENSG00000272510.1	AL121992.3	-1.038490853	9.369766794	4.27E-10
ENSG00000130821.15	SLC6A8	-1.039492941	8.733283487	1.85E-09
ENSG00000152779.13	SLC16A12	-1.039621583	2.732995982	0.001849
ENSG00000201071.1	RF00019	-1.04007403	1.930146597	0.011745
ENSG00000089250.18	NOS1	-1.040522309	2.874295132	0.001336
ENSG00000155792.9	DEPTOR	-1.045288821	10.79780954	1.59E-11
ENSG00000265142.8	MIR133A1HG	-1.046470076	5.319929388	4.79E-06
ENSG00000172379.20	ARNT2	-1.046891409	6.266291027	5.42E-07
ENSG00000164900.4	GBX1	-1.046948425	3.477736953	0.000333
ENSG00000254459.1	AP002812.2	-1.047214438	8.202567005	6.27E-09
ENSG00000269680.1	AC008760.1	-1.048268804	21.39628185	4.02E-22
ENSG00000255449.1	AP002812.5	-1.048386281	8.19658388	6.36E-09
ENSG00000135409.10	AMHR2	-1.048918385	3.797369798	0.000159
ENSG00000104267.9	CA2	-1.049179627	5.844043007	1.43E-06
ENSG00000196091.13	MYBPC1	-1.049189844	4.778363408	1.67E-05
ENSG00000072195.14	SPEG	-1.051619811	4.625294524	2.37E-05
ENSG00000237159.5	CNTFR-AS1	-1.053272511	2.727804911	0.001872
ENSG00000106031.8	HOXA13	-1.055151245	3.288658054	0.000514
ENSG00000273156.1	AC124016.2	-1.05574066	3.065126281	0.000861
ENSG00000087884.14	AAMDC	-1.056009716	8.660185609	2.19E-09
ENSG00000232926.1	AC000078.1	-1.056999311	5.741375713	1.81E-06
ENSG00000072954.6	TMEM38A	-1.059980933	5.906380923	1.24E-06
ENSG00000266964.5	FXYD1	-1.06070858	12.26714256	5.41E-13
ENSG00000069011.15	PITX1	-1.061745265	2.769267035	0.001701
ENSG00000165152.8	TMEM246	-1.062716609	12.18877558	6.47E-13
ENSG00000189241.6	TSPYL1	-1.062976057	10.13373764	7.35E-11
ENSG00000177791.11	MYOZ1	-1.064478997	5.839295786	1.45E-06
ENSG00000238133.6	MAP3K20-AS1	-1.066050372	4.854494157	1.40E-05
ENSG00000111640.14	GAPDH	-1.066484545	7.274397205	5.32E-08
ENSG00000221857.7	AC020907.2	-1.068282121	12.27679586	5.29E-13
ENSG00000178568.14	ERBB4	-1.068759472	5.469808359	3.39E-06
ENSG00000238923.1	RNU7-1	-1.069580635	1.978334955	0.010512
ENSG00000234899.9	SOX9-AS1	-1.070411624	5.352090101	4.45E-06
ENSG00000184058.14	TBX1	-1.070733705	2.423688389	0.00377
ENSG00000125733.17	TRIP10	-1.071230367	22.72987208	1.86E-23

ENSG00000251603.1	AC092611.2	-1.071453125	4.356022434	4.41E-05
ENSG00000240038.6	AMY2B	-1.076186638	21.10350168	7.88E-22
ENSG00000277399.4	GPR179	-1.076684772	1.637697542	0.02303
ENSG00000257647.1	AC124312.2	-1.077150949	1.338433872	0.045874
ENSG00000148339.12	SLC25A25	-1.077614646	6.319318263	4.79E-07
ENSG00000225151.10	GOLGA2P7	-1.078736449	4.959069212	1.10E-05
ENSG00000145626.11	UGT3A1	-1.079970091	2.462848512	0.003445
ENSG00000257261.5	AC008014.1	-1.080382114	6.691274318	2.04E-07
ENSG00000228470.1	AL929236.1	-1.080584793	2.495147471	0.003198
ENSG00000280332.1	AC020917.4	-1.082808958	11.23425906	5.83E-12
ENSG00000110693.17	SOX6	-1.086149028	9.814291538	1.53E-10
ENSG00000272703.1	AP005137.2	-1.08861421	4.321222102	4.77E-05
ENSG00000126803.9	HSPA2	-1.089401538	5.241182725	5.74E-06
ENSG00000269968.1	AC006064.4	-1.089941425	7.623470855	2.38E-08
ENSG00000005981.12	ASB4	-1.090422815	4.456955585	3.49E-05
ENSG00000156885.5	COX6A2	-1.092378963	5.701086391	1.99E-06
ENSG00000257514.5	AC117505.1	-1.092478343	4.987480319	1.03E-05
ENSG00000152430.17	BOLL	-1.09330135	5.211354116	6.15E-06
ENSG00000226101.1	LINC02097	-1.095027452	4.23170413	5.87E-05
ENSG00000168333.13	PPDPFL	-1.095808364	3.291981521	0.000511
ENSG00000261863.1	LINC01996	-1.100177317	3.455441352	0.00035
ENSG00000198881.9	ASB12	-1.101119033	6.876114136	1.33E-07
ENSG00000101400.5	SNTA1	-1.102182378	7.503484423	3.14E-08
ENSG00000277543.1	AC026471.5	-1.102724569	5.780648577	1.66E-06
ENSG00000180209.11	MYLPF	-1.10332893	4.993125355	1.02E-05
ENSG00000161649.12	CD300LG	-1.103539248	5.54983209	2.82E-06
ENSG00000276314.1	SNORD107	-1.104181661	2.422821485	0.003777
ENSG00000174429.3	ABRA	-1.104689809	3.590957628	0.000256
ENSG00000187583.10	PLEKHN1	-1.10489006	4.156002965	6.98E-05
ENSG00000135447.16	PPP1R1A	-1.105090127	5.468324253	3.40E-06
ENSG00000127324.8	TSPAN8	-1.105362476	4.9295358	1.18E-05
ENSG00000189134.3	NKAPL	-1.107533751	13.67947324	2.09E-14
ENSG00000249491.1	EGFLAM-AS1	-1.108158339	4.999784847	1.00E-05
ENSG00000279489.1	AL355377.2	-1.108285804	2.69383405	0.002024
ENSG00000261616.1	AC036108.3	-1.108368927	8.35669573	4.40E-09
ENSG00000108823.15	SGCA	-1.109820285	8.9828065	1.04E-09
ENSG00000260398.1	AC068700.1	-1.111572553	4.747192918	1.79E-05
ENSG00000244256.3	RN7SL130P	-1.112279707	2.553769728	0.002794
ENSG00000171992.12	SYNPO	-1.113918806	6.545974805	2.84E-07
ENSG00000133878.8	DUSP26	-1.11426011	6.216687772	6.07E-07
ENSG00000258461.5	AC012651.1	-1.114470699	8.408568008	3.90E-09
ENSG00000166289.5	PLEKHF1	-1.116425463	10.27731867	5.28E-11
ENSG00000246596.7	AC139795.1	-1.117810562	9.241362989	5.74E-10
ENSG00000267557.1	AC008474.1	-1.118607877	2.732655728	0.001851
ENSG00000200170.1	RF00019	-1.119096473	2.816325346	0.001526
ENSG00000136279.20	DBNL	-1.119316076	7.776582054	1.67E-08
ENSG00000092529.23	CAPN3	-1.119670407	8.259688259	5.50E-09

ENSG00000246465.1	AC138904.1	-1.120730347	5.194539787	6.39E-06
ENSG00000104381.12	GDAP1	-1.122175495	10.12507594	7.50E-11
ENSG00000175564.12	UCP3	-1.124408745	4.111175223	7.74E-05
ENSG00000268555.1	AC123912.4	-1.125172494	1.887280422	0.012963
ENSG00000187642.9	PERM1	-1.128156706	4.015301907	9.65E-05
ENSG00000276445.1	AC005393.1	-1.128434976	5.134845901	7.33E-06
ENSG00000199477.1	SNORA31	-1.128540575	9.413661175	3.86E-10
ENSG00000100628.11	ASB2	-1.13000947	5.029321303	9.35E-06
ENSG00000149596.6	JPH2	-1.130814911	5.837126312	1.46E-06
ENSG00000230102.7	LINC02028	-1.13281814	2.160980336	0.006903
ENSG00000254519.3	AC044839.1	-1.133403554	3.431582514	0.00037
ENSG00000179094.15	PER1	-1.134234282	6.112338376	7.72E-07
ENSG00000262769.1	AC025627.1	-1.137089023	6.036725496	9.19E-07
ENSG00000229618.2	AC011287.1	-1.137334122	2.026441082	0.009409
ENSG00000117480.15	FAAH	-1.140052663	15.92503984	1.19E-16
ENSG00000172731.13	LRRC20	-1.141327654	6.438556777	3.64E-07
ENSG00000284214.1	MIR29C	-1.143547487	8.500402766	3.16E-09
ENSG00000204179.10	PTPN20	-1.144818008	5.56045565	2.75E-06
ENSG00000105711.11	SCN1B	-1.147127839	10.87884818	1.32E-11
ENSG00000142856.16	ITGB3BP	-1.149731778	7.891674733	1.28E-08
ENSG00000285373.1	LINC02478	-1.149866815	3.78647705	0.000164
ENSG00000229619.3	MBNL1-AS1	-1.150179979	8.738048899	1.83E-09
ENSG00000106113.18	CRHR2	-1.150926718	5.561085657	2.75E-06
ENSG00000147697.8	GSDMC	-1.152686113	3.387368379	0.00041
ENSG00000125848.9	FLRT3	-1.152745864	6.428025315	3.73E-07
ENSG00000137198.9	GMPR	-1.154980768	8.492119831	3.22E-09
ENSG00000141469.17	SLC14A1	-1.156025711	6.420400085	3.80E-07
ENSG00000166407.13	LMO1	-1.157355861	3.677256199	0.00021
ENSG00000258987.1	AL132642.1	-1.162448188	5.282609976	5.22E-06
ENSG00000175984.14	DENND2C	-1.162623554	7.688791953	2.05E-08
ENSG00000197530.12	MIB2	-1.162755596	19.91351042	1.22E-20
ENSG00000142494.13	SLC47A1	-1.163872418	8.714545894	1.93E-09
ENSG00000204396.10	VWA7	-1.164123246	6.564231132	2.73E-07
ENSG00000234438.4	KBTBD13	-1.165406027	4.388440045	4.09E-05
ENSG00000214097.4	SMCO1	-1.168998884	5.360434529	4.36E-06
ENSG00000079739.16	PGM1	-1.169545864	7.18073386	6.60E-08
ENSG00000196781.14	TLE1	-1.171078372	8.80412882	1.57E-09
ENSG00000280233.1	AC015813.7	-1.171221493	3.687317564	0.000205
ENSG00000234758.1	AC034228.3	-1.171457653	1.899069755	0.012616
ENSG00000188338.14	SLC38A3	-1.172619395	3.576496549	0.000265
ENSG00000244295.2	RPS20P21	-1.173110613	5.173762534	6.70E-06
ENSG00000188580.14	NKAIN2	-1.174990132	4.444009396	3.60E-05
ENSG00000187837.3	HIST1H1C	-1.176337814	11.39294523	4.05E-12
ENSG00000272369.1	AC008035.1	-1.177082762	6.822624691	1.50E-07
ENSG00000283689.1	AC018553.2	-1.178139397	1.79845223	0.015906
ENSG00000101306.10	MYLK2	-1.178490963	4.899033325	1.26E-05
ENSG00000127252.5	HRASLS	-1.181474233	6.197241945	6.35E-07

ENSG00000170577.7	SIX2	-1.182653878	13.38814846	4.09E-14
ENSG00000189431.7	RASSF10	-1.182962979	1.556302215	0.027778
ENSG00000163995.19	ABLIM2	-1.182966776	7.660877888	2.18E-08
ENSG00000235587.2	GAPDHP65	-1.186316415	3.896637737	0.000127
ENSG00000116771.5	AGMAT	-1.186835499	5.146052046	7.14E-06
ENSG00000144908.13	ALDH1L1	-1.188080797	6.230972163	5.88E-07
ENSG00000134240.11	HMGCS2	-1.190176608	1.654347827	0.022164
ENSG00000142675.17	CNKSR1	-1.191628811	7.670345248	2.14E-08
ENSG00000261069.3	AC124312.3	-1.193834585	5.352090101	4.45E-06
ENSG00000118514.13	ALDH8A1	-1.194407443	3.383446478	0.000414
ENSG00000265787.2	CYP4F35P	-1.198133398	2.288397633	0.005148
ENSG00000107147.12	KCNT1	-1.198839328	5.965999412	1.08E-06
ENSG00000058404.19	CAMK2B	-1.199271149	6.265876759	5.42E-07
ENSG00000179271.2	GADD45GIP1	-1.199408569	20.53722733	2.90E-21
ENSG00000130005.12	GAMT	-1.19992123	8.267092659	5.41E-09
ENSG00000284203.1	MIR29B2	-1.200697904	3.526561557	0.000297
ENSG00000107758.15	PPP3CB	-1.202164337	16.94265206	1.14E-17
ENSG00000164708.5	PGAM2	-1.203199593	5.947518599	1.13E-06
ENSG00000144644.14	GADL1	-1.204379571	4.84661189	1.42E-05
ENSG00000162650.16	ATXN7L2	-1.206126131	11.97156432	1.07E-12
ENSG00000239775.1	AC017116.1	-1.206518755	5.898520825	1.26E-06
ENSG00000161896.11	IP6K3	-1.20747067	7.132267428	7.37E-08
ENSG00000228804.5	AC072022.1	-1.207749532	10.99790532	1.00E-11
ENSG00000207197.1	SNORD116-12	-1.209313114	3.937678494	0.000115
ENSG00000263370.1	AC104564.1	-1.211250021	5.239429587	5.76E-06
ENSG00000057294.14	PKP2	-1.211548887	6.18281106	6.56E-07
ENSG00000258584.2	FAM181A-AS1	-1.211599722	1.97181287	0.010671
ENSG00000224097.5	AC021148.1	-1.212444963	2.723166871	0.001892
ENSG00000119938.8	PPP1R3C	-1.212573949	8.257392433	5.53E-09
ENSG00000255468.7	AP001107.9	-1.21324667	10.26872889	5.39E-11
ENSG00000273597.1	AL355377.1	-1.214396423	1.9329266	0.01167
ENSG00000205236.6	AC105052.1	-1.215496034	8.984406564	1.04E-09
ENSG00000143365.17	RORC	-1.215992482	6.993091577	1.02E-07
ENSG00000141401.11	IMPA2	-1.217317324	12.84242703	1.44E-13
ENSG00000203709.11	MIR29B2CHG	-1.221768774	11.11782256	7.62E-12
ENSG00000283928.1	MIR637	-1.224115957	7.272448912	5.34E-08
ENSG00000253051.1	RF00322	-1.227522105	10.22484062	5.96E-11
ENSG00000101134.11	DOK5	-1.227935542	9.211103871	6.15E-10
ENSG00000284117.1	MIR6883	-1.22921752	1.382489585	0.041449
ENSG00000196104.10	SPOCK3	-1.229408702	1.353012021	0.04436
ENSG00000125878.6	TCF15	-1.230313111	6.174692671	6.69E-07
ENSG00000279609.1	AL158070.2	-1.231145098	4.971648823	1.07E-05
ENSG00000284094.1	MIR6073	-1.231337509	7.828271024	1.49E-08
ENSG00000225083.1	GRTP1-AS1	-1.231688682	9.804988681	1.57E-10
ENSG00000175946.8	KLHL38	-1.231858571	5.443816348	3.60E-06
ENSG00000203875.11	SNHG5	-1.234056436	10.05755604	8.76E-11
ENSG00000158571.10	PFKFB1	-1.235671119	7.565202523	2.72E-08

ENSG00000254343.2	AC091563.1	-1.23586158	12.47900447	3.32E-13
ENSG00000248538.7	AC022784.1	-1.236113155	11.44329862	3.60E-12
ENSG00000173281.4	PPP1R3B	-1.236157461	11.19804228	6.34E-12
ENSG00000101210.11	EEF1A2	-1.236182634	7.277342451	5.28E-08
ENSG00000243305.1	AC026347.1	-1.23860992	1.548409902	0.028287
ENSG00000279619.1	AC020907.5	-1.24106458	15.65195297	2.23E-16
ENSG00000270919.1	AC108451.2	-1.244488158	1.565880627	0.027172
ENSG00000277494.1	GPIHBP1	-1.24635046	11.49937864	3.17E-12
ENSG00000155367.15	PPM1J	-1.248087389	6.31179634	4.88E-07
ENSG00000198722.14	UNC13B	-1.250961585	21.27364392	5.33E-22
ENSG00000164976.8	MYORG	-1.251208166	6.121146228	7.57E-07
ENSG00000274124.1	AC074029.3	-1.252002884	2.028990546	0.009354
ENSG00000250218.1	ALDH1L1-AS1	-1.252397872	6.254829897	5.56E-07
ENSG00000081248.10	CACNA1S	-1.256318516	7.508215956	3.10E-08
ENSG00000006788.13	MYH13	-1.256711515	4.970917705	1.07E-05
ENSG00000196218.12	RYR1	-1.258210105	6.126248108	7.48E-07
ENSG00000114315.3	HES1	-1.261573518	10.27950686	5.25E-11
ENSG00000182568.16	SATB1	-1.261991438	14.5770942	2.65E-15
ENSG00000204219.10	TCEA3	-1.263735898	11.1596904	6.92E-12
ENSG00000266844.1	AC093330.1	-1.263878552	9.473576048	3.36E-10
ENSG00000285781.1	AL117340.1	-1.264847061	3.870337582	0.000135
ENSG00000151655.18	ITIH2	-1.268867608	5.774438897	1.68E-06
ENSG00000183386.9	FHL3	-1.269423584	6.112338376	7.72E-07
ENSG00000086967.9	MYBPC2	-1.270048852	4.689265527	2.05E-05
ENSG00000149654.9	CDH22	-1.270698081	1.390024066	0.040736
ENSG00000278156.1	TSC22D1-AS1	-1.273545589	7.310160765	4.90E-08
ENSG00000269445.1	AC067969.2	-1.275585013	6.293271072	5.09E-07
ENSG00000250222.1	AC008443.4	-1.279356035	13.84156106	1.44E-14
ENSG00000265158.1	LRRC37A7P	-1.279663218	3.8893854	0.000129
ENSG00000174669.11	SLC29A2	-1.282795553	9.154321134	7.01E-10
ENSG00000143632.14	ACTA1	-1.285788001	6.791230758	1.62E-07
ENSG00000164237.8	CMBL	-1.291804933	9.844165917	1.43E-10
ENSG00000186326.3	RGS9BP	-1.293930787	4.375922618	4.21E-05
ENSG00000068976.13	PYGM	-1.296926486	6.69994424	2.00E-07
ENSG00000237298.9	TTN-AS1	-1.303878222	6.078880347	8.34E-07
ENSG00000123689.5	G0S2	-1.31017871	4.839988061	1.45E-05
ENSG00000236508.1	ATP13A5-AS1	-1.31647927	4.411604093	3.88E-05
ENSG00000149925.18	ALDOA	-1.322906095	7.716332043	1.92E-08
ENSG00000285043.1	AC093512.2	-1.323180253	7.713877097	1.93E-08
ENSG00000267784.1	AC010680.1	-1.326676786	7.119205924	7.60E-08
ENSG00000271952.1	LINC01954	-1.32816388	1.312100665	0.048742
ENSG00000184185.9	KCNJ12	-1.329161795	8.859811579	1.38E-09
ENSG00000169031.19	COL4A3	-1.330272605	11.65051255	2.24E-12
ENSG00000146809.12	ASB15	-1.332466035	6.750815547	1.77E-07
ENSG00000146054.17	TRIM7	-1.333185455	10.9250727	1.19E-11
ENSG00000132702.12	HAPLN2	-1.333891668	1.801776438	0.015784
ENSG00000250994.1	AC005355.1	-1.333945789	2.249657541	0.005628

ENSG00000248738.6	AC037441.1	-1.336700032	3.868960798	0.000135
ENSG00000108515.17	ENO3	-1.336812423	7.924242501	1.19E-08
ENSG00000229659.1	RPL26P6	-1.339907108	6.57416963	2.67E-07
ENSG00000141526.16	SLC16A3	-1.341404833	5.645186676	2.26E-06
ENSG00000254340.1	AC022784.5	-1.345056266	8.404807684	3.94E-09
ENSG00000246022.2	ALDH1L1-AS2	-1.345470649	6.479836726	3.31E-07
ENSG00000224843.6	LINC00240	-1.346322682	3.669253922	0.000214
ENSG00000267417.1	AC138474.1	-1.350163897	5.991094254	1.02E-06
ENSG00000113916.17	BCL6	-1.35109502	11.59914793	2.52E-12
ENSG00000166343.9	MSS51	-1.353040735	7.502703791	3.14E-08
ENSG00000252061.1	RNU6-415P	-1.353571019	2.107148766	0.007814
ENSG00000177548.12	RABEP2	-1.35428996	13.9566147	1.11E-14
ENSG00000140986.7	RPL3L	-1.354450955	8.48676216	3.26E-09
ENSG00000104369.4	JPH1	-1.359938683	7.913785183	1.22E-08
ENSG00000155657.26	TTN	-1.36610986	7.108887657	7.78E-08
ENSG00000272732.1	AC004982.1	-1.366846639	3.400842552	0.000397
ENSG00000262006.1	AC005920.4	-1.367067669	1.681427755	0.020824
ENSG00000164142.15	FAM160A1	-1.367948036	6.233682057	5.84E-07
ENSG00000260047.2	BCAP31P2	-1.368294371	2.456455279	0.003496
ENSG00000122756.14	CNTFR	-1.370207816	13.01674522	9.62E-14
ENSG00000187527.10	ATP13A5	-1.370647013	6.100002116	7.94E-07
ENSG00000188993.3	LRRC66	-1.373431091	15.63968396	2.29E-16
ENSG00000186481.16	ANKRD20A5P	-1.374566705	5.125336077	7.49E-06
ENSG00000241735.2	FABP5P3	-1.375044797	3.93930201	0.000115
ENSG00000060138.12	YBX3	-1.375900982	13.17594979	6.67E-14
ENSG00000272949.1	AC093668.2	-1.376829599	13.91089282	1.23E-14
ENSG00000105808.17	RASA4	-1.377606181	11.08407703	8.24E-12
ENSG00000283294.1	AP005212.4	-1.377727272	3.226459952	0.000594
ENSG00000130595.18	TNNT3	-1.385273158	6.840435196	1.44E-07
ENSG00000185100.10	ADSSL1	-1.385765041	8.004806719	9.89E-09
ENSG00000101470.9	TNNC2	-1.386478562	6.678904561	2.09E-07
ENSG00000277785.1	SNORD116-21	-1.387879975	2.660795239	0.002184
ENSG00000250041.2	AC069360.1	-1.39046189	8.446955302	3.57E-09
ENSG00000263772.1	AC017100.2	-1.392368944	2.431936996	0.003699
ENSG00000278944.1	AC027045.1	-1.392687976	6.441031446	3.62E-07
ENSG00000184544.11	DHRS7C	-1.392910974	7.315155219	4.84E-08
ENSG00000230442.5	AC006333.1	-1.393663768	7.847643012	1.42E-08
ENSG00000177508.11	IRX3	-1.394715835	3.922164094	0.00012
ENSG00000227258.5	SMIM2-AS1	-1.399060067	3.497564244	0.000318
ENSG00000279192.1	PWAR5	-1.402465043	5.632714461	2.33E-06
ENSG00000285938.1	AC072022.2	-1.404039214	9.075702913	8.40E-10
ENSG00000072422.16	RHOBTB1	-1.408303147	11.01219125	9.72E-12
ENSG00000270885.1	RASL10B	-1.40886478	7.14083506	7.23E-08
ENSG00000105643.9	ARRDC2	-1.414341837	12.01488967	9.66E-13
ENSG00000231167.3	YBX1P2	-1.416057186	1.922580952	0.011951
ENSG00000285844.1	FO393414.3	-1.425028208	4.751015925	1.77E-05
ENSG00000236496.2	GPS2P1	-1.425479	1.726554716	0.018769

ENSG00000157782.9	CABP1	-1.427163909	4.600684445	2.51E-05
ENSG00000213225.7	NOC2LP1	-1.435494997	1.97877039	0.010501
ENSG00000179262.9	RAD23A	-1.438658197	18.00460573	9.89E-19
ENSG00000274904.1	AC093512.1	-1.439338761	7.597267133	2.53E-08
ENSG00000104879.4	CKM	-1.441791361	7.910347431	1.23E-08
ENSG00000169184.5	MN1	-1.442489812	15.17974464	6.61E-16
ENSG00000279289.1	AL136164.3	-1.442604254	2.925719184	0.001187
ENSG00000284479.1	AC009477.2	-1.443973055	1.496906279	0.031849
ENSG00000145949.10	MYLK4	-1.445854283	4.393321483	4.04E-05
ENSG00000169758.12	TMEM266	-1.447974026	11.08523592	8.22E-12
ENSG00000279172.1	AC020904.3	-1.448868768	10.79129796	1.62E-11
ENSG00000004468.12	CD38	-1.452592449	14.63445733	2.32E-15
ENSG00000214970.8	AC005323.1	-1.457473071	6.217290532	6.06E-07
ENSG00000163673.7	DCLK3	-1.463055868	4.929209454	1.18E-05
ENSG00000080709.15	KCNN2	-1.464507077	4.171769053	6.73E-05
ENSG00000140416.20	TPM1	-1.46535614	8.519580362	3.02E-09
ENSG00000178821.12	TMEM52	-1.467696079	8.12086938	7.57E-09
ENSG00000105204.13	DYRK1B	-1.468580079	14.58027155	2.63E-15
ENSG00000185760.15	KCNQ5	-1.468983254	8.132230317	7.38E-09
ENSG00000108342.12	CSF3	-1.469800815	2.605364142	0.002481
ENSG00000223774.5	AL513217.1	-1.471655172	12.77500741	1.68E-13
ENSG00000124374.8	PAIP2B	-1.479443994	7.516020927	3.05E-08
ENSG00000259986.1	AC103876.1	-1.481768342	1.324295409	0.047392
ENSG00000271141.1	AC010680.4	-1.49176777	7.676340267	2.11E-08
ENSG00000146477.5	SLC22A3	-1.4989252	8.547149793	2.84E-09
ENSG00000119125.16	GDA	-1.505185934	2.941697362	0.001144
ENSG00000279668.2	AC024610.2	-1.505978726	2.433797175	0.003683
ENSG00000227456.7	LINC00310	-1.50827777	11.6659918	2.16E-12
ENSG00000259422.1	AC091100.1	-1.510441228	8.218482666	6.05E-09
ENSG00000279551.1	AC025263.3	-1.510955012	1.399032645	0.039899
ENSG00000164309.14	CMYA5	-1.513917162	9.883297343	1.31E-10
ENSG00000261217.2	BCAP31P1	-1.517685185	3.620393878	0.00024
ENSG00000250900.6	AC008443.5	-1.518387427	12.15131818	7.06E-13
ENSG00000003989.17	SLC7A2	-1.519900947	10.37562787	4.21E-11
ENSG00000170525.20	PFKFB3	-1.520076667	6.515705632	3.05E-07
ENSG00000130598.15	TNNI2	-1.527115369	6.977655178	1.05E-07
ENSG00000172361.5	CFAP53	-1.534234109	7.626328417	2.36E-08
ENSG00000163431.12	LMOD1	-1.53767206	13.54023105	2.88E-14
ENSG00000265356.2	AC004147.4	-1.538147255	1.834356973	0.014643
ENSG00000148795.6	CYP17A1	-1.538726404	12.58537644	2.60E-13
ENSG00000267423.1	AC005616.1	-1.540362511	5.345696173	4.51E-06
ENSG00000228366.1	AL592437.1	-1.547769542	5.017663585	9.60E-06
ENSG00000084628.9	NKAIN1	-1.553758805	8.332354305	4.65E-09
ENSG00000233996.1	AC013439.1	-1.560498013	2.071451846	0.008483
ENSG00000268055.1	AC067969.1	-1.561769046	7.548239337	2.83E-08
ENSG00000233297.4	RASA4DP	-1.562948597	5.779974774	1.66E-06
ENSG00000162552.14	WNT4	-1.563849938	11.83641481	1.46E-12

ENSG00000134020.7	PEBP4	-1.573522965	11.13864489	7.27E-12
ENSG00000156463.17	SH3RF2	-1.573601244	10.08841853	8.16E-11
ENSG00000129204.16	USP6	-1.579191723	10.36895545	4.28E-11
ENSG00000100373.9	UPK3A	-1.584695136	6.860212007	1.38E-07
ENSG00000174407.13	MIR1-1HG	-1.584931067	8.420313615	3.80E-09
ENSG00000172318.5	B3GALT1	-1.59187457	6.162964164	6.87E-07
ENSG00000270681.1	AC095055.1	-1.595245968	2.864034123	0.001368
ENSG00000261614.1	YBX3P1	-1.596697771	5.167385752	6.80E-06
ENSG00000224884.1	AC034187.1	-1.601762767	5.427806883	3.73E-06
ENSG00000239732.3	TLR9	-1.608321819	16.1041636	7.87E-17
ENSG00000284508.1	MIR133A2	-1.618535967	8.318739705	4.80E-09
ENSG00000270574.1	AC010680.2	-1.620695322	9.529793621	2.95E-10
ENSG00000231585.1	AC034228.2	-1.631733488	1.310465456	0.048925
ENSG00000101542.9	CDH20	-1.635390186	3.417381571	0.000382
ENSG00000170667.14	RASA4B	-1.646140234	13.80727506	1.56E-14
ENSG00000196581.10	AJAP1	-1.653538458	2.139501967	0.007253
ENSG00000107165.12	TYRP1	-1.659734601	1.637999853	0.023014
ENSG00000265768.1	MIR4506	-1.669579292	3.056172091	0.000879
ENSG00000125414.18	MYH2	-1.685325352	7.858806184	1.38E-08
ENSG00000199017.2	MIR1-1	-1.691695028	1.594096585	0.025463
ENSG00000271011.1	AC010680.3	-1.697678216	7.68327397	2.07E-08
ENSG00000130222.10	GADD45G	-1.703759903	7.266083652	5.42E-08
ENSG00000157087.18	ATP2B2	-1.703825705	8.165940721	6.82E-09
ENSG00000250105.1	AP002748.1	-1.711029158	3.659008539	0.000219
ENSG00000248514.1	AC008443.3	-1.718419793	12.29832455	5.03E-13
ENSG00000248746.5	ACTN3	-1.721722707	3.425139882	0.000376
ENSG00000272736.5	AC005323.2	-1.733633886	8.110587016	7.75E-09
ENSG00000266265.3	KLF14	-1.751280744	2.405918895	0.003927
ENSG00000107859.9	PITX3	-1.752535042	10.30004528	5.01E-11
ENSG00000185269.11	NOTUM	-1.762753053	7.482332512	3.29E-08
ENSG00000237372.2	UNQ6494	-1.772778231	1.843798072	0.014329
ENSG00000266385.1	AC005899.5	-1.784351607	1.503445905	0.031373
ENSG00000109061.9	MYH1	-1.787878459	4.225204905	5.95E-05
ENSG00000163491.16	NEK10	-1.79195725	13.78037197	1.66E-14
ENSG00000229065.1	AL354893.2	-1.792370855	1.351773051	0.044486
ENSG00000215018.9	COL28A1	-1.802977473	15.33344739	4.64E-16
ENSG00000141576.15	RNF157	-1.819398128	12.46193826	3.45E-13
ENSG00000260442.5	ATP2A1-AS1	-1.824000984	8.151883716	7.05E-09
ENSG00000267128.1	RNF157-AS1	-1.828151229	12.62902569	2.35E-13
ENSG00000159259.7	CHAF1B	-1.830529989	17.74966315	1.78E-18
ENSG00000196296.13	ATP2A1	-1.831247255	8.267822656	5.40E-09
ENSG00000156219.16	ART3	-1.858373653	15.27534552	5.30E-16
ENSG00000171811.13	CFAP46	-1.858875735	8.588073496	2.58E-09
ENSG00000171885.14	AQP4	-1.882780414	8.282430859	5.22E-09
ENSG00000258545.5	RHOXF1-AS1	-1.899997632	4.166648459	6.81E-05
ENSG00000262855.1	AC012146.4	-1.900890022	10.81410133	1.53E-11
ENSG00000279598.1	AC009948.5	-1.929699031	6.542335439	2.87E-07

ENSG00000259495.2	AC016705.2	-1.939757078	5.073282225	8.45E-06
ENSG00000181781.9	ODF3L2	-1.957916815	10.90840809	1.23E-11
ENSG00000270277.1	AC009948.3	-1.964780556	6.658284227	2.20E-07
ENSG00000250878.3	METTL21EP	-1.980581804	5.657924506	2.20E-06
ENSG00000188613.6	NANOS1	-2.048168662	13.09915179	7.96E-14
ENSG00000226413.2	OR8T1P	-2.067881009	1.851873599	0.014065
ENSG00000273424.1	AL008582.1	-2.073446642	2.049238229	0.008928
ENSG00000164588.6	HCN1	-2.088759757	3.679246981	0.000209
ENSG00000215187.10	FAM166B	-2.110181214	12.27679586	5.29E-13
ENSG00000183783.6	KCTD8	-2.12954767	3.829245731	0.000148
ENSG00000270956.1	AC009948.4	-2.165314617	6.510577039	3.09E-07
ENSG00000047936.10	ROS1	-2.180329373	3.476278986	0.000334
ENSG00000219186.2	FTH1P19	-2.182299252	1.751800135	0.017709
ENSG00000163032.11	VSNL1	-2.190390466	3.350343309	0.000446
ENSG00000278445.1	AL137246.2	-2.195053261	6.813657988	1.54E-07
ENSG00000282024.1	AL009031.1	-2.214307319	3.715776221	0.000192
ENSG00000284791.1	OR7E47P	-2.221968409	11.53443367	2.92E-12
ENSG00000150783.9	TEX12	-2.225442086	2.08248082	0.00827
ENSG00000285873.1	AL445253.1	-2.227573318	3.695989791	0.000201
ENSG00000175262.14	C1orf127	-2.230265687	16.95854922	1.10E-17
ENSG00000257542.5	OR7E47P	-2.247407913	11.17663013	6.66E-12
ENSG00000284574.1	MIR6787	-2.293987431	1.922306134	0.011959
ENSG00000188536.13	HBA2	-2.30614573	5.518507	3.03E-06
ENSG00000089101.17	CFAP61	-2.339710695	17.40728254	3.91E-18
ENSG00000203952.9	CCDC160	-2.341039757	1.481767465	0.032979
ENSG00000255426.1	AC044839.3	-2.346910042	4.028369059	9.37E-05
ENSG00000150750.7	C11orf53	-2.36226017	1.45743424	0.034879
ENSG00000218073.1	AL021407.3	-2.386414067	1.438247556	0.036455
ENSG00000229425.2	AJ009632.2	-2.390047105	2.899725603	0.00126
ENSG00000244734.4	HBB	-2.408104622	6.468731003	3.40E-07
ENSG00000259664.2	LINC02254	-2.433129588	1.611222577	0.024478
ENSG00000198574.5	SH2D1B	-2.451337009	7.185185337	6.53E-08
ENSG00000206172.8	HBA1	-2.454266063	6.430000259	3.72E-07
ENSG00000137766.17	UNC13C	-2.464631661	5.255568721	5.55E-06
ENSG00000232451.1	AC018467.1	-2.478230819	3.281555626	0.000523
ENSG00000254638.1	AP002884.2	-2.554147822	1.750923878	0.017745
ENSG00000131067.16	GGT7	-2.580345881	31.32726029	4.71E-32
ENSG00000237505.7	PKN2-AS1	-2.593661603	3.740691746	0.000182
ENSG00000162989.4	KCNJ3	-2.599989747	3.679561318	0.000209
ENSG00000250978.5	AC079467.1	-2.683899479	10.33965086	4.57E-11
ENSG00000214617.9	SLC6A10P	-2.771645999	2.362803916	0.004337
ENSG00000187889.12	FYB2	-2.809395322	2.721099863	0.001901
ENSG00000248587.7	GDNF-AS1	-2.842166915	19.63190997	2.33E-20
ENSG00000250056.5	LINC01018	-2.886299116	2.326308747	0.004717
ENSG00000261529.1	AC100774.1	-2.942941821	1.57863806	0.026385
ENSG00000169885.9	CALML6	-2.94510635	11.37735944	4.19E-12
ENSG00000256199.1	AC078962.2	-2.969594697	1.659800508	0.021888

ENSG00000158578.20	ALAS2	-3.032333813	2.792321181	0.001613
ENSG00000249203.1	LINC02224	-3.065792215	3.095700999	0.000802
ENSG00000168621.14	GDNF	-3.105603273	22.01165613	9.74E-23
ENSG00000004939.14	SLC4A1	-3.153268677	2.167386684	0.006802
ENSG00000164418.20	GRIK2	-3.230987256	6.247489991	5.66E-07
ENSG00000197085.11	NPSR1-AS1	-3.266398821	2.162900902	0.006872
ENSG00000171246.5	NPTX1	-3.294486887	2.938172205	0.001153
ENSG00000226476.3	LINC01748	-3.503455463	1.775047027	0.016786
ENSG00000231340.1	ACTG1P10	-3.545147938	1.494349608	0.032037
ENSG00000235285.1	SMIM2-IT1	-3.672275298	2.93622698	0.001158
ENSG00000248461.1	LINC02119	-3.78628294	2.678416367	0.002097
ENSG00000204460.3	LINC01854	-3.88067367	14.43861882	3.64E-15
ENSG00000232445.1	AC006329.1	-3.9247751	1.340390331	0.045668
ENSG00000198555.7	AC133561.1	-4.039760899	1.520709759	0.03015
ENSG00000285685.1	AC115284.3	-4.068195355	2.120143899	0.007583
ENSG00000223107.1	RNU2-72P	-4.183142725	1.54042403	0.028812
ENSG00000226519.1	LINC00390	-4.215836983	2.912697919	0.001223
ENSG00000256347.1	OR8R1P	-4.367215109	1.336139984	0.046117
ENSG00000250003.2	LINC02107	-4.742636934	4.067517344	8.56E-05
ENSG00000227176.1	AC092641.1	-4.811599266	1.451982489	0.03532
ENSG00000252461.1	RF00416	-4.993971017	1.893810419	0.01277
ENSG00000269964.3	MEI4	-5.413665862	2.189466049	0.006464
ENSG00000228956.8	SATB1-AS1	-5.414256076	3.214124155	0.000611
ENSG00000272485.1	AL392183.1	-5.765963807	5.069587173	8.52E-06

Supplemental Table 2: Differenctially expressed genes in males with inclusion body myositis

name	description	log2FoldCh	pvalue	padj
ENSG0000(	IGHV4-59	9.570427	17.57242	2.68E-18
ENSG0000(	KLHDC7B	9.215615	17.23445	5.83E-18
ENSG0000(	IGHV1-69D	8.953483	8.395591	4.02E-09
ENSG0000(	IGHJ2	8.706046	11.21634	6.08E-12
ENSG0000(	IGKV1D-39	8.578423	6.531611	2.94E-07
ENSG0000(	IGHV3-11	8.533909	15.64252	2.28E-16
ENSG0000(	SLC12A5-A	8.462279	16.13993	7.25E-17
ENSG0000(	IGHV5-10-:	8.447972	5.333466	4.64E-06
ENSG0000(	GZMK	8.426116	14.29163	5.11E-15
ENSG0000(	IGKV2-24	8.394443	16.50202	3.15E-17
ENSG0000(	PLA2G2D	8.316947	14.68023	2.09E-15
ENSG0000(	DHRS9	8.293338	21.57295	2.67E-22
ENSG0000(	DMBT1	8.213158	10.71396	1.93E-11
ENSG0000(	IGHV4-34	8.177058	9.556345	2.78E-10
ENSG0000(	IGHV2-5	8.166803	8.046428	8.99E-09
ENSG0000(	IGLV6-57	8.160935	7.974447	1.06E-08
ENSG0000(	RGS1	8.154314	19.14162	7.22E-20
ENSG0000(	IGHV3-48	8.142759	10.82301	1.50E-11
ENSG0000(	CXCR3	8.123391	16.07437	8.43E-17
ENSG0000(	IGKV1-13	8.12253	4.955621	1.11E-05
ENSG0000(	IGHV1-24	8.070201	4.900254	1.26E-05
ENSG0000(	CCL8	8.012941	18.56376	2.73E-19
ENSG0000(	AC099063.	7.92124	20.97631	1.06E-21
ENSG0000(	CCL4	7.779963	17.60121	2.50E-18
ENSG0000(	IGKV1-8	7.763837	10.30416	4.96E-11
ENSG0000(	EOMES	7.740723	13.19104	6.44E-14
ENSG0000(	CCL19	7.668108	7.291434	5.11E-08
ENSG0000(	KIAA1324	7.626777	18.67541	2.11E-19
ENSG0000(	AC243829.	7.609573	17.69398	2.02E-18
ENSG0000(	IGHJ1	7.606774	10.90723	1.24E-11
ENSG0000(	CD200R1	7.58627	20.49884	3.17E-21
ENSG0000(	IGHV3-49	7.579713	5.645367	2.26E-06
ENSG0000(	ZNF683	7.568935	6.454482	3.51E-07
ENSG0000(	CCL3	7.530416	17.04974	8.92E-18
ENSG0000(	SERPINB12	7.477842	7.550333	2.82E-08
ENSG0000(	AL671883.:	7.468501	12.71195	1.94E-13
ENSG0000(	TREM2	7.464717	11.13461	7.33E-12
ENSG0000(	TIFAB	7.44339	10.20537	6.23E-11
ENSG0000(	ABCB5	7.411111	4.510683	3.09E-05
ENSG0000(	CD70	7.400222	11.18818	6.48E-12
ENSG0000(	IGHV3-64D	7.308476	2.674411	0.002116
ENSG0000(	IGHV4-61	7.294545	7.762236	1.73E-08
ENSG0000(	YPEL4	7.285568	22.16511	6.84E-23
ENSG0000(	FAM30A	7.267511	10.57191	2.68E-11
ENSG0000(	THEMIS	7.263963	10.12574	7.49E-11

ENSG0000(	PDCD1	7.240559	17.61017	2.45E-18
ENSG0000(	IGLV7-43	7.22827	5.160597	6.91E-06
ENSG0000(	BHLHE22	7.219852	19.58256	2.61E-20
ENSG0000(	LY86	7.188815	15.24074	5.74E-16
ENSG0000(	SUCNR1	7.187338	12.40596	3.93E-13
ENSG0000(	TNFSF8	7.176195	14.58221	2.62E-15
ENSG0000(	CD69	7.173002	15.47423	3.36E-16
ENSG0000(	IGKV1-17	7.172887	8.573133	2.67E-09
ENSG0000(	CXCL13	7.170603	8.193093	6.41E-09
ENSG0000(	STAB2	7.165034	6.745857	1.80E-07
ENSG0000(	KLHL4	7.151695	14.68023	2.09E-15
ENSG0000(	LINC02257	7.14784	14.46442	3.43E-15
ENSG0000(	IGKV2-30	7.12477	8.349988	4.47E-09
ENSG0000(	ADGRG5	7.079406	11.57789	2.64E-12
ENSG0000(	IGLV8-61	7.069634	5.030967	9.31E-06
ENSG0000(	CHI3L2	7.056834	9.366704	4.30E-10
ENSG0000(	TNFRSF18	7.056153	13.87434	1.34E-14
ENSG0000(	IGHV4-4	7.053181	5.691853	2.03E-06
ENSG0000(	IGHV4-31	7.045429	4.93643	1.16E-05
ENSG0000(	IGLC7	7.037568	5.585405	2.60E-06
ENSG0000(	LINC01315	7.01274	14.24003	5.75E-15
ENSG0000(	IGLV7-46	7.009475	6.628151	2.35E-07
ENSG0000(	IGKV1-12	6.977023	11.17999	6.61E-12
ENSG0000(	GFI1	6.967805	13.14827	7.11E-14
ENSG0000(	CYP4F11	6.957235	9.489515	3.24E-10
ENSG0000(	IGLV4-69	6.925145	3.724651	0.000189
ENSG0000(	RNU2-2P	6.916503	9.524358	2.99E-10
ENSG0000(	IGHV3-43	6.906477	5.850654	1.41E-06
ENSG0000(	IGHV3-20	6.896786	6.626064	2.37E-07
ENSG0000(	MIR155HG	6.886795	11.00506	9.88E-12
ENSG0000(	SLFN12L	6.864198	11.25629	5.54E-12
ENSG0000(	MIR650	6.861647	8.635249	2.32E-09
ENSG0000(	AP000688.	6.859159	6.405134	3.93E-07
ENSG0000(	GNGT2	6.854022	17.62621	2.36E-18
ENSG0000(	CYP2S1	6.851278	14.1804	6.60E-15
ENSG0000(	AC008013.	6.816471	6.211961	6.14E-07
ENSG0000(	IGKV6-21	6.797557	4.09044	8.12E-05
ENSG0000(	ADORA3	6.7971	13.32179	4.77E-14
ENSG0000(	CYSLTR2	6.78788	11.63118	2.34E-12
ENSG0000(	C4orf50	6.78723	9.044627	9.02E-10
ENSG0000(	AC110995.	6.786108	10.48529	3.27E-11
ENSG0000(	IGKV1-16	6.783677	10.3553	4.41E-11
ENSG0000(	TRAJ39	6.774288	4.66029	2.19E-05
ENSG0000(	IGHV2-26	6.77245	2.504663	0.003129
ENSG0000(	ANXA8	6.758965	6.582903	2.61E-07
ENSG0000(	CLEC4G	6.749478	11.38043	4.16E-12
ENSG0000(	AC060234.	6.742248	11.19597	6.37E-12

ENSG0000(	ARL11	6.739367	10.90781	1.24E-11
ENSG0000(	DCANP1	6.701365	6.547114	2.84E-07
ENSG0000(	KRT222	6.679012	5.59412	2.55E-06
ENSG0000(	IGHV3-73	6.676262	4.037619	9.17E-05
ENSG0000(	CD1E	6.665795	4.645234	2.26E-05
ENSG0000(	AC068789.	6.659447	11.4781	3.33E-12
ENSG0000(	SLC8A2	6.642465	7.793106	1.61E-08
ENSG0000(	WNT2	6.615606	3.336968	0.00046
ENSG0000(	AL513303.:	6.605529	7.757038	1.75E-08
ENSG0000(	IGFL2-AS1	6.601227	6.306948	4.93E-07
ENSG0000(	LCN15	6.570892	8.833774	1.47E-09
ENSG0000(	PTPRH	6.569831	8.539656	2.89E-09
ENSG0000(	CHIT1	6.56861	6.656492	2.21E-07
ENSG0000(	JAKMIP1	6.56745	6.426005	3.75E-07
ENSG0000(	ASCL2	6.564785	7.821889	1.51E-08
ENSG0000(	EID3	6.540103	17.69398	2.02E-18
ENSG0000(	CRTAM	6.520235	7.622825	2.38E-08
ENSG0000(	SDS	6.51775	6.568282	2.70E-07
ENSG0000(	KCNK1	6.516845	7.164622	6.85E-08
ENSG0000(	AL357054.4	6.499136	11.34936	4.47E-12
ENSG0000(	FASLG	6.49886	6.422488	3.78E-07
ENSG0000(	C2-AS1	6.49468	9.418572	3.81E-10
ENSG0000(	RASL11B	6.494527	6.193197	6.41E-07
ENSG0000(	ENPP6	6.491879	6.129766	7.42E-07
ENSG0000(	HRASLS2	6.489955	7.557537	2.77E-08
ENSG0000(	LINC01915	6.476831	7.533517	2.93E-08
ENSG0000(	VNN1	6.473626	7.636018	2.31E-08
ENSG0000(	IGKJ3	6.472836	10.38383	4.13E-11
ENSG0000(	MIR214	6.471158	8.054495	8.82E-09
ENSG0000(	GPR171	6.470535	8.964747	1.08E-09
ENSG0000(	FGF16	6.454241	8.739668	1.82E-09
ENSG0000(	MFSD2A	6.444489	10.86254	1.37E-11
ENSG0000(	IGHV3-66	6.431781	2.774394	0.001681
ENSG0000(	FXYD3	6.431753	6.391507	4.06E-07
ENSG0000(	MIR3142H(	6.428339	7.624715	2.37E-08
ENSG0000(	AC092645.	6.428247	12.30867	4.91E-13
ENSG0000(	FCRL5	6.421795	5.31637	4.83E-06
ENSG0000(	SHISAL1	6.410928	9.174236	6.70E-10
ENSG0000(	C3orf80	6.395955	11.07665	8.38E-12
ENSG0000(	IGKV3-15	6.39221	10.34509	4.52E-11
ENSG0000(	LINC01550	6.383635	14.25608	5.55E-15
ENSG0000(	TNFRSF9	6.379023	8.789351	1.62E-09
ENSG0000(	MTUS2	6.353409	10.37745	4.19E-11
ENSG0000(	SLC9A9-AS:	6.346836	13.03628	9.20E-14
ENSG0000(	LAMP3	6.327623	7.538858	2.89E-08
ENSG0000(	RF02271	6.321867	18.25622	5.54E-19
ENSG0000(	LINC01504	6.314435	10.54254	2.87E-11

ENSG0000(	IGKV2-29	6.31028	1.893718	0.012773
ENSG0000(	ANKRD22	6.293525	7.135157	7.33E-08
ENSG0000(	MT1F	6.288745	8.587023	2.59E-09
ENSG0000(	TRBV20-1	6.286403	4.438162	3.65E-05
ENSG0000(	AC020907.	6.282562	6.194965	6.38E-07
ENSG0000(	IGLV5-45	6.279407	3.055903	0.000879
ENSG0000(	KLRC4	6.269454	7.395325	4.02E-08
ENSG0000(	IL10	6.269338	9.71649	1.92E-10
ENSG0000(	IGHV3-72	6.269274	2.626496	0.002363
ENSG0000(	AC005264.	6.255987	8.692677	2.03E-09
ENSG0000(	CTLA4	6.25596	8.2035	6.26E-09
ENSG0000(	MYH16	6.255001	4.972226	1.07E-05
ENSG0000(	ALOX15	6.253786	3.125633	0.000749
ENSG0000(	IGLV1-40	6.248733	9.239983	5.75E-10
ENSG0000(	Z84488.1	6.242866	7.452075	3.53E-08
ENSG0000(	RPTN	6.237765	3.607509	0.000247
ENSG0000(	IGHV4-55	6.235745	4.285321	5.18E-05
ENSG0000(	AL513122.:	6.228909	10.81856	1.52E-11
ENSG0000(	CD300LB	6.22886	7.974624	1.06E-08
ENSG0000(	IGKV1-6	6.225872	6.114457	7.68E-07
ENSG0000(	TRPV4	6.221861	10.11257	7.72E-11
ENSG0000(	IFNG	6.220946	5.951572	1.12E-06
ENSG0000(	LINC02384	6.213569	7.254081	5.57E-08
ENSG0000(	EPOP	6.210317	8.921815	1.20E-09
ENSG0000(	CEACAM21	6.208221	7.486032	3.27E-08
ENSG0000(	LINC00900	6.207321	9.252976	5.59E-10
ENSG0000(	CD101	6.206907	7.097049	8.00E-08
ENSG0000(	AP000662.	6.200008	11.12253	7.54E-12
ENSG0000(	AC010186.	6.194855	8.744616	1.80E-09
ENSG0000(	TMSB4Y	6.191773	13.74626	1.79E-14
ENSG0000(	OGFR-AS1	6.180933	13.21381	6.11E-14
ENSG0000(	ADAMDEC:	6.1693	4.165464	6.83E-05
ENSG0000(	FLG-AS1	6.167087	9.058916	8.73E-10
ENSG0000(	AC022034.	6.162512	8.76112	1.73E-09
ENSG0000(	AC093484.	6.158199	9.252976	5.59E-10
ENSG0000(	KCNK3	6.156533	7.145056	7.16E-08
ENSG0000(	IGHV1-46	6.146694	7.863559	1.37E-08
ENSG0000(	IGHV2-70	6.13461	3.003808	0.000991
ENSG0000(	CALHM6	6.130424	11.63118	2.34E-12
ENSG0000(	MIR3120	6.124144	5.579899	2.63E-06
ENSG0000(	AL450992.:	6.118642	12.35336	4.43E-13
ENSG0000(	SOWAHD	6.114164	6.050032	8.91E-07
ENSG0000(	IGHV3-64	6.111583	3.621128	0.000239
ENSG0000(	AC034238.	6.110593	7.697563	2.01E-08
ENSG0000(	HCG4P8	6.108635	10.10068	7.93E-11
ENSG0000(	FGF21	6.107851	2.951855	0.001117
ENSG0000(	ATP8B3	6.086735	7.260782	5.49E-08

ENSG0000(	IGKV1D-13	6.080757	1.772791	0.016874
ENSG0000(	AC245100.	6.062439	12.82179	1.51E-13
ENSG0000(	GCSAM	6.057406	10.36268	4.34E-11
ENSG0000(	NUGGC	6.057025	4.961042	1.09E-05
ENSG0000(	TRIM17	6.055696	7.192641	6.42E-08
ENSG0000(	ARC	6.046756	7.020409	9.54E-08
ENSG0000(	FER1L6	6.044536	6.911148	1.23E-07
ENSG0000(	LINC00892	6.040961	5.841667	1.44E-06
ENSG0000(	HTR2B	6.040615	5.762177	1.73E-06
ENSG0000(	GDF7	6.031118	8.046269	8.99E-09
ENSG0000(	LINC01503	6.026252	10.27613	5.30E-11
ENSG0000(	CRYBB1	6.022979	6.033361	9.26E-07
ENSG0000(	IGHV3-7	6.017017	9.664113	2.17E-10
ENSG0000(	IGLV1-47	6.01362	5.487909	3.25E-06
ENSG0000(	KCNA3	6.009728	4.129703	7.42E-05
ENSG0000(	AC023794.	6.000623	5.612384	2.44E-06
ENSG0000(	IGKV3D-15	5.999949	3.517912	0.000303
ENSG0000(	ADH1A	5.995912	2.890122	0.001288
ENSG0000(	RAB36	5.986208	5.700342	1.99E-06
ENSG0000(	CHKB-DT	5.982225	8.911918	1.22E-09
ENSG0000(	AK5	5.981126	10.29267	5.10E-11
ENSG0000(	AL359752.:	5.980224	9.729204	1.87E-10
ENSG0000(	AC133065.	5.974933	6.699036	2.00E-07
ENSG0000(	NECAB2	5.974417	4.878214	1.32E-05
ENSG0000(	AL391832.:	5.972127	10.81083	1.55E-11
ENSG0000(	IGHV3-21	5.956786	7.490517	3.23E-08
ENSG0000(	AL033527.:	5.952842	6.697704	2.01E-07
ENSG0000(	LINC00944	5.950284	5.565819	2.72E-06
ENSG0000(	AC116407.	5.947991	8.832505	1.47E-09
ENSG0000(	TEX41	5.947889	3.450879	0.000354
ENSG0000(	AC060766.	5.947288	12.3332	4.64E-13
ENSG0000(	RPS2P32	5.946788	10.38383	4.13E-11
ENSG0000(	AC025188.	5.944463	8.555337	2.78E-09
ENSG0000(	SIGLEC7	5.942933	5.439556	3.63E-06
ENSG0000(	P2RY10	5.938703	4.129814	7.42E-05
ENSG0000(	RNU6-146F	5.938173	12.72211	1.90E-13
ENSG0000(	AL157935.:	5.938164	7.134338	7.34E-08
ENSG0000(	IGKV2-28	5.93373	9.507325	3.11E-10
ENSG0000(	GALNT14	5.930209	13.78717	1.63E-14
ENSG0000(	PTMAP2	5.928627	10.07998	8.32E-11
ENSG0000(	TRBJ1-5	5.927492	3.436689	0.000366
ENSG0000(	TMIGD3	5.918885	5.670886	2.13E-06
ENSG0000(	C4BPB	5.898633	6.882993	1.31E-07
ENSG0000(	CCL4L2	5.890939	3.425624	0.000375
ENSG0000(	AC130371.	5.890392	5.620345	2.40E-06
ENSG0000(	XCL2	5.890258	2.870096	0.001349
ENSG0000(	ZNF365	5.883938	2.321951	0.004765

ENSG0000(	IGHJ5	5.876363	13.28636	5.17E-14
ENSG0000(	CEACAM16	5.874453	5.610585	2.45E-06
ENSG0000(	FCGR1B	5.865454	4.033225	9.26E-05
ENSG0000(	AP000919.	5.850664	8.173582	6.71E-09
ENSG0000(	FOLR1	5.849095	4.689259	2.05E-05
ENSG0000(	VEPH1	5.846774	4.587319	2.59E-05
ENSG0000(	AL158071.:	5.846767	6.646949	2.25E-07
ENSG0000(	GYG2P1	5.845833	9.857053	1.39E-10
ENSG0000(	C15orf48	5.841216	2.897505	0.001266
ENSG0000(	RNA5SP28:	5.838334	5.672485	2.13E-06
ENSG0000(	STYK1	5.834523	8.230913	5.88E-09
ENSG0000(	TRGC2	5.834147	4.021771	9.51E-05
ENSG0000(	AC009093.	5.831447	6.984189	1.04E-07
ENSG0000(	GSEC	5.83136	8.087633	8.17E-09
ENSG0000(	KCNJ5	5.825277	19.39131	4.06E-20
ENSG0000(	C8orf88	5.817305	6.619108	2.40E-07
ENSG0000(	WNT10A	5.812533	3.974585	0.000106
ENSG0000(	IGKV1-39	5.810893	13.13146	7.39E-14
ENSG0000(	LIPC	5.807213	4.703046	1.98E-05
ENSG0000(	DNAH14	5.79851	6.819426	1.52E-07
ENSG0000(	LY6D	5.792848	2.813455	0.001537
ENSG0000(	AC091576.	5.789419	5.56589	2.72E-06
ENSG0000(	WDR86-AS	5.776882	4.398179	4.00E-05
ENSG0000(	FCRL3	5.774975	5.309851	4.90E-06
ENSG0000(	CLNK	5.773602	3.865146	0.000136
ENSG0000(	CCL3L1	5.770662	3.350827	0.000446
ENSG0000(	CRYBG2	5.766574	5.686897	2.06E-06
ENSG0000(	C2CD6	5.764751	8.447901	3.57E-09
ENSG0000(	RGS13	5.760124	8.241604	5.73E-09
ENSG0000(	C9orf84	5.758733	5.500659	3.16E-06
ENSG0000(	IGKV3-7	5.756076	2.762662	0.001727
ENSG0000(	IGHV3-13	5.752035	2.297384	0.005042
ENSG0000(	GSDMA	5.750818	3.846912	0.000142
ENSG0000(	C1orf147	5.744515	4.745106	1.80E-05
ENSG0000(	SIGLEC14	5.734273	3.829254	0.000148
ENSG0000(	HIGD1AP1:	5.731765	3.337509	0.00046
ENSG0000(	AL139405.:	5.716923	3.263317	0.000545
ENSG0000(	C1orf220	5.707323	5.550965	2.81E-06
ENSG0000(	IGLV3-1	5.707216	10.82275	1.50E-11
ENSG0000(	GASAL1	5.705025	5.73267	1.85E-06
ENSG0000(	IGKV2D-28	5.700302	2.301593	0.004994
ENSG0000(	CNTNAP4	5.692124	8.259854	5.50E-09
ENSG0000(	LRG1	5.691706	4.331601	4.66E-05
ENSG0000(	F2RL2	5.688641	2.67278	0.002124
ENSG0000(	LINC00163	5.687615	3.912501	0.000122
ENSG0000(	LINC01094	5.687225	6.455918	3.50E-07
ENSG0000(	LAG3	5.683446	14.83455	1.46E-15

ENSG0000(	AP000779.	5.680124	6.885954	1.30E-07
ENSG0000(	EBI3	5.677062	5.570654	2.69E-06
ENSG0000(	SLC44A3-A	5.671231	4.39717	4.01E-05
ENSG0000(	AC022415.	5.671088	8.215552	6.09E-09
ENSG0000(	AC010761.	5.670148	5.517889	3.03E-06
ENSG0000(	UNC5B-AS:	5.667784	2.695183	0.002018
ENSG0000(	TIGIT	5.667682	8.359258	4.37E-09
ENSG0000(	SLC12A8	5.665415	5.497829	3.18E-06
ENSG0000(	IGHV4-28	5.664196	2.686955	0.002056
ENSG0000(	APOBEC3H	5.663846	3.316537	0.000482
ENSG0000(	XCL1	5.659856	3.217466	0.000606
ENSG0000(	ZNF311	5.658209	5.500147	3.16E-06
ENSG0000(	FAM72B	5.647271	4.774364	1.68E-05
ENSG0000(	HLA-DPB2	5.646563	2.290753	0.00512
ENSG0000(	AL592146.:	5.642047	3.186617	0.000651
ENSG0000(	IGKV3-11	5.632786	12.84154	1.44E-13
ENSG0000(	AC023510.	5.630453	5.578688	2.64E-06
ENSG0000(	AC211486.	5.621793	5.494484	3.20E-06
ENSG0000(	AC016597.	5.616488	8.2613	5.48E-09
ENSG0000(	RYR2	5.614521	5.513326	3.07E-06
ENSG0000(	INSYN2	5.614039	6.382676	4.14E-07
ENSG0000(	AC005736.	5.613265	6.81472	1.53E-07
ENSG0000(	AC004865.	5.611184	6.57862	2.64E-07
ENSG0000(	LHFPL1	5.611105	6.252773	5.59E-07
ENSG0000(	IGHV3-33	5.610265	7.196207	6.36E-08
ENSG0000(	AC096921.	5.608958	4.511454	3.08E-05
ENSG0000(	AC015911.	5.608348	3.23072	0.000588
ENSG0000(	FOLH1	5.608302	2.702526	0.001984
ENSG0000(	IGLV2-18	5.602333	1.878326	0.013233
ENSG0000(	FAM19A2	5.596387	6.461726	3.45E-07
ENSG0000(	IGLV9-49	5.591191	1.863449	0.013695
ENSG0000(	RNASE6	5.590543	12.45107	3.54E-13
ENSG0000(	ADM2	5.588235	5.37221	4.24E-06
ENSG0000(	TNFRSF13E	5.585888	3.609441	0.000246
ENSG0000(	BTLA	5.583444	3.215378	0.000609
ENSG0000(	IGKV1D-12	5.574791	1.534285	0.029222
ENSG0000(	AP001043.	5.57375	5.405141	3.93E-06
ENSG0000(	OR2A20P	5.56905	3.824102	0.00015
ENSG0000(	AL136419.:	5.568977	8.13709	7.29E-09
ENSG0000(	AK8	5.567541	5.502239	3.15E-06
ENSG0000(	AC233309.	5.567537	3.764052	0.000172
ENSG0000(	AC116407.	5.566776	6.67731	2.10E-07
ENSG0000(	AC092111.	5.561146	5.50014	3.16E-06
ENSG0000(	ICOS	5.559945	2.647469	0.002252
ENSG0000(	HLA-DRB9	5.559711	3.14963	0.000709
ENSG0000(	ADCYAP1	5.556143	2.589044	0.002576
ENSG0000(	TTC24	5.554818	8.903486	1.25E-09

ENSG0000(	ITGB8	5.554553	12.61244	2.44E-13
ENSG0000(	BCL11A	5.552026	5.476681	3.34E-06
ENSG0000(	AC245100.	5.551448	6.551044	2.81E-07
ENSG0000(	EDARADD	5.548636	8.114837	7.68E-09
ENSG0000(	MIR2277	5.541747	5.444538	3.59E-06
ENSG0000(	TLR8	5.534333	12.61244	2.44E-13
ENSG0000(	EML2-AS1	5.530869	5.539491	2.89E-06
ENSG0000(	GDF6	5.5212	2.567831	0.002705
ENSG0000(	MS4A1	5.519902	2.185534	0.006523
ENSG0000(	CYSRT1	5.518808	8.05245	8.86E-09
ENSG0000(	NMRAL2P	5.516585	2.595965	0.002535
ENSG0000(	CALML3	5.515628	2.180322	0.006602
ENSG0000(	LINC01934	5.511509	3.685694	0.000206
ENSG0000(	AC083841.	5.511124	2.203676	0.006256
ENSG0000(	AC090825.	5.509444	4.406333	3.92E-05
ENSG0000(	ASGR1	5.508743	4.421152	3.79E-05
ENSG0000(	GAPT	5.496432	2.547646	0.002834
ENSG0000(	DTHD1	5.495701	2.625113	0.002371
ENSG0000(	LPAR4	5.49547	3.191507	0.000643
ENSG0000(	STARD13-A	5.49424	5.229357	5.90E-06
ENSG0000(	FRMPD3	5.493375	4.333728	4.64E-05
ENSG0000(	MUC12	5.493189	3.775827	0.000168
ENSG0000(	GCNT3	5.492871	2.174237	0.006695
ENSG0000(	AC025271.	5.491745	2.547941	0.002832
ENSG0000(	IGKV1-9	5.489338	4.512802	3.07E-05
ENSG0000(	TSPAN10	5.488076	5.421771	3.79E-06
ENSG0000(	HCN2	5.48168	5.079974	8.32E-06
ENSG0000(	AP000757.	5.480231	5.332331	4.65E-06
ENSG0000(	RUFY4	5.479712	2.626054	0.002366
ENSG0000(	IGLV2-8	5.477281	6.542403	2.87E-07
ENSG0000(	NBAT1	5.472006	4.267728	5.40E-05
ENSG0000(	CRYBB3	5.470461	4.459742	3.47E-05
ENSG0000(	IFNG-AS1	5.467595	3.620113	0.00024
ENSG0000(	TRBV7-2	5.466007	2.177211	0.00665
ENSG0000(	AC124301.	5.46124	2.178884	0.006624
ENSG0000(	CLEC9A	5.461176	2.618505	0.002407
ENSG0000(	ADRA1A	5.456321	4.45891	3.48E-05
ENSG0000(	C2CD4D	5.454769	4.361555	4.35E-05
ENSG0000(	BTNL8	5.454455	2.588172	0.002581
ENSG0000(	LINC00891	5.448977	3.763485	0.000172
ENSG0000(	MZB1	5.448665	8.768463	1.70E-09
ENSG0000(	KCNB2	5.447182	2.592799	0.002554
ENSG0000(	IGKV1D-33	5.44638	1.787135	0.016325
ENSG0000(	ZNF578	5.443015	8.082804	8.26E-09
ENSG0000(	GABRA2	5.4404	2.536555	0.002907
ENSG0000(	LINC01426	5.438867	2.61384	0.002433
ENSG0000(	IMPG2	5.4386	5.47081	3.38E-06

ENSG0000(	TNFRSF17	5.437584	2.194044	0.006397
ENSG0000(	MIR6748	5.431845	6.59078	2.57E-07
ENSG0000(	C8orf48	5.427341	4.393037	4.05E-05
ENSG0000(	AL035530.:	5.425625	4.400371	3.98E-05
ENSG0000(	AL390879.:	5.421472	3.610262	0.000245
ENSG0000(	IGHV3-23	5.417273	10.60654	2.47E-11
ENSG0000(	AL157394.:	5.405215	6.37275	4.24E-07
ENSG0000(	AC245100.	5.403715	5.310535	4.89E-06
ENSG0000(	DCAF12L2	5.402961	4.367324	4.29E-05
ENSG0000(	AC131025.	5.402316	3.062601	0.000866
ENSG0000(	NBEAP1	5.400254	2.974731	0.00106
ENSG0000(	TCTEX1D4	5.400173	3.676193	0.000211
ENSG0000(	AL160270.:	5.396552	2.960734	0.001095
ENSG0000(	AC015911.	5.39518	2.562326	0.00274
ENSG0000(	AC116036.	5.391189	6.136892	7.30E-07
ENSG0000(	MGC32805	5.391167	2.518627	0.00303
ENSG0000(	AC083837.	5.385149	3.555445	0.000278
ENSG0000(	AL391069.:	5.384713	4.179364	6.62E-05
ENSG0000(	CDHR1	5.380855	3.433253	0.000369
ENSG0000(	DRAIC	5.380667	5.112774	7.71E-06
ENSG0000(	AC009244.	5.379347	3.564659	0.000272
ENSG0000(	AL139352.:	5.370889	2.109685	0.007768
ENSG0000(	AL161669.:	5.366787	2.481582	0.003299
ENSG0000(	AC009955.	5.363503	6.315501	4.84E-07
ENSG0000(	PRR29-AS1	5.361833	5.087125	8.18E-06
ENSG0000(	AC009509.	5.360033	3.581066	0.000262
ENSG0000(	PABPC1P4	5.358304	2.480812	0.003305
ENSG0000(	IGLV2-14	5.357201	12.15289	7.03E-13
ENSG0000(	PPP1R26-A	5.355344	3.036872	0.000919
ENSG0000(	AL732437.:	5.353434	2.082098	0.008278
ENSG0000(	AC010503.	5.351612	3.019299	0.000957
ENSG0000(	AC092329.	5.349141	5.345958	4.51E-06
ENSG0000(	LYPD5	5.341153	4.396918	4.01E-05
ENSG0000(	KRT5	5.339715	2.963434	0.001088
ENSG0000(	SNORA25	5.338029	3.071056	0.000849
ENSG0000(	PHF24	5.337423	3.693862	0.000202
ENSG0000(	PAQR5	5.335563	3.007419	0.000983
ENSG0000(	IGLV2-28	5.331771	1.433056	0.036893
ENSG0000(	HIST2H2BF	5.329844	2.990033	0.001023
ENSG0000(	AC067945.	5.327896	3.60798	0.000247
ENSG0000(	IGLV1-51	5.320771	9.052572	8.86E-10
ENSG0000(	LINC01852	5.319751	3.631981	0.000233
ENSG0000(	PTPRD-AS1	5.319501	2.885767	0.001301
ENSG0000(	AC093627.	5.310867	2.996249	0.001009
ENSG0000(	AC005077.	5.307719	3.537164	0.00029
ENSG0000(	NPFFR2	5.307499	3.581119	0.000262
ENSG0000(	FKBP9P1	5.30393	4.151002	7.06E-05

ENSG0000(	SLC6A4	5.301314	4.19323	6.41E-05
ENSG0000(	AC135721.	5.300082	5.067086	8.57E-06
ENSG0000(	CD40LG	5.299604	2.504395	0.00313
ENSG0000(	AL161935.:	5.299045	3.560133	0.000275
ENSG0000(	TRBV5-1	5.297995	2.054631	0.008818
ENSG0000(	GPR88	5.282278	5.091728	8.10E-06
ENSG0000(	NIPAL4	5.281386	3.536582	0.000291
ENSG0000(	IGKV3D-11	5.277729	2.035168	0.009222
ENSG0000(	SEC22B4P	5.273066	3.039582	0.000913
ENSG0000(	AC104447.	5.268145	7.604226	2.49E-08
ENSG0000(	CCDC170	5.267242	8.595515	2.54E-09
ENSG0000(	LINC01480	5.266323	3.576222	0.000265
ENSG0000(	AC040970.	5.263676	2.955105	0.001109
ENSG0000(	NAGS	5.243242	4.257875	5.52E-05
ENSG0000(	AC106886.	5.242093	4.211147	6.15E-05
ENSG0000(	LINC00865	5.235852	2.931637	0.00117
ENSG0000(	TRAV19	5.231475	2.872346	0.001342
ENSG0000(	FCRLA	5.231038	2.923383	0.001193
ENSG0000(	DUOX2	5.229611	1.689995	0.020418
ENSG0000(	VWA5B1	5.228154	4.22863	5.91E-05
ENSG0000(	AL645939.!	5.217494	2.929157	0.001177
ENSG0000(	TRBJ2-2	5.215631	2.42172	0.003787
ENSG0000(	AC136424.	5.214571	1.68287	0.020755
ENSG0000(	AC023908.	5.214184	2.013537	0.009693
ENSG0000(	AC087878.	5.212864	2.433699	0.003684
ENSG0000(	IGHGP	5.206065	19.37342	4.23E-20
ENSG0000(	AIRE	5.201855	2.377256	0.004195
ENSG0000(	TRIM67	5.201738	2.386377	0.004108
ENSG0000(	LNCTAM34	5.200321	3.515501	0.000305
ENSG0000(	MMP23B	5.198262	2.923495	0.001193
ENSG0000(	IGHV3-69-:	5.1964	1.653719	0.022196
ENSG0000(	LRRTM2	5.192845	4.258947	5.51E-05
ENSG0000(	RAB33A	5.192636	2.881409	0.001314
ENSG0000(	DLGAP2	5.188694	2.915793	0.001214
ENSG0000(	C1QL4	5.188195	1.654033	0.02218
ENSG0000(	TTC34	5.183587	4.245028	5.69E-05
ENSG0000(	LINC00570	5.181714	2.422627	0.003779
ENSG0000(	AC131097.	5.177874	2.95561	0.001108
ENSG0000(	RFPL1S	5.175595	4.202775	6.27E-05
ENSG0000(	PEAK3	5.165418	4.193274	6.41E-05
ENSG0000(	ADGRE4P	5.161315	2.403461	0.003949
ENSG0000(	IYD	5.159146	1.677239	0.021026
ENSG0000(	AC004706.	5.159073	3.460959	0.000346
ENSG0000(	ASPDH	5.151089	3.379322	0.000418
ENSG0000(	WNT3	5.150822	2.90267	0.001251
ENSG0000(	FLJ22447	5.133948	1.979729	0.010478
ENSG0000(	IGHV3-53	5.133271	3.086048	0.00082

ENSG0000(	AL356056.:	5.124092	3.347718	0.000449
ENSG0000(	IGHV5-51	5.123334	7.485382	3.27E-08
ENSG0000(	AL158163.:	5.122756	4.96429	1.09E-05
ENSG0000(	APOBEC3B	5.122486	2.379139	0.004177
ENSG0000(	CFAP300	5.121058	1.974826	0.010597
ENSG0000(	IGLV3-19	5.118458	6.446705	3.58E-07
ENSG0000(	AC002472.	5.115522	2.37455	0.004221
ENSG0000(	CD180	5.111018	6.855372	1.40E-07
ENSG0000(	AP000944.	5.108054	3.336069	0.000461
ENSG0000(	RN7SKP17!	5.106665	2.818414	0.001519
ENSG0000(	AC067930.	5.106665	2.818414	0.001519
ENSG0000(	AP000759.	5.104996	4.112344	7.72E-05
ENSG0000(	AC023794.	5.102061	1.351648	0.044499
ENSG0000(	DHRS4L1	5.101514	4.892715	1.28E-05
ENSG0000(	GPR85	5.100669	2.826228	0.001492
ENSG0000(	RF01875	5.097492	2.352296	0.004443
ENSG0000(	SNX29P2	5.088461	1.947299	0.01129
ENSG0000(	IGKV2D-29	5.086467	1.346606	0.045019
ENSG0000(	ENO4	5.084678	2.825551	0.001494
ENSG0000(	TRBJ1-1	5.081628	2.323175	0.004751
ENSG0000(	AC145676.	5.081051	2.301075	0.004999
ENSG0000(	AC018529.	5.079474	2.84433	0.001431
ENSG0000(	SLITRK2	5.072315	1.343384	0.045354
ENSG0000(	C9orf106	5.069275	3.394819	0.000403
ENSG0000(	IGHJ4	5.068577	15.23693	5.80E-16
ENSG0000(	SYNGR3	5.06813	2.77219	0.00169
ENSG0000(	LRRC6	5.06619	2.833456	0.001467
ENSG0000(	AC010201.	5.064753	1.947659	0.011281
ENSG0000(	AC009093.	5.057491	1.613999	0.024322
ENSG0000(	AC012313.	5.057414	4.136645	7.30E-05
ENSG0000(	CYP7B1	5.056781	3.387898	0.000409
ENSG0000(	SIGLEC17P	5.052912	2.857861	0.001387
ENSG0000(	AP001363.	5.052188	2.343498	0.004534
ENSG0000(	AC000403.	5.0519	2.85721	0.001389
ENSG0000(	IGHG4	5.05044	8.61655	2.42E-09
ENSG0000(	IGKV1-5	5.048988	9.086181	8.20E-10
ENSG0000(	AL451074.:	5.048019	3.346744	0.00045
ENSG0000(	GYPE	5.043194	2.339213	0.004579
ENSG0000(	HLA-W	5.042676	4.847334	1.42E-05
ENSG0000(	SNORA73B	5.041954	1.583657	0.026082
ENSG0000(	TFEC	5.040803	11.03335	9.26E-12
ENSG0000(	MIR6845	5.035606	2.758149	0.001745
ENSG0000(	CCL18	5.033326	11.99461	1.01E-12
ENSG0000(	PTP4A2P2	5.029695	3.289858	0.000513
ENSG0000(	AL139246.!	5.029192	2.763901	0.001722
ENSG0000(	TTC22	5.028084	2.322155	0.004763
ENSG0000(	IGHV3-30	5.023362	9.324691	4.73E-10

ENSG0000(	LINC00648	5.023196	1.605748	0.024789
ENSG0000(	AC002059.	5.021784	3.23087	0.000588
ENSG0000(	CCL26	5.01983	2.346336	0.004505
ENSG0000(	IGKV1-33	5.019639	8.7643	1.72E-09
ENSG0000(	LINC00484	5.016832	2.813922	0.001535
ENSG0000(	AL049775.:	5.015227	3.283068	0.000521
ENSG0000(	IGHV1-18	5.014775	6.976656	1.06E-07
ENSG0000(	REP15	5.012302	3.313524	0.000486
ENSG0000(	MIR147B	5.002438	1.929806	0.011754
ENSG0000(	ETV7	5.001751	10.52325	3.00E-11
ENSG0000(	FAM178B	5.001329	2.347624	0.004491
ENSG0000(	NKX3-2	5.001192	1.309782	0.049003
ENSG0000(	AP000282.	5.000729	1.568541	0.027006
ENSG0000(	AC008074.	4.999922	4.888591	1.29E-05
ENSG0000(	CD1B	4.998825	1.895202	0.012729
ENSG0000(	ZBED2	4.992616	2.680049	0.002089
ENSG0000(	WARS2-IT1	4.991308	2.28999	0.005129
ENSG0000(	LINC01579	4.990779	2.738987	0.001824
ENSG0000(	AC244517.	4.989905	3.312162	0.000487
ENSG0000(	OMG	4.988735	2.813323	0.001537
ENSG0000(	AL137856.:	4.987867	2.76728	0.001709
ENSG0000(	LINC01260	4.985441	1.581803	0.026194
ENSG0000(	TONSL-AS1	4.981918	2.78772	0.00163
ENSG0000(	LINC01871	4.978126	1.891186	0.012847
ENSG0000(	SFTA1P	4.977689	1.913531	0.012203
ENSG0000(	SIGLEC5	4.971979	2.689538	0.002044
ENSG0000(	INSM1	4.968847	1.888014	0.012942
ENSG0000(	DUOXA1	4.966789	1.901957	0.012533
ENSG0000(	IDH1-AS1	4.96663	3.186743	0.000651
ENSG0000(	SIGLEC6	4.962021	2.336463	0.004608
ENSG0000(	SIT1	4.961012	8.960103	1.10E-09
ENSG0000(	AC107398.	4.957578	2.302056	0.004988
ENSG0000(	RF00285	4.956421	2.719671	0.001907
ENSG0000(	AL096816.:	4.956218	1.873182	0.013391
ENSG0000(	RN7SL138F	4.955125	2.766232	0.001713
ENSG0000(	MYMK	4.952789	7.006442	9.85E-08
ENSG0000(	SLC7A5P1	4.951356	3.266305	0.000542
ENSG0000(	AL031985.:	4.95103	3.257642	0.000553
ENSG0000(	FHAD1	4.949506	2.70092	0.001991
ENSG0000(	AC004834.	4.948248	2.225816	0.005945
ENSG0000(	AC005865.	4.945725	2.67376	0.00212
ENSG0000(	HIST1H2AK	4.944324	2.700671	0.001992
ENSG0000(	CCL13	4.941272	16.25199	5.60E-17
ENSG0000(	CRYBB2	4.940492	2.705088	0.001972
ENSG0000(	FANCD2OS	4.940377	3.28509	0.000519
ENSG0000(	DNAJB13	4.938177	2.708209	0.001958
ENSG0000(	HS3ST3A1	4.937288	2.721671	0.001898

ENSG0000(	LILRB4	4.933863	9.666718	2.15E-10
ENSG0000(	PHEX	4.929095	2.265158	0.005431
ENSG0000(	CYP19A1	4.926998	2.268149	0.005393
ENSG0000(	TRBV28	4.925846	1.542876	0.02865
ENSG0000(	AC008011.	4.925025	5.293249	5.09E-06
ENSG0000(	AC147651.	4.923234	2.665988	0.002158
ENSG0000(	TRBV7-9	4.916518	1.53006	0.029508
ENSG0000(	HPSE	4.909811	8.258925	5.51E-09
ENSG0000(	AL449106.:	4.905651	3.247349	0.000566
ENSG0000(	IGKJ5	4.903637	8.495736	3.19E-09
ENSG0000(	AC017000.	4.9023	2.244115	0.0057
ENSG0000(	PMCH	4.899994	3.844071	0.000143
ENSG0000(	FAM187A	4.899725	1.887584	0.012954
ENSG0000(	AC243829.	4.897695	2.244072	0.005701
ENSG0000(	KLHL7-DT	4.892395	2.237269	0.005791
ENSG0000(	AC008467.	4.892314	2.26312	0.005456
ENSG0000(	ABCB11	4.891227	1.514587	0.030578
ENSG0000(	AC233702.	4.887244	3.205845	0.000623
ENSG0000(	IGHG1	4.878284	20.59958	2.51E-21
ENSG0000(	VN1R82P	4.872953	3.243239	0.000571
ENSG0000(	ACPP	4.872702	2.216362	0.006076
ENSG0000(	AC091132.	4.87264	3.23142	0.000587
ENSG0000(	LINC00856	4.872557	2.189296	0.006467
ENSG0000(	TRBJ1-2	4.869627	1.516649	0.030433
ENSG0000(	CDKN2B-AS	4.861485	2.688583	0.002048
ENSG0000(	AC005776.	4.86079	3.232318	0.000586
ENSG0000(	FIRRE	4.857378	2.696106	0.002013
ENSG0000(	ANKRD36B	4.856466	7.927202	1.18E-08
ENSG0000(	SEPT14P2	4.856329	4.724203	1.89E-05
ENSG0000(	RPL7P18	4.855128	1.521939	0.030065
ENSG0000(	GBP6	4.850279	8.531428	2.94E-09
ENSG0000(	AL133215.:	4.847635	2.622603	0.002384
ENSG0000(	WNK3	4.847053	2.223263	0.00598
ENSG0000(	AC116025.	4.844488	2.210565	0.006158
ENSG0000(	OTUB2	4.844363	3.926198	0.000119
ENSG0000(	ENPP3	4.843706	1.844022	0.014321
ENSG0000(	MMP9	4.843096	7.698371	2.00E-08
ENSG0000(	IGHJ6	4.842353	14.52122	3.01E-15
ENSG0000(	LINC02139	4.841659	3.178737	0.000663
ENSG0000(	AP003472.	4.839885	2.201646	0.006286
ENSG0000(	AC099850.	4.839228	2.641198	0.002285
ENSG0000(	STPG3	4.831918	1.815469	0.015294
ENSG0000(	IGHJ3P	4.831177	1.499137	0.031686
ENSG0000(	ADAMTS7F	4.830644	2.20315	0.006264
ENSG0000(	MIR6892	4.830134	2.651742	0.00223
ENSG0000(	AC124014.	4.828608	1.825104	0.014959
ENSG0000(	AKR7L	4.827475	2.608764	0.002462

ENSG0000(	AC026369.	4.826503	2.205963	0.006224
ENSG0000(	AC010210.	4.818254	2.634872	0.002318
ENSG0000(	VSTM5	4.816555	1.83369	0.014666
ENSG0000(	DDX25	4.813067	2.668929	0.002143
ENSG0000(	TRBV9	4.810445	1.464591	0.034309
ENSG0000(	DGCR5	4.809327	1.789766	0.016227
ENSG0000(	FCER2	4.809072	4.136645	7.30E-05
ENSG0000(	AC090136.	4.808741	2.642599	0.002277
ENSG0000(	NBEAP5	4.805062	1.503493	0.031369
ENSG0000(	IGHJ3	4.804756	8.989119	1.03E-09
ENSG0000(	AP000525.	4.804488	1.815028	0.01531
ENSG0000(	IGLV1-44	4.80359	5.908898	1.23E-06
ENSG0000(	AC010424.	4.802779	2.161834	0.006889
ENSG0000(	IGLV2-11	4.80255	6.720925	1.90E-07
ENSG0000(	IGHM	4.799703	21.24357	5.71E-22
ENSG0000(	TRBJ1-4	4.798638	2.130835	0.007399
ENSG0000(	LINC02446	4.798437	1.769741	0.016993
ENSG0000(	ENDOU	4.797923	1.803985	0.015704
ENSG0000(	MIR4785	4.797712	3.089163	0.000814
ENSG0000(	RORB	4.796379	1.820147	0.01513
ENSG0000(	CLDN10	4.796264	1.511942	0.030765
ENSG0000(	UBD	4.793758	9.603989	2.49E-10
ENSG0000(	AC018738.	4.792251	1.463948	0.03436
ENSG0000(	CXCL3	4.791196	1.447892	0.035654
ENSG0000(	OSMR-AS1	4.789683	2.182201	0.006574
ENSG0000(	RNU1-123F	4.788318	2.603791	0.00249
ENSG0000(	NCF4-AS1	4.787674	9.403817	3.95E-10
ENSG0000(	RF00285	4.787269	2.15265	0.007036
ENSG0000(	CLEC5A	4.786766	1.475005	0.033496
ENSG0000(	SH2D6	4.784848	1.787985	0.016294
ENSG0000(	IGKV4-1	4.784278	10.0901	8.13E-11
ENSG0000(	AL359710.:	4.778803	2.153888	0.007016
ENSG0000(	AP003774.	4.775387	2.601852	0.002501
ENSG0000(	AC098614.	4.773775	7.670343	2.14E-08
ENSG0000(	CCL22	4.773248	2.129502	0.007422
ENSG0000(	ADCY8	4.770813	2.570464	0.002689
ENSG0000(	SHLD2P1	4.769781	2.141167	0.007225
ENSG0000(	DBX2	4.768867	2.175997	0.006668
ENSG0000(	CYP4F22	4.765553	1.46669	0.034144
ENSG0000(	AL157871.:	4.76274	2.115607	0.007663
ENSG0000(	B3GNT7	4.761593	5.889715	1.29E-06
ENSG0000(	AL033527.!	4.76025	2.182201	0.006574
ENSG0000(	SLC30A3	4.757485	1.475072	0.033491
ENSG0000(	AC008737.	4.755538	1.78816	0.016287
ENSG0000(	AL355102.:	4.755225	5.83786	1.45E-06
ENSG0000(	EMBP1	4.7545	2.577057	0.002648
ENSG0000(	AC006064.	4.754213	2.624885	0.002372

ENSG0000(	LINC01993.	4.753134	1.783076	0.016479
ENSG0000(	SIGLEC8	4.750057	1.8085	0.015542
ENSG0000(	LGALS7B	4.746714	1.464088	0.034349
ENSG0000(	AC022509.	4.74131	2.156696	0.006971
ENSG0000(	LGALS2	4.739471	7.119968	7.59E-08
ENSG0000(	PADI3	4.737533	1.458817	0.034768
ENSG0000(	CPA1	4.73632	1.750334	0.017769
ENSG0000(	LINC01914	4.734749	1.765566	0.017157
ENSG0000(	IGLC2	4.73262	18.27198	5.35E-19
ENSG0000(	RTP5	4.732052	2.1137	0.007697
ENSG0000(	AC116347.	4.730169	1.74462	0.018004
ENSG0000(	RAB44	4.724782	2.597645	0.002526
ENSG0000(	AL691459.:	4.72244	1.745151	0.017982
ENSG0000(	BLK	4.722119	2.078386	0.008349
ENSG0000(	RTKN2	4.721299	2.137487	0.007286
ENSG0000(	PNOC	4.721065	1.765944	0.017142
ENSG0000(	AC245100.	4.719281	3.039136	0.000914
ENSG0000(	SCN11A	4.717312	1.468203	0.034025
ENSG0000(	GCSAML	4.715516	1.77275	0.016875
ENSG0000(	LINC01684	4.714437	1.770308	0.01697
ENSG0000(	TDO2	4.713663	6.372406	4.24E-07
ENSG0000(	FCGR1A	4.706193	4.908132	1.24E-05
ENSG0000(	GPR78	4.706116	5.647667	2.25E-06
ENSG0000(	PCDHB18P	4.705181	2.583552	0.002609
ENSG0000(	AL022323.4	4.702863	2.138048	0.007277
ENSG0000(	CHST13	4.702068	2.122497	0.007542
ENSG0000(	LRTM1	4.700915	3.133903	0.000735
ENSG0000(	AC138430.	4.699371	1.73924	0.018229
ENSG0000(	KRT81	4.699099	1.742259	0.018103
ENSG0000(	CATSPERZ	4.691824	2.11861	0.00761
ENSG0000(	IGLJ2	4.691808	15.13124	7.39E-16
ENSG0000(	CCR5	4.691407	11.09894	7.96E-12
ENSG0000(	TTLL9	4.689422	2.099319	0.007956
ENSG0000(	TEX14	4.688946	2.130759	0.0074
ENSG0000(	AC008937.	4.685606	1.75543	0.017562
ENSG0000(	AC105446.	4.68233	1.75556	0.017557
ENSG0000(	TNIP3	4.678802	1.742428	0.018096
ENSG0000(	AC008429.	4.677028	1.746364	0.017932
ENSG0000(	AC092903.	4.676601	1.447892	0.035654
ENSG0000(	AC106872.	4.674506	2.097745	0.007985
ENSG0000(	P2RY13	4.673562	8.334202	4.63E-09
ENSG0000(	AC103691.	4.67277	1.751042	0.01774
ENSG0000(	METTL27	4.671633	4.787901	1.63E-05
ENSG0000(	AC019197.	4.669008	2.114621	0.00768
ENSG0000(	IL12A	4.667821	1.741794	0.018122
ENSG0000(	AC079414.	4.667452	1.436851	0.036572
ENSG0000(	AL161669.:	4.66721	1.42404	0.037667

ENSG0000(	TFAP2C	4.666062	2.104752	0.007857
ENSG0000(	ELOVL3	4.665491	1.429911	0.037161
ENSG0000(	FAM225B	4.665184	2.119916	0.007587
ENSG0000(	AL031118.:	4.664487	1.753293	0.017648
ENSG0000(	ZNF831	4.664453	7.326399	4.72E-08
ENSG0000(	CXCL11	4.664205	9.639415	2.29E-10
ENSG0000(	SUMO2P17	4.660372	1.705486	0.019702
ENSG0000(	GPR174	4.658516	5.768945	1.70E-06
ENSG0000(	SHD	4.655413	6.390523	4.07E-07
ENSG0000(	AF127936.	4.655102	2.076577	0.008383
ENSG0000(	PRR7-AS1	4.654434	2.54521	0.00285
ENSG0000(	AL359183.:	4.65375	1.733825	0.018458
ENSG0000(	AC104564.	4.653602	1.726635	0.018766
ENSG0000(	CPZ	4.653485	5.465464	3.42E-06
ENSG0000(	AL008729.:	4.650928	2.116196	0.007653
ENSG0000(	RHOH	4.645036	5.977098	1.05E-06
ENSG0000(	SLAMF1	4.644702	4.960209	1.10E-05
ENSG0000(	TYMSOS	4.642199	2.081222	0.008294
ENSG0000(	BNC1	4.641376	2.100103	0.007941
ENSG0000(	RHEBP1	4.637667	1.417572	0.038232
ENSG0000(	CXCL9	4.633887	16.75466	1.76E-17
ENSG0000(	AC018755.	4.633835	1.402132	0.039616
ENSG0000(	IGHV1-2	4.630153	5.801169	1.58E-06
ENSG0000(	KLRC1	4.629247	1.710954	0.019456
ENSG0000(	FTLP3	4.629238	2.111505	0.007736
ENSG0000(	ZMYND15	4.627082	5.333875	4.64E-06
ENSG0000(	AC009133.	4.62498	1.711084	0.01945
ENSG0000(	AC100793.	4.62401	1.415753	0.038393
ENSG0000(	TMEM150I	4.623222	7.240302	5.75E-08
ENSG0000(	AL137779.:	4.622257	2.513113	0.003068
ENSG0000(	PATL2	4.622159	8.098121	7.98E-09
ENSG0000(	AL390719.:	4.617774	1.415651	0.038402
ENSG0000(	SEZ6	4.614503	2.422185	0.003783
ENSG0000(	AC009093.	4.605888	1.403299	0.039509
ENSG0000(	AC064799.	4.605871	2.472933	0.003366
ENSG0000(	AL353719.:	4.599879	2.420407	0.003798
ENSG0000(	AC007731.	4.598331	2.074735	0.008419
ENSG0000(	P2RY12	4.598257	4.416774	3.83E-05
ENSG0000(	SNORA48	4.592759	2.046216	0.00899
ENSG0000(	CD79A	4.589912	6.189921	6.46E-07
ENSG0000(	SNORD95	4.587757	2.515026	0.003055
ENSG0000(	AC016629.	4.58536	2.033943	0.009248
ENSG0000(	AL161891.:	4.584224	1.725614	0.01881
ENSG0000(	AC134669.	4.578224	8.585446	2.60E-09
ENSG0000(	POU5F1P3	4.577762	2.040686	0.009106
ENSG0000(	IGKJ1	4.575212	14.85654	1.39E-15
ENSG0000(	AL158198.:	4.572306	1.389928	0.040745

ENSG0000(	AC024940.	4.572157	2.035168	0.009222
ENSG0000(	MMP2-AS1	4.570447	1.388458	0.040883
ENSG0000(	TNF	4.569727	5.787809	1.63E-06
ENSG0000(	LINC00539	4.567938	2.031259	0.009306
ENSG0000(	AC078880.	4.566243	1.652143	0.022277
ENSG0000(	FUT2	4.56333	1.695636	0.020154
ENSG0000(	AL139300.:	4.561116	1.396938	0.040092
ENSG0000(	SIGLEC9	4.552902	4.75405	1.76E-05
ENSG0000(	AC093890.	4.545721	1.687948	0.020514
ENSG0000(	AC147067.	4.545296	1.680926	0.020848
ENSG0000(	CYP2B7P	4.542228	2.009406	0.009786
ENSG0000(	C22orf31	4.542128	2.430233	0.003713
ENSG0000(	AC011939.	4.541687	1.67612	0.02108
ENSG0000(	AC127070.	4.541349	1.6593	0.021913
ENSG0000(	TMEM191/	4.541026	1.68458	0.020674
ENSG0000(	OASL	4.539769	8.167883	6.79E-09
ENSG0000(	ASB9	4.537732	6.219191	6.04E-07
ENSG0000(	AC008033.	4.535983	1.374798	0.042189
ENSG0000(	AC087284.	4.535724	1.372375	0.042425
ENSG0000(	AC007448.	4.535404	1.657375	0.02201
ENSG0000(	AL022323.:	4.532828	1.98502	0.010351
ENSG0000(	AC087623.	4.531504	1.379005	0.041783
ENSG0000(	KCNK4-TEX	4.531139	1.676347	0.021069
ENSG0000(	AC012313.	4.529518	2.440779	0.003624
ENSG0000(	IGHA1	4.524109	14.37478	4.22E-15
ENSG0000(	BEAN1-AS1	4.522405	1.365667	0.043086
ENSG0000(	RF02110	4.522282	1.994907	0.010118
ENSG0000(	AC010531.	4.518972	2.908773	0.001234
ENSG0000(	AP001021.	4.518455	1.99786	0.010049
ENSG0000(	AC018410.	4.516013	2.405444	0.003931
ENSG0000(	SULT1A3	4.514268	1.356539	0.044001
ENSG0000(	AL512631.:	4.513053	1.651335	0.022318
ENSG0000(	CD86	4.511994	8.853485	1.40E-09
ENSG0000(	IL5RA	4.509322	1.645891	0.0226
ENSG0000(	AC002128.	4.508019	2.027575	0.009385
ENSG0000(	AC009226.	4.505683	1.360652	0.043586
ENSG0000(	AL589743.!	4.504051	1.360443	0.043607
ENSG0000(	AL161457.:	4.503901	1.982796	0.010404
ENSG0000(	LINC00942	4.5025	5.400786	3.97E-06
ENSG0000(	AL034417.:	4.49741	1.630487	0.023416
ENSG0000(	C19orf81	4.49516	5.004653	9.89E-06
ENSG0000(	TMEM163	4.492008	1.627396	0.023583
ENSG0000(	C16orf54	4.490516	8.003222	9.93E-09
ENSG0000(	AC087501.	4.490148	1.67175	0.021294
ENSG0000(	AC055822.	4.48921	2.011144	0.009747
ENSG0000(	NGF	4.48754	1.651928	0.022288
ENSG0000(	AC002525.	4.487027	1.625217	0.023702

ENSG0000(	MST1L	4.486151	1.646175	0.022585
ENSG0000(	VN1R81P	4.485895	2.376797	0.0042
ENSG0000(	CORT	4.485852	1.982699	0.010406
ENSG0000(	AC090772.	4.484676	1.355978	0.044058
ENSG0000(	TMEM232	4.484443	1.997309	0.010062
ENSG0000(	CNTD2	4.484318	1.361681	0.043483
ENSG0000(	AL512422.:	4.479911	1.621452	0.023908
ENSG0000(	FCGR1CP	4.475561	1.350605	0.044606
ENSG0000(	CCDC181	4.474685	1.648293	0.022475
ENSG0000(	LINC02362	4.474285	1.353923	0.044267
ENSG0000(	STPG3-AS1	4.472626	1.349353	0.044735
ENSG0000(	LAX1	4.47137	5.60013	2.51E-06
ENSG0000(	TFR2	4.471297	1.362893	0.043362
ENSG0000(	CPNE7	4.470323	1.347884	0.044887
ENSG0000(	DBIL5P	4.469579	1.979717	0.010478
ENSG0000(	CNTN4-AS1	4.46883	1.355491	0.044107
ENSG0000(	ECT2L	4.468808	1.639579	0.022931
ENSG0000(	GPR84	4.466555	1.343587	0.045333
ENSG0000(	TOMM20P	4.466376	1.337579	0.045964
ENSG0000(	AC096733.	4.466223	1.64874	0.022452
ENSG0000(	HOXA1	4.465347	1.647489	0.022517
ENSG0000(	AC244153.	4.461248	1.637907	0.023019
ENSG0000(	EN2	4.45302	5.857231	1.39E-06
ENSG0000(	IGKC	4.45122	17.29214	5.10E-18
ENSG0000(	LINC00574	4.446779	1.973306	0.010634
ENSG0000(	LINC02511	4.445926	1.353807	0.044278
ENSG0000(	EVI2A	4.445424	8.05245	8.86E-09
ENSG0000(	AMER2	4.445404	1.327169	0.047079
ENSG0000(	AC103810.	4.443914	1.345653	0.045118
ENSG0000(	AL592429.:	4.439675	1.957285	0.011034
ENSG0000(	AC040934.	4.438928	1.94399	0.011377
ENSG0000(	IGHG2	4.433762	14.40411	3.94E-15
ENSG0000(	LINC01655	4.433232	1.599484	0.025149
ENSG0000(	IGLV3-21	4.431236	8.22986	5.89E-09
ENSG0000(	AL391069.:	4.427148	1.928768	0.011782
ENSG0000(	AC004585.	4.422527	1.333004	0.046451
ENSG0000(	E2F7	4.422509	1.606458	0.024748
ENSG0000(	TMPRSS3	4.420274	5.049838	8.92E-06
ENSG0000(	AC027237.	4.419131	1.626272	0.023644
ENSG0000(	AC244205.	4.41618	14.45332	3.52E-15
ENSG0000(	IGKJ2	4.415441	14.40562	3.93E-15
ENSG0000(	AL645929.:	4.414248	8.929728	1.18E-09
ENSG0000(	AC004923.	4.411811	1.606735	0.024732
ENSG0000(	CEND1	4.411163	1.591332	0.025625
ENSG0000(	AC114689.	4.410233	1.621044	0.023931
ENSG0000(	AC015712.	4.408956	1.618514	0.024071
ENSG0000(	C2CD4A	4.408487	1.310129	0.048963

ENSG0000(	AC106820.	4.407277	1.930201	0.011744
ENSG0000(	IGLJ1	4.406588	11.67994	2.09E-12
ENSG0000(	RDM1P3	4.406582	1.3307	0.046698
ENSG0000(	CXorf21	4.406285	8.056107	8.79E-09
ENSG0000(	AC106886.	4.403016	1.929624	0.011759
ENSG0000(	AC018761.	4.402416	1.611156	0.024482
ENSG0000(	LINC02044	4.402232	1.589362	0.025742
ENSG0000(	AC090152.	4.400722	1.941759	0.011435
ENSG0000(	AL031847.:	4.400486	1.949262	0.011239
ENSG0000(	DLGAP3	4.397803	1.609753	0.024561
ENSG0000(	RF01973	4.396021	1.310386	0.048934
ENSG0000(	SLC7A4	4.389288	3.917071	0.000121
ENSG0000(	SIGLEC11	4.38788	6.14807	7.11E-07
ENSG0000(	AC134312.	4.383484	1.301959	0.049893
ENSG0000(	CCDC116	4.382678	1.315726	0.048336
ENSG0000(	IGKJ4	4.381865	13.13484	7.33E-14
ENSG0000(	LMX1B	4.377394	3.629448	0.000235
ENSG0000(	AL359715.:	4.373108	1.59232	0.025567
ENSG0000(	SNORD36B	4.370177	1.304407	0.049613
ENSG0000(	LYPD3	4.369331	1.306626	0.04936
ENSG0000(	UTS2R	4.367188	5.995496	1.01E-06
ENSG0000(	GPR183	4.367059	7.350882	4.46E-08
ENSG0000(	AC092821.	4.363143	1.601849	0.025012
ENSG0000(	BREA2	4.363053	1.590858	0.025653
ENSG0000(	ZNF454	4.362265	1.596092	0.025346
ENSG0000(	GPR152	4.362075	1.911093	0.012272
ENSG0000(	AC106881.	4.360515	1.311139	0.04885
ENSG0000(	AC087286.	4.347094	1.582562	0.026148
ENSG0000(	IGLJ3	4.34422	10.36718	4.29E-11
ENSG0000(	RF02164	4.343213	1.570381	0.026892
ENSG0000(	BNC2-AS1	4.341694	1.592252	0.025571
ENSG0000(	AL353807.:	4.341606	1.301493	0.049947
ENSG0000(	AC027702.	4.337701	1.915698	0.012142
ENSG0000(	AC011455.	4.333729	1.571989	0.026792
ENSG0000(	TCF23	4.333167	2.842012	0.001439
ENSG0000(	LY96	4.330786	9.440951	3.62E-10
ENSG0000(	M1AP	4.328656	1.919718	0.01203
ENSG0000(	AC017083.	4.327982	1.585215	0.025989
ENSG0000(	LY9	4.324764	3.332221	0.000465
ENSG0000(	AC139491.	4.313609	1.557385	0.027709
ENSG0000(	RPL35P2	4.3104	1.562749	0.027368
ENSG0000(	LINC01686	4.305186	1.550675	0.02814
ENSG0000(	KLHL35	4.300039	6.171004	6.75E-07
ENSG0000(	CCR2	4.298964	8.15511	7.00E-09
ENSG0000(	AC005837.	4.297133	1.54967	0.028205
ENSG0000(	PYHIN1	4.291308	4.805845	1.56E-05
ENSG0000(	RHEBP2	4.289753	1.886266	0.012994

ENSG0000(	TRG-AS1	4.289644	4.881861	1.31E-05
ENSG0000(	SLC34A3	4.287701	1.534908	0.02918
ENSG0000(	IRF4	4.2834	8.588285	2.58E-09
ENSG0000(	MRPL37P1	4.282252	1.533681	0.029263
ENSG0000(	MIR6805	4.277642	1.542876	0.02865
ENSG0000(	AC004540.	4.273287	3.476457	0.000334
ENSG0000(	AC004771.	4.269465	1.551371	0.028095
ENSG0000(	LINC01239	4.268744	4.82734	1.49E-05
ENSG0000(	AC112236.	4.264337	4.608813	2.46E-05
ENSG0000(	CD2	4.261629	7.101632	7.91E-08
ENSG0000(	AP002360.	4.252676	1.53654	0.029071
ENSG0000(	AL365361.:	4.240762	3.543839	0.000286
ENSG0000(	TOX	4.231566	5.760785	1.73E-06
ENSG0000(	CXCL10	4.228394	13.83192	1.47E-14
ENSG0000(	AC026471.	4.223901	1.509094	0.030967
ENSG0000(	AC112484.	4.222329	1.509089	0.030968
ENSG0000(	OR2I1P	4.215451	13.12177	7.55E-14
ENSG0000(	RF00017	4.209174	1.805845	0.015637
ENSG0000(	SAMD3	4.206481	6.791972	1.61E-07
ENSG0000(	TLR7	4.205497	7.490255	3.23E-08
ENSG0000(	AL132800.:	4.202107	1.497645	0.031795
ENSG0000(	LINC01619	4.198691	1.492613	0.032165
ENSG0000(	AC008063.	4.186812	3.350305	0.000446
ENSG0000(	ZDHHC22	4.182629	3.757864	0.000175
ENSG0000(	AC006511.	4.180095	1.495062	0.031984
ENSG0000(	CHI3L1	4.177863	7.045706	9.00E-08
ENSG0000(	CD244	4.176071	3.474648	0.000335
ENSG0000(	AC046130.	4.170023	1.492359	0.032184
ENSG0000(	EGR2	4.165501	8.425985	3.75E-09
ENSG0000(	AC016683.	4.164493	1.475267	0.033476
ENSG0000(	TNNT2	4.15507	13.07479	8.42E-14
ENSG0000(	IL12RB1	4.15177	7.486032	3.27E-08
ENSG0000(	LINC00092	4.136065	1.793824	0.016076
ENSG0000(	ERICD	4.133729	1.47853	0.033225
ENSG0000(	C1QB	4.130684	20.66342	2.17E-21
ENSG0000(	AL451067.:	4.1251	4.899942	1.26E-05
ENSG0000(	FCRL6	4.123883	5.352189	4.44E-06
ENSG0000(	GZMA	4.122201	5.78892	1.63E-06
ENSG0000(	CDKN2A	4.113746	8.318773	4.80E-09
ENSG0000(	UBASH3A	4.1108	3.252764	0.000559
ENSG0000(	AC009802.	4.104973	4.634409	2.32E-05
ENSG0000(	LRRC70	4.102619	3.073618	0.000844
ENSG0000(	RHEX	4.101836	4.379165	4.18E-05
ENSG0000(	FOXI2	4.101149	3.338208	0.000459
ENSG0000(	ENO1P1	4.099326	1.468428	0.034007
ENSG0000(	AC091182.	4.099189	5.560275	2.75E-06
ENSG0000(	GBP5	4.096325	16.51925	3.03E-17

ENSG0000(	AC020612.	4.094211	1.454602	0.035107
ENSG0000(	AL645933.:	4.091412	10.0657	8.60E-11
ENSG0000(	KYNU	4.086286	5.555063	2.79E-06
ENSG0000(	BATF	4.080867	7.67095	2.13E-08
ENSG0000(	ARMH1	4.064776	5.767189	1.71E-06
ENSG0000(	IGLL5	4.063618	13.03628	9.20E-14
ENSG0000(	NAT1	4.063461	9.758008	1.75E-10
ENSG0000(	HOXB-AS2	4.049272	1.428857	0.037251
ENSG0000(	SH2D1A	4.047632	6.911531	1.23E-07
ENSG0000(	LINC01679	4.04638	6.941137	1.15E-07
ENSG0000(	BCL2A1	4.035881	1.672392	0.021262
ENSG0000(	IGLV2-23	4.033299	6.183909	6.55E-07
ENSG0000(	RF02180	4.023151	1.411191	0.038798
ENSG0000(	TRAT1	4.017372	2.190312	0.006452
ENSG0000(	AP000915.	4.014228	4.869555	1.35E-05
ENSG0000(	FMO1	4.004341	3.44575	0.000358
ENSG0000(	LRFN1	3.99569	6.06171	8.68E-07
ENSG0000(	GPR65	3.988338	5.640452	2.29E-06
ENSG0000(	MARCO	3.987978	9.740789	1.82E-10
ENSG0000(	HLA-DQB2	3.984586	6.968862	1.07E-07
ENSG0000(	SLAMF6	3.982494	4.407397	3.91E-05
ENSG0000(	PRSS12	3.976941	5.752428	1.77E-06
ENSG0000(	MILR1	3.973618	7.826365	1.49E-08
ENSG0000(	AC015656.	3.970044	5.49678	3.19E-06
ENSG0000(	PDCD4-AS1	3.966482	7.919877	1.20E-08
ENSG0000(	IGLC1	3.964158	11.94095	1.15E-12
ENSG0000(	IGHV3-74	3.961655	4.591324	2.56E-05
ENSG0000(	FPR3	3.956458	13.12399	7.52E-14
ENSG0000(	PCBP3	3.949672	5.260928	5.48E-06
ENSG0000(	IGLC3	3.936158	12.41518	3.84E-13
ENSG0000(	C1QA	3.927783	26.38586	4.11E-27
ENSG0000(	RUBCNL	3.926589	10.98312	1.04E-11
ENSG0000(	DLGAP1-AS	3.92639	6.391507	4.06E-07
ENSG0000(	LPAR5	3.922736	4.009905	9.77E-05
ENSG0000(	IGKV3-20	3.917249	10.83122	1.47E-11
ENSG0000(	BCAS1	3.916183	5.973402	1.06E-06
ENSG0000(	CLEC4E	3.914735	2.384844	0.004122
ENSG0000(	AC008429.	3.91377	6.817302	1.52E-07
ENSG0000(	IGHA2	3.907379	7.634704	2.32E-08
ENSG0000(	CD3G	3.905787	3.721526	0.00019
ENSG0000(	AL049775.:	3.905045	3.634993	0.000232
ENSG0000(	PLA2G7	3.899659	2.764101	0.001721
ENSG0000(	AP005205.	3.89535	2.916099	0.001213
ENSG0000(	LINC00475	3.8844	3.918319	0.000121
ENSG0000(	CD300C	3.870464	2.964251	0.001086
ENSG0000(	FAM135B	3.869664	2.69533	0.002017
ENSG0000(	WFDC21P	3.868158	4.41371	3.86E-05

ENSG0000(	MIR1244-2	3.867066	3.01788	0.00096
ENSG0000(	CST7	3.860336	5.766353	1.71E-06
ENSG0000(	IGHV3-15	3.859242	5.659252	2.19E-06
ENSG0000(	ABCD2	3.859202	4.415631	3.84E-05
ENSG0000(	PNMA6A	3.854125	3.460745	0.000346
ENSG0000(	JCHAIN	3.852724	11.80748	1.56E-12
ENSG0000(	AC138207.	3.837825	11.08199	8.28E-12
ENSG0000(	SNX29P2	3.826023	1.786771	0.016339
ENSG0000(	AC116348.	3.824856	2.637241	0.002305
ENSG0000(	C1QC	3.818965	24.41093	3.88E-25
ENSG0000(	SCG2	3.818913	5.529513	2.95E-06
ENSG0000(	TSKS	3.81531	3.121223	0.000756
ENSG0000(	TRBJ2-2P	3.804153	1.748029	0.017864
ENSG0000(	HCAR2	3.803784	1.892868	0.012798
ENSG0000(	PTPN7	3.800714	6.431403	3.70E-07
ENSG0000(	LIPG	3.79435	5.085881	8.21E-06
ENSG0000(	VNN2	3.778658	3.210905	0.000615
ENSG0000(	CCL28	3.76721	3.055836	0.000879
ENSG0000(	MXRA5Y	3.765795	1.75844	0.017441
ENSG0000(	AC242842.	3.765008	3.409527	0.000389
ENSG0000(	PLCH2	3.739977	4.704806	1.97E-05
ENSG0000(	CCR4	3.730595	2.282206	0.005221
ENSG0000(	C3AR1	3.72716	12.42343	3.77E-13
ENSG0000(	GZMH	3.725047	5.171299	6.74E-06
ENSG0000(	AL161785.:	3.695546	3.016165	0.000963
ENSG0000(	GZMM	3.690046	4.767367	1.71E-05
ENSG0000(	TNFAIP8L2	3.687675	4.539298	2.89E-05
ENSG0000(	IPCEF1	3.682992	5.033679	9.25E-06
ENSG0000(	LINC02593	3.682203	2.665988	0.002158
ENSG0000(	IRF8	3.67838	19.34108	4.56E-20
ENSG0000(	MIR222HG	3.670718	3.910636	0.000123
ENSG0000(	IFI30	3.66631	14.83386	1.47E-15
ENSG0000(	C4B	3.665867	6.810264	1.55E-07
ENSG0000(	NAPSB	3.662086	5.016364	9.63E-06
ENSG0000(	ZC3H12D	3.654781	4.524208	2.99E-05
ENSG0000(	AL160272.:	3.638941	4.589521	2.57E-05
ENSG0000(	DUSP15	3.636896	7.034676	9.23E-08
ENSG0000(	EXOC3L4	3.635357	8.739668	1.82E-09
ENSG0000(	GPR132	3.622395	5.870427	1.35E-06
ENSG0000(	TBX21	3.617979	3.351766	0.000445
ENSG0000(	IGKV1-27	3.612595	2.901291	0.001255
ENSG0000(	CD8A	3.604181	8.549205	2.82E-09
ENSG0000(	RANBP3L	3.60337	2.681749	0.002081
ENSG0000(	TNFRSF10C	3.591732	2.214074	0.006108
ENSG0000(	TFF3	3.582865	3.43067	0.000371
ENSG0000(	AC109460.	3.57546	3.26112	0.000548
ENSG0000(	C2	3.57174	25.37289	4.24E-26

ENSG0000(	LRRC4C	3.565624	6.629842	2.35E-07
ENSG0000(	ACTL10	3.564807	2.705752	0.001969
ENSG0000(	FCER1A	3.563226	4.865602	1.36E-05
ENSG0000(	ACP5	3.562759	13.97518	1.06E-14
ENSG0000(	TTC16	3.549248	1.832012	0.014723
ENSG0000(	LINC00173	3.548154	3.672421	0.000213
ENSG0000(	AC092919.	3.544661	3.062376	0.000866
ENSG0000(	AC046185.	3.543339	2.976352	0.001056
ENSG0000(	TNFRSF10A	3.536407	3.036872	0.000919
ENSG0000(	AC244502.	3.529747	2.562326	0.00274
ENSG0000(	TPSD1	3.523507	1.936874	0.011564
ENSG0000(	AC074386.	3.521499	3.685694	0.000206
ENSG0000(	TNFSF4	3.520914	4.79739	1.59E-05
ENSG0000(	TKTL1	3.51609	4.383944	4.13E-05
ENSG0000(	CD163	3.515973	13.19477	6.39E-14
ENSG0000(	IL2RA	3.509311	2.923008	0.001194
ENSG0000(	TRDC	3.507588	1.80716	0.01559
ENSG0000(	MSR1	3.505968	12.45107	3.54E-13
ENSG0000(	AC098864.	3.503329	7.556425	2.78E-08
ENSG0000(	PTPN22	3.503161	6.539942	2.88E-07
ENSG0000(	AL157955.:	3.495709	1.797093	0.015955
ENSG0000(	CD33	3.491644	5.45358	3.52E-06
ENSG0000(	GZMB	3.490928	4.101111	7.92E-05
ENSG0000(	AC091152.	3.488589	2.880031	0.001318
ENSG0000(	CARMIL3	3.485231	2.900286	0.001258
ENSG0000(	LINC01410	3.479788	3.142436	0.00072
ENSG0000(	AC092143.	3.47721	3.358253	0.000438
ENSG0000(	SP140	3.477146	6.274291	5.32E-07
ENSG0000(	ICAM5	3.47653	2.966441	0.00108
ENSG0000(	HCST	3.475924	6.195995	6.37E-07
ENSG0000(	TRBC1	3.473072	4.475392	3.35E-05
ENSG0000(	AL118508.:	3.470247	4.015498	9.65E-05
ENSG0000(	AC138207.	3.467428	2.78492	0.001641
ENSG0000(	TMEM236	3.466768	3.454565	0.000351
ENSG0000(	TMEM225I	3.466462	3.395241	0.000402
ENSG0000(	PLA2G5	3.464926	1.635696	0.023137
ENSG0000(	AC243960.	3.459322	1.565716	0.027182
ENSG0000(	GPR39	3.443248	1.729354	0.018649
ENSG0000(	LTK	3.44204	3.969919	0.000107
ENSG0000(	AC090559.	3.42768	5.268211	5.39E-06
ENSG0000(	AC087477.	3.426372	2.429889	0.003716
ENSG0000(	AF165147.	3.425245	1.510752	0.03085
ENSG0000(	AC116348.	3.425076	3.711678	0.000194
ENSG0000(	C15orf53	3.420238	3.576222	0.000265
ENSG0000(	LYZ	3.41914	12.14458	7.17E-13
ENSG0000(	C4orf48	3.413526	1.982984	0.0104
ENSG0000(	CPNE5	3.412779	5.825254	1.50E-06

ENSG0000(	GBP1	3.412515	15.4733	3.36E-16
ENSG0000(	GDF15	3.405156	5.206506	6.22E-06
ENSG0000(	IL21R	3.404902	4.287631	5.16E-05
ENSG0000(	FCGR3A	3.400865	9.347257	4.50E-10
ENSG0000(	HLA-DQA1	3.395902	15.63439	2.32E-16
ENSG0000(	CCL2	3.392512	12.73508	1.84E-13
ENSG0000(	TLR1	3.391789	6.085721	8.21E-07
ENSG0000(	GBP7	3.38856	1.606239	0.024761
ENSG0000(	AC138207.	3.388451	4.715267	1.93E-05
ENSG0000(	RFX8	3.386468	1.664657	0.021644
ENSG0000(	TYROBP	3.384891	19.14162	7.22E-20
ENSG0000(	MATK	3.382008	4.086407	8.20E-05
ENSG0000(	DLEU1-AS1	3.380368	1.616563	0.024179
ENSG0000(	CD52	3.37879	5.272248	5.34E-06
ENSG0000(	COL9A2	3.375164	5.576643	2.65E-06
ENSG0000(	CD1C	3.374099	4.267728	5.40E-05
ENSG0000(	PLA2G2A	3.371849	4.376748	4.20E-05
ENSG0000(	TWIST1	3.371725	4.072365	8.47E-05
ENSG0000(	TNFAIP6	3.368079	5.625437	2.37E-06
ENSG0000(	GRIA3	3.367886	2.869071	0.001352
ENSG0000(	AC138207.	3.366974	2.392722	0.004048
ENSG0000(	SCIN	3.366277	2.695311	0.002017
ENSG0000(	CD5	3.359898	4.285898	5.18E-05
ENSG0000(	PNMA3	3.357123	5.712335	1.94E-06
ENSG0000(	SAMSN1	3.356391	5.304601	4.96E-06
ENSG0000(	AL121985.:	3.355815	3.498706	0.000317
ENSG0000(	TNFSF14	3.353157	4.902972	1.25E-05
ENSG0000(	FOLR2	3.352132	18.27198	5.35E-19
ENSG0000(	SIGLEC16	3.35095	3.005318	0.000988
ENSG0000(	ALDH3A1	3.350132	3.442514	0.000361
ENSG0000(	MS4A4A	3.349287	16.94127	1.14E-17
ENSG0000(	CCDC74A	3.347784	2.840998	0.001442
ENSG0000(	HSD17B3	3.346948	4.043421	9.05E-05
ENSG0000(	IL17B	3.343355	2.446452	0.003577
ENSG0000(	MYH8	3.33776	8.03175	9.30E-09
ENSG0000(	LEFTY2	3.336298	1.525629	0.029811
ENSG0000(	SLC22A15	3.336165	5.999533	1.00E-06
ENSG0000(	TNFSF13B	3.331708	11.57789	2.64E-12
ENSG0000(	CLEC10A	3.331066	14.84149	1.44E-15
ENSG0000(	C16orf89	3.330168	2.970288	0.001071
ENSG0000(	GPR137B	3.327819	7.68458	2.07E-08
ENSG0000(	AC008555.	3.320424	2.873245	0.001339
ENSG0000(	PCED1B-AS	3.319485	4.242536	5.72E-05
ENSG0000(	CR1	3.319462	4.283922	5.20E-05
ENSG0000(	FLT3	3.318294	1.821829	0.015072
ENSG0000(	C5orf58	3.31648	4.764775	1.72E-05
ENSG0000(	NCF1B	3.314935	5.168281	6.79E-06

ENSG0000(	TRBJ2-5	3.313286	1.387071	0.041014
ENSG0000(	GPAA1P2	3.306827	1.623376	0.023803
ENSG0000(	GNA15	3.304818	7.428794	3.73E-08
ENSG0000(	AC245100.	3.300906	5.564432	2.73E-06
ENSG0000(	ZCCHC18	3.296835	1.946586	0.011309
ENSG0000(	ARHGEF34I	3.294942	1.626307	0.023642
ENSG0000(	PLEK	3.292758	16.66766	2.15E-17
ENSG0000(	MYL4	3.292172	3.877722	0.000133
ENSG0000(	CCDC89	3.291757	2.708209	0.001958
ENSG0000(	AC020656.	3.291339	10.2427	5.72E-11
ENSG0000(	PIK3CG	3.290875	7.503606	3.14E-08
ENSG0000(	PRICKLE2-A	3.28703	1.966941	0.010791
ENSG0000(	AC027559.	3.280304	1.623969	0.02377
ENSG0000(	SIRPG	3.276308	1.37148	0.042513
ENSG0000(	PTPRO	3.276288	5.645367	2.26E-06
ENSG0000(	ADGRB1	3.272032	5.96717	1.08E-06
ENSG0000(	MPEG1	3.269233	22.10334	7.88E-23
ENSG0000(	SPINK2	3.263377	1.375175	0.042153
ENSG0000(	PTHLH	3.262641	3.205151	0.000624
ENSG0000(	XCR1	3.261342	3.371845	0.000425
ENSG0000(	ENAM	3.257241	9.71209	1.94E-10
ENSG0000(	SIRPB2	3.257229	5.168128	6.79E-06
ENSG0000(	S100A3	3.256586	3.99203	0.000102
ENSG0000(	IKZF3	3.253269	6.860284	1.38E-07
ENSG0000(	DOK2	3.250065	14.25599	5.55E-15
ENSG0000(	IL21R-AS1	3.248306	3.537257	0.00029
ENSG0000(	AC244021.	3.247918	3.256034	0.000555
ENSG0000(	CD209	3.245479	17.61017	2.45E-18
ENSG0000(	GPBAR1	3.244383	3.292493	0.00051
ENSG0000(	TMSB4XP8	3.241354	7.257658	5.53E-08
ENSG0000(	AC025576.	3.223628	1.769628	0.016997
ENSG0000(	CD4	3.22175	20.54173	2.87E-21
ENSG0000(	F13A1	3.221174	19.14839	7.11E-20
ENSG0000(	CASS4	3.220217	4.495987	3.19E-05
ENSG0000(	RTN4R	3.219887	3.634993	0.000232
ENSG0000(	SENCR	3.216847	1.881577	0.013135
ENSG0000(	BATF3	3.216476	5.582728	2.61E-06
ENSG0000(	AC004846.	3.216287	4.438878	3.64E-05
ENSG0000(	CD8B	3.216207	3.0664	0.000858
ENSG0000(	AL023806.:	3.212594	2.275663	0.005301
ENSG0000(	MMP27	3.210771	2.307974	0.004921
ENSG0000(	SNORA16A	3.209565	2.188375	0.006481
ENSG0000(	IQGAP2	3.207816	12.53686	2.90E-13
ENSG0000(	MEFV	3.204621	1.599883	0.025126
ENSG0000(	AD000864.	3.204446	6.141537	7.22E-07
ENSG0000(	TRBJ2-3	3.199266	3.690985	0.000204
ENSG0000(	LINC00248	3.197724	1.629612	0.023463

ENSG0000(	SH2D2A	3.195498	3.455116	0.000351
ENSG0000(	AOAH	3.195346	5.017978	9.59E-06
ENSG0000(	MEI1	3.194712	5.688208	2.05E-06
ENSG0000(	CD163L1	3.191827	12.19361	6.40E-13
ENSG0000(	MS4A6A	3.189905	19.32332	4.75E-20
ENSG0000(	LCP1	3.189454	16.4077	3.91E-17
ENSG0000(	FUT7	3.188519	1.518398	0.030311
ENSG0000(	TRBJ2-1	3.185221	1.614995	0.024266
ENSG0000(	CD14	3.184858	14.84149	1.44E-15
ENSG0000(	ZBP1	3.167798	4.718128	1.91E-05
ENSG0000(	FAM90A1	3.163629	2.165466	0.006832
ENSG0000(	MS4A7	3.163326	20.74599	1.79E-21
ENSG0000(	ITGB2-AS1	3.159706	3.730599	0.000186
ENSG0000(	SLC24A2	3.159174	2.789449	0.001624
ENSG0000(	LAPTM5	3.15866	17.67865	2.10E-18
ENSG0000(	AL390755.:	3.156831	1.545667	0.028466
ENSG0000(	AP003396.	3.149736	8.964741	1.08E-09
ENSG0000(	Z83844.2	3.148201	6.841574	1.44E-07
ENSG0000(	SLC6A12	3.14005	1.315463	0.048366
ENSG0000(	FCGR2A	3.140009	13.09528	8.03E-14
ENSG0000(	PRRX2	3.135522	3.685497	0.000206
ENSG0000(	RARRES3	3.13508	19.25474	5.56E-20
ENSG0000(	CCL5	3.134429	9.265217	5.43E-10
ENSG0000(	HCP5	3.133273	17.23345	5.84E-18
ENSG0000(	PKHD1	3.132602	1.658744	0.021941
ENSG0000(	C5AR1	3.129478	4.411836	3.87E-05
ENSG0000(	PDZK1	3.126519	1.568078	0.027035
ENSG0000(	ATAD3C	3.125851	2.136923	0.007296
ENSG0000(	HLA-DOA	3.125538	14.32942	4.68E-15
ENSG0000(	PBX4	3.124243	4.027441	9.39E-05
ENSG0000(	BLM	3.123264	2.769164	0.001702
ENSG0000(	AL023653.:	3.121315	2.548807	0.002826
ENSG0000(	AL360270.:	3.121227	3.058401	0.000874
ENSG0000(	HPGDS	3.120612	5.13799	7.28E-06
ENSG0000(	AC011462.	3.120373	2.347627	0.004491
ENSG0000(	MRC1	3.119869	18.26597	5.42E-19
ENSG0000(	MYCL	3.117876	4.302413	4.98E-05
ENSG0000(	CYTIP	3.115769	4.108995	7.78E-05
ENSG0000(	THY1	3.108278	9.312617	4.87E-10
ENSG0000(	CENPM	3.108016	3.716304	0.000192
ENSG0000(	CSTA	3.105802	2.324757	0.004734
ENSG0000(	HSPA6	3.101991	4.334465	4.63E-05
ENSG0000(	AL133467.:	3.101768	2.107208	0.007813
ENSG0000(	IL22RA1	3.094004	1.762893	0.017263
ENSG0000(	AL590764.:	3.093902	10.81856	1.52E-11
ENSG0000(	AL356489.:	3.092958	1.308173	0.049184
ENSG0000(	B3GAT1	3.090894	1.979089	0.010493

ENSG0000(	AL031848.:	3.089064	2.896583	0.001269
ENSG0000(	ANXA2P2	3.087424	4.307028	4.93E-05
ENSG0000(	RAET1E	3.086087	3.11878	0.000761
ENSG0000(	PENK	3.083816	8.939526	1.15E-09
ENSG0000(	BX255923.	3.079974	2.358658	0.004379
ENSG0000(	HLA-DRA	3.076343	20.24323	5.71E-21
ENSG0000(	SKAP1	3.074581	3.764911	0.000172
ENSG0000(	LILRA6	3.071267	5.671729	2.13E-06
ENSG0000(	AC005722.	3.070651	1.954911	0.011094
ENSG0000(	AL035587.:	3.07043	1.702585	0.019834
ENSG0000(	WHRN	3.069127	5.22814	5.91E-06
ENSG0000(	AC012349.	3.068769	7.131426	7.39E-08
ENSG0000(	C2CD4B	3.064932	1.867168	0.013578
ENSG0000(	NFAM1	3.064922	7.471246	3.38E-08
ENSG0000(	NCF1C	3.061237	6.326732	4.71E-07
ENSG0000(	DUXAP9	3.060551	1.939958	0.011483
ENSG0000(	PPP2R2B	3.060259	3.236692	0.00058
ENSG0000(	KIAA1549L	3.059642	2.881911	0.001312
ENSG0000(	LINC00426	3.058372	1.61713	0.024147
ENSG0000(	CTSK	3.052333	7.683619	2.07E-08
ENSG0000(	SPNS3	3.052052	1.416884	0.038293
ENSG0000(	TMEM154	3.049312	5.048546	8.94E-06
ENSG0000(	BICDL1	3.048762	3.043709	0.000904
ENSG0000(	PSMB9	3.048367	20.62996	2.34E-21
ENSG0000(	DOCK2	3.048073	13.51738	3.04E-14
ENSG0000(	CLEC12A	3.047962	1.668457	0.021456
ENSG0000(	HLA-DOB	3.047047	5.091987	8.09E-06
ENSG0000(	DKFZP434H	3.045169	1.462867	0.034446
ENSG0000(	CYBB	3.035181	13.39276	4.05E-14
ENSG0000(	ZNF572	3.031887	1.309784	0.049002
ENSG0000(	PTPRC	3.029586	13.28636	5.17E-14
ENSG0000(	AIM2	3.025914	2.572903	0.002674
ENSG0000(	PFN4	3.025585	1.700793	0.019916
ENSG0000(	AC242842.	3.021407	3.14518	0.000716
ENSG0000(	Z95114.3	3.019136	2.48996	0.003236
ENSG0000(	AC073332.	3.018579	4.461174	3.46E-05
ENSG0000(	PSMB8	3.017505	23.63528	2.32E-24
ENSG0000(	RTP4	3.012332	4.871321	1.34E-05
ENSG0000(	PLA2G4A	3.009862	5.185715	6.52E-06
ENSG0000(	CTSS	3.005997	16.99225	1.02E-17
ENSG0000(	ITGA4	3.005933	6.615753	2.42E-07
ENSG0000(	EMB	3.000905	21.60554	2.48E-22
ENSG0000(	ASPN	2.997483	6.362241	4.34E-07
ENSG0000(	TRAC	2.996526	10.37452	4.22E-11
ENSG0000(	CD53	2.995366	14.01041	9.76E-15
ENSG0000(	AL512791.:	2.99366	1.973173	0.010637
ENSG0000(	TRBJ2-7	2.991611	2.001069	0.009975

ENSG0000(	ANKRD36B	2.985206	1.680556	0.020866
ENSG0000(	AC012645.	2.985034	12.33211	4.65E-13
ENSG0000(	STX1B	2.983509	10.90538	1.24E-11
ENSG0000(	IL2RG	2.983414	10.25927	5.50E-11
ENSG0000(	AL512506.:	2.978979	1.605652	0.024794
ENSG0000(	NFIA-AS2	2.976363	1.397375	0.040052
ENSG0000(	AC092958.	2.974918	1.916545	0.012119
ENSG0000(	EVI2B	2.973576	8.61655	2.42E-09
ENSG0000(	SNCAIP	2.971904	4.668172	2.15E-05
ENSG0000(	NCKAP1L	2.970528	19.48356	3.28E-20
ENSG0000(	MIR4292	2.968264	1.941541	0.011441
ENSG0000(	FAM227A	2.968195	1.907851	0.012364
ENSG0000(	DNASE1L3	2.964545	3.224809	0.000596
ENSG0000(	ZNF215	2.963351	2.62006	0.002399
ENSG0000(	MS4A14	2.962222	5.318102	4.81E-06
ENSG0000(	CD84	2.962194	14.14122	7.22E-15
ENSG0000(	GFRA2	2.95737	9.537409	2.90E-10
ENSG0000(	BOK-AS1	2.956627	3.295615	0.000506
ENSG0000(	AC121757.	2.951316	1.559542	0.027571
ENSG0000(	HLA-DPA1	2.950529	18.81921	1.52E-19
ENSG0000(	ABHD11-A!	2.94883	2.540268	0.002882
ENSG0000(	AL021328.:	2.944561	1.352635	0.044398
ENSG0000(	KLRB1	2.944293	1.660573	0.021849
ENSG0000(	NUDT11	2.940903	1.850084	0.014123
ENSG0000(	IL2RB	2.939796	4.179754	6.61E-05
ENSG0000(	AL136116.:	2.939337	1.356371	0.044018
ENSG0000(	OTOF	2.936323	2.983116	0.00104
ENSG0000(	MIR199A2	2.935058	2.111459	0.007736
ENSG0000(	AIF1	2.934804	13.51738	3.04E-14
ENSG0000(	HLA-DPB1	2.934015	17.5134	3.07E-18
ENSG0000(	POSTN	2.93386	9.122696	7.54E-10
ENSG0000(	RF01979	2.930342	3.078964	0.000834
ENSG0000(	SECTM1	2.930234	9.514908	3.06E-10
ENSG0000(	SASH3	2.929557	5.438281	3.65E-06
ENSG0000(	ITGB2	2.929295	12.87556	1.33E-13
ENSG0000(	AL645939.:	2.927103	17.48572	3.27E-18
ENSG0000(	CCL27	2.926855	1.442609	0.03609
ENSG0000(	HLA-DQB1-	2.925631	9.033528	9.26E-10
ENSG0000(	CYP2D7	2.922433	2.095806	0.00802
ENSG0000(	IL10RA	2.921748	15.25322	5.58E-16
ENSG0000(	HASPIN	2.92156	2.119916	0.007587
ENSG0000(	TMEM200/	2.921331	4.778009	1.67E-05
ENSG0000(	WFDC3	2.921203	1.624302	0.023752
ENSG0000(	AP001372.	2.916145	2.920097	0.001202
ENSG0000(	APOL4	2.914999	17.70797	1.96E-18
ENSG0000(	ADAP1	2.914757	8.638495	2.30E-09
ENSG0000(	CRIP3	2.914549	1.564143	0.027281

ENSG0000(	WNT10B	2.911815	1.977694	0.010527
ENSG0000(	MGP	2.909186	8.146283	7.14E-09
ENSG0000(	CRISPLD1	2.906809	3.243369	0.000571
ENSG0000(	SLAMF7	2.904567	10.19812	6.34E-11
ENSG0000(	CTSW	2.903822	8.056107	8.79E-09
ENSG0000(	AC138028.	2.901611	3.7807	0.000166
ENSG0000(	SHANK1	2.898185	2.920407	0.001201
ENSG0000(	MCOLN2	2.897857	2.465364	0.003425
ENSG0000(	MIR4271	2.89544	2.864983	0.001365
ENSG0000(	RNU4-2	2.894047	4.619672	2.40E-05
ENSG0000(	C12orf60	2.893997	8.084076	8.24E-09
ENSG0000(	LUM	2.893224	7.400839	3.97E-08
ENSG0000(	CLIC3	2.892415	1.385468	0.041165
ENSG0000(	FCER1G	2.89	11.30081	5.00E-12
ENSG0000(	CCDC15	2.88775	1.399964	0.039814
ENSG0000(	GSC	2.886673	2.657716	0.002199
ENSG0000(	XKR6	2.884764	2.522396	0.003003
ENSG0000(	SLC29A3	2.881863	5.97382	1.06E-06
ENSG0000(	OPRL1	2.879502	3.058512	0.000874
ENSG0000(	LINC01121	2.879224	2.151244	0.007059
ENSG0000(	CD3D	2.875708	5.269693	5.37E-06
ENSG0000(	NCF2	2.875094	11.20976	6.17E-12
ENSG0000(	AC099548.	2.874189	1.592047	0.025583
ENSG0000(	SLC22A18A	2.871007	1.705486	0.019702
ENSG0000(	PHF21B	2.868721	2.102094	0.007905
ENSG0000(	P2RX7	2.865555	7.893654	1.28E-08
ENSG0000(	AL671883.:	2.863131	20.00314	9.93E-21
ENSG0000(	TRPC6	2.862776	1.427998	0.037325
ENSG0000(	IL1RL2	2.860462	1.54124	0.028758
ENSG0000(	FGF7	2.859484	10.58453	2.60E-11
ENSG0000(	PIK3AP1	2.858771	6.203156	6.26E-07
ENSG0000(	ZNF826P	2.858183	3.060019	0.000871
ENSG0000(	BLNK	2.853714	5.233924	5.84E-06
ENSG0000(	IKBKE	2.853657	5.657065	2.20E-06
ENSG0000(	IGF2-AS	2.852028	2.911977	0.001225
ENSG0000(	CCDC153	2.85174	2.768656	0.001704
ENSG0000(	ARHGAP30	2.850937	14.23109	5.87E-15
ENSG0000(	EPSTI1	2.850559	13.5661	2.72E-14
ENSG0000(	SLA	2.847189	10.8832	1.31E-11
ENSG0000(	PIK3R5	2.843469	5.58594	2.59E-06
ENSG0000(	RAB39B	2.842538	1.305007	0.049544
ENSG0000(	BDKRB2	2.838728	5.34155	4.55E-06
ENSG0000(	NCKAP5	2.83677	2.990237	0.001023
ENSG0000(	MXRA5	2.83623	11.577	2.65E-12
ENSG0000(	LILRB5	2.833335	19.21416	6.11E-20
ENSG0000(	KIF14	2.831267	1.728744	0.018675
ENSG0000(	AC092171.	2.830443	1.36014	0.043637

ENSG0000(	LILRA2	2.827052	1.319821	0.047883
ENSG0000(	LGALS17A	2.825973	4.083763	8.25E-05
ENSG0000(	CD6	2.825667	4.414512	3.85E-05
ENSG0000(	AC011484.	2.823066	2.674579	0.002116
ENSG0000(	PTGES	2.821377	4.386364	4.11E-05
ENSG0000(	AL138756.:	2.821281	2.475737	0.003344
ENSG0000(	MOXD1	2.817027	2.679674	0.002091
ENSG0000(	AC012629.	2.815665	3.517912	0.000303
ENSG0000(	RALY-AS1	2.814525	3.167399	0.00068
ENSG0000(	MIR5001	2.809579	2.643426	0.002273
ENSG0000(	PSTPIP1	2.808314	8.107862	7.80E-09
ENSG0000(	FCHO1	2.807414	3.617311	0.000241
ENSG0000(	WDFY4	2.806348	9.913018	1.22E-10
ENSG0000(	LILRA1	2.802353	1.591714	0.025603
ENSG0000(	IRX1	2.802093	1.328761	0.046907
ENSG0000(	CCDC80	2.799764	11.6249	2.37E-12
ENSG0000(	HLA-C	2.792891	26.38586	4.11E-27
ENSG0000(	AC060234.	2.792223	3.762918	0.000173
ENSG0000(	OMD	2.792178	6.594128	2.55E-07
ENSG0000(	PTAFR	2.791926	10.62824	2.35E-11
ENSG0000(	JHY	2.787849	2.5929	0.002553
ENSG0000(	AC063949.	2.787326	3.040098	0.000912
ENSG0000(	HLA-H	2.783661	9.557363	2.77E-10
ENSG0000(	FLJ46906	2.783433	2.948646	0.001126
ENSG0000(	GPR34	2.779858	7.655366	2.21E-08
ENSG0000(	TMEM176/	2.779826	18.27198	5.35E-19
ENSG0000(	AL590385.:	2.773303	7.459181	3.47E-08
ENSG0000(	SCN5A	2.772413	3.545897	0.000285
ENSG0000(	AC015911.	2.771066	1.670372	0.021361
ENSG0000(	HLA-DQB1	2.768114	8.882684	1.31E-09
ENSG0000(	HLA-F	2.767609	18.55648	2.78E-19
ENSG0000(	FCGR2B	2.767448	7.520374	3.02E-08
ENSG0000(	TLR2	2.76643	4.110088	7.76E-05
ENSG0000(	SELPLG	2.766369	9.504898	3.13E-10
ENSG0000(	NPAS3	2.766233	3.428016	0.000373
ENSG0000(	TMEM176I	2.763428	16.53629	2.91E-17
ENSG0000(	ESPNL	2.757346	1.794258	0.01606
ENSG0000(	AC018647.	2.756273	1.938854	0.011512
ENSG0000(	TWIST2	2.754938	2.178816	0.006625
ENSG0000(	AL596094.:	2.752474	1.802742	0.015749
ENSG0000(	HLA-B	2.752096	21.26998	5.37E-22
ENSG0000(	AC093732.	2.751393	2.392673	0.004049
ENSG0000(	DIRAS1	2.751094	5.24855	5.64E-06
ENSG0000(	CSF1R	2.747638	19.24762	5.65E-20
ENSG0000(	SUSD3	2.747304	1.658505	0.021953
ENSG0000(	DBH	2.747106	3.319442	0.000479
ENSG0000(	IFIT3	2.746074	13.70713	1.96E-14

ENSG0000(	FUOM	2.743786	6.380986	4.16E-07
ENSG0000(	AC000123.	2.743692	1.896425	0.012693
ENSG0000(	LYVE1	2.741722	8.249253	5.63E-09
ENSG0000(	NCF1	2.740545	7.217718	6.06E-08
ENSG0000(	LCK	2.736637	4.521357	3.01E-05
ENSG0000(	TLR6	2.735004	3.109477	0.000777
ENSG0000(	VSIG10L	2.732427	3.692745	0.000203
ENSG0000(	STAT1	2.731136	12.41697	3.83E-13
ENSG0000(	AC010328.	2.729819	1.454107	0.035147
ENSG0000(	KLRK1	2.728446	8.078561	8.35E-09
ENSG0000(	AC010654.	2.727857	1.493942	0.032067
ENSG0000(	AC016888.	2.727417	8.553522	2.80E-09
ENSG0000(	CD68	2.727102	15.12991	7.41E-16
ENSG0000(	FXYD2	2.727023	3.953685	0.000111
ENSG0000(	HLA-DMB	2.726528	17.60121	2.50E-18
ENSG0000(	AL365475.:	2.725819	1.799879	0.015853
ENSG0000(	AL645941.:	2.724833	16.6744	2.12E-17
ENSG0000(	CCR1	2.721924	8.708619	1.96E-09
ENSG0000(	PRDM6	2.721364	2.282155	0.005222
ENSG0000(	PAMR1	2.720454	10.25927	5.50E-11
ENSG0000(	CD74	2.71989	17.50052	3.16E-18
ENSG0000(	RBP5	2.71935	6.053958	8.83E-07
ENSG0000(	AC010605.	2.718475	1.884012	0.013061
ENSG0000(	HIST3H2A	2.711401	3.425166	0.000376
ENSG0000(	TMEM119	2.710901	10.5661	2.72E-11
ENSG0000(	OR2A1-AS1	2.708367	1.591185	0.025634
ENSG0000(	ITGB7	2.707942	9.543675	2.86E-10
ENSG0000(	RUNX3	2.706293	7.088453	8.16E-08
ENSG0000(	IGSF6	2.701757	12.02403	9.46E-13
ENSG0000(	LINC00506	2.701507	1.764742	0.017189
ENSG0000(	ITGAM	2.700958	14.14491	7.16E-15
ENSG0000(	TRBC2	2.699689	7.573531	2.67E-08
ENSG0000(	LINC01497	2.698089	1.791881	0.016148
ENSG0000(	RRN3P2	2.69659	2.260942	0.005484
ENSG0000(	VCAM1	2.696287	12.93196	1.17E-13
ENSG0000(	HLA-DRB1	2.695795	16.83973	1.45E-17
ENSG0000(	CD300A	2.692321	3.591881	0.000256
ENSG0000(	AL034550.:	2.69212	1.36054	0.043597
ENSG0000(	APOBEC3G	2.689375	14.33084	4.67E-15
ENSG0000(	TBXAS1	2.685487	9.145111	7.16E-10
ENSG0000(	SVOP	2.685115	3.077578	0.000836
ENSG0000(	HDX	2.684896	4.366101	4.30E-05
ENSG0000(	KLRC4-KLRI	2.683694	9.478454	3.32E-10
ENSG0000(	AL031595.:	2.681192	7.146164	7.14E-08
ENSG0000(	ADAMTSL1	2.681035	4.439964	3.63E-05
ENSG0000(	CPXM1	2.680228	5.210019	6.17E-06
ENSG0000(	SIGLEC1	2.677957	10.93105	1.17E-11

ENSG0000(	CLEC7A	2.677436	5.501546	3.15E-06
ENSG0000(	BCL11B	2.675314	2.465309	0.003425
ENSG0000(	LILRB1	2.674587	4.23101	5.87E-05
ENSG0000(	PYCARD-AS	2.674422	18.50947	3.09E-19
ENSG0000(	CSF2RB	2.67191	12.87732	1.33E-13
ENSG0000(	HLA-J	2.671282	10.04212	9.08E-11
ENSG0000(	PPM1E	2.670133	3.282906	0.000521
ENSG0000(	CD3E	2.669504	5.747751	1.79E-06
ENSG0000(	PCA3	2.669175	6.750796	1.78E-07
ENSG0000(	C9orf139	2.668813	2.520367	0.003017
ENSG0000(	C1R	2.665629	13.51738	3.04E-14
ENSG0000(	TMEM121	2.664832	2.667089	0.002152
ENSG0000(	AC245595.	2.662438	1.841437	0.014407
ENSG0000(	NKG7	2.657274	3.261548	0.000548
ENSG0000(	PYCARD	2.656763	18.27198	5.35E-19
ENSG0000(	WISP2	2.655977	6.495562	3.19E-07
ENSG0000(	LAD1	2.653474	6.937901	1.15E-07
ENSG0000(	PIMREG	2.652885	1.379376	0.041747
ENSG0000(	NELL2	2.651516	3.353083	0.000444
ENSG0000(	SDK2	2.648952	3.221229	0.000601
ENSG0000(	HCK	2.647644	7.517709	3.04E-08
ENSG0000(	OTULINL	2.645466	10.12574	7.49E-11
ENSG0000(	SPI1	2.643318	13.0041	9.91E-14
ENSG0000(	ANO9	2.642082	2.832704	0.00147
ENSG0000(	PRUNE2	2.640273	14.92314	1.19E-15
ENSG0000(	ADAP2	2.639273	10.97704	1.05E-11
ENSG0000(	DLGAP1-AS	2.63858	7.832755	1.47E-08
ENSG0000(	MIR6891	2.637254	17.39272	4.05E-18
ENSG0000(	PSMB8-AS1	2.636129	18.64888	2.24E-19
ENSG0000(	AC241377.	2.63593	2.096381	0.00801
ENSG0000(	BATF2	2.635528	8.452889	3.52E-09
ENSG0000(	AC003072.	2.633801	2.052171	0.008868
ENSG0000(	CMA1	2.633494	3.037691	0.000917
ENSG0000(	LAIR1	2.630885	13.87422	1.34E-14
ENSG0000(	CCDC8	2.629526	12.58453	2.60E-13
ENSG0000(	EMILIN2	2.627968	9.451037	3.54E-10
ENSG0000(	APOL1	2.620839	17.18342	6.56E-18
ENSG0000(	SYK	2.619886	14.50292	3.14E-15
ENSG0000(	PARVG	2.615284	10.0901	8.13E-11
ENSG0000(	TNFAIP8L3	2.615073	10.83355	1.47E-11
ENSG0000(	RGS2	2.614415	15.30268	4.98E-16
ENSG0000(	ALOX5AP	2.610571	11.45871	3.48E-12
ENSG0000(	AL499627.1	2.609924	2.537852	0.002898
ENSG0000(	POU2AF1	2.604788	2.249669	0.005628
ENSG0000(	CORO1A	2.604253	9.503116	3.14E-10
ENSG0000(	FNDC1	2.602698	6.131751	7.38E-07
ENSG0000(	CTHRC1	2.602087	6.493174	3.21E-07

ENSG0000(	AC073283.	2.600596	1.603356	0.024926
ENSG0000(	AC254633.	2.599096	1.935999	0.011588
ENSG0000(	FRMD1	2.598558	3.428016	0.000373
ENSG0000(	BTK	2.598188	8.590527	2.57E-09
ENSG0000(	AC084398.	2.592697	2.463082	0.003443
ENSG0000(	MTX1P1	2.592462	4.515955	3.05E-05
ENSG0000(	IGSF1	2.591704	3.35152	0.000445
ENSG0000(	DACT2	2.590289	2.338886	0.004583
ENSG0000(	AL109918.:	2.589687	4.010615	9.76E-05
ENSG0000(	IKZF1	2.58801	7.296962	5.05E-08
ENSG0000(	CARMIL2	2.587282	3.280838	0.000524
ENSG0000(	GPR156	2.58655	1.666969	0.021529
ENSG0000(	GBP3	2.582058	13.83046	1.48E-14
ENSG0000(	AC010761.	2.58069	2.079796	0.008322
ENSG0000(	UBE2L6	2.578569	22.49551	3.20E-23
ENSG0000(	CYSLTR1	2.57733	3.292493	0.00051
ENSG0000(	FAM72A	2.576517	2.031663	0.009297
ENSG0000(	LINC02361	2.574877	1.452217	0.035301
ENSG0000(	C11orf45	2.574865	1.441798	0.036158
ENSG0000(	GBP1P1	2.574839	6.083274	8.26E-07
ENSG0000(	WEE2	2.574661	2.301175	0.004998
ENSG0000(	AL671277.:	2.574518	3.135885	0.000731
ENSG0000(	TYMP	2.573715	10.87593	1.33E-11
ENSG0000(	MIR3615	2.573341	7.836166	1.46E-08
ENSG0000(	AC116348.	2.573092	3.758322	0.000174
ENSG0000(	AL845552.:	2.572012	1.491956	0.032214
ENSG0000(	MPZL3	2.571387	2.567831	0.002705
ENSG0000(	ARHGAP15	2.569645	4.521161	3.01E-05
ENSG0000(	AC022075.	2.569318	5.260617	5.49E-06
ENSG0000(	RARRES1	2.566755	3.889688	0.000129
ENSG0000(	CILP	2.566657	9.147395	7.12E-10
ENSG0000(	HHAT	2.565686	4.575081	2.66E-05
ENSG0000(	C6	2.564	3.392579	0.000405
ENSG0000(	NOD2	2.563842	5.640528	2.29E-06
ENSG0000(	SERPINA1	2.563678	5.674596	2.12E-06
ENSG0000(	CHST11	2.563198	7.195699	6.37E-08
ENSG0000(	CLEC4A	2.563068	2.930391	0.001174
ENSG0000(	AC099063.	2.559804	6.390523	4.07E-07
ENSG0000(	ADAM28	2.559656	2.88874	0.001292
ENSG0000(	FCGBP	2.555141	3.844909	0.000143
ENSG0000(	TMSB4XP4	2.554593	2.301505	0.004995
ENSG0000(	XPNPEP2	2.554545	2.85626	0.001392
ENSG0000(	TAP1	2.553562	15.45412	3.51E-16
ENSG0000(	TIMP1	2.546417	12.79855	1.59E-13
ENSG0000(	HLA-A	2.545295	21.91184	1.23E-22
ENSG0000(	AC090907.	2.544748	3.308551	0.000491
ENSG0000(	MRGPRF-A	2.541352	1.638638	0.022981

ENSG0000(	CCND2-AS1	2.538844	2.201897	0.006282
ENSG0000(	B2M	2.537058	25.96411	1.09E-26
ENSG0000(	CD96	2.536581	3.606936	0.000247
ENSG0000(	NHLRC4	2.534669	2.028694	0.009361
ENSG0000(	MIR3606	2.534262	8.120158	7.58E-09
ENSG0000(	BTN3A2	2.53344	11.90542	1.24E-12
ENSG0000(	AC051619.	2.533382	2.22751	0.005922
ENSG0000(	SLC37A2	2.531992	10.33611	4.61E-11
ENSG0000(	GRIN2D	2.531215	1.664657	0.021644
ENSG0000(	RAB42	2.529478	2.292151	0.005103
ENSG0000(	TAGAP	2.529111	7.062457	8.66E-08
ENSG0000(	AP006621.	2.52824	2.289936	0.005129
ENSG0000(	CHRDL1	2.527067	6.907535	1.24E-07
ENSG0000(	C7	2.525933	4.426092	3.75E-05
ENSG0000(	AC066613.	2.525558	4.331783	4.66E-05
ENSG0000(	APOBEC3C	2.521271	26.90856	1.23E-27
ENSG0000(	HMSD	2.519083	4.27848	5.27E-05
ENSG0000(	CAMK4	2.518674	2.251922	0.005599
ENSG0000(	FGL2	2.51828	20.66301	2.17E-21
ENSG0000(	SOCS1	2.516092	3.360198	0.000436
ENSG0000(	FAM180B	2.51468	3.698803	0.0002
ENSG0000(	CD48	2.513208	3.545472	0.000285
ENSG0000(	COL3A1	2.510741	8.2613	5.48E-09
ENSG0000(	TNFAIP8	2.510324	14.12835	7.44E-15
ENSG0000(	IRF5	2.507175	8.62843	2.35E-09
ENSG0000(	FYB1	2.501108	7.73404	1.84E-08
ENSG0000(	HACD4	2.500759	6.86723	1.36E-07
ENSG0000(	RGL1	2.498611	12.63361	2.32E-13
ENSG0000(	IFI27	2.498457	9.260047	5.49E-10
ENSG0000(	APBB1IP	2.494171	5.465068	3.43E-06
ENSG0000(	SLC9A3R1	2.492658	20.74599	1.79E-21
ENSG0000(	ITPKA	2.490701	1.580211	0.02629
ENSG0000(	LGI2	2.490037	7.227954	5.92E-08
ENSG0000(	AC068987.	2.489745	1.774937	0.01679
ENSG0000(	LRRC17	2.48843	9.444812	3.59E-10
ENSG0000(	LOX	2.487036	4.88417	1.31E-05
ENSG0000(	GATA6	2.4841	1.844291	0.014312
ENSG0000(	CCDC150	2.484014	2.669353	0.002141
ENSG0000(	GNAS-AS1	2.482245	3.171227	0.000674
ENSG0000(	C4A	2.479974	6.075768	8.40E-07
ENSG0000(	ECM2	2.477619	8.712605	1.94E-09
ENSG0000(	ADA2	2.475656	14.5992	2.52E-15
ENSG0000(	AL671277.1	2.470602	18.44454	3.59E-19
ENSG0000(	BTG3-AS1	2.466648	2.260176	0.005493
ENSG0000(	AC004687.	2.466501	2.924446	0.00119
ENSG0000(	CCL21	2.465394	2.390875	0.004066
ENSG0000(	INSRR	2.465218	1.451736	0.03534

ENSG0000(	GCNT1	2.464721	24.39752	4.00E-25
ENSG0000(	FAM86HP	2.461129	2.823013	0.001503
ENSG0000(	GRIA1	2.461116	1.744397	0.018014
ENSG0000(	MIR198	2.460276	6.93433	1.16E-07
ENSG0000(	FAM218A	2.458872	1.723802	0.018889
ENSG0000(	ICAM1	2.458397	15.42796	3.73E-16
ENSG0000(	USP27X-AS	2.458022	2.065514	0.0086
ENSG0000(	FBP1	2.454721	7.223775	5.97E-08
ENSG0000(	AC006487.	2.45416	2.873999	0.001337
ENSG0000(	AP003392.	2.453647	1.333321	0.046417
ENSG0000(	ADRB1	2.452963	2.541338	0.002875
ENSG0000(	PRG4	2.451736	3.754279	0.000176
ENSG0000(	RND2	2.449704	4.851916	1.41E-05
ENSG0000(	COL1A2	2.449554	8.359363	4.37E-09
ENSG0000(	HSD17B14	2.446802	4.385	4.12E-05
ENSG0000(	LCP2	2.444541	12.41393	3.86E-13
ENSG0000(	AL591721.:	2.441121	2.776651	0.001672
ENSG0000(	DUXAP8	2.440598	2.310667	0.00489
ENSG0000(	IGSF22	2.440593	2.723805	0.001889
ENSG0000(	TMEM229I	2.440131	9.005822	9.87E-10
ENSG0000(	FNDC10	2.437916	1.383775	0.041326
ENSG0000(	CARD11	2.437608	2.728532	0.001868
ENSG0000(	NCF4	2.435214	8.734263	1.84E-09
ENSG0000(	RENBP	2.434927	14.75046	1.78E-15
ENSG0000(	ATP8B4	2.43379	6.626952	2.36E-07
ENSG0000(	CPA3	2.426678	4.883977	1.31E-05
ENSG0000(	MAP1A	2.426178	20.70065	1.99E-21
ENSG0000(	ADAMTS2	2.423509	6.874651	1.33E-07
ENSG0000(	AC011511.	2.423362	9.547807	2.83E-10
ENSG0000(	FGD2	2.422636	10.51204	3.08E-11
ENSG0000(	HCLS1	2.421637	12.35718	4.39E-13
ENSG0000(	ARL4C	2.418963	12.44253	3.61E-13
ENSG0000(	CCRL2	2.418814	3.438624	0.000364
ENSG0000(	SHTN1	2.417408	13.76064	1.74E-14
ENSG0000(	HOXB5	2.414136	1.383106	0.04139
ENSG0000(	RNASE1	2.413558	10.69926	2.00E-11
ENSG0000(	HJURP	2.413088	1.353345	0.044326
ENSG0000(	RAC2	2.411957	8.450434	3.54E-09
ENSG0000(	ADRA1B	2.409198	2.173725	0.006703
ENSG0000(	HLA-G	2.407487	7.364422	4.32E-08
ENSG0000(	TMEM45A	2.407166	5.780793	1.66E-06
ENSG0000(	GIPR	2.4064	1.346644	0.045015
ENSG0000(	LGALS3BP	2.405982	15.85533	1.40E-16
ENSG0000(	DCLK1	2.405854	14.3489	4.48E-15
ENSG0000(	GPC3	2.405194	8.277164	5.28E-09
ENSG0000(	ADAM32	2.403519	1.677964	0.020991
ENSG0000(	CAPG	2.402365	10.83355	1.47E-11

ENSG0000(	VAV1	2.4021	8.501009	3.15E-09
ENSG0000(	CYTH4	2.400546	14.79627	1.60E-15
ENSG0000(	ANO7L1	2.3985	2.165615	0.006829
ENSG0000(	ADAMTS14	2.39845	3.669831	0.000214
ENSG0000(	APOC1	2.395565	8.678754	2.10E-09
ENSG0000(	MYH3	2.395488	6.095524	8.03E-07
ENSG0000(	RASAL3	2.394238	7.366936	4.30E-08
ENSG0000(	SNX10	2.393798	8.153372	7.02E-09
ENSG0000(	AC010761.	2.390698	2.302227	0.004986
ENSG0000(	C1S	2.390577	12.61244	2.44E-13
ENSG0000(	DENND2D	2.39016	5.304601	4.96E-06
ENSG0000(	COL1A1	2.388945	6.818232	1.52E-07
ENSG0000(	ASCL5	2.38723	1.887237	0.012965
ENSG0000(	SLC41A2	2.385585	3.901248	0.000126
ENSG0000(	ANPEP	2.381236	5.775989	1.67E-06
ENSG0000(	AL359317.:	2.379728	3.561236	0.000275
ENSG0000(	RIBC1	2.379012	1.6845	0.020678
ENSG0000(	AC008105.	2.37897	4.248527	5.64E-05
ENSG0000(	AHNAK2	2.373713	5.536721	2.91E-06
ENSG0000(	C1QTNF4	2.373406	1.988426	0.01027
ENSG0000(	AC139495.	2.373007	2.062862	0.008652
ENSG0000(	SH3BP1	2.372974	10.46273	3.45E-11
ENSG0000(	AC011481.	2.372615	8.705629	1.97E-09
ENSG0000(	AC093484.	2.37251	3.601689	0.00025
ENSG0000(	LINC01943	2.369801	8.708787	1.96E-09
ENSG0000(	CD44	2.368339	13.58879	2.58E-14
ENSG0000(	AL109930.:	2.366771	1.503978	0.031334
ENSG0000(	TRAM1L1	2.365216	2.788709	0.001627
ENSG0000(	TNFSF9	2.3645	2.024204	0.009458
ENSG0000(	VASH2	2.361842	8.478112	3.33E-09
ENSG0000(	GK-AS1	2.358703	3.51685	0.000304
ENSG0000(	CLDN23	2.35735	2.559592	0.002757
ENSG0000(	CTSG	2.356315	6.023346	9.48E-07
ENSG0000(	AC002398.	2.354346	1.955983	0.011067
ENSG0000(	CLDND2	2.354288	1.893858	0.012769
ENSG0000(	NT5E	2.353535	5.87925	1.32E-06
ENSG0000(	GGTA1P	2.351668	14.89574	1.27E-15
ENSG0000(	FSTL1	2.35166	6.397123	4.01E-07
ENSG0000(	LILRB2	2.349966	7.906371	1.24E-08
ENSG0000(	HLA-DMA	2.342923	16.07437	8.43E-17
ENSG0000(	TLL1	2.340652	4.217547	6.06E-05
ENSG0000(	TNXA	2.339056	2.159101	0.006933
ENSG0000(	MICB	2.338224	5.102187	7.90E-06
ENSG0000(	SNORA16A	2.338159	1.339753	0.045735
ENSG0000(	SNAI1	2.335756	1.634524	0.023199
ENSG0000(	RAB7B	2.332347	11.03045	9.32E-12
ENSG0000(	DCN	2.327119	5.969048	1.07E-06

ENSG0000(	COTL1	2.326114	10.54509	2.85E-11
ENSG0000(	DERL3	2.325511	5.250519	5.62E-06
ENSG0000(	ITGAL	2.321169	6.432921	3.69E-07
ENSG0000(	C5AR2	2.32022	3.11878	0.000761
ENSG0000(	AC007336.	2.31967	6.299593	5.02E-07
ENSG0000(	AL034397.:	2.318893	1.371543	0.042507
ENSG0000(	IFI27L2	2.318466	18.86777	1.36E-19
ENSG0000(	ODF3B	2.316518	9.454313	3.51E-10
ENSG0000(	CENPK	2.314485	1.633113	0.023275
ENSG0000(	RHEBL1	2.313176	2.61723	0.002414
ENSG0000(	AC015922.	2.311452	2.916548	0.001212
ENSG0000(	CD44-AS1	2.310929	6.167407	6.80E-07
ENSG0000(	RPS10P7	2.309941	3.405957	0.000393
ENSG0000(	THEMIS2	2.30973	8.280281	5.24E-09
ENSG0000(	C1RL	2.306826	11.96902	1.07E-12
ENSG0000(	SIGLEC10	2.305943	1.403638	0.039479
ENSG0000(	ITK	2.300782	4.211678	6.14E-05
ENSG0000(	C9orf66	2.299196	1.493871	0.032072
ENSG0000(	FAAP24	2.298908	2.188873	0.006473
ENSG0000(	GNAT2	2.296117	1.518182	0.030326
ENSG0000(	AC069444.	2.292506	1.399964	0.039814
ENSG0000(	FBXL16	2.291243	2.028572	0.009363
ENSG0000(	MYBPHL	2.289728	1.551708	0.028073
ENSG0000(	WAS	2.28828	8.982323	1.04E-09
ENSG0000(	MYBPH	2.288181	2.971547	0.001068
ENSG0000(	MFAP2	2.285183	3.828543	0.000148
ENSG0000(	LINC02397	2.284544	3.313829	0.000485
ENSG0000(	VCAN-AS1	2.28452	5.776868	1.67E-06
ENSG0000(	PINLYP	2.283235	2.159749	0.006922
ENSG0000(	FAM171A2	2.282272	4.010615	9.76E-05
ENSG0000(	ABI3BP	2.281607	8.193036	6.41E-09
ENSG0000(	AC011450.	2.281145	4.497836	3.18E-05
ENSG0000(	BBS12	2.280942	2.572903	0.002674
ENSG0000(	CD83	2.280428	6.558275	2.77E-07
ENSG0000(	BTN3A3	2.279222	18.71501	1.93E-19
ENSG0000(	IFIT2	2.27765	8.64631	2.26E-09
ENSG0000(	TNFRSF13C	2.277511	1.414978	0.038461
ENSG0000(	KIF19	2.277471	1.675934	0.02109
ENSG0000(	FP565260.:	2.277183	1.675465	0.021112
ENSG0000(	RPL23AP1	2.277076	5.516412	3.05E-06
ENSG0000(	ADGRG2	2.276931	1.626466	0.023634
ENSG0000(	ZEB2-AS1	2.276663	3.615429	0.000242
ENSG0000(	CHL1	2.275448	3.707243	0.000196
ENSG0000(	AC022150.	2.273637	1.515304	0.030528
ENSG0000(	BGN	2.272426	6.345483	4.51E-07
ENSG0000(	FGF9	2.272305	3.642333	0.000228
ENSG0000(	TACR1	2.270738	2.152548	0.007038

ENSG0000(	TUBA3FP	2.268484	1.941846	0.011433
ENSG0000(	RHBDL2	2.267896	1.484474	0.032774
ENSG0000(	EDA2R	2.266984	7.82391	1.50E-08
ENSG0000(	STAP2	2.266594	4.283922	5.20E-05
ENSG0000(	EPHB3	2.265227	4.524208	2.99E-05
ENSG0000(	PABPC5	2.263833	3.339567	0.000458
ENSG0000(	MNDA	2.262918	3.642978	0.000228
ENSG0000(	AC060766.	2.262059	3.827326	0.000149
ENSG0000(	C9	2.26033	19.02812	9.37E-20
ENSG0000(	MYO1G	2.259492	4.672638	2.13E-05
ENSG0000(	S1PR4	2.257698	1.639083	0.022957
ENSG0000(	LIF	2.256738	2.73963	0.001821
ENSG0000(	ALOX5	2.256413	11.12588	7.48E-12
ENSG0000(	NTM	2.253376	4.950119	1.12E-05
ENSG0000(	LPAR1	2.253185	6.137529	7.29E-07
ENSG0000(	PTPRCAP	2.252123	7.705553	1.97E-08
ENSG0000(	AL022318.4	2.251868	17.15441	7.01E-18
ENSG0000(	GALNT17	2.247925	8.965413	1.08E-09
ENSG0000(	GMFG	2.246312	8.919873	1.20E-09
ENSG0000(	KCNQ1	2.246028	5.962019	1.09E-06
ENSG0000(	LINC01138	2.245297	4.288789	5.14E-05
ENSG0000(	VGLL3	2.24519	8.503739	3.14E-09
ENSG0000(	LRRC4	2.243973	3.610279	0.000245
ENSG0000(	AC010260.	2.24267	2.093565	0.008062
ENSG0000(	SLC7A7	2.242635	8.538455	2.89E-09
ENSG0000(	AL021707.(	2.241673	1.350255	0.044642
ENSG0000(	TPPP3	2.241083	8.808495	1.55E-09
ENSG0000(	GMIP	2.23774	11.84808	1.42E-12
ENSG0000(	P2RX4	2.237644	9.959559	1.10E-10
ENSG0000(	SFRP4	2.237356	5.071531	8.48E-06
ENSG0000(	CNTN4	2.236868	3.442277	0.000361
ENSG0000(	AC078795.	2.236504	1.461918	0.034521
ENSG0000(	TMEM35A	2.235146	1.540548	0.028804
ENSG0000(	PTPN6	2.230985	10.6789	2.09E-11
ENSG0000(	TNFRSF11A	2.230338	2.735845	0.001837
ENSG0000(	ARHGAP8	2.229418	1.645693	0.02261
ENSG0000(	TRIM9	2.22865	7.344121	4.53E-08
ENSG0000(	CMKLR1	2.226671	17.3238	4.74E-18
ENSG0000(	APOBR	2.225492	4.822553	1.50E-05
ENSG0000(	ABCC3	2.224975	3.961213	0.000109
ENSG0000(	C3	2.224137	10.73342	1.85E-11
ENSG0000(	ST8SIA2	2.223786	4.546629	2.84E-05
ENSG0000(	GBP4	2.223277	10.29267	5.10E-11
ENSG0000(	AC010533.	2.222894	3.319622	0.000479
ENSG0000(	HIST1H4I	2.222883	5.756956	1.75E-06
ENSG0000(	FLG	2.222849	1.446386	0.035778
ENSG0000(	MMP2	2.221446	6.027546	9.39E-07

ENSG0000(	NCS1	2.220883	17.90725	1.24E-18
ENSG0000(	BAALC-AS1	2.217652	1.627396	0.023583
ENSG0000(	SNORA57	2.217057	1.397719	0.04002
ENSG0000(	CRIP1	2.216372	9.096824	8.00E-10
ENSG0000(	WDR17	2.216017	1.414732	0.038483
ENSG0000(	ZNF214	2.21422	1.817499	0.015223
ENSG0000(	GBP2	2.211897	12.37129	4.25E-13
ENSG0000(	AP000802.	2.208896	3.422591	0.000378
ENSG0000(	MC1R	2.205616	8.8599	1.38E-09
ENSG0000(	CXCL16	2.204627	13.2164	6.08E-14
ENSG0000(	FBLN7	2.204136	4.851592	1.41E-05
ENSG0000(	CHRNA1	2.2036	5.168279	6.79E-06
ENSG0000(	PCDH18	2.203343	7.516306	3.05E-08
ENSG0000(	APOBEC3F	2.202345	6.118838	7.61E-07
ENSG0000(	ANXA1	2.199984	9.572725	2.67E-10
ENSG0000(	FCGR2C	2.199576	7.640584	2.29E-08
ENSG0000(	JAZF1-AS1	2.199534	2.850237	0.001412
ENSG0000(	PDGFD	2.196267	5.434721	3.68E-06
ENSG0000(	GPR176	2.196263	4.121172	7.57E-05
ENSG0000(	CARD16	2.195499	5.442949	3.61E-06
ENSG0000(	HNMT	2.195003	10.92805	1.18E-11
ENSG0000(	AC022165.	2.194279	1.896206	0.0127
ENSG0000(	GLIPR2	2.192448	11.83281	1.47E-12
ENSG0000(	EMID1	2.191542	4.832443	1.47E-05
ENSG0000(	AC105942.	2.190297	4.650299	2.24E-05
ENSG0000(	MS4A4E	2.189029	4.058641	8.74E-05
ENSG0000(	EFEMP1	2.188627	7.885215	1.30E-08
ENSG0000(	BIRC3	2.188447	24.99385	1.01E-25
ENSG0000(	CACNA1E	2.187747	1.387798	0.040945
ENSG0000(	AC005779.	2.184441	1.848869	0.014162
ENSG0000(	DENND1C	2.182517	4.432443	3.69E-05
ENSG0000(	DOCK10	2.178813	10.82641	1.49E-11
ENSG0000(	ZNF503	2.176176	2.718733	0.001911
ENSG0000(	PTGES3P1	2.176172	8.520578	3.02E-09
ENSG0000(	CP	2.176168	10.06632	8.58E-11
ENSG0000(	HLA-DQA2	2.172397	2.154417	0.007008
ENSG0000(	MFAP5	2.171569	5.949963	1.12E-06
ENSG0000(	COL19A1	2.171022	1.349993	0.044669
ENSG0000(	AC005515.	2.167936	1.500122	0.031614
ENSG0000(	ID2-AS1	2.167656	1.50888	0.030983
ENSG0000(	JAK3	2.167635	4.56453	2.73E-05
ENSG0000(	HGF	2.164557	6.466302	3.42E-07
ENSG0000(	AC093162.	2.164232	1.865471	0.013631
ENSG0000(	HLA-DRB6	2.164221	6.630593	2.34E-07
ENSG0000(	PROCR	2.163456	4.060774	8.69E-05
ENSG0000(	AC106782.	2.162101	2.565253	0.002721
ENSG0000(	TBC1D30	2.160995	1.95764	0.011025

ENSG0000(	PNMA8A	2.160937	6.693565	2.03E-07
ENSG0000(	C7orf31	2.157651	3.123178	0.000753
ENSG0000(	CYBRD1	2.157116	5.968849	1.07E-06
ENSG0000(	LINC01139	2.156882	1.882456	0.013108
ENSG0000(	BTN3A1	2.156759	14.25505	5.56E-15
ENSG0000(	NLRC5	2.154051	10.47322	3.36E-11
ENSG0000(	AL356652.:	2.153312	3.589172	0.000258
ENSG0000(	AC019117.	2.150337	5.143073	7.19E-06
ENSG0000(	PI16	2.146975	4.915964	1.21E-05
ENSG0000(	AC092118.	2.146481	1.305854	0.049448
ENSG0000(	AL158151.:	2.145171	2.383396	0.004136
ENSG0000(	IRF1	2.143726	13.03729	9.18E-14
ENSG0000(	AC011558.	2.143485	3.448886	0.000356
ENSG0000(	KLRD1	2.142517	2.943158	0.00114
ENSG0000(	UNC93B1	2.141091	9.713694	1.93E-10
ENSG0000(	LBX2	2.141055	3.337967	0.000459
ENSG0000(	PSD4	2.139619	9.104343	7.86E-10
ENSG0000(	AL135926.:	2.139408	10.17364	6.70E-11
ENSG0000(	LST1	2.137695	4.818411	1.52E-05
ENSG0000(	AC063952.	2.134728	5.829895	1.48E-06
ENSG0000(	CORO2A	2.134563	3.986571	0.000103
ENSG0000(	PDE10A	2.132627	3.571014	0.000269
ENSG0000(	IFI35	2.132142	13.45364	3.52E-14
ENSG0000(	DAB2	2.131574	14.72231	1.90E-15
ENSG0000(	TPSAB1	2.130315	9.415994	3.84E-10
ENSG0000(	STXBP2	2.129917	6.721917	1.90E-07
ENSG0000(	NGFR	2.128824	7.462822	3.44E-08
ENSG0000(	KCNAB3	2.128636	1.621299	0.023917
ENSG0000(	ISG20	2.126984	6.393726	4.04E-07
ENSG0000(	AL359094.:	2.1263	4.372321	4.24E-05
ENSG0000(	AL928654.:	2.124439	8.720034	1.91E-09
ENSG0000(	MCUB	2.124221	10.79852	1.59E-11
ENSG0000(	JAML	2.123952	6.103897	7.87E-07
ENSG0000(	ROR2	2.120771	1.330183	0.046754
ENSG0000(	APOBEC3D	2.120009	6.917048	1.21E-07
ENSG0000(	AC109361.	2.118884	1.614842	0.024275
ENSG0000(	HTRA1	2.118391	5.721878	1.90E-06
ENSG0000(	GALNT6	2.117995	4.14212	7.21E-05
ENSG0000(	HSPA7	2.117002	6.19498	6.38E-07
ENSG0000(	CFH	2.116948	14.46442	3.43E-15
ENSG0000(	AF117829.	2.114097	3.312032	0.000487
ENSG0000(	RAB32	2.113853	7.820981	1.51E-08
ENSG0000(	ULK4	2.113491	1.508312	0.031023
ENSG0000(	BTN2A2	2.112833	15.36789	4.29E-16
ENSG0000(	AC064836.	2.111739	1.323178	0.047514
ENSG0000(	SLC17A9	2.110368	3.489284	0.000324
ENSG0000(	ADAMTS16	2.106623	1.955983	0.011067

ENSG0000(	EMP3	2.106463	8.372587	4.24E-09
ENSG0000(	DPT	2.106403	9.940103	1.15E-10
ENSG0000(	LRP1-AS	2.103829	3.685761	0.000206
ENSG0000(	SPN	2.103566	5.45358	3.52E-06
ENSG0000(	PLTP	2.10227	10.02015	9.55E-11
ENSG0000(	HLA-DRB5	2.100908	6.532975	2.93E-07
ENSG0000(	ZNF296	2.10078	1.761706	0.01731
ENSG0000(	RASGRP1	2.098473	4.026041	9.42E-05
ENSG0000(	CPVL	2.094817	12.5343	2.92E-13
ENSG0000(	CEP295NL	2.094538	5.185715	6.52E-06
ENSG0000(	HUNK	2.091924	2.60275	0.002496
ENSG0000(	GPNMB	2.090459	5.499001	3.17E-06
ENSG0000(	NPNT	2.087703	8.876285	1.33E-09
ENSG0000(	KCNN4	2.087166	4.683674	2.07E-05
ENSG0000(	ESPL1	2.086921	1.445826	0.035824
ENSG0000(	PRKCB	2.085867	4.547563	2.83E-05
ENSG0000(	SMIM5	2.085791	1.318102	0.048073
ENSG0000(	S100A11	2.084968	11.40392	3.95E-12
ENSG0000(	NTRK2	2.084325	4.677583	2.10E-05
ENSG0000(	KCNT2	2.083555	2.196026	0.006368
ENSG0000(	AC244517.	2.082306	1.72486	0.018843
ENSG0000(	ABRACL	2.080921	4.982186	1.04E-05
ENSG0000(	RAC3	2.080385	1.309439	0.049041
ENSG0000(	METRNL	2.074982	6.32857	4.69E-07
ENSG0000(	HRH1	2.074467	8.236614	5.80E-09
ENSG0000(	EGR3	2.073321	3.251958	0.00056
ENSG0000(	S100A4	2.073117	9.458713	3.48E-10
ENSG0000(	HRASLS5	2.072643	1.676103	0.021081
ENSG0000(	PTPRE	2.068581	10.93753	1.15E-11
ENSG0000(	SLC9A9	2.068083	12.77833	1.67E-13
ENSG0000(	AOX1	2.067912	4.727686	1.87E-05
ENSG0000(	AC007750.	2.067287	7.306517	4.94E-08
ENSG0000(	AC103858.	2.064514	4.173096	6.71E-05
ENSG0000(	PCOLCE	2.063778	9.783995	1.64E-10
ENSG0000(	GSN-AS1	2.063593	19.38539	4.12E-20
ENSG0000(	CYP26B1	2.063127	7.128531	7.44E-08
ENSG0000(	PTGIR	2.061596	3.99478	0.000101
ENSG0000(	DEF6	2.061268	7.717827	1.92E-08
ENSG0000(	RASSF2	2.05926	13.83691	1.46E-14
ENSG0000(	C9orf92	2.056588	1.319772	0.047888
ENSG0000(	AC022966.	2.05653	4.869018	1.35E-05
ENSG0000(	AC062029.	2.055604	2.422627	0.003779
ENSG0000(	WIPF1	2.053745	11.16263	6.88E-12
ENSG0000(	CD248	2.053565	5.254492	5.57E-06
ENSG0000(	AC010894.	2.051978	7.356411	4.40E-08
ENSG0000(	TMC8	2.05151	5.270618	5.36E-06
ENSG0000(	HIST1H2BK	2.046217	7.340138	4.57E-08

ENSG0000(	AC015922.	2.045893	3.56106	0.000275
ENSG0000(	ARSD-AS1	2.045348	3.58376	0.000261
ENSG0000(	NRCAM	2.043632	3.668003	0.000215
ENSG0000(	PCOLCE-AS	2.04357	10.55243	2.80E-11
ENSG0000(	P2RY14	2.043073	9.50917	3.10E-10
ENSG0000(	TMEM169	2.041637	1.676119	0.021081
ENSG0000(	TIMP2	2.040942	4.724203	1.89E-05
ENSG0000(	MIR4709	2.040251	7.610473	2.45E-08
ENSG0000(	COLEC12	2.039717	10.99841	1.00E-11
ENSG0000(	INE2	2.037161	1.906453	0.012404
ENSG0000(	CETP	2.034074	1.376309	0.042043
ENSG0000(	IL34	2.033235	8.515572	3.05E-09
ENSG0000(	TAF1A-AS1	2.032941	1.455525	0.035033
ENSG0000(	FAS	2.032024	26.90856	1.23E-27
ENSG0000(	FERMT3	2.031803	11.36334	4.33E-12
ENSG0000(	VENTX	2.03128	3.807478	0.000156
ENSG0000(	LOXL1	2.028753	8.167057	6.81E-09
ENSG0000(	PKD1L3	2.028403	1.751089	0.017738
ENSG0000(	ADRA2A	2.027752	4.635751	2.31E-05
ENSG0000(	CASP1	2.02393	10.84991	1.41E-11
ENSG0000(	SLC8A1-AS	2.022399	5.98984	1.02E-06
ENSG0000(	KAZALD1	2.022293	11.21673	6.07E-12
ENSG0000(	ELL3	2.021282	10.5622	2.74E-11
ENSG0000(	SERPINA3	2.021129	2.667089	0.002152
ENSG0000(	APOL6	2.020321	13.00071	9.98E-14
ENSG0000(	LY75	2.019653	4.445948	3.58E-05
ENSG0000(	THEM5	2.019601	1.550675	0.02814
ENSG0000(	AP003392.	2.01766	2.338298	0.004589
ENSG0000(	SSC5D	2.016885	5.418154	3.82E-06
ENSG0000(	KCTD17	2.014888	9.772544	1.69E-10
ENSG0000(	MFAP4	2.013369	5.599862	2.51E-06
ENSG0000(	CLEC4GP1	2.01076	2.014451	0.009673
ENSG0000(	PCDHB11	2.010009	2.218719	0.006043
ENSG0000(	TMPPE	2.0086	2.485634	0.003269
ENSG0000(	AC099521.	2.006053	1.62066	0.023952
ENSG0000(	PLCB2	2.004654	8.057947	8.75E-09
ENSG0000(	CLSTN3	2.003444	10.87593	1.33E-11
ENSG0000(	CA13	2.001628	1.900914	0.012563
ENSG0000(	BIN2	2.001005	3.669052	0.000214
ENSG0000(	AC008105.	2.001002	11.48177	3.30E-12
ENSG0000(	MUSK	2.000174	3.355832	0.000441
ENSG0000(	SLC22A18	2.000129	4.947681	1.13E-05
ENSG0000(	ITGAX	1.998109	4.858401	1.39E-05
ENSG0000(	RBM47	1.996781	5.556056	2.78E-06
ENSG0000(	IFITM10	1.996007	8.397467	4.00E-09
ENSG0000(	FAM107B	1.995689	8.032197	9.29E-09
ENSG0000(	KCNMB1	1.995637	2.794655	0.001605

ENSG0000(	AC018653.	1.994915	3.027732	0.000938
ENSG0000(	DMRT3	1.994329	1.421777	0.037864
ENSG0000(	C3orf14	1.990397	4.089495	8.14E-05
ENSG0000(	ALG1L9P	1.989359	1.317824	0.048103
ENSG0000(	CNN2	1.989342	9.052239	8.87E-10
ENSG0000(	APOE	1.98754	7.853236	1.40E-08
ENSG0000(	AL049829.:	1.987138	1.858964	0.013837
ENSG0000(	AL159163.:	1.986617	11.16717	6.80E-12
ENSG0000(	C14orf132	1.986022	10.58749	2.59E-11
ENSG0000(	OLFML3	1.985785	8.31296	4.86E-09
ENSG0000(	1-Mar	1.985068	7.257178	5.53E-08
ENSG0000(	CASP4	1.985037	13.45312	3.52E-14
ENSG0000(	SYT17	1.983738	1.835425	0.014607
ENSG0000(	C11orf21	1.981885	2.373044	0.004236
ENSG0000(	AC012435.	1.981505	2.907656	0.001237
ENSG0000(	IFI16	1.978836	8.789297	1.62E-09
ENSG0000(	RAB31	1.977734	12.75599	1.75E-13
ENSG0000(	AL022323.:	1.976789	2.180458	0.0066
ENSG0000(	ARL4D	1.976138	10.67949	2.09E-11
ENSG0000(	NUF2	1.975366	1.340937	0.04561
ENSG0000(	GPR153	1.972743	14.62174	2.39E-15
ENSG0000(	HSD3B7	1.972313	11.81524	1.53E-12
ENSG0000(	IRF9	1.972304	16.15361	7.02E-17
ENSG0000(	PRR5	1.972211	3.914111	0.000122
ENSG0000(	FBN1	1.97046	5.361445	4.35E-06
ENSG0000(	OAF	1.969626	5.189851	6.46E-06
ENSG0000(	AC012615.	1.968982	3.372295	0.000424
ENSG0000(	NLGN4Y	1.968811	4.050715	8.90E-05
ENSG0000(	NECAB1	1.968431	1.40132	0.03969
ENSG0000(	EFHD2	1.968138	10.3143	4.85E-11
ENSG0000(	TPSB2	1.966759	3.490533	0.000323
ENSG0000(	CGAS	1.966333	3.01242	0.000972
ENSG0000(	KCNH2	1.966097	2.100295	0.007938
ENSG0000(	AC025048.	1.961177	2.01063	0.009758
ENSG0000(	PARP8	1.959107	7.358775	4.38E-08
ENSG0000(	VCAN	1.957286	5.349361	4.47E-06
ENSG0000(	CMTM3	1.95585	10.6434	2.27E-11
ENSG0000(	SERPING1	1.954375	6.81472	1.53E-07
ENSG0000(	MMP16	1.954262	2.818922	0.001517
ENSG0000(	ARPC1B	1.952612	10.86805	1.36E-11
ENSG0000(	DPYD-AS1	1.952364	2.161198	0.006899
ENSG0000(	CARMIL1	1.952069	7.894826	1.27E-08
ENSG0000(	TBX19	1.951853	2.664622	0.002165
ENSG0000(	SNX7	1.950529	13.09772	7.99E-14
ENSG0000(	ARAP2	1.950336	9.872524	1.34E-10
ENSG0000(	HSH2D	1.946239	2.016061	0.009637
ENSG0000(	PCK2	1.946226	9.155741	6.99E-10

ENSG0000(	MYD88	1.945353	8.520723	3.01E-09
ENSG0000(	C1QTNF3	1.945231	3.756421	0.000175
ENSG0000(	CR559946.	1.944752	1.549471	0.028218
ENSG0000(	LYN	1.941587	9.412878	3.86E-10
ENSG0000(	PARP14	1.940114	8.815963	1.53E-09
ENSG0000(	AC135048.	1.939736	11.74195	1.81E-12
ENSG0000(	ZBTB7C	1.939461	6.964033	1.09E-07
ENSG0000(	FAP	1.938965	8.187846	6.49E-09
ENSG0000(	ARHGAP9	1.937826	6.493967	3.21E-07
ENSG0000(	VIM-AS1	1.936907	7.852294	1.41E-08
ENSG0000(	GPX8	1.936369	6.566044	2.72E-07
ENSG0000(	GCH1	1.934961	6.13962	7.25E-07
ENSG0000(	GRAP2	1.934498	2.227275	0.005925
ENSG0000(	ZNF321P	1.934082	2.250083	0.005622
ENSG0000(	SLCO2B1	1.931588	16.74183	1.81E-17
ENSG0000(	AL160272.:	1.931545	2.945581	0.001133
ENSG0000(	CYBA	1.930924	11.92919	1.18E-12
ENSG0000(	CNN3	1.928668	7.648193	2.25E-08
ENSG0000(	SOCS3	1.928633	5.879186	1.32E-06
ENSG0000(	AP000919.	1.926384	1.574184	0.026657
ENSG0000(	MYO1F	1.92453	6.277172	5.28E-07
ENSG0000(	TMED3	1.92297	7.372741	4.24E-08
ENSG0000(	DNM1	1.92219	4.184794	6.53E-05
ENSG0000(	LINC02381	1.921902	3.334875	0.000463
ENSG0000(	CCDC88B	1.921602	6.590511	2.57E-07
ENSG0000(	PTGFRN	1.921292	9.260047	5.49E-10
ENSG0000(	NFKBID	1.92029	2.881796	0.001313
ENSG0000(	GPRIN1	1.919619	2.236635	0.005799
ENSG0000(	AC119403.	1.9172	1.402003	0.039628
ENSG0000(	AC092821.	1.916828	3.204751	0.000624
ENSG0000(	ACTC1	1.916102	2.268846	0.005385
ENSG0000(	PIM2	1.915619	7.112394	7.72E-08
ENSG0000(	TLR3	1.915261	7.452075	3.53E-08
ENSG0000(	SCN9A	1.913063	4.928372	1.18E-05
ENSG0000(	CYTOR	1.912983	6.964823	1.08E-07
ENSG0000(	AL158151.:	1.911392	8.40694	3.92E-09
ENSG0000(	DOCK11	1.91133	6.391507	4.06E-07
ENSG0000(	LY75-CD30	1.909617	9.688119	2.05E-10
ENSG0000(	AC105285.	1.909337	2.83862	0.00145
ENSG0000(	C5	1.909043	2.236933	0.005795
ENSG0000(	AL773545.:	1.908668	1.760861	0.017344
ENSG0000(	SLC2A10	1.908052	5.723795	1.89E-06
ENSG0000(	TRANK1	1.906595	10.14385	7.18E-11
ENSG0000(	NABP1	1.906154	9.621172	2.39E-10
ENSG0000(	ARNTL2	1.904534	3.273708	0.000532
ENSG0000(	SAMD9	1.90392	8.032575	9.28E-09
ENSG0000(	GALNT16	1.902758	4.64642	2.26E-05

ENSG0000(	EPS8L2	1.901763	7.852777	1.40E-08
ENSG0000(	AL133415.:	1.900709	7.092026	8.09E-08
ENSG0000(	GFPT2	1.899364	3.742791	0.000181
ENSG0000(	CLIC1	1.898903	11.06525	8.60E-12
ENSG0000(	DUSP5	1.898157	5.761384	1.73E-06
ENSG0000(	VIM	1.898056	7.213309	6.12E-08
ENSG0000(	KLHL6	1.89611	7.243647	5.71E-08
ENSG0000(	PAQR8	1.895287	9.741396	1.81E-10
ENSG0000(	AC106739.	1.894857	1.650367	0.022368
ENSG0000(	DDAH2	1.894842	8.362169	4.34E-09
ENSG0000(	PLPPR4	1.894726	3.930234	0.000117
ENSG0000(	STAMBPL1	1.89466	7.490255	3.23E-08
ENSG0000(	LSAMP	1.892397	1.887713	0.012951
ENSG0000(	LRMP	1.892119	3.958272	0.00011
ENSG0000(	PLAC9	1.889977	7.315675	4.83E-08
ENSG0000(	FMNL1	1.889601	9.625753	2.37E-10
ENSG0000(	TMSB4X	1.88928	10.71247	1.94E-11
ENSG0000(	OLFML1	1.888452	6.534551	2.92E-07
ENSG0000(	CLIP3	1.887616	11.24448	5.70E-12
ENSG0000(	S1PR2	1.887459	4.147248	7.12E-05
ENSG0000(	SAMD5	1.886926	2.664622	0.002165
ENSG0000(	NFKBIE	1.886483	11.23997	5.75E-12
ENSG0000(	ELN-AS1	1.885553	5.066221	8.59E-06
ENSG0000(	C6orf132	1.885263	1.671804	0.021291
ENSG0000(	SERPINB8	1.884892	7.927202	1.18E-08
ENSG0000(	CKB	1.884359	8.001005	9.98E-09
ENSG0000(	ACTRT3	1.881773	1.609753	0.024561
ENSG0000(	ALDH3B1	1.881337	12.53857	2.89E-13
ENSG0000(	SLC1A2	1.881279	2.439365	0.003636
ENSG0000(	PTGS1	1.880734	5.95538	1.11E-06
ENSG0000(	LINC01116	1.880512	2.623219	0.002381
ENSG0000(	CCDC200	1.880178	5.722939	1.89E-06
ENSG0000(	AC013451.	1.879279	2.677017	0.002104
ENSG0000(	CADM3	1.879151	4.967473	1.08E-05
ENSG0000(	LGALS9	1.879011	6.611152	2.45E-07
ENSG0000(	SIK1B	1.877412	1.719165	0.019091
ENSG0000(	VWA5A	1.87525	14.39282	4.05E-15
ENSG0000(	MEDAG	1.874841	5.919054	1.20E-06
ENSG0000(	ASPHD2	1.873446	2.110321	0.007757
ENSG0000(	CDT1	1.871712	1.403506	0.039491
ENSG0000(	SLC15A3	1.870809	13.5162	3.05E-14
ENSG0000(	ID2	1.86929	10.98312	1.04E-11
ENSG0000(	IL18R1	1.868856	3.867937	0.000136
ENSG0000(	TRIM46	1.86602	1.409501	0.038949
ENSG0000(	MYOF	1.865865	10.26919	5.38E-11
ENSG0000(	TTC39C	1.861961	4.591475	2.56E-05
ENSG0000(	PLEKHO2	1.86167	10.90481	1.25E-11

ENSG0000(	DBH-AS1	1.861647	2.620361	0.002397
ENSG0000(	PTPRJ	1.858842	9.644565	2.27E-10
ENSG0000(	AC106782.	1.858444	2.191865	0.006429
ENSG0000(	NAIP	1.857326	1.746212	0.017939
ENSG0000(	SH3BGRL	1.856759	9.474194	3.36E-10
ENSG0000(	C10orf25	1.854669	2.120103	0.007584
ENSG0000(	AKNA	1.854439	9.321072	4.77E-10
ENSG0000(	AC002116.	1.85405	8.708619	1.96E-09
ENSG0000(	GEM	1.854038	6.302771	4.98E-07
ENSG0000(	MAN1A1	1.853898	11.41681	3.83E-12
ENSG0000(	TCN2	1.85236	9.154395	7.01E-10
ENSG0000(	NHLH2	1.852278	2.23443	0.005829
ENSG0000(	COL5A2	1.851831	8.76112	1.73E-09
ENSG0000(	PRR5-ARH(	1.851564	4.346854	4.50E-05
ENSG0000(	DUXAP10	1.850837	1.828599	0.014839
ENSG0000(	RPP25	1.850717	5.989594	1.02E-06
ENSG0000(	CUZD1	1.850002	1.685322	0.020639
ENSG0000(	RAB20	1.849962	8.902546	1.25E-09
ENSG0000(	MMP14	1.848774	6.144996	7.16E-07
ENSG0000(	STMN3	1.840541	11.39895	3.99E-12
ENSG0000(	DRAM1	1.839183	7.408801	3.90E-08
ENSG0000(	HRH2	1.837702	1.676877	0.021044
ENSG0000(	SULF1	1.835018	5.795834	1.60E-06
ENSG0000(	AL669918.:	1.834342	10.18653	6.51E-11
ENSG0000(	PHGDH	1.833781	4.111205	7.74E-05
ENSG0000(	CDON	1.833112	4.961822	1.09E-05
ENSG0000(	C1QTNF7	1.833035	1.729177	0.018656
ENSG0000(	AC011731.	1.83203	1.709524	0.01952
ENSG0000(	DOCK8	1.83157	9.030056	9.33E-10
ENSG0000(	SOX11	1.831009	2.766232	0.001713
ENSG0000(	MCTP2	1.82993	1.845903	0.014259
ENSG0000(	NDC80	1.829901	1.479145	0.033178
ENSG0000(	FAM20A	1.82856	9.205403	6.23E-10
ENSG0000(	DSCAML1	1.826932	2.865779	0.001362
ENSG0000(	CADM3-AS	1.822929	4.919007	1.21E-05
ENSG0000(	SPATA2L	1.821844	3.832288	0.000147
ENSG0000(	TAP2	1.821613	10.78479	1.64E-11
ENSG0000(	PLP1	1.820538	3.6981	0.0002
ENSG0000(	SVEP1	1.819813	5.435254	3.67E-06
ENSG0000(	LOXL1-AS1	1.819757	7.282488	5.22E-08
ENSG0000(	HCG4B	1.819237	2.626923	0.002361
ENSG0000(	ELN	1.818606	5.502786	3.14E-06
ENSG0000(	AQP3	1.818452	6.784418	1.64E-07
ENSG0000(	SRPX	1.817532	5.785637	1.64E-06
ENSG0000(	MIR7847	1.816239	13.39276	4.05E-14
ENSG0000(	TMEM107	1.815113	7.506853	3.11E-08
ENSG0000(	ACKR1	1.814397	4.193235	6.41E-05

ENSG0000(	RUNX1	1.814228	12.10091	7.93E-13
ENSG0000(	ARPC5	1.812173	10.85397	1.40E-11
ENSG0000(	ITGA11	1.811843	4.827901	1.49E-05
ENSG0000(	TMEM106/	1.811326	7.276635	5.29E-08
ENSG0000(	ANXA2	1.810721	11.86925	1.35E-12
ENSG0000(	TMEM136	1.809837	7.817341	1.52E-08
ENSG0000(	PARP9	1.809505	10.90538	1.24E-11
ENSG0000(	PDCD1LG2	1.808208	4.60477	2.48E-05
ENSG0000(	AC009511.	1.808122	1.892466	0.01281
ENSG0000(	AC008105.	1.807195	6.549028	2.82E-07
ENSG0000(	SYT15	1.803369	1.865488	0.013631
ENSG0000(	JUNB	1.802991	7.448649	3.56E-08
ENSG0000(	PCDHB7	1.801758	1.652066	0.022281
ENSG0000(	AC004846.	1.801718	3.449491	0.000355
ENSG0000(	CDK18	1.801399	6.388421	4.09E-07
ENSG0000(	CAMK1D	1.799097	6.711011	1.95E-07
ENSG0000(	SOD3	1.798792	5.023724	9.47E-06
ENSG0000(	S100A10	1.796138	8.587023	2.59E-09
ENSG0000(	FAM102B	1.795337	8.943435	1.14E-09
ENSG0000(	EMP1	1.794512	5.585245	2.60E-06
ENSG0000(	CLEC3B	1.793259	5.381304	4.16E-06
ENSG0000(	IFITM1	1.793247	5.54025	2.88E-06
ENSG0000(	AC010186.	1.792833	2.773256	0.001686
ENSG0000(	AL139274.:	1.79277	2.25211	0.005596
ENSG0000(	RGS17	1.788502	1.654057	0.022179
ENSG0000(	TNFRSF21	1.788059	11.1795	6.61E-12
ENSG0000(	TMEM98	1.78479	5.448496	3.56E-06
ENSG0000(	RCN3	1.784381	5.583725	2.61E-06
ENSG0000(	SPON2	1.78298	6.644227	2.27E-07
ENSG0000(	CDKN1A	1.782829	4.656467	2.21E-05
ENSG0000(	ST8SIA1	1.781874	3.632072	0.000233
ENSG0000(	PAK3	1.781002	2.315628	0.004835
ENSG0000(	CSF1	1.780966	11.38043	4.16E-12
ENSG0000(	AHR	1.779596	10.59089	2.57E-11
ENSG0000(	AC009163.:	1.779263	1.371564	0.042505
ENSG0000(	DKK2	1.778271	3.433955	0.000368
ENSG0000(	CLMP	1.776671	5.083945	8.24E-06
ENSG0000(	DOK1	1.776586	6.54525	2.85E-07
ENSG0000(	PHLDA2	1.776498	2.317014	0.004819
ENSG0000(	PLSCR4	1.776409	5.136845	7.30E-06
ENSG0000(	CYP1B1	1.775973	6.459842	3.47E-07
ENSG0000(	MYO15A	1.774929	2.07588	0.008397
ENSG0000(	SCO2	1.774894	7.301904	4.99E-08
ENSG0000(	SHOX2	1.774257	3.393399	0.000404
ENSG0000(	HEPH	1.773717	4.49696	3.18E-05
ENSG0000(	DAPK1	1.773691	6.871696	1.34E-07
ENSG0000(	GPR68	1.770765	9.966577	1.08E-10

ENSG0000(	BISPR	1.770652	3.637565	0.00023
ENSG0000(	AL139241.:	1.770058	4.768403	1.70E-05
ENSG0000(	COL6A6	1.769783	5.090974	8.11E-06
ENSG0000(	SRGN	1.769095	7.794418	1.61E-08
ENSG0000(	LRRCC1	1.769046	4.04606	8.99E-05
ENSG0000(	CCND2	1.768283	5.232465	5.86E-06
ENSG0000(	AC022413.	1.768181	1.714231	0.019309
ENSG0000(	TBC1D10C	1.768069	5.861218	1.38E-06
ENSG0000(	MMRN1	1.765598	1.475072	0.033491
ENSG0000(	PCED1B	1.764393	5.599644	2.51E-06
ENSG0000(	FBXO27	1.761188	2.448051	0.003564
ENSG0000(	FKBP10	1.76086	5.905634	1.24E-06
ENSG0000(	TNFAIP2	1.760615	8.651456	2.23E-09
ENSG0000(	A1BG-AS1	1.760428	4.317768	4.81E-05
ENSG0000(	RNASE4	1.759565	4.508307	3.10E-05
ENSG0000(	DNAAF4	1.758916	1.664962	0.021629
ENSG0000(	HERC5	1.758676	3.467919	0.00034
ENSG0000(	PNMA2	1.75862	1.923364	0.01193
ENSG0000(	TRIB3	1.758551	3.251941	0.00056
ENSG0000(	ATP8B2	1.758454	5.779849	1.66E-06
ENSG0000(	ADD3	1.757664	6.745494	1.80E-07
ENSG0000(	PLAUR	1.757361	6.856808	1.39E-07
ENSG0000(	ADD3-AS1	1.756902	3.594746	0.000254
ENSG0000(	AL163636.:	1.756229	4.504265	3.13E-05
ENSG0000(	FBXO41	1.754665	3.510832	0.000308
ENSG0000(	AC117503.	1.754005	2.249986	0.005624
ENSG0000(	AC007620.	1.753938	7.759078	1.74E-08
ENSG0000(	CMAHP	1.753365	10.90743	1.24E-11
ENSG0000(	BASP1	1.753344	5.041774	9.08E-06
ENSG0000(	NNMT	1.752282	4.029712	9.34E-05
ENSG0000(	CDH11	1.752231	5.044123	9.03E-06
ENSG0000(	SDC1	1.750729	3.607266	0.000247
ENSG0000(	NLRP3	1.750317	2.920159	0.001202
ENSG0000(	TMSB10	1.748884	11.10799	7.80E-12
ENSG0000(	CBR3	1.748592	5.320979	4.78E-06
ENSG0000(	TCAF2	1.748519	2.988135	0.001028
ENSG0000(	LVRN	1.747605	2.448326	0.003562
ENSG0000(	PITPNM1	1.747375	10.92881	1.18E-11
ENSG0000(	CTSO	1.746856	10.23889	5.77E-11
ENSG0000(	ZNF582-AS	1.745735	1.824891	0.014966
ENSG0000(	TESPA1	1.743976	2.057535	0.008759
ENSG0000(	SELP	1.743748	4.090457	8.12E-05
ENSG0000(	AC069368.	1.743649	11.34691	4.50E-12
ENSG0000(	NCAM1	1.740519	7.420228	3.80E-08
ENSG0000(	DOK6	1.739239	1.302379	0.049845
ENSG0000(	ROBO3	1.737948	6.161343	6.90E-07
ENSG0000(	TIAM1	1.735299	6.57862	2.64E-07

ENSG0000(	ERBB3	1.734493	2.911113	0.001227
ENSG0000(	ADCY7	1.733886	12.90304	1.25E-13
ENSG0000(	PHETA2	1.733736	5.92941	1.18E-06
ENSG0000(	TMEM173	1.733192	10.83492	1.46E-11
ENSG0000(	UBXN10	1.732974	2.389514	0.004078
ENSG0000(	PRTFDC1	1.732942	5.214376	6.10E-06
ENSG0000(	TP53I3	1.732394	10.05832	8.74E-11
ENSG0000(	TRIM21	1.731731	14.74874	1.78E-15
ENSG0000(	MMP11	1.731727	5.583116	2.61E-06
ENSG0000(	AC002472.	1.73088	2.50234	0.003145
ENSG0000(	RNASET2	1.729747	9.897963	1.26E-10
ENSG0000(	L1CAM	1.729079	6.57862	2.64E-07
ENSG0000(	FBXL13	1.728036	2.648248	0.002248
ENSG0000(	RARRES2	1.726111	4.421983	3.78E-05
ENSG0000(	AC080038.	1.725374	6.694127	2.02E-07
ENSG0000(	AC010319.	1.725271	1.815501	0.015293
ENSG0000(	AC007362.	1.723909	1.394693	0.0403
ENSG0000(	CXCL14	1.723457	6.850872	1.41E-07
ENSG0000(	IFIH1	1.723019	6.845613	1.43E-07
ENSG0000(	Z99774.1	1.722977	1.54839	0.028289
ENSG0000(	SLC8A1	1.722726	15.26229	5.47E-16
ENSG0000(	AC104758.	1.7225	1.700812	0.019915
ENSG0000(	TLR4	1.721969	8.760632	1.74E-09
ENSG0000(	LINC01140	1.721964	6.299593	5.02E-07
ENSG0000(	PRSS36	1.71995	6.775947	1.68E-07
ENSG0000(	TXNDC5	1.719576	9.219986	6.03E-10
ENSG0000(	PRKAG2-AS	1.719542	1.399964	0.039814
ENSG0000(	LINC00623	1.718502	3.39729	0.000401
ENSG0000(	RNF212	1.717968	2.415072	0.003845
ENSG0000(	TRIM14	1.717647	8.848229	1.42E-09
ENSG0000(	CMTM7	1.717286	7.768067	1.71E-08
ENSG0000(	ELAVL2	1.717256	1.797051	0.015957
ENSG0000(	PRDM1	1.717041	7.083615	8.25E-08
ENSG0000(	AC244100.	1.71694	4.295273	5.07E-05
ENSG0000(	HHEX	1.716651	6.734128	1.84E-07
ENSG0000(	LGMN	1.715829	15.15009	7.08E-16
ENSG0000(	MARVELD1	1.71574	5.766723	1.71E-06
ENSG0000(	MR1	1.713485	10.52129	3.01E-11
ENSG0000(	U52112.1	1.712799	7.474212	3.36E-08
ENSG0000(	AP000943.	1.712288	2.405088	0.003935
ENSG0000(	C10orf142	1.711714	1.932543	0.01168
ENSG0000(	LBX2-AS1	1.710702	5.166662	6.81E-06
ENSG0000(	PLXNB2	1.710648	12.07886	8.34E-13
ENSG0000(	SARDH	1.710422	10.93753	1.15E-11
ENSG0000(	NRROS	1.70771	7.260782	5.49E-08
ENSG0000(	TRIM22	1.707212	8.80399	1.57E-09
ENSG0000(	HS3ST1	1.705887	1.310455	0.048927

ENSG0000(	NRSN2	1.705318	8.522189	3.00E-09
ENSG0000(	AL137026.:	1.704945	1.633474	0.023256
ENSG0000(	PPP1R18	1.704375	13.18783	6.49E-14
ENSG0000(	IER5L	1.704166	8.347925	4.49E-09
ENSG0000(	CLIC2	1.703935	8.383316	4.14E-09
ENSG0000(	C1RL-AS1	1.703679	5.83734	1.45E-06
ENSG0000(	PIK3CD	1.702932	7.850968	1.41E-08
ENSG0000(	AL445183.:	1.700674	4.839383	1.45E-05
ENSG0000(	SCARA5	1.700455	4.615571	2.42E-05
ENSG0000(	PCDHB9	1.700245	1.409312	0.038966
ENSG0000(	FRMD4B	1.700179	7.887852	1.29E-08
ENSG0000(	FZD10	1.697225	3.856938	0.000139
ENSG0000(	CFD	1.696917	5.139839	7.25E-06
ENSG0000(	AL139260.:	1.696795	2.765803	0.001715
ENSG0000(	GIMAP2	1.696537	8.698402	2.00E-09
ENSG0000(	AC026316.	1.69646	1.455988	0.034995
ENSG0000(	CERCAM	1.696264	5.585405	2.60E-06
ENSG0000(	STK17B	1.693096	10.91535	1.22E-11
ENSG0000(	PLCXD3	1.692513	1.381536	0.04154
ENSG0000(	IFIT5	1.691965	11.30092	5.00E-12
ENSG0000(	MXRA8	1.691737	5.77928	1.66E-06
ENSG0000(	ZNF525	1.691511	3.381699	0.000415
ENSG0000(	TBX18	1.690972	4.378292	4.19E-05
ENSG0000(	SLC47A2	1.690814	2.412198	0.003871
ENSG0000(	AC017002.	1.690471	5.471159	3.38E-06
ENSG0000(	IL33	1.690338	6.210004	6.17E-07
ENSG0000(	AC008734.	1.688499	1.337569	0.045965
ENSG0000(	TRPV2	1.688198	8.992159	1.02E-09
ENSG0000(	HLA-V	1.688191	1.363696	0.043282
ENSG0000(	PDLIM4	1.687571	8.188532	6.48E-09
ENSG0000(	TRIM34	1.686807	5.738755	1.82E-06
ENSG0000(	MTHFD1L	1.686165	7.473959	3.36E-08
ENSG0000(	RGS19	1.686109	4.583671	2.61E-05
ENSG0000(	ZNF816-ZN	1.685998	2.062862	0.008652
ENSG0000(	SLC49A3	1.684965	14.5394	2.89E-15
ENSG0000(	NPL	1.684201	7.979858	1.05E-08
ENSG0000(	CCL15-CCL:	1.683309	4.144978	7.16E-05
ENSG0000(	GAL3ST4	1.683107	4.678675	2.10E-05
ENSG0000(	AJUBA	1.682734	5.936571	1.16E-06
ENSG0000(	OLFML2A	1.682547	7.873338	1.34E-08
ENSG0000(	TRAF3IP3	1.682166	5.215534	6.09E-06
ENSG0000(	MIR6821	1.681201	2.711703	0.001942
ENSG0000(	CEP19	1.680977	2.511333	0.003081
ENSG0000(	IL7	1.679187	5.234462	5.83E-06
ENSG0000(	TRPM2	1.679105	5.539108	2.89E-06
ENSG0000(	ZNF382	1.678769	4.470544	3.38E-05
ENSG0000(	CD302	1.678523	7.326595	4.71E-08

ENSG0000(	ZNF486	1.677546	2.866192	0.001361
ENSG0000(	SPON1	1.675178	1.589067	0.025759
ENSG0000(	HLA-E	1.675105	14.75046	1.78E-15
ENSG0000(	ISLR	1.6746	5.690157	2.04E-06
ENSG0000(	TSHZ2	1.674349	5.5922	2.56E-06
ENSG0000(	SPTLC3	1.674165	5.445326	3.59E-06
ENSG0000(	LILRB3	1.671263	4.315314	4.84E-05
ENSG0000(	MS4A2	1.670959	2.48134	0.003301
ENSG0000(	AL133346.:	1.670883	3.273792	0.000532
ENSG0000(	UAP1L1	1.670761	9.557363	2.77E-10
ENSG0000(	HLA-F-AS1	1.670594	9.367982	4.29E-10
ENSG0000(	AL163636.:	1.670228	4.139602	7.25E-05
ENSG0000(	TRIM38	1.670136	23.53068	2.95E-24
ENSG0000(	TNFSF13	1.670091	7.057425	8.76E-08
ENSG0000(	AC108863.	1.669839	9.913839	1.22E-10
ENSG0000(	AC103691.	1.669687	1.472584	0.033683
ENSG0000(	NR2F1-AS1	1.669295	1.363638	0.043287
ENSG0000(	SYTL1	1.668978	2.530837	0.002946
ENSG0000(	MIR34AHG	1.668149	5.23149	5.87E-06
ENSG0000(	RUNX2	1.667876	3.868419	0.000135
ENSG0000(	TRABD2A	1.666544	8.916087	1.21E-09
ENSG0000(	MAMDC2-/	1.66603	7.012309	9.72E-08
ENSG0000(	LOXL4	1.664609	5.057088	8.77E-06
ENSG0000(	NMI	1.664425	10.86263	1.37E-11
ENSG0000(	MRC2	1.662335	5.811014	1.55E-06
ENSG0000(	LRRC37A4F	1.662278	3.28509	0.000519
ENSG0000(	AL358334.:	1.661826	3.361496	0.000435
ENSG0000(	ARL4A	1.661739	5.764686	1.72E-06
ENSG0000(	PPM1M	1.66128	9.044627	9.02E-10
ENSG0000(	MT1M	1.66096	1.633474	0.023256
ENSG0000(	SNX22	1.66016	7.079481	8.33E-08
ENSG0000(	KRT80	1.65954	1.446292	0.035786
ENSG0000(	AC084876.	1.658584	1.560679	0.027499
ENSG0000(	PAPLN	1.658139	5.313862	4.85E-06
ENSG0000(	ADGRG6	1.658079	8.452889	3.52E-09
ENSG0000(	EPHA3	1.657964	2.811283	0.001544
ENSG0000(	PRR5L	1.657946	4.446497	3.58E-05
ENSG0000(	DDR2	1.657738	4.430518	3.71E-05
ENSG0000(	GLT8D2	1.657381	4.760082	1.74E-05
ENSG0000(	CCL14	1.656089	4.04589	9.00E-05
ENSG0000(	RF01978	1.65536	1.738456	0.018262
ENSG0000(	TMEM51	1.655084	5.077166	8.37E-06
ENSG0000(	ANG	1.654572	4.15731	6.96E-05
ENSG0000(	NXPH3	1.653686	3.961602	0.000109
ENSG0000(	PDE6B	1.652675	2.109776	0.007766
ENSG0000(	IFITM3	1.652617	5.385447	4.12E-06
ENSG0000(	PODN	1.652482	4.775858	1.68E-05

ENSG0000(	ZNF503	1.647622	4.724203	1.89E-05
ENSG0000(	EPHA7	1.647146	1.474392	0.033543
ENSG0000(	KCNE3	1.645094	4.451335	3.54E-05
ENSG0000(	GNB4	1.64496	9.592972	2.55E-10
ENSG0000(	MSC	1.644483	14.71616	1.92E-15
ENSG0000(	LHFPL2	1.643016	5.918267	1.21E-06
ENSG0000(	SDC2	1.64282	8.362603	4.34E-09
ENSG0000(	DDX60	1.641784	7.892436	1.28E-08
ENSG0000(	AC116667.	1.640534	1.765944	0.017142
ENSG0000(	SRC	1.64021	8.903486	1.25E-09
ENSG0000(	NES	1.640174	5.496538	3.19E-06
ENSG0000(	GSN	1.638373	7.281601	5.23E-08
ENSG0000(	EVC2	1.637248	5.415488	3.84E-06
ENSG0000(	PROS1	1.636273	5.349058	4.48E-06
ENSG0000(	CLEC11A	1.635972	10.03433	9.24E-11
ENSG0000(	LMO3	1.63568	2.466294	0.003417
ENSG0000(	SGCE	1.635649	8.153372	7.02E-09
ENSG0000(	FEZ1	1.634282	4.550591	2.81E-05
ENSG0000(	EPB41L3	1.633895	8.989119	1.03E-09
ENSG0000(	MIR7703	1.632999	11.99783	1.00E-12
ENSG0000(	TCEAL7	1.632623	5.138526	7.27E-06
ENSG0000(	AC066613.	1.632455	2.971041	0.001069
ENSG0000(	TUBA1A	1.632384	8.821977	1.51E-09
ENSG0000(	IGF1	1.630814	9.367982	4.29E-10
ENSG0000(	MICALL2	1.630692	7.768067	1.71E-08
ENSG0000(	INPP5D	1.630205	8.941373	1.14E-09
ENSG0000(	MEG8	1.629248	3.460959	0.000346
ENSG0000(	AP000781.	1.629172	4.869555	1.35E-05
ENSG0000(	FGD3	1.629141	5.695734	2.01E-06
ENSG0000(	SLC38A1	1.628929	4.468243	3.40E-05
ENSG0000(	FN1	1.62861	7.590406	2.57E-08
ENSG0000(	KCNAB2	1.628528	7.269704	5.37E-08
ENSG0000(	SRGAP2C	1.628523	10.29127	5.11E-11
ENSG0000(	STK32A	1.628182	1.445255	0.035871
ENSG0000(	PPIB	1.627351	6.955534	1.11E-07
ENSG0000(	APOL3	1.626955	11.9247	1.19E-12
ENSG0000(	PHOSPHO1	1.626885	4.098814	7.97E-05
ENSG0000(	MAFF	1.626651	3.544024	0.000286
ENSG0000(	LINC01500	1.625798	2.358313	0.004382
ENSG0000(	XRCC4	1.625759	5.316016	4.83E-06
ENSG0000(	CALHM2	1.624829	6.731903	1.85E-07
ENSG0000(	NUDT18	1.623021	6.262567	5.46E-07
ENSG0000(	LSP1	1.622931	12.49176	3.22E-13
ENSG0000(	DTX1	1.622844	3.526405	0.000298
ENSG0000(	VASN	1.622443	2.605219	0.002482
ENSG0000(	DNMT3B	1.621929	4.419262	3.81E-05
ENSG0000(	EEF1A1	1.621261	12.02415	9.46E-13

ENSG0000(	TRIM6-TRII	1.620679	4.983395	1.04E-05
ENSG0000(	SRRM3	1.620556	1.400948	0.039724
ENSG0000(	HAPLN3	1.620537	5.006645	9.85E-06
ENSG0000(	CTTNBP2	1.620068	2.015309	0.009654
ENSG0000(	SERPINF2	1.619301	3.457209	0.000349
ENSG0000(	LRP8	1.618918	4.355852	4.41E-05
ENSG0000(	TNXB	1.618145	5.049472	8.92E-06
ENSG0000(	LRRN4CL	1.617779	4.490003	3.24E-05
ENSG0000(	AL049830.:	1.617089	1.509412	0.030945
ENSG0000(	AC007938.	1.61697	4.416774	3.83E-05
ENSG0000(	A1BG	1.616895	5.206468	6.22E-06
ENSG0000(	CPQ	1.616802	7.477789	3.33E-08
ENSG0000(	AL357033.4	1.616761	1.403594	0.039483
ENSG0000(	CD37	1.616754	7.239464	5.76E-08
ENSG0000(	SMC5-AS1	1.615541	8.632766	2.33E-09
ENSG0000(	AL049839.:	1.614601	4.920218	1.20E-05
ENSG0000(	LAT	1.61329	6.555123	2.79E-07
ENSG0000(	PRF1	1.612113	3.814598	0.000153
ENSG0000(	UST	1.612101	5.386147	4.11E-06
ENSG0000(	ZNF610	1.61113	2.292515	0.005099
ENSG0000(	AC009086.	1.611117	8.357163	4.39E-09
ENSG0000(	CXCR4	1.610959	6.326732	4.71E-07
ENSG0000(	LRRK1	1.610352	6.296577	5.05E-07
ENSG0000(	MDK	1.610016	6.525577	2.98E-07
ENSG0000(	AC093535.	1.608736	2.11955	0.007594
ENSG0000(	RAP2B	1.608464	11.65221	2.23E-12
ENSG0000(	GVINP1	1.608424	3.685694	0.000206
ENSG0000(	VAMP8	1.608134	8.883128	1.31E-09
ENSG0000(	SLC43A3	1.607908	4.893631	1.28E-05
ENSG0000(	PLEKHA4	1.606268	14.4885	3.25E-15
ENSG0000(	GAS7	1.60587	7.578796	2.64E-08
ENSG0000(	GPR1	1.605522	3.178107	0.000664
ENSG0000(	FREM1	1.603771	5.573048	2.67E-06
ENSG0000(	GAS2L3	1.6026	2.128244	0.007443
ENSG0000(	ST8SIA4	1.601922	6.967178	1.08E-07
ENSG0000(	DCST1	1.601425	1.572744	0.026746
ENSG0000(	RFX2	1.601164	6.718915	1.91E-07
ENSG0000(	PLEKHA2	1.60076	12.84298	1.44E-13
ENSG0000(	DAP	1.600526	6.394122	4.04E-07
ENSG0000(	CTSC	1.598164	7.475293	3.35E-08
ENSG0000(	TMEM255A	1.598154	3.01456	0.000967
ENSG0000(	COL6A2	1.597706	8.302007	4.99E-09
ENSG0000(	ARHGAP25	1.597539	7.456033	3.50E-08
ENSG0000(	FOS	1.595342	2.186634	0.006507
ENSG0000(	RAB37	1.595271	4.481251	3.30E-05
ENSG0000(	F2R	1.5943	5.81299	1.54E-06
ENSG0000(	LTB	1.594285	2.02407	0.009461

ENSG0000(	TRIM6	1.594022	1.817314	0.01523
ENSG0000(	APCDD1L	1.593913	1.680596	0.020864
ENSG0000(	SLC4A8	1.591343	1.733132	0.018487
ENSG0000(	MRAP2	1.590997	2.201671	0.006285
ENSG0000(	NEGR1	1.589661	5.791536	1.62E-06
ENSG0000(	PRPS2	1.588705	5.905041	1.24E-06
ENSG0000(	MEST	1.588104	6.097953	7.98E-07
ENSG0000(	ZNF781	1.587695	1.370491	0.04261
ENSG0000(	FOXP3	1.58666	2.70338	0.00198
ENSG0000(	ADH1C	1.585081	4.038568	9.15E-05
ENSG0000(	SOX5	1.584747	1.485533	0.032694
ENSG0000(	IRF6	1.584265	1.30085	0.050021
ENSG0000(	NTRK1	1.582622	2.292515	0.005099
ENSG0000(	CABP4	1.581714	6.190375	6.45E-07
ENSG0000(	ARHGAP36	1.579482	2.071984	0.008473
ENSG0000(	HLA-K	1.578989	4.472257	3.37E-05
ENSG0000(	ZAP70	1.577749	3.976842	0.000105
ENSG0000(	P3H3	1.577599	4.072866	8.46E-05
ENSG0000(	PTGIS	1.576998	2.690054	0.002041
ENSG0000(	LIPA	1.576946	11.48177	3.30E-12
ENSG0000(	DACT3	1.576008	3.900412	0.000126
ENSG0000(	ARHGAP45	1.57466	6.608465	2.46E-07
ENSG0000(	AC144831.	1.574189	2.626251	0.002365
ENSG0000(	CARD6	1.57415	6.796026	1.60E-07
ENSG0000(	RAB3IL1	1.573728	5.602045	2.50E-06
ENSG0000(	BLOC1S5-T	1.57307	8.72195	1.90E-09
ENSG0000(	SLC2A3	1.572741	6.621063	2.39E-07
ENSG0000(	CDC7	1.572684	3.447121	0.000357
ENSG0000(	GSTM5	1.571108	2.827283	0.001488
ENSG0000(	ZNF385D	1.570373	8.62843	2.35E-09
ENSG0000(	OLFML2B	1.568441	10.07269	8.46E-11
ENSG0000(	AP003071.	1.564568	2.530836	0.002946
ENSG0000(	AP005205.	1.564258	2.111667	0.007733
ENSG0000(	AL133351.:	1.562685	1.997948	0.010047
ENSG0000(	SEC24D	1.56255	8.920012	1.20E-09
ENSG0000(	NRK	1.562549	1.705152	0.019717
ENSG0000(	KIF7	1.562517	5.900839	1.26E-06
ENSG0000(	EDIL3	1.561548	4.066713	8.58E-05
ENSG0000(	MIAT	1.560888	3.47405	0.000336
ENSG0000(	PERP	1.560417	7.04353	9.05E-08
ENSG0000(	SEL1L3	1.559963	6.804564	1.57E-07
ENSG0000(	SULT1A2	1.559506	1.361094	0.043542
ENSG0000(	LSP1P4	1.557857	1.746939	0.017909
ENSG0000(	STAB1	1.556771	8.15555	6.99E-09
ENSG0000(	AC133065.	1.556349	5.96529	1.08E-06
ENSG0000(	MFRP	1.555385	2.72345	0.00189
ENSG0000(	POU2F2	1.555133	3.403097	0.000395

ENSG0000(	ARHGAP20	1.554584	2.060736	0.008695
ENSG0000(	HELLPAR	1.553967	10.01342	9.70E-11
ENSG0000(	SIRPB1	1.553835	1.460074	0.034668
ENSG0000(	CLSTN2	1.55382	4.416563	3.83E-05
ENSG0000(	C1QTNF5	1.552304	2.711231	0.001944
ENSG0000(	SLC25A43	1.551676	6.141537	7.22E-07
ENSG0000(	HAVCR2	1.55141	5.365895	4.31E-06
ENSG0000(	FBXO17	1.551141	4.219665	6.03E-05
ENSG0000(	MRGPRF	1.55071	3.368848	0.000428
ENSG0000(	MAL	1.549235	2.041208	0.009095
ENSG0000(	ZNF300	1.549076	2.435121	0.003672
ENSG0000(	S100A2	1.548466	1.380763	0.041614
ENSG0000(	IL4R	1.54515	7.478154	3.33E-08
ENSG0000(	ZC3HAV1	1.545135	13.98705	1.03E-14
ENSG0000(	FLT3LG	1.544372	8.588357	2.58E-09
ENSG0000(	MCTP1	1.543742	5.266099	5.42E-06
ENSG0000(	BST1	1.542405	4.122076	7.55E-05
ENSG0000(	SCRN1	1.54234	5.333875	4.64E-06
ENSG0000(	TNFRSF14	1.541708	7.358968	4.38E-08
ENSG0000(	BEX4	1.539549	6.845613	1.43E-07
ENSG0000(	AC087289.	1.538904	11.98428	1.04E-12
ENSG0000(	AL139339.:	1.53864	2.43818	0.003646
ENSG0000(	LINC02202	1.538354	3.499842	0.000316
ENSG0000(	GLMP	1.538169	8.866194	1.36E-09
ENSG0000(	RIPK2	1.538017	5.115088	7.67E-06
ENSG0000(	IFITM2	1.537922	4.855603	1.39E-05
ENSG0000(	JAM3	1.533605	10.24711	5.66E-11
ENSG0000(	SOX8	1.532954	7.435722	3.67E-08
ENSG0000(	SLC1A5	1.532683	6.917048	1.21E-07
ENSG0000(	CASP8	1.530252	8.902546	1.25E-09
ENSG0000(	CHRNG	1.529722	2.274734	0.005312
ENSG0000(	TRIM69	1.529131	6.976295	1.06E-07
ENSG0000(	FMO4	1.528832	2.599881	0.002513
ENSG0000(	MAMDC2	1.528555	10.82964	1.48E-11
ENSG0000(	SYNE3	1.528513	5.835279	1.46E-06
ENSG0000(	SLC24A4	1.527651	1.465051	0.034273
ENSG0000(	SPAG4	1.527365	2.222747	0.005988
ENSG0000(	CD276	1.527053	6.904513	1.25E-07
ENSG0000(	PRKCD	1.526948	6.184257	6.54E-07
ENSG0000(	GBGT1	1.526403	4.727686	1.87E-05
ENSG0000(	PSD3	1.526325	4.892128	1.28E-05
ENSG0000(	AC009041.	1.525675	8.297873	5.04E-09
ENSG0000(	SAT1	1.525262	6.789299	1.62E-07
ENSG0000(	KCNK6	1.524893	7.869712	1.35E-08
ENSG0000(	MDGA1	1.524828	5.844932	1.43E-06
ENSG0000(	EEF1A1P5	1.523815	10.38383	4.13E-11
ENSG0000(	RPS6KA1	1.523637	4.858842	1.38E-05

ENSG0000(	TRIM47	1.522381	10.12042	7.58E-11
ENSG0000(	ZNF329	1.521538	10.11335	7.70E-11
ENSG0000(	ORAI2	1.521371	10.9659	1.08E-11
ENSG0000(	TTYH3	1.519774	8.760553	1.74E-09
ENSG0000(	AC022107.	1.519638	1.542684	0.028663
ENSG0000(	ELF4	1.519179	7.539636	2.89E-08
ENSG0000(	GSDME	1.517287	7.352557	4.44E-08
ENSG0000(	CTSZ	1.516274	9.647181	2.25E-10
ENSG0000(	ALPL	1.515851	2.451233	0.003538
ENSG0000(	APOL2	1.515804	10.63111	2.34E-11
ENSG0000(	FCHSD1	1.51506	6.782775	1.65E-07
ENSG0000(	NHSL1	1.514905	4.105241	7.85E-05
ENSG0000(	CLIC4	1.514817	19.82609	1.49E-20
ENSG0000(	VAT1	1.514321	5.374925	4.22E-06
ENSG0000(	EFS	1.512925	4.211147	6.15E-05
ENSG0000(	MYCN	1.512013	2.672813	0.002124
ENSG0000(	AL135818.:	1.511727	1.85184	0.014066
ENSG0000(	CYP1B1-AS	1.510978	4.635751	2.31E-05
ENSG0000(	ADAMTS4	1.510748	2.005544	0.009873
ENSG0000(	LRP1	1.509594	4.851916	1.41E-05
ENSG0000(	PARVA	1.508805	7.196899	6.35E-08
ENSG0000(	PTPRF	1.508764	3.923565	0.000119
ENSG0000(	SRGAP1	1.507087	6.427204	3.74E-07
ENSG0000(	SLFN13	1.506383	5.804028	1.57E-06
ENSG0000(	SAMD9L	1.505984	6.545258	2.85E-07
ENSG0000(	IL16	1.505499	5.085676	8.21E-06
ENSG0000(	LGALS3	1.505436	7.587706	2.58E-08
ENSG0000(	EHD3	1.505141	10.87593	1.33E-11
ENSG0000(	ADAM12	1.50509	5.437051	3.66E-06
ENSG0000(	CAVIN3	1.504115	11.10799	7.80E-12
ENSG0000(	SERTAD4	1.504105	3.32185	0.000477
ENSG0000(	KCNK5	1.50394	1.430929	0.037074
ENSG0000(	HDC	1.503762	2.316416	0.004826
ENSG0000(	ARHGAP22	1.501204	6.972258	1.07E-07
ENSG0000(	PNMA8B	1.500762	3.503489	0.000314
ENSG0000(	DUSP4	1.500636	4.813556	1.54E-05
ENSG0000(	CEP152	1.500445	2.541158	0.002876
ENSG0000(	C2orf81	1.500287	2.093924	0.008055
ENSG0000(	BEND6	1.500131	2.148638	0.007102
ENSG0000(	SCAMP5	1.50003	4.591475	2.56E-05
ENSG0000(	AC007298.	1.497536	1.396601	0.040124
ENSG0000(	ECM1	1.497096	4.237352	5.79E-05
ENSG0000(	HS3ST3B1	1.496996	2.256681	0.005538
ENSG0000(	F10	1.495594	4.783895	1.64E-05
ENSG0000(	ENPP2	1.494383	3.75701	0.000175
ENSG0000(	EPHB2	1.493996	4.566626	2.71E-05
ENSG0000(	HIST1H2BN	1.493988	1.594027	0.025467

ENSG0000(	CST3	1.492543	11.06746	8.56E-12
ENSG0000(	L3MBTL4	1.490894	6.248687	5.64E-07
ENSG0000(	GPR173	1.490465	3.834264	0.000146
ENSG0000(	ELOVL7	1.490364	1.61799	0.0241
ENSG0000(	TRERF1	1.489151	7.339829	4.57E-08
ENSG0000(	CASP17P	1.487139	5.81	1.55E-06
ENSG0000(	LFNG	1.487111	4.989147	1.03E-05
ENSG0000(	TMEM35B	1.486485	5.974585	1.06E-06
ENSG0000(	FAT1	1.486249	9.787973	1.63E-10
ENSG0000(	PGM2	1.485703	6.485688	3.27E-07
ENSG0000(	TGFBR3	1.48561	3.7027	0.000198
ENSG0000(	CD247	1.485249	1.882266	0.013114
ENSG0000(	BRSK1	1.484847	2.058886	0.008732
ENSG0000(	ANGPTL6	1.484778	5.982709	1.04E-06
ENSG0000(	COL5A1	1.483675	5.50014	3.16E-06
ENSG0000(	ANXA4	1.483155	5.824493	1.50E-06
ENSG0000(	STAT4	1.481607	2.47994	0.003312
ENSG0000(	HSD11B2	1.481573	1.673476	0.021209
ENSG0000(	FZD10-DT	1.481436	5.441357	3.62E-06
ENSG0000(	AC005041.	1.481373	2.158912	0.006936
ENSG0000(	SUSD2	1.480932	6.046857	8.98E-07
ENSG0000(	FBLIM1	1.480853	6.863862	1.37E-07
ENSG0000(	TSPAN32	1.480542	1.956847	0.011045
ENSG0000(	ARRB2	1.480084	8.215025	6.10E-09
ENSG0000(	GALNT3	1.479452	1.821744	0.015075
ENSG0000(	TMEM86A	1.479394	10.51021	3.09E-11
ENSG0000(	EVC	1.479393	6.083274	8.26E-07
ENSG0000(	ERAP2	1.479284	2.870235	0.001348
ENSG0000(	MARCKS	1.479253	6.726369	1.88E-07
ENSG0000(	ARHGDIB	1.479052	6.986392	1.03E-07
ENSG0000(	SYNGAP1-/	1.478227	1.767008	0.0171
ENSG0000(	ZNF888	1.478081	3.309753	0.00049
ENSG0000(	TCEAL9	1.476707	5.483877	3.28E-06
ENSG0000(	PMAIP1	1.476143	3.58805	0.000258
ENSG0000(	BMF-AS1	1.475416	2.401694	0.003966
ENSG0000(	PRCP	1.47529	7.417871	3.82E-08
ENSG0000(	RAB8B	1.474932	7.884491	1.30E-08
ENSG0000(	SPHK1	1.474239	5.37221	4.24E-06
ENSG0000(	FBLN5	1.474158	6.771656	1.69E-07
ENSG0000(	DMXL2	1.472236	7.451764	3.53E-08
ENSG0000(	TENM1	1.469329	3.397767	0.0004
ENSG0000(	STAT6	1.468567	6.769897	1.70E-07
ENSG0000(	ZFP82	1.468301	9.119766	7.59E-10
ENSG0000(	SCD5	1.467116	2.601442	0.002504
ENSG0000(	AL357060.:	1.466853	4.566591	2.71E-05
ENSG0000(	PTPN13	1.466515	4.656467	2.21E-05
ENSG0000(	SPACA9	1.466358	3.002191	0.000995

ENSG0000(	CBR3-AS1	1.466034	4.591033	2.56E-05
ENSG0000(	GNG2	1.465897	8.046269	8.99E-09
ENSG0000(	PLXNB1	1.465631	4.513777	3.06E-05
ENSG0000(	C19orf66	1.465205	7.163623	6.86E-08
ENSG0000(	ADAM8	1.465056	3.478486	0.000332
ENSG0000(	MEIS3	1.464788	4.827901	1.49E-05
ENSG0000(	PTGFR	1.46443	4.335328	4.62E-05
ENSG0000(	AC011510.	1.464329	4.612413	2.44E-05
ENSG0000(	ARSD	1.464218	8.205015	6.24E-09
ENSG0000(	TAGLN2	1.46402	8.188221	6.48E-09
ENSG0000(	CNPY4	1.462835	5.014061	9.68E-06
ENSG0000(	AL359762.:	1.461236	1.631172	0.023379
ENSG0000(	IGFLR1	1.459981	8.026525	9.41E-09
ENSG0000(	GPC6	1.459818	7.545692	2.85E-08
ENSG0000(	SFXN3	1.459384	5.687332	2.05E-06
ENSG0000(	EMILIN1	1.458961	5.600296	2.51E-06
ENSG0000(	AL355581.:	1.458894	1.890455	0.012869
ENSG0000(	TSKU	1.458847	3.619109	0.00024
ENSG0000(	AC020931.	1.458386	6.503845	3.13E-07
ENSG0000(	VAV3	1.457858	3.094524	0.000804
ENSG0000(	SPIN4	1.456119	1.839378	0.014475
ENSG0000(	BAIAP2-DT	1.455937	6.470253	3.39E-07
ENSG0000(	WASF1	1.455329	1.781094	0.016554
ENSG0000(	AC020915.	1.45418	7.471264	3.38E-08
ENSG0000(	TICRR	1.453479	2.588206	0.002581
ENSG0000(	BMX	1.453138	1.535529	0.029139
ENSG0000(	FYN	1.45177	6.6206	2.40E-07
ENSG0000(	TMC6	1.45093	6.151247	7.06E-07
ENSG0000(	AC093673.	1.449623	4.971835	1.07E-05
ENSG0000(	AC134043.	1.449406	2.90921	0.001233
ENSG0000(	VIT	1.449252	5.145957	7.15E-06
ENSG0000(	SRPX2	1.448794	3.317153	0.000482
ENSG0000(	MREG	1.448368	5.392077	4.05E-06
ENSG0000(	FXYD5	1.448036	6.2234	5.98E-07
ENSG0000(	LACC1	1.447283	6.975376	1.06E-07
ENSG0000(	FBXO36	1.447218	2.1716	0.006736
ENSG0000(	LPAR2	1.446636	1.486879	0.032593
ENSG0000(	BOK	1.446513	4.553995	2.79E-05
ENSG0000(	DACT1	1.44645	4.563002	2.74E-05
ENSG0000(	TMEM14A	1.445957	4.734401	1.84E-05
ENSG0000(	LPIN2	1.44556	7.954786	1.11E-08
ENSG0000(	MVP	1.44526	13.41717	3.83E-14
ENSG0000(	CORO1B	1.445243	8.472853	3.37E-09
ENSG0000(	COL6A1	1.444914	8.090105	8.13E-09
ENSG0000(	SLC12A7	1.444816	10.06632	8.58E-11
ENSG0000(	RAB34	1.443944	4.775914	1.68E-05
ENSG0000(	NCAM1-AS	1.443189	2.836656	0.001457

ENSG0000(	PRR7	1.442633	1.881743	0.01313
ENSG0000(	FNDC4	1.441996	2.056462	0.008781
ENSG0000(	TLNRD1	1.441407	10.93769	1.15E-11
ENSG0000(	PARP12	1.441061	9.618653	2.41E-10
ENSG0000(	FBXL7	1.441041	5.777062	1.67E-06
ENSG0000(	RSAD2	1.440382	4.489614	3.24E-05
ENSG0000(	ATP10D	1.440256	4.960209	1.10E-05
ENSG0000(	STARD5	1.440147	4.766227	1.71E-05
ENSG0000(	JAZF1	1.439716	5.079334	8.33E-06
ENSG0000(	CLDN11	1.439324	2.51697	0.003041
ENSG0000(	FAM110A	1.439199	5.890807	1.29E-06
ENSG0000(	CMTM6	1.438034	6.964823	1.08E-07
ENSG0000(	COL6A3	1.436817	4.933097	1.17E-05
ENSG0000(	RABGAP1L	1.436342	8.209512	6.17E-09
ENSG0000(	PTPRD	1.43628	5.292942	5.09E-06
ENSG0000(	AC008649.	1.435823	3.536226	0.000291
ENSG0000(	CMTM1	1.434747	2.519458	0.003024
ENSG0000(	BTN2A3P	1.431843	4.819907	1.51E-05
ENSG0000(	NUMBL	1.430732	8.953597	1.11E-09
ENSG0000(	ADGRE2	1.43055	1.726901	0.018754
ENSG0000(	PSME1	1.430117	14.71616	1.92E-15
ENSG0000(	MLLT11	1.428061	7.292636	5.10E-08
ENSG0000(	NPC2	1.427897	6.780361	1.66E-07
ENSG0000(	MAML2	1.427436	8.764793	1.72E-09
ENSG0000(	LINC01145	1.426109	3.03412	0.000924
ENSG0000(	AC004593.	1.424283	7.17283	6.72E-08
ENSG0000(	PLSCR1	1.423244	4.479129	3.32E-05
ENSG0000(	GABBR1	1.423197	4.924894	1.19E-05
ENSG0000(	SOAT1	1.422727	9.13749	7.29E-10
ENSG0000(	COL8A1	1.422725	3.314041	0.000485
ENSG0000(	IGFBP4	1.422022	6.623838	2.38E-07
ENSG0000(	AL137009.:	1.421578	4.810924	1.55E-05
ENSG0000(	AC008622.	1.421504	4.329171	4.69E-05
ENSG0000(	GAS1	1.420675	3.968433	0.000108
ENSG0000(	AC092535.	1.420549	1.841009	0.014421
ENSG0000(	PALM	1.420477	3.274156	0.000532
ENSG0000(	DDX60L	1.420049	4.536299	2.91E-05
ENSG0000(	SLAMF8	1.419853	4.053311	8.84E-05
ENSG0000(	EVA1B	1.418357	4.702157	1.99E-05
ENSG0000(	MFHAS1	1.418146	11.52008	3.02E-12
ENSG0000(	EBF1	1.416787	4.536216	2.91E-05
ENSG0000(	RELL2	1.416638	3.375267	0.000421
ENSG0000(	LINC01232	1.416427	5.795358	1.60E-06
ENSG0000(	CHN1	1.416119	7.636642	2.31E-08
ENSG0000(	UBA7	1.415036	8.271384	5.35E-09
ENSG0000(	SERPINB9P	1.413867	1.406595	0.039211
ENSG0000(	AXL	1.413858	4.52873	2.96E-05

ENSG0000(	FGR	1.410053	2.429889	0.003716
ENSG0000(	AL772307.:	1.410021	1.380777	0.041612
ENSG0000(	ATP2A3	1.409951	5.703127	1.98E-06
ENSG0000(	AC023906.	1.40983	1.646979	0.022543
ENSG0000(	PCDHB15	1.40957	2.496993	0.003184
ENSG0000(	CADPS2	1.409162	4.037293	9.18E-05
ENSG0000(	MYO7A	1.408305	5.922008	1.20E-06
ENSG0000(	SPATA13	1.408257	11.20976	6.17E-12
ENSG0000(	PHLDA1	1.407932	8.936001	1.16E-09
ENSG0000(	PLVAP	1.406236	5.603715	2.49E-06
ENSG0000(	AC012313.	1.403352	10.94357	1.14E-11
ENSG0000(	WDR76	1.402448	4.302965	4.98E-05
ENSG0000(	MIR5193	1.400097	5.956149	1.11E-06
ENSG0000(	CNPY3	1.399688	8.668306	2.15E-09
ENSG0000(	OSR1	1.399377	5.48305	3.29E-06
ENSG0000(	HLA-L	1.399178	5.47172	3.38E-06
ENSG0000(	ZNF813	1.3984	2.966441	0.00108
ENSG0000(	ANTXR2	1.398078	8.396217	4.02E-09
ENSG0000(	NRP2	1.396691	6.623173	2.38E-07
ENSG0000(	SAMHD1	1.3959	8.425985	3.75E-09
ENSG0000(	AC068733.	1.395859	9.821338	1.51E-10
ENSG0000(	NEK8	1.393648	3.576222	0.000265
ENSG0000(	VSTM4	1.392936	7.660609	2.18E-08
ENSG0000(	PKD2	1.392824	4.378863	4.18E-05
ENSG0000(	CCDC152	1.392742	7.011322	9.74E-08
ENSG0000(	HR	1.39266	3.109628	0.000777
ENSG0000(	LRCH2	1.39248	2.089474	0.008138
ENSG0000(	BST2	1.391748	3.970026	0.000107
ENSG0000(	AC016876.	1.391615	1.653233	0.022221
ENSG0000(	LIMD2	1.390534	4.313403	4.86E-05
ENSG0000(	TGFBI	1.390292	6.668608	2.14E-07
ENSG0000(	HOXB7	1.389996	2.064273	0.008624
ENSG0000(	ZNF521	1.389656	4.249606	5.63E-05
ENSG0000(	TLDC2	1.388008	8.196416	6.36E-09
ENSG0000(	ADORA1	1.387862	4.916312	1.21E-05
ENSG0000(	SH3TC1	1.387719	8.472853	3.37E-09
ENSG0000(	DTX3L	1.387425	8.151406	7.06E-09
ENSG0000(	ELMO1	1.387226	6.803908	1.57E-07
ENSG0000(	GALNT12	1.387218	2.326012	0.004721
ENSG0000(	LPXN	1.386552	6.906756	1.24E-07
ENSG0000(	AC096677.	1.386164	1.764943	0.017181
ENSG0000(	AL157786.:	1.386123	5.492133	3.22E-06
ENSG0000(	MIR7705	1.38533	4.631092	2.34E-05
ENSG0000(	BOC	1.384178	5.522551	3.00E-06
ENSG0000(	DIRC3	1.383657	2.096506	0.008007
ENSG0000(	HAAO	1.382112	5.713105	1.94E-06
ENSG0000(	MYADM	1.38209	6.872755	1.34E-07

ENSG0000(	BACE2	1.381832	5.119632	7.59E-06
ENSG0000(	MAP3K7CL	1.381684	2.532366	0.002935
ENSG0000(	GLI3	1.380231	4.615511	2.42E-05
ENSG0000(	BTG3	1.380138	5.706838	1.96E-06
ENSG0000(	DGKA	1.379967	5.285792	5.18E-06
ENSG0000(	ETV1	1.379641	5.445708	3.58E-06
ENSG0000(	PYGL	1.379368	5.463628	3.44E-06
ENSG0000(	KLF16	1.37895	8.368168	4.28E-09
ENSG0000(	DZIP1	1.37821	4.697719	2.01E-05
ENSG0000(	TBC1D16	1.377941	10.97704	1.05E-11
ENSG0000(	RF01872	1.37777	5.400084	3.98E-06
ENSG0000(	QPRT	1.377605	7.16984	6.76E-08
ENSG0000(	PDE8B	1.377497	2.297524	0.005041
ENSG0000(	CBR1	1.376722	12.36782	4.29E-13
ENSG0000(	LYL1	1.375397	4.029712	9.34E-05
ENSG0000(	DBN1	1.375052	6.059581	8.72E-07
ENSG0000(	RPL22L1	1.375026	5.664103	2.17E-06
ENSG0000(	LINC00869	1.37459	8.54139	2.87E-09
ENSG0000(	AC008894.	1.374465	5.450061	3.55E-06
ENSG0000(	RAB11FIP4	1.373687	3.507447	0.000311
ENSG0000(	SERPINB9	1.373116	7.62561	2.37E-08
ENSG0000(	MIR671	1.373097	5.533002	2.93E-06
ENSG0000(	TRABD2B	1.372983	2.558821	0.002762
ENSG0000(	TPM4	1.372689	6.008839	9.80E-07
ENSG0000(	PLEKHA6	1.372554	4.478853	3.32E-05
ENSG0000(	ZNF670	1.371698	2.176593	0.006659
ENSG0000(	PSME2	1.371347	12.05399	8.83E-13
ENSG0000(	AC010261.	1.370383	1.722859	0.01893
ENSG0000(	ANKDD1A	1.369009	5.316943	4.82E-06
ENSG0000(	SLC25A22	1.36709	6.87087	1.35E-07
ENSG0000(	AL359220.:	1.366887	1.321566	0.047691
ENSG0000(	SPEF2	1.366834	1.88708	0.012969
ENSG0000(	AC097534.	1.366697	1.646929	0.022546
ENSG0000(	FKBP1B	1.366569	1.969483	0.010728
ENSG0000(	GADD45B	1.366489	8.064974	8.61E-09
ENSG0000(	MEIS3P1	1.365741	2.911968	0.001225
ENSG0000(	PARPBP	1.365548	1.957819	0.01102
ENSG0000(	FER1L4	1.364645	3.275134	0.000531
ENSG0000(	MSC-AS1	1.364539	13.98062	1.05E-14
ENSG0000(	TMEM51-A	1.363838	3.606773	0.000247
ENSG0000(	PIP4K2A	1.363594	8.283897	5.20E-09
ENSG0000(	TGFB2-AS1	1.363578	1.569591	0.026941
ENSG0000(	PLEKHA5	1.363447	5.166662	6.81E-06
ENSG0000(	RFTN2	1.363189	3.374282	0.000422
ENSG0000(	OSBPL3	1.362392	4.977416	1.05E-05
ENSG0000(	MSX1	1.361423	3.314992	0.000484
ENSG0000(	CABLES1	1.361376	3.18578	0.000652

ENSG0000(	HELZ2	1.360432	4.515799	3.05E-05
ENSG0000(	KIF21B	1.358752	4.982186	1.04E-05
ENSG0000(	AC234582.	1.35851	4.23713	5.79E-05
ENSG0000(	AC092070.	1.358451	5.009353	9.79E-06
ENSG0000(	TRAF4	1.358139	7.552503	2.80E-08
ENSG0000(	LRRC1	1.357724	3.175567	0.000667
ENSG0000(	CRIP2	1.357456	8.440633	3.63E-09
ENSG0000(	AC110597.	1.357241	3.897799	0.000127
ENSG0000(	PLXNC1	1.356181	10.85748	1.39E-11
ENSG0000(	SP140L	1.356127	12.62298	2.38E-13
ENSG0000(	KCTD12	1.355371	10.01764	9.60E-11
ENSG0000(	PCDHB10	1.355112	1.814443	0.015331
ENSG0000(	EFNA5	1.354359	2.474194	0.003356
ENSG0000(	PCDHAC2	1.354009	1.709524	0.01952
ENSG0000(	TRAFD1	1.352829	10.57391	2.67E-11
ENSG0000(	SHISA5	1.352135	6.26283	5.46E-07
ENSG0000(	AC008894.	1.351574	6.318748	4.80E-07
ENSG0000(	TRIM5	1.351261	7.384387	4.13E-08
ENSG0000(	AC026691.	1.350362	1.794097	0.016066
ENSG0000(	CPNE8	1.349942	5.854976	1.40E-06
ENSG0000(	RINL	1.349229	5.674966	2.11E-06
ENSG0000(	CMIP	1.348502	8.003594	9.92E-09
ENSG0000(	AC006460.	1.348452	2.029256	0.009349
ENSG0000(	MEG9	1.348333	3.460393	0.000346
ENSG0000(	GNA14	1.347963	2.333753	0.004637
ENSG0000(	CELSR1	1.347772	3.361496	0.000435
ENSG0000(	DOK3	1.347704	6.614353	2.43E-07
ENSG0000(	TRAF5	1.347401	8.329498	4.68E-09
ENSG0000(	U91328.2	1.347096	17.69398	2.02E-18
ENSG0000(	CDC25B	1.346509	6.958653	1.10E-07
ENSG0000(	AC008440.	1.346125	5.292253	5.10E-06
ENSG0000(	CBLN1	1.34592	1.493392	0.032108
ENSG0000(	ITM2A	1.345344	6.932986	1.17E-07
ENSG0000(	HHIPL1	1.34493	3.673716	0.000212
ENSG0000(	ABHD17C	1.34451	1.988468	0.010269
ENSG0000(	GJA1	1.344026	4.567709	2.71E-05
ENSG0000(	IL17RA	1.343819	5.610585	2.45E-06
ENSG0000(	HSD17B11	1.343319	5.065497	8.60E-06
ENSG0000(	GADD45A	1.342179	3.731955	0.000185
ENSG0000(	C1QTNF2	1.342107	2.160956	0.006903
ENSG0000(	DHX58	1.342019	5.910233	1.23E-06
ENSG0000(	LINC00702	1.341308	4.721765	1.90E-05
ENSG0000(	6-Sep	1.340815	6.601178	2.51E-07
ENSG0000(	NAP1L3	1.339852	2.634351	0.002321
ENSG0000(	MID1	1.339494	4.138648	7.27E-05
ENSG0000(	LBH	1.338973	13.13484	7.33E-14
ENSG0000(	EIF5A2	1.338928	3.891633	0.000128

ENSG0000(	NCOA7	1.338861	6.285473	5.18E-07
ENSG0000(	INTS6L	1.33875	4.479804	3.31E-05
ENSG0000(	TOX2	1.338304	2.640032	0.002291
ENSG0000(	NOTCH2	1.337966	6.497778	3.18E-07
ENSG0000(	NR2F1	1.337858	1.577204	0.026473
ENSG0000(	S100A6	1.337471	6.479683	3.31E-07
ENSG0000(	NUAK2	1.337031	2.040518	0.009109
ENSG0000(	FBLN2	1.33684	3.982281	0.000104
ENSG0000(	TRAM2	1.336119	5.181294	6.59E-06
ENSG0000(	EXOSC7	1.335524	4.721931	1.90E-05
ENSG0000(	ADAMTSL2	1.335481	3.071886	0.000847
ENSG0000(	AC022034.	1.335479	1.932056	0.011693
ENSG0000(	ZDHHC15	1.334589	1.733135	0.018487
ENSG0000(	AL358333.:	1.334493	4.756167	1.75E-05
ENSG0000(	THBS3	1.333989	4.324343	4.74E-05
ENSG0000(	ACSL5	1.331946	9.0357	9.21E-10
ENSG0000(	LPCAT2	1.331837	6.30128	5.00E-07
ENSG0000(	PTPRN2	1.331789	5.547321	2.84E-06
ENSG0000(	LINC00963	1.331591	10.2799	5.25E-11
ENSG0000(	SESN3	1.331215	9.266758	5.41E-10
ENSG0000(	AL139384.:	1.330508	2.337093	0.004602
ENSG0000(	AC011611.	1.330349	5.148229	7.11E-06
ENSG0000(	CDC42EP5	1.330179	3.149913	0.000708
ENSG0000(	LTBP1	1.329546	4.573967	2.67E-05
ENSG0000(	PAG1	1.328868	5.75164	1.77E-06
ENSG0000(	CLCF1	1.328851	2.601442	0.002504
ENSG0000(	TTC7A	1.328564	5.626482	2.36E-06
ENSG0000(	IDO1	1.327399	1.912873	0.012222
ENSG0000(	TBC1D8B	1.326969	1.524585	0.029882
ENSG0000(	SPIRE2	1.325557	1.3307	0.046698
ENSG0000(	AKR1C3	1.325118	4.306655	4.94E-05
ENSG0000(	IMPDH1	1.32506	5.860471	1.38E-06
ENSG0000(	TAX1BP3	1.324439	7.182625	6.57E-08
ENSG0000(	CMPK2	1.324302	4.563002	2.74E-05
ENSG0000(	FST	1.324057	2.737769	0.001829
ENSG0000(	HYMAI	1.32367	3.11483	0.000768
ENSG0000(	RBBP8	1.323595	4.80409	1.57E-05
ENSG0000(	AP000688.	1.322975	10.50366	3.14E-11
ENSG0000(	LCNL1	1.322913	1.506816	0.03113
ENSG0000(	C5orf56	1.32272	10.68839	2.05E-11
ENSG0000(	HELB	1.321914	5.580093	2.63E-06
ENSG0000(	MAP3K8	1.321822	5.688208	2.05E-06
ENSG0000(	GABRE	1.321141	2.151244	0.007059
ENSG0000(	TPST1	1.320568	3.487987	0.000325
ENSG0000(	TGIF2	1.320443	7.092807	8.08E-08
ENSG0000(	SCPEP1	1.319989	6.750796	1.78E-07
ENSG0000(	STX11	1.319714	2.624412	0.002375

ENSG0000(	SRGAP2	1.319184	9.742481	1.81E-10
ENSG0000(	ABCG2	1.319026	1.484021	0.032808
ENSG0000(	VAV2	1.317372	8.571453	2.68E-09
ENSG0000(	C2CD2	1.315851	7.339704	4.57E-08
ENSG0000(	BCAT1	1.315561	3.854857	0.00014
ENSG0000(	AC068234.	1.314256	3.083744	0.000825
ENSG0000(	AF186192.:	1.314254	1.302152	0.049871
ENSG0000(	ACOT7	1.314245	7.533517	2.93E-08
ENSG0000(	CTSH	1.313107	4.577684	2.64E-05
ENSG0000(	UGDH	1.311114	4.021641	9.51E-05
ENSG0000(	PCDHA4	1.310102	1.311586	0.048799
ENSG0000(	ETNK2	1.309874	2.295546	0.005064
ENSG0000(	MAN2B2	1.309521	6.085203	8.22E-07
ENSG0000(	GINS1	1.30951	1.391201	0.040625
ENSG0000(	NECAP2	1.30857	6.893577	1.28E-07
ENSG0000(	PQLC3	1.307603	6.53226	2.94E-07
ENSG0000(	TPBG	1.30746	4.751795	1.77E-05
ENSG0000(	EHD4	1.304557	6.482246	3.29E-07
ENSG0000(	PROCA1	1.304487	2.886661	0.001298
ENSG0000(	SLC35D2	1.303685	7.21142	6.15E-08
ENSG0000(	AKAP12	1.302803	4.486656	3.26E-05
ENSG0000(	NMRAL1	1.300202	5.362433	4.34E-06
ENSG0000(	GNB5	1.298532	6.285316	5.18E-07
ENSG0000(	ALPK1	1.298138	8.317163	4.82E-09
ENSG0000(	SHKBP1	1.297865	9.514908	3.06E-10
ENSG0000(	MGST1	1.297164	1.734291	0.018438
ENSG0000(	KIT	1.296667	3.89672	0.000127
ENSG0000(	PCSK5	1.296412	4.076938	8.38E-05
ENSG0000(	MEOX2	1.296381	4.76157	1.73E-05
ENSG0000(	PIEZO1	1.295741	7.518826	3.03E-08
ENSG0000(	RIN3	1.29535	6.306948	4.93E-07
ENSG0000(	NRXN2	1.294624	4.262556	5.46E-05
ENSG0000(	SERP2	1.292998	2.90921	0.001233
ENSG0000(	AC087623.	1.290383	4.193089	6.41E-05
ENSG0000(	GLDN	1.290277	1.876942	0.013276
ENSG0000(	AC108516.	1.290142	3.875497	0.000133
ENSG0000(	KCNC3	1.289947	4.715814	1.92E-05
ENSG0000(	TENT5A	1.289441	4.165205	6.84E-05
ENSG0000(	AC116903.	1.289352	4.215613	6.09E-05
ENSG0000(	TLR5	1.289108	4.73065	1.86E-05
ENSG0000(	TRIQK	1.288779	3.95453	0.000111
ENSG0000(	AC019257.	1.28857	2.939991	0.001148
ENSG0000(	SELENOP	1.288316	6.55218	2.80E-07
ENSG0000(	TBC1D2B	1.287953	4.492905	3.21E-05
ENSG0000(	SH3PXD2B	1.287304	5.882904	1.31E-06
ENSG0000(	WWTR1	1.287239	11.1699	6.76E-12
ENSG0000(	PDE3B	1.287191	1.375436	0.042127

ENSG0000(	BDNF-AS	1.287151	1.999365	0.010015
ENSG0000(	ZNF559	1.286296	5.358246	4.38E-06
ENSG0000(	SAMD10	1.28625	3.555639	0.000278
ENSG0000(	PDGFRA	1.286198	6.173185	6.71E-07
ENSG0000(	ALDH1L2	1.285766	5.568532	2.70E-06
ENSG0000(	SUSD1	1.285735	7.058593	8.74E-08
ENSG0000(	MERTK	1.285666	4.601832	2.50E-05
ENSG0000(	ZNF497	1.285084	4.635751	2.31E-05
ENSG0000(	SPA17	1.285005	2.071111	0.00849
ENSG0000(	NOVA1	1.28491	3.287336	0.000516
ENSG0000(	GDF10	1.284645	2.11416	0.007688
ENSG0000(	MLKL	1.284253	5.237803	5.78E-06
ENSG0000(	LINCMD1	1.283894	1.462867	0.034446
ENSG0000(	TNFSF10	1.283673	5.878221	1.32E-06
ENSG0000(	FAM24B	1.283565	1.469762	0.033903
ENSG0000(	TUBA1C	1.283241	13.83691	1.46E-14
ENSG0000(	AFF3	1.28313	2.348921	0.004478
ENSG0000(	AC022167.	1.282935	5.999322	1.00E-06
ENSG0000(	SLC16A4	1.282344	3.275603	0.00053
ENSG0000(	TMEM37	1.282237	2.180458	0.0066
ENSG0000(	ABI3	1.282206	4.595368	2.54E-05
ENSG0000(	ANGPTL4	1.281323	2.027908	0.009378
ENSG0000(	SP100	1.280579	12.36782	4.29E-13
ENSG0000(	TGFBR2	1.280339	4.704806	1.97E-05
ENSG0000(	SELENOM	1.279918	5.37221	4.24E-06
ENSG0000(	SRGAP2B	1.279453	3.781287	0.000165
ENSG0000(	CD200	1.279275	3.43108	0.000371
ENSG0000(	AC129507.	1.27876	8.020999	9.53E-09
ENSG0000(	ANKRD65	1.278536	6.100663	7.93E-07
ENSG0000(	BCL3	1.278082	5.692847	2.03E-06
ENSG0000(	AC023906.	1.278071	2.164475	0.006847
ENSG0000(	AL136982.7	1.278048	3.112122	0.000772
ENSG0000(	SOCS2	1.277973	3.439912	0.000363
ENSG0000(	RAB8A	1.276975	6.16828	6.79E-07
ENSG0000(	GNB3	1.276857	2.835782	0.00146
ENSG0000(	ST14	1.276203	2.221806	0.006001
ENSG0000(	GDF11	1.275309	7.035649	9.21E-08
ENSG0000(	AL117190.1	1.274592	5.087125	8.18E-06
ENSG0000(	OSTF1	1.274471	8.297873	5.04E-09
ENSG0000(	NDRG1	1.273739	5.62052	2.40E-06
ENSG0000(	TRPC1	1.272462	3.71216	0.000194
ENSG0000(	ABCA6	1.271917	3.476698	0.000334
ENSG0000(	LINC00342	1.271057	3.568924	0.00027
ENSG0000(	FAM102A	1.269864	8.686765	2.06E-09
ENSG0000(	DUBR	1.269792	3.120883	0.000757
ENSG0000(	ADAMTS7	1.269199	4.260546	5.49E-05
ENSG0000(	NT5DC2	1.268953	4.69176	2.03E-05

ENSG0000(	HELLS	1.268675	2.413063	0.003863
ENSG0000(	SERPINH1	1.268231	5.752701	1.77E-06
ENSG0000(	AC114490.	1.267722	5.232465	5.86E-06
ENSG0000(	AGPAT4	1.267167	3.846927	0.000142
ENSG0000(	KIRREL1	1.266696	3.86608	0.000136
ENSG0000(	P3H2	1.265643	2.292805	0.005096
ENSG0000(	HTRA3	1.265593	3.340947	0.000456
ENSG0000(	AL160269.:	1.265274	6.242283	5.72E-07
ENSG0000(	AC092118.	1.265215	1.816384	0.015262
ENSG0000(	C10orf55	1.265066	7.395325	4.02E-08
ENSG0000(	ZNF439	1.263589	1.532267	0.029358
ENSG0000(	FUCA2	1.26308	7.002998	9.93E-08
ENSG0000(	HERC6	1.261397	3.660756	0.000218
ENSG0000(	AC124068.	1.261344	9.446002	3.58E-10
ENSG0000(	FMN1	1.26127	4.324998	4.73E-05
ENSG0000(	NAALADL1	1.260531	3.723821	0.000189
ENSG0000(	AC144652.	1.259842	1.663561	0.021699
ENSG0000(	AC127502.	1.258787	1.445183	0.035877
ENSG0000(	EGLN3	1.258704	3.49319	0.000321
ENSG0000(	AL392172.:	1.257842	3.825163	0.00015
ENSG0000(	GM2A	1.257077	13.85264	1.40E-14
ENSG0000(	SPIN1	1.256468	6.769158	1.70E-07
ENSG0000(	ACKR3	1.255639	2.633596	0.002325
ENSG0000(	ADIRF	1.255128	3.09973	0.000795
ENSG0000(	ARHGEF5	1.254246	1.565716	0.027182
ENSG0000(	ARHGAP24	1.253012	11.62631	2.36E-12
ENSG0000(	RARG	1.253005	3.167677	0.00068
ENSG0000(	NINJ1	1.252958	11.22918	5.90E-12
ENSG0000(	B3GALNT1	1.252926	3.711678	0.000194
ENSG0000(	RRBP1	1.252015	5.42704	3.74E-06
ENSG0000(	TSPAN4	1.251579	4.590659	2.57E-05
ENSG0000(	SERPINA5	1.251215	2.456901	0.003492
ENSG0000(	MEIS2	1.25107	1.646852	0.02255
ENSG0000(	AC132217.	1.249669	3.607785	0.000247
ENSG0000(	SOX12	1.24934	7.203494	6.26E-08
ENSG0000(	COL24A1	1.249138	1.984781	0.010357
ENSG0000(	RERGL	1.247648	1.895986	0.012706
ENSG0000(	ITGA3	1.247495	4.55318	2.80E-05
ENSG0000(	ADAM33	1.246904	2.058335	0.008743
ENSG0000(	ZNF699	1.24686	2.115674	0.007662
ENSG0000(	PLAU	1.246799	8.136994	7.29E-09
ENSG0000(	ADH1B	1.245344	2.958433	0.0011
ENSG0000(	CYBC1	1.243535	7.776511	1.67E-08
ENSG0000(	PRICKLE2	1.243501	7.160381	6.91E-08
ENSG0000(	XYLB	1.243025	3.078367	0.000835
ENSG0000(	RTL5	1.242743	4.165205	6.84E-05
ENSG0000(	CYB561	1.242161	5.465866	3.42E-06

ENSG0000(	AC100791.	1.241995	4.717635	1.92E-05
ENSG0000(	AC105020.	1.241186	6.067677	8.56E-07
ENSG0000(	AC091814.	1.241019	4.849982	1.41E-05
ENSG0000(	KAZN	1.240132	5.31637	4.83E-06
ENSG0000(	CHPF	1.239939	6.896055	1.27E-07
ENSG0000(	GSDMD	1.237989	7.490255	3.23E-08
ENSG0000(	CHD3	1.237256	4.518072	3.03E-05
ENSG0000(	AC138028.	1.236494	5.541317	2.88E-06
ENSG0000(	AP001972.	1.236271	2.084531	0.008231
ENSG0000(	LPAR6	1.234921	5.852974	1.40E-06
ENSG0000(	AC138028.	1.234541	7.035649	9.21E-08
ENSG0000(	PMP22	1.233233	3.573493	0.000267
ENSG0000(	CFI	1.23225	3.756421	0.000175
ENSG0000(	IFT52	1.231004	4.362386	4.34E-05
ENSG0000(	MED12L	1.230731	3.848552	0.000142
ENSG0000(	AP002026.	1.229513	1.743443	0.018053
ENSG0000(	AL603832.:	1.22921	1.328295	0.046957
ENSG0000(	AMPD2	1.228081	7.58791	2.58E-08
ENSG0000(	TNFRSF14-.	1.227501	5.66032	2.19E-06
ENSG0000(	CCDC102A	1.227016	4.010615	9.76E-05
ENSG0000(	TRAF1	1.226797	4.60638	2.48E-05
ENSG0000(	SLFN11	1.226741	5.764127	1.72E-06
ENSG0000(	AC104350.	1.225352	1.517689	0.030361
ENSG0000(	PTTG1	1.225085	2.031186	0.009307
ENSG0000(	MFSD1	1.224872	7.771052	1.69E-08
ENSG0000(	KANK2	1.224528	5.888512	1.29E-06
ENSG0000(	NR5A2	1.224413	1.346361	0.045044
ENSG0000(	SLC22A17	1.22428	3.756062	0.000175
ENSG0000(	SDC3	1.223658	7.047388	8.97E-08
ENSG0000(	CD58	1.22331	9.052482	8.86E-10
ENSG0000(	SNAI2	1.222824	4.724203	1.89E-05
ENSG0000(	PCDHGC3	1.222695	5.195875	6.37E-06
ENSG0000(	NHSL2	1.222148	3.333464	0.000464
ENSG0000(	CSPG4	1.221169	5.764686	1.72E-06
ENSG0000(	TCIRG1	1.220908	4.279009	5.26E-05
ENSG0000(	CBX4	1.220013	8.308235	4.92E-09
ENSG0000(	CORO7	1.219295	7.372741	4.24E-08
ENSG0000(	ELOVL5	1.218578	4.473917	3.36E-05
ENSG0000(	AC125611.	1.218244	8.378871	4.18E-09
ENSG0000(	CERKL	1.218072	1.366074	0.043045
ENSG0000(	RASSF1-AS	1.217626	3.763485	0.000172
ENSG0000(	PCDHA10	1.217552	1.386866	0.041033
ENSG0000(	AL807752.'	1.217525	2.504997	0.003126
ENSG0000(	SLC2A13	1.217326	2.005684	0.00987
ENSG0000(	SPRED1	1.217088	6.567372	2.71E-07
ENSG0000(	FTL	1.21692	6.254643	5.56E-07
ENSG0000(	AP001453.	1.216798	2.175109	0.006682

ENSG0000(	AC104794.	1.21668	1.367478	0.042906
ENSG0000(	AL162742.:	1.216189	1.924038	0.011911
ENSG0000(	PXDNL	1.216015	2.02694	0.009399
ENSG0000(	SHC3	1.215964	2.258263	0.005517
ENSG0000(	AC103810.	1.21587	2.425753	0.003752
ENSG0000(	FAM111A	1.215164	6.540116	2.88E-07
ENSG0000(	TTC8	1.215075	2.713428	0.001935
ENSG0000(	P3H4	1.214336	8.028036	9.37E-09
ENSG0000(	FBXO2	1.213142	1.658713	0.021943
ENSG0000(	GPM6B	1.212905	5.449333	3.55E-06
ENSG0000(	AC007319.	1.21147	5.268211	5.39E-06
ENSG0000(	AC007182.	1.211173	6.391507	4.06E-07
ENSG0000(	PTGDS	1.209643	2.528658	0.00296
ENSG0000(	PTGER4	1.209349	7.319206	4.80E-08
ENSG0000(	PLAT	1.209229	4.343175	4.54E-05
ENSG0000(	AC079880.	1.209191	2.855186	0.001396
ENSG0000(	CCDC88C	1.208675	6.19498	6.38E-07
ENSG0000(	FSCN1	1.208395	5.539704	2.89E-06
ENSG0000(	ZNF665	1.208177	2.207168	0.006206
ENSG0000(	AC020659.	1.208159	1.955345	0.011083
ENSG0000(	GLIS3	1.208093	1.544547	0.02854
ENSG0000(	WDR54	1.207766	4.940789	1.15E-05
ENSG0000(	LIMA1	1.207723	3.803289	0.000157
ENSG0000(	SH3BGRL3	1.207432	4.587334	2.59E-05
ENSG0000(	CCNI2	1.204901	5.609544	2.46E-06
ENSG0000(	GRK3	1.204436	3.820536	0.000151
ENSG0000(	GRN	1.204026	8.399909	3.98E-09
ENSG0000(	AC005703.	1.203316	3.960996	0.000109
ENSG0000(	SIRPA	1.203214	4.370628	4.26E-05
ENSG0000(	NAALAD2	1.203079	2.52661	0.002974
ENSG0000(	CARHSP1	1.203063	6.747051	1.79E-07
ENSG0000(	PCDHA2	1.203034	1.400948	0.039724
ENSG0000(	SLC7A1	1.202586	7.249511	5.63E-08
ENSG0000(	CD226	1.202477	2.576565	0.002651
ENSG0000(	TDRD10	1.202274	3.56106	0.000275
ENSG0000(	KDELC1	1.199936	2.440779	0.003624
ENSG0000(	MIR3917	1.199714	5.759334	1.74E-06
ENSG0000(	AC073130.	1.199411	2.421427	0.003789
ENSG0000(	MMEL1	1.198488	3.709957	0.000195
ENSG0000(	LURAP1L	1.197624	2.939053	0.001151
ENSG0000(	TMEM79	1.197043	6.02571	9.43E-07
ENSG0000(	NTN4	1.196731	8.849535	1.41E-09
ENSG0000(	AC004922.	1.196118	7.49375	3.21E-08
ENSG0000(	TRADD	1.19543	7.395871	4.02E-08
ENSG0000(	CSDC2	1.195194	8.945696	1.13E-09
ENSG0000(	AC022098.	1.19509	4.563002	2.74E-05
ENSG0000(	FGFR1	1.194716	4.458207	3.48E-05

ENSG0000(	FAM167B	1.19418	1.514833	0.030561
ENSG0000(	RMI2	1.194005	2.769164	0.001702
ENSG0000(	ITGA9-AS1	1.19398	5.538201	2.90E-06
ENSG0000(	MIR4751	1.193442	11.62516	2.37E-12
ENSG0000(	NAB2	1.193271	9.274492	5.32E-10
ENSG0000(	DTX4	1.192408	8.550665	2.81E-09
ENSG0000(	ITGA9	1.192333	5.179933	6.61E-06
ENSG0000(	ZNF57	1.191796	1.702918	0.019819
ENSG0000(	CRYBG1	1.191093	6.246031	5.68E-07
ENSG0000(	TRPA1	1.190535	4.099772	7.95E-05
ENSG0000(	TLCD2	1.189747	3.357202	0.000439
ENSG0000(	PCDHB14	1.18971	4.537593	2.90E-05
ENSG0000(	LINC00205	1.189505	7.163955	6.86E-08
ENSG0000(	ACTN1	1.189411	5.061903	8.67E-06
ENSG0000(	TBC1D9	1.189313	6.784418	1.64E-07
ENSG0000(	ID4	1.188203	2.866048	0.001361
ENSG0000(	KIFC1	1.18767	1.97052	0.010702
ENSG0000(	AC006441.	1.187443	8.392567	4.05E-09
ENSG0000(	LINC00265	1.187304	2.405482	0.003931
ENSG0000(	ITGB3	1.187014	3.308551	0.000491
ENSG0000(	FAM198B-/	1.186899	4.755046	1.76E-05
ENSG0000(	PRELID1	1.186761	8.748544	1.78E-09
ENSG0000(	RPL3	1.185994	5.830819	1.48E-06
ENSG0000(	KRT86	1.185923	1.409103	0.038985
ENSG0000(	PCDH7	1.185791	2.705088	0.001972
ENSG0000(	AL391422.4	1.185582	3.196627	0.000636
ENSG0000(	PGRMC1	1.185577	4.862544	1.37E-05
ENSG0000(	ASF1B	1.185363	1.315767	0.048332
ENSG0000(	DPYSL2	1.184542	4.567694	2.71E-05
ENSG0000(	C22orf34	1.183984	1.642492	0.022778
ENSG0000(	AC011603.	1.182913	7.254081	5.57E-08
ENSG0000(	PLXNB3	1.182782	3.838382	0.000145
ENSG0000(	MPZL1	1.182504	5.501033	3.15E-06
ENSG0000(	PRRG3	1.182453	1.766379	0.017125
ENSG0000(	CCDC71L	1.182383	5.332099	4.65E-06
ENSG0000(	AC073610.	1.180898	7.235989	5.81E-08
ENSG0000(	AC016747.	1.180557	3.444303	0.000359
ENSG0000(	DUSP6	1.180383	3.560298	0.000275
ENSG0000(	ARSJ	1.179037	1.467151	0.034107
ENSG0000(	AP001458.	1.178682	1.944634	0.01136
ENSG0000(	ARF3	1.178095	6.928722	1.18E-07
ENSG0000(	TNFAIP3	1.178085	7.109116	7.78E-08
ENSG0000(	PIWIL4	1.177872	3.460959	0.000346
ENSG0000(	AC004824.	1.177792	4.118877	7.61E-05
ENSG0000(	FJX1	1.177785	3.341868	0.000455
ENSG0000(	DSEL	1.176441	4.926321	1.18E-05
ENSG0000(	FSTL3	1.175995	2.595498	0.002538

ENSG0000(	AC093155.	1.175449	4.80409	1.57E-05
ENSG0000(	AC133644.	1.172712	5.530445	2.95E-06
ENSG0000(	ALDH18A1	1.172587	8.885473	1.30E-09
ENSG0000(	MANF	1.172519	9.349695	4.47E-10
ENSG0000(	CLEC2D	1.172132	4.810281	1.55E-05
ENSG0000(	WTIP	1.171629	5.042065	9.08E-06
ENSG0000(	GPR162	1.17139	4.004891	9.89E-05
ENSG0000(	LASP1	1.170895	8.140252	7.24E-09
ENSG0000(	FAM171B	1.170846	3.435389	0.000367
ENSG0000(	XYLT1	1.169751	2.824346	0.001498
ENSG0000(	AC096677.	1.168531	2.219914	0.006027
ENSG0000(	MELTF	1.168505	3.396456	0.000401
ENSG0000(	U62317.2	1.167435	4.15002	7.08E-05
ENSG0000(	PTK7	1.167381	5.275276	5.31E-06
ENSG0000(	RARA-AS1	1.167303	4.64642	2.26E-05
ENSG0000(	TUBA1B	1.166419	7.341976	4.55E-08
ENSG0000(	AC006453.	1.165313	2.914377	0.001218
ENSG0000(	S100A16	1.165248	7.452055	3.53E-08
ENSG0000(	PAPSS1	1.165102	7.561854	2.74E-08
ENSG0000(	PRDM8	1.164855	5.344915	4.52E-06
ENSG0000(	LOXL2	1.16472	4.671919	2.13E-05
ENSG0000(	PRKX	1.164566	8.635586	2.31E-09
ENSG0000(	DENND6B	1.164295	4.557278	2.77E-05
ENSG0000(	UBA6-AS1	1.164206	3.914434	0.000122
ENSG0000(	MICAL1	1.164019	7.752983	1.77E-08
ENSG0000(	PNP	1.16386	3.773006	0.000169
ENSG0000(	AC079313.	1.163247	3.918319	0.000121
ENSG0000(	PRKD1	1.162739	4.165205	6.84E-05
ENSG0000(	DYNC2H1	1.162575	3.690032	0.000204
ENSG0000(	CAP1	1.161986	8.083193	8.26E-09
ENSG0000(	TFPI	1.160663	6.594113	2.55E-07
ENSG0000(	GLIS2	1.160517	3.644185	0.000227
ENSG0000(	SP110	1.160355	5.937995	1.15E-06
ENSG0000(	VSTM2L	1.160264	1.856246	0.013924
ENSG0000(	PRICKLE2-A	1.159646	5.835351	1.46E-06
ENSG0000(	HSD11B1	1.159498	2.726023	0.001879
ENSG0000(	FKBP11	1.15925	4.462892	3.44E-05
ENSG0000(	SFT2D2	1.158974	8.005267	9.88E-09
ENSG0000(	FBXL2	1.158947	2.583844	0.002607
ENSG0000(	HPS3	1.158491	8.42492	3.76E-09
ENSG0000(	GBA	1.158384	5.616365	2.42E-06
ENSG0000(	PCDHGA12	1.158281	4.363807	4.33E-05
ENSG0000(	Z97200.1	1.158196	1.912873	0.012222
ENSG0000(	RASGRP4	1.157462	3.816131	0.000153
ENSG0000(	IKBIP	1.15705	4.64642	2.26E-05
ENSG0000(	WWTR1-AS	1.155538	10.2623	5.47E-11
ENSG0000(	CD81	1.155434	5.863338	1.37E-06

ENSG0000(	NID1	1.155243	2.6213	0.002392
ENSG0000(	ATP11C	1.154973	4.784093	1.64E-05
ENSG0000(	PCDHGA9	1.154614	4.368733	4.28E-05
ENSG0000(	ABHD15	1.154428	7.550333	2.82E-08
ENSG0000(	DZIP1L	1.154277	1.791753	0.016153
ENSG0000(	PIPOX	1.154082	1.508184	0.031032
ENSG0000(	SMIM10	1.15319	2.458201	0.003482
ENSG0000(	PEG3	1.152474	6.07477	8.42E-07
ENSG0000(	AC083799.	1.152183	2.490439	0.003233
ENSG0000(	RAI14	1.151572	3.593218	0.000255
ENSG0000(	ATL3	1.151244	3.992811	0.000102
ENSG0000(	AC140134.	1.15104	1.450011	0.03548
ENSG0000(	PDE1A	1.150978	3.725036	0.000188
ENSG0000(	FAM111A-I	1.150371	3.487862	0.000325
ENSG0000(	PCDHGB1	1.150337	4.314949	4.84E-05
ENSG0000(	STK10	1.149247	5.596955	2.53E-06
ENSG0000(	PCDHGB3	1.148444	4.355251	4.41E-05
ENSG0000(	PDIA3	1.148331	7.146334	7.14E-08
ENSG0000(	PCDHGA5	1.147775	4.322438	4.76E-05
ENSG0000(	TAGLN	1.147764	2.611703	0.002445
ENSG0000(	PCDHGB2	1.147174	4.379165	4.18E-05
ENSG0000(	USP51	1.147101	2.454342	0.003513
ENSG0000(	GLYCTK-AS	1.146743	2.346915	0.004499
ENSG0000(	PCDHGA8	1.146362	4.322045	4.76E-05
ENSG0000(	ZNF83	1.145674	4.173096	6.71E-05
ENSG0000(	CLMAT3	1.144976	4.240393	5.75E-05
ENSG0000(	SLC39A10	1.144438	7.173006	6.71E-08
ENSG0000(	PCDHGA7	1.144321	4.318753	4.80E-05
ENSG0000(	LRRC3	1.144094	2.041208	0.009095
ENSG0000(	SPARC	1.144053	4.836916	1.46E-05
ENSG0000(	PCDHGA4	1.143631	4.29409	5.08E-05
ENSG0000(	PLAGL1	1.143628	4.641937	2.28E-05
ENSG0000(	ATP10A	1.143524	2.840243	0.001445
ENSG0000(	LDAH	1.142624	2.758149	0.001745
ENSG0000(	AC011446.	1.142516	4.031941	9.29E-05
ENSG0000(	ZNF487	1.141406	2.533219	0.002929
ENSG0000(	PCDHGA10	1.141043	4.305284	4.95E-05
ENSG0000(	TXN	1.140623	9.851528	1.41E-10
ENSG0000(	PCDHGA3	1.139648	4.258216	5.52E-05
ENSG0000(	MMP25-AS	1.13918	3.660756	0.000218
ENSG0000(	ZNF503-AS	1.138812	2.549776	0.00282
ENSG0000(	SNX33	1.137443	4.197138	6.35E-05
ENSG0000(	SCIMP	1.136558	4.414627	3.85E-05
ENSG0000(	OLFM2	1.135023	1.794369	0.016056
ENSG0000(	PCDHGA11	1.134969	4.259458	5.50E-05
ENSG0000(	ASAP2	1.134749	3.49408	0.000321
ENSG0000(	BMP5	1.134228	3.381149	0.000416

ENSG0000(	MSN	1.134096	8.579735	2.63E-09
ENSG0000(	KLF4	1.13409	3.73883	0.000182
ENSG0000(	STK26	1.133775	2.176786	0.006656
ENSG0000(	PCDHGB5	1.133123	4.291715	5.11E-05
ENSG0000(	AC010619.	1.133048	8.185866	6.52E-09
ENSG0000(	ATP6AP2	1.132854	6.454482	3.51E-07
ENSG0000(	WDFY2	1.13203	5.148507	7.10E-06
ENSG0000(	PCDHGA6	1.131596	4.214641	6.10E-05
ENSG0000(	PCDHGB4	1.131239	4.240232	5.75E-05
ENSG0000(	PSRC1	1.130242	2.300142	0.00501
ENSG0000(	PCDHGC4	1.130219	4.235034	5.82E-05
ENSG0000(	PDIA4	1.129826	6.767055	1.71E-07
ENSG0000(	ZNF624	1.1293	2.688583	0.002048
ENSG0000(	COL21A1	1.128574	2.560912	0.002748
ENSG0000(	SNCG	1.128455	3.072969	0.000845
ENSG0000(	HMCN1	1.12727	2.550886	0.002813
ENSG0000(	PRKY	1.127141	5.942156	1.14E-06
ENSG0000(	AL355310.:	1.127083	4.594085	2.55E-05
ENSG0000(	TGIF1	1.126572	7.225104	5.96E-08
ENSG0000(	MIR3614	1.126545	5.764686	1.72E-06
ENSG0000(	AP1G2	1.126458	5.325291	4.73E-06
ENSG0000(	NFKB2	1.126038	5.506552	3.11E-06
ENSG0000(	PCDHGA2	1.125443	4.141888	7.21E-05
ENSG0000(	CPLX1	1.125196	3.030358	0.000932
ENSG0000(	AC010768.	1.12514	4.723517	1.89E-05
ENSG0000(	FLNA	1.122119	5.559934	2.75E-06
ENSG0000(	PCDHGC5	1.121331	4.171069	6.74E-05
ENSG0000(	SWAP70	1.121258	5.928449	1.18E-06
ENSG0000(	CALD1	1.121075	4.922591	1.20E-05
ENSG0000(	GIMAP4	1.120826	4.7064	1.97E-05
ENSG0000(	PRXL2B	1.120555	5.063797	8.63E-06
ENSG0000(	ZNF788P	1.119838	1.680556	0.020866
ENSG0000(	PDIA5	1.118376	4.418075	3.82E-05
ENSG0000(	ABHD2	1.117459	9.200737	6.30E-10
ENSG0000(	AL390728.(	1.117343	4.471785	3.37E-05
ENSG0000(	TSPAN2	1.11703	8.390644	4.07E-09
ENSG0000(	MFSD4A	1.116327	2.233505	0.005841
ENSG0000(	IGSF10	1.116321	1.971472	0.010679
ENSG0000(	C18orf54	1.116261	1.965	0.010839
ENSG0000(	ZEB2	1.116229	8.910776	1.23E-09
ENSG0000(	EFNA4	1.115115	2.398429	0.003995
ENSG0000(	SERPINE1	1.114916	2.629946	0.002345
ENSG0000(	UGCG	1.114454	5.983918	1.04E-06
ENSG0000(	TWSG1	1.114081	4.804202	1.57E-05
ENSG0000(	CAPN6	1.113701	2.23608	0.005807
ENSG0000(	PCDHGA1	1.113686	4.113611	7.70E-05
ENSG0000(	AC127502.	1.113366	1.866988	0.013584

ENSG0000(	AC073610.	1.113279	7.192276	6.42E-08
ENSG0000(	VDAC1P8	1.112939	5.107796	7.80E-06
ENSG0000(	CDA	1.112747	1.362256	0.043425
ENSG0000(	VASP	1.112023	5.345585	4.51E-06
ENSG0000(	PPP1R3G	1.111545	1.766858	0.017106
ENSG0000(	MIR1244-1	1.111452	5.209408	6.17E-06
ENSG0000(	HVCN1	1.11108	6.216798	6.07E-07
ENSG0000(	PCDHGB7	1.110925	4.198364	6.33E-05
ENSG0000(	GPR160	1.11086	2.049684	0.008919
ENSG0000(	STARD4	1.11061	4.011016	9.75E-05
ENSG0000(	ADAMTSL3	1.110459	3.418461	0.000382
ENSG0000(	TCTN2	1.109575	3.610142	0.000245
ENSG0000(	ZNF845	1.109277	5.911086	1.23E-06
ENSG0000(	PVT1	1.108814	3.327429	0.000471
ENSG0000(	TNFRSF1B	1.108215	4.780861	1.66E-05
ENSG0000(	BTG1	1.108052	8.223814	5.97E-09
ENSG0000(	RASSF5	1.107771	4.38804	4.09E-05
ENSG0000(	CDK6	1.107756	5.440879	3.62E-06
ENSG0000(	PHLDB2	1.105414	2.756323	0.001753
ENSG0000(	TAPBP	1.105264	9.096824	8.00E-10
ENSG0000(	ANGPTL2	1.104966	6.2279	5.92E-07
ENSG0000(	CREB3L2	1.104353	7.210645	6.16E-08
ENSG0000(	SLCO3A1	1.104136	5.30197	4.99E-06
ENSG0000(	ABLIM1	1.103679	5.999322	1.00E-06
ENSG0000(	LINC01011	1.103152	7.68271	2.08E-08
ENSG0000(	AC010542.	1.10268	6.856808	1.39E-07
ENSG0000(	AL035530.:	1.101538	4.718368	1.91E-05
ENSG0000(	OSMR	1.101505	3.901512	0.000125
ENSG0000(	ADGRL1	1.10064	5.671463	2.13E-06
ENSG0000(	AC008966.	1.100492	2.237115	0.005793
ENSG0000(	AC091564.	1.098651	1.77762	0.016687
ENSG0000(	PCDHGB6	1.098558	4.116266	7.65E-05
ENSG0000(	IGF2	1.097462	3.10324	0.000788
ENSG0000(	KDELR3	1.097342	3.362829	0.000434
ENSG0000(	AVPI1	1.096821	4.205827	6.23E-05
ENSG0000(	B3GALT4	1.096554	5.106655	7.82E-06
ENSG0000(	TMEM91	1.096216	2.949688	0.001123
ENSG0000(	AGTRAP	1.096148	8.757959	1.75E-09
ENSG0000(	PCDHB13	1.095509	1.979938	0.010473
ENSG0000(	PPT1	1.095485	7.407267	3.92E-08
ENSG0000(	CLEC2B	1.095291	5.578928	2.64E-06
ENSG0000(	RBP1	1.09503	2.07833	0.00835
ENSG0000(	ZC2HC1A	1.094701	3.460959	0.000346
ENSG0000(	VILL	1.094002	3.573223	0.000267
ENSG0000(	NEK11	1.093946	6.067183	8.57E-07
ENSG0000(	ADIRF-AS1	1.093565	2.598979	0.002518
ENSG0000(	IGF2	1.093425	3.09955	0.000795

ENSG0000(	AC010655.	1.093226	1.848589	0.014171
ENSG0000(	SH3D21	1.093219	3.380223	0.000417
ENSG0000(	STMN1	1.093216	6.299231	5.02E-07
ENSG0000(	AL135999.:	1.093107	4.676157	2.11E-05
ENSG0000(	PRICKLE1	1.092773	4.971825	1.07E-05
ENSG0000(	RHPN1	1.092689	1.950488	0.011208
ENSG0000(	MAN1B1-D	1.092284	5.37221	4.24E-06
ENSG0000(	CDCP1	1.092177	1.362645	0.043387
ENSG0000(	AF131215.:	1.091822	1.512892	0.030698
ENSG0000(	TMEM243	1.091501	3.765657	0.000172
ENSG0000(	PRRG4	1.090424	1.934564	0.011626
ENSG0000(	KDELC2	1.090165	3.5093	0.00031
ENSG0000(	COX6A1	1.090031	5.551762	2.81E-06
ENSG0000(	CD82	1.088627	5.420118	3.80E-06
ENSG0000(	USP18	1.088366	4.329171	4.69E-05
ENSG0000(	TGFB3	1.087029	4.814513	1.53E-05
ENSG0000(	ADGRD1	1.08658	1.991649	0.010194
ENSG0000(	AC008945.	1.08596	5.270618	5.36E-06
ENSG0000(	AC090197.	1.085932	2.249274	0.005633
ENSG0000(	IGFBP3	1.085816	1.77365	0.01684
ENSG0000(	SYTL4	1.085773	2.46258	0.003447
ENSG0000(	AC005332.	1.085609	2.440173	0.003629
ENSG0000(	HILPDA	1.085393	2.486087	0.003265
ENSG0000(	MAP1B	1.084935	2.954902	0.001109
ENSG0000(	MANSC1	1.084502	2.588319	0.00258
ENSG0000(	GJC2	1.083423	1.641914	0.022808
ENSG0000(	DYNLL1	1.083342	8.2035	6.26E-09
ENSG0000(	AC027097.	1.083255	3.363099	0.000433
ENSG0000(	CNRIP1	1.082684	4.515955	3.05E-05
ENSG0000(	PLK3	1.08236	4.42537	3.76E-05
ENSG0000(	TPRG1	1.082288	2.724913	0.001884
ENSG0000(	FZD1	1.081488	2.352296	0.004443
ENSG0000(	EBF2	1.081467	3.828543	0.000148
ENSG0000(	STK32B	1.081375	2.04194	0.009079
ENSG0000(	PLCG2	1.081235	5.101305	7.92E-06
ENSG0000(	IL32	1.080882	1.793661	0.016082
ENSG0000(	AC079601.	1.080796	4.880777	1.32E-05
ENSG0000(	SLC16A2	1.079587	5.436493	3.66E-06
ENSG0000(	NOTCH2NL	1.079472	5.451304	3.54E-06
ENSG0000(	TSHZ3	1.078908	5.973714	1.06E-06
ENSG0000(	AC015712.	1.078548	3.107294	0.000781
ENSG0000(	ASIC1	1.077473	2.721674	0.001898
ENSG0000(	CASQ2	1.076738	1.929974	0.01175
ENSG0000(	ENO2	1.076131	2.620152	0.002398
ENSG0000(	HTR7P1	1.075533	2.264723	0.005436
ENSG0000(	RTN1	1.07549	2.30076	0.005003
ENSG0000(	LRCH1	1.075457	5.317109	4.82E-06

ENSG0000(	TES	1.075048	4.527979	2.96E-05
ENSG0000(	REEP4	1.07494	4.818552	1.52E-05
ENSG0000(	IER2	1.074128	4.323593	4.75E-05
ENSG0000(	ZNF430	1.073956	6.383705	4.13E-07
ENSG0000(	TSPO	1.073487	5.185715	6.52E-06
ENSG0000(	MBOAT2	1.073009	5.385355	4.12E-06
ENSG0000(	TKT	1.072766	3.721337	0.00019
ENSG0000(	CCDC40	1.072704	5.092394	8.08E-06
ENSG0000(	ANLN	1.072296	2.024992	0.009441
ENSG0000(	AC007068.	1.071335	3.615806	0.000242
ENSG0000(	CAPN5	1.070423	4.486178	3.26E-05
ENSG0000(	CRMP1	1.070121	2.795465	0.001602
ENSG0000(	Z84488.2	1.069541	3.817668	0.000152
ENSG0000(	AC135050.	1.06872	4.324267	4.74E-05
ENSG0000(	IL1R1	1.068555	4.387393	4.10E-05
ENSG0000(	CCND1	1.066572	3.699307	0.0002
ENSG0000(	AC104134.	1.066178	3.358253	0.000438
ENSG0000(	TTC21A	1.065522	2.20676	0.006212
ENSG0000(	TMEM67	1.064509	2.228332	0.005911
ENSG0000(	FES	1.064316	4.2241	5.97E-05
ENSG0000(	FAM198B	1.063963	4.032069	9.29E-05
ENSG0000(	AC239799.	1.063773	5.256261	5.54E-06
ENSG0000(	VKORC1	1.063675	4.275871	5.30E-05
ENSG0000(	ZFP36L2	1.062668	8.532646	2.93E-09
ENSG0000(	AL355075.:	1.062418	1.674431	0.021163
ENSG0000(	SNORA63	1.062276	2.003562	0.009918
ENSG0000(	CD81-AS1	1.061849	6.369537	4.27E-07
ENSG0000(	SUMF1	1.061842	3.685571	0.000206
ENSG0000(	ALDH1A3	1.061766	2.855873	0.001394
ENSG0000(	NMNAT2	1.061339	2.035426	0.009217
ENSG0000(	TMEM44-A	1.060982	4.312826	4.87E-05
ENSG0000(	RGMB-AS1	1.060935	1.85928	0.013827
ENSG0000(	ENPP1	1.060853	2.173761	0.006703
ENSG0000(	DPYD	1.060498	5.104407	7.86E-06
ENSG0000(	MGAT2	1.060467	11.46538	3.42E-12
ENSG0000(	LMNTD2	1.059867	1.415565	0.038409
ENSG0000(	ELOVL1	1.059755	7.843539	1.43E-08
ENSG0000(	FZD6	1.059626	4.495016	3.20E-05
ENSG0000(	CRACR2A	1.059546	2.630188	0.002343
ENSG0000(	ETV5	1.058623	3.776612	0.000167
ENSG0000(	KLHL2	1.058545	4.149261	7.09E-05
ENSG0000(	GALNT7	1.058434	5.876891	1.33E-06
ENSG0000(	GGT5	1.058305	5.299756	5.01E-06
ENSG0000(	CD47	1.058179	9.879207	1.32E-10
ENSG0000(	SEMA3C	1.05779	3.663397	0.000217
ENSG0000(	ABCA8	1.057139	3.973409	0.000106
ENSG0000(	EML2	1.055771	6.183909	6.55E-07

ENSG0000(	AL139099.:	1.055233	11.26316	5.46E-12
ENSG0000(	NIPSNAP1	1.054789	10.35991	4.37E-11
ENSG0000(	ZNF286A	1.054781	13.258	5.52E-14
ENSG0000(	ZNF625-ZN	1.054677	4.283922	5.20E-05
ENSG0000(	BEX3	1.054573	4.115478	7.67E-05
ENSG0000(	ZNF217	1.054488	9.547807	2.83E-10
ENSG0000(	PLK2	1.054481	6.070775	8.50E-07
ENSG0000(	SYTL2	1.054446	4.375282	4.21E-05
ENSG0000(	DUSP18	1.054013	4.494605	3.20E-05
ENSG0000(	OSTC	1.053688	6.670989	2.13E-07
ENSG0000(	HOTAIRM1	1.053243	3.444219	0.00036
ENSG0000(	MAN1C1	1.052658	2.873449	0.001338
ENSG0000(	ZNF600	1.051904	4.292597	5.10E-05
ENSG0000(	AC012181.	1.051617	1.942691	0.011411
ENSG0000(	MIR4435-2	1.050323	5.372109	4.25E-06
ENSG0000(	VWA1	1.049991	7.743162	1.81E-08
ENSG0000(	SCO2	1.049724	3.573037	0.000267
ENSG0000(	SPC24	1.049676	2.008546	0.009805
ENSG0000(	NRM	1.049593	5.388755	4.09E-06
ENSG0000(	ITGB4	1.049102	3.341543	0.000455
ENSG0000(	SEMA3A	1.049063	1.574219	0.026655
ENSG0000(	FAIM2	1.049054	1.399964	0.039814
ENSG0000(	RF02217	1.049041	4.480604	3.31E-05
ENSG0000(	AC024909.	1.048854	2.926857	0.001183
ENSG0000(	SLC36A1	1.048686	6.442372	3.61E-07
ENSG0000(	GLB1L	1.048614	2.505133	0.003125
ENSG0000(	B9D2	1.048442	1.838462	0.014506
ENSG0000(	SDCBP	1.048408	6.444656	3.59E-07
ENSG0000(	EPB41L2	1.048334	4.408438	3.90E-05
ENSG0000(	NHS	1.048258	1.758108	0.017454
ENSG0000(	ALCAM	1.048104	3.420926	0.000379
ENSG0000(	DNMT1	1.047766	13.13146	7.39E-14
ENSG0000(	ANXA5	1.047735	4.734691	1.84E-05
ENSG0000(	AC012676.	1.047092	3.22084	0.000601
ENSG0000(	AC018816.	1.046691	2.610521	0.002452
ENSG0000(	SLC27A3	1.046482	3.829254	0.000148
ENSG0000(	UBAC2-AS1	1.045385	2.180701	0.006596
ENSG0000(	MEX3D	1.043921	5.815182	1.53E-06
ENSG0000(	LAMA4	1.043088	3.641228	0.000228
ENSG0000(	AL359878.:	1.042825	1.305873	0.049446
ENSG0000(	ADCY3	1.04274	5.303052	4.98E-06
ENSG0000(	TC2N	1.042649	4.194614	6.39E-05
ENSG0000(	MIR3916	1.042621	1.533694	0.029262
ENSG0000(	EDEM2	1.040782	4.182833	6.56E-05
ENSG0000(	ITPR1	1.040368	5.561575	2.74E-06
ENSG0000(	PRRT3	1.040353	6.173726	6.70E-07
ENSG0000(	TENM4	1.040198	3.915052	0.000122

ENSG0000(	ARHGAP28	1.039896	3.457538	0.000349
ENSG0000(	AC020913.	1.039604	2.875673	0.001331
ENSG0000(	EFEMP2	1.039313	5.233373	5.84E-06
ENSG0000(	SMAP2	1.038031	6.255801	5.55E-07
ENSG0000(	AL158206.:	1.037888	1.474245	0.033555
ENSG0000(	ODF2L	1.037717	4.126687	7.47E-05
ENSG0000(	GPSM3	1.037371	3.583639	0.000261
ENSG0000(	COL9A3	1.036884	1.417501	0.038238
ENSG0000(	KCNQ4	1.036653	2.137352	0.007289
ENSG0000(	SIGIRR	1.036647	5.199215	6.32E-06
ENSG0000(	GORAB	1.036579	4.675728	2.11E-05
ENSG0000(	SPOCK2	1.036528	4.039697	9.13E-05
ENSG0000(	CKLF-CMTI	1.036524	7.200304	6.31E-08
ENSG0000(	EIF2AK3	1.03613	6.180294	6.60E-07
ENSG0000(	TGFB2-OT1	1.035251	3.853805	0.00014
ENSG0000(	CKLF	1.035027	6.884331	1.31E-07
ENSG0000(	FAM83H	1.034414	1.771339	0.01693
ENSG0000(	SNORD55	1.034261	1.908134	0.012356
ENSG0000(	SDF2L1	1.032782	5.815956	1.53E-06
ENSG0000(	LDHB	1.031996	2.681463	0.002082
ENSG0000(	F3	1.031866	3.284755	0.000519
ENSG0000(	FAM57A	1.030965	3.71326	0.000194
ENSG0000(	VSIR	1.030933	3.923915	0.000119
ENSG0000(	IGFBP7-AS:	1.030099	4.392014	4.05E-05
ENSG0000(	SH3BP4	1.029724	7.501962	3.15E-08
ENSG0000(	ANKRD13D	1.029626	4.258947	5.51E-05
ENSG0000(	CATSPERG	1.029179	1.405301	0.039328
ENSG0000(	LRRC25	1.028668	2.340142	0.004569
ENSG0000(	PRKACB	1.028543	7.450782	3.54E-08
ENSG0000(	PPIA	1.028349	6.647064	2.25E-07
ENSG0000(	CYP2T1P	1.028341	2.503191	0.003139
ENSG0000(	CIT	1.028341	3.076046	0.000839
ENSG0000(	DPY19L1	1.028133	4.675166	2.11E-05
ENSG0000(	ERF	1.027762	5.868625	1.35E-06
ENSG0000(	ZNRD1ASP	1.027583	4.301908	4.99E-05
ENSG0000(	SLC17A7	1.027272	3.774337	0.000168
ENSG0000(	REC8	1.027161	2.655275	0.002212
ENSG0000(	WDR78	1.026953	1.732369	0.01852
ENSG0000(	CRABP2	1.026732	2.708856	0.001955
ENSG0000(	SLFN12	1.02591	3.13347	0.000735
ENSG0000(	SLC4A3	1.025735	2.905348	0.001244
ENSG0000(	PFKL	1.025171	9.432808	3.69E-10
ENSG0000(	AC010197.	1.024795	2.653921	0.002219
ENSG0000(	ZC3HAV1L	1.024411	1.399964	0.039814
ENSG0000(	PLOD2	1.024281	3.291048	0.000512
ENSG0000(	MAP9	1.023501	3.251958	0.00056
ENSG0000(	ARPC3	1.023238	6.797204	1.60E-07

ENSG0000(	MYO5B	1.023142	2.4304	0.003712
ENSG0000(	ARMC9	1.022815	3.791627	0.000162
ENSG0000(	TRAM1	1.022776	5.980815	1.05E-06
ENSG0000(	CLVS1	1.022153	2.197727	0.006343
ENSG0000(	AP001972.	1.021834	3.683808	0.000207
ENSG0000(	SEC61B	1.021725	4.259311	5.50E-05
ENSG0000(	ZC4H2	1.021544	4.232312	5.86E-05
ENSG0000(	LRRN3	1.021497	2.825295	0.001495
ENSG0000(	B3GNT9	1.021427	7.269704	5.37E-08
ENSG0000(	UCP2	1.021216	5.398797	3.99E-06
ENSG0000(	AP001528.	1.020465	2.416904	0.003829
ENSG0000(	TIFA	1.020037	4.292597	5.10E-05
ENSG0000(	HRAT92	1.020031	1.300045	0.050113
ENSG0000(	RALGPS2	1.019991	6.744124	1.80E-07
ENSG0000(	TWF1	1.019663	4.132555	7.37E-05
ENSG0000(	FUT8	1.019254	4.674903	2.11E-05
ENSG0000(	NUPR1	1.018979	3.482332	0.000329
ENSG0000(	FAM172A	1.018939	6.216798	6.07E-07
ENSG0000(	ZDHHC13	1.018365	7.364422	4.32E-08
ENSG0000(	ARAP1-AS1	1.018201	4.345855	4.51E-05
ENSG0000(	SMIM14	1.018142	3.153929	0.000702
ENSG0000(	SPATA6L	1.018002	1.499293	0.031674
ENSG0000(	PIM1	1.017491	3.915348	0.000122
ENSG0000(	MELTF-AS1	1.017381	2.292704	0.005097
ENSG0000(	AL354813.:	1.016876	2.146868	0.007131
ENSG0000(	ZNF738	1.016713	4.060935	8.69E-05
ENSG0000(	BMPER	1.015952	3.444167	0.00036
ENSG0000(	ORAI3	1.015903	4.812518	1.54E-05
ENSG0000(	TGFB2	1.015755	3.374398	0.000422
ENSG0000(	CHST7	1.015725	4.977016	1.05E-05
ENSG0000(	RF02216	1.01497	4.869555	1.35E-05
ENSG0000(	CNTRL	1.01468	4.428697	3.73E-05
ENSG0000(	ZNF544	1.014678	5.573961	2.67E-06
ENSG0000(	B3GNT2	1.014586	4.825591	1.49E-05
ENSG0000(	PFKP	1.014496	4.385406	4.12E-05
ENSG0000(	PLD2	1.01428	4.658786	2.19E-05
ENSG0000(	ARL13B	1.012979	4.380711	4.16E-05
ENSG0000(	DISC1	1.012781	3.997695	0.000101
ENSG0000(	MOV10	1.012329	5.211484	6.14E-06
ENSG0000(	SOGA1	1.012321	5.862255	1.37E-06
ENSG0000(	RAB43	1.012039	4.675006	2.11E-05
ENSG0000(	CDC42EP1	1.011906	4.639801	2.29E-05
ENSG0000(	ANKRD44	1.011892	5.103374	7.88E-06
ENSG0000(	MIR675	1.010399	2.062119	0.008667
ENSG0000(	AC025164.	1.010228	5.332858	4.65E-06
ENSG0000(	LAT2	1.010159	3.113595	0.00077
ENSG0000(	MNS1	1.009946	2.686533	0.002058

ENSG0000(	ROM1	1.009134	3.739483	0.000182
ENSG0000(	IGSF3	1.0088	3.50447	0.000313
ENSG0000(	PREX1	1.008058	5.166662	6.81E-06
ENSG0000(	AL451164.:	1.007581	3.178969	0.000662
ENSG0000(	AL691442.:	1.007427	1.398605	0.039939
ENSG0000(	POC5	1.007401	6.443991	3.60E-07
ENSG0000(	AC012073.	1.005459	2.758149	0.001745
ENSG0000(	ZSWIM4	1.004057	6.133572	7.35E-07
ENSG0000(	CASP7	1.003787	10.42526	3.76E-11
ENSG0000(	TGFB3-AS1	1.003741	3.193324	0.000641
ENSG0000(	RP2	1.003661	6.784439	1.64E-07
ENSG0000(	LAYN	1.002981	4.318475	4.80E-05
ENSG0000(	PGM5-AS1	1.00272	1.513029	0.030688
ENSG0000(	LDLRAD3	1.002423	5.852974	1.40E-06
ENSG0000(	GNRH1	1.00223	1.623509	0.023795
ENSG0000(	FCGRT	1.001465	5.002397	9.94E-06
ENSG0000(	NQO1	1.001362	3.188875	0.000647
ENSG0000(	IL27RA	1.001055	2.797818	0.001593
ENSG0000(	RIPK3	1.00105	2.675395	0.002112
ENSG0000(	BCAS4	1.000546	2.078212	0.008352
ENSG0000(	SLC30A1	0.999463	4.289122	5.14E-05
ENSG0000(	AC010422.	0.999304	4.794248	1.61E-05
ENSG0000(	FKBP7	0.998687	3.896678	0.000127
ENSG0000(	MGAT3	0.998335	2.373044	0.004236
ENSG0000(	LDOC1	0.998094	3.322494	0.000476
ENSG0000(	ANKRD29	0.997868	3.217244	0.000606
ENSG0000(	AC016876.	0.997768	5.073715	8.44E-06
ENSG0000(	MIF	0.997677	12.3252	4.73E-13
ENSG0000(	NOSTRIN	0.997534	2.510273	0.003088
ENSG0000(	SCAMP1-A!	0.997503	2.69838	0.002003
ENSG0000(	SESTD1	0.997469	3.611486	0.000245
ENSG0000(	GALNT10	0.996642	4.036914	9.19E-05
ENSG0000(	ARRB1	0.995597	5.257882	5.52E-06
ENSG0000(	COBLL1	0.995159	2.593245	0.002551
ENSG0000(	LYNX1	0.995004	3.870006	0.000135
ENSG0000(	MIF-AS1	0.994613	12.66913	2.14E-13
ENSG0000(	G6PD	0.993938	4.881861	1.31E-05
ENSG0000(	LPIN3	0.993711	2.248947	0.005637
ENSG0000(	ADNP2	0.993703	7.865211	1.36E-08
ENSG0000(	RGS16	0.992999	2.036344	0.009197
ENSG0000(	MYL9	0.992724	4.512377	3.07E-05
ENSG0000(	ZWINT	0.991902	1.930622	0.011732
ENSG0000(	CHIC2	0.991473	6.550517	2.82E-07
ENSG0000(	MIR4680	0.991362	3.671117	0.000213
ENSG0000(	FLI1	0.990587	6.85148	1.41E-07
ENSG0000(	PLPP3	0.990128	3.506137	0.000312
ENSG0000(	NIPAL2	0.989991	3.249558	0.000563

ENSG0000(	ZNF28	0.989484	3.542515	0.000287
ENSG0000(	AC135050.	0.989367	3.806814	0.000156
ENSG0000(	PDGFRL	0.989019	2.801033	0.001581
ENSG0000(	ADGRA2	0.988842	4.38804	4.09E-05
ENSG0000(	FHDC1	0.987297	2.390875	0.004066
ENSG0000(	TMEM123	0.987194	8.185866	6.52E-09
ENSG0000(	AC080013.	0.98716	1.443819	0.03599
ENSG0000(	AP001972.	0.98712	2.022232	0.009501
ENSG0000(	CNTN1	0.986898	1.81275	0.01539
ENSG0000(	TUSC3	0.986846	5.569905	2.69E-06
ENSG0000(	ARHGEF10	0.985484	2.498962	0.00317
ENSG0000(	TSTD1	0.985005	3.899791	0.000126
ENSG0000(	CA5B	0.984472	2.796141	0.001599
ENSG0000(	RAB27A	0.984104	7.83334	1.47E-08
ENSG0000(	BAX	0.98389	6.917048	1.21E-07
ENSG0000(	AC007728.	0.983863	2.456455	0.003496
ENSG0000(	PPIAP22	0.983543	4.600956	2.51E-05
ENSG0000(	AFAP1	0.983525	5.00038	9.99E-06
ENSG0000(	GNAI2	0.983331	7.286542	5.17E-08
ENSG0000(	COLGALT1	0.982812	6.08729	8.18E-07
ENSG0000(	CLIP2	0.981367	3.07256	0.000846
ENSG0000(	AL033528.:	0.981276	3.26112	0.000548
ENSG0000(	ZFP36L1	0.980962	8.761691	1.73E-09
ENSG0000(	SEC23B	0.980718	6.083274	8.26E-07
ENSG0000(	RCN1P2	0.977552	3.182818	0.000656
ENSG0000(	DNALI1	0.977236	4.557702	2.77E-05
ENSG0000(	C3orf58	0.976834	12.04681	8.98E-13
ENSG0000(	DHRS1	0.976403	4.761269	1.73E-05
ENSG0000(	GALNS	0.976395	6.951706	1.12E-07
ENSG0000(	ARHGEF2	0.975002	7.012309	9.72E-08
ENSG0000(	C2orf27A	0.974784	2.451887	0.003533
ENSG0000(	SYT11	0.974473	4.9972	1.01E-05
ENSG0000(	SEC11C	0.973787	5.982616	1.04E-06
ENSG0000(	TP53I11	0.972387	5.829455	1.48E-06
ENSG0000(	KIF11	0.971962	1.703761	0.019781
ENSG0000(	CLDN15	0.971922	2.53408	0.002924
ENSG0000(	XXYLT1	0.971902	3.485629	0.000327
ENSG0000(	MAN2B1	0.971791	5.761347	1.73E-06
ENSG0000(	IL15RA	0.971701	6.299593	5.02E-07
ENSG0000(	TMEM198I	0.971444	3.041627	0.000909
ENSG0000(	CLDN4	0.971262	1.720368	0.019038
ENSG0000(	NBPF14	0.970507	4.385676	4.11E-05
ENSG0000(	MEGF10	0.970024	1.995689	0.0101
ENSG0000(	IL1R1-AS1	0.969948	2.350082	0.004466
ENSG0000(	DOCK1	0.969652	5.368937	4.28E-06
ENSG0000(	AL356481.:	0.969473	3.551987	0.000281
ENSG0000(	CPT1A	0.969264	3.469532	0.000339

ENSG0000(	EFHC1	0.968838	4.302465	4.98E-05
ENSG0000(	ZNF8	0.967339	7.432333	3.70E-08
ENSG0000(	RNU1-125F	0.96677	1.344965	0.045189
ENSG0000(	RRN3P1	0.966148	3.68687	0.000206
ENSG0000(	ZNF85	0.965338	2.093695	0.008059
ENSG0000(	SLC35F2	0.965271	3.925131	0.000119
ENSG0000(	CHRD	0.965054	2.801016	0.001581
ENSG0000(	AL031320.:	0.964317	4.769201	1.70E-05
ENSG0000(	SOX4	0.964037	4.184799	6.53E-05
ENSG0000(	AC022167.	0.963903	5.989783	1.02E-06
ENSG0000(	FLVCR2	0.963758	6.098846	7.96E-07
ENSG0000(	ATXN7L1	0.963553	6.41182	3.87E-07
ENSG0000(	DDX58	0.963367	4.305284	4.95E-05
ENSG0000(	CERS4	0.96232	2.037703	0.009168
ENSG0000(	PARP4	0.962314	10.18121	6.59E-11
ENSG0000(	CDC14B	0.961801	3.849276	0.000141
ENSG0000(	C11orf96	0.961306	2.022595	0.009493
ENSG0000(	NAAA	0.961181	3.146321	0.000714
ENSG0000(	SLC16A8	0.960802	1.532206	0.029363
ENSG0000(	SLC16A14	0.960301	1.638303	0.022998
ENSG0000(	ATP1B3	0.960232	9.514908	3.06E-10
ENSG0000(	C11orf80	0.959843	3.567602	0.000271
ENSG0000(	AP000787.	0.95979	2.802267	0.001577
ENSG0000(	RCC2	0.959184	5.826552	1.49E-06
ENSG0000(	EML3	0.958661	4.019929	9.55E-05
ENSG0000(	ARAP1	0.958633	7.629658	2.35E-08
ENSG0000(	TBC1D10A	0.957916	3.3731	0.000424
ENSG0000(	FAM129B	0.9579	6.271193	5.36E-07
ENSG0000(	C1orf74	0.957856	3.856144	0.000139
ENSG0000(	NR1H3	0.957805	3.717773	0.000192
ENSG0000(	AC004656.	0.957639	3.082035	0.000828
ENSG0000(	MFNG	0.957576	4.187126	6.50E-05
ENSG0000(	SMOC2	0.957423	3.251097	0.000561
ENSG0000(	8-Sep	0.957316	5.660756	2.18E-06
ENSG0000(	IGFBP7	0.957254	4.282352	5.22E-05
ENSG0000(	KLF5	0.956673	2.535519	0.002914
ENSG0000(	FUT4	0.956562	3.170278	0.000676
ENSG0000(	SNX18	0.956396	8.677971	2.10E-09
ENSG0000(	NCAM2	0.956353	1.731362	0.018563
ENSG0000(	ZMAT3	0.956045	9.169323	6.77E-10
ENSG0000(	AC068385.	0.955875	1.641296	0.02284
ENSG0000(	TP73	0.955847	1.673575	0.021204
ENSG0000(	TNFSF12-TI	0.955807	3.994882	0.000101
ENSG0000(	SNTB2	0.955771	3.789316	0.000162
ENSG0000(	MMP25	0.955699	2.201897	0.006282
ENSG0000(	AL359094.:	0.955167	4.775858	1.68E-05
ENSG0000(	SH3KBP1	0.954966	6.394788	4.03E-07

ENSG0000(	RIN2	0.953344	3.139153	0.000726
ENSG0000(	PGAM1	0.953307	10.36984	4.27E-11
ENSG0000(	AKR1C1	0.953073	2.475092	0.003349
ENSG0000(	KCNAB1	0.952994	2.022305	0.009499
ENSG0000(	PTBP3	0.952417	6.193535	6.40E-07
ENSG0000(	PAQR7	0.951924	6.364324	4.32E-07
ENSG0000(	CD34	0.951664	2.795231	0.001602
ENSG0000(	BMF	0.951432	4.260051	5.49E-05
ENSG0000(	GATA3	0.951325	1.766145	0.017134
ENSG0000(	LMNB1	0.951085	2.360902	0.004356
ENSG0000(	ZNF559-ZN	0.950612	2.916548	0.001212
ENSG0000(	AC020913.	0.95049	3.698884	0.0002
ENSG0000(	FANCD2	0.950342	2.327566	0.004704
ENSG0000(	CASP3	0.950142	6.376711	4.20E-07
ENSG0000(	ARMC2	0.950089	2.795543	0.001601
ENSG0000(	APP	0.949273	5.424155	3.77E-06
ENSG0000(	RIMS3	0.949083	2.169882	0.006763
ENSG0000(	AP3S1	0.949023	4.39717	4.01E-05
ENSG0000(	UGGT2	0.948769	3.55538	0.000278
ENSG0000(	SDK1	0.948015	2.102151	0.007904
ENSG0000(	ADAMTS10	0.947305	3.344108	0.000453
ENSG0000(	AL358472.:	0.947096	1.355794	0.044076
ENSG0000(	TCTA	0.946693	11.63118	2.34E-12
ENSG0000(	SLC2A6	0.946621	1.627965	0.023552
ENSG0000(	PRRT3-AS1	0.946342	3.097972	0.000798
ENSG0000(	GCA	0.945769	3.584156	0.000261
ENSG0000(	CORO1C	0.945269	5.704465	1.97E-06
ENSG0000(	TNFRSF10A	0.944994	3.69754	0.000201
ENSG0000(	ACTG1	0.944793	5.058345	8.74E-06
ENSG0000(	MYDGF	0.944204	4.336372	4.61E-05
ENSG0000(	TGFB1	0.943053	8.708619	1.96E-09
ENSG0000(	AC009950.	0.942564	3.013544	0.000969
ENSG0000(	CELF6	0.942153	2.266114	0.005419
ENSG0000(	AC027601.	0.942135	3.074487	0.000842
ENSG0000(	TM4SF19-T	0.94176	1.57062	0.026877
ENSG0000(	CAB39L	0.941188	1.805541	0.015648
ENSG0000(	PGD	0.940222	6.69752	2.01E-07
ENSG0000(	ZNF837	0.939931	2.108601	0.007788
ENSG0000(	PABPC1	0.939236	5.056128	8.79E-06
ENSG0000(	CCNG2	0.938842	3.502048	0.000315
ENSG0000(	ADGRB2	0.93881	1.941559	0.01144
ENSG0000(	NAGLU	0.938221	4.620509	2.40E-05
ENSG0000(	PTPN9	0.938062	4.634269	2.32E-05
ENSG0000(	STARD9	0.937207	2.412678	0.003867
ENSG0000(	HAUS1	0.937108	5.272634	5.34E-06
ENSG0000(	TAF1B	0.936343	3.606553	0.000247
ENSG0000(	PML	0.935887	6.110482	7.75E-07

ENSG0000(	MEX3B	0.935447	3.8289	0.000148
ENSG0000(	PTMS	0.935382	4.858955	1.38E-05
ENSG0000(	DCDC2B	0.93532	1.538533	0.028938
ENSG0000(	PLD1	0.933597	3.128349	0.000744
ENSG0000(	ITGA5	0.933509	4.485652	3.27E-05
ENSG0000(	HAS2	0.932915	1.67038	0.021361
ENSG0000(	SQLE	0.931196	2.672834	0.002124
ENSG0000(	NAV2-AS1	0.931104	1.80623	0.015623
ENSG0000(	AC091564.	0.930903	2.505062	0.003126
ENSG0000(	AL390728.	0.93024	3.703758	0.000198
ENSG0000(	GLI2	0.930196	3.748716	0.000178
ENSG0000(	SOWAHC	0.930049	3.673716	0.000212
ENSG0000(	CKS1B	0.929943	4.605424	2.48E-05
ENSG0000(	ARHGAP27	0.929851	3.72108	0.00019
ENSG0000(	TMCO4	0.92937	4.752027	1.77E-05
ENSG0000(	MT2A	0.929259	3.262914	0.000546
ENSG0000(	H19	0.928238	1.984471	0.010364
ENSG0000(	EZR	0.927844	5.052642	8.86E-06
ENSG0000(	ACCS	0.927109	2.292515	0.005099
ENSG0000(	PTMA	0.927106	5.173286	6.71E-06
ENSG0000(	AL121845.	0.926973	1.986029	0.010327
ENSG0000(	SYDE1	0.926965	2.824919	0.001497
ENSG0000(	LARP6	0.926455	5.144516	7.17E-06
ENSG0000(	MYO9B	0.926432	5.163849	6.86E-06
ENSG0000(	PEX11G	0.926252	4.272862	5.34E-05
ENSG0000(	UNC119	0.925808	5.008481	9.81E-06
ENSG0000(	PCDHB2	0.925781	2.523742	0.002994
ENSG0000(	TMEM216	0.925352	3.946674	0.000113
ENSG0000(	HOXB6	0.925309	1.431789	0.037001
ENSG0000(	CPM	0.925089	4.204783	6.24E-05
ENSG0000(	SH2B3	0.924729	6.183909	6.55E-07
ENSG0000(	ESRRG	0.924421	1.352765	0.044385
ENSG0000(	AC109992.	0.923764	1.371715	0.04249
ENSG0000(	CSK	0.923733	4.9455	1.13E-05
ENSG0000(	AC007192.	0.923377	3.401604	0.000397
ENSG0000(	AK4	0.923362	2.376797	0.0042
ENSG0000(	TIMP3	0.923031	2.957964	0.001102
ENSG0000(	HSP90B1	0.922519	6.202785	6.27E-07
ENSG0000(	AC092115.	0.922475	1.833379	0.014676
ENSG0000(	SPSB4	0.922467	2.811168	0.001545
ENSG0000(	TPP1	0.92237	9.85421	1.40E-10
ENSG0000(	ZNF101	0.921575	4.641697	2.28E-05
ENSG0000(	BICD1	0.92112	4.454018	3.52E-05
ENSG0000(	LINC00294	0.921077	3.906876	0.000124
ENSG0000(	TRPV3	0.92076	1.857582	0.013881
ENSG0000(	RF01972	0.920074	1.950916	0.011197
ENSG0000(	ZNF763	0.919952	3.623048	0.000238

ENSG0000(	FRMD8	0.918997	6.580543	2.63E-07
ENSG0000(	AC005324.	0.918878	3.861172	0.000138
ENSG0000(	TMEM198	0.918634	4.462538	3.45E-05
ENSG0000(	EEF1AKMT	0.918598	3.608597	0.000246
ENSG0000(	TDP1	0.918245	3.412718	0.000387
ENSG0000(	CDCA3	0.918077	3.399478	0.000399
ENSG0000(	CCDC120	0.916808	3.85379	0.00014
ENSG0000(	CHPF2	0.91652	7.742924	1.81E-08
ENSG0000(	VMP1	0.915749	6.801477	1.58E-07
ENSG0000(	RRAS	0.914835	5.427213	3.74E-06
ENSG0000(	TRIM56	0.914753	6.064354	8.62E-07
ENSG0000(	PRMT6	0.913665	5.751571	1.77E-06
ENSG0000(	SLC38A10	0.913175	3.990827	0.000102
ENSG0000(	FAM98C	0.913115	4.908132	1.24E-05
ENSG0000(	MBNL3	0.912929	2.218371	0.006048
ENSG0000(	AC006027.	0.912883	2.880446	0.001317
ENSG0000(	CHFR	0.912743	10.87593	1.33E-11
ENSG0000(	ZNF20	0.912691	1.861757	0.013748
ENSG0000(	TCEAL8	0.911944	4.208724	6.18E-05
ENSG0000(	ITM2C	0.911122	5.022209	9.50E-06
ENSG0000(	PLPP5	0.911108	4.731838	1.85E-05
ENSG0000(	CHST14	0.910993	3.979749	0.000105
ENSG0000(	RHOC	0.909619	6.274291	5.32E-07
ENSG0000(	GAB3	0.909361	4.345191	4.52E-05
ENSG0000(	USP6NL	0.909355	5.604704	2.48E-06
ENSG0000(	MGAT5	0.908788	5.270979	5.36E-06
ENSG0000(	GFRA1	0.90862	1.750079	0.01778
ENSG0000(	PHTF1	0.907255	8.89141	1.28E-09
ENSG0000(	AC009084.	0.907237	5.712651	1.94E-06
ENSG0000(	AP000350.	0.906656	10.90673	1.24E-11
ENSG0000(	TRIP6	0.906523	2.482545	0.003292
ENSG0000(	CC2D2A	0.905472	3.632146	0.000233
ENSG0000(	ARHGEF1	0.905463	4.085927	8.20E-05
ENSG0000(	AC135048.	0.905366	4.529607	2.95E-05
ENSG0000(	AC099489.	0.905359	1.690458	0.020396
ENSG0000(	THSD7A	0.905088	1.579249	0.026348
ENSG0000(	DHFR2	0.904648	3.037442	0.000917
ENSG0000(	LITAF	0.904348	4.085643	8.21E-05
ENSG0000(	HHLA3	0.904343	2.053654	0.008838
ENSG0000(	CAMKK1	0.904241	2.089983	0.008129
ENSG0000(	ADAR	0.903962	4.848212	1.42E-05
ENSG0000(	IFIT1	0.903955	3.549508	0.000282
ENSG0000(	MIR100HG	0.903773	2.991238	0.00102
ENSG0000(	CSRP1	0.903322	3.636712	0.000231
ENSG0000(	PDCD4	0.902963	5.241393	5.74E-06
ENSG0000(	ESYT3	0.90183	1.302266	0.049858
ENSG0000(	SLC30A7	0.901395	3.95928	0.00011

ENSG0000(	PCNX2	0.899439	2.455448	0.003504
ENSG0000(	CX3CL1	0.899269	3.073757	0.000844
ENSG0000(	AC067852.	0.898688	4.408563	3.90E-05
ENSG0000(	AL121987.:	0.897624	4.298467	5.03E-05
ENSG0000(	CBX6	0.897004	8.448359	3.56E-09
ENSG0000(	ZFAS1	0.896672	4.037375	9.18E-05
ENSG0000(	SH3GL1	0.896106	5.113161	7.71E-06
ENSG0000(	LAP3	0.89495	4.582672	2.61E-05
ENSG0000(	ADORA2A	0.894783	2.849821	0.001413
ENSG0000(	ADA	0.894563	4.873232	1.34E-05
ENSG0000(	RAMP2-AS	0.894377	2.096091	0.008015
ENSG0000(	BEGAIN	0.894219	2.216186	0.006079
ENSG0000(	NAB1	0.8942	6.506481	3.12E-07
ENSG0000(	PTK2B	0.894174	5.018853	9.58E-06
ENSG0000(	UBE2E2	0.89357	11.235	5.82E-12
ENSG0000(	STK32C	0.893487	9.878847	1.32E-10
ENSG0000(	INS-IGF2	0.892659	2.016969	0.009617
ENSG0000(	CACHD1	0.892509	1.765271	0.017168
ENSG0000(	PLXNA3	0.892148	3.895754	0.000127
ENSG0000(	PSMB10	0.891872	5.73428	1.84E-06
ENSG0000(	GNAO1	0.891408	3.157379	0.000696
ENSG0000(	AC027097.	0.8902	2.803995	0.00157
ENSG0000(	AKR1A1	0.88979	5.96448	1.09E-06
ENSG0000(	PTTG1IP	0.889488	6.220945	6.01E-07
ENSG0000(	SPATA6	0.888358	2.390875	0.004066
ENSG0000(	JAM2	0.887961	3.919257	0.00012
ENSG0000(	PPP1R9B	0.887891	5.688633	2.05E-06
ENSG0000(	FAM43B	0.887807	1.349952	0.044673
ENSG0000(	DCLRE1C	0.887743	4.648066	2.25E-05
ENSG0000(	ZNF829	0.887661	2.941761	0.001144
ENSG0000(	DEPP1	0.887431	1.995239	0.01011
ENSG0000(	WDR34	0.887381	3.697724	0.000201
ENSG0000(	ACER3	0.887197	6.029949	9.33E-07
ENSG0000(	BMP2	0.886823	2.201843	0.006283
ENSG0000(	SPTAN1	0.886583	5.166591	6.81E-06
ENSG0000(	PIK3IP1	0.885976	2.945327	0.001134
ENSG0000(	MIS18BP1	0.885159	3.539516	0.000289
ENSG0000(	CCDC39	0.884916	5.041774	9.08E-06
ENSG0000(	RBMS1	0.884772	4.546629	2.84E-05
ENSG0000(	PRRX1	0.884743	3.024246	0.000946
ENSG0000(	ARL6IP1	0.884716	5.460729	3.46E-06
ENSG0000(	TRIM4	0.884227	4.234397	5.83E-05
ENSG0000(	SMPD2	0.884155	2.285513	0.005182
ENSG0000(	MIR1282	0.88354	8.907198	1.24E-09
ENSG0000(	NPTXR	0.88303	2.83697	0.001456
ENSG0000(	RILPL2	0.882896	3.400927	0.000397
ENSG0000(	ARAP1-AS2	0.882663	6.918041	1.21E-07

ENSG0000(	KIF3B	0.882033	3.216573	0.000607
ENSG0000(	WARS	0.88201	4.193089	6.41E-05
ENSG0000(	1-Sep	0.881288	1.94996	0.011221
ENSG0000(	PRMT2	0.881037	7.149265	7.09E-08
ENSG0000(	IRAK4	0.881017	4.204031	6.25E-05
ENSG0000(	SGTB	0.881	7.249511	5.63E-08
ENSG0000(	GINS4	0.880496	2.499367	0.003167
ENSG0000(	IQGAP1	0.880351	4.982186	1.04E-05
ENSG0000(	STAC	0.880261	1.349635	0.044706
ENSG0000(	PROSER3	0.879237	3.644185	0.000227
ENSG0000(	NAT14	0.878843	4.256933	5.53E-05
ENSG0000(	UCHL1	0.878546	1.93768	0.011543
ENSG0000(	ZNF675	0.877185	2.759675	0.001739
ENSG0000(	AC023024.	0.877134	1.780174	0.016589
ENSG0000(	ZNF737	0.876495	2.854599	0.001398
ENSG0000(	NECTIN1	0.875945	3.901512	0.000125
ENSG0000(	FAM184A	0.875724	1.752834	0.017667
ENSG0000(	TFAP2E	0.875349	1.858293	0.013858
ENSG0000(	FAXDC2	0.875072	2.57003	0.002691
ENSG0000(	ACTB	0.874804	4.034862	9.23E-05
ENSG0000(	STK4	0.874744	5.801551	1.58E-06
ENSG0000(	MEG3	0.874492	3.848923	0.000142
ENSG0000(	LINC00921	0.874141	1.539577	0.028868
ENSG0000(	ODF2	0.873527	4.831894	1.47E-05
ENSG0000(	AP000757.	0.873313	2.711703	0.001942
ENSG0000(	STAT2	0.872991	3.76715	0.000171
ENSG0000(	SINHCAF	0.872985	4.629718	2.35E-05
ENSG0000(	GMFB	0.872928	4.766945	1.71E-05
ENSG0000(	BCL10	0.872602	4.076558	8.38E-05
ENSG0000(	EZR-AS1	0.871977	3.48683	0.000326
ENSG0000(	ALG1L9P	0.871886	1.56514	0.027218
ENSG0000(	AL021546.:	0.871735	5.47709	3.33E-06
ENSG0000(	DOC2B	0.871729	2.444329	0.003595
ENSG0000(	SLC1A7	0.871595	1.900012	0.012589
ENSG0000(	P3H1	0.87131	3.606936	0.000247
ENSG0000(	COLCA1	0.871243	1.79312	0.016102
ENSG0000(	LTBP4	0.87123	2.09496	0.008036
ENSG0000(	TLE3	0.871194	4.063072	8.65E-05
ENSG0000(	COLQ	0.870395	1.706488	0.019657
ENSG0000(	AC009690.	0.870083	5.195595	6.37E-06
ENSG0000(	NAV2	0.870076	2.611807	0.002445
ENSG0000(	PLCD1	0.869963	3.707782	0.000196
ENSG0000(	HPS1	0.868724	4.184888	6.53E-05
ENSG0000(	TSPAN12	0.868475	3.03656	0.000919
ENSG0000(	PCDHB4	0.868347	4.151632	7.05E-05
ENSG0000(	C15orf39	0.868312	3.545057	0.000285
ENSG0000(	PHF11	0.868233	4.504265	3.13E-05

ENSG0000(	CD109	0.867473	1.795471	0.016015
ENSG0000(	POGLUT1	0.866259	4.814513	1.53E-05
ENSG0000(	VANGL1	0.865866	3.770794	0.00017
ENSG0000(	DAPP1	0.86586	1.570224	0.026901
ENSG0000(	KIAA1551	0.865648	2.977144	0.001054
ENSG0000(	CACNA2D4	0.865631	1.658974	0.021929
ENSG0000(	PGBD5	0.865486	1.78009	0.016592
ENSG0000(	ST3GAL4	0.865227	4.131877	7.38E-05
ENSG0000(	NBDY	0.864526	6.294861	5.07E-07
ENSG0000(	NCEH1	0.864228	5.235743	5.81E-06
ENSG0000(	PDGFC	0.86421	4.161519	6.89E-05
ENSG0000(	MIR3658	0.864057	2.868382	0.001354
ENSG0000(	UCK2	0.862757	5.691853	2.03E-06
ENSG0000(	CAMK1	0.862662	3.055903	0.000879
ENSG0000(	AC009139.	0.860961	1.935614	0.011598
ENSG0000(	PEA15	0.860373	4.258947	5.51E-05
ENSG0000(	RASL11A	0.860338	2.629591	0.002346
ENSG0000(	SLC25A5	0.860276	8.347241	4.50E-09
ENSG0000(	CBLB	0.859956	5.373289	4.23E-06
ENSG0000(	PLBD1	0.859311	2.621404	0.002391
ENSG0000(	AL117190.:	0.858808	2.599236	0.002516
ENSG0000(	ELOVL6	0.858716	1.779006	0.016634
ENSG0000(	XBP1	0.858461	5.795834	1.60E-06
ENSG0000(	FOXC1	0.858249	2.948883	0.001125
ENSG0000(	GAS5-AS1	0.858222	1.870138	0.013485
ENSG0000(	CHN2	0.858123	2.539195	0.002889
ENSG0000(	AC025165.	0.857396	4.506211	3.12E-05
ENSG0000(	CNDP2	0.856597	6.812327	1.54E-07
ENSG0000(	ZFP69	0.856354	2.892059	0.001282
ENSG0000(	MIR3652	0.856004	6.527743	2.97E-07
ENSG0000(	CDC42SE1	0.855996	9.919708	1.20E-10
ENSG0000(	CAMK2N1	0.855476	2.634533	0.00232
ENSG0000(	AP1S1	0.855145	6.393726	4.04E-07
ENSG0000(	FXYD6-FXY	0.853851	2.586102	0.002594
ENSG0000(	SPINT2	0.853536	2.357808	0.004387
ENSG0000(	EAF2	0.853143	2.598443	0.002521
ENSG0000(	SFXN2	0.853055	2.968211	0.001076
ENSG0000(	KBTBD11-C	0.85262	2.541901	0.002871
ENSG0000(	AL135910.:	0.852398	1.423565	0.037708
ENSG0000(	RAP1GAP2	0.852357	1.9647	0.010847
ENSG0000(	AC008581.	0.851931	1.553216	0.027976
ENSG0000(	PCDH19	0.851556	2.826091	0.001492
ENSG0000(	LCA5	0.851504	3.008923	0.00098
ENSG0000(	SPARCL1	0.851159	3.587671	0.000258
ENSG0000(	SS18	0.850893	5.568364	2.70E-06
ENSG0000(	GALNT4	0.850822	3.585946	0.000259
ENSG0000(	AC015795.	0.85033	2.347624	0.004491

ENSG0000(	FER	0.850261	4.047501	8.96E-05
ENSG0000(	SYNGAP1	0.84924	3.58007	0.000263
ENSG0000(	RBMS3	0.849217	2.105044	0.007852
ENSG0000(	UBXN11	0.848862	2.456438	0.003496
ENSG0000(	PLEKHG5	0.848787	3.359302	0.000437
ENSG0000(	CENPA	0.848553	3.983547	0.000104
ENSG0000(	DPEP2	0.848516	2.716609	0.00192
ENSG0000(	AC015912.	0.848511	4.603211	2.49E-05
ENSG0000(	LRRC49	0.847869	2.106223	0.00783
ENSG0000(	MXRA7	0.847535	2.876671	0.001328
ENSG0000(	TCF7	0.847411	1.42405	0.037666
ENSG0000(	NLRP1	0.847226	2.435916	0.003665
ENSG0000(	AC002401.	0.846886	4.195342	6.38E-05
ENSG0000(	ZNF667-AS	0.84688	2.698199	0.002004
ENSG0000(	RAB4B	0.846381	5.358411	4.38E-06
ENSG0000(	LUZP1	0.846358	7.163955	6.86E-08
ENSG0000(	BIN3	0.84586	7.625939	2.37E-08
ENSG0000(	CEBPA	0.844828	1.773497	0.016846
ENSG0000(	TPK1	0.844224	3.441329	0.000362
ENSG0000(	TMEM273	0.843452	3.330839	0.000467
ENSG0000(	AC009690.	0.84334	4.172419	6.72E-05
ENSG0000(	OS9	0.843036	4.842184	1.44E-05
ENSG0000(	TMEM140	0.842929	4.670962	2.13E-05
ENSG0000(	ATF5	0.842922	10.54832	2.83E-11
ENSG0000(	A4GALT	0.842819	3.19951	0.000632
ENSG0000(	AC016876.	0.842553	4.136645	7.30E-05
ENSG0000(	IL4I1	0.842465	5.463309	3.44E-06
ENSG0000(	AL512353.:	0.842184	3.59914	0.000252
ENSG0000(	ACOX2	0.841581	5.37573	4.21E-06
ENSG0000(	AC092069.	0.840867	3.247349	0.000566
ENSG0000(	TMEM100	0.840763	1.659615	0.021897
ENSG0000(	AC080112.	0.840558	3.28545	0.000518
ENSG0000(	AC007292.	0.840517	4.363444	4.33E-05
ENSG0000(	DCHS1	0.840367	2.933676	0.001165
ENSG0000(	IL1RAP	0.839471	2.384405	0.004127
ENSG0000(	GHDC	0.838995	4.968709	1.07E-05
ENSG0000(	FERP1	0.838512	2.219165	0.006037
ENSG0000(	AC009237.	0.838398	2.303125	0.004976
ENSG0000(	AC010542.	0.837821	1.773711	0.016838
ENSG0000(	TNFRSF10E	0.837542	4.306655	4.94E-05
ENSG0000(	CEP164	0.8375	2.581869	0.002619
ENSG0000(	C21orf91	0.83697	4.83251	1.47E-05
ENSG0000(	ZNF718	0.836811	3.056667	0.000878
ENSG0000(	MAPK13	0.836564	3.191431	0.000644
ENSG0000(	KIF20B	0.836187	2.173761	0.006703
ENSG0000(	SEPT4-AS1	0.836011	2.197157	0.006351
ENSG0000(	SUCO	0.835564	7.815253	1.53E-08

ENSG0000(	RASSF9	0.83541	3.892396	0.000128
ENSG0000(	PCYOX1L	0.835126	3.544448	0.000285
ENSG0000(	TNRC6C-AS	0.835002	2.603644	0.002491
ENSG0000(	SEMA6B	0.834109	2.137817	0.007281
ENSG0000(	GIMAP5	0.833745	4.399866	3.98E-05
ENSG0000(	ARHGAP1	0.833662	4.053311	8.84E-05
ENSG0000(	TYRO3	0.833645	1.662228	0.021766
ENSG0000(	AC091564.	0.833505	7.578796	2.64E-08
ENSG0000(	TRIM73	0.833213	1.441016	0.036223
ENSG0000(	MYZAP	0.833178	1.748796	0.017832
ENSG0000(	MPZL2	0.833154	1.561709	0.027434
ENSG0000(	ATP1A1-AS	0.832126	5.539704	2.89E-06
ENSG0000(	SPOCK1	0.832091	1.57662	0.026508
ENSG0000(	AC090971.	0.831996	2.764585	0.00172
ENSG0000(	CATSPER2F	0.831993	5.600845	2.51E-06
ENSG0000(	NECTIN2	0.831939	4.537447	2.90E-05
ENSG0000(	GIMAP1-GI	0.831888	4.348562	4.48E-05
ENSG0000(	CCND3	0.831422	4.799903	1.59E-05
ENSG0000(	NOL4L	0.831211	2.540419	0.002881
ENSG0000(	ZNF107	0.830842	3.514685	0.000306
ENSG0000(	FAM3C	0.830819	2.958953	0.001099
ENSG0000(	AC008738.	0.830753	1.703019	0.019814
ENSG0000(	SMPD3	0.830547	2.463082	0.003443
ENSG0000(	NPHP4	0.829934	5.254492	5.57E-06
ENSG0000(	TMEM234	0.829893	3.47405	0.000336
ENSG0000(	NBPF19	0.829699	3.809374	0.000155
ENSG0000(	ADPGK-AS1	0.829034	2.16133	0.006897
ENSG0000(	EML4	0.828535	4.606306	2.48E-05
ENSG0000(	NTRK3	0.82844	1.412458	0.038685
ENSG0000(	AP000646.	0.828339	3.742468	0.000181
ENSG0000(	CCDC115	0.828335	5.609303	2.46E-06
ENSG0000(	PRKD2	0.827147	3.428538	0.000373
ENSG0000(	IDNK	0.826369	3.177434	0.000665
ENSG0000(	AMIGO2	0.82626	2.55926	0.002759
ENSG0000(	NPY1R	0.825288	1.493942	0.032067
ENSG0000(	RTKN	0.824479	8.532064	2.94E-09
ENSG0000(	AC092279.	0.82433	1.416986	0.038284
ENSG0000(	ADPGK	0.824199	4.617668	2.41E-05
ENSG0000(	SLC23A2	0.823953	4.756522	1.75E-05
ENSG0000(	FOXP1	0.823795	3.948538	0.000113
ENSG0000(	BID	0.823747	7.585699	2.60E-08
ENSG0000(	PLOD3	0.822948	6.045693	9.00E-07
ENSG0000(	NOD1	0.821947	3.940224	0.000115
ENSG0000(	CES2	0.821934	5.795402	1.60E-06
ENSG0000(	DCLRE1B	0.821522	4.858401	1.39E-05
ENSG0000(	TUBB2A	0.820763	4.23769	5.79E-05
ENSG0000(	AL355312.!	0.820542	2.740595	0.001817

ENSG0000(	PTPN18	0.82002	3.326231	0.000472
ENSG0000(	OGFR	0.819874	7.07117	8.49E-08
ENSG0000(	INF2	0.819709	5.619527	2.40E-06
ENSG0000(	TGFB1I1	0.819209	2.263138	0.005456
ENSG0000(	CCDC191	0.818916	2.480388	0.003308
ENSG0000(	LAMA3	0.818642	1.948849	0.01125
ENSG0000(	AC004997.	0.818561	2.92597	0.001186
ENSG0000(	METTL7B	0.818523	1.594111	0.025462
ENSG0000(	GLB1	0.81835	4.550672	2.81E-05
ENSG0000(	DAAM1	0.818155	5.709492	1.95E-06
ENSG0000(	CDC42EP2	0.817617	3.884186	0.000131
ENSG0000(	NME1	0.817424	5.393597	4.04E-06
ENSG0000(	LGALS8-AS	0.817026	1.30856	0.049141
ENSG0000(	GAS5	0.816779	4.542794	2.87E-05
ENSG0000(	MYL5	0.816309	3.910612	0.000123
ENSG0000(	AL358115.:	0.815272	4.201576	6.29E-05
ENSG0000(	AGTR1	0.815255	2.088465	0.008157
ENSG0000(	IDUA	0.815236	3.413192	0.000386
ENSG0000(	NIPSNAP3/	0.815222	9.374175	4.22E-10
ENSG0000(	ALDOC	0.814966	1.534338	0.029219
ENSG0000(	AC025287.	0.814957	3.916476	0.000121
ENSG0000(	FAM86DP	0.814736	2.586768	0.00259
ENSG0000(	PRR11	0.814708	2.698199	0.002004
ENSG0000(	FAM86C1	0.814473	2.666139	0.002157
ENSG0000(	OSBPL10	0.814206	2.989847	0.001024
ENSG0000(	TRIM11	0.814087	3.917036	0.000121
ENSG0000(	MAP3K1	0.814068	3.361496	0.000435
ENSG0000(	ARMCX2	0.813801	5.139635	7.25E-06
ENSG0000(	TACC3	0.813752	3.843467	0.000143
ENSG0000(	PDE1B	0.813724	1.766145	0.017134
ENSG0000(	FGD5	0.813706	2.273058	0.005333
ENSG0000(	PPFIA3	0.813488	2.388884	0.004084
ENSG0000(	ICAM3	0.813011	3.307346	0.000493
ENSG0000(	CAPN12	0.812131	4.076055	8.39E-05
ENSG0000(	RAB29	0.811599	4.803093	1.57E-05
ENSG0000(	MOSPD2	0.810999	3.274004	0.000532
ENSG0000(	CD40	0.810546	3.096138	0.000801
ENSG0000(	AL157871.4	0.81054	3.21352	0.000612
ENSG0000(	ACTA2	0.809481	2.491927	0.003222
ENSG0000(	SIPA1	0.809408	3.268511	0.000539
ENSG0000(	B3GAT3	0.809355	4.78811	1.63E-05
ENSG0000(	ZEB1-AS1	0.808679	6.083826	8.24E-07
ENSG0000(	PPP1R21	0.808551	8.821103	1.51E-09
ENSG0000(	FKBP9	0.80835	3.278087	0.000527
ENSG0000(	COL18A1	0.808012	4.43698	3.66E-05
ENSG0000(	SERPINB1	0.807779	5.848925	1.42E-06
ENSG0000(	FMO3	0.807054	1.323267	0.047504

ENSG0000(	FXYD6	0.806946	2.598443	0.002521
ENSG0000(	MIA-RAB4E	0.806633	4.886121	1.30E-05
ENSG0000(	ZNF117	0.806304	2.212912	0.006125
ENSG0000(	IQCN	0.805263	2.001326	0.00997
ENSG0000(	CKAP4	0.805217	6.697596	2.01E-07
ENSG0000(	ADGRE5	0.805103	7.003494	9.92E-08
ENSG0000(	SNAPC2	0.804694	3.841359	0.000144
ENSG0000(	LINC01547	0.804467	1.923426	0.011928
ENSG0000(	RBPMS-AS:	0.804176	1.983294	0.010392
ENSG0000(	AC006460.	0.804116	3.455891	0.00035
ENSG0000(	KLHL3	0.804098	2.710787	0.001946
ENSG0000(	SPECC1	0.803732	2.182458	0.00657
ENSG0000(	TPM3P9	0.803476	4.246354	5.67E-05
ENSG0000(	TUFT1	0.803153	5.227488	5.92E-06
ENSG0000(	PXDN	0.802933	2.648378	0.002247
ENSG0000(	SLC38A6	0.801655	3.526468	0.000298
ENSG0000(	ATL1	0.800479	2.88044	0.001317
ENSG0000(	TVP23C-CD	0.800478	2.974392	0.001061
ENSG0000(	AC114490.	0.800151	4.676221	2.11E-05
ENSG0000(	SLC9B2	0.800106	2.484257	0.003279
ENSG0000(	AC004696.	0.799983	1.800885	0.015817
ENSG0000(	GAB2	0.799922	2.910793	0.001228
ENSG0000(	RDH11	0.799666	4.806426	1.56E-05
ENSG0000(	TMEM50A	0.799442	6.570343	2.69E-07
ENSG0000(	TSPAN6	0.798298	1.949577	0.011231
ENSG0000(	CDR2L	0.79818	2.47415	0.003356
ENSG0000(	PGLS	0.798115	3.452881	0.000352
ENSG0000(	AC087521.	0.797541	1.570961	0.026856
ENSG0000(	ARSB	0.797324	2.768518	0.001704
ENSG0000(	CKS2	0.797167	2.040686	0.009106
ENSG0000(	KLHL42	0.797086	4.63248	2.33E-05
ENSG0000(	SEC14L2	0.796311	2.221182	0.006009
ENSG0000(	P2RY6	0.79624	1.817653	0.015218
ENSG0000(	FUT11	0.796106	9.033767	9.25E-10
ENSG0000(	MANBA	0.796077	3.163198	0.000687
ENSG0000(	AC016825.	0.795644	1.554189	0.027913
ENSG0000(	THPO	0.795638	2.300293	0.005008
ENSG0000(	AC108449.	0.793474	1.327443	0.04705
ENSG0000(	BMP1	0.793224	2.109685	0.007768
ENSG0000(	IFFO1	0.792963	5.056883	8.77E-06
ENSG0000(	SLC16A9	0.792667	1.450026	0.035479
ENSG0000(	TUB	0.792635	1.966879	0.010792
ENSG0000(	CTDSPL	0.79262	4.263934	5.45E-05
ENSG0000(	ZNF286B	0.792533	1.734327	0.018436
ENSG0000(	ZNF367	0.792171	2.670576	0.002135
ENSG0000(	AGRN	0.791466	2.362705	0.004338
ENSG0000(	NANS	0.790737	4.082434	8.27E-05

ENSG0000(	PPP4R1L	0.790679	3.576852	0.000265
ENSG0000(	OSBPL8	0.79042	4.453233	3.52E-05
ENSG0000(	LPCAT1	0.790329	2.498663	0.003172
ENSG0000(	SGPP1	0.78989	5.168296	6.79E-06
ENSG0000(	ATP1A1	0.789643	5.142383	7.20E-06
ENSG0000(	ACTA2-AS1	0.789613	2.188873	0.006473
ENSG0000(	PCSK6	0.78958	1.782007	0.016519
ENSG0000(	KL	0.788743	2.710229	0.001949
ENSG0000(	AP003392.	0.788723	3.344115	0.000453
ENSG0000(	PARP10	0.788372	6.768172	1.71E-07
ENSG0000(	AC115618.	0.788231	2.29139	0.005112
ENSG0000(	LRFN4	0.787984	3.617101	0.000241
ENSG0000(	AKR1C2	0.787617	1.948418	0.011261
ENSG0000(	TBCEL	0.787552	13.1351	7.33E-14
ENSG0000(	TCF7L1	0.787401	4.934306	1.16E-05
ENSG0000(	AL662884.:	0.786752	4.27802	5.27E-05
ENSG0000(	MANEA	0.786602	2.416372	0.003834
ENSG0000(	TMEM87B	0.786019	3.047064	0.000897
ENSG0000(	ZNF668	0.785884	5.090052	8.13E-06
ENSG0000(	CREBL2	0.78515	3.971884	0.000107
ENSG0000(	AC068631.	0.784572	8.308235	4.92E-09
ENSG0000(	SPATA1	0.784439	2.685568	0.002063
ENSG0000(	SH3BP5	0.784395	2.88733	0.001296
ENSG0000(	RCAN3	0.784383	2.478846	0.00332
ENSG0000(	RNF207	0.784172	3.136233	0.000731
ENSG0000(	ATP2B4	0.783708	6.560694	2.75E-07
ENSG0000(	FADS1	0.783672	2.411981	0.003873
ENSG0000(	AC104662.	0.783369	3.36702	0.00043
ENSG0000(	RADIL	0.783068	2.182201	0.006574
ENSG0000(	RIT1	0.782844	8.744318	1.80E-09
ENSG0000(	AC114490.	0.78271	1.519368	0.030244
ENSG0000(	ITIH5	0.782292	3.237395	0.000579
ENSG0000(	ASNS	0.781674	3.371121	0.000425
ENSG0000(	ITPRIP	0.781543	6.510777	3.08E-07
ENSG0000(	RMDN2	0.781355	3.683334	0.000207
ENSG0000(	HFE	0.7812	2.437039	0.003656
ENSG0000(	FLRT2	0.78078	3.581696	0.000262
ENSG0000(	SPACA6	0.780506	1.494365	0.032036
ENSG0000(	AC009061.	0.780391	1.424741	0.037606
ENSG0000(	NECTIN3	0.780309	1.949998	0.01122
ENSG0000(	ANKRD50	0.780111	3.185317	0.000653
ENSG0000(	CALCRL	0.779681	2.937919	0.001154
ENSG0000(	GNG12	0.779535	2.721592	0.001898
ENSG0000(	MCAM	0.779159	1.847065	0.014221
ENSG0000(	PECAM1	0.778986	2.891021	0.001285
ENSG0000(	KIF2A	0.778412	5.462599	3.45E-06
ENSG0000(	LMNA	0.778402	7.522117	3.01E-08

ENSG0000(	AC008738.	0.777589	1.458259	0.034813
ENSG0000(	BTN2A1	0.777449	10.70799	1.96E-11
ENSG0000(	5-Sep	0.776874	2.401463	0.003968
ENSG0000(	IFFO2	0.776864	3.755291	0.000176
ENSG0000(	TMEM30B	0.776484	1.92868	0.011785
ENSG0000(	CNKSR3	0.776331	2.168159	0.00679
ENSG0000(	NSUN3	0.775939	3.479643	0.000331
ENSG0000(	HPGD	0.775758	1.454789	0.035092
ENSG0000(	CALR	0.775609	4.364633	4.32E-05
ENSG0000(	AC008149.	0.775556	1.859491	0.01382
ENSG0000(	PI4K2B	0.775121	3.108982	0.000778
ENSG0000(	AC135050.	0.774515	4.594596	2.54E-05
ENSG0000(	DDB2	0.774236	3.636316	0.000231
ENSG0000(	NRN1	0.774108	3.374282	0.000422
ENSG0000(	RAD51D	0.774051	3.94397	0.000114
ENSG0000(	BNIP3L	0.773927	3.786624	0.000163
ENSG0000(	GALK1	0.772816	4.156354	6.98E-05
ENSG0000(	FAM171A1	0.77281	3.488451	0.000325
ENSG0000(	AC005020.	0.772626	8.807536	1.56E-09
ENSG0000(	TMEM208	0.77256	4.560572	2.75E-05
ENSG0000(	WASF2	0.772296	3.889688	0.000129
ENSG0000(	CD27	0.772054	4.49001	3.24E-05
ENSG0000(	AC010542.	0.771747	2.456455	0.003496
ENSG0000(	RAB5C	0.771439	4.173363	6.71E-05
ENSG0000(	KCNC4	0.770404	1.472747	0.033671
ENSG0000(	SPTLC2	0.770388	3.646621	0.000226
ENSG0000(	RBM43	0.770016	4.247662	5.65E-05
ENSG0000(	SYCE1L	0.768986	2.272693	0.005337
ENSG0000(	PXYLP1	0.768557	1.967332	0.010781
ENSG0000(	AC093726.	0.767012	1.56687	0.02711
ENSG0000(	TSPAN9	0.766944	3.816131	0.000153
ENSG0000(	AL356124.:	0.766782	2.536555	0.002907
ENSG0000(	GMDS	0.765744	4.917245	1.21E-05
ENSG0000(	NAGA	0.765155	3.428193	0.000373
ENSG0000(	LRRC8C-DT	0.765144	2.047701	0.00896
ENSG0000(	PNMA1	0.765021	5.145487	7.15E-06
ENSG0000(	AC093423.	0.764954	2.012624	0.009714
ENSG0000(	STS	0.764827	4.807005	1.56E-05
ENSG0000(	NPR1	0.76421	1.483703	0.032832
ENSG0000(	AC008537.	0.763932	2.710601	0.001947
ENSG0000(	RSU1	0.763789	4.213064	6.12E-05
ENSG0000(	CHMP4A	0.76357	7.851068	1.41E-08
ENSG0000(	TAPBPL	0.763306	5.580094	2.63E-06
ENSG0000(	11-Sep	0.762626	4.536216	2.91E-05
ENSG0000(	ITM2B	0.762359	5.632393	2.33E-06
ENSG0000(	PLEKHA7	0.762255	2.734167	0.001844
ENSG0000(	AC108449.	0.762115	4.800394	1.58E-05

ENSG0000(	FRG1CP	0.761641	2.6889	0.002047
ENSG0000(	CTSB	0.760782	5.307686	4.92E-06
ENSG0000(	SETDB2	0.760399	7.210645	6.16E-08
ENSG0000(	PLEKHO1	0.759486	4.189161	6.47E-05
ENSG0000(	ANKRD36C	0.759081	1.675465	0.021112
ENSG0000(	SASH1	0.758436	4.03094	9.31E-05
ENSG0000(	AC027307.	0.758271	2.390973	0.004065
ENSG0000(	SMCO4	0.757273	1.794369	0.016056
ENSG0000(	RAP1GDS1	0.756993	3.954134	0.000111
ENSG0000(	CTTNBP2N	0.756793	3.995681	0.000101
ENSG0000(	KCTD7	0.756626	5.622067	2.39E-06
ENSG0000(	LAMP1	0.756606	4.027441	9.39E-05
ENSG0000(	GOLGA8A	0.755867	1.99534	0.010108
ENSG0000(	SLITRK4	0.754752	1.352161	0.044447
ENSG0000(	AL670729.:	0.754704	1.787135	0.016325
ENSG0000(	LZTFL1	0.75464	3.24231	0.000572
ENSG0000(	KLF6	0.753477	3.564661	0.000272
ENSG0000(	GULP1	0.753113	4.050715	8.90E-05
ENSG0000(	IFT57	0.753028	2.845559	0.001427
ENSG0000(	CYFIP1	0.752939	6.332829	4.65E-07
ENSG0000(	ABR	0.752753	7.048795	8.94E-08
ENSG0000(	LGALS8	0.751897	4.38022	4.17E-05
ENSG0000(	NAGK	0.751866	5.260617	5.49E-06
ENSG0000(	PPIP5K2	0.751798	3.280645	0.000524
ENSG0000(	A2M-AS1	0.751635	3.101661	0.000791
ENSG0000(	MYH9	0.751402	4.116266	7.65E-05
ENSG0000(	FKBP14	0.751378	3.099242	0.000796
ENSG0000(	RNASEH2B	0.751243	3.633608	0.000232
ENSG0000(	MAPK10	0.751066	4.82599	1.49E-05
ENSG0000(	DNAJB11	0.750488	11.83423	1.46E-12
ENSG0000(	ZBTB22	0.750382	7.352304	4.44E-08
ENSG0000(	BCR	0.75011	2.525629	0.002981
ENSG0000(	BMP4	0.750101	2.030414	0.009324
ENSG0000(	AL096870.:	0.749882	8.005267	9.88E-09
ENSG0000(	HSD17B7	0.749551	4.165534	6.83E-05
ENSG0000(	RAP1B	0.749159	3.645935	0.000226
ENSG0000(	PDGFRB	0.748564	1.927606	0.011814
ENSG0000(	MAGEH1	0.747855	3.600618	0.000251
ENSG0000(	ODF2-AS1	0.7475	2.865142	0.001364
ENSG0000(	RPL23AP7	0.747333	1.515022	0.030548
ENSG0000(	AC008569.	0.746932	5.35622	4.40E-06
ENSG0000(	AC091167.	0.746448	1.851258	0.014085
ENSG0000(	DYNLT3	0.745726	3.98956	0.000102
ENSG0000(	MAPK8IP1	0.745556	3.349711	0.000447
ENSG0000(	CDCA4	0.745143	3.76715	0.000171
ENSG0000(	GPRIN3	0.744992	2.330872	0.004668
ENSG0000(	DUOX1	0.744599	1.338344	0.045883

ENSG0000(	RHOG	0.744229	4.04606	8.99E-05
ENSG0000(	NEK3	0.743904	2.834079	0.001465
ENSG0000(	SLC36A4	0.743772	2.940253	0.001147
ENSG0000(	TTLL3	0.743501	1.864907	0.013649
ENSG0000(	APH1B	0.743493	3.328132	0.00047
ENSG0000(	YWHAQ	0.742753	4.83476	1.46E-05
ENSG0000(	LYST	0.742716	3.91768	0.000121
ENSG0000(	PTPRK	0.742498	5.901975	1.25E-06
ENSG0000(	C1orf162	0.741819	1.727579	0.018725
ENSG0000(	PTPRA	0.741744	3.829906	0.000148
ENSG0000(	SEPT5-GP1	0.74089	2.178816	0.006625
ENSG0000(	IL10RB	0.740293	4.258947	5.51E-05
ENSG0000(	CERS5	0.740093	3.099744	0.000795
ENSG0000(	RASGEF1A	0.740018	1.527269	0.029698
ENSG0000(	ST6GAL1	0.73966	4.256933	5.53E-05
ENSG0000(	ALG1L6P	0.739559	1.670285	0.021366
ENSG0000(	MARCKSL1	0.739355	2.758149	0.001745
ENSG0000(	FANCG	0.739201	5.136142	7.31E-06
ENSG0000(	BX255925.	0.739113	4.5738	2.67E-05
ENSG0000(	GDAP2	0.738782	4.443829	3.60E-05
ENSG0000(	CDRT4	0.73867	2.021969	0.009507
ENSG0000(	B3GNT8	0.738418	1.75913	0.017413
ENSG0000(	TCF7L2	0.737172	2.914962	0.001216
ENSG0000(	SPRYD3	0.736384	3.115865	0.000766
ENSG0000(	NBPF10	0.735996	1.859895	0.013807
ENSG0000(	CTBS	0.735995	2.316253	0.004828
ENSG0000(	AC114488.	0.735868	1.327569	0.047036
ENSG0000(	KIAA1549	0.735251	2.543029	0.002864
ENSG0000(	SLC30A4	0.734973	3.093475	0.000806
ENSG0000(	ELK3	0.734336	3.296405	0.000505
ENSG0000(	DSTN	0.734267	2.66335	0.002171
ENSG0000(	PARP11	0.734236	4.781712	1.65E-05
ENSG0000(	GLCCI1	0.73412	4.043848	9.04E-05
ENSG0000(	OGG1	0.733939	3.382012	0.000415
ENSG0000(	ACTR3	0.733326	8.761778	1.73E-09
ENSG0000(	RAMP3	0.733048	1.881445	0.013139
ENSG0000(	GNG11	0.732579	2.260162	0.005493
ENSG0000(	EXT1	0.732575	2.849474	0.001414
ENSG0000(	AL078612.:	0.732063	7.213423	6.12E-08
ENSG0000(	STARD13	0.732048	4.668735	2.14E-05
ENSG0000(	PPP1R16B	0.732037	2.590333	0.002568
ENSG0000(	CDK15	0.732011	1.52774	0.029666
ENSG0000(	ARHGAP23	0.73198	4.690267	2.04E-05
ENSG0000(	LPP-AS2	0.731847	3.132562	0.000737
ENSG0000(	FAM214B	0.731564	6.25689	5.53E-07
ENSG0000(	DOK4	0.73148	2.187929	0.006487
ENSG0000(	VWCE	0.731108	2.017363	0.009608

ENSG0000(	P4HA2	0.73089	4.567694	2.71E-05
ENSG0000(	AC090004.	0.730242	4.130893	7.40E-05
ENSG0000(	CEP41	0.730044	2.852625	0.001404
ENSG0000(	ADAM10	0.72994	5.90894	1.23E-06
ENSG0000(	ZC3H12A	0.729897	2.024047	0.009461
ENSG0000(	APOD	0.729395	1.461943	0.034519
ENSG0000(	AC068831.	0.72932	3.056456	0.000878
ENSG0000(	C1orf159	0.728797	2.855337	0.001395
ENSG0000(	ZNF677	0.728523	4.414065	3.85E-05
ENSG0000(	ITGAV	0.728363	3.834385	0.000146
ENSG0000(	APMAP	0.728352	4.000397	9.99E-05
ENSG0000(	PHLDA3	0.727775	2.806044	0.001563
ENSG0000(	TMEM43	0.727519	4.796629	1.60E-05
ENSG0000(	RND3	0.727439	1.512034	0.030759
ENSG0000(	APEX2	0.727383	6.824754	1.50E-07
ENSG0000(	MPO	0.727046	1.529518	0.029545
ENSG0000(	FADS2	0.72663	2.146661	0.007134
ENSG0000(	CIDECP	0.726283	4.621591	2.39E-05
ENSG0000(	MINDY4	0.726261	1.66241	0.021757
ENSG0000(	TRIL	0.726222	1.636846	0.023076
ENSG0000(	VAMP1	0.726075	5.121126	7.57E-06
ENSG0000(	GIMAP7	0.725608	2.49341	0.003211
ENSG0000(	AL591806.:	0.725526	4.437424	3.65E-05
ENSG0000(	AC092849.	0.724715	3.196862	0.000636
ENSG0000(	CCDC146	0.724404	2.488261	0.003249
ENSG0000(	CCDC88A	0.724274	3.523919	0.000299
ENSG0000(	SPTLC1	0.723625	3.334494	0.000463
ENSG0000(	GLS	0.723571	5.953535	1.11E-06
ENSG0000(	FGF2	0.723494	3.282934	0.000521
ENSG0000(	AC027277.	0.722368	3.78183	0.000165
ENSG0000(	SLC19A1	0.722175	3.752986	0.000177
ENSG0000(	NLRC3	0.722134	1.914321	0.012181
ENSG0000(	INSIG1	0.721473	2.652064	0.002228
ENSG0000(	GK	0.721273	3.013176	0.00097
ENSG0000(	TET3	0.720503	3.583469	0.000261
ENSG0000(	NAV1	0.720271	3.167064	0.000681
ENSG0000(	AC012173.	0.719652	3.66305	0.000217
ENSG0000(	FAM13B	0.719546	3.936011	0.000116
ENSG0000(	FBXO5	0.71954	2.306796	0.004934
ENSG0000(	GAS2L1	0.719504	2.526648	0.002974
ENSG0000(	ALG10	0.719002	1.355794	0.044076
ENSG0000(	AC003991.	0.71892	4.091983	8.09E-05
ENSG0000(	LYNX1-SLU	0.71863	1.564924	0.027232
ENSG0000(	TK2	0.718587	2.455029	0.003507
ENSG0000(	TRIM25	0.717806	3.550878	0.000281
ENSG0000(	TMOD3	0.717802	3.95197	0.000112
ENSG0000(	TUBB6	0.717801	5.318253	4.81E-06

ENSG0000(	RAB13	0.717327	2.979503	0.001048
ENSG0000(	DPY19L3	0.717137	2.494558	0.003202
ENSG0000(	GIMAP1	0.717093	2.376797	0.0042
ENSG0000(	RAD51B	0.716722	1.647815	0.0225
ENSG0000(	AMMECR1	0.716643	3.498416	0.000317
ENSG0000(	ITPA	0.716453	7.284136	5.20E-08
ENSG0000(	CHSY1	0.716097	4.197318	6.35E-05
ENSG0000(	RELB	0.715914	2.725326	0.001882
ENSG0000(	TONSL	0.715845	3.096138	0.000801
ENSG0000(	AC005786.	0.715428	2.395282	0.004025
ENSG0000(	HMGN1	0.714814	5.146073	7.14E-06
ENSG0000(	PIGM	0.714733	4.159016	6.93E-05
ENSG0000(	CFL1	0.714691	5.691853	2.03E-06
ENSG0000(	GXYLT2	0.714523	4.258861	5.51E-05
ENSG0000(	MICA	0.714018	1.61806	0.024096
ENSG0000(	NBEAL2	0.713701	2.948208	0.001127
ENSG0000(	AC007382.	0.712824	1.609355	0.024584
ENSG0000(	AC093110.	0.71174	2.602398	0.002498
ENSG0000(	FUT10	0.711355	1.775659	0.016763
ENSG0000(	TMEM255I	0.711297	1.508443	0.031014
ENSG0000(	TMEM135	0.711082	2.954599	0.00111
ENSG0000(	HSPB11	0.71101	3.927219	0.000118
ENSG0000(	RCN1	0.710701	8.161841	6.89E-09
ENSG0000(	AFAP1L2	0.71062	1.880557	0.013166
ENSG0000(	CLUHP3	0.710565	2.288998	0.00514
ENSG0000(	HEXA	0.710198	4.122833	7.54E-05
ENSG0000(	MAGI2-AS3	0.710164	2.370731	0.004259
ENSG0000(	AC009084.	0.709722	2.312485	0.00487
ENSG0000(	AL359736.:	0.709688	4.157092	6.96E-05
ENSG0000(	KCTD1	0.709332	2.236793	0.005797
ENSG0000(	RPS27L	0.708796	5.585405	2.60E-06
ENSG0000(	PROX2	0.708311	1.983902	0.010378
ENSG0000(	RNF213	0.708276	3.989439	0.000102
ENSG0000(	CXCL12	0.707919	4.259231	5.51E-05
ENSG0000(	RABL2A	0.707726	2.000579	0.009987
ENSG0000(	BDH2	0.707721	1.880241	0.013175
ENSG0000(	EFCAB13	0.707635	1.382378	0.041459
ENSG0000(	PTEN	0.706717	5.410189	3.89E-06
ENSG0000(	ZNF254	0.706291	5.946097	1.13E-06
ENSG0000(	RASGEF1B	0.70582	1.335977	0.046134
ENSG0000(	GRK5	0.70576	1.725614	0.01881
ENSG0000(	EDEM1	0.705536	3.385723	0.000411
ENSG0000(	GNB1	0.705358	6.134436	7.34E-07
ENSG0000(	AC126474.	0.704989	2.356843	0.004397
ENSG0000(	ETS1	0.704905	5.951128	1.12E-06
ENSG0000(	HMOX1	0.704889	1.324021	0.047422
ENSG0000(	PSAT1	0.704539	2.091993	0.008091

ENSG0000(	CELF2	0.704135	3.948429	0.000113
ENSG0000(	MOB1A	0.703295	5.283965	5.20E-06
ENSG0000(	TNFRSF25	0.70298	1.901202	0.012554
ENSG0000(	CBL	0.70298	3.491698	0.000322
ENSG0000(	TMED9	0.70275	8.749612	1.78E-09
ENSG0000(	HSPA13	0.701895	5.059286	8.72E-06
ENSG0000(	SNX32	0.701821	5.797815	1.59E-06
ENSG0000(	LRP11	0.701563	2.097296	0.007993
ENSG0000(	C1GALT1C1	0.701314	2.724171	0.001887
ENSG0000(	SDCBP2	0.700569	1.651508	0.02231
ENSG0000(	SLC12A9	0.700283	3.347062	0.00045
ENSG0000(	MYO15B	0.699849	1.478228	0.033248
ENSG0000(	HEYL	0.699724	1.385774	0.041136
ENSG0000(	CCDC39	0.699693	2.046914	0.008976
ENSG0000(	PGF	0.699651	1.669976	0.021381
ENSG0000(	RBPJ	0.699644	3.473469	0.000336
ENSG0000(	AC119674.	0.699566	2.819871	0.001514
ENSG0000(	STX2	0.699529	2.525629	0.002981
ENSG0000(	EAF1-AS1	0.69942	2.793914	0.001607
ENSG0000(	AKT3	0.699267	2.082098	0.008278
ENSG0000(	PHYHD1	0.699032	4.635751	2.31E-05
ENSG0000(	TMEM165	0.698913	4.635751	2.31E-05
ENSG0000(	ROBO1	0.698903	2.711514	0.001943
ENSG0000(	TANC2	0.698629	3.660641	0.000218
ENSG0000(	ZFYVE28	0.698306	3.175714	0.000667
ENSG0000(	PUM3	0.698258	4.173363	6.71E-05
ENSG0000(	AC003991.	0.698256	1.371176	0.042543
ENSG0000(	GDPD5	0.697902	1.530635	0.029469
ENSG0000(	C8orf82	0.696768	6.526374	2.98E-07
ENSG0000(	LOXL3	0.696543	3.071455	0.000848
ENSG0000(	ZDHHC20	0.696138	3.771777	0.000169
ENSG0000(	RAMP2	0.695481	1.76233	0.017285
ENSG0000(	GRAP	0.69546	1.302054	0.049882
ENSG0000(	TREX1	0.694897	3.501403	0.000315
ENSG0000(	MRPL45P2	0.694807	1.503087	0.031399
ENSG0000(	RAB11FIP1	0.694272	3.626252	0.000236
ENSG0000(	BAIAP2	0.69397	1.598136	0.025227
ENSG0000(	FAM91A1	0.693968	3.22121	0.000601
ENSG0000(	PRR13	0.693653	4.84442	1.43E-05
ENSG0000(	CSRP2	0.693634	2.245753	0.005679
ENSG0000(	AC114271.	0.693089	3.237909	0.000578
ENSG0000(	SLC24A1	0.692042	2.784986	0.001641
ENSG0000(	AC040162.	0.691607	4.223186	5.98E-05
ENSG0000(	CORO2B	0.691527	1.35061	0.044606
ENSG0000(	PRAF2	0.691455	2.760433	0.001736
ENSG0000(	LRIG3	0.690736	2.630353	0.002342
ENSG0000(	DENND2A	0.690592	2.025957	0.00942

ENSG0000(	DDX39A	0.690432	5.87361	1.34E-06
ENSG0000(	SHF	0.690388	1.762281	0.017287
ENSG0000(	BMP2K	0.690304	2.915036	0.001216
ENSG0000(	AF111169.:	0.69011	2.532265	0.002936
ENSG0000(	CRISPLD2	0.69009	1.85841	0.013854
ENSG0000(	TCF4	0.689422	3.440572	0.000363
ENSG0000(	AL136295.!	0.688985	4.705555	1.97E-05
ENSG0000(	IGFBP2	0.688479	2.485928	0.003266
ENSG0000(	GNAI3	0.688039	4.656689	2.20E-05
ENSG0000(	ID3	0.688035	1.789124	0.016251
ENSG0000(	SH3BGRL2	0.687665	2.068174	0.008547
ENSG0000(	IQCJ-SCHIP	0.687563	1.356999	0.043954
ENSG0000(	LAMB1	0.687542	2.35759	0.004389
ENSG0000(	PDZD7	0.687272	2.095806	0.00802
ENSG0000(	ZNF582	0.686869	1.978201	0.010515
ENSG0000(	NYNRIN	0.686381	1.895114	0.012732
ENSG0000(	SLX4IP	0.686379	3.468572	0.00034
ENSG0000(	AC115618.	0.686144	2.219165	0.006037
ENSG0000(	ABHD12	0.685979	4.016823	9.62E-05
ENSG0000(	SNX20	0.685477	1.360837	0.043568
ENSG0000(	PTPRS	0.683991	1.52885	0.02959
ENSG0000(	MAGED2	0.683687	3.159256	0.000693
ENSG0000(	TYW1B	0.683647	1.590894	0.025651
ENSG0000(	TNFRSF19	0.683538	1.35033	0.044634
ENSG0000(	SGSH	0.683531	3.521235	0.000301
ENSG0000(	LRRC8C	0.68327	2.049056	0.008932
ENSG0000(	LRRC8D	0.683163	3.401164	0.000397
ENSG0000(	SERP1	0.683117	4.63225	2.33E-05
ENSG0000(	VASH1	0.682994	3.119353	0.00076
ENSG0000(	RABL2B	0.682749	2.287856	0.005154
ENSG0000(	ADGRL4	0.682362	1.493537	0.032097
ENSG0000(	OXCT1	0.682165	1.345572	0.045126
ENSG0000(	HSPA5	0.681967	7.239464	5.76E-08
ENSG0000(	RNF24	0.681357	4.146891	7.13E-05
ENSG0000(	AC005324.	0.681217	2.022801	0.009489
ENSG0000(	KCTD10	0.681032	3.060584	0.00087
ENSG0000(	ESYT1	0.68103	4.546629	2.84E-05
ENSG0000(	RPL23AP82	0.680996	3.638092	0.00023
ENSG0000(	ABCA9	0.680743	1.588893	0.02577
ENSG0000(	CYB5R3	0.680513	3.931614	0.000117
ENSG0000(	FCHSD2	0.680471	4.364267	4.32E-05
ENSG0000(	SLC37A3	0.680452	1.981774	0.010429
ENSG0000(	HIP1	0.68036	2.572505	0.002676
ENSG0000(	TCTN3	0.680359	3.609016	0.000246
ENSG0000(	MOK	0.680271	1.696771	0.020102
ENSG0000(	PASK	0.680257	2.544896	0.002852
ENSG0000(	CDR2	0.680187	2.499532	0.003166

ENSG0000(	BBC3	0.679375	1.532879	0.029317
ENSG0000(	SNX30	0.67908	2.190312	0.006452
ENSG0000(	EME1	0.679054	2.587751	0.002584
ENSG0000(	FAM3C2	0.678689	1.453567	0.035191
ENSG0000(	AC008770.	0.678193	2.328371	0.004695
ENSG0000(	QPCTL	0.678178	2.982442	0.001041
ENSG0000(	TMEM241	0.678011	2.002973	0.009932
ENSG0000(	ZSCAN12	0.677691	1.677147	0.021031
ENSG0000(	RECQL	0.67745	5.493847	3.21E-06
ENSG0000(	AC009113.	0.676682	4.388024	4.09E-05
ENSG0000(	PCSK7	0.676585	2.009324	0.009788
ENSG0000(	IQCG	0.674977	3.924595	0.000119
ENSG0000(	YAE1	0.674445	6.475303	3.35E-07
ENSG0000(	ZNF816	0.674016	2.531881	0.002938
ENSG0000(	AC009090.	0.673995	2.474012	0.003357
ENSG0000(	N4BP2	0.673655	1.986516	0.010315
ENSG0000(	CAV1	0.673445	2.354186	0.004424
ENSG0000(	FKBP2	0.673218	3.81194	0.000154
ENSG0000(	MAPRE1	0.673203	9.613073	2.44E-10
ENSG0000(	TLDC1	0.673091	3.125611	0.000749
ENSG0000(	VDR	0.672723	1.956755	0.011047
ENSG0000(	SPTBN1	0.672636	3.199991	0.000631
ENSG0000(	TCP11L1	0.672585	4.999858	1.00E-05
ENSG0000(	ZNF43	0.671917	2.965196	0.001083
ENSG0000(	MAP7D3	0.671255	3.938261	0.000115
ENSG0000(	AC125257.	0.671215	1.679646	0.02091
ENSG0000(	PLXND1	0.671001	3.23087	0.000588
ENSG0000(	PPM1H	0.670969	1.658557	0.02195
ENSG0000(	CLCN5	0.670963	3.743945	0.00018
ENSG0000(	A2M	0.670864	3.35746	0.000439
ENSG0000(	ZNF841	0.670699	2.559271	0.002759
ENSG0000(	DEGS2	0.670161	2.886378	0.001299
ENSG0000(	BLOC1S3	0.670052	4.424052	3.77E-05
ENSG0000(	PRKAG2	0.669556	1.798306	0.015911
ENSG0000(	GPR161	0.669451	2.789609	0.001623
ENSG0000(	ZDHHC23	0.669408	3.263317	0.000545
ENSG0000(	KIF13B	0.668063	6.962886	1.09E-07
ENSG0000(	CEP135	0.667992	1.9647	0.010847
ENSG0000(	TASP1	0.667899	2.064375	0.008622
ENSG0000(	FGD1	0.667578	2.009785	0.009777
ENSG0000(	MYL6	0.667435	3.459099	0.000347
ENSG0000(	AC145207.	0.666794	3.222707	0.000599
ENSG0000(	PELI1	0.666312	3.328532	0.000469
ENSG0000(	NRSN2-AS1	0.666269	1.582915	0.026127
ENSG0000(	ARHGAP39	0.666255	2.390764	0.004067
ENSG0000(	MMP28	0.666001	1.336284	0.046102
ENSG0000(	MYC	0.665993	2.336588	0.004607

ENSG0000(	NCALD	0.665753	2.32198	0.004765
ENSG0000(	TOR4A	0.664176	1.751601	0.017717
ENSG0000(	NAP1L5	0.66393	5.470838	3.38E-06
ENSG0000(	ATOX1	0.663661	3.901512	0.000125
ENSG0000(	ALDH16A1	0.663413	4.008828	9.80E-05
ENSG0000(	DLL1	0.663211	1.78454	0.016423
ENSG0000(	DNAJB14	0.66312	3.334989	0.000462
ENSG0000(	TRAK2	0.663089	7.021544	9.52E-08
ENSG0000(	PLCE1	0.662989	1.387579	0.040966
ENSG0000(	ZNF185	0.662715	1.773018	0.016865
ENSG0000(	TCTN1	0.662465	2.700779	0.001992
ENSG0000(	ADAMTS9-:	0.662014	1.309953	0.048983
ENSG0000(	EVL	0.661859	3.52642	0.000298
ENSG0000(	CLBA1	0.661609	2.189296	0.006467
ENSG0000(	HIF1A	0.66149	4.173096	6.71E-05
ENSG0000(	TACC1	0.661409	1.870517	0.013474
ENSG0000(	ARID3A	0.661404	2.842339	0.001438
ENSG0000(	AL645465.:	0.661021	2.460713	0.003462
ENSG0000(	PFN1	0.660825	5.636248	2.31E-06
ENSG0000(	PRR3	0.660126	3.12	0.000759
ENSG0000(	CLEC1A	0.659854	1.440163	0.036294
ENSG0000(	FAM122B	0.659853	3.582312	0.000262
ENSG0000(	LAMA2	0.65983	2.678915	0.002095
ENSG0000(	FZD8	0.659452	1.408751	0.039017
ENSG0000(	LLGL2	0.65925	2.005989	0.009863
ENSG0000(	CEP162	0.659099	2.242564	0.005721
ENSG0000(	CARD8-AS1	0.658651	3.374971	0.000422
ENSG0000(	SLC10A7	0.658016	2.151323	0.007058
ENSG0000(	ZNF562	0.657617	3.986258	0.000103
ENSG0000(	YWHAZ	0.657371	6.105121	7.85E-07
ENSG0000(	ACAP2	0.657193	2.852405	0.001405
ENSG0000(	AL929472.:	0.657156	2.19276	0.006416
ENSG0000(	ASAP1	0.65702	4.539852	2.89E-05
ENSG0000(	ITPR3	0.657	1.779609	0.016611
ENSG0000(	ARSA	0.656424	2.229819	0.005891
ENSG0000(	LZTS1	0.656269	1.385442	0.041168
ENSG0000(	TTLL1	0.655927	4.527533	2.97E-05
ENSG0000(	VPS4B	0.655822	4.178984	6.62E-05
ENSG0000(	SNHG12	0.655591	2.690054	0.002041
ENSG0000(	AL132780.:	0.655315	4.338109	4.59E-05
ENSG0000(	ZNF568	0.655194	2.199999	0.00631
ENSG0000(	DSC2	0.655029	1.528344	0.029625
ENSG0000(	NCKAP5L	0.654847	3.460361	0.000346
ENSG0000(	ACSL4	0.6547	6.198526	6.33E-07
ENSG0000(	COMMD6	0.654635	2.67376	0.00212
ENSG0000(	SLC39A7	0.653989	4.002354	9.95E-05
ENSG0000(	HYOU1	0.653951	5.273615	5.33E-06

ENSG0000(	SLC50A1	0.653212	3.438179	0.000365
ENSG0000(	AP000295.	0.652781	5.347057	4.50E-06
ENSG0000(	ENO1	0.651981	3.761811	0.000173
ENSG0000(	SNHG28	0.651626	1.636136	0.023113
ENSG0000(	MYO1E	0.651571	2.553724	0.002794
ENSG0000(	STXBP4	0.651193	2.040518	0.009109
ENSG0000(	RNASEL	0.651173	3.196741	0.000636
ENSG0000(	CFAP36	0.651131	2.557522	0.00277
ENSG0000(	GIMAP6	0.650944	2.784739	0.001642
ENSG0000(	CSTB	0.65046	4.286498	5.17E-05
ENSG0000(	ENTPD1	0.650321	3.39484	0.000403
ENSG0000(	CYP4V2	0.650052	1.832532	0.014705
ENSG0000(	AC069224.	0.650007	1.845794	0.014263
ENSG0000(	KLF3	0.649978	4.291911	5.11E-05
ENSG0000(	CCDC14	0.649772	2.308429	0.004916
ENSG0000(	SLC40A1	0.649715	3.292692	0.00051
ENSG0000(	YEATS2	0.649704	5.580093	2.63E-06
ENSG0000(	SERINC5	0.649607	2.85721	0.001389
ENSG0000(	EHD2	0.649256	2.335627	0.004617
ENSG0000(	TAF1A	0.648972	1.482923	0.032891
ENSG0000(	TTTY15	0.648469	1.913309	0.012209
ENSG0000(	KIF3C	0.647705	2.486416	0.003263
ENSG0000(	CENPC	0.647586	3.008845	0.00098
ENSG0000(	SYP	0.647582	1.610617	0.024512
ENSG0000(	ECT2	0.647393	1.793824	0.016076
ENSG0000(	EVI5	0.647349	3.098444	0.000797
ENSG0000(	KIAA1522	0.647139	3.089681	0.000813
ENSG0000(	ERLIN1	0.647081	4.429778	3.72E-05
ENSG0000(	CAPZA1	0.646902	3.428276	0.000373
ENSG0000(	SLC17A5	0.646824	2.332875	0.004646
ENSG0000(	RAD9B	0.646803	2.011884	0.00973
ENSG0000(	RGS14	0.646585	2.323535	0.004748
ENSG0000(	ZNF529-AS	0.646331	3.496409	0.000319
ENSG0000(	RDM1P5	0.646244	1.888014	0.012942
ENSG0000(	FNDC3B	0.645985	3.124001	0.000752
ENSG0000(	BAZ1A	0.645872	2.759139	0.001741
ENSG0000(	GLCE	0.645858	1.955597	0.011077
ENSG0000(	UBL7-AS1	0.645815	3.075047	0.000841
ENSG0000(	AC005540.	0.645573	2.476181	0.003341
ENSG0000(	DOCK7	0.645201	2.569725	0.002693
ENSG0000(	ARHGDIA	0.64461	4.088405	8.16E-05
ENSG0000(	ZNF627	0.644607	4.230712	5.88E-05
ENSG0000(	SLC39A11	0.644494	2.767367	0.001709
ENSG0000(	C1QTNF1	0.644349	1.51366	0.030644
ENSG0000(	P4HA2-AS1	0.644291	2.305076	0.004954
ENSG0000(	AL035078.4	0.643869	7.054385	8.82E-08
ENSG0000(	MCRIP1	0.643415	4.26257	5.46E-05

ENSG0000(	LDLRAD2	0.643376	2.409038	0.003899
ENSG0000(	TTC26	0.643087	2.136537	0.007302
ENSG0000(	ATXN3	0.642789	3.521235	0.000301
ENSG0000(	PTPN12	0.642374	3.256679	0.000554
ENSG0000(	NBPF26	0.642192	2.147186	0.007125
ENSG0000(	HSPG2	0.641654	3.196862	0.000636
ENSG0000(	TMX1	0.64079	3.729564	0.000186
ENSG0000(	SLC6A6	0.640722	2.703818	0.001978
ENSG0000(	FAM208A	0.64069	8.577697	2.64E-09
ENSG0000(	ADAMTS12	0.640522	2.302227	0.004986
ENSG0000(	MFSD14B	0.640445	4.333728	4.64E-05
ENSG0000(	ACAP1	0.640077	2.200136	0.006308
ENSG0000(	IFT122	0.639943	1.800767	0.015821
ENSG0000(	TRIM16	0.639641	2.008804	0.009799
ENSG0000(	NXT2	0.639454	2.413624	0.003858
ENSG0000(	GJD3	0.639412	1.603672	0.024907
ENSG0000(	TSPYL4	0.639348	4.285898	5.18E-05
ENSG0000(	FRMD4A	0.63908	3.279447	0.000525
ENSG0000(	VEGFC	0.638953	1.404147	0.039432
ENSG0000(	NSMCE4A	0.638537	2.670834	0.002134
ENSG0000(	MDP1	0.638493	3.701594	0.000199
ENSG0000(	ACTN4	0.638173	7.089452	8.14E-08
ENSG0000(	OCIAD2	0.63765	2.840243	0.001445
ENSG0000(	ZNF267	0.637526	3.30206	0.000499
ENSG0000(	NID2	0.637067	2.041351	0.009092
ENSG0000(	ARF4	0.637002	3.473394	0.000336
ENSG0000(	PDXK	0.636845	3.153517	0.000702
ENSG0000(	PTPN2	0.636123	2.412535	0.003868
ENSG0000(	AC020916.	0.635263	3.377717	0.000419
ENSG0000(	RARB	0.634965	2.165478	0.006832
ENSG0000(	EBF3	0.634938	2.495042	0.003199
ENSG0000(	CNTLN	0.634353	1.387851	0.04094
ENSG0000(	JAG1	0.63414	2.269016	0.005383
ENSG0000(	ETV6	0.634074	2.836316	0.001458
ENSG0000(	SMIM3	0.633752	1.520108	0.030192
ENSG0000(	HACD3	0.633229	3.760077	0.000174
ENSG0000(	AP000426.	0.632646	1.612199	0.024423
ENSG0000(	CBX2	0.632593	1.836804	0.014561
ENSG0000(	ITPR2	0.631789	3.024589	0.000945
ENSG0000(	TM4SF1	0.630844	1.501166	0.031538
ENSG0000(	KIAA0556	0.63068	3.14806	0.000711
ENSG0000(	AC092849.	0.630474	3.09812	0.000798
ENSG0000(	ACVRL1	0.629706	3.125633	0.000749
ENSG0000(	FBXO33	0.629503	2.910877	0.001228
ENSG0000(	AL354950.:	0.628931	1.717077	0.019183
ENSG0000(	ZNF141	0.628641	3.149426	0.000709
ENSG0000(	ARFIP1	0.628636	4.346854	4.50E-05

ENSG0000(	FRRS1	0.62836	2.148414	0.007105
ENSG0000(	ZNF790-AS	0.628293	2.635176	0.002316
ENSG0000(	FAM122C	0.62716	3.642327	0.000228
ENSG0000(	AL807752.(	0.626825	1.936873	0.011565
ENSG0000(	RBPMS	0.626221	1.512909	0.030697
ENSG0000(	SRD5A1	0.626216	4.177405	6.65E-05
ENSG0000(	ZNF707	0.62584	3.555915	0.000278
ENSG0000(	ATP13A3	0.625711	3.009698	0.000978
ENSG0000(	RNF19B	0.625515	3.080523	0.000831
ENSG0000(	NEMP2	0.625402	1.63515	0.023166
ENSG0000(	AL162591.:	0.625128	1.969028	0.010739
ENSG0000(	AC079781.	0.624864	3.701676	0.000199
ENSG0000(	SIDT1	0.624733	1.619933	0.023992
ENSG0000(	AL132780.:	0.624494	3.576222	0.000265
ENSG0000(	TNFSF12	0.624399	2.217154	0.006065
ENSG0000(	TMTC3	0.623631	2.630738	0.00234
ENSG0000(	TRIM2	0.623512	2.067172	0.008567
ENSG0000(	CDC42SE2	0.623307	3.128349	0.000744
ENSG0000(	ARPC4-TTL	0.623082	3.220907	0.000601
ENSG0000(	HDAC7	0.622615	1.585884	0.025949
ENSG0000(	ERP29	0.622521	4.496753	3.19E-05
ENSG0000(	AC142391.	0.62245	2.041208	0.009095
ENSG0000(	AL356475.:	0.622443	1.508443	0.031014
ENSG0000(	LINC00910	0.622127	1.788301	0.016282
ENSG0000(	GOLM1	0.621288	2.264453	0.005439
ENSG0000(	KDSR	0.621165	2.068145	0.008548
ENSG0000(	NBPF1	0.62109	3.693997	0.000202
ENSG0000(	GAS6-DT	0.621042	1.821535	0.015082
ENSG0000(	IMPDH1P1	0.620996	2.085808	0.008207
ENSG0000(	ARHGAP31	0.620959	2.127695	0.007453
ENSG0000(	TRAF3	0.620541	5.672485	2.13E-06
ENSG0000(	SLC25A1	0.62043	1.617674	0.024117
ENSG0000(	SULT1A1	0.620347	2.133903	0.007347
ENSG0000(	ZMIZ1	0.620193	2.987481	0.001029
ENSG0000(	GALNT18	0.620107	1.550452	0.028154
ENSG0000(	AL731566.:	0.619963	1.699919	0.019956
ENSG0000(	ARHGAP42	0.619952	2.265011	0.005432
ENSG0000(	DLG2	0.619679	1.870144	0.013485
ENSG0000(	ZNF700	0.619403	2.462373	0.003448
ENSG0000(	YDJC	0.619375	2.07739	0.008368
ENSG0000(	EXT2	0.619224	2.756289	0.001753
ENSG0000(	UBTD2	0.618764	4.072547	8.46E-05
ENSG0000(	DCUN1D3	0.618643	1.819015	0.01517
ENSG0000(	TBC1D12	0.618384	1.93493	0.011616
ENSG0000(	AC087292.	0.618138	1.679092	0.020937
ENSG0000(	ZNF268	0.617535	3.852027	0.000141
ENSG0000(	HEY1	0.617449	1.493537	0.032097

ENSG0000(	ATP9A	0.616269	2.547918	0.002832
ENSG0000(	ALDH7A1	0.616155	2.104752	0.007857
ENSG0000(	HIVEP3	0.616048	2.581869	0.002619
ENSG0000(	RFTN1	0.614657	3.279935	0.000525
ENSG0000(	HLX	0.614504	2.011884	0.00973
ENSG0000(	IL10RB-DT	0.614138	3.214517	0.00061
ENSG0000(	TSPAN13	0.614121	1.694084	0.020226
ENSG0000(	ABHD16B	0.613122	1.796305	0.015984
ENSG0000(	KDM5B	0.612933	4.19136	6.44E-05
ENSG0000(	SETD9	0.612872	1.800885	0.015817
ENSG0000(	GLYCTK	0.61272	2.54778	0.002833
ENSG0000(	YIPF5	0.612308	4.391772	4.06E-05
ENSG0000(	FDXR	0.612269	1.395475	0.040228
ENSG0000(	TVP23C	0.612071	2.0856	0.008211
ENSG0000(	PIGCP1	0.611453	1.886266	0.012994
ENSG0000(	NFATC2	0.611449	1.917902	0.012081
ENSG0000(	GLIPR1	0.611131	2.609389	0.002458
ENSG0000(	ARMCX6	0.610792	2.698199	0.002004
ENSG0000(	RF01957	0.610722	1.635321	0.023157
ENSG0000(	RF01956	0.610529	1.508912	0.03098
ENSG0000(	ECSCR	0.610481	1.92936	0.011766
ENSG0000(	DYNC2LI1	0.610446	1.603767	0.024902
ENSG0000(	ARHGEF25	0.610332	1.78966	0.016231
ENSG0000(	HSPA12A	0.609785	1.970584	0.010701
ENSG0000(	PCAT19	0.609553	1.360001	0.043651
ENSG0000(	WDR19	0.609366	1.42123	0.037911
ENSG0000(	AC106786.	0.608754	3.253109	0.000558
ENSG0000(	C9orf16	0.60871	4.780863	1.66E-05
ENSG0000(	HIF1A-AS1	0.608361	1.34493	0.045193
ENSG0000(	RF01955	0.60825	1.478782	0.033206
ENSG0000(	SERF2	0.608036	6.390523	4.07E-07
ENSG0000(	AL136295.:	0.607837	2.400995	0.003972
ENSG0000(	CD151	0.607583	2.719914	0.001906
ENSG0000(	TMEM214	0.60688	4.576116	2.65E-05
ENSG0000(	ATF7IP2	0.606849	1.723739	0.018891
ENSG0000(	KIF9	0.606528	1.731905	0.018539
ENSG0000(	C4orf3	0.606439	5.596458	2.53E-06
ENSG0000(	ARMC7	0.606245	3.453518	0.000352
ENSG0000(	FAM162B	0.606118	1.455038	0.035072
ENSG0000(	ELMOD2	0.605557	5.01768	9.60E-06
ENSG0000(	AC127024.	0.605514	1.535432	0.029145
ENSG0000(	C19orf71	0.605029	1.903796	0.01248
ENSG0000(	CEP57L1	0.604787	4.302565	4.98E-05
ENSG0000(	AC124319.	0.604778	3.353406	0.000443
ENSG0000(	AL445685.:	0.604553	2.551486	0.002809
ENSG0000(	EMC3-AS1	0.604247	1.333863	0.046359
ENSG0000(	ARHGAP12	0.604078	6.888956	1.29E-07

ENSG0000(	C8orf37	0.603848	1.909337	0.012321
ENSG0000(	ZNF385B	0.603719	1.385442	0.041168
ENSG0000(	MGAT1	0.60349	3.550878	0.000281
ENSG0000(	ZDHHC1	0.603332	2.013981	0.009683
ENSG0000(	RNF125	0.603199	1.761325	0.017325
ENSG0000(	EDNRA	0.602912	1.530253	0.029495
ENSG0000(	OLMALINC	0.602749	1.736878	0.018328
ENSG0000(	ZNF512	0.602656	4.574614	2.66E-05
ENSG0000(	HPCAL1	0.602369	2.736855	0.001833
ENSG0000(	ATP8B1	0.602367	1.954445	0.011106
ENSG0000(	ZDHHC18	0.602289	5.247281	5.66E-06
ENSG0000(	SRI	0.602171	3.603444	0.000249
ENSG0000(	AKAP11	0.601789	3.983638	0.000104
ENSG0000(	C12orf76	0.601344	4.082522	8.27E-05
ENSG0000(	RNF135	0.600804	1.672301	0.021267
ENSG0000(	MAP4K1	0.600653	1.340512	0.045655
ENSG0000(	RAI2	0.600165	1.529798	0.029526
ENSG0000(	RAB35	0.600072	6.631172	2.34E-07
ENSG0000(	CHEK1	0.60004	1.731905	0.018539
ENSG0000(	MIR6080	0.599972	1.323585	0.047469
ENSG0000(	MAP3K14-/	0.599939	2.918523	0.001206
ENSG0000(	STK39	0.599765	2.357203	0.004393
ENSG0000(	ISYNA1	0.59968	3.064656	0.000862
ENSG0000(	AC008906.	0.59959	2.617307	0.002414
ENSG0000(	PLEKHG2	0.59937	2.304505	0.00496
ENSG0000(	HEXB	0.599301	1.919432	0.012038
ENSG0000(	ARPC4	0.599236	4.243424	5.71E-05
ENSG0000(	RTCA-AS1	0.598836	1.380908	0.0416
ENSG0000(	WLS	0.598267	2.963523	0.001088
ENSG0000(	MAST3	0.597869	1.625928	0.023663
ENSG0000(	PALM2-AK/	0.597357	5.570654	2.69E-06
ENSG0000(	HEG1	0.596734	2.452947	0.003524
ENSG0000(	AC004466.	0.595991	1.310887	0.048878
ENSG0000(	SDCBP2-AS	0.595219	2.601886	0.002501
ENSG0000(	TMEM44	0.594699	1.748098	0.017861
ENSG0000(	KLF11	0.593917	1.740565	0.018173
ENSG0000(	AKAP2	0.593836	5.412156	3.87E-06
ENSG0000(	ZNF136	0.593783	2.647494	0.002252
ENSG0000(	HMGB2	0.593696	3.237305	0.000579
ENSG0000(	GLA	0.593037	3.249504	0.000563
ENSG0000(	SRD5A3	0.592956	2.516218	0.003046
ENSG0000(	RPL26L1	0.592788	3.349711	0.000447
ENSG0000(	SEMA4A	0.591423	1.467151	0.034107
ENSG0000(	CDKN2C	0.591081	2.561024	0.002748
ENSG0000(	NBL1	0.591007	2.67706	0.002103
ENSG0000(	CEMP1	0.590888	1.63983	0.022918
ENSG0000(	PCED1A	0.590038	1.889136	0.012908

ENSG0000(	SLC31A1	0.589971	3.047192	0.000897
ENSG0000(	HAUS4	0.589763	4.969589	1.07E-05
ENSG0000(	CTSA	0.589679	4.679742	2.09E-05
ENSG0000(	FANCF	0.589584	1.619096	0.024038
ENSG0000(	AP5Z1	0.589288	2.842012	0.001439
ENSG0000(	MPP2	0.588964	1.504611	0.031289
ENSG0000(	FLNB-AS1	0.588107	1.635894	0.023126
ENSG0000(	AC144548.	0.587545	5.718167	1.91E-06
ENSG0000(	AF111169.	0.586494	2.116525	0.007647
ENSG0000(	KNTC1	0.585886	2.317438	0.004815
ENSG0000(	HM13	0.585525	4.006319	9.86E-05
ENSG0000(	CEP290	0.585443	1.668201	0.021468
ENSG0000(	SEC61A1	0.585436	4.873502	1.34E-05
ENSG0000(	CUL1	-0.58505	4.928967	1.18E-05
ENSG0000(	NKX3-1	-0.58518	1.797904	0.015926
ENSG0000(	FZD7	-0.58528	2.130968	0.007397
ENSG0000(	DHRS11	-0.58536	2.745451	0.001797
ENSG0000(	MAP3K10	-0.5855	2.45517	0.003506
ENSG0000(	FBRSL1	-0.58555	4.10311	7.89E-05
ENSG0000(	AC006213.	-0.58586	1.988932	0.010258
ENSG0000(	AC004854.	-0.58639	1.422189	0.037828
ENSG0000(	AC009065.	-0.58639	3.41596	0.000384
ENSG0000(	AC020765.	-0.5864	3.893025	0.000128
ENSG0000(	AC067852.	-0.58643	1.395793	0.040198
ENSG0000(	DCAF8	-0.5865	4.213064	6.12E-05
ENSG0000(	AL133216.:	-0.58687	1.839944	0.014456
ENSG0000(	TSSC4	-0.58724	6.505302	3.12E-07
ENSG0000(	TAF7	-0.58766	7.757038	1.75E-08
ENSG0000(	MROH7-TT	-0.58823	3.941487	0.000114
ENSG0000(	ANAPC15	-0.58827	3.00037	0.000999
ENSG0000(	NBAS	-0.58839	4.849982	1.41E-05
ENSG0000(	AC130462.	-0.58977	2.931573	0.001171
ENSG0000(	NDUFAF5	-0.59007	2.370136	0.004264
ENSG0000(	HEBP2	-0.5902	4.773313	1.69E-05
ENSG0000(	RPA1	-0.59037	6.134436	7.34E-07
ENSG0000(	TOGARAM	-0.59061	3.386012	0.000411
ENSG0000(	ATF4	-0.5907	4.533042	2.93E-05
ENSG0000(	SENP8	-0.59075	1.85783	0.013873
ENSG0000(	LIMCH1	-0.59106	1.530792	0.029458
ENSG0000(	FOXJ3	-0.59142	10.67001	2.14E-11
ENSG0000(	AC005726.	-0.5915	2.445586	0.003584
ENSG0000(	RWDD4	-0.59176	3.851055	0.000141
ENSG0000(	PCYOX1	-0.59188	3.295045	0.000507
ENSG0000(	TPCN1	-0.59191	4.431589	3.70E-05
ENSG0000(	SLFNL1-AS:	-0.59207	4.239806	5.76E-05
ENSG0000(	RERE	-0.59267	2.791461	0.001616
ENSG0000(	RRAGD	-0.59269	1.553507	0.027957

ENSG0000(	ANO1-AS1	-0.59269	1.742902	0.018076
ENSG0000(	TANGO6	-0.59288	7.768067	1.71E-08
ENSG0000(	DUSP28	-0.59289	1.354471	0.044211
ENSG0000(	SRRD	-0.59291	4.085643	8.21E-05
ENSG0000(	PKD1	-0.59311	3.403097	0.000395
ENSG0000(	AC068790.	-0.59332	3.620453	0.00024
ENSG0000(	AC017083.	-0.59339	4.538071	2.90E-05
ENSG0000(	AC007191.	-0.59377	2.096091	0.008015
ENSG0000(	POLRMT	-0.59381	3.174561	0.000669
ENSG0000(	SMOC1	-0.59382	1.715487	0.019254
ENSG0000(	SFXN4	-0.59419	2.739764	0.001821
ENSG0000(	TXNL1	-0.5945	3.547784	0.000283
ENSG0000(	AC126755.	-0.59456	1.539934	0.028845
ENSG0000(	ATG9A	-0.59456	2.731583	0.001855
ENSG0000(	AP001107.	-0.59471	2.008575	0.009804
ENSG0000(	ADRM1	-0.59476	4.089556	8.14E-05
ENSG0000(	MAPKAPK2	-0.5948	2.683542	0.002072
ENSG0000(	TIMM23B	-0.59507	3.479126	0.000332
ENSG0000(	USP19	-0.59515	4.256769	5.54E-05
ENSG0000(	TMCC1	-0.59523	3.10951	0.000777
ENSG0000(	BEND5	-0.59554	3.078964	0.000834
ENSG0000(	MT-TS1	-0.59566	1.809487	0.015506
ENSG0000(	C15orf61	-0.59567	1.782007	0.016519
ENSG0000(	LONRF1	-0.59683	2.940247	0.001148
ENSG0000(	BECN1	-0.59687	13.55097	2.81E-14
ENSG0000(	HAGH	-0.59695	2.502965	0.003141
ENSG0000(	AL031595.:	-0.59713	2.945798	0.001133
ENSG0000(	AC006942.	-0.59731	2.970678	0.00107
ENSG0000(	COL7A1	-0.59738	2.080162	0.008315
ENSG0000(	ALKBH7	-0.59799	2.50448	0.00313
ENSG0000(	PARM1	-0.598	2.245613	0.005681
ENSG0000(	LRRC47	-0.59851	3.292493	0.00051
ENSG0000(	SMTN	-0.59858	3.2905	0.000512
ENSG0000(	ESRRA	-0.59859	1.608307	0.024643
ENSG0000(	DEK	-0.59894	5.928136	1.18E-06
ENSG0000(	TEF	-0.59929	2.0532	0.008847
ENSG0000(	PCBP1-AS1	-0.59935	5.681992	2.08E-06
ENSG0000(	TRMT1L	-0.59959	2.654278	0.002217
ENSG0000(	POLE	-0.59977	6.047902	8.96E-07
ENSG0000(	ENSA	-0.60008	6.495529	3.19E-07
ENSG0000(	ZNF524	-0.60053	5.952437	1.12E-06
ENSG0000(	AC087289.	-0.60066	2.76166	0.001731
ENSG0000(	DPP8	-0.60084	3.311456	0.000488
ENSG0000(	TCERG1	-0.6015	8.708619	1.96E-09
ENSG0000(	SLC2A11	-0.60153	3.174103	0.00067
ENSG0000(	SPRYD7	-0.60159	2.511262	0.003081
ENSG0000(	AC087190.	-0.60208	3.390105	0.000407

ENSG0000(	PLEKHB1	-0.60289	1.396898	0.040096
ENSG0000(	AC087289.	-0.60291	5.583075	2.61E-06
ENSG0000(	GSE1	-0.60331	4.238441	5.78E-05
ENSG0000(	SUN1	-0.60338	11.16005	6.92E-12
ENSG0000(	AGAP2	-0.6039	3.136068	0.000731
ENSG0000(	HS6ST1	-0.60417	4.196698	6.36E-05
ENSG0000(	CAB39	-0.60501	2.326012	0.004721
ENSG0000(	PTOV1-AS2	-0.60534	9.252976	5.59E-10
ENSG0000(	SPATA2	-0.60603	3.752457	0.000177
ENSG0000(	PNRC1	-0.60654	3.506765	0.000311
ENSG0000(	DNAJC24	-0.60703	3.956055	0.000111
ENSG0000(	PDLIM7	-0.6075	1.879529	0.013197
ENSG0000(	AES	-0.60799	3.116304	0.000765
ENSG0000(	TPI1	-0.60813	1.48386	0.03282
ENSG0000(	TEX2	-0.60817	3.303542	0.000497
ENSG0000(	NEK9	-0.60835	7.314286	4.85E-08
ENSG0000(	AL139011.1	-0.6088	2.401575	0.003967
ENSG0000(	AC124283.	-0.60883	3.777606	0.000167
ENSG0000(	AL354696.1	-0.60905	2.51892	0.003027
ENSG0000(	ATXN1	-0.60913	4.210228	6.16E-05
ENSG0000(	RUSC2	-0.60916	3.606783	0.000247
ENSG0000(	ORAI1	-0.60931	1.521843	0.030072
ENSG0000(	SYPL1	-0.60958	7.67924	2.09E-08
ENSG0000(	SUCLG2-AS	-0.61004	1.676192	0.021077
ENSG0000(	MLST8	-0.61005	5.434201	3.68E-06
ENSG0000(	AC037459.	-0.6102	3.067062	0.000857
ENSG0000(	RHOF	-0.61022	5.668689	2.14E-06
ENSG0000(	LIPT2	-0.61031	1.568481	0.02701
ENSG0000(	MVB12B	-0.61066	3.033222	0.000926
ENSG0000(	MPP5	-0.61085	4.553548	2.80E-05
ENSG0000(	AC137630.	-0.61107	4.313627	4.86E-05
ENSG0000(	RNA5SP392	-0.61108	1.797093	0.015955
ENSG0000(	DMGDH	-0.61109	1.676877	0.021044
ENSG0000(	TPP2	-0.61124	5.427213	3.74E-06
ENSG0000(	AL136980.1	-0.61193	1.859807	0.01381
ENSG0000(	GDE1	-0.61197	3.95453	0.000111
ENSG0000(	PNMT	-0.61212	1.312972	0.048644
ENSG0000(	AL590399.1	-0.61217	1.421719	0.037869
ENSG0000(	RFX3-AS1	-0.61246	2.293603	0.005086
ENSG0000(	MTFR1L	-0.61283	2.495119	0.003198
ENSG0000(	PDE7A	-0.61325	2.093565	0.008062
ENSG0000(	AGPAT3	-0.61351	2.60275	0.002496
ENSG0000(	TPRKB	-0.61372	3.401403	0.000397
ENSG0000(	AL138724.1	-0.61387	3.153268	0.000703
ENSG0000(	RAVER2	-0.61408	2.022441	0.009496
ENSG0000(	CNNM2	-0.61414	4.143851	7.18E-05
ENSG0000(	KPNA1	-0.61435	2.838494	0.00145

ENSG0000(	SRF	-0.61458	2.935727	0.00116
ENSG0000(	AC004980.	-0.6146	2.562099	0.002741
ENSG0000(	AC003681.	-0.61483	5.565271	2.72E-06
ENSG0000(	FAN1	-0.61543	5.413467	3.86E-06
ENSG0000(	STIM1	-0.61544	3.487924	0.000325
ENSG0000(	LIN9	-0.61572	2.714096	0.001932
ENSG0000(	PEX5	-0.61617	6.293819	5.08E-07
ENSG0000(	L2HGDH	-0.61646	1.936009	0.011588
ENSG0000(	AC015688.	-0.61659	2.81407	0.001534
ENSG0000(	CDK16	-0.61662	2.883403	0.001308
ENSG0000(	RAD52	-0.61665	4.132337	7.37E-05
ENSG0000(	AMIGO3	-0.61673	4.47948	3.32E-05
ENSG0000(	DDI2	-0.61682	3.352525	0.000444
ENSG0000(	AC092376.	-0.61709	1.547989	0.028315
ENSG0000(	AL353708.:	-0.61761	2.993311	0.001016
ENSG0000(	FGFRL1	-0.61774	2.227275	0.005925
ENSG0000(	DDIT4	-0.61791	1.566489	0.027134
ENSG0000(	SSH1	-0.61792	4.521405	3.01E-05
ENSG0000(	KAT6B	-0.61809	6.81257	1.54E-07
ENSG0000(	DCAF5	-0.61815	4.29103	5.12E-05
ENSG0000(	DNAJA3	-0.61862	2.582683	0.002614
ENSG0000(	AC005944.	-0.61895	3.178677	0.000663
ENSG0000(	DDIT4-AS1	-0.61898	1.529811	0.029525
ENSG0000(	ZFAND3	-0.61913	4.924348	1.19E-05
ENSG0000(	SPDYE2	-0.61959	1.320958	0.047758
ENSG0000(	ACTR1B	-0.61988	10.53214	2.94E-11
ENSG0000(	FOXO1	-0.62038	1.306626	0.04936
ENSG0000(	QKI	-0.62089	3.389834	0.000408
ENSG0000(	ZNF518B	-0.62102	5.355476	4.41E-06
ENSG0000(	MAST2	-0.62172	2.24385	0.005704
ENSG0000(	TAF4	-0.6218	5.704384	1.98E-06
ENSG0000(	AC129492.	-0.62187	5.038079	9.16E-06
ENSG0000(	HSF1	-0.62248	5.057947	8.75E-06
ENSG0000(	TUBG1	-0.62345	1.971239	0.010685
ENSG0000(	MIR6800	-0.62441	2.570459	0.002689
ENSG0000(	ZNF658B	-0.62442	2.308132	0.004919
ENSG0000(	PPP3CB-AS	-0.62457	1.376292	0.042044
ENSG0000(	MAPKAPK3	-0.62475	1.623897	0.023774
ENSG0000(	KIAA0232	-0.62484	2.299861	0.005013
ENSG0000(	BRICD5	-0.62538	3.368757	0.000428
ENSG0000(	CRKL	-0.62558	5.066581	8.58E-06
ENSG0000(	GMPPB	-0.62565	7.41676	3.83E-08
ENSG0000(	PBX1	-0.62636	5.365895	4.31E-06
ENSG0000(	SLC25A11	-0.62673	1.617993	0.024099
ENSG0000(	LPIN1	-0.6269	1.529718	0.029531
ENSG0000(	Z98884.1	-0.62692	1.417501	0.038238
ENSG0000(	TNNI3K	-0.62741	2.125673	0.007487

ENSG0000(	PAIP1	-0.62759	4.333511	4.64E-05
ENSG0000(	ZDHHC2	-0.62763	2.518501	0.00303
ENSG0000(	C1orf21	-0.62774	4.061764	8.67E-05
ENSG0000(	SNRPN	-0.62793	2.554561	0.002789
ENSG0000(	ATP11A	-0.62807	3.76497	0.000172
ENSG0000(	STAT5B	-0.6281	6.884331	1.31E-07
ENSG0000(	AC093297.	-0.6284	4.339368	4.58E-05
ENSG0000(	AIFM1	-0.62884	2.908262	0.001235
ENSG0000(	EEPD1	-0.62917	2.202788	0.006269
ENSG0000(	COL4A5	-0.6293	1.65828	0.021964
ENSG0000(	HDLBP	-0.6295	5.259839	5.50E-06
ENSG0000(	MACO1	-0.62971	6.775947	1.68E-07
ENSG0000(	MRS2	-0.62982	2.189569	0.006463
ENSG0000(	RPL4P4	-0.63042	4.272414	5.34E-05
ENSG0000(	SNURF	-0.63063	2.540478	0.002881
ENSG0000(	TEAD3	-0.6307	4.020588	9.54E-05
ENSG0000(	AL035448.:	-0.63079	1.684323	0.020686
ENSG0000(	RAD54L	-0.63104	3.085699	0.000821
ENSG0000(	DYNLL2	-0.6318	3.263225	0.000545
ENSG0000(	STK11	-0.63187	4.555969	2.78E-05
ENSG0000(	UBL4A	-0.632	3.446979	0.000357
ENSG0000(	REEP2	-0.63213	1.43749	0.036518
ENSG0000(	FGGY	-0.63232	2.397227	0.004007
ENSG0000(	AC004805.	-0.63267	2.006686	0.009847
ENSG0000(	ANOS1	-0.63283	1.706196	0.01967
ENSG0000(	ARIH2	-0.63299	3.841866	0.000144
ENSG0000(	EEF1D	-0.63322	11.63161	2.34E-12
ENSG0000(	YWHAG	-0.63344	2.548405	0.002829
ENSG0000(	COQ7	-0.63391	3.743945	0.00018
ENSG0000(	CLYBL	-0.63398	2.536938	0.002904
ENSG0000(	SYS1-DBNE	-0.63483	2.629591	0.002346
ENSG0000(	BCAS2	-0.63539	8.283939	5.20E-09
ENSG0000(	TMEM88	-0.63552	1.363747	0.043277
ENSG0000(	HADH	-0.63553	2.446889	0.003574
ENSG0000(	AL031316.:	-0.63576	1.377103	0.041966
ENSG0000(	TAB2	-0.63598	7.035128	9.22E-08
ENSG0000(	BTG2	-0.63601	1.36445	0.043207
ENSG0000(	AR	-0.63605	4.371782	4.25E-05
ENSG0000(	SNX19	-0.63636	5.652207	2.23E-06
ENSG0000(	RPL4	-0.63661	7.537531	2.90E-08
ENSG0000(	THAP9	-0.63682	2.70961	0.001952
ENSG0000(	KAT2B	-0.6371	3.044668	0.000902
ENSG0000(	FGF7P6	-0.63729	1.483078	0.032879
ENSG0000(	AL358937.:	-0.63792	1.661072	0.021824
ENSG0000(	OSBP	-0.63805	4.702157	1.99E-05
ENSG0000(	SRRM2	-0.63818	4.711824	1.94E-05
ENSG0000(	CDC34	-0.63818	4.04168	9.08E-05

ENSG0000(	CUL4A	-0.63884	3.14806	0.000711
ENSG0000(	CS	-0.63899	1.915037	0.012161
ENSG0000(	FGF13	-0.63972	2.69444	0.002021
ENSG0000(	PPP3CC	-0.64008	2.851373	0.001408
ENSG0000(	PRRG1	-0.64013	2.295265	0.005067
ENSG0000(	AFF1	-0.64026	5.490265	3.23E-06
ENSG0000(	AL355987.4	-0.64038	2.415551	0.003841
ENSG0000(	POLR3E	-0.64078	5.112774	7.71E-06
ENSG0000(	VEZF1	-0.64081	9.009063	9.79E-10
ENSG0000(	STAU2	-0.64082	2.044977	0.009016
ENSG0000(	NUDT4	-0.64115	2.660251	0.002186
ENSG0000(	CBLL1	-0.64118	5.601247	2.50E-06
ENSG0000(	AC073111.	-0.64127	2.451934	0.003532
ENSG0000(	AC098582.	-0.64128	2.985501	0.001034
ENSG0000(	CALM3	-0.64148	9.579189	2.64E-10
ENSG0000(	PCBP1	-0.64155	5.255795	5.55E-06
ENSG0000(	IPP	-0.64202	4.077419	8.37E-05
ENSG0000(	AC025419.	-0.64245	2.238938	0.005768
ENSG0000(	AC090181.	-0.64381	1.756644	0.017513
ENSG0000(	FBXW5	-0.64388	3.676343	0.000211
ENSG0000(	AC027307.	-0.64408	1.601849	0.025012
ENSG0000(	DBNDD2	-0.64422	2.615173	0.002426
ENSG0000(	NFATC1	-0.64436	3.838443	0.000145
ENSG0000(	PARVB	-0.64473	2.452426	0.003528
ENSG0000(	H2AFV	-0.64521	8.997643	1.01E-09
ENSG0000(	TMEM108	-0.64531	1.635939	0.023124
ENSG0000(	ABCC9	-0.6455	2.823571	0.001501
ENSG0000(	ABCA5	-0.64578	2.835118	0.001462
ENSG0000(	FLJ37035	-0.6462	2.470731	0.003383
ENSG0000(	CNBD2	-0.64713	2.090034	0.008128
ENSG0000(	MTMR10	-0.6478	6.27746	5.28E-07
ENSG0000(	AC009065.	-0.64789	2.698199	0.002004
ENSG0000(	MYO18A	-0.64859	1.440399	0.036274
ENSG0000(	AC138811.	-0.64871	1.460074	0.034668
ENSG0000(	AC079848.	-0.6489	1.383404	0.041362
ENSG0000(	HDAC4	-0.64914	4.079575	8.33E-05
ENSG0000(	DOHH	-0.64929	4.514716	3.06E-05
ENSG0000(	MRPL38	-0.64953	5.084829	8.23E-06
ENSG0000(	NEURL4	-0.64995	6.629781	2.35E-07
ENSG0000(	TMEM245	-0.64999	5.688208	2.05E-06
ENSG0000(	MAP4	-0.65015	2.260942	0.005484
ENSG0000(	COX5A	-0.65054	1.40206	0.039622
ENSG0000(	AC087289.	-0.65068	5.363041	4.33E-06
ENSG0000(	DTNA	-0.65105	1.361094	0.043542
ENSG0000(	AC067931.	-0.65114	1.590308	0.025686
ENSG0000(	AC124312.	-0.65119	2.636899	0.002307
ENSG0000(	EPN1	-0.65167	6.718915	1.91E-07

ENSG0000(	GRSF1	-0.65175	2.060285	0.008704
ENSG0000(	NPEPPS	-0.65176	6.292034	5.10E-07
ENSG0000(	SETD3	-0.65191	2.956466	0.001105
ENSG0000(	AC005702.	-0.65203	2.665797	0.002159
ENSG0000(	EVI5L	-0.65258	3.129376	0.000742
ENSG0000(	STXBP5	-0.65287	4.992092	1.02E-05
ENSG0000(	AMFR	-0.65382	5.600703	2.51E-06
ENSG0000(	DNAJC21	-0.65395	4.470544	3.38E-05
ENSG0000(	SCMH1	-0.65453	6.269981	5.37E-07
ENSG0000(	HBEGF	-0.65453	1.882556	0.013105
ENSG0000(	AL136295.:	-0.65484	1.60625	0.02476
ENSG0000(	RNF11	-0.65488	6.209219	6.18E-07
ENSG0000(	ZC3H8	-0.65563	6.572826	2.67E-07
ENSG0000(	BRPF3	-0.65604	5.60793	2.47E-06
ENSG0000(	AP001000.	-0.65609	2.187929	0.006487
ENSG0000(	LINC01963	-0.65624	2.078747	0.008342
ENSG0000(	CRY2	-0.65631	3.096216	0.000801
ENSG0000(	ASPSCR1	-0.65633	11.0643	8.62E-12
ENSG0000(	PTP4A2	-0.65661	8.478112	3.33E-09
ENSG0000(	AC090587.	-0.65705	1.375436	0.042127
ENSG0000(	THAP4	-0.65717	5.362457	4.34E-06
ENSG0000(	GOLGA6L9	-0.65777	2.272704	0.005337
ENSG0000(	ARIH2OS	-0.6581	2.655439	0.002211
ENSG0000(	KLHL11	-0.65937	1.751798	0.017709
ENSG0000(	ZNF768	-0.65964	3.110672	0.000775
ENSG0000(	EIF3J	-0.66005	4.039062	9.14E-05
ENSG0000(	RLIMP2	-0.66028	2.678628	0.002096
ENSG0000(	DHRS7	-0.6607	3.10951	0.000777
ENSG0000(	ING2	-0.66083	2.531078	0.002944
ENSG0000(	BEND7	-0.6609	5.956854	1.10E-06
ENSG0000(	ANKRD9	-0.66115	1.678279	0.020976
ENSG0000(	AC018766.	-0.66176	4.460859	3.46E-05
ENSG0000(	TIAF1	-0.66206	1.92394	0.011914
ENSG0000(	UBE2D4	-0.66216	3.036872	0.000919
ENSG0000(	FAM118A	-0.66217	1.724617	0.018853
ENSG0000(	AC067930.	-0.66226	9.657385	2.20E-10
ENSG0000(	AC009065.	-0.66228	3.002681	0.000994
ENSG0000(	LINC-PINT	-0.66255	1.816115	0.015272
ENSG0000(	AC092140.	-0.6627	2.522407	0.003003
ENSG0000(	WDR97	-0.66306	1.803985	0.015704
ENSG0000(	EFR3B	-0.66306	1.604996	0.024832
ENSG0000(	AC009137.	-0.66393	2.768518	0.001704
ENSG0000(	MCRIP2	-0.66393	2.044469	0.009027
ENSG0000(	RNPC3	-0.66402	5.835225	1.46E-06
ENSG0000(	GNAL	-0.66402	4.362386	4.34E-05
ENSG0000(	HDAC11	-0.66475	2.61123	0.002448
ENSG0000(	STBD1	-0.66508	1.566555	0.02713

ENSG0000(	C14orf39	-0.66534	1.360924	0.043559
ENSG0000(	CFAP126	-0.66558	1.413585	0.038585
ENSG0000(	SAMD4A	-0.66562	3.553278	0.00028
ENSG0000(	FBXW4	-0.66564	4.860063	1.38E-05
ENSG0000(	CTH	-0.66579	1.477608	0.033296
ENSG0000(	HMGA1	-0.66589	2.269332	0.005379
ENSG0000(	GINS2	-0.6663	1.783576	0.01646
ENSG0000(	HERC2P2	-0.66634	2.519458	0.003024
ENSG0000(	AC009955.	-0.6664	2.711737	0.001942
ENSG0000(	PRPF39	-0.66645	3.549164	0.000282
ENSG0000(	AL109811.:	-0.66689	3.811944	0.000154
ENSG0000(	TECR	-0.6669	2.551486	0.002809
ENSG0000(	PPM1A	-0.66741	4.98815	1.03E-05
ENSG0000(	GCNT4	-0.66742	1.564093	0.027284
ENSG0000(	MIR6848	-0.66754	3.411453	0.000388
ENSG0000(	TESK1	-0.66762	5.277097	5.28E-06
ENSG0000(	MEF2D	-0.66845	2.77987	0.00166
ENSG0000(	AC067930.	-0.66865	9.514908	3.06E-10
ENSG0000(	CDV3	-0.66872	8.382848	4.14E-09
ENSG0000(	FAM47E-ST	-0.66872	1.555445	0.027833
ENSG0000(	EIF3C	-0.66886	2.5765	0.002652
ENSG0000(	SLC2A4RG	-0.66904	6.713578	1.93E-07
ENSG0000(	TMEM70	-0.66919	1.30085	0.050021
ENSG0000(	MID2	-0.66951	2.797288	0.001595
ENSG0000(	DECR2	-0.67049	5.460368	3.46E-06
ENSG0000(	ZNF710	-0.67073	2.30436	0.004962
ENSG0000(	ANKRD34A	-0.67124	1.827246	0.014885
ENSG0000(	DAB2IP	-0.67127	4.653279	2.22E-05
ENSG0000(	SLC1A4	-0.6713	1.563714	0.027308
ENSG0000(	AC026954.	-0.67158	9.582626	2.61E-10
ENSG0000(	MRPL23	-0.67193	8.308126	4.92E-09
ENSG0000(	AC025682.	-0.67261	7.59584	2.54E-08
ENSG0000(	MITF	-0.67268	3.774095	0.000168
ENSG0000(	PLCL2	-0.67334	3.056569	0.000878
ENSG0000(	RAPGEF1	-0.67397	2.931137	0.001172
ENSG0000(	LRIG1	-0.67403	3.5037	0.000314
ENSG0000(	SYNPO2	-0.6741	1.418364	0.038162
ENSG0000(	PPM1B	-0.67416	2.640438	0.002289
ENSG0000(	PDE4A	-0.67416	3.814684	0.000153
ENSG0000(	VPS72	-0.67422	1.481609	0.032991
ENSG0000(	AC090589.	-0.67497	2.972758	0.001065
ENSG0000(	CDK11B	-0.67497	7.925012	1.19E-08
ENSG0000(	AL442003.:	-0.67513	2.78552	0.001639
ENSG0000(	AC020978.	-0.6754	1.793978	0.01607
ENSG0000(	GPS1	-0.6755	7.420228	3.80E-08
ENSG0000(	EIF1	-0.67577	7.621756	2.39E-08
ENSG0000(	AL118516.:	-0.67701	2.022305	0.009499

ENSG0000(	TMEM161I	-0.67736	2.988918	0.001026
ENSG0000(	SYNE2	-0.67741	2.833174	0.001468
ENSG0000(	AC093503.	-0.67872	8.316763	4.82E-09
ENSG0000(	AC105339.	-0.6789	2.6889	0.002047
ENSG0000(	MCMDC2	-0.67956	4.36527	4.31E-05
ENSG0000(	LMNB2	-0.67957	8.616856	2.42E-09
ENSG0000(	RBM33	-0.67964	5.629451	2.35E-06
ENSG0000(	RPLP1	-0.67973	5.335162	4.62E-06
ENSG0000(	LMTK2	-0.67993	3.517565	0.000304
ENSG0000(	FOXP2	-0.68	1.559012	0.027605
ENSG0000(	PHF10	-0.68002	6.438622	3.64E-07
ENSG0000(	AL449266.:	-0.6801	3.872176	0.000134
ENSG0000(	THRB	-0.68058	4.332822	4.65E-05
ENSG0000(	KLHL21	-0.68108	2.389042	0.004083
ENSG0000(	TOM1L2	-0.68134	3.803283	0.000157
ENSG0000(	SLC41A3	-0.68158	5.523614	2.99E-06
ENSG0000(	TMEM161/	-0.68159	3.334494	0.000463
ENSG0000(	ARIH1	-0.68172	6.095344	8.03E-07
ENSG0000(	RCOR3	-0.68224	7.254081	5.57E-08
ENSG0000(	HDAC5	-0.68239	8.808906	1.55E-09
ENSG0000(	MIR4687	-0.68242	2.678251	0.002098
ENSG0000(	ESRP2	-0.68347	3.285086	0.000519
ENSG0000(	SGF29	-0.68353	7.842098	1.44E-08
ENSG0000(	KIF28P	-0.68383	2.929157	0.001177
ENSG0000(	TMEM56	-0.6847	1.499293	0.031674
ENSG0000(	BARD1	-0.68503	2.773069	0.001686
ENSG0000(	SEMA4D	-0.6854	1.766095	0.017136
ENSG0000(	HOXA10	-0.68555	2.909333	0.001232
ENSG0000(	TLN2	-0.68578	3.901512	0.000125
ENSG0000(	MPP7	-0.6867	2.565717	0.002718
ENSG0000(	NDUFB10	-0.68694	2.027575	0.009385
ENSG0000(	AC010136.	-0.68698	1.560417	0.027516
ENSG0000(	IDE	-0.68703	2.807416	0.001558
ENSG0000(	MIR6511B:	-0.68719	2.940964	0.001146
ENSG0000(	MAP2K7	-0.68754	6.234048	5.83E-07
ENSG0000(	DSE	-0.68759	4.450974	3.54E-05
ENSG0000(	TMEM202-	-0.68768	2.40539	0.003932
ENSG0000(	ZBTB43	-0.688	2.995885	0.00101
ENSG0000(	UBE3A	-0.68816	4.215788	6.08E-05
ENSG0000(	AL020996.:	-0.68833	2.976527	0.001056
ENSG0000(	AKTIP	-0.68849	6.6628	2.17E-07
ENSG0000(	HIPK2	-0.68916	1.856189	0.013925
ENSG0000(	ZNF341	-0.68918	4.743498	1.81E-05
ENSG0000(	FXR1	-0.68927	1.978034	0.010519
ENSG0000(	AUNIP	-0.6895	3.055909	0.000879
ENSG0000(	H1F0	-0.68959	7.507013	3.11E-08
ENSG0000(	LAMB3	-0.69013	1.899082	0.012616

ENSG0000(	AL354920.:	-0.69039	3.328265	0.00047
ENSG0000(	KDM3B	-0.69042	9.201807	6.28E-10
ENSG0000(	CCSER2	-0.6907	3.323387	0.000475
ENSG0000(	CUL3	-0.69087	3.874509	0.000134
ENSG0000(	FBXO3	-0.69105	3.249558	0.000563
ENSG0000(	ACADL	-0.6911	3.857794	0.000139
ENSG0000(	TAPT1-AS1	-0.69151	3.087199	0.000818
ENSG0000(	LONRF3	-0.69151	1.939491	0.011495
ENSG0000(	CCDC9B	-0.69154	1.804498	0.015686
ENSG0000(	SYT2	-0.69163	1.969586	0.010725
ENSG0000(	EPS15L1	-0.69169	3.905556	0.000124
ENSG0000(	AC098650.	-0.69238	3.742468	0.000181
ENSG0000(	AC097662.	-0.69246	1.875943	0.013306
ENSG0000(	C1orf43	-0.69246	3.703522	0.000198
ENSG0000(	BHLHE41	-0.69262	1.63877	0.022974
ENSG0000(	SNHG26	-0.69309	2.022595	0.009493
ENSG0000(	APOPT1	-0.69324	3.001113	0.000997
ENSG0000(	TRAK1	-0.69338	2.133847	0.007348
ENSG0000(	AC068888.	-0.69366	7.625939	2.37E-08
ENSG0000(	GSPT1	-0.69374	5.444851	3.59E-06
ENSG0000(	MRPS25	-0.69379	8.760632	1.74E-09
ENSG0000(	LARP4B	-0.6938	8.568419	2.70E-09
ENSG0000(	AMIGO1	-0.69426	2.296603	0.005051
ENSG0000(	AP000763.	-0.69433	1.49746	0.031808
ENSG0000(	COX7A1	-0.69442	1.448632	0.035593
ENSG0000(	HORMAD2	-0.69547	4.30047	5.01E-05
ENSG0000(	AP001107.	-0.69547	2.835843	0.001459
ENSG0000(	NECAB3	-0.6959	3.105622	0.000784
ENSG0000(	HDGFL2	-0.69599	10.14643	7.14E-11
ENSG0000(	PLCL2-AS1	-0.69605	2.957763	0.001102
ENSG0000(	KEAP1	-0.69625	3.5093	0.00031
ENSG0000(	DNLZ	-0.69644	3.175527	0.000668
ENSG0000(	UBE3C	-0.69646	4.162754	6.87E-05
ENSG0000(	AC004771.	-0.69649	8.946929	1.13E-09
ENSG0000(	AL683813.:	-0.69689	3.249771	0.000563
ENSG0000(	CEP85L	-0.69714	2.202056	0.00628
ENSG0000(	DYM	-0.69717	5.237623	5.79E-06
ENSG0000(	AC243919.	-0.69752	1.614238	0.024309
ENSG0000(	FBXL17	-0.69788	2.270189	0.005368
ENSG0000(	AC073052.	-0.69798	6.280793	5.24E-07
ENSG0000(	NDUFB4	-0.69828	3.239168	0.000577
ENSG0000(	LSMEM2	-0.69863	3.619055	0.00024
ENSG0000(	FBXO34	-0.69894	4.456703	3.49E-05
ENSG0000(	AC008038.	-0.69917	3.474831	0.000335
ENSG0000(	ABTB2	-0.6992	2.706556	0.001965
ENSG0000(	PNPLA7	-0.69992	3.054629	0.000882
ENSG0000(	SMARCD3	-0.70022	2.497187	0.003183

ENSG0000(	MAMSTR	-0.70025	1.356999	0.043954
ENSG0000(	NXN	-0.70083	1.939288	0.0115
ENSG0000(	DUSP7	-0.70087	4.903598	1.25E-05
ENSG0000(	CSDE1	-0.7009	2.449225	0.003554
ENSG0000(	RASSF3	-0.70092	1.657962	0.021981
ENSG0000(	VSIG10	-0.701	3.117898	0.000762
ENSG0000(	AL031733.:	-0.70106	1.672147	0.021274
ENSG0000(	ASH1L	-0.70107	7.486032	3.27E-08
ENSG0000(	GUCA1B	-0.70114	2.341675	0.004553
ENSG0000(	NPEPL1	-0.70115	5.49678	3.19E-06
ENSG0000(	IPO13	-0.70117	2.002381	0.009945
ENSG0000(	AC012184.	-0.70118	2.711737	0.001942
ENSG0000(	PDZD2	-0.70119	1.655014	0.02213
ENSG0000(	ZMYM4-AS	-0.70173	1.629934	0.023446
ENSG0000(	MT-TF	-0.70174	1.842559	0.014369
ENSG0000(	CAHM	-0.70191	2.923181	0.001193
ENSG0000(	PLEKHG3	-0.70254	1.617993	0.024099
ENSG0000(	NDUFS8	-0.70281	3.268517	0.000539
ENSG0000(	AC008878.	-0.703	3.121223	0.000756
ENSG0000(	TMEM184/	-0.70346	3.608235	0.000246
ENSG0000(	AC245297.	-0.70349	1.613684	0.02434
ENSG0000(	ESR2	-0.70387	1.564739	0.027243
ENSG0000(	TMEM233	-0.70391	1.310887	0.048878
ENSG0000(	DNAJB6	-0.704	3.607179	0.000247
ENSG0000(	JMJD6	-0.70402	4.215613	6.09E-05
ENSG0000(	LRPPRC	-0.70431	3.578704	0.000264
ENSG0000(	AC025165.	-0.70434	1.632033	0.023333
ENSG0000(	RTTN	-0.70457	4.28925	5.14E-05
ENSG0000(	PCBD2	-0.7046	2.923008	0.001194
ENSG0000(	BOP1	-0.70474	5.058689	8.74E-06
ENSG0000(	GOLGA4	-0.70492	3.501403	0.000315
ENSG0000(	KLHL25	-0.70503	3.747758	0.000179
ENSG0000(	C22orf39	-0.7053	4.556715	2.78E-05
ENSG0000(	RORA-AS1	-0.70536	2.712035	0.001941
ENSG0000(	PRKAR2A	-0.70543	2.727391	0.001873
ENSG0000(	NREP	-0.70551	1.968824	0.010744
ENSG0000(	EXTL3	-0.70578	4.932508	1.17E-05
ENSG0000(	KHDRBS3	-0.70593	4.7269	1.88E-05
ENSG0000(	GPN3	-0.70611	5.907399	1.24E-06
ENSG0000(	CLUH	-0.70621	2.266114	0.005419
ENSG0000(	HLCS	-0.70623	5.085764	8.21E-06
ENSG0000(	THNSL2	-0.70692	1.368952	0.042761
ENSG0000(	CENPV	-0.70697	3.392771	0.000405
ENSG0000(	HSDL2	-0.70735	2.171055	0.006744
ENSG0000(	TMOD1	-0.70762	2.297827	0.005037
ENSG0000(	2-Mar	-0.70803	7.498853	3.17E-08
ENSG0000(	OTUD7B	-0.71031	5.663536	2.17E-06

ENSG0000(	SH3RF3-AS	-0.71039	1.614995	0.024266
ENSG0000(	UBAP1L	-0.71143	3.673334	0.000212
ENSG0000(	AC009303.	-0.712	2.960822	0.001094
ENSG0000(	ZNF672	-0.71248	3.256679	0.000554
ENSG0000(	USP38	-0.71306	2.21692	0.006068
ENSG0000(	C22orf15	-0.71367	1.438848	0.036404
ENSG0000(	ZNF358	-0.71403	3.649041	0.000224
ENSG0000(	TADA2B	-0.71411	3.125633	0.000749
ENSG0000(	AL132639.:	-0.71431	2.819577	0.001515
ENSG0000(	VPS8	-0.71443	4.785149	1.64E-05
ENSG0000(	ALDH5A1	-0.71504	1.823952	0.014999
ENSG0000(	MAP2K6	-0.71524	1.604823	0.024841
ENSG0000(	EPB41	-0.71539	7.963842	1.09E-08
ENSG0000(	AC135586.	-0.71544	4.441132	3.62E-05
ENSG0000(	AC064807.	-0.71599	1.729284	0.018652
ENSG0000(	PLEC	-0.71614	2.682681	0.002076
ENSG0000(	USP54	-0.71625	1.517921	0.030344
ENSG0000(	PMEPA1	-0.71668	1.758754	0.017428
ENSG0000(	OPLAH	-0.71678	2.043598	0.009045
ENSG0000(	STIMATE	-0.71683	2.766761	0.001711
ENSG0000(	NDUFV2P1	-0.71687	1.672147	0.021274
ENSG0000(	STK33	-0.71716	1.522997	0.029992
ENSG0000(	CAND2	-0.71726	1.309456	0.049039
ENSG0000(	AL604028.:	-0.71761	4.011268	9.74E-05
ENSG0000(	APOM	-0.71782	3.331498	0.000466
ENSG0000(	BAG1	-0.71813	3.574274	0.000267
ENSG0000(	ZNF25	-0.71814	4.934749	1.16E-05
ENSG0000(	SKIL	-0.71855	2.515207	0.003053
ENSG0000(	HEXIM2	-0.71876	1.986809	0.010308
ENSG0000(	RTN2	-0.71982	1.346606	0.045019
ENSG0000(	MIR555	-0.72002	5.030584	9.32E-06
ENSG0000(	NT5C2	-0.72015	3.802897	0.000157
ENSG0000(	SHARPIN	-0.72083	6.826009	1.49E-07
ENSG0000(	NIPSNAP3E	-0.72129	1.836696	0.014565
ENSG0000(	TARID	-0.72136	2.539604	0.002887
ENSG0000(	VPS4A	-0.72162	5.494462	3.20E-06
ENSG0000(	PVRIG	-0.72186	1.362192	0.043432
ENSG0000(	KLHDC3	-0.72243	2.931524	0.001171
ENSG0000(	ERI3	-0.72248	6.132262	7.37E-07
ENSG0000(	RGS5	-0.72295	3.178677	0.000663
ENSG0000(	NUSAP1	-0.72351	2.536783	0.002905
ENSG0000(	PNPLA4	-0.72369	2.504789	0.003128
ENSG0000(	FAM129A	-0.724	1.665891	0.021583
ENSG0000(	UBXN1	-0.72425	11.79461	1.60E-12
ENSG0000(	POLDIP2	-0.72443	2.872031	0.001343
ENSG0000(	AC009065.	-0.72517	3.607509	0.000247
ENSG0000(	AC137630.	-0.72538	4.924439	1.19E-05

ENSG0000(	KCMF1	-0.72554	3.677049	0.00021
ENSG0000(	MIR3654	-0.7258	3.044484	0.000903
ENSG0000(	ARHGEF6	-0.72583	4.875762	1.33E-05
ENSG0000(	MIOS	-0.72656	4.73146	1.86E-05
ENSG0000(	AC025259.	-0.727	1.369842	0.042673
ENSG0000(	TUT4	-0.7272	6.123082	7.53E-07
ENSG0000(	UBE2Q2P2	-0.72725	1.896381	0.012695
ENSG0000(	SMYD2	-0.72731	4.020953	9.53E-05
ENSG0000(	RELT	-0.72734	1.614936	0.02427
ENSG0000(	LAPTM4B	-0.72776	1.832073	0.014721
ENSG0000(	WHAMM	-0.72825	7.842318	1.44E-08
ENSG0000(	AL110504.:	-0.72834	1.782604	0.016497
ENSG0000(	CPEB3	-0.7284	2.120879	0.00757
ENSG0000(	CAMTA2	-0.72878	7.618619	2.41E-08
ENSG0000(	NR4A1	-0.72914	1.341075	0.045596
ENSG0000(	PRKAR2A-/	-0.72937	1.685603	0.020625
ENSG0000(	TRAF3IP1	-0.72973	5.937995	1.15E-06
ENSG0000(	POLR2I	-0.72998	4.406224	3.92E-05
ENSG0000(	SLC25A23	-0.73001	2.12819	0.007444
ENSG0000(	BTBD6	-0.73042	2.911234	0.001227
ENSG0000(	FAF1	-0.73047	5.009353	9.79E-06
ENSG0000(	MPST	-0.73103	5.556009	2.78E-06
ENSG0000(	NDUFB11	-0.73178	3.819607	0.000151
ENSG0000(	THAP11	-0.73195	8.446053	3.58E-09
ENSG0000(	TBCE	-0.73208	5.38215	4.15E-06
ENSG0000(	HSPA1L	-0.73212	2.141038	0.007227
ENSG0000(	JUP	-0.7323	2.896813	0.001268
ENSG0000(	EIF4EBP1	-0.7324	2.937906	0.001154
ENSG0000(	LEAP2	-0.73269	2.654431	0.002216
ENSG0000(	CLIP1	-0.73272	2.465145	0.003427
ENSG0000(	CACNA2D1	-0.73326	1.596309	0.025333
ENSG0000(	MAP10	-0.73329	3.078367	0.000835
ENSG0000(	IER5	-0.7342	2.310947	0.004887
ENSG0000(	MFN2	-0.73424	2.570412	0.002689
ENSG0000(	LYRM7	-0.7348	3.173477	0.000671
ENSG0000(	CSPG4P10	-0.7354	3.212972	0.000612
ENSG0000(	VEGFA	-0.73557	1.553216	0.027976
ENSG0000(	NIPAL1	-0.73608	1.777439	0.016694
ENSG0000(	AC099677.	-0.73628	2.06678	0.008575
ENSG0000(	SLC22A5	-0.73661	1.912049	0.012245
ENSG0000(	SMPD5	-0.73662	2.178816	0.006625
ENSG0000(	PNPLA3	-0.73664	2.845848	0.001426
ENSG0000(	MAP2K2	-0.73665	6.697704	2.01E-07
ENSG0000(	PDZRN3	-0.73747	2.183906	0.006548
ENSG0000(	MLLT6	-0.73749	6.874541	1.33E-07
ENSG0000(	EIF3F	-0.73772	11.95691	1.10E-12
ENSG0000(	RF01892	-0.73817	1.782911	0.016485

ENSG0000(	AD001527.	-0.73833	4.376814	4.20E-05
ENSG0000(	CUTC	-0.73868	2.195514	0.006375
ENSG0000(	ECI1	-0.73887	3.490755	0.000323
ENSG0000(	TSPOAP1	-0.73917	2.406289	0.003924
ENSG0000(	SMAD3	-0.7394	5.302365	4.98E-06
ENSG0000(	SPTBN4	-0.73973	2.923611	0.001192
ENSG0000(	ZNF774	-0.74002	1.356299	0.044025
ENSG0000(	WDTC1	-0.74126	4.435662	3.67E-05
ENSG0000(	FAM84A	-0.74126	1.635939	0.023124
ENSG0000(	POLR2J2	-0.74126	2.016969	0.009617
ENSG0000(	AC008878.	-0.74152	4.04168	9.08E-05
ENSG0000(	EIF5	-0.74214	8.513949	3.06E-09
ENSG0000(	EEF2K	-0.74329	5.37221	4.24E-06
ENSG0000(	FOXO3	-0.7433	1.666246	0.021565
ENSG0000(	AC105345.	-0.74345	2.586102	0.002594
ENSG0000(	FLII	-0.74403	4.066713	8.58E-05
ENSG0000(	AKAP7	-0.74445	4.279031	5.26E-05
ENSG0000(	RABEP1	-0.7449	4.961822	1.09E-05
ENSG0000(	PTPN14	-0.74575	4.295768	5.06E-05
ENSG0000(	AL590094.:	-0.74591	3.902481	0.000125
ENSG0000(	ZNF423	-0.74663	7.321896	4.77E-08
ENSG0000(	GSTM2	-0.74707	6.210593	6.16E-07
ENSG0000(	IFNLR1	-0.74722	1.421085	0.037924
ENSG0000(	NME7	-0.74729	3.793762	0.000161
ENSG0000(	RCSD1	-0.7476	1.947535	0.011284
ENSG0000(	FXN	-0.74799	3.694652	0.000202
ENSG0000(	RABL6	-0.74839	4.430947	3.71E-05
ENSG0000(	MED14	-0.74853	4.862166	1.37E-05
ENSG0000(	LINC02019	-0.74857	1.465905	0.034205
ENSG0000(	ZNRF2P2	-0.74869	3.170919	0.000675
ENSG0000(	PLAGL2	-0.74872	3.756421	0.000175
ENSG0000(	AC004771.	-0.74949	6.720591	1.90E-07
ENSG0000(	SELENOW	-0.75046	2.580411	0.002628
ENSG0000(	ANAPC16	-0.75054	5.655587	2.21E-06
ENSG0000(	ZNF628	-0.75111	7.378381	4.18E-08
ENSG0000(	FAM189A2	-0.75123	1.619599	0.02401
ENSG0000(	COQ8A	-0.75147	1.340512	0.045655
ENSG0000(	SNHG6	-0.75154	6.098229	7.98E-07
ENSG0000(	RWDD4P2	-0.75164	4.317633	4.81E-05
ENSG0000(	AC127496.	-0.75178	2.764414	0.00172
ENSG0000(	AL022238.4	-0.752	5.907224	1.24E-06
ENSG0000(	SLC25A30	-0.75216	1.768343	0.017047
ENSG0000(	ASB16-AS1	-0.75217	1.911712	0.012254
ENSG0000(	NPTN-IT1	-0.7526	2.101511	0.007916
ENSG0000(	EEF2	-0.75271	4.74738	1.79E-05
ENSG0000(	TMEM150/	-0.75321	4.342899	4.54E-05
ENSG0000(	BIN1	-0.7533	1.559657	0.027564

ENSG0000(	EMD	-0.7535	8.752106	1.77E-09
ENSG0000(	FKBP3	-0.75402	2.048397	0.008945
ENSG0000(	HOXA10-A!	-0.75409	3.875497	0.000133
ENSG0000(	AXIN2	-0.75468	2.336528	0.004608
ENSG0000(	AC006994.	-0.75469	1.41365	0.038579
ENSG0000(	AC027682.	-0.75503	1.896418	0.012694
ENSG0000(	CNNM3	-0.7553	2.161009	0.006902
ENSG0000(	PLCL1	-0.75581	1.803025	0.015739
ENSG0000(	KIF13A	-0.75652	4.19673	6.36E-05
ENSG0000(	FXR2	-0.75789	2.95231	0.001116
ENSG0000(	AC007342.	-0.75834	2.902745	0.001251
ENSG0000(	GRB10	-0.75853	5.533914	2.92E-06
ENSG0000(	GTF2IRD2B	-0.75936	10.83492	1.46E-11
ENSG0000(	SLC25A37	-0.75936	7.407267	3.92E-08
ENSG0000(	MYBBP1A	-0.75981	3.849276	0.000141
ENSG0000(	GOLGA2P1	-0.75995	2.762476	0.001728
ENSG0000(	PLIN2	-0.76083	3.137723	0.000728
ENSG0000(	NEDD1	-0.76161	2.57748	0.002646
ENSG0000(	RPP25L	-0.76176	2.953809	0.001112
ENSG0000(	DOP1B	-0.76209	4.253203	5.58E-05
ENSG0000(	AC074143.	-0.76227	6.553754	2.79E-07
ENSG0000(	MLEC	-0.76355	5.452274	3.53E-06
ENSG0000(	AC093305.	-0.76613	1.728242	0.018696
ENSG0000(	KLHL23	-0.76631	4.550672	2.81E-05
ENSG0000(	HECW1	-0.76646	1.340437	0.045663
ENSG0000(	PPP1R9A	-0.76678	3.372526	0.000424
ENSG0000(	COPS3	-0.76688	4.159016	6.93E-05
ENSG0000(	AC026464.	-0.76739	4.95705	1.10E-05
ENSG0000(	RPUSD4	-0.76799	3.126638	0.000747
ENSG0000(	RAB28P5	-0.76922	2.47197	0.003373
ENSG0000(	SVIP	-0.76932	4.617021	2.42E-05
ENSG0000(	SUCLG2	-0.76953	3.930654	0.000117
ENSG0000(	RHOD	-0.76991	1.831968	0.014724
ENSG0000(	CUL5	-0.77003	3.302964	0.000498
ENSG0000(	ZCCHC2	-0.77015	4.844276	1.43E-05
ENSG0000(	EYA4	-0.77036	2.049684	0.008919
ENSG0000(	MYPOP	-0.77106	5.104564	7.86E-06
ENSG0000(	MBNL1	-0.77166	4.314949	4.84E-05
ENSG0000(	RANP4	-0.77211	2.291336	0.005113
ENSG0000(	SLC25A12	-0.77212	1.878829	0.013218
ENSG0000(	SLC25A30-/	-0.77283	1.427238	0.037391
ENSG0000(	KLHL8	-0.77291	5.322021	4.76E-06
ENSG0000(	GKAP1	-0.77293	3.171489	0.000674
ENSG0000(	MRPS9	-0.77321	4.64453	2.27E-05
ENSG0000(	UBE2B	-0.77329	5.130141	7.41E-06
ENSG0000(	IPO5	-0.77332	5.928136	1.18E-06
ENSG0000(	EEF1B2	-0.77349	7.768067	1.71E-08

ENSG0000(	EGLN1	-0.77361	1.896687	0.012686
ENSG0000(	NCBP2L	-0.77396	2.832273	0.001471
ENSG0000(	AC108866.	-0.77419	2.53863	0.002893
ENSG0000(	AC092651.	-0.77425	4.615344	2.42E-05
ENSG0000(	TLE2	-0.77499	3.011973	0.000973
ENSG0000(	AC006449.	-0.7753	7.439151	3.64E-08
ENSG0000(	NR3C2	-0.77586	4.739188	1.82E-05
ENSG0000(	AC073349.	-0.77613	1.810435	0.015473
ENSG0000(	AC005329.	-0.77632	2.662097	0.002177
ENSG0000(	TAF3	-0.7764	9.621172	2.39E-10
ENSG0000(	AC017083.	-0.77802	7.043506	9.05E-08
ENSG0000(	VDAC1	-0.77876	2.121079	0.007567
ENSG0000(	AC003688.	-0.77935	3.495802	0.000319
ENSG0000(	TESK2	-0.78086	3.46559	0.000342
ENSG0000(	KANSL2	-0.78105	3.327429	0.000471
ENSG0000(	MICAL3	-0.78125	5.513326	3.07E-06
ENSG0000(	UBAC1	-0.78287	3.263317	0.000545
ENSG0000(	MARK4	-0.78294	4.975314	1.06E-05
ENSG0000(	FAM220A	-0.78358	2.586044	0.002594
ENSG0000(	FHIT	-0.78383	4.066447	8.58E-05
ENSG0000(	QDPR	-0.78395	6.699036	2.00E-07
ENSG0000(	ASB16	-0.78405	1.746794	0.017915
ENSG0000(	ARAF	-0.78498	5.623127	2.38E-06
ENSG0000(	FKBP4	-0.78539	4.677583	2.10E-05
ENSG0000(	AC010336.	-0.7856	8.367557	4.29E-09
ENSG0000(	PYGO1	-0.78667	2.808344	0.001555
ENSG0000(	DIP2C	-0.78674	5.657065	2.20E-06
ENSG0000(	UBALD2	-0.78739	4.83476	1.46E-05
ENSG0000(	AL355987.:	-0.78754	3.135881	0.000731
ENSG0000(	AL133406.:	-0.78805	3.263829	0.000545
ENSG0000(	CASTOR2	-0.78859	1.391831	0.040567
ENSG0000(	RIC3	-0.78891	3.408647	0.00039
ENSG0000(	LSMEM1	-0.7891	1.695003	0.020184
ENSG0000(	ST3GAL3	-0.78927	2.961669	0.001092
ENSG0000(	PRPS1P2	-0.78968	2.80258	0.001576
ENSG0000(	AC083843.	-0.79006	1.861485	0.013757
ENSG0000(	FRAS1	-0.79012	2.020915	0.00953
ENSG0000(	ST3GAL2	-0.79019	3.931614	0.000117
ENSG0000(	HIGD1B	-0.79092	1.74462	0.018004
ENSG0000(	GHR	-0.79126	4.495016	3.20E-05
ENSG0000(	AC016026.	-0.79197	6.208082	6.19E-07
ENSG0000(	MAOB	-0.7922	3.275532	0.00053
ENSG0000(	AL591848.4	-0.79283	1.895201	0.012729
ENSG0000(	IRS1	-0.7932	2.640573	0.002288
ENSG0000(	VAPB	-0.79361	6.117379	7.63E-07
ENSG0000(	AC138150.	-0.79387	2.172294	0.006725
ENSG0000(	RAF1	-0.794	7.098082	7.98E-08

ENSG0000(	CCDC69	-0.79409	1.979909	0.010473
ENSG0000(	MAF1	-0.79456	6.06171	8.68E-07
ENSG0000(	KLF9	-0.79509	3.06244	0.000866
ENSG0000(	EIF4B	-0.79514	8.873012	1.34E-09
ENSG0000(	PRAG1	-0.7956	4.873869	1.34E-05
ENSG0000(	MT-TD	-0.79585	2.524578	0.002988
ENSG0000(	NIPSNAP2	-0.79633	2.109031	0.00778
ENSG0000(	ST3GAL1	-0.79689	2.544135	0.002857
ENSG0000(	KCNS3	-0.79758	2.126799	0.007468
ENSG0000(	UTAT33	-0.79772	5.870427	1.35E-06
ENSG0000(	AC007375.	-0.79775	1.624296	0.023752
ENSG0000(	TBC1D1	-0.79776	2.186118	0.006515
ENSG0000(	DPF3	-0.79792	1.723541	0.0189
ENSG0000(	YBX1	-0.79812	6.153087	7.03E-07
ENSG0000(	AC006449.	-0.7985	5.214492	6.10E-06
ENSG0000(	UACA	-0.79857	3.934524	0.000116
ENSG0000(	DENND4B	-0.79903	5.809152	1.55E-06
ENSG0000(	NFIX	-0.79927	4.769538	1.70E-05
ENSG0000(	HOMER1	-0.80079	1.558208	0.027656
ENSG0000(	MTATP6P1	-0.80112	1.529113	0.029572
ENSG0000(	NDUFS7	-0.80134	3.130358	0.000741
ENSG0000(	AC073167.	-0.8018	1.397375	0.040052
ENSG0000(	POMZP3	-0.80246	3.159633	0.000692
ENSG0000(	MASP2	-0.8026	5.293249	5.09E-06
ENSG0000(	LGALSL	-0.80507	2.903267	0.001249
ENSG0000(	USO1	-0.80565	2.910329	0.001229
ENSG0000(	EIF4BP6	-0.80587	3.705775	0.000197
ENSG0000(	NEDD4	-0.80642	2.085159	0.008219
ENSG0000(	GPLD1	-0.80677	4.801226	1.58E-05
ENSG0000(	CTDNEP1	-0.8072	4.529607	2.95E-05
ENSG0000(	AC069234.	-0.80787	4.099654	7.95E-05
ENSG0000(	NCOA3	-0.8079	4.991883	1.02E-05
ENSG0000(	UBE2E3	-0.80795	6.372406	4.24E-07
ENSG0000(	RFESD	-0.80992	2.639206	0.002295
ENSG0000(	UBR3	-0.81006	2.338274	0.004589
ENSG0000(	AP000695.	-0.81044	1.34624	0.045057
ENSG0000(	ZFYVE9	-0.81072	5.248315	5.65E-06
ENSG0000(	WWP1	-0.81122	2.731583	0.001855
ENSG0000(	NKX6-2	-0.81179	4.986084	1.03E-05
ENSG0000(	INTS1	-0.81189	5.98521	1.03E-06
ENSG0000(	AP002990.	-0.81203	5.62922	2.35E-06
ENSG0000(	AC005329.	-0.81243	3.555915	0.000278
ENSG0000(	RASL12	-0.81259	2.887862	0.001295
ENSG0000(	CHCHD3	-0.81283	2.674579	0.002116
ENSG0000(	HPN	-0.81318	1.599267	0.025161
ENSG0000(	AL138963.:	-0.81373	3.598257	0.000252
ENSG0000(	STARD4-AS	-0.81376	2.880158	0.001318

ENSG0000(	TBKBP1	-0.81474	3.98532	0.000103
ENSG0000(	RSC1A1	-0.81505	2.047891	0.008956
ENSG0000(	ZBTB16	-0.81636	1.658432	0.021957
ENSG0000(	PWWP2B	-0.81647	2.759675	0.001739
ENSG0000(	AL451062.:	-0.81666	5.40912	3.90E-06
ENSG0000(	USP25	-0.81694	3.676361	0.000211
ENSG0000(	NAA30	-0.8179	3.978305	0.000105
ENSG0000(	EEF1G	-0.81862	5.585916	2.59E-06
ENSG0000(	CAMSAP1	-0.81952	3.488269	0.000325
ENSG0000(	AC097461.	-0.81956	1.883483	0.013077
ENSG0000(	AL592424.:	-0.81957	1.355794	0.044076
ENSG0000(	PCNT	-0.82042	2.060736	0.008695
ENSG0000(	AL358472.!	-0.82069	5.033679	9.25E-06
ENSG0000(	AC245033.	-0.82218	4.019929	9.55E-05
ENSG0000(	PPP3R1	-0.82325	8.38638	4.11E-09
ENSG0000(	ATP23	-0.82329	2.681463	0.002082
ENSG0000(	C19orf47	-0.82377	2.421974	0.003785
ENSG0000(	SERINC2	-0.82489	2.287856	0.005154
ENSG0000(	GAS2	-0.82542	3.402297	0.000396
ENSG0000(	GPD1	-0.82569	1.746	0.017947
ENSG0000(	PACS2	-0.82573	10.24674	5.67E-11
ENSG0000(	KLF13	-0.82616	4.570748	2.69E-05
ENSG0000(	ZNF784	-0.8266	3.269941	0.000537
ENSG0000(	RNLS	-0.82663	8.167883	6.79E-09
ENSG0000(	RBM38	-0.82796	1.701951	0.019863
ENSG0000(	CDKN2AIPI	-0.82869	4.704081	1.98E-05
ENSG0000(	IFRD2	-0.82876	5.953535	1.11E-06
ENSG0000(	KPNA4	-0.82902	3.256037	0.000555
ENSG0000(	DNAJC12	-0.82911	1.311397	0.048821
ENSG0000(	UBE2D1	-0.82915	1.986809	0.010308
ENSG0000(	RALGAPA2	-0.82927	3.676067	0.000211
ENSG0000(	PIK3R1	-0.82999	1.670372	0.021361
ENSG0000(	AP000785.	-0.83147	1.316403	0.048261
ENSG0000(	AC005841.	-0.83171	5.010632	9.76E-06
ENSG0000(	CHCHD10	-0.83208	1.716562	0.019206
ENSG0000(	RNF34	-0.83226	6.413287	3.86E-07
ENSG0000(	AC137056.	-0.8326	3.12915	0.000743
ENSG0000(	AP005717.	-0.83269	2.137352	0.007289
ENSG0000(	EPM2A	-0.83288	2.305024	0.004954
ENSG0000(	STK25	-0.83492	4.863365	1.37E-05
ENSG0000(	NMNAT1	-0.83497	3.172288	0.000673
ENSG0000(	DDX59	-0.83535	7.589168	2.58E-08
ENSG0000(	SALRNA1	-0.83615	2.824323	0.001499
ENSG0000(	SETBP1	-0.83624	5.05134	8.89E-06
ENSG0000(	AL354740.:	-0.83673	7.667765	2.15E-08
ENSG0000(	FITM1	-0.83693	1.368737	0.042782
ENSG0000(	PKM	-0.83714	2.271221	0.005355

ENSG0000(	GRIP2	-0.83791	2.125088	0.007497
ENSG0000(	EML1	-0.83817	7.843929	1.43E-08
ENSG0000(	CELSR2	-0.83823	1.881064	0.01315
ENSG0000(	YBX1P1	-0.8383	4.670876	2.13E-05
ENSG0000(	RGMA	-0.83843	3.772049	0.000169
ENSG0000(	BTNL9	-0.83902	3.030902	0.000931
ENSG0000(	TMPRSS5	-0.8391	4.267319	5.40E-05
ENSG0000(	AL603962.:	-0.84003	3.748583	0.000178
ENSG0000(	SIVA1	-0.84011	12.45107	3.54E-13
ENSG0000(	PDLIM3	-0.84061	1.504611	0.031289
ENSG0000(	FAM53C	-0.84062	6.019259	9.57E-07
ENSG0000(	AGL	-0.84093	1.348394	0.044834
ENSG0000(	ABCB4	-0.84099	2.617749	0.002411
ENSG0000(	ZNF710-AS	-0.8412	2.585468	0.002597
ENSG0000(	SPDYA	-0.84131	2.897505	0.001266
ENSG0000(	AC003688.	-0.84186	1.344039	0.045286
ENSG0000(	AC020909.	-0.84201	1.537077	0.029035
ENSG0000(	HECTD2	-0.84352	3.274004	0.000532
ENSG0000(	ANKRD40	-0.84369	5.153754	7.02E-06
ENSG0000(	RNA5SP21(	-0.84408	3.869581	0.000135
ENSG0000(	ATP5F1D	-0.8448	3.293449	0.000509
ENSG0000(	ZNF106	-0.84503	1.970584	0.010701
ENSG0000(	KLHL33	-0.8453	1.766555	0.017118
ENSG0000(	SLC25A25-/	-0.84634	3.545675	0.000285
ENSG0000(	PLEKHH3	-0.84694	6.445723	3.58E-07
ENSG0000(	AC104116.	-0.847	6.251168	5.61E-07
ENSG0000(	CUEDC1	-0.8474	7.885215	1.30E-08
ENSG0000(	SAMM50	-0.84752	4.391693	4.06E-05
ENSG0000(	CARM1	-0.84762	5.321468	4.77E-06
ENSG0000(	HTD2	-0.84804	3.55279	0.00028
ENSG0000(	MRPS15	-0.84868	5.699622	2.00E-06
ENSG0000(	CDNF	-0.84988	3.756492	0.000175
ENSG0000(	HSF4	-0.85022	6.53226	2.94E-07
ENSG0000(	RPP14	-0.85061	3.859395	0.000138
ENSG0000(	SLC9A5	-0.85061	2.539993	0.002884
ENSG0000(	ACSS2	-0.85071	3.836862	0.000146
ENSG0000(	AC131238.	-0.85098	3.026191	0.000941
ENSG0000(	HINT3	-0.8517	3.117282	0.000763
ENSG0000(	NUDT3	-0.85236	7.215115	6.09E-08
ENSG0000(	DHPS	-0.8527	18.75951	1.74E-19
ENSG0000(	PIP4K2B	-0.85438	6.440008	3.63E-07
ENSG0000(	IKZF4	-0.8546	5.283681	5.20E-06
ENSG0000(	ENC1	-0.8546	2.187598	0.006492
ENSG0000(	PDE4DIP	-0.8547	1.834657	0.014633
ENSG0000(	PLCB1	-0.85486	4.962583	1.09E-05
ENSG0000(	MAPT	-0.85508	1.605255	0.024817
ENSG0000(	AC015914.	-0.85531	2.964732	0.001085

ENSG0000(	PPP6C	-0.85659	12.82961	1.48E-13
ENSG0000(	ANKRD46	-0.85718	4.282944	5.21E-05
ENSG0000(	EIF3CL	-0.85765	3.565333	0.000272
ENSG0000(	JAKMIP3	-0.85808	3.388867	0.000408
ENSG0000(	SAR1B	-0.85827	3.125682	0.000749
ENSG0000(	PHTF2	-0.85847	2.035197	0.009222
ENSG0000(	TPT1	-0.85888	4.230166	5.89E-05
ENSG0000(	ATP1A4	-0.85888	1.483078	0.032879
ENSG0000(	CATSPER3	-0.85903	4.173096	6.71E-05
ENSG0000(	AC010889.	-0.85947	5.363587	4.33E-06
ENSG0000(	AC090772.	-0.85972	7.108977	7.78E-08
ENSG0000(	AC004076.	-0.86123	1.578977	0.026365
ENSG0000(	ZBTB18	-0.86197	3.001113	0.000997
ENSG0000(	PDLIM2	-0.86267	5.094024	8.05E-06
ENSG0000(	SEMA6C	-0.86291	1.967751	0.010771
ENSG0000(	HECTD1	-0.86445	4.397636	4.00E-05
ENSG0000(	ADCY2	-0.86499	2.343262	0.004537
ENSG0000(	ST13	-0.86619	16.60711	2.47E-17
ENSG0000(	COBL	-0.86626	1.730373	0.018605
ENSG0000(	CECR2	-0.86678	1.850644	0.014104
ENSG0000(	YBX1P10	-0.86681	5.104407	7.86E-06
ENSG0000(	ZNF865	-0.86788	4.851592	1.41E-05
ENSG0000(	CIPC	-0.86791	2.430826	0.003708
ENSG0000(	PPP2R3A	-0.86805	1.976912	0.010546
ENSG0000(	NDFIP2	-0.86808	6.718915	1.91E-07
ENSG0000(	SLC16A12	-0.86843	1.337686	0.045953
ENSG0000(	NRG4	-0.8685	3.03753	0.000917
ENSG0000(	PPP1CB	-0.86865	4.158861	6.94E-05
ENSG0000(	AC124947.	-0.86869	1.892283	0.012815
ENSG0000(	BZW2	-0.86963	2.773256	0.001686
ENSG0000(	SLC14A1	-0.86976	1.709445	0.019523
ENSG0000(	OSBPL1A	-0.87004	5.772584	1.69E-06
ENSG0000(	AC087164.	-0.87294	3.049809	0.000892
ENSG0000(	SVIL-AS1	-0.87372	2.65503	0.002213
ENSG0000(	ZNF192P1	-0.87373	2.911968	0.001225
ENSG0000(	DMTN	-0.87388	3.109628	0.000777
ENSG0000(	TRAPPC9	-0.87389	5.747751	1.79E-06
ENSG0000(	PEX26	-0.87411	3.5758	0.000266
ENSG0000(	PFKM	-0.87424	1.724421	0.018862
ENSG0000(	LDHA	-0.87563	2.780942	0.001656
ENSG0000(	FXYD1	-0.87564	3.582312	0.000262
ENSG0000(	RNF126	-0.87565	8.910776	1.23E-09
ENSG0000(	MACROD1	-0.87583	2.372322	0.004243
ENSG0000(	AC239802.	-0.87605	1.599508	0.025147
ENSG0000(	LRRC2	-0.87777	1.44614	0.035798
ENSG0000(	RNF150	-0.8778	2.600001	0.002512
ENSG0000(	AC072039.	-0.8779	1.890487	0.012868

ENSG0000(	PLPP7	-0.87792	1.925128	0.011882
ENSG0000(	PER2	-0.87865	3.278761	0.000526
ENSG0000(	TMEM201	-0.87881	3.392999	0.000405
ENSG0000(	PSTPIP2	-0.87935	2.393887	0.004038
ENSG0000(	TFEB	-0.87962	4.195424	6.38E-05
ENSG0000(	PHKG1	-0.87972	2.702202	0.001985
ENSG0000(	MAPT-AS1	-0.88021	1.300126	0.050104
ENSG0000(	ADCY9	-0.88078	3.757435	0.000175
ENSG0000(	MIR6747	-0.88145	4.017076	9.61E-05
ENSG0000(	TSPAN5	-0.88168	1.732508	0.018514
ENSG0000(	ADSL	-0.88238	3.479643	0.000331
ENSG0000(	AC020907.	-0.8826	3.576734	0.000265
ENSG0000(	ZBTB44	-0.88318	6.699036	2.00E-07
ENSG0000(	MIR5006	-0.88339	2.463406	0.00344
ENSG0000(	CDCA7	-0.88363	1.737335	0.018309
ENSG0000(	PXMP2	-0.88453	5.614509	2.43E-06
ENSG0000(	PRDM10	-0.88481	7.895881	1.27E-08
ENSG0000(	TTC23	-0.88536	4.935535	1.16E-05
ENSG0000(	ALKBH5	-0.88548	4.272862	5.34E-05
ENSG0000(	CAP2	-0.88578	1.812603	0.015396
ENSG0000(	PHKB	-0.88604	3.914211	0.000122
ENSG0000(	DCAF6	-0.88653	3.002486	0.000994
ENSG0000(	SCUBE2	-0.88661	1.594845	0.025419
ENSG0000(	AC073333.	-0.88696	2.833241	0.001468
ENSG0000(	SOS2	-0.88734	4.536347	2.91E-05
ENSG0000(	AC027575.	-0.8885	5.712335	1.94E-06
ENSG0000(	AC005921.	-0.88896	7.892804	1.28E-08
ENSG0000(	AC105052.	-0.88914	2.101007	0.007925
ENSG0000(	WDR47	-0.88937	8.023938	9.46E-09
ENSG0000(	PPP1R2	-0.89011	5.442838	3.61E-06
ENSG0000(	GID4	-0.89044	6.886033	1.30E-07
ENSG0000(	TRIM52-AS	-0.89048	3.158569	0.000694
ENSG0000(	DRD4	-0.89218	2.072642	0.00846
ENSG0000(	RXRA	-0.89238	5.721977	1.90E-06
ENSG0000(	AC012485.	-0.89252	2.613576	0.002435
ENSG0000(	MPPED2	-0.89398	1.706303	0.019665
ENSG0000(	UBFD1	-0.89558	5.55919	2.76E-06
ENSG0000(	COX6A2	-0.89565	1.424854	0.037596
ENSG0000(	DNASE1L1	-0.89568	4.894874	1.27E-05
ENSG0000(	USP2	-0.89688	1.665686	0.021593
ENSG0000(	RMND5A	-0.89866	5.344801	4.52E-06
ENSG0000(	AC008735.	-0.89873	1.709008	0.019543
ENSG0000(	TNKS2	-0.89873	5.514044	3.06E-06
ENSG0000(	AVIL	-0.9021	5.070084	8.51E-06
ENSG0000(	TSC22D1	-0.90211	3.552635	0.00028
ENSG0000(	AC008870.	-0.90221	3.485607	0.000327
ENSG0000(	PIK3CB	-0.90331	4.060935	8.69E-05

ENSG0000(	ACSM5	-0.90417	4.065278	8.60E-05
ENSG0000(	NOXA1	-0.90421	6.248887	5.64E-07
ENSG0000(	AC026471.	-0.90452	1.440197	0.036291
ENSG0000(	AC073389.	-0.90495	1.855711	0.013941
ENSG0000(	GPATCH8	-0.90518	7.435722	3.67E-08
ENSG0000(	DCLK3	-0.90621	1.435463	0.036689
ENSG0000(	TAL2	-0.90699	1.385518	0.041161
ENSG0000(	FAM50B	-0.9072	8.707235	1.96E-09
ENSG0000(	CDC25C	-0.90723	3.750883	0.000177
ENSG0000(	PRKAG3	-0.90798	1.308223	0.049179
ENSG0000(	AC092378.	-0.90835	1.863259	0.013701
ENSG0000(	USP24	-0.91017	4.291911	5.11E-05
ENSG0000(	SRL	-0.91021	1.512909	0.030697
ENSG0000(	INPP5A	-0.91034	4.793416	1.61E-05
ENSG0000(	MID1IP1-A	-0.91056	3.600031	0.000251
ENSG0000(	PCAT7	-0.91065	1.396105	0.040169
ENSG0000(	SCN4A	-0.91077	1.611443	0.024466
ENSG0000(	PKD1P5	-0.91141	2.140484	0.007236
ENSG0000(	LBX1	-0.91282	1.392056	0.040546
ENSG0000(	PKD1L1	-0.91407	3.77073	0.00017
ENSG0000(	AL050343.:	-0.91438	7.077646	8.36E-08
ENSG0000(	NFIC	-0.91444	7.7793	1.66E-08
ENSG0000(	CLIC5	-0.91494	1.756105	0.017535
ENSG0000(	ZNF671	-0.91625	4.473917	3.36E-05
ENSG0000(	MAP3K20	-0.91836	2.698059	0.002004
ENSG0000(	TMEM143	-0.91863	2.721674	0.001898
ENSG0000(	TOB1-AS1	-0.92051	2.145286	0.007157
ENSG0000(	AC008687.	-0.92094	1.323946	0.04743
ENSG0000(	ARHGEF4	-0.92102	4.918346	1.21E-05
ENSG0000(	RBM24	-0.92222	1.580958	0.026245
ENSG0000(	NEU3	-0.92302	4.386364	4.11E-05
ENSG0000(	DICER1-AS:	-0.92399	6.848749	1.42E-07
ENSG0000(	DEPTOR	-0.92453	3.353036	0.000444
ENSG0000(	LANCL1-AS	-0.92464	1.677239	0.021026
ENSG0000(	PHKA1	-0.92481	1.607916	0.024665
ENSG0000(	SVIL	-0.92693	1.96087	0.010943
ENSG0000(	PDCL3P4	-0.92708	3.147087	0.000713
ENSG0000(	ZYG11B	-0.92733	2.923383	0.001193
ENSG0000(	CD300LG	-0.92735	2.202788	0.006269
ENSG0000(	TSPAN8	-0.92833	1.445136	0.035881
ENSG0000(	ACTN2	-0.92856	1.404507	0.0394
ENSG0000(	RN7SL731F	-0.92863	2.857555	0.001388
ENSG0000(	NEURL1	-0.92974	1.58119	0.026231
ENSG0000(	MSRB3	-0.93013	6.325274	4.73E-07
ENSG0000(	AC139795.	-0.93135	2.979222	0.001049
ENSG0000(	RNFT1-DT	-0.93214	2.335898	0.004614
ENSG0000(	AL691432.:	-0.93227	4.541774	2.87E-05

ENSG0000(	AL355877.:	-0.93238	1.558631	0.027629
ENSG0000(	CTNNA3	-0.9342	1.51504	0.030546
ENSG0000(	KLF15	-0.93458	1.696199	0.020128
ENSG0000(	SHMT1	-0.93495	7.500434	3.16E-08
ENSG0000(	GRTP1	-0.93566	10.20537	6.23E-11
ENSG0000(	DAPK2	-0.93627	3.032339	0.000928
ENSG0000(	ATG4D	-0.93633	4.48133	3.30E-05
ENSG0000(	RAB10	-0.93645	4.955818	1.11E-05
ENSG0000(	ASB2	-0.93706	1.315823	0.048326
ENSG0000(	AL669841.:	-0.93718	1.637447	0.023044
ENSG0000(	AC037459.	-0.9383	6.610109	2.45E-07
ENSG0000(	DTWD2	-0.93835	3.506137	0.000312
ENSG0000(	SLC2A4	-0.93949	1.7097	0.019512
ENSG0000(	AC009078.	-0.93965	3.327269	0.000471
ENSG0000(	PHYH	-0.94033	3.454254	0.000351
ENSG0000(	MIR133A1I	-0.94066	1.441177	0.03621
ENSG0000(	C10orf71	-0.94108	1.420311	0.037992
ENSG0000(	FHOD1	-0.94135	2.942716	0.001141
ENSG0000(	LDB3	-0.94309	1.578662	0.026384
ENSG0000(	KBTBD8	-0.9445	1.395698	0.040207
ENSG0000(	AC020917.	-0.94514	5.45358	3.52E-06
ENSG0000(	FYCO1	-0.9454	2.612307	0.002442
ENSG0000(	NRDC	-0.9457	5.315099	4.84E-06
ENSG0000(	PPP1R3A	-0.9459	1.430241	0.037133
ENSG0000(	KIF1B	-0.94595	3.094487	0.000804
ENSG0000(	TARSL2	-0.94748	4.362633	4.34E-05
ENSG0000(	C14orf180	-0.94796	1.983099	0.010397
ENSG0000(	GPD1L	-0.94861	2.446491	0.003577
ENSG0000(	CCDC192	-0.94909	3.936011	0.000116
ENSG0000(	PGP	-0.94951	3.303255	0.000497
ENSG0000(	RNF144B	-0.9498	2.598661	0.00252
ENSG0000(	GTF2IRD1	-0.95022	3.989602	0.000102
ENSG0000(	EFNB2	-0.95139	5.353833	4.43E-06
ENSG0000(	CITED4	-0.95378	3.066033	0.000859
ENSG0000(	CAMK2G	-0.95428	5.170375	6.76E-06
ENSG0000(	AC022400.	-0.95453	4.880546	1.32E-05
ENSG0000(	TPM2	-0.95471	1.749215	0.017815
ENSG0000(	FOXO4	-0.95472	4.395359	4.02E-05
ENSG0000(	SYNPO2L	-0.95515	1.655445	0.022108
ENSG0000(	AMPD1	-0.95581	1.594433	0.025443
ENSG0000(	PDE6C	-0.9559	1.740565	0.018173
ENSG0000(	SGK1	-0.95656	2.278669	0.005264
ENSG0000(	INSR	-0.95704	7.286542	5.17E-08
ENSG0000(	AC004982.	-0.95903	4.927353	1.18E-05
ENSG0000(	B3GALNT2	-0.95949	3.532479	0.000293
ENSG0000(	AL132642.:	-0.96058	1.391071	0.040638
ENSG0000(	NEO1	-0.96093	6.085661	8.21E-07

ENSG0000(	LMCD1	-0.96209	3.521235	0.000301
ENSG0000(	KPNA3	-0.96235	3.645517	0.000226
ENSG0000(	SIMC1	-0.96245	7.242767	5.72E-08
ENSG0000(	PATJ	-0.96398	2.901685	0.001254
ENSG0000(	PRKCQ	-0.96612	1.91129	0.012266
ENSG0000(	GPD2	-0.96627	5.621311	2.39E-06
ENSG0000(	DNAJC16	-0.96643	4.199804	6.31E-05
ENSG0000(	AP005137.	-0.96662	1.369103	0.042746
ENSG0000(	ARNT2	-0.96762	2.317468	0.004814
ENSG0000(	SEMA6D	-0.96828	4.532269	2.94E-05
ENSG0000(	AL355355.:	-0.96918	3.154199	0.000701
ENSG0000(	SUGCT	-0.96924	4.72852	1.87E-05
ENSG0000(	DBNL	-0.96977	2.862383	0.001373
ENSG0000(	OPTN	-0.96981	3.644185	0.000227
ENSG0000(	RNF123	-0.97016	3.578704	0.000264
ENSG0000(	GOT2	-0.97031	2.374626	0.004221
ENSG0000(	SAPCD1-AS	-0.97129	5.314422	4.85E-06
ENSG0000(	ECPAS	-0.97136	5.743025	1.81E-06
ENSG0000(	CACNA2D3	-0.97174	2.145455	0.007154
ENSG0000(	NPHP1	-0.9724	3.009712	0.000978
ENSG0000(	RF00066	-0.97268	1.712778	0.019374
ENSG0000(	MYLPF	-0.97282	1.572028	0.02679
ENSG0000(	TXLNB	-0.97433	1.942087	0.011426
ENSG0000(	TMEM120I	-0.97442	8.985161	1.03E-09
ENSG0000(	MBP	-0.97567	5.061903	8.67E-06
ENSG0000(	DVL1	-0.97581	4.026473	9.41E-05
ENSG0000(	CNNM4	-0.97619	4.038399	9.15E-05
ENSG0000(	HSF2	-0.97698	4.994474	1.01E-05
ENSG0000(	KBTBD12	-0.97729	1.95764	0.011025
ENSG0000(	COL4A4	-0.9803	3.590279	0.000257
ENSG0000(	AC005618.	-0.98086	1.768138	0.017055
ENSG0000(	EGFLAM-A!	-0.98387	1.475529	0.033456
ENSG0000(	FAAH	-0.98425	7.355796	4.41E-08
ENSG0000(	SIX2	-0.98482	4.991883	1.02E-05
ENSG0000(	EIF4BP3	-0.98832	2.095155	0.008032
ENSG0000(	AC241952.	-0.98938	2.850597	0.001411
ENSG0000(	RPL7P3	-0.98953	1.521705	0.030081
ENSG0000(	ASB8	-0.99174	4.754415	1.76E-05
ENSG0000(	RAPGEFL1	-0.99177	4.591475	2.56E-05
ENSG0000(	WRB-SH3B	-0.9921	2.432445	0.003694
ENSG0000(	MRPL23-A!	-0.99352	1.973173	0.010637
ENSG0000(	MAP3K9	-0.99357	2.932741	0.001168
ENSG0000(	TRIB1	-0.99585	3.958272	0.00011
ENSG0000(	AL138781.:	-0.99601	1.549478	0.028218
ENSG0000(	GAPDH	-0.99616	2.519458	0.003024
ENSG0000(	SLC16A3	-0.99703	1.304395	0.049614
ENSG0000(	CEP85	-0.9974	2.583552	0.002609

ENSG0000(	MSANTD1	-0.9994	4.752245	1.77E-05
ENSG0000(	HMGB1P1(	-0.99973	2.60142	0.002504
ENSG0000(	TMTC4	-1.00005	8.739668	1.82E-09
ENSG0000(	TMEM246	-1.00084	5.2654	5.43E-06
ENSG0000(	ADCY1	-1.0031	3.757655	0.000175
ENSG0000(	BX927359.	-1.0041	2.08519	0.008219
ENSG0000(	SH2B2	-1.00545	3.016801	0.000962
ENSG0000(	TAPT1	-1.00552	7.782424	1.65E-08
ENSG0000(	ZNF76	-1.00568	9.221361	6.01E-10
ENSG0000(	AL353622.:	-1.00846	2.385061	0.00412
ENSG0000(	DCUN1D2	-1.00859	4.291842	5.11E-05
ENSG0000(	RAB12	-1.00957	6.485688	3.27E-07
ENSG0000(	KCNJ11	-1.00958	1.719426	0.01908
ENSG0000(	KRBA1	-1.01006	5.320276	4.78E-06
ENSG0000(	MIR6808	-1.01116	3.823168	0.00015
ENSG0000(	HJV	-1.01123	1.797051	0.015957
ENSG0000(	VWA8	-1.01245	3.499489	0.000317
ENSG0000(	AL158850.:	-1.0126	1.895429	0.012722
ENSG0000(	AC105052.	-1.01308	2.681463	0.002082
ENSG0000(	XK	-1.01427	1.417353	0.038251
ENSG0000(	MUM1L1	-1.01501	2.219914	0.006027
ENSG0000(	ZNF853	-1.01634	4.174381	6.69E-05
ENSG0000(	AC006064.	-1.0165	2.714895	0.001928
ENSG0000(	TACC2	-1.01774	3.117069	0.000764
ENSG0000(	KBTBD11	-1.01779	2.572903	0.002674
ENSG0000(	AAMDC	-1.01797	4.150249	7.08E-05
ENSG0000(	UBE2G1	-1.01983	3.945381	0.000113
ENSG0000(	PLCD4	-1.02019	3.319317	0.000479
ENSG0000(	SH3BGR	-1.02148	1.947208	0.011293
ENSG0000(	CEBPB-AS1	-1.02225	3.728218	0.000187
ENSG0000(	KLHL38	-1.02233	1.514069	0.030615
ENSG0000(	SBK1	-1.02372	2.096369	0.00801
ENSG0000(	ZCWPW2	-1.02441	5.436844	3.66E-06
ENSG0000(	APIP	-1.02554	7.006442	9.85E-08
ENSG0000(	TRDN	-1.02565	1.594049	0.025465
ENSG0000(	PKDCC	-1.02591	3.998273	0.0001
ENSG0000(	CLTCL1	-1.02635	2.499532	0.003166
ENSG0000(	AC020907.	-1.02657	4.842167	1.44E-05
ENSG0000(	MIR193BH(	-1.02785	4.638346	2.30E-05
ENSG0000(	ALPK3	-1.02795	1.796576	0.015974
ENSG0000(	MYLK2	-1.0292	1.495515	0.031951
ENSG0000(	MID1IP1	-1.03102	6.12695	7.47E-07
ENSG0000(	TBC1D4	-1.0321	3.761415	0.000173
ENSG0000(	AP002812.	-1.03319	4.060935	8.69E-05
ENSG0000(	TRIP10	-1.03416	11.14685	7.13E-12
ENSG0000(	RPS20P21	-1.03464	1.931074	0.01172
ENSG0000(	AL645941.:	-1.03599	3.261966	0.000547

ENSG0000(	PGM2L1	-1.03602	5.105055	7.85E-06
ENSG0000(	ZNF204P	-1.03607	4.651181	2.23E-05
ENSG0000(	MROH7	-1.03617	2.263138	0.005456
ENSG0000(	FLNC-AS1	-1.03653	1.833749	0.014664
ENSG0000(	TCF15	-1.03766	1.706633	0.01965
ENSG0000(	SLC16A10	-1.03767	1.825473	0.014946
ENSG0000(	DMD	-1.03783	3.407012	0.000392
ENSG0000(	FLNC	-1.0383	1.881558	0.013135
ENSG0000(	CHRNA10	-1.03882	2.078444	0.008347
ENSG0000(	TNK2	-1.03923	6.926463	1.18E-07
ENSG0000(	AP002812.	-1.03981	4.187126	6.50E-05
ENSG0000(	AC012513.	-1.0426	4.610887	2.45E-05
ENSG0000(	OSBPL7	-1.04344	3.04086	0.00091
ENSG0000(	CLCN1	-1.04422	1.731348	0.018563
ENSG0000(	AC126755.	-1.04638	3.232082	0.000586
ENSG0000(	CCNG1	-1.04689	5.416346	3.83E-06
ENSG0000(	AC012510.	-1.04712	3.669575	0.000214
ENSG0000(	AC104564.	-1.04745	2.027749	0.009381
ENSG0000(	GLUL	-1.04781	3.51625	0.000305
ENSG0000(	AC008079.	-1.04919	2.373411	0.004232
ENSG0000(	AC108134.	-1.0498	1.74293	0.018075
ENSG0000(	AC083843.	-1.05084	3.115743	0.000766
ENSG0000(	RIF1	-1.05286	2.655464	0.002211
ENSG0000(	OBSCN-AS1	-1.0538	1.547989	0.028315
ENSG0000(	PGAM2	-1.05481	1.937053	0.01156
ENSG0000(	AP001793.	-1.05525	5.732887	1.85E-06
ENSG0000(	ASB10	-1.05559	1.846307	0.014246
ENSG0000(	GAMT	-1.05608	2.328013	0.004699
ENSG0000(	KCNA7	-1.05666	1.799632	0.015862
ENSG0000(	AC017116.	-1.05895	1.911809	0.012252
ENSG0000(	NKAIN2	-1.05905	1.337579	0.045964
ENSG0000(	AP000695.	-1.05973	1.646862	0.02255
ENSG0000(	MYO18B	-1.06098	1.769342	0.017008
ENSG0000(	PNCK	-1.06124	2.916548	0.001212
ENSG0000(	GPIHBP1	-1.06217	3.137377	0.000729
ENSG0000(	SGCA	-1.06248	2.938919	0.001151
ENSG0000(	AL353593.:	-1.06335	1.764764	0.017188
ENSG0000(	TUBA8	-1.06481	2.267837	0.005397
ENSG0000(	IMPA2	-1.06617	4.196266	6.36E-05
ENSG0000(	DUSP8	-1.06643	3.185459	0.000652
ENSG0000(	SYNM	-1.06864	2.058555	0.008739
ENSG0000(	TOB2	-1.0706	6.881218	1.31E-07
ENSG0000(	ATP1A2	-1.07177	2.031947	0.009291
ENSG0000(	PLEKHF1	-1.07182	4.258861	5.51E-05
ENSG0000(	SOX6	-1.07394	3.920625	0.00012
ENSG0000(	FKBP5	-1.07419	1.683278	0.020736
ENSG0000(	ZNF579	-1.07432	4.414512	3.85E-05

ENSG0000(	AL358473.:	-1.07526	1.416505	0.038326
ENSG0000(	TMEM38A	-1.07559	2.071984	0.008473
ENSG0000(	MYBPC1	-1.07615	1.813818	0.015353
ENSG0000(	PTP4A3	-1.07628	2.780565	0.001657
ENSG0000(	EEF1B2P3	-1.07635	6.061258	8.68E-07
ENSG0000(	AC008760.	-1.07659	15.43215	3.70E-16
ENSG0000(	AC109583.	-1.07719	1.488629	0.032462
ENSG0000(	AC125807.	-1.07835	2.505476	0.003123
ENSG0000(	CFAP58	-1.07848	2.562762	0.002737
ENSG0000(	MIR600HG	-1.07944	9.919708	1.20E-10
ENSG0000(	MDH2	-1.08039	4.683722	2.07E-05
ENSG0000(	SCN1B	-1.0823	3.724651	0.000189
ENSG0000(	AL353593.:	-1.08237	1.647534	0.022515
ENSG0000(	HRASLS	-1.08477	2.057705	0.008756
ENSG0000(	AL135905.:	-1.08543	3.891269	0.000128
ENSG0000(	PTP4A1	-1.08964	3.925131	0.000119
ENSG0000(	Z98949.1	-1.09139	1.791988	0.016144
ENSG0000(	ATP1B1	-1.09288	2.667996	0.002148
ENSG0000(	AC036108.	-1.09388	1.93238	0.011685
ENSG0000(	LONRF2	-1.0942	4.238302	5.78E-05
ENSG0000(	DHCR24	-1.09608	2.009582	0.009782
ENSG0000(	AL138689.:	-1.09702	6.301016	5.00E-07
ENSG0000(	MYOZ3	-1.0991	2.207219	0.006206
ENSG0000(	GPR157	-1.10163	2.6941	0.002023
ENSG0000(	MAPK12	-1.10203	5.17275	6.72E-06
ENSG0000(	MIR6505	-1.10258	1.429971	0.037156
ENSG0000(	MAP3K20-/	-1.10287	2.110003	0.007762
ENSG0000(	AC020904.	-1.10471	3.099744	0.000795
ENSG0000(	FABP5P3	-1.10502	1.385845	0.04113
ENSG0000(	CEBPB	-1.10981	4.177637	6.64E-05
ENSG0000(	NTF4	-1.1104	1.404545	0.039396
ENSG0000(	AP003469.	-1.11074	6.852061	1.41E-07
ENSG0000(	AP001542.	-1.11097	7.41425	3.85E-08
ENSG0000(	NOS1	-1.11262	1.338526	0.045864
ENSG0000(	SCARNA15	-1.11339	2.800394	0.001583
ENSG0000(	AMY2B	-1.11362	12.10091	7.93E-13
ENSG0000(	LINC02097	-1.11426	1.427939	0.03733
ENSG0000(	CHDH	-1.11548	5.373846	4.23E-06
ENSG0000(	PRKAA2	-1.11666	2.529479	0.002955
ENSG0000(	ERBB4	-1.11768	1.895986	0.012706
ENSG0000(	AL390208.:	-1.11797	4.580093	2.63E-05
ENSG0000(	MLXIP	-1.11813	6.581147	2.62E-07
ENSG0000(	RNF128	-1.11818	1.554908	0.027867
ENSG0000(	LRRC14B	-1.11882	1.86249	0.013725
ENSG0000(	OBSCN	-1.12145	1.989401	0.010247
ENSG0000(	MYOZ1	-1.1223	2.254067	0.005571
ENSG0000(	SESN1	-1.12248	5.023724	9.47E-06

ENSG0000(	AC096536.	-1.12357	1.818469	0.015189
ENSG0000(	AC117505.	-1.12358	1.921217	0.011989
ENSG0000(	AC044839.	-1.12378	1.430664	0.037097
ENSG0000(	CES3	-1.12414	2.46572	0.003422
ENSG0000(	AL136164.4	-1.12438	4.307028	4.93E-05
ENSG0000(	MKNK2	-1.12502	3.354724	0.000442
ENSG0000(	GMPR	-1.12563	3.161452	0.00069
ENSG0000(	AC068700.	-1.12971	1.729476	0.018643
ENSG0000(	AGMAT	-1.13229	1.894822	0.01274
ENSG0000(	TNK2-AS1	-1.13301	8.582952	2.61E-09
ENSG0000(	CLDN9	-1.13371	3.724298	0.000189
ENSG0000(	GOLGA2P7	-1.13424	2.644384	0.002268
ENSG0000(	SNHG5	-1.13555	4.870235	1.35E-05
ENSG0000(	MBNL1-AS1	-1.13605	3.541799	0.000287
ENSG0000(	MAN2A2	-1.1365	9.036727	9.19E-10
ENSG0000(	DUSP26	-1.13952	2.154543	0.007006
ENSG0000(	ABCC6	-1.14078	1.953167	0.011139
ENSG0000(	AC036108.	-1.14136	1.912856	0.012222
ENSG0000(	HIST1H1C	-1.1414	4.416774	3.83E-05
ENSG0000(	CNTFR-AS1	-1.14157	2.258594	0.005513
ENSG0000(	MYHAS	-1.14217	1.70095	0.019909
ENSG0000(	ARRDC2	-1.14304	3.687883	0.000205
ENSG0000(	PRKN	-1.14356	3.472139	0.000337
ENSG0000(	ZNF154	-1.1439	4.545523	2.85E-05
ENSG0000(	LINC01128	-1.14488	4.544719	2.85E-05
ENSG0000(	AL158070.1	-1.14569	1.750474	0.017763
ENSG0000(	PPP1R1A	-1.14587	2.484508	0.003277
ENSG0000(	SOX9-AS1	-1.14646	2.249442	0.005631
ENSG0000(	KIF1C	-1.14705	4.097377	7.99E-05
ENSG0000(	DUSP3	-1.14856	5.1048	7.86E-06
ENSG0000(	GADD45GI	-1.15134	8.838792	1.45E-09
ENSG0000(	BOLL	-1.15136	2.286535	0.00517
ENSG0000(	TSPYL1	-1.15514	5.405141	3.93E-06
ENSG0000(	AC068831.	-1.15533	12.65553	2.21E-13
ENSG0000(	SMCO1	-1.15569	2.084215	0.008237
ENSG0000(	SNHG14	-1.15582	5.225237	5.95E-06
ENSG0000(	TRDN-AS1	-1.1603	2.219914	0.006027
ENSG0000(	LINGO4	-1.16064	1.36655	0.042998
ENSG0000(	AL589880.1	-1.16507	4.128656	7.44E-05
ENSG0000(	SNORA31	-1.16521	3.494863	0.00032
ENSG0000(	AC005523.	-1.16544	2.643378	0.002273
ENSG0000(	HPCAL4	-1.16805	1.68393	0.020705
ENSG0000(	MIR29C	-1.16884	4.653763	2.22E-05
ENSG0000(	SLC6A8	-1.17052	4.369841	4.27E-05
ENSG0000(	AP001107.	-1.1712	3.871729	0.000134
ENSG0000(	NKAPL	-1.17191	8.885473	1.30E-09
ENSG0000(	CXorf57	-1.17322	3.675356	0.000211

ENSG0000(	PLEKHN1	-1.17486	1.883467	0.013078
ENSG0000(	ITGB3BP	-1.17932	3.203898	0.000625
ENSG0000(	SLC37A4	-1.17962	6.16828	6.79E-07
ENSG0000(	AC022784.	-1.18065	3.328353	0.00047
ENSG0000(	TUBA4A	-1.1813	2.910667	0.001228
ENSG0000(	HABP2	-1.18138	2.340312	0.004568
ENSG0000(	BUB1B-PAK	-1.1829	3.852371	0.00014
ENSG0000(	FHL3	-1.18315	2.240805	0.005744
ENSG0000(	FEM1A	-1.18339	2.7404	0.001818
ENSG0000(	TSC22D1-A	-1.18368	2.586102	0.002594
ENSG0000(	AC090241.	-1.185	1.93783	0.011539
ENSG0000(	AL121992.:	-1.18538	4.301752	4.99E-05
ENSG0000(	NRAP	-1.18605	2.195129	0.006381
ENSG0000(	FAM78A	-1.1863	4.890103	1.29E-05
ENSG0000(	AC000078.	-1.191	2.374428	0.004223
ENSG0000(	AC021087.	-1.19148	1.544547	0.02854
ENSG0000(	AC004771.	-1.19802	4.332316	4.65E-05
ENSG0000(	PITX1	-1.19955	1.51329	0.03067
ENSG0000(	ACSL6	-1.19982	3.553278	0.00028
ENSG0000(	HOXA13	-1.19986	1.378651	0.041817
ENSG0000(	PERM1	-1.20344	1.799368	0.015872
ENSG0000(	IP6K3	-1.20398	2.413879	0.003856
ENSG0000(	PPP1R3B	-1.2041	5.279806	5.25E-06
ENSG0000(	RASA4	-1.20455	3.442553	0.000361
ENSG0000(	LINC00310	-1.20525	3.276382	0.000529
ENSG0000(	MAFA	-1.20601	1.74789	0.017869
ENSG0000(	LRRC37A7F	-1.21013	1.714911	0.019279
ENSG0000(	ALDOA	-1.21016	2.718079	0.001914
ENSG0000(	AC093512.	-1.21028	2.716737	0.00192
ENSG0000(	NT5C1A	-1.21087	1.618722	0.024059
ENSG0000(	ABLIM2	-1.21386	2.820344	0.001512
ENSG0000(	GDAP1	-1.214	4.706393	1.97E-05
ENSG0000(	AP001099.	-1.21411	1.39241	0.040513
ENSG0000(	RORC	-1.21559	2.358968	0.004376
ENSG0000(	ASB12	-1.2186	2.888385	0.001293
ENSG0000(	SNTA1	-1.22402	3.40498	0.000394
ENSG0000(	VN1R1	-1.22559	4.612147	2.44E-05
ENSG0000(	AC012651.	-1.22847	3.747903	0.000179
ENSG0000(	HSPA2	-1.2309	2.783696	0.001646
ENSG0000(	CIART	-1.23114	2.577588	0.002645
ENSG0000(	SLC25A25	-1.23222	3.622483	0.000239
ENSG0000(	AC008443.	-1.23262	4.955818	1.11E-05
ENSG0000(	CAPN3	-1.23356	3.668329	0.000215
ENSG0000(	SLC47A1	-1.23452	3.708324	0.000196
ENSG0000(	MIR761	-1.23594	4.629678	2.35E-05
ENSG0000(	AC138904.	-1.23672	2.003291	0.009924
ENSG0000(	AC022706.	-1.23731	3.092624	0.000808

ENSG0000(	ACTA1	-1.23766	2.320901	0.004776
ENSG0000(	RN7SKP202	-1.23789	1.659149	0.021921
ENSG0000(	ASB4	-1.23958	2.237269	0.005791
ENSG0000(	JPH2	-1.23999	2.579764	0.002632
ENSG0000(	AC008014.	-1.24278	3.12027	0.000758
ENSG0000(	SLC29A2	-1.24389	3.21229	0.000613
ENSG0000(	SLC38A3	-1.24646	1.529993	0.029513
ENSG0000(	CSF3	-1.24739	1.315246	0.04839
ENSG0000(	PWAR6	-1.24961	5.453364	3.52E-06
ENSG0000(	LRRC20	-1.2512	2.944962	0.001135
ENSG0000(	ANKRD23	-1.25143	2.759139	0.001741
ENSG0000(	PWAR5	-1.25441	2.189906	0.006458
ENSG0000(	ANKRD39	-1.25526	2.65415	0.002217
ENSG0000(	TLL2	-1.2576	1.619195	0.024033
ENSG0000(	AC138474.	-1.25976	1.763901	0.017223
ENSG0000(	HES1	-1.26006	3.826959	0.000149
ENSG0000(	AC027045.	-1.2614	1.922378	0.011957
ENSG0000(	AC026398.	-1.26289	2.62006	0.002399
ENSG0000(	NEB	-1.2629	2.454583	0.003511
ENSG0000(	DHRS7C	-1.26292	2.327083	0.004709
ENSG0000(	PFKFB3	-1.2635	2.249669	0.005628
ENSG0000(	YBX3P1	-1.26369	2.003934	0.00991
ENSG0000(	LINC01996	-1.26396	1.856449	0.013917
ENSG0000(	AC092164.	-1.26447	5.223337	5.98E-06
ENSG0000(	CA2	-1.26751	3.264106	0.000544
ENSG0000(	AC091563.	-1.27004	6.23674	5.80E-07
ENSG0000(	PGM1	-1.27393	3.49417	0.000321
ENSG0000(	SELENOKP3	-1.27443	1.553432	0.027962
ENSG0000(	TLE1	-1.27496	5.898432	1.26E-06
ENSG0000(	EEF1A2	-1.27511	2.607828	0.002467
ENSG0000(	FLRT3	-1.27962	3.081897	0.000828
ENSG0000(	AL592437.1	-1.28188	1.412954	0.038641
ENSG0000(	AC093512.	-1.28262	2.530836	0.002946
ENSG0000(	VWA7	-1.2827	2.615027	0.002426
ENSG0000(	AC018553.	-1.28497	1.429015	0.037238
ENSG0000(	COL4A3	-1.28529	4.183032	6.56E-05
ENSG0000(	YBX3	-1.28567	4.769538	1.70E-05
ENSG0000(	SPEG	-1.28794	2.695311	0.002017
ENSG0000(	TNNC2	-1.28809	2.619805	0.0024
ENSG0000(	PPP1R3C	-1.28907	3.460119	0.000347
ENSG0000(	KBTBD13	-1.28909	1.869649	0.013501
ENSG0000(	DNAJB5	-1.28959	3.847108	0.000142
ENSG0000(	CYP17A1	-1.2908	3.772049	0.000169
ENSG0000(	GRTP1-AS1	-1.29122	6.466156	3.42E-07
ENSG0000(	KCNN2	-1.29191	1.717635	0.019159
ENSG0000(	PAK6	-1.29238	4.793357	1.61E-05
ENSG0000(	DOK5	-1.29634	4.814513	1.53E-05

ENSG0000(	TCEA3	-1.29635	4.46342	3.44E-05
ENSG0000(	CSMD1	-1.29725	1.890351	0.012872
ENSG0000(	SMIM2-AS:	-1.29756	1.731153	0.018572
ENSG0000(	PTPN20	-1.29766	3.685694	0.000206
ENSG0000(	AC036108.	-1.29912	4.53731	2.90E-05
ENSG0000(	SATB1	-1.2997	6.436583	3.66E-07
ENSG0000(	AC092611.	-1.29975	2.064273	0.008624
ENSG0000(	AC025627.	-1.30128	2.766761	0.001711
ENSG0000(	AC093668.	-1.3042	6.073788	8.44E-07
ENSG0000(	AC092611.	-1.30663	3.5093	0.00031
ENSG0000(	ITIH2	-1.31114	5.986063	1.03E-06
ENSG0000(	MIB2	-1.3129	12.20803	6.19E-13
ENSG0000(	PFKFB1	-1.31325	3.402848	0.000396
ENSG0000(	RGS9BP	-1.31878	1.625098	0.023708
ENSG0000(	SNORD116	-1.32215	1.797001	0.015959
ENSG0000(	PPM1J	-1.32702	3.016324	0.000963
ENSG0000(	SLC22A3	-1.32936	2.907484	0.001237
ENSG0000(	MUC3A	-1.33093	3.445523	0.000358
ENSG0000(	AC005616.	-1.33422	1.676103	0.021081
ENSG0000(	MN1	-1.33444	5.759334	1.74E-06
ENSG0000(	ACTG1P4	-1.33494	4.079357	8.33E-05
ENSG0000(	CMBL	-1.33738	4.619758	2.40E-05
ENSG0000(	AC010680.	-1.33752	2.674356	0.002117
ENSG0000(	PPP3CB	-1.33797	8.4832	3.29E-09
ENSG0000(	TRIM7	-1.33844	4.102956	7.89E-05
ENSG0000(	MYH13	-1.33863	2.422921	0.003776
ENSG0000(	PAIP2B	-1.34036	2.973516	0.001063
ENSG0000(	SYNPO	-1.34122	3.757864	0.000175
ENSG0000(	RABEP2	-1.34126	9.424759	3.76E-10
ENSG0000(	AC008474.	-1.34727	1.824002	0.014997
ENSG0000(	AC072022.	-1.34738	7.893654	1.28E-08
ENSG0000(	GSDMC	-1.34753	1.560238	0.027527
ENSG0000(	ENO3	-1.34894	3.279243	0.000526
ENSG0000(	RNU6-415I	-1.34992	1.425708	0.037522
ENSG0000(	CNKSR1	-1.35268	3.499991	0.000316
ENSG0000(	MYLK4	-1.36299	1.565148	0.027218
ENSG0000(	RPL3L	-1.36601	2.991452	0.00102
ENSG0000(	NKILA	-1.36771	5.075808	8.40E-06
ENSG0000(	PKP2	-1.37102	3.907071	0.000124
ENSG0000(	ATXN7L2	-1.37661	7.67095	2.13E-08
ENSG0000(	PYGM	-1.37767	2.831532	0.001474
ENSG0000(	MYBPC2	-1.37886	2.277292	0.005281
ENSG0000(	ALDH1L1-A	-1.3803	2.67261	0.002125
ENSG0000(	LRRC66	-1.38171	7.554994	2.79E-08
ENSG0000(	AC093330.	-1.38272	5.107236	7.81E-06
ENSG0000(	AL355377.:	-1.38385	1.91856	0.012063
ENSG0000(	MIR6073	-1.38466	3.694652	0.000202

ENSG0000(	AC004982.	-1.39315	2.142426	0.007204
ENSG0000(	RAD23A	-1.3961	6.98417	1.04E-07
ENSG0000(	TNNT3	-1.3978	3.092611	0.000808
ENSG0000(	ALDH1L1	-1.40509	3.683334	0.000207
ENSG0000(	CAMK2B	-1.41076	3.073618	0.000844
ENSG0000(	SNORD116	-1.41248	2.058754	0.008735
ENSG0000(	AC005520.	-1.41935	15.5693	2.70E-16
ENSG0000(	RHOBTB1	-1.41995	6.059581	8.72E-07
ENSG0000(	CRHR2	-1.43255	3.400927	0.000397
ENSG0000(	CABP1	-1.43556	1.722313	0.018953
ENSG0000(	AC008035.	-1.43626	4.027096	9.40E-05
ENSG0000(	MIR486-1	-1.43811	1.544547	0.02854
ENSG0000(	TMEM52	-1.44034	3.249384	0.000563
ENSG0000(	MIR29B2CI	-1.44194	6.640744	2.29E-07
ENSG0000(	AC010680.	-1.44784	2.094106	0.008052
ENSG0000(	MYORG	-1.44982	3.444029	0.00036
ENSG0000(	G0S2	-1.45359	3.691535	0.000203
ENSG0000(	AC009948.	-1.45381	1.423565	0.037708
ENSG0000(	AC006333.	-1.45444	2.945219	0.001134
ENSG0000(	MIR637	-1.45503	5.144863	7.16E-06
ENSG0000(	GADD45G	-1.45516	2.795465	0.001602
ENSG0000(	ADSSL1	-1.45808	3.280876	0.000524
ENSG0000(	MIR486-2	-1.45935	1.592338	0.025566
ENSG0000(	UNC13B	-1.46002	10.855	1.40E-11
ENSG0000(	AL355377.:	-1.46047	1.47853	0.033225
ENSG0000(	AC069360.	-1.46154	3.261661	0.000547
ENSG0000(	ABRA	-1.4649	2.447368	0.00357
ENSG0000(	TNNI2	-1.46816	2.993628	0.001015
ENSG0000(	PER1	-1.47319	4.678675	2.10E-05
ENSG0000(	GADL1	-1.47507	2.888022	0.001294
ENSG0000(	FAM160A1	-1.47795	2.696617	0.002011
ENSG0000(	AC022784.	-1.48198	5.768692	1.70E-06
ENSG0000(	CNTFR	-1.48399	8.960446	1.10E-09
ENSG0000(	DYRK1B	-1.48589	5.890212	1.29E-06
ENSG0000(	AC005393.	-1.4878	3.183477	0.000655
ENSG0000(	KCNT1	-1.4908	4.137594	7.28E-05
ENSG0000(	CACNA1S	-1.49257	3.587717	0.000258
ENSG0000(	UPK3A	-1.49447	2.019968	0.009551
ENSG0000(	MIR1-1HG	-1.49501	3.058512	0.000874
ENSG0000(	BCL6	-1.49771	7.695536	2.02E-08
ENSG0000(	CKM	-1.50373	3.283403	0.000521
ENSG0000(	WNT4	-1.50592	5.849388	1.41E-06
ENSG0000(	AC010680.	-1.51023	3.153268	0.000703
ENSG0000(	AC008443.	-1.5117	3.642968	0.000228
ENSG0000(	AC021148.	-1.51645	2.220613	0.006017
ENSG0000(	RF00322	-1.51742	6.095344	8.03E-07
ENSG0000(	TPM1	-1.52038	4.871591	1.34E-05

ENSG0000(	ASB15	-1.52142	3.456406	0.00035
ENSG0000(	MIR29B2	-1.5344	2.286555	0.005169
ENSG0000(	JPH1	-1.53744	3.669831	0.000214
ENSG0000(	AC010680.	-1.54252	3.054268	0.000883
ENSG0000(	TMEM266	-1.5466	5.892841	1.28E-06
ENSG0000(	MIR133A2	-1.55496	3.181496	0.000658
ENSG0000(	RYR1	-1.55732	3.520955	0.000301
ENSG0000(	ATP13A5-A	-1.56063	2.197727	0.006343
ENSG0000(	FO393414.	-1.56102	1.67946	0.020919
ENSG0000(	ALDH1L1-A	-1.56427	4.137609	7.28E-05
ENSG0000(	IRX3	-1.56656	2.885415	0.001302
ENSG0000(	AL513217.:	-1.57232	6.697704	2.01E-07
ENSG0000(	SLC7A2	-1.57378	4.698979	2.00E-05
ENSG0000(	AC067969.	-1.57487	3.607889	0.000247
ENSG0000(	LINC00240	-1.57508	2.295257	0.005067
ENSG0000(	PEBP4	-1.57879	4.010487	9.76E-05
ENSG0000(	KCNJ12	-1.58565	5.759334	1.74E-06
ENSG0000(	AC037441.	-1.58673	2.099731	0.007948
ENSG0000(	SH3RF2	-1.59133	4.227747	5.92E-05
ENSG0000(	AC008443.	-1.59298	5.517366	3.04E-06
ENSG0000(	AC124312.	-1.59454	3.243593	0.000571
ENSG0000(	ALDH8A1	-1.59705	1.561575	0.027443
ENSG0000(	AC009948.	-1.59961	1.544114	0.028568
ENSG0000(	RASA4B	-1.60153	6.336772	4.60E-07
ENSG0000(	GSTM1	-1.60226	1.446346	0.035781
ENSG0000(	AC072022.	-1.60815	6.128325	7.44E-07
ENSG0000(	AC124016.	-1.6116	2.828215	0.001485
ENSG0000(	LMOD1	-1.61381	7.103136	7.89E-08
ENSG0000(	AC123912.	-1.61644	1.941138	0.011451
ENSG0000(	TBX1	-1.61842	2.352043	0.004446
ENSG0000(	MSS51	-1.61928	4.848212	1.42E-05
ENSG0000(	ATP13A5	-1.62216	3.073757	0.000844
ENSG0000(	DENND2C	-1.62297	6.943008	1.14E-07
ENSG0000(	RASL10B	-1.63421	5.260789	5.49E-06
ENSG0000(	CMYA5	-1.63758	4.093607	8.06E-05
ENSG0000(	AL117340.:	-1.6445	2.686919	0.002056
ENSG0000(	TTN-AS1	-1.64498	3.865146	0.000136
ENSG0000(	TLR9	-1.65917	5.89357	1.28E-06
ENSG0000(	TTN	-1.6594	3.892396	0.000128
ENSG0000(	CDH20	-1.65996	2.96785	0.001077
ENSG0000(	B3GALT1	-1.66445	2.778676	0.001665
ENSG0000(	AC067969.	-1.66963	2.966358	0.001081
ENSG0000(	CD38	-1.6738	7.478154	3.33E-08
ENSG0000(	AC091100.	-1.67405	4.005769	9.87E-05
ENSG0000(	AC005323.	-1.6752	3.587864	0.000258
ENSG0000(	HAPLN2	-1.69775	1.436851	0.036572
ENSG0000(	CFAP53	-1.70637	3.466951	0.000341

ENSG0000(	NEK10	-1.71029	5.879186	1.32E-06
ENSG0000(	BCAP31P2	-1.71039	2.015365	0.009652
ENSG0000(	CHAF1B	-1.71883	6.621272	2.39E-07
ENSG0000(	CFAP46	-1.72055	3.483665	0.000328
ENSG0000(	USP6	-1.72492	4.569576	2.69E-05
ENSG0000(	COL28A1	-1.73217	9.95286	1.11E-10
ENSG0000(	KCNQ5	-1.73743	3.814847	0.000153
ENSG0000(	SNORD116	-1.74873	1.552454	0.028025
ENSG0000(	ATP2A1-AS	-1.7494	3.521865	0.000301
ENSG0000(	ART3	-1.74965	4.992177	1.02E-05
ENSG0000(	LINC01886	-1.75724	2.228134	0.005914
ENSG0000(	RPL26P6	-1.76026	4.603865	2.49E-05
ENSG0000(	PRSS45	-1.76323	1.839445	0.014473
ENSG0000(	ATP2A1	-1.76433	3.644787	0.000227
ENSG0000(	NKAIN1	-1.77322	4.868394	1.35E-05
ENSG0000(	PITX3	-1.77851	3.301076	0.0005
ENSG0000(	AC095055.	-1.78	2.535009	0.002917
ENSG0000(	AC016705.	-1.8061	2.22301	0.005984
ENSG0000(	KCTD8	-1.82758	1.723706	0.018893
ENSG0000(	RHOXF1-AS	-1.85027	1.438646	0.036421
ENSG0000(	MYH1	-1.87312	3.046253	0.000899
ENSG0000(	AC005323.	-1.87479	4.282799	5.21E-05
ENSG0000(	BCAP31P1	-1.87799	2.0532	0.008847
ENSG0000(	AQP4	-1.87843	3.510004	0.000309
ENSG0000(	MYH2	-1.89561	4.518072	3.03E-05
ENSG0000(	AC024610.	-1.92758	2.130468	0.007405
ENSG0000(	AC012146.	-1.92904	4.562986	2.74E-05
ENSG0000(	RNF157	-1.97571	6.43653	3.66E-07
ENSG0000(	METTL21EI	-1.97862	2.346868	0.004499
ENSG0000(	AC034187.	-1.98322	4.079404	8.33E-05
ENSG0000(	AC004147.	-1.98866	1.899658	0.012599
ENSG0000(	ATP2B2	-1.99487	4.574614	2.66E-05
ENSG0000(	RNF157-AS	-1.99931	6.504402	3.13E-07
ENSG0000(	CFAP61	-2.00641	5.526503	2.98E-06
ENSG0000(	AC009948.	-2.00714	2.154562	0.007005
ENSG0000(	GDA	-2.04913	2.806286	0.001562
ENSG0000(	AL137246.:	-2.0773	2.411814	0.003874
ENSG0000(	ACTN3	-2.08535	3.225236	0.000595
ENSG0000(	AP002748.	-2.08585	3.196862	0.000636
ENSG0000(	AL445253.:	-2.12349	1.420541	0.037972
ENSG0000(	ODF3L2	-2.14517	6.33594	4.61E-07
ENSG0000(	NOTUM	-2.15693	4.679794	2.09E-05
ENSG0000(	HCN1	-2.15784	2.045191	0.009012
ENSG0000(	NOC2LP1	-2.16894	1.403153	0.039523
ENSG0000(	ANKRD20A	-2.18679	6.890971	1.29E-07
ENSG0000(	HBA2	-2.24552	2.22733	0.005925
ENSG0000(	CYP4F35P	-2.27442	4.029152	9.35E-05

ENSG0000(	HBB	-2.3576	2.614606	0.002429
ENSG0000(	AC018467.	-2.37052	2.045925	0.008997
ENSG0000(	C1orf127	-2.3729	8.623986	2.38E-09
ENSG0000(	NANOS1	-2.38666	8.686765	2.06E-09
ENSG0000(	FAM166B	-2.41817	9.621172	2.39E-10
ENSG0000(	UNC13C	-2.42829	2.834599	0.001464
ENSG0000(	SH2D1B	-2.45663	4.512663	3.07E-05
ENSG0000(	AJ009632.2	-2.46667	3.136233	0.000731
ENSG0000(	AP005212.	-2.46765	5.480002	3.31E-06
ENSG0000(	OR7E47P	-2.50787	4.809291	1.55E-05
ENSG0000(	OR7E47P	-2.51412	5.098105	7.98E-06
ENSG0000(	CALML6	-2.52329	5.946097	1.13E-06
ENSG0000(	KCNJ3	-2.55662	1.63124	0.023375
ENSG0000(	AC079467.	-2.61732	5.146052	7.14E-06
ENSG0000(	PKN2-AS1	-2.73138	2.269767	0.005373
ENSG0000(	GGT7	-2.75745	24.41093	3.88E-25
ENSG0000(	HBA1	-2.76047	3.710087	0.000195
ENSG0000(	AC044839.	-2.83802	2.287074	0.005163
ENSG0000(	RPS4XP5	-2.93273	1.839094	0.014485
ENSG0000(	LINC01018	-2.94227	1.355794	0.044076
ENSG0000(	GDNF-AS1	-3.00898	9.167176	6.80E-10
ENSG0000(	CCDC160	-3.099	1.399421	0.039864
ENSG0000(	GRIK2	-3.17891	4.19245	6.42E-05
ENSG0000(	GDNF	-3.3562	13.3719	4.25E-14
ENSG0000(	AP002884.	-3.70781	1.603368	0.024925
ENSG0000(	LINC01748	-3.86003	1.51839	0.030312
ENSG0000(	SNORD116	-3.86521	1.301297	0.049969
ENSG0000(	LINC01854	-4.19331	8.554036	2.79E-09
ENSG0000(	RNU2-72P	-4.49173	1.341371	0.045565
ENSG0000(	CDCA4P4	-4.79094	1.394075	0.040358
ENSG0000(	AC115284.	-4.97557	1.839445	0.014473
ENSG0000(	AC133561.	-5.02719	1.484021	0.032808
ENSG0000(	AL392183.:	-5.10748	2.277558	0.005278
ENSG0000(	LINC02107	-5.19241	2.300795	0.005003
ENSG0000(	AP005131.	-5.56745	1.309295	0.049057
ENSG0000(	MEI4	-5.77908	1.94491	0.011352
ENSG0000(	LINC02119	-5.81815	2.864778	0.001365
ENSG0000(	LINC00330	-6.16317	1.615679	0.024228

Supplemental Table 3: Differenctially expressed genes in females with inclusion body myositis

name	description	log2FoldCh	pvalue	padj
ENSG0000(	PADI3	22.41593	14.93127	1.17E-15
ENSG0000(	PLA2G2D	9.634275	19.07881	8.34E-20
ENSG0000(	IGHV4-59	9.583385	25.56935	2.70E-26
ENSG0000(	IGHJ2	9.348471	22.648	2.25E-23
ENSG0000(	IGHV1-69D	9.307265	20.16449	6.85E-21
ENSG0000(	RGS1	9.225973	19.62996	2.34E-20
ENSG0000(	KLHDC7B	8.925218	18.07089	8.49E-19
ENSG0000(	IGKV1-6	8.860099	19.66049	2.19E-20
ENSG0000(	TDO2	8.711265	17.47661	3.34E-18
ENSG0000(	IGLV8-61	8.609965	20.10672	7.82E-21
ENSG0000(	IGHV3-49	8.535706	16.86591	1.36E-17
ENSG0000(	IGLV6-57	8.519672	20.86686	1.36E-21
ENSG0000(	ZNF683	8.480979	13.28064	5.24E-14
ENSG0000(	IGHV3-43	8.444078	7.363655	4.33E-08
ENSG0000(	CCL4	8.44352	21.04348	9.05E-22
ENSG0000(	CCL8	8.400279	20.70285	1.98E-21
ENSG0000(	IGKV1D-39	8.361667	14.55695	2.77E-15
ENSG0000(	TIFAB	8.351627	16.43344	3.69E-17
ENSG0000(	SLAMF1	8.298933	20.41953	3.81E-21
ENSG0000(	IGKV6-21	8.272459	12.15211	7.05E-13
ENSG0000(	IGLV4-69	8.252592	12.00058	9.99E-13
ENSG0000(	CCL19	8.247244	15.82165	1.51E-16
ENSG0000(	FAM30A	8.220353	14.15605	6.98E-15
ENSG0000(	IGHV4-61	8.179484	14.62255	2.38E-15
ENSG0000(	AC243829.	8.168392	17.79983	1.59E-18
ENSG0000(	PDCD1	8.100247	16.43344	3.69E-17
ENSG0000(	CCL3	8.000614	16.8713	1.34E-17
ENSG0000(	MCOLN2	7.976279	15.82611	1.49E-16
ENSG0000(	IGLC7	7.968185	9.482838	3.29E-10
ENSG0000(	IGHV3-20	7.957344	9.985021	1.04E-10
ENSG0000(	TMPRSS3	7.90561	13.99961	1.00E-14
ENSG0000(	IGHV1-24	7.877051	13.60935	2.46E-14
ENSG0000(	IGHV4-31	7.829283	7.19752	6.35E-08
ENSG0000(	CXCL13	7.816098	15.11853	7.61E-16
ENSG0000(	CHIT1	7.792509	8.978384	1.05E-09
ENSG0000(	C4orf50	7.792332	17.80023	1.58E-18
ENSG0000(	AL671883.:	7.743056	14.24352	5.71E-15
ENSG0000(	IGKV3-7	7.722681	11.61484	2.43E-12
ENSG0000(	CYSLTR2	7.685441	17.74402	1.80E-18
ENSG0000(	IGHV1-69	7.636961	9.968287	1.08E-10
ENSG0000(	TNFRSF18	7.636951	14.59667	2.53E-15
ENSG0000(	DCANP1	7.621676	14.20473	6.24E-15
ENSG0000(	JAKMIP1	7.611733	14.41418	3.85E-15
ENSG0000(	DMBT1	7.592056	10.62691	2.36E-11
ENSG0000(	TREM2	7.582467	14.06437	8.62E-15

ENSG0000(	SCG2	7.575385	12.12598	7.48E-13
ENSG0000(	TRBJ2-7	7.573634	14.22529	5.95E-15
ENSG0000(	FCRL5	7.558441	10.48187	3.30E-11
ENSG0000(	TOX	7.545708	19.69248	2.03E-20
ENSG0000(	TRBJ2-1	7.525403	11.6763	2.11E-12
ENSG0000(	XCL1	7.516256	10.9277	1.18E-11
ENSG0000(	LINC02593	7.476304	18.70422	1.98E-19
ENSG0000(	LINC00892	7.470019	12.9104	1.23E-13
ENSG0000(	CRTAM	7.459984	11.44307	3.61E-12
ENSG0000(	TNF	7.440065	15.71509	1.93E-16
ENSG0000(	GPR171	7.422052	13.31737	4.82E-14
ENSG0000(	FASLG	7.415266	13.57326	2.67E-14
ENSG0000(	IGKV2-29	7.405426	8.755224	1.76E-09
ENSG0000(	NUGGC	7.401207	15.13631	7.31E-16
ENSG0000(	IGLV5-45	7.343783	6.574204	2.67E-07
ENSG0000(	IGLV7-43	7.284754	7.685712	2.06E-08
ENSG0000(	IGKV2D-28	7.267535	6.54151	2.87E-07
ENSG0000(	CALML3	7.266531	3.131939	0.000738
ENSG0000(	DUSP15	7.264367	22.19992	6.31E-23
ENSG0000(	IGHV3-66	7.255793	6.60316	2.49E-07
ENSG0000(	IFNG	7.224172	12.5245	2.99E-13
ENSG0000(	AC010605.	7.203157	12.59307	2.55E-13
ENSG0000(	AIM2	7.195377	14.72311	1.89E-15
ENSG0000(	LINC02384	7.183952	11.88666	1.30E-12
ENSG0000(	TNFRSF9	7.177629	10.86704	1.36E-11
ENSG0000(	MIR3142H(	7.148369	14.73585	1.84E-15
ENSG0000(	XCL2	7.143444	7.777768	1.67E-08
ENSG0000(	C15orf53	7.135011	12.56436	2.73E-13
ENSG0000(	IGKV3D-15	7.102826	6.881517	1.31E-07
ENSG0000(	Z84488.1	7.101176	12.35	4.47E-13
ENSG0000(	IGHV6-1	7.099071	7.528832	2.96E-08
ENSG0000(	IGHV3-13	7.097965	4.500171	3.16E-05
ENSG0000(	AL732437.:	7.079495	2.532161	0.002937
ENSG0000(	ADAMDEC:	7.062996	8.629971	2.34E-09
ENSG0000(	LAMP3	7.058941	12.96098	1.09E-13
ENSG0000(	P2RY10	7.056163	12.00966	9.78E-13
ENSG0000(	LINC00944	7.032046	13.45496	3.51E-14
ENSG0000(	IGHV4-55	7.012322	5.818798	1.52E-06
ENSG0000(	IL10	6.985921	11.96757	1.08E-12
ENSG0000(	IGLV10-54	6.973299	4.991234	1.02E-05
ENSG0000(	CTLA4	6.960306	10.31082	4.89E-11
ENSG0000(	SPINK2	6.959912	7.405726	3.93E-08
ENSG0000(	LINC00426	6.958034	14.30174	4.99E-15
ENSG0000(	AC060234.	6.94678	12.69858	2.00E-13
ENSG0000(	HRASLS2	6.945303	8.324667	4.74E-09
ENSG0000(	C2-AS1	6.94268	12.58709	2.59E-13
ENSG0000(	CCR4	6.939899	12.98156	1.04E-13

ENSG0000(	ANKRD22	6.937245	8.704828	1.97E-09
ENSG0000(	TRBJ1-5	6.908501	10.76644	1.71E-11
ENSG0000(	TRIM46	6.906173	17.72791	1.87E-18
ENSG0000(	C19orf81	6.899251	14.14079	7.23E-15
ENSG0000(	TRGC2	6.85515	10.42342	3.77E-11
ENSG0000(	SOWAHD	6.848989	8.633759	2.32E-09
ENSG0000(	CEACAM21	6.833581	11.72104	1.90E-12
ENSG0000(	RPTN	6.828663	6.052997	8.85E-07
ENSG0000(	RF01877	6.828569	10.0265	9.41E-11
ENSG0000(	ASCL2	6.827372	10.36145	4.35E-11
ENSG0000(	NCR3	6.826494	11.79167	1.62E-12
ENSG0000(	LINC01934	6.824545	11.81064	1.55E-12
ENSG0000(	LINC02257	6.821462	13.76736	1.71E-14
ENSG0000(	IGHG4	6.806281	12.03013	9.33E-13
ENSG0000(	IGHV1-18	6.798911	11.32908	4.69E-12
ENSG0000(	IGHV2-26	6.791104	11.19107	6.44E-12
ENSG0000(	IGKV1D-33	6.789866	6.210888	6.15E-07
ENSG0000(	EPOP	6.776494	18.37064	4.26E-19
ENSG0000(	RNA5SP33	6.747502	6.832509	1.47E-07
ENSG0000(	IGKV2-40	6.74511	3.519226	0.000303
ENSG0000(	WNT10A	6.732298	9.778728	1.66E-10
ENSG0000(	AL391832.	6.720928	11.74197	1.81E-12
ENSG0000(	AC244502.	6.720506	12.4885	3.25E-13
ENSG0000(	CCL4L2	6.719083	7.846998	1.42E-08
ENSG0000(	C15orf48	6.718619	9.464669	3.43E-10
ENSG0000(	CLNK	6.715811	11.90462	1.25E-12
ENSG0000(	AC078880.	6.68703	5.964614	1.08E-06
ENSG0000(	ICOS	6.677507	9.66229	2.18E-10
ENSG0000(	HCG4P8	6.670538	10.99796	1.00E-11
ENSG0000(	DTHD1	6.657719	8.219056	6.04E-09
ENSG0000(	IGHJ3P	6.649638	7.957913	1.10E-08
ENSG0000(	IGHV2-70	6.645053	4.02743	9.39E-05
ENSG0000(	BTLA	6.638628	9.356001	4.41E-10
ENSG0000(	CRYBG2	6.638532	6.968667	1.07E-07
ENSG0000(	TNFRSF13E	6.598381	5.601172	2.51E-06
ENSG0000(	IGHV3-69-	6.576925	5.437463	3.65E-06
ENSG0000(	GCSAM	6.570863	11.58134	2.62E-12
ENSG0000(	APOBEC3H	6.548121	9.182517	6.57E-10
ENSG0000(	GSDMA	6.544293	8.110751	7.75E-09
ENSG0000(	SERPINB12	6.530804	3.384496	0.000413
ENSG0000(	MFSD2A	6.500154	7.866029	1.36E-08
ENSG0000(	EBI3	6.499636	11.34538	4.51E-12
ENSG0000(	TRBV7-9	6.486269	5.470675	3.38E-06
ENSG0000(	IGHV3-48	6.474715	7.249458	5.63E-08
ENSG0000(	ADGRE4P	6.4685	6.72871	1.87E-07
ENSG0000(	MZB1	6.410254	12.72031	1.90E-13
ENSG0000(	ITPKA	6.409077	9.247406	5.66E-10

ENSG0000(	SLC8A2	6.403204	7.307185	4.93E-08
ENSG0000(	HLA-DRB9	6.400187	7.810073	1.55E-08
ENSG0000(	TRPV4	6.385315	14.3745	4.22E-15
ENSG0000(	PTPRH	6.380318	7.990119	1.02E-08
ENSG0000(	AC073283.	6.36377	7.746308	1.79E-08
ENSG0000(	AL160272.:	6.35787	9.577574	2.65E-10
ENSG0000(	PNOC	6.347211	5.089072	8.15E-06
ENSG0000(	LINC01871	6.34183	6.169695	6.77E-07
ENSG0000(	TRBJ1-1	6.333991	6.609278	2.46E-07
ENSG0000(	FLG-AS1	6.319762	7.742952	1.81E-08
ENSG0000(	IGHV4-28	6.310355	3.930507	0.000117
ENSG0000(	MTUS2	6.302913	4.713292	1.94E-05
ENSG0000(	AC015911.	6.298129	7.671963	2.13E-08
ENSG0000(	FLG	6.29206	9.296803	5.05E-10
ENSG0000(	AC008013.	6.28868	9.107614	7.81E-10
ENSG0000(	FRMPD3	6.277345	7.738612	1.83E-08
ENSG0000(	AP000282.	6.275069	7.110331	7.76E-08
ENSG0000(	SPNS3	6.273625	8.933472	1.17E-09
ENSG0000(	C4BPB	6.272813	7.302519	4.98E-08
ENSG0000(	TMIGD3	6.268445	6.374679	4.22E-07
ENSG0000(	IGLJ3	6.266248	14.20473	6.24E-15
ENSG0000(	IGLV2-28	6.263296	4.43677	3.66E-05
ENSG0000(	AC138207.	6.258252	11.01714	9.61E-12
ENSG0000(	AC046185.	6.247665	9.306347	4.94E-10
ENSG0000(	TRAJ39	6.245826	8.28117	5.23E-09
ENSG0000(	IGHV2-5	6.242992	7.940191	1.15E-08
ENSG0000(	DRAIC	6.238351	9.366115	4.30E-10
ENSG0000(	EDARADD	6.23032	9.084419	8.23E-10
ENSG0000(	AL096816.:	6.230063	8.820604	1.51E-09
ENSG0000(	LINC02446	6.217005	3.802517	0.000158
ENSG0000(	LCN15	6.214299	5.446743	3.57E-06
ENSG0000(	IGLV3-9	6.212549	8.086803	8.19E-09
ENSG0000(	SDS	6.203919	9.205701	6.23E-10
ENSG0000(	FCGR1B	6.183216	7.402629	3.96E-08
ENSG0000(	IGLV3-21	6.182978	18.40278	3.96E-19
ENSG0000(	RPS2P32	6.18283	9.279269	5.26E-10
ENSG0000(	IGHV5-51	6.168369	12.58411	2.61E-13
ENSG0000(	ABCB5	6.168333	3.079826	0.000832
ENSG0000(	IGHV3-11	6.165599	7.431556	3.70E-08
ENSG0000(	GCNT3	6.161321	6.078434	8.35E-07
ENSG0000(	EML2-AS1	6.158471	10.84705	1.42E-11
ENSG0000(	IGHGP	6.156193	23.05557	8.80E-24
ENSG0000(	AL033527.:	6.144421	7.6707	2.13E-08
ENSG0000(	WNT2	6.138081	2.650182	0.002238
ENSG0000(	IGHV3-7	6.130998	15.11853	7.61E-16
ENSG0000(	IGKV2-24	6.128849	7.160973	6.90E-08
ENSG0000(	RTKN2	6.115703	8.52527	2.98E-09

ENSG0000(	AC010503.	6.100279	8.562142	2.74E-09
ENSG0000(	GPR174	6.0937	8.776685	1.67E-09
ENSG0000(	TIMD4	6.090812	2.64601	0.002259
ENSG0000(	IGHV3-73	6.090133	6.687906	2.05E-07
ENSG0000(	AC015911.	6.082041	7.151814	7.05E-08
ENSG0000(	TRBV19	6.080965	3.111134	0.000774
ENSG0000(	FER1L6	6.068815	6.481572	3.30E-07
ENSG0000(	SPON1-AS1	6.063567	3.051331	0.000889
ENSG0000(	GPR156	6.06077	8.953335	1.11E-09
ENSG0000(	ZBED2	6.054822	7.119757	7.59E-08
ENSG0000(	CD1B	6.0537	5.784775	1.64E-06
ENSG0000(	STAP1	6.040934	3.454316	0.000351
ENSG0000(	TRBJ1-2	6.039686	6.131285	7.39E-07
ENSG0000(	IGHV1-69-2	6.038853	1.818961	0.015172
ENSG0000(	CCNA1	6.034067	4.32718	4.71E-05
ENSG0000(	TRAV19	6.028505	7.253112	5.58E-08
ENSG0000(	IGLV1-47	6.020265	10.85276	1.40E-11
ENSG0000(	AC091576.	6.012832	4.363939	4.33E-05
ENSG0000(	LINC00163	5.99555	3.603789	0.000249
ENSG0000(	TRBJ2-2	5.993341	6.022104	9.50E-07
ENSG0000(	STYK1	5.987121	5.11865	7.61E-06
ENSG0000(	CPNE7	5.982998	5.02393	9.46E-06
ENSG0000(	AL359752.1	5.980897	7.16728	6.80E-08
ENSG0000(	TRBJ1-4	5.979299	8.853554	1.40E-09
ENSG0000(	PLPPR1	5.970899	16.22211	6.00E-17
ENSG0000(	TRBV7-2	5.967861	7.07455	8.42E-08
ENSG0000(	ENHO	5.967323	7.873155	1.34E-08
ENSG0000(	RF00003	5.954263	8.902904	1.25E-09
ENSG0000(	IGLV4-60	5.953137	2.125896	0.007483
ENSG0000(	NPFFR2	5.950481	7.325127	4.73E-08
ENSG0000(	IGKV1D-16	5.925154	2.893269	0.001279
ENSG0000(	C1orf147	5.922963	7.065473	8.60E-08
ENSG0000(	AC020915.	5.922049	15.43134	3.70E-16
ENSG0000(	LINC00506	5.919243	14.89754	1.27E-15
ENSG0000(	FXYD4	5.919136	5.966779	1.08E-06
ENSG0000(	AC004585.	5.915926	7.213863	6.11E-08
ENSG0000(	WDR86-AS	5.909618	4.98409	1.04E-05
ENSG0000(	CXCL11	5.909247	14.74558	1.80E-15
ENSG0000(	SLC22A18A	5.908805	7.438396	3.64E-08
ENSG0000(	ERMN	5.90345	8.26306	5.46E-09
ENSG0000(	SYNGR3	5.894083	5.862151	1.37E-06
ENSG0000(	LYPD5	5.89322	8.870547	1.35E-09
ENSG0000(	AC087878.	5.883598	4.985863	1.03E-05
ENSG0000(	AC124301.	5.882495	5.046175	8.99E-06
ENSG0000(	DLGAP2	5.8786	7.391612	4.06E-08
ENSG0000(	LRTM1	5.874644	6.933641	1.17E-07
ENSG0000(	DUXAP9	5.871475	8.324362	4.74E-09

ENSG0000(	LINC00937	5.871279	5.90618	1.24E-06
ENSG0000(	CLEC9A	5.871155	6.74627	1.79E-07
ENSG0000(	SHISAL1	5.868141	7.006128	9.86E-08
ENSG0000(	PMCH	5.866669	8.492357	3.22E-09
ENSG0000(	MYMK	5.862373	10.66826	2.15E-11
ENSG0000(	IGHV1-3	5.861422	4.355338	4.41E-05
ENSG0000(	GBP6	5.859051	12.51602	3.05E-13
ENSG0000(	IGLV9-49	5.858431	4.215786	6.08E-05
ENSG0000(	IGLC3	5.853624	20.67247	2.13E-21
ENSG0000(	FCRL2	5.85109	2.417762	0.003822
ENSG0000(	IL5RA	5.84616	3.388591	0.000409
ENSG0000(	AC233309.	5.843592	8.401393	3.97E-09
ENSG0000(	CDKN2B-AS	5.842668	6.107419	7.81E-07
ENSG0000(	AP005205.	5.841902	4.15042	7.07E-05
ENSG0000(	KCNB2	5.841058	2.516225	0.003046
ENSG0000(	AC134312.	5.840281	4.113443	7.70E-05
ENSG0000(	IGKV1-17	5.837956	10.81845	1.52E-11
ENSG0000(	IGHV3-64	5.826409	3.505634	0.000312
ENSG0000(	TNFSF15	5.819914	4.045591	9.00E-05
ENSG0000(	AC004906.	5.818874	6.038296	9.16E-07
ENSG0000(	RAB39B	5.814327	5.791333	1.62E-06
ENSG0000(	ABCA4	5.812059	8.792491	1.61E-09
ENSG0000(	INSYN2	5.811427	5.060295	8.70E-06
ENSG0000(	TRIM67	5.810924	6.919131	1.20E-07
ENSG0000(	SYNDIG1	5.810769	4.059078	8.73E-05
ENSG0000(	IGLJ1	5.808689	15.27867	5.26E-16
ENSG0000(	FAM151B	5.807152	7.1956	6.37E-08
ENSG0000(	AL365475.1	5.805418	6.829815	1.48E-07
ENSG0000(	AC091152.	5.79988	4.240428	5.75E-05
ENSG0000(	HSD17B13	5.795041	8.061591	8.68E-09
ENSG0000(	IGKV6D-21	5.791134	1.672359	0.021264
ENSG0000(	AL158071.3	5.789984	5.702661	1.98E-06
ENSG0000(	KIRREL3	5.789451	5.716456	1.92E-06
ENSG0000(	AL645939.5	5.787333	4.857153	1.39E-05
ENSG0000(	ANXA8	5.785668	2.888457	0.001293
ENSG0000(	IGHV3-72	5.782398	6.881517	1.31E-07
ENSG0000(	HLA-DPB2	5.779319	2.026061	0.009418
ENSG0000(	HIST2H2BF	5.778258	5.950208	1.12E-06
ENSG0000(	RPGRIP1	5.769475	6.952236	1.12E-07
ENSG0000(	IGLC1	5.769439	17.5824	2.62E-18
ENSG0000(	AC124014.	5.769231	6.931392	1.17E-07
ENSG0000(	AL137856.1	5.768557	4.780912	1.66E-05
ENSG0000(	AC010761.	5.766812	8.495056	3.20E-09
ENSG0000(	AC090192.	5.758503	4.911414	1.23E-05
ENSG0000(	RF01875	5.755922	4.769729	1.70E-05
ENSG0000(	IGLL5	5.746256	18.46289	3.44E-19
ENSG0000(	AC062004.	5.740385	3.990659	0.000102

ENSG0000(	CNTN3	5.729772	4.015202	9.66E-05
ENSG0000(	IGHV4-4	5.729537	6.654599	2.22E-07
ENSG0000(	CCL22	5.725236	6.934722	1.16E-07
ENSG0000(	IGHV3-33	5.723314	14.21875	6.04E-15
ENSG0000(	IGLJ7	5.720764	2.41415	0.003853
ENSG0000(	LINC01094	5.717327	5.836549	1.46E-06
ENSG0000(	ARRDC3-AS	5.715881	5.86463	1.37E-06
ENSG0000(	BX255923.	5.715847	4.042901	9.06E-05
ENSG0000(	AC099548.	5.713252	4.903944	1.25E-05
ENSG0000(	RUFY4	5.711876	5.739784	1.82E-06
ENSG0000(	SAA4	5.707709	3.375044	0.000422
ENSG0000(	MYCNOS	5.700651	8.602258	2.50E-09
ENSG0000(	TRIM60P18	5.699086	10.41799	3.82E-11
ENSG0000(	MUC12	5.686373	5.685307	2.06E-06
ENSG0000(	IGLV3-1	5.677218	13.85652	1.39E-14
ENSG0000(	DNM1P46	5.672426	4.852615	1.40E-05
ENSG0000(	ABCA13	5.670416	2.83935	0.001448
ENSG0000(	PHF24	5.67027	5.727463	1.87E-06
ENSG0000(	ZNF365	5.662098	8.275809	5.30E-09
ENSG0000(	IYD	5.660471	2.368171	0.004284
ENSG0000(	TRBJ2-4	5.647997	4.654249	2.22E-05
ENSG0000(	IGLJ2	5.636939	20.8925	1.28E-21
ENSG0000(	MIR147B	5.636496	3.353496	0.000443
ENSG0000(	STARD13-A	5.63578	4.833391	1.47E-05
ENSG0000(	FCGR1A	5.630939	7.603561	2.49E-08
ENSG0000(	AC127521.	5.627648	5.566171	2.72E-06
ENSG0000(	CXCR3	5.627134	14.24352	5.71E-15
ENSG0000(	IGKV1D-12	5.622894	3.888776	0.000129
ENSG0000(	AC002059.	5.620303	8.026352	9.41E-09
ENSG0000(	IGHV3-30	5.611963	10.6613	2.18E-11
ENSG0000(	TIGD4	5.611927	3.317624	0.000481
ENSG0000(	IGLV1-40	5.60619	16.91342	1.22E-17
ENSG0000(	HCG4	5.605441	5.639685	2.29E-06
ENSG0000(	AL031985.:	5.598574	5.604936	2.48E-06
ENSG0000(	WFDC3	5.594537	3.94231	0.000114
ENSG0000(	AC067945.	5.59442	4.620723	2.39E-05
ENSG0000(	MIR8085	5.591023	8.455223	3.51E-09
ENSG0000(	AL352979.:	5.579501	5.651593	2.23E-06
ENSG0000(	TRBV5-1	5.577412	3.95271	0.000112
ENSG0000(	IGLV7-46	5.573862	6.036741	9.19E-07
ENSG0000(	CD69	5.573654	6.920657	1.20E-07
ENSG0000(	IGHV3-53	5.572087	10.30901	4.91E-11
ENSG0000(	AC245100.	5.568162	6.830554	1.48E-07
ENSG0000(	C1orf220	5.564932	6.529835	2.95E-07
ENSG0000(	AC083837.	5.554264	4.639035	2.30E-05
ENSG0000(	HNRNPA1P	5.553694	3.976426	0.000106
ENSG0000(	IGHG1	5.550774	21.51975	3.02E-22

ENSG0000(	CCL3L1	5.546481	3.264027	0.000544
ENSG0000(	IGLV3-27	5.540881	1.914821	0.012167
ENSG0000(	AC009133.	5.52855	3.828559	0.000148
ENSG0000(	PTP4A2P2	5.520193	3.240634	0.000575
ENSG0000(	INSM1	5.519775	3.262188	0.000547
ENSG0000(	TLR10	5.51933	4.477471	3.33E-05
ENSG0000(	IGKC	5.518775	19.2403	5.75E-20
ENSG0000(	ATP13A4	5.516332	3.661148	0.000218
ENSG0000(	RFPL1S	5.511295	3.89509	0.000127
ENSG0000(	TMPRSS13	5.51066	4.645294	2.26E-05
ENSG0000(	TRBJ1-6	5.506888	3.8159	0.000153
ENSG0000(	F2RL2	5.506557	4.556324	2.78E-05
ENSG0000(	LINC02084	5.504725	3.774831	0.000168
ENSG0000(	BDKRB1	5.50468	2.753472	0.001764
ENSG0000(	AC099063.	5.501005	7.620357	2.40E-08
ENSG0000(	RND1	5.500966	6.5759	2.66E-07
ENSG0000(	IGKV1D-13	5.498066	1.558169	0.027659
ENSG0000(	IGHV3-74	5.497503	9.151865	7.05E-10
ENSG0000(	FLJ22447	5.497324	3.228012	0.000592
ENSG0000(	SUCNR1	5.488397	8.974326	1.06E-09
ENSG0000(	TRAV29DV	5.487784	2.71722	0.001918
ENSG0000(	IGLC6	5.485102	2.220993	0.006012
ENSG0000(	DNAH9	5.48416	4.475075	3.35E-05
ENSG0000(	IL12B	5.474158	3.201197	0.000629
ENSG0000(	AC005722.	5.469525	1.854148	0.013991
ENSG0000(	AC025580.	5.468582	2.689702	0.002043
ENSG0000(	CCL18	5.449753	18.35628	4.40E-19
ENSG0000(	CD1E	5.445903	7.45421	3.51E-08
ENSG0000(	RPL32P1	5.442415	3.748205	0.000179
ENSG0000(	ETV7	5.441368	9.317267	4.82E-10
ENSG0000(	AL390755.:	5.439518	6.839376	1.45E-07
ENSG0000(	AC087501.	5.430651	5.213152	6.12E-06
ENSG0000(	AC244205.	5.429498	19.54876	2.83E-20
ENSG0000(	IGKJ2	5.427736	19.53247	2.93E-20
ENSG0000(	IGKJ5	5.426917	14.24872	5.64E-15
ENSG0000(	LINC01585	5.426142	4.529363	2.96E-05
ENSG0000(	TEX41	5.422398	5.564487	2.73E-06
ENSG0000(	MYH16	5.416185	3.132848	0.000736
ENSG0000(	AC008033.	5.414463	3.145474	0.000715
ENSG0000(	AC090136.	5.41168	3.259651	0.00055
ENSG0000(	FATE1	5.411418	3.76416	0.000172
ENSG0000(	SMPDL3B	5.41004	2.552646	0.002801
ENSG0000(	RGR	5.409788	3.186079	0.000652
ENSG0000(	AC136424.	5.4027	6.467672	3.41E-07
ENSG0000(	IGHJ6	5.399659	17.72791	1.87E-18
ENSG0000(	AC010655.	5.396664	2.549296	0.002823
ENSG0000(	CXorf65	5.395886	3.14534	0.000716

ENSG0000(	LINC00943	5.394914	4.489236	3.24E-05
ENSG0000(	FAM110C	5.390474	3.746727	0.000179
ENSG0000(	IGLC2	5.38969	22.45515	3.51E-23
ENSG0000(	AL109761.:	5.388782	4.531332	2.94E-05
ENSG0000(	PKHD1	5.383796	4.418801	3.81E-05
ENSG0000(	AC023908.	5.381451	6.428949	3.72E-07
ENSG0000(	ADM2	5.381104	4.576143	2.65E-05
ENSG0000(	SLITRK2	5.379052	3.099362	0.000795
ENSG0000(	WARS2-IT1	5.376257	3.674648	0.000212
ENSG0000(	LINC00942	5.37544	2.165949	0.006824
ENSG0000(	IGHV4-39	5.36808	9.710876	1.95E-10
ENSG0000(	AL121944.:	5.367805	5.321443	4.77E-06
ENSG0000(	ANAPC1P1	5.365121	3.629785	0.000235
ENSG0000(	AC040970.	5.357487	4.460368	3.46E-05
ENSG0000(	AK8	5.356013	5.380353	4.17E-06
ENSG0000(	MMP23B	5.351592	4.386945	4.10E-05
ENSG0000(	AL157394.:	5.349926	5.256547	5.54E-06
ENSG0000(	AC116025.	5.339152	4.391406	4.06E-05
ENSG0000(	IGKV1-16	5.329771	8.329296	4.68E-09
ENSG0000(	FAM178B	5.321195	3.632066	0.000233
ENSG0000(	TRBV29-1	5.320515	3.671047	0.000213
ENSG0000(	IL12A	5.318604	4.320054	4.79E-05
ENSG0000(	AF127936.	5.316506	3.067346	0.000856
ENSG0000(	AC131097.	5.316405	3.730513	0.000186
ENSG0000(	KYNU	5.316281	9.80726	1.56E-10
ENSG0000(	HS3ST2	5.314579	2.571981	0.002679
ENSG0000(	AC015911.	5.311463	8.491079	3.23E-09
ENSG0000(	IL22RA1	5.308255	3.67103	0.000213
ENSG0000(	C1QL4	5.304885	1.48063	0.033065
ENSG0000(	AC016957.	5.30226	4.267215	5.40E-05
ENSG0000(	ENO1P1	5.299377	5.302411	4.98E-06
ENSG0000(	IGLV1-44	5.294428	12.9525	1.12E-13
ENSG0000(	POU2AF1	5.290884	9.501602	3.15E-10
ENSG0000(	RTP1	5.288473	1.79896	0.015887
ENSG0000(	TCTEX1D4	5.285477	3.727422	0.000187
ENSG0000(	GPR88	5.283452	3.131904	0.000738
ENSG0000(	MAFG-DT	5.280605	6.352703	4.44E-07
ENSG0000(	AL390719.:	5.277322	3.596808	0.000253
ENSG0000(	AC011455.	5.275517	3.708836	0.000196
ENSG0000(	NALT1	5.272304	3.590039	0.000257
ENSG0000(	FCGR1CP	5.266517	4.310994	4.89E-05
ENSG0000(	AC005077.	5.262215	3.100111	0.000794
ENSG0000(	ZNF257	5.260449	3.093701	0.000806
ENSG0000(	NRIP3	5.258327	5.174192	6.70E-06
ENSG0000(	IGKV3D-7	5.252336	1.742603	0.018088
ENSG0000(	AC026356.	5.249467	4.315429	4.84E-05
ENSG0000(	IGHV3-23	5.243282	19.73173	1.85E-20

ENSG0000(	IGKJ1	5.240823	18.39536	4.02E-19
ENSG0000(	FAAH2	5.23839	4.979053	1.05E-05
ENSG0000(	OR2A20P	5.233258	2.537385	0.002901
ENSG0000(	IGKV1D-8	5.232027	1.71271	0.019377
ENSG0000(	CFHR1	5.224611	3.663613	0.000217
ENSG0000(	IGKV1-13	5.223905	6.000011	1.00E-06
ENSG0000(	IGKV3-15	5.221637	14.65468	2.21E-15
ENSG0000(	CYP19A1	5.218742	4.390062	4.07E-05
ENSG0000(	AC090152.	5.213151	4.331586	4.66E-05
ENSG0000(	AL033527.!	5.212639	4.278409	5.27E-05
ENSG0000(	AC124301.	5.212003	3.055746	0.00088
ENSG0000(	GDF6	5.210184	2.994767	0.001012
ENSG0000(	IGHD	5.209083	3.008193	0.000981
ENSG0000(	AIRE	5.209041	2.478213	0.003325
ENSG0000(	TRGC1	5.207584	2.977702	0.001053
ENSG0000(	IGLJ6	5.20709	1.755019	0.017578
ENSG0000(	TONSL-AS1	5.204366	5.180194	6.60E-06
ENSG0000(	TNN	5.202746	2.414563	0.00385
ENSG0000(	PABPC1P4	5.20092	3.603546	0.000249
ENSG0000(	MIR2277	5.199784	5.15955	6.93E-06
ENSG0000(	OMG	5.196956	6.287772	5.15E-07
ENSG0000(	AC130371.	5.18136	2.987629	0.001029
ENSG0000(	AC005865.	5.180538	3.049976	0.000891
ENSG0000(	AC243964.	5.17711	2.496885	0.003185
ENSG0000(	NTN5	5.17682	4.985266	1.03E-05
ENSG0000(	IGKJ4	5.170311	19.88052	1.32E-20
ENSG0000(	AC026803.	5.169582	3.586358	0.000259
ENSG0000(	GALNT13	5.168422	2.923703	0.001192
ENSG0000(	LINC00891	5.167541	2.96589	0.001082
ENSG0000(	CD80	5.164277	2.468673	0.003399
ENSG0000(	CACNA1F	5.161828	4.135618	7.32E-05
ENSG0000(	TNIP3	5.155319	2.980752	0.001045
ENSG0000(	LINC01480	5.153652	2.916596	0.001212
ENSG0000(	AC011416.	5.151964	4.311712	4.88E-05
ENSG0000(	AL365361.:	5.147842	11.01143	9.74E-12
ENSG0000(	IGKV2-28	5.147598	20.75847	1.74E-21
ENSG0000(	AC127070.	5.142181	4.138038	7.28E-05
ENSG0000(	AC006033.	5.138711	2.930854	0.001173
ENSG0000(	HLA-W	5.136161	4.183638	6.55E-05
ENSG0000(	ABHD15-AS	5.135206	3.456854	0.000349
ENSG0000(	AP001351.	5.1346	2.977702	0.001053
ENSG0000(	RN7SL138F	5.134483	2.973122	0.001064
ENSG0000(	AL139352.:	5.134191	3.517794	0.000304
ENSG0000(	AC022509.	5.132525	3.528614	0.000296
ENSG0000(	AC034238.	5.128812	7.892076	1.28E-08
ENSG0000(	PHEX	5.117056	3.509401	0.000309
ENSG0000(	RPL23AP32	5.115177	3.412773	0.000387

ENSG0000(	AC243829.	5.114174	2.441421	0.003619
ENSG0000(	AC145422.	5.112991	4.259974	5.50E-05
ENSG0000(	SHANK2	5.109445	4.157721	6.95E-05
ENSG0000(	AL160270.:	5.109008	2.406729	0.00392
ENSG0000(	AC012313.	5.108352	2.976252	0.001056
ENSG0000(	CDCA2	5.105337	5.041828	9.08E-06
ENSG0000(	LINC01267	5.104472	2.065708	0.008596
ENSG0000(	RF02166	5.101804	2.966138	0.001081
ENSG0000(	BCL2L15	5.099365	3.535861	0.000291
ENSG0000(	CXCL10	5.098383	17.00807	9.82E-18
ENSG0000(	ADORA3	5.094204	9.309612	4.90E-10
ENSG0000(	FAM187A	5.091729	5.195425	6.38E-06
ENSG0000(	SLC44A3-A	5.0915	4.16561	6.83E-05
ENSG0000(	AC100782.	5.091093	2.039913	0.009122
ENSG0000(	IGKV1-9	5.090683	9.971324	1.07E-10
ENSG0000(	CDKN2A	5.086492	15.33998	4.57E-16
ENSG0000(	AL031651.:	5.085005	4.212611	6.13E-05
ENSG0000(	IGKV1-37	5.084522	1.643531	0.022723
ENSG0000(	AL512422.:	5.084244	3.456639	0.000349
ENSG0000(	IGKV1-12	5.081294	12.73991	1.82E-13
ENSG0000(	MAP3K21	5.080746	4.203543	6.26E-05
ENSG0000(	AL031846.:	5.079766	3.400255	0.000398
ENSG0000(	CCL26	5.078821	2.368431	0.004281
ENSG0000(	MIR4649	5.07877	2.403076	0.003953
ENSG0000(	FCRLB	5.075944	2.925346	0.001188
ENSG0000(	DBX2	5.074103	2.425083	0.003758
ENSG0000(	CFAP300	5.071549	3.463888	0.000344
ENSG0000(	TRBV2	5.071403	1.996022	0.010092
ENSG0000(	CXCL9	5.06783	24.67638	2.11E-25
ENSG0000(	BX322639.	5.067142	4.225275	5.95E-05
ENSG0000(	IGKV3-11	5.063244	14.77352	1.68E-15
ENSG0000(	RPL10P19	5.058012	3.409143	0.00039
ENSG0000(	CNTD2	5.056481	2.380893	0.00416
ENSG0000(	AL139349.:	5.05369	3.454512	0.000351
ENSG0000(	IGKJ3	5.051888	10.36329	4.33E-11
ENSG0000(	IGHJ3	5.051008	13.72236	1.90E-14
ENSG0000(	AL359183.:	5.050067	2.39637	0.004014
ENSG0000(	AC021678.	5.048696	1.34755	0.044921
ENSG0000(	RTP5	5.048212	2.376598	0.004201
ENSG0000(	DIAPH3	5.047773	3.459414	0.000347
ENSG0000(	FAM225B	5.047693	4.169797	6.76E-05
ENSG0000(	TRAV13-2	5.047511	2.404119	0.003943
ENSG0000(	AC009185.	5.047459	3.41563	0.000384
ENSG0000(	KLRC4	5.047103	7.196996	6.35E-08
ENSG0000(	HIGD1AP1:	5.045376	3.488154	0.000325
ENSG0000(	TBC1D27P	5.043734	2.365	0.004315
ENSG0000(	TTK	5.041762	4.11706	7.64E-05

ENSG0000(	FANK1	5.040211	4.099734	7.95E-05
ENSG0000(	AC131254.	5.040047	1.664748	0.02164
ENSG0000(	AC008074.	5.039424	2.410364	0.003887
ENSG0000(	NKX6-1	5.039001	1.97181	0.010671
ENSG0000(	PRR29-AS1	5.0367	2.926781	0.001184
ENSG0000(	SUMO2P17	5.032811	3.500567	0.000316
ENSG0000(	LAG3	5.030291	7.644348	2.27E-08
ENSG0000(	LINC00996	5.028325	2.852061	0.001406
ENSG0000(	PPP4R4	5.02461	1.661884	0.021783
ENSG0000(	TRDV1	5.024417	1.642781	0.022762
ENSG0000(	AC011330.	5.022357	4.016422	9.63E-05
ENSG0000(	LINC00484	5.019022	2.881785	0.001313
ENSG0000(	TOMM20P	5.011535	2.833157	0.001468
ENSG0000(	TRAV12-2	5.01119	2.801749	0.001579
ENSG0000(	AC007681.	5.010567	3.351084	0.000446
ENSG0000(	AP001347.	5.00464	2.806426	0.001562
ENSG0000(	IGHV4-34	5.004333	6.379729	4.17E-07
ENSG0000(	LINC00539	5.003802	2.857802	0.001387
ENSG0000(	TRBJ1-3	5.003722	2.801731	0.001579
ENSG0000(	AC087623.	5.002648	4.057588	8.76E-05
ENSG0000(	LINC01619	5.001413	1.990322	0.010225
ENSG0000(	LINC01117	4.9997	2.868807	0.001353
ENSG0000(	AC004847.	4.998627	2.349751	0.004469
ENSG0000(	PDIA2	4.997428	4.003844	9.91E-05
ENSG0000(	CD79A	4.996486	5.328703	4.69E-06
ENSG0000(	FSD1	4.994325	2.329345	0.004684
ENSG0000(	AC006518.	4.993542	3.441604	0.000362
ENSG0000(	TRAV12-3	4.992561	1.968091	0.010762
ENSG0000(	TRBV9	4.990693	1.33763	0.045959
ENSG0000(	FANCD2OS	4.990028	4.104759	7.86E-05
ENSG0000(	ZBTB32	4.98821	3.346027	0.000451
ENSG0000(	IGKV4-1	4.986943	22.60073	2.51E-23
ENSG0000(	AL596094.1	4.984553	4.047985	8.95E-05
ENSG0000(	FTH1P22	4.983568	2.338431	0.004587
ENSG0000(	CLECL1	4.979655	1.936425	0.011576
ENSG0000(	IGFL2-AS1	4.976496	1.964401	0.010854
ENSG0000(	U62317.1	4.974186	2.366146	0.004304
ENSG0000(	CD8B	4.971022	8.309371	4.90E-09
ENSG0000(	AC074131.	4.969279	2.786161	0.001636
ENSG0000(	AC090825.	4.968299	4.092043	8.09E-05
ENSG0000(	LINC00528	4.966601	2.786583	0.001635
ENSG0000(	AC139749.	4.96507	2.75118	0.001773
ENSG0000(	SLC24A2	4.962567	4.823879	1.50E-05
ENSG0000(	AP001021.	4.960661	2.858022	0.001387
ENSG0000(	NIPAL4	4.959215	1.622631	0.023843
ENSG0000(	IGLV1-51	4.956819	12.33078	4.67E-13
ENSG0000(	AL049775.1	4.955441	2.337457	0.004598

ENSG0000(	AC245060.	4.954266	3.367933	0.000429
ENSG0000(	AP003472.	4.952894	2.370802	0.004258
ENSG0000(	HEATR9	4.951836	2.779275	0.001662
ENSG0000(	MIR155HG	4.950438	7.152847	7.03E-08
ENSG0000(	SFTA1P	4.946883	2.839169	0.001448
ENSG0000(	BHLHA15	4.946682	1.946285	0.011317
ENSG0000(	AC010201.	4.946631	2.797764	0.001593
ENSG0000(	IGLV2-34	4.941489	1.600607	0.025084
ENSG0000(	IL21R	4.93999	8.704828	1.97E-09
ENSG0000(	OR2I1P	4.939853	16.6626	2.17E-17
ENSG0000(	IGHJ5	4.93765	13.61443	2.43E-14
ENSG0000(	TRBV6-5	4.9359	1.936838	0.011565
ENSG0000(	CRYBB3	4.928379	2.357001	0.004395
ENSG0000(	CCDC74B	4.928045	2.343219	0.004537
ENSG0000(	KLRC1	4.925398	1.905422	0.012433
ENSG0000(	AC002472.	4.92113	3.319296	0.000479
ENSG0000(	AC016683.	4.920667	2.332028	0.004656
ENSG0000(	EHHADH-A	4.910384	3.357633	0.000439
ENSG0000(	AL031118.:	4.907258	3.316352	0.000483
ENSG0000(	SH2D6	4.906249	2.796227	0.001599
ENSG0000(	AC068775.	4.905145	2.275911	0.005298
ENSG0000(	TTC24	4.904148	10.90529	1.24E-11
ENSG0000(	4-Mar	4.904018	1.883071	0.01309
ENSG0000(	PIFO	4.901936	1.889712	0.012891
ENSG0000(	VWC2	4.901425	2.291105	0.005116
ENSG0000(	SLAMF6	4.900241	7.334756	4.63E-08
ENSG0000(	NECAB2	4.899644	1.57982	0.026314
ENSG0000(	AC245100.	4.897793	2.344177	0.004527
ENSG0000(	COLEC11	4.895103	2.309033	0.004909
ENSG0000(	RF00285	4.894266	2.789244	0.001625
ENSG0000(	ENO4	4.894103	2.274013	0.005321
ENSG0000(	CD19	4.893759	2.316533	0.004825
ENSG0000(	AC009630.	4.892634	2.803532	0.001572
ENSG0000(	KRT5	4.891903	1.560493	0.027511
ENSG0000(	MATN4	4.891523	1.932598	0.011679
ENSG0000(	CPA5	4.886837	2.302072	0.004988
ENSG0000(	SUDS3P1	4.884702	2.756169	0.001753
ENSG0000(	IGKV2D-29	4.884481	1.303445	0.049723
ENSG0000(	AC067817.	4.883095	2.758515	0.001744
ENSG0000(	EXO1	4.876883	3.29873	0.000503
ENSG0000(	AC018761.	4.876401	3.310153	0.00049
ENSG0000(	TRAV13-1	4.876194	1.853502	0.014012
ENSG0000(	THEMIS	4.86884	6.122824	7.54E-07
ENSG0000(	CCL13	4.868832	16.66715	2.15E-17
ENSG0000(	GZMK	4.865577	7.817809	1.52E-08
ENSG0000(	TRG-AS1	4.863663	6.051222	8.89E-07
ENSG0000(	SGO1	4.862745	3.282162	0.000522

ENSG0000(	ADGRG5	4.861041	7.921688	1.20E-08
ENSG0000(	HAS1	4.857404	2.274152	0.005319
ENSG0000(	AC079949.	4.853374	2.745809	0.001796
ENSG0000(	AL512408.:	4.852739	3.916445	0.000121
ENSG0000(	AC008533.	4.847702	2.231451	0.005869
ENSG0000(	PNMA3	4.844468	6.128318	7.44E-07
ENSG0000(	AL133215.:	4.837786	2.762672	0.001727
ENSG0000(	SLC12A5	4.835066	1.863514	0.013693
ENSG0000(	CAMSAP3	4.829784	2.268902	0.005384
ENSG0000(	RAB33A	4.829184	1.884053	0.01306
ENSG0000(	AL031847.:	4.827711	2.72592	0.00188
ENSG0000(	MAS1L	4.824307	2.279128	0.005259
ENSG0000(	IGLV2-14	4.82405	14.59351	2.55E-15
ENSG0000(	TTC34	4.823889	2.27077	0.005361
ENSG0000(	AC239859.	4.820865	1.893196	0.012788
ENSG0000(	CD70	4.818615	4.511376	3.08E-05
ENSG0000(	AL445231.:	4.816963	2.654702	0.002215
ENSG0000(	IGHJ4	4.816918	24.90178	1.25E-25
ENSG0000(	IGKV1-5	4.816687	16.09473	8.04E-17
ENSG0000(	CLDN10	4.812699	1.539285	0.028888
ENSG0000(	UBD	4.811967	13.93091	1.17E-14
ENSG0000(	CEND1	4.810562	2.69252	0.00203
ENSG0000(	ARRDC5	4.808537	1.540136	0.028831
ENSG0000(	MIR6872	4.802808	3.211864	0.000614
ENSG0000(	DDX25	4.802526	1.874198	0.01336
ENSG0000(	AP000523.	4.801336	2.238905	0.005769
ENSG0000(	BNC1	4.798085	2.234951	0.005822
ENSG0000(	F7	4.790744	2.718543	0.001912
ENSG0000(	CALML3-AS	4.789399	1.84499	0.014289
ENSG0000(	FAM83D	4.788171	2.693562	0.002025
ENSG0000(	U62317.4	4.783222	2.234191	0.005832
ENSG0000(	MIR142	4.781305	4.724877	1.88E-05
ENSG0000(	LINC02154	4.779109	2.203385	0.006261
ENSG0000(	AL139158.:	4.775032	1.543264	0.028624
ENSG0000(	AC092143.	4.771998	4.060929	8.69E-05
ENSG0000(	GPR158	4.771063	2.206921	0.00621
ENSG0000(	AL445472.:	4.764688	2.625867	0.002367
ENSG0000(	AL133371.:	4.763628	2.670997	0.002133
ENSG0000(	GFI1	4.763264	5.482582	3.29E-06
ENSG0000(	IGHA1	4.762861	20.67247	2.13E-21
ENSG0000(	TRDC	4.761994	6.970225	1.07E-07
ENSG0000(	IGKV2-30	4.761779	10.59826	2.52E-11
ENSG0000(	BEND3P1	4.758502	2.648066	0.002249
ENSG0000(	MIR650	4.754315	8.128868	7.43E-09
ENSG0000(	AL499627.:	4.751745	13.71351	1.93E-14
ENSG0000(	AC018638.	4.749741	2.199772	0.006313
ENSG0000(	MIR9-3HG	4.748594	2.185401	0.006525

ENSG0000(	KCNIP1	4.744601	1.807368	0.015582
ENSG0000(	AL158198.:	4.741272	3.1366	0.00073
ENSG0000(	GP6	4.741168	2.643364	0.002273
ENSG0000(	RNA5SP33:	4.739833	1.82468	0.014973
ENSG0000(	REP15	4.738842	2.182856	0.006564
ENSG0000(	BX571818.	4.737031	2.182233	0.006573
ENSG0000(	ZC3H12D	4.730321	8.799672	1.59E-09
ENSG0000(	BATF	4.72907	6.489772	3.24E-07
ENSG0000(	GALNT14	4.728257	8.298751	5.03E-09
ENSG0000(	AC011445.	4.723526	2.647556	0.002251
ENSG0000(	AC002525.	4.722955	1.833854	0.01466
ENSG0000(	PLEKHD1	4.720535	3.829896	0.000148
ENSG0000(	AC104009.	4.72023	1.795386	0.016018
ENSG0000(	TRBV6-2	4.717044	1.49027	0.032339
ENSG0000(	IGKV2D-30	4.707321	1.492372	0.032183
ENSG0000(	MIR4775	4.705789	2.1683	0.006787
ENSG0000(	CCR5	4.702519	17.0213	9.52E-18
ENSG0000(	AC119428.	4.702287	1.811926	0.01542
ENSG0000(	AC090772.	4.700316	2.593777	0.002548
ENSG0000(	IPCEF1	4.696351	7.294682	5.07E-08
ENSG0000(	TRBV20-1	4.695439	4.650797	2.23E-05
ENSG0000(	PRSS30P	4.691265	1.480326	0.033088
ENSG0000(	AL606469.:	4.690697	1.812081	0.015414
ENSG0000(	TRBJ2-5	4.688196	4.852615	1.40E-05
ENSG0000(	LINC00877	4.686602	2.154314	0.007009
ENSG0000(	TRDJ1	4.679055	1.778844	0.01664
ENSG0000(	SLC12A3	4.678981	1.783412	0.016466
ENSG0000(	AC110048.	4.674315	1.490384	0.032331
ENSG0000(	SP140	4.67261	5.861217	1.38E-06
ENSG0000(	LINC02352	4.669618	2.148668	0.007101
ENSG0000(	KCNA3	4.669551	5.429489	3.72E-06
ENSG0000(	HTRA4	4.669316	1.476676	0.033367
ENSG0000(	AC110285.	4.667306	2.15993	0.006919
ENSG0000(	AL359198.:	4.659473	3.136454	0.00073
ENSG0000(	LINC01426	4.655701	1.779185	0.016627
ENSG0000(	SNX29P2	4.655246	2.164715	0.006844
ENSG0000(	TRAV2	4.654323	2.151654	0.007053
ENSG0000(	AC090825.	4.65414	2.167505	0.0068
ENSG0000(	AC009570.	4.652401	2.114142	0.007689
ENSG0000(	TRBV11-2	4.650746	1.443463	0.036019
ENSG0000(	HPSE2	4.650359	7.804421	1.57E-08
ENSG0000(	LINC01260	4.649648	1.774336	0.016814
ENSG0000(	AL391832.:	4.648256	2.169623	0.006767
ENSG0000(	AC139491.	4.645384	2.127385	0.007458
ENSG0000(	CCL17	4.644206	1.455822	0.035009
ENSG0000(	MARK2P16	4.643886	1.774745	0.016798
ENSG0000(	LINC00920	4.64127	1.453305	0.035212

ENSG0000(	SLC6A17	4.640878	1.776813	0.016718
ENSG0000(	CNTN4-AS1	4.63792	1.461157	0.034581
ENSG0000(	SAMD3	4.636337	7.658994	2.19E-08
ENSG0000(	AL139246.!	4.63404	1.775002	0.016788
ENSG0000(	MIR770	4.633381	1.765208	0.017171
ENSG0000(	AC110995.	4.631424	5.276629	5.29E-06
ENSG0000(	AC087284.	4.630414	1.440747	0.036245
ENSG0000(	ASGR2	4.629771	2.107124	0.007814
ENSG0000(	AC068724.	4.628777	2.141702	0.007216
ENSG0000(	GPR55	4.627927	2.108928	0.007782
ENSG0000(	AL034417.:	4.618988	1.471149	0.033795
ENSG0000(	AC008429.	4.618372	1.458408	0.034801
ENSG0000(	XKR9	4.618014	2.120946	0.007569
ENSG0000(	RIMS2	4.601518	2.10315	0.007886
ENSG0000(	MESTIT1	4.600447	1.75707	0.017496
ENSG0000(	AL023653.:	4.599818	6.4606	3.46E-07
ENSG0000(	CHD5	4.598807	2.116692	0.007644
ENSG0000(	KRT81	4.598366	2.1095	0.007771
ENSG0000(	TMC1	4.590113	2.08892	0.008149
ENSG0000(	TRBV3-1	4.582434	1.445578	0.035844
ENSG0000(	AC002128.	4.578747	2.0892	0.008143
ENSG0000(	ITFG2-AS1	4.573751	2.086001	0.008203
ENSG0000(	TESMIN	4.572155	2.097674	0.007986
ENSG0000(	AC009093.	4.570909	1.751141	0.017736
ENSG0000(	TRBV12-4	4.56687	1.420452	0.037979
ENSG0000(	IGKV1-39	4.562532	19.89954	1.26E-20
ENSG0000(	KRT4	4.561352	1.722325	0.018953
ENSG0000(	AC005220.	4.560473	1.443683	0.036001
ENSG0000(	AC003101.	4.555737	1.734637	0.018423
ENSG0000(	NEURL3	4.552064	1.736619	0.018339
ENSG0000(	RF01876	4.55183	1.720858	0.019017
ENSG0000(	AL021707.:	4.550327	1.694081	0.020226
ENSG0000(	AC008467.	4.54996	2.057961	0.008751
ENSG0000(	IGHA2	4.547741	8.366227	4.30E-09
ENSG0000(	AC073367.	4.547349	2.100791	0.007929
ENSG0000(	DKFZP434I	4.544275	1.717826	0.01915
ENSG0000(	NEUROG3	4.540469	1.416591	0.038319
ENSG0000(	AC013652.	4.539743	1.708656	0.019559
ENSG0000(	AC087482.	4.538869	1.411336	0.038785
ENSG0000(	AL590666.:	4.53163	1.724272	0.018868
ENSG0000(	TM6SF2	4.530214	2.078387	0.008349
ENSG0000(	CLCA2	4.528796	1.402176	0.039612
ENSG0000(	CLDN2	4.52793	2.084932	0.008224
ENSG0000(	AC009095.	4.526784	2.039913	0.009122
ENSG0000(	AL161725.:	4.526191	2.037997	0.009162
ENSG0000(	AC233300.	4.525556	1.717305	0.019173
ENSG0000(	WDR93	4.516441	1.382588	0.041439

ENSG0000(	GPR152	4.514605	1.725904	0.018797
ENSG0000(	AC119044.	4.507324	1.702649	0.019831
ENSG0000(	AC012236.	4.506577	1.690045	0.020415
ENSG0000(	AC116353.	4.504107	1.68784	0.020519
ENSG0000(	CA7	4.502565	1.387696	0.040955
ENSG0000(	ARHGEF19-	4.502363	1.713208	0.019355
ENSG0000(	C2CD4A	4.501361	1.408386	0.039049
ENSG0000(	AL512306.:	4.501254	1.693533	0.020252
ENSG0000(	RAB39A	4.500767	1.710094	0.019494
ENSG0000(	RF00019	4.495908	1.404146	0.039432
ENSG0000(	IGLV2-8	4.494844	8.949097	1.12E-09
ENSG0000(	LINC01624	4.493633	2.068715	0.008537
ENSG0000(	FTLP3	4.49078	2.032651	0.009276
ENSG0000(	SLC27A2	4.490587	1.395388	0.040236
ENSG0000(	TRAV17	4.490552	1.686813	0.020568
ENSG0000(	KLRB1	4.490183	4.790839	1.62E-05
ENSG0000(	LAX1	4.489867	7.190731	6.45E-08
ENSG0000(	AC116407.	4.487762	1.658642	0.021946
ENSG0000(	SNORD36C	4.486977	2.049823	0.008916
ENSG0000(	AL365295.:	4.485042	2.066452	0.008581
ENSG0000(	RF01874	4.477751	1.675338	0.021118
ENSG0000(	ALOX15	4.475486	2.216566	0.006073
ENSG0000(	RF02164	4.475373	1.702623	0.019832
ENSG0000(	AC009955.	4.473643	1.699244	0.019987
ENSG0000(	RN7SKP17!	4.473145	1.384186	0.041287
ENSG0000(	AC067930.	4.473145	1.384186	0.041287
ENSG0000(	NUDT10	4.467342	1.671488	0.021307
ENSG0000(	GRHL2	4.467061	1.389521	0.040783
ENSG0000(	AC106886.	4.466125	1.678519	0.020964
ENSG0000(	UNC80	4.464522	1.641821	0.022813
ENSG0000(	AC119428.	4.46413	1.664987	0.021628
ENSG0000(	HAR1A	4.462059	1.678741	0.020954
ENSG0000(	SAA2-SAA4	4.459929	5.439384	3.64E-06
ENSG0000(	MIR6843	4.457973	1.673397	0.021213
ENSG0000(	AC010615.	4.457745	1.665261	0.021614
ENSG0000(	AC239798.	4.456798	1.373626	0.042303
ENSG0000(	SHLD2P1	4.450608	1.366987	0.042955
ENSG0000(	ERICD	4.450462	2.044489	0.009026
ENSG0000(	AL137784.:	4.444197	2.003416	0.009922
ENSG0000(	LINC01474	4.443614	1.643544	0.022723
ENSG0000(	AC024145.	4.443412	1.65413	0.022175
ENSG0000(	AC011495.	4.442685	1.658829	0.021937
ENSG0000(	TMEM179	4.442289	1.641043	0.022854
ENSG0000(	EEF1A1P3	4.436946	1.993744	0.010145
ENSG0000(	AC011447.	4.436117	1.648493	0.022465
ENSG0000(	AC105940.	4.435557	2.012851	0.009708
ENSG0000(	CYP8B1	4.431465	1.366699	0.042983

ENSG0000(	CD3G	4.429101	6.412926	3.86E-07
ENSG0000(	RNF175	4.428303	1.359416	0.04371
ENSG0000(	AC093890.	4.427773	1.369347	0.042722
ENSG0000(	AC002074.	4.425898	1.360149	0.043637
ENSG0000(	RN7SKP17(	4.425602	1.363874	0.043264
ENSG0000(	LINC02044	4.423201	1.967731	0.010771
ENSG0000(	AC005722.	4.421889	1.367167	0.042937
ENSG0000(	RHBDL3	4.420055	1.355343	0.044122
ENSG0000(	BRINP1	4.419686	5.018138	9.59E-06
ENSG0000(	AC009093.	4.419528	1.359955	0.043656
ENSG0000(	IRX1	4.407854	1.639458	0.022937
ENSG0000(	SLC9A9-AS:	4.405704	9.213736	6.11E-10
ENSG0000(	OLR1	4.401502	1.350489	0.044618
ENSG0000(	AL596330.:	4.401279	1.65623	0.022068
ENSG0000(	DYNC1I2P1	4.39395	1.635998	0.023121
ENSG0000(	LTK	4.393147	9.71399	1.93E-10
ENSG0000(	TRAV4	4.392703	1.343593	0.045332
ENSG0000(	ASPDH	4.391777	1.346885	0.04499
ENSG0000(	MYCL	4.387357	9.774708	1.68E-10
ENSG0000(	MYLK-AS2	4.385045	1.639043	0.022959
ENSG0000(	RHEBP1	4.384071	1.341566	0.045544
ENSG0000(	ADAMTS7F	4.382437	1.345971	0.045085
ENSG0000(	IL21R-AS1	4.382024	6.535196	2.92E-07
ENSG0000(	SCRT1	4.378469	1.628019	0.023549
ENSG0000(	KCNG2	4.378311	1.616861	0.024162
ENSG0000(	TRBV5-4	4.369362	1.328317	0.046955
ENSG0000(	PIP5KL1	4.369171	1.628738	0.02351
ENSG0000(	IGHG2	4.356103	17.85583	1.39E-18
ENSG0000(	CD1A	4.353722	1.308129	0.049189
ENSG0000(	ZFPM2-AS1	4.352698	1.336666	0.046061
ENSG0000(	MEI1	4.352101	6.970225	1.07E-07
ENSG0000(	AC106872.	4.351074	1.337817	0.045939
ENSG0000(	AC027702.	4.350503	1.614615	0.024288
ENSG0000(	GPR84	4.33982	1.329101	0.04687
ENSG0000(	CCDC116	4.33891	1.310036	0.048974
ENSG0000(	GZMA	4.334016	9.357429	4.39E-10
ENSG0000(	CACNA1I	4.333511	1.623658	0.023787
ENSG0000(	SEPT7P9	4.329614	1.934787	0.01162
ENSG0000(	AC015712.	4.323984	1.590718	0.025662
ENSG0000(	AC018529.	4.323971	1.319821	0.047883
ENSG0000(	MOXD1	4.317833	6.050311	8.91E-07
ENSG0000(	AC073046.	4.31733	1.940986	0.011455
ENSG0000(	MIR6875	4.316945	1.602085	0.024999
ENSG0000(	GPRC5D-AS	4.315746	1.313913	0.048539
ENSG0000(	TTC16	4.315358	4.611471	2.45E-05
ENSG0000(	AC008429.	4.315009	1.324496	0.04737
ENSG0000(	AP000688.	4.310251	2.934178	0.001164

ENSG0000(	SNORD36B	4.310059	1.594077	0.025464
ENSG0000(	AC106786.	4.307078	1.314385	0.048486
ENSG0000(	CD2	4.304734	10.41513	3.84E-11
ENSG0000(	FTOP1	4.300795	1.323728	0.047454
ENSG0000(	LGR6	4.298891	8.260639	5.49E-09
ENSG0000(	PBX4	4.29848	6.691744	2.03E-07
ENSG0000(	AP003717.	4.296474	1.311176	0.048845
ENSG0000(	TRBV12-3	4.296214	1.585981	0.025943
ENSG0000(	LIPJ	4.29605	1.305164	0.049526
ENSG0000(	LINC01504	4.294217	6.189893	6.46E-07
ENSG0000(	TFAP2C	4.292074	1.3083	0.04917
ENSG0000(	ULBP3	4.289656	1.599068	0.025173
ENSG0000(	NPAS1	4.287646	1.595946	0.025354
ENSG0000(	SIT1	4.287572	8.161478	6.89E-09
ENSG0000(	NMRAL2P	4.286338	1.303581	0.049707
ENSG0000(	AC005776.	4.286182	1.572491	0.026761
ENSG0000(	FCRL6	4.285288	8.556902	2.77E-09
ENSG0000(	FOXI2	4.285159	2.93283	0.001167
ENSG0000(	AC009299.	4.281261	1.568532	0.027007
ENSG0000(	SCRG1	4.278288	1.300013	0.050117
ENSG0000(	AL831711.:	4.272518	1.571248	0.026838
ENSG0000(	ZNF80	4.263725	1.565132	0.027219
ENSG0000(	SGO1-AS1	4.256268	1.570933	0.026858
ENSG0000(	SLFN12L	4.248684	3.587773	0.000258
ENSG0000(	AC108463.	4.248601	1.562207	0.027403
ENSG0000(	DSTNP1	4.231095	1.557924	0.027674
ENSG0000(	TMEM150I	4.229271	5.975016	1.06E-06
ENSG0000(	AP006284.	4.220459	7.629599	2.35E-08
ENSG0000(	AC138466.	4.203528	1.538424	0.028945
ENSG0000(	PDE6G	4.201875	3.427468	0.000374
ENSG0000(	AC018553.	4.196479	1.51749	0.030375
ENSG0000(	FBXO27	4.190332	8.611944	2.44E-09
ENSG0000(	PTPN22	4.184896	5.882876	1.31E-06
ENSG0000(	SIRPG	4.184793	3.911816	0.000123
ENSG0000(	PYHIN1	4.182791	5.384543	4.13E-06
ENSG0000(	FANCB	4.179377	1.520501	0.030165
ENSG0000(	ALDH3A1	4.17636	5.078796	8.34E-06
ENSG0000(	AC133065.	4.16853	5.030002	9.33E-06
ENSG0000(	TNNT2	4.161792	24.25068	5.61E-25
ENSG0000(	TIGIT	4.158773	4.665301	2.16E-05
ENSG0000(	AC010761.	4.1244	10.99598	1.01E-11
ENSG0000(	AC009226.	4.120123	1.502084	0.031471
ENSG0000(	AL645933.:	4.09626	13.36788	4.29E-14
ENSG0000(	AL671277.:	4.078512	5.271494	5.35E-06
ENSG0000(	BLM	4.071029	7.550314	2.82E-08
ENSG0000(	IL31RA	4.068238	8.120432	7.58E-09
ENSG0000(	CHI3L1	4.066855	9.405158	3.93E-10

ENSG0000(	AC063977.	4.064776	1.472164	0.033716
ENSG0000(	AC091182.	4.059073	7.365517	4.31E-08
ENSG0000(	CXCR2P1	4.040539	2.94635	0.001131
ENSG0000(	BICDL1	4.038861	9.66229	2.18E-10
ENSG0000(	AL049775.:	4.029491	2.836983	0.001456
ENSG0000(	CD8A	4.029281	12.52663	2.97E-13
ENSG0000(	ATP8B3	4.024658	5.706681	1.96E-06
ENSG0000(	UBASH3A	4.018787	3.93603	0.000116
ENSG0000(	AC087457.	4.016632	3.148459	0.00071
ENSG0000(	EOMES	4.012197	4.882479	1.31E-05
ENSG0000(	IMPG2	4.008192	3.38738	0.00041
ENSG0000(	SCML4	3.983303	4.16269	6.88E-05
ENSG0000(	CLEC4G	3.976068	3.560708	0.000275
ENSG0000(	UTS2R	3.96443	6.52188	3.01E-07
ENSG0000(	AC009093.	3.963227	3.290953	0.000512
ENSG0000(	AL445490.:	3.957388	3.714151	0.000193
ENSG0000(	GZMH	3.949986	6.9323	1.17E-07
ENSG0000(	AL357054.4	3.94969	6.709361	1.95E-07
ENSG0000(	SLC12A5-A	3.948515	5.958454	1.10E-06
ENSG0000(	AC244021.	3.948204	6.353694	4.43E-07
ENSG0000(	HLA-DQA1	3.947278	18.46289	3.44E-19
ENSG0000(	MMP9	3.943174	8.81004	1.55E-09
ENSG0000(	LINC00598	3.940274	6.412041	3.87E-07
ENSG0000(	RHOH	3.925981	4.389847	4.08E-05
ENSG0000(	AP006621.	3.916423	9.244545	5.69E-10
ENSG0000(	AP000757.	3.913633	5.280889	5.24E-06
ENSG0000(	CD244	3.911317	4.69235	2.03E-05
ENSG0000(	PLCH2	3.900711	5.693846	2.02E-06
ENSG0000(	FAM135B	3.900105	3.065599	0.00086
ENSG0000(	FCRLA	3.898958	1.789108	0.016251
ENSG0000(	HLA-DOB	3.882938	7.16446	6.85E-08
ENSG0000(	IKZF3	3.874586	12.55177	2.81E-13
ENSG0000(	FFAR4	3.859986	2.332374	0.004652
ENSG0000(	CALHM6	3.858228	6.968667	1.07E-07
ENSG0000(	GBP5	3.857548	14.93127	1.17E-15
ENSG0000(	CYP2D7	3.84705	3.600597	0.000251
ENSG0000(	LYPD1	3.846442	3.579345	0.000263
ENSG0000(	MYBPH	3.843325	24.67638	2.11E-25
ENSG0000(	ZNF831	3.840563	5.015802	9.64E-06
ENSG0000(	FLT3	3.837661	3.77564	0.000168
ENSG0000(	CYP4F11	3.835014	1.783667	0.016456
ENSG0000(	LINC01176	3.830894	5.878201	1.32E-06
ENSG0000(	RASL11B	3.825503	4.171446	6.74E-05
ENSG0000(	HASPIN	3.822443	3.87232	0.000134
ENSG0000(	AL157935.:	3.822039	2.541141	0.002876
ENSG0000(	TRAT1	3.815458	3.808873	0.000155
ENSG0000(	SLAMF7	3.80798	16.69956	2.00E-17

ENSG0000(	GPR39	3.807203	5.195867	6.37E-06
ENSG0000(	EXOC3L4	3.803392	10.9104	1.23E-11
ENSG0000(	FCRL3	3.797674	3.155337	0.000699
ENSG0000(	AL109930.:	3.791642	5.266843	5.41E-06
ENSG0000(	CRYBB1	3.791269	2.215259	0.006092
ENSG0000(	LY86	3.787483	5.772372	1.69E-06
ENSG0000(	ZNF396	3.786079	6.801568	1.58E-07
ENSG0000(	LINC00954	3.774643	3.644987	0.000226
ENSG0000(	SLC7A4	3.766808	3.207225	0.000621
ENSG0000(	CYP2S1	3.765403	5.207359	6.20E-06
ENSG0000(	FXYD3	3.764902	2.05494	0.008812
ENSG0000(	TNMD	3.76328	2.785559	0.001638
ENSG0000(	SPON1	3.75651	6.691205	2.04E-07
ENSG0000(	LINC00900	3.75357	9.675041	2.11E-10
ENSG0000(	IRF4	3.749321	8.1521	7.05E-09
ENSG0000(	ADAMTS3	3.747378	3.142108	0.000721
ENSG0000(	SAMSN1	3.74695	6.882219	1.31E-07
ENSG0000(	CDS1	3.746563	3.652797	0.000222
ENSG0000(	TRIM29	3.741893	4.63868	2.30E-05
ENSG0000(	SAA2	3.741741	4.611063	2.45E-05
ENSG0000(	CPA4	3.741184	3.514669	0.000306
ENSG0000(	SNAI1	3.730694	3.904837	0.000124
ENSG0000(	AC098864.	3.724427	5.884047	1.31E-06
ENSG0000(	CYP21A2	3.723453	3.932032	0.000117
ENSG0000(	AL021328.:	3.722771	2.825149	0.001496
ENSG0000(	TMEM200(	3.719288	2.526885	0.002972
ENSG0000(	FBLL1	3.705204	1.708277	0.019576
ENSG0000(	CCL5	3.699452	14.43519	3.67E-15
ENSG0000(	IL12RB1	3.696697	8.115603	7.66E-09
ENSG0000(	TNFSF4	3.695763	8.334491	4.63E-09
ENSG0000(	RARRES3	3.694306	22.81268	1.54E-23
ENSG0000(	PATL2	3.692495	7.688846	2.05E-08
ENSG0000(	ZBP1	3.686661	3.774503	0.000168
ENSG0000(	ZFP69B	3.681049	5.365188	4.31E-06
ENSG0000(	MIR222HG	3.666059	2.123719	0.007521
ENSG0000(	CBX3P2	3.661567	3.422127	0.000378
ENSG0000(	HJURP	3.654444	5.090134	8.13E-06
ENSG0000(	GPR18	3.64793	2.066318	0.008584
ENSG0000(	MAPK15	3.647464	2.749497	0.00178
ENSG0000(	FAM90A1	3.63608	3.330858	0.000467
ENSG0000(	C4B	3.632974	6.77272	1.69E-07
ENSG0000(	MYL4	3.631983	9.559824	2.76E-10
ENSG0000(	EGR2	3.625656	5.186725	6.51E-06
ENSG0000(	CPZ	3.62549	4.744893	1.80E-05
ENSG0000(	TCAF2	3.608872	6.319167	4.80E-07
ENSG0000(	LIPG	3.605732	5.986607	1.03E-06
ENSG0000(	MIR5001	3.602541	3.826022	0.000149

ENSG0000(	CD200R1	3.591111	5.477047	3.33E-06
ENSG0000(	WFDC21P	3.589003	3.776254	0.000167
ENSG0000(	FAM227A	3.588745	3.869371	0.000135
ENSG0000(	AOC4P	3.584708	1.889467	0.012898
ENSG0000(	PNMA6A	3.58308	3.239118	0.000577
ENSG0000(	MKX	3.581434	1.594121	0.025461
ENSG0000(	LINC02362	3.580929	2.627994	0.002355
ENSG0000(	KCNK15-AS	3.577934	2.534782	0.002919
ENSG0000(	APOBEC3B	3.576309	2.227202	0.005926
ENSG0000(	CD40LG	3.575894	3.200381	0.00063
ENSG0000(	RNU6-146F	3.574976	3.008343	0.000981
ENSG0000(	AL512329.:	3.572906	1.473167	0.033638
ENSG0000(	AC116348.	3.571052	5.772372	1.69E-06
ENSG0000(	B3GALT2	3.567687	3.139494	0.000725
ENSG0000(	CHI3L2	3.566286	5.111462	7.74E-06
ENSG0000(	LINC01679	3.559386	3.414936	0.000385
ENSG0000(	AC008429.	3.556475	2.44492	0.00359
ENSG0000(	AC114980.	3.555511	2.334307	0.004631
ENSG0000(	AL355102.:	3.554881	3.387748	0.000409
ENSG0000(	CENPM	3.550116	5.807213	1.56E-06
ENSG0000(	DGKB	3.541136	4.169003	6.78E-05
ENSG0000(	HLA-DQB1-	3.539752	13.78146	1.65E-14
ENSG0000(	FPR3	3.531269	12.2413	5.74E-13
ENSG0000(	DHRS9	3.529925	6.502301	3.15E-07
ENSG0000(	ABCD2	3.521243	4.429755	3.72E-05
ENSG0000(	C10orf67	3.520049	1.433398	0.036864
ENSG0000(	NAGS	3.517012	3.887448	0.00013
ENSG0000(	GBP1	3.513309	20.68924	2.05E-21
ENSG0000(	LMTK3	3.510405	3.047063	0.000897
ENSG0000(	KLHL4	3.506864	4.833181	1.47E-05
ENSG0000(	GPR78	3.505689	4.426487	3.75E-05
ENSG0000(	AP000662.	3.497719	5.232482	5.85E-06
ENSG0000(	C2	3.496843	27.41873	3.81E-28
ENSG0000(	RNA5SP28:	3.493984	2.275566	0.005302
ENSG0000(	TTC22	3.49361	2.548147	0.00283
ENSG0000(	JPH3	3.492588	1.909188	0.012326
ENSG0000(	MILR1	3.487777	8.261574	5.48E-09
ENSG0000(	AC090971.	3.485536	2.952021	0.001117
ENSG0000(	PRUNE2	3.478371	22.69316	2.03E-23
ENSG0000(	AC241377.	3.477669	4.692595	2.03E-05
ENSG0000(	AC022415.	3.475499	2.227118	0.005928
ENSG0000(	AF131215.(	3.474821	5.915054	1.22E-06
ENSG0000(	AC226101.	3.471996	3.209458	0.000617
ENSG0000(	AL513122.:	3.468509	5.443062	3.61E-06
ENSG0000(	HMSD	3.464916	5.676891	2.10E-06
ENSG0000(	MATK	3.463625	8.360027	4.36E-09
ENSG0000(	AL845552.:	3.462248	1.615975	0.024212

ENSG0000(	ST8SIA2	3.461168	9.510497	3.09E-10
ENSG0000(	FAM72B	3.458116	2.554285	0.002791
ENSG0000(	AC068789.	3.456319	3.965177	0.000108
ENSG0000(	RPRM	3.453641	1.672414	0.021261
ENSG0000(	AC012313.	3.450038	3.68289	0.000208
ENSG0000(	AC019069.	3.448993	2.164667	0.006844
ENSG0000(	RORB	3.4479	1.540715	0.028793
ENSG0000(	P2RY12	3.437061	4.416703	3.83E-05
ENSG0000(	HSD17B3	3.433385	2.970756	0.00107
ENSG0000(	LINC01503	3.433017	1.932311	0.011687
ENSG0000(	AC245100.	3.432738	2.588903	0.002577
ENSG0000(	CD86	3.42615	6.019956	9.55E-07
ENSG0000(	SVOP	3.424737	5.951521	1.12E-06
ENSG0000(	HLA-DQB1	3.414832	16.24339	5.71E-17
ENSG0000(	CNIH2	3.412779	2.119196	0.0076
ENSG0000(	FOLR1	3.409693	1.63551	0.023147
ENSG0000(	TRBJ2-6	3.406394	2.110575	0.007752
ENSG0000(	FGF16	3.40519	1.851614	0.014073
ENSG0000(	AL157955.1	3.405139	2.009247	0.009789
ENSG0000(	GZMM	3.404083	5.174134	6.70E-06
ENSG0000(	SIK1	3.401613	1.605144	0.024823
ENSG0000(	TRAC	3.397911	10.29565	5.06E-11
ENSG0000(	HLA-DOA	3.39456	16.48384	3.28E-17
ENSG0000(	AL592146.1	3.387566	2.136824	0.007298
ENSG0000(	C1QTNF4	3.387187	4.63066	2.34E-05
ENSG0000(	CCL28	3.377741	1.355824	0.044073
ENSG0000(	TRBJ2-2P	3.376391	2.717007	0.001919
ENSG0000(	XKR6	3.372514	3.220456	0.000602
ENSG0000(	B3GAT1	3.365142	4.321342	4.77E-05
ENSG0000(	PTPN7	3.354656	9.475717	3.34E-10
ENSG0000(	GJD4	3.354237	1.30344	0.049723
ENSG0000(	PCA3	3.349139	7.827284	1.49E-08
ENSG0000(	AD000864.	3.337809	10.28457	5.19E-11
ENSG0000(	TMEM191A	3.336436	1.755189	0.017572
ENSG0000(	HCP5	3.33578	23.93171	1.17E-24
ENSG0000(	MMP2-AS1	3.327714	1.513398	0.030662
ENSG0000(	ACP5	3.327231	15.54984	2.82E-16
ENSG0000(	CYP4F22	3.322267	2.339285	0.004578
ENSG0000(	DERL3	3.315867	12.09617	8.01E-13
ENSG0000(	AC002398.	3.31499	3.280272	0.000524
ENSG0000(	AL138976.1	3.308369	2.112588	0.007716
ENSG0000(	RAG1	3.305228	2.448214	0.003563
ENSG0000(	AC025271.	3.303498	1.553747	0.027942
ENSG0000(	RF02271	3.302029	2.160824	0.006905
ENSG0000(	TRBC1	3.297194	8.551837	2.81E-09
ENSG0000(	HCST	3.294836	9.980072	1.05E-10
ENSG0000(	PAMR1	3.294672	13.84856	1.42E-14

ENSG0000(	AC084398.	3.294252	5.05206	8.87E-06
ENSG0000(	C1QB	3.290648	12.96816	1.08E-13
ENSG0000(	LILRB4	3.288179	10.63836	2.30E-11
ENSG0000(	XCR1	3.288149	4.060929	8.69E-05
ENSG0000(	NLRP2	3.282073	4.834063	1.47E-05
ENSG0000(	TCAF2P1	3.270864	2.618426	0.002408
ENSG0000(	SKAP1	3.269496	4.881198	1.31E-05
ENSG0000(	ASCL5	3.268434	5.638673	2.30E-06
ENSG0000(	NPAS3	3.259619	4.101308	7.92E-05
ENSG0000(	LY9	3.256014	3.051592	0.000888
ENSG0000(	E2F2	3.247502	2.825706	0.001494
ENSG0000(	TSPAN10	3.243517	2.288714	0.005144
ENSG0000(	OASL	3.240959	7.441609	3.62E-08
ENSG0000(	CA12	3.240312	2.778559	0.001665
ENSG0000(	TRBJ2-3	3.234172	3.147042	0.000713
ENSG0000(	HLA-DRA	3.232087	16.46861	3.40E-17
ENSG0000(	Z99774.1	3.231142	6.616671	2.42E-07
ENSG0000(	TRBC2	3.229665	12.33793	4.59E-13
ENSG0000(	AOAH	3.229405	6.090032	8.13E-07
ENSG0000(	GALNT5	3.226574	1.941105	0.011452
ENSG0000(	WNK3	3.223464	2.302174	0.004987
ENSG0000(	ZNF321P	3.222207	5.686281	2.06E-06
ENSG0000(	LMNTD2	3.219295	4.942379	1.14E-05
ENSG0000(	STAT1	3.21923	22.69316	2.03E-23
ENSG0000(	TFEC	3.218263	6.913294	1.22E-07
ENSG0000(	KLRC4-KLRI	3.218142	10.98802	1.03E-11
ENSG0000(	BLK	3.216467	1.446718	0.03575
ENSG0000(	LCK	3.215452	9.284158	5.20E-10
ENSG0000(	AL031590.1	3.214853	1.458695	0.034778
ENSG0000(	NOD2	3.208908	6.970064	1.07E-07
ENSG0000(	BCL11B	3.208741	3.054342	0.000882
ENSG0000(	ZNF311	3.206974	1.949311	0.011238
ENSG0000(	KRT222	3.195815	2.433128	0.003689
ENSG0000(	KLRK1	3.195394	10.3874	4.10E-11
ENSG0000(	COL19A1	3.192269	3.323666	0.000475
ENSG0000(	ACTC1	3.192106	14.98181	1.04E-15
ENSG0000(	PPP2R2B	3.19194	5.772372	1.69E-06
ENSG0000(	ZNF572	3.190505	1.913243	0.012211
ENSG0000(	LINC01238	3.189528	2.876244	0.00133
ENSG0000(	TMEM225I	3.189458	1.909383	0.01232
ENSG0000(	CNTNAP4	3.187682	1.631611	0.023355
ENSG0000(	HPSE	3.184445	3.897846	0.000127
ENSG0000(	CTSW	3.180254	8.917055	1.21E-09
ENSG0000(	AC087645.	3.176123	1.876049	0.013303
ENSG0000(	GASAL1	3.17076	1.452284	0.035295
ENSG0000(	GPR183	3.169826	4.030845	9.31E-05
ENSG0000(	HS3ST3A1	3.166506	1.431019	0.037066

ENSG0000(	SHANK1	3.166307	5.05652	8.78E-06
ENSG0000(	CD6	3.164942	8.069258	8.53E-09
ENSG0000(	AC068631.	3.163146	3.180252	0.00066
ENSG0000(	IL2RG	3.162995	11.28993	5.13E-12
ENSG0000(	TNFRSF11E	3.156765	2.295933	0.005059
ENSG0000(	LGALS2	3.155429	5.55638	2.78E-06
ENSG0000(	RF00285	3.155097	1.423533	0.037711
ENSG0000(	AL359513.:	3.154451	1.80917	0.015518
ENSG0000(	ZNF280B	3.14926	5.239971	5.75E-06
ENSG0000(	ESPNL	3.146692	2.58868	0.002578
ENSG0000(	STX1B	3.146143	10.98536	1.03E-11
ENSG0000(	CCR2	3.144265	4.44314	3.60E-05
ENSG0000(	HMGB1P3	3.143399	5.778179	1.67E-06
ENSG0000(	SEC22B4P	3.141611	2.527617	0.002967
ENSG0000(	CDC37L1-D	3.137972	1.637756	0.023027
ENSG0000(	HLA-DQB2	3.134087	6.902138	1.25E-07
ENSG0000(	AC243960.	3.129985	2.507598	0.003107
ENSG0000(	IFI30	3.124274	14.14979	7.08E-15
ENSG0000(	DMRT3	3.122867	2.458306	0.003481
ENSG0000(	IRF8	3.11893	11.66064	2.18E-12
ENSG0000(	CD3E	3.117575	9.486084	3.27E-10
ENSG0000(	PRRX2	3.116277	4.184115	6.54E-05
ENSG0000(	MYH3	3.115092	14.69977	2.00E-15
ENSG0000(	AC018653.	3.113809	4.664807	2.16E-05
ENSG0000(	MARCO	3.109772	6.663528	2.17E-07
ENSG0000(	AC004890.	3.101812	2.014123	0.00968
ENSG0000(	SOHLH2	3.100607	3.426095	0.000375
ENSG0000(	MIR6821	3.098441	5.467172	3.41E-06
ENSG0000(	PPM1E	3.098359	4.874048	1.34E-05
ENSG0000(	PTPRO	3.098147	6.343589	4.53E-07
ENSG0000(	WHRN	3.096482	6.949095	1.12E-07
ENSG0000(	ITK	3.096011	6.442713	3.61E-07
ENSG0000(	AL137784.:	3.093851	1.626984	0.023606
ENSG0000(	PSMB8	3.091759	22.81268	1.54E-23
ENSG0000(	KLRD1	3.087466	4.817022	1.52E-05
ENSG0000(	TET2-AS1	3.082911	1.341572	0.045544
ENSG0000(	AC016757.	3.08054	1.459876	0.034684
ENSG0000(	ARC	3.07972	2.116974	0.007639
ENSG0000(	AL645922.:	3.078884	22.40139	3.97E-23
ENSG0000(	AL590764.:	3.077283	10.67739	2.10E-11
ENSG0000(	MT1F	3.077166	2.820958	0.00151
ENSG0000(	AC008011.	3.074927	1.75667	0.017512
ENSG0000(	ITGB7	3.070651	9.979934	1.05E-10
ENSG0000(	MSR1	3.067557	8.060996	8.69E-09
ENSG0000(	AL035587.:	3.066483	3.226364	0.000594
ENSG0000(	AL121985.:	3.059629	5.794942	1.60E-06
ENSG0000(	PLA2G5	3.05444	4.398898	3.99E-05

ENSG0000(	APOBEC3G	3.050876	17.14295	7.20E-18
ENSG0000(	GZMB	3.045423	5.010635	9.76E-06
ENSG0000(	PCED1B-AS	3.044302	5.672978	2.12E-06
ENSG0000(	TFR2	3.042231	2.061843	0.008673
ENSG0000(	PRR7-AS1	3.040492	1.366805	0.042973
ENSG0000(	CD101	3.035804	3.084718	0.000823
ENSG0000(	C8orf88	3.033223	2.647691	0.002251
ENSG0000(	AC005538.	3.030753	4.511291	3.08E-05
ENSG0000(	NCKAP5	3.029028	3.366492	0.00043
ENSG0000(	MSL3P1	3.025862	4.461602	3.45E-05
ENSG0000(	MIR6819	3.024285	1.530141	0.029503
ENSG0000(	TBC1D8-AS	3.023186	1.829379	0.014812
ENSG0000(	ANGPTL5	3.020923	4.75856	1.74E-05
ENSG0000(	ZC2HC1B	3.019222	1.302405	0.049842
ENSG0000(	PENK	3.017078	12.99599	1.01E-13
ENSG0000(	CDC7	3.016108	5.727463	1.87E-06
ENSG0000(	CD52	3.013127	6.039773	9.12E-07
ENSG0000(	TBX21	3.012684	4.422591	3.78E-05
ENSG0000(	SIGLEC11	3.010191	2.04945	0.008924
ENSG0000(	CSTA	3.009741	1.715809	0.019239
ENSG0000(	PSMB9	3.008106	18.73699	1.83E-19
ENSG0000(	CD48	3.005686	6.238765	5.77E-07
ENSG0000(	AC241585.	3.003189	4.495836	3.19E-05
ENSG0000(	KCNK1	3.002957	1.385146	0.041196
ENSG0000(	LPAR5	3.002462	3.361733	0.000435
ENSG0000(	TYMSOS	3.002397	1.31562	0.048348
ENSG0000(	DNAH5	2.995638	1.462337	0.034488
ENSG0000(	HLA-DPB1	2.995175	14.97417	1.06E-15
ENSG0000(	CPNE5	2.993111	3.697439	0.000201
ENSG0000(	BATF2	2.991588	11.70609	1.97E-12
ENSG0000(	AC103974.	2.988145	2.036891	0.009186
ENSG0000(	AC011468.	2.980925	1.673397	0.021213
ENSG0000(	PLA2G7	2.976438	3.313188	0.000486
ENSG0000(	MATN1-AS	2.97567	1.441251	0.036203
ENSG0000(	HLA-DPA1	2.973679	15.28176	5.23E-16
ENSG0000(	ADTRP	2.973088	1.428777	0.037258
ENSG0000(	LSAMP	2.9711	3.700867	0.000199
ENSG0000(	C1orf105	2.967617	3.022302	0.00095
ENSG0000(	AC005264.	2.961509	2.800847	0.001582
ENSG0000(	POSTN	2.961201	12.91152	1.23E-13
ENSG0000(	SAA1	2.957544	8.08763	8.17E-09
ENSG0000(	SH2D2A	2.95666	4.79333	1.61E-05
ENSG0000(	AL121839.:	2.955677	4.458255	3.48E-05
ENSG0000(	HLA-DRB1	2.95561	16.80453	1.57E-17
ENSG0000(	AC116348.	2.954858	3.582298	0.000262
ENSG0000(	ARHGAP8	2.954232	3.509361	0.000309
ENSG0000(	AC022075.	2.953811	6.753505	1.76E-07

ENSG0000(	AKNAD1	2.948969	2.627062	0.00236
ENSG0000(	CD300C	2.947867	2.711942	0.001941
ENSG0000(	AC138207.	2.943124	2.40256	0.003958
ENSG0000(	IKBKE	2.939894	5.797497	1.59E-06
ENSG0000(	RF00015	2.937069	1.570261	0.026899
ENSG0000(	AC005332.	2.933178	1.88919	0.012907
ENSG0000(	DUX4L50	2.932699	1.966154	0.01081
ENSG0000(	AL512506.:	2.93233	2.262463	0.005464
ENSG0000(	C1QA	2.930046	11.35891	4.38E-12
ENSG0000(	GIPR	2.929942	1.743008	0.018071
ENSG0000(	HAP1	2.928279	1.399659	0.039842
ENSG0000(	AC004540.	2.925782	3.022351	0.00095
ENSG0000(	AKR7A3	2.923686	3.238948	0.000577
ENSG0000(	AL021707.(	2.920234	2.194902	0.006384
ENSG0000(	LINC00638	2.916795	1.643364	0.022732
ENSG0000(	PARD6G-A!	2.915651	1.469409	0.033931
ENSG0000(	SPOCD1	2.915064	1.462086	0.034508
ENSG0000(	AL022323.:	2.915028	1.303859	0.049675
ENSG0000(	CARD11	2.912703	7.577443	2.65E-08
ENSG0000(	AL645939.:	2.912284	18.90457	1.25E-19
ENSG0000(	ANKRD1	2.911031	14.99339	1.02E-15
ENSG0000(	LINC01550	2.904202	4.789288	1.62E-05
ENSG0000(	CD3D	2.902807	6.34492	4.52E-07
ENSG0000(	TNFSF8	2.901776	4.50404	3.13E-05
ENSG0000(	TAP1	2.901489	19.57716	2.65E-20
ENSG0000(	EPSTI1	2.899821	14.71506	1.93E-15
ENSG0000(	PLA2G2A	2.895158	5.415796	3.84E-06
ENSG0000(	KCNJ5	2.894255	6.102155	7.90E-07
ENSG0000(	CD5	2.892054	6.102155	7.90E-07
ENSG0000(	RBP5	2.891006	6.698209	2.00E-07
ENSG0000(	TNFAIP8L3	2.886539	9.881737	1.31E-10
ENSG0000(	CBY3	2.886037	1.593095	0.025521
ENSG0000(	MYH8	2.885356	7.343852	4.53E-08
ENSG0000(	TFP1	2.882311	1.392664	0.040489
ENSG0000(	AC025048.	2.876559	4.037145	9.18E-05
ENSG0000(	AC245100.	2.874429	4.060929	8.69E-05
ENSG0000(	AC005410.	2.874015	1.442784	0.036076
ENSG0000(	PLEK	2.872768	12.77414	1.68E-13
ENSG0000(	OTUB2	2.867862	1.416691	0.03831
ENSG0000(	NAT1	2.865362	4.708524	1.96E-05
ENSG0000(	AC104699.	2.865328	2.165341	0.006834
ENSG0000(	AP000759.	2.863781	2.178633	0.006628
ENSG0000(	HLA-DMB	2.861951	15.9895	1.02E-16
ENSG0000(	AC010761.	2.860373	6.475063	3.35E-07
ENSG0000(	FAM180A	2.857129	1.75333	0.017647
ENSG0000(	PSMB8-AS:	2.855853	18.58176	2.62E-19
ENSG0000(	AC092123.	2.855678	1.365373	0.043115

ENSG0000(	METTL27	2.851255	1.944621	0.01136
ENSG0000(	TMEM132I	2.850875	2.076715	0.008381
ENSG0000(	AL031432.:	2.833891	1.778991	0.016634
ENSG0000(	CCBE1	2.830897	1.874397	0.013354
ENSG0000(	TMEM229I	2.827233	10.45072	3.54E-11
ENSG0000(	MYB	2.816705	1.329101	0.04687
ENSG0000(	IGLV2-18	2.815023	1.67358	0.021204
ENSG0000(	BHLHE22	2.811657	5.498233	3.18E-06
ENSG0000(	TNFSF14	2.810073	5.016393	9.63E-06
ENSG0000(	DRAXIN	2.807654	4.17008	6.76E-05
ENSG0000(	AC242842.	2.807565	2.632089	0.002333
ENSG0000(	KIAA1324	2.807317	2.350041	0.004466
ENSG0000(	BTN3A2	2.807244	16.06172	8.68E-17
ENSG0000(	GBP7	2.806608	3.935811	0.000116
ENSG0000(	LYPD3	2.806527	1.362304	0.043421
ENSG0000(	LINC00173	2.805294	4.325913	4.72E-05
ENSG0000(	RN7SL832F	2.804571	1.359703	0.043681
ENSG0000(	APOL4	2.794896	13.69084	2.04E-14
ENSG0000(	KRT80	2.787435	7.94781	1.13E-08
ENSG0000(	TNFRSF13C	2.78737	2.169815	0.006764
ENSG0000(	IL2RB	2.78727	6.609778	2.46E-07
ENSG0000(	CD96	2.786611	5.327858	4.70E-06
ENSG0000(	AL391069.:	2.781791	2.756954	0.00175
ENSG0000(	MS4A14	2.779296	4.685237	2.06E-05
ENSG0000(	C1QC	2.775032	11.3067	4.94E-12
ENSG0000(	ADRB1	2.772085	4.385301	4.12E-05
ENSG0000(	CLEC10A	2.771432	10.18041	6.60E-11
ENSG0000(	CCND2-AS1	2.771329	6.711552	1.94E-07
ENSG0000(	VCAM1	2.766889	14.20948	6.17E-15
ENSG0000(	COL22A1	2.760911	1.70638	0.019662
ENSG0000(	AL645941.:	2.758309	16.25039	5.62E-17
ENSG0000(	RALY-AS1	2.752826	2.609529	0.002457
ENSG0000(	MYRF	2.751699	3.995098	0.000101
ENSG0000(	MS4A7	2.751442	14.67034	2.14E-15
ENSG0000(	RUNX3	2.751036	8.832833	1.47E-09
ENSG0000(	IL10RA	2.750885	14.541	2.88E-15
ENSG0000(	ADAP1	2.748231	9.882606	1.31E-10
ENSG0000(	TNFSF13B	2.745376	9.881737	1.31E-10
ENSG0000(	BATF3	2.74164	4.465444	3.42E-05
ENSG0000(	C4A	2.738271	6.214147	6.11E-07
ENSG0000(	MS4A4A	2.734134	8.170126	6.76E-09
ENSG0000(	HLA-C	2.732682	20.16449	6.85E-21
ENSG0000(	LYZ	2.728815	6.667247	2.15E-07
ENSG0000(	PHF21B	2.728579	2.277649	0.005277
ENSG0000(	HLA-F	2.724978	17.60366	2.49E-18
ENSG0000(	PTPRCAP	2.721942	11.22129	6.01E-12
ENSG0000(	NHLH2	2.721122	4.175441	6.68E-05

ENSG0000(	CCDC170	2.720701	4.25967	5.50E-05
ENSG0000(	AL157871.:	2.719939	1.410309	0.038877
ENSG0000(	APOL1	2.719654	22.81268	1.54E-23
ENSG0000(	LRIT3	2.71943	1.68344	0.020728
ENSG0000(	FAM218A	2.718806	1.915935	0.012136
ENSG0000(	LILRB1	2.717367	7.186594	6.51E-08
ENSG0000(	CD180	2.717289	3.610544	0.000245
ENSG0000(	RNASE6	2.716358	5.932411	1.17E-06
ENSG0000(	SH2D1A	2.716153	3.568323	0.00027
ENSG0000(	AC009802.	2.713736	1.994117	0.010136
ENSG0000(	MYO1G	2.712598	8.644421	2.27E-09
ENSG0000(	LGALS17A	2.712148	8.91408	1.22E-09
ENSG0000(	ARL4C	2.706373	17.41448	3.85E-18
ENSG0000(	LINC01239	2.706369	2.11558	0.007663
ENSG0000(	1-Dec	2.705565	3.086621	0.000819
ENSG0000(	CLDN22	2.704005	1.843257	0.014346
ENSG0000(	CD53	2.70366	10.39617	4.02E-11
ENSG0000(	AC141557.	2.701827	1.977787	0.010525
ENSG0000(	ORC1	2.700848	1.66875	0.021441
ENSG0000(	AL671277.:	2.700601	20.63791	2.30E-21
ENSG0000(	FIRRE	2.700199	1.663341	0.02171
ENSG0000(	AP000919.	2.699129	3.898686	0.000126
ENSG0000(	ADH1A	2.698931	1.414497	0.038504
ENSG0000(	CST7	2.697413	6.789326	1.62E-07
ENSG0000(	AIFM3	2.69648	2.474594	0.003353
ENSG0000(	EVI2A	2.692982	6.580888	2.62E-07
ENSG0000(	AC020656.	2.690287	7.345372	4.51E-08
ENSG0000(	NKG7	2.687949	5.142605	7.20E-06
ENSG0000(	TYMP	2.685166	16.10853	7.79E-17
ENSG0000(	AC062029.	2.684382	1.714657	0.01929
ENSG0000(	TLR7	2.683137	4.477471	3.33E-05
ENSG0000(	BCAS1	2.683026	3.647564	0.000225
ENSG0000(	GBP1P1	2.682616	9.87104	1.35E-10
ENSG0000(	AL022318.4	2.681266	17.01965	9.56E-18
ENSG0000(	NAPSB	2.680577	6.029327	9.35E-07
ENSG0000(	APOBEC3D	2.680482	11.05568	8.80E-12
ENSG0000(	CD84	2.676945	11.82687	1.49E-12
ENSG0000(	AC004900.	2.675027	2.690453	0.00204
ENSG0000(	CPXM1	2.674786	6.688266	2.05E-07
ENSG0000(	AC011731.	2.673065	2.401571	0.003967
ENSG0000(	HLA-B	2.672454	18.92624	1.19E-19
ENSG0000(	AC004687.	2.671165	5.202381	6.28E-06
ENSG0000(	SIGLEC16	2.670852	3.748528	0.000178
ENSG0000(	FCHO1	2.669936	5.164723	6.84E-06
ENSG0000(	PCK1	2.668235	1.741967	0.018115
ENSG0000(	AC018904.	2.663221	1.4637	0.034379
ENSG0000(	AC005831.	2.661231	2.260798	0.005485

ENSG0000(	B2M	2.657364	18.66494	2.16E-19
ENSG0000(	NCKAP1L	2.655836	11.26297	5.46E-12
ENSG0000(	CCL2	2.655572	8.015687	9.65E-09
ENSG0000(	AP000802.	2.654788	3.389681	0.000408
ENSG0000(	ZNF215	2.652918	1.906587	0.0124
ENSG0000(	RASAL3	2.652226	10.68999	2.04E-11
ENSG0000(	SDC1	2.65164	7.975449	1.06E-08
ENSG0000(	ITGAL	2.642659	9.528997	2.96E-10
ENSG0000(	DIRAS1	2.642232	8.892013	1.28E-09
ENSG0000(	CHRNA1	2.634598	12.25398	5.57E-13
ENSG0000(	ANO9	2.633901	4.488885	3.24E-05
ENSG0000(	HLA-A	2.632481	20.02686	9.40E-21
ENSG0000(	SHD	2.630252	2.80914	0.001552
ENSG0000(	SOCS1	2.629462	4.330472	4.67E-05
ENSG0000(	AL671883.:	2.628814	14.65603	2.21E-15
ENSG0000(	AC004494.	2.625646	3.482408	0.000329
ENSG0000(	TBC1D10C	2.623789	8.23042	5.88E-09
ENSG0000(	CLEC12A	2.623676	2.799472	0.001587
ENSG0000(	MS4A6A	2.623387	10.87434	1.34E-11
ENSG0000(	CERKL	2.623192	4.553944	2.79E-05
ENSG0000(	RAET1E-AS	2.623183	2.43972	0.003633
ENSG0000(	TRARG1	2.620674	2.928905	0.001178
ENSG0000(	BANK1	2.619925	2.097036	0.007998
ENSG0000(	CCDC150	2.618287	6.049969	8.91E-07
ENSG0000(	AL008729.:	2.614979	1.530891	0.029452
ENSG0000(	HLA-DQA2	2.613079	2.705739	0.001969
ENSG0000(	AC242842.	2.609454	2.016828	0.00962
ENSG0000(	TOX2	2.606199	3.389756	0.000408
ENSG0000(	HTR7	2.605813	3.935464	0.000116
ENSG0000(	AC098679.	2.605506	1.328532	0.046932
ENSG0000(	CETP	2.604299	2.630028	0.002344
ENSG0000(	NUF2	2.603946	2.605954	0.002478
ENSG0000(	VNN1	2.603741	2.451947	0.003532
ENSG0000(	CIDEA	2.603316	1.861954	0.013742
ENSG0000(	CD4	2.602344	11.55999	2.75E-12
ENSG0000(	CDH17	2.602184	1.38126	0.041566
ENSG0000(	RASGRP1	2.60134	4.306267	4.94E-05
ENSG0000(	CD163	2.600966	8.764464	1.72E-09
ENSG0000(	DENND1C	2.600168	5.294215	5.08E-06
ENSG0000(	HLA-DRB5	2.600118	9.604011	2.49E-10
ENSG0000(	EID3	2.59853	2.822331	0.001505
ENSG0000(	MS4A1	2.585202	1.490399	0.03233
ENSG0000(	A1BG-AS1	2.584592	6.882486	1.31E-07
ENSG0000(	IFIT3	2.583131	14.65468	2.21E-15
ENSG0000(	GCNT1	2.580936	18.44139	3.62E-19
ENSG0000(	GBP2	2.5808	27.79333	1.61E-28
ENSG0000(	AC099850.	2.578521	1.575389	0.026583

ENSG0000(	DOK2	2.577063	11.53715	2.90E-12
ENSG0000(	EGR3	2.574242	4.733644	1.85E-05
ENSG0000(	PANO1	2.569732	2.194261	0.006394
ENSG0000(	CD74	2.568175	12.99541	1.01E-13
ENSG0000(	AC011603.	2.567948	7.203325	6.26E-08
ENSG0000(	TCF23	2.563504	3.033556	0.000926
ENSG0000(	RRN3P2	2.562953	2.890203	0.001288
ENSG0000(	SASH3	2.561768	6.096253	8.01E-07
ENSG0000(	MIAT	2.560931	13.40977	3.89E-14
ENSG0000(	DUXAP8	2.558913	3.397686	0.0004
ENSG0000(	ITGA4	2.558443	4.919034	1.20E-05
ENSG0000(	AC012349.	2.557555	9.411221	3.88E-10
ENSG0000(	BLNK	2.557462	8.240884	5.74E-09
ENSG0000(	AC011511.	2.55592	14.20313	6.26E-15
ENSG0000(	ERBB3	2.55499	9.083371	8.25E-10
ENSG0000(	APOBEC3C	2.553069	24.37494	4.22E-25
ENSG0000(	UBE2L6	2.552773	22.00998	9.77E-23
ENSG0000(	CXorf21	2.551273	2.255715	0.00555
ENSG0000(	LCP1	2.550295	9.059594	8.72E-10
ENSG0000(	AC116348.	2.548316	4.163522	6.86E-05
ENSG0000(	EMB	2.548207	9.631518	2.34E-10
ENSG0000(	GYG2	2.54774	3.033801	0.000925
ENSG0000(	NLRC5	2.547565	18.33864	4.59E-19
ENSG0000(	DCLK1	2.546477	19.89954	1.26E-20
ENSG0000(	AIF1	2.544163	7.612129	2.44E-08
ENSG0000(	AL512791.:	2.543628	2.194965	0.006383
ENSG0000(	CASS4	2.541646	4.35113	4.46E-05
ENSG0000(	IKZF1	2.537827	7.661206	2.18E-08
ENSG0000(	COL8A2	2.533039	8.498995	3.17E-09
ENSG0000(	MIR6891	2.530263	11.44548	3.59E-12
ENSG0000(	DDN	2.530251	6.574041	2.67E-07
ENSG0000(	CTSS	2.529566	9.715984	1.92E-10
ENSG0000(	AC134669.	2.527637	4.600806	2.51E-05
ENSG0000(	AC092919.	2.527461	1.514806	0.030563
ENSG0000(	AC093732.	2.527005	3.474276	0.000336
ENSG0000(	ADIPOQ-AS	2.526155	2.948577	0.001126
ENSG0000(	LPAR4	2.520408	1.313058	0.048634
ENSG0000(	BTN3A3	2.519908	21.74538	1.80E-22
ENSG0000(	AL390066.:	2.51789	1.372047	0.042457
ENSG0000(	MAP1A	2.510915	24.19913	6.32E-25
ENSG0000(	ARMH1	2.510835	2.11811	0.007619
ENSG0000(	LRRC43	2.510678	1.690674	0.020386
ENSG0000(	TRIM9	2.51017	5.52534	2.98E-06
ENSG0000(	ANKRD36B	2.508825	1.328673	0.046917
ENSG0000(	HLA-K	2.508648	7.442967	3.61E-08
ENSG0000(	AC105118.	2.505836	1.410812	0.038832
ENSG0000(	ADIPOQ	2.502612	2.938024	0.001153

ENSG0000(	LAIR1	2.502522	13.76715	1.71E-14
ENSG0000(	TMEM200/	2.495663	4.961386	1.09E-05
ENSG0000(	S100A3	2.495592	2.355229	0.004413
ENSG0000(	DOCK2	2.492249	8.854914	1.40E-09
ENSG0000(	F13A1	2.489744	8.328916	4.69E-09
ENSG0000(	DNAH14	2.488607	1.463707	0.034379
ENSG0000(	EVI2B	2.486113	4.337734	4.59E-05
ENSG0000(	ICAM1	2.485744	16.43344	3.69E-17
ENSG0000(	AC005840.	2.484745	3.220041	0.000603
ENSG0000(	MED12L	2.483965	6.358339	4.38E-07
ENSG0000(	TYROBP	2.481687	8.945193	1.13E-09
ENSG0000(	CD28	2.480817	2.739986	0.00182
ENSG0000(	FYB1	2.479131	7.17539	6.68E-08
ENSG0000(	BCL2A1	2.477761	1.43192	0.03699
ENSG0000(	GFRA2	2.47776	5.161207	6.90E-06
ENSG0000(	IRF1	2.476985	15.54984	2.82E-16
ENSG0000(	COL14A1	2.474095	7.722326	1.90E-08
ENSG0000(	LINC00475	2.473813	1.353026	0.044358
ENSG0000(	AC048380.	2.472961	1.856204	0.013925
ENSG0000(	LINC01135	2.471578	3.189953	0.000646
ENSG0000(	TMSB4XP4	2.471441	2.863736	0.001369
ENSG0000(	LEF1	2.471063	2.160823	0.006905
ENSG0000(	LINC00484	2.470278	3.745331	0.00018
ENSG0000(	HRASLS5	2.4701	2.385456	0.004117
ENSG0000(	AL133467.:	2.466788	1.862326	0.01373
ENSG0000(	PMAIP1	2.466082	4.172315	6.72E-05
ENSG0000(	CAPG	2.46406	16.52543	2.98E-17
ENSG0000(	PTGES3P1	2.464038	17.01575	9.64E-18
ENSG0000(	SBK2	2.463747	2.447559	0.003568
ENSG0000(	ZNF681	2.46335	2.071318	0.008486
ENSG0000(	NCS1	2.462634	23.0101	9.77E-24
ENSG0000(	RAB42	2.460258	5.388241	4.09E-06
ENSG0000(	AC138028.	2.460214	2.110611	0.007752
ENSG0000(	DUSP4	2.457373	14.57865	2.64E-15
ENSG0000(	SLC47A2	2.454855	3.383278	0.000414
ENSG0000(	KIAA1755	2.452728	10.24317	5.71E-11
ENSG0000(	ZNF625	2.452039	4.833391	1.47E-05
ENSG0000(	SNX10	2.451453	9.941259	1.14E-10
ENSG0000(	CDK18	2.450931	19.92709	1.18E-20
ENSG0000(	PTGER2	2.45046	2.840015	0.001445
ENSG0000(	PLCE1-AS1	2.449497	2.816499	0.001526
ENSG0000(	BIRC3	2.449433	12.88895	1.29E-13
ENSG0000(	ZNF426-DT	2.44782	2.23434	0.00583
ENSG0000(	APOL6	2.447753	28.10649	7.83E-29
ENSG0000(	ITGB2-AS1	2.446554	2.400251	0.003979
ENSG0000(	PLIN1	2.443619	2.839169	0.001448
ENSG0000(	BRWD1-AS	2.440567	2.35852	0.00438

ENSG0000(	FCGR3A	2.438639	5.042195	9.07E-06
ENSG0000(	PIM2	2.436992	10.74038	1.82E-11
ENSG0000(	BTN3A1	2.435575	17.45724	3.49E-18
ENSG0000(	ZCCHC18	2.435533	2.82	0.001514
ENSG0000(	HLA-DRB6	2.430003	8.483802	3.28E-09
ENSG0000(	RPL24P8	2.422813	1.386567	0.041061
ENSG0000(	LAD1	2.422555	11.4154	3.84E-12
ENSG0000(	AC109460.	2.422507	2.79591	0.0016
ENSG0000(	SLC23A3	2.42124	3.003647	0.000992
ENSG0000(	RMI2	2.420336	4.470223	3.39E-05
ENSG0000(	P2RX7	2.419472	10.50807	3.10E-11
ENSG0000(	AL118508.:	2.419081	1.484179	0.032796
ENSG0000(	KCNN4	2.418665	6.486689	3.26E-07
ENSG0000(	BTK	2.418203	4.800804	1.58E-05
ENSG0000(	AC096677.	2.416569	4.777469	1.67E-05
ENSG0000(	CD1C	2.414223	4.418801	3.81E-05
ENSG0000(	FCER2	2.413753	2.350041	0.004466
ENSG0000(	FAM72A	2.413085	2.174979	0.006684
ENSG0000(	SERPINB9P	2.411156	2.965327	0.001083
ENSG0000(	PNMA8A	2.408621	8.198402	6.33E-09
ENSG0000(	KREMEN2	2.40542	2.526885	0.002972
ENSG0000(	KCNK5	2.405204	4.312851	4.87E-05
ENSG0000(	AC093162.	2.401443	2.377229	0.004195
ENSG0000(	RBP4	2.400154	2.314121	0.004852
ENSG0000(	SIGLEC1	2.399545	15.3831	4.14E-16
ENSG0000(	AC087477.	2.398337	3.852923	0.00014
ENSG0000(	AC098614.	2.397333	3.035089	0.000922
ENSG0000(	CENPK	2.394996	1.748996	0.017824
ENSG0000(	AL451067.:	2.391378	2.558149	0.002766
ENSG0000(	CARMIL2	2.391249	6.384891	4.12E-07
ENSG0000(	SEL1L3	2.389905	9.782479	1.65E-10
ENSG0000(	VASH2	2.389633	9.791863	1.61E-10
ENSG0000(	PTPRC	2.384095	6.211514	6.14E-07
ENSG0000(	MAP7	2.383174	5.838734	1.45E-06
ENSG0000(	FP565260.:	2.380705	1.796168	0.015989
ENSG0000(	ZMYND15	2.379774	3.872306	0.000134
ENSG0000(	AC006141.	2.378484	1.429619	0.037186
ENSG0000(	CFB	2.378064	13.51993	3.02E-14
ENSG0000(	CYBB	2.376198	9.341822	4.55E-10
ENSG0000(	OLIG1	2.375696	4.219586	6.03E-05
ENSG0000(	LAPTM5	2.375668	8.632855	2.33E-09
ENSG0000(	RTP4	2.374662	4.172315	6.72E-05
ENSG0000(	LY75	2.374471	4.931104	1.17E-05
ENSG0000(	TNFAIP8	2.37349	12.16147	6.90E-13
ENSG0000(	IQGAP2	2.372969	9.348489	4.48E-10
ENSG0000(	C3AR1	2.369415	6.269654	5.37E-07
ENSG0000(	CD83	2.368364	9.166819	6.81E-10

ENSG0000(	KCND1	2.367937	3.478807	0.000332
ENSG0000(	VSIG4	2.36772	5.968836	1.07E-06
ENSG0000(	ASPN	2.362518	5.658612	2.19E-06
ENSG0000(	CD209	2.360037	10.50807	3.10E-11
ENSG0000(	LY96	2.358797	4.033008	9.27E-05
ENSG0000(	ARL11	2.358359	2.040024	0.00912
ENSG0000(	AC010186.	2.35728	1.61539	0.024244
ENSG0000(	PCED1B	2.356871	7.760933	1.73E-08
ENSG0000(	OTULINL	2.356325	10.88814	1.29E-11
ENSG0000(	AC016888.	2.356006	12.9974	1.01E-13
ENSG0000(	DCST1	2.354633	2.118272	0.007616
ENSG0000(	CYTIP	2.352837	7.09986	7.95E-08
ENSG0000(	AC245100.	2.351085	3.693848	0.000202
ENSG0000(	CCDC78	2.35049	1.349762	0.044693
ENSG0000(	HELB	2.346506	7.038131	9.16E-08
ENSG0000(	CORO1A	2.346069	7.302977	4.98E-08
ENSG0000(	IL17B	2.343129	1.879872	0.013186
ENSG0000(	JAK3	2.33666	5.98463	1.04E-06
ENSG0000(	ARHGAP30	2.335874	8.86765	1.36E-09
ENSG0000(	CHRNG	2.335281	5.056203	8.79E-06
ENSG0000(	AC099063.	2.332582	9.992511	1.02E-10
ENSG0000(	IPO5P1	2.332029	2.688271	0.00205
ENSG0000(	RF00019	2.331167	1.546118	0.028437
ENSG0000(	HYDIN2	2.330051	1.402611	0.039572
ENSG0000(	AC119403.	2.327826	1.842027	0.014387
ENSG0000(	AC012645.	2.326956	5.913751	1.22E-06
ENSG0000(	TNC	2.326716	13.42405	3.77E-14
ENSG0000(	FAM171A2	2.326128	2.833084	0.001469
ENSG0000(	MIR3615	2.325454	12.70268	1.98E-13
ENSG0000(	NCAM1	2.325326	16.88594	1.30E-17
ENSG0000(	ALOX5AP	2.323974	7.345372	4.51E-08
ENSG0000(	FGD2	2.323241	11.85972	1.38E-12
ENSG0000(	GLUD2	2.322665	1.386113	0.041104
ENSG0000(	CD68	2.321226	11.18247	6.57E-12
ENSG0000(	TWIST1	2.319356	2.371231	0.004254
ENSG0000(	AP000580.	2.31741	1.990548	0.01022
ENSG0000(	GBP4	2.315852	14.20313	6.26E-15
ENSG0000(	SLA	2.314927	4.93395	1.16E-05
ENSG0000(	CYSLTR1	2.314295	4.766145	1.71E-05
ENSG0000(	FMO1	2.313795	1.370108	0.042647
ENSG0000(	ODF3B	2.313117	14.85175	1.41E-15
ENSG0000(	OTOF	2.311852	3.47291	0.000337
ENSG0000(	GPR132	2.305356	8.470263	3.39E-09
ENSG0000(	Z83844.2	2.301995	6.285953	5.18E-07
ENSG0000(	HM13-IT1	2.300829	1.58006	0.026299
ENSG0000(	AC106739.	2.300578	1.538424	0.028945
ENSG0000(	ITGB2	2.300121	7.718021	1.91E-08

ENSG0000(	NEFH	2.300061	2.483428	0.003285
ENSG0000(	HESX1	2.298905	1.351102	0.044555
ENSG0000(	AL022344.:	2.298423	1.627538	0.023576
ENSG0000(	ITGB8	2.297316	2.144482	0.00717
ENSG0000(	SFRP2	2.291795	3.891954	0.000128
ENSG0000(	GPR65	2.28818	4.30212	4.99E-05
ENSG0000(	VAV1	2.286303	4.627271	2.36E-05
ENSG0000(	SCIN	2.285521	2.364107	0.004324
ENSG0000(	TRAM1L1	2.283555	2.157899	0.006952
ENSG0000(	MPEG1	2.281943	8.061618	8.68E-09
ENSG0000(	GNAT2	2.28112	1.748755	0.017834
ENSG0000(	IRF5	2.27833	6.030092	9.33E-07
ENSG0000(	SPN	2.278293	7.078958	8.34E-08
ENSG0000(	HLA-DMA	2.277876	14.32788	4.70E-15
ENSG0000(	CALB2	2.277513	1.441033	0.036222
ENSG0000(	SCNN1B	2.276164	1.418727	0.038131
ENSG0000(	AC004551.	2.266423	3.629738	0.000235
ENSG0000(	NCF1C	2.262729	5.173723	6.70E-06
ENSG0000(	ADA2	2.262724	13.37769	4.19E-14
ENSG0000(	CCR1	2.262508	5.560044	2.75E-06
ENSG0000(	CLDN23	2.259838	3.123192	0.000753
ENSG0000(	C16orf54	2.258455	3.815207	0.000153
ENSG0000(	C16orf89	2.256552	4.048156	8.95E-05
ENSG0000(	C5orf58	2.25642	4.399189	3.99E-05
ENSG0000(	FBP1	2.25521	8.723417	1.89E-09
ENSG0000(	IFI6	2.255108	10.20102	6.29E-11
ENSG0000(	BRIP1	2.250731	1.31372	0.04856
ENSG0000(	TUBA3FP	2.250073	2.236979	0.005795
ENSG0000(	FCER1G	2.249283	5.949569	1.12E-06
ENSG0000(	PSD4	2.24857	9.194123	6.40E-10
ENSG0000(	PSTPIP1	2.247617	8.530075	2.95E-09
ENSG0000(	CCDC169-S	2.24722	2.027584	0.009385
ENSG0000(	PARVG	2.246619	5.936655	1.16E-06
ENSG0000(	AP003031.	2.243557	2.497984	0.003177
ENSG0000(	AC122688.	2.242585	1.463636	0.034385
ENSG0000(	HLA-G	2.237326	4.451562	3.54E-05
ENSG0000(	ISG20	2.234651	6.27712	5.28E-07
ENSG0000(	C6	2.232261	4.33552	4.62E-05
ENSG0000(	GNA15	2.232215	4.780104	1.66E-05
ENSG0000(	MC1R	2.23122	8.160498	6.91E-09
ENSG0000(	PDZK1P1	2.229191	1.479538	0.033148
ENSG0000(	CA13	2.226876	2.37961	0.004172
ENSG0000(	SH3BP1	2.226169	7.15448	7.01E-08
ENSG0000(	AL031595.:	2.22549	4.72479	1.88E-05
ENSG0000(	CABP4	2.224893	8.488324	3.25E-09
ENSG0000(	MICB	2.224137	5.812708	1.54E-06
ENSG0000(	AC138207.	2.223567	4.191471	6.43E-05

ENSG0000(	DENND2D	2.223224	5.550121	2.82E-06
ENSG0000(	IFI27L2	2.218862	12.02813	9.37E-13
ENSG0000(	CTXN3	2.218621	4.203378	6.26E-05
ENSG0000(	AL669918.:	2.217348	17.56968	2.69E-18
ENSG0000(	PPP1R26-A	2.215281	1.765208	0.017171
ENSG0000(	NUDT11	2.21474	1.490109	0.032351
ENSG0000(	IGSF6	2.21421	5.707244	1.96E-06
ENSG0000(	C3orf80	2.213941	1.550285	0.028165
ENSG0000(	CCDC8	2.213041	8.56462	2.73E-09
ENSG0000(	FOLR2	2.212942	7.574751	2.66E-08
ENSG0000(	CTD-2201I:	2.212263	7.99715	1.01E-08
ENSG0000(	CRISPLD1	2.20911	4.073869	8.44E-05
ENSG0000(	PTHLH	2.208797	1.723613	0.018897
ENSG0000(	KIAA1549L	2.208545	2.261981	0.00547
ENSG0000(	LINC01635	2.207155	1.507676	0.031069
ENSG0000(	SYT17	2.207133	2.794927	0.001604
ENSG0000(	BX470102.	2.206207	1.844268	0.014313
ENSG0000(	CYP27C1	2.205586	3.355935	0.000441
ENSG0000(	SUSD3	2.202204	1.557997	0.02767
ENSG0000(	IGSF22	2.202043	3.671178	0.000213
ENSG0000(	TMC8	2.199777	7.585102	2.60E-08
ENSG0000(	ARHGAP36	2.197654	4.308409	4.92E-05
ENSG0000(	NLRP3	2.197617	3.752591	0.000177
ENSG0000(	WDFY4	2.197328	8.614643	2.43E-09
ENSG0000(	LMX1B	2.196639	1.710702	0.019467
ENSG0000(	AC005515.	2.196086	2.558119	0.002766
ENSG0000(	AC099521.	2.195364	1.403265	0.039513
ENSG0000(	BTN2A2	2.195277	13.92293	1.19E-14
ENSG0000(	GPR68	2.193922	8.570285	2.69E-09
ENSG0000(	EPS8L2	2.19365	16.28147	5.23E-17
ENSG0000(	NMUR1	2.192358	1.686813	0.020568
ENSG0000(	MRC1	2.188954	6.199614	6.32E-07
ENSG0000(	RAC2	2.188125	6.444295	3.60E-07
ENSG0000(	AL357060.:	2.181507	6.833754	1.47E-07
ENSG0000(	AC037198.	2.180738	4.468608	3.40E-05
ENSG0000(	OAS1	2.177254	9.204291	6.25E-10
ENSG0000(	BDKRB2	2.174051	6.503095	3.14E-07
ENSG0000(	SCN5A	2.1723	5.639685	2.29E-06
ENSG0000(	CP	2.170017	9.269102	5.38E-10
ENSG0000(	AC010186.	2.16587	2.163843	0.006857
ENSG0000(	AC092111.	2.165697	1.422794	0.037775
ENSG0000(	C11orf45	2.165623	1.405496	0.03931
ENSG0000(	ITGAM	2.16479	9.298418	5.03E-10
ENSG0000(	AL034550.:	2.163311	2.270664	0.005362
ENSG0000(	SLC9A3R1	2.162691	13.47243	3.37E-14
ENSG0000(	AC105020.	2.162044	2.128147	0.007445
ENSG0000(	TLR8	2.160114	2.289249	0.005137

ENSG0000(	CYTH4	2.158666	7.709512	1.95E-08
ENSG0000(	AC138207.	2.158533	2.826346	0.001492
ENSG0000(	NES	2.157576	14.91166	1.23E-15
ENSG0000(	AC090907.	2.153482	4.626107	2.37E-05
ENSG0000(	MLLT11	2.151964	15.74507	1.80E-16
ENSG0000(	SLC22A15	2.151295	4.076599	8.38E-05
ENSG0000(	ARNTL2	2.149957	6.925017	1.19E-07
ENSG0000(	TRAF3IP3	2.149924	7.990324	1.02E-08
ENSG0000(	DEF6	2.149195	6.278526	5.27E-07
ENSG0000(	PDCD1LG2	2.14535	6.65951	2.19E-07
ENSG0000(	AC004846.	2.144309	2.741579	0.001813
ENSG0000(	PIK3AP1	2.143852	4.458186	3.48E-05
ENSG0000(	AL035530.:	2.143695	1.408052	0.039079
ENSG0000(	C9orf139	2.143632	2.473445	0.003362
ENSG0000(	LCP2	2.139786	8.870547	1.35E-09
ENSG0000(	GALNT17	2.139732	7.740326	1.82E-08
ENSG0000(	PRR5-ARH(	2.139658	6.535863	2.91E-07
ENSG0000(	HLA-V	2.138494	3.242712	0.000572
ENSG0000(	PARPBP	2.136944	2.68137	0.002083
ENSG0000(	VENTX	2.134173	3.527016	0.000297
ENSG0000(	S100A2	2.132955	4.38709	4.10E-05
ENSG0000(	ADAP2	2.128559	9.86785	1.36E-10
ENSG0000(	BOK-AS1	2.127497	2.587809	0.002583
ENSG0000(	FSIP1	2.126948	4.515378	3.05E-05
ENSG0000(	YPEL4	2.125451	3.790666	0.000162
ENSG0000(	PTK6	2.12394	2.557387	0.002771
ENSG0000(	LINC01133	2.123513	2.37533	0.004214
ENSG0000(	DLGAP1-AS	2.123456	8.25496	5.56E-09
ENSG0000(	CD163L1	2.118209	6.603229	2.49E-07
ENSG0000(	AC090559.	2.117549	2.32085	0.004777
ENSG0000(	NCF1B	2.117525	2.607741	0.002468
ENSG0000(	NELL2	2.115961	1.964716	0.010846
ENSG0000(	GPBAR1	2.111939	2.360382	0.004361
ENSG0000(	GRAP2	2.111124	2.881785	0.001313
ENSG0000(	LGI2	2.110609	4.861608	1.38E-05
ENSG0000(	KCNAB2	2.1094	11.74088	1.82E-12
ENSG0000(	CD33	2.10787	6.000909	9.98E-07
ENSG0000(	AL645929.:	2.107126	2.31629	0.004827
ENSG0000(	SUSD4	2.106556	5.424018	3.77E-06
ENSG0000(	ABCC3	2.102881	13.26301	5.46E-14
ENSG0000(	AP000915.	2.101151	2.951954	0.001117
ENSG0000(	PTPRF	2.099038	6.609278	2.46E-07
ENSG0000(	KCNC1	2.097181	3.698798	0.0002
ENSG0000(	KLB	2.095932	1.377197	0.041957
ENSG0000(	AC064836.	2.095756	2.879592	0.001319
ENSG0000(	PIK3R5	2.094387	5.861161	1.38E-06
ENSG0000(	LRRC17	2.093632	8.528476	2.96E-09

ENSG0000(	CD14	2.093169	7.693667	2.02E-08
ENSG0000(	C3orf52	2.093006	2.541141	0.002876
ENSG0000(	GPR34	2.091399	3.23374	0.000584
ENSG0000(	SLC41A2	2.090427	7.967738	1.08E-08
ENSG0000(	IRF9	2.090113	17.64192	2.28E-18
ENSG0000(	IFI27	2.088097	9.232735	5.85E-10
ENSG0000(	SCD	2.08742	2.560717	0.00275
ENSG0000(	SECTM1	2.08703	7.896581	1.27E-08
ENSG0000(	SDSL	2.08701	5.048485	8.94E-06
ENSG0000(	JHY	2.08636	2.152371	0.007041
ENSG0000(	THEMIS2	2.083819	8.358189	4.38E-09
ENSG0000(	STAT4	2.081244	6.98082	1.05E-07
ENSG0000(	RUNX1	2.079532	14.7032	1.98E-15
ENSG0000(	ANO7L1	2.078003	3.158295	0.000695
ENSG0000(	ARAP2	2.077497	9.153946	7.02E-10
ENSG0000(	FAM86EP	2.07672	2.435245	0.003671
ENSG0000(	TAP2	2.076676	16.23482	5.82E-17
ENSG0000(	PRSS27	2.075891	1.773735	0.016837
ENSG0000(	KLHL6	2.075407	4.078367	8.35E-05
ENSG0000(	CRIP1	2.074871	7.496141	3.19E-08
ENSG0000(	LILRB5	2.073683	10.20102	6.29E-11
ENSG0000(	AL512306.:	2.072594	2.335709	0.004616
ENSG0000(	C1R	2.071746	11.10599	7.83E-12
ENSG0000(	AC073332.	2.071345	1.647436	0.02252
ENSG0000(	ARHGAP15	2.065751	4.428283	3.73E-05
ENSG0000(	ID2	2.064972	19.77569	1.68E-20
ENSG0000(	GRIN2D	2.062388	1.487236	0.032566
ENSG0000(	NRCAM	2.062258	4.889736	1.29E-05
ENSG0000(	TMEM236	2.061921	1.693614	0.020248
ENSG0000(	AC011450.	2.06105	4.12051	7.58E-05
ENSG0000(	TACR1	2.060724	2.971572	0.001068
ENSG0000(	ABRACL	2.05986	6.225714	5.95E-07
ENSG0000(	CLSTN3	2.059705	19.28113	5.23E-20
ENSG0000(	SLC17A9	2.055392	7.543051	2.86E-08
ENSG0000(	CCRL2	2.052255	3.603789	0.000249
ENSG0000(	ADAMTS14	2.050756	3.141396	0.000722
ENSG0000(	MXRA5	2.050588	7.368381	4.28E-08
ENSG0000(	HCLS1	2.048685	8.288902	5.14E-09
ENSG0000(	APOBEC3F	2.048672	9.558668	2.76E-10
ENSG0000(	FOS	2.046546	2.825149	0.001496
ENSG0000(	LBX2	2.045424	2.820804	0.001511
ENSG0000(	HCG4B	2.044293	3.346223	0.000451
ENSG0000(	AC109361.	2.043234	2.558174	0.002766
ENSG0000(	AF131215.:	2.037985	4.529363	2.96E-05
ENSG0000(	AC006111.	2.037629	1.452038	0.035315
ENSG0000(	TMEM156	2.037527	2.07003	0.008511
ENSG0000(	AC063949.	2.036038	2.30596	0.004944

ENSG0000(	SLC44A3	2.035919	1.570933	0.026858
ENSG0000(	THEM5	2.035753	1.863477	0.013694
ENSG0000(	CD44	2.034849	15.14004	7.24E-16
ENSG0000(	NFKBIE	2.03348	11.94683	1.13E-12
ENSG0000(	RUBCNL	2.033112	3.381672	0.000415
ENSG0000(	C4orf48	2.032109	3.487804	0.000325
ENSG0000(	TMEM176I	2.031084	10.19616	6.37E-11
ENSG0000(	CD247	2.027869	4.511376	3.08E-05
ENSG0000(	SNX7	2.025453	10.20956	6.17E-11
ENSG0000(	PARP9	2.024867	16.91342	1.22E-17
ENSG0000(	RASSF5	2.023862	9.253742	5.58E-10
ENSG0000(	GCH1	2.020688	11.66551	2.16E-12
ENSG0000(	CDHR1	2.019856	1.526239	0.029769
ENSG0000(	LINC01410	2.018815	1.795546	0.016012
ENSG0000(	PTTG1	2.015384	3.774656	0.000168
ENSG0000(	TSSK6	2.014807	3.005363	0.000988
ENSG0000(	ASPHD2	2.012869	3.683232	0.000207
ENSG0000(	PARP14	2.010761	13.21052	6.16E-14
ENSG0000(	SELPLG	2.010172	4.899943	1.26E-05
ENSG0000(	AL928654.:	2.008977	7.584264	2.60E-08
ENSG0000(	PCDHB11	2.004989	2.482054	0.003296
ENSG0000(	C14orf132	2.003979	11.6143	2.43E-12
ENSG0000(	FNDC4	2.002594	6.155077	7.00E-07
ENSG0000(	HDX	2.002586	3.189246	0.000647
ENSG0000(	PLCXD2	1.998425	3.897954	0.000126
ENSG0000(	FAM111B	1.997311	1.524435	0.029893
ENSG0000(	LRFN1	1.993003	3.929921	0.000118
ENSG0000(	FCGR2B	1.992582	4.306267	4.94E-05
ENSG0000(	JUNB	1.99226	8.1328	7.37E-09
ENSG0000(	AC008649.	1.991222	5.661079	2.18E-06
ENSG0000(	SYTL1	1.989753	3.837872	0.000145
ENSG0000(	LRRC4C	1.989474	4.454666	3.51E-05
ENSG0000(	CACNB4	1.986918	3.390956	0.000406
ENSG0000(	APOE	1.986467	9.300102	5.01E-10
ENSG0000(	C3	1.985712	10.36694	4.30E-11
ENSG0000(	AC093063.	1.984625	1.744049	0.018028
ENSG0000(	TMSB4XP8	1.980828	1.994611	0.010125
ENSG0000(	C7	1.979746	4.037145	9.18E-05
ENSG0000(	CYP3A5	1.977572	2.249728	0.005627
ENSG0000(	IGSF10	1.974877	4.590113	2.57E-05
ENSG0000(	SIGLEC9	1.974463	1.461178	0.03458
ENSG0000(	AC092958.	1.97232	1.300085	0.050109
ENSG0000(	THBS1	1.971684	6.308053	4.92E-07
ENSG0000(	HSPA7	1.970924	6.297173	5.04E-07
ENSG0000(	PRR5	1.970593	8.01158	9.74E-09
ENSG0000(	FOSB	1.970516	2.298932	0.005024
ENSG0000(	AC135048.	1.970371	19.49026	3.23E-20

ENSG0000(	AC010319.	1.968816	2.48053	0.003307
ENSG0000(	RAB20	1.96822	9.897218	1.27E-10
ENSG0000(	ELOVL7	1.967755	2.101162	0.007922
ENSG0000(	PIK3CG	1.965554	2.869356	0.001351
ENSG0000(	AC008543.	1.964061	1.343448	0.045347
ENSG0000(	L3MBTL4	1.959322	7.959567	1.10E-08
ENSG0000(	TBX19	1.958408	3.664985	0.000216
ENSG0000(	WSCD2	1.958138	1.764829	0.017186
ENSG0000(	HSH2D	1.95743	2.184095	0.006545
ENSG0000(	ADAM28	1.957318	2.375184	0.004215
ENSG0000(	DBH	1.956564	1.797667	0.015934
ENSG0000(	THBS4	1.955447	6.967761	1.08E-07
ENSG0000(	PIEZO2	1.953252	1.689444	0.020444
ENSG0000(	PARP8	1.953228	8.928334	1.18E-09
ENSG0000(	FKBP11	1.952867	9.408576	3.90E-10
ENSG0000(	C6orf132	1.950325	2.793324	0.001609
ENSG0000(	DENND5B-.	1.949873	2.179222	0.006619
ENSG0000(	MUSK	1.949582	4.035748	9.21E-05
ENSG0000(	AKNA	1.948763	9.526742	2.97E-10
ENSG0000(	PYCARD	1.947495	8.71705	1.92E-09
ENSG0000(	APOC1	1.946714	5.836549	1.46E-06
ENSG0000(	SYK	1.946358	5.844493	1.43E-06
ENSG0000(	AC010969.	1.946057	2.245588	0.005681
ENSG0000(	SLCO2B1	1.945498	10.19413	6.40E-11
ENSG0000(	GPR137B	1.945335	7.80931	1.55E-08
ENSG0000(	NFKBID	1.944978	6.154981	7.00E-07
ENSG0000(	AC060234.	1.944051	2.224609	0.005962
ENSG0000(	AC011481.	1.943943	6.925923	1.19E-07
ENSG0000(	TBXAS1	1.943854	5.832281	1.47E-06
ENSG0000(	AC103858.	1.943146	4.635351	2.32E-05
ENSG0000(	TWIST2	1.943137	1.629427	0.023473
ENSG0000(	VWA2	1.943137	1.94963	0.01123
ENSG0000(	RANBP17	1.939678	1.371786	0.042483
ENSG0000(	DOCK10	1.938728	8.510494	3.09E-09
ENSG0000(	CYP26B1	1.938081	7.739113	1.82E-08
ENSG0000(	AC008105.	1.935287	8.723634	1.89E-09
ENSG0000(	HSD3B7	1.933243	20.56477	2.72E-21
ENSG0000(	MGP	1.932369	6.754741	1.76E-07
ENSG0000(	PIPOX	1.932027	1.760177	0.017371
ENSG0000(	EDA2R	1.931925	6.64338	2.27E-07
ENSG0000(	BUB1B	1.931723	1.968646	0.010749
ENSG0000(	PRKAR2B	1.931172	4.08534	8.22E-05
ENSG0000(	TMEM176/	1.931094	11.59666	2.53E-12
ENSG0000(	HOXB5	1.931027	1.414497	0.038504
ENSG0000(	AC003072.	1.930633	1.57553	0.026575
ENSG0000(	EEF1A1P6	1.930595	3.777527	0.000167
ENSG0000(	TM4SF19-/	1.928288	2.254813	0.005561

ENSG0000(	SLC6A16	1.92437	2.395586	0.004022
ENSG0000(	SNORA44	1.923556	1.503667	0.031357
ENSG0000(	C1S	1.921592	11.34234	4.55E-12
ENSG0000(	AC066613.	1.920758	4.874048	1.34E-05
ENSG0000(	IGSF1	1.920689	3.545611	0.000285
ENSG0000(	LINC00861	1.917251	1.464241	0.034337
ENSG0000(	CR559946.	1.917186	1.579717	0.02632
ENSG0000(	SLC37A2	1.916056	8.567775	2.71E-09
ENSG0000(	MREG	1.914998	7.249227	5.63E-08
ENSG0000(	PHOSPHO1	1.910021	8.490997	3.23E-09
ENSG0000(	AL359076.:	1.909943	1.498934	0.0317
ENSG0000(	FGF7	1.909367	7.800727	1.58E-08
ENSG0000(	C2CD4D	1.906682	1.445445	0.035855
ENSG0000(	PTPRE	1.906278	10.14831	7.11E-11
ENSG0000(	IFI35	1.905836	13.63389	2.32E-14
ENSG0000(	RBM47	1.905299	5.079396	8.33E-06
ENSG0000(	SLC7A7	1.904396	8.870209	1.35E-09
ENSG0000(	NFAM1	1.90389	2.783572	0.001646
ENSG0000(	TNFAIP6	1.901576	4.878808	1.32E-05
ENSG0000(	NEU4	1.895431	2.06127	0.008684
ENSG0000(	LINC01315	1.894453	1.820232	0.015128
ENSG0000(	FBXL16	1.893855	2.187837	0.006489
ENSG0000(	GNGT2	1.892867	2.575081	0.00266
ENSG0000(	ZNF793-AS	1.892441	1.446767	0.035746
ENSG0000(	CAMK4	1.891912	2.145982	0.007145
ENSG0000(	HERC5	1.891222	13.91753	1.21E-14
ENSG0000(	CTSC	1.888737	8.654486	2.22E-09
ENSG0000(	RGL1	1.887496	9.657285	2.20E-10
ENSG0000(	LINC00623	1.886787	7.371966	4.25E-08
ENSG0000(	GBP3	1.886093	7.488645	3.25E-08
ENSG0000(	TRIM69	1.884473	12.01243	9.72E-13
ENSG0000(	NCF1	1.883956	4.037034	9.18E-05
ENSG0000(	SLC4A8	1.881782	2.299926	0.005013
ENSG0000(	AL161785.:	1.881591	1.481633	0.032989
ENSG0000(	PERP	1.880929	9.992511	1.02E-10
ENSG0000(	SLAMF8	1.879591	8.824704	1.50E-09
ENSG0000(	CHST11	1.875061	9.469124	3.40E-10
ENSG0000(	SRRM3	1.874872	4.49597	3.19E-05
ENSG0000(	L1CAM	1.874666	12.70967	1.95E-13
ENSG0000(	HLA-J	1.872889	3.457522	0.000349
ENSG0000(	CD44-AS1	1.872625	9.35013	4.47E-10
ENSG0000(	CDT1	1.872341	2.952027	0.001117
ENSG0000(	CSF1R	1.872146	6.999272	1.00E-07
ENSG0000(	PYCARD-AS	1.872129	8.364643	4.32E-09
ENSG0000(	SOX11	1.871317	5.030002	9.33E-06
ENSG0000(	PTAFR	1.870072	5.711083	1.94E-06
ENSG0000(	CMKLR1	1.868409	10.46157	3.45E-11

ENSG0000(	RARRES1	1.867829	4.516651	3.04E-05
ENSG0000(	SERPINB8	1.865198	7.738612	1.83E-08
ENSG0000(	PRF1	1.864808	5.39379	4.04E-06
ENSG0000(	AP003396.	1.864317	5.204986	6.24E-06
ENSG0000(	B3GNT7	1.862617	2.214086	0.006108
ENSG0000(	AC023906.	1.862492	3.855025	0.00014
ENSG0000(	S100B	1.862119	2.17673	0.006657
ENSG0000(	TRIM38	1.862071	19.89954	1.26E-20
ENSG0000(	CR1	1.861613	2.242011	0.005728
ENSG0000(	C12orf60	1.858666	6.367861	4.29E-07
ENSG0000(	EMILIN2	1.857982	6.638663	2.30E-07
ENSG0000(	RGS17	1.857552	1.304685	0.049581
ENSG0000(	APBB1IP	1.857273	6.472064	3.37E-07
ENSG0000(	CTSK	1.856407	5.227665	5.92E-06
ENSG0000(	FLJ46906	1.856034	1.732593	0.01851
ENSG0000(	SLC22A18	1.855744	7.692775	2.03E-08
ENSG0000(	DUSP5	1.855057	6.308801	4.91E-07
ENSG0000(	WAS	1.854333	4.499418	3.17E-05
ENSG0000(	GRIN3A	1.852616	2.232246	0.005858
ENSG0000(	LGALS3BP	1.851966	18.03241	9.28E-19
ENSG0000(	AC066613.	1.850607	2.736484	0.001834
ENSG0000(	TENT5B	1.849393	2.15084	0.007066
ENSG0000(	COTL1	1.8484	9.170128	6.76E-10
ENSG0000(	AL117350.:	1.847123	1.500993	0.031551
ENSG0000(	SLC29A3	1.844038	7.230815	5.88E-08
ENSG0000(	TNFAIP8L2	1.843009	2.375784	0.004209
ENSG0000(	LINC01943	1.842448	7.69917	2.00E-08
ENSG0000(	TMEM37	1.837853	5.173973	6.70E-06
ENSG0000(	CENPW	1.836223	2.735709	0.001838
ENSG0000(	GPRIN1	1.835587	1.616526	0.024181
ENSG0000(	AC010864.	1.834868	1.386977	0.041023
ENSG0000(	AEBP1	1.834746	5.969003	1.07E-06
ENSG0000(	TMEM169	1.833416	3.165841	0.000683
ENSG0000(	BOK	1.833383	5.230841	5.88E-06
ENSG0000(	C11orf21	1.833363	3.029419	0.000935
ENSG0000(	GALNT6	1.833176	5.924845	1.19E-06
ENSG0000(	CENPE	1.831417	1.81902	0.01517
ENSG0000(	AL355581.:	1.831258	5.424654	3.76E-06
ENSG0000(	MIR7703	1.82868	12.52663	2.97E-13
ENSG0000(	LMO3	1.828389	3.833308	0.000147
ENSG0000(	KIF21B	1.826487	8.91408	1.22E-09
ENSG0000(	TSPAN32	1.826463	2.458037	0.003483
ENSG0000(	WNT10B	1.824536	2.737415	0.001831
ENSG0000(	PITPNM1	1.822952	12.4909	3.23E-13
ENSG0000(	VSIG10L2	1.821652	6.566035	2.72E-07
ENSG0000(	VSTM2L	1.821136	5.624224	2.38E-06
ENSG0000(	RAB11FIP4	1.821126	4.432076	3.70E-05

ENSG0000(	ATP8B4	1.819878	7.312274	4.87E-08
ENSG0000(	OGN	1.819514	5.479865	3.31E-06
ENSG0000(	ISG15	1.819133	8.479011	3.32E-09
ENSG0000(	PTPN6	1.816874	6.571153	2.68E-07
ENSG0000(	CCND2	1.81564	17.1654	6.83E-18
ENSG0000(	AL138756.:	1.814008	4.173663	6.70E-05
ENSG0000(	IFITM10	1.813534	5.124015	7.52E-06
ENSG0000(	AC092111.	1.812574	1.724731	0.018848
ENSG0000(	PTGER3	1.810999	1.437204	0.036542
ENSG0000(	CCDC88B	1.809114	6.411372	3.88E-07
ENSG0000(	HLA-H	1.809039	4.097464	7.99E-05
ENSG0000(	LINC00844	1.808554	2.003067	0.00993
ENSG0000(	PCOLCE2	1.808391	2.69336	0.002026
ENSG0000(	PARP12	1.805315	16.85359	1.40E-17
ENSG0000(	THY1	1.80444	4.791825	1.62E-05
ENSG0000(	AL049829.:	1.804284	1.408756	0.039016
ENSG0000(	ELAVL2	1.804178	1.665656	0.021595
ENSG0000(	GDF15	1.802782	1.331572	0.046605
ENSG0000(	FCGR2C	1.802282	4.197135	6.35E-05
ENSG0000(	SLC9A9	1.802234	9.449059	3.56E-10
ENSG0000(	HS3ST3B1	1.80211	3.937111	0.000116
ENSG0000(	XRCC4	1.800294	9.392362	4.05E-10
ENSG0000(	AC008105.	1.797984	4.390399	4.07E-05
ENSG0000(	PLCB2	1.797369	5.760986	1.73E-06
ENSG0000(	S100A4	1.796941	9.701052	1.99E-10
ENSG0000(	AC100861.	1.795685	2.043409	0.009049
ENSG0000(	APOL2	1.795551	15.86326	1.37E-16
ENSG0000(	TNFRSF11A	1.794285	3.086698	0.000819
ENSG0000(	C1RL	1.791368	9.71399	1.93E-10
ENSG0000(	CLEC7A	1.790284	2.385215	0.004119
ENSG0000(	EPHB2	1.788928	10.29565	5.06E-11
ENSG0000(	PRDM1	1.787416	7.014161	9.68E-08
ENSG0000(	BIN2	1.787025	3.736743	0.000183
ENSG0000(	MTHFD1L	1.7863	6.894526	1.27E-07
ENSG0000(	Z93930.2	1.786133	2.070457	0.008502
ENSG0000(	STAP2	1.785447	3.918945	0.000121
ENSG0000(	SLC15A3	1.78544	11.10633	7.83E-12
ENSG0000(	TIMP1	1.785011	9.980417	1.05E-10
ENSG0000(	TMEM178I	1.781999	8.404047	3.94E-09
ENSG0000(	TMEM40	1.780854	2.678779	0.002095
ENSG0000(	TSHZ2	1.779542	10.14028	7.24E-11
ENSG0000(	LILRB2	1.778046	4.202894	6.27E-05
ENSG0000(	TRIM14	1.776659	12.03013	9.33E-13
ENSG0000(	FMOD	1.776209	3.457295	0.000349
ENSG0000(	NMI	1.773218	11.80815	1.56E-12
ENSG0000(	AC084876.	1.772447	2.837233	0.001455
ENSG0000(	OSCAR	1.772163	1.763405	0.017242

ENSG0000(	AP3B2	1.771455	2.126278	0.007477
ENSG0000(	FGL2	1.771023	6.880833	1.32E-07
ENSG0000(	AL135818.:	1.770567	2.144869	0.007164
ENSG0000(	SIK1B	1.769065	2.389999	0.004074
ENSG0000(	ZAP70	1.768681	6.011103	9.75E-07
ENSG0000(	IQGAP3	1.768327	2.268333	0.005391
ENSG0000(	STK17B	1.766242	8.768376	1.70E-09
ENSG0000(	GGTA1P	1.763478	5.740133	1.82E-06
ENSG0000(	FAM24B	1.761614	1.984053	0.010374
ENSG0000(	RELL2	1.761507	6.918274	1.21E-07
ENSG0000(	ADGRG2	1.759885	2.355138	0.004414
ENSG0000(	S100A6	1.759835	13.73976	1.82E-14
ENSG0000(	RPL23AP1	1.759103	9.005503	9.87E-10
ENSG0000(	CCDC89	1.758431	1.373626	0.042303
ENSG0000(	PLD4	1.757206	4.924528	1.19E-05
ENSG0000(	SCO2	1.756957	12.70133	1.99E-13
ENSG0000(	CFH	1.755892	6.291258	5.11E-07
ENSG0000(	P2RY14	1.755873	5.541115	2.88E-06
ENSG0000(	RENBP	1.755602	5.812756	1.54E-06
ENSG0000(	HIST3H2A	1.754286	4.179463	6.62E-05
ENSG0000(	EMP3	1.753878	7.888988	1.29E-08
ENSG0000(	LILRA6	1.753704	2.553662	0.002795
ENSG0000(	P3H2	1.753163	7.372511	4.24E-08
ENSG0000(	SHTN1	1.75237	7.583014	2.61E-08
ENSG0000(	MIR4709	1.751611	8.38834	4.09E-09
ENSG0000(	FBXO41	1.750054	6.645124	2.26E-07
ENSG0000(	CFP	1.749883	3.518996	0.000303
ENSG0000(	LEP	1.74926	1.423045	0.037753
ENSG0000(	CCDC169	1.748776	1.774745	0.016798
ENSG0000(	TTC39C	1.747536	10.33137	4.66E-11
ENSG0000(	GPR153	1.747232	12.76442	1.72E-13
ENSG0000(	AC007728.	1.746936	10.2209	6.01E-11
ENSG0000(	RPLP1P6	1.746125	1.388783	0.040852
ENSG0000(	BISPR	1.746043	6.107419	7.81E-07
ENSG0000(	ATF3	1.745443	5.460794	3.46E-06
ENSG0000(	ADRA2A	1.744356	2.415505	0.003841
ENSG0000(	AL353586.:	1.742468	3.357397	0.000439
ENSG0000(	PTGES	1.738771	4.175329	6.68E-05
ENSG0000(	NCF2	1.736144	3.184722	0.000654
ENSG0000(	MOCOS	1.73418	2.539473	0.002888
ENSG0000(	CXCR4	1.732502	4.7863	1.64E-05
ENSG0000(	DPP4	1.731414	3.641768	0.000228
ENSG0000(	CDKN1A	1.730471	6.586513	2.59E-07
ENSG0000(	FMNL1	1.729714	7.64642	2.26E-08
ENSG0000(	TNFRSF21	1.729286	11.56534	2.72E-12
ENSG0000(	PCK2	1.728139	9.352785	4.44E-10
ENSG0000(	SCAMP5	1.726465	5.598898	2.52E-06

ENSG0000(	FER1L4	1.726099	2.860897	0.001378
ENSG0000(	AC005332.	1.725921	10.83748	1.45E-11
ENSG0000(	AC103925.	1.725349	1.603888	0.024895
ENSG0000(	AC068587.	1.725063	1.318719	0.048004
ENSG0000(	GABRE	1.724511	3.819367	0.000152
ENSG0000(	GRK3	1.7231	7.787997	1.63E-08
ENSG0000(	NEK8	1.721295	4.53035	2.95E-05
ENSG0000(	AC005779.	1.720706	1.567389	0.027078
ENSG0000(	HDAC2-AS2	1.719246	1.921798	0.011973
ENSG0000(	CARD16	1.717724	5.843213	1.43E-06
ENSG0000(	P2RX4	1.717719	8.439796	3.63E-09
ENSG0000(	NTM	1.716926	4.315537	4.84E-05
ENSG0000(	NPNT	1.716495	8.912697	1.22E-09
ENSG0000(	CHODL	1.714932	1.656224	0.022069
ENSG0000(	CASP8	1.713475	9.697244	2.01E-10
ENSG0000(	AC254633.	1.711406	1.534944	0.029178
ENSG0000(	GPNMB	1.711354	6.412626	3.87E-07
ENSG0000(	PLA2G4A	1.710945	3.771706	0.000169
ENSG0000(	HLA-F-AS1	1.71011	11.20322	6.26E-12
ENSG0000(	SRC	1.707247	13.4921	3.22E-14
ENSG0000(	RPS10P7	1.704808	3.721156	0.00019
ENSG0000(	FAS	1.704177	12.03963	9.13E-13
ENSG0000(	SPIRE2	1.703667	3.229901	0.000589
ENSG0000(	CD7	1.702673	2.118272	0.007616
ENSG0000(	FNDC1	1.702073	3.802395	0.000158
ENSG0000(	AC093673.	1.701399	7.48161	3.30E-08
ENSG0000(	TPPP3	1.700184	5.969003	1.07E-06
ENSG0000(	HSD17B14	1.699591	4.355903	4.41E-05
ENSG0000(	CHRDL1	1.696834	5.008808	9.80E-06
ENSG0000(	1-Mar	1.696488	6.603065	2.49E-07
ENSG0000(	AC125611.	1.695949	18.44721	3.57E-19
ENSG0000(	CCDC200	1.69521	4.558938	2.76E-05
ENSG0000(	DOCK8	1.69501	7.365517	4.31E-08
ENSG0000(	TNFSF9	1.694189	1.551113	0.028112
ENSG0000(	KCNC4	1.693485	11.24179	5.73E-12
ENSG0000(	BEND6	1.691109	2.588645	0.002578
ENSG0000(	WDR76	1.690244	3.753936	0.000176
ENSG0000(	TGFB3-AS1	1.687791	4.000483	9.99E-05
ENSG0000(	SOCS3	1.687637	5.274408	5.32E-06
ENSG0000(	AL590385.:	1.685736	3.226709	0.000593
ENSG0000(	STXBP2	1.685017	5.068165	8.55E-06
ENSG0000(	SIRPB2	1.684674	2.210557	0.006158
ENSG0000(	HUNK	1.683815	2.283881	0.005201
ENSG0000(	IFIT2	1.683619	9.448789	3.56E-10
ENSG0000(	UNC93B1	1.682948	10.32415	4.74E-11
ENSG0000(	LINC01138	1.680521	5.397865	4.00E-06
ENSG0000(	ARL4D	1.679977	9.784141	1.64E-10

ENSG0000(	PAQR8	1.679355	9.10906	7.78E-10
ENSG0000(	XG	1.679338	4.790886	1.62E-05
ENSG0000(	TMC6	1.679011	12.5491	2.82E-13
ENSG0000(	TNFAIP2	1.678768	15.23068	5.88E-16
ENSG0000(	CASP1	1.678543	7.780356	1.66E-08
ENSG0000(	CHL1	1.678278	2.896677	0.001269
ENSG0000(	LINC02397	1.677635	2.970431	0.00107
ENSG0000(	RPS6KA1	1.677585	5.751145	1.77E-06
ENSG0000(	CXCL16	1.677127	9.968287	1.08E-10
ENSG0000(	HAPLN3	1.676448	8.579521	2.63E-09
ENSG0000(	AC092807.	1.675819	1.925921	0.01186
ENSG0000(	SERPINA1	1.675662	2.026577	0.009406
ENSG0000(	TMEM132(	1.675341	3.213693	0.000611
ENSG0000(	LRRC37A4F	1.675285	4.836016	1.46E-05
ENSG0000(	BX470102.	1.675213	2.368431	0.004281
ENSG0000(	ZNF57	1.673465	3.120239	0.000758
ENSG0000(	1-Sep	1.673266	6.271319	5.35E-07
ENSG0000(	A1BG	1.673216	8.526035	2.98E-09
ENSG0000(	CTSZ	1.673171	12.64615	2.26E-13
ENSG0000(	WIPF1	1.672117	8.982131	1.04E-09
ENSG0000(	MAMDC2	1.671156	5.936715	1.16E-06
ENSG0000(	TAGAP	1.670938	3.488154	0.000325
ENSG0000(	KCTD17	1.670897	10.80906	1.55E-11
ENSG0000(	PRR5L	1.670247	8.595168	2.54E-09
ENSG0000(	DRAM1	1.670108	11.31606	4.83E-12
ENSG0000(	FAM180B	1.669135	3.481086	0.00033
ENSG0000(	FGF9	1.668963	3.205673	0.000623
ENSG0000(	LGMN	1.667861	12.92972	1.18E-13
ENSG0000(	CYTOR	1.666794	10.23481	5.82E-11
ENSG0000(	AC133065.	1.666374	7.527473	2.97E-08
ENSG0000(	AP002026.	1.665814	2.357903	0.004386
ENSG0000(	PLTP	1.665072	7.205412	6.23E-08
ENSG0000(	SLC7A11	1.664872	1.644043	0.022696
ENSG0000(	HRH1	1.664402	5.454991	3.51E-06
ENSG0000(	HPGDS	1.663269	2.884668	0.001304
ENSG0000(	DTX3L	1.662558	15.7179	1.91E-16
ENSG0000(	POU2F2	1.662525	3.754983	0.000176
ENSG0000(	PTGIR	1.662291	5.835533	1.46E-06
ENSG0000(	MS4A4E	1.661424	3.533398	0.000293
ENSG0000(	PANX1	1.660626	11.15398	7.01E-12
ENSG0000(	NECAB1	1.659158	1.70944	0.019524
ENSG0000(	GSN-AS1	1.658264	11.53484	2.92E-12
ENSG0000(	AC008105.	1.656384	6.286735	5.17E-07
ENSG0000(	CLCF1	1.655533	5.776941	1.67E-06
ENSG0000(	ARHGAP45	1.654923	7.940286	1.15E-08
ENSG0000(	CGAS	1.653469	4.293409	5.09E-05
ENSG0000(	ERAP2	1.653091	2.716387	0.001921

ENSG0000(	MCUB	1.652416	7.813104	1.54E-08
ENSG0000(	BTN2A3P	1.651064	7.613606	2.43E-08
ENSG0000(	GMFG	1.649392	4.374675	4.22E-05
ENSG0000(	CRHBP	1.645435	1.570933	0.026858
ENSG0000(	PRSS12	1.64453	2.403234	0.003952
ENSG0000(	PDE3B	1.644199	2.805991	0.001563
ENSG0000(	CEP55	1.642532	1.578356	0.026402
ENSG0000(	SP100	1.642353	24.19913	6.32E-25
ENSG0000(	GDF11	1.641952	8.804218	1.57E-09
ENSG0000(	MGST1	1.64164	2.850578	0.001411
ENSG0000(	SERPINE1	1.640134	4.320054	4.79E-05
ENSG0000(	MYOF	1.639377	12.58602	2.59E-13
ENSG0000(	MIR7846	1.639087	2.987517	0.001029
ENSG0000(	SP140L	1.639013	22.648	2.25E-23
ENSG0000(	AC007991.	1.638907	1.455465	0.035038
ENSG0000(	CPVL	1.638525	6.056429	8.78E-07
ENSG0000(	KLHL35	1.637756	2.990479	0.001022
ENSG0000(	IDO1	1.637668	2.985948	0.001033
ENSG0000(	TUBA1C	1.633335	24.12745	7.46E-25
ENSG0000(	PAX8	1.633097	2.393113	0.004045
ENSG0000(	CYBA	1.631979	8.337619	4.60E-09
ENSG0000(	CCDC96	1.63102	2.024197	0.009458
ENSG0000(	ELL3	1.630235	10.41513	3.84E-11
ENSG0000(	AC011611.	1.62966	9.967674	1.08E-10
ENSG0000(	LIPA	1.627566	11.55427	2.79E-12
ENSG0000(	SERPINA3	1.626314	2.910722	0.001228
ENSG0000(	SARDH	1.626148	4.59933	2.52E-05
ENSG0000(	ADGRB1	1.625569	1.756993	0.017499
ENSG0000(	RSAD2	1.623895	7.02822	9.37E-08
ENSG0000(	LINC00869	1.623505	11.24127	5.74E-12
ENSG0000(	ANXA1	1.622978	7.12963	7.42E-08
ENSG0000(	PKD1L2	1.622303	3.524731	0.000299
ENSG0000(	FCGBP	1.62104	1.941024	0.011455
ENSG0000(	TRAFD1	1.620782	14.37559	4.21E-15
ENSG0000(	CLEC4A	1.619904	1.690758	0.020382
ENSG0000(	MICALL2	1.619113	10.33137	4.66E-11
ENSG0000(	TLR3	1.618817	8.637824	2.30E-09
ENSG0000(	EFEMP1	1.617592	5.334449	4.63E-06
ENSG0000(	TRIM21	1.616774	11.91784	1.21E-12
ENSG0000(	LAT	1.616636	7.297212	5.04E-08
ENSG0000(	AC012181.	1.616275	2.754764	0.001759
ENSG0000(	GADD45A	1.615849	8.912697	1.22E-09
ENSG0000(	ATP10A	1.615615	9.813055	1.54E-10
ENSG0000(	U52112.1	1.61449	8.897094	1.27E-09
ENSG0000(	AC009041.	1.612899	7.464488	3.43E-08
ENSG0000(	PROCA1	1.611393	3.546467	0.000284
ENSG0000(	PTPRJ	1.611213	6.878307	1.32E-07

ENSG0000(	APOL3	1.610878	10.61996	2.40E-11
ENSG0000(	FAM107B	1.610536	8.39432	4.03E-09
ENSG0000(	GIMAP2	1.609896	7.150874	7.07E-08
ENSG0000(	IFIH1	1.609196	9.558668	2.76E-10
ENSG0000(	AC090617.	1.608881	1.446767	0.035746
ENSG0000(	CPM	1.608344	12.4379	3.65E-13
ENSG0000(	LINC01145	1.60723	4.325801	4.72E-05
ENSG0000(	AL159163.:	1.607165	7.875206	1.33E-08
ENSG0000(	MICE	1.605659	1.523732	0.029941
ENSG0000(	SPI1	1.604397	4.593174	2.55E-05
ENSG0000(	RAB31	1.603682	7.149522	7.09E-08
ENSG0000(	LPXN	1.601106	9.642837	2.28E-10
ENSG0000(	NT5E	1.600718	4.817707	1.52E-05
ENSG0000(	CCDC88C	1.600409	13.25332	5.58E-14
ENSG0000(	NPL	1.599775	7.592957	2.55E-08
ENSG0000(	C3orf14	1.599469	3.955691	0.000111
ENSG0000(	DDN-AS1	1.598595	5.883997	1.31E-06
ENSG0000(	OGFR-AS1	1.597884	1.826852	0.014899
ENSG0000(	CASP4	1.597839	8.549003	2.82E-09
ENSG0000(	XPNPEP2	1.596899	3.109251	0.000778
ENSG0000(	PVALB	1.595273	1.556229	0.027782
ENSG0000(	TMEM119	1.595154	6.927934	1.18E-07
ENSG0000(	FCGR2A	1.594738	2.982067	0.001042
ENSG0000(	TMEM136	1.593537	7.158745	6.94E-08
ENSG0000(	RAP2B	1.593163	14.33503	4.62E-15
ENSG0000(	RPS6KA6	1.591942	2.278523	0.005266
ENSG0000(	CSF1	1.591578	14.74219	1.81E-15
ENSG0000(	DUSP2	1.59135	3.587773	0.000258
ENSG0000(	ITGAX	1.587309	3.021422	0.000952
ENSG0000(	RPP25	1.586588	2.995888	0.00101
ENSG0000(	DPT	1.585881	7.80188	1.58E-08
ENSG0000(	AC011558.	1.585316	4.664382	2.17E-05
ENSG0000(	AC091057.	1.584619	1.603289	0.024929
ENSG0000(	AHR	1.583941	8.56807	2.70E-09
ENSG0000(	ZBED6CL	1.583105	1.478563	0.033223
ENSG0000(	AL441992.:	1.583029	1.557924	0.027674
ENSG0000(	EPB41L3	1.582897	14.24352	5.71E-15
ENSG0000(	RNASE1	1.582578	5.641009	2.29E-06
ENSG0000(	LTB	1.582324	2.254986	0.005559
ENSG0000(	AP000919.	1.580681	2.08579	0.008207
ENSG0000(	PTN	1.579185	5.062532	8.66E-06
ENSG0000(	AL135926.:	1.575813	7.44013	3.63E-08
ENSG0000(	LINC01140	1.575579	5.303903	4.97E-06
ENSG0000(	HK3	1.574321	2.461199	0.003458
ENSG0000(	KCNC3	1.573147	4.843342	1.43E-05
ENSG0000(	OPRL1	1.571941	4.686305	2.06E-05
ENSG0000(	PINLYP	1.570268	1.878741	0.013221

ENSG0000(	CNN2	1.56939	10.01885	9.58E-11
ENSG0000(	CORO2A	1.568346	7.592957	2.55E-08
ENSG0000(	FERMT3	1.568306	7.065094	8.61E-08
ENSG0000(	AHNAK2	1.567223	6.239456	5.76E-07
ENSG0000(	HAVCR2	1.565548	8.529899	2.95E-09
ENSG0000(	PHLDA1	1.563915	10.95541	1.11E-11
ENSG0000(	TRAF1	1.562151	11.15182	7.05E-12
ENSG0000(	QPCT	1.561953	1.694428	0.02021
ENSG0000(	AC006487.	1.561871	2.02532	0.009434
ENSG0000(	GMIP	1.560877	5.149266	7.09E-06
ENSG0000(	LGALS9	1.560768	7.186594	6.51E-08
ENSG0000(	CGNL1	1.560724	6.60903	2.46E-07
ENSG0000(	LINC01252	1.560553	1.478346	0.033239
ENSG0000(	ADAMTS2	1.560218	4.098682	7.97E-05
ENSG0000(	CLDN4	1.557563	1.693533	0.020252
ENSG0000(	FXYD2	1.556986	2.034394	0.009239
ENSG0000(	SLC1A5	1.55552	10.50046	3.16E-11
ENSG0000(	CCDC80	1.554592	4.214322	6.10E-05
ENSG0000(	IL7R	1.552439	2.456938	0.003492
ENSG0000(	STMN3	1.551571	12.03963	9.13E-13
ENSG0000(	KAZALD1	1.550896	5.329179	4.69E-06
ENSG0000(	RAB32	1.549951	6.919869	1.20E-07
ENSG0000(	AC008738.	1.548799	4.812716	1.54E-05
ENSG0000(	SCN9A	1.547256	4.097846	7.98E-05
ENSG0000(	AC017002.	1.544958	5.266891	5.41E-06
ENSG0000(	EML2	1.544477	17.64192	2.28E-18
ENSG0000(	ECM2	1.544254	5.97562	1.06E-06
ENSG0000(	HCK	1.543969	2.977702	0.001053
ENSG0000(	KCNMB1	1.543929	2.504118	0.003132
ENSG0000(	ZNF486	1.542906	2.843219	0.001435
ENSG0000(	MFAP2	1.539949	3.581472	0.000262
ENSG0000(	U91328.2	1.539078	9.475687	3.34E-10
ENSG0000(	EEF1A1	1.53702	9.306347	4.94E-10
ENSG0000(	CMTM7	1.536993	9.073732	8.44E-10
ENSG0000(	LINC01116	1.536968	2.788933	0.001626
ENSG0000(	SLFN13	1.535995	9.422053	3.78E-10
ENSG0000(	TGFBI	1.535364	8.799672	1.59E-09
ENSG0000(	TRIM22	1.534886	9.087881	8.17E-10
ENSG0000(	ADAM12	1.534677	4.374764	4.22E-05
ENSG0000(	CCDC40	1.532168	13.79207	1.61E-14
ENSG0000(	PALM2	1.53166	1.348911	0.044781
ENSG0000(	MFHAS1	1.531652	15.01127	9.74E-16
ENSG0000(	LY75-CD30	1.531456	5.50346	3.14E-06
ENSG0000(	LACC1	1.531306	7.489363	3.24E-08
ENSG0000(	AL109918.:	1.530958	2.752754	0.001767
ENSG0000(	MDK	1.530694	10.98269	1.04E-11
ENSG0000(	AC009118.	1.530599	1.395318	0.040242

ENSG0000(	LCN10	1.530218	1.394323	0.040335
ENSG0000(	SLITRK4	1.529661	5.131186	7.39E-06
ENSG0000(	TGFB2-OT1	1.52905	10.86705	1.36E-11
ENSG0000(	APBA2	1.528489	1.558359	0.027647
ENSG0000(	C11orf80	1.527943	9.992314	1.02E-10
ENSG0000(	MIR34AHG	1.527878	8.338734	4.58E-09
ENSG0000(	SLC1A2	1.52711	2.323096	0.004752
ENSG0000(	ZNF826P	1.526919	1.748863	0.017829
ENSG0000(	ANXA2P2	1.52571	2.642605	0.002277
ENSG0000(	S100A11	1.525346	9.568968	2.70E-10
ENSG0000(	C5orf56	1.524714	10.72362	1.89E-11
ENSG0000(	BCAT1	1.523972	5.075615	8.40E-06
ENSG0000(	PSME1	1.522819	16.70291	1.98E-17
ENSG0000(	SCPEP1	1.522448	15.07331	8.45E-16
ENSG0000(	DACT2	1.522206	1.310036	0.048974
ENSG0000(	NTRK2	1.521261	4.126088	7.48E-05
ENSG0000(	UCHL1	1.521084	7.679271	2.09E-08
ENSG0000(	AC023906.	1.520225	4.010574	9.76E-05
ENSG0000(	VAMP8	1.519464	8.565258	2.72E-09
ENSG0000(	AC105942.	1.518742	6.886154	1.30E-07
ENSG0000(	LIPE	1.517844	2.524771	0.002987
ENSG0000(	INE2	1.517474	2.872221	0.001342
ENSG0000(	CARMIL1	1.516214	5.622882	2.38E-06
ENSG0000(	PSME2	1.51612	13.88566	1.30E-14
ENSG0000(	ARSI	1.515295	3.00348	0.000992
ENSG0000(	COL9A2	1.513856	2.248547	0.005642
ENSG0000(	SPTLC3	1.513727	7.697272	2.01E-08
ENSG0000(	AC055720.	1.51351	2.053049	0.00885
ENSG0000(	P2RY13	1.512946	1.557694	0.027689
ENSG0000(	SSTR2	1.512544	1.31359	0.048575
ENSG0000(	TMEM35A	1.512296	1.564711	0.027245
ENSG0000(	DAPP1	1.511743	2.777902	0.001668
ENSG0000(	PLEKHA2	1.511218	10.07616	8.39E-11
ENSG0000(	CADM3	1.511183	4.267215	5.40E-05
ENSG0000(	GAS2L3	1.511135	2.034993	0.009226
ENSG0000(	TMED3	1.511091	6.437099	3.66E-07
ENSG0000(	TGFB2-AS1	1.511004	3.220177	0.000602
ENSG0000(	EFHD2	1.510717	8.047682	8.96E-09
ENSG0000(	IL18R1	1.509931	4.797193	1.60E-05
ENSG0000(	IGFN1	1.509643	1.737216	0.018314
ENSG0000(	DAPK1	1.50938	15.70772	1.96E-16
ENSG0000(	CDON	1.509071	6.663528	2.17E-07
ENSG0000(	DOK1	1.507961	7.529953	2.95E-08
ENSG0000(	CAMK1D	1.506921	7.677066	2.10E-08
ENSG0000(	MAMDC2-/	1.506402	4.031417	9.30E-05
ENSG0000(	ESPL1	1.504575	1.645987	0.022595
ENSG0000(	SCARA3	1.499652	4.138291	7.27E-05

ENSG0000(	CBR1	1.499425	10.49164	3.22E-11
ENSG0000(	DGKA	1.497316	9.598392	2.52E-10
ENSG0000(	RGS19	1.497239	6.108165	7.80E-07
ENSG0000(	VWA5A	1.496192	10.79647	1.60E-11
ENSG0000(	UAP1L1	1.495436	11.62031	2.40E-12
ENSG0000(	FAM86HP	1.49467	1.944423	0.011365
ENSG0000(	BRSK1	1.494528	2.341504	0.004555
ENSG0000(	IRF6	1.491933	1.623117	0.023817
ENSG0000(	TNFSF13	1.491715	10.91251	1.22E-11
ENSG0000(	PVT1	1.491035	3.456639	0.000349
ENSG0000(	TRPV2	1.48985	8.171815	6.73E-09
ENSG0000(	TRANK1	1.489303	10.74369	1.80E-11
ENSG0000(	HLA-E	1.489123	10.09203	8.09E-11
ENSG0000(	PPP1R18	1.489068	11.07222	8.47E-12
ENSG0000(	PLCXD3	1.488949	1.819817	0.015142
ENSG0000(	NRROS	1.48868	7.145136	7.16E-08
ENSG0000(	CXADR	1.487662	1.540136	0.028831
ENSG0000(	CDCP1	1.487576	3.570627	0.000269
ENSG0000(	TRPM2	1.487107	4.464689	3.43E-05
ENSG0000(	NMRAL1	1.487105	7.117573	7.63E-08
ENSG0000(	LINC00963	1.486095	16.87538	1.33E-17
ENSG0000(	RAB37	1.485934	4.527145	2.97E-05
ENSG0000(	ALOX5	1.485501	3.624153	0.000238
ENSG0000(	CLIC1	1.484243	10.3874	4.10E-11
ENSG0000(	GSC	1.484205	1.587793	0.025835
ENSG0000(	IL34	1.484094	6.755175	1.76E-07
ENSG0000(	EEF1A1P5	1.483188	8.916425	1.21E-09
ENSG0000(	CEBPA	1.483033	4.269664	5.37E-05
ENSG0000(	GALNT8	1.480318	1.782042	0.016518
ENSG0000(	AC009084.	1.480284	1.348839	0.044788
ENSG0000(	ADCY7	1.480235	9.952161	1.12E-10
ENSG0000(	ELOVL5	1.478682	5.270662	5.36E-06
ENSG0000(	AC096677.	1.478139	2.469513	0.003392
ENSG0000(	AC144652.	1.476452	1.894762	0.012742
ENSG0000(	TGFB3	1.475752	7.395834	4.02E-08
ENSG0000(	SLC25A22	1.475196	13.45892	3.48E-14
ENSG0000(	MR1	1.475019	8.718819	1.91E-09
ENSG0000(	RIPK2	1.474949	8.852443	1.40E-09
ENSG0000(	FMO4	1.474178	3.546718	0.000284
ENSG0000(	GLIPR2	1.47412	6.857819	1.39E-07
ENSG0000(	KNL1	1.472392	1.350669	0.0446
ENSG0000(	SMC5-AS1	1.472346	4.475216	3.35E-05
ENSG0000(	TSKU	1.469332	3.048561	0.000894
ENSG0000(	C1QTNF3	1.469286	3.565852	0.000272
ENSG0000(	FRMD4B	1.468459	7.170923	6.75E-08
ENSG0000(	AC022034.	1.466627	3.6884	0.000205
ENSG0000(	AC108863.	1.466097	9.831503	1.47E-10

ENSG0000(	CADM3-AS	1.465129	4.555898	2.78E-05
ENSG0000(	LSP1P4	1.4645	2.828131	0.001485
ENSG0000(	ATP2A3	1.464462	6.371331	4.25E-07
ENSG0000(	MYD88	1.464036	8.845133	1.43E-09
ENSG0000(	TRAF5	1.46382	11.46715	3.41E-12
ENSG0000(	BBS12	1.463674	2.103094	0.007887
ENSG0000(	MEG9	1.463144	10.80087	1.58E-11
ENSG0000(	CDO1	1.462634	3.539334	0.000289
ENSG0000(	NNMT	1.458829	4.474787	3.35E-05
ENSG0000(	LPIN2	1.458702	14.24872	5.64E-15
ENSG0000(	IFI16	1.45805	7.039108	9.14E-08
ENSG0000(	LOXL1	1.456834	7.02558	9.43E-08
ENSG0000(	DPYSL5	1.455682	1.520398	0.030172
ENSG0000(	BEAN1	1.455121	2.068155	0.008548
ENSG0000(	RNASET2	1.455096	7.71103	1.95E-08
ENSG0000(	AC012615.	1.4546	5.594264	2.55E-06
ENSG0000(	IGFLR1	1.454106	5.884047	1.31E-06
ENSG0000(	PLEKHO2	1.453804	8.174579	6.69E-09
ENSG0000(	TNFAIP3	1.453456	11.13523	7.32E-12
ENSG0000(	LOXL1-AS1	1.450708	7.024578	9.45E-08
ENSG0000(	ZNF815P	1.450185	1.628444	0.023526
ENSG0000(	AC010245.	1.449215	1.467764	0.034059
ENSG0000(	ARPC1B	1.448821	9.750014	1.78E-10
ENSG0000(	PIK3CD	1.448663	6.272727	5.34E-07
ENSG0000(	NAIP	1.448359	1.416902	0.038291
ENSG0000(	TRABD2A	1.448254	6.97938	1.05E-07
ENSG0000(	OMD	1.448021	2.60806	0.002466
ENSG0000(	TRERF1	1.447795	7.325127	4.73E-08
ENSG0000(	CORO1B	1.447594	8.990886	1.02E-09
ENSG0000(	CSF2RB	1.447512	3.982338	0.000104
ENSG0000(	DNM1	1.445247	5.65314	2.22E-06
ENSG0000(	IFIT5	1.443184	11.03131	9.30E-12
ENSG0000(	FUOM	1.443049	4.915987	1.21E-05
ENSG0000(	LINC00702	1.442247	5.059003	8.73E-06
ENSG0000(	LIMD2	1.442005	5.211199	6.15E-06
ENSG0000(	AC008622.	1.439557	3.901147	0.000126
ENSG0000(	ATP8B2	1.438919	12.88065	1.32E-13
ENSG0000(	MRAP2	1.438882	1.496301	0.031893
ENSG0000(	ELN-AS1	1.438498	5.496726	3.19E-06
ENSG0000(	MPZL3	1.437717	1.925009	0.011885
ENSG0000(	AC092821.	1.437484	2.912755	0.001222
ENSG0000(	PTGFR	1.437273	2.376985	0.004198
ENSG0000(	FCHSD1	1.437054	10.54858	2.83E-11
ENSG0000(	RIPK3	1.436781	6.961874	1.09E-07
ENSG0000(	CCDC3	1.436732	6.224923	5.96E-07
ENSG0000(	LRRK1	1.434909	11.76567	1.72E-12
ENSG0000(	ZBTB7C	1.434852	7.630474	2.34E-08

ENSG0000(	C1QTNF7	1.433796	2.188555	0.006478
ENSG0000(	CLEC2D	1.433137	7.235758	5.81E-08
ENSG0000(	STK26	1.431177	7.250443	5.62E-08
ENSG0000(	AC093484.	1.430958	1.98712	0.010301
ENSG0000(	PHGDH	1.430242	3.774137	0.000168
ENSG0000(	MEX3A	1.42913	1.384572	0.04125
ENSG0000(	OAS2	1.429071	4.436538	3.66E-05
ENSG0000(	TGFB2	1.429069	8.819547	1.52E-09
ENSG0000(	RYR2	1.428492	1.516809	0.030422
ENSG0000(	S100A10	1.42788	7.675138	2.11E-08
ENSG0000(	PTGFRN	1.427821	7.047365	8.97E-08
ENSG0000(	ZNF300	1.427712	4.497946	3.18E-05
ENSG0000(	TRIB3	1.427065	5.008717	9.80E-06
ENSG0000(	PLA1A	1.426921	3.845994	0.000143
ENSG0000(	ARSD-AS1	1.42669	5.101426	7.92E-06
ENSG0000(	GABBR1	1.425947	7.902456	1.25E-08
ENSG0000(	HTR7P1	1.425892	1.999112	0.01002
ENSG0000(	LST1	1.425882	2.365477	0.00431
ENSG0000(	SPACA9	1.425053	3.436437	0.000366
ENSG0000(	EMID1	1.424064	4.782227	1.65E-05
ENSG0000(	HACD4	1.423826	5.838523	1.45E-06
ENSG0000(	ID4	1.421308	5.173501	6.71E-06
ENSG0000(	AC008738.	1.421086	3.497616	0.000318
ENSG0000(	FAM20A	1.420738	7.695811	2.01E-08
ENSG0000(	ARSD	1.420669	10.71273	1.94E-11
ENSG0000(	ZC3HAV1	1.419486	16.48709	3.26E-17
ENSG0000(	BGN	1.419362	5.98463	1.04E-06
ENSG0000(	ISM1	1.419169	3.925523	0.000119
ENSG0000(	LIF	1.418757	1.703368	0.019798
ENSG0000(	CLEC4GP1	1.417408	2.365227	0.004313
ENSG0000(	JAML	1.416187	1.931145	0.011718
ENSG0000(	KIAA1671	1.414797	11.00594	9.86E-12
ENSG0000(	FBXL13	1.414338	3.500385	0.000316
ENSG0000(	AP000688.	1.413852	8.74841	1.78E-09
ENSG0000(	TFAP2E	1.413484	3.388598	0.000409
ENSG0000(	LINC01232	1.413165	9.515053	3.05E-10
ENSG0000(	AC127024.	1.413163	4.838514	1.45E-05
ENSG0000(	GPR176	1.41311	9.173126	6.71E-10
ENSG0000(	PRSS36	1.412924	5.679555	2.09E-06
ENSG0000(	TRIM34	1.412832	8.719814	1.91E-09
ENSG0000(	AC093484.	1.41188	2.090006	0.008128
ENSG0000(	SYT15	1.411516	2.702411	0.001984
ENSG0000(	PRKX	1.41109	7.965752	1.08E-08
ENSG0000(	SAMD9L	1.410657	6.716506	1.92E-07
ENSG0000(	NMB	1.409945	1.984602	0.010361
ENSG0000(	ST8SIA4	1.409492	4.423871	3.77E-05
ENSG0000(	MERTK	1.407552	6.685305	2.06E-07

ENSG0000(	ANTXR1	1.407542	6.090538	8.12E-07
ENSG0000(	AC009511.	1.407536	1.513478	0.030656
ENSG0000(	GSDMD	1.407361	11.36493	4.32E-12
ENSG0000(	LUM	1.406717	3.700366	0.000199
ENSG0000(	AL357033.4	1.40566	3.828015	0.000149
ENSG0000(	STEAP2	1.404054	2.35157	0.004451
ENSG0000(	FBLN7	1.403783	4.711833	1.94E-05
ENSG0000(	PRPS2	1.40304	7.901669	1.25E-08
ENSG0000(	MLKL	1.402683	8.965232	1.08E-09
ENSG0000(	TNFRSF14	1.401531	9.411221	3.88E-10
ENSG0000(	CD24	1.400386	2.434499	0.003677
ENSG0000(	AC007068.	1.399954	6.395888	4.02E-07
ENSG0000(	AL365273.1	1.399566	2.548013	0.002831
ENSG0000(	PRR7	1.395965	2.227069	0.005928
ENSG0000(	HNMT	1.395956	5.823519	1.50E-06
ENSG0000(	NOX4	1.395699	2.304536	0.00496
ENSG0000(	GNAS-AS1	1.395636	1.908435	0.012347
ENSG0000(	AC069368.	1.395322	6.942826	1.14E-07
ENSG0000(	NABP1	1.395168	6.643392	2.27E-07
ENSG0000(	MMP2	1.394726	4.044432	9.03E-05
ENSG0000(	DUXAP10	1.394121	1.733813	0.018458
ENSG0000(	VCAN-AS1	1.394061	3.821579	0.000151
ENSG0000(	TMSB4X	1.393461	6.344114	4.53E-07
ENSG0000(	FBLIM1	1.393032	8.888017	1.29E-09
ENSG0000(	FAM83G	1.392482	9.0787	8.34E-10
ENSG0000(	AC010186.	1.392125	2.063631	0.008637
ENSG0000(	AL158151.1	1.391673	5.808974	1.55E-06
ENSG0000(	AP001972.	1.389313	3.621893	0.000239
ENSG0000(	CLSTN2	1.389251	4.301208	5.00E-05
ENSG0000(	AC004593.	1.388043	7.957913	1.10E-08
ENSG0000(	AOX1	1.387461	2.415636	0.00384
ENSG0000(	AC007620.	1.387373	7.499931	3.16E-08
ENSG0000(	MIR198	1.386667	3.376546	0.00042
ENSG0000(	AC009950.	1.385164	5.898803	1.26E-06
ENSG0000(	TM4SF19	1.385104	2.014711	0.009667
ENSG0000(	ZNF682	1.384778	5.672376	2.13E-06
ENSG0000(	MVP	1.384647	16.65769	2.20E-17
ENSG0000(	TMEM106A	1.384298	7.369482	4.27E-08
ENSG0000(	ANXA2	1.384043	8.770134	1.70E-09
ENSG0000(	CELSR1	1.3831	4.942379	1.14E-05
ENSG0000(	AC007336.	1.382513	3.711071	0.000195
ENSG0000(	ABI3BP	1.382389	5.889912	1.29E-06
ENSG0000(	FAM83H	1.382135	4.936508	1.16E-05
ENSG0000(	SEMA3D	1.381482	1.607034	0.024715
ENSG0000(	HERC6	1.380665	8.524782	2.99E-09
ENSG0000(	CD226	1.380175	4.245429	5.68E-05
ENSG0000(	ROBO3	1.378671	8.873458	1.34E-09

ENSG0000(	PCDH18	1.377808	3.61172	0.000245
ENSG0000(	TMSB10	1.377781	8.407132	3.92E-09
ENSG0000(	TECTA	1.377023	2.773427	0.001685
ENSG0000(	LOX	1.374458	2.617834	0.002411
ENSG0000(	RFX2	1.374079	5.640383	2.29E-06
ENSG0000(	TP53I3	1.374045	7.159972	6.92E-08
ENSG0000(	PRELP	1.373605	2.093563	0.008062
ENSG0000(	SOX8	1.373534	4.221683	6.00E-05
ENSG0000(	VIM	1.372699	6.286805	5.17E-07
ENSG0000(	PLEKHH2	1.371102	3.940916	0.000115
ENSG0000(	TTYH3	1.37081	10.37047	4.26E-11
ENSG0000(	NOV	1.369062	2.99736	0.001006
ENSG0000(	AC022007.	1.368146	2.421728	0.003787
ENSG0000(	SLC7A1	1.367816	15.61132	2.45E-16
ENSG0000(	METRNL	1.367706	6.843464	1.43E-07
ENSG0000(	SLC35F2	1.36715	7.537223	2.90E-08
ENSG0000(	CLIC2	1.36668	5.235403	5.82E-06
ENSG0000(	GSDME	1.366338	8.174579	6.69E-09
ENSG0000(	RARRES2	1.366186	3.196668	0.000636
ENSG0000(	LRRC56	1.3653	3.316698	0.000482
ENSG0000(	IL4R	1.364646	9.151423	7.06E-10
ENSG0000(	SLC49A3	1.364282	8.278589	5.27E-09
ENSG0000(	AC022107.	1.363665	2.142436	0.007204
ENSG0000(	AC016747.	1.363405	5.855423	1.40E-06
ENSG0000(	S1PR2	1.363196	5.342337	4.55E-06
ENSG0000(	CD37	1.363134	6.19253	6.42E-07
ENSG0000(	UGCG	1.363004	13.96212	1.09E-14
ENSG0000(	CSPG4	1.362448	6.762309	1.73E-07
ENSG0000(	DMXL2	1.361869	7.079082	8.34E-08
ENSG0000(	KCNK6	1.361277	8.071189	8.49E-09
ENSG0000(	TMEM107	1.360456	4.578685	2.64E-05
ENSG0000(	SEMA7A	1.360008	9.838815	1.45E-10
ENSG0000(	LPAR1	1.359513	4.080672	8.30E-05
ENSG0000(	TLDC2	1.359491	8.095281	8.03E-09
ENSG0000(	LURAP1	1.359061	1.41297	0.038639
ENSG0000(	PCDHA9	1.358489	1.528165	0.029637
ENSG0000(	AC005041.	1.358454	3.43258	0.000369
ENSG0000(	DDX60	1.35797	6.505637	3.12E-07
ENSG0000(	SPINT2	1.356139	5.684483	2.07E-06
ENSG0000(	PCDHAC1	1.355437	1.893045	0.012792
ENSG0000(	ARL4A	1.355154	6.384156	4.13E-07
ENSG0000(	ALDH3B1	1.35309	10.74394	1.80E-11
ENSG0000(	CPXM2	1.352487	4.567074	2.71E-05
ENSG0000(	MAN1A1	1.352021	5.167186	6.80E-06
ENSG0000(	HMOX1	1.351706	9.07449	8.42E-10
ENSG0000(	MYO1F	1.351698	2.694426	0.002021
ENSG0000(	PLXNB2	1.35061	13.31641	4.83E-14

ENSG0000(	RTN1	1.349882	3.714939	0.000193
ENSG0000(	INPP5D	1.348342	4.686305	2.06E-05
ENSG0000(	FGD3	1.347031	3.637993	0.00023
ENSG0000(	MIR5193	1.346392	7.900664	1.26E-08
ENSG0000(	TUBA1A	1.346224	9.751158	1.77E-10
ENSG0000(	DOCK11	1.34563	4.17011	6.76E-05
ENSG0000(	PCDHA1	1.345302	1.50379	0.031348
ENSG0000(	GFPT2	1.345217	4.123997	7.52E-05
ENSG0000(	GALNT3	1.344902	1.371492	0.042512
ENSG0000(	SAMHD1	1.344193	8.475347	3.35E-09
ENSG0000(	PIP4K2A	1.344109	7.588821	2.58E-08
ENSG0000(	FLT3LG	1.343885	7.468599	3.40E-08
ENSG0000(	TCF7	1.343666	4.971698	1.07E-05
ENSG0000(	ARHGEF5	1.343237	2.929246	0.001177
ENSG0000(	SESN3	1.341694	4.942227	1.14E-05
ENSG0000(	HELLS	1.341378	5.914542	1.22E-06
ENSG0000(	PCDHB7	1.341013	3.118966	0.00076
ENSG0000(	PSAT1	1.339836	1.962874	0.010892
ENSG0000(	ITIH3	1.339218	6.367861	4.29E-07
ENSG0000(	AC011451.	1.338142	1.312505	0.048696
ENSG0000(	CLIP3	1.338139	10.38142	4.16E-11
ENSG0000(	ALPK1	1.337827	11.59759	2.53E-12
ENSG0000(	AL133415.:	1.336958	5.914221	1.22E-06
ENSG0000(	BEX2	1.336482	2.429412	0.00372
ENSG0000(	GCNT2	1.336336	1.365484	0.043104
ENSG0000(	PCDHA5	1.335231	1.867391	0.013571
ENSG0000(	SNX22	1.334742	7.442269	3.61E-08
ENSG0000(	SFT2D2	1.333264	15.09249	8.08E-16
ENSG0000(	ABHD17C	1.332566	3.890868	0.000129
ENSG0000(	ENPP2	1.331159	5.79336	1.61E-06
ENSG0000(	PCDHA8	1.331135	1.481513	0.032998
ENSG0000(	CTGF	1.330549	4.016924	9.62E-05
ENSG0000(	DIRAS3	1.329423	1.681408	0.020825
ENSG0000(	CD300A	1.328251	2.262242	0.005467
ENSG0000(	C1RL-AS1	1.327679	10.30218	4.99E-11
ENSG0000(	PCDHA6	1.32511	1.464241	0.034337
ENSG0000(	TLR2	1.324622	1.395432	0.040232
ENSG0000(	GNB5	1.324299	9.526045	2.98E-10
ENSG0000(	AC010894.	1.32393	4.479172	3.32E-05
ENSG0000(	SRGN	1.323561	4.111242	7.74E-05
ENSG0000(	TIAM1	1.323315	5.529974	2.95E-06
ENSG0000(	KLF16	1.323217	9.526742	2.97E-10
ENSG0000(	GEM	1.323063	5.943305	1.14E-06
ENSG0000(	AL603832.:	1.321772	3.437063	0.000366
ENSG0000(	DAB2	1.321227	5.585693	2.60E-06
ENSG0000(	ANXA4	1.320012	8.957409	1.10E-09
ENSG0000(	ARHGAP9	1.319312	2.467201	0.00341

ENSG0000(	UBA7	1.319042	10.47699	3.33E-11
ENSG0000(	TRAF4	1.318995	9.931644	1.17E-10
ENSG0000(	AC007938.	1.318985	2.185629	0.006522
ENSG0000(	PRKCD	1.318832	6.655327	2.21E-07
ENSG0000(	ZNF385D	1.317577	6.044004	9.04E-07
ENSG0000(	NUDT18	1.317434	7.281423	5.23E-08
ENSG0000(	PCDHA13	1.317281	1.449693	0.035506
ENSG0000(	LYVE1	1.317148	1.897658	0.012657
ENSG0000(	CABLES1	1.316987	4.323402	4.75E-05
ENSG0000(	CMPK2	1.31671	6.437392	3.65E-07
ENSG0000(	TBC1D16	1.314319	9.784141	1.64E-10
ENSG0000(	COL1A1	1.313873	2.743751	0.001804
ENSG0000(	THBS2	1.31352	2.861958	0.001374
ENSG0000(	PAG1	1.313276	5.251674	5.60E-06
ENSG0000(	FAM102B	1.313276	7.152688	7.04E-08
ENSG0000(	CTHRC1	1.312732	2.277441	0.005279
ENSG0000(	CLMP	1.312308	3.380776	0.000416
ENSG0000(	AC129507.	1.312266	8.549003	2.82E-09
ENSG0000(	CRYBG1	1.311125	10.9277	1.18E-11
ENSG0000(	PNMA2	1.310985	2.009465	0.009784
ENSG0000(	AC092279.	1.31073	1.547864	0.028323
ENSG0000(	REC8	1.30991	7.846998	1.42E-08
ENSG0000(	HPS3	1.309477	8.258019	5.52E-09
ENSG0000(	MEG8	1.309097	3.739749	0.000182
ENSG0000(	C2orf81	1.308393	4.047819	8.96E-05
ENSG0000(	AC015922.	1.308384	1.394893	0.040282
ENSG0000(	CHRND	1.308182	3.78397	0.000164
ENSG0000(	TMEM158	1.306581	4.772482	1.69E-05
ENSG0000(	GPX8	1.305956	4.780104	1.66E-05
ENSG0000(	CKB	1.305713	3.843342	0.000143
ENSG0000(	ABCG2	1.305691	1.385419	0.04117
ENSG0000(	CTSH	1.303778	5.20948	6.17E-06
ENSG0000(	AC104758.	1.303665	2.878082	0.001324
ENSG0000(	RNF43	1.303511	1.310806	0.048887
ENSG0000(	PRKCB	1.30324	2.244604	0.005694
ENSG0000(	ZNF217	1.303129	9.568968	2.70E-10
ENSG0000(	GNA14	1.302464	1.563399	0.027328
ENSG0000(	BRCA2	1.302282	1.575318	0.026588
ENSG0000(	NLRC3	1.301525	5.574244	2.67E-06
ENSG0000(	GNG2	1.3007	7.93004	1.17E-08
ENSG0000(	UNC5CL	1.300614	1.787518	0.016311
ENSG0000(	PLEKHO1	1.300294	9.797243	1.59E-10
ENSG0000(	TMEM45A	1.299839	4.117094	7.64E-05
ENSG0000(	PTK2B	1.299819	11.54407	2.86E-12
ENSG0000(	AC012313.	1.299696	8.87839	1.32E-09
ENSG0000(	AC011446.	1.299263	6.325274	4.73E-07
ENSG0000(	UBAC2-AS1	1.297949	1.846199	0.01425

ENSG0000(	TLR1	1.297222	1.657264	0.022016
ENSG0000(	AC018816.	1.296848	7.824474	1.50E-08
ENSG0000(	KRT16P6	1.296239	1.323888	0.047436
ENSG0000(	SCIMP	1.295435	5.177036	6.65E-06
ENSG0000(	HHAT	1.295422	2.39697	0.004009
ENSG0000(	RASGEF1A	1.295354	1.922067	0.011966
ENSG0000(	ZNF101	1.29473	6.355253	4.41E-07
ENSG0000(	FRMD1	1.294308	2.504578	0.003129
ENSG0000(	PLPPR4	1.293102	2.502754	0.003142
ENSG0000(	ACOT4	1.292555	2.521222	0.003011
ENSG0000(	SRGAP2C	1.291932	7.375469	4.21E-08
ENSG0000(	ORAI2	1.290911	9.015626	9.65E-10
ENSG0000(	CMAHP	1.290906	5.534849	2.92E-06
ENSG0000(	PPIB	1.290188	7.035404	9.22E-08
ENSG0000(	PCDHA12	1.289428	1.390433	0.040697
ENSG0000(	C2CD2	1.289039	10.76895	1.70E-11
ENSG0000(	ARPC5	1.288898	7.981321	1.04E-08
ENSG0000(	AP000813.	1.288401	1.368909	0.042765
ENSG0000(	SH3BGRL	1.287606	6.655475	2.21E-07
ENSG0000(	ST8SIA1	1.285417	5.684453	2.07E-06
ENSG0000(	NCAM1-AS	1.284504	2.641901	0.002281
ENSG0000(	DAP	1.284072	8.29551	5.06E-09
ENSG0000(	IER5L	1.283941	6.105094	7.85E-07
ENSG0000(	AC091825.	1.283384	2.183289	0.006557
ENSG0000(	PRRT4	1.282948	4.727704	1.87E-05
ENSG0000(	CHN2	1.282333	7.31831	4.80E-08
ENSG0000(	SLC12A7	1.281739	9.179783	6.61E-10
ENSG0000(	CYLD	1.281684	12.3564	4.40E-13
ENSG0000(	AC009086.	1.281148	5.80613	1.56E-06
ENSG0000(	MID1	1.281017	7.400731	3.97E-08
ENSG0000(	ARHGAP25	1.280784	4.582476	2.62E-05
ENSG0000(	ACOT7	1.275628	8.26306	5.46E-09
ENSG0000(	RAB7B	1.275422	4.753763	1.76E-05
ENSG0000(	AC092718.	1.274833	1.517663	0.030362
ENSG0000(	GVINP1	1.274114	3.332784	0.000465
ENSG0000(	ADORA1	1.273936	5.350974	4.46E-06
ENSG0000(	CORO7	1.273169	9.183022	6.56E-10
ENSG0000(	DENND6B	1.2726	5.983086	1.04E-06
ENSG0000(	AC100791.	1.272267	4.788202	1.63E-05
ENSG0000(	VAV2	1.271947	15.55101	2.81E-16
ENSG0000(	ELN	1.271903	4.400863	3.97E-05
ENSG0000(	NCF4	1.271121	1.474586	0.033529
ENSG0000(	CYS1	1.26997	4.098821	7.96E-05
ENSG0000(	STX11	1.269476	3.547302	0.000284
ENSG0000(	ANGPTL6	1.269308	8.447057	3.57E-09
ENSG0000(	HGF	1.267246	3.035844	0.000921
ENSG0000(	VIM-AS1	1.265273	5.431522	3.70E-06

ENSG0000(	PCDHA7	1.264867	1.342592	0.045437
ENSG0000(	CMTM3	1.264682	5.200564	6.30E-06
ENSG0000(	LRP8	1.264276	7.873189	1.34E-08
ENSG0000(	PCDHA11	1.264006	1.63407	0.023224
ENSG0000(	TSPAN11	1.263141	2.64807	0.002249
ENSG0000(	TTC8	1.262734	8.785118	1.64E-09
ENSG0000(	ITGA11	1.262228	3.039495	0.000913
ENSG0000(	WISP2	1.261809	3.103952	0.000787
ENSG0000(	ACSL5	1.26125	7.661292	2.18E-08
ENSG0000(	FSTL1	1.259898	3.193123	0.000641
ENSG0000(	SNCAIP	1.259544	1.501661	0.031502
ENSG0000(	AL139384.:	1.259419	1.353294	0.044331
ENSG0000(	AL355075.:	1.259344	5.952543	1.12E-06
ENSG0000(	FBXO36	1.257614	1.566797	0.027115
ENSG0000(	GPC3	1.257578	3.230714	0.000588
ENSG0000(	PDE6B	1.256857	2.522353	0.003004
ENSG0000(	PCDHA10	1.256607	1.718893	0.019103
ENSG0000(	AL359532.:	1.256228	1.311252	0.048837
ENSG0000(	AC105020.	1.255399	5.760822	1.73E-06
ENSG0000(	CHN1	1.255389	5.861064	1.38E-06
ENSG0000(	IL16	1.255046	5.405755	3.93E-06
ENSG0000(	LINC00265	1.254812	5.316131	4.83E-06
ENSG0000(	AF117829.	1.253253	2.961835	0.001092
ENSG0000(	PPARG	1.252865	2.28267	0.005216
ENSG0000(	SH3BP4	1.250759	5.789696	1.62E-06
ENSG0000(	SYNE3	1.248876	6.652739	2.22E-07
ENSG0000(	PLXNB1	1.248529	7.627171	2.36E-08
ENSG0000(	EBF1	1.247819	4.205449	6.23E-05
ENSG0000(	CD276	1.247351	7.782814	1.65E-08
ENSG0000(	PEG10	1.247229	5.628389	2.35E-06
ENSG0000(	ATP9A	1.24704	10.71273	1.94E-11
ENSG0000(	PLXNB3	1.246554	2.311289	0.004883
ENSG0000(	FOXP3	1.24644	3.486001	0.000327
ENSG0000(	MX2	1.245155	5.003034	9.93E-06
ENSG0000(	LDAH	1.24445	4.267215	5.40E-05
ENSG0000(	LY6E	1.243598	5.897665	1.27E-06
ENSG0000(	HIST1H4I	1.243536	2.889139	0.001291
ENSG0000(	AC002116.	1.243254	7.545005	2.85E-08
ENSG0000(	TMEM173	1.242672	10.26439	5.44E-11
ENSG0000(	C19orf66	1.242358	9.201243	6.29E-10
ENSG0000(	AC007066.	1.241038	1.64356	0.022722
ENSG0000(	ATP10D	1.24091	5.164417	6.85E-06
ENSG0000(	TAPBP	1.240822	12.56436	2.73E-13
ENSG0000(	AC087289.	1.237575	8.507656	3.11E-09
ENSG0000(	TLL1	1.236718	2.002199	0.009949
ENSG0000(	ZNF845	1.236649	6.82892	1.48E-07
ENSG0000(	EGLN3	1.23626	6.057938	8.75E-07

ENSG0000(	FMN1	1.236172	2.944048	0.001138
ENSG0000(	TGIF2	1.236052	8.775299	1.68E-09
ENSG0000(	PHLDA3	1.235548	13.81748	1.52E-14
ENSG0000(	AMPD3	1.235412	4.638772	2.30E-05
ENSG0000(	SOD3	1.232779	3.900972	0.000126
ENSG0000(	PPM1M	1.231366	8.456689	3.49E-09
ENSG0000(	LINC01547	1.230854	2.240963	0.005742
ENSG0000(	RAB8B	1.230608	6.343589	4.53E-07
ENSG0000(	PQLC3	1.23037	6.144645	7.17E-07
ENSG0000(	BASP1	1.230253	3.454512	0.000351
ENSG0000(	CTSO	1.230217	6.67523	2.11E-07
ENSG0000(	AC019117.	1.230086	2.939735	0.001149
ENSG0000(	KIFC1	1.2297	2.3438	0.004531
ENSG0000(	TICRR	1.229248	3.733519	0.000185
ENSG0000(	SAMD10	1.22889	3.404866	0.000394
ENSG0000(	TRIM6-TRII	1.228044	6.583933	2.61E-07
ENSG0000(	PNP	1.227795	7.832099	1.47E-08
ENSG0000(	FST	1.227418	3.588461	0.000258
ENSG0000(	ELF4	1.226798	8.347591	4.49E-09
ENSG0000(	MYO15A	1.225436	1.795187	0.016026
ENSG0000(	NOVA1	1.225104	3.771971	0.000169
ENSG0000(	TMEM14A	1.224746	5.962236	1.09E-06
ENSG0000(	PGM5-AS1	1.224475	2.075521	0.008404
ENSG0000(	RINL	1.223877	6.947833	1.13E-07
ENSG0000(	LYN	1.222948	3.48528	0.000327
ENSG0000(	AL160269.:	1.222228	12.24939	5.63E-13
ENSG0000(	OAS3	1.221865	5.058989	8.73E-06
ENSG0000(	SAT1	1.221782	5.812254	1.54E-06
ENSG0000(	TNFRSF1B	1.221214	7.170923	6.75E-08
ENSG0000(	EMP1	1.220097	4.201512	6.29E-05
ENSG0000(	SPHK1	1.220088	8.853554	1.40E-09
ENSG0000(	MMP14	1.219878	4.516202	3.05E-05
ENSG0000(	F2R	1.218981	6.147498	7.12E-07
ENSG0000(	RGS2	1.218795	2.512187	0.003075
ENSG0000(	CERS4	1.218659	5.883997	1.31E-06
ENSG0000(	TMEM86A	1.218291	6.101942	7.91E-07
ENSG0000(	MEST	1.217178	3.386703	0.00041
ENSG0000(	SERPING1	1.216962	5.814407	1.53E-06
ENSG0000(	PLEKHA5	1.215749	5.843213	1.43E-06
ENSG0000(	TBC1D32	1.215625	1.654199	0.022172
ENSG0000(	LFNG	1.215266	6.077846	8.36E-07
ENSG0000(	GNB4	1.214972	6.74237	1.81E-07
ENSG0000(	SDK1	1.214667	3.346233	0.000451
ENSG0000(	SFXN3	1.213941	8.579521	2.63E-09
ENSG0000(	6-Sep	1.213633	7.787997	1.63E-08
ENSG0000(	AC020931.	1.213631	7.244509	5.69E-08
ENSG0000(	PI16	1.213582	2.117061	0.007637

ENSG0000(	EPHB3	1.213534	3.774656	0.000168
ENSG0000(	PLK3	1.211651	8.168869	6.78E-09
ENSG0000(	AL022323.:	1.211473	4.585397	2.60E-05
ENSG0000(	BTG3	1.211411	6.056146	8.79E-07
ENSG0000(	WASF1	1.211318	2.284729	0.005191
ENSG0000(	C17orf58	1.211086	3.964416	0.000109
ENSG0000(	BTBD11	1.210705	2.08751	0.008175
ENSG0000(	CYBRD1	1.210347	3.189806	0.000646
ENSG0000(	PDLIM4	1.210066	4.759495	1.74E-05
ENSG0000(	SAMD9	1.209373	3.193029	0.000641
ENSG0000(	PCDHAC2	1.20935	1.642967	0.022753
ENSG0000(	VCAN	1.208457	3.144087	0.000718
ENSG0000(	HHIPL1	1.207928	3.533398	0.000293
ENSG0000(	CST3	1.20769	6.722005	1.90E-07
ENSG0000(	RASSF7	1.207593	5.638956	2.30E-06
ENSG0000(	AC022414.	1.207548	1.841978	0.014389
ENSG0000(	EFNA4	1.207165	1.348541	0.044819
ENSG0000(	TTC7A	1.206905	8.199548	6.32E-09
ENSG0000(	TRIM47	1.205158	10.24317	5.71E-11
ENSG0000(	RASGEF1B	1.204499	6.212979	6.12E-07
ENSG0000(	COL1A2	1.204295	3.319635	0.000479
ENSG0000(	PCDHA2	1.20403	1.951133	0.011191
ENSG0000(	IFI44L	1.203522	3.310153	0.00049
ENSG0000(	CLU	1.202738	4.856597	1.39E-05
ENSG0000(	ASPM	1.202518	1.834119	0.014651
ENSG0000(	IFITM1	1.201229	6.852117	1.41E-07
ENSG0000(	SEC24D	1.199771	6.4894	3.24E-07
ENSG0000(	MAP3K7CL	1.19726	2.037195	0.009179
ENSG0000(	C9	1.196918	4.066672	8.58E-05
ENSG0000(	AC116903.	1.1966	6.862859	1.37E-07
ENSG0000(	POLE2	1.195349	2.013104	0.009703
ENSG0000(	LGALS3	1.195074	7.893554	1.28E-08
ENSG0000(	PYGL	1.195073	4.533868	2.93E-05
ENSG0000(	AC117503.	1.195043	1.632982	0.023282
ENSG0000(	NRSN2	1.194149	8.955922	1.11E-09
ENSG0000(	AL139339.:	1.193933	7.214142	6.11E-08
ENSG0000(	SH3TC1	1.193535	8.240202	5.75E-09
ENSG0000(	PRRG4	1.192571	3.165418	0.000683
ENSG0000(	CNN3	1.191852	6.667334	2.15E-07
ENSG0000(	PCDHA4	1.19182	1.938101	0.011532
ENSG0000(	AL133346.:	1.190419	2.495677	0.003194
ENSG0000(	RASSF2	1.189687	3.659167	0.000219
ENSG0000(	SDF2L1	1.189598	5.874166	1.34E-06
ENSG0000(	FAM111A	1.189597	8.760387	1.74E-09
ENSG0000(	AC008771.	1.189391	1.437658	0.036504
ENSG0000(	MIR6515	1.18803	2.220993	0.006012
ENSG0000(	AC083799.	1.188023	1.57086	0.026862

ENSG0000(	HLA-L	1.186869	3.258235	0.000552
ENSG0000(	SDC2	1.186157	4.642745	2.28E-05
ENSG0000(	SCAMP1-A!	1.185485	4.114978	7.67E-05
ENSG0000(	MIR922	1.18482	2.293628	0.005086
ENSG0000(	IER2	1.184621	6.030152	9.33E-07
ENSG0000(	GLI3	1.1843	5.285846	5.18E-06
ENSG0000(	ABHD15	1.183158	6.516357	3.05E-07
ENSG0000(	CMTM6	1.181194	5.851623	1.41E-06
ENSG0000(	SIDT1	1.180226	6.070029	8.51E-07
ENSG0000(	LINC02381	1.179828	2.802223	0.001577
ENSG0000(	NIPSNAP1	1.179624	13.47243	3.37E-14
ENSG0000(	FGF18	1.179098	1.568532	0.027007
ENSG0000(	PCOLCE	1.178946	5.876372	1.33E-06
ENSG0000(	TLNRD1	1.178546	9.524735	2.99E-10
ENSG0000(	TLR4	1.178163	3.084547	0.000823
ENSG0000(	CLEC2B	1.177322	4.611426	2.45E-05
ENSG0000(	AC099489.	1.177158	1.814725	0.015321
ENSG0000(	RUNX2	1.176534	2.34758	0.004492
ENSG0000(	MDGA1	1.17537	5.022638	9.49E-06
ENSG0000(	PLEKHG4	1.175191	4.153371	7.02E-05
ENSG0000(	AL136982.7	1.174615	3.042149	0.000908
ENSG0000(	BEGAIN	1.174356	3.270742	0.000536
ENSG0000(	TMEM35B	1.173881	6.333935	4.64E-07
ENSG0000(	ADORA2A	1.173459	3.769667	0.00017
ENSG0000(	WDR54	1.173266	5.846168	1.43E-06
ENSG0000(	AGTRAP	1.173211	7.440097	3.63E-08
ENSG0000(	ZNF85	1.173188	1.723613	0.018897
ENSG0000(	DCST2	1.172523	1.483278	0.032864
ENSG0000(	RF01684	1.172262	1.978607	0.010505
ENSG0000(	AC005726.	1.172113	2.154901	0.007
ENSG0000(	ZNF600	1.172066	4.380099	4.17E-05
ENSG0000(	SLC35D2	1.171957	10.66242	2.18E-11
ENSG0000(	BCL3	1.171649	9.392362	4.05E-10
ENSG0000(	HR	1.171387	3.121317	0.000756
ENSG0000(	MAN1B1-D	1.171282	5.632528	2.33E-06
ENSG0000(	TYRO3	1.170046	2.525431	0.002982
ENSG0000(	ASF1B	1.169907	1.976659	0.010552
ENSG0000(	LRRC4	1.169705	1.850162	0.01412
ENSG0000(	TLCD2	1.169595	5.161207	6.90E-06
ENSG0000(	FBLN1	1.168427	3.874224	0.000134
ENSG0000(	LVRN	1.168125	1.890333	0.012873
ENSG0000(	ZNF382	1.167759	2.098709	0.007967
ENSG0000(	MSC	1.167484	6.389852	4.08E-07
ENSG0000(	PLCE1	1.167439	4.001306	9.97E-05
ENSG0000(	VGLL3	1.167302	3.990446	0.000102
ENSG0000(	RTN4R	1.166479	2.311707	0.004879
ENSG0000(	SPIN4	1.166264	2.792046	0.001614

ENSG0000(	AL358334.:	1.165726	2.595059	0.002541
ENSG0000(	GAL3ST4	1.165668	3.617109	0.000241
ENSG0000(	SPATA2L	1.165602	4.93852	1.15E-05
ENSG0000(	OLFML1	1.164775	4.659226	2.19E-05
ENSG0000(	PTGDR	1.164355	1.882147	0.013118
ENSG0000(	PRKACB	1.163873	9.613623	2.43E-10
ENSG0000(	RNU1-125F	1.162572	2.385411	0.004117
ENSG0000(	IFI44	1.162485	4.302976	4.98E-05
ENSG0000(	NFKB2	1.162386	8.122049	7.55E-09
ENSG0000(	LBX2-AS1	1.161797	3.34954	0.000447
ENSG0000(	MAN2B1	1.161761	8.118239	7.62E-09
ENSG0000(	FJX1	1.161509	4.519497	3.02E-05
ENSG0000(	EHD3	1.161468	6.655327	2.21E-07
ENSG0000(	ADAMTSL1	1.161054	2.104059	0.007869
ENSG0000(	AC097534.	1.160702	1.965706	0.010822
ENSG0000(	DDAH2	1.160485	5.447407	3.57E-06
ENSG0000(	PTGS1	1.159698	3.288205	0.000515
ENSG0000(	EFCAB7	1.158564	3.334057	0.000463
ENSG0000(	LRMP	1.158041	1.628444	0.023526
ENSG0000(	SPC24	1.157197	1.474567	0.03353
ENSG0000(	ADGRG6	1.156688	4.131378	7.39E-05
ENSG0000(	HIST1H2BK	1.156228	4.182631	6.57E-05
ENSG0000(	ACKR1	1.155514	5.331065	4.67E-06
ENSG0000(	COL3A1	1.155095	2.128147	0.007445
ENSG0000(	ADIRF	1.152255	3.039528	0.000913
ENSG0000(	MFAP5	1.151317	2.32339	0.004749
ENSG0000(	NPC2	1.151193	5.549122	2.82E-06
ENSG0000(	QPRT	1.151042	4.885384	1.30E-05
ENSG0000(	EZR	1.150349	11.90462	1.25E-12
ENSG0000(	PROCR	1.150338	1.948503	0.011259
ENSG0000(	TCEAL7	1.148858	3.148459	0.00071
ENSG0000(	COLEC12	1.148489	4.768234	1.71E-05
ENSG0000(	NAV2-AS1	1.148049	3.142108	0.000721
ENSG0000(	CBR3	1.147824	3.714953	0.000193
ENSG0000(	AL355310.:	1.14757	5.275033	5.31E-06
ENSG0000(	PCDHB15	1.147079	3.094055	0.000805
ENSG0000(	VAV3	1.146583	1.395072	0.040265
ENSG0000(	TNFRSF12A	1.146087	3.140116	0.000724
ENSG0000(	SCRN1	1.145898	3.686281	0.000206
ENSG0000(	AC003070.	1.14518	3.44891	0.000356
ENSG0000(	STMN1	1.14509	11.20906	6.18E-12
ENSG0000(	SH3BGRL3	1.144637	6.266959	5.41E-07
ENSG0000(	ALDOC	1.144619	2.854965	0.001396
ENSG0000(	AC144831.	1.1444	2.47465	0.003352
ENSG0000(	TNRC6C-AS	1.144097	5.990655	1.02E-06
ENSG0000(	EPHA3	1.143113	3.221059	0.000601
ENSG0000(	SFRP1	1.142265	2.448533	0.00356

ENSG0000(	AC079880.	1.14198	3.406674	0.000392
ENSG0000(	SFRP4	1.141304	1.87493	0.013337
ENSG0000(	AJUBA	1.141099	4.046222	8.99E-05
ENSG0000(	CD248	1.141078	2.753472	0.001764
ENSG0000(	PLAUR	1.140605	4.13808	7.28E-05
ENSG0000(	CD302	1.140398	4.593314	2.55E-05
ENSG0000(	RDH16	1.14011	1.347497	0.044927
ENSG0000(	MAML2	1.139986	5.456123	3.50E-06
ENSG0000(	WDFY2	1.139853	7.258936	5.51E-08
ENSG0000(	CDKN2B	1.139795	3.498	0.000318
ENSG0000(	ANPEP	1.139596	2.188983	0.006472
ENSG0000(	CELSR3	1.139364	4.341027	4.56E-05
ENSG0000(	MAP4K1	1.138598	5.37415	4.23E-06
ENSG0000(	STAT6	1.137467	8.109695	7.77E-09
ENSG0000(	GLCCI1	1.136777	6.777521	1.67E-07
ENSG0000(	ARHGDIB	1.13626	4.692595	2.03E-05
ENSG0000(	MANF	1.136079	5.493814	3.21E-06
ENSG0000(	A2ML1-AS1	1.135126	3.406674	0.000392
ENSG0000(	PIK3R6	1.134582	1.540009	0.02884
ENSG0000(	MMP19	1.134529	5.928682	1.18E-06
ENSG0000(	ADGRD1-A	1.134247	1.315883	0.048319
ENSG0000(	MFSD1	1.13405	6.659608	2.19E-07
ENSG0000(	CCDC65	1.132822	1.897969	0.012648
ENSG0000(	IL7	1.132586	6.780961	1.66E-07
ENSG0000(	FXYD5	1.132264	5.359873	4.37E-06
ENSG0000(	FANCD2	1.131875	4.112569	7.72E-05
ENSG0000(	LAP3	1.131543	8.770549	1.70E-09
ENSG0000(	MAFF	1.131438	4.340728	4.56E-05
ENSG0000(	AMPD2	1.130609	5.846168	1.43E-06
ENSG0000(	SMAP2	1.1305	6.333935	4.64E-07
ENSG0000(	CCDC152	1.129117	4.021621	9.51E-05
ENSG0000(	MIR7847	1.129012	5.713632	1.93E-06
ENSG0000(	RBBP8	1.128618	4.424158	3.77E-05
ENSG0000(	TMEM51	1.128508	3.27863	0.000526
ENSG0000(	CELF6	1.128477	2.659916	0.002188
ENSG0000(	DTX1	1.127453	2.987564	0.001029
ENSG0000(	NHSL1	1.126175	3.400255	0.000398
ENSG0000(	RABGAP1L	1.125926	6.234773	5.82E-07
ENSG0000(	AL157786.1	1.123542	3.719978	0.000191
ENSG0000(	IL32	1.123261	2.433606	0.003685
ENSG0000(	TCN2	1.122812	6.594129	2.55E-07
ENSG0000(	FZD10-DT	1.122666	4.486039	3.27E-05
ENSG0000(	LHFPL2	1.122501	6.536444	2.91E-07
ENSG0000(	KCNIP2-AS1	1.121676	1.607345	0.024698
ENSG0000(	SERPINB9	1.121662	5.656293	2.21E-06
ENSG0000(	AC010655.	1.121612	2.967803	0.001077
ENSG0000(	OLFML2A	1.120954	5.069944	8.51E-06

ENSG0000(	OLFM2	1.12077	2.78186	0.001652
ENSG0000(	MIR3917	1.12038	8.872611	1.34E-09
ENSG0000(	DLEU2L	1.120045	1.415608	0.038405
ENSG0000(	CYBC1	1.119486	8.118239	7.62E-09
ENSG0000(	C17orf67	1.119485	1.448829	0.035577
ENSG0000(	GSTM5	1.11926	3.452946	0.000352
ENSG0000(	SRPX	1.118284	4.481095	3.30E-05
ENSG0000(	ANTXR2	1.116993	5.751019	1.77E-06
ENSG0000(	PRDM8	1.115968	7.70712	1.96E-08
ENSG0000(	MYO5B	1.115963	1.416691	0.03831
ENSG0000(	TRIM5	1.115942	6.874556	1.33E-07
ENSG0000(	TENM4	1.115105	5.136014	7.31E-06
ENSG0000(	AC012181.	1.114276	2.114844	0.007676
ENSG0000(	PDE10A	1.113932	2.412238	0.00387
ENSG0000(	CCL15-CCL:	1.1138	3.535861	0.000291
ENSG0000(	SPATA13	1.113759	5.563174	2.73E-06
ENSG0000(	ABHD2	1.111317	9.382216	4.15E-10
ENSG0000(	PAX8-AS1	1.111076	1.537007	0.02904
ENSG0000(	AC010531.	1.109575	1.501599	0.031507
ENSG0000(	LDHB	1.109482	5.781466	1.65E-06
ENSG0000(	DNM3OS	1.108762	4.111592	7.73E-05
ENSG0000(	ERRFI1	1.108522	9.71053	1.95E-10
ENSG0000(	PXDNL	1.108314	2.054489	0.008821
ENSG0000(	AL355987.:	1.107584	1.529059	0.029576
ENSG0000(	SLC2A10	1.107417	3.523204	0.0003
ENSG0000(	LLGL2	1.106048	7.16376	6.86E-08
ENSG0000(	HTRA1	1.105326	4.060471	8.70E-05
ENSG0000(	HELZ2	1.105066	8.370816	4.26E-09
ENSG0000(	CAPN6	1.103524	2.854965	0.001396
ENSG0000(	AC020915.	1.103337	9.240098	5.75E-10
ENSG0000(	AC010197.	1.102745	5.766083	1.71E-06
ENSG0000(	NGFR	1.102684	2.558577	0.002763
ENSG0000(	XBP1	1.102443	5.732131	1.85E-06
ENSG0000(	RASSF1-AS	1.101904	6.858753	1.38E-07
ENSG0000(	ETV5	1.101602	3.813955	0.000153
ENSG0000(	FBXO2	1.100987	1.758123	0.017453
ENSG0000(	BCAS4	1.100589	3.136473	0.00073
ENSG0000(	ST14	1.100544	3.323314	0.000475
ENSG0000(	VDR	1.100511	5.147177	7.13E-06
ENSG0000(	IQCN	1.099229	4.832195	1.47E-05
ENSG0000(	CCL14	1.098114	3.551663	0.000281
ENSG0000(	OSTF1	1.097574	6.713385	1.93E-07
ENSG0000(	LSP1	1.097395	5.643196	2.27E-06
ENSG0000(	NBPF14	1.097197	6.505864	3.12E-07
ENSG0000(	PARP15	1.09678	4.673591	2.12E-05
ENSG0000(	LINC00205	1.096139	6.097592	7.99E-07
ENSG0000(	CASP17P	1.095566	4.668456	2.15E-05

ENSG0000(	ABLIM1	1.095378	5.018849	9.58E-06
ENSG0000(	CSDC2	1.095016	10.35462	4.42E-11
ENSG0000(	ADAM8	1.094666	1.994785	0.010121
ENSG0000(	GLMP	1.094506	6.808695	1.55E-07
ENSG0000(	KLF4	1.094458	3.451306	0.000354
ENSG0000(	CILP	1.094065	6.374679	4.22E-07
ENSG0000(	AC124068.	1.093345	8.747016	1.79E-09
ENSG0000(	CDC42SE1	1.092505	12.26608	5.42E-13
ENSG0000(	AL157838.:	1.092388	2.241093	0.00574
ENSG0000(	VILL	1.092296	7.611642	2.45E-08
ENSG0000(	AQP11	1.09227	2.251798	0.0056
ENSG0000(	MIR3606	1.091846	1.795715	0.016006
ENSG0000(	SNHG28	1.091675	5.141387	7.22E-06
ENSG0000(	ARRB2	1.090949	4.583705	2.61E-05
ENSG0000(	AC007382.	1.090801	1.629451	0.023472
ENSG0000(	AL137009.:	1.090753	3.196224	0.000636
ENSG0000(	LRRN3	1.08994	2.787645	0.001631
ENSG0000(	CYB561	1.088834	7.044904	9.02E-08
ENSG0000(	SEMA4A	1.088793	4.418316	3.82E-05
ENSG0000(	MIR3916	1.088468	1.484815	0.032748
ENSG0000(	FAM102A	1.08746	7.93038	1.17E-08
ENSG0000(	CDC14B	1.087318	10.05004	8.91E-11
ENSG0000(	LINC00337	1.087256	2.909917	0.001231
ENSG0000(	MOV10	1.087086	10.97875	1.05E-11
ENSG0000(	EFNA5	1.08652	2.352653	0.00444
ENSG0000(	AL139099.4	1.085138	2.811676	0.001543
ENSG0000(	RN7SL1	1.085138	2.811676	0.001543
ENSG0000(	ZNF8	1.084595	5.672376	2.13E-06
ENSG0000(	SINHCAF	1.083934	7.799112	1.59E-08
ENSG0000(	BAIAP2-DT	1.08389	5.522205	3.00E-06
ENSG0000(	GAB3	1.083146	6.489772	3.24E-07
ENSG0000(	DCN	1.082565	3.01133	0.000974
ENSG0000(	FN1	1.080608	3.556781	0.000277
ENSG0000(	ZNF610	1.079927	1.358302	0.043823
ENSG0000(	TNFRSF14-,	1.079565	6.483698	3.28E-07
ENSG0000(	FBXL2	1.079028	1.75301	0.01766
ENSG0000(	USP18	1.078357	6.157064	6.97E-07
ENSG0000(	TSPAN9	1.078246	7.677066	2.10E-08
ENSG0000(	STAMBPL1	1.077289	3.346121	0.000451
ENSG0000(	ITGA3	1.07706	6.315401	4.84E-07
ENSG0000(	NRSN2-AS1	1.0768	2.132528	0.00737
ENSG0000(	EML4	1.076788	9.309742	4.90E-10
ENSG0000(	CALHM2	1.07662	6.966977	1.08E-07
ENSG0000(	KLHL2	1.076502	6.044951	9.02E-07
ENSG0000(	AC093155.	1.076452	4.062439	8.66E-05
ENSG0000(	MICAL1	1.076373	6.425861	3.75E-07
ENSG0000(	ITGA9-AS1	1.076334	5.078796	8.34E-06

ENSG0000(	IL10RB	1.076251	7.081556	8.29E-08
ENSG0000(	SDCBP2	1.076134	1.487244	0.032565
ENSG0000(	CD27	1.075955	6.210945	6.15E-07
ENSG0000(	MANEA	1.074229	3.126837	0.000747
ENSG0000(	DOK3	1.074173	4.078272	8.35E-05
ENSG0000(	CEP152	1.073759	3.245744	0.000568
ENSG0000(	SNHG28	1.073507	4.443404	3.60E-05
ENSG0000(	TC2N	1.072307	3.178829	0.000662
ENSG0000(	AL022476.:	1.072137	1.752784	0.017669
ENSG0000(	CASK-AS1	1.071963	3.106053	0.000783
ENSG0000(	TRPV3	1.071731	2.200532	0.006302
ENSG0000(	DKK3	1.071539	3.384752	0.000412
ENSG0000(	NINL	1.071429	7.574751	2.66E-08
ENSG0000(	BICD1	1.070911	3.57834	0.000264
ENSG0000(	SQLE	1.070781	6.238765	5.77E-07
ENSG0000(	PLEKHA4	1.070068	5.86024	1.38E-06
ENSG0000(	BID	1.069919	13.04248	9.07E-14
ENSG0000(	RNASEL	1.068772	13.02976	9.34E-14
ENSG0000(	AC138028.	1.067893	4.310334	4.89E-05
ENSG0000(	ZNF888	1.067657	3.189703	0.000646
ENSG0000(	TNFRSF10A	1.067384	5.694621	2.02E-06
ENSG0000(	PIM1	1.067347	5.91631	1.21E-06
ENSG0000(	HENMT1	1.067026	4.132715	7.37E-05
ENSG0000(	SP110	1.066414	6.796539	1.60E-07
ENSG0000(	PDIA3	1.066118	9.173846	6.70E-10
ENSG0000(	LGALS8-AS	1.066047	1.492344	0.032185
ENSG0000(	MMP16	1.065923	1.610652	0.02451
ENSG0000(	WWTR1	1.065698	10.147	7.13E-11
ENSG0000(	Z84488.2	1.064951	3.75738	0.000175
ENSG0000(	LRMDA	1.064582	2.329758	0.00468
ENSG0000(	ZNF665	1.064167	1.605972	0.024776
ENSG0000(	KCNQ1	1.063897	3.225394	0.000595
ENSG0000(	SLC8A1	1.063846	5.330164	4.68E-06
ENSG0000(	RND2	1.063343	1.788376	0.016279
ENSG0000(	HILPDA	1.061232	3.126271	0.000748
ENSG0000(	SH3PXD2B	1.06049	4.512939	3.07E-05
ENSG0000(	AL358333.:	1.059933	5.51709	3.04E-06
ENSG0000(	DUSP18	1.059847	7.55474	2.79E-08
ENSG0000(	TRPA1	1.059451	1.846972	0.014224
ENSG0000(	DNMT1	1.05929	16.23482	5.82E-17
ENSG0000(	ZEB2	1.059099	7.341715	4.55E-08
ENSG0000(	SLC25A43	1.058833	5.623163	2.38E-06
ENSG0000(	DDX58	1.05867	11.64067	2.29E-12
ENSG0000(	PROSER2-A	1.058536	3.678381	0.00021
ENSG0000(	PLAU	1.058523	7.119778	7.59E-08
ENSG0000(	HELLPAR	1.058504	3.993522	0.000102
ENSG0000(	PAPLN	1.05814	3.515058	0.000305

ENSG0000(	UBXN11	1.058056	4.713214	1.94E-05
ENSG0000(	ADIRF-AS1	1.057913	3.092788	0.000808
ENSG0000(	MIR4680	1.056662	4.345082	4.52E-05
ENSG0000(	DSEL	1.056403	4.754778	1.76E-05
ENSG0000(	LRRCC1	1.05592	2.258732	0.005511
ENSG0000(	KIF7	1.055012	5.156296	6.98E-06
ENSG0000(	TNFRSF19	1.054906	4.226208	5.94E-05
ENSG0000(	STARD5	1.053685	3.512618	0.000307
ENSG0000(	FREM1	1.053475	3.026655	0.00094
ENSG0000(	IL17RA	1.053425	4.217989	6.05E-05
ENSG0000(	AC244100.	1.053256	3.356623	0.00044
ENSG0000(	PCDH7	1.053232	3.064905	0.000861
ENSG0000(	PRTFDC1	1.053148	3.786391	0.000164
ENSG0000(	DACT3	1.052564	3.914671	0.000122
ENSG0000(	RAB27A	1.052258	7.477828	3.33E-08
ENSG0000(	GADD45B	1.05224	5.173362	6.71E-06
ENSG0000(	JAZF1	1.051885	5.030002	9.33E-06
ENSG0000(	AC134043.	1.05183	1.635611	0.023141
ENSG0000(	TM4SF19-T	1.051248	4.931344	1.17E-05
ENSG0000(	AC110597.	1.050572	2.962429	0.00109
ENSG0000(	EZR-AS1	1.05007	5.938191	1.15E-06
ENSG0000(	TCIRG1	1.048776	5.495583	3.19E-06
ENSG0000(	ISLR	1.048513	3.963332	0.000109
ENSG0000(	TEX9	1.047231	1.337974	0.045923
ENSG0000(	ENO2	1.046835	7.436885	3.66E-08
ENSG0000(	AKR1C3	1.046238	2.826569	0.001491
ENSG0000(	ADNP2	1.046116	8.779816	1.66E-09
ENSG0000(	CLVS1	1.046019	2.928048	0.00118
ENSG0000(	SOAT1	1.045916	5.237579	5.79E-06
ENSG0000(	AC114490.	1.045395	3.51774	0.000304
ENSG0000(	AC126474.	1.045033	4.62555	2.37E-05
ENSG0000(	ZNF582-AS	1.044985	2.498379	0.003174
ENSG0000(	MEDAG	1.04481	2.343118	0.004538
ENSG0000(	AP000295.	1.044724	8.313968	4.85E-09
ENSG0000(	PFKP	1.044493	8.759394	1.74E-09
ENSG0000(	CADPS2	1.044339	5.647803	2.25E-06
ENSG0000(	JAM3	1.044112	6.245585	5.68E-07
ENSG0000(	AC234582.	1.043927	5.595304	2.54E-06
ENSG0000(	CHGB	1.042545	1.803776	0.015712
ENSG0000(	PAPSS1	1.042251	7.270286	5.37E-08
ENSG0000(	IGF1	1.041591	3.295718	0.000506
ENSG0000(	ANKRD44	1.041552	3.339924	0.000457
ENSG0000(	ZMAT3	1.041288	7.293557	5.09E-08
ENSG0000(	ITM2A	1.040225	4.096019	8.02E-05
ENSG0000(	AC080112.	1.039712	5.25337	5.58E-06
ENSG0000(	C1orf74	1.039665	5.089817	8.13E-06
ENSG0000(	CNPY3	1.038802	6.845602	1.43E-07

ENSG0000(	GPSM1	1.038641	8.790606	1.62E-09
ENSG0000(	INTS6L	1.038497	4.920281	1.20E-05
ENSG0000(	CASQ2	1.037142	4.706787	1.96E-05
ENSG0000(	PCOLCE-AS	1.036642	4.87952	1.32E-05
ENSG0000(	MSC-AS1	1.036636	4.945423	1.13E-05
ENSG0000(	GAS7	1.036445	3.743414	0.000181
ENSG0000(	SELENOP	1.036086	3.567618	0.000271
ENSG0000(	CDRT4	1.035151	9.555531	2.78E-10
ENSG0000(	PARP10	1.034934	12.06877	8.54E-13
ENSG0000(	ASNS	1.034528	7.318059	4.81E-08
ENSG0000(	AC244669.	1.033948	2.099932	0.007945
ENSG0000(	NECAP2	1.033663	6.447989	3.56E-07
ENSG0000(	AL157871.4	1.03291	3.84099	0.000144
ENSG0000(	LINC00672	1.032538	1.458849	0.034766
ENSG0000(	SPSB4	1.03178	4.664807	2.16E-05
ENSG0000(	EHD4	1.031646	7.58168	2.62E-08
ENSG0000(	PLP1	1.031413	1.517619	0.030366
ENSG0000(	AC114490.	1.031072	5.326672	4.71E-06
ENSG0000(	FAT1	1.030901	4.846064	1.43E-05
ENSG0000(	MFSD4A	1.030289	2.569462	0.002695
ENSG0000(	OSR1	1.030225	3.44339	0.00036
ENSG0000(	BST1	1.030047	3.158398	0.000694
ENSG0000(	SLC2A3	1.029984	3.763309	0.000172
ENSG0000(	RIMS3	1.029372	2.747254	0.00179
ENSG0000(	AC091814.	1.028956	3.573279	0.000267
ENSG0000(	RNASE4	1.02872	2.238905	0.005769
ENSG0000(	NEK11	1.028463	3.547302	0.000284
ENSG0000(	PRAL	1.027989	4.604992	2.48E-05
ENSG0000(	SLC37A1	1.027894	11.32241	4.76E-12
ENSG0000(	TRADD	1.027539	10.82735	1.49E-11
ENSG0000(	MAPK13	1.027514	5.957073	1.10E-06
ENSG0000(	TNFSF10	1.027453	6.471344	3.38E-07
ENSG0000(	SPTBN2	1.027408	7.231325	5.87E-08
ENSG0000(	MMEL1	1.027112	3.329503	0.000468
ENSG0000(	ARHGAP20	1.026594	1.586551	0.025909
ENSG0000(	SETDB2	1.026557	5.997119	1.01E-06
ENSG0000(	FTL	1.025874	5.357931	4.39E-06
ENSG0000(	FRZB	1.025544	1.821003	0.015101
ENSG0000(	IRAK4	1.025384	6.64435	2.27E-07
ENSG0000(	AC010768.	1.025371	4.713292	1.94E-05
ENSG0000(	CTSB	1.024486	7.595801	2.54E-08
ENSG0000(	DDX60L	1.024347	5.067642	8.56E-06
ENSG0000(	PAK3	1.024332	1.752087	0.017698
ENSG0000(	SLC38A1	1.024294	2.20807	0.006193
ENSG0000(	THBS3	1.023592	6.213825	6.11E-07
ENSG0000(	GPR173	1.023428	6.756125	1.75E-07
ENSG0000(	PHTF1	1.02337	6.477266	3.33E-07

ENSG0000(	CES4A	1.023161	3.173232	0.000671
ENSG0000(	EVC	1.022889	5.952543	1.12E-06
ENSG0000(	CHFR	1.022235	12.77414	1.68E-13
ENSG0000(	RPL39L	1.022056	2.175647	0.006673
ENSG0000(	CLIC4	1.021532	10.02185	9.51E-11
ENSG0000(	AC108449.	1.021192	6.632965	2.33E-07
ENSG0000(	TPBG	1.021027	5.45682	3.49E-06
ENSG0000(	SUSD2	1.020281	3.808607	0.000155
ENSG0000(	ZBTB22	1.020096	10.44119	3.62E-11
ENSG0000(	TNFRSF25	1.020078	4.787742	1.63E-05
ENSG0000(	ARID3A	1.020021	6.526044	2.98E-07
ENSG0000(	MX1	1.019744	3.548612	0.000283
ENSG0000(	RPL22L1	1.019732	2.322109	0.004763
ENSG0000(	C10orf55	1.01968	5.614982	2.43E-06
ENSG0000(	KCNK15	1.019668	1.662037	0.021775
ENSG0000(	LINC00910	1.019529	5.963599	1.09E-06
ENSG0000(	PTK7	1.019518	3.239855	0.000576
ENSG0000(	AL163636.:	1.019127	2.195687	0.006373
ENSG0000(	AP003392.	1.019074	10.57881	2.64E-11
ENSG0000(	AC133644.	1.019034	6.42231	3.78E-07
ENSG0000(	OTUD1	1.019001	1.543318	0.028621
ENSG0000(	SRGAP2	1.018495	9.27851	5.27E-10
ENSG0000(	KCND2	1.017871	1.744438	0.018012
ENSG0000(	CCDC71L	1.017395	5.706681	1.96E-06
ENSG0000(	FAM111A-I	1.017333	2.980499	0.001046
ENSG0000(	LPAR6	1.017016	3.525149	0.000298
ENSG0000(	MXRA8	1.015872	4.930337	1.17E-05
ENSG0000(	TMEM144	1.015455	7.160771	6.91E-08
ENSG0000(	DHX58	1.015144	5.557938	2.77E-06
ENSG0000(	TAGLN2	1.013283	4.443642	3.60E-05
ENSG0000(	PREX1	1.012747	5.601064	2.51E-06
ENSG0000(	U62317.2	1.011814	8.382129	4.15E-09
ENSG0000(	SLC16A6	1.010443	1.371295	0.042531
ENSG0000(	GJD3	1.009224	2.233574	0.00584
ENSG0000(	CDC42SE2	1.009174	6.019429	9.56E-07
ENSG0000(	NCAPH	1.009066	1.431871	0.036994
ENSG0000(	SULF1	1.009038	3.107886	0.00078
ENSG0000(	ZNF503	1.008866	3.316277	0.000483
ENSG0000(	CLEC11A	1.008651	3.01774	0.00096
ENSG0000(	COL5A2	1.008603	3.157722	0.000695
ENSG0000(	RCN1P2	1.006778	6.359724	4.37E-07
ENSG0000(	ST6GAL1	1.006338	5.953398	1.11E-06
ENSG0000(	SEC23B	1.00554	7.665221	2.16E-08
ENSG0000(	RHPN1	1.005537	4.48306	3.29E-05
ENSG0000(	MTX1P1	1.005343	2.178878	0.006624
ENSG0000(	NMNAT2	1.005004	3.705997	0.000197
ENSG0000(	PARP4	1.00468	9.475687	3.34E-10

ENSG0000(	TMEM241	1.004619	2.977134	0.001054
ENSG0000(	MAP3K8	1.004543	5.498233	3.18E-06
ENSG0000(	ARMC9	1.004245	6.243058	5.71E-07
ENSG0000(	STK4	1.004077	7.101729	7.91E-08
ENSG0000(	FBXO17	1.003754	3.802013	0.000158
ENSG0000(	FBN1	1.003123	1.6751	0.02113
ENSG0000(	AC084125.	1.002798	1.847686	0.014201
ENSG0000(	MEIS3	1.002506	5.301782	4.99E-06
ENSG0000(	CCDC146	1.002388	4.602983	2.49E-05
ENSG0000(	EEF1AKMT	1.002283	1.953855	0.011121
ENSG0000(	CRACR2A	1.002273	3.575803	0.000266
ENSG0000(	SLC8A1-AS	1.001829	3.60414	0.000249
ENSG0000(	IRAK2	1.001795	6.510526	3.09E-07
ENSG0000(	LINC00924	1.000873	2.276012	0.005296
ENSG0000(	GPSM3	1.000242	4.73724	1.83E-05
ENSG0000(	ZEB2-AS1	0.999684	1.300075	0.05011
ENSG0000(	NOTCH2	0.999668	4.730248	1.86E-05
ENSG0000(	PSRC1	0.998873	1.469936	0.033889
ENSG0000(	IFFO2	0.997883	8.581193	2.62E-09
ENSG0000(	MIR3658	0.997769	2.489075	0.003243
ENSG0000(	PCDHB14	0.997549	3.452005	0.000353
ENSG0000(	ZFP36L2	0.996751	8.769313	1.70E-09
ENSG0000(	KLRG1	0.996157	3.217297	0.000606
ENSG0000(	AC104134.	0.99588	3.130936	0.00074
ENSG0000(	PLAC9	0.995624	2.905125	0.001244
ENSG0000(	AC015795.	0.995619	4.826983	1.49E-05
ENSG0000(	ETNK2	0.994692	1.851612	0.014073
ENSG0000(	MMP11	0.993606	2.502278	0.003146
ENSG0000(	SDC3	0.993551	4.953564	1.11E-05
ENSG0000(	RHBDF2	0.993356	5.88721	1.30E-06
ENSG0000(	CRABP2	0.99272	5.29437	5.08E-06
ENSG0000(	GRN	0.992205	8.595168	2.54E-09
ENSG0000(	BEX4	0.991891	4.797482	1.59E-05
ENSG0000(	EDEM2	0.991371	5.498915	3.17E-06
ENSG0000(	SLCO3A1	0.991326	8.601845	2.50E-09
ENSG0000(	B3GNT9	0.99113	14.58859	2.58E-15
ENSG0000(	AQP3	0.990234	2.836018	0.001459
ENSG0000(	AK4	0.990027	2.67977	0.00209
ENSG0000(	AOC3	0.989748	1.499667	0.031647
ENSG0000(	HSP90B1	0.989034	4.914991	1.22E-05
ENSG0000(	SSC5D	0.988807	2.814869	0.001532
ENSG0000(	ARSJ	0.988499	1.54025	0.028824
ENSG0000(	PLSCR1	0.988378	5.159153	6.93E-06
ENSG0000(	DNMT3B	0.988071	4.634362	2.32E-05
ENSG0000(	ZWINT	0.987825	2.29831	0.005031
ENSG0000(	MAP1LC3C	0.987534	1.376387	0.042035
ENSG0000(	IMPDH1P1	0.987426	5.386269	4.11E-06

ENSG0000(	CTTNBP2	0.987272	1.377197	0.041957
ENSG0000(	LCTL	0.985872	2.870835	0.001346
ENSG0000(	CMIP	0.985618	6.532189	2.94E-07
ENSG0000(	ZNF497	0.985509	7.380493	4.16E-08
ENSG0000(	TKT	0.985377	2.421048	0.003793
ENSG0000(	MBOAT2	0.985239	4.25967	5.50E-05
ENSG0000(	CASP3	0.984035	5.674047	2.12E-06
ENSG0000(	ARSB	0.983837	6.504786	3.13E-07
ENSG0000(	PDIA4	0.983345	8.925354	1.19E-09
ENSG0000(	AGPAT4	0.982858	5.997937	1.00E-06
ENSG0000(	FUCA2	0.982468	4.753156	1.77E-05
ENSG0000(	VAT1	0.981815	4.274738	5.31E-05
ENSG0000(	SLFN12	0.981652	3.13402	0.000734
ENSG0000(	AC016876.	0.981552	6.709237	1.95E-07
ENSG0000(	ARF3	0.98118	6.717028	1.92E-07
ENSG0000(	TTC39A	0.980725	3.808607	0.000155
ENSG0000(	AZIN2	0.979881	6.540181	2.88E-07
ENSG0000(	UNC119	0.979524	9.534226	2.92E-10
ENSG0000(	MYCN	0.979385	1.529241	0.029564
ENSG0000(	MAP9	0.978825	1.623658	0.023787
ENSG0000(	AC063952.	0.978409	2.001792	0.009959
ENSG0000(	IQCG	0.978204	4.352642	4.44E-05
ENSG0000(	AC105285.	0.977905	1.33252	0.046503
ENSG0000(	MMP25-AS	0.977821	2.451434	0.003536
ENSG0000(	AC010542.	0.977703	3.352571	0.000444
ENSG0000(	ZSWIM4	0.977687	9.835567	1.46E-10
ENSG0000(	SLC6A9	0.977169	3.360055	0.000436
ENSG0000(	P3H3	0.976972	3.709138	0.000195
ENSG0000(	CSK	0.976951	7.393277	4.04E-08
ENSG0000(	AC008440.	0.975688	4.964817	1.08E-05
ENSG0000(	AC245060.	0.975111	2.2337	0.005838
ENSG0000(	GMDS	0.974956	10.26738	5.40E-11
ENSG0000(	ZNF503-AS	0.974746	2.998502	0.001003
ENSG0000(	SLC36A1	0.974619	5.118493	7.61E-06
ENSG0000(	TES	0.974196	6.692786	2.03E-07
ENSG0000(	MYADM	0.973824	5.606044	2.48E-06
ENSG0000(	CLSPN	0.973733	1.563399	0.027328
ENSG0000(	STAB1	0.973461	3.404588	0.000394
ENSG0000(	NTNG2	0.973362	1.611498	0.024463
ENSG0000(	TSTD1	0.972788	7.247984	5.65E-08
ENSG0000(	PABPC1L	0.972447	5.078796	8.34E-06
ENSG0000(	AC004824.	0.972087	3.520103	0.000302
ENSG0000(	MGAT2	0.972032	7.219152	6.04E-08
ENSG0000(	LINC00467	0.97182	5.464039	3.44E-06
ENSG0000(	CBR3-AS1	0.97103	3.446174	0.000358
ENSG0000(	KCNIP2	0.970887	1.328369	0.046949
ENSG0000(	PROSER2	0.970198	4.235681	5.81E-05

ENSG0000(	ALDH18A1	0.969948	8.578531	2.64E-09
ENSG0000(	POU4F1	0.96955	1.536442	0.029078
ENSG0000(	MIR671	0.969252	3.345997	0.000451
ENSG0000(	MIS18BP1	0.968182	4.050453	8.90E-05
ENSG0000(	AC053503.	0.967936	1.590744	0.02566
ENSG0000(	TDRD7	0.967849	11.00594	9.86E-12
ENSG0000(	ARHGAP28	0.967844	4.142311	7.21E-05
ENSG0000(	BST2	0.967097	4.974206	1.06E-05
ENSG0000(	CRMP1	0.966626	3.359287	0.000437
ENSG0000(	AP001972.	0.966391	4.455047	3.51E-05
ENSG0000(	AC073610.	0.966142	5.949469	1.12E-06
ENSG0000(	WARS	0.965788	4.199403	6.32E-05
ENSG0000(	LAT2	0.965632	3.069442	0.000852
ENSG0000(	AL139099.:	0.965351	7.070366	8.50E-08
ENSG0000(	FYN	0.964701	5.373259	4.23E-06
ENSG0000(	MARCKS	0.963576	4.879987	1.32E-05
ENSG0000(	TIMP2	0.963532	2.701244	0.00199
ENSG0000(	TMEM98	0.962916	3.417694	0.000382
ENSG0000(	FUT4	0.962762	7.44013	3.63E-08
ENSG0000(	FEZ1	0.962527	4.192126	6.43E-05
ENSG0000(	ALCAM	0.961832	3.280517	0.000524
ENSG0000(	CD82	0.961394	4.699136	2.00E-05
ENSG0000(	CDK20	0.961137	3.963332	0.000109
ENSG0000(	PCLAF	0.961011	1.326672	0.047133
ENSG0000(	ZC4H2	0.960685	5.300746	5.00E-06
ENSG0000(	TNS3	0.960612	10.27391	5.32E-11
ENSG0000(	IL33	0.960585	3.644987	0.000226
ENSG0000(	AC010422.	0.960336	5.689636	2.04E-06
ENSG0000(	AC092143.	0.960083	1.734297	0.018438
ENSG0000(	AC004846.	0.95862	2.248645	0.005641
ENSG0000(	AC068385.	0.958437	2.234951	0.005822
ENSG0000(	SH3BP5	0.958296	8.193011	6.41E-09
ENSG0000(	MARVELD1	0.957369	3.925014	0.000119
ENSG0000(	LRRC1	0.957364	2.377421	0.004194
ENSG0000(	AP1G2	0.957301	5.008808	9.80E-06
ENSG0000(	SEC14L2	0.956636	3.116064	0.000765
ENSG0000(	AC016876.	0.95652	3.028331	0.000937
ENSG0000(	RGMB-AS1	0.956044	1.375596	0.042112
ENSG0000(	LILRB3	0.955631	1.785795	0.016376
ENSG0000(	AC138028.	0.955582	5.153411	7.02E-06
ENSG0000(	RAB34	0.955289	4.272237	5.34E-05
ENSG0000(	GSN	0.955002	4.455477	3.50E-05
ENSG0000(	CPQ	0.954961	4.034616	9.23E-05
ENSG0000(	PCNX2	0.954734	7.11668	7.64E-08
ENSG0000(	HSD17B11	0.954605	3.220013	0.000603
ENSG0000(	FZD10	0.954344	2.103094	0.007887
ENSG0000(	TMEM198	0.954228	6.076464	8.39E-07

ENSG0000(	CPNE8	0.954192	4.833391	1.47E-05
ENSG0000(	BTG1	0.953983	6.479167	3.32E-07
ENSG0000(	SGCE	0.95394	4.216281	6.08E-05
ENSG0000(	PLSCR4	0.953919	3.036696	0.000919
ENSG0000(	PPP1R3G	0.953745	4.46898	3.40E-05
ENSG0000(	ANXA5	0.953741	5.604936	2.48E-06
ENSG0000(	AP000943.	0.953698	4.832195	1.47E-05
ENSG0000(	LRFN4	0.953538	5.653192	2.22E-06
ENSG0000(	AL359762.:	0.952933	1.933581	0.011653
ENSG0000(	CDC25B	0.952716	6.26165	5.47E-07
ENSG0000(	AL355001.:	0.952632	4.169008	6.78E-05
ENSG0000(	NRXN2	0.952566	6.188616	6.48E-07
ENSG0000(	CDCA4	0.952457	8.916817	1.21E-09
ENSG0000(	USP6NL	0.951716	3.300104	0.000501
ENSG0000(	PIEZO1	0.951298	5.976932	1.05E-06
ENSG0000(	FKBP10	0.951288	4.828287	1.48E-05
ENSG0000(	HMGB3	0.951113	5.197301	6.35E-06
ENSG0000(	AL135999.:	0.950566	4.827468	1.49E-05
ENSG0000(	APOBR	0.950435	1.64418	0.022689
ENSG0000(	C2orf27A	0.950155	2.454993	0.003508
ENSG0000(	PLEKHA6	0.949986	4.971628	1.07E-05
ENSG0000(	PHF11	0.949507	6.443632	3.60E-07
ENSG0000(	ATCAY	0.949431	2.905584	0.001243
ENSG0000(	POC1A	0.949417	1.591238	0.025631
ENSG0000(	ICMT	0.949372	3.621196	0.000239
ENSG0000(	AC108516.	0.948826	3.050581	0.00089
ENSG0000(	ARHGAP24	0.948745	5.560455	2.75E-06
ENSG0000(	MYLK	0.94832	4.482006	3.30E-05
ENSG0000(	AP005205.	0.947646	2.675319	0.002112
ENSG0000(	TBC1D9	0.947432	4.360625	4.36E-05
ENSG0000(	SRGAP1	0.947041	4.996124	1.01E-05
ENSG0000(	ABI3	0.946155	3.610608	0.000245
ENSG0000(	IL15RA	0.945979	6.593966	2.55E-07
ENSG0000(	AC093797.	0.945967	1.393018	0.040456
ENSG0000(	CEP126	0.945142	2.213374	0.006118
ENSG0000(	SELP	0.944938	1.954358	0.011108
ENSG0000(	DBN1	0.944844	4.719737	1.91E-05
ENSG0000(	AC024909.	0.944628	2.876056	0.00133
ENSG0000(	GM2A	0.944528	6.422477	3.78E-07
ENSG0000(	MCTP1	0.943566	3.607134	0.000247
ENSG0000(	CAMKK1	0.943364	3.703282	0.000198
ENSG0000(	XAF1	0.942911	4.197976	6.34E-05
ENSG0000(	PPT1	0.942847	6.756502	1.75E-07
ENSG0000(	AC020659.	0.942237	3.176394	0.000666
ENSG0000(	AP001610.	0.942099	2.814834	0.001532
ENSG0000(	AL136295.!	0.942024	7.216086	6.08E-08
ENSG0000(	ANKRD13D	0.941237	6.423391	3.77E-07

ENSG0000(	FLVCR2	0.940069	5.348114	4.49E-06
ENSG0000(	CD40	0.94004	6.595844	2.54E-07
ENSG0000(	AC007182.	0.939397	2.233574	0.00584
ENSG0000(	SERTAD4	0.938955	2.591019	0.002564
ENSG0000(	NKD1	0.938188	1.830672	0.014768
ENSG0000(	SLC4A11	0.937689	2.178389	0.006631
ENSG0000(	AC092070.	0.936933	4.157721	6.95E-05
ENSG0000(	AP000781.	0.93678	5.035766	9.21E-06
ENSG0000(	IFITM3	0.936608	5.319262	4.79E-06
ENSG0000(	PLCG2	0.93575	3.658897	0.000219
ENSG0000(	LTB4R	0.93568	6.64435	2.27E-07
ENSG0000(	NRP2	0.935386	3.497008	0.000318
ENSG0000(	TBCEL	0.934957	5.719549	1.91E-06
ENSG0000(	NHS	0.934047	1.801051	0.015811
ENSG0000(	UST	0.933734	3.428397	0.000373
ENSG0000(	NAALADL1	0.933282	2.991641	0.001019
ENSG0000(	LIMA1	0.932686	5.194194	6.39E-06
ENSG0000(	SLC43A3	0.932553	5.127653	7.45E-06
ENSG0000(	AC008676.	0.931866	2.094915	0.008037
ENSG0000(	AC007192.	0.931799	5.841799	1.44E-06
ENSG0000(	PROSER3	0.93157	7.386694	4.10E-08
ENSG0000(	FAM214B	0.931532	10.9538	1.11E-11
ENSG0000(	SAMD5	0.931493	1.891496	0.012838
ENSG0000(	TMEM243	0.931231	3.586681	0.000259
ENSG0000(	ADGRD1	0.930406	2.595265	0.002539
ENSG0000(	CAB39L	0.930158	3.546718	0.000284
ENSG0000(	RAB29	0.929734	8.404047	3.94E-09
ENSG0000(	MYZAP	0.929275	2.385955	0.004112
ENSG0000(	TRAF3	0.929139	6.698224	2.00E-07
ENSG0000(	S100A16	0.929029	9.207745	6.20E-10
ENSG0000(	TMEM87B	0.928887	5.667275	2.15E-06
ENSG0000(	SRGAP2B	0.928883	3.748859	0.000178
ENSG0000(	ADD3	0.92842	3.064905	0.000861
ENSG0000(	SLFN11	0.928118	6.754901	1.76E-07
ENSG0000(	NBDY	0.927615	5.91722	1.21E-06
ENSG0000(	TRIQK	0.926323	3.683822	0.000207
ENSG0000(	SPOCK1	0.925756	2.34763	0.004491
ENSG0000(	CYP2J2	0.925676	3.260834	0.000548
ENSG0000(	PNMA8B	0.925178	1.68628	0.020593
ENSG0000(	AC008894.	0.924219	6.29967	5.02E-07
ENSG0000(	SHISA5	0.924	7.365517	4.31E-08
ENSG0000(	TLR5	0.92379	4.604685	2.48E-05
ENSG0000(	OAF	0.922458	4.821579	1.51E-05
ENSG0000(	TIFA	0.921709	4.070632	8.50E-05
ENSG0000(	NUMBL	0.921621	7.122756	7.54E-08
ENSG0000(	ATP1A1-AS	0.921457	7.22505	5.96E-08
ENSG0000(	TRIM6	0.921013	1.758471	0.017439

ENSG0000(	AL033528.:	0.920617	2.929246	0.001177
ENSG0000(	SHKBP1	0.920077	5.565513	2.72E-06
ENSG0000(	ZNF28	0.919046	2.964413	0.001085
ENSG0000(	IFNAR2	0.918908	8.012927	9.71E-09
ENSG0000(	RNF227	0.918733	5.233047	5.85E-06
ENSG0000(	AP003071.	0.918647	1.503667	0.031357
ENSG0000(	CCDC122	0.918452	1.661884	0.021783
ENSG0000(	AC005786.	0.91818	4.987458	1.03E-05
ENSG0000(	MAN2B2	0.917932	5.114985	7.67E-06
ENSG0000(	OSBPL3	0.91769	2.599455	0.002515
ENSG0000(	GLYCTK-AS	0.917173	2.190153	0.006454
ENSG0000(	AC093864.	0.916759	1.351712	0.044493
ENSG0000(	HIST2H2BD	0.916023	2.107124	0.007814
ENSG0000(	G6PD	0.915963	5.288428	5.15E-06
ENSG0000(	AC027097.	0.915061	4.385395	4.12E-05
ENSG0000(	EDEM1	0.915	7.142706	7.20E-08
ENSG0000(	TCTEX1D2	0.914545	3.367837	0.000429
ENSG0000(	SMPD2	0.914318	4.045058	9.01E-05
ENSG0000(	SLC39A10	0.914068	4.731792	1.85E-05
ENSG0000(	AL160236.:	0.913621	1.948622	0.011256
ENSG0000(	NRK	0.913167	1.723024	0.018922
ENSG0000(	GLB1L	0.91271	2.157955	0.006951
ENSG0000(	NFATC2	0.912647	4.0619	8.67E-05
ENSG0000(	ODF2-AS1	0.912635	4.113109	7.71E-05
ENSG0000(	STARD4	0.912525	3.822523	0.00015
ENSG0000(	NCOA7	0.91241	4.13169	7.38E-05
ENSG0000(	DNA2	0.912055	2.476902	0.003335
ENSG0000(	AL445183.:	0.911321	2.207305	0.006204
ENSG0000(	AC007728.	0.911042	2.347076	0.004497
ENSG0000(	GPC6	0.910535	2.413496	0.003859
ENSG0000(	FAT2	0.910467	1.467764	0.034059
ENSG0000(	SLC30A1	0.909796	4.30212	4.99E-05
ENSG0000(	PCDHB10	0.909118	1.7945	0.016051
ENSG0000(	CKLF	0.908472	3.792694	0.000161
ENSG0000(	AC020913.	0.908312	3.78531	0.000164
ENSG0000(	C5	0.908056	1.52953	0.029544
ENSG0000(	ADAMTS7	0.907989	4.050453	8.90E-05
ENSG0000(	PREPL	0.907923	4.604685	2.48E-05
ENSG0000(	STK10	0.907826	3.533398	0.000293
ENSG0000(	ARMC2	0.907772	3.078994	0.000834
ENSG0000(	PPL	0.907219	2.738508	0.001826
ENSG0000(	GALNT7	0.906507	5.275173	5.31E-06
ENSG0000(	ELOVL6	0.905904	2.35826	0.004383
ENSG0000(	PRCP	0.905561	3.837243	0.000145
ENSG0000(	ITGA9	0.905008	3.430892	0.000371
ENSG0000(	MIR1244-1	0.904935	4.568094	2.70E-05
ENSG0000(	PCYOX1L	0.90489	4.619848	2.40E-05

ENSG0000(	TMEM79	0.904096	6.894722	1.27E-07
ENSG0000(	GALNT16	0.903524	2.358495	0.00438
ENSG0000(	NEGR1	0.903478	2.391483	0.00406
ENSG0000(	AC012435.	0.90321	1.4326	0.036932
ENSG0000(	DUSP6	0.902926	2.968646	0.001075
ENSG0000(	MBOAT1	0.902427	2.808309	0.001555
ENSG0000(	DKK2	0.902233	1.703392	0.019797
ENSG0000(	KMO	0.902152	4.728068	1.87E-05
ENSG0000(	HACD2	0.901935	2.617985	0.00241
ENSG0000(	ATP1B3	0.901669	8.207236	6.21E-09
ENSG0000(	LARP6	0.901151	7.039108	9.14E-08
ENSG0000(	KIAA1549	0.900567	2.61312	0.002437
ENSG0000(	PRELID1	0.89914	6.264453	5.44E-07
ENSG0000(	AC004922.	0.898745	8.702836	1.98E-09
ENSG0000(	CLEC3B	0.898714	2.352886	0.004437
ENSG0000(	ARAP1-AS1	0.898445	6.146363	7.14E-07
ENSG0000(	AC007750.	0.898257	2.135643	0.007317
ENSG0000(	LPCAT2	0.897995	4.012525	9.72E-05
ENSG0000(	ANG	0.897416	1.755677	0.017552
ENSG0000(	SPATA6L	0.897245	2.044189	0.009033
ENSG0000(	CD47	0.897107	6.600684	2.51E-07
ENSG0000(	CERCAM	0.896318	3.84025	0.000144
ENSG0000(	CNDP2	0.895944	9.059594	8.72E-10
ENSG0000(	RIN3	0.895451	4.928837	1.18E-05
ENSG0000(	MYO9B	0.894951	6.827954	1.49E-07
ENSG0000(	AL451164.:	0.893854	4.810911	1.55E-05
ENSG0000(	PTBP3	0.893752	6.957904	1.10E-07
ENSG0000(	SCARA5	0.893706	2.018296	0.009587
ENSG0000(	IL27RA	0.893451	3.611332	0.000245
ENSG0000(	PRKCZ-AS1	0.892924	2.045764	0.009
ENSG0000(	SLC3A1	0.892469	4.669042	2.14E-05
ENSG0000(	NFKBIZ	0.892069	5.376609	4.20E-06
ENSG0000(	SVEP1	0.891986	2.323526	0.004748
ENSG0000(	UCK2	0.891895	4.966023	1.08E-05
ENSG0000(	F3	0.891536	3.369924	0.000427
ENSG0000(	TMEM206	0.891339	4.138939	7.26E-05
ENSG0000(	FSTL3	0.89127	4.046928	8.98E-05
ENSG0000(	AC073610.	0.891145	5.744818	1.80E-06
ENSG0000(	CPLANE2	0.890978	1.315702	0.048339
ENSG0000(	ZNF788P	0.890686	2.923918	0.001191
ENSG0000(	ITPR1	0.890513	7.801399	1.58E-08
ENSG0000(	AL118506.:	0.890213	5.179244	6.62E-06
ENSG0000(	AL358115.:	0.890104	4.194522	6.39E-05
ENSG0000(	PAQR7	0.889961	10.98166	1.04E-11
ENSG0000(	COL6A6	0.889607	1.377501	0.041928
ENSG0000(	UGDH	0.889023	4.021621	9.51E-05
ENSG0000(	SCO2	0.887983	6.754741	1.76E-07

ENSG0000(	SMPD3	0.887949	3.046237	0.000899
ENSG0000(	FAM222A	0.887371	2.039303	0.009135
ENSG0000(	TWF1	0.887291	3.753683	0.000176
ENSG0000(	RAI14	0.887274	3.746727	0.000179
ENSG0000(	AC027097.	0.887026	3.42847	0.000373
ENSG0000(	AC011603.	0.885964	6.734169	1.84E-07
ENSG0000(	OLFML2B	0.885455	2.953973	0.001112
ENSG0000(	SNORA63	0.884838	1.691484	0.020348
ENSG0000(	TNFRSF10E	0.884465	10.60875	2.46E-11
ENSG0000(	PSD3	0.883841	2.375075	0.004216
ENSG0000(	PLXNC1	0.883805	2.713424	0.001935
ENSG0000(	FGR	0.883604	1.774268	0.016816
ENSG0000(	PKD2	0.883132	4.240119	5.75E-05
ENSG0000(	TUBA1B	0.883069	6.699613	2.00E-07
ENSG0000(	PDE1B	0.882935	2.239237	0.005765
ENSG0000(	NBEAL2	0.882542	4.961386	1.09E-05
ENSG0000(	AC109992.	0.882427	2.239772	0.005757
ENSG0000(	SLC16A4	0.882346	3.542089	0.000287
ENSG0000(	PDXK	0.88216	5.80335	1.57E-06
ENSG0000(	ZNF117	0.882141	2.498423	0.003174
ENSG0000(	ZFP69	0.881556	3.074451	0.000842
ENSG0000(	ABHD16B	0.881026	4.964817	1.08E-05
ENSG0000(	TBX18	0.88089	2.070457	0.008502
ENSG0000(	TPP1	0.878755	7.99715	1.01E-08
ENSG0000(	HSPA13	0.878727	2.752886	0.001767
ENSG0000(	NTN1	0.878694	1.501397	0.031521
ENSG0000(	PIGCP1	0.877544	3.456594	0.000349
ENSG0000(	AC092115.	0.877524	1.973217	0.010636
ENSG0000(	LINC00342	0.877268	3.726634	0.000188
ENSG0000(	GIMAP5	0.876372	5.077232	8.37E-06
ENSG0000(	PHETA2	0.875787	3.356004	0.000441
ENSG0000(	TDP1	0.87558	5.992265	1.02E-06
ENSG0000(	RNF19B	0.875394	8.378711	4.18E-09
ENSG0000(	CAP1	0.874894	4.970696	1.07E-05
ENSG0000(	KCNH2	0.874487	1.523533	0.029955
ENSG0000(	OXCT1	0.874309	2.406557	0.003921
ENSG0000(	CARD14	0.874162	5.291127	5.12E-06
ENSG0000(	IGFBP4	0.873623	3.497008	0.000318
ENSG0000(	TFPI	0.873452	4.987458	1.03E-05
ENSG0000(	TSPAN4	0.873028	6.107286	7.81E-07
ENSG0000(	MAP3K5	0.872489	5.349479	4.47E-06
ENSG0000(	BMP1	0.872406	6.00061	9.99E-07
ENSG0000(	KIF23	0.872342	1.343172	0.045376
ENSG0000(	NANS	0.871867	5.563277	2.73E-06
ENSG0000(	HS6ST2	0.871635	2.234951	0.005822
ENSG0000(	LINC02202	0.871588	1.974614	0.010602
ENSG0000(	KLF5	0.870791	3.149934	0.000708

ENSG0000(	SHOX2	0.870546	2.569544	0.002694
ENSG0000(	SLC25A5	0.870071	7.780356	1.66E-08
ENSG0000(	IL17RD	0.870009	2.180204	0.006604
ENSG0000(	GOLM1	0.869913	8.131093	7.39E-09
ENSG0000(	ARHGEF1	0.869264	5.23512	5.82E-06
ENSG0000(	ANLN	0.869242	2.159596	0.006925
ENSG0000(	AURKB	0.869205	2.142848	0.007197
ENSG0000(	MMD	0.868428	1.400921	0.039726
ENSG0000(	CKLF-CMTN	0.868332	3.805776	0.000156
ENSG0000(	ADM5	0.867468	1.618329	0.024081
ENSG0000(	AL390728.(	0.86698	2.500741	0.003157
ENSG0000(	LYL1	0.866743	4.344775	4.52E-05
ENSG0000(	EVA1B	0.866239	3.952759	0.000111
ENSG0000(	TBC1D2B	0.865795	5.186725	6.51E-06
ENSG0000(	TSPAN5	0.865664	2.956077	0.001106
ENSG0000(	PCDHB12	0.865652	1.429578	0.03719
ENSG0000(	SNX20	0.865364	2.938762	0.001151
ENSG0000(	HSD11B2	0.86534	1.846404	0.014243
ENSG0000(	TPK1	0.86513	3.957132	0.00011
ENSG0000(	COL16A1	0.865069	4.102445	7.90E-05
ENSG0000(	STAT2	0.864702	11.60904	2.46E-12
ENSG0000(	INSIG1	0.864391	4.842258	1.44E-05
ENSG0000(	LINC00294	0.864022	5.223834	5.97E-06
ENSG0000(	AC125257.	0.863371	5.276053	5.30E-06
ENSG0000(	MYH10	0.862984	2.841646	0.00144
ENSG0000(	ZNF286A	0.862759	7.104645	7.86E-08
ENSG0000(	AC068631.	0.862639	5.187092	6.50E-06
ENSG0000(	CEP295NL	0.862513	2.007366	0.009832
ENSG0000(	ENPP5	0.862006	2.568376	0.002702
ENSG0000(	LITAF	0.861982	4.262641	5.46E-05
ENSG0000(	ANGPTL2	0.861867	4.711276	1.94E-05
ENSG0000(	MEIS3P1	0.861628	1.883995	0.013062
ENSG0000(	ITM2C	0.861204	3.295415	0.000507
ENSG0000(	ARPC3	0.860987	5.054501	8.82E-06
ENSG0000(	AC006978.	0.860703	4.640305	2.29E-05
ENSG0000(	TBC1D10A	0.860429	4.759495	1.74E-05
ENSG0000(	C21orf91	0.860169	6.573342	2.67E-07
ENSG0000(	PTPRD	0.859961	2.173682	0.006704
ENSG0000(	KCNH3	0.859386	1.888401	0.01293
ENSG0000(	ATL3	0.859092	3.868637	0.000135
ENSG0000(	SWAP70	0.85897	4.963992	1.09E-05
ENSG0000(	PDCD4	0.858899	5.956651	1.10E-06
ENSG0000(	RIMKLB	0.858722	8.907358	1.24E-09
ENSG0000(	AC010619.	0.858543	6.049969	8.91E-07
ENSG0000(	MYO1D	0.858518	3.436437	0.000366
ENSG0000(	NINJ1	0.858516	4.820848	1.51E-05
ENSG0000(	PARVA	0.858473	3.723972	0.000189

ENSG0000(	NFE2L3	0.857763	3.702323	0.000198
ENSG0000(	SLC12A9	0.857556	7.700994	1.99E-08
ENSG0000(	ERFE	0.857542	1.457327	0.034888
ENSG0000(	C18orf54	0.857498	1.518185	0.030326
ENSG0000(	IFITM2	0.857015	4.295118	5.07E-05
ENSG0000(	AC008945.	0.856611	2.439793	0.003633
ENSG0000(	UBE2S	0.856053	4.286525	5.17E-05
ENSG0000(	AL163636.:	0.856016	1.573724	0.026686
ENSG0000(	NAV2	0.855455	6.667334	2.15E-07
ENSG0000(	AC092535.	0.855314	1.941024	0.011455
ENSG0000(	GSAP	0.855292	4.085878	8.21E-05
ENSG0000(	NBPF19	0.855066	5.204688	6.24E-06
ENSG0000(	MOSPD2	0.854577	4.401754	3.97E-05
ENSG0000(	SH3D21	0.854379	4.690442	2.04E-05
ENSG0000(	TCEAL9	0.854093	3.053893	0.000883
ENSG0000(	RAB4B	0.85371	7.234584	5.83E-08
ENSG0000(	CCND1	0.853602	4.170588	6.75E-05
ENSG0000(	BTBD19	0.853527	3.625905	0.000237
ENSG0000(	SERP2	0.853135	2.648056	0.002249
ENSG0000(	GBGT1	0.853048	3.724619	0.000189
ENSG0000(	AC006441.	0.852664	4.860774	1.38E-05
ENSG0000(	SUSD5	0.852508	2.50567	0.003121
ENSG0000(	DDX12P	0.852297	2.708499	0.001957
ENSG0000(	AL035071.:	0.852117	4.6619	2.18E-05
ENSG0000(	NQO1	0.852075	1.934864	0.011618
ENSG0000(	PDGFC	0.851836	5.456191	3.50E-06
ENSG0000(	EVC2	0.85182	2.135009	0.007328
ENSG0000(	AC068733.	0.851054	5.548593	2.83E-06
ENSG0000(	ARAP1-AS2	0.849888	5.514305	3.06E-06
ENSG0000(	SEPT4-AS1	0.849562	4.453692	3.52E-05
ENSG0000(	CMTM1	0.849462	1.446435	0.035774
ENSG0000(	EIF2AK3	0.849441	4.29992	5.01E-05
ENSG0000(	GAB2	0.848872	3.473289	0.000336
ENSG0000(	AC015922.	0.847813	2.356783	0.004398
ENSG0000(	CD200	0.847425	2.608681	0.002462
ENSG0000(	CHIC1	0.847311	3.370102	0.000426
ENSG0000(	SMCO4	0.847099	2.7032	0.001981
ENSG0000(	DHRS1	0.846996	5.284949	5.19E-06
ENSG0000(	ATP1A1	0.846956	6.608063	2.47E-07
ENSG0000(	GIMAP4	0.846662	3.621784	0.000239
ENSG0000(	PODN	0.846629	2.173099	0.006713
ENSG0000(	PASK	0.846549	4.113109	7.71E-05
ENSG0000(	ZNF559	0.846391	3.587509	0.000259
ENSG0000(	ITPRIPL1	0.846353	2.053684	0.008837
ENSG0000(	AC083949.	0.846192	2.39937	0.003987
ENSG0000(	ADH1B	0.846107	1.723614	0.018897
ENSG0000(	RELB	0.846097	3.837243	0.000145

ENSG0000(	AC022966.	0.845635	2.143732	0.007182
ENSG0000(	WEE1	0.845609	4.260842	5.48E-05
ENSG0000(	LOXL2	0.844911	2.955792	0.001107
ENSG0000(	IL15	0.843507	4.510279	3.09E-05
ENSG0000(	EMC3-AS1	0.843295	3.326859	0.000471
ENSG0000(	AC073130.	0.843028	3.323084	0.000475
ENSG0000(	RAB8A	0.842973	5.751019	1.77E-06
ENSG0000(	NBPF25P	0.84287	1.590034	0.025702
ENSG0000(	CARD6	0.842588	3.277089	0.000528
ENSG0000(	SPIN1	0.842405	5.471795	3.37E-06
ENSG0000(	NAP1L3	0.842344	2.01546	0.00965
ENSG0000(	SOX12	0.842341	4.023722	9.47E-05
ENSG0000(	EFS	0.841938	3.047724	0.000896
ENSG0000(	MBNL3	0.84173	2.112661	0.007715
ENSG0000(	RNF213	0.841646	8.152876	7.03E-09
ENSG0000(	MAPK10	0.841454	5.057503	8.76E-06
ENSG0000(	MIF	0.840627	5.672625	2.13E-06
ENSG0000(	LRRN4CL	0.840371	2.344207	0.004527
ENSG0000(	SLX4IP	0.840241	5.706491	1.97E-06
ENSG0000(	MGAT3	0.840127	3.231568	0.000587
ENSG0000(	ATP6AP2	0.839528	5.593758	2.55E-06
ENSG0000(	ARHGAP27	0.839029	4.870639	1.35E-05
ENSG0000(	SLC27A3	0.83891	4.52219	3.00E-05
ENSG0000(	CBLB	0.838755	6.048169	8.95E-07
ENSG0000(	GALNT12	0.838751	1.400298	0.039783
ENSG0000(	ELMO1	0.838504	3.294251	0.000508
ENSG0000(	IL10RB-DT	0.838219	5.861217	1.38E-06
ENSG0000(	AC011510.	0.838137	1.608504	0.024632
ENSG0000(	BMS1P3	0.837581	1.566219	0.027151
ENSG0000(	CAVIN3	0.837002	5.327485	4.70E-06
ENSG0000(	VSTM4	0.836707	4.498549	3.17E-05
ENSG0000(	ROR1	0.836649	3.889552	0.000129
ENSG0000(	SIGIRR	0.836181	4.738676	1.83E-05
ENSG0000(	ODF2	0.836049	8.422567	3.78E-09
ENSG0000(	BAX	0.8358	8.172252	6.73E-09
ENSG0000(	TNFSF12-TI	0.835423	8.407132	3.92E-09
ENSG0000(	AC002472.	0.835392	2.066452	0.008581
ENSG0000(	BTN2A1	0.835298	12.64581	2.26E-13
ENSG0000(	ZNF329	0.834422	3.857709	0.000139
ENSG0000(	MIF-AS1	0.833944	5.664296	2.17E-06
ENSG0000(	IMPDH1	0.833497	4.696108	2.01E-05
ENSG0000(	FRMD8	0.833482	7.677066	2.10E-08
ENSG0000(	AKR1A1	0.83337	6.263538	5.45E-07
ENSG0000(	SYNGAP1-/	0.833069	2.027177	0.009393
ENSG0000(	ECM1	0.832999	2.786145	0.001636
ENSG0000(	AC093726.	0.832434	1.530597	0.029472
ENSG0000(	IFT52	0.831997	2.702433	0.001984

ENSG0000(	AC116366.	0.831756	4.18169	6.58E-05
ENSG0000(	OCIAD2	0.831618	4.235681	5.81E-05
ENSG0000(	HFE	0.831139	8.651009	2.23E-09
ENSG0000(	SH2B3	0.830778	6.278526	5.27E-07
ENSG0000(	GRAMD2B	0.830246	3.31141	0.000488
ENSG0000(	PGBD5	0.830238	3.352754	0.000444
ENSG0000(	MIR4658	0.829628	1.519532	0.030232
ENSG0000(	MIA-RAB4E	0.828675	6.582254	2.62E-07
ENSG0000(	CDK15	0.828444	2.112588	0.007716
ENSG0000(	IDUA	0.828362	5.850028	1.41E-06
ENSG0000(	TMCO4	0.828144	5.345648	4.51E-06
ENSG0000(	BACE2	0.828119	3.95271	0.000112
ENSG0000(	ODF2L	0.827057	3.351701	0.000445
ENSG0000(	AC020913.	0.827033	2.825871	0.001493
ENSG0000(	ST6GALNA(	0.827029	6.386217	4.11E-07
ENSG0000(	AC011479.	0.826629	2.279518	0.005254
ENSG0000(	PRMT6	0.826602	5.10511	7.85E-06
ENSG0000(	NPHP4	0.826543	4.858322	1.39E-05
ENSG0000(	MST1R	0.826515	3.344526	0.000452
ENSG0000(	ZC3HAV1L	0.826355	1.448173	0.035631
ENSG0000(	ARSA	0.826233	5.704045	1.98E-06
ENSG0000(	HMGCLL1	0.825244	1.842011	0.014388
ENSG0000(	RTL5	0.825244	3.843933	0.000143
ENSG0000(	PRXL2B	0.824989	5.116976	7.64E-06
ENSG0000(	ATXN7L1	0.824503	6.049108	8.93E-07
ENSG0000(	UBA6-AS1	0.824331	4.32228	4.76E-05
ENSG0000(	TVP23C-CD	0.823918	7.705547	1.97E-08
ENSG0000(	SLC22A4	0.823774	2.967505	0.001078
ENSG0000(	MEX3D	0.823584	4.797482	1.59E-05
ENSG0000(	AL590560.:	0.823023	1.649927	0.022391
ENSG0000(	KDELR3	0.822973	2.571	0.002685
ENSG0000(	OSMR	0.822708	4.919233	1.20E-05
ENSG0000(	TAPBPL	0.822542	7.548123	2.83E-08
ENSG0000(	ZNF813	0.822276	3.400344	0.000398
ENSG0000(	AP006623.	0.822133	1.678281	0.020976
ENSG0000(	DNAJB11	0.822126	6.353838	4.43E-07
ENSG0000(	ZNF430	0.821733	4.085878	8.21E-05
ENSG0000(	CNPY4	0.819934	4.733588	1.85E-05
ENSG0000(	AC005324.	0.819709	4.149841	7.08E-05
ENSG0000(	MAPK8IP1	0.819256	4.852381	1.40E-05
ENSG0000(	TMCC1-AS:	0.818844	2.834933	0.001462
ENSG0000(	HYOU1	0.817743	10.3141	4.85E-11
ENSG0000(	PLA2R1	0.817711	2.449026	0.003556
ENSG0000(	STX2	0.817478	6.41341	3.86E-07
ENSG0000(	WWTR1-AS	0.816382	3.720966	0.00019
ENSG0000(	AC092279.	0.816184	2.194902	0.006384
ENSG0000(	RRN3P1	0.815941	5.906372	1.24E-06

ENSG0000(	KIAA0513	0.815809	6.502357	3.15E-07
ENSG0000(	MTMR11	0.815737	2.444119	0.003597
ENSG0000(	PROS1	0.815629	2.27649	0.005291
ENSG0000(	RASGRP4	0.81541	1.950722	0.011202
ENSG0000(	ADGRE5	0.815292	4.884748	1.30E-05
ENSG0000(	CD81	0.81426	6.183617	6.55E-07
ENSG0000(	PRICKLE2	0.814256	3.259266	0.00055
ENSG0000(	RPL3	0.814045	2.50732	0.003109
ENSG0000(	NAB2	0.813733	5.184992	6.53E-06
ENSG0000(	PTPRN2	0.813556	3.771688	0.000169
ENSG0000(	CYP11A1	0.813026	2.234951	0.005822
ENSG0000(	REEP4	0.812835	6.467672	3.41E-07
ENSG0000(	LINC01588	0.812522	1.385122	0.041198
ENSG0000(	RGS16	0.812469	2.056169	0.008787
ENSG0000(	EVL	0.812052	5.001206	9.97E-06
ENSG0000(	SPINT1	0.81194	1.926735	0.011838
ENSG0000(	AC092069.	0.811574	8.638408	2.30E-09
ENSG0000(	ACSL4	0.811413	9.403583	3.95E-10
ENSG0000(	KIF11	0.81118	2.300521	0.005006
ENSG0000(	CLDN15	0.81082	2.961075	0.001094
ENSG0000(	DTX4	0.810449	5.024267	9.46E-06
ENSG0000(	KIF13B	0.810442	8.704828	1.97E-09
ENSG0000(	DPYD	0.810306	3.59758	0.000253
ENSG0000(	ZNF700	0.81015	6.977199	1.05E-07
ENSG0000(	NSUN3	0.809356	5.661923	2.18E-06
ENSG0000(	CTTNBP2N	0.809154	5.661572	2.18E-06
ENSG0000(	NOTCH2NL	0.809069	2.592422	0.002556
ENSG0000(	MYOG	0.808477	3.636229	0.000231
ENSG0000(	DEGS2	0.807211	4.146259	7.14E-05
ENSG0000(	TNXB	0.806325	1.799195	0.015878
ENSG0000(	TRAM1	0.805982	4.156089	6.98E-05
ENSG0000(	TP73	0.805724	1.675206	0.021125
ENSG0000(	PI4K2B	0.805093	3.521951	0.000301
ENSG0000(	OGFR	0.804705	6.501051	3.15E-07
ENSG0000(	AC006027.	0.804601	7.040924	9.10E-08
ENSG0000(	FOXH1	0.804226	1.526707	0.029737
ENSG0000(	GRAMD1B	0.80396	3.515895	0.000305
ENSG0000(	AL390728.4	0.803755	3.741115	0.000182
ENSG0000(	AC019257.	0.802775	2.494708	0.003201
ENSG0000(	STX1A	0.802198	5.534513	2.92E-06
ENSG0000(	THSD7A	0.80219	2.631473	0.002336
ENSG0000(	KCNAB1	0.802155	1.457993	0.034834
ENSG0000(	PIWIL4	0.802117	3.96528	0.000108
ENSG0000(	PRKD2	0.801688	5.149845	7.08E-06
ENSG0000(	CYR61	0.801172	1.568532	0.027007
ENSG0000(	VDAC1P8	0.801076	3.930839	0.000117
ENSG0000(	TXN	0.800977	4.302388	4.98E-05

ENSG0000(	PLEKHA7	0.800962	6.132633	7.37E-07
ENSG0000(	COX6A1	0.800381	3.436906	0.000366
ENSG0000(	STON2	0.80021	5.838734	1.45E-06
ENSG0000(	MIR4435-2	0.8	6.227748	5.92E-07
ENSG0000(	SFMBT2	0.79984	5.804443	1.57E-06
ENSG0000(	AC008537.	0.799827	4.511291	3.08E-05
ENSG0000(	SLC5A10	0.799452	3.55012	0.000282
ENSG0000(	SPA17	0.799229	2.333395	0.004641
ENSG0000(	TGFB1	0.798884	6.279313	5.26E-07
ENSG0000(	TRPC1	0.798555	2.836815	0.001456
ENSG0000(	PDZD4	0.79842	2.42364	0.00377
ENSG0000(	CYFIP2	0.798179	4.128564	7.44E-05
ENSG0000(	AC090197.	0.798139	2.213585	0.006115
ENSG0000(	CFD	0.798043	1.513478	0.030656
ENSG0000(	ITGB3	0.797864	1.989044	0.010255
ENSG0000(	SNCG	0.797654	2.125533	0.00749
ENSG0000(	AC005261.	0.797047	1.672253	0.021269
ENSG0000(	TGFBR2	0.796619	3.512307	0.000307
ENSG0000(	TACC3	0.795995	3.7099	0.000195
ENSG0000(	NME1	0.795901	6.494042	3.21E-07
ENSG0000(	TTC21A	0.795738	1.926772	0.011837
ENSG0000(	TMEM198I	0.795562	5.025614	9.43E-06
ENSG0000(	CDK3	0.795482	4.72479	1.88E-05
ENSG0000(	TRAM2	0.795467	4.224117	5.97E-05
ENSG0000(	ANKRD65	0.795453	3.474276	0.000336
ENSG0000(	LASP1	0.794917	5.140135	7.24E-06
ENSG0000(	AC233723.	0.794718	3.028479	0.000937
ENSG0000(	HOXB6	0.794508	2.718543	0.001912
ENSG0000(	DYNLT3	0.794111	6.298083	5.03E-07
ENSG0000(	LMNB1	0.793539	3.784552	0.000164
ENSG0000(	NUPR1	0.793483	2.620256	0.002397
ENSG0000(	LINC01521	0.793287	1.643361	0.022732
ENSG0000(	PDGFD	0.793192	1.697142	0.020084
ENSG0000(	AP000646.	0.792823	2.836168	0.001458
ENSG0000(	TENT5A	0.79192	2.601717	0.002502
ENSG0000(	LRRC25	0.791392	2.037542	0.009172
ENSG0000(	TSGA10	0.791366	1.384286	0.041278
ENSG0000(	MYLK-AS1	0.791328	2.152479	0.007039
ENSG0000(	SMAD9	0.791179	2.788785	0.001626
ENSG0000(	NIPSNAP3/	0.790682	6.107111	7.81E-07
ENSG0000(	KPNA2	0.790678	6.846335	1.42E-07
ENSG0000(	SLC39A11	0.790199	5.145443	7.15E-06
ENSG0000(	ZNF738	0.789327	4.596011	2.54E-05
ENSG0000(	TMEM165	0.789233	8.224989	5.96E-09
ENSG0000(	LBH	0.789088	3.82297	0.00015
ENSG0000(	RF01872	0.78884	2.907399	0.001238
ENSG0000(	BCL10	0.78834	4.093351	8.07E-05

ENSG0000(	RGS11	0.788249	3.585094	0.00026
ENSG0000(	ARMCX2	0.788193	7.405726	3.93E-08
ENSG0000(	PABPC1	0.787811	4.761654	1.73E-05
ENSG0000(	CHPF	0.787184	5.983692	1.04E-06
ENSG0000(	ZNF43	0.786479	3.497008	0.000318
ENSG0000(	MSN	0.786134	5.891578	1.28E-06
ENSG0000(	USP30-AS1	0.786054	3.311248	0.000488
ENSG0000(	AC008770.	0.785585	3.958	0.00011
ENSG0000(	IDNK	0.785384	3.738209	0.000183
ENSG0000(	ESYT1	0.784991	6.657805	2.20E-07
ENSG0000(	NEK3	0.78391	5.823474	1.50E-06
ENSG0000(	BX255925.	0.783874	5.573804	2.67E-06
ENSG0000(	RNF212	0.783089	1.678237	0.020978
ENSG0000(	CASC15	0.782677	1.56557	0.027191
ENSG0000(	11-Sep	0.782638	7.670347	2.14E-08
ENSG0000(	TGIF1	0.781846	4.594319	2.54E-05
ENSG0000(	CRLF3	0.781087	6.353838	4.43E-07
ENSG0000(	PPIA	0.780379	4.895863	1.27E-05
ENSG0000(	ADCY3	0.779657	5.399748	3.98E-06
ENSG0000(	CHPF2	0.779623	6.249682	5.63E-07
ENSG0000(	SPON2	0.779387	4.276496	5.29E-05
ENSG0000(	TEP1	0.779256	7.145499	7.15E-08
ENSG0000(	MFSD13A	0.779247	2.836622	0.001457
ENSG0000(	DTNB	0.77888	2.81245	0.00154
ENSG0000(	ARMCX6	0.778876	6.670028	2.14E-07
ENSG0000(	CXCL12	0.778755	6.333935	4.64E-07
ENSG0000(	SHC3	0.778718	1.825044	0.014961
ENSG0000(	AXL	0.778694	2.283557	0.005205
ENSG0000(	ARHGDIG	0.778234	3.422234	0.000378
ENSG0000(	AL645465.:	0.777922	3.47291	0.000337
ENSG0000(	ZNF816	0.777527	1.907796	0.012365
ENSG0000(	DDHD1	0.777199	7.228222	5.91E-08
ENSG0000(	PGAM1	0.77653	7.02822	9.37E-08
ENSG0000(	KCNE3	0.775999	2.49355	0.00321
ENSG0000(	BNIP3L	0.775906	2.92198	0.001197
ENSG0000(	SOWAHC	0.77579	4.426895	3.74E-05
ENSG0000(	AP002761.	0.775453	3.170432	0.000675
ENSG0000(	STK32C	0.775174	8.480717	3.31E-09
ENSG0000(	OSBPL10	0.773988	3.089014	0.000815
ENSG0000(	DDB2	0.773943	7.618549	2.41E-08
ENSG0000(	TTLL1	0.773333	7.150443	7.07E-08
ENSG0000(	PRMT2	0.773186	9.22953	5.89E-10
ENSG0000(	LRP1	0.773093	1.980376	0.010462
ENSG0000(	AP000350.	0.773019	5.101147	7.92E-06
ENSG0000(	RCC2	0.772254	6.965071	1.08E-07
ENSG0000(	SLC45A3	0.772042	1.701117	0.019901
ENSG0000(	AC008966.	0.771927	1.643842	0.022707

ENSG0000(	MICA	0.771668	6.486506	3.26E-07
ENSG0000(	AC008906.	0.771196	3.969109	0.000107
ENSG0000(	ELOVL1	0.771182	6.970738	1.07E-07
ENSG0000(	CKAP2	0.77066	3.220456	0.000602
ENSG0000(	LDOC1	0.770658	5.072462	8.46E-06
ENSG0000(	BCYRN1	0.770372	1.915993	0.012134
ENSG0000(	KCTD12	0.769938	3.007352	0.000983
ENSG0000(	RAP1GDS1	0.769523	3.903397	0.000125
ENSG0000(	ROM1	0.769461	4.346513	4.50E-05
ENSG0000(	AC015712.	0.769432	3.218852	0.000604
ENSG0000(	ADGRL1	0.768939	3.207225	0.000621
ENSG0000(	AC025423.	0.76865	6.116598	7.65E-07
ENSG0000(	AP000593.	0.768526	1.38862	0.040868
ENSG0000(	KCNQ4	0.767951	2.297158	0.005045
ENSG0000(	CHIC2	0.767911	4.694227	2.02E-05
ENSG0000(	LRP5L	0.76676	2.587596	0.002585
ENSG0000(	MYO7A	0.766298	2.195687	0.006373
ENSG0000(	APEX2	0.766144	6.941415	1.14E-07
ENSG0000(	RGS6	0.765846	2.665198	0.002162
ENSG0000(	PLK2	0.765439	3.92319	0.000119
ENSG0000(	AC073111.	0.765377	5.329085	4.69E-06
ENSG0000(	TMOD3	0.765335	5.147177	7.13E-06
ENSG0000(	HABP4	0.764424	13.82112	1.51E-14
ENSG0000(	CYFIP1	0.763972	9.834464	1.46E-10
ENSG0000(	EHHADH	0.763704	2.675552	0.002111
ENSG0000(	AL591485.:	0.763692	1.987863	0.010283
ENSG0000(	GIMAP1-GI	0.763638	4.015173	9.66E-05
ENSG0000(	PGD	0.763623	8.151046	7.06E-09
ENSG0000(	SELENOM	0.763107	3.945502	0.000113
ENSG0000(	ARAP1	0.762577	7.63084	2.34E-08
ENSG0000(	FAM49A	0.762195	4.971628	1.07E-05
ENSG0000(	KIAA1522	0.762184	8.228507	5.91E-09
ENSG0000(	ERI1	0.762145	4.959855	1.10E-05
ENSG0000(	PLAGL1	0.761652	4.659303	2.19E-05
ENSG0000(	AC108134.	0.761286	2.708367	0.001957
ENSG0000(	GORAB	0.760374	3.95618	0.000111
ENSG0000(	ZNF562	0.760353	8.644047	2.27E-09
ENSG0000(	LINC01358	0.760155	1.764549	0.017197
ENSG0000(	ZC3H12A	0.760146	3.912488	0.000122
ENSG0000(	CCDC24	0.760095	2.782224	0.001651
ENSG0000(	SDCBP	0.75992	5.08346	8.25E-06
ENSG0000(	BOC	0.759315	3.651041	0.000223
ENSG0000(	AC079781.	0.75931	4.732891	1.85E-05
ENSG0000(	CKS2	0.759259	2.520194	0.003019
ENSG0000(	MKI67	0.758942	2.348763	0.00448
ENSG0000(	SERPINI1	0.758392	2.746399	0.001793
ENSG0000(	TRIM56	0.757472	5.751019	1.77E-06

ENSG0000(	CIT	0.756882	2.485582	0.003269
ENSG0000(	AP001372.	0.756832	1.422737	0.03778
ENSG0000(	NRM	0.756791	4.018667	9.58E-05
ENSG0000(	EEF1AKMT	0.756508	2.996585	0.001008
ENSG0000(	PRR11	0.756418	4.094291	8.05E-05
ENSG0000(	AL031320.:	0.756185	4.10886	7.78E-05
ENSG0000(	ACER3	0.756103	3.94111	0.000115
ENSG0000(	ACTR3	0.755917	9.068715	8.54E-10
ENSG0000(	CDH24	0.755825	1.609322	0.024585
ENSG0000(	AC006453.	0.755343	1.812267	0.015408
ENSG0000(	PHLDA2	0.754853	1.579554	0.02633
ENSG0000(	FBXL7	0.754796	2.48053	0.003307
ENSG0000(	RALGPS2	0.754518	1.768132	0.017056
ENSG0000(	PTGER4	0.754235	2.787241	0.001632
ENSG0000(	ZNF177	0.754139	3.958755	0.00011
ENSG0000(	SRGAP3	0.753699	4.454756	3.51E-05
ENSG0000(	RAB3IL1	0.753283	2.861906	0.001374
ENSG0000(	GIMAP7	0.752931	2.829512	0.001481
ENSG0000(	TMEM91	0.752834	4.107763	7.80E-05
ENSG0000(	HIST2H2BC	0.752831	1.85484	0.013969
ENSG0000(	AP001458.	0.752777	1.979157	0.010492
ENSG0000(	ASAP2	0.751867	2.485693	0.003268
ENSG0000(	MYEF2	0.751846	4.054734	8.82E-05
ENSG0000(	AC004656.	0.751176	2.525217	0.002984
ENSG0000(	APMAP	0.750973	4.859225	1.38E-05
ENSG0000(	STARD9	0.750894	4.542317	2.87E-05
ENSG0000(	SLC4A3	0.750347	2.173675	0.006704
ENSG0000(	RNF207	0.750241	2.787604	0.001631
ENSG0000(	CCDC102A	0.750229	4.642745	2.28E-05
ENSG0000(	PHLDB2	0.749717	1.472754	0.03367
ENSG0000(	ABCA7	0.748988	2.70537	0.001971
ENSG0000(	CCDC136	0.748958	2.170138	0.006759
ENSG0000(	SRPX2	0.748731	1.546333	0.028423
ENSG0000(	MFRP	0.748555	1.882152	0.013117
ENSG0000(	KIAA1551	0.747783	2.119089	0.007602
ENSG0000(	AC007319.	0.747554	2.141319	0.007222
ENSG0000(	CELF2	0.7469	4.505337	3.12E-05
ENSG0000(	DYRK3	0.746824	1.502788	0.03142
ENSG0000(	TMEM44-A	0.745838	3.11213	0.000772
ENSG0000(	ZNF512	0.745807	13.17566	6.67E-14
ENSG0000(	C1QTNF5	0.745617	1.856137	0.013927
ENSG0000(	NR1H3	0.745429	4.737202	1.83E-05
ENSG0000(	ADAMTSL3	0.745259	2.162842	0.006873
ENSG0000(	SNX33	0.745233	3.726634	0.000188
ENSG0000(	RMDN2	0.745168	3.014616	0.000967
ENSG0000(	ORAI3	0.745104	3.94231	0.000114
ENSG0000(	DDR2	0.745023	1.397717	0.040021

ENSG0000(	AC008569.	0.7447	3.288568	0.000515
ENSG0000(	IQGAP1	0.744624	3.418349	0.000382
ENSG0000(	EMILIN1	0.744192	3.621784	0.000239
ENSG0000(	AL591806.:	0.744164	7.217237	6.06E-08
ENSG0000(	ARHGEF19	0.743932	2.472793	0.003367
ENSG0000(	ABHD12	0.743901	6.340977	4.56E-07
ENSG0000(	PFKL	0.743704	4.84507	1.43E-05
ENSG0000(	GATA3	0.742883	1.829605	0.014805
ENSG0000(	MPO	0.74242	1.562023	0.027414
ENSG0000(	TPM4	0.742069	4.042836	9.06E-05
ENSG0000(	ADD3-AS1	0.741463	1.399635	0.039844
ENSG0000(	ALDH1A3	0.741389	2.999701	0.001001
ENSG0000(	AKR1C1	0.741107	2.034381	0.009239
ENSG0000(	AC091564.	0.741087	3.502185	0.000315
ENSG0000(	CNTRL	0.740508	2.337471	0.004598
ENSG0000(	AL355596.:	0.740199	1.512036	0.030758
ENSG0000(	SLC50A1	0.740096	5.760807	1.73E-06
ENSG0000(	FDXR	0.740066	2.494476	0.003203
ENSG0000(	GBA	0.739894	4.547645	2.83E-05
ENSG0000(	PRRT2	0.739304	2.967479	0.001078
ENSG0000(	IFFO1	0.738948	6.782863	1.65E-07
ENSG0000(	ARHGAP22	0.738896	3.075456	0.000841
ENSG0000(	P2RY6	0.738624	2.37673	0.0042
ENSG0000(	PML	0.738499	8.322441	4.76E-09
ENSG0000(	CKS1B	0.738266	3.95271	0.000112
ENSG0000(	FAM171A1	0.737696	5.275173	5.31E-06
ENSG0000(	IL4I1	0.737569	3.860356	0.000138
ENSG0000(	PTPN2	0.73713	4.020102	9.55E-05
ENSG0000(	PGM2	0.73695	3.952467	0.000112
ENSG0000(	MAP6	0.73649	1.930294	0.011741
ENSG0000(	OSTC	0.736072	3.460027	0.000347
ENSG0000(	PALM	0.73603	1.941613	0.011439
ENSG0000(	GLS	0.735861	8.349195	4.48E-09
ENSG0000(	B3GALT4	0.735315	2.829114	0.001482
ENSG0000(	CASP7	0.735002	8.228152	5.91E-09
ENSG0000(	AL512353.:	0.734976	4.62832	2.35E-05
ENSG0000(	B3GALNT1	0.734615	2.832668	0.00147
ENSG0000(	NHSL2	0.734545	4.101308	7.92E-05
ENSG0000(	DHFR2	0.734321	3.916962	0.000121
ENSG0000(	DZIP1L	0.734121	1.691875	0.020329
ENSG0000(	CATSPERG	0.733849	1.329115	0.046869
ENSG0000(	ITPA	0.733759	8.799672	1.59E-09
ENSG0000(	NOL4L	0.733168	5.765466	1.72E-06
ENSG0000(	DSC2	0.732865	1.747834	0.017872
ENSG0000(	SGPP1	0.732336	3.644987	0.000226
ENSG0000(	ADAR	0.732109	7.875861	1.33E-08
ENSG0000(	B9D2	0.731662	2.2006	0.006301

ENSG0000(	SYP	0.731315	2.465267	0.003426
ENSG0000(	PRICKLE2-A	0.730996	2.277649	0.005277
ENSG0000(	HPS1	0.730912	5.338176	4.59E-06
ENSG0000(	ACTN1	0.730567	4.349403	4.47E-05
ENSG0000(	FAM184A	0.730396	1.357205	0.043933
ENSG0000(	LRRC49	0.730141	2.571	0.002685
ENSG0000(	WIPF3	0.729941	2.513493	0.003066
ENSG0000(	PTPN13	0.729847	1.753854	0.017626
ENSG0000(	AC239799.	0.729765	2.107124	0.007814
ENSG0000(	CREBL2	0.729764	4.272493	5.34E-05
ENSG0000(	RERG	0.729508	1.645092	0.022642
ENSG0000(	CCDC120	0.729375	3.804775	0.000157
ENSG0000(	AMIGO2	0.729122	2.996434	0.001008
ENSG0000(	FAM122C	0.728829	8.09127	8.10E-09
ENSG0000(	KANK2	0.728806	4.942379	1.14E-05
ENSG0000(	OXCT1-AS1	0.728774	1.478227	0.033249
ENSG0000(	AC020916.	0.728684	3.868521	0.000135
ENSG0000(	RCN3	0.728681	4.764602	1.72E-05
ENSG0000(	AC112220.	0.728608	3.071248	0.000849
ENSG0000(	ZNRD1ASP	0.728511	1.5268	0.02973
ENSG0000(	VASP	0.728439	3.843933	0.000143
ENSG0000(	RASSF4	0.728186	3.434149	0.000368
ENSG0000(	FAM216A	0.727903	3.408005	0.000391
ENSG0000(	CA5B	0.727311	3.466066	0.000342
ENSG0000(	BX664615.	0.727309	1.527025	0.029715
ENSG0000(	MEGF10	0.727204	1.766878	0.017105
ENSG0000(	CBX4	0.726834	3.713095	0.000194
ENSG0000(	SH3BP5-AS	0.726216	5.772859	1.69E-06
ENSG0000(	SEC61B	0.726012	2.702411	0.001984
ENSG0000(	PGRMC1	0.725674	2.976292	0.001056
ENSG0000(	TCEAL8	0.725329	3.311248	0.000488
ENSG0000(	AKAP11	0.725169	4.184431	6.54E-05
ENSG0000(	AC099518.	0.724562	4.509326	3.10E-05
ENSG0000(	DPY19L1	0.724404	5.186539	6.51E-06
ENSG0000(	FAP	0.724336	1.464241	0.034337
ENSG0000(	GLT8D2	0.724026	1.663625	0.021696
ENSG0000(	SLC2A6	0.723537	2.502914	0.003141
ENSG0000(	BEX3	0.723177	3.206528	0.000622
ENSG0000(	RAB43	0.723114	4.047395	8.97E-05
ENSG0000(	PRXL2C	0.722768	5.00289	9.93E-06
ENSG0000(	AC024267.	0.72234	1.686941	0.020562
ENSG0000(	KCTD7	0.722313	7.766776	1.71E-08
ENSG0000(	CD58	0.722241	4.012525	9.72E-05
ENSG0000(	PGLS	0.720569	5.726733	1.88E-06
ENSG0000(	SLC30A7	0.720504	3.309876	0.00049
ENSG0000(	NAGK	0.720486	7.325127	4.73E-08
ENSG0000(	C5orf51	0.720104	4.626085	2.37E-05

ENSG0000(	GINS4	0.719891	1.440476	0.036268
ENSG0000(	AC124319.	0.719859	1.474077	0.033568
ENSG0000(	TRIM11	0.719745	5.657071	2.20E-06
ENSG0000(	PPP1R9B	0.719571	7.043897	9.04E-08
ENSG0000(	BAZ1A	0.719035	5.058953	8.73E-06
ENSG0000(	CYP1B1	0.718423	1.813014	0.015381
ENSG0000(	ORMDL2	0.718053	5.713632	1.93E-06
ENSG0000(	ARHGEF3	0.717685	4.984983	1.04E-05
ENSG0000(	FBXO33	0.717166	4.62207	2.39E-05
ENSG0000(	ZFP36L1	0.715979	4.254604	5.56E-05
ENSG0000(	RCN1	0.715942	8.527853	2.97E-09
ENSG0000(	POGLUT1	0.715717	4.834098	1.47E-05
ENSG0000(	ARHGEF17	0.7156	6.277837	5.27E-07
ENSG0000(	NLRP1	0.71551	3.863812	0.000137
ENSG0000(	AC127502.	0.715482	1.831617	0.014736
ENSG0000(	IFIT1	0.715374	4.773962	1.68E-05
ENSG0000(	PDE8B	0.71523	2.082763	0.008265
ENSG0000(	CYB5A	0.715166	3.098444	0.000797
ENSG0000(	SMIM14	0.714707	3.153985	0.000701
ENSG0000(	PIGM	0.714323	6.461102	3.46E-07
ENSG0000(	C20orf96	0.714116	2.877602	0.001326
ENSG0000(	SLC17A5	0.713414	3.900227	0.000126
ENSG0000(	RAP1B	0.713326	4.77652	1.67E-05
ENSG0000(	EXOSC7	0.713119	2.568849	0.002699
ENSG0000(	CLDND2	0.712801	1.335115	0.046226
ENSG0000(	THPO	0.71207	3.739525	0.000182
ENSG0000(	SLC38A10	0.712069	6.821506	1.51E-07
ENSG0000(	TUBB6	0.711953	8.657991	2.20E-09
ENSG0000(	SPARC	0.711522	1.824682	0.014973
ENSG0000(	ACKR4	0.71152	2.289943	0.005129
ENSG0000(	PIF1	0.711097	2.063248	0.008645
ENSG0000(	PRICKLE1	0.710792	3.207036	0.000621
ENSG0000(	TP53I11	0.710465	3.688493	0.000205
ENSG0000(	RHNO1	0.709918	4.833979	1.47E-05
ENSG0000(	AC108734.	0.709844	1.817648	0.015218
ENSG0000(	SPRED1	0.709763	3.18285	0.000656
ENSG0000(	ZNF625-ZN	0.709579	3.534506	0.000292
ENSG0000(	ARHGEF2	0.709332	6.951412	1.12E-07
ENSG0000(	CCDC39	0.70924	1.976513	0.010556
ENSG0000(	IGSF11	0.708858	1.927518	0.011816
ENSG0000(	ZNF83	0.708552	2.589254	0.002575
ENSG0000(	ADM	0.708213	1.988908	0.010259
ENSG0000(	ZDHHC13	0.708148	3.035641	0.000921
ENSG0000(	GCA	0.708127	1.68583	0.020614
ENSG0000(	AL391069.:	0.707059	2.874857	0.001334
ENSG0000(	CDC42EP2	0.707002	3.712499	0.000194
ENSG0000(	WDR31	0.706647	1.759306	0.017406

ENSG0000(	LHFPL6	0.706448	2.123588	0.007523
ENSG0000(	CLUHP3	0.70615	3.362043	0.000434
ENSG0000(	FLNA	0.705992	4.018852	9.58E-05
ENSG0000(	RFWD3	0.705909	7.053955	8.83E-08
ENSG0000(	C1QTNF1	0.705842	1.812267	0.015408
ENSG0000(	PLAT	0.705805	2.347997	0.004487
ENSG0000(	NAP1L2	0.705424	3.141396	0.000722
ENSG0000(	MAP1B	0.705305	1.712977	0.019365
ENSG0000(	OLFML3	0.705027	1.655657	0.022097
ENSG0000(	SPOCK2	0.704915	2.312245	0.004873
ENSG0000(	TSPO	0.704802	6.544866	2.85E-07
ENSG0000(	VMP1	0.704659	5.745927	1.80E-06
ENSG0000(	CASP10	0.704612	4.225275	5.95E-05
ENSG0000(	FAM98C	0.704567	5.877042	1.33E-06
ENSG0000(	ESYT3	0.70444	1.313036	0.048637
ENSG0000(	CHRD	0.70416	3.568412	0.00027
ENSG0000(	Z97989.1	0.704052	2.132528	0.00737
ENSG0000(	PRKCZ	0.703491	5.084427	8.23E-06
ENSG0000(	ZNF568	0.703423	2.484152	0.00328
ENSG0000(	FAM172A	0.703319	4.080609	8.31E-05
ENSG0000(	STK39	0.703254	2.730306	0.001861
ENSG0000(	GRHL1	0.702875	1.450343	0.035453
ENSG0000(	ZNF675	0.70262	2.71959	0.001907
ENSG0000(	ZNF385B	0.702319	1.53665	0.029064
ENSG0000(	ETV6	0.702151	3.85819	0.000139
ENSG0000(	ZNF124	0.702102	2.734232	0.001844
ENSG0000(	PPIAP22	0.701822	4.003416	9.92E-05
ENSG0000(	FGFR1	0.701719	4.030414	9.32E-05
ENSG0000(	INPP4B	0.700426	2.666202	0.002157
ENSG0000(	FLRT2	0.699836	2.985948	0.001033
ENSG0000(	KNTC1	0.699833	2.547545	0.002834
ENSG0000(	IL1R1	0.699559	4.136661	7.30E-05
ENSG0000(	PRKAG2	0.699541	2.72592	0.00188
ENSG0000(	DYNLL1	0.699156	4.280581	5.24E-05
ENSG0000(	AVPI1	0.699155	2.392541	0.00405
ENSG0000(	ADAMTS12	0.699007	1.752784	0.017669
ENSG0000(	DNASE2	0.69894	2.601576	0.002503
ENSG0000(	RBPJ	0.69887	3.573062	0.000267
ENSG0000(	TP53INP1	0.69872	2.321951	0.004765
ENSG0000(	HIF1A	0.698199	4.937743	1.15E-05
ENSG0000(	PRRT3	0.697437	3.484648	0.000328
ENSG0000(	CFI	0.697129	3.116974	0.000764
ENSG0000(	GJA1	0.697011	1.777641	0.016686
ENSG0000(	AL035078.4	0.696752	8.350044	4.47E-09
ENSG0000(	RHCE	0.696546	3.222259	0.000599
ENSG0000(	TRAF2	0.696523	5.587747	2.58E-06
ENSG0000(	CD81-AS1	0.696373	3.252461	0.000559

ENSG0000(	GGT5	0.695986	2.222065	0.005997
ENSG0000(	DUBR	0.695502	2.718427	0.001912
ENSG0000(	TGFBR3	0.695166	1.351467	0.044518
ENSG0000(	FLI1	0.695024	2.96955	0.001073
ENSG0000(	MRC2	0.694854	2.273236	0.00533
ENSG0000(	TSPAN33	0.694854	4.70118	1.99E-05
ENSG0000(	P2RX5	0.694777	2.925346	0.001188
ENSG0000(	AC027601.	0.694762	4.519132	3.03E-05
ENSG0000(	AL121845.:	0.694486	2.33593	0.004614
ENSG0000(	AC068234.	0.694476	1.574749	0.026623
ENSG0000(	SH3KBP1	0.693496	3.870208	0.000135
ENSG0000(	GNG7	0.692966	2.521753	0.003008
ENSG0000(	SOGA1	0.692171	5.45682	3.49E-06
ENSG0000(	PXYLP1	0.691752	3.548922	0.000283
ENSG0000(	SUMF1	0.69173	3.17142	0.000674
ENSG0000(	EIF5A2	0.691464	2.200696	0.006299
ENSG0000(	MGAT5	0.691448	3.220456	0.000602
ENSG0000(	AC090971.	0.691038	2.357817	0.004387
ENSG0000(	LRCH1	0.690849	3.271871	0.000535
ENSG0000(	ZNF521	0.690663	1.719166	0.019091
ENSG0000(	PELI1	0.690247	6.054648	8.82E-07
ENSG0000(	AGAP11	0.689643	1.821867	0.015071
ENSG0000(	KIRREL1	0.689622	2.385803	0.004113
ENSG0000(	AC009690.	0.688995	5.832124	1.47E-06
ENSG0000(	MIR675	0.688277	1.694428	0.02021
ENSG0000(	TSPAN2	0.688272	2.300681	0.005004
ENSG0000(	RP2	0.688269	3.872306	0.000134
ENSG0000(	PDZD7	0.687909	2.915125	0.001216
ENSG0000(	ZDHHC23	0.687876	1.540884	0.028782
ENSG0000(	LPP-AS2	0.687361	2.617301	0.002414
ENSG0000(	GLYCTK	0.687005	3.59133	0.000256
ENSG0000(	HEPH	0.686704	2.063419	0.008641
ENSG0000(	BMP2K	0.686098	3.569584	0.000269
ENSG0000(	CHMP4A	0.686015	6.329846	4.68E-07
ENSG0000(	AC011365.	0.685348	1.439971	0.03631
ENSG0000(	LGALS8	0.685071	5.638956	2.30E-06
ENSG0000(	ACTB	0.685	4.13627	7.31E-05
ENSG0000(	COL5A1	0.684835	1.573512	0.026699
ENSG0000(	AL136982.:	0.684216	1.516928	0.030414
ENSG0000(	ADPGK	0.683782	4.37633	4.20E-05
ENSG0000(	GNAO1	0.683605	2.843657	0.001433
ENSG0000(	RILPL2	0.6832	3.883796	0.000131
ENSG0000(	PLEKHH1	0.682955	4.611409	2.45E-05
ENSG0000(	CLMAT3	0.682932	1.387346	0.040988
ENSG0000(	PLPP5	0.682801	3.711865	0.000194
ENSG0000(	FADS1	0.68241	2.409137	0.003898
ENSG0000(	CCND3	0.682159	3.747443	0.000179

ENSG0000(	CLBA1	0.682017	3.736743	0.000183
ENSG0000(	AC009690.	0.681715	4.883911	1.31E-05
ENSG0000(	E2F8	0.681637	2.198804	0.006327
ENSG0000(	OSBPL8	0.681588	2.816478	0.001526
ENSG0000(	RTKN	0.681105	7.280821	5.24E-08
ENSG0000(	PTGR1	0.680886	2.802679	0.001575
ENSG0000(	COLGALT1	0.680576	5.862696	1.37E-06
ENSG0000(	CALR	0.680466	8.918798	1.21E-09
ENSG0000(	IER3	0.680349	2.255776	0.005549
ENSG0000(	NOD1	0.679892	8.155504	6.99E-09
ENSG0000(	TTLL3	0.67909	6.637423	2.30E-07
ENSG0000(	GALNT10	0.678804	3.125576	0.000749
ENSG0000(	EBF2	0.678748	2.17116	0.006743
ENSG0000(	NIPAL2	0.678705	2.344198	0.004527
ENSG0000(	TAF1B	0.678434	2.316307	0.004827
ENSG0000(	DISC1	0.67838	3.549044	0.000282
ENSG0000(	MFNG	0.678279	2.917878	0.001208
ENSG0000(	INF2	0.678227	5.601559	2.50E-06
ENSG0000(	AC009113.	0.677924	3.407031	0.000392
ENSG0000(	MCAM	0.677924	2.023861	0.009465
ENSG0000(	RFX5	0.677924	4.142379	7.20E-05
ENSG0000(	RGS14	0.677458	3.173616	0.00067
ENSG0000(	RAB23	0.677284	3.035217	0.000922
ENSG0000(	4-Sep	0.677142	4.062054	8.67E-05
ENSG0000(	PTPN9	0.676821	3.8479	0.000142
ENSG0000(	AL136982.(	0.676768	1.329105	0.04687
ENSG0000(	CCDC183-A	0.676674	2.05494	0.008812
ENSG0000(	CDC20	0.676604	1.550077	0.028179
ENSG0000(	NBPF20	0.676194	3.326401	0.000472
ENSG0000(	EML3	0.675766	3.549044	0.000282
ENSG0000(	CHSY1	0.675684	4.619848	2.40E-05
ENSG0000(	ARRB1	0.675429	3.658053	0.00022
ENSG0000(	AC008894.	0.675382	2.867651	0.001356
ENSG0000(	SYNGAP1	0.675312	8.552068	2.80E-09
ENSG0000(	TSPAN13	0.675223	4.25967	5.50E-05
ENSG0000(	ZNF837	0.675005	3.036707	0.000919
ENSG0000(	BLOC1S3	0.67461	4.930416	1.17E-05
ENSG0000(	CAMKK2	0.674482	7.710685	1.95E-08
ENSG0000(	AC087741.	0.674298	2.773685	0.001684
ENSG0000(	CCNG2	0.674004	2.731403	0.001856
ENSG0000(	CDH11	0.67397	2.08892	0.008149
ENSG0000(	ZNF627	0.673688	4.135993	7.31E-05
ENSG0000(	SLC38A6	0.673546	4.332864	4.65E-05
ENSG0000(	PLOD2	0.673005	1.881838	0.013127
ENSG0000(	CBL	0.67278	5.561324	2.75E-06
ENSG0000(	ERV3-1	0.672615	1.525999	0.029785
ENSG0000(	AC009163.	0.672564	1.365217	0.04313

ENSG0000(	NAT14	0.672427	4.132701	7.37E-05
ENSG0000(	RAD51D	0.672416	5.591116	2.56E-06
ENSG0000(	AC104532.	0.672351	4.113928	7.69E-05
ENSG0000(	TEDC1	0.671841	3.022545	0.000949
ENSG0000(	MAST3	0.671815	2.144972	0.007162
ENSG0000(	SLC20A1	0.671314	5.709797	1.95E-06
ENSG0000(	TMEM67	0.671215	1.479546	0.033148
ENSG0000(	PDIA5	0.670997	3.392733	0.000405
ENSG0000(	NBPF10	0.670822	2.700124	0.001995
ENSG0000(	KLHL3	0.670208	3.872672	0.000134
ENSG0000(	CALD1	0.669723	2.78368	0.001646
ENSG0000(	AL356481.:	0.669624	3.882628	0.000131
ENSG0000(	CHD3	0.669611	3.469653	0.000339
ENSG0000(	KPNA2P3	0.669367	4.69709	2.01E-05
ENSG0000(	LINC00654	0.669255	1.819512	0.015153
ENSG0000(	UGGT2	0.669076	1.707987	0.019589
ENSG0000(	PLEKHG2	0.668962	3.594928	0.000254
ENSG0000(	FCHSD2	0.668875	8.435893	3.67E-09
ENSG0000(	ASAP1	0.668514	5.656777	2.20E-06
ENSG0000(	ZNF836	0.668396	3.458974	0.000348
ENSG0000(	SULT1A1	0.668356	4.289023	5.14E-05
ENSG0000(	ENTPD1	0.668326	4.055932	8.79E-05
ENSG0000(	FES	0.668274	4.071986	8.47E-05
ENSG0000(	RND3	0.668161	2.362182	0.004343
ENSG0000(	SGTB	0.668052	3.92319	0.000119
ENSG0000(	AL035530.:	0.667756	1.624058	0.023765
ENSG0000(	RF02217	0.667713	1.755019	0.017578
ENSG0000(	A2MP1	0.667459	1.978607	0.010505
ENSG0000(	AL928654.:	0.667318	1.948627	0.011256
ENSG0000(	HMGN1	0.667178	4.72479	1.88E-05
ENSG0000(	ZNF544	0.666468	5.967185	1.08E-06
ENSG0000(	IL12RB2	0.666097	1.837006	0.014554
ENSG0000(	ARHGAP42	0.666017	2.000442	0.00999
ENSG0000(	ACAP1	0.665854	2.132923	0.007363
ENSG0000(	DPYSL2	0.665776	3.031497	0.00093
ENSG0000(	YWHAZ	0.665756	6.464103	3.43E-07
ENSG0000(	CATSPER2F	0.66559	6.374679	4.22E-07
ENSG0000(	FAM122B	0.665225	5.516266	3.05E-06
ENSG0000(	AL136982.4	0.664651	1.351891	0.044474
ENSG0000(	AL021546.:	0.66459	3.272237	0.000534
ENSG0000(	CCDC191	0.664508	2.132923	0.007363
ENSG0000(	GLB1	0.664333	4.671358	2.13E-05
ENSG0000(	XXYLT1	0.664182	3.447971	0.000356
ENSG0000(	PIK3IP1	0.664049	2.500782	0.003157
ENSG0000(	RRBP1	0.664035	3.434717	0.000368
ENSG0000(	PPP1R26	0.66382	3.327887	0.00047
ENSG0000(	CDK6	0.663724	1.958395	0.011005

ENSG0000(	TMEM273	0.663605	3.030248	0.000933
ENSG0000(	IRAK1	0.663536	8.764464	1.72E-09
ENSG0000(	SNX30	0.66317	3.936541	0.000116
ENSG0000(	CBX6	0.663032	6.268474	5.39E-07
ENSG0000(	ZNF624	0.662941	1.411745	0.038749
ENSG0000(	TRIM62	0.662373	3.133463	0.000735
ENSG0000(	MYDGF	0.661986	4.702081	1.99E-05
ENSG0000(	CEP164	0.661865	6.78623	1.64E-07
ENSG0000(	LDLRAD3	0.661859	2.316277	0.004828
ENSG0000(	LPCAT1	0.661732	5.29475	5.07E-06
ENSG0000(	CAPZA1	0.661077	4.336823	4.60E-05
ENSG0000(	AC079601.	0.660647	2.891817	0.001283
ENSG0000(	ALDH1B1	0.660544	1.849712	0.014135
ENSG0000(	PRKD1	0.660345	1.52414	0.029913
ENSG0000(	MELTF	0.659971	2.069781	0.008516
ENSG0000(	PLEKHG5	0.659853	3.206781	0.000621
ENSG0000(	AL078612.:	0.659727	6.782078	1.65E-07
ENSG0000(	HSPA5	0.659494	7.896946	1.27E-08
ENSG0000(	RBM43	0.658961	4.65018	2.24E-05
ENSG0000(	HHEX	0.658726	1.983242	0.010393
ENSG0000(	PRSS23	0.658697	5.158979	6.93E-06
ENSG0000(	TIMELESS	0.658561	5.611617	2.45E-06
ENSG0000(	PLD2	0.658497	4.294018	5.08E-05
ENSG0000(	AC022098.	0.657837	2.470665	0.003383
ENSG0000(	PEX11G	0.657601	2.332597	0.004649
ENSG0000(	RBMS1	0.65757	4.768234	1.71E-05
ENSG0000(	TTPAL	0.657405	5.511511	3.08E-06
ENSG0000(	AC002401.	0.656905	4.799367	1.59E-05
ENSG0000(	CHST7	0.656761	2.144869	0.007164
ENSG0000(	CCDC115	0.656727	4.984631	1.04E-05
ENSG0000(	TANC2	0.656727	4.251801	5.60E-05
ENSG0000(	ATOX1	0.656071	3.010154	0.000977
ENSG0000(	RNU4-5P	0.656031	5.35639	4.40E-06
ENSG0000(	COL6A2	0.655696	2.390015	0.004074
ENSG0000(	TMEM216	0.655643	2.785559	0.001638
ENSG0000(	SS18	0.65557	6.123423	7.53E-07
ENSG0000(	AC025164.	0.655382	1.711538	0.01943
ENSG0000(	SLC36A4	0.655298	2.061452	0.008681
ENSG0000(	LINC01563	0.653362	2.089057	0.008146
ENSG0000(	KIAA0556	0.653289	5.016264	9.63E-06
ENSG0000(	ANKRD29	0.65263	2.003869	0.009911
ENSG0000(	C1orf162	0.652596	2.30837	0.004916
ENSG0000(	MRGPRF	0.652472	1.646781	0.022554
ENSG0000(	MIR4751	0.652424	2.379157	0.004177
ENSG0000(	SYTL2	0.652391	2.268326	0.005391
ENSG0000(	YEATS2-AS	0.651469	4.647686	2.25E-05
ENSG0000(	PLXNA3	0.650958	4.857571	1.39E-05

ENSG0000(	SIPA1L1	0.650859	4.32229	4.76E-05
ENSG0000(	CNRIP1	0.650826	1.879631	0.013194
ENSG0000(	ZNF823	0.650687	3.536288	0.000291
ENSG0000(	FANCF	0.6506	3.266864	0.000541
ENSG0000(	PFN1	0.649878	5.666323	2.16E-06
ENSG0000(	TAX1BP3	0.649671	3.400512	0.000398
ENSG0000(	WDR34	0.649391	3.810867	0.000155
ENSG0000(	FAM129B	0.649262	6.767131	1.71E-07
ENSG0000(	TSHZ3	0.649121	3.006112	0.000986
ENSG0000(	AC011477.	0.649011	2.044189	0.009033
ENSG0000(	TCTA	0.64889	4.966183	1.08E-05
ENSG0000(	CSTB	0.648672	6.575332	2.66E-07
ENSG0000(	GDPD5	0.648641	4.335754	4.62E-05
ENSG0000(	PTCH2	0.647459	1.94105	0.011454
ENSG0000(	SLC23A2	0.647244	5.173362	6.71E-06
ENSG0000(	GLIS2	0.647148	3.064785	0.000861
ENSG0000(	TUFT1	0.646896	3.376622	0.00042
ENSG0000(	SPAG5	0.646493	2.699023	0.002
ENSG0000(	AC114490.	0.646286	4.002532	9.94E-05
ENSG0000(	LINC01569	0.646222	1.347471	0.044929
ENSG0000(	CCDC102B	0.645973	3.595134	0.000254
ENSG0000(	GALNT4	0.645811	2.337793	0.004594
ENSG0000(	OLMALINC	0.645765	2.237638	0.005786
ENSG0000(	DDX39A	0.645763	6.144645	7.17E-07
ENSG0000(	LEPR	0.645585	1.322041	0.047639
ENSG0000(	ITGAV	0.645477	3.611657	0.000245
ENSG0000(	MEX3B	0.645146	1.89059	0.012865
ENSG0000(	PIP5K1B	0.645125	1.637798	0.023025
ENSG0000(	CANX	0.645052	6.595844	2.54E-07
ENSG0000(	MYL9	0.644563	2.319888	0.004788
ENSG0000(	SBNO2	0.644289	5.300746	5.00E-06
ENSG0000(	TMEM43	0.644261	7.622885	2.38E-08
ENSG0000(	AC099343.	0.644111	4.746798	1.79E-05
ENSG0000(	PTMA	0.642898	4.661063	2.18E-05
ENSG0000(	DMPK	0.642597	2.322812	0.004755
ENSG0000(	PEA15	0.642318	5.053622	8.84E-06
ENSG0000(	CLIP2	0.642281	3.750397	0.000178
ENSG0000(	UBASH3B	0.642189	1.642433	0.022781
ENSG0000(	ZNF367	0.64217	2.972631	0.001065
ENSG0000(	CACNB3	0.641922	2.547137	0.002837
ENSG0000(	FRRS1	0.641661	2.391483	0.00406
ENSG0000(	SLC17A7	0.64069	1.882924	0.013094
ENSG0000(	FAM3C	0.640658	3.389827	0.000408
ENSG0000(	CD274	0.640561	1.626277	0.023644
ENSG0000(	FRMD6	0.640302	1.778461	0.016655
ENSG0000(	DACT1	0.640269	1.373361	0.042329
ENSG0000(	RECK	0.640173	1.516589	0.030438

ENSG0000(	SNX8	0.64011	6.854335	1.40E-07
ENSG0000(	CEP41	0.64006	3.473289	0.000336
ENSG0000(	AL162417.:	0.640004	7.974834	1.06E-08
ENSG0000(	TTC9	0.640003	1.882918	0.013094
ENSG0000(	RFTN2	0.639864	1.998261	0.01004
ENSG0000(	USP51	0.63962	1.40374	0.039469
ENSG0000(	GSDMB	0.63958	2.100791	0.007929
ENSG0000(	TMEM50A	0.639513	7.004511	9.90E-08
ENSG0000(	RALGDS	0.639338	7.139695	7.25E-08
ENSG0000(	MIR3652	0.639316	3.004928	0.000989
ENSG0000(	MAGED2	0.638914	4.15262	7.04E-05
ENSG0000(	AC006001.	0.638668	2.234805	0.005824
ENSG0000(	CREB3L2	0.638284	4.107763	7.80E-05
ENSG0000(	LRRC27	0.63805	4.685694	2.06E-05
ENSG0000(	CLCN5	0.637641	2.945006	0.001135
ENSG0000(	CPT1C	0.637297	1.432039	0.03698
ENSG0000(	HAAO	0.637281	2.234406	0.005829
ENSG0000(	AL133355.:	0.636259	2.228005	0.005916
ENSG0000(	ZNRF2	0.635692	3.125135	0.00075
ENSG0000(	COMMD8	0.635582	3.231728	0.000587
ENSG0000(	NAGA	0.635459	4.81447	1.53E-05
ENSG0000(	NDOR1	0.635077	5.769356	1.70E-06
ENSG0000(	SOD2-OT1	0.63504	1.475519	0.033457
ENSG0000(	LTBP1	0.634615	2.644002	0.00227
ENSG0000(	NT5DC3	0.634611	2.505322	0.003124
ENSG0000(	MOK	0.634247	2.641687	0.002282
ENSG0000(	ARHGAP39	0.633806	2.44909	0.003556
ENSG0000(	TWSG1	0.633562	2.463903	0.003436
ENSG0000(	AC007098.	0.633371	2.701485	0.001988
ENSG0000(	GK	0.633357	2.788958	0.001626
ENSG0000(	MDM2	0.633078	4.787137	1.63E-05
ENSG0000(	GXYLT2	0.63246	2.601848	0.002501
ENSG0000(	SPX	0.632383	1.589682	0.025723
ENSG0000(	CIITA	0.631827	2.61151	0.002446
ENSG0000(	TRIP6	0.631756	4.202088	6.28E-05
ENSG0000(	MOB1A	0.631721	5.226637	5.93E-06
ENSG0000(	DLL1	0.631511	3.496202	0.000319
ENSG0000(	ARHGAP1	0.631437	5.677876	2.10E-06
ENSG0000(	TASP1	0.63104	3.152277	0.000704
ENSG0000(	AC006460.	0.630647	2.403326	0.003951
ENSG0000(	AC116366.	0.630606	4.511291	3.08E-05
ENSG0000(	Z83844.3	0.630586	3.725998	0.000188
ENSG0000(	DCLRE1C	0.630482	2.942066	0.001143
ENSG0000(	CERS5	0.63044	4.649305	2.24E-05
ENSG0000(	SUCO	0.630186	3.198964	0.000632
ENSG0000(	C19orf71	0.630164	2.448533	0.00356
ENSG0000(	AC010883.	0.63016	1.4024	0.039591

ENSG0000(	MAFB	0.629859	2.829694	0.00148
ENSG0000(	UCP2	0.629752	3.254051	0.000557
ENSG0000(	GSR	0.629736	4.690493	2.04E-05
ENSG0000(	RAB6B	0.629588	2.466986	0.003412
ENSG0000(	AL096870.:	0.629053	4.855697	1.39E-05
ENSG0000(	TAGLN	0.628278	1.488003	0.032508
ENSG0000(	RUBCN	0.628088	8.327522	4.70E-09
ENSG0000(	ATF7IP2	0.628016	2.177942	0.006638
ENSG0000(	ZNF254	0.627972	2.806617	0.001561
ENSG0000(	AC025423.	0.627861	3.01086	0.000975
ENSG0000(	RAP1GAP2	0.627429	2.021017	0.009528
ENSG0000(	BBC3	0.627018	1.867319	0.013573
ENSG0000(	RAB9B	0.626073	2.898942	0.001262
ENSG0000(	AC005261.	0.625695	3.464954	0.000343
ENSG0000(	MEOX2	0.625568	1.683774	0.020712
ENSG0000(	FHDC1	0.625379	2.095701	0.008022
ENSG0000(	DOK4	0.625342	3.422706	0.000378
ENSG0000(	ARID5A	0.62507	2.669466	0.002141
ENSG0000(	AZIN1-AS1	0.624975	5.316255	4.83E-06
ENSG0000(	NAGLU	0.624972	3.665098	0.000216
ENSG0000(	GNAI3	0.624739	3.964579	0.000108
ENSG0000(	PC	0.624705	2.547844	0.002832
ENSG0000(	PLD1	0.624655	2.01211	0.009725
ENSG0000(	NFATC4	0.624626	2.044489	0.009026
ENSG0000(	MYO5A	0.624464	3.356847	0.00044
ENSG0000(	SLC44A1	0.624374	2.76933	0.001701
ENSG0000(	DPYSL3	0.624323	2.794317	0.001606
ENSG0000(	TMEM123	0.62401	4.153664	7.02E-05
ENSG0000(	ZEB1-AS1	0.623509	5.851623	1.41E-06
ENSG0000(	AC127024.	0.623287	3.039528	0.000913
ENSG0000(	ICAM3	0.623278	1.978607	0.010505
ENSG0000(	IKBIP	0.623212	1.976807	0.010549
ENSG0000(	SYT11	0.622997	2.472097	0.003372
ENSG0000(	EME1	0.622993	2.096198	0.008013
ENSG0000(	ANXA2R	0.622918	2.87835	0.001323
ENSG0000(	WTIP	0.62249	2.382504	0.004145
ENSG0000(	SLC25A5-A	0.622427	5.906584	1.24E-06
ENSG0000(	SEMA3F	0.622306	2.270177	0.005368
ENSG0000(	PAPSS2	0.62176	1.352222	0.04444
ENSG0000(	CDC42EP5	0.621623	2.010723	0.009756
ENSG0000(	ZNF559-ZN	0.621395	3.238416	0.000578
ENSG0000(	STS	0.620971	3.062407	0.000866
ENSG0000(	DPP3	0.620944	4.745122	1.80E-05
ENSG0000(	TOP2A	0.620771	1.691521	0.020346
ENSG0000(	SFXN2	0.620705	1.727804	0.018715
ENSG0000(	N4BP2L1	0.620549	4.885745	1.30E-05
ENSG0000(	CDR2	0.620202	4.331732	4.66E-05

ENSG0000(	MYF5	0.62014	1.82945	0.01481
ENSG0000(	RAD51B	0.619741	2.771576	0.001692
ENSG0000(	NPTXR	0.619722	1.484815	0.032748
ENSG0000(	VMAC	0.619605	3.447197	0.000357
ENSG0000(	ATP11C	0.619448	2.265428	0.005427
ENSG0000(	MORN4	0.619102	2.408715	0.003902
ENSG0000(	LRRC8D	0.618853	4.321992	4.76E-05
ENSG0000(	B3GAT3	0.618084	5.969003	1.07E-06
ENSG0000(	AC004997.	0.617727	3.42847	0.000373
ENSG0000(	NBPF11	0.617651	5.697529	2.01E-06
ENSG0000(	HVCN1	0.617522	2.622598	0.002385
ENSG0000(	NBPF12	0.617144	4.672374	2.13E-05
ENSG0000(	ZNFX1	0.616709	5.906407	1.24E-06
ENSG0000(	CDC6	0.616657	1.390701	0.040672
ENSG0000(	ZNF345	0.616518	3.095187	0.000803
ENSG0000(	RCBTB1	0.616216	5.237024	5.79E-06
ENSG0000(	MAP7D3	0.615867	3.570872	0.000269
ENSG0000(	TPST1	0.615127	1.41721	0.038264
ENSG0000(	FRG1CP	0.61498	1.34493	0.045193
ENSG0000(	RF02216	0.614901	1.546244	0.028429
ENSG0000(	ALDH1A1	0.61439	2.158776	0.006938
ENSG0000(	PPFIA3	0.614057	2.178878	0.006624
ENSG0000(	TIGD1	0.613906	1.663478	0.021703
ENSG0000(	CAPRIN2	0.613887	2.31817	0.004807
ENSG0000(	SLC30A4	0.613692	2.834933	0.001462
ENSG0000(	SLIT3	0.613687	1.349762	0.044693
ENSG0000(	CENPF	0.613429	1.989044	0.010255
ENSG0000(	VSIR	0.61301	3.345997	0.000451
ENSG0000(	TMEM140	0.612887	3.9298	0.000118
ENSG0000(	AC124319.	0.612821	3.806451	0.000156
ENSG0000(	PSMB10	0.612747	2.317973	0.004809
ENSG0000(	PUM3	0.612616	4.058405	8.74E-05
ENSG0000(	SPATA33	0.612015	2.64513	0.002264
ENSG0000(	FUT8	0.611648	2.35852	0.00438
ENSG0000(	IER3-AS1	0.611421	1.768267	0.01705
ENSG0000(	SLC16A2	0.611159	2.717299	0.001917
ENSG0000(	TRIM27	0.610831	8.318802	4.80E-09
ENSG0000(	LAMA4	0.610618	1.366226	0.04303
ENSG0000(	LOXL3	0.61	4.157038	6.97E-05
ENSG0000(	CDCA3	0.609851	1.851767	0.014068
ENSG0000(	AC027307.	0.609735	1.49276	0.032154
ENSG0000(	STYXL1	0.60968	3.930839	0.000117
ENSG0000(	RARA-AS1	0.609666	2.185647	0.006522
ENSG0000(	CAMK1	0.609472	2.686099	0.00206
ENSG0000(	CD9	0.608708	2.552403	0.002803
ENSG0000(	CAMK2D	0.608428	3.036696	0.000919
ENSG0000(	RIT1	0.607776	7.791847	1.61E-08

ENSG0000(	ZFAS1	0.607175	1.653435	0.022211
ENSG0000(	FERP1	0.607151	2.287101	0.005163
ENSG0000(	SDCBP2-AS	0.606822	3.699703	0.0002
ENSG0000(	KDELC1	0.606724	1.368479	0.042808
ENSG0000(	CBWD6	0.606695	1.547864	0.028323
ENSG0000(	ALDH1L2	0.606534	1.989357	0.010248
ENSG0000(	IGFBP3	0.606347	1.329101	0.04687
ENSG0000(	RRAGC	0.606086	7.363014	4.33E-08
ENSG0000(	H19	0.605857	1.486527	0.032619
ENSG0000(	GALNS	0.605763	3.25985	0.00055
ENSG0000(	AC012073.	0.605716	2.048184	0.00895
ENSG0000(	ENO1	0.605412	6.97938	1.05E-07
ENSG0000(	B3GNT2	0.60527	2.48754	0.003254
ENSG0000(	RHOC	0.605074	3.724619	0.000189
ENSG0000(	UBE2J1	0.604203	2.116067	0.007655
ENSG0000(	TRNAU1AP	0.603889	7.139695	7.25E-08
ENSG0000(	ERF	0.603722	4.357968	4.39E-05
ENSG0000(	PROX2	0.603714	2.467146	0.003411
ENSG0000(	PTPRA	0.603306	4.408483	3.90E-05
ENSG0000(	BAIAP2	0.603172	2.27886	0.005262
ENSG0000(	RDH11	0.603157	3.236101	0.000581
ENSG0000(	PNMA1	0.603013	4.579905	2.63E-05
ENSG0000(	AC011530.	0.602961	2.110947	0.007746
ENSG0000(	MFSD12	0.602549	8.755224	1.76E-09
ENSG0000(	GNG12	0.602396	2.601079	0.002506
ENSG0000(	VWCE	0.60231	1.856169	0.013926
ENSG0000(	SACM1L	0.602153	4.641131	2.28E-05
ENSG0000(	AC092953.	0.602016	2.726064	0.001879
ENSG0000(	AL670729.:	0.601342	2.226336	0.005938
ENSG0000(	AC010542.	0.600362	1.713451	0.019344
ENSG0000(	KIF2A	0.600338	5.20948	6.17E-06
ENSG0000(	WLS	0.600324	3.308879	0.000491
ENSG0000(	CD160	0.600095	1.435905	0.036652
ENSG0000(	ELF1	0.599833	5.09037	8.12E-06
ENSG0000(	TK2	0.599764	3.933034	0.000117
ENSG0000(	EFHC1	0.599744	3.201197	0.000629
ENSG0000(	DM1-AS	0.599521	2.060254	0.008705
ENSG0000(	NUP93	0.599452	6.36098	4.36E-07
ENSG0000(	GPX3	0.59922	2.994208	0.001013
ENSG0000(	TTF2	0.599132	4.892933	1.28E-05
ENSG0000(	GDAP2	0.598974	2.031399	0.009303
ENSG0000(	DCLRE1B	0.59863	3.06409	0.000863
ENSG0000(	CD27-AS1	0.598319	5.3161	4.83E-06
ENSG0000(	PLOD3	0.597969	5.407237	3.92E-06
ENSG0000(	KIF3B	0.597673	3.045524	0.0009
ENSG0000(	SLC35C1	0.597455	3.509361	0.000309
ENSG0000(	ADPGK-AS:	0.597269	1.783464	0.016464

ENSG0000(	DNAJC1	0.596122	1.629451	0.023472
ENSG0000(	FKBP7	0.595109	2.485574	0.003269
ENSG0000(	HOXB7	0.594989	1.412397	0.03869
ENSG0000(	SATB2	0.594616	1.783374	0.016467
ENSG0000(	DENND3	0.594547	2.275	0.005309
ENSG0000(	FCGRT	0.59449	3.92384	0.000119
ENSG0000(	AC135048.	0.594298	3.001498	0.000997
ENSG0000(	SERPINB1	0.59426	4.400259	3.98E-05
ENSG0000(	LINC01534	0.593669	1.387696	0.040955
ENSG0000(	TRPS1	0.593177	2.345196	0.004517
ENSG0000(	C1orf198	0.593032	2.882817	0.00131
ENSG0000(	CTDSPL	0.59255	2.940915	0.001146
ENSG0000(	PLCD1	0.592185	3.837094	0.000146
ENSG0000(	TMEM170/	0.592112	5.670556	2.14E-06
ENSG0000(	LUZP1	0.591848	3.925122	0.000119
ENSG0000(	HIP1	0.591838	5.658612	2.19E-06
ENSG0000(	RF01972	0.591769	1.388238	0.040904
ENSG0000(	ATP6V1A	0.591667	7.245532	5.68E-08
ENSG0000(	CSGALNAC	0.591627	6.411723	3.88E-07
ENSG0000(	HIVEP3	0.591612	3.252126	0.00056
ENSG0000(	TAF1A	0.591489	2.803532	0.001572
ENSG0000(	ATP13A3	0.591154	7.574751	2.66E-08
ENSG0000(	CREG1	0.591102	6.273955	5.32E-07
ENSG0000(	SPTLC2	0.590854	3.88601	0.00013
ENSG0000(	AC020934.	0.59064	1.601245	0.025047
ENSG0000(	COL6A3	0.590525	1.489709	0.032381
ENSG0000(	ADAM19	0.589474	2.373894	0.004228
ENSG0000(	CACHD1	0.58926	1.759771	0.017387
ENSG0000(	PTTG1IP	0.588846	5.954668	1.11E-06
ENSG0000(	QSOX2	0.588513	3.950425	0.000112
ENSG0000(	ZNF48	0.58847	2.840043	0.001445
ENSG0000(	TUSC3	0.587744	2.54114	0.002876
ENSG0000(	DNAH1	0.587734	6.511156	3.08E-07
ENSG0000(	SAP25	0.587067	1.494239	0.032045
ENSG0000(	AC080038.	0.587027	1.359576	0.043694
ENSG0000(	MPZL1	0.586726	2.469875	0.003389
ENSG0000(	OS9	0.586544	4.564531	2.73E-05
ENSG0000(	ERAP1	0.586261	5.754412	1.76E-06
ENSG0000(	TMEM51-A	0.586247	1.4961	0.031908
ENSG0000(	TGDS	0.586074	3.747774	0.000179
ENSG0000(	F11R	0.58585	3.803343	0.000157
ENSG0000(	AIFM2	0.585783	1.344976	0.045188
ENSG0000(	AC009139.	0.585682	1.379587	0.041727
ENSG0000(	ASPRV1	0.58566	2.404294	0.003942
ENSG0000(	AP001029.	0.585456	2.322518	0.004759
ENSG0000(	DUS3L	-0.58516	6.032834	9.27E-07
ENSG0000(	AL136309.4	-0.58524	3.023982	0.000946

ENSG0000(	SMG1P1	-0.58527	3.414141	0.000385
ENSG0000(	BAG3	-0.58528	3.170432	0.000675
ENSG0000(	CNST	-0.58545	3.239459	0.000576
ENSG0000(	AC007743.	-0.58574	2.597584	0.002526
ENSG0000(	SMOX	-0.58603	4.32302	4.75E-05
ENSG0000(	MPST	-0.58609	2.037262	0.009178
ENSG0000(	ATP5PF	-0.58624	4.405796	3.93E-05
ENSG0000(	EPN1	-0.58635	6.594461	2.54E-07
ENSG0000(	PATJ	-0.58655	1.431083	0.037061
ENSG0000(	MFGE8	-0.58743	5.883671	1.31E-06
ENSG0000(	AC008763.	-0.58789	6.150997	7.06E-07
ENSG0000(	RPS11	-0.58793	3.148639	0.00071
ENSG0000(	AUNIP	-0.58864	2.750932	0.001774
ENSG0000(	SLC7A5P2	-0.58888	1.741206	0.018147
ENSG0000(	RORA	-0.58932	3.634362	0.000232
ENSG0000(	NDUFB2-A!	-0.58972	4.251624	5.60E-05
ENSG0000(	C1QTNF9	-0.58979	2.256755	0.005537
ENSG0000(	OSBPL1A	-0.58986	3.666788	0.000215
ENSG0000(	SLFNL1-AS:	-0.59002	4.442425	3.61E-05
ENSG0000(	RPL14P1	-0.59009	2.617301	0.002414
ENSG0000(	ELL	-0.59116	5.303207	4.98E-06
ENSG0000(	RPS2P46	-0.59132	1.357617	0.043892
ENSG0000(	PCBD2	-0.59137	2.938267	0.001153
ENSG0000(	PYGO1	-0.59173	2.833097	0.001469
ENSG0000(	CDKN1C	-0.59181	4.278237	5.27E-05
ENSG0000(	NEXN-AS1	-0.59187	1.770978	0.016944
ENSG0000(	CCNB1IP1	-0.59191	3.660995	0.000218
ENSG0000(	AC093627.	-0.59198	1.998483	0.010035
ENSG0000(	AC105345.	-0.59232	1.993088	0.01016
ENSG0000(	ZNF232	-0.59233	6.170988	6.75E-07
ENSG0000(	TMED1	-0.5926	6.602666	2.50E-07
ENSG0000(	ESRP2	-0.59264	2.397934	0.004
ENSG0000(	USP38	-0.59267	3.484864	0.000327
ENSG0000(	SYPL1	-0.59271	10.37698	4.20E-11
ENSG0000(	DNAJA3	-0.59295	3.782582	0.000165
ENSG0000(	NDUFC2-K(	-0.59364	8.426328	3.75E-09
ENSG0000(	DUSP7	-0.59371	3.297338	0.000504
ENSG0000(	MRPL34	-0.59416	4.057508	8.76E-05
ENSG0000(	HOXA9	-0.59418	2.993482	0.001015
ENSG0000(	USP53	-0.59425	4.348726	4.48E-05
ENSG0000(	AL020996.:	-0.59434	2.707717	0.00196
ENSG0000(	FAM189A2	-0.59452	2.500044	0.003162
ENSG0000(	KMT2E-AS:	-0.59456	4.519852	3.02E-05
ENSG0000(	PPP1CB	-0.5948	3.610774	0.000245
ENSG0000(	FMC1	-0.59482	4.062439	8.66E-05
ENSG0000(	FOXO4	-0.59502	2.459367	0.003472
ENSG0000(	PRX	-0.59543	1.808709	0.015534

ENSG0000(	AC126696.	-0.59563	3.156646	0.000697
ENSG0000(	LAPTM4B	-0.5959	2.849909	0.001413
ENSG0000(	AC093899.	-0.59607	4.051769	8.88E-05
ENSG0000(	AL162231.4	-0.59631	3.414018	0.000385
ENSG0000(	MZT2A	-0.59643	4.198762	6.33E-05
ENSG0000(	DCTPP1	-0.59656	8.460661	3.46E-09
ENSG0000(	NDUFS3	-0.59678	5.741655	1.81E-06
ENSG0000(	FBXO32	-0.59721	2.767781	0.001707
ENSG0000(	GPS1	-0.59743	8.067433	8.56E-09
ENSG0000(	AL021368.1	-0.59746	2.367298	0.004292
ENSG0000(	PHKB	-0.59755	3.140457	0.000724
ENSG0000(	LINC02067	-0.59757	1.97728	0.010537
ENSG0000(	HINT3	-0.59766	3.157133	0.000696
ENSG0000(	GNG5	-0.5977	4.122299	7.55E-05
ENSG0000(	AC079848.	-0.59803	1.893399	0.012782
ENSG0000(	MT-TP	-0.59837	2.304354	0.004962
ENSG0000(	DHRS7B	-0.59838	12.15795	6.95E-13
ENSG0000(	FBXO34	-0.59867	8.443931	3.60E-09
ENSG0000(	BCAR1	-0.59882	5.203357	6.26E-06
ENSG0000(	ZNF106	-0.59883	1.845748	0.014264
ENSG0000(	SIX1	-0.59891	3.34867	0.000448
ENSG0000(	DDI2	-0.5991	4.803026	1.57E-05
ENSG0000(	AC138811.	-0.59917	2.139474	0.007253
ENSG0000(	USP2	-0.59943	2.11172	0.007732
ENSG0000(	SPDYA	-0.59949	3.108929	0.000778
ENSG0000(	RPL21	-0.59977	2.849442	0.001414
ENSG0000(	MRPL58	-0.59983	6.645124	2.26E-07
ENSG0000(	NFATC3	-0.59991	6.64435	2.27E-07
ENSG0000(	ZGRF1	-0.6001	2.94247	0.001142
ENSG0000(	MAST2	-0.60033	3.070518	0.00085
ENSG0000(	CSTF2T	-0.60069	6.644454	2.27E-07
ENSG0000(	AC087289.	-0.60072	4.7727	1.69E-05
ENSG0000(	NDRG2	-0.60079	2.460413	0.003464
ENSG0000(	BEND7	-0.60089	4.620723	2.39E-05
ENSG0000(	GPLD1	-0.60098	2.686807	0.002057
ENSG0000(	EIF3CL	-0.60104	1.751689	0.017714
ENSG0000(	UBAP2	-0.60111	3.057285	0.000876
ENSG0000(	MAP4K3	-0.6012	6.473015	3.37E-07
ENSG0000(	FUZ	-0.60138	4.219721	6.03E-05
ENSG0000(	AC091060.	-0.60182	6.321027	4.77E-07
ENSG0000(	MT-ND4L	-0.60197	1.557436	0.027705
ENSG0000(	TRIM28	-0.60222	6.040028	9.12E-07
ENSG0000(	SETBP1	-0.60226	4.645294	2.26E-05
ENSG0000(	AP003721.	-0.60229	2.344747	0.004521
ENSG0000(	AC138150.	-0.60242	1.97392	0.010619
ENSG0000(	GTPBP4	-0.60276	1.904159	0.012469
ENSG0000(	RPS23	-0.60289	3.802291	0.000158

ENSG0000(	ZNF418	-0.60294	3.240889	0.000574
ENSG0000(	AL645941.:	-0.60294	2.053293	0.008845
ENSG0000(	ANKRD46	-0.60298	3.094055	0.000805
ENSG0000(	CTH	-0.60306	1.596145	0.025343
ENSG0000(	UBL4A	-0.6034	3.497372	0.000318
ENSG0000(	SULT6B1	-0.60349	3.926693	0.000118
ENSG0000(	CBLL1	-0.60367	7.570095	2.69E-08
ENSG0000(	SLC9A5	-0.60377	1.890133	0.012879
ENSG0000(	TOGARAM	-0.60381	5.535541	2.91E-06
ENSG0000(	CEBPD	-0.60396	1.353294	0.044331
ENSG0000(	NCOA3	-0.60397	3.376694	0.00042
ENSG0000(	COPS9	-0.60399	4.788566	1.63E-05
ENSG0000(	SUCLG2-AS	-0.60435	1.37832	0.041848
ENSG0000(	AC010335.	-0.60467	2.890368	0.001287
ENSG0000(	MIR6501	-0.60472	3.723996	0.000189
ENSG0000(	JSRP1	-0.60498	7.529887	2.95E-08
ENSG0000(	AC104794.	-0.605	2.271562	0.005351
ENSG0000(	DAB2IP	-0.6053	3.76986	0.00017
ENSG0000(	UQCRB	-0.60554	5.076293	8.39E-06
ENSG0000(	ARL6IP4	-0.60556	9.06309	8.65E-10
ENSG0000(	AC009065.	-0.60572	2.946863	0.00113
ENSG0000(	AC124312.	-0.60598	1.75301	0.01766
ENSG0000(	MRPL12	-0.60612	3.746049	0.000179
ENSG0000(	MRPL20	-0.60697	7.333521	4.64E-08
ENSG0000(	SEMA6C	-0.60709	2.274013	0.005321
ENSG0000(	AL138724.:	-0.60723	3.686118	0.000206
ENSG0000(	AC034102.	-0.6075	3.422642	0.000378
ENSG0000(	ZNF671	-0.60788	2.961075	0.001094
ENSG0000(	WRB	-0.60799	6.67413	2.12E-07
ENSG0000(	KCNA7	-0.60804	1.49414	0.032052
ENSG0000(	COX10	-0.60813	2.325006	0.004731
ENSG0000(	NUBP2	-0.60825	7.24919	5.63E-08
ENSG0000(	PMF1-BGL/	-0.60847	6.453306	3.52E-07
ENSG0000(	AC006504.	-0.60872	1.608634	0.024624
ENSG0000(	IMPDH2	-0.60878	7.147702	7.12E-08
ENSG0000(	AL354920.:	-0.6088	4.403491	3.95E-05
ENSG0000(	AL109955.:	-0.60888	1.85199	0.014061
ENSG0000(	ATP5MC1	-0.60894	3.297253	0.000504
ENSG0000(	THRB	-0.60902	3.462255	0.000345
ENSG0000(	AMPD1	-0.60958	1.933607	0.011652
ENSG0000(	PHPT1	-0.61019	4.424158	3.77E-05
ENSG0000(	KIAA0232	-0.61043	6.052997	8.85E-07
ENSG0000(	ANOS1	-0.61058	2.568752	0.002699
ENSG0000(	MPC2	-0.61097	3.719491	0.000191
ENSG0000(	CCDC84	-0.61103	4.541949	2.87E-05
ENSG0000(	CHRAC1	-0.61114	4.66715	2.15E-05
ENSG0000(	FXN	-0.61145	3.292086	0.00051

ENSG0000(	UQCRFS1P	-0.61147	2.020678	0.009535
ENSG0000(	NDFIP2	-0.61163	5.597418	2.53E-06
ENSG0000(	AL354892.:	-0.61164	1.556768	0.027748
ENSG0000(	MIR6717	-0.61182	2.507998	0.003105
ENSG0000(	RN7SKP70	-0.61186	3.807534	0.000156
ENSG0000(	KLHL41	-0.61191	4.424158	3.77E-05
ENSG0000(	HDAC11	-0.61194	1.790368	0.016204
ENSG0000(	HECTD2	-0.61211	2.671735	0.002129
ENSG0000(	FBXW5	-0.61232	5.507694	3.11E-06
ENSG0000(	PRR34	-0.6126	2.34135	0.004557
ENSG0000(	H1F0	-0.61277	8.361801	4.35E-09
ENSG0000(	UQCRHL	-0.61287	2.118612	0.00761
ENSG0000(	TARSL2	-0.613	2.483815	0.003282
ENSG0000(	DDIT4L	-0.61329	1.718832	0.019106
ENSG0000(	ASB14	-0.61357	4.011106	9.75E-05
ENSG0000(	MALSU1	-0.61359	6.090032	8.13E-07
ENSG0000(	AC026464.	-0.61387	5.346917	4.50E-06
ENSG0000(	AP000692.	-0.61389	2.489075	0.003243
ENSG0000(	AC022509.	-0.61394	3.438391	0.000364
ENSG0000(	CAP2	-0.61395	2.651913	0.002229
ENSG0000(	NOC4L	-0.61411	7.088804	8.15E-08
ENSG0000(	PPP6C	-0.61419	9.417723	3.82E-10
ENSG0000(	KLHL33	-0.61481	1.337545	0.045968
ENSG0000(	ST3GAL1	-0.61491	2.526885	0.002972
ENSG0000(	CNNM2	-0.61506	5.751019	1.77E-06
ENSG0000(	KCNS3	-0.61515	2.403777	0.003947
ENSG0000(	FKBP4	-0.61517	5.503719	3.14E-06
ENSG0000(	NBEAL1	-0.61524	1.944423	0.011365
ENSG0000(	ATG10	-0.61547	3.817952	0.000152
ENSG0000(	AL392046.:	-0.61554	2.028808	0.009358
ENSG0000(	PARVB	-0.61602	3.866931	0.000136
ENSG0000(	PLPPR2	-0.61608	3.882043	0.000131
ENSG0000(	AKT1S1	-0.61635	6.497631	3.18E-07
ENSG0000(	FBXO21	-0.61652	2.904527	0.001246
ENSG0000(	AC108488.	-0.61666	2.56552	0.002719
ENSG0000(	AC012313.	-0.61667	3.32736	0.000471
ENSG0000(	AL358852.:	-0.61685	2.757192	0.001749
ENSG0000(	GOT2	-0.61762	1.713048	0.019362
ENSG0000(	CTF1	-0.61766	2.970431	0.00107
ENSG0000(	C19orf53	-0.61769	6.144504	7.17E-07
ENSG0000(	HADH	-0.61848	2.677829	0.0021
ENSG0000(	GNAL	-0.61909	5.645982	2.26E-06
ENSG0000(	CEP85	-0.61941	2.418577	0.003814
ENSG0000(	COX7C	-0.61974	5.29475	5.07E-06
ENSG0000(	ZNF620	-0.61983	3.480621	0.000331
ENSG0000(	NFATC1	-0.61997	3.002686	0.000994
ENSG0000(	CELSR2	-0.62001	1.598504	0.025206

ENSG0000(	MYBBP1A	-0.62009	3.027425	0.000939
ENSG0000(	AC027682.	-0.62039	1.318718	0.048005
ENSG0000(	TCAP	-0.62142	1.775506	0.016768
ENSG0000(	CLPP	-0.62149	6.270736	5.36E-07
ENSG0000(	FHOD1	-0.62168	1.986679	0.010311
ENSG0000(	AL445183.:	-0.62174	2.272762	0.005336
ENSG0000(	AC130343.	-0.62189	1.762083	0.017295
ENSG0000(	PNMT	-0.62201	1.435519	0.036684
ENSG0000(	PKD1	-0.62202	4.058377	8.74E-05
ENSG0000(	WDR38	-0.62213	2.912372	0.001224
ENSG0000(	UBAP1L	-0.62234	4.118938	7.60E-05
ENSG0000(	HDAC5	-0.62245	7.152247	7.04E-08
ENSG0000(	BANF1	-0.62287	5.026757	9.40E-06
ENSG0000(	PSMA7	-0.62323	6.267169	5.41E-07
ENSG0000(	AC005696.	-0.6242	2.061612	0.008677
ENSG0000(	AC074044.	-0.62429	3.052694	0.000886
ENSG0000(	C6orf136	-0.62499	3.33116	0.000466
ENSG0000(	MIR24-1	-0.62506	2.84823	0.001418
ENSG0000(	WASH8P	-0.62517	1.818218	0.015198
ENSG0000(	TLN2	-0.6256	2.216294	0.006077
ENSG0000(	FGFRL1	-0.62635	2.642605	0.002277
ENSG0000(	SNRNP25	-0.62646	5.79608	1.60E-06
ENSG0000(	AC132938.	-0.6266	2.082193	0.008276
ENSG0000(	ACSL6	-0.62704	1.734206	0.018441
ENSG0000(	MIR6511B:	-0.62744	2.487236	0.003257
ENSG0000(	FYCO1	-0.62774	1.53445	0.029211
ENSG0000(	HSBP1L1	-0.62787	2.221098	0.00601
ENSG0000(	ANAPC15	-0.62812	5.457511	3.49E-06
ENSG0000(	SMARCD3	-0.62838	4.537211	2.90E-05
ENSG0000(	ANAPC11	-0.62894	5.728074	1.87E-06
ENSG0000(	TMEM161I	-0.62903	2.718251	0.001913
ENSG0000(	PMF1	-0.62906	6.23366	5.84E-07
ENSG0000(	MIR3074	-0.62951	2.643207	0.002274
ENSG0000(	MYADML2	-0.63035	2.059645	0.008717
ENSG0000(	ACSS2	-0.63048	2.570938	0.002686
ENSG0000(	AC008453.	-0.6311	1.368104	0.042845
ENSG0000(	MT-ATP6	-0.63122	2.068993	0.008531
ENSG0000(	HEBP2	-0.63126	6.876315	1.33E-07
ENSG0000(	LRRC30	-0.63135	1.604551	0.024857
ENSG0000(	UBE2D1	-0.63164	3.096203	0.000801
ENSG0000(	ANKRD34A	-0.63174	2.187916	0.006488
ENSG0000(	KLHL11	-0.63195	1.732307	0.018522
ENSG0000(	AC022400.	-0.63266	2.56634	0.002714
ENSG0000(	IGF2BP2	-0.63448	2.230571	0.005881
ENSG0000(	AC079807.	-0.63456	2.489075	0.003243
ENSG0000(	SLC1A1	-0.63464	2.546747	0.00284
ENSG0000(	MYPOP	-0.63484	4.485247	3.27E-05

ENSG0000(	ACACB	-0.63514	1.976925	0.010546
ENSG0000(	AC012510.	-0.63568	2.440513	0.003626
ENSG0000(	CEP350	-0.63678	4.678118	2.10E-05
ENSG0000(	DVL1	-0.63684	2.315808	0.004833
ENSG0000(	ZNF524	-0.63695	6.625158	2.37E-07
ENSG0000(	HECTD1	-0.63702	3.832088	0.000147
ENSG0000(	SLC25A4	-0.63705	1.955004	0.011092
ENSG0000(	ANKH	-0.63732	5.854167	1.40E-06
ENSG0000(	WWP1	-0.63738	3.497561	0.000318
ENSG0000(	COX17	-0.63743	7.014161	9.68E-08
ENSG0000(	ACO2	-0.63777	2.316307	0.004827
ENSG0000(	DPP6	-0.63795	1.523216	0.029977
ENSG0000(	PLCD3	-0.63829	2.422929	0.003776
ENSG0000(	MT-RNR1	-0.63842	2.898942	0.001262
ENSG0000(	MT-ATP8	-0.63889	1.877553	0.013257
ENSG0000(	SMDT1	-0.63892	5.768549	1.70E-06
ENSG0000(	AP003396.	-0.63902	1.922255	0.01196
ENSG0000(	FRAS1	-0.63945	1.770051	0.01698
ENSG0000(	ECPAS	-0.63954	4.068677	8.54E-05
ENSG0000(	ZNF710-AS	-0.63959	1.673449	0.021211
ENSG0000(	ECHDC2	-0.64056	3.48528	0.000327
ENSG0000(	HSPB1	-0.64071	2.570791	0.002687
ENSG0000(	TUT4	-0.64106	6.992738	1.02E-07
ENSG0000(	KCNJ11	-0.64138	2.154551	0.007006
ENSG0000(	ZNF628	-0.64152	6.079821	8.32E-07
ENSG0000(	STK25	-0.64178	4.942379	1.14E-05
ENSG0000(	AL355472.:	-0.64202	4.504144	3.13E-05
ENSG0000(	INTS1	-0.64234	4.164906	6.84E-05
ENSG0000(	SLC25A23	-0.64235	3.965624	0.000108
ENSG0000(	TMTC4	-0.64236	3.904381	0.000125
ENSG0000(	EIF3G	-0.64251	6.186386	6.51E-07
ENSG0000(	RPL13AP5	-0.64258	3.739458	0.000182
ENSG0000(	SYNM	-0.6437	1.437946	0.03648
ENSG0000(	INPP5A	-0.64376	2.837412	0.001454
ENSG0000(	NDUFA2	-0.64414	6.91425	1.22E-07
ENSG0000(	FUNDC2	-0.64416	5.264063	5.44E-06
ENSG0000(	NECAB3	-0.64435	3.189809	0.000646
ENSG0000(	AC136469.	-0.64469	7.098919	7.96E-08
ENSG0000(	AL354733.:	-0.64481	3.771479	0.000169
ENSG0000(	INSR	-0.64485	3.400255	0.000398
ENSG0000(	KBTBD11	-0.64507	2.021657	0.009514
ENSG0000(	PRKCQ	-0.64517	3.624525	0.000237
ENSG0000(	AL031595.:	-0.6455	4.574063	2.67E-05
ENSG0000(	TBC1D17	-0.64602	4.864534	1.37E-05
ENSG0000(	LIPT2	-0.6461	2.830707	0.001477
ENSG0000(	VAPB	-0.64642	4.285825	5.18E-05
ENSG0000(	MIR6784	-0.64748	1.38499	0.041211

ENSG0000(	AC133552.	-0.64753	2.710829	0.001946
ENSG0000(	AC007846.	-0.6477	1.453338	0.03521
ENSG0000(	B3GALNT2	-0.64773	3.383237	0.000414
ENSG0000(	C10orf71	-0.64778	1.846421	0.014242
ENSG0000(	KAT2A	-0.64837	2.873033	0.00134
ENSG0000(	RPL10A	-0.64852	4.336698	4.61E-05
ENSG0000(	MRPL10	-0.64863	10.62524	2.37E-11
ENSG0000(	PRICKLE3	-0.64881	5.689597	2.04E-06
ENSG0000(	CAV3	-0.64901	3.218494	0.000605
ENSG0000(	USP24	-0.64911	3.409282	0.00039
ENSG0000(	AC073111.	-0.64953	3.041701	0.000908
ENSG0000(	RPL4P4	-0.65017	5.167333	6.80E-06
ENSG0000(	RPS28	-0.65025	3.511605	0.000308
ENSG0000(	FLJ42351	-0.65028	1.979476	0.010484
ENSG0000(	AC073389.	-0.65036	1.884233	0.013055
ENSG0000(	PDE4B	-0.65045	5.307084	4.93E-06
ENSG0000(	AC073333.	-0.65071	2.645708	0.002261
ENSG0000(	AR	-0.65081	3.567213	0.000271
ENSG0000(	COQ7	-0.65102	5.154401	7.01E-06
ENSG0000(	FILIP1L	-0.65123	4.025037	9.44E-05
ENSG0000(	RPL13	-0.65138	4.449241	3.55E-05
ENSG0000(	PLOD1	-0.65172	10.25534	5.55E-11
ENSG0000(	MSRB2	-0.65177	5.38255	4.14E-06
ENSG0000(	AP000721.	-0.6519	5.739872	1.82E-06
ENSG0000(	WHAMMP:	-0.65231	3.447738	0.000357
ENSG0000(	PIK3CB	-0.65256	5.0181	9.59E-06
ENSG0000(	AL136164.4	-0.65279	2.794142	0.001606
ENSG0000(	THAP4	-0.6529	6.025831	9.42E-07
ENSG0000(	AC009119.	-0.65329	1.890274	0.012874
ENSG0000(	UPK3BL1	-0.65383	1.357676	0.043886
ENSG0000(	AHCYL1	-0.65424	6.919014	1.20E-07
ENSG0000(	SMG1P3	-0.65433	2.712448	0.001939
ENSG0000(	RPS5	-0.65491	4.962627	1.09E-05
ENSG0000(	TRAPPC9	-0.65498	3.370884	0.000426
ENSG0000(	AL035448.:	-0.65499	3.217949	0.000605
ENSG0000(	MRPL38	-0.65509	5.011258	9.74E-06
ENSG0000(	RAD21-AS1	-0.65527	3.374659	0.000422
ENSG0000(	CNGA1	-0.65653	1.53829	0.028954
ENSG0000(	AC010422.	-0.65671	11.36906	4.27E-12
ENSG0000(	NDUFB7	-0.6568	5.578122	2.64E-06
ENSG0000(	JMJD6	-0.65688	4.176762	6.66E-05
ENSG0000(	SLC25A11	-0.65777	3.589493	0.000257
ENSG0000(	GPD1L	-0.65797	1.873036	0.013396
ENSG0000(	PANK4	-0.65801	5.117317	7.63E-06
ENSG0000(	HOXA3	-0.65817	3.275679	0.00053
ENSG0000(	CWC15	-0.65847	8.172324	6.72E-09
ENSG0000(	PKD1P6-NI	-0.65879	5.290931	5.12E-06

ENSG0000(	AC068831.	-0.65927	2.814461	0.001533
ENSG0000(	WFIKKN2	-0.65929	1.554871	0.027869
ENSG0000(	TAF3	-0.6593	5.76665	1.71E-06
ENSG0000(	AC145207.	-0.6594	1.535894	0.029114
ENSG0000(	COL4A6	-0.65964	1.386977	0.041023
ENSG0000(	LMOD3	-0.6598	3.306247	0.000494
ENSG0000(	ZNF511-PR	-0.65987	6.004461	9.90E-07
ENSG0000(	MTFR1L	-0.66005	3.94111	0.000115
ENSG0000(	ERI3	-0.66108	6.690985	2.04E-07
ENSG0000(	IFNLR1	-0.66125	1.759771	0.017387
ENSG0000(	LRP4	-0.66165	2.928088	0.00118
ENSG0000(	MASP2	-0.66168	3.585094	0.00026
ENSG0000(	AL136084.:	-0.6619	1.457272	0.034892
ENSG0000(	SAPCD1-AS	-0.66192	2.711013	0.001945
ENSG0000(	DAPK2	-0.6623	2.541804	0.002872
ENSG0000(	IQCH-AS1	-0.66235	4.720746	1.90E-05
ENSG0000(	RPL4	-0.66253	7.960022	1.10E-08
ENSG0000(	NT5M	-0.66285	3.400255	0.000398
ENSG0000(	C1orf43	-0.66287	9.46513	3.43E-10
ENSG0000(	KCND3	-0.66293	1.929579	0.01176
ENSG0000(	ZBTB44	-0.66306	5.591116	2.56E-06
ENSG0000(	NDUFS6	-0.66331	5.169133	6.77E-06
ENSG0000(	GTF2IRD2B	-0.66473	5.244793	5.69E-06
ENSG0000(	PLCD4	-0.66481	2.637387	0.002305
ENSG0000(	AC135586.	-0.66489	3.334588	0.000463
ENSG0000(	KLHL30	-0.66525	2.591019	0.002564
ENSG0000(	PPP2R3A	-0.66544	3.195675	0.000637
ENSG0000(	THAP11	-0.6659	7.750116	1.78E-08
ENSG0000(	ATP8A1	-0.66623	3.491279	0.000323
ENSG0000(	COX14	-0.66688	5.178577	6.63E-06
ENSG0000(	RFX7	-0.66708	6.457729	3.49E-07
ENSG0000(	ANO5	-0.66732	2.926797	0.001184
ENSG0000(	AP003465.	-0.66779	5.942767	1.14E-06
ENSG0000(	MRPL48	-0.66847	5.962594	1.09E-06
ENSG0000(	TNKS2	-0.66862	6.703124	1.98E-07
ENSG0000(	CAMK2G	-0.66874	3.083281	0.000826
ENSG0000(	TSSC4	-0.66874	9.282472	5.22E-10
ENSG0000(	NEO1	-0.66944	3.563547	0.000273
ENSG0000(	PRR34-AS1	-0.66952	2.804182	0.00157
ENSG0000(	TSPOAP1	-0.66956	3.486341	0.000326
ENSG0000(	POP7	-0.6698	10.75201	1.77E-11
ENSG0000(	ADCY1	-0.67	2.454582	0.003511
ENSG0000(	C10orf71-A	-0.67102	2.678827	0.002095
ENSG0000(	SHMT1	-0.67174	2.718438	0.001912
ENSG0000(	UBAC1	-0.67178	5.787844	1.63E-06
ENSG0000(	ARHGAP10	-0.67206	8.386533	4.11E-09
ENSG0000(	SHARPIN	-0.6721	7.276481	5.29E-08

ENSG0000(	MYO7B	-0.67241	2.08971	0.008134
ENSG0000(	DSG2	-0.67246	1.643057	0.022748
ENSG0000(	HOXA-AS2	-0.67262	3.707407	0.000196
ENSG0000(	AC004951.	-0.67323	5.917801	1.21E-06
ENSG0000(	CHCHD3	-0.67355	3.709566	0.000195
ENSG0000(	NDUFA12	-0.67357	6.582254	2.62E-07
ENSG0000(	UBXN1	-0.67397	6.966357	1.08E-07
ENSG0000(	TUBA4A	-0.67452	2.676235	0.002107
ENSG0000(	PDK2	-0.67467	2.979765	0.001048
ENSG0000(	RAF1	-0.6753	6.8812	1.31E-07
ENSG0000(	APOPT1	-0.67578	5.122233	7.55E-06
ENSG0000(	KEAP1	-0.67593	5.39379	4.04E-06
ENSG0000(	VEZF1	-0.67606	8.012077	9.73E-09
ENSG0000(	AC074032.	-0.67651	6.014347	9.68E-07
ENSG0000(	AURKAIP1	-0.67702	5.345648	4.51E-06
ENSG0000(	LINC02019	-0.67717	1.587948	0.025826
ENSG0000(	FRMD3	-0.67741	3.129185	0.000743
ENSG0000(	NFU1	-0.67758	7.300712	5.00E-08
ENSG0000(	C14orf39	-0.67787	3.099362	0.000795
ENSG0000(	HOXA6	-0.67821	2.841487	0.00144
ENSG0000(	OSBPL11	-0.67846	3.110289	0.000776
ENSG0000(	AL513550.:	-0.67899	4.634362	2.32E-05
ENSG0000(	BLOC1S1	-0.679	6.97386	1.06E-07
ENSG0000(	AMIGO3	-0.67905	4.132985	7.36E-05
ENSG0000(	PHTF2	-0.67915	2.770049	0.001698
ENSG0000(	PRKG1	-0.6797	2.641761	0.002282
ENSG0000(	EIF1	-0.6798	7.39791	4.00E-08
ENSG0000(	LEAP2	-0.67983	3.758239	0.000174
ENSG0000(	PLIN2	-0.67984	4.211243	6.15E-05
ENSG0000(	TDRD6	-0.68004	2.074578	0.008422
ENSG0000(	SHROOM4	-0.68095	3.938266	0.000115
ENSG0000(	SMIM26	-0.68102	5.271212	5.36E-06
ENSG0000(	EMC9	-0.68108	4.491563	3.22E-05
ENSG0000(	DUSP8	-0.68126	2.040798	0.009103
ENSG0000(	BTBD6	-0.68131	3.746727	0.000179
ENSG0000(	SYNE2	-0.6817	7.760192	1.74E-08
ENSG0000(	NDUFB9	-0.68172	6.542299	2.87E-07
ENSG0000(	PNPLA7	-0.68235	2.555327	0.002784
ENSG0000(	ADCY9	-0.68312	2.845207	0.001428
ENSG0000(	SLC19A2	-0.68319	1.647407	0.022521
ENSG0000(	CEP85L	-0.68321	6.071864	8.47E-07
ENSG0000(	DTWD2	-0.68364	3.336591	0.000461
ENSG0000(	METTL26	-0.68414	6.207157	6.21E-07
ENSG0000(	CCDC192	-0.68466	2.698625	0.002002
ENSG0000(	NEURL1	-0.68471	2.046912	0.008976
ENSG0000(	EN1	-0.68494	2.478711	0.003321
ENSG0000(	AC233992.	-0.68499	4.84507	1.43E-05

ENSG0000(	LINC-PINT	-0.68501	2.448214	0.003563
ENSG0000(	UTAT33	-0.68579	4.785696	1.64E-05
ENSG0000(	RNF123	-0.6858	3.005002	0.000989
ENSG0000(	MIR5006	-0.6858	1.901832	0.012536
ENSG0000(	CUL5	-0.68625	5.464122	3.43E-06
ENSG0000(	AL445465.:	-0.6864	2.332374	0.004652
ENSG0000(	ABCB4	-0.68678	2.98689	0.001031
ENSG0000(	C2orf88	-0.687	2.316236	0.004828
ENSG0000(	EMD	-0.68756	7.406035	3.93E-08
ENSG0000(	PFKM	-0.68793	3.356938	0.00044
ENSG0000(	GPATCH8	-0.68815	7.276776	5.29E-08
ENSG0000(	PRAG1	-0.6883	6.117763	7.62E-07
ENSG0000(	RPS21	-0.68882	2.741993	0.001811
ENSG0000(	INKA1	-0.68899	3.274187	0.000532
ENSG0000(	TXNIP	-0.68901	1.896907	0.012679
ENSG0000(	AC005841.	-0.68903	6.224923	5.96E-07
ENSG0000(	RPUSD4	-0.68965	4.731792	1.85E-05
ENSG0000(	ZNF154	-0.69041	2.538992	0.002891
ENSG0000(	RPS13	-0.69043	5.251344	5.61E-06
ENSG0000(	AC005944.	-0.69051	5.032448	9.28E-06
ENSG0000(	ZFAT-AS1	-0.6915	1.712587	0.019383
ENSG0000(	EBAG9	-0.69152	9.331518	4.66E-10
ENSG0000(	AC009065.	-0.69199	2.61916	0.002403
ENSG0000(	SYNGR1	-0.69215	9.700152	1.99E-10
ENSG0000(	TPTEP1	-0.69225	2.917016	0.001211
ENSG0000(	MYOT	-0.6929	2.558286	0.002765
ENSG0000(	AC022706.	-0.69319	1.563683	0.02731
ENSG0000(	AL669841.:	-0.69338	1.327609	0.047032
ENSG0000(	MT-TL1	-0.69343	1.673449	0.021211
ENSG0000(	COX5B	-0.69377	4.932189	1.17E-05
ENSG0000(	AP005263.	-0.69381	6.767131	1.71E-07
ENSG0000(	RPL14	-0.69386	6.884833	1.30E-07
ENSG0000(	AC130462.	-0.69395	4.013117	9.70E-05
ENSG0000(	AC034236.	-0.69411	2.83392	0.001466
ENSG0000(	PHB2	-0.69602	10.29693	5.05E-11
ENSG0000(	DCAF6	-0.69612	4.073242	8.45E-05
ENSG0000(	ZNF865	-0.69663	3.474824	0.000335
ENSG0000(	AP003392.	-0.69708	2.923735	0.001192
ENSG0000(	PLCL1	-0.69731	2.380613	0.004163
ENSG0000(	SCMH1	-0.69773	12.70133	1.99E-13
ENSG0000(	ZYG11B	-0.69796	3.067407	0.000856
ENSG0000(	KPNA3	-0.69812	4.573298	2.67E-05
ENSG0000(	DENND2C	-0.69911	1.816667	0.015252
ENSG0000(	RPL13A	-0.69919	4.869819	1.35E-05
ENSG0000(	PACS2	-0.6994	5.546584	2.84E-06
ENSG0000(	NDUFV2	-0.69985	6.50207	3.15E-07
ENSG0000(	SAR1B	-0.70024	3.627757	0.000236

ENSG0000(	ST13	-0.70026	10.75048	1.78E-11
ENSG0000(	AL109811.:	-0.7004	3.28527	0.000518
ENSG0000(	RPL32	-0.70049	4.092651	8.08E-05
ENSG0000(	RPL8	-0.70074	6.444295	3.60E-07
ENSG0000(	TATDN1	-0.70091	7.234923	5.82E-08
ENSG0000(	MRPS25	-0.7011	7.703366	1.98E-08
ENSG0000(	RPL35	-0.70116	3.642899	0.000228
ENSG0000(	SLC41A3	-0.70148	9.118431	7.61E-10
ENSG0000(	ZNF25	-0.70158	13.30491	4.96E-14
ENSG0000(	ELOB	-0.70198	6.146014	7.14E-07
ENSG0000(	ATP5PO	-0.70408	7.24484	5.69E-08
ENSG0000(	IDH2	-0.70413	2.570826	0.002686
ENSG0000(	AC004080.	-0.70449	4.892933	1.28E-05
ENSG0000(	AC106791.	-0.70456	2.589403	0.002574
ENSG0000(	MT-TD	-0.70477	1.981749	0.010429
ENSG0000(	MRPL11	-0.705	7.507028	3.11E-08
ENSG0000(	GRIP2	-0.70516	2.46395	0.003436
ENSG0000(	SAMD14	-0.70531	3.295533	0.000506
ENSG0000(	MIR196B	-0.70536	1.875801	0.013311
ENSG0000(	MIR600HG	-0.70557	2.246268	0.005672
ENSG0000(	PKM	-0.70572	2.740313	0.001818
ENSG0000(	AC005520.	-0.70603	1.696634	0.020108
ENSG0000(	RPL7AP6	-0.70604	3.773199	0.000169
ENSG0000(	MID2	-0.70627	4.706779	1.96E-05
ENSG0000(	UBE2G1	-0.70643	4.150953	7.06E-05
ENSG0000(	AP000311.	-0.70663	7.442967	3.61E-08
ENSG0000(	ALPK2	-0.70692	2.043922	0.009038
ENSG0000(	RPS2	-0.70735	6.925087	1.19E-07
ENSG0000(	AC005523.	-0.70784	1.86701	0.013583
ENSG0000(	MRPS9	-0.7079	5.423671	3.77E-06
ENSG0000(	AC010323.	-0.70795	7.15448	7.01E-08
ENSG0000(	TECR	-0.7081	5.703705	1.98E-06
ENSG0000(	AL136295.:	-0.70836	4.521713	3.01E-05
ENSG0000(	CISD1	-0.70873	6.673037	2.12E-07
ENSG0000(	CDC25C	-0.70914	2.208439	0.006188
ENSG0000(	PPP1R2	-0.70935	5.826791	1.49E-06
ENSG0000(	DES	-0.70994	2.87988	0.001319
ENSG0000(	AC138904.	-0.71011	2.728816	0.001867
ENSG0000(	TAPT1-AS1	-0.71017	3.076616	0.000838
ENSG0000(	EPM2A	-0.7103	3.806955	0.000156
ENSG0000(	FEM1A	-0.7103	1.858059	0.013866
ENSG0000(	MPP7	-0.71049	2.65656	0.002205
ENSG0000(	UBE2D4	-0.71066	5.981702	1.04E-06
ENSG0000(	RAB12	-0.71126	6.70365	1.98E-07
ENSG0000(	LDB3	-0.71187	2.103807	0.007874
ENSG0000(	RPL10	-0.71187	7.480227	3.31E-08
ENSG0000(	MLYCD	-0.71193	2.385419	0.004117

ENSG0000(	LINC00649	-0.71229	8.276756	5.29E-09
ENSG0000(	RAB28P5	-0.71239	3.358874	0.000438
ENSG0000(	VDAC1	-0.71253	3.67606	0.000211
ENSG0000(	SYT2	-0.71298	3.291315	0.000511
ENSG0000(	VPS72	-0.71306	4.324254	4.74E-05
ENSG0000(	YIPF7	-0.71315	4.865615	1.36E-05
ENSG0000(	UBR3	-0.71343	3.894599	0.000127
ENSG0000(	DPF3	-0.71365	2.356783	0.004398
ENSG0000(	AES	-0.7138	5.511588	3.08E-06
ENSG0000(	BZW2	-0.71424	3.413022	0.000386
ENSG0000(	AL133406.:	-0.71436	4.08264	8.27E-05
ENSG0000(	LINC01128	-0.71439	2.806499	0.001561
ENSG0000(	CDK16	-0.71488	6.97938	1.05E-07
ENSG0000(	MYHAS	-0.71547	1.381855	0.041509
ENSG0000(	COX7B	-0.71606	5.599351	2.52E-06
ENSG0000(	VGLL2	-0.71618	1.671488	0.021307
ENSG0000(	AC093525.	-0.7162	11.11228	7.72E-12
ENSG0000(	NDUFA6	-0.71623	4.930416	1.17E-05
ENSG0000(	ATP1B1	-0.71675	2.941456	0.001144
ENSG0000(	WDR47	-0.71718	3.41563	0.000384
ENSG0000(	SMAD3	-0.71749	5.785623	1.64E-06
ENSG0000(	CUEDC1	-0.71756	4.259974	5.50E-05
ENSG0000(	UBFD1	-0.71816	6.220822	6.01E-07
ENSG0000(	POLR2J4	-0.71858	6.839307	1.45E-07
ENSG0000(	ATL2	-0.71861	5.686935	2.06E-06
ENSG0000(	HHATL-AS1	-0.7189	3.176917	0.000665
ENSG0000(	MAPK12	-0.71908	3.456594	0.000349
ENSG0000(	FOXP2	-0.71914	4.251624	5.60E-05
ENSG0000(	DUSP3	-0.71937	4.442164	3.61E-05
ENSG0000(	NOP16	-0.71951	8.975859	1.06E-09
ENSG0000(	EEF1D	-0.7198	7.734	1.85E-08
ENSG0000(	PTOV1	-0.7199	8.421112	3.79E-09
ENSG0000(	ATP5MC2	-0.7207	8.239493	5.76E-09
ENSG0000(	AC124947.	-0.72105	1.480204	0.033098
ENSG0000(	AC007342.	-0.72111	3.413038	0.000386
ENSG0000(	UBA52	-0.72139	6.711552	1.94E-07
ENSG0000(	KHDRBS3	-0.72146	5.087498	8.18E-06
ENSG0000(	AL137798.:	-0.72151	3.766028	0.000171
ENSG0000(	PNRC1	-0.72204	8.853157	1.40E-09
ENSG0000(	PKD1P6	-0.72215	6.116358	7.65E-07
ENSG0000(	CEBPB-AS1	-0.72216	2.110763	0.007749
ENSG0000(	SMG1P7	-0.7225	2.626039	0.002366
ENSG0000(	PAM16	-0.72276	5.562088	2.74E-06
ENSG0000(	RPS25	-0.72302	4.920281	1.20E-05
ENSG0000(	CACNA2D3	-0.72313	2.179222	0.006619
ENSG0000(	ACTG1P4	-0.72317	2.136337	0.007306
ENSG0000(	DDX59	-0.72386	6.232923	5.85E-07

ENSG0000(	AC027307.	-0.72387	2.229216	0.005899
ENSG0000(	SCX	-0.72444	2.516133	0.003047
ENSG0000(	HOXA10	-0.72452	4.131533	7.39E-05
ENSG0000(	SRRM2	-0.72462	5.698659	2.00E-06
ENSG0000(	ADAMTSL5	-0.72489	2.348257	0.004485
ENSG0000(	TMEM202-	-0.72562	4.854767	1.40E-05
ENSG0000(	APIP	-0.72572	4.37636	4.20E-05
ENSG0000(	AC138811.	-0.72611	4.24386	5.70E-05
ENSG0000(	PLCB1	-0.7263	3.81828	0.000152
ENSG0000(	LBX1-AS1	-0.72641	1.684145	0.020695
ENSG0000(	AC026954.	-0.7269	9.337161	4.60E-10
ENSG0000(	AL136172.:	-0.72713	1.399792	0.03983
ENSG0000(	PER1	-0.72726	1.612026	0.024433
ENSG0000(	OBSCN	-0.72793	1.754663	0.017593
ENSG0000(	COX8A	-0.7281	5.027593	9.38E-06
ENSG0000(	TMTC1	-0.72848	7.155375	6.99E-08
ENSG0000(	QDPR	-0.72854	4.686161	2.06E-05
ENSG0000(	AC092378.	-0.7287	1.811937	0.015419
ENSG0000(	RIC3	-0.72929	4.711498	1.94E-05
ENSG0000(	EEF2K	-0.72954	5.812254	1.54E-06
ENSG0000(	AC053503.	-0.72978	2.804038	0.00157
ENSG0000(	NT5C2	-0.73061	5.898709	1.26E-06
ENSG0000(	SLC35E4	-0.73068	4.37379	4.23E-05
ENSG0000(	CDNF	-0.7312	5.611174	2.45E-06
ENSG0000(	ANKRD39	-0.73122	3.574109	0.000267
ENSG0000(	RPS16	-0.73127	6.722602	1.89E-07
ENSG0000(	AL591848.4	-0.7314	3.201719	0.000628
ENSG0000(	CTDNEP1	-0.73146	6.586513	2.59E-07
ENSG0000(	PKIA	-0.73178	3.586395	0.000259
ENSG0000(	UQCR11	-0.73188	6.088431	8.16E-07
ENSG0000(	URGCP-MR	-0.73227	6.612745	2.44E-07
ENSG0000(	LRP4-AS1	-0.73313	2.444259	0.003595
ENSG0000(	SBK1	-0.73355	3.074918	0.000842
ENSG0000(	BEND5	-0.73368	10.27913	5.26E-11
ENSG0000(	FAM118A	-0.73408	1.951951	0.01117
ENSG0000(	AL360270.:	-0.73442	5.11931	7.60E-06
ENSG0000(	POPDC3	-0.73448	4.443749	3.60E-05
ENSG0000(	EIF4BP6	-0.73463	3.443493	0.00036
ENSG0000(	SPATA9	-0.73463	2.430226	0.003713
ENSG0000(	IDI2-AS1	-0.73509	1.654199	0.022172
ENSG0000(	AC067930.	-0.73558	6.691744	2.03E-07
ENSG0000(	PKNOX2	-0.7356	3.735629	0.000184
ENSG0000(	ATP6V0C	-0.73566	10.86876	1.35E-11
ENSG0000(	GATM	-0.73595	2.775383	0.001677
ENSG0000(	CIPC	-0.7361	4.338497	4.59E-05
ENSG0000(	ANKRD23	-0.73613	3.618247	0.000241
ENSG0000(	U47924.1	-0.73624	3.892403	0.000128

ENSG0000(	SLC25A36	-0.73657	7.45306	3.52E-08
ENSG0000(	AC010619.	-0.73667	5.73126	1.86E-06
ENSG0000(	CMSS1	-0.7373	4.594229	2.55E-05
ENSG0000(	AP001793.	-0.73762	6.740559	1.82E-07
ENSG0000(	P2RY1	-0.738	1.3073	0.049283
ENSG0000(	EEF2	-0.73822	12.70947	1.95E-13
ENSG0000(	CEBPB	-0.73842	2.539009	0.002891
ENSG0000(	PDE4D	-0.73904	3.928736	0.000118
ENSG0000(	AP003084.	-0.73914	2.342306	0.004547
ENSG0000(	ATF4	-0.7398	4.891456	1.28E-05
ENSG0000(	NCBP2L	-0.7399	3.468883	0.00034
ENSG0000(	TBKBP1	-0.74018	3.648924	0.000224
ENSG0000(	AC067930.	-0.74055	7.490768	3.23E-08
ENSG0000(	DNASE1L1	-0.74114	6.340977	4.56E-07
ENSG0000(	ECHDC3	-0.74174	3.330314	0.000467
ENSG0000(	CSPG4P12	-0.74325	1.509586	0.030932
ENSG0000(	GOLGA7B	-0.74357	1.414311	0.03852
ENSG0000(	KLHDC3	-0.74449	4.485466	3.27E-05
ENSG0000(	ALDH1A2	-0.74457	3.336371	0.000461
ENSG0000(	XIST	-0.74481	3.259508	0.00055
ENSG0000(	HSF1	-0.74559	7.054836	8.81E-08
ENSG0000(	AL022238.4	-0.74564	6.83349	1.47E-07
ENSG0000(	CCDC69	-0.74586	3.802291	0.000158
ENSG0000(	AC026398.	-0.74597	2.31391	0.004854
ENSG0000(	GPR157	-0.74807	2.609529	0.002457
ENSG0000(	NDUFA7	-0.74851	6.944409	1.14E-07
ENSG0000(	NDUFA4L2	-0.7487	5.109092	7.78E-06
ENSG0000(	MIR3610	-0.74875	3.520055	0.000302
ENSG0000(	CGREF1	-0.74903	4.32302	4.75E-05
ENSG0000(	SERINC2	-0.74937	3.460535	0.000346
ENSG0000(	CDH4	-0.74974	3.640646	0.000229
ENSG0000(	POLR2J2	-0.74991	1.387617	0.040962
ENSG0000(	RPL27	-0.75011	4.003416	9.92E-05
ENSG0000(	LSMEM2	-0.75027	5.225417	5.95E-06
ENSG0000(	SAMM50	-0.75054	8.421372	3.79E-09
ENSG0000(	MID1IP1-A	-0.75252	3.059512	0.000872
ENSG0000(	SNHG26	-0.75265	3.647996	0.000225
ENSG0000(	MTLN	-0.75268	2.990855	0.001021
ENSG0000(	KANSL1-AS	-0.75289	1.307442	0.049267
ENSG0000(	MRPS24	-0.75291	6.519197	3.03E-07
ENSG0000(	AC005702.	-0.75317	7.16376	6.86E-08
ENSG0000(	DBNDD2	-0.75373	4.082379	8.27E-05
ENSG0000(	AC137630.	-0.75379	8.644047	2.27E-09
ENSG0000(	BAG1	-0.7538	6.582522	2.62E-07
ENSG0000(	UTP11	-0.75382	5.762942	1.73E-06
ENSG0000(	PTOV1-AS2	-0.75394	8.424801	3.76E-09
ENSG0000(	RXRA	-0.75447	4.420767	3.80E-05

ENSG0000(	LGR4	-0.75474	5.049989	8.91E-06
ENSG0000(	SYS1-DBND	-0.75618	4.362799	4.34E-05
ENSG0000(	EYA4	-0.7568	5.814967	1.53E-06
ENSG0000(	VEGFA	-0.75746	2.004741	0.009891
ENSG0000(	SRL	-0.75784	3.107875	0.00078
ENSG0000(	MAP2K2	-0.75796	6.828345	1.48E-07
ENSG0000(	AC104831.	-0.75815	1.731931	0.018538
ENSG0000(	TMOD4	-0.75831	4.139997	7.24E-05
ENSG0000(	MAMSTR	-0.75895	2.872723	0.001341
ENSG0000(	LNCOC1	-0.75904	2.980704	0.001045
ENSG0000(	TMEM120I	-0.75912	4.863723	1.37E-05
ENSG0000(	AC004982.	-0.75948	3.633911	0.000232
ENSG0000(	DSE	-0.75957	4.291066	5.12E-05
ENSG0000(	HOXD8	-0.75957	1.597002	0.025293
ENSG0000(	CIAO2B	-0.7611	9.455319	3.50E-10
ENSG0000(	MLST8	-0.76121	6.451053	3.54E-07
ENSG0000(	IRX3	-0.76148	1.856137	0.013927
ENSG0000(	HOXA10-A!	-0.76212	4.608162	2.47E-05
ENSG0000(	CENPV	-0.76215	4.35715	4.39E-05
ENSG0000(	COQ8A	-0.76236	2.241872	0.00573
ENSG0000(	UBXN7-AS:	-0.76262	1.537998	0.028974
ENSG0000(	KLHL25	-0.7627	3.193978	0.00064
ENSG0000(	AVIL	-0.76273	3.493953	0.000321
ENSG0000(	AL353622.:	-0.76274	1.554934	0.027865
ENSG0000(	MAN2A2	-0.76296	3.904102	0.000125
ENSG0000(	HIGD1B	-0.76302	2.730845	0.001858
ENSG0000(	KCNJ2	-0.76314	2.392541	0.00405
ENSG0000(	COBL	-0.76452	3.033132	0.000927
ENSG0000(	AL590094.:	-0.76458	2.745809	0.001796
ENSG0000(	PRRG1	-0.76489	3.62601	0.000237
ENSG0000(	AC036108.	-0.7653	1.838445	0.014506
ENSG0000(	RPL37A	-0.7656	5.609928	2.46E-06
ENSG0000(	AC003688.	-0.76562	7.023502	9.47E-08
ENSG0000(	NR4A1	-0.76593	1.34987	0.044682
ENSG0000(	MRPL23	-0.76637	7.079082	8.34E-08
ENSG0000(	AC245140.	-0.76681	8.266154	5.42E-09
ENSG0000(	CARM1	-0.76723	4.869264	1.35E-05
ENSG0000(	PRKAG3	-0.76903	2.582993	0.002612
ENSG0000(	LSMEM1	-0.76951	2.57813	0.002642
ENSG0000(	DNAJB5	-0.77001	3.693484	0.000203
ENSG0000(	RNF150	-0.77014	2.987296	0.00103
ENSG0000(	ZFHX3	-0.77068	5.584818	2.60E-06
ENSG0000(	THAP9	-0.77172	7.396949	4.01E-08
ENSG0000(	SMAD7	-0.77197	2.951864	0.001117
ENSG0000(	NFIC	-0.77385	5.169826	6.76E-06
ENSG0000(	AC107027.	-0.7739	3.802797	0.000157
ENSG0000(	NDUFA4	-0.7744	6.638663	2.30E-07

ENSG0000(	GHR	-0.77502	4.962627	1.09E-05
ENSG0000(	COL4A5	-0.77509	4.211908	6.14E-05
ENSG0000(	AC015914.	-0.77548	4.047229	8.97E-05
ENSG0000(	AC072039.	-0.77664	3.42847	0.000373
ENSG0000(	RILP	-0.77682	6.326955	4.71E-07
ENSG0000(	USP25	-0.77712	5.838614	1.45E-06
ENSG0000(	NIPSNAP2	-0.77735	4.741635	1.81E-05
ENSG0000(	FAM78A	-0.77745	4.313802	4.86E-05
ENSG0000(	FAM107A	-0.77814	2.624602	0.002374
ENSG0000(	DUPD1	-0.7785	2.295013	0.00507
ENSG0000(	RPS15	-0.77871	6.438912	3.64E-07
ENSG0000(	KLHL23	-0.77893	4.759696	1.74E-05
ENSG0000(	PGPEP1L	-0.77935	2.484335	0.003278
ENSG0000(	UACA	-0.77991	2.777277	0.00167
ENSG0000(	XK	-0.78007	3.834663	0.000146
ENSG0000(	RN7SKP202	-0.7802	1.372047	0.042457
ENSG0000(	KLF9	-0.78185	6.465919	3.42E-07
ENSG0000(	VEGFD	-0.78199	2.072172	0.008469
ENSG0000(	FEZ2	-0.78247	1.523216	0.029977
ENSG0000(	AL358473.1	-0.78251	2.325359	0.004728
ENSG0000(	AC087362.	-0.78275	1.364903	0.043162
ENSG0000(	ASB11	-0.78285	2.797764	0.001593
ENSG0000(	IFRD2	-0.78332	6.117857	7.62E-07
ENSG0000(	MIR6850	-0.78366	5.427217	3.74E-06
ENSG0000(	AC005393.	-0.78463	1.933718	0.011649
ENSG0000(	RPL18AP3	-0.78468	6.377607	4.19E-07
ENSG0000(	C15orf61	-0.78487	4.613722	2.43E-05
ENSG0000(	ALKBH7	-0.78517	5.78755	1.63E-06
ENSG0000(	PHYH	-0.78525	4.463559	3.44E-05
ENSG0000(	AC241952.	-0.7856	4.291066	5.12E-05
ENSG0000(	ZMYM4-AS	-0.78561	1.616861	0.024162
ENSG0000(	RF00066	-0.78566	2.14606	0.007144
ENSG0000(	MBP	-0.78589	5.284949	5.19E-06
ENSG0000(	EIF4EBP1	-0.78672	2.56184	0.002743
ENSG0000(	CLYBL	-0.78756	3.46607	0.000342
ENSG0000(	TOMM7	-0.78807	6.708713	1.96E-07
ENSG0000(	HSF2	-0.78857	9.996155	1.01E-10
ENSG0000(	RPL18A	-0.78909	7.947842	1.13E-08
ENSG0000(	AC021087.	-0.78909	2.139095	0.007259
ENSG0000(	RERE	-0.78927	5.292618	5.10E-06
ENSG0000(	PEX26	-0.78927	4.261923	5.47E-05
ENSG0000(	PTPN14	-0.7899	6.007068	9.84E-07
ENSG0000(	ANAPC16	-0.79017	8.528658	2.96E-09
ENSG0000(	RPL26P6	-0.79042	1.768895	0.017026
ENSG0000(	JAKMIP3	-0.79069	3.627921	0.000236
ENSG0000(	RASL12	-0.79153	6.719538	1.91E-07
ENSG0000(	MLXIP	-0.79154	4.53072	2.95E-05

ENSG0000(	MLIP	-0.79165	5.160627	6.91E-06
ENSG0000(	TTC23	-0.79205	7.4885	3.25E-08
ENSG0000(	FLT1	-0.7923	4.959121	1.10E-05
ENSG0000(	CACNG1	-0.79231	4.067532	8.56E-05
ENSG0000(	PLAG1	-0.79346	2.000163	0.009996
ENSG0000(	UBE2E3	-0.79396	7.739208	1.82E-08
ENSG0000(	TAPT1	-0.79415	6.851157	1.41E-07
ENSG0000(	IKZF2	-0.79415	4.567074	2.71E-05
ENSG0000(	LBX1	-0.79431	2.329509	0.004683
ENSG0000(	CCER2	-0.7947	1.615696	0.024227
ENSG0000(	SPEG	-0.79481	1.976513	0.010556
ENSG0000(	ZNF76	-0.79504	7.461162	3.46E-08
ENSG0000(	AL136131.:	-0.79542	1.688903	0.020469
ENSG0000(	AC108134.	-0.79605	1.629649	0.023461
ENSG0000(	ARAF	-0.79615	7.623713	2.38E-08
ENSG0000(	AGBL1	-0.79633	1.755677	0.017552
ENSG0000(	ALDH8A1	-0.79751	2.102676	0.007894
ENSG0000(	ENC1	-0.79779	3.136364	0.000731
ENSG0000(	NDUFA3	-0.79784	7.14389	7.18E-08
ENSG0000(	PTPRU	-0.79826	4.101334	7.92E-05
ENSG0000(	CISH	-0.7991	1.313894	0.048541
ENSG0000(	LRRC14B	-0.79924	2.330791	0.004669
ENSG0000(	ST3GAL3	-0.79925	4.019835	9.55E-05
ENSG0000(	HIGD2A	-0.79928	9.390004	4.07E-10
ENSG0000(	AC016739.	-0.79954	3.399426	0.000399
ENSG0000(	FAM129A	-0.79956	2.946716	0.001131
ENSG0000(	SLC25A30	-0.79991	3.104794	0.000786
ENSG0000(	INO80C	-0.80004	12.5245	2.99E-13
ENSG0000(	PDE11A	-0.80091	1.440452	0.03627
ENSG0000(	AL135905.:	-0.80122	4.060316	8.70E-05
ENSG0000(	LPIN1	-0.80129	4.013591	9.69E-05
ENSG0000(	P2RY2	-0.80162	4.077733	8.36E-05
ENSG0000(	SUGCT	-0.80191	3.421392	0.000379
ENSG0000(	SLC2A12	-0.8024	2.989069	0.001025
ENSG0000(	PTP4A1	-0.803	4.074351	8.43E-05
ENSG0000(	ACADL	-0.80369	2.32269	0.004757
ENSG0000(	AC068338.	-0.80387	2.861958	0.001374
ENSG0000(	TTC3P1	-0.80455	3.23433	0.000583
ENSG0000(	AC074143.	-0.80497	3.414955	0.000385
ENSG0000(	AC005225.	-0.80659	1.304328	0.049622
ENSG0000(	CRHR2	-0.80722	1.821212	0.015093
ENSG0000(	TACC2	-0.80761	3.481937	0.00033
ENSG0000(	MED25	-0.80832	4.158303	6.95E-05
ENSG0000(	EMC10	-0.80855	2.838923	0.001449
ENSG0000(	AC083809.	-0.80937	2.787594	0.001631
ENSG0000(	GLUL	-0.80939	2.465482	0.003424
ENSG0000(	NPTN-IT1	-0.80955	3.571744	0.000268

ENSG0000(	WRB-SH3B	-0.80963	3.56751	0.000271
ENSG0000(	RPS15A	-0.81071	4.926225	1.19E-05
ENSG0000(	CSPG4P10	-0.81171	3.056079	0.000879
ENSG0000(	RF01891	-0.81206	3.690084	0.000204
ENSG0000(	DCUN1D2	-0.81276	4.197377	6.35E-05
ENSG0000(	MSANTD1	-0.81318	2.122274	0.007546
ENSG0000(	PRKAA2	-0.81325	4.334806	4.63E-05
ENSG0000(	MIR761	-0.8133	4.646835	2.26E-05
ENSG0000(	AL451062.:	-0.81391	7.110331	7.76E-08
ENSG0000(	AC004943.	-0.81414	7.111704	7.73E-08
ENSG0000(	ADSL	-0.81414	6.20208	6.28E-07
ENSG0000(	PNPLA4	-0.81437	5.407499	3.91E-06
ENSG0000(	AC005225.	-0.81625	2.118111	0.007619
ENSG0000(	HMGB1P1(	-0.81654	1.589564	0.02573
ENSG0000(	LINGO4	-0.81796	2.214022	0.006109
ENSG0000(	HOXD9	-0.81809	1.74877	0.017833
ENSG0000(	POLE	-0.8182	6.876689	1.33E-07
ENSG0000(	SH3BGR	-0.81851	3.377464	0.000419
ENSG0000(	ZNF853	-0.81898	5.611174	2.45E-06
ENSG0000(	TRDN-AS1	-0.81906	2.294966	0.00507
ENSG0000(	GRTP1	-0.81916	3.590757	0.000257
ENSG0000(	MCRIP2	-0.81961	3.921064	0.00012
ENSG0000(	UBE2B	-0.82005	7.374597	4.22E-08
ENSG0000(	TBC1D4	-0.82024	4.802034	1.58E-05
ENSG0000(	SNHG19	-0.8203	4.643433	2.27E-05
ENSG0000(	COX6B1	-0.82147	7.6427	2.28E-08
ENSG0000(	TXLNB	-0.82182	3.992291	0.000102
ENSG0000(	NDUFV2P1	-0.8236	3.270742	0.000536
ENSG0000(	AC006942.	-0.82458	4.970198	1.07E-05
ENSG0000(	AC003688.	-0.82517	3.084194	0.000824
ENSG0000(	AL132639.:	-0.82584	4.550536	2.81E-05
ENSG0000(	RUSC2	-0.8274	5.333341	4.64E-06
ENSG0000(	CA2	-0.82786	2.583203	0.002611
ENSG0000(	ATG4D	-0.82792	5.827728	1.49E-06
ENSG0000(	MIR6747	-0.82878	5.605378	2.48E-06
ENSG0000(	PRPF39	-0.82898	9.159622	6.92E-10
ENSG0000(	AC105105.	-0.82899	2.64403	0.00227
ENSG0000(	AK3	-0.8293	3.900227	0.000126
ENSG0000(	ZNF204P	-0.83013	3.092146	0.000809
ENSG0000(	PCNT	-0.83033	3.279576	0.000525
ENSG0000(	SIMC1	-0.83152	4.729334	1.86E-05
ENSG0000(	BCHE	-0.83161	2.05494	0.008812
ENSG0000(	BX640514.	-0.83248	1.678781	0.020952
ENSG0000(	COX4I2	-0.8328	2.192618	0.006418
ENSG0000(	MPPED2	-0.83288	1.725302	0.018823
ENSG0000(	OBSCN-AS1	-0.83292	1.550453	0.028154
ENSG0000(	MCMDC2	-0.83311	5.054287	8.82E-06

ENSG0000(	MAOB	-0.83397	2.884329	0.001305
ENSG0000(	AC008038.	-0.83402	9.66229	2.18E-10
ENSG0000(	RGS5	-0.83408	6.47667	3.34E-07
ENSG0000(	TTC21B-AS	-0.83433	2.600576	0.002509
ENSG0000(	NDUFB4	-0.83452	7.725761	1.88E-08
ENSG0000(	MAF1	-0.83514	7.430476	3.71E-08
ENSG0000(	AL354712.:	-0.83558	2.073819	0.008437
ENSG0000(	RPLP1	-0.83667	7.282327	5.22E-08
ENSG0000(	RF01892	-0.83781	3.455268	0.000351
ENSG0000(	TMEM150/	-0.83805	9.374438	4.22E-10
ENSG0000(	SLC16A10	-0.83834	2.43831	0.003645
ENSG0000(	KLF13	-0.83916	5.883671	1.31E-06
ENSG0000(	CHDH	-0.84003	2.198818	0.006327
ENSG0000(	POLR2J3	-0.84006	4.409497	3.89E-05
ENSG0000(	EFNB2	-0.84059	12.02463	9.45E-13
ENSG0000(	CDKN2AIPI	-0.84321	7.658994	2.19E-08
ENSG0000(	TRIB1	-0.84367	3.618262	0.000241
ENSG0000(	MAPKAPK3	-0.84392	5.754969	1.76E-06
ENSG0000(	PKD1P5	-0.84407	2.262463	0.005464
ENSG0000(	AC093668.	-0.8442	2.74031	0.001818
ENSG0000(	WDR97	-0.84431	2.53604	0.00291
ENSG0000(	GKAP1	-0.84503	5.665645	2.16E-06
ENSG0000(	PITX2	-0.84552	3.270561	0.000536
ENSG0000(	ACOT6	-0.84651	2.284414	0.005195
ENSG0000(	HOXD3	-0.8471	1.977717	0.010526
ENSG0000(	TMEM201	-0.84844	5.40269	3.96E-06
ENSG0000(	ECI1	-0.84862	6.111925	7.73E-07
ENSG0000(	RPL32P29	-0.84952	3.07433	0.000843
ENSG0000(	AP001542.	-0.84981	4.344775	4.52E-05
ENSG0000(	PRKAR2A-/	-0.84992	3.61269	0.000244
ENSG0000(	DUSP13	-0.84995	4.549634	2.82E-05
ENSG0000(	HMGA1	-0.85063	7.15448	7.01E-08
ENSG0000(	ITGB6	-0.85158	4.347691	4.49E-05
ENSG0000(	TPRKB	-0.85188	8.765664	1.72E-09
ENSG0000(	AC099677.	-0.85344	2.569544	0.002694
ENSG0000(	AC009831.	-0.85349	2.477553	0.00333
ENSG0000(	EEF1B2P3	-0.85384	4.587318	2.59E-05
ENSG0000(	AC127029.	-0.85489	2.386175	0.00411
ENSG0000(	UBALD2	-0.85504	5.606044	2.48E-06
ENSG0000(	ARHGEF4	-0.85536	8.864808	1.37E-09
ENSG0000(	AC092611.	-0.85602	2.387801	0.004094
ENSG0000(	SCN4A	-0.8564	3.570258	0.000269
ENSG0000(	PPP1R14A	-0.85675	3.534153	0.000292
ENSG0000(	SNORD116	-0.85784	1.874397	0.013354
ENSG0000(	CLCN1	-0.85787	3.726189	0.000188
ENSG0000(	MIR3654	-0.85862	6.872444	1.34E-07
ENSG0000(	AL096711.:	-0.85879	3.636229	0.000231

ENSG0000(	PDLIM7	-0.8588	3.212723	0.000613
ENSG0000(	SYNPO	-0.85999	2.763244	0.001725
ENSG0000(	RYR1	-0.85999	2.627108	0.00236
ENSG0000(	EIF3F	-0.86074	12.64615	2.26E-13
ENSG0000(	AL353593.:	-0.86149	1.866691	0.013593
ENSG0000(	AGL	-0.86229	3.592439	0.000256
ENSG0000(	MBNL1	-0.86236	8.824704	1.50E-09
ENSG0000(	CFAP58	-0.86282	1.814638	0.015324
ENSG0000(	AL390208.:	-0.8629	6.905866	1.24E-07
ENSG0000(	LY6K	-0.86308	3.933034	0.000117
ENSG0000(	PDLIM2	-0.86353	3.804775	0.000157
ENSG0000(	AC025259.	-0.86392	1.517345	0.030385
ENSG0000(	SLC2A4	-0.86414	3.318783	0.00048
ENSG0000(	LONRF2	-0.86451	4.044432	9.03E-05
ENSG0000(	MIR6800	-0.86458	4.0178	9.60E-05
ENSG0000(	RPL29	-0.86467	7.466292	3.42E-08
ENSG0000(	SGF29	-0.86487	8.368893	4.28E-09
ENSG0000(	FAM50B	-0.86518	5.141387	7.22E-06
ENSG0000(	RPSAP26	-0.86523	1.698593	0.020017
ENSG0000(	AC015909.	-0.86595	2.194965	0.006383
ENSG0000(	AC126755.	-0.86609	2.854994	0.001396
ENSG0000(	PLPP7	-0.86622	4.160656	6.91E-05
ENSG0000(	AC010300.	-0.86677	1.903119	0.012499
ENSG0000(	PRKN	-0.86716	5.368442	4.28E-06
ENSG0000(	NEDD4	-0.8674	5.883286	1.31E-06
ENSG0000(	AL158071.4	-0.86818	1.635611	0.023141
ENSG0000(	F8	-0.86846	12.75596	1.75E-13
ENSG0000(	ZNF784	-0.86857	4.160978	6.90E-05
ENSG0000(	PPM1N	-0.869	5.504278	3.13E-06
ENSG0000(	TNK2-AS1	-0.86992	6.537708	2.90E-07
ENSG0000(	PXMP2	-0.87126	3.825825	0.000149
ENSG0000(	SNORD62B	-0.87218	5.141086	7.23E-06
ENSG0000(	MKNK2	-0.87239	3.650646	0.000224
ENSG0000(	GOLGA2P1	-0.87283	3.223435	0.000598
ENSG0000(	AL138689.:	-0.87349	9.902004	1.25E-10
ENSG0000(	SLC37A4	-0.87384	3.177888	0.000664
ENSG0000(	TMEM233	-0.87497	4.889672	1.29E-05
ENSG0000(	LINC01996	-0.87512	1.535566	0.029136
ENSG0000(	FAM86JP	-0.87669	2.13363	0.007351
ENSG0000(	AC067969.	-0.87707	2.697249	0.002008
ENSG0000(	TTN-AS1	-0.87733	2.121336	0.007562
ENSG0000(	OPTN	-0.87854	3.990659	0.000102
ENSG0000(	SLC6A1	-0.8786	2.936633	0.001157
ENSG0000(	TRDN	-0.87885	2.093368	0.008066
ENSG0000(	AC107886.	-0.87922	2.728404	0.001869
ENSG0000(	CCNG1	-0.87978	5.444615	3.59E-06
ENSG0000(	MIR324	-0.88053	2.757243	0.001749

ENSG0000(	DNAJC16	-0.88092	5.722471	1.89E-06
ENSG0000(	ABHD1	-0.8811	4.964426	1.09E-05
ENSG0000(	AP001893.	-0.88224	1.388238	0.040904
ENSG0000(	TAL2	-0.88276	2.786145	0.001636
ENSG0000(	RFESD	-0.88328	2.939628	0.001149
ENSG0000(	DRD4	-0.88407	2.302869	0.004979
ENSG0000(	SESN1	-0.88469	6.353245	4.43E-07
ENSG0000(	LGALS4	-0.88531	1.754037	0.017618
ENSG0000(	ADCY2	-0.88589	5.065043	8.61E-06
ENSG0000(	RNF126	-0.88597	8.629092	2.35E-09
ENSG0000(	MRLN	-0.88691	3.869819	0.000135
ENSG0000(	DDIT4-AS1	-0.88794	2.675115	0.002113
ENSG0000(	AC083843.	-0.888	2.500388	0.003159
ENSG0000(	ZBTB16	-0.89073	2.651913	0.002229
ENSG0000(	AL353593.:	-0.89094	1.901832	0.012536
ENSG0000(	VWA8	-0.89145	4.246944	5.66E-05
ENSG0000(	COX5A	-0.89181	5.816864	1.52E-06
ENSG0000(	CASTOR2	-0.89215	4.169003	6.78E-05
ENSG0000(	CRLF1	-0.89228	4.591561	2.56E-05
ENSG0000(	PNCK	-0.89271	3.456911	0.000349
ENSG0000(	RF01882	-0.89292	2.745947	0.001795
ENSG0000(	RF00322	-0.89372	3.711299	0.000194
ENSG0000(	AC007969.	-0.89481	3.212597	0.000613
ENSG0000(	MYL1	-0.89644	4.033991	9.25E-05
ENSG0000(	MED14OS	-0.89671	4.901162	1.26E-05
ENSG0000(	RTN2	-0.89688	5.383836	4.13E-06
ENSG0000(	GRB10	-0.89821	5.124685	7.50E-06
ENSG0000(	OSBPL7	-0.89826	3.360685	0.000436
ENSG0000(	ADAMTS7F	-0.89832	2.701244	0.00199
ENSG0000(	AC036108.	-0.89956	3.65775	0.00022
ENSG0000(	SIVA1	-0.89974	9.468034	3.40E-10
ENSG0000(	AC018766.	-0.89989	8.885112	1.30E-09
ENSG0000(	AC036108.	-0.90006	2.311805	0.004877
ENSG0000(	SLC25A25	-0.90053	2.645749	0.002261
ENSG0000(	AC008035.	-0.90103	2.514463	0.003059
ENSG0000(	SLC6A8	-0.9011	4.355884	4.41E-05
ENSG0000(	AL121992.:	-0.90116	4.882479	1.31E-05
ENSG0000(	CXorf57	-0.9012	2.994603	0.001013
ENSG0000(	GPD2	-0.90122	4.169003	6.78E-05
ENSG0000(	MAP2K6	-0.90132	2.968258	0.001076
ENSG0000(	HOXA4	-0.90146	3.871369	0.000134
ENSG0000(	AC008014.	-0.90254	3.882146	0.000131
ENSG0000(	HOXA5	-0.9027	4.032822	9.27E-05
ENSG0000(	CNBD2	-0.90349	7.084207	8.24E-08
ENSG0000(	SOS2	-0.90489	8.954001	1.11E-09
ENSG0000(	AC093305.	-0.90516	4.196402	6.36E-05
ENSG0000(	AL353708.:	-0.90566	7.454585	3.51E-08

ENSG0000(	TOB1-AS1	-0.90581	3.396184	0.000402
ENSG0000(	AC037459.	-0.90673	3.796533	0.00016
ENSG0000(	AP002990.	-0.90675	12.00966	9.78E-13
ENSG0000(	AC007375.	-0.90688	3.908164	0.000124
ENSG0000(	KIAA0408	-0.90708	3.770588	0.00017
ENSG0000(	SNHG6	-0.90761	5.668198	2.15E-06
ENSG0000(	AC005329.	-0.90796	4.684021	2.07E-05
ENSG0000(	KCNT1	-0.9087	2.009247	0.009789
ENSG0000(	PRSS56	-0.90968	2.951683	0.001118
ENSG0000(	POLR2J3	-0.91047	5.878125	1.32E-06
ENSG0000(	RNF128	-0.91058	3.046329	0.000899
ENSG0000(	MDH2	-0.91067	5.059574	8.72E-06
ENSG0000(	NDUFS8	-0.91143	6.529346	2.96E-07
ENSG0000(	AC091390.	-0.91225	4.15804	6.95E-05
ENSG0000(	LGR5	-0.91227	1.42915	0.037226
ENSG0000(	LMCD1	-0.9123	3.251129	0.000561
ENSG0000(	EEF1G	-0.9131	11.96974	1.07E-12
ENSG0000(	JUP	-0.91316	5.896766	1.27E-06
ENSG0000(	ASB4	-0.91399	2.3438	0.004531
ENSG0000(	ZNF579	-0.91648	4.662151	2.18E-05
ENSG0000(	DHPS	-0.91672	11.31362	4.86E-12
ENSG0000(	GYPC	-0.91673	8.286053	5.18E-09
ENSG0000(	ALDH1L1-A	-0.91821	2.052589	0.00886
ENSG0000(	NDUFB11	-0.91898	9.212556	6.13E-10
ENSG0000(	SNAI3	-0.91903	1.9298	0.011754
ENSG0000(	LRRC38	-0.92137	2.749422	0.001781
ENSG0000(	B4GALNT3	-0.9232	2.431864	0.003699
ENSG0000(	AC021242.	-0.92334	3.282969	0.000521
ENSG0000(	HJV	-0.92342	5.704958	1.97E-06
ENSG0000(	NDUFB10	-0.92477	5.67104	2.13E-06
ENSG0000(	NUDT8	-0.9254	3.118775	0.000761
ENSG0000(	CES3	-0.92594	2.50567	0.003121
ENSG0000(	DDIT4	-0.92613	2.826509	0.001491
ENSG0000(	TMEM143	-0.92635	4.219586	6.03E-05
ENSG0000(	TRIM52-AS	-0.92645	5.163793	6.86E-06
ENSG0000(	ZIC4	-0.92733	3.228549	0.000591
ENSG0000(	AC020765.	-0.92743	9.395834	4.02E-10
ENSG0000(	RPP25L	-0.92753	6.55235	2.80E-07
ENSG0000(	TARID	-0.92931	4.428372	3.73E-05
ENSG0000(	MACROD1	-0.93093	5.633468	2.33E-06
ENSG0000(	TFEB	-0.93137	6.910089	1.23E-07
ENSG0000(	AC126755.	-0.9315	2.065708	0.008596
ENSG0000(	TPM2	-0.93158	4.787733	1.63E-05
ENSG0000(	C19orf47	-0.93178	8.32277	4.76E-09
ENSG0000(	TBC1D1	-0.93205	8.697028	2.01E-09
ENSG0000(	PKP2	-0.93316	2.952321	0.001116
ENSG0000(	PRDM10	-0.93355	8.298775	5.03E-09

ENSG0000(	MAP10	-0.93359	5.442866	3.61E-06
ENSG0000(	POLR2I	-0.93428	7.603944	2.49E-08
ENSG0000(	RPL38	-0.93467	8.099003	7.96E-09
ENSG0000(	MUM1L1	-0.93513	5.571487	2.68E-06
ENSG0000(	AC004080.	-0.93559	4.222511	5.99E-05
ENSG0000(	HOXA13	-0.93573	2.162264	0.006882
ENSG0000(	RPL7P3	-0.93593	2.851599	0.001407
ENSG0000(	CITED4	-0.936	3.246961	0.000566
ENSG0000(	PHKG1	-0.93791	5.808149	1.56E-06
ENSG0000(	TMEM159	-0.9385	5.805892	1.56E-06
ENSG0000(	PTGDR2	-0.93872	2.907421	0.001238
ENSG0000(	AC245297.	-0.9398	2.462562	0.003447
ENSG0000(	HYAL1	-0.94032	6.207255	6.21E-07
ENSG0000(	AC044860.	-0.94068	1.344884	0.045198
ENSG0000(	PITX1	-0.94157	1.402301	0.0396
ENSG0000(	AC000078.	-0.94209	3.214929	0.00061
ENSG0000(	MRPS15	-0.94287	8.791955	1.61E-09
ENSG0000(	PTPN20	-0.94391	1.984816	0.010356
ENSG0000(	NRG4	-0.94429	3.054361	0.000882
ENSG0000(	MID1IP1	-0.94473	5.936715	1.16E-06
ENSG0000(	ASB10	-0.94495	3.645395	0.000226
ENSG0000(	POLR2J	-0.94525	8.728972	1.87E-09
ENSG0000(	HSPA2	-0.94641	2.497714	0.003179
ENSG0000(	ART5	-0.94661	3.583145	0.000261
ENSG0000(	DMD	-0.9468	4.852841	1.40E-05
ENSG0000(	AD001527.	-0.94781	7.720617	1.90E-08
ENSG0000(	ASB8	-0.94825	8.644047	2.27E-09
ENSG0000(	SNORD107	-0.9485	1.562915	0.027358
ENSG0000(	TMEM70	-0.94902	2.277678	0.005276
ENSG0000(	RNPC3	-0.9493	5.558268	2.77E-06
ENSG0000(	FITM1	-0.94955	5.531878	2.94E-06
ENSG0000(	ALDH1L1	-0.94981	2.502697	0.003143
ENSG0000(	AC097359.	-0.95227	4.860212	1.38E-05
ENSG0000(	NDUFS7	-0.95419	5.143304	7.19E-06
ENSG0000(	GPR179	-0.95473	1.596682	0.025312
ENSG0000(	ZNF192P1	-0.95557	3.69102	0.000204
ENSG0000(	FKBP5	-0.95683	2.348763	0.00448
ENSG0000(	EPS15L1	-0.95934	7.294589	5.07E-08
ENSG0000(	NIPAL1	-0.96143	3.260244	0.000549
ENSG0000(	PCDH9	-0.96188	3.208199	0.000619
ENSG0000(	AC005682.	-0.96235	2.452829	0.003525
ENSG0000(	MIR4442	-0.96438	1.820232	0.015128
ENSG0000(	RGMA	-0.96471	6.303249	4.97E-07
ENSG0000(	TNK2	-0.96634	5.485992	3.27E-06
ENSG0000(	GADL1	-0.96662	2.182856	0.006564
ENSG0000(	SEMA6D	-0.96668	4.652382	2.23E-05
ENSG0000(	MIR637	-0.96699	2.200809	0.006298

ENSG0000(	AC025627.	-0.96768	3.170608	0.000675
ENSG0000(	AC007998.	-0.96835	14.69953	2.00E-15
ENSG0000(	GTF2IRD1	-0.96898	5.897665	1.27E-06
ENSG0000(	MT1X	-0.96952	3.318991	0.00048
ENSG0000(	TSPYL1	-0.97108	4.50283	3.14E-05
ENSG0000(	IDI2	-0.97342	1.636143	0.023113
ENSG0000(	AC083843.	-0.97414	3.712942	0.000194
ENSG0000(	NOS1	-0.97536	1.784149	0.016438
ENSG0000(	AC005329.	-0.97568	5.361834	4.35E-06
ENSG0000(	SNTA1	-0.97682	4.519801	3.02E-05
ENSG0000(	GSDMC	-0.97751	2.183561	0.006553
ENSG0000(	HSF4	-0.97759	3.77952	0.000166
ENSG0000(	AC007920.	-0.97815	1.782482	0.016501
ENSG0000(	FLRT3	-0.97896	3.7269	0.000188
ENSG0000(	LONRF3	-0.97919	4.50404	3.13E-05
ENSG0000(	MSRB3	-0.97969	6.462448	3.45E-07
ENSG0000(	C22orf15	-0.98067	4.411111	3.88E-05
ENSG0000(	AC026461.	-0.98102	3.490833	0.000323
ENSG0000(	ZIC1	-0.98171	2.46218	0.00345
ENSG0000(	PKDCC	-0.98413	4.180557	6.60E-05
ENSG0000(	CA8	-0.98577	3.773122	0.000169
ENSG0000(	AL024497.:	-0.98586	4.813099	1.54E-05
ENSG0000(	NRGN	-0.98617	1.381188	0.041573
ENSG0000(	RET	-0.98628	3.592796	0.000255
ENSG0000(	ATP1A2	-0.98657	5.590215	2.57E-06
ENSG0000(	AC022784.	-0.98702	5.559699	2.76E-06
ENSG0000(	RXRG	-0.98702	3.760821	0.000173
ENSG0000(	ATP23	-0.98748	4.092006	8.09E-05
ENSG0000(	EEF1B2	-0.98757	11.34408	4.53E-12
ENSG0000(	AC073167.	-0.98785	2.812727	0.001539
ENSG0000(	TOB2	-0.98792	5.186673	6.51E-06
ENSG0000(	CAMK2B	-0.9881	3.686853	0.000206
ENSG0000(	RNFT1-DT	-0.99031	4.918796	1.21E-05
ENSG0000(	PGM2L1	-0.99157	4.699082	2.00E-05
ENSG0000(	AC008250.	-0.99324	3.471316	0.000338
ENSG0000(	ASB12	-0.99332	4.913299	1.22E-05
ENSG0000(	TUBA8	-0.99507	3.957236	0.00011
ENSG0000(	TPPP2	-0.9955	5.038095	9.16E-06
ENSG0000(	ATP5F1D	-0.99731	5.36717	4.29E-06
ENSG0000(	AC093668.	-0.99834	6.580455	2.63E-07
ENSG0000(	TTN	-0.99997	3.339476	0.000458
ENSG0000(	LRRC77P	-1.00116	2.75914	0.001741
ENSG0000(	YBX1P10	-1.00248	8.540769	2.88E-09
ENSG0000(	CDCA7	-1.00302	5.018406	9.59E-06
ENSG0000(	FHL2	-1.00329	3.408053	0.000391
ENSG0000(	AL158850.:	-1.00553	4.874956	1.33E-05
ENSG0000(	AC012651.	-1.0066	5.130415	7.41E-06

ENSG0000(	PFN1P2	-1.00714	2.802977	0.001574
ENSG0000(	SCN7A	-1.00715	3.167359	0.00068
ENSG0000(	CACNA1S	-1.00837	4.839849	1.45E-05
ENSG0000(	MYRIP	-1.00849	4.305576	4.95E-05
ENSG0000(	SMTNL2	-1.0089	4.005769	9.87E-05
ENSG0000(	C9orf131	-1.00958	1.741206	0.018147
ENSG0000(	MIB2	-1.00962	7.366462	4.30E-08
ENSG0000(	SH2B2	-1.01144	4.557396	2.77E-05
ENSG0000(	CAPN3	-1.01164	5.086864	8.19E-06
ENSG0000(	AC008079.	-1.01241	4.258399	5.52E-05
ENSG0000(	SOX9-AS1	-1.01274	3.842285	0.000144
ENSG0000(	SPDYE6	-1.0139	3.142893	0.00072
ENSG0000(	LANCL1-AS	-1.014	3.284926	0.000519
ENSG0000(	PLEKHH3	-1.01413	5.997331	1.01E-06
ENSG0000(	NOXA1	-1.01449	3.894599	0.000127
ENSG0000(	MIR29B2CI	-1.01535	4.485247	3.27E-05
ENSG0000(	COL4A4	-1.01581	6.915847	1.21E-07
ENSG0000(	KBTBD13	-1.01773	3.120247	0.000758
ENSG0000(	MYOZ1	-1.01823	5.002917	9.93E-06
ENSG0000(	FMO2	-1.01886	5.986579	1.03E-06
ENSG0000(	TMEM108	-1.01977	8.848394	1.42E-09
ENSG0000(	HOXD-AS2	-1.02036	2.312665	0.004868
ENSG0000(	TMEM240	-1.02148	4.407561	3.91E-05
ENSG0000(	JPH2	-1.02408	3.783002	0.000165
ENSG0000(	AL161668.4	-1.02659	5.195838	6.37E-06
ENSG0000(	AC008760.	-1.02701	7.249893	5.62E-08
ENSG0000(	MIR1-2	-1.02813	5.641704	2.28E-06
ENSG0000(	AC138904.	-1.02952	3.89509	0.000127
ENSG0000(	GOLGA2P7	-1.03029	2.130245	0.007409
ENSG0000(	PGP	-1.03041	6.286805	5.17E-07
ENSG0000(	MYBPC1	-1.03073	4.027687	9.38E-05
ENSG0000(	ERBB4	-1.03146	4.879987	1.32E-05
ENSG0000(	FKBP3	-1.03148	8.041353	9.09E-09
ENSG0000(	MAP3K20-A	-1.03332	3.015861	0.000964
ENSG0000(	PDE11A	-1.03585	1.678199	0.02098
ENSG0000(	HFM1	-1.03596	5.638149	2.30E-06
ENSG0000(	CNKSR1	-1.03713	4.812155	1.54E-05
ENSG0000(	LRRC20	-1.03763	3.826101	0.000149
ENSG0000(	GDAP1	-1.03782	5.331501	4.66E-06
ENSG0000(	YBX1P1	-1.03809	11.89872	1.26E-12
ENSG0000(	COX7A1	-1.03869	5.739582	1.82E-06
ENSG0000(	BRICD5	-1.03895	6.244651	5.69E-07
ENSG0000(	SLC25A30-A	-1.04068	3.806162	0.000156
ENSG0000(	YBX1	-1.04225	17.79276	1.61E-18
ENSG0000(	PLEKHN1	-1.04283	2.532078	0.002937
ENSG0000(	TLE2	-1.04353	8.788859	1.63E-09
ENSG0000(	AMY2B	-1.04576	8.299046	5.02E-09

ENSG0000(	HIST1H3E	-1.04607	3.165841	0.000683
ENSG0000(	UNC13B	-1.05017	10.80157	1.58E-11
ENSG0000(	TMEM38A	-1.05076	6.424227	3.77E-07
ENSG0000(	SCARNA2	-1.05167	3.791252	0.000162
ENSG0000(	AL356488.:	-1.05167	3.791252	0.000162
ENSG0000(	SPDYE2	-1.05316	3.499195	0.000317
ENSG0000(	NKAPL	-1.05353	4.697859	2.01E-05
ENSG0000(	SLC11A1	-1.05369	1.387189	0.041003
ENSG0000(	VWA7	-1.054	4.635351	2.32E-05
ENSG0000(	KCNJ12	-1.0549	3.140116	0.000724
ENSG0000(	BOLL	-1.05692	3.234219	0.000583
ENSG0000(	PERM1	-1.05926	2.553662	0.002795
ENSG0000(	BCAP31P1	-1.06085	1.30692	0.049326
ENSG0000(	AP002812.	-1.06179	4.502321	3.15E-05
ENSG0000(	AP002812.	-1.06484	4.638772	2.30E-05
ENSG0000(	PGM1	-1.06546	3.744644	0.00018
ENSG0000(	ATXN7L2	-1.06869	4.965971	1.08E-05
ENSG0000(	MYORG	-1.06914	2.803342	0.001573
ENSG0000(	PPP3CB	-1.06924	7.974834	1.06E-08
ENSG0000(	MIR133A1	-1.0695	6.36098	4.36E-07
ENSG0000(	AC117505.	-1.06974	4.046222	8.99E-05
ENSG0000(	AC245033.	-1.074	4.328929	4.69E-05
ENSG0000(	TLE1	-1.07542	3.25323	0.000558
ENSG0000(	GAPDHP1	-1.07658	3.789392	0.000162
ENSG0000(	PPP1R1A	-1.07691	3.255227	0.000556
ENSG0000(	AL645728.:	-1.08	2.154239	0.007011
ENSG0000(	MSS51	-1.08151	2.748817	0.001783
ENSG0000(	LINC02097	-1.0856	3.793023	0.000161
ENSG0000(	HERC2P3	-1.0866	2.134573	0.007335
ENSG0000(	GBX1	-1.08665	2.708367	0.001957
ENSG0000(	ZNF469	-1.09172	4.992993	1.02E-05
ENSG0000(	AAMDC	-1.09216	4.964817	1.08E-05
ENSG0000(	IRX5	-1.09241	1.638407	0.022993
ENSG0000(	AC072022.	-1.09318	3.992783	0.000102
ENSG0000(	PPP1R3A	-1.0933	5.251344	5.61E-06
ENSG0000(	SLC38A3	-1.09575	2.387154	0.004101
ENSG0000(	DUSP26	-1.09694	6.626939	2.36E-07
ENSG0000(	SLC47A1	-1.09748	5.291796	5.11E-06
ENSG0000(	ATP13A5-A	-1.09965	2.217887	0.006055
ENSG0000(	AC068700.	-1.10062	3.818335	0.000152
ENSG0000(	MIR6073	-1.10162	3.840218	0.000144
ENSG0000(	SNORA31	-1.10369	7.201342	6.29E-08
ENSG0000(	TSC22D1	-1.10645	8.136	7.31E-09
ENSG0000(	SOX6	-1.1071	5.967582	1.08E-06
ENSG0000(	BTNL9	-1.11101	3.799211	0.000159
ENSG0000(	MASP1	-1.11869	3.066118	0.000859
ENSG0000(	TRIP10	-1.11895	10.72362	1.89E-11

ENSG0000(	AC011287.	-1.12159	1.445976	0.035812
ENSG0000(	AC037441.	-1.12569	1.744617	0.018005
ENSG0000(	ITGB3BP	-1.12651	5.19695	6.35E-06
ENSG0000(	MIR29C	-1.12791	3.638601	0.00023
ENSG0000(	ZNF30-AS1	-1.12816	1.38126	0.041566
ENSG0000(	AC009078.	-1.12858	3.892988	0.000128
ENSG0000(	PAX3	-1.13011	8.759394	1.74E-09
ENSG0000(	ASB15	-1.13179	3.370324	0.000426
ENSG0000(	RN7SL130F	-1.13286	1.503311	0.031383
ENSG0000(	TMEM246	-1.13425	6.240674	5.75E-07
ENSG0000(	PPP1R3C	-1.13494	5.532591	2.93E-06
ENSG0000(	DKFZp779M	-1.13587	2.684638	0.002067
ENSG0000(	FABP3	-1.138	3.763404	0.000172
ENSG0000(	CHCHD10	-1.13887	6.154275	7.01E-07
ENSG0000(	LINC00240	-1.14299	1.362869	0.043364
ENSG0000(	GAPDH	-1.14363	5.606044	2.48E-06
ENSG0000(	MLF1	-1.14539	2.946645	0.001131
ENSG0000(	TPT1	-1.14603	11.72104	1.90E-12
ENSG0000(	ATP13A5	-1.14835	2.920415	0.001201
ENSG0000(	MYBPC2	-1.14847	2.590654	0.002567
ENSG0000(	TMEM88	-1.14955	8.099003	7.96E-09
ENSG0000(	ITIH6	-1.1544	3.787849	0.000163
ENSG0000(	AC044839.	-1.15521	2.304363	0.004962
ENSG0000(	ANXA3	-1.15611	5.16304	6.87E-06
ENSG0000(	ARNT2	-1.1563	4.922608	1.20E-05
ENSG0000(	ABLIM2	-1.15658	6.852117	1.41E-07
ENSG0000(	AC093330.	-1.15794	4.169059	6.78E-05
ENSG0000(	DOK5	-1.15826	4.221683	6.00E-05
ENSG0000(	AC005323.	-1.15991	2.571833	0.00268
ENSG0000(	HPN	-1.16278	3.376689	0.00042
ENSG0000(	SPAAR	-1.16354	7.302977	4.98E-08
ENSG0000(	AC011815.	-1.1636	1.534355	0.029218
ENSG0000(	PLEKHF1	-1.16379	6.11041	7.76E-07
ENSG0000(	AC092153.	-1.16438	7.800327	1.58E-08
ENSG0000(	SGCA	-1.16559	8.069258	8.53E-09
ENSG0000(	MYH13	-1.16797	2.469051	0.003396
ENSG0000(	PFKFB1	-1.16891	4.363939	4.33E-05
ENSG0000(	AC006064.	-1.16896	5.52112	3.01E-06
ENSG0000(	MIR133A1I	-1.17091	9.41389	3.86E-10
ENSG0000(	LINC01697	-1.17211	2.942502	0.001142
ENSG0000(	DEPTOR	-1.17347	8.752215	1.77E-09
ENSG0000(	GRTP1-AS1	-1.17481	3.347615	0.000449
ENSG0000(	RTN4RL1	-1.17494	3.512307	0.000307
ENSG0000(	MBNL1-AS:	-1.17544	5.714301	1.93E-06
ENSG0000(	AL138963.:	-1.17575	13.45201	3.53E-14
ENSG0000(	PPM1J	-1.17765	3.362379	0.000434
ENSG0000(	KCNQ5	-1.18354	5.446542	3.58E-06

ENSG0000(	SMCO1	-1.18836	3.895806	0.000127
ENSG0000(	AC020909.	-1.18847	3.41117	0.000388
ENSG0000(	JPH1	-1.18885	4.84507	1.43E-05
ENSG0000(	SLC38A4	-1.19082	2.887932	0.001294
ENSG0000(	PIK3R1	-1.19419	8.28643	5.17E-09
ENSG0000(	GMPR	-1.19436	6.031333	9.30E-07
ENSG0000(	UBE2Q2P2	-1.19739	2.281867	0.005226
ENSG0000(	RSC1A1	-1.19888	7.281795	5.23E-08
ENSG0000(	KCNA5	-1.20021	4.163688	6.86E-05
ENSG0000(	UGT3A1	-1.20027	2.095701	0.008022
ENSG0000(	EEF1A2	-1.20424	7.042257	9.07E-08
ENSG0000(	AC105052.	-1.20624	1.322627	0.047574
ENSG0000(	AC091563.	-1.21071	5.714261	1.93E-06
ENSG0000(	GREM1	-1.21071	2.727274	0.001874
ENSG0000(	HIST1H1C	-1.21643	7.103654	7.88E-08
ENSG0000(	AC020917.	-1.21669	5.398769	3.99E-06
ENSG0000(	SLC16A12	-1.21707	1.37328	0.042337
ENSG0000(	AP005717.	-1.21841	9.584307	2.60E-10
ENSG0000(	IP6K3	-1.21948	7.056833	8.77E-08
ENSG0000(	SCN1B	-1.22025	8.223265	5.98E-09
ENSG0000(	G0S2	-1.22175	2.058361	0.008743
ENSG0000(	PYGM	-1.22293	4.511291	3.08E-05
ENSG0000(	FXYD1	-1.22457	10.45918	3.47E-11
ENSG0000(	RORC	-1.2256	7.406268	3.92E-08
ENSG0000(	CD38	-1.22761	6.961874	1.09E-07
ENSG0000(	RASL10B	-1.22802	2.614072	0.002432
ENSG0000(	AC072022.	-1.22845	3.404267	0.000394
ENSG0000(	BCL6	-1.23012	4.512982	3.07E-05
ENSG0000(	ITIH2	-1.23037	1.599756	0.025133
ENSG0000(	EGFLAM-A!	-1.23043	4.058231	8.75E-05
ENSG0000(	SATB1	-1.23073	7.808222	1.56E-08
ENSG0000(	AC020907.	-1.23226	10.54514	2.85E-11
ENSG0000(	LMO1	-1.23246	3.982338	0.000104
ENSG0000(	NFE2	-1.23454	1.300387	0.050074
ENSG0000(	ALG1L	-1.23899	3.412205	0.000387
ENSG0000(	TCEA3	-1.24117	7.404776	3.94E-08
ENSG0000(	RF01881	-1.242	4.745895	1.80E-05
ENSG0000(	AP005137.	-1.24664	3.78538	0.000164
ENSG0000(	CD300LG	-1.24674	3.343684	0.000453
ENSG0000(	AGMAT	-1.24834	3.827984	0.000149
ENSG0000(	PPDPFL	-1.25057	2.728936	0.001867
ENSG0000(	GADD45GI	-1.25076	11.00594	9.86E-12
ENSG0000(	AC034187.	-1.251	1.662579	0.021748
ENSG0000(	MYLPF	-1.25321	4.694412	2.02E-05
ENSG0000(	CMBL	-1.25809	5.255023	5.56E-06
ENSG0000(	FAM160A1	-1.25821	3.759629	0.000174
ENSG0000(	DBNL	-1.25837	5.063968	8.63E-06

ENSG0000(	AP001107.	-1.25908	6.721397	1.90E-07
ENSG0000(	SELENOW	-1.2609	10.87954	1.32E-11
ENSG0000(	CNTFR	-1.26474	4.633742	2.32E-05
ENSG0000(	LRRTM4	-1.26998	1.59876	0.025191
ENSG0000(	RGS9BP	-1.27182	3.52081	0.000301
ENSG0000(	HRASLS	-1.27353	5.022155	9.50E-06
ENSG0000(	HES1	-1.2741	10.03819	9.16E-11
ENSG0000(	NKAIN2	-1.27427	3.6625	0.000218
ENSG0000(	PPP1R3B	-1.27494	5.65585	2.21E-06
ENSG0000(	FO393414.	-1.28649	4.753023	1.77E-05
ENSG0000(	COX6A2	-1.28819	7.458031	3.48E-08
ENSG0000(	FAAH	-1.28928	8.056027	8.79E-09
ENSG0000(	AMHR2	-1.28998	3.199177	0.000632
ENSG0000(	AC026471.	-1.29947	7.635949	2.31E-08
ENSG0000(	KLF10	-1.30151	4.766826	1.71E-05
ENSG0000(	AC097662.	-1.30316	6.031333	9.30E-07
ENSG0000(	HPN-AS1	-1.31422	3.669416	0.000214
ENSG0000(	ALDH1L1-A	-1.31486	4.027013	9.40E-05
ENSG0000(	AC139795.	-1.31693	6.199614	6.32E-07
ENSG0000(	AL158070.:	-1.31874	3.401934	0.000396
ENSG0000(	ADSSL1	-1.32065	5.560604	2.75E-06
ENSG0000(	AC010680.	-1.32342	5.553184	2.80E-06
ENSG0000(	MYLK2	-1.32386	4.650151	2.24E-05
ENSG0000(	SLC29A2	-1.32515	7.345372	4.51E-08
ENSG0000(	AL929236.:	-1.32578	1.408756	0.039016
ENSG0000(	RPS20P21	-1.32972	3.413957	0.000386
ENSG0000(	AC008443.	-1.33143	9.204291	6.25E-10
ENSG0000(	AC091100.	-1.33167	3.817884	0.000152
ENSG0000(	AL021026.:	-1.33169	6.287742	5.16E-07
ENSG0000(	ASB2	-1.3318	6.347551	4.49E-07
ENSG0000(	ENO3	-1.33245	5.012289	9.72E-06
ENSG0000(	TSPAN8	-1.33359	5.414717	3.85E-06
ENSG0000(	AC069360.	-1.33517	6.73026	1.86E-07
ENSG0000(	GAPDHP65	-1.33542	2.788059	0.001629
ENSG0000(	TRIM7	-1.33591	7.813272	1.54E-08
ENSG0000(	AC006333.	-1.33706	5.658612	2.19E-06
ENSG0000(	ACTA1	-1.33875	5.992812	1.02E-06
ENSG0000(	SIX2	-1.34031	8.973042	1.06E-09
ENSG0000(	PGAM2	-1.34051	4.787137	1.63E-05
ENSG0000(	AC004982.	-1.34224	1.313913	0.048539
ENSG0000(	SNHG5	-1.34276	5.147177	7.13E-06
ENSG0000(	AC017116.	-1.34313	4.806727	1.56E-05
ENSG0000(	GAMT	-1.34574	8.873458	1.34E-09
ENSG0000(	TMEM266	-1.34671	4.841687	1.44E-05
ENSG0000(	RPL3L	-1.35108	7.677066	2.10E-08
ENSG0000(	LRRC37A7F	-1.35257	2.231287	0.005871
ENSG0000(	FHL3	-1.36363	4.409815	3.89E-05

ENSG0000(	ATP2B2	-1.36563	3.506575	0.000311
ENSG0000(	AL132642.:	-1.36583	6.155194	7.00E-07
ENSG0000(	MSTN	-1.36613	4.137846	7.28E-05
ENSG0000(	NKAIN1	-1.36759	3.619075	0.00024
ENSG0000(	IMPA2	-1.36776	9.385831	4.11E-10
ENSG0000(	AL513217.:	-1.37187	5.739582	1.82E-06
ENSG0000(	LRRC66	-1.37361	7.363957	4.33E-08
ENSG0000(	RF00019	-1.37504	2.395909	0.004019
ENSG0000(	RABEP2	-1.37726	5.52076	3.01E-06
ENSG0000(	AC104564.	-1.37916	2.971144	0.001069
ENSG0000(	COL4A3	-1.37916	8.434408	3.68E-09
ENSG0000(	TNNT3	-1.37999	3.882343	0.000131
ENSG0000(	CMYA5	-1.38091	7.126774	7.47E-08
ENSG0000(	CFAP53	-1.38136	3.533398	0.000293
ENSG0000(	CKM	-1.3858	5.276896	5.29E-06
ENSG0000(	TSC22D1-A	-1.38733	5.755824	1.75E-06
ENSG0000(	RHOBTB1	-1.40503	4.961562	1.09E-05
ENSG0000(	TPM1	-1.4108	3.741166	0.000181
ENSG0000(	SLC14A1	-1.41157	5.200564	6.30E-06
ENSG0000(	AC017100.	-1.41241	1.474477	0.033537
ENSG0000(	MYH2	-1.41492	3.310771	0.000489
ENSG0000(	TCF15	-1.41792	5.430338	3.71E-06
ENSG0000(	TPRG1	-1.41832	3.267135	0.000541
ENSG0000(	TGFBR3L	-1.42088	1.396845	0.040101
ENSG0000(	GOLGA6L1(	-1.42377	1.598504	0.025206
ENSG0000(	AC020907.	-1.42487	12.4379	3.65E-13
ENSG0000(	AC105052.	-1.42606	7.426615	3.74E-08
ENSG0000(	NOTUM	-1.42739	3.009928	0.000977
ENSG0000(	AC138474.	-1.42751	5.622304	2.39E-06
ENSG0000(	CABP1	-1.42841	3.008711	0.00098
ENSG0000(	GPIHBP1	-1.43378	11.2036	6.26E-12
ENSG0000(	AC010680.	-1.43499	5.555452	2.78E-06
ENSG0000(	FAM181A-/	-1.43642	1.507581	0.031076
ENSG0000(	KLHL38	-1.43774	5.53832	2.90E-06
ENSG0000(	ALDOA	-1.446	5.706918	1.96E-06
ENSG0000(	AC093512.	-1.44643	5.706491	1.97E-06
ENSG0000(	AC093668.	-1.44902	7.396949	4.01E-08
ENSG0000(	USP6	-1.44903	6.535196	2.92E-07
ENSG0000(	UCP3	-1.45074	4.797193	1.60E-05
ENSG0000(	AC008443.	-1.45559	6.601775	2.50E-07
ENSG0000(	DYRK1B	-1.45993	8.917055	1.21E-09
ENSG0000(	AC067969.	-1.46295	4.932606	1.17E-05
ENSG0000(	LMOD1	-1.46603	6.117033	7.64E-07
ENSG0000(	SLC7A2	-1.47494	5.702636	1.98E-06
ENSG0000(	CSF3R	-1.47652	2.164787	0.006842
ENSG0000(	YBX3	-1.48113	9.201235	6.29E-10
ENSG0000(	RAD23A	-1.48676	11.05568	8.80E-12

ENSG0000(	TNNC2	-1.4949	4.360057	4.36E-05
ENSG0000(	TMEM52	-1.49821	5.334994	4.62E-06
ENSG0000(	B3GALT1	-1.50021	3.703282	0.000198
ENSG0000(	AC022784.	-1.50081	4.982391	1.04E-05
ENSG0000(	SMIM2-AS:	-1.50706	1.675655	0.021103
ENSG0000(	HMGCS2	-1.52584	2.122584	0.007541
ENSG0000(	DHRS7C	-1.52904	6.476692	3.34E-07
ENSG0000(	AC027045.	-1.53135	6.181993	6.58E-07
ENSG0000(	LINC02478	-1.54521	4.660231	2.19E-05
ENSG0000(	MYLK4	-1.54987	3.790346	0.000162
ENSG0000(	AC026347.	-1.55051	1.787862	0.016298
ENSG0000(	LINC01505	-1.55206	1.355824	0.044073
ENSG0000(	AC005323.	-1.55405	2.8567	0.001391
ENSG0000(	MN1	-1.55534	9.382216	4.15E-10
ENSG0000(	RASA4	-1.55738	8.966823	1.08E-09
ENSG0000(	SH3RF2	-1.56159	6.353418	4.43E-07
ENSG0000(	S100A9	-1.56718	1.506873	0.031126
ENSG0000(	TLR9	-1.57391	10.23481	5.82E-11
ENSG0000(	PEBP4	-1.57601	8.979281	1.05E-09
ENSG0000(	PWAR5	-1.57867	3.56577	0.000272
ENSG0000(	CDH20	-1.58054	1.491651	0.032237
ENSG0000(	TNNI2	-1.59456	4.139261	7.26E-05
ENSG0000(	PAIP2B	-1.6025	4.705905	1.97E-05
ENSG0000(	AC093512.	-1.6092	6.070075	8.51E-07
ENSG0000(	KCNN2	-1.61221	2.454681	0.00351
ENSG0000(	WNT4	-1.62218	5.500839	3.16E-06
ENSG0000(	AL136164.:	-1.65967	2.033794	0.009251
ENSG0000(	SLC22A3	-1.664	6.146014	7.14E-07
ENSG0000(	NANOS1	-1.66526	4.501444	3.15E-05
ENSG0000(	RNF157-AS	-1.67008	5.888305	1.29E-06
ENSG0000(	ARRDC2	-1.67201	9.059594	8.72E-10
ENSG0000(	RNF157	-1.67364	5.883671	1.31E-06
ENSG0000(	FABP5P3	-1.674	2.523155	0.002998
ENSG0000(	AC015813.	-1.67613	3.897416	0.000127
ENSG0000(	MIR1-1HG	-1.69105	6.304496	4.96E-07
ENSG0000(	MIR133A2	-1.69346	5.706918	1.96E-06
ENSG0000(	RASA4B	-1.69414	7.01162	9.74E-08
ENSG0000(	MYH1	-1.69421	1.977418	0.010534
ENSG0000(	UPK3A	-1.71144	8.099003	7.96E-09
ENSG0000(	PITX3	-1.73748	9.555108	2.79E-10
ENSG0000(	PFKFB3	-1.74879	4.512982	3.07E-05
ENSG0000(	AC010680.	-1.75374	8.223265	5.98E-09
ENSG0000(	CSF3	-1.7543	1.457236	0.034895
ENSG0000(	YBX1P2	-1.75546	1.580539	0.02627
ENSG0000(	AC005616.	-1.76001	4.430631	3.71E-05
ENSG0000(	LINC00310	-1.76201	9.147528	7.12E-10
ENSG0000(	AC020904.	-1.77615	8.323296	4.75E-09

ENSG0000(	SLC16A3	-1.77731	8.847204	1.42E-09
ENSG0000(	CYP17A1	-1.77982	9.319048	4.80E-10
ENSG0000(	ODF3L2	-1.79623	4.518822	3.03E-05
ENSG0000(	AL592437.:	-1.80008	4.195466	6.38E-05
ENSG0000(	NEK10	-1.85856	8.294157	5.08E-09
ENSG0000(	COL28A1	-1.86107	6.466961	3.41E-07
ENSG0000(	OR7E47P	-1.88176	6.437512	3.65E-07
ENSG0000(	AC012146.	-1.88474	6.040055	9.12E-07
ENSG0000(	AQP4	-1.89444	5.166328	6.82E-06
ENSG0000(	FAM166B	-1.89855	4.78411	1.64E-05
ENSG0000(	AC044839.	-1.91007	1.74116	0.018148
ENSG0000(	ATP2A1	-1.92389	4.221683	6.00E-05
ENSG0000(	ATP2A1-AS	-1.92807	4.131603	7.39E-05
ENSG0000(	AC008443.	-1.93358	10.99881	1.00E-11
ENSG0000(	OR7E47P	-1.95096	6.545113	2.85E-07
ENSG0000(	ART3	-1.96861	12.77414	1.68E-13
ENSG0000(	RHOXF1-AS	-1.97197	3.485921	0.000327
ENSG0000(	CHAF1B	-1.97943	13.21666	6.07E-14
ENSG0000(	AC010680.	-1.9878	8.255892	5.55E-09
ENSG0000(	YBX3P1	-1.99484	3.075534	0.00084
ENSG0000(	METTL21EI	-1.99641	4.082379	8.27E-05
ENSG0000(	GADD45G	-2.00572	4.986823	1.03E-05
ENSG0000(	HCN1	-2.03484	1.759481	0.017399
ENSG0000(	RASA4DP	-2.05396	5.700159	1.99E-06
ENSG0000(	CFAP46	-2.05596	5.875184	1.33E-06
ENSG0000(	C1orf127	-2.08438	7.806913	1.56E-08
ENSG0000(	DCLK3	-2.08501	3.67103	0.000213
ENSG0000(	AC016705.	-2.1023	2.845658	0.001427
ENSG0000(	HBA1	-2.14786	2.8258	0.001493
ENSG0000(	FTH1P19	-2.17531	1.322239	0.047617
ENSG0000(	S100A8	-2.29944	2.13112	0.007394
ENSG0000(	GGT7	-2.30407	12.40622	3.92E-13
ENSG0000(	AL445253.:	-2.33675	2.421892	0.003785
ENSG0000(	MIR4506	-2.33779	3.256636	0.000554
ENSG0000(	AL137246.:	-2.34115	5.155405	6.99E-06
ENSG0000(	AJAP1	-2.36536	2.215158	0.006093
ENSG0000(	HBA2	-2.3845	3.244711	0.000569
ENSG0000(	AC009948.	-2.40663	6.673037	2.12E-07
ENSG0000(	ROS1	-2.43252	2.986764	0.001031
ENSG0000(	SH2D1B	-2.45212	3.403319	0.000395
ENSG0000(	KCTD8	-2.46196	2.084106	0.008239
ENSG0000(	AC009948.	-2.47197	12.26608	5.42E-13
ENSG0000(	HBB	-2.47215	3.934006	0.000116
ENSG0000(	PKN2-AS1	-2.50615	1.700785	0.019917
ENSG0000(	TYRP1	-2.52147	2.25324	0.005582
ENSG0000(	AC009948.	-2.52574	13.21695	6.07E-14
ENSG0000(	UNC13C	-2.55244	2.905699	0.001243

ENSG0000(	CXCR2	-2.56199	1.643737	0.022712
ENSG0000(	FCGR3B	-2.61306	1.754354	0.017605
ENSG0000(	VSNL1	-2.61382	2.282448	0.005219
ENSG0000(	AL009031.:	-2.62729	2.512031	0.003076
ENSG0000(	AC018467.	-2.62842	1.397708	0.040021
ENSG0000(	KCNJ3	-2.63711	2.153181	0.007028
ENSG0000(	CFAP61	-2.64685	12.98156	1.04E-13
ENSG0000(	GDNF-AS1	-2.72547	10.72423	1.89E-11
ENSG0000(	AC079467.	-2.7734	5.254523	5.57E-06
ENSG0000(	AQP4-AS1	-2.89932	1.443947	0.035979
ENSG0000(	GDNF	-2.94513	10.33137	4.66E-11
ENSG0000(	METTL21C	-3.01283	3.512307	0.000307
ENSG0000(	MIR6787	-3.01994	1.944251	0.01137
ENSG0000(	ALAS2	-3.02307	1.578143	0.026415
ENSG0000(	RF01880	-3.08219	1.496817	0.031855
ENSG0000(	FYB2	-3.11254	2.074101	0.008431
ENSG0000(	CALML6	-3.13923	5.738187	1.83E-06
ENSG0000(	GRIK2	-3.34099	2.603792	0.00249
ENSG0000(	LINC02224	-3.40945	2.201045	0.006294
ENSG0000(	SLC4A1	-3.4989	1.635029	0.023172
ENSG0000(	NPSR1-AS1	-3.57492	1.411535	0.038767
ENSG0000(	LINC01854	-3.6535	5.83851	1.45E-06
ENSG0000(	NPTX1	-3.66311	2.114288	0.007686
ENSG0000(	CRYM	-3.77912	1.321906	0.047653
ENSG0000(	SLC6A20	-3.82544	1.693436	0.020256
ENSG0000(	SMIM2-IT1	-4.01345	2.082193	0.008276
ENSG0000(	LINC00390	-4.21914	1.839884	0.014458
ENSG0000(	LINC02107	-4.38196	1.856868	0.013904
ENSG0000(	ACTG1P10	-5.06599	1.605312	0.024813
ENSG0000(	PF4	-5.08214	1.513425	0.03066
ENSG0000(	AC025580.	-5.2605	1.657913	0.021983
ENSG0000(	PAX1	-5.67792	1.424637	0.037615
ENSG0000(	SLITRK3	-5.76923	1.997018	0.010069
ENSG0000(	AC010680.	-5.83447	1.514671	0.030572
ENSG0000(	AC092641.	-5.84309	2.795788	0.0016
ENSG0000(	SATB1-AS1	-6.07358	4.231027	5.87E-05
ENSG0000(	AL392183.:	-6.22524	2.323096	0.004752
ENSG0000(	S100A12	-6.61829	1.960727	0.010946
